# Exploring Water Beyond the Solvent: Insights into Density Fluctuations and EGFP Unfolding via Luminescence Thermometry

**DOI:** 10.1021/acs.jpcb.5c06143

**Published:** 2025-11-06

**Authors:** Yongwei Guo, Fernando E. Maturi, Ramon S. Raposo Filho, Carlos D. S. Brites, Luís D. Carlos

**Affiliations:** Phantom-g, CICECO−Aveiro Institute of Materials, Physics Department, 56062 University of Aveiro, 3810-193 Aveiro, Portugal

## Abstract

Water plays an active role in protein stability, but directly probing its density fluctuations at the protein interface remains challenging. Here, we use enhanced green fluorescent protein (EGFP) to investigate how low-density (LD) and high-density (HD) water motifs modulate unfolding in H_2_O and D_2_O. Fluorescence quenching during heating–cooling cycles indicates that unfolding begins at approximately 55 °C in H_2_O and 64 °C in D_2_O, consistent with the stabilizing effect of isotopic substitution. Circular dichroism corroborates this shift, with higher melting temperatures in D_2_O (83 vs 79 °C in H_2_O). EGFP Brownian velocity measurements, through luminescence thermometry, reveal bilinear temperature dependence with crossover temperatures of 55 °C in H_2_O and 65 °C in D_2_O, indicating that LD motifs persist longer in heavy water. Together, these results establish a fully optical strategy that directly links hydration-water structure to protein stability, providing a new route to study hydration-mediated dynamics in biomolecules.

## Graphical abstract

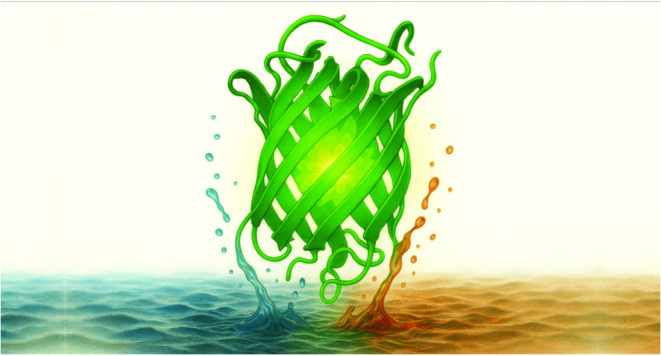

## 1 Introduction

Proteins are fundamental to life, with their structural and functional diversity underpinning essential processes such as metabolism, signaling, and cellular architecture.^1^ Among them, fluorescent proteins – especially green fluorescent protein (GFP)^2^ and engineered variants, such as enhanced GFP^3^ (EGFP) – have transformed biology by enabling live-cell imaging^4^ and sensing.^5,6^ EGFP combines higher brightness, efficient folding, and thermal stability, making it an excellent model for probing how the aqueous environment shapes protein function.

Water, once regarded as a passive solvent, is now recognized as a dynamic determinant of protein stability and activity.^7,8^ It mediates intermolecular interactions, stabilizes higher-order structures, and drives protein folding through hydrophobic collapse.^7^ Even submonolayer hydration is sufficient to sustain biological activity.^9,10^ Within the two-state model, liquid water comprises interconverting low-density (LD) and high-density (HD) motifs,^11–13^ whose competition arises from a hypothesized liquid–liquid critical point.^14,15^ Evidence from both simulations^16,17^ and experiments,^18–24^ shows that these fluctuations, sensitive to temperature and isotopic substitution,^11,13,23–25^ directly modulate the hydration shell of proteins,^10,26,27^ and thereby their stability.^7,28^

Despite advances from X-ray and neutron scattering,^18^ nuclear magnetic resonance (NMR),^29^ infrared (IR),^30^ terahertz spectroscopy,^31^ and simulations,^26^ isolating the contribution of hydration water from protein effects remains challenging.^32^ Fluorescent proteins are particularly sensitive to their hydration environment, yet this relationship is poorly explored.^7^ Experimental and computational studies consistently link unfolding to changes in hydration hydrogen-bond networks: GFP perturbs its first hydration shell,^33^ unfolding begins with its hydration collapse,^34^ and a critical temperature range (47–57 °C) marks the loss of LD structuring and onset of unfolding.^10,32,35^ These findings highlight hydration water as a key driver of protein stability and motivate the use of EGFP as a probe of LD/HD motif fluctuations.

Isotopic substitution provides another lens on hydration effects. Heavy water (D_2_O) is widely used in neutron scattering and NMR studies,^36^ yet its impact on protein stability remains only partially understood.^37^ Typically, deuteration increases melting temperatures by several degrees,^38^ reflecting a more rigid native fold.^39^

Here, we use EGFP to directly link the fluctuations between LD and HD motifs in the hydration shell to protein unfolding in H_2_O and D_2_O. By combining fluorescence quenching during heating–cooling cycles with temperature-dependent Brownian velocity measurements, we provide experimental evidence that LD motifs sustain folding and demonstrate a fully optical platform for probing hydration-mediated stability.

## 2 Experimental and Methods

### 2.1 Materials and Preparation of EGFP Suspensions

Purified recombinant EGFP (EGFP: 28 kDa, 35.71 μM) fused to 6 × His-tag was purchased from ChromoTek (storage buffer: 25 mM TAPS, pH 8.5, 500 mM NaCl, 5 mM EDTA, 0.09% sodium azide), which was diluted in ultrapure water to prepare aqueous suspensions at various concentrations (0.36–3.57 μM, corresponding to 0.2–2.2 × 10^21^ proteins per m^3^, Table S1, Supporting Information). D_2_O was obtained from Sigma-Aldrich. To prepare EGFP suspensions in D_2_O, the aqueous samples were freeze-dried, and the resulting powder was then redispersed in D_2_O. Ultrasonication was used to ensure complete dispersion, yielding final concentrations of 2.14 and 2.86 μM (Table S1, Supporting Information). The total mass fraction of buffer components from the EGFP stock solution in the diluted suspensions is approximately 0.04–0.38%. This leads to a minor variation in the pH of the suspensions (7.2–7.7 for 0.36–3.57 μM EGFP), with negligible impact on the results. The colloidal stability, circular dichroism, and absorbance spectra of the EGFP suspensions are shown in Figures S1–S7, Supporting Information.

### 2.2 Photoluminescence

Emission spectra were collected using the setup shown in Figure S8, Supporting Information. The suspensions were excited at 407 ± 7 nm using a multichannel LED source (MCLS, Sandhouse Design) with a power of 4.0 × 10^–4^ W. The excitation beam was collimated using an adjustable collimator and two plano-convex lenses (LA1145, Thorlabs), resulting in a circular 6.8 × 10^–4^ m radius beam (Figure S8d,e, Supporting Information), corresponding to an excitation power density of 0.03 W·cm^–2^. The emitted signal was collected using a portable spectrometer (Maya 2000 Pro, Ocean Optics) coupled to an optical fiber (P600–1-UV–vis, Ocean Optics). An edge-pass filter (FESH0750, Thorlabs) was used to cut off the excitation signal. Spectral acquisition was performed with a resolution of 0.5 nm and an integration time of 0.250 s. Measurements were conducted with 0.50 mL of EGFP suspension in a semimicro quartz cuvette (9F-Q-10, Starna).

### 2.3 Temperature-dependent emission spectra

To record the temperature-dependent emission spectra of the EGFP suspensions, the quartz cuvette was positioned on a temperature-controlled cuvette holder (TLC 50/E, Quantum Northwest) controlled by a dedicated temperature controller (QNW TC 1, Quantum Northwest). The temperature was changed by 5.0 °C every 8 min, and the emission spectra were acquired after the temperature reached a stable value. The temperature is continuously monitored by a K-type thermocouple (KA01–3, TME Thermometers) with a thermal resolution of 0.1 °C. In the heating–cooling cycles (maximum heating rate 0.07 °C· s^–1^), a total of 100 emission spectra were recorded at each temperature.

### 2.4 Brownian Velocity

The EGFP Brownian velocity was determined by following the procedure provided in Supporting Information (Section 9).

## 3 Results and Discussion

### 3.1 Structure and Fluorescence

Figure 1a shows the three-dimensional structure of EGFP and the amino acid composition of its chromophore. In its native state, EGFP preserves the characteristic β-barrel structure of GFP,^40^ which is crucial for stability and function and encloses the chromophore to enhance photostability^41,42^ (Figures S9, S10, Supporting Information).

**Figure 1:**
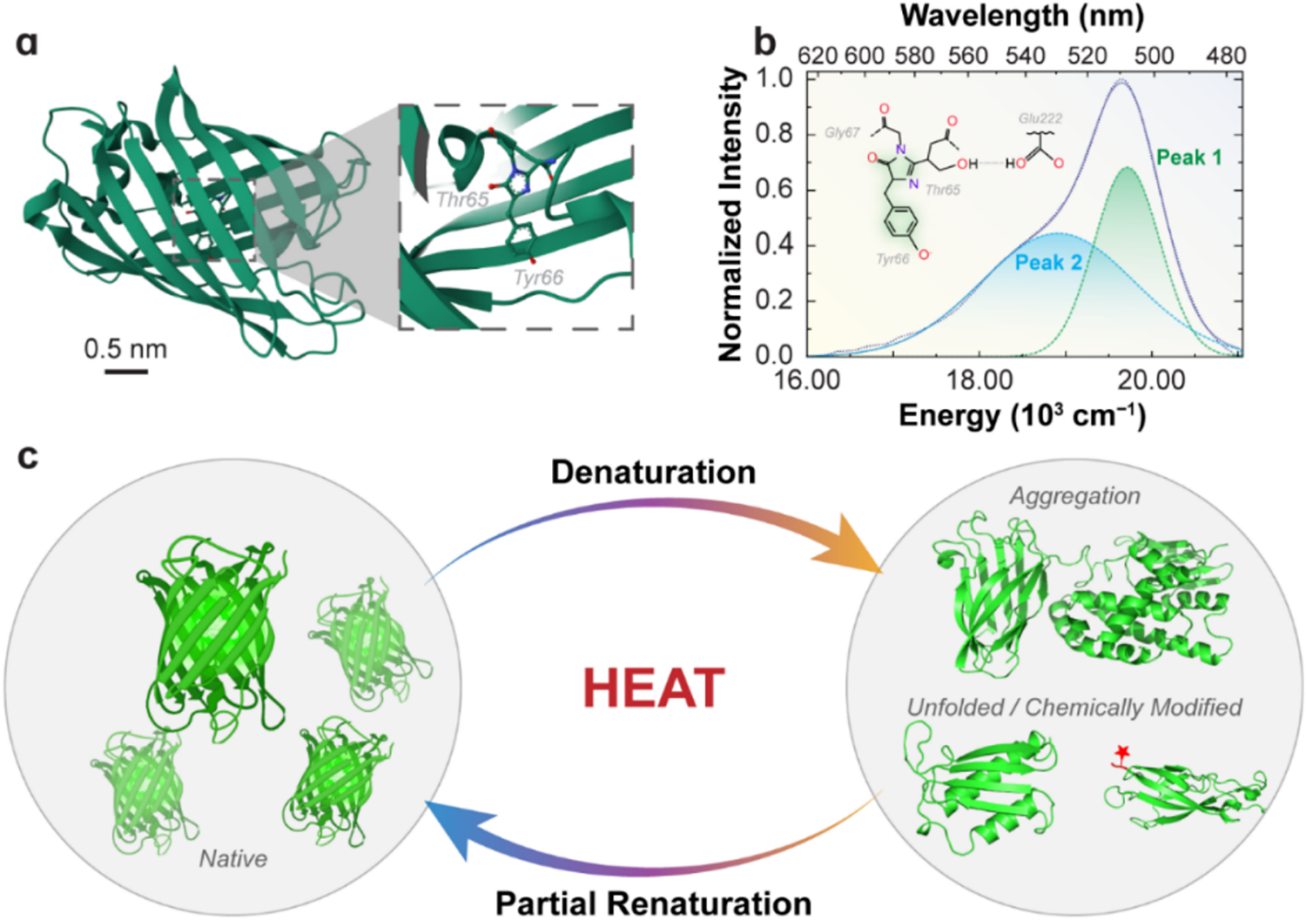
(a) The 3D scaled representation of the EGFP structure based on the Protein Data Bank. The magnification presents the EGFP chromophore, downloaded from the RCSB PDB (RCSB.org) of 4EUL. (b) Emission spectrum of a 0.71 μM EGFP aqueous suspension at 25.0 °C with deconvolution into two Gaussian components (peak 1, green area, and peak 2, blue area). The solid lines represent the fitted envelopes (*R* ^2^ > 0.996). The inset presents the anionic form of the EGFP chromophore with the corresponding amino acid residues (Gly67, Thr65, Tyr66, and Glu222). (c) Schematic illustration of EGFP thermal unfolding, aggregation, and partial refolding.

The F64L mutation improves folding efficiency at 37 °C, while threonine at position 65 (Thr65) facilitates folding and promotes the ionization of the adjacent glutamic acid residue at position 222 (Glu222), thereby increasing thermal stability.^43,44^ These features result in a higher fraction of correctly folded proteins and, consequently, higher fluorescence intensity.^44^

EGFP emits visible fluorescence through a sophisticated mechanism that relies on the intrinsic properties of its chromophore and the finely tuned protein environment.^44,45^ Recent studies suggest that upon light absorption, the chromophore, initially in a neutral state, undergoes excited-state proton transfer, rapidly converting into its anionic form (inset of Figure 1b).^46^ This proton transfer is facilitated by the hydrogen bonds within the β-barrel, with Thr65 and Glu222 residues playing critical roles in stabilizing the deprotonated state.^44^ The resultant anionic chromophore exhibits red-shifted emission with high fluorescence quantum yield and thermal stability.^46^ The emission characteristics of EGFP are governed not only by the chemical structure of the chromophore but also by dynamic interactions with the surrounding protein matrix, which collectively fine-tune the energy landscape for efficient fluorescence.^44,45^ Figure 1b presents the emission spectrum of 0.71 μM (0.43 × 10^21^ proteins per m^3^, Table S1, Supporting Information) EGFP suspension, characterized by a broad peak around 510 nm (peak 1) and a shoulder at 540 nm (peak 2), both attributed to the anionic form.^6,47^ For the other concentrations, the emission is similar as EGFP is highly resistant to concentration quenching, even at concentrations as high as ∼10^24^ proteins per m^3^,^48^ more than 3 orders of magnitude above our experimental conditions. This resilience arises from EGFP’s β-barrel structure.

### 3.2 Fluorescence Temperature Dependence and Melting Temperature Determination

In proteins, the melting temperature (*T*_m_) defines the point at which half the protein population is unfolded; above this transition, aggregation, misfolding, and chemical modifications occur.^49–51^ While primarily dictated by amino acid sequence,^52^ thermal denaturation can shift depending on protein concentration and heating rate.^53^ *T*_m_ is traditionally measured by calorimetric and spectroscopic methods or light scattering,^49,50^ although fluorescence quenching has also been employed (Figure S11, Supporting Information).^52^

For EGFP, heating disrupts the hydrogen bonds that stabilize the chromophore, exposing it to solvent interactions that promote irreversible unfolding (Figure 1c).^41,51^ This destabilization reduces fluorescence intensity and induces a redshift in peak energy (Figure 2). The spectral shift arises from a dipolar charge density redistribution between the phenolic and imidazolinone groups of the chromophore, which alters its dipole moment and reorganizes the surrounding solvent, lowering emission energy.^52^ Partial refolding after cooling is supported by dynamic light scattering and absorbance measurements of samples heated near *T*_m_ and returned to room temperature (Figures S2 and S7a, Supporting Information).

**Figure 2:**
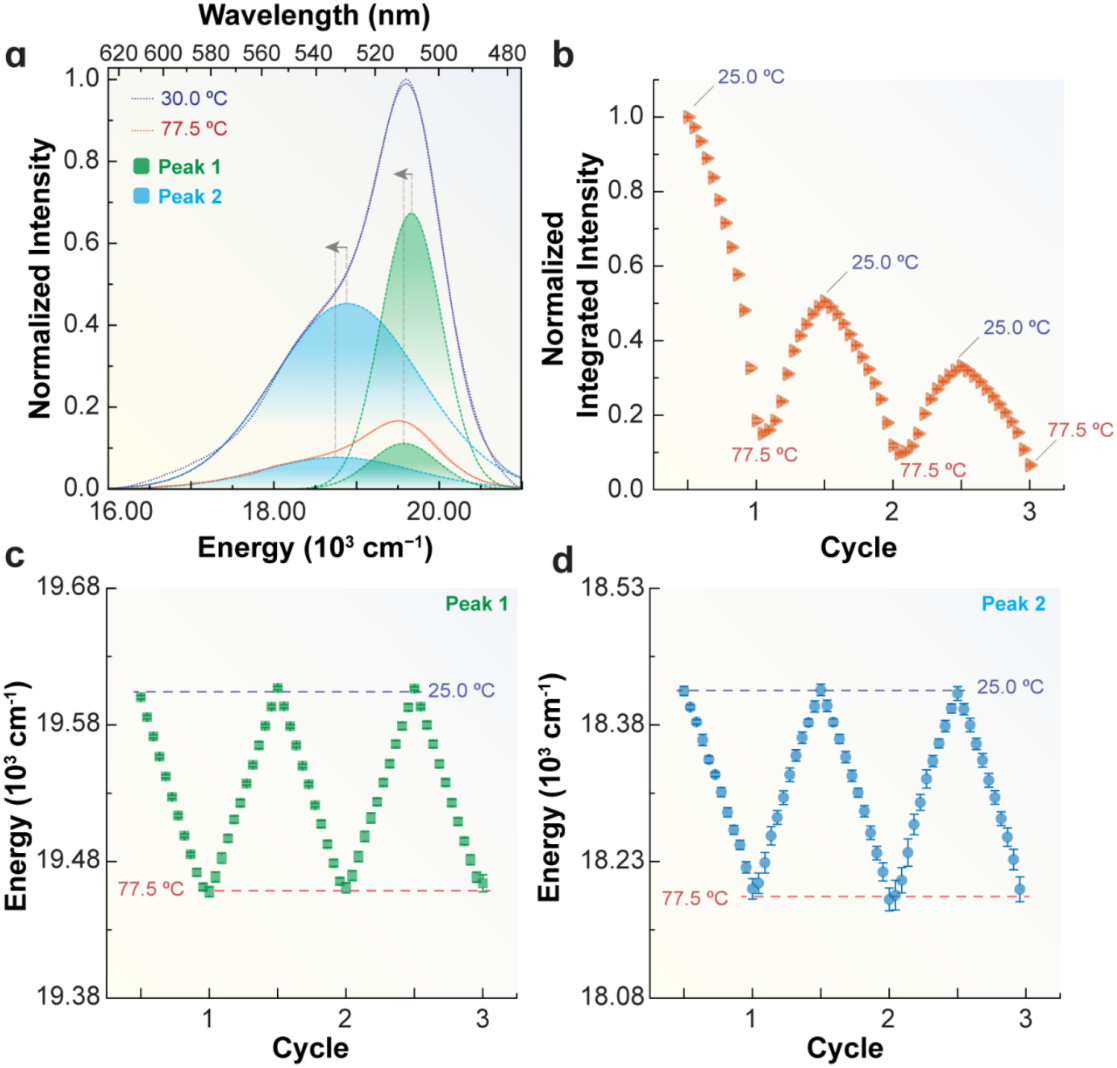
(a) Emission spectra of a 2.86 μM EGFP aqueous suspension at 30.0 and 77.5 °C deconvoluted into two Gaussian components (peak 1, green area, and peak 2, blue area). The solid lines represent the fitted envelopes (*R* ^2^ > 0.997). Arrows indicate the redshift of the peak energies as the temperature increases. (b) Integrated emission intensity and (c) peak 1 and (d) peak 2 energies upon heating–cooling cycles. The repeatability of both peaks is 99.98% at 25.0 °C (Table S5, Supporting Information).

We estimated *T*_m_ by monitoring EGFP fluorescence during heating–cooling cycles (25.0–95.0 °C). For a 1.43 μM EGFP suspension, intensity decreased progressively during heating (orange regions), with only partial recovery on cooling (blue regions), Figure 3a. Recovery efficiency declined as the maximum temperature increased (Figures 3b and S12, Supporting Information).

**Figure 3:**
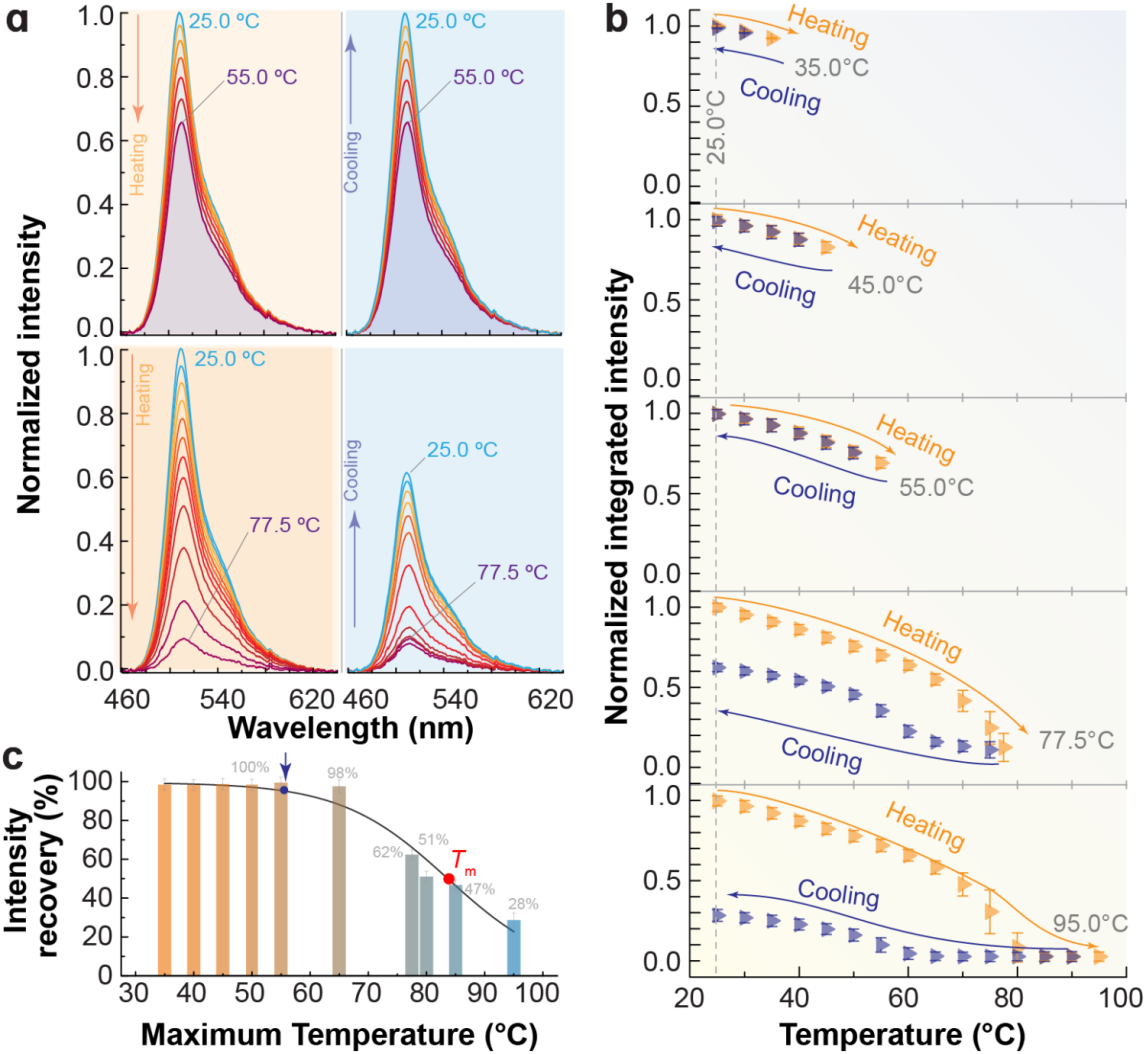
(a) Temperature-dependent emission spectra of a 1.43 μM EGFP suspension during heating and cooling. (b) Normalized emission integrated intensity (475–625 nm) over heating–cooling cycles; recovery decreases with increasing maximum temperature. The points correspond to the mean value of the emission integrated area across the 100 recorded spectra, while the error bars represent the corresponding standard deviation. (c) Fluorescence intensity recovery fraction after cycles up to 95.0 °C fitted with a Boltzmann function (*r* ^2^ > 0.962, Table S3, Supporting Information).

Quantitative analysis (Figure 3c) shows that recovery is complete only up to 56 ± 3 °C, which we define as the onset of unfolding (blue arrow in the figure); above this point, irreversible changes reduce recovery to ∼28% at 95.0 °C. Fitting with a sigmoidal function yields *T*_m_ = 84 ± 1 °C (Table S3, Supporting Information). At higher concentration (2.86 μM), the onset shifts slightly to 55 ± 2 °C and *T*_m_ decreases to 78 ± 1 °C (Figures S13, S14 and Table S3, Supporting Information). Circular dichroism corroborates these values, *T*_m_ = 79 ± 1 °C (Figure S5 and Table S2, Supporting Information).

All measured values agree with literature for EGFP (80.3 °C^54^) and GFP (76 °C^55^ and 78 °C,^56^ Figure S11, Supporting Information), validating fluorescence quenching as a reliable method for probing protein thermal stability.

### 3.3 Luminescence Thermometry and Temperature Dependence of the EGFP Brownian Velocity

The temperature-dependent fluorescence of GFP^57,58^ and EGFP^6,59^ makes these proteins attractive candidates for thermal sensing. We previously demonstrated that luminescence thermometry with EGFP can be performed by analyzing the energy, width, and integrated area of its two emission peaks individually or through a multiparametric approach.^59^ However, that method was validated only within a limited temperature range below *T*_m_ (25.0–50.0 °C). In this study, we extend the temperature window to 70.0 °C by focusing on the energy of peak 1. Beyond its straightforward numerical analysis, this parameter remains robust across repeated heating–cooling cycles, regardless of the maximum temperature reached (up to 77.5 °C) or EGFP concentration (Figures 2c,d and S15, Supporting Information), unlike the peak area, which varies significantly above 55.0 °C (Figure 3c).

While fluorescence cross-correlation spectroscopy^60,61^ and microparticle-tracking velocimetry^62^ are widely used to probe protein dynamics, their applications have typically been limited to near-ambient temperatures. This limitation is critical, as temperature is a key factor influencing protein function and folding (as discussed previously). In our previous work,^6^ we demonstrated the temperature dependence of EGFP protein Brownian velocity across physiological temperatures (30.0–50.0 °C). Here, we extend these measurements to 70.0 °C to explore the fluctuations between LD and HD motifs in the hydration shell of EGFP (through the framework of the two-state model of liquid water^11–13^ ) and its connection to protein stability. We adapted our previously established technique – originally developed for sub-100 nm colloidal NaYF_4_:Yb^3+^/Er^3+^ upconverting nanoparticles in both water and organic solvents^23,24,63^ – to analyze EGFP aqueous suspensions at distinct concentrations (0.36 to 3.57 μM).

Our results align closely with those reported by Di Rienzo et al.^60^ for GFP in water (0.10 μM) and within the cytoplasm of CHO-K1 cells at 37.0 °C using fluorescence cross-correlation spectroscopy, as previously noted.^6^ As shown in Figure 4a, the Brownian velocity increases linearly with temperature, consistent with earlier findings for NaYF_4_:Yb^3+^/Er^3+^ upconverting nanoparticles^23,24,64^ and EGFP aqueous suspensions.^6^ The increase of the Brownian velocity with increasing temperature aligns with the predictions of the Stokes–Einstein–Sutherland theory in the diffusive regime.^65^

**Figure 4:**
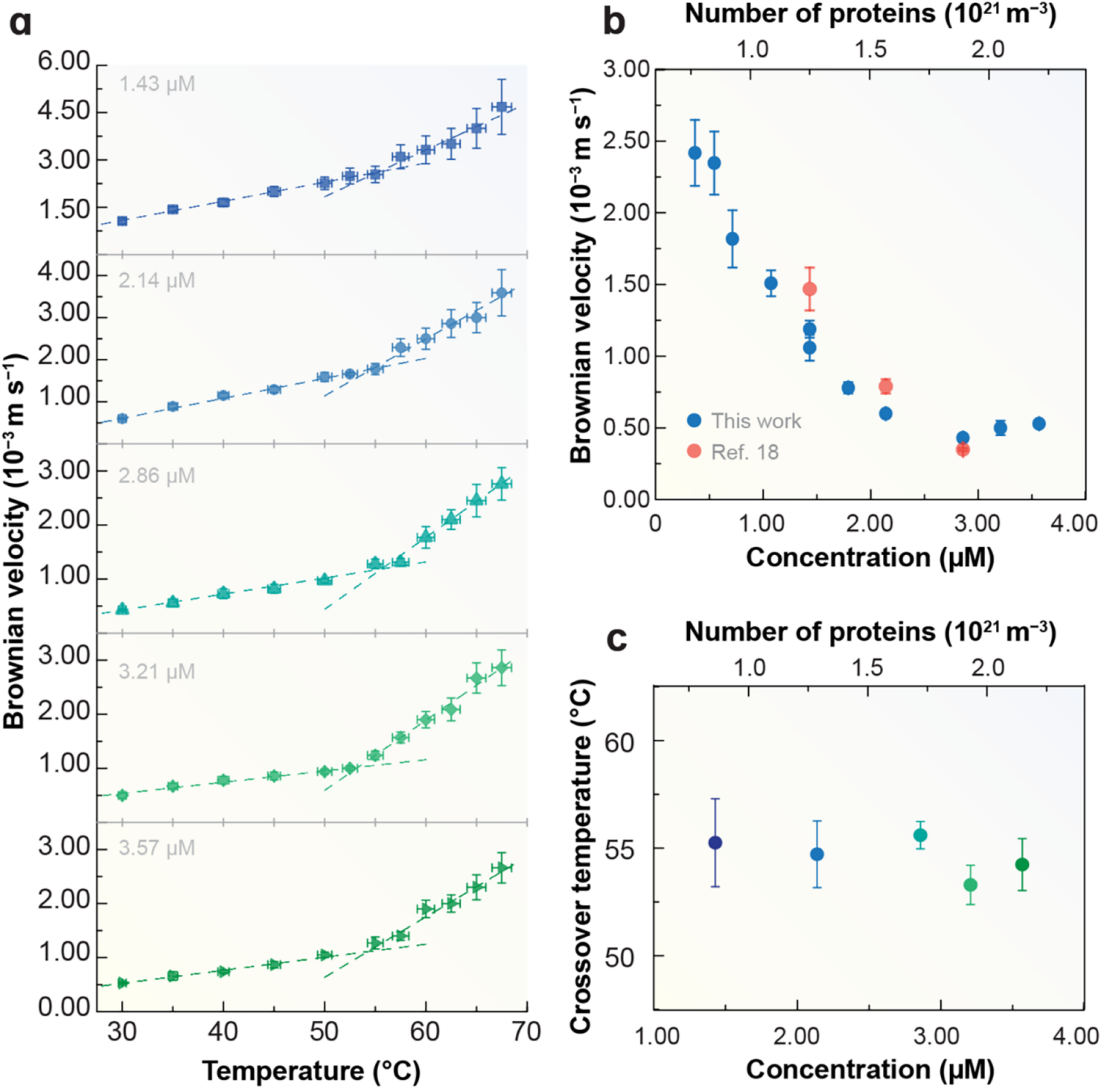
(a) Temperature-dependent Brownian velocity of EGFP in aqueous suspensions (1.43–3.57 μM). The horizontal and vertical error bars were calculated as discussed in Sections 9 and 10, Supporting Information. (b) Brownian velocity at 30.0 °C across concentrations from 0.36 to 3.57 μM, including data (red circles) from our previous work^6^ for comparison. (c) Crossover temperature extracted from bilinear fits, independent of EGFP concentration (Table S8 and Section 10, Supporting Information).

Figure 4b shows the Brownian velocity at 30.0 °C for EGFP concentrations ranging from 0.36 to 3.57 μM. Expanding our previous data set (1.43, 2.14, and 2.86 μM),^6^ we observe consistent results: velocity remains constant between 0.36 and 0.54 μM at (2.4 ± 0.2) × 10^–3^ m s^–1^, then decreases to (0.43 ± 0.02) × 10^–3^ m s^–1^ as the concentration increases to 2.86 μM, before stabilizing around (0.53 ± 0.02) × 10^–3^ m s^–1^ for concentrations up to 3.57 μM. The decrease of Brownian velocity values with increasing EGFP concentration aligns with previous findings by Di Rienzo et al.^60^ and Baum et al.^61^ and is attributed to protein–protein interactions.

More intriguingly, we observe a bilinear temperature dependence of Brownian velocity that is independent of EGFP concentration between 1.43 and 3.57 μM (Figure 4a). At lower concentrations, the low signal-to-noise ratio and its quenching with increasing temperature preclude reliable measurements. For example, at 0.71 μM, peak 1 energy cannot be accurately measured above 60.0 °C (Figure S16, Supporting Information). Additionally, the required multiple heating–cooling cycles to identify the onset temperature from the redshift of peak 1 accelerate EGFP degradation, preventing data collection above 60.0 °C (Figure S17, Supporting Information).

### 3.4 Density Fluctuations in EGFP First Hydration Shell

Bilinear thermal responses have been observed in a range of aqueous systems, including quantum dots,^19^ plasmonic^66^ and luminescent^67^ nanoparticles, organic dyes,^22,68^ and lanthanide-based materials,^23,24,64,69^ with crossover temperatures (*T*_c_) typically between 45 and 65 °C. This range coincides with the isothermal compressibility minimum of water,^70^ suggesting a structural origin. Within the two-state model, we have previously interpreted *T*_c_ as marking the onset of fluctuations between HD and LD motifs upon cooling.^23,24^ Below *T*_c_, transient LD motifs emerge within the predominant HD liquid motifs, increasing the effective protein–solvent hydrodynamic mass, reducing mobility; above *T*_c_, the progressive loss of LD motifs yields a more homogeneous solvent and enhanced diffusion.

For EGFP, Brownian velocity measurements revealed a clear bilinear dependence with *T*_c_ = 55 ± 2 °C (Figure 4c), independent of concentration across 1.43–3.57 μM. This concentration invariance confirms that the crossover arises from intrinsic solvent structure properties rather than macromolecular effects. The measured *T*_c_ also agrees with previous estimates for hydration-water fluctuations in lysozyme,^10,29,32^ underscoring the generality of the phenomenon.

Isotopic substitution further modulates this balance. In D_2_O (2.86 μM), EGFP unfolding onset shifts upward (64 ± 2 °C vs 55 ± 2 °C in H_2_O) and the crossover temperature increases to 65 ± 2 °C (Figure 5, and Table S8, Supporting Information). Circular dichroism similarly indicates a higher melting temperature in D_2_O (83 ± 1 °C vs 79 ± 1 °C in H_2_O, Figures S5, S6 and Table S2, Supporting Information). Together, these results show that LD motifs persist to higher temperatures in heavy water, delaying HD dominance and enhancing hydration-shell stability. This isotopic effect is consistent with the displacement of the Widom line and the more ordered hydrogen-bond network reported for D_2_O.^71^

**Figure 5:**
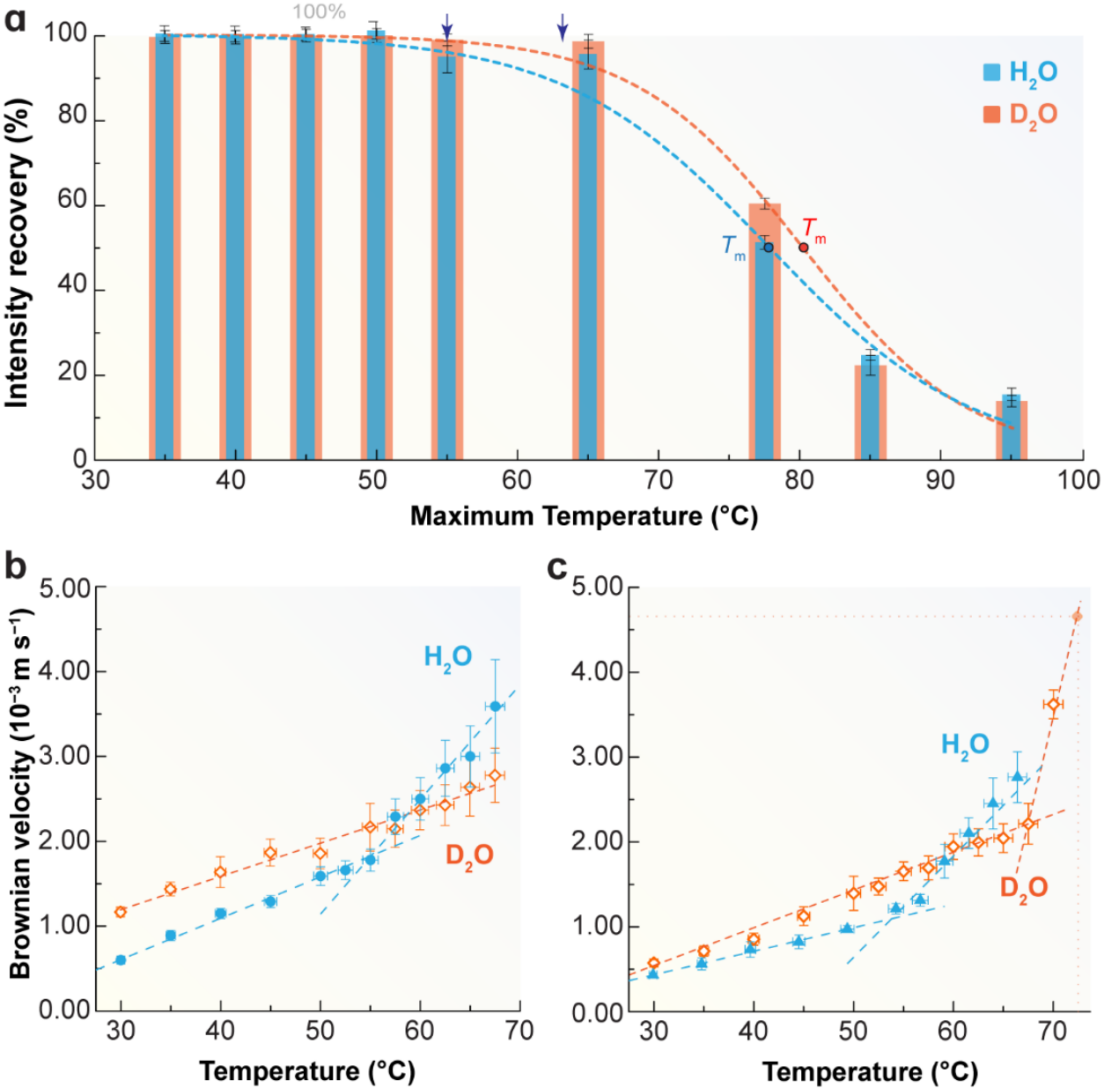
(a) Fluorescence intensity recovery (2.86 μM) after heating–cooling cycles in H_2_O and D_2_O (Figures S13, S20, Supporting Information). The lines are the best fit to the data using Boltzmann functions (*r* ^2^ > 0.988, Tables S3, S4, Supporting Information). The points mark the melting temperature *T*_m_, while the arrows mark the onset of protein unfolding. Temperature-dependent Brownian velocity in H_2_O and D_2_O for (b) 2.14 μM and (c) 2.86 μM. Dashed lines are the best linear fits to the data (*R* ^2^ > 0.944, Table S8, Supporting Information) used to determine *T*_c_.

This interpretation aligns with pioneering studies by Mallamace and co-workers, who examined the connection between hydration water and protein stability using calorimetry, neutron scattering, NMR, and Fourier transform IR spectroscopy.^10,72,73^ Their studies on lysozyme hydration water demonstrated that protein flexibility and unfolding limits are dictated by the lifetime and strength of the hydrogen-bond network: hydration water comprises a mixture of LD and HD motifs up to ∼315 K, above which hydrogen-bond lifetimes shorten and unfolding begins, progressing to irreversible denaturation beyond ∼345 K, when the hydration shell is entirely composed of HD motifs. These results established that the loss of LD motifs coincides with the disappearance of stabilizing hydration interactions.^10,72,73^

## 4 Conclusions

We have demonstrated that EGFP serves as a sensitive probe for hydration-shell dynamics and protein stability in both H_2_O and D_2_O. Fluorescence quenching during heating–cooling cycles revealed that unfolding begins around 55 °C in H_2_O and 64 °C in D_2_O, while circular dichroism confirmed higher melting temperatures in heavy water (83 °C vs 79 °C). EGFP Brownian velocity measurements, through luminescence thermometry, uncovered bilinear thermal behavior with crossover temperatures of 55 °C in H_2_O and 65 °C in D_2_O, consistent with fluctuations between LD and HD water motifs. These results provide direct experimental validation of hydration-water fluctuations – specifically the persistence of LD motifs – govern protein thermal stability, with isotopic substitution extending the stability window. Beyond fundamental validation, this approach can be generalized to other fluorescent proteins and applied to the design of thermally robust protein-based sensors and probes, extending its relevance from biophysical studies to practical biotechnological applications.

## Supplementary Material





## Notes

Calculation of EGFP molecule numbers; colloidal stability and characterization; temperature-dependent circular dichroism and absorption spectra; details of the optical setup and source stability; additional analysis of fluorescence and thermometric parameters; methods for determining Brownian velocity and crossover temperatures (PDF)
A data set of time-dependent peak 1 energies as a function of temperature and EGFP concentration (PDF)
