# Supplementary material for: Exploring Water Beyond the Solvent: Insights into Density Fluctuations and EGFP Unfolding via Luminescence Thermometry
Source: J Phys Chem B. 2025 Nov 6;129(46):12042–50. doi: 10.1021/acs.jpcb.5c06143 (PMC12772516; doi:10.1021/acs.jpcb.5c06143)
Supplement: Supplementary file 1 [file jp5c06143_si_001.pdf]

Supporting Information

Exploring Water Beyond the Solvent: Insights into Density Fluctuations and EGFP Unfolding via Luminescence Thermometry

Yongwei Guo, Fernando E. Maturi, Ramon S. Raposo Filho, Carlos D. S. Brites,* Luís D. Carlos*

Phantom-g, CICECO–Aveiro Institute of Materials, Physics Department, University of Aveiro, 3810-193, Aveiro, Portugal

*Corresponding authors. E-mail: carlos.brites@ua.pt; lcarlos@ua.pt

Contents

1. Number of EGFP molecules 2
2. Colloidal characterization 3
3. Circular dichroism 6
4. Absorption spectra of EGFP aqueous suspensions 9
5. Experimental setup and stability of the excitation source 10
6. Emission features of EGFP aqueous suspensions 11
   6.1. Lineshape Correction and Spectral Deconvolution 11
   6.2. Reproducibility 11
   6.3. Photostability 11
   6.4. Thermal denaturation: Literature data 12
   6.5. Temperature dependence: Emission intensity 13
   6.6. Temperature dependence: Emission peak energy 16
   6.7. Emission quenching at low concentrations 17
7. Optical features of EGFP in D_2O 18
8. Thermometric features 20
   8.1. Repeatability 20
9. Brownian velocity of EGFP 22
   9.1. Uncertainty in the thermocouple measurements 34
   9.2. Reproducibility 35
10. Determination of crossover temperature 36
11. References 37

1. Number of EGFP molecules

The number of EGFP molecules (N) in water (H_2O) and heavy water (D_2O) was calculated based on the mass concentration (m) using:

$$N = \frac{m}{MN_A} \quad \text{(S1)}$$

where M is the molecular weight of EGFP ($M = 28$ kDa = 28 kg mol^{-1}) and N_A is the Avogadro's constant ($N_A = 6.022\times10^{23}$ mol^{-1}). The calculated number of EGFP molecules is presented in **Table S1**.

Table S1. Conversion from mass concentration to the molar concentration and number of EGFP molecules in both H_2O and D_2O suspensions.

Mass concentration (10^{-3} kg m^{-3})	Molar concentration (μM)	Number of proteins per m^3 ($\times10^{21}$)
10	0.36	0.22
15	0.54	0.32
20	0.71	0.43
30	1.07	0.65
40	1.43	0.86
50	1.79	1.1
60	2.14	1.3
80	2.86	1.7
90	3.21	1.9
100	3.57	2.2

2. Colloidal characterization

The hydrodynamic diameter (d_h) and zeta potential (ζ) of EGFP in H_2O and D_2O were measured using a Malvern Zetasizer Nano ZS equipment (ZEN3600, Malvern Instruments) operating with a 632.8 nm laser. Temperature-dependent measurements of d_h were performed with a 1 °C min^{-1} heating rate in a folded capillary cell (DTS1070, Malvern Instruments). Each measurement consisted of three runs, each with twelve scans, and the resulting size distributions and zeta potentials were fitted to log-normal and Gaussian functions, respectively. The mean values and standard deviations were used to determine the average and uncertainty values, respectively.

The hydrodynamic diameter distribution of EGFP in water, at concentrations ranging from 0.71 to 3.57 μM, was measured at 25.0 °C both in fresh samples and after Brownian velocity measurements. These measurements involved temperature increases to 77.5 °C and multiple heating and cooling cycles between 25.0 and 77.5 °C (**Figure S1**). Statistical analysis revealed no significant correlation between hydrodynamic diameter and either concentration or temperature. The d_h of an illustrative example of EGFP in H_2O during heating and cooling cycles and ζ values of aqueous suspensions of EGFP at different concentrations are shown in **Figure S2** and **Figure S3**, respectively. The increase in the EGFP hydrodynamic diameter (**Figure S2)** is attributed to protein conformational changes, resembling those observed in human TRPM4 structures.[1]

EGFP was dispersed in D_2O at two illustrative concentrations (2.14 and 2.86 μM). **Figure S4** presents the values of d_h and ζ of the 2.86 μM suspension in D_2O.

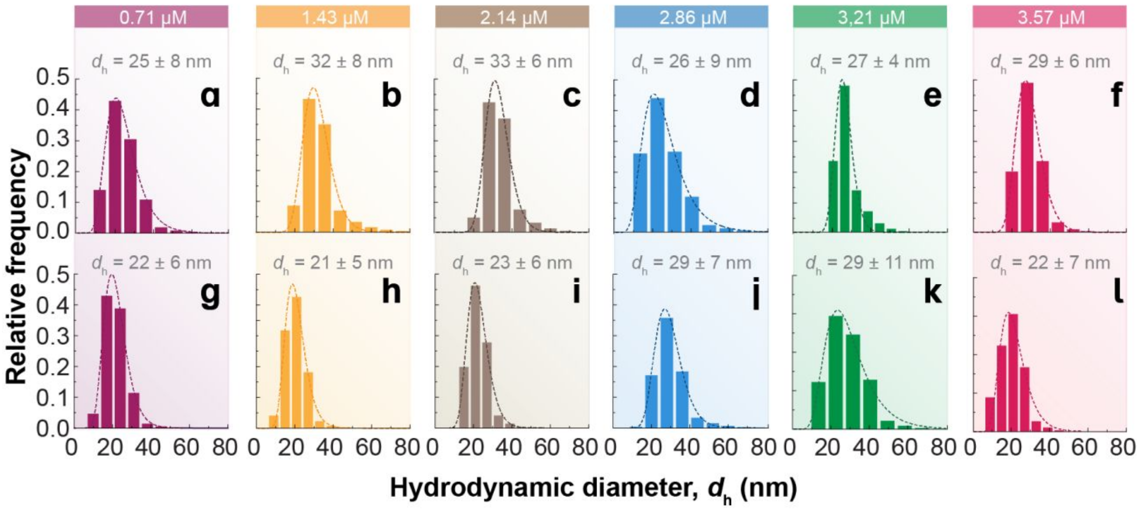

Figure S1. Hydrodynamic diameter of EGFP in the studied aqueous suspensions measured at increasing concentrations (indicated in each panel). (a)−(f) Fresh samples. (g)−(l) Samples after Brownian velocity measurements. The interrupted lines represent the best fit to the experimental data using log-normal functions ($r^2 > 0.975$).

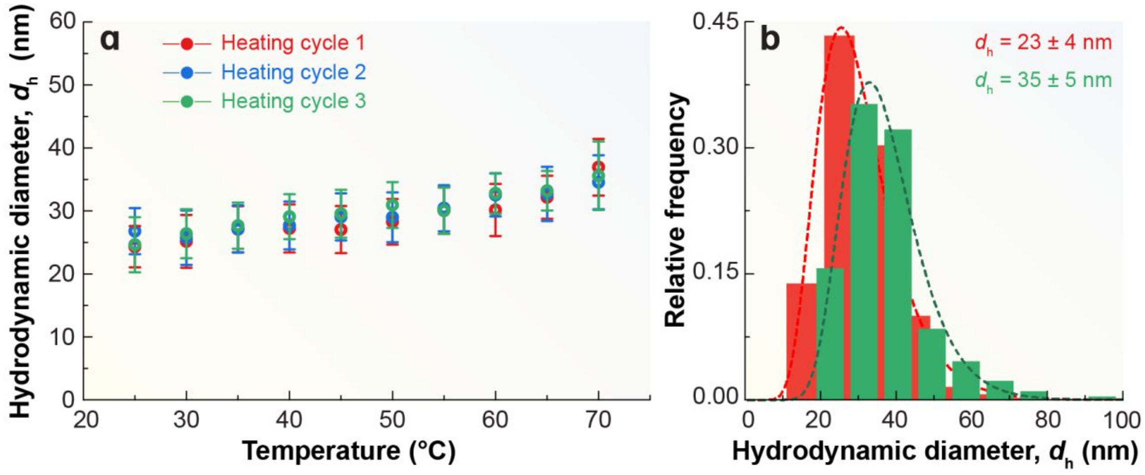

Figure S2. (a) Hydrodynamic diameter of EGFP measured in the 0.71 μM aqueous suspension in three heating/cooling cycles (25.0−70.0 °C). (b) Size distributions of cycle 1 (red) at 25.0 °C and cycle 3 (green) at 70.0 °C. The interrupted lines represent the best fit to the experimental data using log-normal functions ($r^2 > 0.980$).

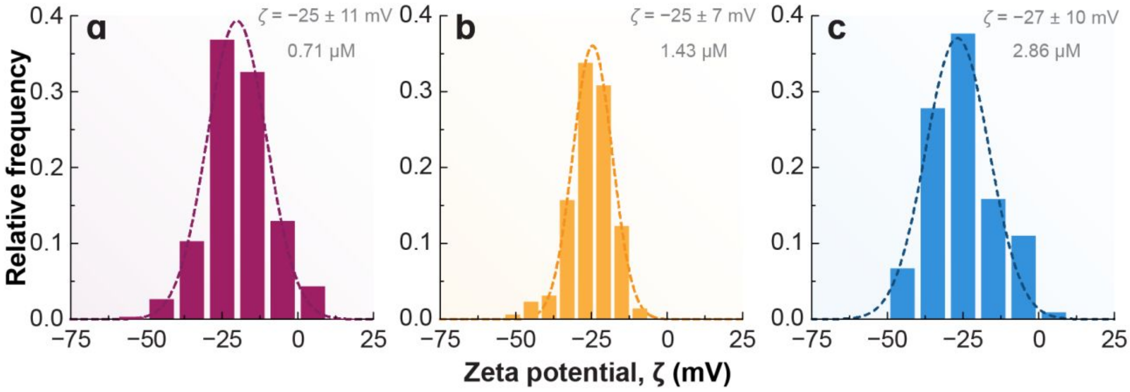

Figure S3. Zeta potential of (a) 0.71 μM, (b) 1.43 μM, and (c) 2.86 μM EGFP aqueous suspensions measured at room temperature. The interrupted lines represent the best fit to the experimental data using Gaussian functions ($r^2 > 0.960$).

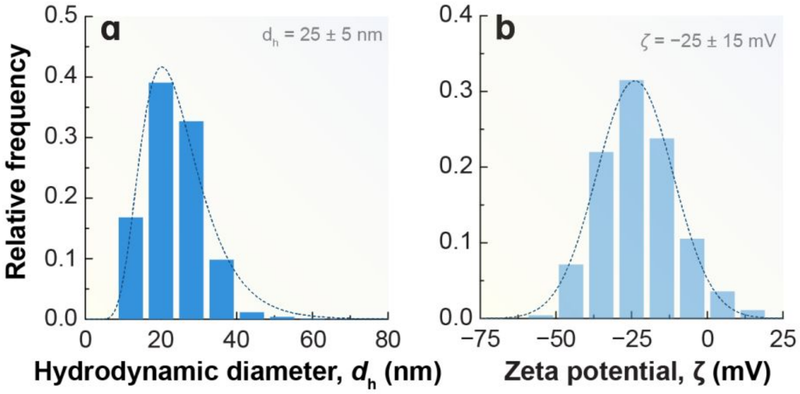

Figure S4. (a) Hydrodynamic diameter and (b) zeta potential of 2.86 μM EGFP in D_2O measured at room temperature. The interrupted lines represent the best fit to the experimental data using log-normal functions ($r^2 > 0.972$) and Gaussian functions ($r^2 > 0.994$), respectively.

The hydrodynamic diameter of EGFP measured in our experiments exceeds its molecular dimensions (4.2 × 2.4 nm), in agreement with several previous reports (Table S2). This discrepancy arises because the hydrodynamic radius reflects the effective size of the protein in solution, including contributions from the hydration shell, solvent–ion interactions, and conformational fluctuations, rather than its geometric size. Additionally, the spherical approximation used in hydrodynamic analyses may overestimate the radius of the β-barrel-shaped EGFP. Partial dimerization under the experimental conditions could further increase the apparent size.[2, 3]

Table S2. Reported hydrodynamic diameters (*d*□) of various EGFPs measured under different experimental conditions, including temperature (*T*) and concentration (*c*).

d_h (nm)	*T* (ºC)	*c* (µM)	Measurement Conditions	Equipment Information	Ref.
10	37	0.1	150 mM NaCl, 5 mM PBS (pH=7.4)	Malvern Zetasizer Nano ZS, (φ=173°).	4
2.2±0.6	n.r.	0.05	n.r.	ALV instrument (φ=90°).	5
3.6±0.2	5	5−10	50 mM PBS, 0.1 M NaCl (pH=8.0)	Malvern Zetasizer Nano-S (φ=173°)	6
6 ± 1	55				
9.35	25	10	0.5 M imidazole, 0.05 M NaH_2PO_4, and 0.1 M NaCl (pH=8.0).	ALV/DLS/SLS-5022F photon correlation spectrometer (φ=90°).	7
25 ± 5	25	0.71−3.57	Water (pH=7.2-7.7)	Malvern Zetasizer NanoZS (φ=173°).	*This work*

n.r. – not reported
φ - detection angle

3. Circular dichroism

Far-UV CD spectroscopy (190–250 nm) was selected because it primarily reflects changes in secondary structure through peptide-bond absorption, providing high sensitivity to β-sheet disruption during EGFP unfolding.[8, 9] Although CD in the visible region (250–500 nm) can probe tertiary structure and chromophore environment, its signals are typically much weaker and require higher protein concentrations or longer pathlengths.[10, 11] Given the predominantly β-barrel structure of EGFP and the experimental constraints, far-UV CD represents the most direct and informative method for monitoring its unfolding process.

Circular dichroism (CD) was employed to monitor the EGFP thermal unfolding in H_2O and D_2O suspensions (2.86 μM). Temperature-dependent CD measurements were conducted on a spectropolarimeter (J-1500, Jasco) equipped with a 150 W Xe lamp. A continuous nitrogen purge (10 L min^{-1}) was used throughout to protect the optical components and maintain baseline stability across the measured wavelength range (200–250 nm). Spectra were recorded with a spectral resolution of 0.5 nm and an integration time of 4.0 s per point. Temperature control was achieved using a single-position Peltier thermostatted cell holder (PTC-517, Jasco), with a step of 2.0 °C and a heating rate of 2.0 °C min^{-1} from 25 to 95 °C.

After removing the blank signal of the solvent and quartz cuvette (100-2-40, Hellma Analytics, pathlength L = 0.4 cm), the raw CD data (in 10^{-3} deg) were converted to units of mean residue ellipticity (θ, in degrees squared centimeter per dmol) using:[9, 12]

$$[\theta] = \frac{[\text{CD data}]}{CnL} \qquad \text{(S2)}$$

where $C = 2.9\times10^{-3}$ mol m^{-3} is the concentration of EGFP, and n = 238 represents the number of EGFP residues.

The temperature dependence of θ and the corresponding thermal denaturation curve, obtained at 215 nm, characteristic of the dominant β-sheet structure of EGFP,[13] are shown in **Figure S5** (H_2O) and **Figure S6** (D_2O).

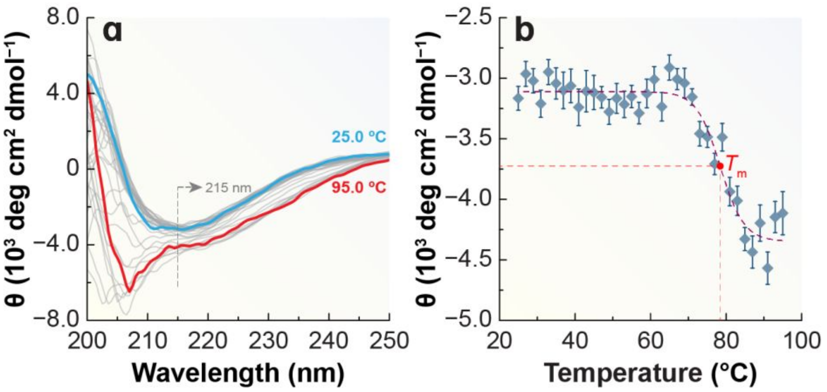

Figure S5. (a) Temperature dependence of the mean residue ellipticity θ of EGFP in H_2O at 2.86 µM. The dashed line indicates the minimum θ value at 215 nm. (b) Thermal evolution of θ (at 215 nm). The dashed line is the best fit to the data (diamonds) using **Eq. S3** (**Table S2**). The red point marks the melting temperature T_m.

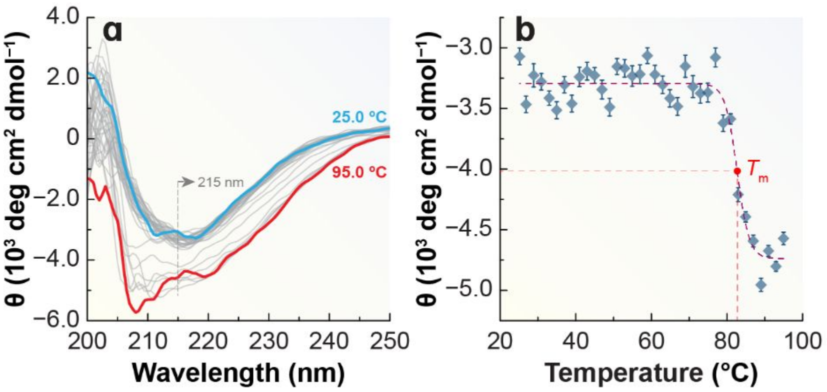

Figure S6. (a) Temperature dependence of the mean residue ellipticity θ of EGFP in D_2O at 2.86 µM. The dashed line indicates the minimum θ value at 215 nm. (b) Thermal evolution of θ (at 215 nm). The dashed line is the best fit to the data (diamonds) using **Eq. S3** (**Table S2**). The red point marks the melting temperature T_m.

The mean residue ellipticity $[\theta]$ of EGFP in H_2O and D_2O were fitted to a sigmoidal Boltzmann function, using the OriginLab® software:

$$[\theta]\,(T) = [\theta_{min}] + \frac{[\theta_{max}] - [\theta_{min}]}{1 + \exp\left(\frac{T - T_m}{\Gamma_\theta}\right)} \qquad (\text{S3})$$

where $[\theta_{min}]$ and$[\theta_{max}]$ are the minimum and maximum values of $[\theta]$, Γ_θ is a temperature range characteristic of the protein, and T_m is the melting temperature of the protein (corresponding temperature of the inflection point). The parameter Γ_θ governs the steepness of the thermal transition: smaller values indicate sharper transitions around T_m, while larger values correspond to more gradual changes in $[\theta]$ with temperature.

Table S2. Fitting parameters of $[\boldsymbol{\theta}](\boldsymbol{T})$ (25.0–95.0 °C) of EGFP in H_2O and D_2O (**Figure S5** and **Figure S6**) using **Eq. S3**.

Concentration (μM)	Solvent	Fitting parameter	Value	r^2
2.86	H_2O	$[\theta_{\mathrm{min}}]$	−4.3 ± 0.1	0.912
		$[\theta_{\mathrm{max}}]$	−3.1 ± 0.1	
		T_{m}	79 ± 1	
		Γ_{θ}	3.1 ± 0.7	
2.86	D_2O	$[\theta_{\mathrm{min}}]$	−4.7 ± 0.1	0.959
		$[\theta_{\mathrm{max}}]$	−3.3 ± 0.1	
		T_{m}	83 ± 1	
		Γ_{θ}	1.6 ± 0.4	

4. Absorption spectra of EGFP aqueous suspensions

Absorption spectra were recorded using a UV-Vis spectrophotometer (V-780, JASCO) at room temperature (25.0 °C) with a spectral resolution of 0.5 nm. Measurements were performed in a 10 mm pathlength quartz cuvette (9F-Q-10, Starna Cells).

The absorbance spectra shown in **Figure S7**, discussed in **Figure 1b** of our recent publication,[14] resemble previously reported data.[15] Moreover, small variations on the extinction coefficient of different batches of the same concentration, observed below 300 nm, result from varying solution storage and preparation conditions and suggest subtle alterations in the local environment of EGFP within the aqueous suspension, **Figure S7d**. Additionally, the decrease in the extinction coefficient at 480 nm, ascribed to the anionic chromophore[14], **Figure S7a**, indicates that the temperature-induced structural alterations impact the chromophore environment.

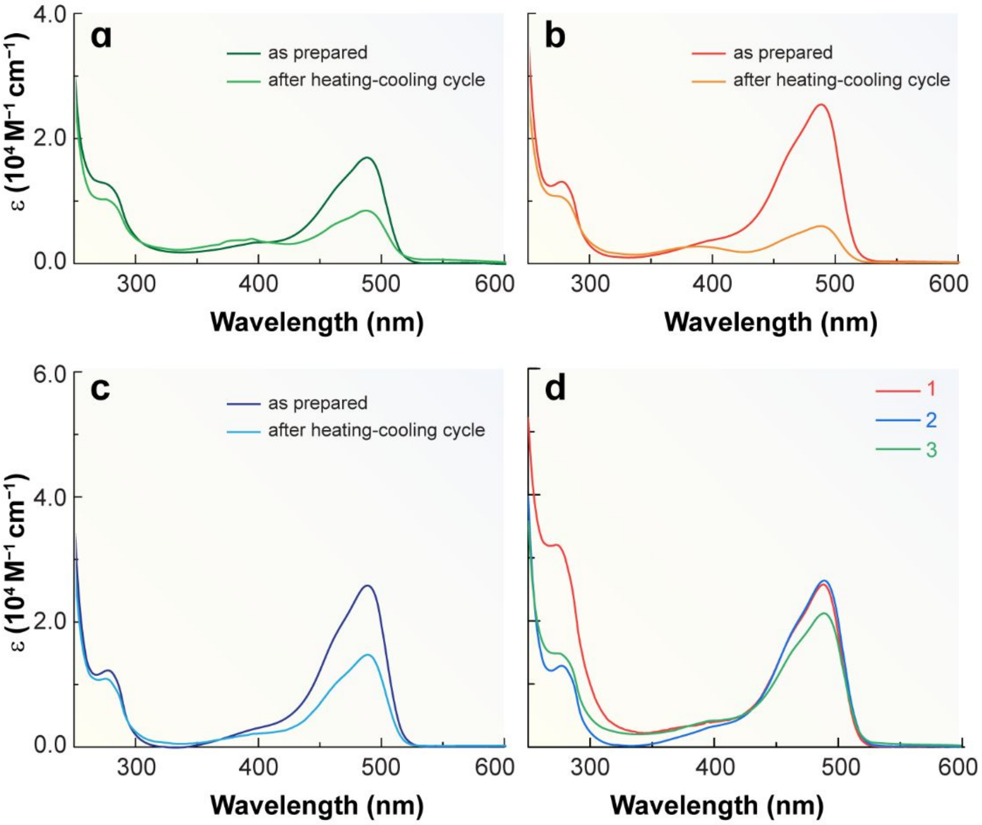

Figure S7. Room temperature absorbance spectra of EGFP aqueous suspensions before and after a heating/cooling temperature cycle for (a) 1.43 μM, (b) 2.86 μM, and (c) 3.57 μM. (d) Absorbance spectra in three distinct EGFP 2.14 μM aqueous suspension batches.

5. Experimental setup and stability of the excitation source

The fluorescent properties of EGFP in both H_2O and D_2O suspensions were studied in the experimental setup shown in **Figure S8a**. For stable performance and optimal working conditions, an LED peaking at 407 nm (**Figure S8b)** was turned on 30 min before starting the measurements (**Figure S8c)** and the beam spot was monitored over 120 min using a CCD beam profiler (BC106VIS/M, Thorlabs). A stable beam size was observed, with a radius of 0.68×10^{-3} m and an area of 1.5×10^{-6} m^2, **Figure S8d** and **S8e**.

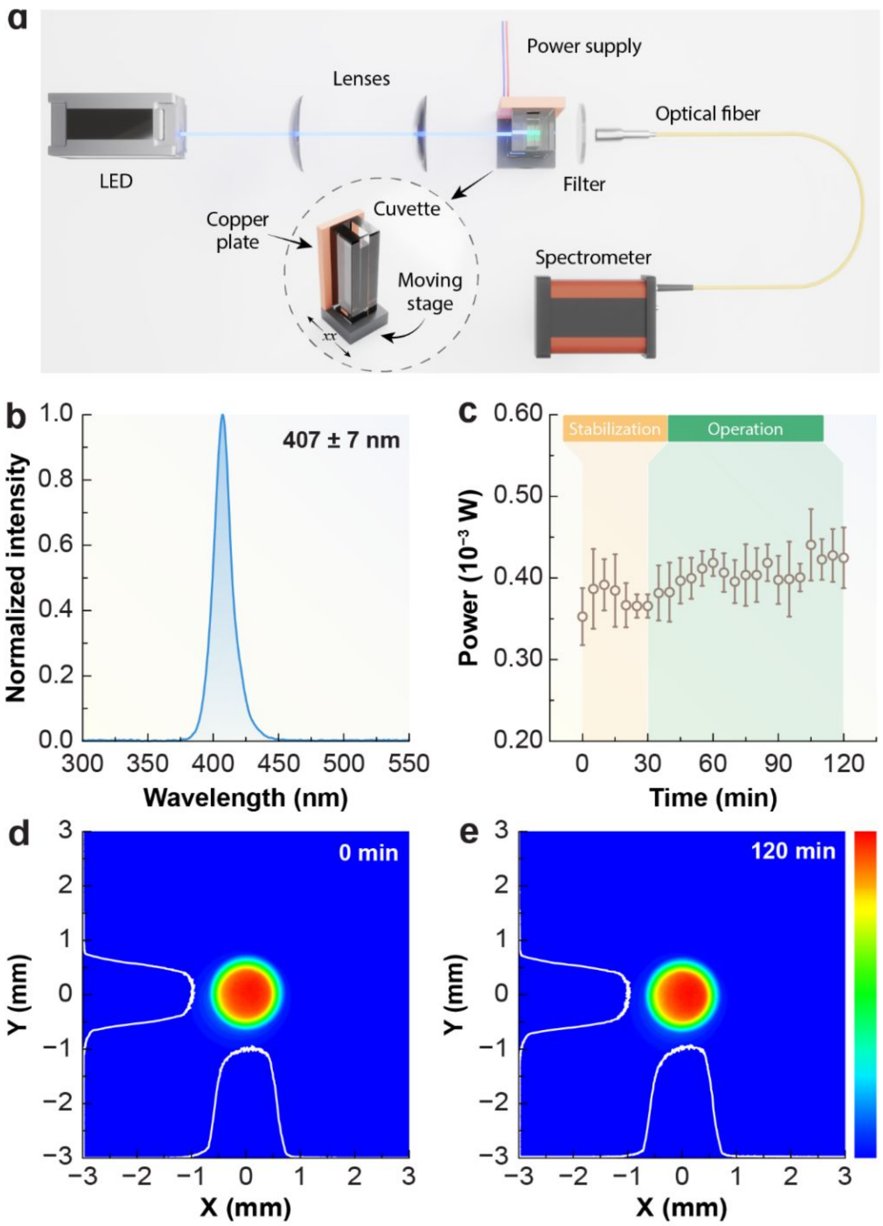

Figure S8. (a) Schematic of the experimental setup used to measure the fluorescent properties and Brownian velocity of EGFP. (b) Emission profile of the LED. (c) Power stability of the LED over time. (d) Beam profile of the LED emission at 0 min and (e) after 120 min of operation.

6. Emission features of EGFP aqueous suspensions

6.1. Lineshape Correction and Spectral Deconvolution

Emission spectra were converted from wavelength (nm) to energy (cm^{-1}) using the Jacobian transformation, as described in our previous work.[14] The corrected spectra were deconvoluted by fitting two Gaussian functions using Matlab®. The peak energies were retrieved from the Gaussian centers, with peaks 1 and 2 corresponding to the higher and lower energy components, respectively.

6.2. Reproducibility

The temperature dependence of the normalized emission intensity and peak 1 energy exhibits negligible variations across different batches for the same concentration, as shown in **Figure S9** for the illustrative example of 2.14 µM.

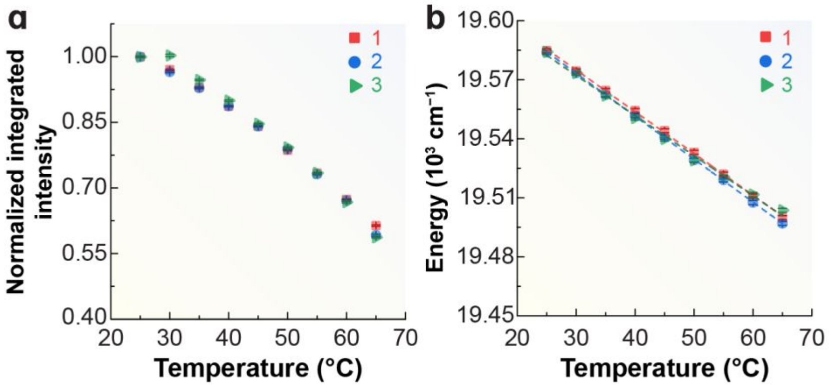

Figure S9. (a) Temperature-dependent normalized emission intensity, and (b) emission peak 1 energy of three distinct batches of 2.14 µM EGFP aqueous suspension. The dashed lines are the best fits corresponding to the three groups of data.

6.3. Photostability

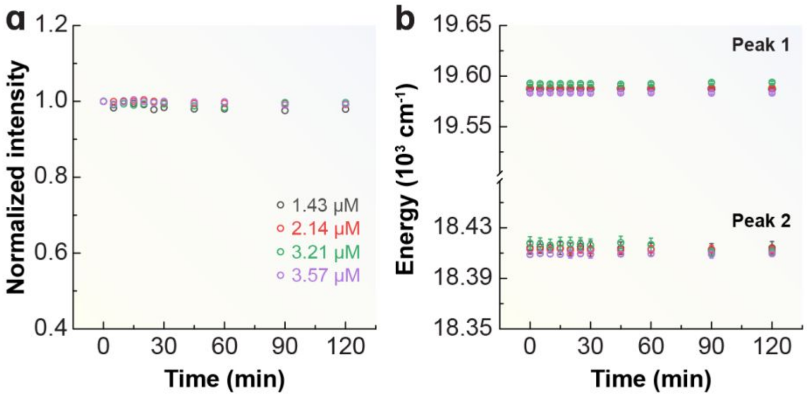

Figure S10. Time dependence of (a) normalized emission integrated intensity, (b) peak 1 and peak 2 energies of selected EGFP aqueous suspensions upon continuous excitation at 25.0 °C.

6.4. Thermal denaturation: Literature data

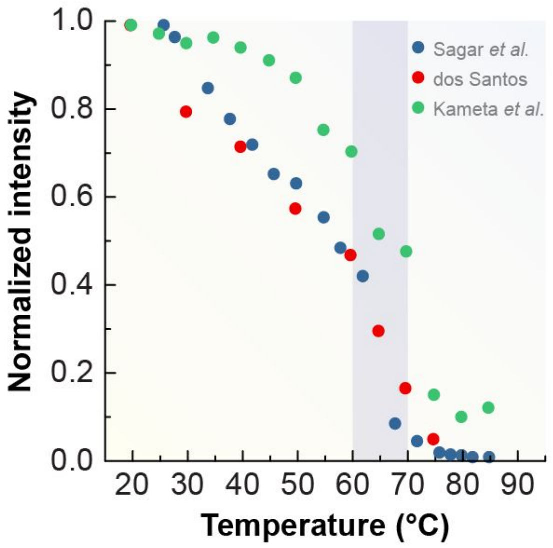

Figure S11. Thermal denaturation of GFP fluorescence, as reported by Sagar et *al.*,[16] dos Santos,[17] and Kameta *et al.*.[18] The shadowed area marks the onset of the protein unfolding.

6.5. Temperature dependence: Emission intensity

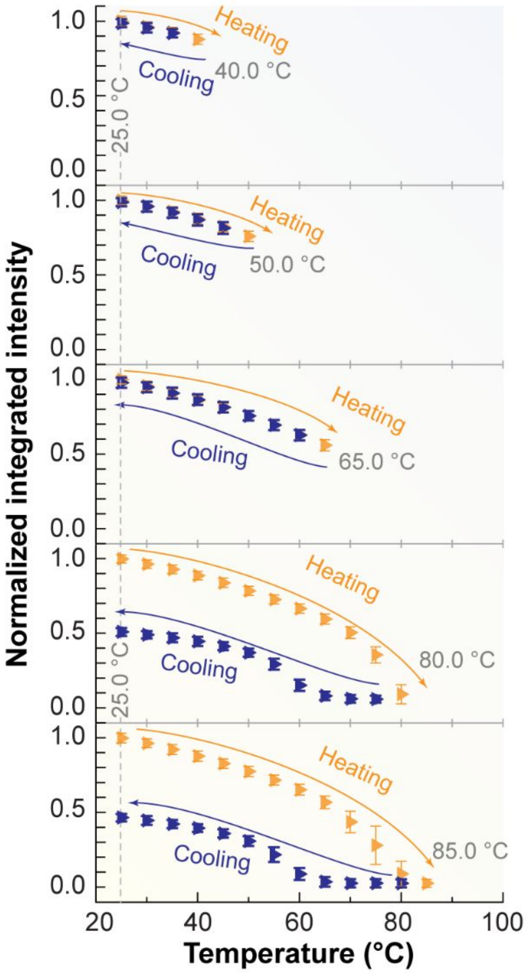

Figure S12. Normalized emission integrated intensity of 1.43 μM EGFP aqueous suspension during heating and cooling cycles with different maximum temperatures. The data points correspond to the mean value of the emission integrated area (475−625 nm) over the 100 recorded spectra, while the error bar is the corresponding standard deviation.

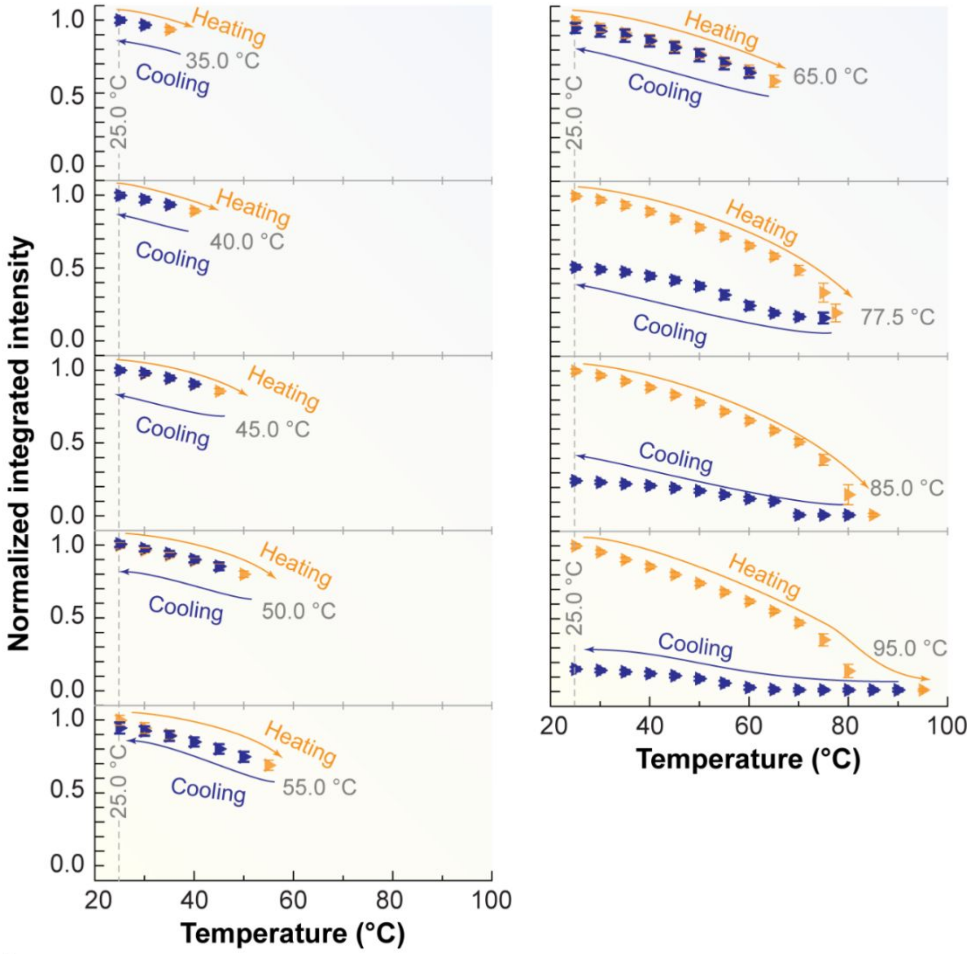

Figure S13. Normalized emission integrated intensity of 2.86 μM EGFP aqueous suspension as a function of temperature during heating and cooling cycles with different maximum temperatures. The temperature step between each point is 5.0 °C.

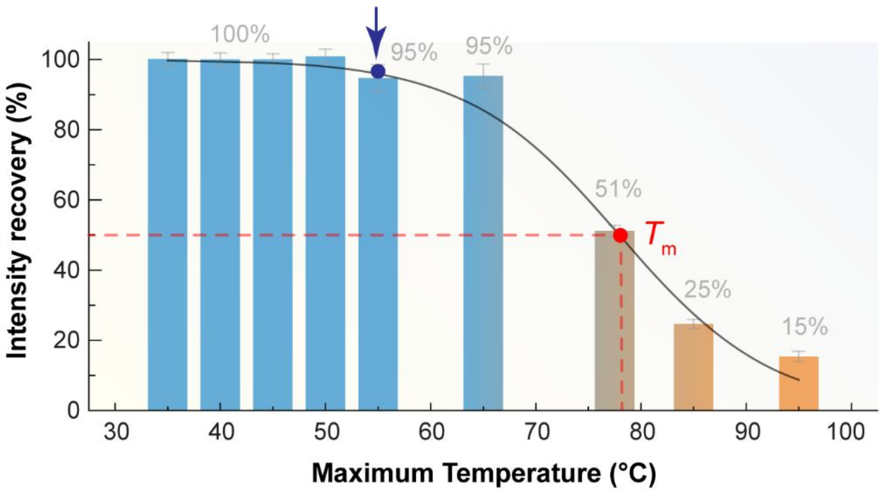

Figure S14. Fluorescence intensity recovery (2.86 μM) after heating/cooling cycles in H_2O. The lines are the best fit to the data using Boltzmann functions (r^2>0.988, **Table S3**). The points mark the melting temperature T_m, while the arrows mark the onset of protein unfolding.

The fluorescence intensity recovery ratios of EGFP in H_2O and D_2O were fitted to a sigmoidal Boltzmann function, using OriginLab® software:

$$\beta(T) = \frac{1}{1 + \exp\left(\frac{T - T_{\mathrm{m}}}{\Gamma}\right)} \tag{S4}$$

where Γ is a temperature range characteristic of the protein and T_{m} is the melting temperature (temperature in the inflection point, corresponding to β=0.5). The parameter Γ governs the steepness of the thermal transition: smaller values indicate sharper transitions around T_{m}, while larger values correspond to more gradual changes in β with temperature.

Table S3. Fitting parameters of the fluorescence intensity recovery of EGFP in H_2O (1.43 and 2.86 µM) after heating and cooling cycles between 25.0 and 95.0 °C in **Figure 3c** and **Figure 5c** using **Eq. S4**.

Concentration (µM)	Solvent	Fitting parameter	Value	r^2
1.43	H_2O	T_{m}	84 ± 1	0.962
		Γ	9 ± 1	
2.86		T_{m}	78 ± 1	0.989
		Γ	7 ± 1	

6.6. Temperature dependence: Emission peak energy

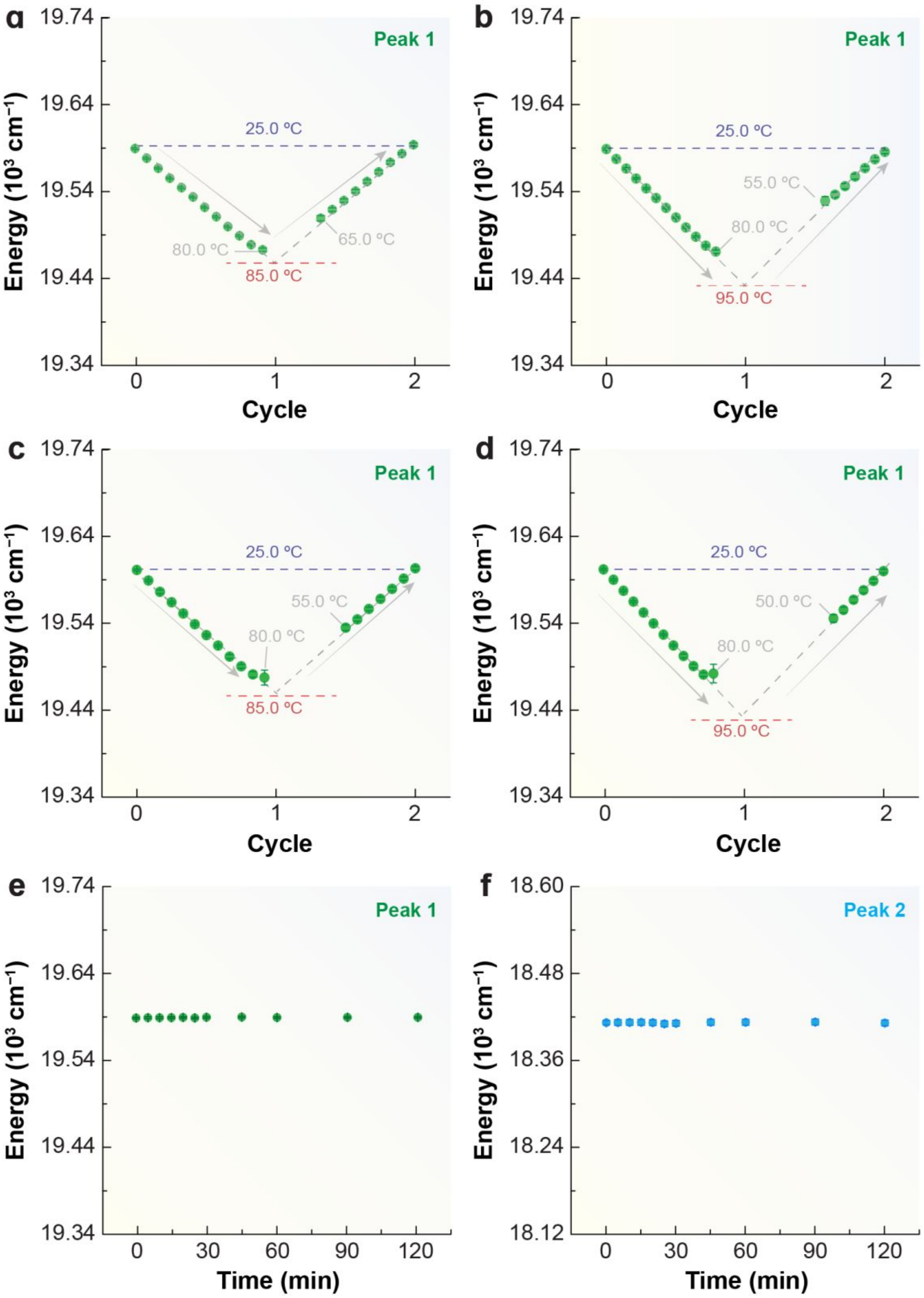

Figure S15. Temperature-dependent peak 1 energy of EGFP aqueous suspensions measured between 25.0−85.0 °C and 25.0−95.0 °C for (a)−(b) 2.86 µM and (c)−(d) 1.43 µM. Missing data points indicate temperatures at which the fluorescence intensity was too low for reliable peak analysis. Real-time tracking of 2.86 µM (e) peak 1 and (f) peak 2 energies at 25.0 °C, over 120 min of continuous irradiation.

6.7. Emission quenching at low concentrations

Emission quenching of EGFP at lower concentrations and high temperatures limits our measurement range under the current conditions. **Figure S16** shows that the emission intensity of the 0.71 μM sample rapidly decreases at temperatures above 60.0 °C, resulting in an increased dispersion of the time-dependent peak 1 energy data points, making it difficult to determine the onset time t_0 from the redshift of peak 1 (see the Experimental Section). This dispersion is significantly greater than that of high-concentration samples (**Figure S17**). Additionally, measuring the Brownian velocity necessitates multiple heating and cooling cycles, further increasing the dispersion.

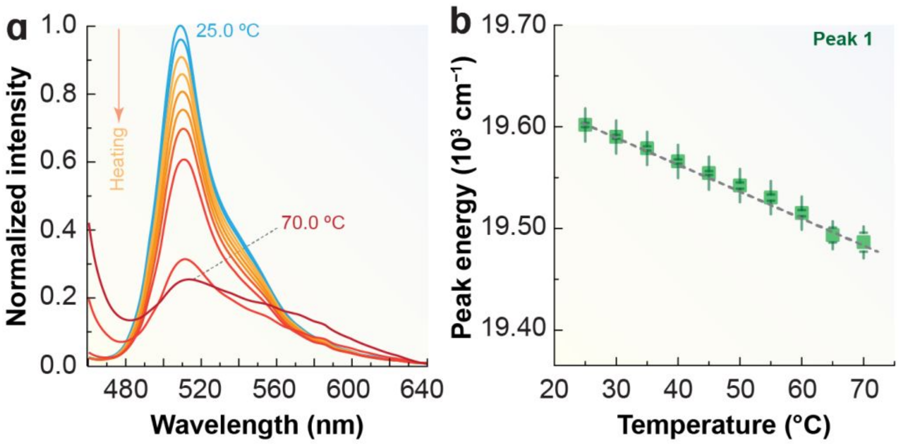

Figure S16. (a) Emission spectra and (b) peak 1 energy of the 0.71 μM EGFP aqueous suspension as a function of temperature in the 25.0−75.0 °C range.

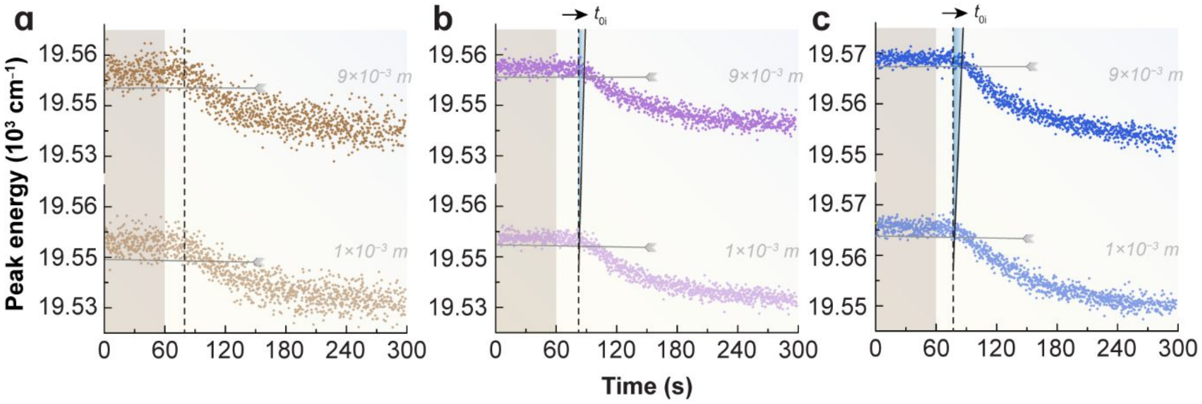

Figure S17. Time-dependence of peak 1 energy recorded at an initial temperature of 55.0 °C and distinct positions ($x_1 = 1\times10^{-3}$ m and $x_5 = 9\times10^{-3}$ m) along the cuvette path length (**Figure S8a)** for distinct EGFP aqueous suspensions: (a) 0.71 μM, (b) 1.43 μM, and (c) 2.86 μM. The dark shadowed area for $t < 60$ s corresponds to the time interval at which the suspension is at the initial temperature (heating off).

7. Optical features of EGFP in D_2O

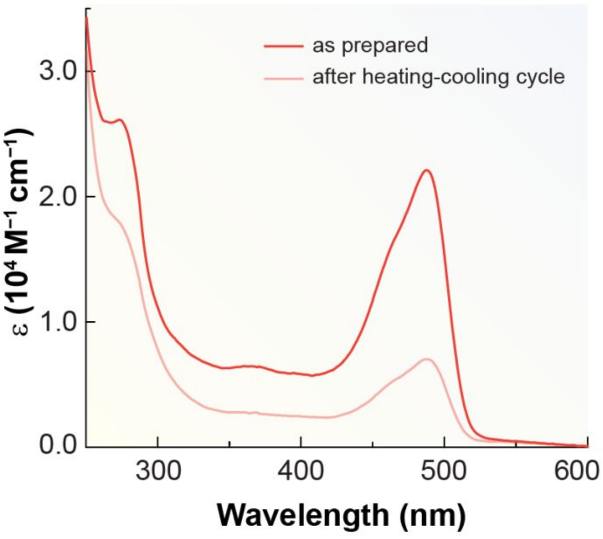

Figure S18. Absorbance spectra of 2.86 μM EGFP suspension in D_2O before and after a heating/cooling temperature cycle at 25.0 °C.

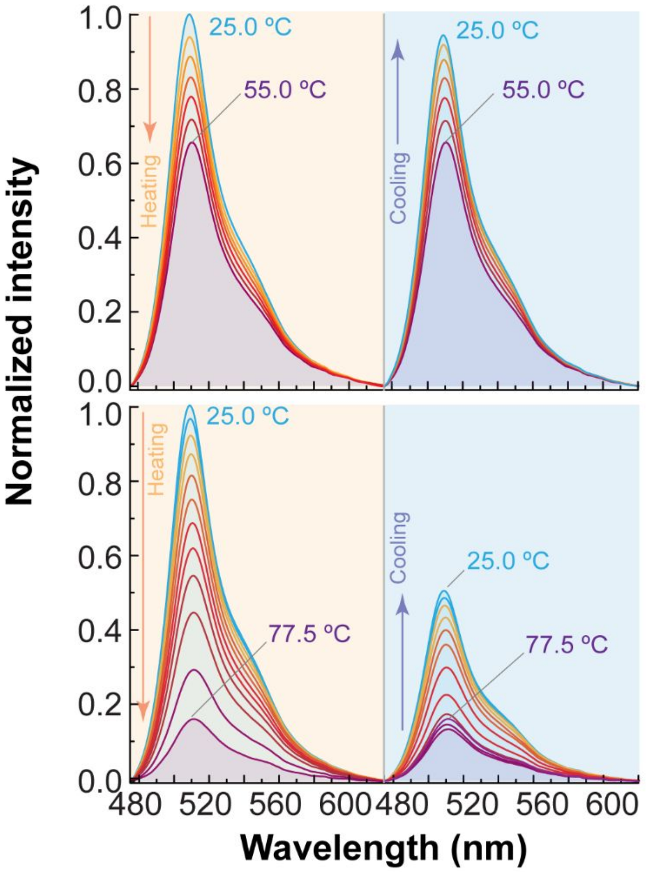

Figure S19. Emission spectra of 2.86 μM EGFP suspension in D_2O as a function of temperature in the 25.0−55.0 °C and 25.0−77.5 °C ranges upon heating and cooling.

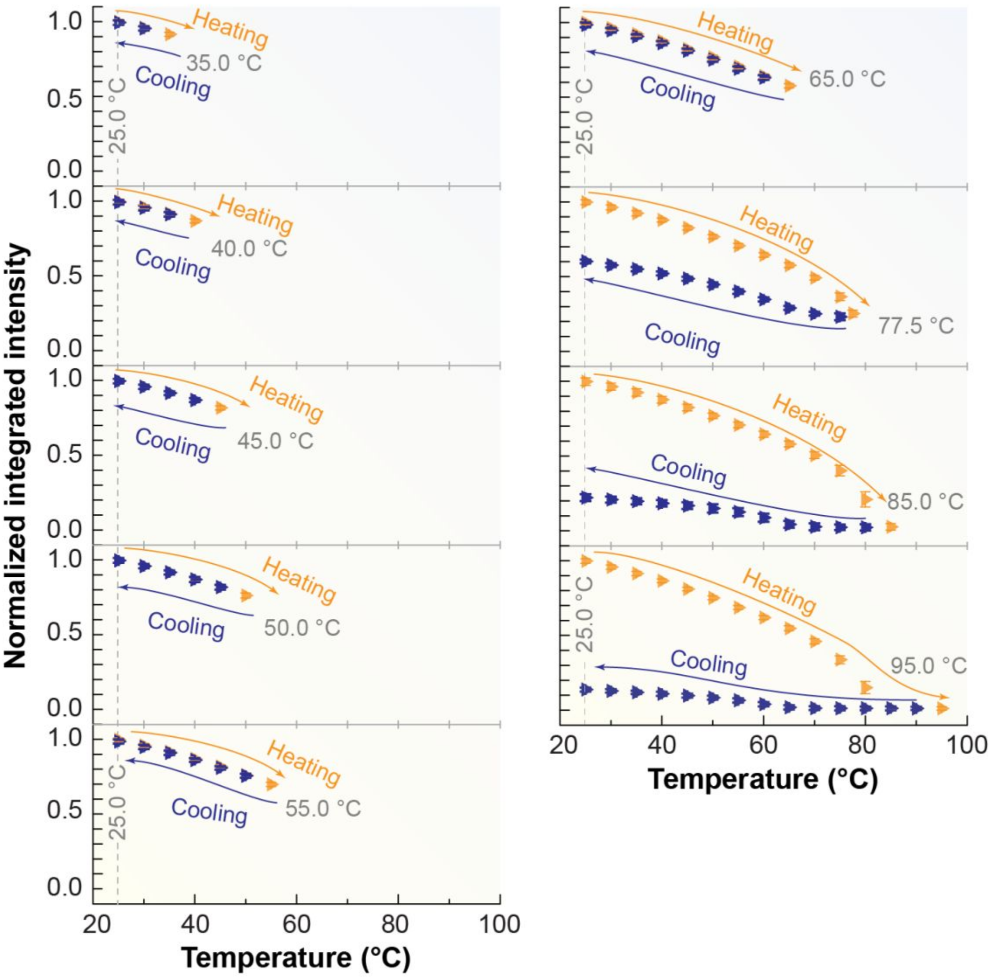

Figure S20. Normalized emission integrated intensity of 2.86 μM EGFP suspension in D_2O during heating and cooling cycles with different maximum temperatures. The data points correspond to the mean value of the emission integrated area (475−625 nm) over the 100 recorded spectra, while the error bar is the corresponding standard deviation.

Table S4. Fitting parameters of the fluorescence intensity recovery of 2.86 µM EGFP suspension in D_2O after heating and cooling cycles between 25.0 and 95.0 °C, (**Figure 5c** and **Figure S20**), using **Eq. S4**.

Fitting parameter	Value	r^2
T_m	80 ± 1	0.988
Γ	6 ± 1	

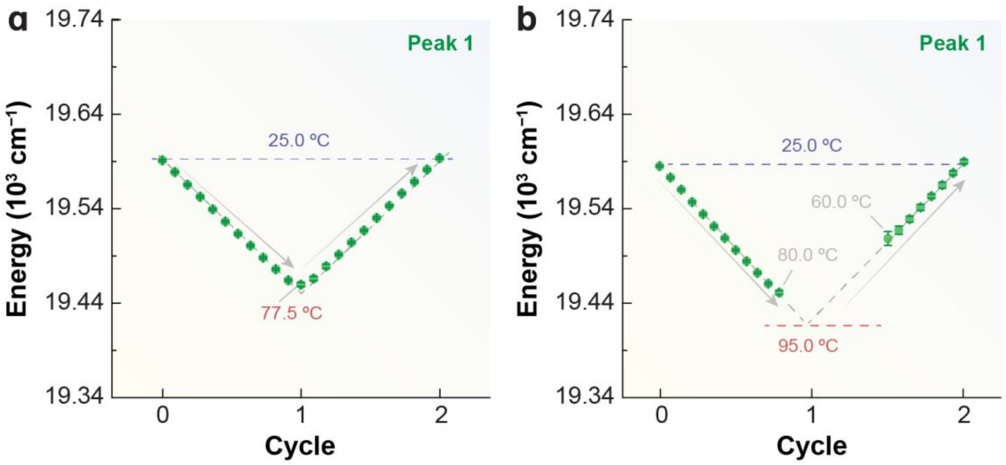

Figure S21. Temperature-dependent peak 1 energy of 2.86 µM EGFP suspension in D_2O measured between (a) 25.0−77.5 °C and (b) 25.0−95.0 °C. Missing data points indicate temperatures at which the fluorescence intensity was too low for reliable peak analysis.

8. Thermometric features

8.1. Repeatability

The repeatability of a thermometric probe is evaluated by assessing its response to repeated measurements under identical conditions. A measurement is considered repeatable when multiple readings of a given quantity, obtained using the same instrument or method over a specified period, exhibit consistency. Consequently, in a repeatability study, any observed variation in measurements of the same subject is attributable solely to errors inherent within the measurement process.[19, 20] The repeatability (R) is determined by:

$$R = 1 - \frac{\max\left(|\bar{\Delta} - \Delta_i|\right)}{\bar{\Delta}} \quad \text{(S5)}$$

where $\bar{\Delta}$ represents the mean thermometric parameter Δ (the integrated fluorescence emission band) and Δ_i each measurement of that parameter.

Table S5. Repeatability of peak 1 of EGFP energy at different concentrations and solvents during heating and cooling cycles with different maximum temperatures.

Concentration (μM)	Solvent	Temperature (°C)	Repeatability (%)
1.43	H_2O	25.0	99.98
		30.0	99.98
		35.0	99.97
		40.0	99.97
		45.0	99.97
		50.0	99.97
		55.0	99.93
		60.0	99.67
		65.0	99.94
		70.0	99.92
		75.0	99.93
		77.5	99.99
2.86	H_2O	25.0	99.97
		30.0	99.96
		35.0	99.96
		40.0	99.96
		45.0	99.96
		50.0	99.96
		55.0	99.96
		60.0	99.90
		65.0	99.95
		70.0	99.97
		75.0	99.97
		77.5	99.98
2.86	D_2O	25.0	99.97
		30.0	99.97
		35.0	99.96
		40.0	99.96
		45.0	99.96
		50.0	99.96
		55.0	99.96
		60.0	99.95
		65.0	99.75
		70.0	99.98
		75.0	99.98
		77.5	99.99

9. Brownian velocity of EGFP

The EGFP Brownian velocity was determined using the experimental setup shown in **Figure S8a**, under a heat flux applied along the *xx*-direction within the cuvette. This heat flux was generated by a Kapton thermofoil heater (HK6906, Minco) connected to a temperature controller (E5CN, Omron). The excitation beam was focused within the cuvette at a defined position (x_1) along the *xx*-direction, see **Figure S8a**. Before heating, the temperature was stabilized at 30.0 °C for 60 s. The Kapton heater then increased the temperature by 10.0 °C, and emission spectra were recorded for 300 s. Peak 1 energy was calculated using lineshape correction and spectral deconvolution. To determine the onset time t_{01}, the time-dependent peak 1 energy was denoised using a discrete wavelet transformation. During the initial 60 s (before the Kapton thermofoil heater was turned on), peak 1 energy fluctuated within its uncertainty, calculated as the standard deviation of its distribution. As the temperature increased, peak 1 exhibited a redshift, and t_{01} was identified as the moment when its energy decreased beyond the uncertainty threshold (**Figure S22**). This process was repeated at four additional positions (x_i, i=2–5) along the cuvette to obtain the corresponding t_{0i} values. The Brownian velocity was determined from the slope of the x_i versus t_{0i} (i=1–5) plot (Figure S23). The same procedure was applied for initial temperatures up to 72.5 °C (see **Figure S24**, **Figure S25**, **Figure S26**, **Figure S27**, and **Figure S28**). A dataset is available as Supporting Information.

The discrete wavelet transformation (DWT) procedure, a nonlinear noise reduction method,[21, 22] was employed to denoise the as-measured peak energy data. The denoising procedure was implemented in four steps using a custom Matlab® script. In summary, the temporal dependence of the peak energy was imported and then normalized to the [0-1] range. Next, the DWT denoising procedure was applied (threshold parameter of 15, 5 stages), and the noise was determined as the difference between the measured and the denoised peak energy values. The histogram of the noise values was computed for all the denoised curves, invariantly following a Gaussian profile (r^2 > 0.980) centered at zero. For all the measurements, the noise is consistent with an additive white Gaussian signal, which validates the use of the DWT denoising procedure. Illustrative denoised curves are presented in **Figure S23** for distinct EGFP aqueous suspensions.

The corresponding x_i versus t_{0i} plots are presented in **Figure S24**. Note that the Brownian velocity corresponds to the slope of the best linear fit to the experimental data. Additional temperature dataset results are provided as Supplementary Material. We estimate the uncertainty in the Brownian velocity from the uncertainty in the slope of the best linear fit to

the x_i versus t_{0i} data. In general, the uncertainty in the Brownian velocity increases with temperature due to the lowest coefficient of determination (r^2).

To reduce the impact of self-absorption on temperature measurement, as discussed in our previous paper,[14] the time-dependent peak 1 energy of the EGFP emission spectra was collected from the back of the cuvette (**Figure S22a)**, and converted to temperature (**Figure S21c)** using calibration curves obtained at different positions (**Figure S21b)**. The observed temperature shift over time, about 10°C, matches our experimental heating parameters. Additionally, the increased onset time, t_{0i} (i = 1−5) from x_1−x_5, can be clearly discerned in the normalized denoised curve, inset of **Figure S21c**. However, since t_0 can be directly determined based on the temperature dependence of peak 1 energy, we will omit temperature conversion and focus on analyzing the time-dependent changes in peak energy to streamline t_0 determination.

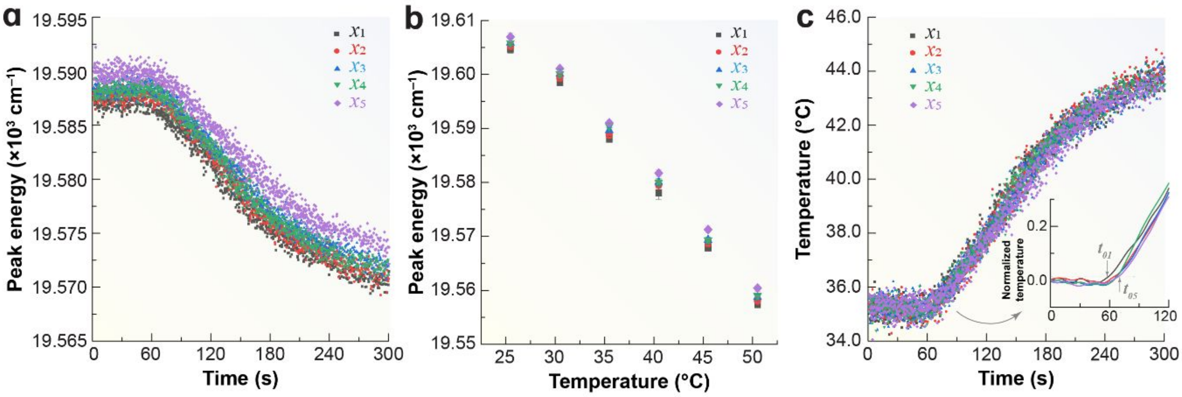

Figure S22. (a) Time-dependent energy of peak 1 in the 2.86 µM EGFP aqueous suspension recorded at 35.0 °C across distinct positions along the cuvette length. (b) Corresponding calibration curve showing the relationship between the energy of peak 1 and temperature. (c) Time-dependent temperature variation, derived from the energy of peak 1 (as calibrated in (b)) measured at the same distinct positions along the cuvette length. The inset shows the denoising curves.

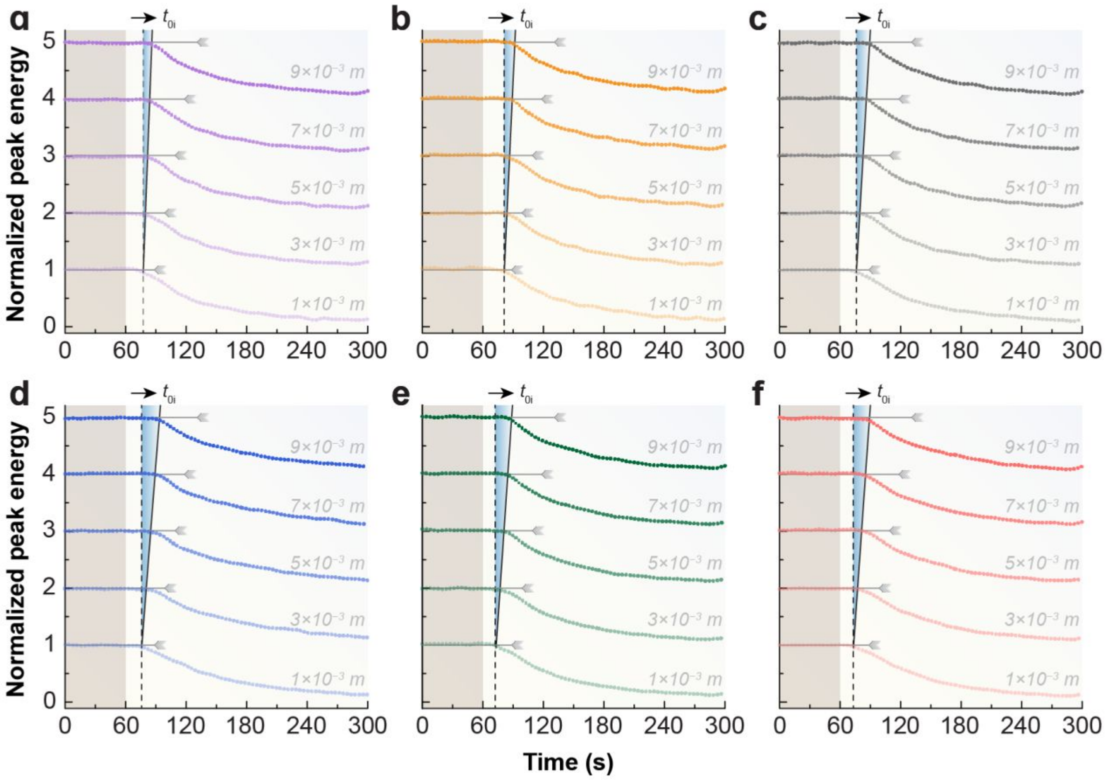

Figure S23. Illustrative time-dependence of normalized peak 1 energy recorded at an initial temperature of 30.0 °C and distinct positions along the cuvette pathlength (**Figure S8a**) for different EGFP aqueous suspensions: (a) 1.43 µM, (b) 1.43 µM (after a heating/cooling cycle), (c) 2.14 µM, (d) 2.86 µM, (e) 3.21 µM, and (f) 3,57 µM. The curves were shifted vertically for better visualization. The onset time t_{0i} at which the peak energy changes upon heating is marked as a full line, and the dashed line corresponds to the t_{0i} value at x_i. The dark shadowed area for $t < 60$ s corresponds to the time interval at which the suspension is at the initial temperature (heating off). The horizontal lines are guides to the eyes, marking the standard deviation of the normalized peak 1 energy, used to determine t_{0i}.

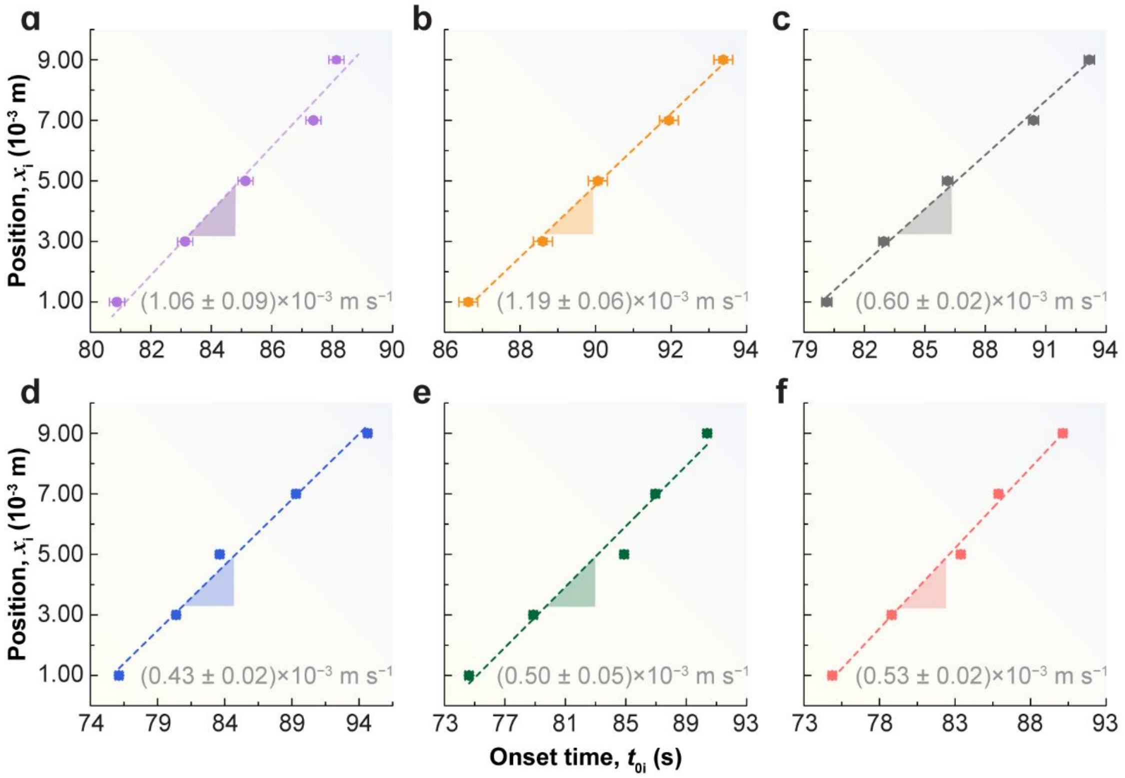

Figure S24. Illustrative x_i versus t_{0i} plots for distinct EGFP aqueous suspensions obtained from the time dependence of normalized peak 1 energy recorded at an initial temperature of 30.0 °C: (a) 1.43 µM, (b) 1.43 µM (after a heating/cooling cycle), (c) 2.14 µM, (d) 2.86 µM, (e) 3.21 µM, and (f) 3,57 µM. The numbers are the slope of the best linear fit to the data (**Table S6**). The error in the slope values is the uncertainty in the Brownian velocity. The error bars in x_i and t_{0i} are, respectively, the uncertainty in the position of the moving stage from **Figure S8a** (10^{-4} m) and the integration time used during spectra acquisition (0.250 s).

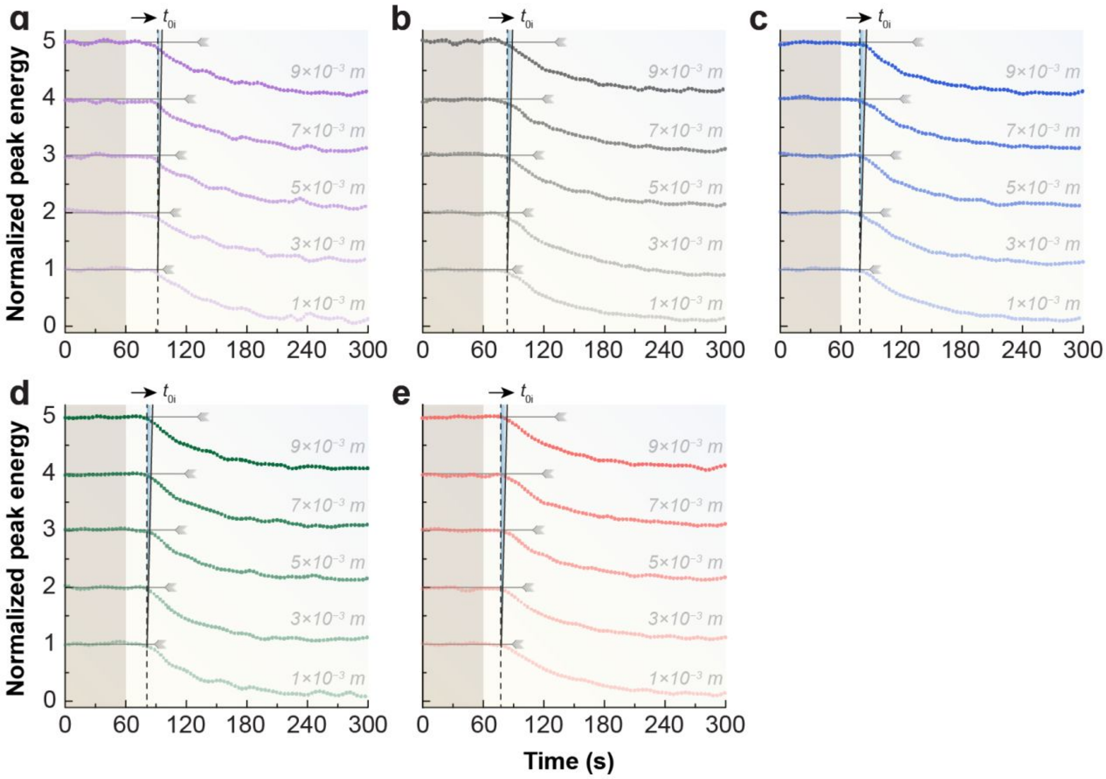

Figure S25. Illustrative time dependence of normalized peak 1 energy recorded at an initial temperature of 67.5 °C for distinct EGFP aqueous suspensions: (a) 1.43 µM, (b) 2.14 µM, (c) 2.86 µM, (d) 3,21 µM, and (e) 3,57 µM. The curves were shifted vertically for better visualization. The onset time t_{0i} at which the peak energy changes upon heating is marked as a full line, and the dashed line corresponds to the t_{0i} value at x_i. The dark shadowed area for $t <$ 60 s corresponds to the time interval at which the suspension is at the initial temperature (heating off). The horizontal lines are guides to the eyes, marking the standard deviation of the normalized peak 1 energy, used to determine t_{0i}.

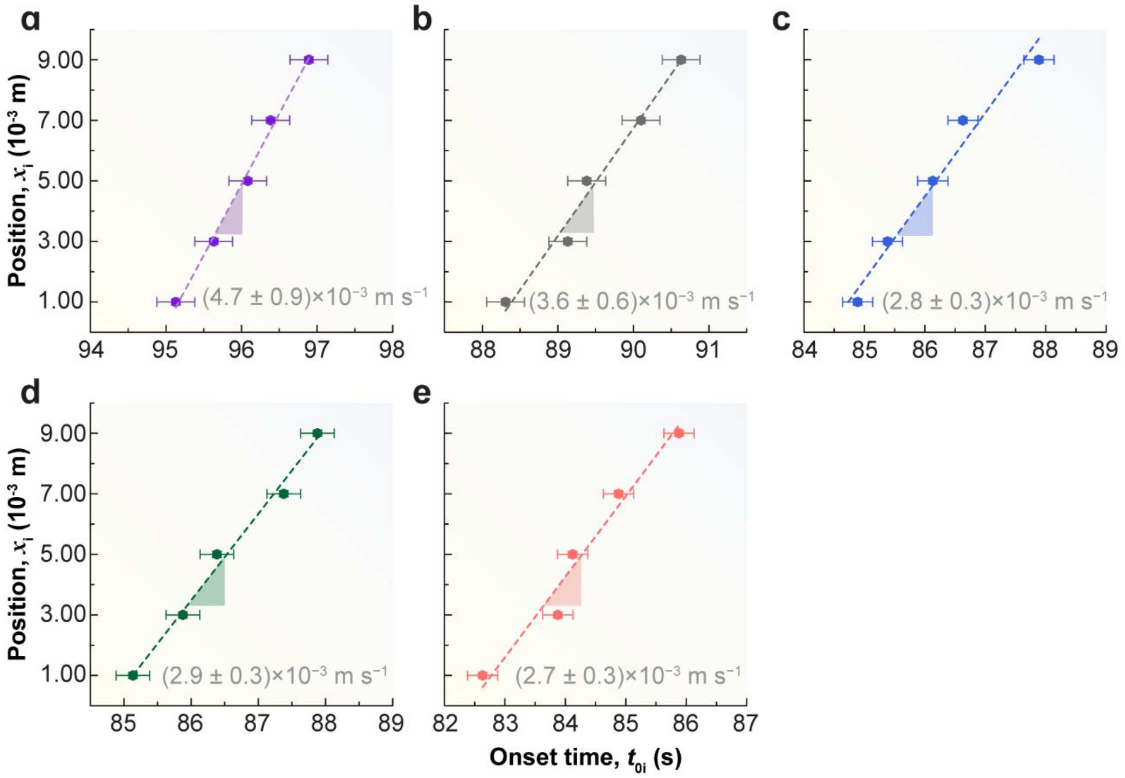

Figure S26. Illustrative x_i versus t_{0i} plots for distinct EGFP aqueous suspensions obtained from the time-dependence of normalized peak 1 energy recorded at an initial temperature of 67.5 °C: (a) 1.43 µM, (b) 2.14 µM, (c) 2.86 µM, (d) 3,21 µM, and (e) 3,57 µM. The numbers are the slope of the best linear fit to the data (**Table S6**). The error in the slope values is the uncertainty in the Brownian velocity. The error bars in x_i and t_{0i} are, respectively, the uncertainty in the position of the moving stage from **Figure S8a** (10^{-4} m) and the integration time used during spectra acquisition (0.250 s).

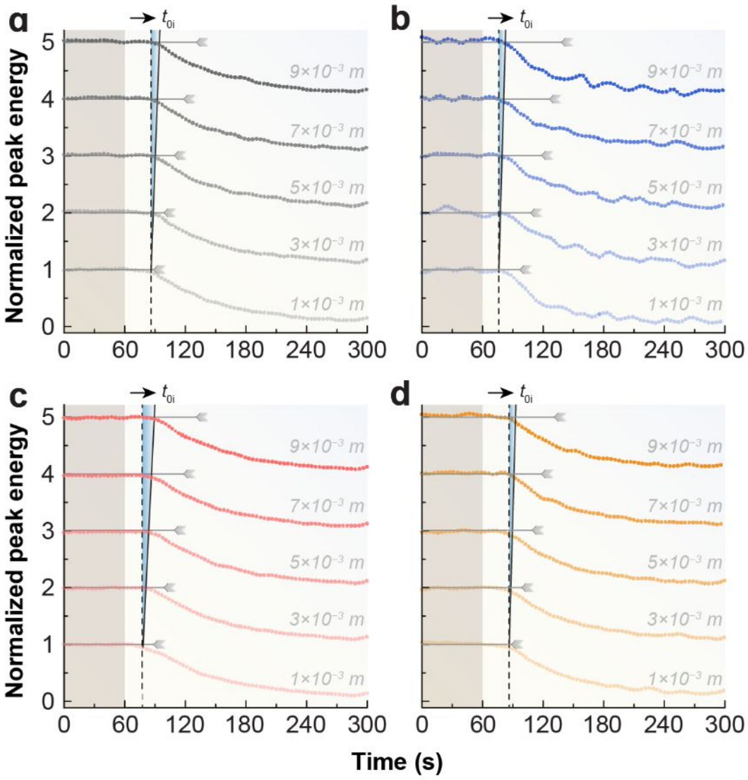

Figure S27. Illustrative time-dependence of normalized peak 1 energy recorded for EGFP suspensions in D_2O at 2.14 and 2.86 μM concentrations and (a) and (c) 30.0 °C, (b) and (d) 67.5 °C initial temperatures. The curves were shifted vertically for better visualization. The onset time t_{0i} at which the peak energy changes upon heating is marked as a full line, and the dashed line corresponds to the t_{0i} value at x_i. The dark shadowed area for $t < 60$ s corresponds to the time interval at which the suspension is at the initial temperature (heating off). The horizontal lines are guides to the eyes, marking the standard deviation of the normalized peak 1 energy, used to determine t_{0i}.

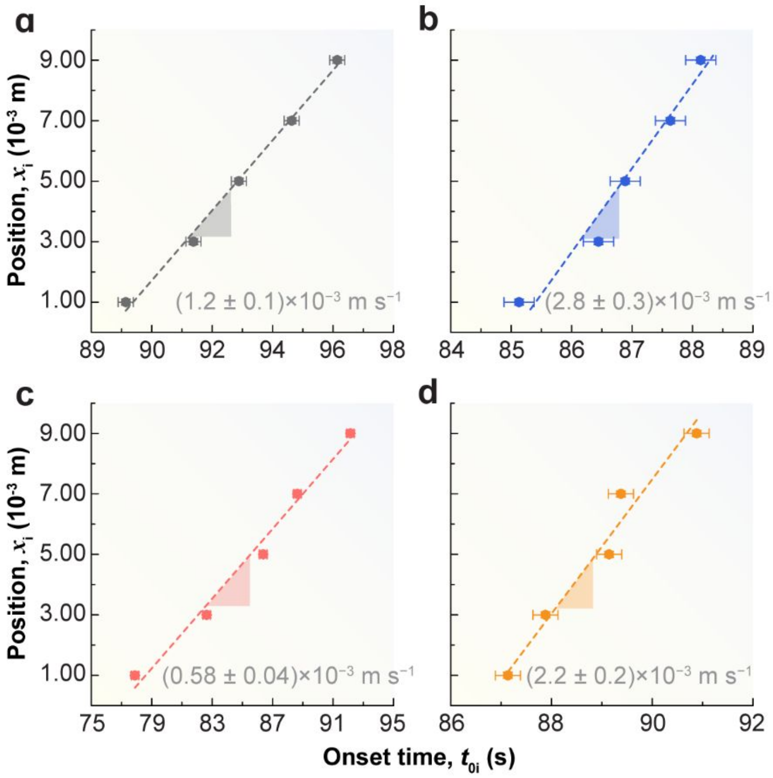

Figure S28. Illustrative x_i versus t_{0i} plots for EGFP suspensions in D_2O at 2.14 and 2.86 μM concentrations and (a) and (c) 30.0 °C, (b) and (d) 67.5 °C initial temperatures. The numbers are the slope of the best linear fit to the data (**Table S7**). The error in the slope values is the uncertainty in the Brownian velocity. The error bars in x_i and t_{0i} are, respectively, the uncertainty in the position of the moving stage from **Figure S8a** (10^{-4} m) and the integration time used during spectra acquisition (0.250 s).

Measuring the Brownian velocity of EGFP in D_2O suspensions becomes difficult at low concentrations (e.g., 2.14 μM) and temperatures exceeding 67.5 °C due to weak signal intensity. This limitation hinders the determination of the onset time and, therefore, the Brownian velocity, explaining the data range in **Figure 5b**(up to 65 °C). In contrast, at a higher concentration of 2.86 μM, we were able to measure the Brownian velocity at 70.0 °C, **Figure S29a,b**, observing the same bilinear temperature dependence previously as in H_2O, **Figure 5c**. Nevertheless, the considerable fluctuations observed in the time-dependence of peak 1 energy at an initial temperature of 72.5 °C restrict the inference of the onset time to only the two extreme positions of the cuvette path length (**Figure S29c**). This limitation prevents the determination of the Brownian velocity at this temperature. Notwithstanding this limitation, the Brownian velocity extrapolated to 72.5 °C, 4.6×10^{-3} m s^{-1}, **Figure 5c**, assuming a linear dependence above 65 °C, is compatible with the discerned onset times. As observed in H_2O, increasing the EGFP concentration from 2.14 to 2.86 μM leads to a reduction in its Brownian velocity. The Brownian

velocities in both H_2O and D_2O are comparable, consistent with our previous findings for UCNPs.[22] Furthermore, the slopes of the linear relationships observed below T_c in both light and heavy water are also similar.

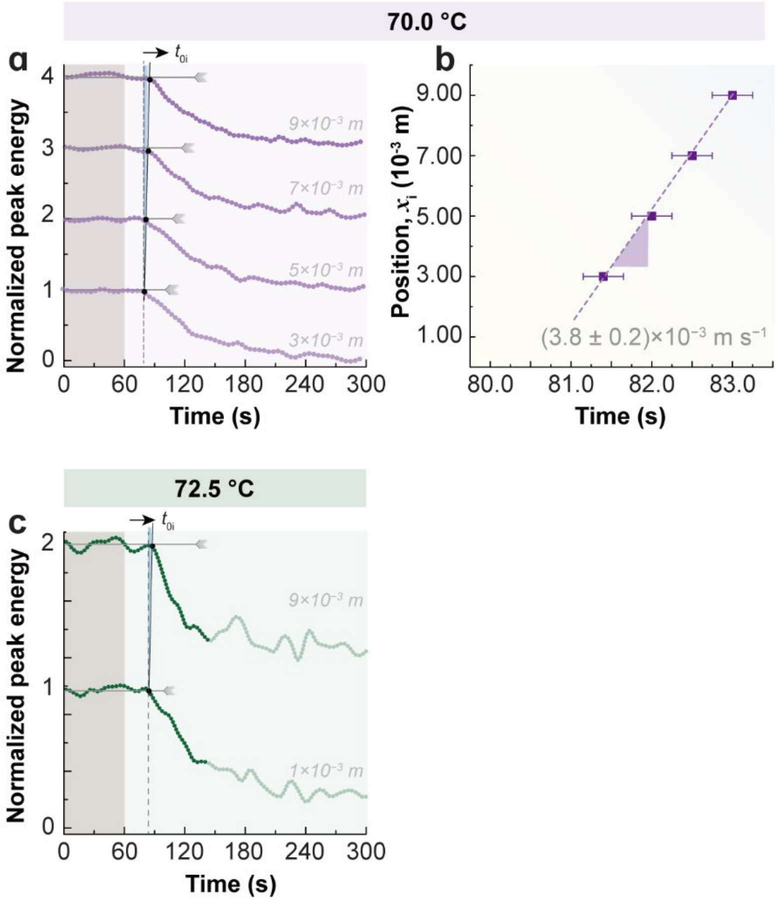

Figure S29. (a) Time-dependence of peak 1 energy recorded at an initial temperature of 70.0 °C and distinct positions ($x_2 = 3\times10^{-3}$ m and $x_5 = 9\times10^{-3}$ m) along the cuvette path length (**Figure S8a)** for EGFP D_2O suspension at 2.86 µM. The point at $x_1 = 1\times10^{-3}$ m was not considered because the fluctuations in the signal precluded the determination of the t_{01} value. (b) Corresponding x_i versus t_{0i} plot. (c) Time-dependence of peak 1 energy recorded at an initial temperature of 72.5 °C at $x_1 = 1\times10^{-3}$ m and $x_5 = 9\times10^{-3}$ m for EGFP D_2O suspension at 2.86 µM. The dark shadowed areas in a) and c) for t < 60 s correspond to the time interval at which the suspension is at the initial temperature (heating off).

Table S6. Slope of x_i versus t_{0i} data for distinct EGFP for all the temperatures in H_2O.

Concentration (μM)	Temperature (°C)	Slope	r^2
0.36	30.0	2.4 ± 0.2	0.997
0.54	30.0	2.3 ± 0.2	0.989
0.71	30.0	1.8 ± 0.2	0.966
1.07	30.0	1.5 ± 0.1	0.998
1.43	30.0	1.1 ± 0.1	0.978
	35.0	1.4 ± 0.1	0.994
	40.0	1.6 ± 0.1	0.996
	45.0	2.0 ± 0.2	0.992
	50.0	2.3 ± 0.2	0.996
	52.5	2.5 ± 0.3	0.996
	55.0	2.5 ± 0.3	0.981
	57.5	3.1 ± 0.4	0.983
	60.0	3.3 ± 0.4	0.996
	62.5	3.5 ± 0.5	0.994
	65.0	4.0 ± 0.6	0.999
	67.5	4.7 ± 0.9	0.994
1.43 (second cycle)	30.0	1.2 ± 0.1	0.997
	35.0	1.5 ± 0.1	0.993
	40.0	1.8 ± 0.1	0.992
	45.0	2.1 ± 0.2	0.992
	50.0	2.3 ± 0.2	0.993
	52.5	2.4 ± 0.2	0.997
	55.0	2.9 ± 0.3	0.995
	57.5	3.3 ± 0.4	0.996
	60.0	3.3 ± 0.1	0.994
1.79	30.0	0.77 ± 0.03	0.993
2.14	30.0	0.60 ± 0.02	0.994
	35.0	0.9 ± 0.1	0.988
	40.0	1.2 ± 0.1	0.992
	45.0	1.3 ± 0.1	0.998
	50.0	1.6 ± 0.1	0.985
	52.5	1.7 ± 0.1	0.998
	55.0	1.8 ± 0.1	0.996
	57.5	2.3 ± 0.2	0.988
	60.0	2.5 ± 0.3	0.996
	62.5	2.9 ± 0.3	0.995
	65.0	3.0 ± 0.4	0.995
	67.5	3.6 ± 0.6	0.979
2.86	30.0	0.43 ± 0.02	0.990
	35.0	0.6 ± 0.1	0.954
	40.0	0.7 ± 0.1	0.956
	45.0	0.8 ± 0.1	0.974
	50.0	0.97 ± 0.04	0.997
	55.0	1.2 ± 0.1	0.992
	57.5	1.3 ± 0.1	0.996
	60.0	1.8 ± 0.2	0.961
	62.5	2.1 ± 0.2	0.997

	65.0	2.5 ± 0.3	0.953
	67.5	2.8 ± 0.3	0.967
3.21	30.0	0.5 ± 0.1	0.975
	35.0	0.7 ± 0.1	0.982
	40.0	0.8 ± 0.1	0.979
	45.0	0.9 ± 0.1	0.979
	50.0	0.94 ± 0.04	0.995
	52.5	1.00 ± 0.04	0.998
	55.0	1.2 ± 0.1	0.986
	57.5	1.6 ± 0.1	0.987
	60.0	1.9 ± 0.2	0.997
	62.5	2.1 ± 0.2	0.971
	65.0	2.7 ± 0.3	0.999
	67.5	2.9 ± 0.3	0.990
3.57	30.0	0.53 ± 0.02	0.993
	35.0	0.7 ± 0.1	0.955
	40.0	0.74 ± 0.03	0.994
	45.0	0.9 ± 0.1	0.985
	50.0	1.1 ± 0.1	0.997
	55.0	1.3 ± 0.1	0.979
	57.5	1.4 ± 0.1	0.999
	60.0	1.9 ± 0.2	0.979
	62.5	2.0 ± 0.2	0.992
	65.0	2.3 ± 0.2	0.997
	67.5	2.7 ± 0.3	0.965

Table S7. Slope of x_i versus t_{0i} data for distinct EGFP for all the temperatures in D_2O.

Concentration (μM)	Temperature (°C)	Slope	r^2
	30.0	1.2 ± 0.1	0.992
	35.0	1.4 ± 0.1	0.994
	40.0	1.6 ± 0.2	0.963
	45.0	1.9 ± 0.2	0.977
	50.0	1.9 ± 0.2	0.972
2.14	55.0	2.2 ± 0.3	0.951
	57.5	2.1 ± 0.2	0.967
	60.0	2.4 ± 0.2	0.973
	62.5	2.4 ± 0.2	0.982
	65.0	2.6 ± 0.3	0.953
	67.5	2.8 ± 0.3	0.961
	30.0	0.6 ± 0.1	0.985
	35.0	0.7 ± 0.1	0.980
	40.0	0.9 ± 0.1	0.981
	45.0	1.1 ± 0.1	0.971
	50.0	1.4 ± 0.2	0.941
	52.5	1.5 ± 0.1	0.985
2.86	55.0	1.7 ± 0.1	0.988
	57.5	1.7 ± 0.1	0.980
	60.0	2.0 ± 0.2	0.988
	62.5	2.0 ± 0.2	0.982
	65.0	2.1 ± 0.2	0.980
	67.5	2.2 ± 0.2	0.966
	70.0	3.8 ± 0.2	0.997

9.1. Uncertainty in the thermocouple measurements

The EGFP suspensions were heated from one side of the cuvette, inducing, therefore, a temperature gradient (**Figure S8a)**. To quantify this gradient, **Figure S30a** shows temperatures recorded at three different positions along the *xx* direction of the cuvette, inset of **Figure S8a**. Measurements were performed using an immersed thermocouple (KA01-3, TME Thermometers, 0.1 °C resolution), with ten readings averaged at each location. Temperature uncertainty was estimated as the maximum deviation relative to the mean value, as shown in **Figure S30b**.

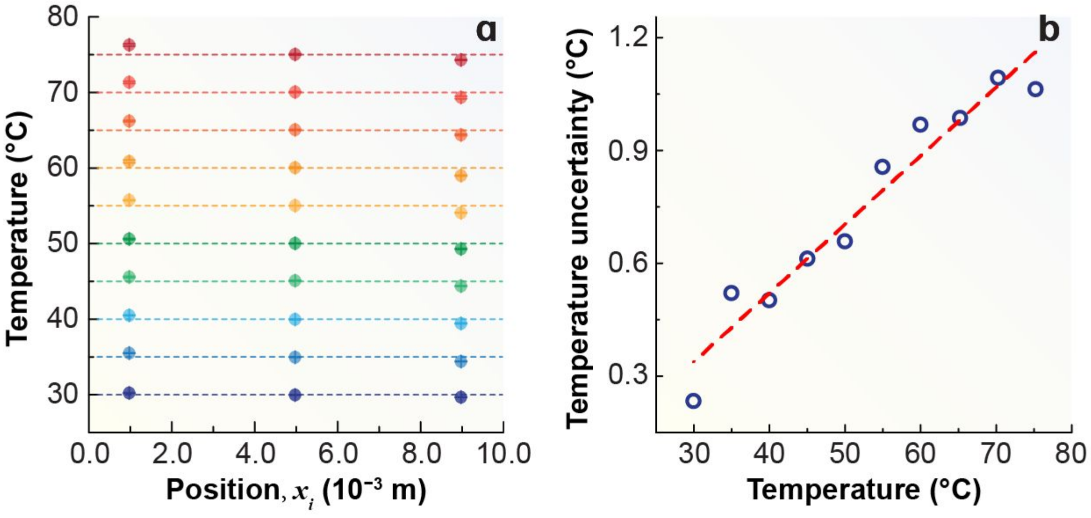

Figure S30. (a) Temperature measurements and (b) corresponding uncertainties of the 0.71 μM EGFP aqueous suspension in the 30.0–80.0 °C range. The dashed line is the best linear fit to the data shown in panel a (r^2>0.934).

9.2. Reproducibility

Since peak 1 energy remains stable after heating/cooling cycles (**Figure S14**) and EGFP Brownian velocity measurements depend exclusively on it, we expect these measurements to be highly reproducible. To verify this, we measured the Brownian velocity at a selected concentration (1.43 μM in H_2O) after a heating/cooling cycle and compared the results with those from the first cycle, observing excellent reproducibility (**Figure S31)**. The absence of data points at high temperatures in the second measurement is attributed to the weaker EGFP emission intensity, as discussed in **Figure S16**.

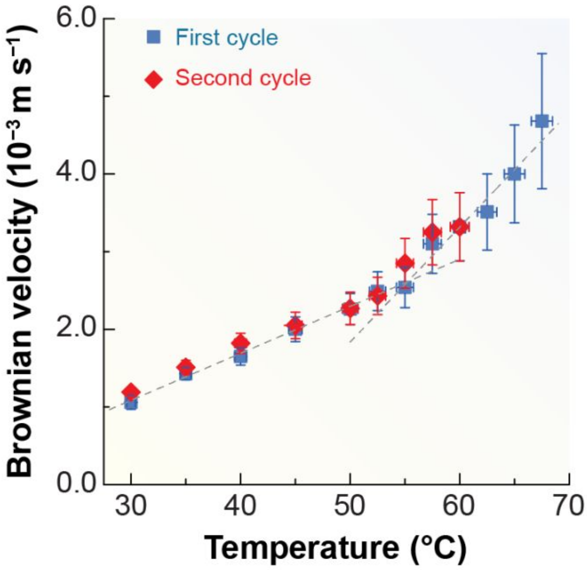

Figure S31. Temperature-dependent Brownian velocity of EGFP (1.43 μM in H_2O) measured in two consecutive heating cycles. The horizontal and vertical error bars were calculated as discussed in **Section 8**.

10. Determination of crossover temperature

The crossover temperature (T_c) corresponds to the temperature of the intersection of the two straight lines representing the temperature dependence of the Brownian velocity of the EGFP in aqueous suspensions between 30.0 and 67.5 °C. Calculating all the possible combinations of two straight lines through a Matlab® script that fits the bilinear pattern in the experimental temperature range, and determining the best fit by maximizing the product of r^2 from each fitted line. The uncertainty of T_c (δT_c) is given by:

$$\delta T_c = \frac{T_c^{max} - T_c^{min}}{2} = \frac{\sigma_1 + \sigma_2}{s_1 - s_2} \qquad \text{(S6)}$$

where T_c^{max} and T_c^{min} are the maximum and minimum predicted values of T_c, respectively, σ_1 and σ_1, and s_1 and s_2 are the standard errors of the estimate and the slope of the linear dependency below and above T_c, respectively. σ is defined as:

$$\sigma = \sqrt{\frac{\sum(v - v')^2}{N - 2}} \qquad \text{(S7)}$$

where v and v' are the measured and the fitted values of the Brownian velocity, respectively, and N is the number of fitted data points.

Table S8. Combined r^2 and crossover temperature of the temperature-dependent Brownian velocity of EGFP at different concentrations from **Figures 4a** and **5c**.

Concentration (µM)	Solvent	Combined r^2	T_c (°C)
1.43		0.944	55 ± 2
2.14		0.962	55 ± 2
2.86	H_2O	0.956	56 ± 1
3.21		0.959	53 ± 1
3.57		0.951	54 ± 1
2.86	D_2O	0.953	65 ± 2

11. References

(1) Hu, J.; Park, S. J.; Walter, T.; Orozco, I. J.; O'Dea, G.; Ye, X.; Du, J.; Lu, W. Physiological temperature drives trpm4 ligand recognition and gating. *Nature* **2024**, *630* (8016), 509-515.

(2) Zacharias, D. A.; Violin, J. D.; Newton, A. C.; Tsien, R. Y. Partitioning of lipid-modified monomeric GFPs into membrane microdomains of live cells. *Science* **2002**, *296* (5569), 913-916.

(3) Matz, M. V.; Fradkov, A. F.; Labas, Y. A.; Savitsky, A. P.; Zaraisky, A. G.; Markelov, M. L.; Lukyanov, S. A. Fluorescent proteins from nonbioluminescent anthozoa species. *Nat. Biotechnol.* **1999**, *17* (10), 969-973.

(4) Liu, L.; Zhang, X. Z.; Fedeli, S.; Cicek, Y. A.; Ndugire, W.; Rotello, V. M. Controlled bio-orthogonal catalysis using nanozyme-protein complexes via modulation of electrostatic interactions. *Materials* **2024**, *17* (7), 1507.

(5) Nolles, A.; Westphal, A. H.; de Hoop, J. A.; Fokkink, R. G.; Kleijn, J. M.; van Berkel, W. J. H.; Borst, J. W. Encapsulation of GFP in complex coacervate core micelles. *Biomacromolecules* **2015**, *16* (5), 1542-1549.

(6) Lavigueur, C.; García, J. G.; Hendriks, L.; Hoogenboom, R.; Cornelissen, J. J. L. M.; Nolte, R. J. M. Thermoresponsive giant biohybrid amphiphiles. *Polym. Chem.* **2011**, *2* (2), 333-340.

(7) Shi, R. N.; Pan, Q.; Guan, Y.; Hua, Z. D.; Huang, Y.; Zhao, M. P.; Li, Y. Z. Imidazole as a catalyst for in vitro refolding of enhanced green fluorescent protein. *Arch. Biochem. Biophys.* **2007**, *459* (1), 122-128.

(8) Kelly, S. M.; Jess, T. J.; Price, N. C. How to study proteins by circular dichroism. *Biochim. Biophys. Acta - Proteins Proteom.* **2005**, *1751* (2), 119-139.

(9) Greenfield, N. J. Using circular dichroism collected as a function of temperature to determine the thermodynamics of protein unfolding and binding interactions. *Nat. Protoc.* **2006**, *1* (6), 2527-2535.

(10) Miles, A. J.; Janes, R. W.; Wallace, B. A. Tools and methods for circular dichroism spectroscopy of proteins: A tutorial review. *Chem. Soc. Rev.* **2021**, *50* (15), 8400-8413.

(11) Kelly, S. M.; Price, N. C. The use of circular dichroism in the investigation of protein structure and function. *Curr. Protein Pept. Sci.* **2000**, *1* (4), 349-384.

(12) Myers, J. K.; Pace, C. N.; Scholtz, J. M. Helix propensities are identical in proteins and peptides. *Biochemistry-Us* **1997**, *36* (36), 10923-10929.

(13) Sreerama, N.; Woody, R. W. Structural composition of β- and β-proteins. *Protein Sci.* **2003**, *12* (2), 384-388.

(14) Guo, Y. W.; Maturi, F. E.; Brites, C. D. S.; Carlos, L. D. Exploring green fluorescent protein Brownian motion: Temperature and concentration dependencies through luminescence thermometry. *Adv. Physics Res.* **2024**, *3* (11), 2400085.

(15) Zhou, Y.; Huo, S. D.; Loznik, M.; Göstl, R.; Boersma, A. J.; Herrmann, A. Controlling optical and catalytic activity of genetically engineered proteins by ultrasound. *Angew. Chem. Int. Edit.* **2021**, *60* (3), 1493-1497.

(16) Sagar, D. M.; Aoudjane, S.; Gaudet, M.; Aeppli, G.; Dalby, P. A. Optically induced thermal gradients for protein characterization in nanolitre-scale samples in microfluidic devices. *Sci. Rep.* **2013**, *3*, 2130.

(17) dos Santos, A. M. Thermal effect on green fluorescent protein anionic and neutral chromophore forms fluorescence. *J. Fluoresc.* **2012**, *22* (1), 151-154.

(18) Kameta, N.; Minamikawa, H.; Someya, Y.; Yui, H.; Masuda, M.; Shimizu, T. Confinement effect of organic nanotubes toward green fluorescent protein (GFP) depending on the inner diameter size (retracted article). *Chem. Eur. J.* **2010**, *16* (14), 4217-4223.

(19) Brites, C. D. S.; Marin, R.; Suta, M.; Neto, A. C. N.; Ximendes, E.; Jaque, D.; Carlos, L. D. Spotlight on luminescence thermometry: Basics, challenges, and cutting-edge applications. *Adv. Mater.* **2023**, *35* (36), 2302749.
(20) Brites, C. D. S.; Millán, A.; Carlos, L. D. Lanthanides in luminescent thermometry. In *Handbook on the physics and chemistry of rare earths*, Bünzli, J.-C. G., Pecharsky, V. K. Eds.; Vol. 49; Elsevier Science, B. V., 2016; pp 339-427.
(21) Brites, C. D. S.; Zhuang, B. L.; Debasu, M. L.; Ding, D.; Qin, X.; Maturi, F. E.; Lim, W. W. Y.; Soh, D.; Rocha, J.; Yi, Z. G.; et al. Decoding a percolation phase transition of water at ~330 K with a nanoparticle ruler. *J. Phys. Chem. Lett.* **2020**, *11* (16), 6704-6711.
(22) Maturi, F. E.; Raposo Filho, R. S.; Brites, C. D. S.; Fan, J.; He, R.; Zhuang, B.; Liu, X.; Carlos, L. D. Deciphering density fluctuations in the hydration water of Brownian nanoparticles via upconversion thermometry. *J. Phys. Chem. Lett.* **2024**, *15* (9), 2606-2615.
