# Supplementary material for: Exploring Water Beyond the Solvent: Insights into Density Fluctuations and EGFP Unfolding via Luminescence Thermometry
Source: J Phys Chem B. 2025 Nov 6;129(46):12042–50. doi: 10.1021/acs.jpcb.5c06143 (PMC12772516; doi:10.1021/acs.jpcb.5c06143)
Supplement: Supplementary file 2 [file jp5c06143_si_002.pdf]

Concentration (μM)	0.36									
Temperature (°C)	30									
Position, x_i	x_1		x_2		x_3		x_4		x_5	
	t (s)	P (cm^{-1})	t (s)	P (cm^{-1})	t (s)	P (cm^{-1})	t (s)	P (cm^{-1})	t (s)	P (cm^{-1})
	0	19610.52	0	19613.24	0	19616.7	0	19616.38	0	19617.41
	0.66251	19616.64	0.66113	19615.72	0.66151	19608.83	0.66234	19615.06	0.73148	19620.04
	0.85609	19609.04	0.85816	19609.36	0.85885	19613.89	0.85838	19620.5	0.87183	19614.93
	1.11239	19611.96	1.10996	19620.53	1.10707	19619.67	1.10482	19612.96	1.10717	19617.04
	1.35418	19609.8	1.35756	19614.56	1.35378	19619.32	1.35653	19617.41	1.35563	19615.88
	1.60719	19617	1.60448	19614.85	1.60338	19615.98	1.60387	19613.48	1.60722	19618.82
	1.85481	19612.04	1.85608	19608.84	1.85897	19619.33	1.85618	19612.44	1.85557	19620.59
	2.10882	19619.98	2.11073	19618.14	2.10575	19615.68	2.10416	19616	2.10414	19614.67
	2.35495	19618.24	2.35558	19618.52	2.35523	19613.95	2.35626	19615.53	2.35512	19617.33
	2.60629	19614.78	2.60372	19615.65	2.60445	19613.5	2.60339	19611.81	2.60554	19620.9
	2.85393	19610.3	2.85669	19611.13	2.8583	19612.99	2.85611	19616.6	2.85631	19614.07
	3.10804	19611.19	3.13196	19615.64	3.11132	19619.27	3.10486	19618.91	3.10514	19619.02
	3.35556	19611.5	3.35872	19612.48	3.35495	19615.49	3.35807	19617.64	3.35456	19612.72
	3.60699	19608.69	3.60607	19616.24	3.60492	19614.43	3.60446	19611.89	3.60478	19615.52
	3.85388	19604.06	3.85562	19614.61	3.85592	19609.83	3.85776	19616.86	3.85509	19616.03
	4.11105	19611.51	4.10934	19616.83	4.10475	19608.21	4.10336	19611.79	4.10596	19615.63
	4.35665	19611.79	4.35751	19614.32	4.35579	19607.91	4.3605	19616.76	4.35579	19614.07
	4.60789	19613.27	4.60533	19612.75	4.60973	19608.24	4.6054	19617.34	4.60625	19616.42
	4.85487	19613.76	4.85676	19610.04	4.86018	19615.58	4.8577	19622.15	4.85573	19615.46
	5.10764	19608.56	5.10974	19610.05	5.10577	19611.77	5.10563	19615.1	5.10703	19617.97
	5.35549	19609.88	5.35726	19614.02	5.35482	19614.16	5.35875	19613.88	5.35679	19619.66
	5.61003	19612.42	5.60557	19613.18	5.60538	19617.69	5.6064	19615.31	5.6053	19618.22
	5.85856	19617.61	5.8599	19618.67	5.85959	19606.99	5.85667	19615.91	5.85532	19611.68
	6.1085	19612.5	6.11023	19615.68	6.10587	19611.9	6.10552	19620.93	6.10555	19616.41
	6.36168	19608.13	6.35929	19613.84	6.35585	19615.24	6.35845	19618.97	6.35679	19617.47
	6.60789	19609.2	6.60657	19616.84	6.60556	19620.16	6.6049	19613.39	6.60781	19613.97
	6.85706	19614.43	6.85865	19617	6.86034	19615.6	6.85748	19608.25	6.85564	19615.65
	7.11274	19610.62	7.11165	19611.95	7.10657	19612.79	7.10561	19616.77	7.10525	19621.54
	7.35716	19610.91	7.35709	19615.47	7.35511	19619.16	7.35783	19615.29	7.35567	19620.68
	7.60669	19616.26	7.60596	19607.85	7.60495	19617.12	7.60607	19619	7.60785	19619.34
	7.85576	19613.64	7.85767	19611.92	7.85976	19620.89	7.85848	19616.87	7.8566	19614.09
	8.11113	19613.12	8.11218	19611.04	8.10665	19619.73	8.10596	19611.6	8.10586	19614.88
	8.35822	19610.65	8.35841	19610.62	8.35727	19614.71	8.35818	19618.27	8.3572	19612.82
	8.60778	19612.99	8.60804	19615.82	8.60521	19619.34	8.60593	19611.6	8.60877	19618.36
	8.85595	19611.08	8.85804	19614.24	8.85966	19617.28	8.86026	19608.48	8.85721	19620.29
	9.13156	19613.45	9.11206	19616.72	9.10719	19614.72	9.10579	19613.99	9.10587	19616.94
	9.35778	19615.12	9.35912	19613.7	9.37046	19611.42	9.35787	19617.54	9.35658	19618.38
	9.60819	19609.42	9.6066	19610.74	9.60669	19611.22	9.60541	19613.44	9.61217	19616.44
	9.85604	19612.41	9.85794	19608.3	9.85972	19611.6	9.85836	19617.51	9.85764	19620.85
	10.10936	19615.76	10.11184	19612.69	10.10697	19612.22	10.10714	19615.51	10.1067	19617.49
	10.35951	19614.55	10.36056	19614.81	10.357	19615.79	10.35888	19614.4	10.35972	19611.24
	10.61057	19609.94	10.60735	19611.66	10.60786	19613.8	10.60717	19612.06	10.60917	19615.07
	10.85766	19610.39	10.85853	19614.85	10.86057	19611.62	10.85989	19615.37	10.85888	19618.22
	11.11092	19604.43	11.11391	19614.72	11.10808	19612.74	11.10817	19619.57	11.10843	19610.92
	11.35861	19614.21	11.3623	19612.53	11.357	19611.63	11.36023	19616.2	11.35862	19617.28
	11.6105	19616.34	11.60861	19613.84	11.60777	19619.07	11.60686	19619.37	11.60831	19614.04
	11.85887	19617.74	11.85933	19610.11	11.86193	19614.73	11.85905	19617.18	11.85774	19617.78
	12.11225	19610.94	12.11265	19614.06	12.10827	19614.05	12.10832	19615.79	12.1093	19616.06
	12.35743	19608.46	12.36047	19618.02	12.35771	19615.43	12.36093	19607.77	12.35999	19617.95
	12.60958	19608.13	12.60853	19614.26	12.60694	19607.69	12.60745	19615.92	12.6082	19612.89
	12.85849	19618.51	12.85987	19617.54	12.85921	19620.97	12.85861	19617.93	12.8576	19618.24
	13.11264	19609.02	13.11268	19617.78	13.10872	19616.27	13.10789	19612.69	13.10831	19618.25
	13.35853	19608.59	13.36004	19609.64	13.35755	19611.87	13.36236	19616.3	13.36008	19609.11
	13.60991	19616.07	13.60795	19621.25	13.60664	19615.23	13.60805	19614.1	13.6082	19617.3
	13.85802	19615.81	13.85996	19617.22	13.85999	19610.05	13.86281	19612.21	13.85831	19622.33
	14.11237	19610.26	14.1151	19608.57	14.10934	19615.14	14.10861	19609.84	14.10798	19613.22
	14.35945	19610.49	14.36002	19609.86	14.35848	19612.39	14.35985	19619	14.35911	19612.95
	14.61124	19607.98	14.60852	19619.5	14.60731	19613.26	14.60903	19615.21	14.61071	19618.55
	14.85743	19612.19	14.8604	19613.53	14.86214	19616.27	14.86088	19617.77	14.85927	19619.14
	15.11235	19614.73	15.11419	19614.57	15.1115	19617.28	15.10904	19609.79	15.10821	19619.28
	15.36359	19611.08	15.36162	19610.9	15.35926	19611.98	15.36076	19616.86	15.36057	19617.15
	15.61197	19612.33	15.60888	19611.47	15.61009	19612.19	15.60892	19612.47	15.60989	19616.96
	15.8607	19614.76	15.86109	19619.08	15.86072	19618.7	15.86093	19610.07	15.86149	19622.92
	16.11258	19611.6	16.11518	19610.12	16.11019	19613.28	16.11062	19617.15	16.11053	19614.67

16.35944	19607.1	16.36454	19614.14	16.35987	19619.04	16.3625	19617.36	16.3601	19613.34
16.61122	19612.11	16.61023	19610.66	16.61002	19618.81	16.60845	19619.59	16.60902	19622.71
16.85916	19611.71	16.86041	19614.94	16.86148	19609.36	16.86054	19611.69	16.86738	19615
17.11147	19614.04	17.11374	19613.97	17.11101	19610.57	17.10902	19613.69	17.11082	19617.53
17.35887	19612.03	17.36161	19612.39	17.35822	19612.98	17.36648	19616.03	17.35922	19618.37
17.61221	19613.23	17.61016	19612.39	17.60921	19614.59	17.60933	19616.24	17.61022	19612.09
17.86044	19610.13	17.86133	19616.87	17.86379	19618.08	17.8626	19617.13	17.86002	19614.98
18.11235	19609.31	18.11391	19616.7	18.11063	19620.29	18.10831	19611.93	18.11154	19615.15
18.35898	19613.31	18.36147	19614.81	18.35903	19616.1	18.36196	19615.85	18.36028	19616.36
18.61138	19613.68	18.61079	19617.12	18.61349	19615.87	18.60989	19620.54	18.61001	19614.4
18.86019	19614.34	18.86313	19615.89	18.86264	19608.27	18.86381	19612.35	18.86007	19616.58
19.11651	19615.81	19.11517	19607.5	19.11136	19609.87	19.10957	19615.37	19.11072	19621.34
19.35987	19609.53	19.36161	19611.24	19.35997	19613.4	19.36185	19610.73	19.36135	19618.08
19.61187	19610.35	19.60926	19619.72	19.60971	19617.23	19.61054	19622.81	19.61406	19616.47
19.86144	19607.65	19.86207	19611.22	19.86552	19616.56	19.86287	19617.48	19.85993	19613.17
20.11389	19612.84	20.11599	19615.39	20.11162	19610.17	20.10913	19620.56	20.1103	19620.66
20.36181	19609.66	20.36325	19612.42	20.3618	19614.24	20.36238	19617.07	20.36216	19616.31
20.61425	19611.71	20.61114	19621.78	20.61053	19611.95	20.61066	19613.5	20.61269	19610.57
20.86097	19612.55	20.86399	19610.53	20.86462	19617.97	20.86339	19617.41	20.86182	19615.73
21.11431	19612.51	21.11674	19612.59	21.11197	19616.95	21.1124	19619.85	21.11097	19612.5
21.3623	19609.07	21.36634	19614.66	21.36193	19613.75	21.36266	19621.53	21.36146	19614.36
21.61527	19613.61	21.61097	19609.88	21.61176	19616.75	21.61063	19619.17	21.61339	19611.54
21.8618	19609.79	21.86401	19605.69	21.86272	19620.6	21.86279	19613.78	21.86348	19622.63
22.11413	19607.69	22.11676	19613.41	22.11153	19620.86	22.11213	19614.14	22.11177	19619.12
22.36186	19610.49	22.36451	19617.72	22.36094	19616.73	22.36446	19615.27	22.36161	19619.62
22.61505	19619.2	22.61269	19611.48	22.61192	19615.47	22.61159	19621.35	22.61212	19614.15
22.86254	19614.48	22.86294	19610.29	22.86481	19621.27	22.86357	19611.82	22.8643	19616.07
23.11669	19618.66	23.13897	19606.28	23.11348	19612.7	23.11236	19618.6	23.11391	19615.49
23.36265	19612.14	23.36504	19616	23.36113	19619.11	23.36428	19615.12	23.36154	19618.86
23.61533	19613.96	23.61321	19612.83	23.61111	19615.41	23.61329	19608.57	23.61475	19614.84
23.8635	19613.33	23.86486	19612.02	23.86513	19620.89	23.86739	19616.26	23.86297	19618.42
24.13588	19615.44	24.1164	19611.18	24.11303	19618.57	24.11187	19614.62	24.11529	19615.86
24.36399	19609.38	24.36551	19610.85	24.36333	19616.09	24.36499	19616.45	24.36331	19619.84
24.61382	19611.65	24.61368	19612.91	24.61101	19610.22	24.61187	19615.17	24.61206	19617.72
24.86206	19611.42	24.86456	19611.93	24.86162	19620.01	24.86512	19616.27	24.86281	19613.58
25.11825	19613.73	25.11613	19613.14	25.11258	19618.94	25.11105	19614.82	25.11489	19611.71
25.3643	19610.05	25.36479	19611.72	25.36315	19609.28	25.36465	19612.18	25.36328	19620.78
25.61522	19612.4	25.61264	19616.31	25.61256	19615.28	25.61184	19622.26	25.61164	19618.61
25.8617	19613.72	25.86522	19614.81	25.8618	19614.52	25.8651	19617.37	25.86302	19615.51
26.11514	19610.79	26.11925	19613.1	26.11355	19613.74	26.11283	19611.15	26.11367	19619.83
26.36227	19607.37	26.36734	19613.98	26.36253	19618.02	26.36415	19612.06	26.36268	19613.75
26.6158	19610.21	26.61173	19610.46	26.61295	19622.47	26.61139	19617.34	26.61224	19615.96
26.86244	19614.26	26.86502	19612.13	26.86238	19618.86	26.86449	19613.18	26.86283	19622.13
27.11489	19612.19	27.11769	19618.39	27.11293	19617.25	27.11327	19614.48	27.11456	19619.7
27.36313	19610.54	27.36585	19611.43	27.36398	19615.3	27.36514	19617.88	27.36508	19616.35
27.61614	19611.49	27.61232	19615.79	27.61419	19613.06	27.61287	19617.15	27.6138	19613.57
27.8677	19609.74	27.86519	19610.71	27.86409	19613.85	27.86438	19608.88	27.86304	19613.44
28.11619	19610.13	28.12105	19607.83	28.11326	19615.26	28.11304	19610.58	28.11355	19620.81
28.36255	19609.58	28.36739	19615.69	28.37832	19610.83	28.36546	19612.63	28.36423	19618.55
28.61608	19611.65	28.61365	19617.18	28.6134	19615.81	28.61328	19618.86	28.61456	19613.28
28.86447	19607.57	28.86491	19611.27	28.86513	19620.67	28.86613	19618.36	28.86269	19619.11
29.11901	19609.8	29.11833	19620.21	29.11441	19611.79	29.11265	19617.6	29.11354	19617.03
29.3636	19614.98	29.367	19610.77	29.36356	19616.84	29.3662	19610.61	29.36361	19611.88
29.61695	19610.98	29.61489	19619.53	29.61302	19610.96	29.61391	19620.68	29.61746	19617.24
29.86376	19614.05	29.87036	19618.52	29.86607	19614.28	29.86553	19611.67	29.8649	19612.79
30.11714	19612.65	30.1184	19623.56	30.11517	19614.53	30.11336	19612.78	30.11361	19617.84
30.37	19613.46	30.36793	19614.25	30.36569	19618.72	30.36757	19616.96	30.36528	19611.36
30.61835	19617.21	30.616	19613.24	30.614	19607.74	30.61591	19616.48	30.61686	19616.02
30.86521	19616.14	30.86737	19611.07	30.86512	19615.44	30.86816	19619.15	30.86616	19612.66
31.11899	19607.82	31.12035	19612.92	31.11496	19607.8	31.11796	19617.86	31.11511	19616.88
31.36604	19613.34	31.37011	19614.12	31.36577	19613.62	31.36886	19615.52	31.36613	19618.36
31.61831	19611.3	31.61537	19609.31	31.61551	19612.16	31.61816	19613.57	31.61758	19620.98
31.8651	19611.35	31.86757	19611.26	31.86564	19614.15	31.87845	19614.03	31.86659	19616.37
32.11802	19614.13	32.12152	19612.46	32.11729	19613.57	32.1181	19615.69	32.11639	19611.91
32.36686	19611.31	32.36888	19611.08	32.36576	19614.61	32.37136	19612.98	32.36665	19623
32.61945	19612.52	32.61521	19614.65	32.61518	19617.37	32.61761	19612.14	32.61586	19618.41
32.86975	19613.45	32.86722	19621.82	32.86584	19611.45	32.87708	19614.81	32.86563	19621.9
33.11719	19609.98	33.12105	19606.08	33.11523	19617.75	33.11649	19616.18	33.11614	19614.77
33.36645	19614.09	33.3702	19610.17	33.36459	19619	33.36824	19611.93	33.36566	19616.1
33.61918	19614.68	33.61551	19612.06	33.61509	19610.72	33.61467	19611.18	33.61724	19610.59

33.86561	19612.72	33.8699	19614.33	33.86686	19616.67	33.86942	19615.76	33.86543	19610.15
34.11935	19614.74	34.12846	19617.69	34.11711	19613.46	34.11527	19614.41	34.1167	19618.49
34.36532	19614.71	34.36842	19612.17	34.36525	19616.86	34.36982	19616.55	34.36643	19616.7
34.61827	19617.93	34.61633	19611.62	34.61525	19609.98	34.61498	19620.54	34.61631	19614.66
34.86654	19611.08	34.86837	19613.6	34.86675	19615.37	34.86654	19616.16	34.8655	19617.83
35.11904	19613.33	35.12024	19613.35	35.11733	19611.31	35.11515	19608.66	35.11708	19618.72
35.36588	19614.16	35.36793	19619.96	35.36623	19611.68	35.37065	19618.56	35.36681	19623.78
35.6188	19610.73	35.61672	19612.38	35.61494	19623.22	35.61534	19615.4	35.61746	19618.69
35.86694	19610.94	35.86942	19612.75	35.86601	19617.41	35.86778	19613.61	35.86672	19621.91
36.11951	19613.23	36.12022	19609.72	36.11771	19614.69	36.1155	19614.48	36.1161	19617.37
36.36662	19616.1	36.37089	19610.27	36.3678	19616.74	36.36977	19614.71	36.36682	19613.54
36.61875	19614.42	36.6159	19612.86	36.61699	19619.66	36.61726	19610.94	36.61969	19611.08
36.86683	19611.09	36.86883	19610.33	36.86665	19609.07	36.86778	19616.84	36.86761	19617.51
37.11936	19609.8	37.1214	19607.17	37.11772	19616.26	37.1158	19613.95	37.11667	19619.52
37.36666	19611.77	37.36964	19612.35	37.36728	19614.39	37.36915	19612.65	37.36682	19615.1
37.62119	19612.15	37.61623	19614.92	37.62564	19612.03	37.6172	19617.92	37.6192	19619.25
37.86662	19619.52	37.86853	19609.63	37.86719	19613.89	37.87001	19618.54	37.86873	19618.88
38.11841	19614.23	38.12314	19619.54	38.11768	19606.06	38.11591	19616.57	38.11832	19617.69
38.3748	19609.02	38.37076	19616.7	38.36793	19615.72	38.37028	19612.88	38.36675	19615.19
38.62037	19610.03	38.61728	19617.9	38.61696	19621.56	38.61773	19618.78	38.61872	19616.15
38.86894	19614.55	38.87045	19612.19	38.86952	19616.01	38.87224	19612.73	38.86947	19622.76
39.12197	19608.3	39.12242	19613.19	39.12331	19615.59	39.11632	19614	39.11885	19617.55
39.36658	19613.75	39.37018	19614.86	39.36761	19616.69	39.36888	19612.45	39.36653	19614.61
39.62048	19620.25	39.61868	19618.77	39.61635	19616.86	39.61626	19617.41	39.6268	19622.84
39.86905	19611.02	39.87001	19616.31	39.86816	19611.46	39.86924	19614.35	39.86778	19614.27
40.12132	19613.03	40.12048	19612.33	40.11978	19612.9	40.11791	19621.1	40.11926	19613.27
40.36802	19605.97	40.37171	19615.13	40.36951	19615.71	40.37143	19614.81	40.36984	19615.61
40.6201	19614.5	40.62068	19610.17	40.61782	19612.23	40.61784	19614.21	40.62018	19619.18
40.86898	19612.3	40.87196	19613.73	40.86853	19618.92	40.87148	19614.26	40.86935	19616.76
41.12191	19617.68	41.125	19613.26	41.1225	19614.01	41.11979	19617.19	41.12119	19617.91
41.36794	19610.12	41.37803	19606.01	41.37058	19609.29	41.37776	19612.2	41.37051	19615.32
41.62111	19613.79	41.61913	19614.26	41.61962	19609.11	41.62006	19615.27	41.61923	19618.24
41.86935	19613.11	41.87321	19615.7	41.87041	19617.31	41.87125	19610.81	41.87011	19615.76
42.1228	19611.14	42.12359	19611.83	42.12034	19612.94	42.11841	19611.08	42.12222	19615.6
42.37072	19616.85	42.37231	19607.05	42.37034	19615.94	42.37169	19608.71	42.37116	19606.89
42.62482	19609.6	42.61929	19610.35	42.62045	19610.54	42.62023	19608.82	42.63006	19619.29
42.8736	19608.27	42.87241	19612.5	42.87147	19610.98	42.87222	19618.41	42.86962	19620.1
43.12352	19612.51	43.14966	19618.51	43.11932	19608.64	43.12072	19615.79	43.12148	19619.66
43.3692	19606.93	43.37401	19613.15	43.37049	19613.18	43.37207	19613.64	43.37078	19616.88
43.62195	19611.49	43.62009	19616.1	43.62049	19611.29	43.61958	19611.07	43.62021	19615.05
43.87074	19609.68	43.87171	19616.31	43.87062	19614.21	43.87524	19615.38	43.86991	19615.83
44.12529	19615.1	44.12492	19610	44.1208	19617.37	44.1209	19615.46	44.12095	19620.38
44.37034	19613.09	44.37322	19610.26	44.37125	19619.24	44.37306	19613.07	44.37185	19613.87
44.62296	19611.08	44.62127	19612.21	44.62006	19616.54	44.62008	19613.93	44.62826	19614.12
44.87117	19607.38	44.87402	19613.68	44.87222	19615.54	44.87194	19614.73	44.87118	19610.67
45.12419	19618.82	45.12317	19611.26	45.1221	19622.51	45.12031	19619.04	45.12118	19613.7
45.37045	19609.17	45.37335	19617.71	45.37151	19605.18	45.37324	19619.2	45.37064	19614.44
45.6229	19609.69	45.62127	19613.15	45.62205	19610.3	45.62074	19613.66	45.62336	19617.29
45.87073	19615.92	45.87347	19616.59	45.87175	19616.92	45.87287	19614.71	45.87275	19606.96
46.12443	19608.44	46.12537	19613.05	46.12154	19614.17	46.1203	19614.76	46.12162	19621.96
46.37158	19613.77	46.37627	19610.71	46.37176	19613.17	46.37304	19614.62	46.37067	19618.39
46.62275	19618.22	46.62059	19612.29	46.61966	19609.62	46.62172	19616.38	46.62363	19619.19
46.87073	19619.76	46.87385	19609.08	46.87362	19620.13	46.87428	19619.8	46.87388	19611.96
47.12467	19613.78	47.12596	19617.13	47.12296	19609.19	47.12035	19616.61	47.12203	19611.94
47.37254	19612.42	47.37537	19611.48	47.37259	19616.9	47.3743	19616.12	47.37222	19612.77
47.62535	19612.65	47.62194	19611.23	47.62181	19619.55	47.62243	19617.97	47.62377	19613.91
47.8733	19617.53	47.87414	19605.72	47.87246	19612.84	47.87486	19614.97	47.87512	19619.84
48.12332	19620.12	48.12732	19616.23	48.12224	19620.28	48.12085	19619.04	48.12734	19619.42
48.37154	19610.16	48.37515	19610.82	48.37313	19612.31	48.37421	19619.12	48.372	19619.95
48.62382	19606.5	48.62236	19616.51	48.62166	19605	48.62135	19620.55	48.62378	19613.37
48.87323	19613.11	48.87441	19615.57	48.87303	19616.04	48.87636	19614.22	48.87286	19615.33
49.12494	19613.65	49.1244	19614.33	49.12053	19614.09	49.12022	19614.02	49.12332	19613.08
49.37054	19608.29	49.37405	19613.55	49.36982	19612.06	49.37342	19616.54	49.37174	19613.11
49.62376	19608.51	49.62316	19612.16	49.62093	19614.19	49.62159	19615.8	49.62126	19620.42
49.8731	19615.1	49.87395	19610.8	49.87269	19611.03	49.87977	19622.19	49.87217	19612.32
50.12314	19610.62	50.12467	19616.15	50.12179	19615.73	50.12205	19617.4	50.12257	19619.04
50.3715	19611.4	50.37451	19607.65	50.3716	19620.5	50.37358	19619.41	50.37208	19618.81
50.62364	19611.96	50.62411	19619.45	50.62021	19613.77	50.6206	19612.01	50.62096	19617.69
50.87358	19608.64	50.87546	19616.19	50.87243	19610.23	50.87309	19620.19	50.87322	19620.45
51.12546	19613.25	51.12453	19612.07	51.12182	19617.55	51.12294	19613.17	51.12282	19622.43

51.37073	19616.81	51.37572	19611.58	51.3722	19616.5	51.37521	19614.95	51.37247	19614.51
51.62504	19607.05	51.62381	19609.33	51.62074	19614.27	51.62159	19617.96	51.62422	19615.62
51.87319	19611.72	51.87477	19610.84	51.87074	19615.93	51.87306	19612.8	51.87575	19612.74
52.1368	19608.63	52.12586	19616.17	52.12246	19614.25	52.12102	19615.41	52.12136	19624
52.37236	19608.42	52.37566	19615.65	52.37443	19617.94	52.37521	19612.52	52.37261	19619.07
52.62591	19615.17	52.62323	19613.08	52.62216	19618.04	52.62367	19613.93	52.62563	19612.28
52.87376	19609.78	52.87484	19614.28	52.87338	19617.91	52.87416	19612.85	52.87351	19611.62
53.12505	19614.95	53.12716	19612.99	53.12264	19619.9	53.12067	19614.17	53.12382	19615.44
53.37335	19613.86	53.3768	19610.65	53.37322	19616.3	53.37539	19616.3	53.37283	19618.38
53.62597	19609.13	53.62356	19613.5	53.62662	19621.39	53.62381	19616.14	53.62413	19620.45
53.87278	19609.08	53.87449	19614.4	53.87411	19620.25	53.87869	19618.71	53.87316	19616.29
54.12708	19614.31	54.12615	19615.51	54.12353	19615.23	54.12178	19617.06	54.12496	19619.64
54.37257	19615.89	54.37721	19611.46	54.3764	19617.13	54.37585	19616.65	54.37315	19617.33
54.62603	19614.5	54.62439	19614.78	54.62229	19621.79	54.6237	19613.82	54.62669	19608.51
54.87417	19617.7	54.87478	19616.58	54.87491	19617.82	54.8762	19616.4	54.87384	19621.49
55.12524	19614.5	55.12624	19618.82	55.12474	19613.41	55.12216	19617.77	55.12542	19615.34
55.37209	19616.76	55.3757	19616.65	55.37444	19617.69	55.37615	19616.13	55.37347	19615.01
55.62556	19610.93	55.62435	19618.42	55.62264	19614.55	55.62313	19616.42	55.62552	19614.58
55.87525	19613.6	55.87732	19614.93	55.87393	19613.75	55.87587	19615.03	55.87562	19612.94
56.12739	19613.23	56.12738	19615.06	56.12517	19616.08	56.12355	19615.61	56.12344	19614.83
56.37363	19604.8	56.37923	19616.02	56.3753	19618.36	56.37616	19613.35	56.37363	19618.54
56.62571	19610.96	56.62343	19613.25	56.62338	19612	56.6247	19618.81	56.62609	19616.92
56.87374	19611.01	56.87664	19610.88	56.87309	19611.46	56.87566	19620.4	56.87511	19618.98
57.12717	19612.6	57.12584	19611.15	57.12743	19617.32	57.12392	19617.69	57.12383	19612.12
57.37472	19616.84	57.37567	19619.89	57.37612	19618.43	57.37811	19611.55	57.37374	19615.35
57.62695	19609.12	57.62383	19613.19	57.62419	19608.36	57.62403	19621.51	57.62613	19613.75
57.8733	19607.93	57.87769	19614.79	57.87378	19615.3	57.87647	19615.76	57.87593	19619.83
58.12602	19618.68	58.12594	19613.27	58.12461	19615.14	58.12523	19613.93	58.12429	19617.36
58.37445	19610.57	58.37774	19606.93	58.37767	19618.31	58.37806	19612.73	58.37339	19620.28
58.62725	19609.44	58.62407	19612.64	58.62475	19613.66	58.62466	19606.37	58.62666	19618.58
58.87566	19613.96	58.87761	19611.76	58.87466	19612.88	58.87813	19618.67	58.87702	19615.42
59.13125	19609.43	59.13078	19611.87	59.12481	19613.65	59.12418	19608.41	59.12588	19610.75
59.37377	19608.39	59.37854	19612.3	59.37582	19613.86	59.37728	19617.63	59.37441	19623.09
59.62744	19614.23	59.62593	19615.1	59.62606	19619.7	59.62593	19619.09	59.62524	19613.58
59.87789	19612.19	59.88153	19617.89	59.87841	19619.85	59.87854	19617.69	59.87905	19608.79
60.13146	19613.45	60.12511	19613.21	60.12576	19620.06	60.12447	19609.89	60.127	19613.01
60.37623	19614.69	60.37975	19611.89	60.3789	19611.54	60.37891	19614.41	60.37647	19621.74
60.62918	19613.21	60.62841	19613.68	60.6264	19621.13	60.62768	19613.92	60.62876	19612.78
60.8765	19610.15	60.88099	19610.38	60.87878	19615.47	60.87931	19615.04	60.87724	19618.62
61.13033	19609.78	61.12767	19611.87	61.12818	19616.32	61.1259	19611.15	61.12933	19617.33
61.37782	19610.44	61.38164	19611.89	61.37977	19609.24	61.37943	19612.06	61.38094	19616.96
61.63137	19615.51	61.62712	19608.78	61.62579	19611.91	61.62769	19614.41	61.62882	19619.2
61.87692	19615.06	61.88061	19612.9	61.87687	19615.21	61.8809	19609.55	61.87718	19614.81
62.12903	19609.55	62.13086	19618.6	62.12984	19617.93	62.12773	19618.6	62.12872	19612.18
62.37797	19608.09	62.37874	19612.84	62.38182	19610.69	62.37961	19617.07	62.37973	19616.19
62.63118	19612.12	62.62887	19611.62	62.62711	19614.54	62.62687	19616.35	62.62717	19618.12
62.878	19611.14	62.88193	19611.95	62.87694	19612.44	62.88027	19616.63	62.87785	19615.98
63.1326	19613.48	63.15123	19613.72	63.12835	19616.27	63.12815	19621.13	63.12843	19620.91
63.37747	19610.31	63.38175	19617.89	63.38147	19609.93	63.38831	19608.11	63.3794	19616.46
63.63038	19617.85	63.6268	19609.25	63.62841	19615.46	63.62627	19614.69	63.63026	19616.81
63.87881	19606.77	63.88027	19611.51	63.87687	19613.55	63.88325	19613.02	63.87826	19618.9
64.1334	19612.13	64.13028	19601.89	64.12911	19619.4	64.12763	19616.67	64.12866	19615.94
64.38101	19607.38	64.38254	19613.51	64.38112	19619.03	64.38116	19608.99	64.37966	19610.41
64.62844	19607.64	64.62779	19611.69	64.62673	19612.54	64.62681	19618.09	64.62904	19617.77
64.87817	19612.41	64.87896	19610.63	64.87694	19615.03	64.87884	19615.56	64.87796	19608.99
65.13143	19609.94	65.12879	19613.67	65.12745	19618.28	65.12548	19604.87	65.12687	19616.03
65.37708	19613.89	65.37981	19606.06	65.37754	19615.62	65.37851	19614.89	65.37668	19613.93
65.62827	19607.35	65.62835	19607.03	65.62865	19610.07	65.62718	19622.55	65.62966	19617
65.87663	19609.55	65.88124	19614.22	65.87919	19616.94	65.88105	19615.27	65.87836	19617.02
66.13185	19611.05	66.12688	19618.74	66.12794	19617.58	66.12689	19611.52	66.12843	19619.09
66.37867	19614.35	66.3822	19610.82	66.37701	19612.76	66.38025	19622.61	66.3766	19613.96
66.63058	19611.13	66.62751	19607.92	66.62732	19619.57	66.62739	19615.87	66.6299	19618.89
66.87763	19616.88	66.88072	19614.34	66.88038	19618.39	66.88036	19611.14	66.87879	19616.62
67.13175	19614.4	67.12967	19605.29	67.12929	19614.47	67.12729	19616.31	67.13007	19616.14
67.37855	19616.27	67.38001	19615.57	67.38389	19620.09	67.37922	19613.19	67.37866	19618.8
67.63174	19606.49	67.62687	19611.12	67.62627	19612.58	67.62739	19613.99	67.62938	19616.46
67.87763	19616.3	67.8818	19611.45	67.8783	19611.22	67.8809	19616.34	67.87785	19615.59
68.13135	19615.03	68.13036	19612.2	68.12963	19617.38	68.12924	19611.59	68.13097	19615.29
68.3788	19611.74	68.3826	19614.31	68.37944	19620.21	68.39117	19616.84	68.37921	19611.06
68.63134	19606.8	68.62791	19615.38	68.62996	19611.2	68.62737	19615.68	68.63261	19618.64

68.8799	19613.55	68.88161	19623.54	68.87808	19617.14	68.88067	19615.66	68.87851	19610.01
69.13432	19612.35	69.13663	19613.68	69.12924	19618.72	69.12833	19610.8	69.1305	19614.78
69.37869	19612.1	69.38222	19615.18	69.37792	19614.95	69.38183	19609.99	69.37979	19622.47
69.6316	19612.31	69.62922	19614.67	69.62924	19613.89	69.6284	19615.81	69.63358	19616.78
69.87911	19613.12	69.88405	19618.57	69.87886	19615.8	69.88022	19613.67	69.87931	19613.38
70.13261	19611.74	70.13058	19609.69	70.12993	19609.54	70.12776	19612.92	70.13122	19616.68
70.38238	19604.58	70.38182	19618.55	70.38019	19618.75	70.38086	19615.18	70.3817	19620.69
70.63097	19609.75	70.63019	19618.15	70.62963	19612.32	70.62932	19610.14	70.63122	19620.38
70.87927	19608.6	70.88532	19611.01	70.87974	19615.5	70.88296	19616.23	70.88062	19621.94
71.1339	19609.56	71.12999	19614.96	71.12973	19619.13	71.12734	19619.28	71.13014	19616.93
71.37906	19613.78	71.38332	19608.27	71.38065	19619.62	71.38183	19610.44	71.37944	19615.76
71.63266	19617.78	71.63007	19616.77	71.63125	19617.94	71.62911	19616.98	71.63288	19616.19
71.87863	19613.6	71.88299	19617.38	71.88126	19621.95	71.88246	19619.67	71.88063	19614.88
72.13351	19610.97	72.12998	19621.31	72.12928	19613.32	72.12915	19616.08	72.13041	19617.49
72.37997	19607.27	72.38132	19621.23	72.38024	19618.87	72.3817	19610.83	72.37956	19611.71
72.6316	19616.54	72.62944	19615.16	72.63032	19616.24	72.62832	19614.02	72.63114	19615.38
72.88001	19613.62	72.88479	19614.87	72.88334	19612.12	72.8823	19612.61	72.88179	19614.88
73.13321	19611.49	73.13219	19613.62	73.1318	19611.76	73.13133	19613.5	73.13176	19613.28
73.39196	19611.95	73.38357	19619.33	73.38002	19610.78	73.39004	19612.73	73.37918	19616.99
73.63334	19611	73.62894	19609.62	73.62939	19618.31	73.62991	19620.62	73.63233	19617.35
73.87994	19613.35	73.88485	19607.52	73.88236	19614.2	73.88576	19617.51	73.88198	19617.64
74.13438	19618.63	74.13185	19609.75	74.13325	19618.62	74.1314	19612.56	74.13601	19616.32
74.38019	19618.43	74.38435	19613.69	74.38065	19614.27	74.38346	19620.36	74.38135	19614.55
74.63386	19614.41	74.62998	19614.84	74.62879	19617.76	74.63086	19611.35	74.63249	19617.47
74.88729	19614.01	74.88532	19610.73	74.88085	19614.05	74.88464	19612.73	74.88115	19617.69
75.13471	19610.44	75.13196	19607.35	75.13439	19616.78	75.13084	19615.52	75.13411	19616.28
75.3799	19607.74	75.38556	19612.72	75.38204	19613.05	75.38528	19614.14	75.38361	19617.58
75.63298	19614.94	75.63139	19614.07	75.63463	19616.73	75.63143	19611.04	75.63375	19618.81
75.8816	19613.28	75.88656	19615.26	75.88046	19614.81	75.88387	19613.54	75.88273	19614.38
76.13626	19612.39	76.13222	19613.17	76.13253	19611.77	76.13022	19611.2	76.13301	19614.7
76.38119	19613.53	76.38767	19610.34	76.3825	19617.04	76.38303	19620.15	76.38365	19622.15
76.63387	19608.62	76.63165	19619.72	76.63361	19614.11	76.63182	19616.01	76.63472	19609.48
76.88152	19614.05	76.88501	19612.34	76.88157	19619.32	76.88506	19614.72	76.88285	19613.68
77.13512	19606.42	77.13634	19615	77.13233	19612.3	77.12969	19615.02	77.1345	19618.82
77.38306	19609.17	77.38369	19614.12	77.38278	19612.26	77.38427	19621.73	77.3824	19616.35
77.63513	19606.17	77.63263	19613.56	77.63373	19612.82	77.63199	19615.22	77.63664	19623.76
77.88121	19606.72	77.88538	19613.29	77.88343	19610.2	77.88764	19616.51	77.88272	19613.64
78.13472	19608.6	78.13415	19618.95	78.13252	19614.53	78.13071	19614.05	78.13337	19622.7
78.38213	19609.47	78.38478	19612.74	78.38214	19623.55	78.38504	19614.51	78.38225	19611.27
78.63545	19611.15	78.63197	19620.22	78.63334	19608.62	78.63148	19618.71	78.63492	19620.42
78.88101	19608.11	78.88485	19612.49	78.88463	19615.1	78.88748	19608.9	78.88362	19605.81
79.13743	19616.15	79.13502	19614.69	79.134	19616.56	79.13389	19612.63	79.13417	19620.36
79.3831	19613.42	79.38624	19614.69	79.38143	19620.66	79.38742	19615.8	79.38119	19621.47
79.63597	19610.18	79.63104	19613.86	79.63451	19611.94	79.6325	19608.91	79.63653	19621.03
79.8838	19612.88	79.88487	19615.1	79.88466	19608.38	79.88506	19617.06	79.88397	19620.27
80.13452	19614.6	80.13407	19608.21	80.13506	19616.92	80.13257	19609.61	80.13545	19615.08
80.38846	19612.81	80.38809	19614.36	80.38568	19611.9	80.3891	19614.9	80.38387	19621.95
80.63542	19608.29	80.634	19607.85	80.63377	19616.25	80.63578	19618.08	80.63696	19617.9
80.88466	19604.59	80.88421	19605.89	80.88375	19605.11	80.88493	19621.21	80.8849	19608.26
81.13588	19612.54	81.13473	19609.25	81.1356	19615.26	81.13246	19617.12	81.13562	19619.86
81.38199	19613.23	81.39222	19608.46	81.38511	19617.48	81.38668	19617.88	81.38361	19625.2
81.63603	19612.65	81.63562	19608.1	81.63587	19609.84	81.63408	19618.47	81.63599	19616.12
81.88441	19606.62	81.88721	19615.14	81.8829	19613.39	81.89065	19615.31	81.88376	19615.52
82.13665	19611.21	82.13431	19615.63	82.1347	19612.52	82.13242	19616.23	82.13687	19616.35
82.38317	19610.78	82.38566	19612.26	82.38613	19612.81	82.3857	19619.25	82.38497	19616.07
82.63851	19605.96	82.63476	19615.93	82.63733	19618.54	82.6342	19611.37	82.63564	19616.95
82.88726	19615.1	82.88646	19614.6	82.88411	19615.22	82.88769	19614.71	82.88534	19619.86
83.1371	19608.49	83.13802	19615.76	83.1359	19616.85	83.13289	19615.28	83.13875	19616.15
83.39422	19603.11	83.3854	19613.38	83.38387	19613.53	83.38646	19611.6	83.38458	19622.94
83.63737	19608.2	83.6348	19617.93	83.63598	19609.33	83.63409	19614.57	83.63698	19614.79
83.88483	19609.78	83.88684	19615.17	83.88562	19609.13	83.88909	19619.19	83.88516	19609.99
84.13858	19608.38	84.13747	19619.88	84.13417	19612.65	84.13415	19616.61	84.13737	19614.64
84.38543	19609.66	84.38726	19607.55	84.38494	19611.49	84.38644	19614.95	84.38452	19613.44
84.63831	19614.02	84.63376	19615.51	84.6378	19614.88	84.63362	19615.42	84.6375	19615.97
84.88567	19610.37	84.88759	19614.78	84.88723	19616.2	84.8868	19612.74	84.88643	19613.65
85.1516	19606.64	85.13622	19618.18	85.13527	19610.08	85.13586	19619.15	85.14143	19618.44
85.38832	19616.43	85.39612	19616.71	85.38406	19612.36	85.3888	19614.12	85.38502	19620.22
85.63851	19610.31	85.64078	19613.07	85.63853	19608.53	85.63291	19612.19	85.6386	19616.14
85.88704	19613.38	85.8889	19619.19	85.88617	19607.46	85.88783	19615.67	85.88739	19616.35
86.13753	19605.93	86.1382	19616.78	86.13738	19615.18	86.13488	19616.84	86.13634	19608.54

86.38435	19610.12	86.39324	19609.83	86.38583	19614.22	86.38958	19609.95	86.38757	19616.84
86.63944	19613.96	86.63561	19610.14	86.63533	19616.7	86.636	19615.7	86.63773	19616.52
86.88705	19610.11	86.88973	19617.93	86.8864	19609.83	86.88637	19617.05	86.88924	19616
87.1404	19617.24	87.13619	19613.5	87.13965	19613.06	87.13422	19614.16	87.13754	19622.45
87.38409	19607.38	87.38784	19611.32	87.38768	19617.53	87.38892	19613.38	87.38608	19613.25
87.6383	19613.18	87.63607	19606.73	87.63604	19612.42	87.63671	19618.35	87.63895	19617.33
87.8875	19610.74	87.88894	19615.61	87.88605	19608.8	87.88784	19611.25	87.88691	19622.53
88.13999	19603.39	88.13762	19612.36	88.14259	19611.07	88.13488	19610.1	88.13926	19612.04
88.38702	19606.52	88.38871	19615.11	88.38836	19615.43	88.38748	19611.09	88.38756	19615.09
88.63726	19602.82	88.63611	19610.76	88.63762	19617.69	88.63673	19612.94	88.63817	19616.85
88.88622	19607.62	88.88839	19613.4	88.88602	19612.56	88.89157	19616.41	88.88685	19619.85
89.14101	19608.95	89.13837	19605.24	89.13819	19609.32	89.13496	19612.16	89.13815	19617.65
89.38793	19607.38	89.38966	19614.43	89.38669	19611.5	89.38913	19615.46	89.38787	19620.35
89.63882	19608.83	89.63564	19612.15	89.63777	19617.58	89.63882	19615.26	89.63896	19620.31
89.88929	19611.05	89.88866	19609.9	89.88599	19612.93	89.8897	19620.05	89.88646	19616.25
90.14036	19608.01	90.13772	19608.46	90.13709	19613.02	90.13658	19608.87	90.13724	19617.28
90.38867	19612.32	90.39006	19613.9	90.38793	19610.6	90.39224	19611.6	90.38912	19613.58
90.63997	19608.96	90.63748	19613.4	90.64011	19610.61	90.63606	19609.11	90.64212	19611.67
90.88729	19604.92	90.88968	19611.72	90.88842	19611.35	90.88862	19605.6	90.88791	19619.26
91.13952	19606.06	91.13774	19621.04	91.1375	19612.4	91.14544	19609.09	91.13806	19615.86
91.3884	19607.7	91.39355	19610.75	91.38778	19614.17	91.39814	19612.39	91.39014	19619.33
91.63979	19607.2	91.63914	19612.23	91.63978	19616	91.63602	19610.88	91.64152	19611.67
91.88754	19610.12	91.89069	19615.17	91.88887	19611.92	91.89003	19617.6	91.88848	19615.39
92.13946	19606.28	92.14215	19616.54	92.1397	19615.54	92.14659	19619.89	92.13788	19611.67
92.38877	19609.94	92.38974	19615.43	92.38694	19614.02	92.39286	19611.06	92.39084	19615.82
92.64052	19610.73	92.63757	19612.35	92.63689	19615.04	92.63808	19610.76	92.64102	19612.07
92.88861	19607.81	92.89103	19607.36	92.88967	19615.11	92.8907	19611.71	92.88861	19614.75
93.14117	19604.22	93.13975	19616.16	93.13956	19609.96	93.13811	19620.26	93.13917	19610.91
93.38926	19604.58	93.39207	19615.22	93.3892	19615.26	93.39148	19617.27	93.39004	19616.81
93.64065	19611.61	93.63674	19606.1	93.63665	19611.36	93.63769	19609.88	93.64183	19615.33
93.89001	19602.98	93.89132	19604.78	93.88767	19612.83	93.89464	19617.21	93.89059	19611.4
94.14351	19613.16	94.14816	19607.32	94.14026	19609.79	94.139	19608.95	94.14594	19612.19
94.39024	19608.86	94.39924	19606.16	94.39021	19611.31	94.39198	19612.38	94.38867	19617.01
94.64109	19608.05	94.6374	19610.07	94.63946	19613.54	94.63865	19615.04	94.6435	19615.26
94.88967	19606.9	94.89137	19609.2	94.88717	19614.14	94.89255	19612.99	94.89042	19614.64
95.14238	19611.2	95.14079	19615.62	95.1388	19611.07	95.14122	19612.53	95.14047	19616.67
95.38973	19600.64	95.39144	19607.92	95.38955	19610.78	95.41438	19613.18	95.39033	19615.44
95.64123	19608.46	95.63898	19609.36	95.64053	19610.31	95.63707	19612.11	95.64245	19612.91
95.88867	19601.66	95.89229	19619.16	95.8909	19611.26	95.89118	19608.34	95.89102	19613.51
96.14223	19605.83	96.13973	19610.49	96.14212	19613.86	96.14009	19610.23	96.142	19614.68
96.39178	19606.85	96.3949	19613.55	96.38911	19603.98	96.39349	19610.91	96.39062	19611.72
96.64165	19606.26	96.64006	19611.2	96.63948	19618.96	96.63786	19608.07	96.64284	19616.04
96.89157	19606.72	96.89127	19611.89	96.89052	19615.89	96.89057	19613.54	96.89246	19615.61
97.14353	19608.77	97.14099	19616.16	97.14434	19612.12	97.14118	19611.58	97.14296	19618.07
97.39252	19604.41	97.39205	19609.48	97.39011	19613.27	97.39205	19613.51	97.39252	19613.86
97.64319	19607.3	97.64457	19610.74	97.64088	19612.81	97.63879	19611.39	97.64144	19609.12
97.89103	19611.51	97.89214	19613.23	97.90134	19613.24	97.89133	19616.16	97.89064	19614.09
98.14233	19605.74	98.14046	19609.23	98.14207	19611.67	98.14048	19612.1	98.14519	19610.87
98.39181	19606.71	98.39187	19613.86	98.39234	19610.46	98.39314	19612.03	98.39346	19615.33
98.64408	19604.32	98.64036	19607.91	98.64252	19613.3	98.6406	19612.88	98.64485	19616.26
98.89215	19608.21	98.89381	19609.73	98.88955	19614.12	98.89545	19622.66	98.8911	19612.06
99.14557	19600.7	99.14115	19603.13	99.14024	19609.14	99.14083	19610.62	99.14401	19609.73
99.38986	19609.78	99.39257	19616.15	99.39185	19610.52	99.39442	19611.07	99.3932	19611.4
99.6425	19603.35	99.64042	19606.33	99.64153	19612.21	99.64031	19609.63	99.64537	19610.42
99.89171	19605.83	99.89446	19609.25	99.89273	19615.37	99.89307	19610.7	99.89077	19607.83
100.1426	19609.61	100.1422	19611.77	100.1423	19611.42	100.1415	19610.34	100.1458	19614.43
100.4092	19602.18	100.3967	19607.73	100.3939	19607.58	100.397	19614.32	100.4005	19611.19
100.6464	19607.69	100.6448	19604.6	100.6433	19608.14	100.642	19608.43	100.646	19612.86
100.8918	19602.51	100.8959	19602.29	100.8981	19613.26	100.8965	19608.42	100.8941	19612.74
101.1446	19605.48	101.1442	19608.72	101.1498	19614.69	101.1446	19613.72	101.1472	19612.98
101.394	19602.04	101.3978	19610.87	101.3944	19612.29	101.3962	19606.56	101.3977	19616.47
101.6462	19606.95	101.6428	19603.83	101.6432	19613.74	101.6417	19616.16	101.6462	19611.19
101.8931	19604.78	101.8954	19612.27	101.8951	19609	101.8957	19611.14	101.8936	19611.47
102.1448	19606.42	102.1427	19608.34	102.1466	19608.59	102.1457	19612	102.1458	19619.31
102.3944	19607.4	102.3954	19616.28	102.3954	19605.3	102.3975	19613.51	102.3952	19610.26
102.6464	19609.68	102.6462	19608.9	102.646	19614.28	102.6424	19608.78	102.6521	19611.36
102.8952	19606.8	102.8974	19606.72	102.8955	19611.59	102.8953	19607.95	102.8996	19610.96
103.1453	19606.83	103.143	19610.78	103.1452	19608.88	103.1448	19611.56	103.1454	19616.32
103.3937	19608.58	103.3983	19610.87	103.3952	19609.62	103.3993	19609.24	103.3944	19613.32
103.646	19596.27	103.6465	19606.84	103.6471	19612.88	103.6446	19608.92	103.6492	19607.48

103.8951	19603.06	103.8982	19608.6	103.8944	19612.58	103.8993	19610.91	103.8956	19610.15
104.1494	19613.75	104.1434	19609.45	104.147	19610.09	104.1449	19609.93	104.1463	19614.21
104.3937	19604.45	104.3982	19607.04	104.3954	19603.72	104.3979	19611.48	104.3962	19612.67
104.646	19607.66	104.6444	19603.7	104.6464	19605.49	104.6452	19612.9	104.6494	19609.76
104.8962	19603.57	104.8987	19612.04	104.8961	19609.89	104.8976	19607.43	104.8976	19612.47
105.1489	19605.75	105.1457	19605.48	105.145	19604.06	105.1458	19607.75	105.1469	19606.9
105.3942	19603.89	105.3974	19610.79	105.3957	19611.49	105.3978	19612.92	105.3983	19611.07
105.647	19599.37	105.6446	19605.47	105.6502	19605.19	105.6483	19610.01	105.6564	19621.84
105.8957	19600.48	105.8976	19613.27	105.8969	19607.62	105.8984	19612.16	105.9078	19615.21
106.15	19603.15	106.1472	19605.53	106.1478	19612.47	106.1468	19606.12	106.1501	19613.9
106.3963	19602.11	106.3993	19610.13	106.3953	19608.82	106.4025	19615.69	106.3968	19613.19
106.6492	19605.19	106.6462	19607.93	106.6464	19608.16	106.6453	19606.37	106.648	19607.25
106.8958	19611.32	106.8974	19608.4	106.8988	19608.55	106.8977	19615.95	106.8984	19610.27
107.1477	19606.76	107.1459	19604.02	107.1608	19608.51	107.1464	19603.14	107.1494	19610.5
107.3957	19599.98	107.4001	19602.52	107.3973	19611.46	107.4	19607.16	107.3969	19608
107.6492	19604.14	107.6442	19612.02	107.6464	19606.53	107.6478	19609.79	107.6494	19610.95
107.8974	19601.18	107.8982	19601.5	107.8986	19604.61	107.8993	19611.61	107.8989	19611.04
108.1489	19606.05	108.1464	19609.6	108.1522	19600.45	108.1449	19602.69	108.1492	19606.5
108.3991	19604.98	108.3996	19605.63	108.3975	19609.08	108.3986	19610.39	108.3983	19602.58
108.6501	19605.3	108.6468	19608.5	108.6474	19608.26	108.647	19606.02	108.6488	19616.85
108.8974	19604.2	108.8989	19604.28	108.897	19604.93	108.9023	19610.6	108.8978	19606.78
109.152	19605.8	109.1459	19597.77	109.1492	19610.34	109.1487	19610.35	109.1484	19608.64
109.3957	19600.84	109.3999	19610.54	109.3976	19609.48	109.3999	19607.98	109.3987	19605.67
109.6504	19601.02	109.6485	19593.29	109.6492	19607.12	109.6467	19607.17	109.6518	19612.38
109.8982	19595.37	109.9002	19609.3	109.8986	19604.76	109.8989	19606.85	109.8982	19614.64
110.1499	19601.3	110.1467	19601.02	110.1487	19610.84	110.1475	19605.61	110.1496	19606.11
110.3972	19600.68	110.4006	19612.7	110.3977	19612.11	110.4046	19609.37	110.3988	19610.83
110.6549	19603.79	110.6486	19602.28	110.6517	19601.03	110.6469	19610.94	110.651	19613.68
110.8987	19603.73	110.9006	19600.35	110.899	19604.1	110.9006	19606.76	110.8986	19603.6
111.1498	19597.65	111.1466	19609.92	111.1488	19607.76	111.148	19607.34	111.1498	19605.44
111.3974	19598.99	111.4037	19603.56	111.3966	19611.46	111.4021	19600.37	111.399	19605.74
111.6516	19594.16	111.6484	19598.59	111.65	19604.83	111.6462	19610.45	111.6537	19603.88
111.8976	19602.52	111.9015	19604.31	111.9003	19602.21	111.9012	19606.98	111.9012	19606.38
112.1496	19602.57	112.1482	19606.94	112.1508	19603.77	112.1484	19607.11	112.1525	19603.97
112.4101	19601.78	112.4016	19604.15	112.3975	19607.28	112.4032	19608.59	112.3975	19613.12
112.6552	19600.41	112.6472	19599.78	112.6484	19603.4	112.6474	19610.89	112.6519	19610.93
112.8969	19596.54	112.9005	19607.46	112.8994	19604.29	112.8993	19606.14	112.9	19609.8
113.1497	19604.75	113.1476	19601.59	113.1539	19604.1	113.1469	19599.74	113.1532	19606.38
113.3993	19603.03	113.4044	19601.26	113.3982	19602.97	113.4008	19610.21	113.3989	19612.08
113.6551	19600.14	113.6476	19610.54	113.6493	19606.93	113.6487	19605.98	113.6522	19608.13
113.9012	19600.41	113.9006	19604.58	113.899	19603.19	113.9041	19605.63	113.9018	19608.17
114.155	19595.64	114.1493	19607.5	114.1534	19613.09	114.1471	19608.58	114.154	19610.59
114.3975	19600.36	114.4043	19601.73	114.3994	19601.8	114.4012	19614.9	114.4003	19608.96
114.6523	19599.26	114.6492	19604.41	114.6497	19609.27	114.6511	19610.76	114.6546	19612.01
114.9007	19598.98	114.9021	19604.62	114.8985	19597.78	114.903	19606.68	114.9006	19604.59
115.1532	19600.12	115.1485	19604.2	115.1506	19600.75	115.1487	19606.91	115.154	19609.18
115.3996	19601.88	115.4043	19602.31	115.3999	19609.74	115.4031	19607.18	115.4004	19611.7
115.6527	19599.81	115.653	19609.25	115.6511	19609.56	115.6474	19603.13	115.6526	19606.14
115.9	19600.49	115.9021	19598.63	115.8991	19604.78	115.9016	19610.77	115.9011	19611.62
116.1535	19595.94	116.1479	19601.95	116.1504	19607.55	116.15	19603.69	116.1542	19606.4
116.3997	19601.97	116.4066	19607.31	116.4003	19606.42	116.4041	19606.54	116.3997	19606.87
116.6522	19594.83	116.6513	19607.01	116.6517	19607.2	116.6484	19605.58	116.6547	19608.09
116.9002	19597.73	116.9036	19602.5	116.9014	19602.08	116.9019	19607.95	116.9024	19604.65
117.1521	19598.57	117.1487	19605.64	117.1533	19601.14	117.1505	19605.91	117.153	19605.99
117.3999	19600.64	117.4038	19611.95	117.3995	19599.29	117.4058	19605.78	117.399	19602.61
117.6539	19600.72	117.6536	19594.15	117.6531	19602.53	117.6487	19601.63	117.6568	19606.1
117.9008	19595.98	117.9037	19602.54	117.901	19608.68	117.9024	19606.87	117.9025	19612.19
118.1523	19599.11	118.1484	19602.79	118.1542	19601.55	118.1497	19605.9	118.1532	19603.72
118.4015	19597.94	118.4041	19596.86	118.4019	19605.1	118.4068	19602.74	118.4011	19607.11
118.6555	19593.18	118.6516	19603.14	118.6522	19603.4	118.6502	19600.07	118.6592	19608.09
118.9049	19599.68	118.9059	19603.09	118.9016	19599.84	118.9062	19603.23	118.9054	19601.9
119.1553	19598.34	119.1505	19598.56	119.1552	19599.09	119.1515	19604.55	119.1543	19604.26
119.403	19598.15	119.408	19602.55	119.404	19603.58	119.4054	19607.16	119.4008	19607.94
119.6593	19597.38	119.6546	19602.93	119.6579	19605.5	119.6558	19602.39	119.6578	19607.71
119.9028	19599.98	119.9044	19598.9	119.9012	19603.34	119.9049	19600.36	119.9046	19605
120.153	19593.12	120.1514	19602.64	120.1555	19599.48	120.1499	19603.27	120.1557	19608.94
120.402	19595.94	120.404	19595.8	120.4032	19606.5	120.4053	19594.96	120.4044	19607.92
120.653	19604.06	120.648	19601.65	120.6492	19607.1	120.6484	19606.93	120.6551	19599.2
120.9023	19597.27	120.9019	19603.15	120.8987	19604.67	120.9046	19599.61	120.9008	19607.03
121.1551	19593.09	121.1493	19592.15	121.1519	19603.64	121.1504	19608.72	121.1535	19600.51

121.3991	19596.71	121.4063	19601.76	121.4028	19599.69	121.4149	19605.29	121.4052	19604.23
121.6528	19594.21	121.6474	19600.65	121.6508	19604.05	121.6501	19605.61	121.6569	19595.17
121.9012	19591.66	121.901	19603.14	121.9024	19604.04	121.9034	19606.55	121.9003	19604.42
122.1524	19599.54	122.1494	19602.95	122.1531	19606.12	122.1508	19603.09	122.1522	19607.02
122.4032	19593.15	122.4138	19602.65	122.3999	19601	122.4065	19601.4	122.4032	19599.84
122.6524	19597.18	122.6508	19598.13	122.6517	19595.98	122.6473	19604.14	122.6548	19604.75
122.8998	19599.06	122.9055	19604.99	122.9016	19603.64	122.9021	19600.34	122.9035	19604.61
123.1535	19591.84	123.1517	19600.5	123.1559	19595	123.1506	19599.28	123.1529	19603.86
123.3999	19605.33	123.4034	19591.83	123.3995	19609.16	123.4081	19612.43	123.4049	19608.14
123.6539	19602.12	123.651	19600.08	123.6533	19597.14	123.6487	19600.5	123.6548	19596.76
123.9025	19593.19	123.904	19600.06	123.9028	19603.06	123.9048	19595.27	123.9035	19600.15
124.1544	19597.42	124.1498	19600.6	124.1558	19599.37	124.1509	19600.49	124.1542	19603.47
124.4006	19598.44	124.4044	19602.55	124.4002	19601.74	124.4093	19604.38	124.4021	19602.53
124.6544	19596.04	124.6518	19599.32	124.6508	19601.87	124.6499	19602.12	124.6551	19604.24
124.9018	19599.21	124.9045	19602.38	124.902	19602.14	124.9044	19600.7	124.9037	19609.34
125.1527	19592.49	125.1491	19601.49	125.158	19599.32	125.1516	19603.8	125.1534	19610.62
125.4008	19599.51	125.4058	19596.47	125.401	19599.38	125.4237	19598.31	125.4039	19595.95
125.6529	19595.92	125.6524	19597.9	125.6515	19604.66	125.6503	19593.68	125.653	19605.9
125.9059	19597.59	125.9051	19600.34	125.9005	19601.15	125.9055	19598.13	125.9049	19600.88
126.1546	19595.1	126.1502	19598.38	126.1553	19605.64	126.1503	19601.82	126.1551	19599.35
126.4003	19590.77	126.4064	19604.21	126.4034	19602.46	126.4045	19596.24	126.4031	19601.36
126.6553	19599.16	126.6515	19603.93	126.6524	19599.12	126.6514	19599.66	126.6542	19609.64
126.9031	19597.5	126.9043	19598.12	126.9011	19603.84	126.9051	19597.21	126.9037	19595.93
127.1565	19592.85	127.1521	19601.05	127.16	19593.26	127.1515	19599.71	127.156	19598.31
127.4009	19599.88	127.4073	19596.35	127.4024	19598.71	127.4059	19606.66	127.4034	19601.6
127.6539	19597.8	127.6546	19604.01	127.6556	19599.02	127.6513	19603.38	127.6536	19606.49
127.9023	19591.33	127.9066	19597.63	127.9027	19602.32	127.9049	19607	127.9035	19605.92
128.1582	19591.23	128.1528	19601.3	128.1565	19599.01	128.1514	19601.64	128.1534	19607.14
128.4129	19592.71	128.4059	19596.36	128.4017	19603.97	128.4071	19600.07	128.4037	19604.3
128.6562	19594.65	128.6513	19599.26	128.6543	19602.78	128.6529	19596.95	128.6561	19590.97
128.9049	19591.66	128.9061	19601.98	128.9041	19597.93	128.9055	19602.43	128.9088	19596.58
129.1594	19594.48	129.1529	19600.49	129.1567	19607.77	129.1525	19594.85	129.1538	19595.24
129.4037	19591.59	129.4067	19593.03	129.4024	19599.89	129.407	19603.78	129.4043	19595
129.6579	19599.77	129.6521	19599.1	129.6544	19597.98	129.6512	19600.49	129.6588	19600.33
129.9021	19597.62	129.9055	19603.54	129.9049	19597.34	129.9043	19599.26	129.9062	19601.2
130.1582	19593.18	130.1522	19594.03	130.1583	19605.42	130.1518	19604.03	130.1568	19606.02
130.4056	19596.06	130.4059	19597.8	130.403	19598.77	130.408	19601.49	130.403	19604.72
130.6584	19595.37	130.6572	19601.88	130.6536	19595.7	130.6535	19594.27	130.6579	19611.92
130.9055	19590.87	130.9067	19592.92	130.9028	19601	130.9064	19598.66	130.9063	19603.35
131.1576	19591.53	131.1529	19597.59	131.1624	19596.06	131.152	19601.27	131.1556	19601.94
131.4016	19587.89	131.4081	19586.7	131.4065	19596.2	131.4068	19599.65	131.4045	19606.06
131.6568	19589.12	131.6577	19602.76	131.6561	19602.5	131.6533	19595.84	131.6573	19600.79
131.908	19596.89	131.9066	19598.29	131.9042	19602.44	131.9078	19602.17	131.9061	19596.81
132.1569	19598.93	132.1553	19594.61	132.1583	19601.94	132.1535	19600.41	132.1583	19606.7
132.4046	19597.55	132.4064	19610.17	132.4056	19605.46	132.4067	19599.79	132.4048	19602.29
132.6583	19592.12	132.6553	19593.25	132.6547	19598.65	132.6539	19598.72	132.6562	19600.63
132.9104	19594.49	132.9069	19601.73	132.9044	19595.16	132.9069	19600.64	132.9065	19601.59
133.1584	19596.06	133.1548	19592.96	133.1571	19596.93	133.1536	19595.29	133.1602	19597.8
133.4065	19594.19	133.4071	19598.6	133.4051	19591.73	133.4083	19604.98	133.406	19597.8
133.6574	19595.01	133.6551	19593.71	133.6583	19597.82	133.6532	19598.1	133.6579	19599.91
133.9068	19592.73	133.9081	19601.63	133.9054	19600.94	133.9088	19600.61	133.9043	19596.01
134.1616	19590.08	134.1555	19597.74	134.1589	19601.85	134.1571	19597.53	134.1576	19597.71
134.408	19599.75	134.408	19595.5	134.4048	19599.46	134.4093	19605.71	134.4072	19603.66
134.6606	19590.41	134.657	19600.06	134.6575	19604.57	134.6544	19596.14	134.6599	19600.77
134.9053	19597.53	134.9099	19597	134.9074	19596.29	134.9067	19598.67	134.9066	19602.71
135.1571	19597.11	135.1549	19596.07	135.1605	19600.56	135.1542	19603.2	135.1588	19603.28
135.4046	19589.51	135.4089	19595.12	135.4041	19597.12	135.4102	19602.49	135.408	19596.03
135.6625	19598.74	135.6579	19594.65	135.6591	19599.05	135.6556	19599.19	135.661	19602.27
135.9083	19591.5	135.9111	19594.44	135.9078	19597.41	135.9077	19599.72	135.9076	19600.58
136.1594	19588.09	136.1542	19597.07	136.1645	19594.39	136.1545	19594.51	136.1564	19602.84
136.4053	19592.96	136.4111	19593.23	136.4069	19600.41	136.4116	19596.36	136.4078	19598.86
136.66	19594.36	136.6576	19597.73	136.6587	19602.25	136.6562	19594.82	136.6608	19601.91
136.909	19594.37	136.9087	19597.67	136.9066	19598.19	136.9082	19593.47	136.9126	19602.73
137.1595	19599.69	137.1544	19606.76	137.1606	19598.52	137.1568	19598.66	137.1588	19594.05
137.4046	19595.2	137.4082	19598.29	137.4072	19600.91	137.4098	19596.59	137.4082	19595.85
137.6596	19587.53	137.6634	19592.71	137.6558	19599.37	137.6564	19599.05	137.662	19601.83
137.9077	19596.81	137.9093	19600.56	137.907	19595.1	137.9093	19596.99	137.9114	19599.15
138.1602	19590.63	138.1552	19591.22	138.16	19596.97	138.1568	19602.45	138.1594	19607.36
138.4071	19591.48	138.4098	19592.23	138.4066	19601.53	138.4118	19601.24	138.4064	19596.69
138.6601	19594.59	138.6576	19596.8	138.6571	19601.39	138.6564	19597.14	138.6616	19596.98

138.9082	19587.82	138.9105	19601.92	138.9077	19604.64	138.9106	19601.28	138.9113	19595.14
139.1623	19594.58	139.1557	19589.07	139.1615	19597.27	139.1577	19599.73	139.16	19593.24
139.4078	19593.68	139.4096	19592.81	139.4067	19598.29	139.4128	19599.88	139.4063	19602.41
139.6599	19595.41	139.6574	19595.71	139.6589	19598.92	139.6742	19604.94	139.6608	19596.28
139.908	19592.96	139.9083	19596.31	139.9081	19600.47	139.9088	19598.66	139.9113	19597.11
140.1585	19593.89	140.1568	19597.01	140.1646	19594.54	140.1576	19603.5	140.1606	19600.28
140.4088	19589.64	140.4105	19595.96	140.4083	19599.51	140.4123	19603.82	140.4066	19599.55
140.6617	19592.04	140.6572	19596.4	140.6593	19594.19	140.6592	19597.51	140.6592	19599.89
140.9078	19586.5	140.9109	19594.43	140.907	19591.32	140.909	19590.72	140.9136	19594.46
141.162	19588.55	141.159	19592.43	141.1611	19596.97	141.1573	19593.12	141.1604	19601.76
141.4084	19597.36	141.4165	19591.63	141.4089	19595.7	141.4114	19601.76	141.4065	19599.16
141.6617	19592.5	141.6731	19593.03	141.6663	19601.99	141.6595	19599.49	141.6626	19600.59
141.9078	19589.75	141.9084	19593.6	141.9113	19595.54	141.9106	19593.87	141.9109	19603.29
142.1612	19591.56	142.1567	19592.14	142.1642	19597.6	142.1568	19602.61	142.1595	19599.94
142.4091	19591.64	142.4224	19598.35	142.4132	19598.86	142.4132	19599.91	142.4086	19597.94
142.6634	19591.43	142.6607	19598.68	142.6632	19597.03	142.6609	19598.61	142.6626	19600.37
142.9089	19595	142.9103	19594.21	142.9166	19597.64	142.9127	19602.6	142.9127	19596.89
143.161	19591.79	143.1569	19596.75	143.1625	19600.67	143.1577	19591.69	143.1634	19601.48
143.4208	19589.24	143.4111	19597.48	143.4136	19594.19	143.412	19596.58	143.4102	19596.67
143.6602	19592.63	143.6603	19602.72	143.6599	19601.97	143.6593	19598.41	143.6608	19602.02
143.9094	19589.81	143.9116	19594.5	143.9084	19601.52	143.914	19591.46	143.9125	19602.6
144.1641	19586.55	144.1567	19594.01	144.1628	19592.97	144.1596	19595.7	144.1627	19597.27
144.4107	19595.31	144.412	19604.35	144.4123	19596.41	144.4175	19593.47	144.4114	19598.77
144.6632	19595.6	144.664	19592.6	144.6614	19603.93	144.6603	19600.65	144.662	19597.31
144.9104	19588.61	144.9133	19594.91	144.9127	19597.51	144.9117	19606.46	144.9113	19594.98
145.1634	19597.19	145.1581	19591.85	145.1614	19597.88	145.1612	19595.71	145.163	19601.22
145.4099	19590.17	145.4123	19590.75	145.4104	19600.14	145.4149	19597.7	145.4101	19600.77
145.66	19593.55	145.6596	19593.85	145.6618	19595.53	145.6597	19596.71	145.6648	19598.28
145.9133	19594.99	145.9119	19594.65	145.9101	19588.7	145.9103	19590.47	145.9144	19604.25
146.1648	19593.02	146.1608	19597.52	146.1647	19593.54	146.1595	19597.08	146.1642	19592.61
146.4114	19598.44	146.4159	19597.15	146.4105	19600.53	146.4146	19595.31	146.4109	19592.63
146.6618	19591.82	146.6587	19596.19	146.6618	19596.57	146.6603	19595.59	146.6642	19596.17
146.9106	19592.54	146.9127	19596.64	146.9107	19590.5	146.9118	19595.98	146.9125	19603.6
147.1632	19593.84	147.164	19588.45	147.1766	19585.32	147.1612	19603.15	147.1647	19593.86
147.413	19595.15	147.4141	19586.51	147.4138	19598.18	147.4145	19595.44	147.4112	19592.4
147.6646	19591.7	147.6617	19596.43	147.6628	19596.24	147.6621	19594.77	147.6627	19606.33
147.9098	19589.91	147.9136	19597.26	147.911	19602.24	147.9133	19599.5	147.9122	19593.85
148.1616	19596.43	148.1599	19593.05	148.1649	19599.34	148.1594	19595.05	148.1624	19600.26
148.413	19586.17	148.4125	19592.47	148.4131	19599.95	148.413	19593.04	148.415	19590.41
148.6638	19588.03	148.6611	19593.98	148.6614	19594.43	148.6611	19599.28	148.6631	19600.92
148.9134	19585.77	148.9146	19596.64	148.911	19589.89	148.9152	19592	148.9135	19597.73
149.1671	19593.09	149.1602	19591.18	149.1669	19598.24	149.1599	19597.36	149.1636	19595.1
149.4104	19590.29	149.4132	19599.54	149.4121	19598.81	149.4153	19600.81	149.4126	19595.12
149.6654	19590.01	149.6606	19589.55	149.6616	19593.49	149.6609	19595.03	149.6647	19591.5
149.912	19585.42	149.9146	19594.52	149.9105	19596.24	149.9126	19597.26	149.9126	19594.1
150.1661	19595.18	150.1601	19598.98	150.1646	19594.68	150.1611	19601.59	150.1645	19603.9
150.4155	19599.45	150.4173	19598.23	150.4177	19595.24	150.4178	19598.09	150.4167	19595.15
150.6678	19592.04	150.6637	19602.16	150.6667	19596.48	150.6634	19597.64	150.6714	19598.33
150.9173	19589.07	150.9173	19588.07	150.9154	19593.06	150.9166	19594.99	150.9146	19598.21
151.1686	19593.1	151.1642	19583.11	151.1672	19596.06	151.1652	19585.52	151.1697	19600.2
151.428	19583.13	151.4197	19594.57	151.4155	19597.29	151.4214	19603.34	151.4196	19602.29
151.6707	19590.59	151.6645	19593.02	151.669	19594.72	151.6649	19595.97	151.6698	19592.25
151.9163	19593.16	151.917	19588.93	151.9173	19595.15	151.9162	19595.87	151.9164	19598.46
152.1684	19590.81	152.1646	19592.04	152.1833	19592.17	152.1779	19591.82	152.1678	19596.86
152.4164	19592.29	152.4187	19587.74	152.4176	19588.16	152.4312	19598.03	152.4187	19596.57
152.6725	19594.03	152.6679	19593.66	152.6674	19591.44	152.6655	19603.51	152.6709	19593.26
152.9183	19587.41	152.9177	19595.46	152.9161	19591.65	152.9194	19587.38	152.9171	19595.67
153.1705	19588.66	153.1644	19585.45	153.1728	19597.6	153.1661	19597.45	153.1691	19598.7
153.4182	19592.85	153.4178	19592.56	153.4174	19593.72	153.421	19596.8	153.4201	19598.4
153.6698	19587.74	153.6685	19590.89	153.6678	19595	153.6646	19599.99	153.6689	19608.27
153.9162	19586.54	153.9195	19602.92	153.9158	19593.21	153.9222	19600.63	153.9195	19591.71
154.1706	19591.75	154.1668	19589.69	154.1713	19594.13	154.1662	19598.77	154.1708	19593.45
154.419	19589.63	154.4249	19593.73	154.4174	19586	154.4424	19600.69	154.4199	19592.17
154.6841	19589.87	154.6756	19597.51	154.669	19598.11	154.6655	19597.27	154.6686	19596.53
154.9243	19589.76	154.9214	19586.73	154.9171	19603.09	154.9193	19594.32	154.9178	19600.77
155.1696	19595.2	155.1668	19593.74	155.1698	19593.01	155.1684	19597.66	155.1714	19596.97
155.4184	19584.65	155.4236	19596.47	155.4174	19596.37	155.4214	19590.65	155.4194	19595.45
155.6697	19593.31	155.6675	19581.64	155.6694	19593.3	155.6657	19593.83	155.6696	19601.83
155.918	19592.97	155.9225	19593.42	155.919	19593.25	155.9211	19596.24	155.9179	19596.02
156.1711	19589.85	156.1671	19597.11	156.1725	19595.86	156.1686	19592.99	156.1707	19600.62

156.4329	19594.02	156.4242	19597.62	156.4177	19594.66	156.4226	19601.36	156.4195	19587.91
156.6719	19588.75	156.6674	19596.66	156.6694	19597.09	156.6666	19593.61	156.6696	19593.68
156.9194	19585.44	156.9205	19590.73	156.9183	19593.1	156.9187	19595.84	156.9191	19586.76
157.1713	19587.77	157.1677	19596.63	157.1729	19602.59	157.1672	19589.11	157.17	19595.37
157.4205	19583.69	157.432	19597	157.4176	19594.57	157.4214	19593.05	157.4182	19599.36
157.672	19586.7	157.6694	19589.92	157.6706	19597.25	157.6664	19598.47	157.6722	19593.28
157.9212	19591.78	157.9252	19596.81	157.9188	19593.46	157.9207	19599.93	157.9196	19596.41
158.1706	19588.33	158.1671	19592.07	158.1923	19587.17	158.1666	19606.57	158.1722	19592.68
158.421	19589.32	158.4235	19593.06	158.4185	19594.58	158.4217	19589.17	158.4197	19589.07
158.6716	19585.05	158.6691	19586.27	158.6708	19595.64	158.6692	19594.38	158.6732	19593.41
158.919	19590.72	158.9236	19591.98	158.921	19588.05	158.9219	19592.27	158.9197	19598.57
159.1755	19595.09	159.1657	19593.21	159.1747	19592.68	159.169	19591.8	159.172	19595.26
159.4215	19583.22	159.4215	19590.38	159.4224	19597.14	159.4222	19597.14	159.4199	19593.82
159.6738	19588.61	159.6695	19593.93	159.6727	19593.04	159.6669	19598.99	159.6725	19597.13
159.9203	19592.51	159.923	19589.15	159.9209	19588.87	159.9216	19599.49	159.9234	19592.85
160.1719	19589.71	160.1696	19587.79	160.1745	19597.43	160.1687	19601.83	160.1812	19592.81
160.4217	19596.12	160.4215	19594.61	160.42	19599	160.4249	19594.28	160.4213	19591.44
160.6721	19597.68	160.6655	19589.59	160.668	19594.88	160.6636	19597.1	160.68	19596.05
160.9165	19580.44	160.9185	19600.47	160.9203	19599.31	160.9185	19591.95	160.918	19594.25
161.1713	19590.47	161.1699	19589.45	161.1692	19595.65	161.1686	19596.83	161.1717	19598.65
161.4182	19587.78	161.4351	19586.41	161.4177	19587.24	161.421	19590.48	161.4174	19597.9
161.6671	19592.17	161.6659	19593.93	161.6722	19593	161.6641	19587.48	161.6735	19591.33
161.9188	19586.6	161.9205	19588	161.918	19598.26	161.9218	19594.04	161.9189	19594.35
162.1713	19595.5	162.1719	19590	162.1721	19593.6	162.165	19587.13	162.1708	19594.04
162.4319	19590.43	162.4207	19590.67	162.4161	19603.99	162.4216	19593.18	162.4182	19587.79
162.6722	19588.78	162.6667	19594.24	162.67	19589.3	162.6651	19595.96	162.6719	19595.36
162.917	19585.65	162.9208	19599.84	162.9188	19595.3	162.9195	19588.76	162.9348	19595.59
163.1754	19587.2	163.1659	19589.36	163.1735	19588.7	163.1655	19593.64	163.1759	19594.97
163.4261	19589.37	163.4192	19588	163.4177	19585.13	163.4213	19593.97	163.4197	19596.67
163.6701	19589.18	163.668	19587.91	163.6731	19592.44	163.666	19593.16	163.6784	19589.97
163.9177	19592.81	163.9216	19588.78	163.917	19598.88	163.9241	19597.54	163.9198	19595.32
164.1712	19585.42	164.1658	19587.92	164.1753	19591.85	164.1697	19596.42	164.1714	19596.05
164.4233	19585.97	164.42	19595.09	164.4187	19590.09	164.4207	19591.53	164.4199	19597.35
164.6711	19594.95	164.6676	19598.27	164.6719	19597.64	164.6667	19590.51	164.6722	19596.33
164.9167	19585.67	164.9213	19592.51	164.918	19599.17	164.9222	19594.28	164.92	19590.71
165.1708	19589.66	165.1662	19593.76	165.1745	19590.13	165.1673	19590.79	165.1713	19591.85
165.423	19592.09	165.4218	19593.17	165.4179	19585.73	165.4214	19594.71	165.4208	19596.8
165.6708	19591.43	165.6675	19589.56	165.672	19594.48	165.6662	19600.02	165.6757	19600.65
165.9197	19588.74	165.921	19595.4	165.9171	19594.41	165.922	19588.64	165.9201	19597.07
166.172	19585.43	166.1684	19584.76	166.1736	19592.9	166.1679	19590.04	166.1718	19599.82
166.4227	19592.23	166.4251	19588	166.4184	19596.37	166.4232	19597.16	166.4205	19598.59
166.675	19586.56	166.6681	19593.17	166.6727	19590.9	166.666	19602.44	166.6737	19596.71
166.9196	19596.66	166.9203	19590.8	166.9194	19595.65	166.9228	19587.85	166.9197	19593.29
167.1726	19585.22	167.1677	19600.97	167.2107	19592.15	167.1697	19589.85	167.1724	19593.38
167.4243	19591.06	167.4225	19588.19	167.4234	19595.37	167.4243	19593.33	167.4207	19601.38
167.6743	19583.32	167.6703	19590.56	167.6725	19594.09	167.6687	19594.24	167.672	19593.3
167.9234	19585.06	167.9225	19591.61	167.9203	19592.45	167.9216	19592.04	167.9234	19598.99
168.1718	19584.64	168.1701	19594.07	168.1745	19592.1	168.1684	19591.06	168.1716	19595.4
168.4257	19580.51	168.4263	19591.53	168.4196	19598.8	168.423	19588.9	168.4204	19596.77
168.6708	19591.33	168.6701	19584.95	168.6738	19590.92	168.6679	19593.62	168.6732	19595
168.9204	19586	168.9219	19597.68	168.9208	19592.08	168.9245	19597.89	168.9242	19596.43
169.1753	19594.93	169.17	19588.13	169.178	19594.92	169.1674	19591.79	169.1754	19597.12
169.4223	19589.8	169.4237	19588.91	169.4193	19591.12	169.4502	19592.9	169.4203	19593.53
169.671	19590.5	169.6697	19589.78	169.6729	19592.09	169.6745	19595.1	169.6742	19594.08
169.9227	19590.22	169.923	19591.3	169.92	19597.07	169.9235	19593.05	169.9234	19595.62
170.1732	19583.27	170.17	19597.9	170.1759	19597.18	170.1705	19595.24	170.1763	19591.67
170.4238	19578.57	170.4235	19588.5	170.4206	19589.87	170.4225	19590.26	170.4218	19600.19
170.6728	19585.23	170.6704	19592.53	170.6745	19591.49	170.6682	19591.65	170.6724	19588.92
170.9204	19586.36	170.9243	19583.49	170.9241	19593.5	170.9227	19591.8	170.9267	19597.3
171.1728	19592.41	171.1708	19586.35	171.1754	19585.91	171.169	19589.42	171.1734	19592.89
171.424	19585.68	171.433	19583.83	171.4204	19589.18	171.4284	19594.6	171.4242	19598.05
171.6743	19587.39	171.6699	19587.3	171.676	19590.41	171.6687	19588.36	171.6728	19603.06
171.9203	19592.67	171.9229	19589	171.9203	19589.26	171.9219	19603.34	171.9239	19589.65
172.1735	19584.11	172.1709	19591.9	172.1764	19594.1	172.1706	19586.14	172.1749	19584.41
172.4463	19588.4	172.4246	19588.64	172.4221	19587.19	172.4263	19593.11	172.4222	19597.78
172.6923	19588.57	172.6694	19591.93	172.6734	19588.2	172.6703	19589.46	172.6752	19589
172.9197	19584.16	172.924	19595.83	172.9229	19599.67	172.9217	19589.93	172.9286	19593.3
173.1735	19591.64	173.1699	19588.5	173.1871	19591.66	173.1696	19594.29	173.1737	19591.73
173.4244	19585.31	173.4232	19590.2	173.425	19602.21	173.4244	19591.56	173.4225	19592.74
173.6743	19589.5	173.6706	19583.62	173.6745	19589.77	173.67	19588.43	173.6728	19595.45

173.9239	19581.23	173.9249	19589.13	173.9215	19592.15	173.9265	19590.71	173.9239	19596.61
174.1751	19584.11	174.1707	19587.61	174.1785	19586.46	174.1694	19591.88	174.1748	19597.93
174.4241	19595.71	174.4239	19591.01	174.4213	19588.34	174.4301	19595.29	174.4273	19593.29
174.6736	19588.11	174.6717	19589.36	174.6749	19589.75	174.671	19594.69	174.6754	19595.19
174.9228	19588.72	174.9241	19593.97	174.9221	19593.33	174.9242	19592.19	174.9352	19592.44
175.1756	19582.08	175.1705	19589.73	175.1803	19591.26	175.1706	19596.37	175.1771	19594.59
175.4267	19582.82	175.4238	19588.42	175.4235	19592.56	175.4257	19593.12	175.424	19589.7
175.6725	19590.52	175.6708	19585.93	175.6742	19586.29	175.6699	19591.66	175.6789	19595.78
175.9228	19588.79	175.925	19590.9	175.9222	19589.21	175.925	19594.79	175.9261	19592.73
176.1748	19586.03	176.174	19582.15	176.1791	19598.64	176.1726	19593.04	176.177	19593.23
176.4263	19583.6	176.4314	19587.84	176.4214	19591.48	176.4268	19592.95	176.424	19596.71
176.6759	19589.21	176.6727	19590.9	176.6748	19596.2	176.6701	19593.03	176.6762	19587.03
176.9262	19583.99	176.9269	19588.66	176.9222	19594.04	176.9252	19591.59	176.9259	19591.15
177.1754	19587.63	177.1748	19587.17	177.1815	19596.42	177.172	19592.7	177.1814	19590.67
177.4284	19592.79	177.4284	19588.25	177.4217	19585.16	177.4282	19589.65	177.4283	19590.61
177.6746	19591	177.6744	19591.05	177.6763	19588.33	177.672	19585.25	177.6765	19577.9
177.923	19589.26	177.9279	19589.34	177.9226	19591.55	177.9242	19589.93	177.9257	19593.36
178.178	19584.33	178.1762	19588.39	178.1796	19592.69	178.1712	19595.76	178.1768	19587.9
178.43	19586.23	178.4271	19586.11	178.422	19585.55	178.4272	19590.77	178.4245	19594.6
178.6746	19587.33	178.6733	19592.72	178.6756	19586.18	178.6774	19594.17	178.6784	19593.47
178.9255	19588.8	178.9297	19588.85	178.9289	19594.1	178.9285	19592.77	178.9276	19598.78
179.179	19583.09	179.1749	19589.29	179.1833	19594.38	179.1732	19590.93	179.1786	19588
179.4297	19589.54	179.4301	19587.73	179.4232	19594.66	179.4279	19596.17	179.4274	19589.86
179.6752	19595.35	179.6728	19589.49	179.6755	19594.36	179.6736	19589.13	179.6763	19590.67
179.9269	19590.82	179.9259	19586.35	179.9256	19596.17	179.9273	19588.33	179.9281	19586.52
180.1815	19583.56	180.1778	19588.69	180.1845	19593.85	180.1753	19593.61	180.1765	19595.64
180.4301	19589.1	180.4292	19587.42	180.4244	19591.11	180.4308	19590.14	180.4257	19594.14
180.679	19591.99	180.6743	19588.99	180.6756	19588.9	180.6733	19588.6	180.6801	19596.51
180.925	19586.31	180.9266	19590.19	180.9254	19583.82	180.9276	19589.15	180.9274	19588.72
181.1781	19576.28	181.1757	19588.28	181.1826	19589.85	181.176	19598.32	181.1778	19591.91
181.4303	19591.7	181.4343	19587.85	181.4257	19590.72	181.4299	19594.55	181.4279	19587.07
181.6761	19585.41	181.6749	19590.24	181.6756	19587.45	181.6734	19596.13	181.6777	19596.21
181.9265	19576.91	181.928	19588.26	181.9268	19587.71	181.9276	19587.71	181.9263	19590.09
182.1778	19587.31	182.1819	19593.49	182.1831	19591.52	182.176	19590.25	182.1775	19587.53
182.4311	19597.12	182.4297	19585.91	182.4298	19587.45	182.429	19598.09	182.4289	19597.38
182.6774	19577.76	182.675	19587.2	182.6786	19590.34	182.674	19585.45	182.6785	19592.09
182.9293	19585.13	182.927	19588.23	182.9262	19587.22	182.9261	19595.14	182.928	19594.89
183.1783	19582.05	183.1768	19590.05	183.183	19590.79	183.1785	19588.8	183.1786	19595.29
183.4314	19584.09	183.4304	19590.78	183.4272	19593.44	183.4294	19590.72	183.4329	19592.32
183.6782	19583.98	183.6757	19584.85	183.6773	19588.09	183.6746	19586.59	183.6805	19591.07
183.9266	19590.05	183.9278	19591.06	183.9271	19586.7	183.9289	19599.17	183.9277	19588.45
184.1817	19585.73	184.1764	19587.12	184.1849	19579.19	184.1763	19592.89	184.1785	19590.51
184.4309	19585.16	184.4317	19585.37	184.4256	19596.8	184.4634	19586.15	184.4302	19589.46
184.6771	19579.7	184.6755	19593.86	184.6779	19589.29	184.6823	19594.39	184.6799	19596.93
184.9275	19584.05	184.9282	19592.66	184.9278	19597.32	184.9277	19593.02	184.9286	19596.82
185.1797	19592.74	185.1767	19589.4	185.1832	19587.78	185.1761	19592.15	185.1806	19592.24
185.4328	19578.88	185.4329	19584.55	185.4275	19589.27	185.4288	19600.78	185.4311	19594.54
185.6775	19582.48	185.6754	19589.96	185.678	19590.78	185.6746	19588.53	185.6807	19596.36
185.9275	19581.88	185.9276	19590.33	185.9271	19593.94	185.929	19594.34	185.9287	19583.34
186.184	19586.62	186.1783	19584.61	186.1845	19587.03	186.178	19587.02	186.1801	19590.66
186.433	19587.05	186.4351	19588.09	186.4273	19586.85	186.4334	19586.59	186.431	19593.91
186.6824	19584.78	186.6778	19591.67	186.6791	19585.59	186.675	19595.14	186.6801	19591.91
186.9299	19588.09	186.9271	19587.44	186.9272	19586.44	186.9289	19592.97	186.9303	19590.8
187.1829	19579.07	187.1768	19595.28	187.2005	19594.04	187.1789	19587.5	187.1791	19597.92
187.4509	19585.17	187.4356	19588.58	187.4283	19590.6	187.4317	19597.24	187.4305	19586.67
187.6813	19586.89	187.6774	19585.01	187.6788	19582.34	187.6784	19593.32	187.6795	19585.46
187.931	19587.25	187.9294	19589.45	187.9297	19583.58	187.9281	19585.09	187.9308	19591.45
188.1806	19589.5	188.1822	19588.01	188.1936	19587.64	188.1778	19588.34	188.1813	19589.83
188.4345	19584.66	188.4333	19588.71	188.4302	19594.22	188.4382	19594.61	188.432	19589.59
188.6806	19594.79	188.6769	19588.24	188.6794	19594.28	188.677	19591.82	188.6821	19592.68
188.9294	19586.88	188.9296	19593.38	188.9288	19588.2	188.9327	19585	188.9295	19588.7
189.1831	19590.05	189.1799	19588.21	189.186	19586.2	189.1775	19593.21	189.1814	19587.87
189.4327	19582.04	189.4326	19579.1	189.4291	19588.88	189.4321	19591.97	189.4307	19597.72
189.6798	19585.53	189.6773	19585.89	189.6805	19591.15	189.6769	19591.61	189.6842	19594.07
189.9294	19584.61	189.9288	19590.88	189.9309	19587.53	189.931	19588.31	189.9335	19597.03
190.1819	19593.32	190.1784	19583.57	190.1856	19588.29	190.1785	19592.7	190.1833	19585.39
190.4338	19584.39	190.4312	19592.3	190.4314	19584.47	190.4328	19583.39	190.4319	19594.94
190.6846	19584.36	190.6781	19585.06	190.6798	19588.81	190.6772	19591.26	190.6812	19585.88
190.9299	19582.67	190.9309	19590.85	190.9308	19586.41	190.9329	19585.26	190.9309	19591.43
191.1807	19591.63	191.1791	19589	191.1868	19587.66	191.1783	19594.13	191.1816	19592.28

191.4345	19582.49	191.4371	19593.26	191.4281	19588.47	191.4389	19589.62	191.4308	19591.72
191.6834	19587.95	191.6798	19593.08	191.6856	19584.31	191.6771	19586.95	191.6832	19586.31
191.9315	19588	191.9353	19589.68	191.9345	19584.91	191.9308	19586.18	191.935	19596.15
192.1817	19583.99	192.1784	19592.97	192.1852	19601.69	192.1827	19593.64	192.1824	19593.02
192.4324	19591.57	192.4328	19593.86	192.4302	19593.08	192.4353	19588.48	192.4316	19595.42
192.6849	19587.12	192.6795	19586.43	192.6809	19589.52	192.6797	19586.61	192.684	19594.77
192.9302	19582.54	192.9321	19584.46	192.9314	19583.02	192.9342	19595.26	192.9337	19592.5
193.1828	19582.38	193.1795	19585.04	193.1876	19586.13	193.1843	19589.11	193.1822	19590.81
193.4324	19586.78	193.4336	19590.7	193.4295	19592.92	193.4418	19591.75	193.4325	19593.25
193.6811	19586.09	193.6785	19588.67	193.6819	19588.98	193.6883	19592.96	193.6862	19582.24
193.9316	19584.62	193.9329	19587.31	193.9315	19593.57	193.9387	19590.01	193.9312	19588.99
194.1841	19581	194.1837	19589.18	194.1894	19587.72	194.1846	19585.43	194.1837	19588.55
194.4366	19592.81	194.4374	19585.89	194.4314	19592.34	194.441	19593.94	194.4321	19594.2
194.682	19583.77	194.6788	19591.01	194.6862	19583.75	194.6822	19591.52	194.6886	19588.27
194.9339	19584.88	194.9331	19589.04	194.9327	19584.6	194.938	19596.53	194.9322	19593.15
195.1847	19592.81	195.182	19587.1	195.1874	19586.65	195.1829	19584.06	195.1851	19596.66
195.4364	19582.67	195.4392	19579.9	195.4297	19590.63	195.4373	19588.63	195.4312	19586.14
195.6849	19582.95	195.679	19584.7	195.6833	19592.25	195.6811	19590.51	195.6845	19589.56
195.9314	19587.12	195.9309	19592.67	195.9326	19589.61	195.9336	19589.09	195.9352	19592.33
196.1839	19581.79	196.1829	19578.87	196.1895	19593.18	196.184	19589.21	196.1837	19597.9
196.4367	19588.36	196.4389	19586.68	196.4311	19591.26	196.4361	19594.38	196.4344	19592.62
196.6848	19583.04	196.68	19589.61	196.6827	19594.27	196.6811	19591.06	196.6849	19595.19
196.9313	19580.4	196.9367	19584.4	196.932	19593.21	196.9326	19593.61	196.9338	19593.03
197.1832	19576.23	197.1821	19587.09	197.1881	19580.59	197.1797	19587.29	197.1844	19586.27
197.4376	19584.45	197.4371	19584.18	197.4316	19592.48	197.4367	19589.79	197.4338	19599.7
197.6849	19585.73	197.6825	19585.21	197.6884	19581.63	197.6816	19594.32	197.6867	19595.56
197.9326	19590.42	197.9339	19587.04	197.9315	19588.57	197.9346	19587.78	197.9347	19595.6
198.1835	19590.38	198.1816	19583.45	198.1892	19592.26	198.1811	19587.54	198.1837	19590.75
198.4374	19582.54	198.4354	19591.74	198.4322	19592.2	198.4375	19588.92	198.4346	19594.43
198.6848	19580.15	198.6809	19582.09	198.6846	19584.14	198.6834	19587.8	198.6853	19594.52
198.9328	19579.03	198.934	19585.19	198.9327	19590.77	198.9357	19592.39	198.9359	19596.79
199.1878	19587.77	199.1809	19592.21	199.1969	19590.02	199.1816	19596.18	199.1864	19592.4
199.4365	19582.24	199.4357	19583.6	199.4345	19588.52	199.4495	19590.14	199.4352	19589.1
199.6859	19580.75	199.6818	19587.9	199.6862	19588.17	199.6816	19587.31	199.6865	19585.5
199.9343	19587.47	199.9347	19584.75	199.9342	19585.44	199.94	19591.51	199.9367	19588.29
200.1847	19586.19	200.1818	19590.47	200.1903	19578.47	200.1829	19585.75	200.1854	19588.51
200.4429	19584.47	200.4422	19579.13	200.4373	19590.24	200.4436	19589.73	200.439	19587.44
200.69	19580.08	200.6868	19585.51	200.689	19590.53	200.6864	19592.66	200.6949	19597.25
200.9362	19585.67	200.9396	19589.34	200.9383	19595.58	200.9397	19587.23	200.941	19598.57
201.195	19582.32	201.1877	19586.36	201.1975	19594.5	201.1857	19589.45	201.1894	19598.56
201.4418	19584.04	201.4628	19588.97	201.4421	19588.84	201.446	19590.98	201.4391	19589.02
201.6887	19580.46	201.6883	19586.32	201.6888	19595.65	201.6862	19579.95	201.6902	19586.89
201.9422	19583.8	201.9424	19584.87	201.9371	19581.66	201.9371	19579.08	201.9406	19593.41
202.1915	19583.1	202.1886	19592.36	202.1962	19584.45	202.1864	19579.27	202.1903	19597.06
202.4624	19581.78	202.4432	19586.2	202.4377	19587.51	202.4447	19593.24	202.44	19590.19
202.6929	19577.93	202.6882	19589.58	202.6906	19592.6	202.6874	19590.36	202.6937	19591.53
202.9378	19584.88	202.9391	19589.5	202.9366	19586.74	202.9398	19591.4	202.939	19593.33
203.1923	19580.23	203.1885	19592.83	203.1955	19584.64	203.1863	19589.37	203.1919	19586.82
203.4431	19579.65	203.4447	19580.96	203.4401	19584.97	203.4452	19592.73	203.4395	19594.75
203.6917	19582.14	203.689	19583.67	203.6895	19590.48	203.689	19598.55	203.6929	19588.91
203.9394	19582.6	203.9414	19587.77	203.9392	19592.19	203.9425	19594.27	203.9391	19588.48
204.1948	19584.82	204.1897	19587.39	204.2074	19590.59	204.1875	19581.86	204.1929	19595.42
204.4446	19583.5	204.4435	19587.37	204.4437	19596.52	204.4446	19584.36	204.4411	19590.96
204.6929	19578.2	204.689	19585.58	204.6989	19585.83	204.6896	19590.39	204.6929	19588.45
204.9394	19580.3	204.9423	19589.32	204.9419	19588.19	204.9397	19586.19	204.9412	19585.35
205.1959	19587.23	205.1886	19591.57	205.1976	19589.61	205.1891	19586.07	205.1927	19594.54
205.4456	19584.49	205.4445	19591.49	205.4418	19591.36	205.4472	19589.24	205.4413	19592.16
205.6917	19582.4	205.6894	19591.21	205.6938	19589.67	205.6878	19589.21	205.6942	19592.42
205.9397	19589.31	205.941	19586.84	205.9429	19591.9	205.9424	19586.39	205.9427	19583.36
206.1936	19590.29	206.1909	19589.33	206.2014	19593	206.1889	19586.06	206.1948	19581.28
206.4442	19583	206.449	19594.48	206.4397	19590.87	206.4472	19589.1	206.4405	19587.87
206.6917	19574.86	206.6891	19591.68	206.6943	19591.72	206.6889	19584.82	206.6935	19585.13
206.9401	19591.89	206.9414	19580.04	206.9396	19587.02	206.9419	19588.61	206.9459	19589.26
207.1948	19584.2	207.1896	19584.17	207.2133	19589.06	207.1898	19598.36	207.1948	19589.66
207.4462	19584.73	207.447	19581.53	207.444	19590.35	207.446	19585.05	207.4412	19585.38
207.6938	19578.61	207.6913	19587.93	207.6923	19584.33	207.6897	19591.42	207.6945	19580.6
207.9427	19583.9	207.942	19583.38	207.9399	19586.58	207.9467	19584.9	207.9434	19587.22
208.1959	19584.73	208.1898	19591.27	208.2014	19586.15	208.1913	19589.66	208.1931	19590.48
208.4457	19582.59	208.445	19591.87	208.4385	19588.96	208.4513	19588.19	208.4421	19588.3
208.6983	19573.58	208.6898	19583.4	208.6941	19593.43	208.6902	19584.99	208.6939	19588.19

208.9401	19588.09	208.9433	19587.88	208.9394	19590.57	208.9442	19584.91	208.9414	19593.01
209.1959	19583.34	209.1914	19586.79	209.1999	19586.53	209.191	19585.51	209.1961	19589.36
209.4463	19578.73	209.4492	19593.01	209.4413	19593.48	209.4482	19595.93	209.4417	19590.15
209.6941	19589.63	209.6926	19589.74	209.6912	19586.45	209.6903	19590.37	209.6936	19583.25
209.9416	19589.63	209.9495	19586.3	209.9404	19596.32	209.944	19593.39	209.9451	19595.55
210.1932	19581.57	210.1966	19593.1	210.1978	19584.9	210.1909	19593.71	210.194	19589.29
210.4477	19585.35	210.4463	19584	210.4428	19592.46	210.4521	19588.8	210.4432	19587.27
210.6949	19586.35	210.6971	19579.38	210.695	19590.48	210.6923	19594.21	210.6955	19591.91
210.9416	19577.31	210.9452	19587.61	210.9421	19590.85	210.9432	19595.87	210.9441	19583.17
211.1948	19582.46	211.1921	19585	211.1997	19585.41	211.1913	19596.64	211.1945	19588.37
211.4484	19579.75	211.4487	19584.55	211.4414	19587.77	211.4505	19590.4	211.4435	19582.52
211.6924	19583.64	211.691	19584.96	211.6949	19583.55	211.6911	19589.32	211.6959	19589.94
211.9436	19579.23	211.9433	19586.7	211.9428	19581.43	211.9442	19582.38	211.9451	19595.52
212.197	19584.91	212.1929	19590.36	212.2001	19582.1	212.192	19593.13	212.1958	19585.79
212.4474	19577.76	212.4477	19580.72	212.4481	19596.49	212.4623	19588.45	212.4447	19593.93
212.695	19586.21	212.6923	19582.09	212.6983	19594.6	212.7033	19588.9	212.6972	19592.43
212.9432	19576.81	212.9433	19586.81	212.9441	19588.34	212.9446	19591.01	212.9449	19590.47
213.1974	19580.9	213.1926	19588.11	213.2018	19585.53	213.1927	19590.22	213.1949	19585.26
213.4532	19580.7	213.4479	19587.63	213.4431	19585.72	213.4482	19592.28	213.4464	19594.89
213.6911	19581.63	213.692	19585.07	213.6941	19590.12	213.6927	19592.1	213.6948	19587.63
213.9477	19577.94	213.9438	19583.55	213.9424	19577.14	213.9468	19591.33	213.9453	19590.73
214.1963	19588.55	214.193	19589.07	214.2041	19591.57	214.1919	19586.1	214.1974	19590.8
214.4491	19583.3	214.4526	19592.97	214.4385	19596.73	214.4749	19589.19	214.4461	19588.98
214.6957	19585.78	214.6988	19586.95	214.6956	19581.95	214.7178	19591.31	214.6968	19593.16
214.9465	19583.88	214.952	19583.45	214.9436	19590.24	214.9528	19592.11	214.9439	19587.9
215.1963	19578.89	215.1943	19587.66	215.2008	19590.68	215.1927	19591.43	215.1954	19587.64
215.455	19585.02	215.4492	19588.29	215.4439	19585.69	215.4483	19587.87	215.4456	19586.99
215.7005	19587.39	215.6941	19591.13	215.6954	19588.86	215.6934	19587.84	215.6963	19588.24
215.9452	19582	215.9447	19579.14	215.9455	19591.58	215.9456	19590.82	215.9447	19580.11
216.1982	19589.41	216.1977	19585.85	216.2013	19587.13	216.1931	19589.44	216.1974	19588.51
216.4503	19582.94	216.4529	19584.22	216.4456	19587.86	216.4492	19587.47	216.4486	19584.73
216.6962	19587.44	216.6906	19593.48	216.696	19585.93	216.6923	19590	216.6968	19586.8
216.9463	19583.28	216.946	19580.38	216.9446	19585.06	216.9453	19581.24	216.9468	19586.68
217.1975	19584.02	217.1954	19587.69	217.2007	19592.42	217.1931	19584.16	217.1972	19587.83
217.4695	19587.59	217.4518	19583.9	217.4442	19588.71	217.4527	19585.92	217.447	19589.85
217.7107	19584.12	217.6972	19586.49	217.6968	19580.59	217.6907	19578.73	217.6994	19594.79
217.9491	19576.76	217.9447	19586.01	217.944	19584.96	217.9445	19584.62	217.9473	19585.79
218.1991	19584.87	218.1961	19581.31	218.2031	19592.4	218.1939	19594.45	218.1975	19586.23
218.4504	19590.24	218.4502	19590.62	218.4661	19585.35	218.4503	19593.85	218.4462	19592.69
218.6975	19578.7	218.6962	19589.24	218.7081	19586.37	218.6957	19591.95	218.6964	19586.14
218.9458	19579.25	218.9489	19582.01	218.9476	19581.21	218.9503	19594.52	218.9471	19577.25
219.2014	19581.08	219.1949	19583.89	219.2055	19590.34	219.1931	19595.73	219.1989	19584.49
219.4502	19582.1	219.4521	19587.89	219.4429	19586.84	219.4507	19584.73	219.4477	19593.2
219.698	19585.43	219.6924	19590.64	219.6977	19593.28	219.6953	19581.59	219.7015	19587.44
219.9513	19583.31	219.9485	19579.26	219.9463	19588.06	219.9489	19585.38	219.9488	19587.78
220.1944	19581.34	220.1958	19588.59	220.203	19587.04	220.1949	19594.33	220.1985	19593.3
220.4503	19581.99	220.4506	19587.68	220.4435	19586.89	220.453	19587.12	220.4476	19586.33
220.6987	19587.21	220.6979	19590.72	220.7029	19589.47	220.692	19578.27	220.6981	19581.25
220.9547	19577.74	220.9484	19580.9	220.9426	19584.47	220.9489	19584.64	220.9502	19586.54
221.1999	19590.13	221.1965	19585.92	221.2022	19590.81	221.1974	19588.53	221.1986	19585.88
221.4522	19584.59	221.4821	19583.45	221.4431	19582.54	221.4549	19588.15	221.4544	19591.51
221.6974	19586.82	221.9548	19584.81	221.6973	19593.5	221.6932	19587.76	221.698	19594.21
221.9486	19590.11	222.2042	19585.86	221.9461	19591.27	221.9485	19586.13	221.9487	19586.1
222.2027	19583.69	222.4596	19591.9	222.2036	19587.58	222.196	19593.66	222.1994	19590.68
222.4483	19583.65	222.7019	19585.92	222.4437	19591.32	222.4542	19585.83	222.4495	19591.27
222.6998	19579.85	222.9551	19584.19	222.6985	19590.04	222.6944	19587.79	222.7018	19591.47
222.95	19580.49	223.2043	19583.51	222.9475	19592.26	222.9471	19583.99	222.9446	19588.67
223.2031	19583.65	223.4599	19581.78	223.2024	19589.05	223.1991	19590.02	223.2177	19593.34
223.4508	19582.11	223.7039	19587.11	223.4439	19587.8	223.461	19583.7	223.4623	19590.06
223.6935	19581.49	223.9566	19587.05	223.6989	19584.2	223.7119	19596.35	223.7075	19578.86
223.9486	19582	224.2017	19587.08	223.951	19589.97	223.9596	19580.24	223.9524	19586.42
224.204	19580.38	224.4617	19579.17	224.201	19588.25	224.2029	19590.21	224.1952	19590.64
224.459	19581.28	224.7043	19580.34	224.4441	19580.05	224.4523	19586.43	224.4498	19592.95
224.7042	19588.13	224.9592	19580.44	224.7005	19582.08	224.6961	19593.48	224.6995	19587.08
224.9489	19584.75	225.2009	19586.87	224.9431	19577.99	224.95	19580.02	224.9512	19594.87
225.2044	19579.22	225.4638	19587.06	225.2069	19584.08	225.1979	19594.23	225.1966	19585.23
225.452	19585.65	225.7089	19584.7	225.4499	19589.11	225.4547	19585.75	225.4507	19586.06
225.6983	19579.66	225.9566	19584.55	225.6993	19584.03	225.6992	19584.55	225.6956	19586.52
225.9512	19583.98	226.2043	19580.67	225.9471	19591.62	225.9528	19589.88	225.952	19586.67
226.1963	19583.85	226.4661	19586.25	226.2103	19585.05	226.1955	19582.69	226.1971	19581.04

226.4637	19576.12	226.7111	19580.2	226.4549	19584.44	226.4555	19591.86	226.4515	19583.81
226.7176	19588.64	226.9586	19588.86	226.702	19583.95	226.7016	19583.66	226.6963	19592.51
226.9628	19580.31	227.2043	19589.57	226.9467	19586.72	226.9477	19583.2	226.952	19590.74
227.2152	19584.6	227.4793	19584.53	227.2272	19583.89	227.1991	19584.68	227.1979	19590.22
227.4631	19583.16	227.7291	19584.17	227.7044	19587.31	227.4547	19591.99	227.4505	19587.92
227.7101	19585.08	227.9782	19582	227.9503	19587.92	227.6997	19581	227.699	19581.88
227.958	19582.77	228.2222	19582.99	228.2101	19584.4	227.9439	19586.57	227.9534	19591.55
228.207	19578.5	228.4736	19589.76	228.4575	19590.28	228.1989	19588.09	228.2009	19588.43
228.4608	19585.02	228.719	19578.02	228.7063	19595.95	228.4549	19585.84	228.4464	19587.84
228.7087	19585.22	228.9701	19582.23	228.9509	19581.15	228.7027	19589.75	228.705	19593.34
228.9575	19581.35	229.2163	19589.09	229.2126	19583.95	228.9506	19588.24	228.9544	19588.37
229.2091	19582.44	229.4684	19585.18	229.4588	19581.5	229.1961	19583.19	229.2023	19590.2
229.4597	19578.83	229.7142	19588.7	229.7067	19584.36	229.473	19591.95	229.4488	19595.46
229.7084	19583.08	229.9615	19591.75	229.952	19591.21	229.7218	19590.61	229.7016	19594.99
229.959	19581.94	230.2079	19584.59	230.2206	19584.87	229.9751	19585.19	229.9509	19587.37
230.2113	19582.59	230.4596	19583.37	230.4734	19589.11	230.2206	19589.13	230.1997	19587.28
230.4628	19583.09	230.7068	19584.91	230.725	19584.92	230.4752	19592.86	230.4481	19581.35
230.7123	19576.18	230.9556	19583.81	230.974	19595.49	230.7278	19585.23	230.7014	19585.43
230.9596	19589	231.2035	19578.68	231.2278	19588.25	231.2036	19587.59	230.9526	19587.59
231.212	19582.92	231.4659	19586.33	231.4779	19583.89	231.4588	19585.84	231.2012	19581.92
231.4652	19592.71	231.7167	19592.7	231.728	19588.44	231.707	19590.77	231.4505	19577.78
231.7174	19585.94	231.9701	19584.14	231.9784	19584.57	231.9565	19586.86	231.7019	19579.74
231.9681	19589.96	232.22	19577.95	232.2332	19582.66	232.2028	19587.19	231.954	19589.65
232.2287	19577.98	232.4731	19586.21	232.7122	19583.57	232.4598	19592.29	232.2043	19584.61
232.7106	19583.03	232.7215	19583.15	232.9586	19584.2	232.7082	19588.53	232.4531	19588.83
232.9752	19582.96	232.9729	19584.2	233.2147	19583.52	232.9582	19589.66	232.7025	19591.71
233.2313	19576.11	233.2221	19580.41	233.466	19585.75	233.2123	19582.59	232.9522	19591.33
233.4878	19583.69	233.4769	19583.77	233.7158	19585.93	233.4672	19587.71	233.205	19587.19
233.9671	19581.32	233.7257	19590.26	233.9663	19584.09	233.7149	19583.51	233.4552	19583.35
234.2217	19578.78	233.9789	19586.94	234.2241	19584.07	233.9738	19587.5	233.7109	19591.47
234.4765	19585.22	234.2312	19583.11	234.4756	19589.47	234.2221	19577.82	233.9615	19587.36
234.7301	19582.36	234.4887	19583.98	234.7289	19582.86	234.4772	19594.29	234.2149	19584.08
234.9813	19584.16	234.9688	19591.13	234.9775	19588.97	234.7291	19585.21	234.465	19583.87
235.236	19580.36	235.2232	19577.23	235.2342	19583.31	234.9809	19588.7	234.718	19587.98
235.495	19578.33	235.4791	19587.94	235.486	19580.15	235.4666	19585.69	234.9688	19590.35
235.9754	19573.75	235.7302	19583.49	235.9656	19588.08	235.7197	19585.17	235.2209	19583.73
236.2366	19585.77	235.9833	19585.63	236.2237	19585.85	235.9712	19582.89	235.4734	19581.78
236.4962	19585.96	236.2337	19583.44	236.4773	19586.6	236.2204	19585.93	235.7256	19589.26
236.7612	19584.23	236.4924	19586.56	236.7287	19586.96	236.4772	19586.99	235.98	19577.44
237.2487	19573.89	236.9746	19589.58	236.9796	19596.66	236.7293	19582.3	236.4619	19586.19
237.5066	19584.49	237.2245	19580	237.2372	19588.44	236.9835	19583.87	236.7163	19586.83
237.7633	19579.25	237.4832	19585.02	237.7239	19582.19	237.2376	19582.81	236.9689	19587.8
238.0176	19582.37	237.7377	19583.29	237.9758	19584.44	237.7175	19586.69	237.2256	19590.67
238.5038	19579.23	237.9924	19583.24	238.2344	19584.21	237.9725	19585.46	237.4956	19585.45
238.7621	19582.88	238.2492	19586.05	238.4871	19587.36	238.2136	19585.16	237.9781	19586.17
239.0154	19579.22	238.7349	19582.46	238.7438	19586.5	238.4763	19591.61	238.2362	19590.45
239.2736	19585.55	238.9913	19583.44	238.9976	19581.71	238.7176	19584.21	238.4915	19586.05
239.7582	19576.32	239.2456	19580.33	239.48	19589.76	238.9734	19586.15	238.7479	19590.7
240.0139	19576.08	239.4932	19581.76	239.735	19585.71	239.2154	19583.74	239.0034	19592.95
240.2728	19579.54	239.7345	19583.1	239.9875	19594.84	239.4764	19584.95	239.4865	19586.35
240.53	19577.59	239.9998	19588.98	240.2536	19582.57	239.7241	19587.88	239.7428	19590.57
241.0089	19577.15	240.2435	19586.74	240.7381	19589.85	239.9736	19588.74	240.0016	19588.42
241.2628	19591.16	240.4914	19574.95	240.9853	19587.4	240.2157	19588.19	240.2596	19589.15
241.513	19581.59	240.7302	19588.14	241.2349	19585.03	240.4767	19587.73	240.7387	19588.59
241.7588	19580.98	240.9833	19583.04	241.4767	19588.44	240.7202	19594.18	240.9951	19593.87
242.0085	19581.17	241.2304	19588.69	241.7339	19579.61	240.9665	19585.05	241.24	19582.81
242.2625	19581.52	241.5069	19580.12	241.9841	19582.58	241.2136	19580.88	241.4926	19592.98
242.5103	19585.49	241.9897	19585.46	242.2366	19589.11	241.4746	19588.18	241.7431	19581.51
242.7601	19575.3	242.2346	19586.47	242.4806	19585.95	241.7146	19589.61	241.99	19586.15
243.0052	19583.75	242.4907	19579.09	242.7337	19587.93	241.9685	19588.17	242.245	19589.51
243.2636	19583.46	242.7348	19579.26	242.9816	19591.67	242.2146	19587.41	242.4914	19590.35
243.512	19578.57	242.9881	19588.35	243.2381	19581.41	242.4734	19585.59	242.7384	19585.33
243.7587	19585.27	243.2339	19589.28	243.4812	19582.18	242.7151	19578.14	242.9894	19583.84
244.0096	19582.21	243.4904	19587.52	243.7343	19584.21	242.9717	19583.64	243.2412	19588.87
244.2593	19584.45	243.7359	19587.03	243.9775	19584.47	243.2171	19588.19	243.4879	19579.78
244.5123	19589.64	243.9888	19581.69	244.2397	19584.16	243.4719	19584.89	243.7397	19585.95
244.7623	19587.43	244.2382	19581	244.4831	19586.74	243.7151	19590.72	243.9867	19586.46
245.0086	19583.17	244.4919	19583.58	244.7348	19588.82	243.9717	19580.1	244.2434	19584.61
245.2616	19581.64	244.7366	19589.03	244.9783	19585	244.2179	19587.87	244.4912	19591.07
245.5171	19581.54	244.9911	19581.02	245.2395	19589.31	244.4895	19587.55	244.7372	19588.33

245.7662	19576.87	245.2353	19585.84	245.4847	19584.54	244.74	19587.06	244.9928	19578.95
246.0137	19586.35	245.4911	19585.26	245.7294	19582.08	245.2188	19582.59	245.2403	19587.9
246.2634	19579.19	245.7354	19588.68	245.9829	19580.57	245.476	19585.94	245.4888	19582.46
246.5133	19581.4	245.99	19588.73	246.2369	19581.31	245.7195	19588.98	245.7444	19591.83
246.7624	19583.36	246.2374	19582.37	246.4806	19581.92	245.9739	19585.14	245.9919	19588.06
247.0105	19579.68	246.4947	19578.99	246.7357	19585.01	246.2191	19592.9	246.2384	19588.32
247.2641	19580.06	246.7384	19583.86	246.9804	19584.81	246.476	19589.53	246.4924	19586.29
247.5361	19583.4	246.9894	19587.29	247.2557	19594.96	246.7204	19583.69	246.7389	19587.29
248.0186	19574.27	247.2376	19581.27	247.5025	19584.79	246.9748	19586.03	246.9887	19583.92
248.2714	19580.49	247.4933	19580.14	247.7509	19592.11	247.2202	19585.53	247.2439	19592.01
248.5246	19587.77	247.7391	19584.95	247.9972	19588.63	247.4767	19585.18	247.4933	19583.43
248.7745	19576.15	247.998	19579.21	248.25	19590.56	247.72	19586.25	247.7395	19581.98
249.0238	19582.25	248.2324	19587.28	248.5001	19590.77	247.9755	19580.93	247.9967	19582.93
249.2746	19581.01	248.4928	19581.99	248.7493	19583.48	248.2203	19586.96	248.2465	19587.96
249.5255	19585.91	248.7382	19587.74	248.9993	19584.83	248.4773	19595.44	248.4941	19586.94
249.7764	19584.39	248.9929	19578.42	249.2531	19584.52	248.7194	19587.95	248.7419	19590.86
250.0267	19581.9	249.2372	19585	249.4987	19583.69	248.9759	19589.85	248.9945	19592.94
250.2829	19579.1	249.4922	19584.24	249.7473	19584.41	249.2199	19583.85	249.247	19588.49
250.535	19579.22	249.7377	19581.23	249.9951	19590.52	249.4763	19588.55	249.496	19585.69
250.7882	19583.02	249.992	19585.58	250.2496	19580.42	249.7221	19585.88	249.7431	19591.07
251.0397	19591.12	250.2398	19583.94	250.4956	19588.29	249.9768	19588.24	249.9958	19587.51
251.5295	19576.18	250.4951	19584.42	250.7453	19580.1	250.2233	19586.75	250.246	19586.1
251.7786	19581.9	250.7376	19585.03	250.9942	19584.91	250.4804	19585.01	250.4951	19584.07
252.0279	19576.76	250.9912	19585.13	251.238	19577.84	250.7363	19590.94	250.7451	19585.22
252.2842	19579.14	251.2406	19577.31	251.4892	19585.78	250.9874	19585.13	250.9925	19593.25
252.5259	19578.28	251.5003	19579.29	251.7374	19582.39	251.2347	19592.26	251.2434	19589.29
252.7805	19584.06	251.7508	19579.32	251.9857	19585.5	251.4893	19594.17	251.4932	19577.46
253.0276	19576.45	252.0022	19576.01	252.2415	19589.61	251.7361	19585.43	251.7458	19586.37
253.2819	19575.61	252.2541	19579.35	252.4863	19585.6	251.988	19594.8	251.9924	19587.28
253.5314	19584.29	252.5087	19585.85	252.7383	19590.91	252.2358	19585.82	252.2469	19593.34
253.7797	19581.86	252.994	19584.47	252.9863	19578.85	252.4988	19584.53	252.4942	19585.14
254.0299	19584.91	253.2465	19583.48	253.2424	19576.55	252.9856	19585.16	252.7464	19584.44
254.277	19581.69	253.5046	19585.7	253.4855	19578.19	253.2337	19580.5	252.9956	19579.98
254.5303	19581.37	253.7552	19584.71	253.7389	19580.9	253.4885	19585.37	253.245	19586.48
254.7817	19583.3	254.0095	19588.48	253.9873	19591.87	253.737	19588.94	253.4929	19583.56
255.0291	19581.42	254.2655	19583.14	254.2422	19584.41	253.991	19592.08	253.7477	19582.17
255.2807	19579.4	254.7519	19585	254.4804	19585.45	254.2408	19587.11	253.9953	19577.95
255.5308	19581.87	255.0101	19586.64	254.7386	19581.02	254.4964	19583.34	254.2463	19586.68
255.7811	19578.23	255.2635	19583.84	254.9867	19583.93	254.7448	19584.65	254.4975	19588.42
256.0293	19579.24	255.5197	19588.74	255.2431	19584.21	254.9975	19584.83	254.7401	19587.98
256.2828	19576.76	256.0079	19580.43	255.4826	19587.43	255.2469	19588.97	254.9939	19588.61
256.5296	19576.4	256.2615	19577.12	255.7382	19594.19	255.5033	19585.13	255.2468	19584.55
256.7847	19583.59	256.5204	19577.67	255.9875	19587.93	255.9916	19589.45	255.5002	19585.76
257.0289	19580.78	256.772	19584.37	256.2454	19593.51	256.2421	19585.26	255.7465	19585.42
257.2814	19577.93	257.0277	19585.58	256.4863	19589.46	256.4996	19583.32	255.9949	19589.78
257.5351	19578.43	257.5319	19587.78	256.7413	19583.86	256.7526	19580.13	256.242	19586.98
257.78	19581.01	258.0226	19581	256.9895	19582.4	257.0097	19588.94	256.4969	19586.39
258.0297	19580.22	258.2768	19581.96	257.2475	19584.5	257.5041	19586	256.7455	19587.55
258.2801	19582.59	258.5345	19582.17	257.4885	19585.61	257.7566	19576.39	256.9943	19589.56
258.5337	19586.3	258.7893	19582.46	257.7403	19586.09	258.0155	19586.09	257.2462	19586.39
258.7818	19580.89	259.2768	19585.36	257.9882	19582.5	258.5077	19587.13	257.4975	19583.41
259.0333	19574.64	259.5338	19584.98	258.2453	19587.07	258.7621	19577.8	257.7481	19588.87
259.2791	19590.13	259.7875	19581.77	258.4888	19587.01	259.0353	19591.79	257.9929	19588.43
259.5366	19581.12	260.0433	19574.06	258.7401	19584.11	259.5279	19581	258.2472	19580.64
259.7811	19586.23	260.5339	19585.74	258.9885	19584.16	259.7823	19591.19	258.495	19592.96
260.0304	19577.21	260.7928	19585.88	259.2503	19582.72	260.0388	19593.51	258.7469	19585.96
260.2808	19581.8	261.0481	19589.9	259.4979	19585.45	260.5304	19573.59	258.997	19592.2
260.535	19577.15	261.5578	19584.86	259.7462	19594.63	260.7887	19580	259.2431	19586.34
260.7811	19579.97	262.0472	19588.98	259.9915	19575.81	261.0451	19584.55	259.4957	19583.09
261.0313	19579.23	262.3038	19584.13	260.2429	19586.07	261.5385	19579.39	259.7487	19584.2
261.282	19579.06	262.5719	19572.87	260.4844	19583.15	261.7914	19584.97	259.9942	19586.08
261.5309	19581.15	263.0619	19581.27	260.7424	19584.14	262.05	19584.28	260.248	19583.06
261.7844	19578.89	263.3187	19580.69	260.9849	19588.95	262.5433	19585.7	260.4927	19587.99
262.0325	19579.43	263.5794	19588.23	261.2444	19587.81	262.7996	19582.09	260.7485	19584.34
262.2778	19582.36	264.0689	19579.29	261.4868	19586.63	263.0598	19590.81	260.9944	19585.68
262.557	19585.76	264.3294	19585.36	261.7424	19587.57	263.5557	19590.05	261.2498	19587.47
263.0489	19579.96	264.8209	19584.67	261.9868	19588.38	263.8112	19588.05	261.4955	19587.22
263.3095	19581.9	265.0785	19573.15	262.2452	19581.21	264.0747	19585	261.7479	19587.68
263.5744	19589.28	265.3408	19581.08	262.4885	19590.69	264.5659	19579.51	261.9953	19587.64
264.068	19581.38	265.8294	19580.9	262.7465	19580.74	264.8219	19591.33	262.2458	19583.04

264.3337	19576.92	266.0887	19582.51	263.0021	19581.08	265.0813	19591.99	262.498	19582.37
264.8233	19568.54	266.3509	19587.96	263.2638	19586.37	265.5733	19590.17	262.7452	19584.2
265.0844	19574.8	266.8439	19574.58	263.7551	19583.12	265.8298	19580.46	263.004	19584.09
265.344	19590.49	267.103	19575.09	264.0136	19587.45	266.0873	19581.28	263.266	19585.73
265.8353	19580.56	267.3687	19582.39	264.2774	19587.47	266.5817	19592.33	263.7592	19584.26
266.0996	19580.05	267.8614	19579.99	264.7699	19591.16	266.8451	19585.45	264.0184	19587.47
266.5954	19578.7	268.1208	19580.35	265.028	19586.26	267.336	19588.1	264.2784	19583.23
266.8576	19578.39	268.3891	19588.66	265.29	19580.75	267.5964	19586.15	264.7729	19593.48
267.3482	19579.05	268.8817	19574.86	265.7825	19586.22	267.8573	19584.79	265.0311	19592.68
267.6127	19582.99	269.1413	19581.16	266.0412	19581.1	268.3482	19591.23	265.2895	19587.4
268.1073	19583.04	269.4076	19581.52	266.3042	19584.24	268.6072	19579.9	265.785	19580.41
268.3713	19580.68	269.8997	19586.09	266.7992	19580.58	268.8688	19581.91	266.0466	19589.37
268.8635	19580.07	270.1608	19585.41	267.0576	19587.67	269.3615	19592.97	266.5389	19581.03
269.1297	19576.63	270.6522	19582.87	267.552	19583.28	269.6359	19586.06	266.8003	19585.6
269.6224	19579.09	270.9153	19582.95	267.8127	19589.26	270.1296	19579.8	267.2952	19583.41
269.8832	19586.76	271.4125	19583.57	268.3089	19583.73	270.3935	19584.4	267.5547	19583.11
270.3802	19585.81	271.6731	19584.54	268.5684	19582.35	270.8864	19590.3	268.0471	19583.26
270.645	19581.87	272.1639	19586.09	269.061	19587.19	271.1477	19591.87	268.3091	19592.52
271.1402	19577.58	272.4303	19579.69	269.3272	19589.04	271.6403	19587.64	268.8027	19585.31
271.4065	19581.72	272.9255	19571.67	269.8227	19583.04	271.9009	19578.7	269.064	19581.87
271.899	19584.62	273.1873	19580.44	270.0818	19582.7	272.3958	19583.31	269.5591	19591.61
272.162	19583.43	273.6801	19582.58	270.5728	19584.36	272.661	19585.4	269.8188	19583.28
272.6576	19587.03	273.9427	19575.4	270.8343	19587.81	273.1539	19576.32	270.3138	19583.9
272.9186	19583.41	274.4402	19584.4	271.0938	19587.98	273.4179	19580.66	270.5779	19584.91
273.4179	19581.45	274.706	19584.44	271.5871	19592.61	273.937	19584.47	271.0729	19594.14
273.6815	19588.69	275.2028	19584.74	271.8504	19590.6	274.1987	19581.69	271.3362	19585
274.18	19584.96	275.471	19581.11	272.3476	19585.15	274.6954	19587.96	271.832	19588.58
274.4454	19580.45	275.9662	19588.28	272.6075	19580.74	274.9577	19585.42	272.0933	19589.32
274.9386	19583.67	276.2296	19578.03	273.102	19578.57	275.4547	19580.74	272.5895	19587.38
275.2074	19579.35	276.7215	19582.08	273.368	19594.85	275.7153	19580.7	272.8512	19586.53
275.7034	19579.28	276.9925	19576.35	273.8635	19580.45	276.2198	19582.71	273.3472	19589.86
275.9654	19578.66	277.4924	19591.16	274.1292	19583.15	276.4876	19581.09	273.6114	19590.86
276.4625	19577.11	277.7561	19585.33	274.6239	19584.46	276.9826	19584.52	274.1111	19583.1
276.7306	19579.91	278.2501	19581.35	274.8873	19588.39	277.2444	19587.06	274.3743	19590.93
277.2286	19580.81	278.5212	19590.15	275.3832	19586.88	277.7386	19579.19	274.8698	19580.74
277.5117	19586.42	279.0201	19589.08	275.6503	19577.32	278.0036	19586.51	275.1338	19587.02
278.0057	19579.86	279.2855	19581.29	276.1492	19581.24	278.5034	19579.08	275.6323	19584.1
278.274	19583.66	279.7793	19579.77	276.4122	19579.56	278.7661	19587.5	275.8977	19583.04
278.7719	19582.09	280.0482	19583	276.9095	19585.49	279.2606	19584.3	276.3959	19583.12
279.0385	19578.12	280.546	19580.8	277.179	19593.03	279.5297	19580.49	276.6603	19584.84
279.5374	19580.1	280.8124	19585.29	277.6755	19587.47	280.0267	19588.73	277.1561	19585.14
279.8074	19582.4	281.309	19583.67	277.9435	19590.44	280.2878	19584.38	277.4187	19581.8
280.3029	19579.3	281.5948	19579.15	278.4506	19578.74	280.7862	19586.43	277.9141	19581.84
280.5725	19574.37	282.0918	19576.5	278.9478	19583.3	281.0521	19577.46	278.1792	19583.42
281.0694	19575.8	282.3628	19577.07	279.2232	19581.53	281.551	19588.14	278.6772	19583.22
281.3344	19587.67	282.8623	19577.9	279.7211	19590.91	281.8162	19583.84	278.9419	19584.05
281.8312	19583.01	283.1305	19579.79	279.9888	19580.09	282.3166	19585.28	279.4381	19588.04
282.1004	19578.79	283.6278	19573.26	280.484	19582.72	282.5859	19589.34	279.7056	19583.2
282.6017	19580.93	283.8964	19579.06	280.7504	19590.31	283.0824	19578.74	280.2032	19591.34
282.873	19579.83	284.3997	19581.64	281.2527	19576.04	283.3494	19588.56	280.4702	19580.83
283.3706	19582.19	284.8979	19582.25	281.7545	19586.53	283.8488	19592.36	280.969	19582.68
283.6507	19578.97	285.167	19580.22	282.0203	19585.03	284.1166	19584.9	281.2357	19586.81
284.1525	19580.64	285.6634	19583.85	282.5201	19587.17	284.616	19577.91	281.7371	19588.02
284.424	19589.17	285.9308	19576.72	282.7888	19580.97	284.8825	19596.65	282.0038	19585
284.921	19577.81	286.4409	19581.48	283.2938	19576.25	285.3825	19590.12	282.5006	19585.42
285.191	19582.07	286.9405	19584.27	283.7974	19581.02	285.6527	19579.12	282.7705	19583.59
285.6904	19579.97	287.2074	19577.96	284.3043	19580.01	286.1513	19588.74	283.2726	19592.64
285.959	19581.9	287.7063	19574.17	284.8058	19590.21	286.419	19582.29	283.556	19589.25
286.462	19576.01	287.977	19581.91	285.0767	19580.61	286.9233	19587.1	284.0548	19591.88
286.7364	19581.93	288.4808	19580.4	285.5742	19574.85	287.4231	19591.84	284.3226	19582.74
287.2376	19587.29	288.9861	19577.45	285.8451	19582.27	287.6796	19588.07	284.8273	19588.47
287.5134	19581.48	289.494	19586.18	286.3484	19590.75	288.1775	19588.31	285.0953	19589.22
288.0122	19582.79	289.9952	19578.86	286.8486	19579.52	288.4575	19583.57	285.5951	19585.08
288.284	19582.62	290.2502	19584.15	287.1176	19582.79	288.959	19581.79	285.8635	19594.59
288.7841	19574.24	290.7518	19576.76	287.6207	19580.41	289.2125	19584.56	286.3627	19584.66
289.0551	19583.27	291.0097	19579.72	288.1224	19577.55	289.7108	19581.37	286.632	19585.06
289.5574	19576.7	291.5197	19576.53	288.3954	19579.24	289.9712	19583.16	287.1325	19591.26
289.83	19576.55	292.0197	19581.64	288.9014	19579.95	290.4748	19584.63	287.4008	19583.26
290.3309	19579.37	292.2766	19585.58	289.4072	19583.61	290.9756	19590.14	287.9023	19588.22
290.6144	19582.82	292.7753	19583.33	289.9073	19583.62	291.2324	19586.33	288.1734	19593.35

291.1153	19579.36	293.0331	19578.49	290.1837	19586.57	291.73	19582.52	288.6777	19587.2
291.392	19580.31	293.537	19585.64	290.6882	19583.89	291.9885	19583.44	289.1803	19587.57
291.8913	19582.95	294.0373	19581.61	290.9563	19584.76	292.4937	19583	289.6871	19590.59
292.1647	19577.31	294.2955	19570.59	291.4574	19592.2	292.9967	19589.55	290.1888	19588.32
292.6674	19589.26	294.7974	19590.82	291.7283	19580.15	293.2539	19588.4	290.4598	19580.88
292.9401	19584.74	295.3047	19582.97	292.233	19583.26	293.7554	19591.24	290.9627	19592.57
293.4427	19575.24	295.8053	19582.49	292.7342	19583.63	294.017	19582.85	291.2356	19592.3
293.7158	19582.34	296.0647	19585.28	293.0048	19583.13	294.5268	19587.81	291.7384	19576.23
294.2198	19578.98	296.5704	19577.84	293.5067	19578.08	295.0297	19587.57	292.242	19585.88
294.7224	19575.5	297.0728	19582.68	293.7788	19581.61	295.2872	19580.22	292.7459	19586.32
294.9954	19581.34	297.3318	19591.09	294.2862	19581.72	295.7877	19581.98	293.2486	19590.86
295.5019	19583.2	297.8359	19577.72	294.7921	19588.75	296.0466	19579.73	293.5196	19580.91
296.0041	19574.77	298.3409	19578.81	295.0643	19580.87	296.5533	19581.36	294.0231	19586.59
296.2806	19576.32	298.8419	19586.86	295.5661	19588.25	297.0549	19584.9	294.5305	19589.19
296.7826	19575.14	299.1011	19576.01	295.8404	19570.02	297.3141	19590.14	295.0382	19588.12
297.0577	19586.47	299.6061	19584.73	296.3463	19578.47	297.8158	19581.08	295.5394	19583.29
297.5668	19577.11	300.1135	19584.52	296.8497	19585.48	298.0753	19582.75	295.8134	19584.66
298.0687	19586.51			297.3549	19586.47	298.5815	19580.4	296.3162	19579.74
298.3436	19586.87			297.8573	19583.13	299.0859	19584.56	296.5896	19584.79
298.8464	19576.84			298.13	19586.95	299.5912	19572.27	297.0924	19587.89
299.123	19578.4			298.6335	19583.05	300.0939	19592.03	297.3656	19589.06
299.6299	19575.17			298.9085	19586.37			297.869	19590.13
300.1341	19579.83			299.4107	19577.84			298.3718	19595.93
				299.6878	19583.66			298.6606	19589.26
				300.1978	19576.2			299.1681	19585.73
								299.6775	19584.3
								300.183	19589.33

0.54												
30												
x_1		x_2		x_3		x_4		x_5		x_1		x
t (s)	P (cm^{-1})	t (s)	P (cm^{-1})	t (s)	P (cm^{-1})	t (s)	P (cm^{-1})	t (s)	P (cm^{-1})	t (s)	P (cm^{-1})	t (s)
0	19609.83	0	19609.65	0	19609.71	0	19608.01	0	19610.83	0	19610.2	0
0.65847	19609.45	0.66183	19610.8	0.66442	19608.6	0.65717	19607.96	0.66235	19607.35	0.68244	19609.83	0.76503
0.85506	19609.72	0.85676	19606.68	0.85362	19607.41	0.848	19607.44	0.85458	19614.53	0.84906	19613.54	1.13014
1.10179	19608.24	1.10352	19607.57	1.10226	19608.42	1.09908	19610.93	1.1041	19610.02	1.09725	19615.02	1.36861
1.35136	19610.35	1.3549	19611.1	1.35092	19611.68	1.34596	19610.77	1.35094	19608.6	1.35035	19611.63	1.62215
1.60149	19606.97	1.60159	19608.96	1.60182	19607.2	1.59612	19611.09	1.602	19606.81	1.59693	19607.72	1.8779
1.85189	19610.55	1.85545	19611.35	1.85173	19613.7	1.84662	19608.01	1.85218	19609.71	1.84729	19610.77	2.12442
2.10149	19607.04	2.10202	19606.7	2.10146	19612.41	2.09674	19609.48	2.1019	19612.15	2.09555	19614.25	2.37696
2.35142	19608.54	2.35534	19607.99	2.35057	19611.18	2.34722	19609.02	2.351	19612.37	2.34684	19612.98	2.62364
2.60074	19606.93	2.60307	19612.4	2.601	19611.41	2.59576	19612.57	2.60143	19605.79	2.59576	19609.7	2.87481
2.85305	19605.94	2.8549	19603.18	2.85162	19606.91	2.84612	19611.04	2.85184	19611.74	2.84712	19607.03	3.13157
3.10124	19608.99	3.10216	19607.83	3.10166	19607.43	3.09572	19608.77	3.10247	19608.97	3.09749	19609.12	3.37603
3.35206	19607.71	3.35577	19605.02	3.35168	19606.64	3.34695	19610.06	3.35211	19610.5	3.34633	19610.95	3.62718
3.60175	19604.25	3.60242	19611	3.60685	19609.03	3.59657	19607.26	3.60199	19610	3.5954	19608.34	3.87483
3.85414	19611.64	3.85496	19609.5	3.85057	19610.57	3.84531	19611.15	3.85062	19608.17	3.84569	19609.45	4.12712
4.10079	19606.41	4.10192	19606.93	4.10067	19606.47	4.09612	19606.43	4.10188	19611.01	4.09693	19607.88	4.37135
4.35244	19610.4	4.35712	19608.52	4.35169	19609.88	4.34599	19609.64	4.35213	19610.41	4.34777	19612.07	4.62322
4.60288	19608.8	4.60281	19605.71	4.60352	19607.91	4.59781	19610.12	4.60297	19609.2	4.59594	19609.26	4.87322
4.85375	19609.23	4.85757	19605.73	4.85292	19607.88	4.84754	19604.95	4.85213	19605.86	4.84589	19611.56	5.12688
5.10144	19608.56	5.10758	19611.79	5.10217	19607.52	5.10426	19611.49	5.10562	19610.48	5.09671	19614.57	5.38026
5.35168	19611.39	5.35831	19610.2	5.35564	19606.47	5.3537	19603.1	5.35276	19609.04	5.34732	19614.04	5.63725
5.60666	19612.71	5.60301	19608.22	5.61727	19611.44	5.59722	19608.49	5.60187	19613.7	5.59793	19607.04	5.8768
5.8594	19606.86	5.85643	19609.98	5.85379	19607.6	5.84635	19608.41	5.85309	19608.32	5.8474	19610.26	6.13508
6.10266	19608.18	6.10338	19605.45	6.10295	19609.69	6.09751	19609.87	6.10344	19604.76	6.09702	19610.37	6.37791
6.35202	19610.23	6.35809	19607.61	6.35289	19609.02	6.3536	19609.36	6.35232	19610.22	6.34873	19613.27	6.61999
6.60201	19609.93	6.60259	19607.33	6.6042	19609.1	6.59844	19612.94	6.60297	19608	6.59853	19614.09	6.86738
6.85478	19606.86	6.85773	19609.76	6.85336	19613.61	6.84741	19611.69	6.85343	19608.96	6.84776	19608.98	7.1248
7.10409	19610.93	7.10432	19608.44	7.10504	19610.37	7.09688	19608.64	7.10413	19609.72	7.09625	19612.78	7.36929
7.35226	19611.02	7.35735	19609.14	7.3529	19611.31	7.34668	19607.53	7.35288	19606.42	7.34654	19608.03	7.62991
7.60161	19609.55	7.60303	19607.09	7.60285	19608.95	7.59947	19607.62	7.60279	19609.59	7.5978	19612.69	7.87761
7.85443	19610.43	7.85667	19607.77	7.85236	19607.29	7.84778	19604.39	7.85216	19610.02	7.84788	19615.11	8.1267
8.10253	19606.37	8.10386	19611.51	8.10422	19607.06	8.09701	19612.17	8.1047	19611.48	8.09822	19610.14	8.37246
8.35385	19611.44	8.35819	19605.68	8.354	19610.64	8.34847	19612.63	8.35393	19609.82	8.34774	19609.23	8.62152
8.60305	19607.11	8.60347	19608.97	8.61109	19608.33	8.59827	19608	8.6031	19607.3	8.5975	19609.75	8.87295
8.85462	19609.41	8.8562	19609.51	8.85332	19610.67	8.84805	19610.48	8.85265	19609.32	8.8478	19611.64	9.12554
9.10258	19606.93	9.10298	19612.41	9.10393	19616.15	9.09731	19609.47	9.10409	19612.3	9.09874	19610.91	9.37374
9.35286	19611.02	9.35977	19608.23	9.35234	19606.86	9.34743	19610.09	9.35422	19607.65	9.34775	19614.32	9.62231
9.603	19604.72	9.60602	19611.74	9.60425	19610.56	9.59777	19610.43	9.60352	19611.19	9.59886	19608.93	9.87118
9.85517	19607.56	9.85734	19609.21	9.85354	19608.55	9.84861	19607.24	9.85293	19604.79	9.84746	19612.25	10.11943
10.10295	19607.39	10.10326	19604.37	10.10295	19609.4	10.0986	19609.38	10.1054	19612.47	10.09847	19611.88	10.37324
10.35397	19608.98	10.35832	19606.85	10.35408	19608.36	10.34878	19611.52	10.35471	19611.09	10.34967	19609.83	10.62499
10.60522	19609.5	10.60438	19608.21	10.60546	19606.6	10.59894	19608.39	10.60502	19610.21	10.60038	19610.09	10.87269
10.85688	19607.27	10.85882	19608.64	10.85489	19608.54	10.84859	19610.39	10.85375	19610.78	10.8482	19612.24	11.12963
11.10379	19608.14	11.10578	19611.02	11.1075	19607.55	11.09991	19608.53	11.10629	19612.11	11.098	19609.52	11.37466
11.35368	19610.39	11.3615	19606.58	11.35497	19607.79	11.35587	19604.33	11.35509	19608.55	11.35114	19611.16	11.62492
11.60433	19607.91	11.60457	19604.89	11.6059	19611.34	11.59903	19606.71	11.6055	19606.69	11.60192	19609.77	11.87292
11.8572	19606.9	11.85795	19609.4	11.85383	19609.03	11.8487	19606.59	11.85464	19608.31	11.84933	19611.95	12.12838
12.10482	19607.53	12.10545	19609.15	12.10597	19610.28	12.09946	19607.35	12.10509	19605.04	12.09905	19610.71	12.37395
12.35469	19605.79	12.35897	19607.39	12.35621	19610.68	12.3505	19606.18	12.35447	19608.7	12.35109	19603.53	12.62546
12.60327	19608.49	12.60512	19609.45	12.6052	19609.45	12.60396	19609.41	12.60473	19608.79	12.60126	19609.73	12.8751
12.85554	19606.77	12.85748	19606.35	12.85347	19608.78	12.84838	19608.56	12.85418	19606.84	12.85104	19611.2	13.12525
13.10558	19605.9	13.10486	19608.5	13.10417	19608.06	13.09856	19611.1	13.10438	19610.01	13.09864	19609.36	13.37567
13.35553	19604.89	13.36002	19608.08	13.35492	19610.88	13.34898	19604.94	13.35443	19606.38	13.34842	19611.7	13.62368
13.60395	19608.75	13.60475	19606.68	13.60688	19609.09	13.60035	19609.21	13.60503	19610.23	13.59984	19611.32	13.87036
13.85573	19611.4	13.85893	19604.59	13.85413	19609.78	13.84989	19607.75	13.85502	19607.02	13.85104	19614.27	14.12427
14.10399	19609.17	14.10507	19610.73	14.10441	19611.68	14.09868	19609.42	14.10577	19613.33	14.10081	19606.27	14.37625
14.35568	19609.94	14.35989	19604.96	14.35527	19608.51	14.34875	19604.67	14.35449	19609.6	14.34902	19609.49	14.62532
14.60522	19610.23	14.60581	19607.02	14.60724	19609.34	14.60038	19607	14.60494	19608.3	14.60027	19608.75	14.87468
14.85614	19606.51	14.85966	19609.87	14.85447	19608.46	14.85019	19609.16	14.85602	19609.37	14.85375	19608.38	15.12626
15.10391	19606.71	15.10486	19609.61	15.10539	19609.98	15.09938	19609.62	15.10755	19609.2	15.1019	19615.58	15.37529
15.35548	19606.05	15.36155	19605.39	15.35527	19611.09	15.34976	19609.04	15.35739	19609.8	15.35048	19608.28	15.62732
15.60669	19611.23	15.60665	19607.41	15.60829	19608.24	15.6007	19608.91	15.60555	19609.06	15.59954	19609.09	15.87518
15.85891	19609.1	15.86018	19608.59	15.85642	19604.02	15.85112	19609.4	15.85576	19607.45	15.8501	19616.54	16.12745
16.10578	19608.23	16.10733	19610.97	16.10601	19608.31	16.10132	19606.64	16.10757	19612.29	16.10345	19613.55	16.37507

16.35588	19607.55	16.36122	19608.01	16.35548	19608.36	16.3506	19609.2	16.35721	19607.06	16.3519	19611.93	16.62651
16.60495	19609.13	16.60555	19608.37	16.60698	19609.4	16.59959	19606.71	16.60609	19611.11	16.59984	19609.92	16.8736
16.85655	19606.5	16.85913	19605.4	16.85576	19609.49	16.85044	19609.87	16.85502	19610.15	16.85126	19612.14	17.13053
17.10636	19603.21	17.10704	19606.11	17.10755	19610.67	17.10121	19609.05	17.10745	19609.65	17.09996	19612.05	17.37589
17.35578	19605.09	17.36305	19606.51	17.35502	19611.18	17.35747	19607.2	17.35626	19607.58	17.35112	19609.74	17.62682
17.60505	19610.12	17.60747	19611.38	17.60772	19610.79	17.60068	19608.15	17.6067	19606.07	17.6013	19615.29	17.87552
17.85741	19609.42	17.85966	19609.19	17.85559	19613.1	17.84972	19607.12	17.85608	19611.85	17.85044	19613.42	18.12717
18.10603	19608.86	18.10703	19610.45	18.10818	19610.63	18.1	19611.07	18.10812	19609.96	18.10028	19609.87	18.37726
18.35707	19608.19	18.36195	19608.06	18.35627	19608.67	18.35114	19608.25	18.35621	19608.21	18.35068	19608.6	18.63206
18.60646	19606.72	18.60678	19608.26	18.60694	19606.79	18.60142	19607.57	18.6071	19611.51	18.6012	19610.85	18.87607
18.85761	19607.96	18.86086	19608.68	18.85601	19610.28	18.85051	19603.55	18.85603	19608.33	18.85096	19606.63	19.12782
19.10661	19608.78	19.10653	19604.93	19.10804	19609.57	19.10061	19606.28	19.10898	19610.63	19.09999	19608.04	19.37647
19.35689	19608.31	19.3626	19606.84	19.35669	19611.05	19.35045	19611.19	19.35561	19610.51	19.35044	19613.09	19.6293
19.60679	19608.41	19.60787	19610.19	19.61097	19604.73	19.60148	19612.57	19.60666	19610.01	19.60114	19613.08	19.87514
19.87679	19608.94	19.86006	19610.73	19.85612	19607.34	19.85273	19608.42	19.85734	19609.35	19.85251	19611.86	20.12888
20.10662	19607.18	20.10602	19605.23	20.10724	19608.89	20.10178	19612.28	20.10935	19605.28	20.10141	19610.57	20.37729
20.35759	19610.82	20.3608	19608.47	20.35696	19604.34	20.35193	19607.89	20.35712	19609.7	20.3518	19609.31	20.63048
20.60862	19607.32	20.60768	19609.51	20.6098	19608.75	20.60237	19607.55	20.60688	19608.38	20.60142	19614.08	20.87687
20.85943	19610.44	20.86203	19610.03	20.85818	19608.52	20.85264	19609.05	20.85827	19609.44	20.85267	19608.72	21.13
21.10747	19604.29	21.10839	19607.96	21.10829	19607.27	21.10366	19609.41	21.11006	19607.81	21.10288	19614.17	21.37682
21.35738	19609.44	21.36244	19609.67	21.35677	19610.01	21.3539	19612.55	21.35782	19606.95	21.35527	19611.19	21.62936
21.60841	19607.73	21.60757	19613.48	21.60998	19610.3	21.60224	19609.86	21.60752	19609.63	21.60121	19609.27	21.87818
21.86103	19606.22	21.86243	19607.29	21.85825	19608.98	21.85196	19608.27	21.85726	19612.09	21.85148	19611.51	22.13075
22.10757	19609.08	22.10881	19607.5	22.11054	19605.5	22.10274	19610.53	22.11125	19606.55	22.10305	19610.62	22.37701
22.35753	19611.77	22.365	19609.25	22.35745	19614.06	22.35363	19613.24	22.35866	19607.87	22.3539	19608.1	22.62914
22.60846	19611.35	22.61082	19608.26	22.60927	19608.05	22.60346	19609.7	22.60856	19610.71	22.60313	19610.25	22.87961
22.86113	19607.62	22.86129	19608.3	22.85786	19612.19	22.85476	19612.21	22.85829	19606.3	22.85193	19612.09	23.1331
23.10909	19611.17	23.10877	19607.91	23.11162	19607.56	23.10285	19609.66	23.11002	19611.13	23.10298	19608.7	23.3763
23.35793	19611.25	23.36428	19610.2	23.35906	19607.13	23.35424	19610.79	23.35829	19611.65	23.35327	19607.18	23.6314
23.60806	19606.6	23.609	19611.11	23.61425	19608.87	23.6043	19606.36	23.60918	19607.71	23.60353	19609.14	23.87702
23.86743	19609.63	23.86194	19612.65	23.85976	19608.79	23.85285	19609.77	23.85947	19607.71	23.85342	19604.91	24.12775
24.11148	19608.91	24.109	19612.95	24.1097	19604.33	24.10312	19613.3	24.10993	19614.69	24.10343	19608.27	24.37805
24.36046	19606.75	24.36564	19609.14	24.3584	19611.12	24.35329	19609.66	24.35753	19608.94	24.35344	19609.14	24.62863
24.60839	19607.33	24.60858	19609.7	24.61019	19607.8	24.60423	19607.78	24.60787	19611.37	24.60409	19609.64	24.87847
24.85965	19609.79	24.86284	19609.77	24.85921	19610.47	24.85387	19614.77	24.85964	19612.83	24.85438	19610.84	25.13013
25.1085	19610.21	25.10799	19607.36	25.11054	19608.98	25.10249	19612.77	25.11203	19609.55	25.10453	19611.65	25.37991
25.35865	19608.47	25.36522	19605.7	25.3582	19608.81	25.35259	19612	25.35839	19605.07	25.35243	19607.04	25.62987
25.60887	19606.85	25.61038	19612.42	25.60991	19606.42	25.60344	19607	25.60784	19608.2	25.60376	19611.77	25.87779
25.86241	19607.96	25.86483	19606.33	25.85744	19613.43	25.85382	19611.45	25.85955	19612.54	25.85413	19612.05	26.13255
26.10853	19611.53	26.10858	19610.74	26.11141	19608.97	26.10492	19608.45	26.11244	19611.45	26.10402	19613.63	26.38205
26.35778	19607.38	26.36388	19612.04	26.35902	19608.27	26.35478	19607.68	26.35967	19610.39	26.35453	19610.22	26.63003
26.60839	19609.56	26.60938	19608.08	26.61989	19608.77	26.60312	19610.59	26.61008	19607.85	26.60284	19611.21	26.87749
26.86267	19611.45	26.86442	19611.9	26.8581	19608.46	26.85405	19608.41	26.85891	19612.31	26.85353	19610.37	27.1348
27.11074	19609.12	27.11143	19609.83	27.10887	19606.33	27.1052	19611.43	27.1111	19609.15	27.10474	19610.04	27.37691
27.35785	19613.63	27.36304	19610.21	27.3583	19612.53	27.36012	19605.98	27.35908	19607.82	27.35448	19606.13	27.63046
27.60847	19609.65	27.61008	19609.41	27.6129	19607.31	27.60426	19607.26	27.61023	19610.56	27.60321	19612.95	27.8806
27.8625	19603.92	27.86326	19610.45	27.85932	19607.64	27.85427	19610.76	27.86028	19610.3	27.85264	19611.24	28.13428
28.11026	19605.73	28.11122	19609.15	28.11155	19609.47	28.10358	19608.36	28.11189	19611.61	28.10415	19614.08	28.37967
28.35977	19609.32	28.36421	19608.29	28.35859	19610.58	28.35481	19610	28.359	19609.95	28.35503	19615.22	28.63268
28.60794	19610.27	28.60937	19602.02	28.61027	19607.86	28.6052	19610.59	28.60957	19604.76	28.60508	19613.78	28.8794
28.86283	19609.63	28.86345	19609.64	28.8592	19608.63	28.85612	19608.54	28.86141	19603.83	28.85286	19615.19	29.13136
29.1102	19607.37	29.11034	19608.15	29.11381	19609.83	29.10324	19609.48	29.11226	19609.14	29.10327	19611.13	29.38072
29.36018	19609.36	29.36585	19608.18	29.35947	19607.78	29.35339	19608.15	29.35882	19609.92	29.35381	19614.99	29.63204
29.61018	19606.01	29.61052	19607.74	29.61245	19604.72	29.60441	19610.93	29.6097	19609.79	29.60528	19611.99	29.87832
29.86305	19605.77	29.86286	19610.66	29.85905	19609.56	29.85614	19612.54	29.86164	19607.21	29.8553	19608.96	30.13072
30.1122	19611.23	30.11164	19609.7	30.1144	19611.2	30.10692	19608.57	30.11438	19612.9	30.10494	19610.86	30.3794
30.36085	19605.43	30.36693	19606.7	30.36174	19607.14	30.3551	19608.34	30.36033	19608.88	30.35854	19609.8	30.6334
30.61194	19607.63	30.61258	19607.62	30.61253	19612.66	30.60563	19606.54	30.61041	19611.3	30.60762	19611.49	30.88043
30.86542	19608.92	30.86628	19612.82	30.86009	19609.08	30.85681	19610.86	30.86208	19609.23	30.85686	19613.81	31.14057
31.1107	19607.11	31.11202	19608.36	31.11216	19611.5	31.10676	19608.77	31.11494	19613.5	31.10704	19612.14	31.38074
31.36042	19610.46	31.36458	19608.35	31.36126	19610.05	31.35649	19610.78	31.36199	19614.31	31.35823	19612.13	31.63253
31.61141	19608.11	31.61189	19606.19	31.61394	19607.36	31.60628	19605.67	31.61055	19607.15	31.60602	19614.25	31.8805
31.86545	19606.44	31.86648	19610.9	31.86189	19604.37	31.8563	19611.88	31.8614	19608.48	31.85668	19609.43	32.13675
32.11207	19608.58	32.11236	19607.04	32.11443	19609.12	32.10665	19609.5	32.11576	19607.34	32.1076	19614.36	32.39494
32.36095	19609.02	32.3678	19609.47	32.36061	19609.04	32.35757	19612.32	32.36261	19611.29	32.35944	19613.89	32.63266
32.61035	19605.59	32.61263	19612.01	32.61329	19613.26	32.60766	19607.49	32.61068	19607.79	32.60445	19612.37	32.88097
32.86428	19608.85	32.86826	19610.17	32.86132	19611.17	32.85554	19610.02	32.88566	19611.11	32.85535	19608.56	33.13595
33.11249	19611.66	33.11268	19609.73	33.11033	19613.05	33.10529	19611.08	33.11467	19612.28	33.10556	19608.85	33.3821
33.36147	19608.27	33.36775	19610.44	33.36016	19611.96	33.35605	19614.18	33.36352	19608.5	33.35827	19612.22	33.63479
33.61039	19610.32	33.6116	19609.35	33.61372	19607.09	33.6071	19611.41	33.6122	19608.22	33.60467	19615.25	33.88082

33.86485	19607.57	33.86454	19608.45	33.86209	19608.84	33.85658	19607.79	33.86064	19611.68	33.85515	19609.96	34.13939
34.11142	19607.55	34.11183	19608.88	34.11408	19610.19	34.10533	19608.42	34.11405	19608.18	34.10609	19609.48	34.38013
34.36262	19608.29	34.36759	19610.62	34.36102	19612.31	34.35558	19609.77	34.36191	19607.07	34.35766	19611.19	34.6339
34.60995	19609.17	34.61257	19608.53	34.61247	19604.8	34.60736	19611.94	34.6125	19609.05	34.60569	19615.6	34.88194
34.86395	19602.54	34.86688	19606.19	34.86103	19607.09	34.8569	19611.41	34.8626	19610.45	34.85723	19614.66	35.13674
35.11102	19611.84	35.11139	19609.29	35.11337	19612.21	35.10588	19608.71	35.11541	19615.06	35.10848	19607.67	35.38132
35.36267	19606.98	35.36837	19608.68	35.36248	19605.97	35.35547	19607.55	35.36113	19610.36	35.35719	19613.16	35.63697
35.61256	19611.77	35.61303	19603.94	35.61332	19609.82	35.60603	19611.52	35.61189	19610.06	35.60743	19610.9	35.88276
35.86476	19608.27	35.86694	19611.26	35.86156	19609.44	35.85764	19608.93	35.86318	19606.1	35.8592	19614.46	36.13643
36.11074	19610.55	36.11249	19607.47	36.11325	19606.86	36.1073	19605.33	36.11698	19610.94	36.10584	19616.07	36.3794
36.36084	19610.33	36.36708	19608.44	36.36396	19607.73	36.35631	19609.39	36.36127	19607.36	36.35678	19613.2	36.63523
36.61548	19605.36	36.61276	19611.35	36.61642	19613.35	36.60632	19608.6	36.61176	19609.05	36.60723	19610.04	36.88123
36.86628	19605.93	36.86672	19604.8	36.86148	19610.37	36.85692	19607.89	36.86294	19610.14	36.85914	19611.18	37.13931
37.11252	19607.03	37.11362	19606.59	37.11346	19613.95	37.10817	19607.72	37.11649	19608.19	37.10732	19609.56	37.38433
37.36192	19607.4	37.37522	19608.6	37.36349	19608.69	37.35787	19608.21	37.36309	19606.46	37.35659	19609.04	37.63467
37.6116	19609.91	37.61421	19609.34	37.61627	19612.04	37.60603	19606.73	37.61203	19607.97	37.60669	19607.98	37.88208
37.86772	19607.99	37.86588	19609.51	37.86116	19610.35	37.85712	19608.61	37.86205	19610.3	37.85874	19614.77	38.13579
38.11309	19607.7	38.11333	19611.99	38.11314	19609.7	38.10729	19607.38	38.11671	19604.75	38.10857	19610.17	38.3846
38.36287	19609.34	38.3697	19609.64	38.36199	19607.15	38.35842	19610.27	38.36374	19606.52	38.3582	19609.71	38.63386
38.61236	19610.37	38.61267	19608.23	38.61739	19607.73	38.60861	19605.66	38.61348	19609.85	38.60712	19611.07	38.88163
38.86583	19606.89	38.86584	19612.06	38.86375	19609.27	38.85733	19610.8	38.86268	19610.23	38.8576	19609.93	39.13705
39.11313	19602.89	39.11368	19609.03	39.11463	19610.53	39.1069	19608.6	39.11569	19611.99	39.10871	19612.33	39.38353
39.36401	19608.57	39.36956	19611.91	39.36249	19610.82	39.35724	19610.53	39.36299	19608.08	39.35859	19612.99	39.63309
39.6147	19609.88	39.61433	19608.13	39.61679	19608.11	39.60806	19607.19	39.61435	19608.67	39.60912	19611.12	39.8842
39.86747	19604.14	39.86564	19608.02	39.86393	19611.25	39.85994	19607.31	39.86469	19608.81	39.85758	19611.25	40.13823
40.11244	19611.16	40.11273	19610.03	40.1162	19609.96	40.10851	19605.65	40.1175	19608.61	40.1075	19608.29	40.38327
40.3654	19610.49	40.38863	19609.9	40.36459	19605.64	40.3583	19609.02	40.36477	19607	40.36019	19608.03	40.63725
40.61588	19608.82	40.616	19608.37	40.61814	19608.2	40.60915	19608.24	40.61529	19609.74	40.61167	19618.34	40.88556
40.86976	19605.08	40.86979	19608.06	40.86486	19608.57	40.86007	19607.07	40.86646	19607.99	40.85928	19611.76	41.13944
41.11391	19608.7	41.11485	19615.52	41.11901	19607.25	41.11121	19606.46	41.12112	19611.66	41.10886	19613.07	41.38573
41.36356	19606.31	41.36832	19607	41.36557	19614.45	41.35992	19611.74	41.36438	19604.96	41.35994	19609.95	41.63735
41.61625	19607.4	41.61517	19609.51	41.62849	19610.18	41.60895	19606.79	41.61451	19608.57	41.61198	19614.03	41.88534
41.87158	19602.91	41.86947	19608.17	41.86477	19609.57	41.85867	19610.11	41.86563	19610.25	41.86165	19611.77	42.14759
42.11516	19609.63	42.11589	19610.48	42.11765	19608.47	42.11107	19607.62	42.12043	19612.82	42.11546	19610.15	42.39074
42.3644	19606.25	42.37238	19608.63	42.36607	19613.02	42.36058	19608.67	42.36624	19608.06	42.35892	19609.64	42.63694
42.61547	19610.65	42.61699	19607.34	42.61891	19606.04	42.6098	19608.43	42.61456	19610.39	42.6088	19610.61	42.88646
42.86869	19607.98	42.8704	19609.21	42.86588	19609.1	42.85902	19608.33	42.86484	19608.03	42.86001	19610.17	43.14285
43.11618	19606.95	43.11694	19608.56	43.11782	19608.84	43.10962	19607.88	43.12096	19608.57	43.11066	19608.61	43.38747
43.3646	19603.22	43.37059	19610.32	43.36546	19609.86	43.36135	19608.67	43.36693	19606.88	43.3603	19611.63	43.63697
43.6149	19605.38	43.61505	19611.68	43.62059	19608.68	43.61114	19611.55	43.61609	19607.87	43.60939	19609.7	43.88649
43.87444	19612.78	43.86928	19608.57	43.86668	19608.28	43.86028	19610.29	43.86486	19607.97	43.86004	19611.32	44.14248
44.11634	19610.92	44.11658	19610.49	44.11929	19605.73	44.11018	19608.51	44.11998	19610.15	44.11153	19612.8	44.38878
44.36613	19607.99	44.37268	19608.14	44.36562	19608.33	44.36099	19607.42	44.36718	19611.66	44.36069	19609.19	44.63892
44.61491	19608.95	44.61631	19611.56	44.61856	19607.78	44.61157	19609.68	44.61707	19605.85	44.60929	19611.12	44.88614
44.87016	19604.65	44.86941	19606.39	44.86606	19608.62	44.86236	19608.3	44.86671	19608.65	44.86002	19609.03	45.14123
45.11552	19607.72	45.11668	19605.17	45.11872	19608.38	45.11067	19611.88	45.1197	19609.37	45.11072	19611.38	45.38632
45.36725	19607.7	45.37415	19609.63	45.36728	19610.77	45.36052	19612.26	45.36621	19613.06	45.36197	19610.78	45.64172
45.61688	19609.41	45.61729	19610.76	45.61967	19614.49	45.61083	19609.21	45.61759	19606.89	45.61172	19608.84	45.88856
45.87113	19610.17	45.87041	19607.44	45.86523	19608.32	45.86211	19611.32	45.86786	19607.8	45.86	19611.16	46.14312
46.11575	19610.64	46.11586	19608.52	46.11957	19608.37	46.11212	19610.74	46.12449	19606.87	46.11047	19611.85	46.3914
46.36672	19611.59	46.3709	19609.18	46.36864	19609.71	46.36176	19610.18	46.36773	19607.45	46.36309	19612.32	46.63985
46.61757	19609.62	46.61848	19609.15	46.61941	19609.72	46.61066	19609.99	46.61543	19608.13	46.61182	19613.27	46.89291
46.87741	19609.54	46.87195	19607.91	46.86601	19608.49	46.86108	19612.27	46.86771	19611.97	46.86304	19613.55	47.14696
47.11714	19609.51	47.11607	19607.72	47.1219	19607.48	47.11231	19606	47.12587	19611.6	47.10978	19614.49	47.38841
47.36612	19608.01	47.37026	19611.01	47.36856	19605.1	47.36302	19612.74	47.36704	19605.77	47.36039	19614	47.64592
47.61691	19608.52	47.61955	19608.51	47.61959	19611.97	47.61275	19605.63	47.61753	19610.08	47.61099	19618.36	47.8892
47.87158	19608.31	47.87066	19610.02	47.86644	19610.78	47.86103	19608.29	47.86714	19611.02	47.86406	19608.25	48.14146
48.1174	19606.59	48.11911	19607.34	48.11962	19611.56	48.11199	19608.65	48.12465	19612	48.11325	19614.27	48.38809
48.36773	19607.18	48.37642	19605.83	48.36959	19608.41	48.36236	19609.26	48.3686	19610.49	48.36085	19612.79	48.63938
48.61487	19608.89	48.61633	19608.98	48.61924	19609.51	48.61192	19611.68	48.6154	19611.79	48.61053	19613.54	48.88548
48.87004	19607.84	48.87071	19607.6	48.86784	19610.49	48.86224	19607.47	48.86656	19611.25	48.86289	19610.03	49.14109
49.11784	19611.5	49.11751	19610.86	49.11962	19611.53	49.1123	19608.54	49.12201	19608.02	49.11171	19612.1	49.38729
49.36811	19610.47	49.37183	19610.41	49.36713	19609.8	49.35999	19607.58	49.36843	19609.08	49.36217	19614.16	49.64032
49.6164	19609.79	49.61686	19610.73	49.62025	19611.28	49.61269	19612.25	49.61722	19609.25	49.61176	19611.77	49.88888
49.89155	19606.37	49.8702	19611.95	49.86757	19612.1	49.86248	19605.78	49.86567	19616.16	49.86086	19610.53	50.14153
50.11733	19607.82	50.11711	19612.21	50.1183	19610.88	50.11306	19607.8	50.12149	19607.67	50.1122	19609.43	50.3885
50.368	19610.79	50.37588	19607.19	50.3674	19611.96	50.36039	19609.11	50.36805	19608.15	50.36595	19607.94	50.64476
50.6168	19608.32	50.6187	19608.99	50.62045	19613.61	50.61057	19605.3	50.6174	19607.97	50.61199	19612.77	50.88884
50.87081	19609.34	50.87211	19608.65	50.86692	19606.32	50.86286	19609.91	50.86703	19608.83	50.86118	19610.44	51.1445
51.11652	19608.61	51.11725	19608.93	51.12156	19609.16	51.11367	19611.02	51.12085	19606.76	51.11185	19615.46	51.39138

51.36894	19605.32	51.37201	19609.48	51.36797	19605.72	51.36231	19612.74	51.36737	19607.33	51.36441	19614.13	51.63889
51.61785	19607.4	51.61886	19606.6	51.62947	19611.04	51.61198	19605.25	51.61843	19607.23	51.61214	19611.98	51.8905
51.87324	19607.52	51.87223	19605.07	51.86674	19609.81	51.86193	19607.28	51.86823	19607.72	51.86126	19614.88	52.14528
52.11649	19608.78	52.11697	19606.71	52.12018	19613.1	52.11396	19610.47	52.12331	19613.67	52.1118	19605.8	52.39044
52.36749	19606.26	52.37126	19609.36	52.36888	19608.9	52.36518	19606.49	52.36741	19608.51	52.36368	19606.81	52.64664
52.61793	19603.93	52.62115	19608.75	52.6207	19611.37	52.61267	19609.6	52.61708	19606.13	52.61333	19612.04	52.8899
52.87374	19606.16	52.873	19610.6	52.86686	19607.08	52.86182	19609.5	52.86811	19609.07	52.86189	19606.8	53.14306
53.1184	19609.57	53.1189	19605.85	53.11924	19607.8	53.11336	19612.32	53.1272	19608.56	53.11203	19613.88	53.39081
53.3684	19603.64	53.37368	19607.93	53.36884	19611.67	53.36277	19609.35	53.36665	19606.59	53.36322	19612.56	53.64279
53.6177	19613.48	53.61766	19610.89	53.62437	19608.35	53.61721	19608.45	53.61663	19609.31	53.61371	19613.09	53.88972
53.87415	19608.52	53.87048	19609.02	53.86854	19610.84	53.86435	19611.63	53.86761	19609.37	53.86294	19611.25	54.14353
54.1185	19607.21	54.1199	19607.17	54.12199	19608.69	54.11384	19607.93	54.12431	19611.7	54.11942	19609.81	54.39339
54.36899	19609.46	54.37441	19607.24	54.36722	19607.84	54.37242	19609.78	54.37018	19609.12	54.36256	19610.69	54.64218
54.61733	19603.98	54.6184	19610.27	54.62262	19607.85	54.61297	19611.2	54.61738	19611.67	54.61273	19617.83	54.8913
54.87334	19609.42	54.87226	19608.45	54.86887	19608.51	54.86421	19608.79	54.86792	19605.77	54.86418	19614.96	55.14272
55.11914	19607.3	55.11848	19609.1	55.11976	19613.06	55.11467	19609.45	55.12577	19607.47	55.11305	19611.53	55.39292
55.36887	19606.51	55.37568	19609.4	55.36746	19608.66	55.36425	19611.04	55.3699	19612.51	55.36281	19607.86	55.64312
55.61817	19607.32	55.61869	19609.32	55.62248	19609.91	55.61459	19611.72	55.61913	19609.25	55.61287	19609.19	55.89213
55.87408	19609.81	55.87108	19613.57	55.86887	19608.55	55.86343	19609.45	55.8677	19606.52	55.86478	19607.51	56.14477
56.11925	19608.42	56.11914	19608.91	56.12406	19610.99	56.11493	19608.34	56.12434	19606.46	56.11955	19608.6	56.39044
56.36886	19607.48	56.37571	19608.14	56.36888	19611.15	56.36445	19606.31	56.37087	19603.08	56.36495	19609.78	56.64622
56.61795	19607.16	56.62082	19605.79	56.62215	19611.09	56.61386	19609.86	56.62053	19606.31	56.61305	19611.37	56.88826
56.87533	19610.2	56.87363	19610.18	56.86746	19613.57	56.86717	19614.51	56.86897	19607.2	56.86378	19609.07	57.14702
57.11985	19604.07	57.11902	19608.78	57.12215	19609.62	57.11417	19608.6	57.12382	19608.02	57.11603	19616.72	57.39196
57.378	19604.2	57.37416	19609.42	57.36966	19609.13	57.36494	19608.75	57.37073	19608.94	57.36387	19608.77	57.64193
57.61781	19609.46	57.62173	19607.01	57.62418	19608.07	57.61449	19609.87	57.62081	19603.91	57.61266	19609.21	57.89015
57.87557	19606.08	57.87431	19610.97	57.86778	19609.9	57.86354	19611.01	57.87015	19605.47	57.86398	19610.89	58.15209
58.11982	19606.96	58.11877	19612.34	58.12324	19608.44	58.11468	19608.1	58.12543	19609.92	58.11618	19611.05	58.39191
58.37025	19606.94	58.37509	19608.38	58.37025	19608.87	58.36588	19608.91	58.36969	19609.36	58.36459	19612.17	58.64416
58.62048	19606.87	58.61947	19611.06	58.62536	19610.49	58.61483	19606.88	58.61947	19610.57	58.6134	19616.58	58.88954
58.87772	19605.71	58.87427	19609.72	58.86942	19611.41	58.86393	19610.8	58.87278	19609.24	58.86524	19616.11	59.14759
59.11932	19605.57	59.12123	19605.98	59.12247	19610.4	59.11365	19606.91	59.12629	19608.65	59.1153	19612.84	59.39318
59.37038	19613.66	59.37652	19610.43	59.36987	19611.36	59.3644	19607.96	59.37118	19611.84	59.36535	19611.82	59.6445
59.62072	19610.05	59.62019	19607.99	59.62538	19608.28	59.6144	19610.24	59.61943	19607.3	59.61492	19610.75	59.88991
59.87718	19607.83	59.87685	19608.78	59.87323	19607.29	59.86791	19610.89	59.87377	19608.12	59.86998	19611.72	60.15021
60.11975	19608.98	60.1226	19611.25	60.12186	19608.89	60.11556	19608.17	60.12906	19607.4	60.1173	19607.92	60.39313
60.37217	19609.14	60.38037	19608.43	60.37101	19608.38	60.3656	19611.72	60.37418	19608.52	60.36838	19615.18	60.64809
60.62131	19606.55	60.62121	19608.1	60.62783	19609.8	60.61552	19610.33	60.62205	19608.32	60.61882	19606.45	60.89444
60.8789	19609.28	60.87623	19610.35	60.8726	19607.57	60.86687	19607.27	60.87094	19608.53	60.86694	19611.89	61.14954
61.12277	19607.12	61.12305	19605.7	61.12506	19607.06	61.11788	19610.5	61.12807	19608.31	61.11622	19615.09	61.39591
61.3714	19606.94	61.37837	19611.1	61.3708	19606.8	61.3674	19608.85	61.37323	19611.72	61.36774	19607.9	61.64737
61.62163	19606.98	61.62143	19609.09	61.64622	19607.81	61.61674	19613.64	61.6233	19610.05	61.61675	19609.95	61.89639
61.87829	19608.01	61.87649	19608.87	61.87227	19612.8	61.86625	19607.92	61.87561	19611.44	61.86822	19611.95	62.14995
62.12274	19607.57	62.12235	19609.55	62.12764	19611.77	62.11808	19609.34	62.12787	19608.15	62.11734	19610.98	62.39525
62.37236	19609.04	62.37844	19609.18	62.37333	19609.62	62.36827	19609.36	62.3734	19610.39	62.36768	19611.88	62.65619
62.62157	19607.63	62.62784	19604.56	62.62621	19610.46	62.61793	19605.97	62.62359	19611.87	62.616	19612.47	62.8988
62.87843	19609.77	62.87668	19607.64	62.87147	19609.93	62.8664	19607.11	62.8771	19610.32	62.86858	19606.65	63.16121
63.1229	19610.65	63.12292	19605.61	63.12943	19606.76	63.11655	19611	63.12949	19610.08	63.11879	19612.25	63.39696
63.37275	19606.35	63.37929	19610.77	63.37376	19609.61	63.36866	19608.1	63.37325	19605.43	63.37019	19611.24	63.65149
63.62328	19611.69	63.62452	19608.13	63.62713	19609.29	63.61881	19608.03	63.62271	19610.72	63.61602	19611.18	63.89595
63.87989	19608.81	63.87963	19608.89	63.87277	19607.53	63.86774	19606.93	63.87684	19609.85	63.86719	19612.97	64.15183
64.12284	19608.45	64.12324	19612.08	64.12558	19611.84	64.11711	19607.12	64.1305	19609.65	64.1179	19609.36	64.39531
64.37271	19610.77	64.37891	19607.77	64.3726	19610.51	64.36872	19609.72	64.37241	19608.51	64.37119	19610.89	64.64638
64.62338	19605.48	64.62298	19610.01	64.62642	19609.38	64.61731	19608.93	64.62142	19610.76	64.61702	19602.89	64.89206
64.88058	19608.82	64.8765	19607.75	64.87355	19607.42	64.86783	19608.44	64.87306	19609.2	64.8653	19610.1	65.15529
65.12184	19608.37	65.12364	19607.63	65.12275	19612.96	65.11844	19606.96	65.13413	19609.92	65.11637	19608.78	65.39774
65.37036	19607.36	65.37777	19609.71	65.37048	19604.28	65.38267	19609.56	65.38297	19610.06	65.3677	19609.91	65.64953
65.62188	19609.03	65.62334	19607.25	65.62614	19613.71	65.61568	19610.96	65.62185	19608.15	65.61724	19613.62	65.89488
65.87794	19609.63	65.87745	19607.48	65.88684	19609.43	65.86625	19610.19	65.87433	19609.55	65.86709	19607.03	66.15214
66.1233	19611.55	66.12432	19609.18	66.12842	19610.52	66.11779	19608.98	66.12854	19609.2	66.11579	19613.43	66.39601
66.37214	19610.3	66.37875	19611.48	66.37188	19611.85	66.36835	19611.42	66.37616	19611	66.36858	19610.97	66.64862
66.62181	19610.85	66.62275	19609.52	66.62814	19610.97	66.61684	19607.56	66.62341	19609.28	66.61768	19612.19	66.89516
66.87807	19611.53	66.87696	19604.68	66.8717	19608.66	66.8671	19609.43	66.87346	19606.61	66.86833	19612.03	67.15472
67.12393	19607.94	67.12356	19607.67	67.12751	19609.38	67.11867	19606.18	67.14782	19608.85	67.11791	19617.06	67.39518
67.37339	19609.98	67.37904	19609.85	67.37341	19608.73	67.36875	19612.83	67.37484	19609	67.36657	19612.49	67.64664
67.62353	19607.15	67.63484	19608.99	67.62884	19607.16	67.61729	19608.1	67.62409	19608.62	67.61741	19611.92	67.89633
67.87847	19611.66	67.87573	19610.05	67.87147	19606.98	67.86671	19610.23	67.87511	19609.79	67.86922	19610.78	68.14881
68.12236	19609.32	68.12378	19611.65	68.12905	19606.88	68.11812	19608.09	68.12904	19611.92	68.11946	19614.46	68.39622
68.37407	19608.86	68.37871	19613.15	68.374	19611.55	68.37049	19608.41	68.37703	19607.9	68.36693	19610.28	68.64803
68.62493	19605.19	68.62444	19610.19	68.62895	19608.72	68.61798	19610.77	68.62281	19610.6	68.61706	19614.58	68.89645

68.87928	19607.9	68.87918	19609.58	68.87214	19610.7	68.86774	19605.07	68.87614	19609.67	68.86867	19610.39	69.15335
69.1227	19610.07	69.12369	19610.62	69.12758	19610.24	69.11774	19609.27	69.13643	19607.13	69.11924	19613.42	69.39569
69.37373	19606.2	69.37862	19610.73	69.3743	19609.06	69.36905	19608.54	69.38379	19607.44	69.36869	19613.86	69.64681
69.62566	19607.95	69.62402	19609.87	69.6317	19608.03	69.61996	19608.36	69.62346	19607.61	69.61786	19610.24	69.89475
69.88069	19605.47	69.87777	19613.21	69.87455	19607.07	69.86881	19608.13	69.87755	19608.9	69.86704	19612.38	70.15319
70.12343	19608.19	70.1241	19607.78	70.12738	19611.11	70.11804	19609.64	70.12902	19618.27	70.11771	19611.71	70.39497
70.37213	19605.8	70.38782	19605.48	70.37364	19607.61	70.36937	19612.99	70.37771	19604.87	70.36969	19610.95	70.64918
70.62544	19609.7	70.62295	19609.12	70.63265	19609.16	70.61926	19609.38	70.62525	19606.56	70.62015	19610.58	70.89558
70.88081	19607.49	70.87943	19610.98	70.87514	19615.98	70.87017	19607.49	70.87408	19608.91	70.86825	19608.81	71.15238
71.12452	19610.3	71.12541	19609.98	71.13021	19606.33	71.11806	19607.44	71.12858	19609.67	71.11757	19610.76	71.39582
71.37316	19607.83	71.38088	19611.66	71.37357	19608.74	71.38047	19608	71.37604	19612.98	71.37177	19609.67	71.64814
71.62533	19608.76	71.62362	19610.09	71.64022	19607.57	71.61863	19609.44	71.62499	19609.1	71.62117	19610.47	71.8963
71.88121	19606.84	71.87893	19612.62	71.87421	19610.19	71.87076	19607.29	71.87542	19610.45	71.87033	19610.28	72.15404
72.12524	19606.24	72.1257	19611.79	72.12903	19608.75	72.12014	19609.98	72.12925	19609.96	72.11768	19607.55	72.39611
72.37422	19610.46	72.38101	19605.77	72.37522	19608.88	72.37026	19606.46	72.37746	19606.51	72.36798	19610.45	72.65033
72.62388	19604.97	72.62751	19610.62	72.62924	19605.58	72.61789	19612.35	72.62537	19608.4	72.61981	19614.93	72.89997
72.88035	19607.1	72.87936	19607.69	72.87425	19607.49	72.86886	19613.27	72.87679	19608.41	72.86995	19615.95	73.15271
73.12544	19607.05	73.12456	19606.46	73.12795	19610.27	73.12006	19606.58	73.13145	19609.88	73.12073	19614.18	73.39925
73.37449	19606.9	73.38137	19611.72	73.37624	19609.26	73.37137	19607.44	73.37805	19609.33	73.36807	19610.72	73.64839
73.62432	19610.49	73.62687	19608.31	73.63157	19608.96	73.61904	19608.1	73.62399	19607.91	73.61883	19611.87	73.89882
73.88213	19605.83	73.88054	19611.4	73.87443	19606.81	73.86851	19609.15	73.87645	19609.51	73.869	19612.06	74.15467
74.12537	19607.48	74.12417	19607.78	74.12963	19609.75	74.11943	19608.59	74.13234	19607.86	74.11983	19607.33	74.39738
74.37741	19608.93	74.38037	19612.11	74.37507	19608.57	74.37331	19608.01	74.3766	19608.19	74.37136	19608.73	74.6444
74.62621	19611.42	74.62648	19606.2	74.63443	19609.52	74.62056	19610.05	74.62416	19608.57	74.61891	19612	74.8987
74.882	19611.63	74.88135	19611.67	74.87629	19611.71	74.86871	19607.96	74.87581	19609.31	74.8753	19611.62	75.15975
75.1238	19610.69	75.12557	19611.83	75.12922	19611.16	75.11923	19610.62	75.13231	19611.29	75.12312	19615.12	75.39273
75.376	19605.24	75.3806	19606.56	75.37449	19609.78	75.3707	19607.51	75.38354	19607.29	75.37118	19607.99	75.65152
75.62723	19612.84	75.62571	19607.74	75.63133	19611.91	75.62156	19609.52	75.62622	19606.38	75.62185	19610.46	75.8999
75.88285	19608.72	75.88129	19612.21	75.87973	19610.04	75.87252	19609.94	75.87689	19606.48	75.87024	19609.35	76.16061
76.12515	19605.66	76.12771	19608.03	76.13639	19611.23	76.11963	19609.71	76.13361	19611.42	76.11987	19608	76.39852
76.37468	19608.61	76.38175	19611.27	76.37556	19613.43	76.37107	19608.93	76.3792	19606.56	76.37356	19615.48	76.6524
76.62493	19607.55	76.62767	19610.04	76.64363	19612.02	76.62042	19607.84	76.62552	19605.89	76.62083	19612.21	76.89948
76.88245	19608.93	76.88116	19605.97	76.87599	19610.15	76.87243	19609.92	76.87737	19609.33	76.87076	19612.5	77.15611
77.12683	19611.68	77.1275	19604.87	77.13114	19609.61	77.1214	19609.31	77.13538	19609.61	77.11863	19610.79	77.3992
77.37651	19607.06	77.38452	19610.13	77.37743	19608.31	77.37356	19606.21	77.37861	19608.42	77.37095	19611.89	77.65263
77.62863	19611.32	77.63073	19605.55	77.63323	19609.92	77.62132	19612.01	77.62836	19613.98	77.61979	19612.93	77.90039
77.88328	19608.5	77.87974	19607.17	77.87541	19608.81	77.87245	19607.24	77.8778	19605.19	77.8727	19606.96	78.15506
78.12691	19607.9	78.12667	19606.73	78.13075	19609.02	78.12174	19608.87	78.13263	19609.03	78.12265	19606.42	78.40001
78.37745	19607.8	78.38201	19609.41	78.377	19611.16	78.37218	19609.52	78.37843	19607.25	78.37108	19612.76	78.65122
78.62742	19611.34	78.62746	19608.65	78.63164	19607.32	78.62089	19607.45	78.62719	19606.98	78.62044	19610.61	78.90006
78.88339	19609.42	78.88084	19606.24	78.87586	19612.9	78.87175	19608.84	78.87993	19608.84	78.8731	19611.9	79.15604
79.12634	19607.41	79.1254	19609.89	79.12984	19614.19	79.12247	19611.7	79.1326	19607.4	79.1225	19606.34	79.40022
79.37717	19603.22	79.39151	19607.73	79.38165	19609.75	79.37378	19609.16	79.37942	19606.87	79.37284	19604.78	79.65323
79.6278	19604.32	79.6284	19605.96	79.63512	19608.85	79.62245	19607.07	79.62677	19602.8	79.62077	19612.25	79.89949
79.90431	19607.68	79.88113	19605.77	79.87677	19607.15	79.87239	19608.71	79.87978	19606.29	79.87181	19611.54	80.15625
80.12609	19606.49	80.12827	19607.03	80.13059	19606.93	80.12222	19607.49	80.1345	19604.37	80.12155	19609.21	80.40225
80.38643	19605.86	80.38563	19613.32	80.37865	19611.98	80.37542	19608.28	80.38294	19607.9	80.37552	19612.98	80.65114
80.62908	19605.28	80.62639	19605.37	80.63599	19612.04	80.62248	19610.56	80.62694	19610.85	80.62254	19610.87	80.90074
80.88591	19608.73	80.88115	19609.13	80.87855	19607.65	80.87162	19608.51	80.88022	19610.26	80.87113	19609.59	81.15642
81.12862	19608.36	81.12863	19607.28	81.13047	19607.84	81.12261	19611.84	81.13422	19610.32	81.12154	19607.85	81.39967
81.37688	19607.82	81.38387	19607.33	81.37689	19611.19	81.37514	19610.77	81.38231	19605.53	81.37405	19609.85	81.6525
81.63338	19611.2	81.62746	19606.44	81.63572	19612.12	81.624	19608.6	81.62673	19607.73	81.62345	19613.02	81.90349
81.89154	19608.88	81.88136	19606.69	81.88033	19609.29	81.87893	19605.12	81.87958	19609.52	81.87332	19609.84	82.15832
82.13212	19604.43	82.13304	19604.25	82.13565	19611.19	82.12135	19604.57	82.13486	19610.37	82.12065	19604.74	82.40262
82.37938	19607.6	82.38565	19610.32	82.37909	19608.79	82.37378	19609.41	82.3826	19608.38	82.37249	19610.44	82.65296
82.62751	19606.96	82.63321	19608.05	82.63438	19609.76	82.62385	19609.28	82.62957	19607.75	82.6235	19611.85	82.90249
82.89084	19603.52	82.88151	19608.4	82.87936	19608.71	82.87509	19612.01	82.87809	19609.46	82.87336	19605.97	83.15844
83.13081	19605.76	83.12817	19606.63	83.13356	19616.77	83.12387	19607.81	83.13576	19611.03	83.12206	19612.37	83.40125
83.39255	19609.63	83.38376	19607.5	83.37837	19606.14	83.37572	19607.83	83.38344	19613.89	83.37238	19608.38	83.65538
83.62845	19607.37	83.62892	19609.49	83.63692	19609.82	83.62313	19606.7	83.63226	19605.43	83.62196	19609.73	83.90109
83.88533	19607.57	83.88401	19607.98	83.87843	19609.18	83.87474	19608.35	83.88056	19606.63	83.87373	19608.98	84.15888
84.1294	19608.09	84.12762	19610.68	84.13416	19607.22	84.12521	19606.93	84.13471	19611.87	84.12428	19613.98	84.40107
84.3793	19609.73	84.3834	19608.08	84.38069	19609.25	84.37577	19610.8	84.38096	19609.08	84.37189	19608.43	84.65414
84.63629	19607.51	84.63005	19606.5	84.63886	19606.01	84.62259	19608.79	84.62944	19609.08	84.62298	19604.46	84.90272
84.88743	19606.58	84.8849	19611.8	84.87933	19611.21	84.87238	19608.4	84.88176	19611.01	84.87408	19611.58	85.1609
85.12767	19608.63	85.13022	19610.98	85.1334	19606.79	85.12452	19607.72	85.13723	19613.33	85.12483	19609.63	85.40401
85.37873	19607.33	85.38508	19607.28	85.38029	19608.08	85.3768	19607.87	85.37957	19610.92	85.37303	19609.71	85.65578
85.6302	19606.8	85.62848	19611.29	85.63386	19612.23	85.6237	19605.44	85.62866	19608.36	85.62222	19608.15	85.90398
85.89515	19607.72	85.8841	19609.98	85.87811	19612	85.87309	19609.54	85.88162	19611.07	85.87381	19613.41	86.16241
86.1305	19607.6	86.13066	19606.45	86.13399	19612.47	86.12367	19607.91	86.13699	19611.56	86.1264	19608.73	86.40397

86.3794	19608.05	86.38693	19606.23	86.38005	19607.28	86.37704	19606.8	86.3841	19607.67	86.37628	19606.75	86.65605
86.63074	19605.46	86.62864	19609.47	86.6391	19607.67	86.62475	19610.76	86.62845	19605.69	86.62313	19611.81	86.9041
86.88664	19606.9	86.88723	19611.97	86.88039	19610.08	86.87407	19607.74	86.88187	19608.99	86.87355	19612.5	87.16251
87.13116	19604.98	87.13204	19605.46	87.13344	19609.49	87.12484	19606.17	87.13608	19608.3	87.12665	19610.62	87.40165
87.38979	19607.57	87.38619	19607.96	87.37972	19613.3	87.38558	19607.79	87.38292	19607.3	87.37525	19606.3	87.65547
87.6293	19607.53	87.63191	19607.02	87.63812	19605.95	87.62774	19611.08	87.63194	19610.97	87.62424	19609.72	87.90644
87.88842	19604.88	87.88372	19612.8	87.88208	19609.07	87.87539	19607.79	87.88028	19611.56	87.87361	19607.06	88.16083
88.13147	19608.59	88.13185	19608.32	88.13378	19610.66	88.12413	19609.1	88.13535	19604.65	88.1262	19606.01	88.40379
88.38073	19605.77	88.38803	19604.35	88.38037	19606.78	88.37724	19605.19	88.38424	19609.13	88.37519	19611.57	88.65635
88.63806	19606.77	88.63207	19611.68	88.63705	19607.23	88.62603	19604.27	88.6313	19610.16	88.6254	19608.12	88.9046
88.88998	19602.87	88.88601	19606.7	88.88195	19608.83	88.8936	19607.7	88.88373	19602.86	88.87611	19604.68	89.16095
89.13137	19609.18	89.12967	19608.06	89.13636	19606.78	89.13583	19610.41	89.13917	19611.74	89.12433	19608.58	89.40446
89.38176	19604.69	89.41163	19609.39	89.38012	19607.89	89.38326	19609.01	89.39255	19611.35	89.37405	19609.31	89.65597
89.63103	19607.12	89.63111	19609.46	89.63966	19605.04	89.62843	19610.64	89.63212	19605.1	89.6255	19603.81	89.90483
89.89296	19606.63	89.88533	19608.03	89.88052	19611.3	89.87632	19605.96	89.88273	19609.08	89.87582	19603.58	90.16149
90.13432	19606.31	90.13346	19607.62	90.13534	19610.1	90.12736	19607.38	90.1371	19610.96	90.12652	19612.77	90.4055
90.38364	19606.6	90.38892	19604.41	90.3817	19611.5	90.37942	19609.72	90.38537	19607.66	90.37531	19606.36	90.65709
90.63147	19607.65	90.62964	19603.53	90.65355	19607.76	90.62604	19609.59	90.63195	19609.44	90.62448	19612.91	90.90503
90.89069	19605.77	90.8854	19607.75	90.88202	19605.26	90.87667	19605.21	90.88438	19612.51	90.87583	19609.73	91.16104
91.13094	19604.69	91.13327	19603.16	91.13505	19605.09	91.12719	19611.2	91.13902	19606.49	91.12711	19605.64	91.40395
91.38253	19605.76	91.38929	19607.55	91.38198	19607.31	91.38287	19606.82	91.3856	19607.68	91.37877	19612.04	91.65844
91.63262	19604.87	91.63128	19604.14	91.64026	19606.37	91.63014	19608.71	91.63391	19610.64	91.62416	19606.69	91.90609
91.88964	19606.99	91.88574	19604.02	91.88199	19608.62	91.87668	19607.48	91.88085	19608.96	91.87596	19607.45	92.16574
92.12991	19605.74	92.1331	19607.91	92.1364	19604.27	92.12898	19609.88	92.13795	19603.6	92.12572	19612.26	92.40519
92.38063	19606.45	92.39012	19605.41	92.38246	19605.86	92.38076	19605.5	92.38639	19608.21	92.37769	19608.7	92.69178
92.63192	19609.78	92.63519	19605.31	92.64194	19604.13	92.62851	19602.67	92.63456	19608.19	92.62702	19606.15	92.90559
92.88937	19610.27	92.90184	19611.63	92.88346	19607.29	92.87643	19610.63	92.88377	19608.51	92.87578	19606.23	93.16468
93.1325	19606.04	93.1386	19605.8	93.1357	19606.35	93.12648	19606.44	93.15934	19605.35	93.12713	19605.5	93.40847
93.38076	19605.13	93.39014	19608.97	93.38165	19602.1	93.3824	19605.94	93.38498	19607.78	93.3781	19609.38	93.65827
93.63245	19606.74	93.63401	19607.02	93.64328	19604.64	93.62886	19609.36	93.63436	19609.59	93.62749	19605.11	93.90734
93.89084	19609.07	93.88792	19607.46	93.88439	19607.11	93.87731	19608.59	93.88568	19606.22	93.87794	19606.25	94.16563
94.13449	19607.38	94.13253	19606.6	94.13668	19610.17	94.12824	19605.24	94.14043	19607.7	94.12647	19610.49	94.40428
94.38484	19602.86	94.3888	19607.09	94.38181	19609.1	94.37976	19606.11	94.38376	19604.63	94.37853	19606.62	94.65674
94.63118	19604.74	94.63318	19605.22	94.64998	19606.52	94.62733	19608.86	94.63338	19605.66	94.62769	19607.25	94.90934
94.89088	19602.79	94.88806	19609.33	94.88439	19608.35	94.8783	19607.78	94.88644	19607.68	94.87876	19600.22	95.1636
95.13601	19607.24	95.13452	19605.81	95.13847	19605.46	95.12906	19608.84	95.13988	19609.8	95.12882	19609.41	95.40768
95.38497	19604.31	95.38882	19608.15	95.38534	19604.08	95.379	19607.12	95.38605	19605.65	95.37642	19605.97	95.66047
95.63406	19602.63	95.63167	19605.22	95.64491	19607.28	95.62951	19606.58	95.63336	19607.51	95.62841	19604.99	95.90857
95.88912	19607.51	95.88775	19606.73	95.88397	19609.24	95.8782	19606.84	95.88403	19609.9	95.87926	19608.04	96.16579
96.13227	19606.34	96.13488	19604.94	96.13947	19604.06	96.12878	19612.43	96.14086	19604.47	96.1298	19608.2	96.40846
96.38442	19605.28	96.39232	19606.14	96.38379	19605.69	96.38356	19603.17	96.40354	19608.46	96.37871	19607.27	96.66044
96.63401	19606.66	96.63343	19607.15	96.64089	19608.61	96.62766	19606.87	96.63301	19609.84	96.62786	19607.87	96.90732
96.88989	19607.47	96.88711	19604.7	96.88142	19605.55	96.8788	19606.29	96.88488	19607.13	96.87676	19607.7	97.16482
97.13234	19607.15	97.13464	19604.37	97.13555	19605.21	97.12999	19608.26	97.13904	19610.65	97.1291	19606.75	97.40937
97.38419	19608.48	97.39048	19609.23	97.38376	19604.83	97.38257	19605.13	97.38883	19605.85	97.37834	19602.82	97.66874
97.63527	19603.47	97.63695	19603.02	97.644	19604.37	97.63197	19609.33	97.63545	19607.82	97.63108	19609.23	97.91023
97.89433	19605.34	97.88655	19608.42	97.88424	19609.22	97.87842	19609.56	97.8833	19606.37	97.87655	19610.02	98.165
98.14393	19612.81	98.13379	19612.17	98.13689	19605.27	98.12753	19606.69	98.1399	19609.24	98.12818	19607.24	98.41257
98.38289	19604.13	98.3907	19607.48	98.38269	19602.47	98.3956	19610.31	98.38868	19608.46	98.37904	19608.35	98.66038
98.63428	19605.55	98.63545	19606.03	98.64381	19605.6	98.63401	19611.52	98.63682	19609.89	98.63094	19603.68	98.91027
98.89122	19608.33	98.89381	19608.53	98.88533	19606.95	98.87947	19608.12	98.88685	19605.63	98.8782	19607	99.16682
99.13442	19608.64	99.13431	19608.06	99.13735	19605.46	99.13237	19605.03	99.13885	19607.34	99.12757	19605.18	99.40354
99.38271	19606.22	99.39382	19606.7	99.38482	19606.75	99.38181	19604.9	99.38738	19607.41	99.37974	19608.95	99.66697
99.6338	19603.79	99.63752	19606.67	99.65222	19603.14	99.62734	19606.1	99.63465	19603.91	99.63041	19607.39	99.90603
99.89211	19602.14	99.89009	19603.74	99.88818	19605.25	99.87928	19605.01	99.8878	19604.62	99.88062	19608.24	100.1688
100.1369	19604.89	100.1352	19606.55	100.141	19609.8	100.1295	19604.41	100.1407	19603.51	100.1278	19605.35	100.4097
100.3875	19602.78	100.3967	19606.5	100.3871	19604	100.3966	19606.06	100.3895	19608.32	100.3793	19605.64	100.6636
100.6356	19601.35	100.6353	19603.92	100.6519	19606.9	100.6308	19603	100.6363	19606.98	100.633	19611.35	100.9103
100.8931	19605.69	100.893	19607.93	100.8883	19608.17	100.8809	19607.96	100.8913	19604.65	100.8831	19602.98	101.1662
101.138	19606.22	101.1366	19605.73	101.1432	19605.31	101.1319	19604.88	101.1442	19603.57	101.1326	19607.93	101.411
101.3874	19605.09	101.3966	19601.11	101.3882	19609.51	101.3866	19605.99	101.3917	19606.64	101.3795	19606.84	101.6652
101.6385	19606.11	101.6374	19608.48	101.6468	19602.16	101.6326	19603.54	101.6368	19603.4	101.6336	19609.95	101.9115
101.8925	19605.92	101.8924	19604.8	101.8876	19606.29	101.8812	19608.88	101.8911	19608.62	101.8829	19609.25	102.1718
102.1353	19607.03	102.1391	19600.55	102.1415	19603.33	102.1318	19603.73	102.1438	19607.08	102.1327	19603.68	102.4108
102.3876	19604.15	102.3961	19606.21	102.3859	19607.14	102.3865	19607.8	102.3919	19604.27	102.3826	19605.59	102.6612
102.6374	19604.17	102.6399	19606.21	102.6487	19605.1	102.6321	19606.29	102.6386	19606.5	102.6347	19603.6	102.9174
102.8949	19604.12	102.8911	19602.35	102.8883	19603.33	102.8841	19606.89	102.8895	19605.92	102.8821	19602.82	103.1696
103.1362	19606.55	103.1376	19605.9	103.1433	19602.53	103.1325	19603.87	103.1438	19605.94	103.1332	19610.42	103.4122
103.3866	19603.47	103.3969	19604.26	103.3861	19603.21	103.3867	19605.97	103.3935	19604.54	103.3829	19603.63	103.6644
103.6404	19604.88	103.6378	19609.21	103.6564	19606.83	103.6338	19605.06	103.6384	19607.87	103.6327	19607.83	103.9112

103.8961	19606.32	103.8928	19606.27	103.8893	19604.95	103.8839	19608.19	103.89	19606.18	103.8814	19605.5	104.1674
104.1394	19606.45	104.1367	19605.75	104.1453	19607.23	104.134	19602.95	104.1451	19605.6	104.1334	19604.15	104.4116
104.3864	19600.28	104.3937	19604.18	104.3897	19604.57	104.3859	19603.49	104.3917	19604.02	104.3826	19603	104.6629
104.6378	19606.48	104.6384	19605.23	104.6483	19601.7	104.6327	19604.64	104.6394	19608.91	104.6333	19605.63	104.9125
104.8952	19604.59	104.8941	19606.48	104.8884	19602.01	104.8852	19602.36	104.8953	19609.12	104.8839	19605.66	105.1709
105.1391	19608.84	105.1392	19604.92	105.1428	19603.9	105.1336	19605.21	105.1448	19606.66	105.1327	19601.85	105.4125
105.3872	19605.36	105.3947	19602.07	105.3907	19602.87	105.3876	19602.64	105.3944	19604.83	105.3824	19605.45	105.6662
105.637	19604.54	105.6411	19607.12	105.6494	19606.53	105.6306	19605.05	105.6376	19606.04	105.6334	19600.68	105.902
105.8959	19608.09	105.8937	19603.37	105.891	19603.88	105.8827	19603.69	105.8938	19605.36	105.8841	19603.57	106.1684
106.141	19601.05	106.1392	19604.27	106.143	19602.46	106.1356	19600.5	106.1469	19607.25	106.1324	19607.13	106.4135
106.3893	19602.42	106.3963	19608.32	106.3893	19603.86	106.3892	19603.97	106.3967	19603.94	106.3848	19608.96	106.6661
106.6379	19602.87	106.6381	19604.21	106.6514	19605.43	106.6329	19607.05	106.6371	19605.82	106.634	19604.56	106.9121
106.8959	19602.79	106.8949	19604.59	106.8894	19603.02	106.8841	19603.21	106.8923	19604.91	106.8834	19607.9	107.1793
107.1397	19608.5	107.1436	19603	107.1438	19604.46	107.1352	19603.06	107.1497	19604.17	107.1341	19597.55	107.4126
107.3896	19605.55	107.4015	19605.16	107.3884	19600.73	107.3886	19599.16	107.3958	19608.36	107.3827	19606.1	107.6648
107.64	19607.03	107.6412	19603.22	107.6495	19604.54	107.6351	19604.56	107.64	19609.08	107.6342	19607.98	107.9122
107.8953	19603.2	107.8938	19601.86	107.8896	19599.41	107.8839	19600.65	107.8926	19603.54	107.8871	19602.93	108.1707
108.139	19603.4	108.141	19602.25	108.1451	19604.48	108.1336	19605.46	108.1455	19604.6	108.1345	19608.34	108.4119
108.389	19599.67	108.3986	19603.97	108.3898	19604.1	108.3903	19605.33	108.3934	19604.31	108.3833	19601.99	108.6732
108.64	19603.04	108.6404	19604.97	108.6522	19603.58	108.6354	19605.81	108.6401	19603.75	108.6339	19604.38	108.9132
108.8958	19602.28	108.8945	19602.75	108.8901	19601.17	108.8856	19608.29	108.8952	19605.55	108.8861	19604.82	109.1717
109.1406	19604.23	109.1394	19599.72	109.1469	19603.41	109.1333	19604.57	109.1469	19605.05	109.1361	19605.61	109.4132
109.389	19607.98	109.4212	19602.85	109.3917	19602.93	109.3886	19602.92	109.3958	19605.44	109.3829	19606.29	109.6677
109.6404	19604.48	109.6406	19602.75	109.6525	19603.82	109.636	19604.7	109.6405	19600.69	109.6332	19603.34	109.9158
109.8975	19605.96	109.8945	19604.99	109.8917	19604.38	109.8866	19601.17	109.8947	19603.06	109.8863	19601.65	110.1783
110.1425	19601.48	110.1399	19602.17	110.1471	19605.41	110.1373	19605.75	110.146	19608.64	110.1376	19603	110.4091
110.3882	19604	110.3968	19601.57	110.3918	19605.65	110.3884	19603.4	110.3952	19600.11	110.3852	19602.24	110.6682
110.6396	19603.54	110.6424	19602.28	110.6524	19597.66	110.6352	19603.2	110.6406	19604.5	110.6338	19600.9	110.9152
110.896	19605.52	110.8965	19602.11	110.8904	19602.58	110.8857	19602.77	110.8969	19604.19	110.8853	19605.91	111.1724
111.1424	19603.58	111.1437	19601.45	111.1472	19603.3	111.1363	19606.07	111.1477	19601.83	111.1375	19606.42	111.4145
111.3944	19603.99	111.4053	19602.19	111.3911	19603.03	111.3918	19601.74	111.3947	19602.13	111.3883	19599.21	111.666
111.6391	19602.57	111.6395	19602.01	111.6508	19606.12	111.6352	19606.43	111.641	19606.77	111.6345	19600.84	111.9148
111.8974	19603.95	111.8968	19603.33	111.891	19608.62	111.8862	19599.27	111.8966	19601.36	111.8846	19605.06	112.1754
112.1432	19599.91	112.1429	19603.33	112.1465	19604.3	112.1368	19603.3	112.1484	19606.58	112.1373	19603.33	112.4151
112.3926	19605.46	112.4032	19596.43	112.3916	19603.13	112.3919	19599.95	112.4096	19608.23	112.3854	19602.31	112.6684
112.6399	19601.82	112.6445	19603.33	112.6553	19602.75	112.6381	19601.1	112.6401	19604.55	112.6368	19604.5	112.9183
112.8977	19601.47	112.8971	19600.14	112.8921	19601.3	112.8891	19606.75	112.8972	19602.26	112.8858	19600.78	113.1747
113.1425	19600.39	113.1428	19603.77	113.1472	19602.29	113.136	19600.69	113.1503	19608.66	113.1363	19603.02	113.4171
113.3926	19605.65	113.4052	19607.06	113.3904	19603.37	113.3944	19603.71	113.3969	19601.57	113.3862	19605.9	113.6675
113.642	19605.2	113.6412	19604.21	113.6549	19604.41	113.637	19605.95	113.6415	19604.64	113.6361	19606.04	113.9171
113.8971	19600.93	113.8957	19600.92	113.8931	19603.87	113.8892	19603.97	113.8966	19603.69	113.8879	19602.41	114.1726
114.142	19603.18	114.1408	19603	114.1481	19604.05	114.136	19606.63	114.1478	19600.13	114.137	19603.55	114.4165
114.3927	19603.97	114.4012	19602.39	114.3906	19603.01	114.394	19601.43	114.3953	19606.44	114.3841	19600.96	114.6687
114.6423	19600.14	114.6442	19599.3	114.654	19605.89	114.6378	19602.44	114.6436	19602.79	114.6359	19600.83	114.9176
114.8962	19600.68	114.8968	19604.7	114.893	19605.68	114.8892	19605.04	114.8967	19604.34	114.8869	19607.57	115.1743
115.1424	19602.48	115.1409	19599.38	115.1477	19603.29	115.1374	19601.58	115.147	19600.16	115.1408	19602.8	115.4153
115.391	19598.27	115.4033	19601.97	115.3938	19604.31	115.3905	19600.16	115.3984	19603.92	115.3856	19604.01	115.667
115.6421	19606.29	115.6423	19600.34	115.6554	19601.85	115.6383	19600.33	115.6418	19599.25	115.6379	19603.22	115.9177
115.898	19597.18	115.899	19601.83	115.8933	19606.76	115.8898	19600.18	115.8975	19606.99	115.888	19605.27	116.1752
116.1425	19606.39	116.1413	19600.39	116.1489	19601.12	116.1396	19605.08	116.149	19602.76	116.1422	19603.17	116.4186
116.3914	19602.5	116.4026	19600.81	116.3935	19604.94	116.3933	19603.31	116.3993	19608.11	116.3892	19601.35	116.6704
116.644	19605.33	116.6427	19599.42	116.6564	19603.57	116.6389	19604.12	116.642	19607.01	116.6363	19602.66	116.9181
116.8996	19603.22	116.8981	19600.69	116.8926	19605.4	116.8908	19601.82	116.8995	19598.18	116.9065	19601.74	117.1783
117.1448	19600.1	117.1446	19602.25	117.148	19604.14	117.1387	19603.56	117.1495	19604.85	117.1452	19596.22	117.4132
117.3937	19606.4	117.4049	19601.82	117.3934	19601.75	117.396	19599.23	117.4001	19604.71	117.3892	19604.48	117.6701
117.6539	19602.39	117.6455	19599.21	117.6557	19598.14	117.6383	19606.57	117.6437	19601.52	117.6378	19593.73	117.918
117.8986	19599.58	117.8971	19601.31	117.8925	19603.9	117.889	19600.17	117.8984	19604.48	117.8873	19596.3	118.1743
118.1465	19600.2	118.145	19603.99	118.1504	19602.54	118.1396	19598.9	118.1497	19601.37	118.1409	19599.29	118.4202
118.3954	19600.11	118.4064	19600.68	118.3974	19601.42	118.3945	19601.03	118.4003	19602.59	118.3889	19604.74	118.6715
118.6436	19600.43	118.6429	19602.54	118.6571	19603.21	118.6406	19602.11	118.6453	19598.91	118.6394	19604.71	118.921
118.9008	19602.83	118.8976	19599.38	118.8956	19599.64	118.8898	19605.45	118.8995	19603.87	118.8909	19600.31	119.1779
119.1477	19601.36	119.1449	19601.4	119.149	19604.2	119.1383	19601.17	119.1494	19598.9	119.1374	19604.27	119.4193
119.395	19601.16	119.4074	19601.16	119.3964	19600.99	119.3945	19604.22	119.4002	19606.69	119.3885	19600.51	119.6699
119.6483	19601.29	119.6505	19598.8	119.6617	19606.53	119.6448	19604.97	119.6494	19603.77	119.6433	19599	119.9207
119.8982	19604.6	119.8971	19601.93	119.8965	19599.46	119.8947	19603.41	119.8948	19601.85	119.8916	19597.45	120.1748
120.1444	19599.16	120.1455	19602.07	120.1502	19600.22	120.1395	19601.29	120.1491	19599.25	120.1403	19604.38	120.4181
120.3951	19598.67	120.4039	19599.7	120.3942	19598.01	120.3928	19600.86	120.4	19601.37	120.3871	19600.41	120.666
120.6425	19601.49	120.6428	19596.72	120.6755	19599.82	120.6372	19603.03	120.6433	19603.42	120.6367	19600.15	120.915
120.8962	19602.9	120.8968	19601.83	120.8932	19599.57	120.888	19600.32	120.8972	19603.7	120.8878	19597.85	121.177
121.1432	19600.64	121.1429	19600.48	121.1484	19600.91	121.139	19601.18	121.147	19601.87	121.1417	19600.05	121.4169

121.391	19601.71	121.4033	19598.51	121.3921	19597.84	121.3927	19604.85	121.3969	19603.4	121.3897	19598.24	121.6716
121.6429	19601.04	121.6442	19601.11	121.6546	19601.69	121.6378	19604.75	121.6436	19600.68	121.6359	19598.07	121.9147
121.8986	19601.56	121.8991	19599.6	121.8937	19603.21	121.8865	19603.14	121.8978	19599.93	121.888	19597.88	122.1734
122.1439	19600.06	122.1409	19600.48	122.1484	19601.99	122.138	19601.76	122.147	19603.11	122.1395	19601.64	122.4124
122.3919	19598.53	122.4028	19598.65	122.3935	19603.15	122.3954	19601.41	122.3964	19601.75	122.39	19603.73	122.6642
122.6421	19597.92	122.6469	19604.42	122.6553	19597.71	122.6373	19602	122.6426	19606.09	122.6367	19598.43	122.9132
122.9013	19598.86	122.8988	19600.95	122.8922	19600.45	122.8877	19601.76	122.8975	19599.88	122.888	19602.15	123.1776
123.1436	19598.63	123.1419	19600.71	123.1501	19602.98	123.1384	19600.09	123.1483	19600.83	123.1393	19600.64	123.4139
123.3921	19602.38	123.4054	19600.3	123.3946	19601.51	123.393	19604.15	123.3993	19603.44	123.3908	19604.43	123.6654
123.6435	19596.87	123.6441	19602.97	123.6554	19600.14	123.6389	19599.61	123.6425	19597.96	123.6391	19594.62	123.9133
123.9004	19596.96	123.8996	19602.75	123.894	19600.78	123.8877	19604.44	123.8977	19603.78	123.8883	19597.6	124.1729
124.1446	19601.96	124.1419	19602.95	124.1495	19603.78	124.14	19599.43	124.1483	19597.52	124.1379	19595.95	124.4142
124.3952	19601.75	124.4038	19603.02	124.3937	19603.02	124.393	19600.06	124.3976	19604.04	124.391	19599.02	124.6672
124.6451	19600.4	124.6437	19601.41	124.6578	19600.95	124.639	19604.14	124.6447	19603.5	124.6399	19597.77	124.9139
124.8969	19600.25	124.9	19601.25	124.8966	19600.76	124.8902	19599.92	124.8983	19602.5	124.8902	19597.95	125.1772
125.1452	19599.05	125.1457	19603.7	125.1491	19604.63	125.1395	19597.51	125.1505	19600.47	125.1389	19596.7	125.4171
125.3948	19601.36	125.4037	19599.98	125.3927	19602.04	125.3954	19602.14	125.4002	19604.85	125.4019	19595.63	125.7155
125.645	19600.4	125.6446	19595.29	125.6611	19601.48	125.6381	19599.32	125.6464	19602.76	125.6422	19603.88	125.9454
125.8984	19599.18	125.8991	19597.67	125.8947	19602.14	125.8919	19601.84	125.897	19600.36	125.8902	19601.04	126.1972
126.1444	19598.51	126.1447	19600.71	126.1685	19598.67	126.1398	19599.49	126.1676	19601.26	126.1419	19596.51	126.4321
126.394	19598.86	126.4043	19601.35	126.3973	19599.25	126.3963	19600.71	126.4	19599.97	126.3883	19599.05	126.6712
126.6449	19599.96	126.6441	19598.02	126.6582	19600.44	126.6379	19603.82	126.6465	19602.36	126.6403	19598.13	126.9141
126.8982	19599.6	126.9	19601.7	126.8947	19602.84	126.8886	19601.88	126.9035	19599.18	126.89	19596.19	127.1739
127.1444	19596.88	127.146	19600.7	127.1501	19603.44	127.1398	19599.53	127.1472	19602.12	127.1414	19595.74	127.4152
127.3964	19601.47	127.4048	19598.98	127.3961	19602.58	127.3968	19603.81	127.4106	19600.79	127.3878	19599.13	127.6678
127.6443	19598.77	127.6488	19599.42	127.6586	19604.93	127.6384	19603.58	127.646	19601.47	127.6363	19602.76	127.9149
127.9019	19598.77	127.899	19602.17	127.8942	19602.48	127.8911	19600.61	127.901	19600.61	127.89	19598.21	128.1733
128.1463	19601.38	128.1473	19600.62	128.15	19602.41	128.1417	19599.89	128.1516	19601.37	128.1429	19597.48	128.4158
128.3961	19603.56	128.4057	19598.46	128.3961	19600.95	128.4008	19599.69	128.3992	19597.44	128.3894	19598.8	128.6685
128.6437	19600.32	128.6479	19600.37	128.6578	19603.42	128.6404	19604.85	128.6434	19597.88	128.6368	19599.15	128.9148
128.8991	19598.42	128.9007	19603.84	128.898	19596.43	128.888	19603.01	128.9028	19598.61	128.8891	19596.66	129.177
129.1469	19602.92	129.1449	19594.83	129.1494	19602.33	129.1405	19601.3	129.1504	19597.32	129.1418	19598.47	129.4154
129.3972	19599.16	129.421	19599.11	129.3946	19599.32	129.397	19601.08	129.3999	19602.56	129.3904	19597.31	129.6679
129.6459	19601.43	129.6464	19602.94	129.6629	19597.49	129.6433	19601.11	129.6448	19598.5	129.6408	19597.38	129.9145
129.9005	19602.22	129.901	19599.89	129.9047	19599.74	129.8915	19600.95	129.9003	19603.21	129.8892	19588.59	130.1734
130.1456	19597.79	130.1463	19599.94	130.1497	19596.2	130.1402	19600.27	130.1501	19597.46	130.1406	19596.65	130.416
130.3972	19598.08	130.406	19599.17	130.3968	19600.34	130.4073	19598.63	130.4008	19601.07	130.3908	19593.27	130.6664
130.6462	19600.12	130.6465	19600.31	130.6596	19598.28	130.6405	19602.11	130.6459	19601.57	130.6416	19599.65	130.9143
130.8986	19597.04	130.903	19597.43	130.8971	19596.03	130.8923	19601.63	130.9085	19602.43	130.8916	19597.33	131.1741
131.1458	19603.83	131.1476	19598.63	131.1525	19601.8	131.1421	19605.75	131.1515	19599.88	131.146	19595.69	131.416
131.3975	19601.54	131.4067	19605.23	131.3968	19598.51	131.399	19600.08	131.4017	19600.68	131.3956	19595.19	131.6671
131.6483	19600.03	131.6483	19596.47	131.6602	19601.86	131.641	19602.28	131.6481	19599.28	131.6415	19595.16	131.9151
131.8994	19600.3	131.9039	19600.91	131.8958	19595.68	131.8929	19601.29	131.8985	19594.56	131.8926	19600.38	132.1747
132.146	19598.71	132.1464	19602.03	132.1514	19601.11	132.1431	19597.63	132.1492	19601.06	132.1425	19596.72	132.4179
132.3971	19600.69	132.4046	19598.4	132.397	19602.07	132.3998	19600.76	132.4027	19601.06	132.3903	19596.31	132.6685
132.6472	19599.66	132.6504	19598.17	132.6597	19597.13	132.6431	19602.24	132.6477	19599.48	132.6432	19595.66	132.9148
132.9012	19599.46	132.9035	19595.56	132.8955	19599.72	132.8922	19597.25	132.9014	19602.42	132.8926	19597.6	133.1751
133.1488	19599.99	133.1484	19600.59	133.1525	19598.48	133.1468	19599.83	133.1532	19601.59	133.1457	19592.21	133.4154
133.3978	19594.95	133.4065	19600.72	133.3982	19599.1	133.3997	19601.12	133.4008	19598.56	133.3911	19594.53	133.6706
133.6477	19599.69	133.6474	19598.56	133.6612	19602.24	133.6415	19598.64	133.6474	19598.7	133.641	19593.63	133.9156
133.9027	19601.07	133.9013	19597.55	133.898	19601.3	133.8929	19602.43	133.9044	19595.6	133.8927	19594.2	134.177
134.1476	19601.69	134.1496	19597.12	134.1511	19601.02	134.1453	19597.26	134.153	19598.14	134.1434	19597.74	134.4159
134.3998	19598.27	134.4072	19597.93	134.3972	19602.31	134.3988	19596.28	134.4034	19597.11	134.393	19592.89	134.6709
134.6462	19597.07	134.6483	19599.27	134.6606	19598.27	134.6514	19596.1	134.6464	19598.14	134.6407	19595.47	134.9151
134.9011	19598.9	134.9018	19598.49	134.9019	19600.28	134.8947	19599.07	134.9036	19602.08	134.8911	19596.85	135.1726
135.1495	19603.26	135.1485	19599.65	135.1539	19598.86	135.143	19596.59	135.1551	19605.29	135.1565	19595.48	135.4131
135.3993	19600.22	135.4079	19597.39	135.3997	19600.1	135.3997	19596.51	135.4025	19598.44	135.3973	19594.07	135.6673
135.6473	19599.96	135.6475	19600.36	135.6616	19600.87	135.6448	19597.06	135.6478	19598.94	135.6423	19593.58	135.9152
135.9021	19601.2	135.9042	19598.01	135.8972	19599.1	135.8948	19602.82	135.9006	19600.98	135.8925	19596.18	136.1733
136.1491	19600.6	136.1503	19602.51	136.1537	19599.81	136.1442	19596.82	136.155	19599.32	136.1464	19594.69	136.4176
136.4002	19603.47	136.4077	19597.81	136.3997	19599.37	136.3995	19599.53	136.4052	19597.43	136.3953	19596.66	136.6699
136.6458	19600.84	136.6499	19598.72	136.6618	19596.53	136.6438	19602.51	136.649	19607.16	136.6427	19593.6	136.9172
136.916	19596.22	136.9021	19601.9	136.8977	19596.1	136.8966	19599.85	136.9032	19602.26	136.8937	19592.08	137.1793
137.1491	19603.49	137.1487	19603.44	137.1534	19603.09	137.1451	19598.07	137.156	19599.92	137.1452	19600.65	137.4187
137.4008	19598.16	137.4067	19602.06	137.4002	19599.94	137.4014	19596.27	137.4047	19604.21	137.3919	19597.19	137.6689
137.6484	19598.23	137.6524	19601.82	137.6611	19595.62	137.6448	19598.65	137.6504	19596.65	137.6432	19591.06	137.917
137.8995	19599.61	137.9048	19600.43	137.9013	19596.39	137.8937	19595.22	137.9021	19596.96	137.8947	19589.37	138.1737
138.1478	19601.26	138.1479	19599.02	138.1537	19604.23	138.1461	19602.54	138.1534	19599.36	138.1466	19595.09	138.4181
138.3994	19600.18	138.4077	19600.16	138.3991	19602.47	138.4022	19602.07	138.4034	19598.06	138.3929	19591.78	138.6702
138.6495	19600.16	138.6521	19599.26	138.6602	19601.18	138.646	19595.92	138.6517	19598.59	138.6435	19593.85	138.918

138.9047	19596.43	138.9047	19600.03	138.9023	19601.28	138.8941	19600.64	138.9036	19599.1	138.8952	19596.28	139.1788
139.149	19599.52	139.1506	19600.24	139.1537	19601.71	139.147	19598.87	139.1535	19599.56	139.1479	19598.2	139.4191
139.3989	19596.67	139.4093	19601.01	139.3985	19601.32	139.3996	19598.02	139.403	19599.65	139.3949	19596.06	139.6704
139.6488	19594.8	139.6512	19601.2	139.6629	19601.25	139.645	19596.67	139.65	19602.18	139.6429	19592.29	139.921
139.9083	19600.11	139.9056	19599.57	139.9031	19597.32	139.895	19602.88	139.9054	19598.75	139.8965	19596.19	140.1791
140.1501	19595.21	140.1507	19596.63	140.1553	19596.41	140.1473	19601.62	140.1647	19602.06	140.1472	19593.01	140.4193
140.4013	19598.59	140.4101	19597.67	140.3996	19601.59	140.4031	19602.52	140.4057	19597.65	140.3947	19596.34	140.6703
140.6492	19599.99	140.6501	19596.17	140.6609	19599.24	140.6453	19600.82	140.6475	19596.34	140.6436	19592.47	140.919
140.9037	19598	140.9032	19597.39	140.901	19601.32	140.8951	19593.94	140.9034	19603.06	140.8956	19592.41	141.1772
141.1506	19595.94	141.1508	19596.15	141.155	19599.79	141.144	19601.24	141.1549	19594.48	141.1468	19597.89	141.4228
141.4	19592.83	141.4108	19599.42	141.4015	19598.87	141.4078	19598.61	141.4042	19597.99	141.3955	19597.63	141.6767
141.6517	19598.41	141.6535	19598.82	141.6776	19598.16	141.6511	19600.01	141.6501	19602.02	141.6448	19596.82	141.9203
141.9017	19600.5	141.906	19598.4	141.8998	19601	141.8966	19600.64	141.9058	19597.91	141.8948	19596.03	142.1864
142.1504	19600.78	142.1513	19600.05	142.1555	19601.46	142.1453	19594.95	142.1532	19602.72	142.1474	19590.49	142.4296
142.4022	19596.17	142.4103	19598.06	142.4005	19594.14	142.4015	19599.76	142.4062	19601.75	142.3967	19591.87	142.6843
142.6515	19598.59	142.6552	19601.33	142.6639	19599.91	142.6441	19600.15	142.6487	19596.73	142.6461	19595.13	142.9224
142.905	19597.31	142.9055	19598.62	142.9008	19600.53	142.8962	19597.1	142.9027	19602.42	142.8968	19592.55	143.1804
143.1494	19594.48	143.1503	19598.34	143.1559	19600.47	143.1464	19597.6	143.1567	19598.95	143.1492	19599.31	143.4209
143.4009	19600.64	143.4102	19595.91	143.4	19596.96	143.404	19599.08	143.4076	19597.84	143.3941	19587	143.6756
143.6516	19595	143.6517	19600.73	143.6615	19597.53	143.6483	19599.17	143.6513	19599.65	143.6461	19590.19	143.9238
143.9025	19599.51	143.9067	19598.51	143.9015	19594.18	143.8956	19598.59	143.9053	19596.79	143.8969	19591.2	144.1802
144.1508	19597.95	144.1508	19596.32	144.1575	19599.25	144.147	19598.93	144.1533	19600.94	144.1506	19592.8	144.4202
144.4049	19595.66	144.4132	19599.65	144.401	19599.23	144.4022	19601.59	144.4062	19599.74	144.3959	19594.1	144.6748
144.6526	19600.44	144.652	19591.92	144.6652	19597.49	144.6458	19595.4	144.6522	19597.71	144.6456	19596.49	144.9222
144.9048	19596.06	144.9069	19597.2	144.9062	19601.33	144.894	19598.89	144.9074	19595.5	144.898	19592.05	145.1784
145.1527	19600.16	145.1525	19597.77	145.1584	19596.71	145.1464	19601.21	145.1547	19598.48	145.1526	19590.38	145.4194
145.4005	19593.04	145.4097	19596.19	145.4034	19599.33	145.4022	19600.47	145.4073	19598.11	145.3967	19596.29	145.6736
145.6521	19601	145.6525	19595.17	145.6612	19603.33	145.6472	19600.03	145.6506	19598.03	145.6457	19593.1	145.9225
145.905	19597.34	145.9068	19597.33	145.9016	19599.54	145.8977	19595.37	145.9071	19601.78	145.8985	19587.48	146.1795
146.1531	19600.38	146.1536	19599.26	146.1572	19599.14	146.1471	19597.09	146.1554	19599.49	146.1516	19595.45	146.4204
146.4015	19597.08	146.412	19596.37	146.4018	19601.31	146.4051	19598.52	146.408	19602.25	146.3975	19594.45	146.6739
146.6512	19599.65	146.6521	19600.64	146.6631	19596.6	146.6481	19602.91	146.6517	19594.78	146.6459	19593.88	146.9229
146.9042	19597.12	146.9078	19594.5	146.9017	19597.28	146.8986	19597.45	146.9087	19598.71	146.8959	19592.12	147.1857
147.1555	19599.96	147.1542	19600.97	147.1563	19599.54	147.1491	19595.35	147.1556	19596.9	147.1506	19592.64	147.4209
147.4034	19602.75	147.4151	19595.23	147.4029	19600.31	147.4036	19600.95	147.4088	19596.23	147.3971	19592.62	147.6761
147.6573	19597.16	147.6561	19596.41	147.6653	19597.96	147.6652	19597.86	147.6523	19596.26	147.6463	19587.64	147.9232
147.9028	19594.84	147.9074	19598.14	147.9019	19597.25	147.9024	19596.71	147.9068	19599.1	147.898	19589.76	148.1793
148.1537	19596.04	148.1555	19593.09	148.159	19598.35	148.1486	19596.14	148.1561	19598.73	148.1533	19592.51	148.4233
148.4026	19597.49	148.4143	19597.48	148.4024	19600.1	148.4045	19597.64	148.4088	19601.91	148.3973	19590.22	148.6758
148.6523	19596.45	148.6525	19596.19	148.6641	19601.47	148.6492	19596.03	148.6521	19603.55	148.6487	19592.39	148.9228
148.9041	19595.57	148.9071	19597.09	148.9031	19599.59	148.8986	19599.66	148.9074	19598.06	148.8983	19595.84	149.1773
149.1519	19594.77	149.1531	19592.6	149.1579	19593.11	149.1484	19600.22	149.1556	19597.69	149.1509	19592.27	149.4222
149.4026	19598.05	149.4131	19595.25	149.4021	19599.93	149.4078	19597.93	149.412	19597.02	149.397	19595.5	149.6756
149.6545	19594.89	149.6528	19592.02	149.6711	19600.9	149.6468	19598.01	149.6557	19600.81	149.6474	19592.41	149.9227
149.9099	19598.63	149.9087	19598.05	149.9138	19600.21	149.8987	19599.98	149.906	19597.65	149.8982	19592.78	150.1835
150.1516	19595.93	150.1534	19598.83	150.159	19594.83	150.1502	19597.98	150.1546	19601.84	150.1493	19586.32	150.4232
150.408	19597.48	150.4372	19596.86	150.4068	19598.01	150.4081	19596.92	150.4125	19599.29	150.3991	19594.51	150.6792
150.6579	19597.31	150.6566	19595.97	150.6685	19599.64	150.6518	19602.61	150.6576	19592.21	150.6512	19585.5	150.9266
150.9108	19599.51	150.9129	19597.84	150.9065	19597.19	150.9013	19598.87	150.912	19600.65	150.9014	19594.17	151.1808
151.157	19598.95	151.1579	19600.44	151.1614	19599	151.1505	19598.34	151.1579	19599.52	151.154	19598.27	151.4264
151.4069	19597.7	151.4185	19593.45	151.4076	19596.15	151.4127	19596.27	151.4123	19596.64	151.4036	19588.01	151.6819
151.6565	19597.29	151.659	19600.83	151.6714	19594.55	151.653	19598.11	151.6576	19596.88	151.6503	19590.63	151.9266
151.9127	19598.33	151.9126	19599.07	151.9075	19599.04	151.9031	19598.33	151.9149	19597.01	151.9018	19590.2	152.1874
152.1582	19601.3	152.1587	19596.58	152.1632	19596.44	152.1513	19598.12	152.1601	19595.43	152.1548	19596.66	152.4307
152.4095	19599.11	152.4206	19602.51	152.4075	19597.99	152.4089	19598.94	152.416	19600.1	152.4032	19587.02	152.6836
152.6587	19597.31	152.66	19595	152.6704	19596.95	152.6532	19601.49	152.6582	19599.83	152.6506	19593.75	152.9603
152.9103	19596.75	152.914	19595.41	152.9083	19594.22	152.9022	19594.48	152.9124	19595.06	152.9007	19590.62	153.1874
153.1572	19597.29	153.1732	19597.25	153.1635	19598.01	153.1523	19595.7	153.1621	19600.5	153.154	19596.1	153.4312
153.4094	19600.66	153.4237	19594.19	153.4084	19597.67	153.4103	19596.09	153.432	19596.85	153.4094	19583.35	153.685
153.6602	19595.88	153.6571	19599.04	153.6694	19595.75	153.6528	19596.85	153.6599	19600.4	153.6546	19594.55	153.9328
153.9092	19597.42	153.913	19594.93	153.9082	19594.76	153.905	19597.88	153.9124	19600.45	153.9063	19593.14	154.1884
154.1577	19601.41	154.1592	19599.21	154.1644	19593.63	154.1542	19595.12	154.1621	19595.52	154.1585	19591.9	154.434
154.4078	19593.33	154.422	19599.79	154.4069	19594.51	154.4129	19596.12	154.4157	19601.41	154.4097	19593.91	154.6847
154.6598	19599.63	154.6599	19599.88	154.6731	19592.87	154.6537	19596.34	154.6596	19592.23	154.6536	19591	154.9323
154.9103	19599.8	154.9132	19598.89	154.9111	19598.31	154.9067	19596.08	154.9138	19597.31	154.9038	19588.92	155.1871
155.1584	19592.29	155.1592	19595.3	155.166	19597	155.1554	19600.92	155.1603	19595.86	155.1549	19592.98	155.4329
155.4078	19596.29	155.4193	19595.88	155.4073	19598.04	155.413	19598.61	155.4188	19601.23	155.405	19589.35	155.6859
155.6608	19597.37	155.6585	19597.43	155.6739	19595.9	155.6544	19600.62	155.6594	19598.35	155.6533	19587.5	155.932
155.9103	19596.85	155.9158	19597.48	155.9168	19594.65	155.9034	19598.24	155.9162	19592.75	155.9066	19593.02	156.1923
156.1592	19597.22	156.1599	19596.21	156.1635	19597.04	156.1539	19596.33	156.1596	19598.23	156.1574	19594.34	156.4319

156.4077	19595.33	156.4216	19600.75	156.4083	19596.93	156.4116	19596.07	156.4148	19596.79	156.4071	19586.17	156.6847
156.6603	19595.96	156.6614	19596.62	156.6713	19599.94	156.6532	19603.5	156.6591	19602.41	156.6526	19585.85	156.9332
156.938	19597.1	156.9189	19597.15	156.9117	19594.4	156.9028	19601.61	156.916	19594.78	156.9045	19593.6	157.1931
157.166	19595.63	157.1599	19595.23	157.1648	19599.32	157.1543	19598.98	157.162	19596.35	157.1573	19597.06	157.4304
157.4107	19592.85	157.4212	19598.35	157.4094	19599.14	157.4122	19595.89	157.4166	19601.69	157.4048	19592.68	157.6875
157.6594	19597.57	157.6641	19598.73	157.6737	19597.57	157.6544	19599.04	157.6602	19595.62	157.6525	19595.46	157.9342
157.9125	19593.27	157.9134	19597.61	157.9115	19597.88	157.905	19596.14	157.9167	19596.07	157.9043	19594.63	158.1906
158.1605	19595.35	158.1599	19597.56	158.1649	19596.42	158.154	19597.75	158.1642	19594.58	158.1568	19592.31	158.4327
158.4091	19599.04	158.4197	19597.04	158.4101	19597.14	158.4148	19602.9	158.4176	19599.23	158.4049	19591.82	158.6892
158.6609	19601.22	158.6611	19595.72	158.6736	19599.29	158.6568	19595.49	158.659	19599.92	158.6528	19594.15	158.9332
158.9111	19596.84	158.9164	19597.39	158.9098	19596.63	158.9058	19596.8	158.915	19600.54	158.9048	19587.27	159.1908
159.1607	19597.09	159.1596	19596.73	159.1661	19595.18	159.1555	19594.71	159.1632	19599.99	159.1564	19591.15	159.4333
159.411	19591.48	159.4219	19600.6	159.4108	19596.89	159.4133	19598	159.4169	19598.57	159.4088	19594.44	159.6864
159.6606	19597.33	159.6607	19597.96	159.6728	19593.33	159.6562	19596.38	159.6596	19596.14	159.6535	19589.58	159.9313
159.9132	19593.99	159.9164	19599.27	159.9117	19596.24	159.907	19591.77	159.9148	19597.81	159.9039	19585.44	160.1891
160.1594	19596.87	160.1624	19598.63	160.1638	19598.99	160.1542	19597.85	160.1597	19599.39	160.1572	19589.68	160.4358
160.4116	19598.24	160.4211	19597.38	160.418	19599.55	160.4154	19597.93	160.4174	19598.33	160.4045	19587.73	160.6844
160.6589	19592.93	160.6578	19594.42	160.6717	19596.46	160.6524	19597.19	160.6591	19598.73	160.6531	19598.07	160.9342
160.906	19596.38	160.9165	19596.67	160.9161	19599.39	160.9035	19598.92	160.9136	19596.31	160.9052	19591.51	161.1894
161.1579	19595.51	161.1614	19596.19	161.1633	19595.59	161.1531	19596.56	161.1564	19593.95	161.1546	19589.4	161.4313
161.4079	19594.8	161.424	19596.86	161.4072	19599.38	161.4107	19593.46	161.4135	19600.02	161.4037	19587.76	161.6854
161.6588	19598.7	161.6572	19594.99	161.6843	19597.43	161.653	19596.7	161.661	19594.18	161.6527	19590.36	161.9383
161.9098	19594.76	161.9132	19596.1	161.9221	19597.13	161.9028	19594.22	161.9137	19595.06	161.905	19589.32	162.1893
162.1583	19597.7	162.1602	19593.89	162.1638	19598.87	162.1541	19595.94	162.1592	19593.83	162.1555	19590.01	162.4326
162.4096	19597.92	162.4204	19596.06	162.41	19599.78	162.4115	19599.38	162.4135	19596.87	162.4031	19589.45	162.6835
162.6586	19594.8	162.662	19595.75	162.6733	19596.42	162.6526	19594.39	162.6597	19595.55	162.6534	19591.7	162.9333
162.9118	19596.19	162.9118	19597.58	162.9087	19591.65	162.9213	19599.03	162.9152	19598.2	162.9031	19593.58	163.1897
163.1595	19600.32	163.1596	19594.9	163.164	19598.76	163.1674	19601.21	163.1624	19597.64	163.1566	19591.48	163.4331
163.4084	19598.5	163.4189	19596.19	163.4094	19594.33	163.4182	19594.45	163.4145	19598.57	163.4057	19593.33	163.6851
163.6572	19598.88	163.6596	19595.51	163.6731	19596.73	163.6611	19598.55	163.6581	19596.35	163.6551	19590.2	163.9332
163.9106	19595.14	163.9157	19596.91	163.9082	19597.86	163.9129	19595.83	163.9149	19598.09	163.9051	19591.62	164.187
164.1607	19592.75	164.1628	19594.9	164.1642	19600.07	164.1657	19596.41	164.1624	19595.45	164.1559	19588.82	164.4343
164.4112	19590.72	164.422	19595.14	164.4105	19592.18	164.4192	19596.16	164.4154	19600.36	164.4066	19592.31	164.6856
164.6578	19595.6	164.6625	19592.44	164.6704	19597.51	164.6586	19594.5	164.6576	19594.93	164.6562	19588.67	164.9332
164.9093	19596.87	164.915	19597.96	164.911	19598.63	164.9105	19592.75	164.914	19599.07	164.9037	19585	165.1882
165.1596	19599.33	165.1642	19598.3	165.1642	19596.38	165.1742	19594.07	165.163	19600.67	165.1575	19587.36	165.4324
165.4118	19590.33	165.4201	19595.7	165.4094	19596.19	165.4187	19598.44	165.4156	19594.82	165.407	19590.52	165.6851
165.6663	19598.21	165.6613	19594.66	165.6696	19597.52	165.6599	19600.55	165.6601	19595.91	165.6559	19593.47	165.9336
165.9139	19592.27	165.9157	19597.54	165.911	19596.06	165.9108	19596.99	165.9132	19596.54	165.9046	19586.17	166.1883
166.1645	19601.42	166.1615	19596.02	166.1683	19590.44	166.1636	19595.73	166.1614	19596.27	166.1587	19594.92	166.4346
166.4148	19593.48	166.4219	19594.99	166.4083	19596.94	166.4171	19594.85	166.4153	19596.04	166.4072	19584.15	166.6864
166.662	19597.35	166.6665	19595.13	166.6715	19594.97	166.6617	19596.39	166.6598	19597.13	166.6575	19590.02	166.9351
166.9111	19597.87	166.9198	19595.99	166.9116	19600.28	166.908	19599.92	166.9146	19597.77	166.9068	19588.02	167.1938
167.1616	19592.53	167.1632	19599.7	167.165	19596.2	167.161	19597.02	167.1598	19602.14	167.1578	19591.35	167.4345
167.4112	19593.64	167.4239	19594.41	167.4104	19593.83	167.4156	19597.8	167.4167	19597.72	167.4058	19586.73	167.6871
167.6605	19598.97	167.6636	19594.34	167.6691	19596.11	167.6629	19598.47	167.6607	19596.76	167.6628	19592.39	167.9346
167.911	19594.14	167.9195	19596.32	167.9113	19597.7	167.9122	19595.93	167.915	19601.12	167.909	19591.63	168.1911
168.161	19594.04	168.1638	19592.84	168.1659	19598.11	168.1597	19595.92	168.1622	19599.64	168.1585	19588.06	168.4346
168.411	19598.82	168.4218	19595.13	168.4114	19598.81	168.4156	19596.94	168.4182	19595.45	168.4045	19591.08	168.6871
168.6594	19591.04	168.6617	19595.48	168.6742	19598.62	168.6607	19596.47	168.6603	19596.13	168.6558	19594.56	168.9358
168.9124	19597.03	168.9156	19595.68	168.9107	19599.63	168.9096	19593.66	168.9171	19594.25	168.9059	19592.22	169.1898
169.1627	19596.02	169.1622	19596.1	169.1662	19599.22	169.1598	19595.54	169.1644	19594.66	169.1576	19585.18	169.432
169.4133	19595.2	169.4226	19597.22	169.4123	19595.5	169.4165	19597.98	169.4157	19600.17	169.4058	19593.17	169.6958
169.6661	19595.23	169.6647	19594.71	169.6695	19596.66	169.6621	19599.37	169.6609	19596.89	169.6588	19590.11	169.9349
169.9106	19598.29	169.9169	19598.45	169.9139	19594.27	169.9121	19593.23	169.9163	19599.19	169.9109	19588.69	170.1904
170.1615	19597.23	170.164	19594.89	170.1662	19597.51	170.1611	19595.32	170.1629	19594.38	170.1588	19588.1	170.4357
170.413	19594.75	170.4403	19592.35	170.4118	19597.1	170.4166	19596.86	170.4173	19595.28	170.4077	19586.91	170.6896
170.6654	19597.13	170.6623	19597.19	170.6797	19599.91	170.6606	19596.04	170.6617	19593.97	170.658	19585.44	170.9365
170.9143	19595.28	170.9201	19596.88	170.9212	19596.83	170.9095	19594.7	170.9155	19601.59	170.9081	19584.18	171.1908
171.1603	19594.57	171.1666	19597.37	171.1676	19600.53	171.1711	19597.33	171.163	19596.72	171.1593	19593.64	171.4369
171.4115	19592.01	171.424	19594.81	171.4106	19596.57	171.4222	19597.85	171.4289	19596.32	171.4099	19586.95	171.6881
171.6632	19595.85	171.6627	19597.92	171.6753	19598.2	171.6619	19595.48	171.663	19593.58	171.6571	19592.75	171.9377
171.9151	19598.9	171.9204	19596.18	171.9138	19597.47	171.911	19592.16	171.916	19595.63	171.9089	19584.55	172.1911
172.1618	19596.32	172.1666	19594.19	172.1677	19596.03	172.1625	19591.36	172.1627	19595.23	172.1584	19588.12	172.4354
172.4118	19594.17	172.4241	19591.57	172.4129	19599.42	172.4178	19598.89	172.4168	19596.77	172.407	19588.54	172.6886
172.663	19596.33	172.6643	19598.32	172.6733	19594.58	172.6646	19600.03	172.6631	19595.2	172.6572	19586.6	172.9366
172.9134	19592.52	172.9178	19597.57	172.9132	19592.93	172.9141	19597.78	172.9189	19599.77	172.9087	19594.18	173.1913
173.163	19594.86	173.165	19595.78	173.1679	19597.34	173.1846	19595.58	173.1637	19594.12	173.159	19591.06	173.4367
173.4134	19599.16	173.423	19595.14	173.4128	19594.24	173.4248	19593.77	173.4197	19595.26	173.4055	19587.06	173.6901
173.661	19593.28	174.2019	19594.1	173.6748	19595.1	173.6636	19595.33	173.6623	19596.73	173.6567	19589.75	173.9366

173.9139	19596.83	174.4501	19594.55	173.9138	19595.04	173.9134	19595.23	173.9168	19596.07	173.9094	19588.93	174.1914
174.1641	19596.42	174.6923	19594.34	174.1681	19593.8	174.1639	19594.14	174.1639	19595.36	174.1601	19590.67	174.4365
174.4143	19594.3	174.9465	19597.91	174.4136	19596.93	174.4176	19591.93	174.4237	19595.39	174.4063	19584.17	174.6903
174.6638	19597.56	175.1946	19594.58	174.6746	19597.03	174.6672	19598.39	174.6664	19596.39	174.6561	19586.32	174.9377
174.9167	19596.27	175.4491	19597.23	174.9169	19593.85	174.9137	19594.19	174.9176	19599.28	174.9067	19588.54	175.1936
175.1778	19595.66	175.6919	19589.35	175.1695	19598.08	175.1668	19593.86	175.1625	19596.59	175.1609	19591.28	175.438
175.4143	19595.44	175.951	19596.49	175.4172	19594.11	175.4195	19598.22	175.4196	19597.2	175.4083	19589.82	175.6909
175.6639	19594.28	176.1981	19592.82	175.6746	19600.1	175.664	19598.09	175.6668	19597.75	175.6575	19590.89	175.9385
175.9157	19591.21	176.4504	19593.79	175.9182	19596.35	175.9263	19597.22	175.9177	19597.71	175.9084	19587.23	176.1917
176.1713	19598.38	176.6919	19594.43	176.1685	19594.54	176.1685	19600.21	176.1643	19598.77	176.1619	19586.32	176.4372
176.4173	19596.31	176.9441	19595.36	176.4126	19593.81	176.4258	19597.52	176.419	19597.94	176.4129	19590.25	176.6886
176.6645	19596.23	177.1944	19597.9	176.6757	19592.89	176.6696	19596.03	176.6649	19597.78	176.6602	19583.79	176.9396
176.9205	19593.97	177.4495	19597.54	176.9159	19593.77	176.9214	19595.39	176.9198	19596.45	176.9061	19591.38	177.1959
177.164	19593.67	177.6946	19593.48	177.1722	19596.77	177.1767	19593.33	177.1637	19594.85	177.1631	19591.69	177.438
177.4151	19590.67	177.9438	19595.75	177.4141	19590	177.424	19595.32	177.4192	19593.09	177.4088	19589.37	177.6895
177.6636	19593.76	178.1946	19594.7	177.6758	19594.02	177.6693	19594.08	177.6658	19595.53	177.6595	19587.39	177.9392
177.9266	19597	178.4517	19591.92	177.9179	19594.26	177.9194	19597.53	177.92	19590.39	177.9093	19589.84	178.1961
178.1647	19593.48	178.6925	19592.15	178.1688	19597.14	178.1695	19597.52	178.1687	19597.08	178.1641	19585.89	178.4379
178.4148	19599.36	178.9465	19593.75	178.4151	19597.75	178.4238	19593.05	178.4193	19598.13	178.4104	19587.57	178.6897
178.6635	19595.72	179.1978	19592.9	178.6764	19595.52	178.6707	19594.59	178.6677	19594.87	178.6622	19589.12	178.9384
178.9189	19592.62	179.4548	19598.6	178.9173	19599.09	178.9182	19593.92	178.92	19593.96	178.9162	19587.09	179.192
179.1652	19590.22	179.6992	19596	179.1685	19596.95	179.1687	19596.82	179.1652	19593.46	179.1667	19593.13	179.4376
179.4151	19594.5	179.9499	19596.6	179.416	19597.83	179.4239	19596.04	179.4239	19595.39	179.4105	19589.55	179.6909
179.6666	19593.38	180.1989	19596.12	179.6763	19593.71	179.6712	19594.62	179.6673	19595.56	179.6604	19588.8	179.9405
179.9158	19592.99	180.451	19595.34	179.9196	19596.26	179.9228	19598.22	179.921	19596.78	179.9123	19586.78	180.197
180.1648	19598.29	180.6943	19592.47	180.1702	19596.37	180.1691	19594.53	180.1649	19592.75	180.1638	19588.89	180.4393
180.4171	19594.55	180.9481	19592.6	180.4163	19592.89	180.4244	19596.07	180.4213	19591.66	180.4099	19590.88	180.6905
180.6666	19596.3	181.1983	19595.75	180.6762	19597.92	180.669	19594.79	180.6674	19595.92	180.6599	19584.2	180.9392
180.9171	19593.73	181.4538	19594.76	180.9163	19596.12	180.925	19593.25	180.92	19596.36	180.9129	19590.04	181.1964
181.1655	19598.91	181.6944	19595.95	181.1718	19596.17	181.1762	19592.62	181.1675	19594.55	181.1634	19585.87	181.4404
181.4157	19595.21	181.9517	19596.65	181.4157	19596.01	181.4346	19594.59	181.4234	19597.33	181.413	19586.12	181.6942
181.6659	19600.04	182.2	19596.91	181.6956	19597.9	181.6754	19597.92	181.6679	19597.96	181.6615	19585	181.9419
181.9171	19595.25	182.4539	19598.08	181.9182	19592.2	181.9339	19593.27	181.9235	19596.65	181.9106	19587.77	182.1957
182.1649	19596.53	182.697	19596.08	182.1696	19595.31	182.1854	19599.45	182.1663	19593.71	182.1633	19586.69	182.4404
182.4189	19595.36	182.951	19593.27	182.416	19594.2	182.431	19598.24	182.4224	19597.61	182.4126	19588.15	182.6949
182.666	19596.99	183.1998	19596.25	182.676	19591.94	182.679	19595.23	182.6694	19593.33	182.662	19587.36	182.9417
182.9184	19591.96	183.4552	19597.58	182.9185	19593.93	182.9255	19593.51	182.9234	19596.66	182.911	19589.85	183.1974
183.1668	19594.84	183.6947	19597.48	183.171	19597.36	183.1736	19594.67	183.1664	19591.77	183.164	19587	183.4399
183.4159	19595.79	183.9479	19591.79	183.4171	19593.9	183.4314	19592.04	183.4226	19596.52	183.4124	19586.05	183.6932
183.6674	19593.94	184.2003	19595.47	183.677	19595.97	183.6743	19597.57	183.6913	19595.57	183.6622	19592.61	183.941
183.9171	19593.56	184.4558	19592.45	183.9188	19596.42	183.9235	19595.92	183.9213	19597	183.9121	19587.42	184.1977
184.1663	19596.2	184.696	19591.67	184.1724	19597.39	184.1817	19595.04	184.1679	19591.93	184.1644	19585.3	184.4401
184.4172	19595.04	184.9477	19595.68	184.4167	19594.39	184.4302	19592.46	184.4238	19594.18	184.4213	19582.6	184.6938
184.6683	19592.68	185.1976	19595.09	184.6771	19594.82	184.6747	19593.56	184.6697	19596.03	184.6641	19589.8	184.9434
184.9194	19595.79	185.4541	19593.6	184.921	19595.68	184.919	19597.98	184.9226	19593.39	184.9149	19582.87	185.1942
185.1824	19592.19	185.6971	19595.98	185.173	19592.93	185.1697	19589.2	185.1658	19600.79	185.165	19583.13	185.4427
185.4184	19596.2	185.9505	19590.32	185.4218	19593.11	185.4246	19593.87	185.4238	19598.63	185.4148	19587.75	185.6943
185.6697	19594.88	186.1971	19597.29	185.6755	19600.1	185.6713	19594.1	185.6745	19592.86	185.6636	19585.51	185.9415
185.9176	19594.78	186.4616	19595.66	185.9287	19591.46	185.9197	19595.48	185.922	19592.95	185.9127	19586.59	186.1962
186.1674	19595.6	186.6985	19595.84	186.1737	19595.7	186.1688	19596.95	186.1665	19596.84	186.1682	19589.91	186.4413
186.4186	19596.15	186.9515	19593.2	186.4293	19597.51	186.4288	19599.5	186.4242	19598.11	186.4166	19582.6	186.6938
186.67	19595.5	187.1989	19597.58	186.679	19593.37	186.6914	19592.74	186.669	19595.75	186.6635	19586.97	186.941
186.9182	19597.51	187.4575	19595.17	186.9194	19595.98	186.9146	19591.55	186.924	19599.53	186.9132	19593.33	187.2002
187.1678	19597.96	187.6968	19596.5	187.1741	19594.01	187.1756	19594.64	187.1683	19598.48	187.165	19586.15	187.4406
187.4196	19594.7	187.952	19590.75	187.418	19599.84	187.4283	19595.17	187.4216	19596.85	187.4138	19587.31	187.6936
187.6687	19591.79	188.1992	19595.06	187.6789	19594.5	187.6795	19595.92	187.6689	19596.58	187.665	19589.3	187.941
187.9209	19593.35	188.4589	19594.51	187.9197	19597.47	187.9148	19598.31	187.9266	19597.49	187.9128	19587.94	188.2013
188.1693	19594.72	188.696	19595.24	188.1728	19596.72	188.1643	19594.85	188.1689	19592.64	188.1659	19582.33	188.4419
188.4185	19594.04	188.9495	19591.98	188.4187	19597.26	188.4267	19596.26	188.4306	19597.88	188.4133	19588.65	188.695
188.6698	19592.69	189.199	19595	188.6785	19594.63	188.6651	19590.15	188.668	19594.84	188.6644	19582.05	188.9444
188.9203	19597.17	189.4563	19595.36	188.919	19595.66	188.9138	19598.41	188.926	19598.3	188.9132	19587.99	189.1994
189.1719	19590.56	189.699	19595.17	189.1734	19599.71	189.164	19592.88	189.1696	19596.92	189.1693	19587.22	189.443
189.4187	19595.1	189.9507	19594.66	189.4195	19597.11	189.4215	19590.61	189.423	19598.69	189.4149	19587.1	189.6954
189.6703	19590.14	190.198	19593.82	189.6779	19596.77	189.6664	19596.25	189.6677	19595.11	189.6652	19596.32	189.9421
189.9184	19597.79	190.4666	19595.77	189.9237	19597.5	189.9147	19593.75	189.9233	19593.73	189.9143	19586.26	190.1983
190.1716	19594.49	190.6988	19592.81	190.1744	19595.62	190.1771	19594.73	190.1694	19590.72	190.1665	19590.64	190.4412
190.4197	19591.13	190.9504	19591.62	190.42	19594.01	190.4262	19595.26	190.4244	19591.53	190.4135	19582.83	190.6944
190.6703	19589.66	191.1996	19596.86	190.6791	19595.31	190.6656	19594.21	190.6702	19597.6	190.6665	19592.12	190.9436
190.9257	19593.65	191.4605	19595.83	190.9203	19593.46	190.9151	19595.67	190.9243	19596.65	190.916	19590.23	191.1968
191.1711	19590.94	191.6996	19592.59	191.1752	19595.24	191.1655	19593.66	191.1696	19598.61	191.169	19587.34	191.4425

191.42	19596.69	191.9524	19593.1	191.4235	19593.76	191.4232	19592.71	191.4264	19594.15	191.4165	19589.31	191.6948
191.6709	19593.73	192.1992	19597.66	191.6828	19590.75	191.6665	19595.61	191.6703	19596.66	191.6669	19582.95	191.9424
191.9208	19594.72	192.4561	19590.7	191.9213	19597.99	191.9153	19595.63	191.9236	19593.1	191.9154	19584.3	192.2021
192.1692	19595.06	192.7005	19600.07	192.1758	19595.17	192.1655	19596.29	192.1697	19592.45	192.1673	19585.47	192.4427
192.4206	19595.05	192.9527	19594.4	192.4192	19592.41	192.4212	19596.62	192.4279	19590.79	192.4148	19584.39	192.695
192.675	19594.93	193.1997	19599.4	192.6807	19594.39	192.6665	19595.1	192.6712	19594.84	192.6657	19584.36	192.9449
192.9206	19597.59	193.462	19597.68	192.9219	19594.7	192.9159	19593.99	192.9245	19593.37	192.915	19586.47	193.2011
193.1708	19591.46	193.6988	19591.76	193.176	19592.23	193.1671	19591.97	193.1716	19593.29	193.1687	19584.94	193.4433
193.4203	19598.53	193.9531	19592.46	193.4211	19594.07	193.4248	19593.58	193.4264	19596.55	193.4149	19581.52	193.6953
193.6727	19593.75	194.2015	19596.93	193.6828	19591.73	193.6669	19591.93	193.6706	19593.42	193.6683	19588.28	193.9453
193.922	19595.27	194.4603	19593.32	193.9219	19596.59	193.9169	19591.2	193.9269	19595.54	193.9148	19586.59	194.2018
194.1712	19593.4	194.7012	19596.46	194.1772	19597.19	194.1698	19596.26	194.1716	19597.33	194.1691	19591.08	194.4419
194.4205	19589.93	194.9526	19594.65	194.4221	19594.2	194.4254	19593.62	194.4281	19595.56	194.4153	19585.98	194.6968
194.6724	19594.2	195.1987	19595.65	194.682	19594.01	194.6671	19597.91	194.6706	19598.74	194.6667	19584.17	194.9447
194.923	19594.9	195.4593	19592.54	194.9235	19594.82	194.9225	19597.42	194.9272	19598.95	194.9154	19589.71	195.2022
195.1731	19592.39	195.7009	19596.43	195.1783	19595.34	195.1666	19597.71	195.1722	19598.21	195.168	19587.4	195.4445
195.4204	19590.48	195.9546	19595.79	195.4219	19591.15	195.4226	19595.92	195.4258	19599.87	195.4296	19582.13	195.6944
195.6706	19594.5	196.204	19595.21	195.6816	19594.1	195.6692	19596.82	195.6723	19593.35	195.6683	19583.18	195.9457
195.9231	19592	196.4601	19591.17	195.9231	19595.37	195.9198	19591.93	195.9261	19595.4	195.9172	19583.57	196.1998
196.1734	19594.8	196.7001	19592.44	196.1778	19592.2	196.1659	19594.07	196.1725	19595.81	196.1669	19584.68	196.4439
196.4223	19596.33	196.9544	19596.2	196.4222	19591.32	196.4222	19593.16	196.4283	19593.83	196.4175	19586.81	196.6978
196.6698	19592.43	197.2014	19597.56	196.6824	19594.37	196.669	19593.99	196.6718	19594.16	196.6681	19585.13	196.9461
196.922	19592.12	197.4609	19593	196.9234	19592.32	196.9189	19596.92	196.929	19593.02	196.9171	19588.73	197.204
197.1872	19593.65	197.7017	19593.88	197.1778	19594.48	197.1705	19593.91	197.1727	19591.64	197.1699	19580.31	197.4448
197.4241	19594.96	197.9571	19590.55	197.4224	19595.85	197.4246	19594.59	197.4289	19593.91	197.4168	19585.5	197.6965
197.6747	19595.6	198.2018	19595.36	197.6822	19595.77	197.6682	19592.35	197.6724	19597.89	197.6673	19586.87	197.9471
197.9269	19590.2	198.465	19590.39	197.9232	19593.19	197.918	19596.54	197.9284	19593.28	197.924	19581.64	198.1998
198.1766	19590.18	198.7002	19596	198.1794	19595.21	198.1677	19596.34	198.1719	19595.95	198.1691	19585.19	198.4467
198.4232	19593.14	198.9546	19592.93	198.423	19593.5	198.4264	19592.5	198.4295	19596.43	198.4187	19582.76	198.6982
198.6742	19592.24	199.203	19594.43	198.682	19591.8	198.6709	19599.84	198.6723	19593.64	198.6688	19588.51	198.9458
198.9221	19596.09	199.462	19597.07	198.9243	19594.99	198.9228	19594.83	198.9287	19594.74	198.9198	19587.86	199.2048
199.1742	19594.87	199.7004	19595.44	199.179	19595.89	199.168	19596.46	199.1724	19596.13	199.1709	19585.22	199.446
199.4237	19595.94	199.9546	19595.87	199.4215	19594.36	199.4256	19595.24	199.428	19598.77	199.4181	19581.67	199.6984
199.6733	19596.6	200.2016	19588.27	199.6854	19596.4	199.6715	19593.67	199.6726	19587.86	199.6714	19589.41	199.9471
199.9217	19592.82	200.4681	19593.66	199.9269	19595.1	199.921	19589.99	199.9291	19598.69	199.9206	19589.6	200.2028
200.1744	19591.53	200.7075	19597.03	200.1783	19592.89	200.1698	19593.6	200.1757	19592.11	200.1696	19588.62	200.4486
200.4289	19588.59	200.9582	19592.06	200.4289	19594.77	200.4306	19590.57	200.4326	19593.61	200.4253	19582.68	200.7025
200.6792	19592.44	201.2056	19593.12	200.688	19596.89	200.6722	19594.41	200.6787	19598.73	200.6724	19587.02	200.9513
200.928	19596.1	201.4667	19592.14	200.9279	19595.12	200.9262	19597.37	200.935	19597.88	200.9243	19587.58	201.2072
201.1809	19593.97	201.7068	19596.86	201.1837	19593.3	201.1744	19595.47	201.1817	19600.74	201.1748	19586	201.4519
201.4292	19592.74	201.9603	19595.29	201.4317	19594.2	201.4289	19592.05	201.4318	19598.11	201.4245	19588.81	201.7033
201.6786	19594.84	202.2064	19600.52	201.7042	19592.88	201.6782	19591.38	201.6775	19597.77	201.6777	19581.8	201.9517
201.9287	19592.71	202.4674	19593.68	201.9282	19593.74	201.9248	19593.7	201.9349	19593.64	201.926	19581.2	202.2079
202.182	19594.41	202.7093	19591.25	202.1826	19595.15	202.1736	19593.83	202.1792	19596.67	202.1757	19584.42	202.4507
202.4282	19594.34	202.9607	19596.21	202.4289	19593.83	202.4291	19594.32	202.4354	19595.41	202.4216	19582.59	202.7045
202.6777	19592.79	203.2078	19590.64	202.6856	19594.1	202.6749	19590.98	202.6787	19599.02	202.6722	19585.01	202.9527
202.9301	19592.31	203.4663	19592.27	202.9301	19594.6	202.9273	19594.4	202.9335	19595.43	202.927	19582.59	203.2081
203.1777	19592.97	203.7066	19593.09	203.1846	19596.44	203.1747	19595.56	203.178	19600.23	203.1748	19586.73	203.4508
203.4289	19594.8	203.9616	19591.68	203.4286	19596.4	203.4301	19593.13	203.4364	19594.42	203.4221	19584.08	203.7051
203.6804	19592.84	204.2079	19594.95	203.6916	19597.07	203.6745	19591.98	203.6792	19595.59	203.6721	19580.11	203.9544
203.9312	19591.67	204.4693	19593	203.9296	19596.63	203.9263	19590.53	203.9354	19595.06	203.9257	19583.66	204.2077
204.1798	19592.06	204.7078	19594.96	204.1881	19593.93	204.1742	19592.73	204.1775	19596.76	204.1761	19582.54	204.4541
204.4289	19592.7	204.9608	19594.54	204.4294	19596.28	204.4305	19597.09	204.434	19595.58	204.4228	19585.35	204.7061
204.6794	19593.15	205.208	19593.02	204.6876	19594.18	204.6754	19592.85	204.6809	19594.01	204.6738	19584.84	204.953
204.9297	19594.69	205.4695	19592.31	204.9326	19596	204.9253	19594.9	204.9346	19590.04	204.9293	19582.32	205.21
205.1797	19596.95	205.709	19597.76	205.185	19596.44	205.1731	19592.83	205.1795	19593.06	205.1779	19583.79	205.4551
205.4303	19590.67	205.9624	19593.79	205.4291	19592.63	205.4327	19594.99	205.4347	19594.65	205.4234	19584.59	205.7058
205.6808	19592.69	206.215	19594.19	205.6895	19597.25	205.6758	19595.39	205.6822	19595.25	205.6737	19585.86	205.9534
205.9313	19591.34	206.4709	19591.99	205.9305	19592.81	205.9279	19594.11	205.9366	19593	205.9262	19588.08	206.2124
206.1815	19590.01	206.7105	19593.34	206.1847	19593.77	206.1752	19592.9	206.1816	19595.24	206.1767	19587.08	206.4519
206.4326	19597.62	206.9639	19594.1	206.4301	19593.82	206.4305	19593.19	206.4339	19593.72	206.4274	19585.83	206.7068
206.683	19593.77	207.2109	19594.88	206.6894	19596.82	206.6746	19590.93	206.6802	19594.09	206.674	19581.49	206.9553
206.9306	19591.34	207.4698	19594.26	206.9304	19596.81	206.9264	19593.88	206.9358	19593.23	206.9268	19588.32	207.2094
207.179	19595.09	207.7084	19596.67	207.1834	19593.71	207.1765	19596.45	207.1815	19595.95	207.1779	19586.5	207.4551
207.4305	19596.09	207.9608	19595.42	207.4315	19592.51	207.4296	19598.32	207.436	19591.54	207.4258	19587.37	207.7061
207.6808	19593.57	208.2108	19593.37	207.6895	19594.12	207.6817	19596.06	207.6829	19598.98	207.6751	19579.31	207.955
207.932	19594.24	208.4748	19592.73	207.9331	19592.99	207.9378	19593.79	207.936	19593.26	207.9265	19593.13	208.2123
208.1786	19593.4	208.7229	19595.63	208.1865	19595.26	208.1799	19593.13	208.1804	19593.11	208.1791	19582.86	208.4542
208.4303	19594.67	208.9626	19591.36	208.4322	19596.56	208.4319	19592.17	208.437	19596.94	208.4254	19582.55	208.7051
208.682	19594.81	209.2089	19595.57	208.6909	19595.57	208.6773	19594.19	208.6812	19595.82	208.6751	19590.74	208.956

208.9344	19593.81	209.4677	19595.56	208.9324	19593.15	208.9263	19591.48	208.9365	19593.92	208.9276	19582.75	209.2098
209.1823	19595.55	209.7101	19590.63	209.1859	19599.55	209.1754	19590.96	209.1799	19595.48	209.178	19586.56	209.4575
209.4336	19595.69	209.964	19593.5	209.4304	19591.83	209.4324	19590.7	209.4392	19596.75	209.4266	19581.81	209.7063
209.6816	19595.28	210.2099	19599.69	209.6908	19592.55	209.6788	19590.9	209.6812	19595.9	209.6745	19583.87	209.9543
209.9344	19594.72	210.486	19596	209.9347	19593.46	209.927	19592.1	209.9354	19593.93	209.9269	19585.32	210.2039
210.1817	19591.39	210.7106	19595.79	210.1855	19591.46	210.1791	19593.28	210.1809	19592.83	210.179	19586.99	210.4539
210.435	19589.32	210.9637	19591.5	210.4319	19592.56	210.4333	19593.86	210.4369	19593.34	210.4261	19589.51	210.7077
210.6807	19594.83	211.2165	19597.45	210.6929	19597.4	210.6777	19595.75	210.6842	19590.51	210.6758	19588.93	210.9574
210.9342	19593.2	211.4739	19591.35	210.9329	19593.99	210.9278	19589.58	210.9363	19597.82	210.9264	19579.03	211.2107
211.1834	19594.43	211.7113	19591.9	211.1858	19592.61	211.1775	19591.11	211.1816	19593.76	211.1791	19585.11	211.4551
211.4342	19593.47	211.9642	19588.77	211.4309	19592.76	211.4323	19594.99	211.4405	19596.75	211.428	19586.17	211.7057
211.6865	19589.76	212.2139	19596.1	211.6931	19594.9	211.6791	19589.78	211.6824	19594.89	211.6764	19587.15	211.9575
211.9329	19592.77	212.4745	19594.43	211.9312	19590.62	211.928	19594.66	211.9384	19593.65	211.9267	19595.17	212.212
212.1827	19595.99	212.7136	19593.45	212.1862	19590.61	212.1818	19590.24	212.1823	19591.84	212.1788	19587.01	212.4561
212.435	19588.26	212.9643	19590.03	212.4342	19591.29	212.4329	19596.23	212.4372	19596.64	212.4263	19584.45	212.7088
212.6836	19594.39	213.2114	19592.57	212.6923	19594.46	212.6825	19590.27	212.6826	19592.4	212.6755	19582.05	212.9583
212.9334	19593.6	213.4871	19594.53	212.9324	19590.94	212.9286	19593.23	212.9374	19591.73	212.9271	19579.62	213.2439
213.1839	19590.1	213.7168	19594.66	213.1875	19589.34	213.181	19591.51	213.183	19592.69	213.1795	19584.03	213.7119
213.4396	19591.81	213.9642	19590.6	213.4347	19591.29	213.4348	19594.41	213.4419	19595.98	213.4271	19579.89	213.961
213.685	19592.39	214.2128	19596.14	213.6931	19597.58	213.6789	19591.92	213.7008	19590.9	213.6796	19583.02	214.2139
213.9356	19590.91	214.4737	19592.99	213.9331	19599.28	213.9292	19592.93	213.9387	19597.65	213.9274	19587.55	214.4603
214.1842	19591.62	214.7149	19593.91	214.1875	19591.99	214.1801	19597.39	214.1825	19595.61	214.1776	19576.67	214.7139
214.4357	19594.63	214.965	19591.03	214.4342	19594.51	214.4329	19591.06	214.4384	19595.68	214.4299	19583.67	214.9607
214.6851	19593.54	215.2118	19593.26	214.6937	19589.61	214.6781	19593.82	214.6838	19591.71	214.6761	19584.06	215.2187
214.936	19596.04	215.4717	19592.29	214.9357	19591.19	214.9271	19592.74	214.9385	19595.29	214.9291	19585.38	215.4681
215.1889	19595.19	215.713	19589.98	215.1882	19591.59	215.1794	19592.04	215.1885	19591.76	215.1802	19584.72	215.7151
215.4362	19592.07	215.9672	19592.73	215.4337	19592.49	215.4546	19593.16	215.4413	19591.09	215.4306	19583.05	215.9548
215.6847	19595.91	216.2125	19590.79	215.693	19592.46	215.683	19594.44	215.6883	19596.42	215.6772	19589.55	216.2056
215.9357	19591.86	216.4724	19589.89	215.9349	19596.19	215.9282	19596.03	215.9397	19594.16	215.9292	19580.56	216.4546
216.1859	19594.14	216.7135	19592.87	216.1893	19593.04	216.1794	19590.44	216.1879	19594.57	216.1834	19585.73	216.7054
216.4375	19594.3	216.9668	19594.86	216.4338	19590.47	216.4344	19591.56	216.4403	19593.77	216.4324	19577.79	216.9558
216.6849	19592.33	217.2152	19595.74	216.696	19590.93	216.6821	19595.18	216.6841	19595.87	216.6773	19583.12	217.2178
216.9354	19594.91	217.4734	19591.95	216.9357	19594.57	216.9373	19596.19	216.9398	19592.89	216.9295	19583.31	217.4606
217.1941	19591.99	217.7145	19591.21	217.1907	19594.27	217.182	19593.14	217.1832	19593.28	217.1806	19584.5	217.7083
217.441	19591	217.9687	19594.14	217.4352	19589.26	217.4345	19590.86	217.4455	19593.93	217.4309	19577.15	217.9583
217.6839	19595.09	218.2142	19596.33	217.697	19596.59	217.6853	19592.01	217.6852	19592.67	217.6772	19586.62	218.2167
217.9367	19591.72	218.4752	19593.89	217.9344	19592.67	217.9303	19590.73	217.9389	19593.75	217.9308	19585.01	218.4637
218.1875	19595.01	218.7148	19592.44	218.1916	19592.86	218.1814	19589.66	218.1856	19595.57	218.1814	19576.25	218.7118
218.4377	19588.34	218.9659	19591.7	218.4366	19595.6	218.445	19592.13	218.4533	19590.84	218.4312	19581.06	218.9626
218.6847	19595.67	219.2143	19591.31	218.6944	19594.86	218.6901	19593.29	218.684	19596.38	218.6794	19582.58	219.2087
218.9352	19592.67	219.4775	19589.51	218.9378	19594.55	218.9366	19592.37	218.9407	19592.89	218.9307	19581.21	219.4547
219.1863	19592.49	219.7239	19594.54	219.1907	19593.84	219.1822	19594.85	219.1884	19596.44	219.1831	19580.88	219.7075
219.4397	19592.15	219.9682	19591.44	219.4372	19590.83	219.4334	19593.67	219.4418	19595.34	219.433	19580.11	219.9581
219.6856	19590.17	220.2165	19594.74	219.6939	19591.63	219.6841	19591.47	219.6862	19592.88	219.6805	19585.45	220.2076
219.9347	19589.65	220.4746	19595.94	219.9377	19593.88	219.9336	19596.3	219.94	19592.08	219.9294	19583.99	220.4531
220.1852	19593.41	220.7174	19590.53	220.1917	19592.07	220.1852	19592.95	220.1871	19597.94	220.183	19584	220.7068
220.4398	19585.04	220.9677	19594.78	220.4374	19586.93	220.4412	19595.42	220.4448	19594.16	220.4316	19581.95	220.9587
220.6867	19596.31	221.2145	19596.91	220.6971	19592.73	220.685	19594.85	220.6887	19591.51	220.6814	19585.37	221.2088
220.9382	19596.67	221.4762	19589.49	220.9393	19593.45	220.9339	19593.02	220.9409	19594.67	220.9351	19578.79	221.4549
221.1856	19590.56	221.7183	19595.73	221.1913	19594.92	221.1821	19596.83	221.1873	19594.75	221.1833	19581.94	221.7079
221.4397	19593.84	221.9686	19594.8	221.4387	19593.05	221.4436	19589.76	221.4434	19596.79	221.4318	19583.31	221.9564
221.6873	19592.03	222.2157	19594.32	221.7166	19593.82	221.6857	19595.36	221.695	19595.48	221.6827	19583.82	222.2092
221.9396	19596.04	222.4752	19594.73	221.9445	19592.04	221.9327	19591.99	221.9421	19598.3	221.9313	19586.94	222.4551
222.187	19595.13	222.7176	19593.58	222.1901	19594.6	222.1838	19591.8	222.1869	19590.94	222.1858	19581.31	222.7085
222.4426	19595.93	222.9679	19593.98	222.4365	19592.66	222.4384	19591.2	222.4559	19594.31	222.4377	19588.87	222.9547
222.6872	19594.08	223.217	19593.07	222.6954	19594.38	222.6842	19591.57	222.6909	19592.69	222.682	19581.57	223.208
222.9394	19594.88	223.4746	19597.47	222.936	19589.86	222.9329	19594.76	222.9437	19591.2	222.9357	19584.87	223.4555
223.1913	19597.12	223.7176	19589.59	223.1919	19590.45	223.1845	19591.67	223.1879	19596.34	223.184	19580.68	223.7101
223.4408	19590.71	223.9685	19592.82	223.4392	19592.75	223.4365	19592.04	223.4446	19596.14	223.4333	19581.41	223.957
223.6887	19591.8	224.2209	19589.68	223.699	19593.16	223.6823	19590.06	223.6926	19591.93	223.685	19580.97	224.2075
223.9408	19591.91	224.476	19592.98	223.9406	19590.59	223.9332	19594.85	223.9418	19591.41	223.9305	19581.04	224.4559
224.1885	19595.41	224.719	19594.28	224.1912	19591.96	224.184	19594.15	224.1889	19592.92	224.1838	19580.18	224.7108
224.4384	19592.99	224.9692	19599.58	224.4402	19592.38	224.4389	19593.57	224.4456	19595.43	224.4335	19576.53	224.9574
224.6884	19598.5	225.2173	19590.96	224.6963	19592.4	224.6837	19592.51	224.6885	19586.04	224.6841	19589.51	225.2087
224.9386	19588.49	225.4769	19592.71	224.94	19592.39	224.9346	19590.81	224.9426	19591.9	224.9326	19584.06	225.4554
225.1877	19590.17	225.7179	19592.7	225.1929	19594.41	225.1861	19589.93	225.1887	19596.51	225.1847	19585.41	225.7098
225.4422	19593.02	225.9694	19588.59	225.4383	19592.59	225.4405	19594.41	225.4439	19595.19	225.4323	19583.64	225.9579
225.6886	19592.17	226.2173	19592.89	225.6987	19595.06	225.6833	19592.14	225.6908	19593.59	225.6838	19583.66	226.2105
225.9386	19591.87	226.477	19596.92	225.9397	19593.93	225.9343	19590.86	225.9425	19596.49	225.9336	19577.23	226.4568
226.1909	19591.7	226.7184	19590.74	226.1947	19592.71	226.1856	19592.39	226.1899	19596.48	226.1844	19583.27	226.7093

226.4419	19588.97	226.9714	19590.84	226.439	19594.76	226.4401	19589.75	226.4441	19590.99	226.4353	19579.87	226.96
226.6896	19592.85	227.2161	19590.95	226.6984	19591.63	226.6836	19592.12	226.6877	19591.68	226.6816	19582.6	227.2109
226.9411	19590.91	227.4764	19590.67	226.9392	19592.77	226.9359	19592.77	226.9439	19589.68	226.9329	19578.25	227.4575
227.1899	19593.7	227.7208	19593.67	227.1918	19590.59	227.1874	19591.9	227.1914	19591.4	227.1883	19583.73	227.7115
227.4395	19591.68	227.9715	19594.21	227.4408	19589.47	227.4428	19591.33	227.4457	19592.06	227.4321	19580.78	227.9576
227.6877	19592.35	228.2188	19591.45	227.6991	19593.61	227.6842	19593.56	227.6913	19591.52	227.6833	19580.49	228.2133
227.9438	19591.98	228.4781	19590.75	227.94	19594.23	227.934	19592.96	227.9439	19595.71	227.9364	19583.15	228.459
228.1915	19593.29	228.721	19589.97	228.1932	19593.55	228.1859	19592.38	228.1894	19595.5	228.1871	19579.44	228.7139
228.4449	19590.43	228.9718	19593.36	228.44	19584.14	228.4452	19595.67	228.4534	19594.76	228.4337	19584.3	228.9591
228.6896	19593.99	229.2201	19593.83	228.6994	19589.62	228.6858	19594.57	228.6868	19594.96	228.6826	19578.31	229.2083
228.9412	19591.75	229.4781	19593.88	228.9378	19590.34	228.9371	19593.49	228.9445	19593.34	228.9382	19582.44	229.4646
229.1919	19592.02	229.7193	19592.28	229.1941	19595.51	229.1875	19590.86	229.189	19593.83	229.1907	19583.22	229.7117
229.4447	19593.45	229.9725	19592.25	229.4398	19591.44	229.442	19594.59	229.447	19591.21	229.436	19579.16	229.96
229.6902	19591.65	230.22	19593.84	229.6995	19590.51	229.6877	19594.02	229.6898	19591.82	229.6892	19582.32	230.2121
229.9448	19589.98	230.4973	19594.21	229.9421	19591.79	229.936	19598.01	229.9434	19593.46	229.9343	19583.18	230.4601
230.1897	19591.4	230.732	19592.21	230.1958	19590.31	230.1907	19591.03	230.1914	19589.07	230.1879	19582.72	230.7193
230.4434	19592.5	230.9685	19593.1	230.4476	19588.94	230.4414	19596.49	230.4471	19594.6	230.4345	19587.59	230.9662
230.6902	19593.93	231.2203	19591.72	230.6991	19592.96	230.6885	19596.45	230.692	19591.73	230.685	19585.76	231.2141
230.9417	19590.92	231.4774	19592.26	230.9417	19592.57	230.9356	19590.67	230.9466	19591.57	230.9367	19583.63	231.4595
231.1912	19592.2	231.7215	19592.53	231.1958	19593.01	231.1843	19591.87	231.1903	19593.8	231.1848	19584.12	231.7229
231.4418	19593.21	231.9741	19594.01	231.4414	19588.41	231.4383	19590.58	231.4466	19594.57	231.4368	19581.9	231.9605
231.6922	19592.71	232.2202	19593.64	231.7008	19592.61	231.6881	19594.61	231.6917	19593.96	231.6844	19580.88	232.2173
231.9438	19593.57	232.4802	19592.03	231.9365	19592.39	231.9355	19594.04	231.9453	19593.18	231.9363	19579.92	232.4622
232.1916	19590.05	232.7191	19594.79	232.1957	19592.34	232.1857	19592.83	232.1914	19596.72	232.1869	19579.56	232.714
232.4421	19588.95	232.9743	19590.45	232.4424	19591.54	232.4398	19591.24	232.447	19593.43	232.4381	19586.97	232.9647
232.6915	19591.97	233.2237	19591.66	232.7021	19593.95	232.6869	19592.79	232.6925	19598.26	232.6855	19584.24	233.2145
232.944	19591.07	233.4775	19592.1	232.9402	19594.35	232.9357	19592.76	232.9457	19589.12	232.9359	19580.77	233.4728
233.1924	19593.22	233.719	19596.01	233.1969	19593.53	233.1881	19592.53	233.1926	19598.82	233.1899	19585.81	233.7153
233.4462	19588.84	233.9738	19593.04	233.4419	19590.24	233.445	19592.61	233.4507	19595.34	233.4348	19580.55	233.9638
233.6914	19593.89	234.2216	19593.26	233.7006	19594	233.689	19593.48	233.6955	19595.3	233.6836	19584.55	234.2184
233.9432	19591.65	234.4801	19590.8	233.9382	19595.44	233.9369	19591.52	233.9542	19593	233.9372	19586.11	234.4668
234.1928	19593.45	234.7175	19589.81	234.1961	19590.87	234.1863	19587.23	234.1994	19591.45	234.1898	19578.71	234.7142
234.4428	19594.87	234.9728	19590.1	234.4383	19590.84	234.4458	19592.87	234.4489	19594.42	234.442	19585.49	234.9639
234.6928	19588.82	235.2209	19591.63	234.7022	19595.99	234.688	19588.31	234.6906	19594.06	234.6849	19580.77	235.2176
234.9446	19589.67	235.4792	19593.17	234.9432	19594.88	234.9376	19593.17	234.952	19594.04	234.9366	19584.81	235.4644
235.1947	19594.42	235.7169	19588.26	235.1981	19593.55	235.1861	19591.5	235.1915	19591.15	235.1931	19582.33	235.7175
235.4481	19588.86	235.9759	19591.22	235.4429	19589.27	235.4451	19595.9	235.4635	19595.66	235.4365	19584.54	235.9605
235.6883	19589.61	236.2221	19592.54	235.7022	19589.11	235.6891	19591.41	235.7064	19589.87	235.6859	19575.08	236.2177
235.9455	19592.96	236.4821	19593.2	235.9422	19593.7	235.9393	19595.2	235.9518	19591.47	235.9359	19578.11	236.4638
236.1973	19593.34	236.7222	19590	236.1979	19594.91	236.1881	19592.47	236.1912	19594.25	236.191	19582.32	236.7155
236.447	19588.43	236.9758	19593.41	236.4418	19594.35	236.4462	19592.57	236.4518	19590.1	236.4391	19581.9	236.9637
236.6935	19587.77	237.2216	19596.63	236.7036	19592.06	236.6883	19593.19	236.6937	19590.25	236.6877	19579.12	237.2169
236.947	19591.32	237.4779	19590.65	236.9432	19592.8	236.9406	19591.1	236.9468	19593.63	236.9389	19577.52	237.4658
237.1986	19595.81	237.7206	19597.95	237.1984	19592.8	237.1884	19592.73	237.1926	19591.67	237.19	19582.8	237.7158
237.4418	19592.25	237.9761	19596.63	237.4394	19595.13	237.4462	19592.48	237.4499	19597.65	237.4397	19579.61	237.9637
237.6949	19591.69	238.2233	19592.18	237.7031	19589.34	237.6904	19588.83	237.691	19595.41	237.6883	19583.14	238.2144
237.9604	19592.67	238.4831	19588.69	237.9452	19593.92	237.9381	19592.71	237.947	19598.24	237.9372	19580.63	238.4607
238.22	19591.87	238.723	19595.13	238.1971	19593.58	238.1903	19595.06	238.1921	19595.57	238.1917	19582.91	238.7174
238.4634	19589.75	238.9766	19591.92	238.4448	19590.83	238.4496	19591.69	238.4524	19591.99	238.44	19581.59	238.9623
238.7048	19592.37	239.2186	19590.39	238.7045	19595.76	238.6908	19594.62	238.6949	19591.14	238.6902	19580.21	239.2208
238.9568	19591.56	239.4961	19588.96	238.9513	19593.89	238.95	19594.16	238.9593	19593.48	238.9487	19581.24	239.4747
239.2071	19593.19	239.7378	19592.49	239.1968	19592.75	239.1874	19595.6	239.2017	19597.6	239.1926	19586.73	239.7252
239.4532	19590.82	239.9819	19595.13	239.4441	19591.7	239.4485	19590.98	239.4506	19586.47	239.4381	19577.73	239.9784
239.6958	19593.64	240.229	19591.59	239.7044	19593.71	239.6889	19591.09	239.6927	19597.05	239.6891	19578.42	240.2269
239.9459	19590.1	240.4789	19593.36	239.9435	19591.9	239.9404	19593.27	239.9491	19590.13	239.94	19581.74	240.4719
240.198	19592.1	240.7163	19596.69	240.1992	19591.73	240.1884	19594.01	240.1911	19595.03	240.1907	19577.52	240.7181
240.4433	19595.29	240.9708	19593.96	240.4458	19595.66	240.446	19593.8	240.4524	19591.87	240.441	19586.62	240.9609
240.692	19589.67	241.2185	19590.9	240.7055	19589.64	240.6847	19590.41	240.6965	19592.09	240.6848	19579.28	241.2151
240.9421	19593.38	241.4786	19592.46	240.948	19592.47	240.9353	19591.96	240.9444	19591.14	240.9514	19584.62	241.4621
241.1922	19592.81	241.7186	19590.74	241.194	19591.4	241.1853	19597.31	241.1913	19593.67	241.1871	19583.83	241.7139
241.4435	19593.79	241.974	19593.23	241.4406	19595.18	241.4624	19592.54	241.4621	19595.89	241.4418	19580.59	241.9602
241.6914	19593.62	242.2201	19588.88	241.7175	19593.62	241.6992	19588.28	241.6971	19593.71	241.686	19586.09	242.2136
241.9427	19593.52	242.4819	19594.61	241.9409	19592.66	241.9374	19592.14	241.9455	19593.44	241.9382	19586.59	242.462
242.1909	19590.67	242.7218	19591.07	242.1945	19591.36	242.1855	19595.23	242.1904	19595.01	242.1868	19582.75	242.719
242.4432	19592.22	242.979	19589.84	242.4407	19596.27	242.4436	19598.44	242.4483	19588.87	242.436	19582.34	242.9663
242.6906	19589.43	243.2204	19592.91	242.7046	19591.81	242.6877	19592.69	242.6921	19596.02	242.6859	19582.65	243.2159
242.9435	19594.01	243.4786	19593.16	242.9421	19594.07	242.9411	19593.19	242.9473	19596.12	242.9361	19576.7	243.4629
243.1925	19593.07	243.7197	19595.65	243.1954	19593.01	243.1856	19595.22	243.1915	19591.51	243.1864	19578.28	243.7184
243.4413	19592.24	243.9744	19593.67	243.4429	19594.56	243.4436	19595.3	243.4507	19592.62	243.4381	19583.56	243.9683
243.6959	19592.34	244.2222	19594.82	243.7005	19593.53	243.6863	19591.32	243.691	19593.09	243.6852	19583.62	244.2139

243.9426	19588.13	244.4805	19589.42	243.9436	19595.87	243.9358	19591.74	243.946	19594.48	243.937	19582.25	244.4641
244.1941	19592.3	244.7206	19592.35	244.196	19589.02	244.1878	19591.16	244.1917	19586.39	244.1888	19578.68	244.7158
244.4421	19592.55	244.9745	19592.88	244.4428	19590.58	244.4445	19591.96	244.4637	19596.55	244.4376	19581.31	244.965
244.6919	19591.79	245.2215	19592.44	244.6987	19591.03	244.6877	19589.48	244.6998	19591.1	244.6867	19582.37	245.2175
244.9494	19592.04	245.4788	19592.43	244.9448	19594.42	244.9377	19589.16	244.9458	19594.3	244.9362	19580.18	245.4631
245.1943	19588.56	245.7242	19592.48	245.1951	19595.75	245.1881	19588.55	245.1917	19593.09	245.1875	19583.28	245.7197
245.4427	19594.69	245.9754	19587.78	245.4411	19594.66	245.4462	19594.54	245.4616	19592.4	245.4408	19579.54	245.9632
245.6935	19594.55	246.2246	19593.33	245.6995	19587.22	245.6877	19597.44	245.6998	19596.49	245.6861	19579.35	246.2175
245.9436	19588.45	246.4788	19588.56	245.9434	19593.46	245.9389	19591.89	245.9471	19595.28	245.9382	19585.1	246.4616
246.1949	19591.12	246.7222	19595.29	246.1973	19595.12	246.1861	19593.9	246.1918	19594.68	246.1956	19585.33	246.7171
246.4456	19589.98	246.9766	19591.37	246.4429	19595.05	246.4446	19597.29	246.4496	19592.53	246.4468	19581.55	246.966
246.6949	19587.69	247.2197	19592.41	246.7258	19591.32	246.6889	19593.85	246.6929	19593.81	246.6869	19584.36	247.2225
246.9428	19592.9	247.4815	19591.28	247.201	19592.57	246.9399	19595.95	246.9465	19596.84	246.9374	19580.3	247.462
247.1956	19594.54	247.722	19593.6	247.4482	19593.14	247.1906	19590.51	247.1932	19592.3	247.1895	19583.66	247.7165
247.4462	19593.43	247.9766	19592.64	247.706	19593.93	247.4468	19597.31	247.4513	19592.93	247.4406	19580.67	247.9679
247.6943	19588.31	248.2222	19589.53	247.9478	19591.17	247.6893	19590.63	247.6942	19592.6	247.6885	19581.85	248.2199
247.9473	19591.44	248.4783	19592.18	248.2008	19588.22	247.9377	19590.37	247.9481	19598.95	247.9388	19578.03	248.46
248.1954	19595.1	248.7182	19594.14	248.4481	19594.09	248.1905	19592.1	248.1928	19592.1	248.1943	19583.17	248.7202
248.442	19590.31	248.9747	19590.11	248.7064	19591.55	248.4465	19595.26	248.4506	19594.18	248.4412	19591.43	248.9616
248.6937	19591.47	249.2219	19591.7	248.95	19599.27	248.6878	19590.48	248.6932	19592.21	248.6867	19579.99	249.2156
248.9443	19594.34	249.4776	19592.24	249.2046	19595	248.9382	19589.66	248.948	19591.24	248.9412	19582.61	249.4657
249.1993	19596.27	249.7215	19595.87	249.4477	19588.93	249.1885	19594.41	249.1936	19595.26	249.1908	19582.55	249.7174
249.4451	19591.14	249.9768	19594.74	249.7052	19589.15	249.445	19590.52	249.4511	19592.16	249.4401	19579.97	249.9648
249.693	19594.95	250.2264	19589.16	249.953	19591.25	249.6916	19589.39	249.6927	19590.74	249.6881	19583.22	250.2164
249.9484	19590.57	250.4906	19589.43	250.2037	19594.44	249.9389	19594.37	249.9484	19590.23	249.9385	19583.92	250.4648
250.1965	19590.56	250.7304	19590.7	250.4488	19592.1	250.1898	19592.29	250.1934	19593.27	250.1889	19576.74	250.7226
250.4475	19589.47	250.975	19591.37	250.7068	19593.17	250.4458	19590.65	250.4518	19593.88	250.4499	19579.77	250.9757
250.6956	19592.18	251.2246	19593.39	250.9522	19586.68	250.6917	19590.95	250.6942	19593.15	250.689	19580.27	251.2312
250.9464	19590.29	251.4802	19591.01	251.2054	19589.83	250.9434	19593.29	250.9485	19593.03	250.9387	19581.19	251.4669
251.1951	19594.25	251.7253	19588.89	251.4502	19591.68	251.1893	19591.99	251.1944	19595.2	251.1888	19587.44	251.7163
251.4506	19590.67	251.9764	19591.22	251.7125	19590.82	251.4474	19591.73	251.4546	19594.76	251.4415	19581.69	251.9718
251.6955	19591.4	252.2255	19590.83	251.9509	19594.27	251.6893	19593.53	251.6972	19594.44	251.6912	19581.27	252.2251
251.9489	19595.31	252.4815	19594.07	252.2041	19592.08	251.9397	19592.84	251.9494	19586.82	251.9398	19581.02	252.4737
252.1982	19593.49	252.7257	19590.45	252.4492	19594.52	252.1904	19591.94	252.193	19593.78	252.1908	19579.72	252.7251
252.4484	19592.38	252.9768	19592.89	252.7093	19595.17	252.4447	19590.42	252.4537	19594.56	252.441	19578.36	252.9778
252.6971	19591.84	253.2235	19593.26	252.948	19588.74	252.6905	19590.09	252.6961	19592.05	252.6907	19582.53	253.2241
252.9483	19593.37	253.4849	19593.68	253.2023	19590.4	252.9402	19598.06	252.9497	19591.04	252.9392	19581.58	253.4854
253.2006	19589.98	253.7288	19592.22	253.4494	19593.16	253.1902	19592.54	253.1961	19592.52	253.1926	19580.67	253.7321
253.445	19588.78	253.9768	19589.22	253.7112	19591.21	253.4462	19589.57	253.4552	19597.17	253.445	19579.47	253.9742
253.6948	19588.13	254.2236	19591.88	253.9504	19590.9	253.6959	19592.49	253.6966	19594.6	253.6914	19581.84	254.2292
253.9489	19594.63	254.4826	19594.24	254.2025	19592.28	253.9406	19595.02	253.9523	19591.94	253.9402	19583.61	254.4722
254.2013	19591.46	254.7242	19589.91	254.4502	19593.39	254.1907	19592.41	254.1945	19595.81	254.1929	19576.49	254.7268
254.4466	19588.48	254.9781	19594.71	254.7142	19590.28	254.4517	19589.19	254.4556	19594.46	254.444	19578.95	254.9687
254.7012	19587.22	255.2254	19590.72	254.9567	19592.38	254.6937	19588.77	254.7027	19591.34	254.6945	19584	255.2239
254.9492	19587.8	255.4831	19590.86	255.1993	19591.45	254.9471	19590.6	254.951	19592.85	254.9412	19584.16	255.4658
255.2042	19593.72	255.7239	19593.19	255.4504	19596.85	255.1913	19590.55	255.1957	19593.23	255.1923	19579.77	255.7176
255.4502	19589.56	255.978	19595.63	255.7129	19592.24	255.4458	19588.68	255.4539	19590.01	255.4403	19586.25	255.9661
255.6985	19592.16	256.2285	19590.8	255.9546	19592.35	255.6956	19585.83	255.6979	19589.61	255.7102	19578.31	256.2185
255.9498	19594.32	256.4853	19593.5	256.2045	19593.21	255.9408	19592.69	255.951	19592.1	255.9534	19575.4	256.4695
256.1988	19593.54	256.7266	19593.45	256.4505	19592.44	256.1918	19590.33	256.1965	19591.7	256.1963	19581.59	256.7199
256.4488	19592.79	256.9771	19592.18	256.7086	19595.58	256.4474	19590.73	256.4562	19589.94	256.4431	19583.53	256.9717
256.6965	19592.54	257.2256	19594.94	256.9526	19587.63	256.6926	19591.02	256.6975	19588.49	256.6954	19576.34	257.2192
256.9476	19589.3	257.4834	19595.54	257.2058	19591.05	256.9448	19592.52	256.9512	19592.43	256.9424	19575.49	257.4662
257.2079	19596.01	257.7279	19593.15	257.4461	19591.86	257.1965	19591.9	257.1977	19590.77	257.1927	19582.08	257.7182
257.4504	19589.92	257.9795	19594.69	257.7118	19589.9	257.4497	19589.97	257.4545	19594.55	257.4419	19578.4	257.9692
257.6995	19590.65	258.2272	19591.93	257.9553	19591.96	257.6935	19594.53	257.6988	19589.06	257.6933	19579.7	258.2171
257.9503	19592.86	258.4823	19591.01	258.2025	19593.56	257.944	19588.86	257.9515	19595.6	257.9423	19583.27	258.4674
258.1998	19593.37	258.7262	19593.65	258.4539	19592.92	258.1912	19589.69	258.1969	19592.39	258.1965	19578.62	258.7173
258.4493	19590.52	258.9809	19589.61	258.7101	19594.05	258.4479	19591.53	258.4562	19590.32	258.4453	19583.58	258.9676
258.6975	19587.1	259.2277	19591.53	258.9535	19593.34	258.6967	19592.36	258.6991	19589.23	258.6987	19584.61	259.2189
258.9524	19591.22	259.4851	19591.17	259.2049	19591.34	258.9439	19587.92	258.9524	19590.99	258.9442	19580.75	259.4676
259.2023	19587.07	259.7251	19588.87	259.4481	19594.17	259.1929	19590.45	259.1981	19593.99	259.1966	19581.82	259.7206
259.4509	19592	259.9783	19593.05	259.711	19591.81	259.4479	19592.71	259.4545	19593.59	259.4437	19582.94	259.9677
259.6967	19592.68	260.2287	19590.37	259.9595	19591.28	259.6947	19588.98	259.699	19591.93	259.6923	19580.66	260.2196
259.9525	19594.71	260.4813	19593.15	260.2055	19595.68	259.9438	19594.37	259.9523	19591.25	259.9445	19578.43	260.4662
260.2025	19592.87	260.7276	19591.96	260.4486	19597.72	260.1938	19591.93	260.1974	19591.85	260.193	19579.1	260.7194
260.4492	19589.61	260.9788	19589.49	260.7111	19589.8	260.4504	19590.1	260.4584	19591.27	260.4467	19582.72	260.9705
260.6974	19590.33	261.2281	19592.14	260.955	19593.48	260.6953	19592.2	260.7008	19593.7	260.6939	19577.95	261.2205
260.9513	19591.14	261.4845	19593.88	261.2018	19592.04	260.9472	19590.38	260.9517	19591.19	260.9458	19575.06	261.4687
261.2012	19593.75	261.7292	19588.52	261.4525	19590.82	261.1941	19590.74	261.1983	19591.43	261.1947	19578.65	261.7207

261.4506	19590.13	261.9789	19594.81	261.7303	19590.16	261.4676	19594.07	261.4578	19592.09	261.4449	19579.96	261.9688
261.6997	19595.87	262.2279	19590.25	262.2152	19589.95	261.7134	19590.82	261.6995	19593.07	261.6923	19581.89	262.2209
261.9527	19589.16	262.4851	19595.41	262.4585	19590.84	261.9598	19591.63	261.9533	19593.92	261.9438	19577.04	262.4684
262.2022	19589.84	262.7301	19588.72	262.7195	19589.94	262.204	19595.49	262.1977	19589.93	262.1931	19581.81	262.7218
262.4495	19592.7	262.9774	19593.41	262.9664	19591.08	262.4561	19592.1	262.457	19598.65	262.4439	19584.36	262.9735
262.6979	19592.39	263.2291	19590.69	263.2157	19591.91	262.7024	19592.62	262.6977	19593.74	262.6937	19578.85	263.2226
262.9563	19593.27	263.4803	19589.28	263.4606	19591.17	262.9485	19592.93	262.9542	19595.7	262.945	19576.15	263.4753
263.2031	19591.53	263.7259	19593.8	263.7151	19592.38	263.1934	19593.18	263.2001	19594.08	263.1948	19581.37	263.7255
263.4504	19588.26	263.9801	19591.45	263.9633	19592.21	263.4509	19587.67	263.4518	19593.18	263.4431	19579.85	263.9723
263.6971	19591.92	264.2277	19590.97	264.2126	19591.69	263.6998	19592.26	263.6978	19590.89	263.695	19581.53	264.2223
263.956	19591.53	264.4858	19594.14	264.4594	19589.24	263.944	19589.32	263.9544	19594.68	263.9444	19583.07	264.4821
264.2064	19586.01	264.7298	19588.48	264.7183	19595.83	264.1994	19593.09	264.199	19589.61	264.1959	19579.11	264.7403
264.4544	19593.06	264.9792	19596.04	264.9724	19593.25	264.4508	19590.68	264.4579	19592.43	264.444	19578.21	265.0057
264.6992	19590.99	265.2256	19591.18	265.2224	19591.36	264.7021	19590.32	264.7103	19594.58	264.6939	19585.81	265.4955
264.9519	19590.31	265.481	19591.91	265.4703	19588.99	264.9494	19589.89	264.9619	19590.91	264.9456	19581.69	265.7534
265.2082	19587.85	265.7296	19596.48	265.7295	19591.19	265.1975	19589.35	265.2084	19589.19	265.1966	19579.34	266.0095
265.4585	19589.43	265.979	19592.22	265.9819	19592.13	265.4519	19592.17	265.4619	19590.77	265.4456	19582.16	266.5016
265.7074	19587.81	266.2283	19592.51	266.2334	19593.15	265.7038	19594.13	265.7105	19588.94	265.6972	19579.59	266.7543
265.9621	19588.21	266.4822	19591.56	266.7221	19594.81	265.9535	19591.1	265.9637	19595.22	265.9446	19579.22	267.0048
266.2112	19593.18	266.7282	19588.97	266.9702	19591.14	266.2015	19593.85	266.2107	19593.7	266.1971	19577.84	267.2601
266.4616	19594.56	266.9783	19592.48	267.2213	19587.86	266.4533	19594.09	266.4631	19590.74	266.4459	19578.24	267.5152
266.7094	19590.22	267.2248	19594.63	267.468	19591.25	266.7069	19590.28	266.713	19591.25	266.6972	19575.84	268.0004
266.967	19590.33	267.4866	19592.68	267.7259	19593.96	266.9552	19589.59	266.965	19592.79	266.946	19579.85	268.2625
267.2175	19592.65	267.7359	19591.05	267.9754	19593.31	267.2053	19590.34	267.2118	19595.53	267.1991	19576.82	268.5195
267.4669	19594.27	267.9917	19590.77	268.2261	19589.45	267.4587	19594.34	267.479	19593.86	267.4463	19575.98	269.0159
267.7146	19592.64	268.2418	19594.34	268.4736	19592.91	267.7093	19591.3	267.9649	19590.51	267.6969	19579.3	269.2785
267.9699	19592.06	268.4975	19591.74	268.7299	19590.35	267.9595	19589.59	268.2128	19589.8	267.9466	19580.59	269.7638
268.2205	19592.49	268.7464	19592.88	268.9815	19592.68	268.2234	19594.04	268.4681	19595.85	268.2077	19586.4	270.0181
268.4717	19594.4	268.9975	19593.08	269.2336	19589.35	268.7069	19594.49	268.718	19587.89	268.4645	19578.86	270.2794
268.7214	19590.64	269.2489	19590.27	269.4826	19590.66	268.9553	19590.66	268.9709	19594.27	268.7158	19578.4	270.534
268.9733	19588.3	269.5042	19595.06	269.7386	19591.23	269.2086	19589.51	269.2214	19594.2	268.9687	19583.45	271.0185
269.4596	19588.41	269.9894	19592.39	270.225	19594.34	269.4632	19591.48	269.4765	19594	269.2217	19583.96	271.2752
269.7101	19590.6	270.2391	19591.14	270.476	19586.1	269.7145	19593.87	269.7271	19592.97	269.7016	19572.53	271.5415
269.9657	19588.86	270.5106	19592.64	270.7315	19591.49	269.9647	19590.62	269.9808	19593.87	269.952	19581.4	272.0278
270.2165	19591.77	270.9973	19592.19	270.9837	19590.78	270.2154	19591.13	270.2294	19589.65	270.2047	19583.27	272.2855
270.4698	19590.48	271.2491	19591.09	271.2384	19591.99	270.4716	19592	270.4844	19593.05	270.455	19580.86	272.541
270.7198	19590.33	271.509	19591.64	271.4871	19593.22	270.7226	19590.48	270.9724	19592.1	270.7065	19579.94	272.798
270.9732	19591.03	271.7581	19592.05	271.7443	19592.86	270.9758	19592.15	271.222	19590.46	270.9582	19578.41	273.293
271.2262	19590.08	272.0095	19593.56	272.2362	19595.61	271.2265	19593.87	271.4805	19592.77	271.2099	19581.49	273.5728
271.711	19594.84	272.2616	19591.44	272.4876	19589.34	271.4864	19592.63	271.7317	19589.97	271.4634	19579.25	274.0723
271.9678	19587.78	272.5181	19589.47	272.7455	19593.41	271.9703	19592.46	271.9861	19590.37	271.7137	19578.76	274.3385
272.2227	19591.66	273.0048	19593.55	272.9968	19588.55	272.2222	19585.97	272.2378	19587.83	271.9676	19576.45	274.8329
272.4763	19587.98	273.2565	19594.63	273.2503	19593.86	272.4803	19589.4	272.7265	19596.71	272.2206	19583.58	275.0922
272.7275	19593.48	273.5147	19589.77	273.5037	19591.98	272.7353	19593.22	272.9839	19589.72	272.4732	19579.75	275.3547
272.9814	19588.63	273.7808	19591.52	273.9884	19597.31	272.993	19588.39	273.2353	19591.54	272.7305	19578.97	275.8409
273.4721	19592.55	274.267	19591.35	274.2419	19591.55	273.4831	19589.1	273.493	19590.11	273.2169	19585.46	276.0991
273.7239	19591.19	274.5238	19592.25	274.4937	19590.93	273.7358	19593.15	273.9963	19591.78	273.4698	19580.25	276.3587
273.9795	19591.41	274.7783	19593.69	274.7517	19591.91	273.9897	19593.9	274.4981	19595.65	273.7217	19576.64	276.8436
274.2332	19591.04	275.0309	19591.28	275.0048	19591.93	274.2404	19593.3	274.7537	19593.31	273.9768	19579.13	277.1007
274.4885	19588.98	275.2841	19594.32	275.4938	19587.19	274.5007	19592.72	275.2389	19590.96	274.2319	19585.11	277.3603
274.9778	19591.23	275.5405	19590.91	275.76	19588.57	274.9843	19592.63	275.4964	19596.45	274.4872	19578.76	277.6219
275.2323	19587.78	276.0266	19591	276.0123	19593.88	275.2362	19593.25	275.7517	19592.92	274.7421	19577.48	278.1145
275.4887	19594.21	276.2799	19589.4	276.4991	19592.26	275.4964	19589.44	276.013	19595.59	275.2249	19576.05	278.3813
275.7404	19593.74	276.5369	19592.96	276.7608	19591.16	275.753	19591.77	276.503	19593.64	275.4802	19578.92	278.8707
275.9992	19590.94	276.7892	19592.83	277.0163	19594.42	276.0098	19588.98	276.7591	19592.73	275.7372	19574.05	279.1321
276.4881	19592.81	277.0451	19595.13	277.5031	19588.65	276.4988	19587.78	277.016	19591.03	275.9906	19579.87	279.393
276.7412	19591.18	277.5384	19590.03	277.7644	19588.64	276.7517	19590.01	277.5131	19590.21	276.2493	19578.03	279.8921
277.0023	19590.89	277.794	19589.97	278.0176	19590.02	277.0088	19591.41	277.7674	19593.84	276.7311	19575.92	280.1658
277.4915	19591.88	278.05	19588.21	278.2779	19591.95	277.2635	19593.3	278.0254	19593.6	276.987	19583.9	280.6635
277.7455	19596.85	278.5455	19593.18	278.7761	19592.05	277.7474	19589.16	278.5161	19592.62	277.2438	19582.6	280.93
278.0056	19597.09	278.7996	19592.26	279.0303	19591.47	278.0045	19593.81	278.771	19592.98	277.5003	19577.78	281.4301
278.4958	19593.13	279.0568	19593.14	279.5219	19593.71	278.2602	19592.38	279.0277	19594.22	277.7581	19575.68	281.6964
278.7495	19588.39	279.3132	19589.82	279.7831	19589	278.5226	19590.75	279.2852	19588.54	278.2431	19584.02	282.1959
279.0081	19591.12	279.8016	19590.07	280.0389	19590.41	279.0074	19588.83	279.7725	19593.01	278.4896	19582.88	282.4803
279.2647	19591.24	280.0599	19593.4	280.5296	19592.82	279.2609	19594.87	280.0311	19591.36	278.7423	19582.36	282.979
279.7523	19595.14	280.3196	19590.24	280.7915	19594.03	279.5208	19596.07	280.2865	19596.18	278.9914	19581.53	283.249
280.0108	19590.85	280.8081	19592.23	281.0481	19593.46	279.7835	19590.52	280.5595	19596.61	279.242	19579.32	283.7456
280.2698	19592.93	281.0638	19588.96	281.536	19593.18	280.2683	19593.21	281.0525	19588.58	279.4909	19575.97	284.0152
280.7626	19591.02	281.3223	19590.17	281.8133	19594.8	280.5295	19591.49	281.3165	19593.55	279.7444	19585.52	284.5137
281.0224	19589.35	281.8197	19593.75	282.3044	19589.91	280.7862	19591.6	281.8039	19592.66	279.991	19583.14	284.7795

281.2798	19589.16	282.0791	19591.47	282.5612	19591.64	281.047	19589.35	282.0624	19595.77	280.2417	19589.08	285.2801
281.7673	19589.63	282.5732	19591.51	282.8249	19592.5	281.5565	19587.9	282.3223	19590.19	280.4904	19577.38	285.5476
282.0296	19593.48	282.8338	19589.78	283.3199	19588.4	282.0437	19590.13	282.81	19593.16	280.7431	19578.91	286.041
282.288	19590.07	283.3279	19589.99	283.5753	19591.69	282.2992	19592.62	283.0683	19589.36	280.9926	19578.98	286.3146
282.7761	19589.63	283.589	19592.22	283.8399	19591.84	282.5662	19589.82	283.3296	19592.36	281.2446	19580.35	286.8148
283.0354	19588.58	284.0791	19590.06	284.3306	19593.25	283.0535	19591.29	283.8183	19592.39	281.4912	19582.76	287.0826
283.2948	19589.89	284.3451	19590.69	284.585	19588.64	283.2996	19590.71	284.0796	19591.65	281.7435	19578.75	287.5771
283.7837	19586.49	284.8374	19587.37	284.8495	19593.67	283.559	19592.26	284.3401	19593.28	281.9927	19580.85	287.8407
284.0541	19594.64	285.0939	19591.35	285.3389	19590.67	283.8101	19589.72	284.8282	19593.57	282.244	19585.74	288.3399
284.5501	19587.87	285.3568	19591.14	285.5994	19589.45	284.0583	19592.91	285.0886	19595.94	282.4929	19579.8	288.6109
284.8076	19590.68	285.8475	19589.64	285.8622	19591.64	284.3071	19588.52	285.3518	19592.33	282.7448	19586.39	289.1068
285.0729	19590.69	286.1083	19592.16	286.352	19593.56	284.5615	19588.9	285.8469	19595.42	282.9953	19577.05	289.3745
285.5665	19592.16	286.3713	19595.63	286.6112	19587.32	284.8086	19591.69	286.1079	19592.93	283.2457	19582.78	289.8687
285.8242	19592.9	286.8669	19589.07	286.8796	19588.35	285.0588	19591.97	286.373	19593.76	283.4929	19576.6	290.1476
286.0916	19591.36	287.1251	19591.23	287.3705	19582.99	285.3033	19590.02	286.8631	19587.83	283.7434	19578.56	290.649
286.5842	19591.78	287.6217	19592.57	287.6314	19589.89	285.5583	19587.26	287.1247	19590.53	283.9933	19580.69	290.9133
286.8429	19591.45	287.8866	19591.76	287.8945	19589.07	285.8064	19588.49	287.3926	19591.88	284.2445	19579.55	291.4397
287.1099	19590.34	288.3813	19589.46	288.3847	19595.68	286.0584	19591.15	287.8854	19590.29	284.4925	19576.92	291.9366
287.6043	19590.54	288.644	19590.27	288.6482	19590.72	286.3056	19589.56	288.1443	19589.53	284.7461	19579.29	292.2226
287.8631	19593.19	289.1357	19590.28	289.1422	19589.92	286.5599	19593.41	288.4099	19590.41	284.9944	19580.9	292.7248
288.3593	19586.67	289.3986	19590.39	289.4012	19594.14	286.8101	19588.9	288.9036	19592.11	285.2435	19578.58	292.996
288.6224	19591.26	289.8945	19594.99	289.8977	19591.38	287.0628	19591.34	289.1625	19596.5	285.4941	19579.43	293.4927
289.122	19592.55	290.1581	19593.32	290.1483	19590.73	287.3119	19593.45	289.4333	19591.98	285.7461	19580.17	293.7663
289.3792	19589.37	290.6531	19591.32	290.3964	19591.36	287.5699	19592.1	289.9272	19592.13	285.9936	19581.53	294.2701
289.8717	19589.97	290.9163	19593.6	290.6517	19591.4	288.0595	19593.13	290.1886	19595.23	286.2452	19583.67	294.7731
290.1374	19590.74	291.4123	19592.11	290.9035	19591.84	288.3097	19592.86	290.6805	19592.28	286.4936	19579.72	295.0415
290.6307	19592.8	291.6754	19590.05	291.1548	19589.03	288.5643	19589.17	290.9461	19595.54	286.7446	19586.53	295.5543
290.8924	19593.13	292.1682	19590.01	291.6494	19591.96	288.8127	19587.76	291.4466	19593.42	286.9972	19579.71	296.0559
291.3835	19591.23	292.4338	19587.71	291.9017	19591.27	289.0654	19592.04	291.7073	19594.02	287.249	19577.38	296.3343
291.6438	19592.24	292.9283	19589.23	292.1543	19590.09	289.3142	19592.37	292.1988	19594.97	287.4962	19585.65	296.8354
291.9064	19590.95	293.1885	19592.78	292.4046	19592.17	289.5692	19589.87	292.4689	19590.08	287.746	19580.02	297.1114
292.4014	19589.78	293.6866	19589.08	292.6569	19591.17	289.8206	19587.89	292.9648	19595.15	287.9968	19582.22	297.6179
292.6622	19589.26	293.9505	19586.95	292.9101	19594.05	290.0757	19590.56	293.2274	19594.92	288.2501	19580.65	298.1182
292.924	19591.13	294.4455	19590.49	293.4026	19590.63	290.5721	19589.38	293.7201	19587.88	288.5015	19581.27	298.3965
293.4179	19587.49	294.7131	19589.35	293.6563	19593.91	290.8237	19593.75	293.9863	19588.69	288.7531	19583.68	298.8995
293.6824	19589.63	295.2058	19591.77	293.9108	19592.79	291.078	19591.22	294.4882	19593.84	289.0033	19580.78	299.4074
294.1797	19589.07	295.4733	19592.47	294.407	19593.24	291.3276	19595.98	294.7506	19595.32	289.2546	19576.78	299.9082
294.4415	19592.03	295.9713	19587.92	294.6626	19592.18	291.5805	19590.88	295.2429	19591.05	289.5046	19582.73	300.181
294.9384	19592.72	296.2348	19593.62	294.918	19599.4	292.0725	19589.24	295.5128	19596.64	289.7705	19572.78	
295.2073	19590.35	296.728	19588.82	295.412	19592.64	292.3225	19591.05	296.0099	19591.08	290.2604	19577.92	
295.6992	19590.39	296.9912	19585.57	295.6667	19590.15	292.5803	19587.79	296.2718	19590.81	290.5229	19585.27	
295.9682	19589.03	297.4916	19591.71	295.9236	19592.45	292.8321	19590.22	296.7665	19591.46	290.7873	19582.16	
296.4685	19593.58	297.7576	19594.36	296.4192	19587.73	293.0866	19594.89	297.0332	19588.75	291.2775	19576.89	
296.7307	19586.99	298.2519	19594.1	296.6764	19585.04	293.583	19587.74	297.5328	19591.3	291.5411	19579.74	
297.2422	19591.03	298.523	19593.26	296.9303	19588.22	293.8345	19589.71	297.7963	19589.62	292.032	19580.12	
297.7362	19589.86	299.0192	19590.91	297.4278	19590.37	294.0915	19594.37	298.2916	19590.14	292.2991	19579.18	
298.0047	19586.76	299.2813	19591.48	297.6837	19590.11	294.5904	19590.54	298.5685	19588.94	292.7898	19581.46	
298.5086	19589.49	299.7749	19589.77	298.185	19590.11	294.8433	19590.22	299.0659	19590.34	293.0564	19578.09	
299.0108	19590.97	300.0399	19589.24	298.4387	19591.32	295.1011	19585.56	299.3351	19588.56	293.5449	19581.07	
299.2766	19587.02			298.6941	19590.76	295.5973	19591.04	299.8322	19585.51	293.8124	19580.79	
299.7712	19593.78			299.1921	19589.68	295.8635	19591.41	300.0982	19588.77	294.3044	19583.68	
300.042	19588.18			299.4458	19591.46	296.3527	19591.41			294.5686	19583.29	
				299.7024	19588.94	296.6257	19588.86			295.0599	19576.59	
				300.2015	19591.97	297.1192	19589.13			295.3273	19585.21	
						297.3881	19586.83			295.8197	19576.65	
						297.8792	19591.23			296.0867	19578.29	
						298.1484	19590.68			296.5779	19580.17	
						298.646	19593.51			296.8451	19581.67	
						298.9108	19597.5			297.3375	19579.8	
						299.406	19593.13			297.6023	19578.14	
						299.6741	19590.64			298.0939	19578.39	
						300.1668	19594.51			298.3651	19586.62	
										298.8624	19573.9	
										299.1352	19578.97	
										299.6255	19578.42	
										299.8975	19576.58	
										300.392	19579.79	

0.71							1.07					
30							30					
2	x_3		x_4		x_5		x_1		x_2		x_3	
P (cm^{-1})	t (s)	P (cm^{-1})	t (s)	P (cm^{-1})	t (s)	P (cm^{-1})	t (s)	P (cm^{-1})	t (s)	P (cm^{-1})	t (s)	P (cm^{-1})
19611.05	0	19612.85	0	19609.73	0	19618.92	0	19605.15	0	19604.98	0	19605.94
19611.56	0.70495	19607.65	0.68326	19607.88	0.70586	19619.87	0.66886	19604.13	0.66703	19603.77	0.66334	19606.26
19610.74	0.85825	19617.38	0.85055	19614.76	0.85154	19622.29	0.85595	19603.05	0.85246	19606.39	0.85305	19604.19
19607.8	1.10446	19611.83	1.10519	19608.57	1.10351	19610.08	1.10378	19603.61	1.10384	19606.18	1.10305	19605.26
19612.74	1.35535	19613.37	1.35218	19607.02	1.35737	19614.41	1.35488	19599.59	1.35116	19604.63	1.3518	19604.29
19608.44	1.60528	19616.6	1.60737	19612.19	1.60319	19612.38	1.60248	19602.56	1.60232	19602.96	1.60191	19604.96
19606.96	1.85325	19612.52	1.85129	19610.55	1.84948	19617.69	1.85315	19602.64	1.85166	19604.8	1.85163	19601.49
19611.07	2.10094	19609.33	2.10338	19610.06	2.10069	19616.21	2.10017	19600.39	2.10047	19603.64	2.10089	19605.13
19604.36	2.35464	19610.68	2.35193	19608.85	2.35681	19615.16	2.35262	19605.13	2.35096	19604.63	2.35225	19604.95
19610.47	2.6011	19610.02	2.60603	19610.23	2.60146	19613.92	2.60181	19604.29	2.60167	19604.01	2.6026	19603.72
19608.85	2.85306	19606.48	2.85092	19614.09	2.85047	19612.28	2.85528	19602.69	2.8521	19604.46	2.85239	19603.75
19613.29	3.10127	19612.99	3.10805	19609.53	3.10077	19612.68	3.10056	19600.75	3.10132	19604.45	3.10169	19602.7
19609.14	3.35529	19607.48	3.35204	19612.15	3.3562	19606.8	3.35423	19604.94	3.35097	19602.28	3.35645	19605.83
19606.17	3.60422	19616.53	3.60717	19610.86	3.6015	19616.59	3.60268	19604.08	3.60101	19603.46	3.60283	19603.29
19606.19	3.85571	19606.6	3.85008	19608.75	3.85149	19610.56	3.85555	19605.5	3.85123	19603.06	3.85249	19606.14
19609.34	4.10056	19607.08	4.104	19614.11	4.10086	19616.91	4.10138	19602.87	4.10222	19603.06	4.10198	19606.75
19611.28	4.35426	19615.25	4.35246	19610.16	4.35575	19611.82	4.35393	19603.86	4.35238	19604.06	4.35157	19603.73
19609.77	4.60502	19610.85	4.60746	19607.39	4.60281	19611.25	4.60154	19602.07	4.60166	19603.78	4.60146	19605.79
19610.51	4.85577	19610.81	4.85799	19615.29	4.85237	19611.64	4.85767	19600.51	4.85173	19603.54	4.85256	19603.41
19606.91	5.10833	19612.29	5.10832	19614.72	5.10269	19608.89	5.10715	19602.36	5.10349	19601.67	5.10322	19604.57
19610.44	5.35951	19609.92	5.35075	19609.73	5.35915	19614.29	5.35414	19601.27	5.35678	19604.99	5.35148	19602.73
19610.71	5.60245	19611.98	5.6051	19613.29	5.6047	19615.1	5.60193	19603.56	5.60707	19604.23	5.60173	19603.99
19615.05	5.85304	19615.13	5.85258	19612.82	5.85085	19608.22	5.85499	19601.48	5.85226	19604.43	5.85134	19604.24
19611.19	6.10245	19613.55	6.10488	19609.56	6.10214	19610.33	6.10126	19603.78	6.10173	19600.37	6.10612	19606
19608.55	6.35632	19611.24	6.35267	19609.38	6.35669	19615.31	6.36449	19602.99	6.35208	19604.14	6.35757	19602.76
19608.26	6.60317	19611.61	6.60668	19608.9	6.60355	19609.21	6.60495	19603.87	6.60267	19603.41	6.60391	19605.39
19608.58	6.857	19610.4	6.85221	19617.3	6.8513	19612.97	6.85772	19603.61	6.85409	19603.95	6.85331	19603.21
19611.76	7.10217	19616.36	7.10586	19608.94	7.10164	19613.27	7.10128	19600.88	7.10233	19605.29	7.10216	19601.79
19608.35	7.35527	19610.52	7.3543	19606.36	7.356	19614.74	7.35422	19601.33	7.35375	19603.94	7.35419	19606.36
19610.89	7.60294	19610.18	7.61109	19609.75	7.60585	19611.83	7.6028	19604.38	7.60247	19604.87	7.60326	19604.11
19611.48	7.85609	19616.29	7.85188	19611.49	7.8518	19613.91	7.86102	19603.22	7.85239	19603.68	7.8529	19603.8
19607.45	8.10239	19610.17	8.10591	19605.16	8.10819	19616.77	8.10462	19603.2	8.10378	19603.99	8.10267	19604.4
19602.64	8.35656	19615.13	8.35434	19614.23	8.35655	19615.73	8.35557	19602.85	8.35506	19606.94	8.35356	19602.07
19613.06	8.60443	19610.95	8.60947	19612.26	8.60213	19618.51	8.60288	19605.45	8.60352	19607.56	8.6023	19602.39
19609.43	8.85628	19611.76	8.85209	19607.6	8.85264	19613.49	8.85641	19604.45	8.85214	19603.31	8.85341	19603.31
19608.96	9.10333	19614.17	9.10458	19609.22	9.10448	19611.79	9.10569	19603.28	9.10267	19606.27	9.10384	19605.13
19616.11	9.35484	19609.56	9.35179	19614.49	9.35702	19616.47	9.35562	19603.98	9.35399	19604.26	9.35301	19605.6
19613.78	9.60216	19613.84	9.6098	19614.6	9.60246	19611.59	9.60263	19605.26	9.60435	19605.02	9.60269	19603.89
19609.47	9.85634	19618.5	9.85285	19611.13	9.8527	19612.05	9.85657	19602.81	9.85241	19606.19	9.85319	19602.68
19607.84	10.10342	19610.29	10.10622	19614.49	10.10355	19613.92	10.10344	19603.88	10.10281	19604.5	10.10393	19605.05
19607.91	10.35685	19608.26	10.35289	19607.85	10.35993	19611.62	10.35551	19601.27	10.35391	19603.77	10.35472	19604.23
19613.82	10.60509	19612.23	10.61003	19607.57	10.60977	19612.96	10.60355	19603.92	10.60493	19603.97	10.60447	19603.98
19606.36	10.85522	19608.46	10.85308	19609.73	10.85303	19611.64	10.85885	19604	10.8548	19605.66	10.8535	19602.39
19608.45	11.10421	19608.91	11.10657	19613.83	11.10366	19614.97	11.10377	19604.33	11.1034	19603.55	11.1049	19603.76
19608.77	11.35768	19608.83	11.35506	19613.1	11.3583	19612.07	11.35671	19601.45	11.35419	19603.04	11.35479	19605.48
19613.46	11.60557	19614.49	11.60941	19605.3	11.60592	19611.54	11.60597	19601.95	11.60539	19603.73	11.60557	19604.43
19613.02	11.85662	19610.82	11.85313	19606.08	11.85324	19611.79	11.85775	19603.11	11.85489	19601.99	11.85375	19605.73
19613.25	12.10445	19614.21	12.10761	19608.89	12.10447	19611.36	12.10382	19602.21	12.10515	19603.57	12.10423	19606.46
19612.62	12.35734	19616.46	12.35473	19612.25	12.35785	19610.05	12.35663	19604.47	12.35399	19604.98	12.36077	19605.57
19608.35	12.60401	19609.57	12.61015	19609.27	12.60494	19611.03	12.60439	19605.33	12.60475	19603.99	12.60504	19602.75
19610.79	12.87687	19613.8	12.8531	19607.86	12.85661	19615.81	12.85959	19604.4	12.85391	19604.57	12.85501	19605.42
19609.9	13.10595	19614.73	13.10585	19614.17	13.10577	19611.38	13.10373	19602.21	13.10414	19605.21	13.1038	19605.57
19615.07	13.35798	19608	13.3537	19615.29	13.35756	19617.05	13.35656	19601.11	13.35493	19602.06	13.35427	19606.33
19610.61	13.60453	19614.65	13.60966	19611.66	13.6036	19619.16	13.60449	19601.6	13.61936	19607.38	13.60427	19605.61
19606.49	13.85833	19611.35	13.85407	19614.58	13.85325	19606.94	13.85873	19607.63	13.85698	19602.31	13.85528	19606.1
19611.92	14.10598	19615	14.10618	19609.81	14.10645	19611.33	14.10434	19603.53	14.10428	19601.78	14.10577	19604.06
19608.47	14.35856	19610.66	14.354	19612.67	14.36043	19617.65	14.35629	19606.53	14.35586	19605.49	14.35415	19603.1
19611.23	14.6051	19610.39	14.61094	19612.99	14.60454	19609.92	14.60373	19604.47	14.60518	19603.49	14.60435	19603.9
19610.75	14.85729	19606.98	14.85552	19609.37	14.85375	19610.07	14.85964	19604.09	14.85456	19606.75	14.85506	19605.91
19608.53	15.10496	19613.32	15.10871	19615.3	15.10504	19620.58	15.10549	19604.06	15.10427	19602.67	15.10609	19604
19612.74	15.3589	19611.28	15.35425	19612.33	15.36019	19612.28	15.35679	19603.12	15.356	19606.33	15.35543	19605.26
19612.2	15.60616	19611.91	15.61318	19610.57	15.60544	19612.17	15.60494	19604.18	15.60612	19604.98	15.60489	19603.94
19610.34	15.8585	19615.3	15.85513	19607.64	15.85511	19612.66	15.86242	19603.39	15.85648	19602.33	15.86317	19604.8
19611.33	16.10573	19612.87	16.10825	19613.18	16.10629	19617.23	16.10619	19600.84	16.10517	19602.55	16.10657	19605.57

19609.37	16.35917	19609.72	16.35635	19611.24	16.36101	19615.23	16.35977	19604.68	16.35545	19600.99	16.35713	19606.28
19610.99	16.60548	19615.97	16.6153	19610.76	16.60707	19609.53	16.607	19603.45	16.60692	19603.59	16.60514	19609.05
19609.78	16.85905	19611.42	16.85447	19611.11	16.85582	19615.49	16.85904	19600.87	16.85589	19604.37	16.85541	19606.14
19613.58	17.10631	19611.81	17.10828	19606.85	17.10745	19612.51	17.10568	19603.07	17.10481	19603.91	17.10648	19606.04
19612.21	17.35996	19608.44	17.35596	19609.81	17.35915	19610.02	17.35849	19603.49	17.35538	19604.41	17.3565	19606.66
19611.85	17.60533	19615.72	17.61212	19612.26	17.60562	19611.06	17.60578	19605.88	17.60548	19603.6	17.60648	19603.12
19608.15	17.85938	19613.14	17.85639	19616.34	17.85499	19614.09	17.86267	19601.52	17.85695	19604.8	17.85655	19603.85
19606.26	18.10531	19614.61	18.10823	19610.43	18.10642	19607.87	18.10554	19604.2	18.10645	19605.18	18.10564	19606.01
19611.92	18.35871	19616.48	18.35549	19616.3	18.36131	19610.48	18.35809	19602.3	18.35548	19602.12	18.3555	19604.48
19613.3	18.60632	19610.99	18.61704	19610.3	18.60639	19614.16	18.60639	19603.49	18.60508	19604.93	18.60667	19603.99
19604.44	18.86006	19613.9	18.85799	19615.26	18.85476	19618.21	18.86152	19603.99	18.85667	19604.6	18.8567	19603.91
19608.87	19.10585	19608.54	19.10952	19607.66	19.11168	19614.82	19.10637	19603.58	19.10703	19603.05	19.10725	19605.55
19610.06	19.36107	19614.12	19.35598	19613.09	19.36334	19614.71	19.36008	19603.16	19.35693	19604.42	19.35621	19604.94
19611.75	19.60588	19613.98	19.61205	19611.37	19.60811	19619.01	19.6063	19602.63	19.60609	19605.02	19.6071	19605.27
19613.2	19.85805	19614.52	19.85695	19618.79	19.85491	19614.39	19.8625	19605.04	19.85599	19602.75	19.85678	19604.1
19612.11	20.1086	19612.44	20.11008	19610.27	20.10648	19614.36	20.10788	19603.76	20.10672	19602.85	20.10845	19605.66
19606.84	20.36164	19616.4	20.35793	19612.96	20.35931	19611.5	20.35876	19603.69	20.35833	19603.63	20.3578	19602.1
19610.83	20.60665	19613.5	20.61349	19613.17	20.60844	19617.33	20.60722	19602.88	20.60803	19605.66	20.60699	19604.74
19608.61	20.86255	19613.34	20.86093	19611.99	20.85734	19613.12	20.86466	19603.95	20.85739	19606.8	20.85719	19601.95
19608.91	21.10845	19611.54	21.1107	19615.73	21.10763	19614.52	21.10826	19603.57	21.10687	19606.44	21.10971	19603.23
19608.51	21.3624	19612.61	21.37375	19612.4	21.36154	19615.92	21.36061	19602.23	21.35749	19603.31	21.35915	19603.47
19617.26	21.60706	19609.08	21.61494	19611.3	21.60911	19614.17	21.60887	19603.34	21.61504	19603.84	21.60996	19604.43
19612.51	21.86103	19613.03	21.85797	19608.54	21.85767	19616.3	21.86564	19603.37	21.85772	19604.04	21.85811	19603.15
19609.82	22.10723	19610.06	22.11123	19612.46	22.10843	19621.39	22.10841	19604.86	22.10829	19604.44	22.10984	19602.19
19611.02	22.36175	19614.16	22.35718	19614.56	22.36018	19612.17	22.36039	19601.78	22.35819	19605.29	22.35883	19604.65
19611.33	22.60845	19608.88	22.61369	19614.21	22.60845	19613.4	22.6073	19601.51	22.60778	19606.67	22.60978	19604.58
19612.38	22.85932	19609.2	22.85855	19613.58	22.8574	19612.46	22.86549	19602.51	22.85975	19606.47	22.85803	19604.05
19611.61	23.10738	19613.57	23.11229	19612.44	23.10897	19610.16	23.1085	19602.19	23.10911	19605.22	23.10879	19605.52
19608.12	23.36225	19608.62	23.35748	19608.66	23.36475	19608.46	23.36139	19603.59	23.35805	19605.6	23.35875	19604.02
19613.05	23.60866	19610.62	23.61687	19612.35	23.60876	19617.14	23.60797	19604.21	23.6078	19603.1	23.60908	19605.18
19608.83	23.86267	19616.39	23.85762	19608.32	23.85791	19612.51	23.86322	19599.85	23.85895	19604.26	23.85923	19600.94
19613.43	24.10773	19609.24	24.11326	19611.91	24.10977	19617.2	24.10856	19604.59	24.10965	19606.87	24.10946	19604.67
19613.68	24.36515	19612.06	24.35941	19609.32	24.36178	19608.35	24.36221	19602.73	24.35864	19603.92	24.3585	19602.64
19609.56	24.60787	19610.4	24.61389	19613.44	24.60872	19614.2	24.60868	19602.98	24.6073	19604.88	24.60775	19604.9
19610.77	24.88038	19613.49	24.85753	19612.49	24.85678	19616.73	24.86754	19605.02	24.85825	19601.96	24.85881	19605.09
19608.23	25.10976	19614.59	25.11045	19611.02	25.10841	19613.26	25.10933	19603.12	25.10924	19606.09	25.11016	19606.34
19610.18	25.36135	19613.17	25.36385	19609.7	25.35989	19610.85	25.36087	19604.65	25.35905	19605.03	25.35902	19603.66
19607.02	25.60743	19613.05	25.61801	19610.79	25.61005	19615.6	25.61292	19603.8	25.60813	19604.25	25.60927	19602.55
19613.65	25.86174	19613.54	25.85789	19613.92	25.85775	19611.83	25.86678	19604.54	25.85816	19602.16	25.858	19604.93
19608.53	26.10891	19611.15	26.11322	19608.5	26.10881	19612.78	26.10844	19602.9	26.10868	19605.83	26.10865	19604.77
19612.67	26.36271	19608.28	26.3599	19616	26.36199	19612.53	26.36167	19603.88	26.35909	19604.28	26.36339	19605.96
19611.79	26.60853	19613.9	26.63317	19608.49	26.61148	19608.14	26.61118	19603.52	26.60955	19603.81	26.60984	19605.34
19611.76	26.86244	19611.56	26.85963	19612.68	26.85885	19614.68	26.86432	19602.11	26.85952	19605.25	26.8588	19604.37
19606.51	27.10751	19612.98	27.11243	19611.9	27.10947	19612.84	27.10777	19604.24	27.10856	19604.48	27.10845	19603.74
19611.24	27.36345	19609.29	27.35863	19609.79	27.36066	19609.44	27.36049	19604.94	27.35839	19605.6	27.35908	19602.8
19615.31	27.6092	19616.48	27.61467	19608.38	27.61918	19610.24	27.60909	19602.88	27.60874	19601.65	27.60934	19602.89
19608.06	27.86271	19610.32	27.85888	19614.4	27.8574	19608.01	27.86406	19604.01	27.86116	19604.08	27.86003	19607.46
19615.37	28.10854	19615.51	28.11209	19615.03	28.10938	19612.67	28.10908	19603.14	28.10978	19606.61	28.10981	19604.87
19612.2	28.36217	19611.36	28.35938	19613.8	28.36283	19612.15	28.36034	19603.32	28.35849	19603.52	28.35898	19603.86
19608.74	28.60916	19605.48	28.61615	19615.59	28.6103	19620.66	28.60855	19605.69	28.60881	19601.93	28.60926	19604.69
19609.4	28.86342	19609.21	28.85948	19616.3	28.85752	19612.99	28.86565	19600.65	28.85942	19603.88	28.85865	19603.02
19609.39	29.10852	19610.38	29.1128	19608.19	29.10825	19610.93	29.10986	19602.4	29.11013	19605.4	29.11018	19604.57
19609.48	29.36203	19609.53	29.35996	19611.07	29.3607	19612.52	29.36177	19603.54	29.35989	19604.84	29.3608	19601.05
19611.1	29.61002	19614.1	29.61753	19609.99	29.61038	19609.53	29.61387	19605.67	29.60917	19603.81	29.60959	19603.62
19609.99	29.86177	19614	29.8593	19608.81	29.86025	19612.97	29.86408	19605.37	29.86012	19602.87	29.85947	19603.95
19606.92	30.1105	19609.53	30.11435	19615.27	30.11196	19610.91	30.11148	19602.76	30.1129	19607.2	30.10981	19604.66
19607.91	30.36476	19611.45	30.36178	19616.09	30.36133	19615.59	30.36319	19603.51	30.36161	19603.03	30.36818	19603.9
19609	30.61043	19608.31	30.61805	19610.22	30.61121	19617.76	30.61145	19604.02	30.62002	19603.8	30.61223	19605.23
19606.85	30.86608	19609.85	30.86036	19613.63	30.86098	19612.14	30.86594	19603.69	30.86056	19607.18	30.86148	19606.98
19608.83	31.11113	19611.08	31.11447	19612.46	31.11261	19614.24	31.11016	19603.59	31.11081	19608.11	31.1102	19605.66
19609.79	31.36634	19609.02	31.36173	19612.29	31.36227	19612.47	31.3638	19600.88	31.36129	19604.1	31.36057	19605.15
19608.05	31.61218	19610.33	31.62122	19609.97	31.61874	19609.68	31.61395	19603.18	31.61158	19606.3	31.61225	19604.03
19608.49	31.86504	19611.83	31.86077	19608.49	31.86088	19608.67	31.86778	19605.2	31.86268	19604.67	31.86279	19602.85
19607.5	32.1111	19611.08	32.11376	19610.53	32.11236	19614.93	32.11135	19602.49	32.1109	19605.79	32.11178	19603
19607.56	32.36619	19607	32.36194	19609.45	32.36442	19619.23	32.36354	19602.47	32.36102	19604.95	32.36133	19608.34
19609.56	32.61121	19605.09	32.62471	19612.38	32.61038	19619.92	32.61573	19602.77	32.61435	19604.6	32.61315	19606.84
19611.38	32.86551	19610.09	32.86275	19612.06	32.86019	19615.07	32.868	19601.94	32.86245	19607.17	32.86153	19607.24
19610.59	33.10976	19611.63	33.1142	19610.27	33.11166	19612.22	33.11037	19604.18	33.11086	19604.51	33.11179	19602.14
19614.85	33.36536	19614.09	33.36049	19611.28	33.36484	19608.19	33.3632	19603.57	33.36046	19604.07	33.36157	19600.62
19613.49	33.61166	19604.4	33.61889	19613.18	33.61234	19613.18	33.61207	19604.25	33.61007	19602.39	33.61065	19605.39

19612.83	33.86441	19616.78	33.86093	19605.78	33.85959	19609.66	33.86563	19603.38	33.86243	19605.36	33.86127	19604.36
19609.68	34.11137	19611.73	34.11548	19608.67	34.11139	19615.12	34.11257	19605.17	34.1121	19604.85	34.11202	19606.2
19607.16	34.36663	19614.03	34.36216	19609.98	34.36383	19614.73	34.36446	19601.96	34.36167	19603.64	34.36274	19604.91
19609.51	34.61159	19610.45	34.61932	19610.38	34.61167	19616.38	34.61135	19603.69	34.61185	19603.57	34.61202	19604.7
19610.45	34.86695	19611.01	34.86069	19615.82	34.86055	19615	34.87428	19604.42	34.86209	19602.02	34.86151	19603.78
19611.01	35.11633	19610.2	35.11498	19613.26	35.11172	19610.83	35.1143	19605.61	35.11179	19603.28	35.11125	19601.99
19609.53	35.36585	19608.97	35.36259	19613.49	35.36416	19614.95	35.36398	19603.26	35.36287	19603.68	35.36226	19605.18
19612.26	35.61142	19612.55	35.62083	19608.95	35.61079	19616.65	35.61059	19601.24	35.6128	19602.56	35.61307	19603.59
19610.67	35.86417	19612.96	35.86259	19606.01	35.86104	19615.34	35.86665	19605.61	35.86374	19602.19	35.86326	19604.17
19608.61	36.1147	19608.7	36.11404	19611.37	36.11345	19608.57	36.11369	19602.46	36.112	19604.98	36.11159	19603.21
19607.11	36.3649	19610.03	36.36198	19613.38	36.36087	19611.67	36.37552	19601.75	36.36142	19602.84	36.36314	19604.29
19609.53	36.61148	19611.93	36.6227	19607.9	36.61314	19610.83	36.61431	19603.56	36.6124	19602.96	36.61283	19604.98
19609.18	36.86558	19615.57	36.8663	19615.62	36.86127	19613.79	36.86667	19603.55	36.86461	19605.96	36.86326	19606.09
19611.14	37.11277	19614.61	37.11572	19611.29	37.11384	19608.91	37.11086	19603.79	37.1129	19601.44	37.11305	19601.62
19606.92	37.36719	19617.07	37.36065	19606.46	37.3642	19616.48	37.36561	19601.55	37.36256	19604.36	37.36176	19605
19611.87	37.6114	19612.37	37.62273	19611.44	37.61178	19614.57	37.61217	19603.41	37.61172	19604.96	37.61237	19605.95
19609.91	37.8666	19611	37.86241	19612.03	37.86063	19613.26	37.86791	19604.59	37.86308	19604.17	37.86291	19604.27
19608.37	38.11198	19612.77	38.11654	19604.39	38.11398	19609	38.11229	19600.29	38.11431	19605.72	38.11331	19603.32
19608.65	38.36689	19611.48	38.36287	19609.15	38.36414	19614.93	38.36426	19605.83	38.36337	19604.71	38.36294	19600.34
19612.57	38.61293	19612.11	38.62416	19614.48	38.61351	19615.35	38.61294	19602.31	38.61173	19604.68	38.61207	19605.71
19614.06	38.8646	19614.28	38.86219	19613.38	38.86146	19612.74	38.87197	19604.99	38.86223	19604.34	38.86239	19603.88
19611.11	39.11181	19617.45	39.11638	19611.38	39.11107	19610.73	39.11182	19603.77	39.11374	19601.74	39.11343	19604.1
19611	39.36895	19613.6	39.36236	19613.32	39.36327	19615.01	39.36385	19605.27	39.36371	19603.79	39.36424	19605.2
19612.16	39.61415	19616.57	39.62302	19608.58	39.61395	19609.8	39.61301	19601.52	39.61441	19605.51	39.61405	19604.45
19609.34	39.86512	19608.9	39.86247	19609.6	39.86334	19612.45	39.86872	19601.96	39.86251	19602.91	39.86238	19605
19610.84	40.11272	19619.09	40.11557	19610.43	40.11445	19615.05	40.11377	19602.96	40.11247	19604.49	40.11343	19602.49
19606.38	40.3681	19617.12	40.36393	19608.5	40.36369	19614.5	40.36758	19604.33	40.36468	19602.17	40.36676	19601.59
19611.66	40.61496	19611.57	40.62333	19609.97	40.62293	19611.75	40.61355	19602.28	40.61515	19602.75	40.61608	19603.66
19605.82	40.87558	19616.4	40.86568	19610.54	40.86463	19613.45	40.87218	19599.88	40.86502	19603.41	40.86455	19605.73
19609.3	41.11539	19616.08	41.11824	19608.71	41.11566	19616.35	41.11607	19600.79	41.11461	19605.48	41.11433	19605.65
19610.61	41.36981	19609.73	41.3642	19610.52	41.3686	19613.8	41.36891	19606.12	41.3642	19604.79	41.36656	19606.34
19605.8	41.61406	19610.62	41.62459	19610.14	41.61471	19614.74	41.61565	19602.69	41.61431	19606.72	41.61634	19604.36
19606	41.86894	19615.31	41.8663	19614.72	41.86404	19613.81	41.87241	19601.72	41.86499	19605.45	41.86554	19602.81
19611.79	42.11529	19612.58	42.11744	19606.45	42.11616	19612.19	42.11611	19601.73	42.11522	19602.42	42.11481	19603.95
19609.16	42.36982	19614.44	42.3642	19610.45	42.36683	19617.74	42.36786	19602.83	42.36451	19599.35	42.36544	19603.93
19612.67	42.61536	19609.08	42.62164	19614.73	42.61578	19612.76	42.61415	19604.54	42.61487	19603.79	42.61639	19605.1
19612.28	42.8673	19615.32	42.866	19611.14	42.86469	19619.5	42.87077	19601.26	42.86629	19604.4	42.86638	19606.22
19610.08	43.11487	19614.53	43.11977	19611.28	43.11507	19612.79	43.1154	19603.68	43.11625	19605.77	43.11618	19605.55
19607.18	43.37014	19610.92	43.36525	19615.4	43.36655	19614.76	43.37382	19604.18	43.3651	19605.53	43.36616	19603.76
19603.88	43.61611	19611.17	43.62468	19608.67	43.61639	19611.11	43.61659	19606.53	43.61411	19605.63	43.61569	19605.12
19611.6	43.86937	19606.91	43.86646	19609.5	43.8678	19616.77	43.87014	19602.64	43.86564	19604.14	43.86564	19604.31
19610.84	44.11453	19612.42	44.11986	19608.14	44.11587	19615.87	44.11638	19603.34	44.11642	19604.39	44.11649	19604.08
19612.07	44.37116	19613.42	44.36649	19611.07	44.36684	19613.65	44.36795	19605.61	44.36698	19603.61	44.36787	19606.75
19605.96	44.6166	19608.85	44.62548	19608.74	44.6159	19606.87	44.61646	19602.32	44.61612	19603.96	44.6164	19606.07
19610.28	44.8693	19613.68	44.86535	19613.94	44.86693	19612.46	44.87632	19604.79	44.8657	19605.06	44.86497	19605.79
19607.02	45.11547	19610.42	45.11846	19614.2	45.11758	19617.3	45.11645	19604.96	45.1159	19603.38	45.11539	19605.93
19614.01	45.36974	19610.65	45.3667	19614.84	45.36593	19609.31	45.3697	19603.82	45.36714	19604.54	45.36825	19607.28
19606.09	45.61709	19608.27	45.62623	19611.89	45.6217	19619.44	45.6168	19603.25	45.61702	19602.52	45.61807	19604.48
19608.24	45.87161	19609.54	45.86931	19609.38	45.8672	19608.66	45.87408	19603.9	45.86686	19603.67	45.8671	19605.74
19606.98	46.11725	19610.65	46.11961	19609.27	46.11588	19611.79	46.11694	19603.62	46.11586	19605.1	46.11991	19603.92
19608.8	46.37028	19609.92	46.36677	19611.68	46.36811	19610.7	46.3672	19602.75	46.3667	19602.82	46.36625	19603.73
19615.6	46.6157	19607.98	46.62556	19612.35	46.61866	19613.55	46.61793	19603.31	46.61843	19603.91	46.61771	19604.95
19609.07	46.87164	19612.71	46.86709	19604.6	46.86646	19610.28	46.87702	19604.65	46.86761	19604.77	46.86782	19607.27
19611.22	47.11649	19614.31	47.12031	19610.52	47.11734	19618.04	47.11818	19605.38	47.1169	19603.09	47.1168	19603.64
19610.19	47.37121	19610.37	47.36639	19608.88	47.36992	19616.89	47.37	19603.46	47.36645	19600.28	47.36701	19605.3
19612.3	47.61632	19610.83	47.62474	19606.36	47.61764	19611.49	47.61629	19602.2	47.61698	19600.36	47.61702	19604.57
19614.33	47.86935	19610.36	47.86672	19607.84	47.86559	19609.7	47.8735	19602.78	47.86804	19606.79	47.86779	19603.58
19612.67	48.11685	19615.23	48.12052	19612.77	48.11553	19614.8	48.11799	19602.26	48.11686	19602.71	48.11799	19603.73
19609.66	48.37188	19608.53	48.3665	19609.07	48.36736	19611.94	48.37052	19605.47	48.36609	19603.28	48.36752	19605.36
19611.92	48.61815	19610.83	48.62617	19616.7	48.61632	19609.93	48.61557	19603.02	48.61511	19605.36	48.61619	19604.24
19611.37	48.87141	19614.43	48.86615	19610.82	48.86596	19613.66	48.87109	19600.98	48.86597	19604.12	48.8658	19605.13
19615.61	49.11619	19611.9	49.11968	19606.82	49.11738	19612.8	49.1158	19603.15	49.11715	19604.45	49.1174	19603.39
19605.35	49.37164	19612.77	49.36614	19612.09	49.3656	19607.77	49.36891	19602.43	49.36717	19603.3	49.36732	19604.56
19609.67	49.61685	19611.33	49.62518	19609.93	49.61659	19616.03	49.61696	19602.56	49.6171	19606.8	49.61553	19604.84
19610	49.87281	19610.97	49.8651	19609.11	49.86614	19610.14	49.87416	19601.5	49.86662	19605.65	49.86569	19604.12
19609.79	50.11776	19609.24	50.11967	19617.28	50.11841	19612.89	50.11571	19604.01	50.11664	19603.5	50.11758	19604.1
19611.95	50.37067	19612.94	50.36629	19615.19	50.36863	19613.83	50.37074	19603	50.36789	19606.03	50.37523	19604.11
19612.26	50.61664	19609.65	50.62603	19615.53	50.61713	19615.08	50.61704	19601.96	50.61717	19604.51	50.61874	19605.62
19609.41	50.87223	19611.81	50.86561	19612.61	50.86521	19615.07	50.87453	19601.89	50.86802	19603.54	50.8666	19603.66
19607.54	51.11811	19607.02	51.1202	19612.1	51.11724	19616.68	51.11561	19604.02	51.11624	19604.5	51.11731	19604.31

19610.22	51.37246	19613.37	51.36679	19608.71	51.36826	19617.72	51.36965	19600.93	51.36732	19603.71	51.36695	19604.51
19612.36	51.61692	19612.62	51.62624	19615.45	51.61888	19616.5	51.6191	19603.73	51.61995	19602.3	51.61834	19604.9
19606.33	51.87281	19614.01	51.86779	19611.98	51.86595	19615.25	51.87683	19602.43	51.86694	19604.45	51.86736	19603.22
19611.98	52.11776	19616.29	52.12006	19608.81	52.11707	19612.02	52.11773	19604.64	52.11736	19603.58	52.11689	19604.54
19610.08	52.3732	19613.75	52.36572	19608.86	52.368	19618	52.36874	19602.59	52.36695	19604.45	52.36737	19605.25
19614.74	52.61911	19613.68	52.62679	19609.23	52.61907	19618.14	52.61644	19602.67	52.61823	19601.51	52.61889	19604.32
19609.67	52.87131	19611.7	52.86661	19607.74	52.86789	19608.76	52.87582	19603.88	52.86862	19602.48	52.86865	19603.33
19609.02	53.1176	19611.34	53.12226	19609.69	53.11711	19612.95	53.11788	19604.08	53.11837	19604.67	53.11735	19605.32
19607.26	53.3732	19615.08	53.36751	19614.16	53.36623	19612.13	53.3722	19599.92	53.36702	19604.84	53.36757	19603.25
19610.09	53.61945	19611.08	53.62648	19609.52	53.61779	19614.45	53.61659	19603.37	53.61849	19603.23	53.61806	19604.19
19611.33	53.87226	19611.8	53.86563	19604.32	53.86858	19614.59	53.87499	19604.15	53.86945	19605.07	53.86856	19603.35
19609.08	54.11902	19614.36	54.12154	19611	54.12036	19612.05	54.11843	19604.28	54.12019	19605.85	54.11914	19604.16
19613.24	54.37414	19613.03	54.36859	19606.22	54.36619	19609.28	54.37096	19602.57	54.36707	19603.13	54.36741	19605.69
19610.34	54.61966	19613.96	54.62696	19612.99	54.61856	19610.04	54.61834	19603.3	54.61751	19602.6	54.61728	19604.82
19608.67	54.87318	19613.81	54.86683	19611.6	54.86812	19612.21	54.87577	19604.04	54.86832	19605.77	54.86777	19604.29
19611.38	55.11715	19612.36	55.12143	19609.87	55.11936	19615.6	55.11785	19604.14	55.121	19602.53	55.11948	19605.81
19610.29	55.37243	19609.84	55.36637	19609.85	55.36634	19615.81	55.37068	19603.82	55.36939	19603.28	55.37716	19603.84
19613.51	55.61951	19610.96	55.62778	19611.05	55.61798	19609.18	55.61959	19603.29	55.61904	19604.85	55.62013	19605.33
19608.17	55.87473	19616.12	55.8679	19612.68	55.86802	19615.74	55.87716	19604.75	55.86766	19603.54	55.8677	19604.72
19611.64	56.11834	19616.16	56.12299	19614.21	56.11981	19613.76	56.11788	19603.84	56.11828	19605.63	56.11812	19604.11
19610.19	56.37325	19610.57	56.36716	19606.85	56.36765	19619.88	56.37084	19602.97	56.37011	19604.36	56.36952	19607.37
19613.04	56.61944	19609.15	56.62778	19614.15	56.61848	19612.06	56.62135	19602.79	56.62075	19603.63	56.61981	19608.88
19608.69	56.87307	19609.64	56.86855	19610.25	56.86815	19613.21	56.87797	19604.21	56.86928	19602.98	56.86837	19603.93
19610.64	57.12024	19618.39	57.12292	19611.69	57.12153	19611.74	57.11907	19604	57.12036	19606.54	57.1185	19601.16
19610.81	57.37323	19611.74	57.36736	19612.48	57.36911	19615.8	57.37047	19603.65	57.36917	19605.47	57.36928	19604.29
19606.89	57.61855	19611.43	57.62773	19615.71	57.6203	19611.7	57.61967	19602.35	57.61983	19603.94	57.62042	19602.86
19609.7	57.87335	19612.43	57.86824	19613.47	57.86839	19611.66	57.87667	19600.51	57.8716	19601.51	57.87164	19603.62
19607.74	58.11867	19611.39	58.12283	19612.8	58.11888	19609.99	58.11947	19605.21	58.12099	19605.22	58.11867	19603.17
19607.99	58.37397	19608.97	58.36986	19616.35	58.36842	19614.86	58.37265	19604.25	58.36817	19605.7	58.36889	19606.47
19611.46	58.62124	19614.73	58.62745	19612.41	58.62023	19613.2	58.61897	19605.58	58.6201	19604.4	58.62752	19606.46
19610.06	58.87686	19617.17	58.86899	19614.68	58.86899	19612.19	58.8772	19600.84	58.87008	19604.47	58.86985	19604.18
19607.42	59.11749	19609.82	59.12393	19610.7	59.1229	19617.88	59.11976	19602.92	59.12087	19601.97	59.12028	19604.9
19610.94	59.37564	19611.13	59.36995	19615.46	59.36777	19615.8	59.37241	19604.48	59.36955	19602.74	59.36942	19603.47
19610.5	59.61925	19612.13	59.6294	19612.76	59.6195	19615.86	59.61983	19602.81	59.62079	19605.64	59.61926	19604.81
19610.55	59.8772	19614.3	59.87168	19611.31	59.87066	19612.87	59.87908	19602.29	59.87337	19604.41	59.8723	19605.07
19611.09	60.12062	19609.47	60.12289	19608.48	60.12234	19609.67	60.12112	19601.29	60.12143	19603.65	60.12097	19604.84
19609.83	60.37508	19616.54	60.37049	19612.64	60.37081	19616.35	60.37649	19601.89	60.37185	19604.37	60.37294	19606.87
19607.61	60.62125	19615.77	60.63049	19609.97	60.62066	19616.25	60.6217	19603.57	60.62192	19603.8	60.62163	19603.98
19609	60.87584	19609.85	60.87604	19606.57	60.87001	19610.69	60.87771	19602.32	60.87171	19604.42	60.87073	19603.4
19609.97	61.12183	19605.67	61.12562	19612.95	61.12366	19611.27	61.12131	19603.91	61.12175	19602.42	61.12168	19604.48
19608.95	61.37744	19608	61.36932	19616.16	61.37142	19613.83	61.37601	19602.05	61.37248	19602.07	61.37314	19605.29
19612.79	61.62103	19609.51	61.6307	19614.01	61.62425	19612.24	61.62464	19604.8	61.62339	19604.38	61.6235	19603.16
19608.84	61.87544	19609.87	61.87333	19617.09	61.87097	19611.46	61.88224	19603.97	61.87158	19604.31	61.87151	19604.34
19607.37	62.12142	19617.13	62.12856	19609.1	62.12443	19619.06	62.12145	19603.21	62.12212	19602.92	62.12188	19602.33
19612.87	62.37754	19606.54	62.37085	19608.68	62.3714	19615.63	62.37461	19603.87	62.37283	19601.76	62.37239	19606.78
19609.25	62.62343	19608.29	62.63084	19610.41	62.622	19617.65	62.62429	19602.89	62.62408	19604.52	62.62469	19605.45
19608.39	62.87735	19607.97	62.87288	19617.96	62.87281	19611.43	62.88025	19606.2	62.87344	19605.67	62.87302	19602.67
19610.77	63.12144	19612.68	63.12789	19613.46	63.12583	19608.56	63.12278	19602.84	63.12214	19603.6	63.12237	19603.63
19610.68	63.37709	19611.32	63.37242	19612.48	63.37162	19608.3	63.37428	19604.21	63.37253	19604.36	63.37238	19604.33
19612.69	63.62321	19608.69	63.62817	19610.19	63.62373	19611.33	63.62186	19602.46	63.62359	19602.32	63.62406	19603.57
19611.7	63.87747	19611.5	63.87235	19607.63	63.87257	19615.25	63.88044	19604.04	63.87364	19604.08	63.87341	19605.18
19612.91	64.12221	19612.46	64.12915	19612.15	64.1255	19614.08	64.12357	19603.39	64.12397	19602.9	64.12245	19604.32
19608.34	64.37884	19614.62	64.37255	19614.25	64.37345	19614.32	64.37562	19600.45	64.37212	19604.99	64.37303	19604.13
19612.67	64.62186	19610.82	64.63123	19613.61	64.62093	19612.94	64.62121	19601.8	64.6218	19603.09	64.62334	19601.98
19613.38	64.87633	19612.61	64.87068	19610.72	64.86958	19607.46	64.88261	19603.13	64.87236	19603.78	64.87245	19604
19610.43	65.12216	19612.56	65.12557	19611.49	65.12311	19611.36	65.12264	19602.8	65.12321	19603.35	65.1226	19606.26
19612.85	65.37668	19604.83	65.37088	19611.05	65.37204	19612.95	65.37643	19602.97	65.37204	19607.71	65.37206	19603.11
19612.1	65.62148	19604.85	65.63294	19613.74	65.62422	19610.46	65.62069	19605.08	65.62192	19603.66	65.62404	19605.67
19606.47	65.87774	19610.92	65.87244	19614.5	65.87213	19616.11	65.89503	19602.73	65.87205	19604.57	65.87176	19603.28
19611.31	66.12227	19615.56	66.1267	19609.43	66.12229	19607.76	66.12344	19603.24	66.12299	19605.12	66.12397	19603.68
19612.95	66.37506	19608.71	66.37085	19612.75	66.37165	19613.26	66.37585	19602.32	66.37396	19603.41	66.37368	19604.85
19609.65	66.62261	19612.65	66.62983	19613.2	66.62387	19611.55	66.62436	19604.83	66.62351	19602.57	66.62333	19604.65
19611.04	66.87878	19615.4	66.87172	19610.55	66.87336	19613.26	66.88057	19604.49	66.87199	19603.24	66.87235	19602.8
19612.54	67.12029	19612.17	67.12774	19612.8	67.12484	19613.07	67.12177	19603.37	67.1221	19604.84	67.12373	19603.21
19610.72	67.37749	19612.02	67.37236	19610	67.37087	19619.3	67.37632	19602.61	67.37523	19604.15	67.37489	19604.03
19608.61	67.62236	19609.76	67.63271	19608.84	67.62429	19616.17	67.62326	19602.12	67.6248	19608.95	67.62348	19605.13
19608.6	67.87757	19609.04	67.87131	19611.28	67.87979	19615.7	67.88334	19604.6	67.87529	19603.63	67.87243	19604.63
19608.7	68.12148	19611.76	68.12733	19607.71	68.12533	19615.07	68.13155	19603.1	68.12265	19605.15	68.12288	19605.07
19608.38	68.3779	19611.11	68.3724	19609.98	68.37139	19616.68	68.37653	19603.73	68.37321	19605.03	68.37413	19606.22
19609.82	68.62263	19612.8	68.6324	19610.13	68.62312	19615.83	68.62308	19604.65	68.62393	19604.48	68.62677	19604.18

19610.65	68.8788	19611.81	68.87322	19615.26	68.87172	19612.24	68.88239	19602.27	68.87436	19602.9	68.87215	19604.27
19609.25	69.12333	19615.75	69.12589	19611.85	69.12557	19613.24	69.1246	19603.09	69.12345	19605.82	69.1227	19603.61
19611.18	69.38046	19612.27	69.37167	19612.13	69.37339	19613.04	69.37514	19602.61	69.37288	19603.03	69.37384	19606.28
19611.77	69.62209	19609.24	69.63121	19610.9	69.62539	19617.85	69.62192	19601.61	69.6229	19608.74	69.62518	19602.62
19611.26	69.87803	19612.37	69.87248	19609.31	69.87261	19612.14	69.88128	19602.12	69.87418	19605.42	69.87353	19604.97
19606.89	70.12264	19615.12	70.12821	19613.3	70.12432	19614.51	70.12425	19603.52	70.12452	19605.37	70.12214	19604.06
19607.38	70.37986	19611.13	70.37246	19612.68	70.3732	19615.47	70.37846	19603.89	70.37537	19605.05	70.37381	19605.01
19612.9	70.62554	19613.46	70.63297	19611.86	70.62565	19612.7	70.62327	19602.51	70.62382	19604.26	70.62497	19606.27
19612.39	70.87953	19609.31	70.87368	19611.91	70.87425	19616.13	70.88241	19600.51	70.87326	19605.46	70.87504	19604.14
19611.44	71.12335	19616.22	71.12812	19614.63	71.12328	19611.71	71.12478	19607.66	71.13453	19602.85	71.12423	19602.3
19605.43	71.37904	19608.15	71.37205	19614.18	71.37213	19608.6	71.37781	19604.24	71.37474	19604.19	71.37309	19603.52
19610.36	71.62375	19607.83	71.63393	19612.81	71.62659	19615.96	71.62777	19604.33	71.62258	19605.11	71.62407	19602.31
19613.16	71.88014	19612.57	71.8739	19606.69	71.87319	19617.86	71.88382	19603.83	71.87304	19604.07	71.87437	19604.92
19609.28	72.12393	19607.01	72.12756	19612.49	72.12617	19606.93	72.12425	19602.78	72.12364	19602.07	72.12508	19604.57
19611.51	72.37819	19609.85	72.37289	19614.22	72.37214	19613.83	72.37576	19603	72.37668	19605.56	72.37487	19605.41
19613.77	72.62403	19606.23	72.63536	19612.13	72.62343	19608.37	72.62489	19603.08	72.62519	19602.78	72.62382	19605.99
19611.38	72.88118	19611.37	72.8746	19614.88	72.87469	19606.84	72.8837	19603.13	72.87407	19605	72.87485	19604.3
19604.8	73.12496	19610.6	73.12923	19609.71	73.12552	19617.2	73.12446	19601.83	73.12396	19604.16	73.12463	19605.03
19610.84	73.37984	19608.93	73.37531	19606.07	73.37359	19612.22	73.37597	19603.72	73.37489	19607.51	73.37597	19605.06
19612.02	73.62395	19608.64	73.63492	19604.57	73.62521	19614.97	73.62487	19604.21	73.62565	19605.7	73.62513	19603.72
19612.77	73.88069	19612.33	73.87345	19613.05	73.87304	19607.05	73.8841	19599.7	73.88702	19604.13	73.87387	19605.97
19609.78	74.12583	19616.24	74.1312	19616.48	74.12635	19613.51	74.12559	19600.89	74.12588	19603.65	74.12498	19602.08
19612.51	74.40008	19609.63	74.37517	19609.65	74.37502	19610.68	74.37801	19603.05	74.37494	19604.49	74.37513	19605.07
19607.06	74.62654	19611.2	74.63393	19611.21	74.62628	19614.72	74.62408	19601.31	74.62628	19605.86	74.62552	19604.64
19607.47	74.88044	19615.05	74.87583	19608.1	74.87532	19614.95	74.88479	19604.63	74.87688	19603.74	74.87432	19602.31
19607.25	75.12484	19611.11	75.12856	19615	75.12566	19615.11	75.12634	19603.54	75.12725	19603.22	75.12544	19603.81
19608.75	75.37974	19610.98	75.37476	19611.23	75.37478	19614.46	75.37789	19602.51	75.37612	19602.61	75.37559	19602.43
19610.09	75.62663	19613.31	75.63723	19612.14	75.62498	19610.98	75.62466	19602.34	75.62564	19605.27	75.62593	19605.14
19609.33	75.88304	19613.58	75.87562	19612.67	75.87729	19612.38	75.88569	19601.75	75.87647	19603.76	75.88183	19606.54
19605.17	76.12452	19611.36	76.12843	19612.85	76.12756	19613	76.12548	19598.85	76.12628	19603.56	76.12448	19602.69
19608.99	76.37922	19615.41	76.3755	19612.32	76.3735	19612.54	76.37866	19603.23	76.37738	19604.56	76.37431	19605.04
19609.96	76.6251	19605.82	76.63651	19612.53	76.62676	19610.6	76.62799	19603.89	76.62595	19605.93	76.62494	19604.07
19611.06	76.88272	19613.59	76.87629	19611.57	76.87464	19614.87	76.88145	19601.79	76.87754	19604.73	76.87662	19603.62
19606.04	77.12664	19606.82	77.13054	19615.04	77.12788	19611.88	77.12561	19601.68	77.12703	19604.44	77.12704	19603.92
19608.14	77.38102	19613.81	77.37459	19612.35	77.37618	19607.41	77.37851	19602.17	77.37649	19599.92	77.37698	19605.75
19604.03	77.62426	19607.39	77.63457	19615.81	77.62492	19608.07	77.62769	19601.86	77.62558	19603.75	77.62505	19603.67
19606.71	77.88075	19609.88	77.87502	19605.48	77.87527	19611	77.88424	19603.56	77.87773	19604.2	77.87649	19600.94
19608.32	78.12716	19604.46	78.13044	19614.25	78.12614	19608.51	78.12574	19603.88	78.12798	19604.62	78.12681	19602.63
19612.78	78.38239	19606.98	78.37624	19613.47	78.37692	19617.77	78.37922	19602.14	78.37813	19603.43	78.37836	19600.78
19612.49	78.62698	19614.48	78.63379	19602.19	78.62776	19612.23	78.62693	19602.46	78.62591	19603.82	78.62733	19601.78
19609.77	78.88272	19611.64	78.87436	19609.3	78.87461	19613.08	78.88461	19602.19	78.87536	19602.86	78.87634	19606.31
19615.54	79.12599	19613.86	79.13022	19610.22	79.12792	19609.4	79.12586	19602.41	79.12667	19605.11	79.12617	19604.58
19609.51	79.38802	19612.81	79.37657	19616.33	79.37694	19618.34	79.38132	19603.03	79.37883	19604.23	79.37746	19604.63
19614.18	79.62694	19611.83	79.641	19610.34	79.62838	19611.92	79.62762	19605.11	79.62771	19602.3	79.62822	19603.82
19610.89	79.8816	19615.39	79.88414	19610.15	79.87529	19614.17	79.88311	19601.69	79.87678	19605.85	79.87802	19605.36
19609.33	80.12518	19607.9	80.12998	19612.95	80.12819	19611.95	80.12797	19606.84	80.12698	19602.7	80.12626	19602.47
19612.09	80.38275	19610.76	80.37788	19614.34	80.37701	19612.27	80.38197	19606.25	80.37956	19603.54	80.3789	19604.66
19608.41	80.62774	19613.19	80.63799	19607.45	80.63769	19613.76	80.62809	19603.57	80.6288	19602.45	80.62784	19604.19
19611.25	80.88382	19614.32	80.87669	19605.91	80.87725	19612.05	80.88696	19600.55	80.87807	19603.17	80.87908	19605.32
19611.49	81.12732	19616.91	81.13316	19609.52	81.12955	19619.65	81.12761	19604.03	81.12693	19604.32	81.12767	19606.9
19611.23	81.38335	19612.39	81.37643	19609.53	81.3763	19620.72	81.37964	19603.33	81.37764	19601.73	81.37758	19603.43
19607.72	81.62574	19615.56	81.65135	19616.27	81.62797	19612.98	81.63026	19602.4	81.62817	19604.67	81.62739	19603.93
19609.7	81.88328	19617.28	81.87666	19612.76	81.87685	19618.27	81.8872	19601.17	81.87944	19601.12	81.87856	19604.87
19611.81	82.12734	19608.96	82.13149	19613.84	82.1281	19612.58	82.12857	19602.41	82.1273	19604.05	82.12964	19603.52
19609.74	82.38266	19611.18	82.37728	19617.11	82.37939	19615.89	82.38024	19602.04	82.37819	19605.89	82.38053	19604.58
19609.18	82.62711	19606.66	82.6358	19612.84	82.62845	19613.17	82.62646	19601.7	82.62785	19604.96	82.62672	19604.15
19612.28	82.88211	19606.34	82.87657	19610.86	82.8765	19616.68	82.8867	19602.73	82.87859	19605.74	82.87781	19600.53
19609.5	83.12662	19611.67	83.13273	19609.11	83.12693	19610.92	83.12864	19603.09	83.13011	19605.27	83.12982	19601.8
19607.37	83.38344	19610.75	83.37919	19610.92	83.37964	19614.73	83.38163	19602.44	83.38081	19601.73	83.37955	19604.55
19609.84	83.62977	19615.82	83.63649	19610.73	83.63106	19613.93	83.62846	19602.55	83.62944	19602.6	83.62837	19602.95
19608.88	83.88907	19609.24	83.87655	19612.85	83.87819	19613.42	83.88751	19602.21	83.87807	19605.72	83.8792	19604.5
19615.21	84.126	19607.56	84.13261	19616.31	84.12729	19615.12	84.12842	19601.52	84.13066	19602.63	84.12764	19604.62
19606.3	84.38717	19612.46	84.37886	19614.96	84.3785	19621.32	84.38133	19601.2	84.38077	19604.14	84.3799	19603.23
19612.26	84.62958	19606.1	84.6358	19616.8	84.6308	19606.7	84.63021	19604.94	84.64135	19603.65	84.63007	19604.14
19611.84	84.88413	19611.47	84.8764	19608.69	84.87918	19612.35	84.89107	19603.66	84.889	19605.82	84.88017	19605.02
19611.96	85.13956	19610.79	85.13235	19607.29	85.13035	19610.72	85.12788	19604.44	85.12862	19604.8	85.12763	19604.1
19608.23	85.38364	19610.97	85.37893	19609.28	85.37848	19615.58	85.38035	19602.53	85.38079	19605.43	85.37904	19605
19611.12	85.62831	19611.4	85.63831	19612.32	85.62985	19615.08	85.63128	19601.91	85.62993	19604.49	85.62936	19602.44
19608.06	85.88477	19611.26	85.88105	19612.52	85.87845	19612.95	85.88757	19605.25	85.88223	19606.17	85.88098	19603.74
19611.96	86.12914	19609.54	86.13654	19614.8	86.13057	19613.63	86.1283	19602.5	86.1366	19604.3	86.12896	19602.45

19609.75	86.38367	19613.65	86.37892	19608.65	86.37922	19614.31	86.384	19602.86	86.37905	19603.32	86.37891	19605.22
19610.47	86.62824	19613.23	86.64632	19611.77	86.63089	19608.98	86.63143	19602.07	86.63002	19601.77	86.62942	19605.99
19614.25	86.88676	19614.15	86.87916	19609.85	86.87916	19610.7	86.88579	19601.06	86.88199	19604.7	86.88048	19606.07
19610.27	87.1302	19611.74	87.13423	19611	87.13131	19612.56	87.12978	19600.85	87.13161	19604.22	87.13077	19604.52
19607.74	87.38661	19615.39	87.37996	19610.83	87.38531	19617.89	87.38205	19602.62	87.38039	19603.09	87.38059	19601.41
19609.74	87.62826	19605.61	87.63905	19611.66	87.62957	19613.92	87.63182	19604.56	87.62896	19605.95	87.6286	19603.66
19606.72	87.8855	19611.48	87.87798	19617.23	87.87856	19611.6	87.88645	19600.51	87.87981	19603.59	87.88048	19600.62
19610.56	88.12952	19609.47	88.13421	19610.44	88.1321	19610.02	88.1309	19603.17	88.1313	19603.49	88.13109	19603.68
19611.15	88.3852	19609.5	88.38262	19617.06	88.38041	19615.87	88.38462	19599.89	88.38126	19605.55	88.38227	19604.48
19608.89	88.63112	19611.96	88.6367	19609.4	88.63371	19611.34	88.62949	19602.41	88.63027	19603.99	88.6294	19604.23
19610.82	88.88577	19609.24	88.87865	19611.1	88.87712	19615.02	88.88677	19603.03	88.87943	19603.28	88.87982	19602.13
19610.42	89.12962	19608.05	89.13371	19610.7	89.13149	19607.83	89.13094	19601.45	89.12983	19604.09	89.1304	19602.6
19609.47	89.38916	19613.44	89.38101	19611.37	89.37935	19614.89	89.38258	19601.85	89.38105	19604.53	89.38045	19607.04
19612.39	89.63022	19614.15	89.63671	19611.45	89.63105	19610.59	89.63144	19602.19	89.63168	19602.32	89.63067	19602.8
19606.84	89.8876	19611.47	89.87909	19610.97	89.87966	19615.47	89.89616	19601.75	89.88078	19606.32	89.88117	19602.28
19604.37	90.13028	19613.14	90.13393	19613.34	90.13232	19605.25	90.12891	19602.1	90.13056	19604.23	90.12975	19603.77
19611.79	90.38633	19612	90.38026	19611.45	90.37968	19612.51	90.38233	19601.22	90.38022	19603.74	90.3823	19603.31
19615.31	90.63083	19612.05	90.6391	19606.73	90.63009	19613.34	90.63097	19602.52	90.63159	19603.81	90.63164	19601.42
19608	90.8882	19613.62	90.88126	19605.79	90.87974	19613.23	90.89279	19601.09	90.8825	19602.47	90.8812	19602.17
19605.51	91.13211	19611.18	91.13699	19613.24	91.13262	19611.7	91.1299	19601.43	91.13023	19602.13	91.12989	19605.27
19610.51	91.3859	19616.89	91.3808	19609.96	91.38075	19609.93	91.38173	19601.55	91.38062	19603.04	91.38023	19603.87
19611.32	91.62911	19610.57	91.63971	19609	91.6339	19612.02	91.63448	19602.84	91.63088	19603.37	91.63202	19603.18
19609.4	91.88644	19605.51	91.88129	19613.46	91.8805	19611.67	91.88954	19599.18	91.88265	19602.33	91.88181	19605.52
19609.8	92.13299	19614.8	92.13639	19611.89	92.13198	19608.21	92.13229	19600.84	92.13269	19605.6	92.13075	19603.34
19608.14	92.38728	19607.26	92.38121	19612.8	92.38006	19617.04	92.38367	19603.4	92.38085	19603.36	92.37946	19602.39
19607.25	92.63198	19611.68	92.63866	19611.69	92.6427	19609.13	92.63135	19602.66	92.63145	19600.6	92.6307	19604.76
19608.16	92.88856	19611.09	92.87996	19611.88	92.88308	19613.13	92.88938	19601.19	92.88186	19601.93	92.88325	19603.96
19607.36	93.13056	19604.41	93.13572	19609.36	93.13451	19613.85	93.13223	19602.33	93.13209	19603.61	93.13244	19601.63
19606.62	93.38591	19612.87	93.38169	19607.71	93.38025	19614.4	93.38601	19602.26	93.38227	19603.89	93.3818	19603.6
19610.01	93.63266	19608.83	93.63939	19615.08	93.6314	19614.71	93.63103	19603.6	93.63219	19604.79	93.63108	19604.49
19607.74	93.88718	19613.24	93.87998	19610.21	93.88126	19610.81	93.88727	19601.3	93.88184	19602.21	93.88165	19605.17
19607.92	94.13115	19612.01	94.13575	19612.74	94.13305	19612.49	94.13241	19599.73	94.13313	19603.23	94.13403	19602.25
19605.56	94.38876	19615.08	94.38206	19606.58	94.38219	19610.35	94.38473	19601.57	94.38301	19603.28	94.38393	19605.03
19608.87	94.63104	19608.92	94.64281	19610.85	94.6338	19611.15	94.63285	19600.3	94.63384	19603.86	94.63199	19604.02
19610.67	94.88923	19609.41	94.88176	19613.98	94.87971	19612.11	94.89561	19601.96	94.88156	19602.93	94.88103	19601.99
19612.62	95.13348	19605.64	95.13542	19610.12	95.13336	19619.73	95.14556	19600.72	95.13317	19603.07	95.13328	19604.86
19608.32	95.38669	19603.46	95.38104	19608.88	95.38258	19613.7	95.38615	19602.23	95.38358	19605.07	95.3846	19602.13
19612.35	95.6308	19611.87	95.64232	19606.71	95.63359	19606.95	95.63313	19600.23	95.63532	19603.35	95.63339	19599.77
19613.59	95.88813	19613.72	95.88199	19604.92	95.88275	19615.23	95.89093	19601.58	95.8837	19603.49	95.88229	19602.72
19608.35	96.1331	19607.92	96.13896	19612.9	96.13136	19609.22	96.13366	19601.72	96.13217	19604.51	96.1325	19600.73
19605.57	96.38777	19611.76	96.38061	19608.3	96.38017	19614.72	96.3836	19600.79	96.38249	19602.24	96.3844	19600.59
19608.1	96.63311	19606.96	96.64076	19606.34	96.63622	19603.54	96.63406	19602.94	96.63434	19603.11	96.63393	19604.14
19609.93	96.88781	19608.97	96.88215	19603.9	96.88273	19615.33	96.8904	19602.31	96.8843	19604.85	96.8833	19603.11
19608.17	97.13354	19609.88	97.13748	19607.93	97.13299	19615.02	97.13397	19601.72	97.13405	19604.29	97.13272	19600.95
19608.7	97.38876	19612.81	97.38267	19608.3	97.38104	19614.32	97.38567	19598.87	97.38209	19599.49	97.38378	19602.7
19608.89	97.63331	19612.61	97.64152	19603.04	97.63258	19611.91	97.63219	19601.58	97.63248	19603.48	97.63465	19603.44
19607.13	97.88988	19610.88	97.8817	19608.94	97.88254	19609.04	97.88947	19601.87	97.8845	19603.79	97.88397	19604.07
19603.09	98.13375	19610.28	98.13798	19609.7	98.1377	19615.29	98.13439	19598.2	98.13589	19602.04	98.13223	19602.83
19610.31	98.38858	19610.77	98.38464	19607.93	98.38303	19611.83	98.38617	19600.03	98.38433	19601.7	98.38335	19600.1
19610.58	98.63394	19610.5	98.6416	19615.95	98.63415	19612.18	98.63425	19600.58	98.64647	19602.94	98.63499	19602.37
19605.95	98.90095	19605.86	98.88149	19611.23	98.8844	19614.03	98.8908	19603.15	98.88424	19606.04	98.88501	19604.17
19609.61	99.13505	19614.66	99.13738	19604.36	99.13539	19616.47	99.1331	19601.74	99.13432	19602.61	99.13507	19602.29
19605	99.38904	19606.71	99.38507	19601.46	99.38307	19615.2	99.38674	19601.61	99.38548	19600.27	99.39723	19601.95
19612.22	99.63375	19613.54	99.64084	19611.71	99.63598	19611.73	99.63411	19598.3	99.63611	19601.2	99.63359	19602.68
19610.06	99.8892	19610.59	99.88449	19607.21	99.8815	19606.16	99.89028	19601.06	99.8846	19602.03	99.88291	19602.98
19608.41	100.1354	19608.6	100.1396	19611.8	100.1363	19611.23	100.1333	19598.31	100.1365	19605.98	100.1361	19602.06
19604.81	100.392	19604.2	100.3847	19604.52	100.3843	19607.13	100.3889	19599.21	100.386	19601.07	100.3873	19601.31
19607.18	100.6361	19604.58	100.643	19606.77	100.6477	19607.85	100.6373	19598.9	100.6376	19602.26	100.6361	19602.36
19606.9	100.8912	19609.95	100.8865	19609.38	100.8858	19605.85	100.8977	19598.32	100.8879	19601.7	100.8859	19602.13
19609.93	101.1378	19609.73	101.1415	19611.74	101.1378	19609.12	101.1357	19600.51	101.1366	19602.35	101.1356	19602.39
19607.73	101.3949	19607.36	101.3836	19608.67	101.3847	19612.46	101.388	19598.98	101.3873	19604.04	101.3872	19601.99
19602.34	101.6352	19615.3	101.6433	19611.41	101.6369	19606.77	101.6389	19602.86	101.6372	19601.73	101.6378	19602.07
19604.77	101.8933	19609.69	101.8865	19604.8	101.887	19611.61	101.8969	19601.45	101.8878	19602.46	101.8865	19602.52
19609.05	102.1344	19611.56	102.1396	19613.77	102.1394	19613.33	102.1381	19599.85	102.1386	19603.53	102.1362	19602.15
19606.03	102.3915	19608.09	102.386	19611.25	102.3877	19610.75	102.3911	19599.81	102.3874	19598.21	102.3879	19602.35
19602.17	102.6376	19606.49	102.6454	19612.06	102.64	19611.7	102.6348	19601.42	102.6358	19600.06	102.6378	19603.76
19609.3	102.8932	19607.59	102.8863	19609.18	102.8847	19602.1	102.8921	19600.77	102.8887	19599.38	102.8879	19600.6
19601.39	103.137	19612.4	103.1408	19608.72	103.1373	19609.09	103.1391	19598.37	103.1377	19601.33	103.1375	19599.37
19605.51	103.3928	19609.89	103.3872	19606.23	103.3883	19613.76	103.3916	19599.11	103.3888	19601.04	103.3876	19603.23
19606.89	103.6372	19608.27	103.6441	19611.43	103.6504	19615	103.6375	19600.1	103.6372	19604.4	103.6374	19601.77

19604.79	103.8941	19607.85	103.8856	19604.36	103.8876	19607.29	103.8942	19598.4	103.8864	19601.74	103.8894	19601.5
19607.41	104.138	19606.56	104.1412	19608.8	104.1371	19611.88	104.1375	19599.71	104.1381	19602.75	104.1388	19598.46
19606.88	104.3952	19604.33	104.3866	19608.43	104.3876	19606.93	104.3922	19598.63	104.3883	19603.4	104.3876	19602.96
19611.04	104.6363	19608.05	104.6486	19608.73	104.6557	19610.84	104.6379	19599.61	104.6391	19597.32	104.6369	19602.1
19606.02	104.8929	19607.33	104.8848	19609.97	104.887	19606.46	104.8933	19599.47	104.8872	19600.6	104.8883	19602.49
19600.15	105.1383	19606.99	105.1408	19608.68	105.1394	19612.76	105.1381	19600.05	105.1372	19603.49	105.1397	19602.68
19605.04	105.3943	19613.72	105.386	19605.95	105.3872	19607.24	105.3902	19599.33	105.3894	19598.7	105.3892	19600.48
19608.68	105.6367	19608.56	105.6487	19608.76	105.6401	19614.11	105.6391	19600.64	105.6416	19602.6	105.6381	19602.32
19602.7	105.8935	19609.61	105.8877	19600.91	105.8875	19606.77	105.8939	19598.91	105.8901	19598.44	105.8945	19602.17
19606.38	106.1371	19608.7	106.1439	19607.86	106.1392	19608.93	106.1394	19600.1	106.1376	19600.82	106.1399	19601.44
19607.69	106.3923	19606.59	106.3886	19611.96	106.3889	19611.86	106.391	19599.98	106.3887	19600.89	106.389	19602.52
19605.7	106.6385	19603.6	106.6488	19606.68	106.6415	19611.33	106.6437	19598	106.6424	19601.66	106.6409	19601.15
19607.99	106.8953	19613.08	106.8871	19608.84	106.8876	19607.51	106.8977	19600.74	106.8898	19603.12	106.8888	19599.79
19605.16	107.1375	19611.76	107.1445	19603.12	107.1388	19613.09	107.1375	19597.82	107.1387	19602.06	107.1393	19600.25
19606.23	107.3942	19608.21	107.3878	19608.99	107.3904	19608.52	107.3924	19599.15	107.3885	19600.26	107.3895	19600.87
19606.81	107.6402	19606.24	107.6462	19604.61	107.6433	19604.57	107.6407	19598.89	107.6405	19602.51	107.6396	19602.34
19600.87	107.8966	19605.43	107.8869	19607.33	107.8884	19609.7	107.8978	19599.2	107.8908	19600.58	107.8897	19600.23
19609.39	108.1383	19608.12	108.1442	19604.15	108.1386	19610.59	108.1405	19600.52	108.1398	19598.6	108.1397	19597.86
19602.81	108.3922	19605.35	108.3895	19609.47	108.3876	19610.31	108.3931	19598.42	108.3911	19599.23	108.3902	19599.08
19610.13	108.6388	19607.02	108.6488	19606.14	108.6439	19605.25	108.6383	19600.54	108.6415	19600.22	108.6399	19602.02
19604.26	108.8956	19607.75	108.8889	19604.03	108.8892	19609.93	108.8978	19601.15	108.8904	19601.61	108.8907	19600.21
19606.75	109.1396	19606.96	109.1423	19604.39	109.1398	19606.17	109.141	19600.46	109.141	19598.28	109.1421	19598.79
19604.19	109.4002	19606.2	109.3876	19606.85	109.3886	19608.19	109.3945	19598.68	109.3895	19596.46	109.3901	19599.15
19605.96	109.6396	19606.47	109.65	19604.45	109.6405	19600.2	109.6386	19597.36	109.6422	19601.59	109.6392	19599.04
19611.13	109.8959	19607.68	109.8894	19601.8	109.8896	19603.98	109.8986	19598.57	109.8897	19598.38	109.8911	19601.26
19606.21	110.14	19609.29	110.1432	19604.25	110.1402	19611.38	110.1419	19600.89	110.1409	19597.43	110.1415	19601.09
19604.37	110.3945	19605.95	110.3883	19603.55	110.3899	19611.59	110.3954	19599.34	110.3914	19602.2	110.3923	19599.48
19604.89	110.6395	19606.39	110.6484	19607.95	110.6401	19608.28	110.6403	19595.36	110.6413	19599.5	110.639	19601.4
19601.8	110.896	19602.97	110.8903	19606.2	110.8884	19604.96	110.8958	19599.25	110.89	19599.64	110.8905	19603.35
19603.84	111.1418	19605.19	111.1466	19609.48	111.1407	19620.45	111.1402	19600.15	111.1399	19600.96	111.1415	19599.63
19602.15	111.3949	19600.94	111.3908	19601.8	111.3917	19607.25	111.3927	19599.52	111.3924	19599.77	111.3911	19601.23
19598.76	111.6402	19606.47	111.6517	19606.97	111.6442	19605.67	111.6433	19599.17	111.6471	19597.5	111.6435	19600.48
19603.87	111.8973	19605.18	111.9023	19608.87	111.8902	19602.67	111.8954	19600.27	111.893	19598.43	111.8893	19594.81
19605.18	112.1388	19604.88	112.1449	19601.99	112.1404	19603.19	112.1409	19598.82	112.1403	19598.41	112.1419	19602.78
19604.33	112.3962	19608.95	112.3899	19603.53	112.3907	19606.23	112.3923	19597	112.3914	19598.09	112.3918	19601.73
19607.57	112.6417	19607.33	112.6487	19605.07	112.6438	19609.26	112.6416	19598.45	112.643	19600.95	112.6418	19599.99
19602.02	112.8968	19608.66	112.8905	19607.29	112.8917	19606.06	112.8979	19597.06	112.8935	19602.46	112.8926	19602.31
19602.52	113.1403	19610.09	113.1442	19606.33	113.1418	19610.58	113.1401	19595.61	113.1407	19597.09	113.141	19599.61
19604.12	113.3964	19606.14	113.3897	19600.61	113.3897	19604.97	113.3956	19597.67	113.3899	19601.85	113.3913	19599.41
19605.81	113.64	19609.09	113.6474	19609.61	113.6398	19608.83	113.6416	19596.36	113.6411	19597.53	113.6416	19601.11
19603.95	113.8969	19610.6	113.8911	19603.95	113.8917	19608.74	113.8999	19597.5	113.8939	19600.5	113.8931	19597.65
19599.72	114.1415	19603.89	114.1458	19599.2	114.1432	19603.62	114.1423	19601.17	114.1431	19598.69	114.1404	19597.53
19600.39	114.3995	19610.87	114.3899	19602.35	114.3967	19606.96	114.394	19600.22	114.3925	19600.28	114.3916	19602.09
19603.67	114.64	19604.34	114.6474	19604.16	114.6421	19605.09	114.6424	19600.57	114.6415	19598.88	114.6415	19601.3
19603.17	114.8978	19604.18	114.8906	19605.01	114.8909	19607.59	114.9	19597.49	114.8921	19601.27	114.8933	19599.29
19600.9	115.1411	19606.19	115.146	19600.39	115.1427	19606.34	115.143	19599.49	115.1446	19598.86	115.1473	19598.98
19600.6	115.3978	19601.17	115.3906	19600.55	115.3945	19601.02	115.3966	19600.01	115.3929	19602.14	115.3932	19601.14
19600.92	115.6412	19609.51	115.6494	19600.44	115.6565	19602.58	115.6413	19598.42	115.6434	19595.98	115.6412	19603.13
19604.72	115.8996	19609.71	115.8897	19607.52	115.8928	19601.51	115.8985	19595.57	115.8918	19600.59	115.8965	19598.15
19603.6	116.1434	19602.65	116.1493	19600.38	116.1415	19606.13	116.1425	19597.67	116.1443	19601.85	116.1431	19599.12
19598.84	116.3982	19600.13	116.3916	19602.38	116.3907	19603.88	116.3968	19600.04	116.3945	19599.68	116.3941	19604.32
19606.55	116.6425	19604.22	116.6502	19605.05	116.6436	19608.03	116.6444	19601.44	116.6451	19599.23	116.6417	19598.48
19600.03	116.8996	19601.8	116.8928	19606.91	116.8924	19603.87	116.8987	19597.76	116.896	19598.74	116.8923	19597.9
19601.77	117.1413	19605.43	117.147	19603.02	117.1437	19604.92	117.1595	19595.97	117.1421	19599.08	117.1439	19598.16
19598.74	117.3991	19601.44	117.3927	19601.46	117.3904	19609.35	117.3956	19597.69	117.3931	19599.07	117.3944	19597.35
19602.25	117.6414	19602.51	117.6499	19604.22	117.6436	19601.38	117.6457	19598.36	117.6449	19597.38	117.6449	19601.16
19601.09	117.8992	19602.98	117.8927	19605.01	117.8924	19610.23	117.8998	19597.21	117.8978	19597.71	117.8923	19600.52
19598.96	118.1433	19604.75	118.1474	19600.34	118.1452	19607.96	118.1419	19597.11	118.1438	19599.76	118.1436	19598.21
19599.54	118.3999	19601.55	118.3908	19604.58	118.3922	19612.41	118.3953	19596.2	118.3928	19600.84	118.3953	19597.84
19599.74	118.6413	19604.03	118.6521	19604.54	118.6424	19607.15	118.6435	19597.29	118.647	19598.42	118.6536	19598.98
19595.72	118.8977	19603.97	118.8924	19602.38	118.893	19607.6	118.9032	19598.23	118.8955	19596.87	118.9037	19597.38
19602.57	119.1434	19600.61	119.1491	19605.37	119.145	19604.86	119.1442	19598.22	119.1456	19598.49	119.1465	19599.63
19599.74	119.4016	19600.16	119.3936	19599.09	119.3939	19611.02	119.3957	19599.07	119.3944	19600.45	119.3938	19601.2
19601.65	119.6463	19605.42	119.6572	19602.75	119.6514	19601.89	119.6477	19596.94	119.6499	19599.79	119.6485	19601.2
19601.87	119.9006	19607.38	119.8924	19599.14	119.893	19602.17	119.9005	19595.64	119.8953	19599.4	119.8953	19598.39
19600.49	120.1436	19600.03	120.1493	19601.02	120.1451	19604.9	120.1454	19597.53	120.1446	19598.71	120.1436	19597.53
19598.53	120.3997	19601.48	120.3938	19599.99	120.3944	19596.59	120.3975	19597.95	120.3955	19598.38	120.3942	19598.24
19600.48	120.6423	19607.31	120.6498	19596.08	120.6538	19601.45	120.6416	19594.94	120.6442	19600.81	120.6417	19598.01
19600.76	120.9086	19605.03	120.8907	19598.47	120.8914	19607.64	120.8961	19595.28	120.8931	19599.12	120.8937	19597.95
19600.75	121.1463	19603.52	121.149	19598.82	121.1416	19602.9	121.1423	19596.85	121.1423	19597.32	121.1428	19599.87

19600.11	121.3982	19603.53	121.3905	19596.94	121.3918	19608.46	121.3952	19596.22	121.394	19598.07	121.3936	19599.45
19602.21	121.6437	19598.46	121.6499	19596.65	121.6437	19606.71	121.6461	19596.29	121.6468	19600.7	121.6421	19599.08
19602.13	121.8983	19604.19	121.8925	19607.68	121.8927	19602.48	121.8987	19598.01	121.8958	19598.52	121.8923	19599.12
19596.55	122.1534	19602.06	122.1472	19598.05	122.1432	19605.83	122.1428	19595.68	122.1448	19597.57	122.143	19598.17
19599.33	122.3982	19595.51	122.3917	19599.98	122.3907	19604.2	122.3952	19596.63	122.3921	19596.85	122.3937	19599.28
19594.85	122.6433	19602.17	122.6511	19602.93	122.6416	19603.06	122.6435	19600.62	122.6449	19593.45	122.6428	19599.25
19600.71	122.8981	19603.17	122.8937	19603.59	122.8926	19606.59	122.8992	19597.82	122.8946	19599.61	122.8929	19597.03
19601.7	123.142	19603.34	123.1471	19600.66	123.1442	19600.61	123.1427	19594	123.1433	19598.15	123.1476	19596.17
19599.31	123.3986	19598.46	123.3904	19602.14	123.3937	19603.49	123.3949	19594.53	123.3945	19597.1	123.3936	19599.35
19602.16	123.6418	19601.24	123.6506	19598.99	123.6413	19601.6	123.6439	19596.74	123.6441	19597.41	123.6457	19596.42
19602.02	123.8996	19601.14	123.8937	19603.25	123.8913	19597.06	123.8987	19597.53	123.8944	19597.84	123.8955	19598.21
19601.66	124.1411	19600.77	124.1475	19601.77	124.1443	19606.05	124.1443	19596.41	124.1445	19598.63	124.1433	19598.23
19599.92	124.4016	19602.81	124.3927	19599.79	124.3955	19603.47	124.3961	19598.09	124.3943	19599.02	124.396	19601.07
19595.07	124.6425	19600.94	124.6513	19603.33	124.644	19601.81	124.6415	19598.7	124.6454	19597.12	124.6461	19595.98
19598.14	124.9006	19600.76	124.8918	19597.61	124.8924	19604.49	124.9195	19596.78	124.8942	19597.94	124.8946	19596.22
19602.41	125.1435	19603.18	125.1492	19601.23	125.1436	19602.2	125.1434	19594.05	125.1439	19596.13	125.1424	19597.96
19599.66	125.3982	19599.68	125.394	19598.48	125.3928	19603.72	125.3983	19594.21	125.3956	19596.22	125.3936	19599.25
19596.37	125.6424	19604.29	125.6495	19599.27	125.6432	19603.24	125.6444	19595.47	125.6452	19594.96	125.6438	19598.93
19598.2	125.8998	19597.97	125.8931	19600.11	125.8948	19606.01	125.8978	19598.11	125.8932	19597.47	125.8952	19594.73
19599.92	126.1443	19598.12	126.1496	19601.16	126.1443	19603.18	126.1449	19596.08	126.144	19598.12	126.1443	19596.12
19600.03	126.3994	19604.31	126.394	19598.71	126.3919	19602.26	126.3965	19595.71	126.3949	19599.54	126.3954	19596.88
19597.63	126.6431	19600.43	126.6521	19597.42	126.6471	19597.99	126.6475	19597.75	126.6474	19600.3	126.6438	19599.39
19604.41	126.9041	19595.82	126.895	19600.42	126.8956	19600.13	126.9008	19598.23	126.8943	19596.45	126.8965	19599.17
19594.97	127.1429	19605.98	127.1501	19599.28	127.1458	19602.49	127.1447	19595.13	127.1437	19595.69	127.1453	19601.87
19595.85	127.3988	19603.38	127.3916	19597.41	127.3933	19597.08	127.3958	19596.1	127.394	19599.42	127.3997	19599.3
19598	127.6446	19597.12	127.6527	19599.66	127.6441	19602.1	127.6449	19595.09	127.6475	19600.13	127.6442	19598.7
19600.32	127.9031	19602.3	127.8967	19593.38	127.9069	19601.98	127.9011	19596.49	127.8968	19594.4	127.8952	19595.84
19598.46	128.1433	19602.86	128.1493	19601.1	128.1548	19602.12	128.1451	19593.87	128.1444	19595.27	128.1458	19595.84
19596.1	128.399	19600.51	128.3938	19601.73	128.3948	19597.38	128.3969	19596.76	128.396	19597.06	128.3994	19598.41
19603.14	128.6447	19601.04	128.654	19596.6	128.6465	19602.12	128.6452	19594.15	128.6461	19598.68	128.6468	19595.56
19602.64	128.901	19599.95	128.9075	19601.53	128.895	19600.44	128.9018	19596.46	128.8955	19595.51	128.8945	19600.16
19599.59	129.1446	19597.72	129.1498	19595.82	129.1457	19601.64	129.1475	19596.13	129.1455	19598.38	129.1485	19594.79
19596.9	129.4031	19595.28	129.3947	19596.61	129.3953	19600.24	129.3968	19596.9	129.3952	19598.79	129.3988	19595.01
19598.11	129.6431	19604	129.6528	19595.48	129.6491	19597.36	129.6449	19595.89	129.6624	19596.44	129.6466	19594.26
19593.56	129.903	19605.87	129.8954	19599.44	129.8958	19597.47	129.9004	19593.66	129.8961	19597.86	129.8953	19596.12
19600.43	130.1459	19600.29	130.1501	19598.19	130.1444	19599.27	130.1468	19595.07	130.1455	19595.03	130.1455	19599.89
19600.51	130.3998	19599.65	130.3938	19600.56	130.3965	19603.32	130.3986	19596.87	130.3961	19597.66	130.3997	19594.16
19597.19	130.6449	19597.17	130.6514	19599.91	130.6467	19597.14	130.6457	19592.66	130.6484	19596.08	130.6463	19595.56
19594.05	130.9017	19601.65	130.8959	19599.66	130.8964	19595.04	130.9055	19598.93	130.8955	19596.73	130.8964	19597.97
19595.81	131.1449	19600.54	131.1518	19599.15	131.1467	19598.06	131.1477	19597.98	131.1454	19597.01	131.1457	19593.04
19596.1	131.4026	19596.97	131.3924	19598.25	131.3959	19602.31	131.4001	19594.53	131.3963	19596.09	131.3983	19597.91
19593.1	131.6539	19601.88	131.6544	19595.71	131.6477	19601.75	131.6497	19595.69	131.6505	19596.15	131.6463	19597.02
19596.05	131.9015	19593.9	131.8965	19602.2	131.8986	19598.91	131.8983	19594.01	131.8977	19597.62	131.897	19595.5
19595.23	132.1438	19599.54	132.1517	19601.65	132.1473	19597.04	132.1505	19597.26	132.146	19598.31	132.147	19597.2
19594.58	132.4013	19606.3	132.395	19599.72	132.3968	19595.94	132.399	19597.74	132.396	19593.59	132.4	19594.33
19594.78	132.6459	19597.59	132.6542	19598.13	132.646	19599.88	132.6485	19595.19	132.6509	19594.64	132.6461	19596.29
19599.68	132.9023	19595.07	132.8958	19607.25	132.8973	19597.1	132.8989	19595.64	132.8985	19597.99	132.8981	19595.08
19594.51	133.154	19600.33	133.1511	19601.75	133.1467	19602.48	133.1477	19595.59	133.1462	19597.96	133.1476	19597.79
19594.11	133.401	19599.05	133.3959	19598.77	133.3964	19596.26	133.4009	19595.94	133.3961	19598.52	133.3992	19595.94
19591.17	133.6449	19598.3	133.6533	19601.49	133.6478	19601.23	133.648	19594.95	133.6866	19598.82	133.6458	19596.66
19595.28	133.9247	19602.79	133.8974	19599.46	133.8979	19602.35	133.9028	19598.27	133.9004	19596.14	133.897	19594.88
19597.37	134.1468	19598.41	134.1511	19598.08	134.1466	19598.63	134.149	19592.65	134.1626	19595.33	134.1483	19594.98
19594.82	134.4039	19595.56	134.3958	19597.96	134.3951	19600.08	134.4005	19594.44	134.3981	19593.1	134.4005	19596.85
19599.41	134.6466	19599.25	134.6565	19598.81	134.6488	19594.37	134.6487	19595.91	134.6501	19600.4	134.6476	19597.83
19600.45	134.9033	19599.74	134.898	19599.25	134.8986	19597.99	134.9051	19596.31	134.9002	19595.24	134.8961	19597.6
19595.11	135.1458	19598.04	135.1541	19593.44	135.1481	19598.77	135.1514	19594.87	135.1492	19595.39	135.1469	19595.1
19595.88	135.4025	19598.98	135.3948	19596.56	135.3968	19594.63	135.4028	19593.35	135.3975	19595.97	135.4013	19596.12
19595.98	135.647	19594.81	135.6536	19597.15	135.6468	19597.39	135.647	19595.65	135.6502	19595.89	135.6483	19594.58
19601.31	135.905	19594.05	135.8976	19597.03	135.8977	19602.14	135.9035	19596	135.8973	19599.39	135.8997	19596.74
19598.71	136.146	19596.71	136.1542	19595.2	136.1518	19600.05	136.1476	19598.49	136.1495	19596.94	136.1465	19598.47
19593.22	136.4035	19596.49	136.3989	19599.64	136.4007	19595.2	136.4025	19593.9	136.3988	19597.36	136.3989	19595.83
19593.99	136.6491	19599.04	136.6556	19594.73	136.6489	19604.16	136.6504	19598.47	136.6507	19597.15	136.6488	19594.39
19595.89	136.9041	19596.6	136.9059	19596.86	136.8981	19595.96	136.8987	19595.59	136.8974	19597.71	136.898	19596.79
19598.55	137.1474	19598.48	137.1529	19594.32	137.1503	19592.23	137.1483	19596.41	137.1485	19598.76	137.1475	19593.3
19595.85	137.4025	19600.65	137.3972	19595.57	137.4029	19602.4	137.4017	19594.01	137.3983	19597.65	137.3989	19594.22
19593.4	137.6524	19597.19	137.6559	19598.38	137.6493	19596.53	137.6488	19592.69	137.6531	19592.92	137.6479	19597.37
19598.48	137.9051	19595.76	137.8953	19598.72	137.8966	19596.86	137.9011	19594.19	137.8997	19593.4	137.8989	19597.09
19598.79	138.1482	19600.7	138.1533	19591.02	138.1475	19596.49	138.1505	19597.57	138.1488	19597.13	138.148	19594.82
19589.45	138.4047	19594.8	138.3971	19597.73	138.401	19602.89	138.4018	19594.09	138.3975	19596.46	138.4003	19594.1
19597.74	138.6484	19597.42	138.6551	19595.7	138.6656	19595.46	138.6493	19592.43	138.6524	19596.93	138.6496	19598.35

19592.13	138.9045	19597.34	138.8971	19590.16	138.8995	19600.57	138.9023	19594.05	138.9037	19594.93	138.8996	19596.16
19597.1	139.1472	19596.16	139.1526	19595.03	139.1512	19602.61	139.1478	19594.15	139.1504	19596.09	139.1504	19594.79
19593.02	139.4061	19594.35	139.3979	19597.07	139.3962	19603.59	139.4018	19595.98	139.3974	19598.35	139.4013	19595.37
19589.65	139.6517	19597.01	139.6574	19593.25	139.6511	19596.22	139.6498	19591.98	139.6501	19596.53	139.6484	19599.45
19597.25	139.9061	19595.82	139.8985	19597.01	139.8991	19599.24	139.9046	19591.92	139.9003	19595.61	139.8991	19596.71
19589.15	140.1471	19596.3	140.1544	19597.58	140.1504	19596.15	140.1492	19592.23	140.1517	19596.81	140.1503	19597.97
19590.96	140.405	19597.06	140.3973	19600.25	140.3983	19590.97	140.4036	19597.58	140.4001	19594.29	140.4032	19596.84
19596.01	140.651	19594.63	140.6563	19593.63	140.6481	19597.52	140.6497	19592.21	140.6534	19596.34	140.6485	19592.93
19594.39	140.9071	19597.83	140.8987	19595.87	140.8983	19590.14	140.9049	19594.69	140.8996	19593.65	140.8989	19596.1
19597.05	141.1471	19592.28	141.1559	19593.81	141.1511	19601.79	141.1501	19595.68	141.1536	19593.28	141.15	19596.93
19592.35	141.4051	19590.56	141.3995	19599.73	141.3992	19598.26	141.4029	19594.36	141.4019	19598.09	141.4055	19598.81
19594.97	141.6513	19598.42	141.6548	19600.49	141.6523	19597.51	141.6516	19592.5	141.6573	19595.09	141.651	19595.62
19594.08	141.9071	19593.79	141.919	19592.35	141.899	19597.77	141.9045	19593.71	141.9014	19597.77	141.8985	19595.79
19592.25	142.1506	19595.68	142.1558	19595.99	142.1519	19594.34	142.1531	19593.44	142.151	19596.95	142.1504	19594.11
19591.88	142.405	19594.19	142.4006	19595.62	142.4006	19596.42	142.4076	19594.45	142.4039	19596.29	142.4055	19597.74
19594.18	142.6525	19595.36	142.6569	19598.64	142.6632	19601.82	142.6493	19592.72	142.654	19597	142.6532	19597.34
19591.34	142.912	19595.65	142.899	19592.49	142.8993	19598.43	142.9023	19591.09	142.9019	19594.11	142.9011	19594.85
19593.58	143.1567	19597.23	143.155	19592.83	143.1551	19600.97	143.1505	19594.28	143.1524	19594.6	143.1505	19594.25
19597.53	143.4146	19598.29	143.4008	19592.4	143.4	19601.89	143.4052	19593.92	143.4019	19595.76	143.4025	19597.5
19594.86	143.6643	19594.69	143.657	19594.61	143.6523	19598.17	143.6517	19594.7	143.6566	19595.49	143.6524	19592.28
19591.72	143.9212	19598.52	143.899	19595.73	143.9007	19595.28	143.9064	19594.3	143.9011	19597.24	143.9042	19595.88
19592.84	144.1638	19593.16	144.154	19596.59	144.1515	19599.94	144.1494	19592.74	144.1541	19595.32	144.1511	19596.17
19592.36	144.4191	19598.78	144.3995	19598.35	144.4	19596.51	144.406	19593.99	144.4029	19595.06	144.4082	19596.56
19594.11	144.6614	19591.65	144.6584	19592.52	144.6528	19599.83	144.6533	19593.37	144.6576	19597.47	144.655	19593.62
19592.51	144.9175	19592.94	144.9013	19586.99	144.9017	19596.48	144.9064	19594.7	144.9015	19594.08	144.9034	19596.08
19589.93	145.1599	19595.46	145.1567	19596.21	145.1529	19594.43	145.1509	19598.26	145.1528	19595.38	145.1513	19593.92
19598.11	145.4324	19593.23	145.3986	19592.91	145.4021	19592.09	145.4073	19594.88	145.4026	19593.46	145.4041	19596.12
19591.59	145.6581	19596.13	145.6801	19590.27	145.6536	19598.42	145.6539	19593.33	145.6564	19595.9	145.6512	19599.85
19596.58	145.9091	19594.59	145.9007	19592.48	145.8992	19599.96	145.9096	19590.95	145.903	19594.75	145.9018	19596.43
19597.13	146.1489	19593.87	146.1592	19594.15	146.1522	19597.41	146.1544	19594.41	146.1531	19595.15	146.1527	19597.13
19592.93	146.4106	19595.72	146.4006	19594.82	146.4012	19597.93	146.4068	19595.62	146.401	19596.47	146.4065	19595.81
19591.15	146.6541	19588.35	146.6574	19598.69	146.6545	19596.77	146.6547	19598.26	146.6551	19594.43	146.6518	19594.36
19598.95	146.9094	19593.76	146.9001	19595.59	146.904	19593.77	146.9061	19594.92	146.9015	19594.98	146.9015	19593.77
19595	147.1505	19596.11	147.1565	19593.07	147.1533	19587.41	147.1543	19592.42	147.1534	19596.27	147.1539	19595.26
19594.93	147.4043	19595.19	147.4021	19596.32	147.4018	19596.94	147.4073	19593.56	147.4038	19594.92	147.4074	19595.71
19592.97	147.6584	19592.21	147.659	19589.12	147.6539	19595.46	147.6534	19596.73	147.6562	19593.5	147.6547	19593.65
19596.26	147.9151	19594.5	147.9038	19593.4	147.9018	19598.38	147.9085	19594.76	147.9028	19596.4	147.9022	19591.43
19592.4	148.153	19594.9	148.1567	19590.97	148.1555	19599.2	148.1531	19593.56	148.1527	19597.03	148.152	19592.27
19591.84	148.4068	19601.48	148.402	19592.65	148.4005	19594.05	148.4081	19594.94	148.4039	19595.44	148.4053	19594.17
19594.78	148.6501	19596.14	148.6585	19591.75	148.6531	19595.97	148.653	19592.9	148.6584	19598.69	148.6537	19595.66
19592.04	148.909	19596.95	148.904	19592.88	148.9017	19593.49	148.906	19596.68	148.9022	19593.63	148.9029	19595.64
19583.18	149.1523	19597.89	149.1642	19594.19	149.1569	19598.08	149.1555	19590.9	149.1535	19596.42	149.1541	19593.14
19592.62	149.4098	19593.75	149.4036	19591.46	149.4034	19595.12	149.4094	19591.58	149.4054	19591.44	149.4067	19594.17
19587.4	149.6528	19595.28	149.6611	19595.04	149.6549	19594.19	149.6541	19594.9	149.658	19595.71	149.6539	19593.32
19590.96	149.9089	19595.6	149.9027	19586.45	149.9006	19600.53	149.9147	19593.68	149.9018	19593.18	149.9045	19595.69
19596.14	150.1519	19596.33	150.1576	19591.45	150.1535	19599.69	150.164	19594.95	150.1518	19595.11	150.1549	19596.14
19594.8	150.4091	19594.85	150.414	19593.56	150.4069	19594.97	150.4131	19595.85	150.4057	19593.07	150.4096	19594.83
19594.99	150.6555	19590.31	150.663	19589.15	150.66	19596.71	150.6579	19594.99	150.6604	19592.26	150.6604	19596.79
19591.02	150.924	19589.88	150.9043	19591.31	150.9056	19596.61	150.9088	19591.86	150.9065	19593.17	150.9102	19593.54
19589.39	151.1572	19594.07	151.1631	19591.71	151.1589	19591.86	151.158	19596.66	151.1542	19596.87	151.1572	19595.37
19594	151.4101	19594.84	151.4047	19591.81	151.4069	19593.58	151.4105	19592.32	151.4073	19592.94	151.4106	19594.16
19590.37	151.6573	19600.12	151.6628	19593.33	151.6579	19596.47	151.6587	19592.75	151.6616	19596.81	151.6588	19597.31
19593.74	151.9154	19594.21	151.9058	19591.2	151.9084	19596.04	151.9102	19591.87	151.9092	19596.18	151.9076	19596.12
19591	152.1555	19591.72	152.161	19595.24	152.1576	19592.18	152.1575	19593.28	152.1572	19595.97	152.1588	19596.05
19596.79	152.4117	19593.05	152.4063	19597.17	152.4079	19596.6	152.4107	19592.03	152.4051	19593.03	152.411	19592.97
19591.84	152.6552	19594.28	152.664	19591.1	152.6562	19595.62	152.6566	19592.65	152.6614	19596.13	152.6593	19592.82
19589.68	152.9154	19588.76	152.9073	19587.97	152.9061	19594.96	152.9095	19591.97	152.908	19596.62	152.907	19593.08
19590.38	153.1588	19599.21	153.1619	19588.29	153.1577	19593.09	153.1572	19596.59	153.158	19596.39	153.1575	19593.32
19594.99	153.4127	19597.11	153.4067	19592.67	153.4074	19599.14	153.4131	19594.82	153.4098	19595.16	153.412	19594.75
19592.47	153.656	19590.12	153.6626	19593.04	153.6576	19596.08	153.6568	19594.23	153.6621	19592.7	153.6596	19593.58
19591.39	153.9169	19594.15	153.9066	19591.88	153.9055	19590.23	153.9108	19592.19	153.9102	19592.7	153.9084	19595.27
19590.46	154.158	19596.66	154.1621	19593.71	154.1597	19588.65	154.1593	19592.55	154.1582	19592.4	154.158	19592.5
19593.27	154.4151	19594.79	154.4068	19593.42	154.4076	19594.37	154.4133	19591.06	154.4085	19596.31	154.4138	19593.87
19593.96	154.6558	19592.25	154.6665	19599.61	154.6595	19601.38	154.6598	19594.1	154.6621	19594.28	154.6604	19594.19
19587.82	154.9164	19593.25	154.907	19592.18	154.9056	19602.99	154.9118	19597.41	154.9097	19595.24	154.9094	19594.98
19591.89	155.1565	19587.32	155.1641	19594.94	155.16	19594.15	155.161	19592.59	155.1601	19594.8	155.1578	19595.46
19587.62	155.4135	19594	155.4096	19590.15	155.4086	19591.34	155.4114	19592.05	155.4095	19594.21	155.4137	19594.87
19592.48	155.6581	19596.27	155.6656	19589.45	155.6587	19595.12	155.659	19594.02	155.6678	19591.72	155.6615	19594.08
19590.74	155.9203	19596.27	155.9108	19594.79	155.909	19593.3	155.912	19593.03	155.9075	19592.78	155.9105	19593.21
19594.09	156.1573	19587.76	156.1661	19597.05	156.1589	19590.29	156.158	19593.84	156.1581	19594.84	156.1577	19595.8

19588.78	156.4116	19591.89	156.4098	19591.33	156.4079	19593.69	156.4143	19593.47	156.4106	19593.63	156.425	19594.64
19593.61	156.6591	19590.86	156.665	19599.28	156.6601	19596.9	156.6618	19593.2	156.6648	19595.19	156.6623	19595.69
19591.38	156.9181	19595.29	156.9071	19593.48	156.907	19600.56	156.9136	19592.76	156.9101	19594.58	156.9097	19594.95
19592.92	157.1571	19590.88	157.1637	19591.38	157.1629	19595.52	157.1588	19593.94	157.1594	19594.47	157.1601	19592.29
19588.87	157.4133	19592.27	157.41	19594.28	157.4082	19595.5	157.4101	19594.07	157.4098	19595.07	157.4122	19594.09
19592.31	157.6579	19596.05	157.6667	19589.98	157.6601	19594.29	157.6598	19595.14	157.6648	19594.99	157.6581	19595.36
19589.99	157.9185	19592.65	157.9105	19587.64	157.9075	19594.07	157.9147	19593.58	157.9105	19592.05	157.909	19594.24
19593.59	158.1588	19589.3	158.1654	19594.11	158.16	19593.73	158.1615	19595.59	158.1594	19589.81	158.1598	19594.55
19592.89	158.4143	19590.21	158.409	19592.63	158.4116	19595.35	158.4131	19591.89	158.4105	19592.49	158.4152	19592.07
19592.54	158.6581	19596.73	158.6659	19593.37	158.6616	19597.83	158.6583	19593.91	158.6641	19596.6	158.6595	19593.59
19590.92	158.9193	19594.05	158.9202	19585.76	158.9084	19595.68	158.9117	19592.31	158.9132	19594	158.9108	19592.5
19590.06	159.1597	19590.13	159.1633	19590.11	159.1585	19592.12	159.1609	19594.39	159.1609	19592.52	159.1608	19593.76
19592.27	159.417	19597.08	159.4112	19590.4	159.4089	19593.16	159.4148	19594.97	159.4113	19592.46	159.4176	19593.59
19588.02	159.6579	19590.58	159.6645	19595.44	159.6606	19593.53	159.6597	19592.58	159.6644	19593.74	159.6603	19594.73
19592.46	159.9189	19595.2	159.909	19590.44	159.9085	19597.51	159.914	19596.16	159.9107	19592.96	159.9092	19592.5
19593.65	160.1599	19592.81	160.1638	19591.83	160.1631	19599.79	160.1757	19593.65	160.1604	19592.41	160.1606	19595.98
19588.96	160.415	19589.83	160.4117	19590.57	160.4129	19596.2	160.4126	19593.36	160.4131	19591.46	160.413	19595.76
19588.76	160.6565	19591.01	160.6612	19594.93	160.6595	19594.19	160.6589	19595.32	160.6609	19593.6	160.6604	19591.73
19593.23	160.9161	19592.22	160.9069	19593.1	160.9119	19593.89	160.9114	19595.75	160.9093	19593.12	160.9086	19593.14
19590.65	161.1569	19590.14	161.1656	19587.24	161.1585	19595.76	161.1572	19593.23	161.16	19592.47	161.1567	19591.95
19591.77	161.4135	19592.07	161.4061	19592.18	161.4077	19586.79	161.4124	19591.98	161.4105	19595.23	161.4123	19595.08
19589.65	161.6566	19590.69	161.6638	19592.51	161.6583	19594.38	161.6602	19592.11	161.6639	19592.66	161.6607	19594.06
19592.62	161.9183	19591.89	161.91	19597.79	161.9064	19595.04	161.9105	19593.52	161.9089	19594.77	161.9091	19597.28
19590.26	162.1555	19589.6	162.1625	19593.43	162.157	19591.48	162.1592	19591.66	162.158	19592.94	162.1598	19590.31
19592.22	162.4136	19590.34	162.4083	19597.02	162.4095	19595.7	162.413	19592.27	162.4107	19594.33	162.4103	19593.27
19594.73	162.658	19591.65	162.6636	19589.47	162.6714	19589.9	162.6587	19593.15	162.667	19593.67	162.6591	19592.72
19588.88	162.918	19593.97	162.9094	19591.28	162.9074	19590.36	162.9121	19593.18	162.9098	19595.77	162.9121	19592.25
19588.13	163.1564	19596.52	163.1639	19596.57	163.1574	19592.7	163.1597	19592.45	163.1577	19595.59	163.1615	19597.44
19587.67	163.4121	19591.28	163.4097	19596.57	163.4084	19593.3	163.4098	19591.61	163.4114	19592.62	163.4127	19592.41
19590.91	163.6573	19591.91	163.665	19591.39	163.6603	19598	163.6601	19592.03	163.686	19596.16	163.6641	19596.4
19586.97	163.9175	19587.73	163.91	19592.35	163.9079	19591.09	163.9128	19593.1	163.9099	19592.25	163.9142	19593.86
19593.23	164.1581	19589.54	164.1649	19592.79	164.1582	19598.77	164.164	19590.27	164.1597	19594.75	164.1628	19594.34
19589.55	164.4184	19590.51	164.4094	19593.37	164.4104	19593.6	164.4154	19594.91	164.4092	19596.28	164.4181	19595.73
19584.72	164.6564	19588.11	164.6662	19589.49	164.6653	19593.56	164.6593	19591.57	164.6638	19591.24	164.6701	19594.14
19592.54	164.9189	19590.71	164.9119	19590.46	164.9098	19589.93	164.91	19589.74	164.9118	19592.43	164.9133	19596.41
19592.67	165.1581	19590.83	165.1659	19586.84	165.1616	19593.7	165.159	19591.96	165.1601	19594.29	165.1595	19591.81
19592.21	165.415	19589.33	165.4092	19586.28	165.4097	19594.11	165.4133	19592.16	165.4135	19593.99	165.4143	19591.21
19586.11	165.6581	19590.67	165.6659	19594.81	165.6591	19596.1	165.6602	19592.02	165.666	19595.39	165.662	19591.85
19590.91	165.9184	19595.8	165.9118	19593.59	165.9082	19589.92	165.9114	19590.92	165.9102	19592	165.9123	19593.1
19590.87	166.1596	19590.45	166.1717	19587.76	166.1609	19593.16	166.1593	19594.36	166.1607	19592.54	166.1589	19592.42
19587.64	166.4146	19595.13	166.4092	19585.48	166.4108	19596.67	166.4131	19593.93	166.4107	19594.71	166.4135	19590.48
19590.21	166.6597	19591.69	166.6675	19588	166.6599	19592.75	166.664	19591.2	166.6661	19594.3	166.661	19592.73
19586.78	166.9261	19589.33	166.9102	19587.89	166.909	19596.6	166.9124	19589.89	166.9099	19592.1	166.9128	19596.18
19589.02	167.1595	19588.57	167.1663	19594.63	167.1595	19585.95	167.1607	19594.37	167.1635	19593.13	167.1608	19591.34
19589.39	167.4157	19589.46	167.4117	19586.76	167.4128	19598.82	167.4116	19592.97	167.4103	19596.7	167.4145	19593.19
19589.75	167.6603	19587.14	167.6668	19589.57	167.6626	19597.42	167.6626	19590.87	167.6683	19596.38	167.659	19593.26
19590.39	167.919	19588.93	167.9117	19591.18	167.9084	19590.76	167.9138	19588.91	167.9093	19592.69	167.9124	19593.6
19587.91	168.1597	19597	168.1642	19594.25	168.1596	19597.1	168.1602	19590.93	168.1602	19594.62	168.1614	19589.73
19589.6	168.4148	19596.16	168.4093	19590.61	168.4114	19595.61	168.4126	19592.86	168.4136	19592.43	168.4152	19593.18
19586.66	168.6584	19592.58	168.6645	19592.01	168.6619	19591.5	168.6607	19593.85	168.6719	19596.05	168.6615	19594.9
19588.1	168.919	19590.55	168.9083	19593.33	168.9109	19589.74	168.9149	19592.63	168.9095	19592.63	168.9109	19595.65
19592.64	169.1599	19583.7	169.164	19587.7	169.161	19592.76	169.1641	19591.62	169.1601	19594.5	169.1628	19593.8
19584.27	169.4192	19592.5	169.4111	19583.84	169.4127	19589.33	169.4165	19592.68	169.411	19591.66	169.4164	19591.3
19586.68	169.6583	19591.6	169.6664	19588.03	169.6627	19593.38	169.664	19592.52	169.6681	19592.88	169.6614	19589.29
19589.15	169.9208	19592.82	169.9103	19587.77	169.9139	19597.88	169.9145	19592.42	169.9115	19590.83	169.914	19593.35
19593.01	170.1612	19592.11	170.1663	19590.02	170.162	19593.13	170.1626	19589.43	170.1599	19589.47	170.1592	19593.66
19587.38	170.4152	19590.73	170.4105	19590.36	170.4125	19594.1	170.4151	19595.22	170.4116	19591.2	170.4149	19594.76
19589.39	170.6612	19593.08	170.667	19584.26	170.6603	19591.7	170.6626	19593.83	170.6711	19592.63	170.6603	19593.81
19586.72	170.9271	19589.51	170.912	19588.97	170.9108	19594.27	170.9144	19589.96	170.9138	19594.29	170.916	19594.81
19585.78	171.1646	19590.32	171.1695	19595.92	171.1616	19590.79	171.1627	19590.95	171.161	19591.62	171.162	19591.97
19587.85	171.4144	19590.32	171.4121	19585.75	171.4162	19598.25	171.4156	19591.88	171.4107	19590.12	171.4159	19592.48
19587.38	171.661	19589.03	171.6681	19586.91	171.6744	19597.06	171.6653	19590.92	171.6684	19591.43	171.6624	19592.37
19590.91	171.921	19592.61	171.9119	19590.75	171.9168	19591.35	171.9137	19593.17	171.9134	19594.63	171.9133	19591.11
19586.48	172.1612	19588.31	172.1669	19589.15	172.1601	19593.81	172.1639	19592.67	172.1635	19593.31	172.1608	19594.33
19584.36	172.4163	19592.19	172.415	19593.53	172.415	19589.99	172.4157	19590.54	172.4129	19590.44	172.4161	19591.57
19592.26	172.6612	19588.41	172.666	19590.56	172.6632	19587.43	172.6657	19591.2	172.6699	19590.39	172.6622	19591.83
19588.37	172.9212	19589.79	172.9113	19586.71	172.914	19593.49	172.9146	19593.97	172.9128	19594.68	172.9154	19592.61
19586.87	173.1612	19591.61	173.1665	19591.75	173.162	19595.42	173.1618	19593.08	173.163	19594.37	173.1621	19595.28
19592.45	173.4173	19594.11	173.4147	19589.31	173.4135	19591.14	173.4149	19592.78	173.4131	19592.76	173.4181	19593.9
19589.3	173.6601	19592.7	173.6684	19592.15	173.6627	19590.22	173.6773	19590.44	173.6677	19591.07	173.6612	19593.53

19590.98	173.9206	19586.65	173.9119	19593.82	173.9128	19591.9	173.9257	19592.79	173.9147	19593.45	173.9149	19592.48
19585.62	174.1633	19600.07	174.1679	19588.4	174.1631	19592.56	174.1647	19590.49	174.1637	19591.94	174.1634	19592.53
19590.12	174.4169	19589.42	174.4142	19589.65	174.4138	19595.94	174.4165	19590.19	174.4134	19595.18	174.4212	19593
19591.05	174.6613	19587.17	174.673	19588.11	174.6642	19593.69	174.6651	19591.8	174.6706	19591.33	174.6628	19594.32
19583.47	174.9217	19596.67	174.9133	19587.27	174.9154	19589.82	174.9187	19591.22	174.9139	19594.05	174.9127	19593.38
19584.57	175.1631	19585.91	175.1699	19589.66	175.1638	19591.02	175.1628	19592.94	175.1635	19592.38	175.1632	19594.32
19587.23	175.4203	19590.59	175.4152	19588.61	175.4138	19587.79	175.4186	19590.8	175.4141	19591.52	175.4192	19592.07
19583.5	175.6618	19589.8	175.669	19591.52	175.6777	19590.86	175.6675	19591.79	175.6729	19592.6	175.6633	19591.74
19589.1	175.9214	19593.37	175.9132	19586.15	175.9158	19594.1	175.9206	19589.44	175.9134	19590.54	175.9173	19591.66
19586.05	176.1625	19590.56	176.1698	19581.14	176.1625	19587.26	176.1685	19591.51	176.1625	19588.45	176.1627	19593.78
19587.48	176.4163	19591.32	176.4169	19589.81	176.4143	19589.03	176.4173	19592.29	176.4128	19594.02	176.4293	19590.35
19592.77	176.6657	19591.92	176.6693	19591.55	176.6663	19586.39	176.6672	19590.63	176.6707	19592.24	176.6677	19592.57
19583.44	176.9239	19586.4	176.9141	19590.79	176.9139	19588.79	176.9171	19592.87	176.915	19593.54	176.9153	19590.19
19586.82	177.1631	19589.22	177.1687	19586.6	177.1653	19593.97	177.1651	19591.57	177.1637	19590.15	177.1642	19591.88
19589.87	177.4177	19588.01	177.4178	19582.92	177.4145	19591.32	177.4172	19589.48	177.4127	19592.44	177.4197	19593.26
19584.14	177.6629	19592.12	177.6716	19592.34	177.6694	19591.85	177.6638	19592.97	177.6734	19593.22	177.6646	19591.2
19592.69	177.9228	19588.48	177.9155	19586.17	177.9139	19593.44	177.918	19590.16	177.914	19592.21	177.9156	19592.2
19589.56	178.1657	19596.63	178.1698	19583.96	178.1672	19591.63	178.164	19590.59	178.1648	19591.58	178.1652	19589.75
19588.85	178.4211	19589.75	178.4154	19595.6	178.4149	19590.18	178.4206	19590.25	178.4125	19590.01	178.4224	19592.3
19587.11	178.6625	19585.64	178.6699	19585.41	178.6672	19595.34	178.6668	19593.55	178.6704	19593.45	178.6687	19590.17
19584.95	178.9253	19592.38	178.9207	19584.8	178.9165	19586.12	178.9177	19591.94	178.9165	19592.26	178.9274	19592.65
19590.84	179.165	19592.84	179.1706	19589.5	179.166	19590.4	179.1638	19591.39	179.166	19592.74	179.1708	19593.99
19589.12	179.4223	19592.04	179.4164	19590.81	179.4148	19590.28	179.4186	19590.46	179.4165	19592.85	179.4204	19591.13
19586.12	179.6644	19587.64	179.6709	19588.54	179.6853	19588.65	179.6659	19590.64	179.6723	19591.45	179.6635	19594.4
19587.28	179.9215	19587.22	179.9168	19591.46	179.9151	19594.25	179.9213	19593.21	179.9193	19591.71	179.9157	19590.61
19588.48	180.1652	19589.94	180.1704	19586.52	180.165	19591.85	180.1648	19593.55	180.1686	19590.63	180.1657	19592.5
19588.72	180.4177	19590	180.4159	19588.49	180.4154	19589.49	180.4173	19592.14	180.4251	19591.8	180.4221	19591.37
19587.46	180.6649	19582.6	180.6711	19591.86	180.6677	19589.21	180.6643	19589.1	180.6774	19593.41	180.6653	19592.73
19584.29	180.9249	19588.27	180.9159	19589.29	180.9147	19592.53	180.9192	19589.07	180.9164	19592.39	180.9144	19595.71
19584.48	181.1648	19596.12	181.1732	19585.84	181.1702	19596.25	181.1667	19591.24	181.1662	19589.91	181.1652	19593.36
19586.86	181.4202	19588.9	181.418	19584.55	181.416	19587.03	181.419	19591.73	181.4161	19593.22	181.4211	19591.99
19586.11	181.6676	19590.46	181.6712	19587.86	181.6686	19588.4	181.6687	19591.48	181.684	19596.39	181.666	19593.34
19586.64	181.9256	19589.85	181.9182	19596.88	181.9152	19585.79	181.9198	19591.37	181.9175	19591.71	181.9157	19590.8
19588.18	182.1671	19585.24	182.1699	19585.63	182.1672	19593.19	182.1683	19589.66	182.1663	19593.68	182.1642	19592.32
19587.64	182.4194	19589.21	182.4174	19589.65	182.4153	19599.39	182.4255	19593.01	182.4176	19591.32	182.432	19591.49
19582.71	182.6653	19587.34	182.6723	19587.9	182.6687	19591.91	182.6726	19591.29	182.6746	19591.51	182.6711	19592.89
19586.95	182.9252	19588.97	182.9213	19590.48	182.9142	19590.63	182.9203	19590.74	182.9219	19591.16	182.9162	19589.95
19584.99	183.167	19586.02	183.1743	19591.2	183.1683	19591.92	183.1657	19590.9	183.1654	19591.83	183.1673	19593.39
19587.41	183.4218	19587.9	183.4141	19586.99	183.417	19591.12	183.4215	19589.83	183.4164	19591.52	183.4204	19589.07
19591.69	183.6653	19591.86	183.6702	19590.1	183.6696	19590.31	183.6696	19589.72	183.6846	19590.44	183.6661	19592.24
19590.6	183.9247	19588.93	183.9176	19589.58	183.9155	19588.68	183.9215	19590.77	183.9264	19592.53	183.9183	19592.78
19590.62	184.1672	19590.7	184.1704	19581.99	184.1654	19587.47	184.1656	19591.67	184.1709	19591.39	184.1695	19591.36
19585.24	184.4238	19591.58	184.4231	19594.64	184.4172	19592.84	184.4205	19589.08	184.4165	19593.15	184.4233	19588.21
19587.47	184.6663	19588.97	184.6793	19587.28	184.6688	19590.46	184.6689	19586.78	184.6754	19590.18	184.666	19591.53
19590.94	184.9251	19586.42	184.9168	19585.68	184.9162	19591.72	184.9216	19591.11	184.9185	19593.62	184.9184	19590.05
19589.63	185.1677	19589.63	185.17	19587	185.1674	19586.99	185.1684	19590.6	185.169	19592.45	185.1675	19591.8
19589.06	185.4201	19584.67	185.4156	19589.97	185.4163	19594.2	185.421	19590.33	185.4189	19589.45	185.4255	19593.56
19581.39	185.6667	19593.17	185.6736	19588.14	185.6701	19594.85	185.667	19590.42	185.6796	19593.42	185.6672	19592.97
19587.19	185.9269	19588.7	185.9171	19590.91	185.9163	19588.1	185.9219	19588.56	185.9177	19591.66	185.9202	19592.3
19586.06	186.1673	19587.6	186.1749	19588.82	186.1684	19595.49	186.1681	19590.11	186.1671	19589.19	186.1678	19591.21
19583.77	186.4214	19592.59	186.4192	19590.51	186.4164	19594.77	186.4209	19590.93	186.4181	19591.89	186.4236	19592.12
19586.59	186.669	19587.32	186.6734	19587.56	186.6696	19588.56	186.6741	19591.77	186.6853	19592.38	186.6675	19592.57
19590.81	186.928	19592.65	186.921	19590.86	186.9176	19590.47	186.9222	19589.33	186.9223	19590.39	186.9177	19595.18
19587.32	187.1681	19594.16	187.1743	19584.61	187.1685	19587.44	187.1721	19590.9	187.1726	19592.52	187.1688	19593.22
19591.88	187.4215	19592.94	187.4205	19585.73	187.4183	19594.05	187.4217	19591.61	187.423	19590.8	187.426	19591.91
19587.23	187.667	19592.21	187.676	19590.58	187.6681	19584.82	187.6675	19592.47	187.6758	19590.96	187.6681	19591.58
19589.2	187.9271	19590.53	187.9208	19586.31	187.9171	19588.42	187.9226	19591.51	187.923	19589.32	187.9196	19590.34
19582.86	188.1689	19590.1	188.1737	19585.71	188.1804	19592.67	188.1692	19590.3	188.1709	19592.98	188.1688	19591.13
19590.2	188.4218	19589.69	188.4182	19585.04	188.4268	19590.49	188.4227	19590.32	188.418	19591.55	188.4234	19591.15
19586.4	188.6663	19589.73	188.6735	19588.27	188.6742	19589.91	188.6684	19591.42	188.6797	19593.07	188.669	19592.46
19584.48	188.9268	19583.01	188.9226	19589.83	188.9164	19587.49	188.9218	19591.48	188.9209	19590.99	188.9205	19594.01
19584.24	189.171	19584.6	189.1756	19585.72	189.1692	19587.99	189.1715	19590.05	189.172	19589.34	189.1711	19590.12
19589.18	189.4267	19587.1	189.4198	19591.52	189.4225	19595.47	189.422	19589.35	189.4177	19592.65	189.4286	19593.14
19585.17	189.6671	19585.86	189.6729	19587.3	189.6748	19588.08	189.6701	19590.15	189.6751	19591.57	189.6687	19593.93
19584.85	189.9272	19586.6	189.9201	19590.81	189.9193	19590.01	189.9218	19589.25	189.9227	19592.02	189.9215	19590.73
19588.38	190.1677	19588.86	190.1723	19593.7	190.1692	19589.13	190.1708	19586.83	190.1696	19590.92	190.1716	19592.91
19579.98	190.4238	19588.43	190.4221	19584.38	190.4189	19588.6	190.4229	19588.11	190.4186	19590.8	190.428	19589.58
19588.4	190.6697	19588.8	190.6758	19589.79	190.6739	19588.52	190.67	19592.42	190.6761	19589.65	190.668	19593.04
19587.23	190.9319	19582.36	190.9231	19589.86	190.9199	19588.77	190.9244	19591.8	190.92	19590.7	190.9196	19591.72
19584.68	191.169	19590.28	191.1768	19586.46	191.1713	19591.32	191.169	19589.23	191.1719	19591.2	191.1765	19591.5

19584.74	191.4226	19587.12	191.4202	19583.42	191.4188	19586.54	191.4233	19589.42	191.4194	19591.5	191.4246	19591.8
19590.52	191.6687	19587.2	191.6772	19590.57	191.6749	19589.64	191.6729	19591.97	191.6819	19589.83	191.67	19589.65
19585.95	191.929	19586.18	191.92	19586.14	191.9191	19593.07	191.9239	19589.65	191.9186	19591.76	191.9202	19592.69
19586.04	192.1686	19584.44	192.1749	19593.05	192.1729	19593.66	192.1721	19590.27	192.1694	19590.24	192.1728	19593.56
19584.38	192.4244	19581.17	192.4182	19590.11	192.4213	19591.98	192.4251	19587.85	192.42	19593.49	192.4263	19592.73
19585.26	192.6686	19587.3	192.6755	19586.57	192.6758	19589.1	192.6718	19591.38	192.6983	19591.22	192.6705	19589.89
19584.84	192.9304	19592.14	192.9214	19591.21	192.93	19588.31	192.9279	19591.93	192.9202	19590.44	192.9211	19591.57
19589.69	193.17	19588.09	193.1746	19588	193.1732	19581.56	193.1716	19590.45	193.1686	19592.44	193.1704	19588.73
19584.03	193.4265	19585.72	193.4189	19584.94	193.4204	19593.34	193.4226	19589.54	193.4201	19592.1	193.4256	19593.35
19582.08	193.6681	19583.58	193.6745	19584.53	193.678	19588.35	193.6744	19589.07	193.677	19592.97	193.6708	19591.87
19583.3	193.933	19581.87	193.9195	19586.42	193.9184	19591.39	193.9254	19588.45	193.9221	19593.24	193.9203	19594.03
19586.04	194.1705	19588.56	194.1738	19591.64	194.1721	19588.5	194.1722	19589.88	194.1715	19589.25	194.1733	19594.91
19586.08	194.4269	19589.39	194.4196	19591.87	194.4203	19587.68	194.4226	19587.58	194.4345	19589.6	194.4307	19592.81
19587.24	194.6697	19590.32	194.6768	19591.7	194.6762	19592.44	194.6711	19589.98	194.6772	19592.19	194.6726	19593.77
19587.77	194.9506	19589.76	194.9198	19583.97	194.92	19589.96	194.925	19591.29	194.9229	19589.4	194.9223	19590.94
19584.57	195.1684	19590.58	195.1752	19588.83	195.1723	19586.14	195.1847	19591	195.1772	19590.63	195.1773	19592.38
19581.03	195.4261	19591.07	195.4198	19585.15	195.422	19590.24	195.4238	19591.72	195.4216	19591.54	195.4267	19590.53
19593.02	195.6699	19586.26	195.6768	19583.86	195.6744	19587.2	195.6733	19590	195.6805	19591.35	195.6702	19589.64
19587.62	195.9328	19584.66	195.9211	19584.92	195.9211	19593.68	195.9266	19588.58	195.9215	19591.05	195.9225	19593.19
19587.38	196.1685	19595.8	196.1793	19588.71	196.1826	19588.95	196.1714	19591.54	196.1734	19591.78	196.1745	19592.26
19580.11	196.4241	19590.21	196.4184	19585.96	196.4211	19587.86	196.4254	19592.98	196.4236	19586.86	196.4322	19591.15
19583.72	196.6708	19585.49	196.6761	19587.92	196.6758	19592	196.6746	19590.23	196.6805	19592.83	196.6725	19591.53
19582.69	196.9285	19582.54	196.9206	19581.95	196.92	19586.36	196.9265	19589.78	196.9236	19590.47	196.9211	19593.46
19587.61	197.1706	19584.67	197.1756	19584.97	197.1738	19589.64	197.1711	19589.87	197.1725	19591.24	197.1729	19589.57
19585.77	197.4266	19589.86	197.4205	19588.33	197.4213	19590.19	197.424	19591.62	197.4232	19590.17	197.4271	19591.37
19586.26	197.67	19589.05	197.6769	19579.3	197.6766	19591.78	197.6745	19590.66	197.6798	19588.96	197.6721	19592.31
19584.21	197.9309	19589.27	197.921	19582.16	197.9195	19583.43	197.9297	19589.21	197.9227	19588.15	197.925	19592.48
19586.72	198.172	19582.56	198.1757	19585.22	198.1739	19581.33	198.1744	19591.6	198.1728	19589.79	198.1758	19593.59
19585.74	198.4279	19586.04	198.4215	19587.04	198.4233	19590.36	198.427	19590.89	198.4225	19591.31	198.4379	19588.81
19584.09	198.6722	19584.91	198.6787	19591.56	198.6802	19584.08	198.6721	19591.13	198.683	19590.47	198.6763	19591.2
19583.95	198.9295	19582.93	198.9209	19583.08	198.9214	19592.18	198.9268	19590.83	198.924	19592.36	198.9249	19590.26
19586.85	199.1711	19584.26	199.1761	19590.4	199.1731	19587.06	199.1745	19588.74	199.1734	19592.22	199.171	19590.62
19584.17	199.4313	19587.99	199.4214	19591.99	199.4223	19595.04	199.4285	19591.97	199.4224	19591.08	199.4303	19590.33
19587.56	199.6727	19590.12	199.6785	19589.17	199.6779	19590.04	199.675	19592.63	199.68	19591.91	199.6721	19592.37
19589.69	199.9319	19582.59	199.9229	19587.25	199.9225	19592.64	199.9263	19588.08	199.9258	19590.38	199.9242	19591.82
19582.24	200.1713	19590.48	200.1766	19584.46	200.1752	19581.38	200.1752	19589.99	200.1731	19592.96	200.1748	19589.76
19577.95	200.4311	19594.25	200.4241	19585.42	200.4274	19585.11	200.4316	19587.69	200.4263	19588.23	200.4353	19590.96
19586.81	200.6753	19587.26	200.6818	19587.67	200.6785	19589.11	200.6773	19587.35	200.6885	19593.03	200.6781	19591.96
19583.63	200.9362	19590.17	200.9269	19588.74	200.9264	19591.38	200.9291	19590.8	200.9294	19593.14	200.9279	19591.8
19584.02	201.1754	19585.93	201.1834	19582.96	201.1783	19587.21	201.1782	19589.66	201.1797	19589.81	201.1783	19590.54
19583.64	201.4314	19585.68	201.4251	19592.37	201.428	19592.77	201.4316	19587.7	201.4289	19590.51	201.4379	19592.14
19586.47	201.6776	19582.88	201.6822	19585.19	201.6842	19591.8	201.6806	19590.04	201.6859	19591.21	201.677	19591.43
19588.88	201.9367	19592.06	201.9285	19585.48	201.926	19590.48	201.9321	19589.26	201.9303	19591.58	201.9276	19591.3
19584.65	202.1759	19583.52	202.1973	19587.38	202.1798	19589.6	202.178	19590	202.1786	19592.89	202.1781	19593.02
19582.79	202.4325	19584.57	202.4279	19590.32	202.428	19588.87	202.4311	19589.43	202.4285	19591.27	202.4363	19591.13
19585.61	202.6779	19587.73	202.6818	19585.23	202.6837	19594.77	202.6804	19589.28	202.69	19589.29	202.6804	19590.9
19587.21	202.9351	19592.52	202.9288	19587.17	202.9273	19587.29	202.9342	19590.92	202.9299	19594.21	202.9321	19591.66
19584.92	203.1773	19583.13	203.1808	19587.95	203.178	19589.6	203.1792	19587.44	203.1798	19591.18	203.1819	19591.38
19585.13	203.4332	19586.95	203.4265	19588.65	203.4326	19588.21	203.4333	19587.76	203.429	19590.45	203.4375	19594.63
19585.84	203.6777	19586.56	203.6833	19584.69	203.6818	19587.49	203.6777	19586.7	203.6873	19589.45	203.6792	19587.52
19582.53	203.9377	19590.91	203.9294	19582.06	203.929	19586.44	203.9317	19589.36	203.9298	19591.2	203.9289	19589.39
19582.72	204.1775	19584.41	204.1822	19590.43	204.1795	19589.56	204.1935	19590.81	204.1813	19590.51	204.1781	19589.3
19586.79	204.4354	19582.69	204.4277	19591.33	204.4282	19587.25	204.4324	19590.6	204.4285	19590.49	204.4374	19589
19583.63	204.678	19584.88	204.6823	19592.79	204.6808	19588.45	204.6795	19589.31	204.6911	19590.87	204.6793	19591.9
19586.13	204.9361	19585.89	204.9287	19586.09	204.9287	19585.28	204.9298	19589.01	204.9342	19589.03	204.9281	19592.05
19586.62	205.1782	19590.84	205.1843	19588.65	205.1787	19592.8	205.1792	19590.39	205.1826	19589.95	205.1779	19589.3
19582.75	205.4338	19581.89	205.428	19588.76	205.4288	19596.34	205.4332	19590.62	205.429	19590.71	205.4364	19592.77
19584.51	205.7003	19588.25	205.6827	19585.05	205.6816	19591.28	205.6792	19590.04	205.6875	19590.43	205.6811	19590.38
19581.83	205.9389	19583.2	205.9296	19577.89	205.9278	19593.06	205.933	19592.5	205.9323	19593.2	205.9297	19590.18
19582.72	206.1769	19588.65	206.1836	19585	206.1795	19584.11	206.1794	19587.96	206.1815	19593.19	206.1788	19590.94
19584.37	206.4346	19591.37	206.4269	19586.85	206.4295	19586.41	206.4329	19593.46	206.4284	19590.41	206.4358	19590.7
19582.57	206.6789	19588.54	206.7024	19587.85	206.6863	19589.03	206.6817	19590.33	206.6869	19590.42	206.6795	19591.99
19589.2	206.9371	19584.16	206.9277	19585.17	206.9282	19582.52	206.935	19585.82	206.9327	19591.46	206.9304	19591.94
19583.94	207.1776	19581.02	207.1851	19586.2	207.1799	19588.38	207.1812	19589.22	207.1842	19591.01	207.1804	19589.71
19587.61	207.4354	19587.47	207.4281	19585.26	207.4287	19586.86	207.4338	19588.88	207.4297	19592.35	207.4412	19592.43
19585.71	207.6795	19586.37	207.6825	19587.4	207.6836	19588.72	207.6803	19591.19	207.6917	19589.17	207.6793	19590.97
19586.32	207.9381	19587.24	207.929	19588.33	207.9289	19592.42	207.9337	19590.13	207.9319	19589.54	207.9311	19593.33
19587.89	208.1794	19591.72	208.1832	19586.98	208.1795	19580.8	208.1825	19590.59	208.1828	19590.18	208.1799	19590.74
19586.87	208.4344	19588.78	208.43	19594.1	208.4323	19584.98	208.4336	19589.41	208.4321	19591.9	208.4375	19590.3
19586.11	208.6799	19584.01	208.683	19586.65	208.6842	19591.47	208.6797	19589.61	208.6887	19590.95	208.6818	19592.65

19584.2	208.9391	19582	208.9303	19592.12	208.9299	19587.27	208.9372	19588.9	208.9313	19589.88	208.9306	19589.94
19584.43	209.1805	19586.55	209.1825	19580.63	209.1808	19594.33	209.1847	19588.98	209.1866	19592.31	209.1786	19591.64
19581.39	209.4381	19583.92	209.4286	19589.82	209.4307	19586.92	209.4368	19588.45	209.4315	19590.46	209.4399	19590.08
19580.99	209.6788	19587.44	209.6849	19583.35	209.6902	19586.03	209.6808	19589.13	209.6895	19591.71	209.6921	19589.23
19584.1	209.9359	19583.83	209.9305	19585.83	209.9329	19587.96	209.9375	19589.12	209.9313	19593.22	209.9317	19589.56
19582.5	210.1798	19590.95	210.185	19584.61	210.181	19591.75	210.1978	19587.23	210.1838	19589.09	210.179	19590.36
19582.95	210.4372	19585.59	210.4297	19583.32	210.4311	19584.3	210.4446	19588.69	210.4312	19590.48	210.4388	19590
19584	210.6816	19583.93	210.6865	19587.53	210.6829	19589.96	210.6799	19590.86	210.6936	19591.88	210.6814	19590.46
19583.1	210.9369	19589.2	210.9316	19581.93	210.9316	19587.47	210.9346	19588.56	210.932	19587.76	210.9339	19591.66
19582.02	211.1794	19583.6	211.1848	19584.99	211.1832	19587.91	211.1839	19588.73	211.1839	19588.35	211.1826	19589.17
19584.09	211.4352	19586.77	211.4325	19586.25	211.4314	19585.82	211.436	19588.95	211.4315	19590.87	211.4465	19589.39
19581.5	211.6815	19587.27	211.6847	19588.23	211.6901	19589.45	211.6844	19591.12	211.6942	19592.34	211.6837	19589.63
19583.39	211.941	19586.19	211.9302	19590.66	211.9318	19585.37	211.9411	19591.84	211.9332	19589.86	211.9335	19589.21
19582.45	212.1804	19589.55	212.1855	19585.95	212.1838	19588.69	212.1928	19590.33	212.1859	19589.86	212.1825	19588.58
19584.96	212.4345	19583.82	212.4338	19594.46	212.4325	19590.45	212.4357	19589.36	212.4308	19589.81	212.4397	19591.4
19582.3	212.6811	19585.03	212.6876	19585.8	212.6868	19591.51	212.6846	19590.17	212.6953	19588.88	212.6828	19592.21
19583.82	212.9395	19585.41	212.9317	19582.18	212.9339	19586.81	212.9412	19588.74	212.9321	19591.58	212.9327	19588.25
19583.29	213.1799	19587.56	213.1846	19587.97	213.1843	19591.39	213.187	19590.41	213.1848	19590.8	213.1818	19592.24
19580.27	213.4361	19585.05	213.4321	19584.43	213.4343	19588.14	213.4344	19591.81	213.4325	19587.7	213.439	19588.18
19586.56	213.6807	19589.97	213.6845	19580.97	213.6891	19583.65	213.6833	19588.86	213.6923	19589.69	213.683	19592.28
19583.38	213.9512	19588.8	213.9366	19579.86	213.9333	19583.79	213.9366	19588	213.9345	19589.77	213.9361	19589.7
19581.25	214.1883	19581.49	214.1882	19587.1	214.184	19585.86	214.1853	19588.29	214.1856	19588.95	214.1827	19590.5
19582.35	214.4377	19589.01	214.4315	19583.9	214.4319	19585.41	214.4349	19589.74	214.4326	19587.78	214.44	19589.29
19582.71	214.6824	19588.39	214.6865	19584.16	214.6867	19589.44	214.6829	19588.71	214.6904	19590.35	214.6846	19590.22
19585.51	214.9407	19583.39	214.9349	19583.93	214.9351	19583.73	214.9359	19588.79	214.932	19588.19	214.9352	19592.14
19582.19	215.1815	19585.18	215.187	19590.05	215.1837	19587.88	215.1849	19590.12	215.1855	19590.14	215.1829	19593.16
19584.9	215.4368	19579.99	215.4315	19584.43	215.4348	19586.34	215.437	19587.98	215.4335	19589.33	215.4407	19586.83
19587.26	215.6839	19583.86	215.6889	19581.11	215.6872	19586.18	215.685	19588.6	215.6937	19588.38	215.6862	19591.42
19583.24	215.9412	19582.27	215.9387	19582.92	215.9336	19584.8	215.9359	19589.64	215.9324	19592.06	215.9377	19592.25
19581.5	216.1833	19588.93	216.1876	19584.48	216.1842	19589.15	216.187	19586.47	216.1891	19588.68	216.184	19589.27
19585.38	216.4368	19582.93	216.4326	19587.77	216.434	19587.26	216.4364	19588.49	216.4344	19589.67	216.443	19589.48
19582.33	216.6828	19584.1	216.687	19589.3	216.6886	19590.13	216.686	19587.45	216.6981	19589.95	216.6841	19589.92
19584.69	216.9401	19582.41	216.9346	19583.52	216.9342	19589.66	216.9377	19588.55	216.9348	19587.98	216.9362	19589.33
19581.63	217.1828	19590.09	217.1867	19584.48	217.1857	19591	217.1893	19588.53	217.1857	19589.81	217.1845	19589.07
19580.72	217.4381	19582.94	217.4335	19583.47	217.4337	19590.43	217.4375	19586.97	217.4331	19590.92	217.4424	19589.7
19587.11	217.6824	19589.72	217.6881	19580.99	217.6881	19589.95	217.6846	19592.69	217.6943	19587.75	217.6858	19591.71
19582.99	217.9437	19583.63	217.9371	19586.54	217.9351	19586.08	217.9376	19591.66	217.9362	19591.79	217.9338	19591.21
19584.66	218.1839	19578.55	218.1885	19580.03	218.1853	19591.36	218.1867	19590.91	218.1879	19590.36	218.1838	19589.84
19587.94	218.4415	19587.23	218.4316	19587.74	218.4361	19584.64	218.4379	19586.39	218.4333	19589.57	218.4504	19590.06
19580.28	218.6868	19577.71	218.688	19590.16	218.6874	19590.42	218.6847	19589.79	218.6941	19589.94	218.6853	19590.33
19580.44	218.9439	19581.93	218.9379	19584.18	218.9335	19590.75	218.9375	19589.06	218.9345	19587.65	218.9362	19590.18
19585.3	219.1833	19584.75	219.1887	19583.31	219.1848	19583.6	219.1868	19589.42	219.1878	19588.41	219.1839	19592.78
19588.07	219.4398	19584.96	219.434	19586	219.4355	19579.97	219.4373	19591.04	219.4353	19589.56	219.4488	19587.88
19585.2	219.6856	19583.2	219.6881	19580.56	219.689	19579.92	219.6849	19591.11	219.6999	19591.52	219.6858	19588.26
19582.96	219.9439	19587.37	219.9389	19585.12	219.9351	19585.78	219.9403	19589.76	219.9358	19587.48	219.9382	19590.52
19580	220.1844	19585.86	220.1901	19582.17	220.185	19590.57	220.1878	19590.86	220.1884	19591.71	220.1854	19590.05
19581.68	220.4372	19587.43	220.435	19586.66	220.4358	19587.49	220.4383	19589.73	220.4351	19590.97	220.4432	19591.18
19584.53	220.6855	19577.74	220.6879	19581.7	220.6884	19582.51	220.6852	19586.71	220.6945	19590.04	220.6863	19591.5
19588.91	220.9413	19585.34	220.9373	19579.44	220.9352	19582.21	220.9416	19588.29	220.9356	19587.4	220.9378	19591.27
19582.28	221.1844	19584.13	221.1884	19586.89	221.1861	19590.54	221.1896	19586.64	221.1899	19586.61	221.1864	19590.68
19583.69	221.4384	19581.76	221.4339	19587.17	221.4361	19588.48	221.4392	19590.62	221.4364	19588.99	221.4436	19591.4
19578.26	221.6856	19592.73	221.6947	19583.35	221.6923	19590.18	221.6882	19588.74	221.697	19589.1	221.6884	19590.74
19581.56	221.9443	19582.24	221.9361	19589.75	221.9345	19587.5	221.9402	19588.4	221.9366	19589.04	221.9385	19592.47
19582.39	222.1851	19585.4	222.1885	19586.01	222.1866	19583.05	222.1926	19587.56	222.191	19590.04	222.1871	19589.29
19582.88	222.4412	19582.18	222.4352	19585.53	222.4373	19589.71	222.4393	19589.44	222.438	19589.86	222.4438	19588.99
19585.14	222.6875	19583.18	222.6891	19584.16	222.6914	19585.99	222.6886	19589.54	222.7191	19591.48	222.6867	19590.4
19585.65	222.9437	19587.97	222.9372	19584.21	222.9345	19588.62	222.9422	19589.41	222.9479	19587.43	222.9379	19591.74
19580.14	223.1856	19585.25	223.1883	19581.85	223.1873	19590	223.1875	19587.62	223.1902	19591.62	223.186	19588.57
19581.77	223.4394	19580.84	223.4349	19583.26	223.438	19585.84	223.4449	19590.27	223.4368	19589.57	223.4483	19587.85
19582.01	223.6897	19587.61	223.6905	19588.6	223.6928	19592.35	223.6872	19590.53	223.6973	19592.02	223.6865	19589.52
19585.97	223.9438	19590.61	223.9366	19584.88	223.9364	19580.21	223.9403	19589.09	223.9374	19590.51	223.9396	19590.26
19577.74	224.1862	19585.88	224.1949	19584.49	224.1863	19581.91	224.1911	19588.6	224.1906	19589.86	224.1884	19590.51
19583.07	224.4412	19585.58	224.4347	19582.91	224.4382	19584.35	224.4433	19590.65	224.4371	19592.58	224.4485	19589.27
19581.54	224.6889	19582.19	224.6907	19583.61	224.6916	19588.97	224.6896	19588.4	224.6962	19589.07	224.6892	19588.42
19580.21	224.9471	19582.35	224.938	19588.65	224.9375	19587.58	224.9404	19586.4	224.9388	19589.25	224.9396	19589.22
19585.61	225.1875	19584.81	225.1913	19585.8	225.1867	19587.21	225.1924	19590.99	225.1916	19588.62	225.1907	19589.98
19586.91	225.4386	19580.99	225.437	19588.63	225.437	19588.21	225.4404	19586.12	225.4379	19590.53	225.4493	19588.98
19586.06	225.689	19584.25	225.6909	19584.46	225.6947	19593.29	225.6897	19590.14	225.6988	19591.08	225.6897	19591.81
19585.82	225.9468	19578.08	225.9387	19583.51	225.9366	19588.13	225.9416	19590.67	225.9389	19586.88	225.9386	19588.53
19580.41	226.1876	19581.16	226.1931	19588.04	226.1892	19581.41	226.1927	19586.75	226.1928	19587.03	226.1876	19592.97

19581.09	226.4423	19589.12	226.4375	19584.87	226.4417	19585.55	226.4407	19590.11	226.4391	19588.84	226.4489	19589.57
19583.41	226.6882	19588.46	226.6901	19584.55	226.6928	19591.17	226.6913	19587.69	226.7018	19588.39	226.6887	19588.34
19580.28	226.9428	19582.24	226.9373	19583.24	226.9384	19587.03	226.9417	19585.28	226.9369	19589.04	226.9389	19589.73
19586.58	227.186	19587.85	227.1929	19586.84	227.1946	19590.79	227.1923	19588.04	227.192	19590.24	227.1877	19587.88
19587.64	227.4405	19580.57	227.4367	19579.14	227.4399	19589.98	227.4419	19589.54	227.4394	19589.72	227.4487	19590.07
19580.93	227.6903	19584.79	227.6955	19582.59	227.6931	19588.04	227.6907	19590.17	227.7007	19591.78	227.6906	19589.11
19580.71	227.946	19584.44	227.9429	19586.8	227.9358	19587.25	227.9396	19587.89	227.9392	19590.79	227.9407	19589.91
19582.94	228.1835	19582.35	228.1915	19586.43	228.1903	19584.41	228.19	19585.92	228.1923	19591.78	228.1886	19588.95
19582.31	228.4405	19584.36	228.4426	19585.9	228.4399	19587.52	228.4439	19589.99	228.44	19589.4	228.4508	19589.63
19587.12	228.6887	19583.22	228.692	19588.29	228.6925	19576.87	228.6896	19588.76	228.6987	19591.49	228.6891	19588.59
19582.83	228.9469	19580.56	228.9426	19586.66	228.939	19586.18	228.9433	19587.13	228.9402	19589.25	228.9418	19589.63
19582.76	229.1882	19584.72	229.1935	19581.83	229.1891	19580.83	229.1918	19588.55	229.1936	19587.91	229.1893	19590.58
19580.42	229.4437	19581.92	229.4373	19591.17	229.4406	19580.71	229.4487	19586.02	229.4382	19588.93	229.4514	19589.57
19582.48	229.6914	19587.05	229.6914	19581.49	229.6945	19586.15	229.6902	19590.77	229.6994	19590	229.6851	19590.1
19583.88	229.9489	19580.85	229.9424	19585.57	229.9393	19581.39	229.9423	19588.14	229.9414	19587.82	229.9435	19588.88
19590.25	230.1845	19583.05	230.1934	19586.31	230.1907	19577.75	230.1938	19588.29	230.1972	19589.15	230.1907	19588.5
19583.56	230.4444	19589.94	230.4374	19585.77	230.439	19587.15	230.4427	19586.88	230.4388	19588.91	230.4481	19589.5
19579.59	230.69	19586.23	230.6929	19590.7	230.6932	19587.91	230.6911	19584.52	230.6996	19590.14	230.6905	19588.03
19578.85	230.9482	19583.28	230.9405	19582.58	230.937	19583.68	230.942	19587.01	230.9409	19590.54	230.9448	19587.43
19579.7	231.1867	19581.6	231.1952	19580.87	231.1909	19580.92	231.2057	19587.35	231.1928	19588.74	231.1902	19590.75
19575.69	231.4448	19576.92	231.4413	19582.02	231.4404	19586.67	231.4462	19587.54	231.4394	19590.09	231.4489	19590.27
19579.97	231.6909	19583.55	231.6924	19579.82	231.6949	19585.67	231.6917	19589.67	231.6996	19589.19	231.6914	19591.38
19580.22	231.9458	19587.57	231.942	19586.38	231.9381	19586.99	231.9434	19587.89	231.9396	19588.1	231.9426	19592.28
19580.83	232.1868	19584.27	232.1931	19587.22	232.1909	19584.08	232.1931	19588.01	232.1973	19587.46	232.1912	19591.9
19583.83	232.4424	19586.63	232.4397	19581.22	232.4403	19586.01	232.4455	19586.58	232.4402	19592.27	232.4511	19589.73
19575.81	232.6904	19581.43	232.6947	19593.32	232.6938	19590.28	232.6922	19586.4	232.7039	19590.76	232.6896	19591.74
19582.15	232.9507	19585.25	232.9412	19584.23	232.9398	19581.14	232.9432	19588.77	232.9408	19590.41	232.9444	19589.86
19588.98	233.1872	19588.12	233.1938	19588.65	233.1903	19590.94	233.194	19588.78	233.1951	19592.44	233.1898	19589.87
19583.24	233.4427	19583.65	233.4388	19585.55	233.4463	19581.51	233.4439	19588.79	233.443	19588.62	233.4494	19590.89
19583.92	233.6907	19580.47	233.694	19593.4	233.6938	19577.93	233.6934	19588.57	233.7021	19589.89	233.6892	19588.87
19579.43	233.9491	19586.67	233.9418	19588.72	233.9401	19582.48	233.9447	19587.58	233.9393	19589.86	233.9442	19589.46
19577.23	234.187	19578.28	234.1939	19581.88	234.1937	19588.55	234.194	19586.42	234.2032	19588.23	234.1915	19588.83
19580.86	234.4458	19585.62	234.4392	19580.18	234.4422	19585.27	234.4457	19588.5	234.4506	19587.81	234.4542	19587.56
19586.45	234.6882	19581.85	234.6936	19587.97	234.6953	19581.6	234.6953	19589.3	234.7061	19588.87	234.6946	19586.37
19584.24	234.9493	19584.71	234.9447	19586.55	234.9381	19587.76	234.9472	19590.24	234.9518	19591.79	234.9439	19589.17
19583.5	235.1892	19579.83	235.1961	19580.64	235.1948	19587.29	235.1944	19588.58	235.196	19589.46	235.1923	19588.76
19580.8	235.4469	19590.84	235.4381	19583.18	235.4428	19587.37	235.4486	19587.21	235.4467	19588.5	235.4685	19588.98
19585.59	235.6871	19579.56	235.6959	19580.05	235.6961	19581.96	235.6922	19588.63	235.7087	19591.63	235.7087	19589.96
19584.5	235.9495	19578.45	235.9406	19581.13	235.937	19585.71	235.9451	19590.81	235.9541	19588.25	235.9516	19588.73
19587.37	236.19	19588.18	236.1982	19580.85	236.1953	19589.87	236.1961	19588.36	236.2036	19588.93	236.1888	19588.7
19586.2	236.4459	19591.13	236.4403	19583.77	236.4401	19588.68	236.4477	19589.01	236.4487	19588.85	236.4504	19590.06
19577.04	236.6891	19578.82	236.7025	19587.26	236.6983	19586.04	236.6946	19590.08	236.7062	19590.72	236.6934	19587.32
19583.52	236.946	19582.07	236.9442	19581.47	236.9384	19590.28	236.9464	19587.72	236.9489	19588.86	236.9438	19589.33
19579.18	237.1873	19578.75	237.1951	19591.14	237.1987	19587.46	237.1964	19586.25	237.2066	19587.92	237.1939	19589.1
19581.09	237.4456	19585.22	237.4398	19579.54	237.4431	19594.04	237.4491	19588.09	237.4928	19588.3	237.4535	19590.42
19583.73	237.6895	19580.45	237.6995	19585.37	237.6985	19591.06	237.6905	19587.77	237.9695	19589.43	237.6935	19589.02
19587.47	237.9501	19580.3	237.9427	19584.53	237.9411	19586.66	237.9466	19590.05	238.2766	19590.16	237.9445	19590.85
19588.41	238.1922	19584.35	238.1962	19585.35	238.1945	19583.54	238.1971	19587.6	238.7617	19587.92	238.1926	19590.45
19580.05	238.4452	19580.93	238.442	19584.15	238.4422	19589.68	238.4473	19585.16	239.0081	19589.4	238.4518	19588.89
19588.19	238.6915	19576.62	238.699	19578.46	238.6995	19585.63	238.6938	19589.12	239.2865	19589.05	238.6926	19589.94
19584.14	238.9584	19585.68	238.9494	19583.6	238.9499	19585.25	238.9552	19586.91	239.7756	19590.07	238.956	19590.21
19579.33	239.201	19583.33	239.1984	19582.93	239.1956	19588.49	239.1978	19586.52	240.0178	19589.66	239.2144	19591.72
19582.52	239.4477	19577.1	239.4395	19585.21	239.4409	19583.4	239.4497	19588.49	240.2634	19591.47	239.4694	19587.03
19581.24	239.6925	19584.19	239.6973	19585.34	239.6992	19582.06	239.691	19590.26	240.5044	19588.27	239.7131	19591.94
19583.02	239.9516	19587.63	239.9427	19582.4	239.9428	19581.42	239.947	19590.41	240.7632	19588.48	239.9625	19589.01
19579.38	240.1955	19582	240.1969	19576.85	240.1969	19581.95	240.1973	19586.87	241.0065	19589.33	240.2034	19588.57
19581.8	240.4426	19580.98	240.4413	19578.91	240.4418	19583.96	240.4473	19588.2	241.2592	19588.47	240.4541	19590.8
19587.66	240.6879	19578.94	240.6945	19575.58	240.7028	19581.79	240.6973	19586.87	241.5043	19587.05	240.6915	19590.92
19585.61	240.948	19587.84	240.9455	19590.1	240.937	19579.18	240.9448	19588.09	241.7882	19589.64	240.9435	19588.23
19580.49	241.1875	19588.85	241.1934	19584.44	241.1909	19586.92	241.2168	19587.26	242.0266	19587.44	241.1916	19590.4
19581.77	241.4424	19582.95	241.4387	19583.21	241.4393	19584.13	241.449	19588.86	242.2609	19586.81	241.4505	19589.56
19577.7	241.6885	19579.88	241.6934	19582.13	241.696	19581.62	241.6926	19589	242.5095	19588.29	241.6959	19588.52
19584.78	241.9473	19583.79	241.9407	19585.05	241.9384	19579.07	241.9434	19590.63	242.7672	19589.81	241.9414	19592.36
19582.08	242.19	19581.23	242.194	19578.27	242.19	19587.83	242.1944	19584.22	243.0075	19590.31	242.1895	19590.01
19583.06	242.4435	19585.08	242.4377	19588.26	242.4418	19586.21	242.4461	19587.74	243.2602	19588.31	242.4498	19589.54
19589.2	242.6906	19579.8	242.6928	19579.56	242.6957	19585.78	242.6915	19590.98	243.5086	19590.22	242.6936	19587.33
19584.37	242.9475	19571.7	242.9447	19582.2	242.9397	19589.4	242.9442	19586.65	243.7713	19589.04	242.9437	19591.21
19574.63	243.1892	19583.31	243.1934	19585.23	243.191	19584.1	243.1937	19587.6	244.0044	19586.86	243.1906	19586.21
19580.2	243.4418	19586.14	243.4388	19583.56	243.4406	19586.14	243.4457	19587.44	244.262	19586.65	243.4528	19589.08
19582.37	243.6907	19579.01	243.6935	19584.49	243.6948	19584.96	243.6909	19588.29	244.5078	19590.39	243.693	19588.86

19578.39	243.9503	19582.58	243.943	19584.19	243.9394	19585.32	243.9434	19588.65	244.7671	19589.91	243.9449	19590.14
19580.57	244.1899	19584.02	244.1954	19584.86	244.1911	19585.58	244.197	19588.52	245.0088	19587.07	244.1921	19588.35
19581.1	244.4463	19588.92	244.4396	19586.53	244.4409	19584.31	244.4446	19589.75	245.2652	19588.8	244.4504	19588.32
19575.69	244.6912	19581.91	244.6931	19580.44	244.6945	19583.97	244.6911	19587.71	245.5058	19590.44	244.6919	19588.47
19584.21	244.9488	19585.61	244.9477	19578.54	244.9404	19582.67	244.945	19586.43	245.768	19587.38	244.9454	19586.46
19577.59	245.1912	19582.93	245.1967	19579	245.1929	19585.05	245.1988	19589.49	246.0117	19587.52	245.1928	19588.94
19583.22	245.4435	19586.01	245.441	19580.07	245.4405	19585.24	245.4465	19590.72	246.2634	19591.95	245.452	19588.17
19582.32	245.6903	19583.5	245.6993	19577.38	245.6973	19588.49	245.693	19587.68	246.5093	19590.33	245.6918	19589.9
19583.54	245.9479	19577.93	245.9429	19585.65	245.9398	19581.53	245.9487	19588.66	246.7697	19589.62	245.9463	19590.29
19583	246.191	19583	246.1954	19587.4	246.1916	19587.96	246.1951	19589.21	247.0077	19589.53	246.1935	19587.36
19586.67	246.4458	19581.97	246.4431	19582.01	246.4412	19586.45	246.445	19586.71	247.2644	19588.57	246.4539	19589.44
19582.5	246.6904	19581.87	246.6961	19586.15	246.6981	19592.17	246.6936	19589.88	247.5131	19590.13	246.6907	19589.35
19582.93	246.9493	19581.81	246.9462	19580.37	246.9419	19587.13	246.9449	19589.68	247.7677	19585.81	246.9453	19588.85
19583.14	247.1914	19581.93	247.1978	19582.83	247.1928	19584.34	247.1963	19588.38	248.0118	19588.71	247.1913	19591.74
19581.1	247.4453	19578.88	247.4405	19585.01	247.4429	19586.96	247.4462	19588.41	248.2656	19584.94	247.4515	19589.03
19579.88	247.6926	19582.57	247.6949	19585.27	247.6962	19585.54	247.6931	19589.24	248.5185	19587.42	247.6942	19587.78
19581.02	247.9515	19587.52	247.9467	19582.43	247.9406	19579.26	247.9448	19590.8	248.7955	19586.75	247.9455	19587.61
19585.53	248.192	19579.36	248.2001	19580.79	248.1929	19585.18	248.1968	19585.55	249.2787	19591.38	248.196	19588.41
19584.77	248.4439	19588.29	248.4393	19591.13	248.452	19593.91	248.4469	19586.12	249.5263	19585.68	248.4503	19591.71
19585.32	248.693	19587.76	248.6961	19578.2	248.7019	19582.14	248.695	19587.21	249.8197	19590.14	248.694	19588.96
19580.83	248.9491	19576.4	248.9453	19582.51	248.9416	19587.9	248.947	19584.97	250.2955	19591.54	248.9454	19589.06
19575.85	249.1924	19578.74	249.1943	19588.06	249.1944	19585.3	249.1972	19587.96	250.5419	19587.04	249.1935	19587.89
19580.17	249.4461	19584.75	249.4444	19580.53	249.445	19585.52	249.4512	19589.7	250.7996	19586.82	249.4524	19589.08
19580.91	249.6922	19582.16	249.6958	19584	249.6979	19583.41	249.7011	19586.05	251.043	19591.9	249.6924	19589.46
19582.46	249.9512	19582.96	249.9454	19586.51	249.9422	19580.01	249.9456	19588.1	251.2951	19589.81	249.9479	19589.62
19581.3	250.1933	19586.84	250.1949	19585.34	250.1942	19582.8	250.1978	19587.6	251.5434	19588.72	250.1937	19587.28
19579.42	250.4461	19581.52	250.4425	19588.67	250.4449	19587.3	250.4471	19587.72	251.7984	19588.77	250.4521	19589.95
19580.67	250.6935	19579.78	250.6966	19582.96	250.7003	19582.73	250.6977	19589.03	252.0435	19585.81	250.6931	19586.29
19578.91	250.9506	19581.28	250.9481	19576.03	250.9418	19586.44	250.9464	19587.42	252.2966	19589.79	250.9493	19590.79
19578.95	251.1936	19585.31	251.1962	19579.29	251.1964	19586.38	251.2018	19589.51	252.5439	19585.9	251.1944	19589.56
19581.71	251.4469	19584.56	251.4457	19583.47	251.4443	19582.43	251.4491	19586.46	252.8117	19588.22	251.4528	19588.4
19579.43	251.6933	19582.03	251.6961	19580.49	251.6997	19587.15	251.6952	19588.53	253.0432	19589.46	251.6946	19587.19
19577.4	251.9511	19577.79	251.9497	19579.48	251.9436	19581.85	251.9484	19591.38	253.2955	19590.18	251.9481	19585.15
19579.68	252.194	19582.94	252.1964	19580.99	252.1936	19591.83	252.1964	19589.1	253.5436	19589.27	252.1943	19588.78
19582.68	252.4484	19584.35	252.4427	19585.42	252.4438	19586.56	252.4494	19587.53	253.8013	19588.94	252.4525	19587.58
19579	252.695	19583.9	252.6948	19582.2	252.6974	19583.79	252.6991	19588.57	254.0442	19587.81	252.6948	19588.58
19577.93	252.9522	19580.41	252.9451	19587.42	252.9433	19586.75	252.9514	19590.71	254.2952	19589.07	252.9486	19589.47
19582.58	253.1942	19582.59	253.1968	19580.92	253.1965	19581.21	253.1991	19587.37	254.5507	19587.14	253.1952	19588.6
19580.61	253.446	19580.16	253.4452	19587.43	253.447	19585.69	253.4491	19587.67	254.8155	19588.45	253.4521	19590.09
19578.28	253.6958	19582.05	253.6996	19581.59	253.7016	19580.22	253.6958	19586.3	255.043	19588.51	253.6963	19588.54
19581.24	253.9563	19582.96	253.9464	19578.89	253.9438	19588.88	253.9488	19585.88	255.2966	19589.94	253.9486	19588.26
19587.41	254.1946	19587.33	254.1959	19582.17	254.1958	19580.67	254.1986	19587.29	255.5447	19588.1	254.1957	19587.66
19580.58	254.4471	19580.97	254.4461	19578.89	254.4463	19583.52	254.4499	19586.02	255.8027	19588.27	254.4532	19592.23
19578.36	254.6959	19581.01	254.6985	19584.08	254.7015	19589.52	254.7034	19589.26	256.0457	19586.82	254.6963	19587.09
19581.77	254.9552	19585.68	254.9491	19580.22	254.9456	19581.81	254.9541	19589.01	256.2968	19590.02	254.9477	19588.56
19582.69	255.1947	19581.22	255.197	19576.98	255.1958	19585.95	255.2125	19589.72	256.5441	19585.68	255.1939	19590.36
19577.07	255.4485	19581.98	255.4527	19578.9	255.4477	19580.63	255.4466	19589.22	256.8008	19587.95	255.456	19588.67
19583.23	255.6934	19581.91	255.6981	19577.56	255.701	19590.75	255.6991	19584.68	257.0442	19590.35	255.6961	19587.27
19580.56	255.9524	19583.81	255.9476	19581.82	255.9441	19584.07	255.9488	19588.15	257.2975	19589.05	255.9497	19591.4
19580.44	256.1947	19585.08	256.201	19581.97	256.1964	19584.31	256.2034	19591.39	257.5454	19590.01	256.1942	19587.06
19582.83	256.4492	19585.93	256.4448	19581.12	256.4458	19584.7	256.455	19586.96	257.801	19589.01	256.4548	19587.94
19584.73	256.6979	19583.39	256.6978	19582.7	256.7008	19583.82	256.702	19588.13	258.0443	19587.55	256.6971	19587.9
19582.78	256.9542	19581.8	256.9475	19577.78	256.9445	19584.27	256.95	19586.28	258.2986	19587.73	256.95	19591.39
19582.46	257.1993	19584.15	257.1999	19585.43	257.1975	19586.67	257.2007	19588.52	258.5459	19588.82	257.1959	19587.25
19580.49	257.4541	19581.44	257.447	19586.21	257.448	19580.41	257.4494	19586.84	258.8024	19586.56	257.4516	19588.19
19581.22	257.6963	19583.66	257.6977	19588.23	257.7018	19587.1	257.6975	19587.94	259.0459	19588.54	257.6978	19588.71
19585.4	257.9595	19584.79	257.9509	19579.81	257.9459	19581.18	257.9506	19588.03	259.2979	19587.69	257.955	19589.05
19582.98	258.1927	19583.47	258.1987	19590.02	258.1964	19592.12	258.2012	19588.83	259.5452	19586.11	258.1961	19587.53
19582.17	258.4478	19584.08	258.4468	19579.12	258.4476	19584.16	258.4492	19588.37	259.802	19589.38	258.4534	19589.37
19576.84	258.6956	19580.7	258.7001	19586.69	258.7022	19586.55	258.7011	19589.43	260.0482	19588.45	258.6983	19588.94
19583.64	258.9534	19584.7	258.9492	19580.4	258.9453	19581.13	258.9505	19588.25	260.2984	19589.25	258.9515	19588.82
19582.67	259.1989	19575.91	259.1994	19578.82	259.1971	19586.5	259.2011	19585.31	260.546	19586.9	259.1975	19588.49
19583.23	259.4531	19579.83	259.4461	19582.78	259.4517	19581.11	259.4512	19586.65	260.8045	19585.27	259.4554	19589.67
19587.89	259.6969	19582.17	259.7	19582.18	259.704	19588.54	259.6989	19586	261.0478	19587.65	259.6963	19588.61
19584.13	259.9544	19579.3	259.9492	19582.25	259.9454	19583.58	259.9514	19587.54	261.2994	19586.97	259.9525	19587.31
19579.8	260.1959	19584.53	260.1996	19581.96	260.1984	19590.9	260.2024	19588.4	261.5476	19587.41	260.1978	19588.88
19581.19	260.4498	19577.21	260.4479	19584.98	260.4561	19583.82	260.4531	19587.67	261.8043	19589.79	260.4563	19590.67
19576.98	260.6973	19580.06	260.7009	19584.21	260.6987	19589.29	260.6992	19590.61	262.047	19586.74	260.6973	19589.23
19582.8	260.9541	19585.01	260.9483	19585.19	260.9463	19575.34	260.9498	19586.55	262.3	19591.95	260.9543	19590.03
19582.54	261.1966	19577.41	261.2011	19579.38	261.1982	19586.63	261.1997	19586.98	262.5514	19590.57	261.1978	19590.85

19581.86	261.4507	19578.48	261.4475	19586.14	261.4491	19584.2	261.4515	19588.7	262.804	19590.63	261.4587	19589.81
19582.91	261.6976	19580.74	261.7028	19582.32	261.7038	19585.83	261.7041	19586.56	263.0487	19589.21	261.7071	19587.54
19579.35	261.9523	19586.57	261.9492	19585.84	261.9469	19583.49	261.9507	19588.64	263.2998	19586.03	261.9511	19589.7
19582.27	262.1965	19575.53	262.1999	19587.7	262.201	19592.12	262.2013	19589.89	263.5455	19590.74	262.1987	19588.76
19575.73	262.4517	19577.8	262.4645	19581.47	262.4486	19579.04	262.4519	19585.98	263.8043	19588.68	262.459	19590.06
19577.92	262.7	19576.87	262.7032	19580.17	262.7015	19585.67	262.6952	19586.6	264.0486	19588.38	262.7015	19586.17
19577.81	262.9527	19577.33	262.9509	19583.88	262.9484	19584.97	262.9531	19586.89	264.3002	19586.64	262.946	19589.37
19582.05	263.1989	19575.34	263.1987	19580.61	263.1964	19590.08	263.2007	19584.41	264.544	19588.35	263.1984	19592.42
19580.55	263.4492	19581.32	263.4455	19578.27	263.4483	19592.43	263.4485	19588.43	264.8059	19586.26	263.4543	19587.16
19575.14	263.6924	19579.02	263.6964	19579.76	263.7051	19584.96	263.7004	19586.08	265.051	19588.04	263.7012	19589.64
19579.92	263.958	19576.91	263.9467	19583.47	263.9578	19587.41	263.9511	19586.49	265.2979	19586.65	263.951	19588.16
19580.8	264.2056	19577.47	264.1992	19579.48	264.2045	19582.04	264.2011	19585.57	265.5494	19588.02	264.1938	19587.62
19582.78	264.4564	19576.22	264.4459	19574.67	264.4538	19591.03	264.4499	19589.13	265.8016	19588.83	264.4783	19588.52
19582.81	264.7024	19587.34	264.7011	19586.07	264.7057	19581.11	264.6938	19586.83	266.049	19586.45	264.9616	19588.36
19579.44	264.9546	19582.17	264.956	19581.61	264.9543	19577.83	264.9523	19589.74	266.2958	19588.47	265.2074	19589.67
19580.77	265.2028	19583.1	265.206	19581.69	265.205	19586.6	265.203	19588.68	266.5489	19589.99	265.4675	19587.88
19579.43	265.4558	19580.72	265.4543	19583.01	265.4576	19584.96	265.4523	19588.43	266.8034	19587.72	265.713	19588.05
19580.8	265.7013	19578.52	265.7055	19581.37	265.7107	19574.4	265.7001	19585.55	267.049	19589.39	265.9626	19589.33
19577.41	265.9772	19576.53	265.9545	19588.14	265.9587	19583.72	265.9518	19588.63	267.2971	19587.18	266.2079	19586.57
19580.12	266.4593	19583.84	266.2101	19583.59	266.2092	19583.4	266.1999	19585.9	267.5495	19587.52	266.4699	19588.28
19585.69	266.7066	19587.05	266.4613	19584.72	266.4597	19586.17	266.4511	19589.49	267.8027	19587.2	266.714	19588.07
19578.91	266.9628	19582.02	266.7124	19584.93	266.713	19585	266.6983	19589.07	268.0499	19588.65	266.9631	19588.38
19583.94	267.2121	19581.33	266.9658	19580.71	266.9623	19577.31	266.9508	19588.2	268.2994	19591.29	267.2087	19586.51
19579.77	267.4642	19581.12	267.2203	19586.33	267.2141	19586.94	267.2046	19586.59	268.5489	19588.95	267.4628	19589.92
19584.64	267.7126	19581.57	267.4696	19582.04	267.4662	19581.12	267.4532	19586.18	268.8226	19587.53	267.7079	19589.26
19578.8	267.9677	19585.92	267.7287	19587.05	267.7213	19580.02	267.7001	19586.79	269.3074	19588.48	267.9601	19584.9
19582.86	268.2183	19577.07	268.213	19585.67	267.9728	19589.57	267.948	19587.96	269.5547	19587.95	268.2063	19587.47
19578.31	268.4707	19590.05	268.4635	19585.39	268.4568	19589.45	268.2022	19585.09	269.8175	19590.6	268.4674	19588.65
19579.3	268.7194	19581.84	268.7166	19578.71	268.7142	19584.16	268.4532	19585.68	270.0684	19587.87	268.7145	19589.14
19580.39	268.9753	19584.99	268.9702	19582.08	268.9638	19581.7	268.701	19587.45	270.3188	19585.51	268.9654	19589.78
19576.78	269.2273	19585.32	269.2232	19580.58	269.2153	19585.02	268.9535	19588.83	270.5666	19588.61	269.2122	19589.16
19579.04	269.4804	19580.59	269.4742	19581.36	269.4668	19585.25	269.2076	19587.64	270.8216	19586.52	269.4748	19590.82
19579.73	269.9693	19581.3	269.7294	19581.16	269.7256	19579.76	269.4586	19589.82	271.0739	19590.41	269.7278	19588.96
19575.76	270.2243	19581.04	269.9831	19585.21	269.9792	19589.26	269.7071	19585.77	271.3248	19586.04	269.9802	19588.4
19577.9	270.4768	19583.3	270.4657	19578.6	270.4707	19581.84	269.9614	19587.4	271.5735	19590.5	270.2305	19588.37
19581.32	270.7279	19586.89	270.7236	19592.12	270.7292	19584.65	270.2329	19588.35	271.8298	19587.41	270.4935	19586.48
19589.56	270.9872	19580.83	270.9797	19580.65	270.9825	19582.44	270.7241	19587.94	272.3167	19585.98	270.9789	19588.44
19576.17	271.4713	19581.13	271.2357	19576.91	271.4664	19579.74	270.9754	19590.51	272.5649	19590.48	271.2281	19591.01
19578.63	271.7246	19588.57	271.486	19588.17	271.7231	19587.32	271.2303	19587.8	272.8229	19587.21	271.487	19588.97
19580.17	271.9864	19577.11	271.7423	19580.06	271.9774	19581.39	271.4806	19587.04	273.0719	19584.29	271.7371	19589.13
19575.95	272.2429	19582.05	272.2292	19586.07	272.2313	19591.85	271.7297	19587.31	273.3239	19585.77	271.99	19588.32
19580.5	272.7253	19586.04	272.4797	19587.06	272.4862	19586.86	271.9828	19585.61	273.574	19588.95	272.2412	19589.12
19580.27	272.9846	19580.24	272.7342	19586.02	272.9686	19587.04	272.2356	19585.8	273.8328	19587.76	272.4987	19588.2
19579.22	273.2392	19577.68	272.9898	19585.89	273.2248	19581.9	272.4866	19587.17	274.3213	19589.08	272.9861	19588.89
19576.69	273.4946	19587.28	273.2468	19579.33	273.4789	19587.19	272.974	19588.54	274.5724	19587.61	273.2364	19587.63
19579.22	273.7472	19578.28	273.7353	19582.22	273.74	19588.53	273.2275	19585.32	274.8341	19586.72	273.4978	19585.58
19580.8	274.2332	19579.7	273.991	19581.79	274.2293	19586.53	273.4788	19589.05	275.0857	19589.02	273.7485	19590.98
19584.98	274.4903	19582.4	274.2454	19583.03	274.4841	19586.52	273.7299	19588.6	275.5761	19587.53	274.0085	19589.82
19581.54	274.7418	19583	274.4991	19581.8	274.7427	19585.6	273.9854	19587.76	275.835	19587.64	274.5068	19590.34
19576.35	275.0016	19584.56	274.7553	19586.74	274.9975	19585.01	274.2379	19587.83	276.0889	19583.22	274.759	19587.69
19580.82	275.4877	19579.59	275.2461	19584.24	275.4823	19582.78	274.4891	19586.72	276.342	19589.21	275.0124	19588.79
19574.64	275.7396	19587.56	275.4998	19587.9	275.7405	19577.09	274.7413	19588.68	276.5962	19586.74	275.5087	19588.95
19584.54	275.9974	19581.87	275.7543	19587.88	275.9947	19585.16	275.2295	19587.94	277.083	19588.58	275.7602	19589.57
19577.84	276.2517	19579.1	276.012	19580.04	276.2516	19588.51	275.484	19587.45	277.338	19589.04	276.0156	19589.32
19576.94	276.5073	19579.8	276.2695	19582.43	276.7429	19589.48	275.7367	19585.43	277.5917	19585.13	276.5151	19588.99
19583.04	277.0028	19578.55	276.7626	19584.15	276.9988	19585.89	275.9905	19586.39	277.8482	19589.15	277.0195	19586.16
19582.54	277.2596	19584.35	277.0228	19578.88	277.2574	19588.23	276.2445	19586.88	278.0998	19584.77	277.2712	19587.28
19581.69	277.5155	19580.67	277.5072	19580.45	277.7481	19587.03	276.7316	19587.68	278.5862	19588.65	277.5356	19587.16
19582.1	278.0111	19590.43	277.7675	19578.78	278.0059	19585.78	276.9871	19586.23	278.8474	19586.99	278.0265	19589.24
19574.9	278.2663	19585.48	278.0272	19581.75	278.2641	19588.04	277.2412	19586.76	279.0993	19587.62	278.2781	19587.2
19581.66	278.5247	19581.61	278.5133	19584.2	278.7549	19584.22	277.4956	19586.11	279.3545	19587.44	278.5439	19587.81
19579.38	279.0269	19587.24	278.7715	19591.76	279.014	19582.62	277.7472	19586.7	279.8496	19588.79	279.0345	19590.71
19577.78	279.2904	19576.76	279.0411	19580.66	279.2708	19586.73	278.0046	19588.22	280.1021	19588.48	279.2865	19587.19
19580.38	279.7777	19579.71	279.5255	19577.9	279.765	19586.55	278.4926	19585.1	280.3575	19586.93	279.5489	19588.82
19577.28	280.0383	19580.37	279.7849	19581.41	280.0253	19585.3	278.7471	19589.71	280.8533	19588.06	280.0413	19586.42
19578.51	280.2944	19584.16	280.0445	19582.26	280.2843	19586.04	279.0013	19585.53	281.1054	19587.86	280.2971	19588.08
19579.54	280.7796	19584.36	280.5317	19585	280.7738	19585.42	279.2594	19588.07	281.362	19586.79	280.5639	19587.9
19583.24	281.0383	19582.05	280.795	19580.77	281.0335	19582.81	279.7471	19588.04	281.8657	19588.62	281.0545	19585.46
19579.97	281.2965	19581.89	281.055	19584.07	281.2908	19580.52	280.0043	19588.09	282.3559	19586.01	281.3092	19588.6
19583.07	281.5546	19582.94	281.5409	19585.49	281.7826	19585.58	280.2608	19587.35	282.609	19587.98	281.5703	19590.54

19573.05	282.0484	19582.49	281.8053	19581.7	282.0443	19588.58	280.519	19584.54	282.8775	19587.54	282.0619	19589.24
19576.67	282.306	19580.66	282.0642	19577.94	282.5335	19589.5	281.0083	19588.82	283.3684	19586.94	282.3183	19587.03
19580.84	282.5643	19586.46	282.5502	19583.07	282.7954	19588.55	281.2632	19587.67	283.6221	19589.12	282.5795	19590.41
19579.33	283.0563	19583.67	282.8134	19581.89	283.0586	19582.38	281.5192	19588.76	283.8796	19590.76	283.0705	19585.02
19579.89	283.3144	19578.8	283.0735	19583.09	283.5491	19590.02	281.7773	19586.32	284.1345	19590.07	283.3261	19585.52
19576.43	283.8082	19579.84	283.5604	19582.54	283.809	19583.53	282.2679	19589.38	284.6265	19588.35	283.5886	19590.45
19576.5	284.0714	19581.25	283.8252	19581.22	284.0718	19589.96	282.5264	19586.38	284.8858	19588.96	284.0815	19588.05
19579.63	284.3321	19586.43	284.0868	19582.55	284.5648	19586.08	282.7847	19584.79	285.1403	19587.97	284.3378	19588.21
19582.5	284.8223	19584.67	284.5767	19575.67	284.8275	19583.02	283.2787	19585.18	285.6282	19585.1	284.6172	19587.43
19583.18	285.0856	19583.31	284.8434	19581.42	285.3153	19585.48	283.535	19587.8	285.8901	19588.27	285.1099	19587.53
19580.66	285.5753	19577.88	285.3304	19580.61	285.5828	19582.22	283.794	19586.32	286.1474	19588.11	285.3662	19587.07
19580.73	285.8332	19584.69	285.5924	19582.87	286.0762	19591.19	284.2881	19584.72	286.6357	19585.05	285.6303	19588.61
19582.39	286.097	19583.74	285.8599	19583.94	286.3358	19580.74	284.5458	19587.8	286.8947	19587.63	286.1217	19587.29
19580.51	286.5872	19581	286.3509	19580.43	286.6032	19591.44	284.8058	19586.13	287.15	19587.67	286.3818	19591.55
19576.56	286.8473	19576.66	286.613	19581.37	287.0945	19589.27	285.2997	19590.78	287.4081	19588.18	286.643	19590.3
19577.66	287.1101	19582.28	287.1087	19583.79	287.3541	19590.69	285.5575	19586.01	287.9009	19587.56	287.1348	19586.83
19576.47	287.6011	19582.97	287.3701	19586.42	287.6189	19583.22	285.8191	19587.16	288.1593	19588.78	287.3939	19590.14
19580.01	287.8625	19581.82	287.6319	19586.22	288.1209	19584.49	286.314	19588.03	288.4162	19587.34	287.6582	19585.98
19583.57	288.124	19580.61	288.1224	19581.88	288.3823	19582.45	286.5738	19587.13	288.9117	19588.45	288.15	19586.57
19584.12	288.6141	19580.99	288.381	19579.6	288.8721	19580.53	287.0676	19587.85	289.1681	19586.46	288.4064	19587.66
19580.3	288.8837	19579.02	288.6446	19582.5	289.1377	19584.28	287.3315	19588.43	289.6623	19588.15	288.666	19588.15
19577.03	289.38	19579.12	289.137	19582.89	289.6318	19582.69	287.8253	19583.16	289.9231	19586.99	289.158	19585.76
19579.07	289.6445	19580.57	289.3973	19578.2	289.8992	19580.09	288.088	19587.27	290.1822	19586.97	289.4202	19588.8
19579.18	290.1354	19582.56	289.6615	19581.64	290.3891	19584.5	288.5788	19587.16	290.685	19586.77	289.9122	19584.65
19578.12	290.3998	19582.37	290.1554	19585.54	290.6575	19579.3	288.841	19588.27	290.9459	19588.53	290.1743	19587.59
19581.91	290.8937	19578.64	290.4163	19580.06	291.152	19590.92	289.336	19588.78	291.4378	19585.99	290.6661	19588.54
19573.51	291.1598	19584.89	290.9122	19580.32	291.4146	19577.57	289.5944	19587.29	291.7035	19586.7	290.9267	19586.51
19577.49	291.6492	19578.43	291.1802	19579.09	291.9059	19585.91	290.0948	19586.59	292.1975	19588.73	291.1887	19585.93
19582.37	291.9189	19581.97	291.6791	19581.56	292.1712	19579.54	290.3591	19586.48	292.456	19588.09	291.6817	19587.66
19577.56	292.42	19579.04	291.9465	19587.21	292.6673	19581.12	290.8544	19587.77	292.7269	19586.9	291.9473	19588.19
19578.81	292.6827	19581.75	292.4377	19582.79	292.9313	19584.88	291.1206	19587.63	293.223	19586.13	292.446	19591.63
19582.73	293.175	19579.62	292.7027	19581.21	293.4232	19589.02	291.6171	19588.57	293.4836	19586.53	292.7137	19587.49
19575.78	293.4426	19577.78	293.1986	19580.59	293.6936	19582.45	291.886	19587.46	293.9773	19587.6	293.2058	19591.37
19580.62	293.942	19580.75	293.4617	19583.85	294.1868	19575.64	292.3851	19588.32	294.2398	19588.92	293.4721	19588.75
19574.29	294.2065	19580.44	293.9586	19585.44	294.4514	19584.5	292.6478	19585.78	294.7478	19588.33	293.9666	19588.14
19579.67	294.7005	19578.09	294.2265	19581.05	294.9439	19582.02	293.1481	19589.38	295.3036	19588.75	294.2269	19587.42
19580.87	294.972	19581.33	294.7219	19577.99	295.2141	19586.44	293.4158	19586.05	295.8068	19589.9	294.7194	19589.29
19581.06	295.4702	19582.27	294.9925	19585.31	295.7137	19584.28	293.916	19588.31	296.3024	19587.6	294.9824	19588.27
	295.7338	19580.13	295.4854	19584.03	295.9898	19578.66	294.1885	19583.01	296.5626	19586.36	295.4805	19584.68
	296.2269	19581.47	295.7524	19578.87	296.4821	19582.22	294.6831	19586.11	297.0571	19588.21	295.7399	19585.87
	296.4974	19580.97	296.2493	19580.64	296.7532	19584.23	294.9546	19589.24	297.3211	19587.55	296.2313	19587.39
	296.9961	19583.47	296.5146	19585.89	297.247	19582.18	295.4556	19587.85	297.8208	19586.52	296.4989	19589.38
	297.2503	19588.01	297.0105	19580.01	297.5153	19581.68	295.9599	19586.32	298.0859	19585.67	296.9934	19588.38
	297.7422	19573.4	297.2795	19580.28	298.0085	19584.07	296.238	19588.42	298.5792	19587.05	297.254	19586.12
	297.9984	19581.22	297.7742	19582.71	298.2761	19580.47	296.733	19587.9	298.8485	19586.5	297.7463	19588.34
	298.2505	19585.05	298.0419	19585.26	298.7728	19580.59	296.9951	19581.94	299.3442	19588	298.01	19585.45
	298.5055	19584.94	298.5346	19581.75	299.0426	19580.95	297.4967	19586.03	299.6061	19586.76	298.5114	19587.08
	299.0075	19579.56	298.8036	19577.19	299.538	19581.55	297.7603	19587.08	300.1007	19585.09	298.7741	19586.79
	299.2604	19578.49	299.2984	19581.56	299.8095	19582.53	298.2593	19587.44			299.2677	19589.13
	299.7563	19580.18	299.5646	19579.87	300.3165	19578.87	298.5284	19587.83			299.5581	19588.11
	300.0178	19581.52	300.0635	19580.51			299.0233	19585.84			300.0556	19586.65
							299.3013	19586.57				
							299.7986	19585.2				
							300.0635	19587.12				

				1.43								
				30								
x_4		x_5		x_1		x_2		x_3		x_4		x
t (s)	P (cm^{-1})	t (s)	P (cm^{-1})	t (s)	P (cm^{-1})	t (s)	P (cm^{-1})	t (s)	P (cm^{-1})	t (s)	P (cm^{-1})	t (s)
0	19607.32	0	19604.76	0	19602.2	0	19603.83	0	19604.28	0	19602.86	0
0.66526	19604.95	0.65966	19604.77	1.59955	19601.92	0.67683	19602.12	0.66749	19603.45	0.66284	19603.56	0.67306
0.85821	19605.21	0.85289	19606.61	1.85038	19602.66	0.85297	19603.46	0.85629	19603.76	0.85166	19602.81	0.86043
1.10721	19608.02	1.10503	19606.28	2.09834	19603.23	1.1006	19601.88	1.10379	19604.59	1.10003	19604.16	1.10893
1.35129	19604.41	1.35068	19604.21	2.34948	19602.6	1.34911	19603.7	1.3558	19602	1.34923	19602.27	1.35771
1.60063	19604.8	1.60288	19604.44	2.59799	19603.08	1.6	19601.88	1.60488	19605.19	1.59934	19603.7	1.60945
1.85158	19603.42	1.85192	19605.47	2.85109	19602.83	1.85072	19602.65	1.85561	19602.46	1.84899	19603.22	1.85854
2.10441	19604.77	2.10458	19605.1	3.09953	19602.14	2.09917	19602.95	2.10265	19602.9	2.09958	19603.55	2.10769
2.3514	19603.69	2.35076	19606.8	3.35226	19601.72	2.34926	19603.64	2.35464	19604.23	2.34948	19604.12	2.35736
2.60064	19605.3	2.60215	19605.89	3.59738	19601.17	2.59998	19604.31	2.60478	19602.71	2.59838	19602.96	2.60837
2.8511	19604.87	2.8552	19605.4	3.8498	19602.54	2.85018	19602.81	2.85547	19602.72	2.84992	19602.36	2.85782
3.10575	19603.66	3.10531	19603.95	4.09928	19602.48	3.09899	19603.12	3.10297	19603.1	3.09767	19604.74	3.10962
3.35261	19606.63	3.35155	19604.04	4.3525	19602.83	3.34997	19603	3.35498	19602.96	3.34882	19603.65	3.35827
3.60099	19603.87	3.60333	19604.4	4.59774	19601.59	3.59874	19603.53	3.60747	19604.04	3.59916	19603.7	3.60865
3.85112	19604.55	3.85044	19605.36	4.84991	19603.65	3.84853	19603.9	3.85552	19603.52	3.84908	19606.21	3.85722
4.10692	19606.94	4.10465	19607.04	5.09953	19602.76	4.09853	19603.64	4.10378	19602.91	4.09745	19604.14	4.10765
4.35241	19606.58	4.35205	19603.98	5.35081	19602.52	4.35111	19604.46	4.35453	19602.81	4.35004	19605.05	4.35855
4.60217	19609.77	4.6022	19604.88	5.59897	19602.42	4.6001	19601.8	4.60494	19602.24	4.59912	19605.37	4.6108
4.85151	19605.54	4.85533	19604.6	5.85002	19603.32	4.84986	19601.82	4.85556	19605.72	4.8506	19602.76	4.85817
5.10606	19607.68	5.10285	19607.95	6.09937	19602.13	5.10694	19604.2	5.10823	19603.01	5.10079	19603.08	5.1081
5.35337	19605.6	5.35144	19605.52	6.35115	19601.17	5.35651	19601.89	5.35829	19604.67	5.34952	19603.71	5.35781
5.6021	19605.96	5.6083	19606.98	6.60042	19601.95	5.59971	19604.65	5.60561	19603.9	5.60322	19603.66	5.60904
5.85641	19606.83	5.86969	19608.22	6.85197	19603.39	5.85194	19603.14	5.85526	19602.74	5.85251	19603.14	5.85947
6.10839	19606.38	6.11294	19606.11	7.10091	19604.25	6.10072	19602.08	6.10356	19605	6.09983	19604.05	6.10913
6.35294	19604.37	6.35695	19602.57	7.35079	19600.35	6.35044	19603.74	6.35568	19603.38	6.35173	19604.28	6.35859
6.60513	19605.68	6.61169	19607.32	7.59896	19602.24	6.60048	19604.46	6.60588	19603.03	6.60016	19602.27	6.60946
6.85354	19605.61	6.85513	19602.98	7.85209	19603.76	6.85215	19603.45	6.85584	19604.51	6.85073	19603.91	6.85964
7.1079	19604.17	7.11441	19606.73	8.10101	19602.21	7.10194	19604.44	7.10432	19602.24	7.09906	19602.65	7.11014
7.35259	19606.15	7.35131	19603.38	8.35351	19603.25	7.35038	19602.71	7.35611	19602.28	7.35115	19603.39	7.35823
7.60277	19602.79	7.60311	19606.7	8.59878	19603.5	7.5998	19603.21	7.60592	19603.41	7.60018	19602.84	7.60914
7.85358	19603.8	7.85249	19605.54	8.85163	19603.63	7.85058	19604.04	7.85603	19604.13	7.85088	19603.44	7.85926
8.10591	19604.47	8.10803	19605.65	9.10116	19602.94	8.10178	19603.73	8.10514	19604.59	8.09873	19603.06	8.11236
8.35319	19606.14	8.35357	19606.7	9.3516	19601.53	8.35278	19601.93	8.3593	19603.51	8.35109	19602.16	8.36013
8.60212	19605.36	8.60252	19606.2	9.59876	19602.66	8.60117	19603.96	8.60608	19603.19	8.6001	19602.26	8.60867
8.85307	19606.29	8.85266	19606.49	9.85114	19604.62	8.85126	19604.11	8.85626	19603.03	8.85251	19602.99	8.86023
9.10613	19604.42	9.10541	19605.84	10.10167	19602.5	9.10111	19604.16	9.10426	19603.45	9.09981	19602.87	9.1103
9.35285	19606.34	9.36075	19607.15	10.35353	19602.84	9.3514	19603.11	9.35766	19603.39	9.3503	19603.23	9.36019
9.60252	19605.64	9.60277	19604.83	10.60034	19601.53	9.60194	19603.42	9.60752	19601.48	9.60026	19605.1	9.60994
9.85289	19603.96	9.85241	19605.51	10.85419	19602.97	9.85285	19603.67	9.85636	19603.26	9.85227	19605.04	9.85923
10.10704	19604.98	10.10496	19605.47	11.10178	19601.87	10.10236	19604.85	10.10467	19602.07	10.10051	19604.08	10.11063
10.35452	19605.65	10.35415	19605.16	11.35388	19601.98	10.35186	19602.63	10.3584	19603.13	10.35104	19601.83	10.36165
10.60327	19605.66	10.6069	19606.27	11.60119	19602.02	10.60211	19602.34	10.60803	19605.05	10.60041	19603.51	10.6121
10.85314	19605.16	10.85334	19605.75	11.8532	19602.1	10.85429	19603.39	10.85905	19603.64	10.85276	19602.85	10.85989
11.10775	19605.29	11.10768	19604.37	12.10162	19601.91	11.10354	19603	11.10631	19602.51	11.1018	19603.19	11.11242
11.35469	19602.61	11.3541	19608.33	12.35263	19601.16	11.35266	19601.56	11.35772	19603.48	11.35369	19601.44	11.36122
11.60415	19606.53	11.60558	19606.54	12.60198	19602.93	11.60137	19603.81	11.60897	19603.12	11.6008	19604.01	11.61225
11.85338	19604.62	11.8549	19608.26	12.85395	19603.22	11.85286	19601.29	11.85845	19602.78	11.8534	19604.44	11.8621
12.10773	19604.71	12.10834	19606.58	13.1022	19603.15	12.102	19603.33	12.10725	19602.55	12.102	19604.36	12.11149
12.35575	19606.21	12.35537	19605.52	13.35574	19600.05	12.35486	19604.22	12.35899	19603.8	12.35333	19603.21	12.36189
12.60468	19605.25	12.60632	19604.51	13.60075	19602.98	12.60337	19603.56	12.60768	19604.53	12.60238	19602.96	12.61145
12.85455	19604.01	12.85451	19605.46	13.85425	19602.61	12.85207	19602.56	12.85839	19605.1	12.85206	19604.3	12.86193
13.10813	19606.41	13.10747	19605.73	14.10178	19604.72	13.10225	19602.92	13.10721	19604.49	13.10122	19603.87	13.1125
13.35381	19603.77	13.35413	19606.49	14.35264	19601.73	13.35351	19603.14	13.35846	19604.51	13.35387	19604.15	13.36056
13.60467	19606.53	13.60574	19605.06	14.60108	19602.28	13.60348	19603.49	13.60728	19603.42	13.60175	19602.08	13.61253
13.85501	19606.69	13.8541	19606.73	14.85424	19603.32	13.85383	19602.77	13.85822	19603.54	13.85244	19604.19	13.86139
14.10849	19603.96	14.1072	19605.11	15.10367	19602.12	14.1019	19603.52	14.10634	19603.11	14.10067	19603.24	14.1127
14.35394	19605.99	14.35441	19605.71	15.35454	19602.09	14.35474	19603.96	14.35824	19604.34	14.35548	19604.12	14.36119
14.60471	19606.2	14.60651	19606.48	15.60166	19601.11	14.60299	19603.69	14.60886	19605.2	14.60242	19603.1	14.61188
14.85495	19603.82	14.85406	19605.48	15.85456	19602.76	14.85322	19601.47	14.85797	19603.59	14.85362	19603.36	14.86172
15.10729	19605.46	15.10867	19605.5	16.10449	19602.86	15.10348	19601.26	15.10599	19602.19	15.101	19602.67	15.11265
15.35543	19606.2	15.35611	19606.08	16.35611	19602.88	15.35507	19602.87	15.35924	19603.23	15.35433	19603.58	15.36267
15.60491	19608.28	15.60855	19604.7	16.60211	19601.77	15.60452	19604.91	15.60954	19603.4	15.60306	19604.4	15.61335
15.85661	19601.87	15.8551	19605.05	16.85535	19601.14	15.85475	19604.51	15.86002	19603.27	15.85518	19603.36	15.86282
16.10988	19603.31	16.10938	19605.73	17.10334	19602.61	16.10426	19602.37	16.10767	19603.11	16.10265	19603.93	16.11328

16.35669	19606	16.35626	19606.45	17.35486	19602.11	16.35527	19602.69	16.36035	19604.93	16.35321	19602.77	16.36828
16.60491	19605.36	16.60528	19605.98	17.60344	19602.59	16.60381	19602.02	16.61559	19602.94	16.60189	19601.91	16.61378
16.85467	19607.84	16.85498	19602.04	17.85502	19604.72	16.85503	19606.31	16.85997	19604.33	16.85433	19603.42	16.8631
17.10899	19607.83	17.10963	19608.28	18.10294	19602.02	17.1064	19601.53	17.10892	19602.41	17.10323	19604	17.11501
17.35665	19604.18	17.35594	19606.71	18.35749	19601.88	17.35642	19602.7	17.35978	19604	17.35533	19602.87	17.36319
17.60522	19605.48	17.60728	19607.59	18.60312	19600.66	17.6039	19603.86	17.60962	19604.63	17.60189	19602.82	17.61347
17.85512	19604.17	17.85502	19604.12	18.85612	19603.26	17.85462	19602.47	17.85914	19604.07	17.85645	19602.22	17.86415
18.1082	19604.84	18.1129	19603.49	19.10428	19601.63	18.10527	19603.02	18.10931	19604.65	18.10321	19604.26	18.11343
18.35654	19603.13	18.3559	19602.84	19.35529	19600.23	18.35629	19603.17	18.35993	19603.74	18.35484	19604.33	18.36289
18.60633	19604.67	18.60913	19603.57	19.60208	19601.38	18.60345	19605.88	18.61132	19603.58	18.60267	19604.11	18.61392
18.85573	19605.37	18.85604	19605.74	19.85589	19603.44	18.85477	19602.26	18.85948	19603.6	18.85477	19602.76	18.86455
19.10948	19604.72	19.10827	19604.46	20.10435	19603.98	19.10481	19604.2	19.10846	19603.11	19.1026	19603.3	19.11391
19.35633	19601.31	19.35652	19604.18	20.35775	19604.71	19.35704	19604.02	19.36073	19603.61	19.35602	19604.37	19.36278
19.60712	19603.23	19.60887	19604.9	20.60496	19602.11	19.60459	19603.42	19.6105	19604.27	19.60372	19602.64	19.61355
19.85759	19603.44	19.85652	19606.11	20.85636	19602.47	19.85537	19603.22	19.85981	19604.05	19.85561	19604.32	19.86327
20.57322	19603.75	20.11017	19604.68	21.10481	19603.54	20.10396	19602.35	20.10938	19603.98	20.10305	19602.3	20.11393
20.81997	19607.82	20.35631	19604.54	21.35779	19603.01	20.35816	19602.94	20.36256	19603.04	20.35482	19603.3	20.36515
21.07355	19603.65	20.61085	19603.54	21.60479	19602.17	20.607	19603.14	20.61186	19602.69	20.60627	19603.16	20.6148
21.31879	19605.26	20.85766	19608.31	21.85667	19603.76	20.85676	19602.52	20.86056	19603.09	20.85776	19603.41	20.86429
21.57003	19608.4	21.11495	19606.65	22.10507	19601.96	21.10634	19603.01	21.10957	19602.32	21.10527	19602.57	21.11597
21.8208	19603.24	21.35869	19603.9	22.35731	19603.47	21.35698	19603.42	21.3623	19603.53	21.35475	19603.51	21.36552
22.07009	19603.73	21.60902	19605.8	22.60597	19600.66	21.61388	19602.5	21.61345	19602.65	21.60461	19603.3	21.61501
22.31899	19605.66	21.85788	19603.92	22.85888	19602.03	21.86121	19602.04	21.8632	19603.56	21.85804	19604.07	21.86441
22.57059	19605.35	22.11122	19607.22	23.10504	19602.39	22.10713	19604.17	22.10981	19601.53	22.10685	19603.05	22.11535
22.82124	19605.6	22.35905	19606.9	23.35881	19600.06	22.35644	19604.48	22.36204	19604.3	22.35901	19604.98	22.36581
23.07036	19609.68	22.60867	19603.42	23.60517	19603.1	22.60546	19603.61	22.61504	19603.9	22.60472	19603.8	22.61626
23.32007	19607.97	22.85781	19606.37	23.85874	19602.54	22.85828	19604.63	22.86349	19603.33	22.85778	19603.11	22.86514
23.57008	19607.15	23.11025	19604.17	24.10685	19601.45	23.10753	19602.98	23.11058	19604.88	23.10604	19602.36	23.11615
23.82021	19607.03	23.36144	19600.87	24.35764	19603.24	23.35842	19601.65	23.36208	19604.27	23.35786	19603.54	23.36504
24.07082	19603.16	23.61111	19605.33	24.60356	19601.34	23.6067	19602.28	23.61322	19604.16	23.60581	19604.15	23.62067
24.32108	19606.85	23.85796	19605.34	24.86001	19602.34	23.85662	19603.62	23.86287	19604.97	23.85725	19603.1	23.86633
24.57012	19605.34	24.11296	19605.63	25.10685	19602.85	24.10645	19602.66	24.11134	19602.21	24.10489	19604.43	24.11672
24.81925	19605.07	24.35973	19608.53	25.36422	19603.72	24.35924	19604.02	24.36222	19602.62	24.35833	19603.7	24.36478
25.0705	19606.17	24.61201	19605.58	25.60606	19600.73	24.60743	19601.06	24.61167	19602.64	24.6064	19603.44	24.61577
25.32069	19607.2	24.85816	19605.77	25.86063	19602.12	24.85974	19603.5	24.86285	19604.9	24.85974	19604.13	24.86571
25.57157	19604.87	25.11159	19603.3	26.10647	19603.77	25.10625	19602.78	25.11023	19602.52	25.10426	19604.41	25.1167
25.82024	19603.74	25.35958	19606.92	26.35835	19602.59	25.35796	19603.59	25.36288	19603.87	25.35495	19602.79	25.3643
26.0697	19604.97	25.61315	19605.88	26.60704	19603.39	25.6073	19602.76	25.61688	19604.52	25.60556	19603.07	25.61506
26.32004	19604.25	25.85892	19606.63	26.85925	19602	25.85839	19602.9	25.86231	19603.81	25.85953	19602.61	25.86484
26.57142	19603.4	26.11258	19606.94	27.10704	19604.6	26.10692	19602.1	26.11053	19602.83	26.10597	19604.15	26.11722
26.82107	19604.95	26.35894	19606.13	27.35854	19600.73	26.35841	19603.73	26.36361	19603.32	26.35705	19604.31	26.36644
27.06961	19605.84	26.61212	19607.6	27.60567	19601.62	26.60756	19603.16	26.61481	19603.67	26.60532	19604.15	26.61623
27.32011	19605.49	26.85854	19604.7	27.8589	19603.53	26.85789	19601.91	26.86583	19604.05	26.85754	19603.26	26.86521
27.57094	19605.24	27.11428	19606.28	28.10742	19602.99	27.10826	19601.56	27.11055	19602.75	27.1072	19604.47	27.11627
27.8207	19604.87	27.35985	19602.83	28.36132	19602.75	27.3592	19604.23	27.36316	19602.71	27.35796	19601.04	27.36613
28.07152	19605.8	27.6118	19604.65	28.60615	19602.61	27.60741	19603.25	27.6155	19605.08	27.6051	19605.17	27.61738
28.32074	19606.35	27.85836	19602.07	28.85817	19601.93	27.86003	19603.82	27.86369	19603.02	27.85823	19603.51	27.86613
28.56982	19605.41	28.11265	19608.35	29.10847	19603.24	28.10926	19604.82	28.11072	19602.37	28.10618	19603.54	28.11713
28.82106	19605.07	28.35937	19605.11	29.35982	19600.82	28.35871	19602.5	28.36265	19602.56	28.35877	19603.63	28.36723
29.0721	19604.41	28.61267	19608.52	29.60745	19601.39	28.6075	19601.68	28.61685	19603.39	28.60724	19603.39	28.61721
29.32183	19605.02	28.85901	19606.94	29.85836	19602.42	28.85812	19605.08	28.86636	19603.9	28.85895	19603.23	28.8668
29.57056	19606.01	29.1123	19604.77	30.10745	19601.52	29.10824	19603.69	29.11305	19602.82	29.10577	19604.64	29.1175
29.82096	19606.47	29.35872	19601.7	30.36901	19602.99	29.36122	19603.14	29.36475	19604.24	29.35818	19601.91	29.36577
30.07203	19604.39	29.61322	19602.4	30.60855	19602.47	29.60844	19602.89	29.61437	19603.14	29.60756	19604.27	29.61797
30.32323	19604.88	29.86061	19606.04	30.86095	19601.89	29.86152	19604.94	29.86397	19602.95	29.85957	19605.12	29.86815
30.57312	19605.31	30.11483	19606.39	31.108	19602.38	30.10836	19600.78	30.11255	19603.67	30.10739	19603.65	30.11785
30.82201	19604.06	30.3613	19605.13	31.36098	19602.44	30.36196	19603.23	30.36473	19604.05	30.36005	19602.84	30.36701
31.07224	19602.8	30.61436	19603.18	31.60917	19602.46	30.61162	19603.06	30.61656	19604.61	30.60909	19602.01	30.61877
31.32312	19604.72	30.86208	19607.42	31.86594	19605.06	30.86071	19602.09	30.86481	19603.74	30.86011	19603.6	30.86776
31.57395	19606.36	31.11546	19609.35	32.1092	19602.14	31.10939	19602.03	31.11394	19602.51	31.10878	19603.66	31.12059
31.82311	19606.22	31.36073	19607.38	32.36166	19604.6	31.36241	19604.43	31.3668	19605.07	31.36028	19603.34	31.36973
32.0724	19607.31	31.61301	19606.25	32.60697	19604.19	31.61141	19604.73	31.61907	19603.17	31.60711	19603.5	31.61845
32.32369	19604.96	31.86175	19604.87	32.85907	19603.06	31.86179	19602.62	31.86575	19601.97	31.86044	19604.02	31.86913
32.57366	19603.66	32.11746	19606.5	33.10883	19602.66	32.10941	19602.71	32.11283	19604.07	32.10975	19604.41	32.1191
32.8235	19605.64	32.36219	19605.9	33.36408	19602.27	32.36159	19601.11	32.36704	19601.87	32.37604	19603.27	32.36932
33.07179	19603.39	32.61311	19605.02	33.60867	19601.44	32.6105	19603.59	32.61877	19603.83	32.60784	19603.18	32.61929
33.32193	19606.38	32.8593	19604.27	33.85968	19599.51	32.86157	19604.38	32.86596	19604.17	32.86033	19602.62	32.86712
33.57643	19602.97	33.1146	19606.13	34.10816	19603.53	33.10986	19603.03	33.11254	19602.62	33.10957	19604.52	33.11987
33.82492	19604.72	33.36285	19606.09	34.3607	19602.29	33.36095	19604.65	33.36547	19604.29	33.36054	19602.84	33.36882
34.07215	19603.64	33.61231	19605.77	34.60932	19602.33	33.60962	19605.14	33.62407	19604.03	33.60877	19603.77	33.61957

34.32245	19604.63	33.85985	19608.25	34.86102	19602.62	33.86112	19601.75	33.86856	19605.08	33.8593	19602.25	33.86882
34.57346	19604.55	34.11329	19605.82	35.10883	19601.87	34.11149	19604.96	34.11401	19603.77	34.10839	19601.63	34.11936
34.82417	19602.76	34.36067	19605.15	35.36021	19601.01	34.36232	19603.22	34.36668	19603.33	34.36074	19605.86	34.36901
35.0741	19606.9	34.6154	19608.65	35.6086	19601.7	34.60964	19604.63	34.61871	19602.57	34.60995	19602.82	34.61965
35.32289	19606.36	34.861	19605.01	35.86213	19602.05	34.86112	19601.65	34.86606	19603.16	34.86114	19603.46	34.86905
35.57247	19604.5	35.11522	19604.42	36.10993	19601.27	35.11	19602.52	35.11573	19604.18	35.10816	19603.37	35.11985
35.82357	19606.79	35.36119	19606.34	36.36078	19602.78	35.36076	19602.54	35.36765	19601.8	35.35997	19604.34	35.36893
36.07452	19604.2	35.6162	19605.3	36.60901	19602.89	35.61149	19603.39	35.61919	19604.18	35.60964	19604.61	35.62783
36.32625	19604.28	35.86252	19605.79	36.86198	19601.3	35.86172	19602.62	35.86616	19603.83	35.86149	19601.84	35.87017
36.57439	19606.22	36.11626	19604.74	37.11137	19602.27	36.10973	19604.35	36.11426	19604.1	36.11009	19603.19	36.12148
36.8231	19604.48	36.36148	19604.55	37.36265	19601.74	36.36111	19603.95	36.36729	19604.29	36.35901	19601.58	36.36946
37.07357	19602.21	36.61525	19604.74	37.60942	19602.74	36.61119	19602.38	36.61993	19603.37	36.6099	19603.5	36.61903
37.3254	19606.29	36.86274	19605.5	37.86235	19602.87	36.86384	19604.27	36.8672	19603.08	36.86168	19603.64	36.86948
37.57543	19604.68	37.11473	19607.33	38.11045	19601.06	37.11142	19602.4	37.11406	19602.65	37.11073	19604.47	37.12098
37.8244	19605.76	37.3689	19603.29	38.36476	19600.98	37.36112	19604.38	37.3663	19603.47	37.36093	19602.92	37.37051
38.07303	19605.67	37.61448	19605.09	38.61077	19601.15	37.61062	19603.06	37.62036	19603.33	37.60921	19605.12	37.61941
38.3248	19606.98	37.86241	19605.57	38.8617	19603.48	37.86249	19602.08	37.86786	19603.64	37.86144	19604.98	37.8699
38.57471	19607.35	38.11797	19607.13	39.11036	19602.17	38.11156	19603.7	38.11458	19603.98	38.11146	19603.68	38.12304
38.82553	19608.17	38.36368	19605.52	39.36184	19602.67	38.36359	19602.59	38.36661	19603.63	38.36145	19603.46	38.37075
39.07448	19604.22	38.61605	19604.03	39.61051	19602.03	38.61309	19602.79	38.61994	19603.65	38.60989	19603.08	38.62088
39.32366	19602.68	38.86242	19606.91	39.86331	19603.44	38.86285	19603.78	38.86842	19602.7	38.86221	19602.73	38.86883
39.57477	19606.35	39.11721	19606.1	40.11137	19603.8	39.11894	19602.58	39.11636	19602.7	39.11038	19603.99	39.12174
39.8264	19602.42	39.36534	19605.09	40.36377	19603.4	39.36306	19603.24	39.36838	19604.3	39.36192	19603.38	39.37109
40.07546	19608.08	39.61538	19608.66	40.61196	19602.77	39.61355	19604.1	39.6203	19603.82	39.61138	19605.92	39.62149
40.32548	19607.35	39.86287	19604.86	40.86425	19601.75	39.86419	19602.59	39.86813	19603.02	39.86487	19603.51	39.87029
40.57583	19606.38	40.11644	19602.57	41.11362	19602.64	40.11278	19603.99	40.11494	19604.02	40.10993	19604.51	40.12081
40.82725	19605.56	40.36412	19604.57	41.365	19602.26	40.36542	19605.79	40.37004	19603.6	40.3627	19603.64	40.372
41.07828	19606.46	40.6198	19605.05	41.61189	19603.43	40.61355	19602.43	40.62327	19603.04	40.61178	19604.11	40.6224
41.32622	19606.48	40.86456	19607.15	41.86401	19601.2	40.86419	19602.95	40.86884	19602.81	40.86516	19603.14	40.87194
41.5755	19606.03	41.11807	19605.56	42.11396	19603.35	41.1149	19603.09	41.11709	19603.34	41.11409	19603.71	41.12395
41.82778	19605.31	41.36493	19605.45	42.36518	19602.17	41.36478	19603.1	41.36964	19603.76	41.36347	19602.45	41.37068
42.07778	19602.81	41.61935	19601.38	42.61291	19603.37	41.61402	19603.27	41.62249	19604.67	41.61155	19603.96	41.62309
42.32758	19606.09	41.86637	19606.17	42.86545	19603.38	41.86468	19602.24	41.87106	19603.64	41.86463	19600.99	41.87216
42.57641	19606.33	42.11879	19606.24	43.1125	19602.78	42.11733	19602.28	42.11631	19602.92	42.11496	19603.65	42.12343
42.82772	19602.64	42.36433	19607.54	43.36707	19602.62	42.36622	19602.93	42.36959	19603.9	42.36629	19602.93	42.37444
43.07712	19605.71	42.61785	19604.11	43.61388	19601.86	42.61579	19602.55	42.63028	19603.99	42.61194	19604.81	42.62316
43.32827	19603.33	42.86562	19604.98	43.86544	19602.19	42.86411	19603.72	42.87277	19602.25	42.86468	19604.19	42.87328
43.57823	19604.76	43.12071	19606.41	44.11391	19602.34	43.11523	19602.4	43.11902	19602.94	43.11471	19601.56	43.12524
43.82898	19604.71	43.36607	19606.64	44.3649	19603.36	43.36565	19602.61	43.37128	19602.48	43.36479	19604.17	43.37334
44.07676	19605.63	43.61855	19605.2	44.61201	19603.31	43.61554	19603.32	43.62284	19603.55	43.61397	19604.77	43.6236
44.32682	19606.35	43.86438	19604.44	44.86575	19603.22	43.86532	19601.53	43.87111	19601.37	43.86633	19605.32	43.87327
44.57833	19604.6	44.12065	19604.11	45.11403	19603.5	44.11402	19604.16	44.11971	19602.64	44.11497	19604.56	44.12472
44.83037	19604.1	44.36746	19606.21	45.36735	19602.87	44.36476	19602.53	44.37169	19603.2	44.36561	19603.42	44.3738
45.07738	19606.6	44.62079	19606.61	45.61386	19602.36	44.61727	19602.79	44.62546	19604.56	44.61461	19605	44.62451
45.32695	19603.13	44.86593	19605.26	45.86568	19603.08	44.86642	19602.21	44.87039	19604.44	44.8682	19603.55	44.87288
45.57829	19607.97	45.11901	19607.44	46.1144	19600.88	45.1151	19603.49	45.11901	19604.11	45.11564	19603.35	45.12385
45.83019	19604.83	45.36604	19604.82	46.36622	19602.49	45.36552	19603.76	45.37344	19601.57	45.36376	19602.17	45.37376
46.079	19603.31	45.62192	19605.24	46.61639	19602.98	45.61649	19603.57	45.62546	19603.52	45.61376	19605.26	45.62635
46.32842	19607.19	45.86738	19604.7	46.86638	19601.8	45.86764	19603.5	45.87137	19603.01	45.8671	19602.72	45.87461
46.57738	19606.13	46.12009	19603.84	47.11411	19601.43	46.11697	19603.39	46.11808	19604.05	46.11472	19603.59	46.12354
46.82857	19605.6	46.36675	19604.56	47.36568	19602.09	46.36812	19603.13	46.37152	19605.25	46.36407	19602.66	46.37266
47.07867	19604.33	46.62156	19605.29	47.61458	19602.3	46.61592	19602.94	46.62434	19603.36	46.61423	19603.19	46.62561
47.32934	19606.06	46.86832	19605.1	47.86826	19602.96	46.86679	19604.1	46.87264	19603.38	46.86691	19603.26	46.87467
47.57909	19604.26	47.12071	19606.1	48.11624	19601.14	47.11539	19602.79	47.11859	19603.94	47.11608	19604.44	47.12705
47.8287	19604.56	47.37093	19606.55	48.36872	19603.35	47.36746	19603.77	47.37218	19604.6	47.36674	19604.38	47.37318
48.079	19607.91	47.61966	19603.01	48.61462	19602.01	47.61741	19603.67	47.62703	19602.73	47.61397	19603.34	47.6273
48.33073	19607.64	47.867	19604.68	48.86641	19601.45	47.86595	19600.42	47.87251	19604.03	47.86799	19603.49	47.87406
48.5785	19604.75	48.12172	19605.85	49.11557	19602.93	48.11643	19603.38	48.11964	19603.1	48.11542	19603.25	48.12779
48.82761	19603.75	48.36785	19603.85	49.36662	19601.82	48.36778	19603.92	48.37139	19602.51	48.36711	19603.78	48.37616
49.07708	19604.47	48.62015	19606.16	49.61612	19603.62	48.61836	19604.2	48.62386	19602.34	48.61301	19603.37	48.62485
49.32807	19607.53	48.86663	19604.82	49.86597	19602.37	48.86779	19601.98	48.87184	19603.73	48.86533	19603.98	48.87244
49.57981	19605.77	49.12089	19605.12	50.1157	19602.35	49.117	19601.69	49.11927	19603.76	49.11318	19604.81	49.12354
49.82891	19603.82	49.36737	19606.67	50.36712	19603.39	49.36682	19602.04	49.37622	19603.34	49.37518	19603.79	49.37365
50.07762	19606.43	49.62111	19604.32	50.61519	19602.63	49.62093	19603.01	49.63146	19603.84	49.62249	19603.77	49.62858
50.32743	19605.49	49.86701	19605.29	50.86597	19602.88	49.86676	19603.68	49.87346	19603.7	49.87077	19603	49.87249
50.57792	19604.83	50.12013	19606.89	51.11463	19601.62	50.11658	19603.29	50.11969	19603.39	50.11384	19603	50.12279
50.82914	19605.06	50.36582	19604.83	51.37411	19603.32	50.37931	19601.61	50.37172	19602.78	50.36408	19604.6	50.37439
51.07892	19604.06	50.62026	19604.17	51.61518	19603.02	50.61794	19603.41	50.62532	19604.63	50.61444	19604.49	50.6266
51.32814	19607.43	50.86807	19608.1	51.86754	19603.61	50.86934	19603.03	50.87188	19602.95	50.86778	19603.59	50.87521
51.57717	19605.25	51.12208	19605.47	52.11422	19603.58	51.11873	19603.31	51.11872	19603.85	51.11493	19604.38	51.12467

51.82866	19607.08	51.3667	19605.03	52.36618	19601.93	51.36818	19603.43	51.37243	19604.51	51.36662	19602.11	51.37338
52.07975	19604.85	51.62014	19606.6	52.61513	19603.02	51.61781	19602.58	51.62771	19604.25	51.6144	19604.13	51.62611
52.33015	19605.71	51.86715	19605.4	52.86728	19602.38	51.8682	19603.38	51.87497	19604.06	51.86722	19604.15	51.87501
52.57869	19605.11	52.12143	19604.18	53.11778	19604.09	52.11969	19602.34	52.11911	19602.87	52.11614	19603.59	52.12628
52.828	19608.72	52.36781	19605.56	53.37055	19604.42	52.36845	19602.03	52.3719	19604.34	52.36731	19602.93	52.37354
53.07866	19604.17	52.6203	19606.72	53.61413	19603.38	52.61758	19602.37	52.6284	19603.28	52.61367	19603.82	52.62745
53.33102	19605.28	52.86738	19603.4	53.86711	19602.87	52.86719	19602.4	52.87437	19603.59	52.86661	19604.04	52.87507
53.57991	19606.21	53.12317	19603.97	54.11746	19603.56	53.11991	19604.05	53.12049	19603.02	53.11607	19603.64	53.12638
53.82907	19604.99	53.36828	19606.36	54.36811	19603.04	53.36886	19603.22	53.37167	19604.8	53.36739	19603.79	53.37586
54.07935	19605.24	53.62108	19607.13	54.61464	19602.05	53.61916	19603.61	53.62719	19604.82	53.61551	19603.2	53.62636
54.33001	19604.28	53.86696	19602.86	54.8679	19602.69	53.8692	19602.66	53.87518	19604.17	53.86702	19602.46	53.87511
54.58058	19604.87	54.12259	19606.69	55.1175	19602.58	54.11827	19603.47	54.12381	19603.94	54.11567	19605.02	54.12596
54.83042	19603.32	54.36881	19607.45	55.36844	19601.99	54.37009	19602.57	54.37471	19603.62	54.36898	19603.88	54.37621
55.07914	19603.78	54.61957	19603.36	55.6156	19603.25	54.6191	19604.67	54.62821	19604.25	54.61708	19602.83	54.62796
55.32993	19605.13	54.86671	19605.64	55.86742	19603.16	54.86988	19603.65	54.87455	19600.79	54.86735	19602.83	54.87407
55.58051	19604.1	55.12371	19604.15	56.11721	19603.38	55.12072	19602.26	55.12067	19602.44	55.11429	19604.9	55.13059
55.83113	19606.47	55.36946	19603.72	56.37041	19601.75	55.36898	19604.14	55.37526	19602.62	55.36877	19604.17	55.37755
56.07967	19605.73	55.62328	19603.53	56.61723	19600.41	55.61821	19601.37	55.6268	19604.04	55.61869	19602.81	55.62918
56.32971	19605.15	55.86724	19605.83	56.86776	19603.8	55.87031	19604.21	55.87553	19602.58	55.86945	19604.75	55.87529
56.58009	19604.92	56.12141	19604.64	57.11569	19602.07	56.12092	19603.7	56.12073	19604.15	56.11605	19604.04	56.12718
56.83097	19604.29	56.36889	19606.87	57.36878	19602.09	56.36973	19602.15	56.37399	19604.74	56.36808	19602.27	56.37754
57.08087	19605.02	56.6235	19604.7	57.61762	19600.49	56.61811	19602.82	56.6283	19607.25	56.61721	19603.51	56.62972
57.33022	19604.53	56.86911	19605.2	57.87085	19602.79	56.86981	19601.79	56.87693	19603.06	56.86985	19603.57	56.87756
57.58036	19605.76	57.12428	19604.52	58.11776	19603.94	57.12053	19603.24	57.12144	19603.66	57.11765	19602.93	57.12703
57.83034	19606.38	57.36818	19606.34	58.37214	19603.3	57.37015	19602.44	57.37321	19603.4	57.36822	19603.84	57.37716
58.08202	19606.89	57.62413	19606.34	58.61615	19603.85	57.61858	19602.67	57.62869	19601.89	57.61617	19604.66	57.62754
58.33312	19606.55	57.87661	19605.17	58.86966	19602.94	57.86911	19603.49	57.8745	19603.55	57.86954	19602.95	57.87612
58.58084	19603.35	58.12452	19603.73	59.11978	19604.03	58.1229	19604.01	58.12355	19602.96	58.1183	19603.67	58.12916
58.83033	19603.52	58.36873	19604.96	59.37115	19601.82	58.3719	19602.93	58.37543	19601.02	58.36951	19604.14	58.37812
59.08196	19605.65	58.62174	19606.97	59.6158	19602.05	58.61921	19602.16	58.62829	19603.07	58.61712	19602.28	58.62778
59.33313	19603.9	58.86906	19606.1	59.86944	19601.43	58.8697	19601.41	58.87493	19602.53	58.86885	19602.54	58.87666
59.58263	19604.06	59.12606	19605.51	60.1274	19603.64	59.12144	19603.63	59.12305	19604.27	59.11751	19603.16	59.1286
59.83056	19604.09	59.37331	19603.68	60.37225	19603.77	59.37058	19603.19	59.37592	19604.01	59.37182	19604.41	59.37947
60.08081	19603.56	59.62176	19601.95	60.6196	19602.85	59.62022	19604.24	59.6289	19604	59.62065	19602.6	59.62992
60.33505	19605.75	59.87186	19604.76	60.87119	19601.2	59.87133	19603.87	59.87607	19603.4	59.86923	19604.13	59.87731
60.58401	19605.9	60.12442	19606.04	61.11957	19601.38	60.12059	19602.53	60.1229	19603.96	60.11658	19601.94	60.127
60.83367	19606.43	60.37384	19604.05	61.37351	19602.3	60.37263	19602.98	60.37743	19603.59	60.37202	19603.38	60.38035
61.08285	19603.79	60.62448	19605.41	61.62026	19602.1	60.62295	19604.08	60.63171	19604.22	60.6202	19603.13	60.63165
61.33329	19606.33	60.87132	19607.05	61.87212	19603.92	60.87393	19603.37	60.87772	19604.17	60.8729	19603.49	60.88012
61.58284	19606.1	61.12639	19607.23	62.12149	19602.36	61.12365	19602.91	61.12455	19602.34	61.11981	19603.55	61.12993
61.83422	19605.29	61.37401	19605.42	62.37245	19601.86	61.37207	19603.95	61.37712	19603.88	61.37141	19604.19	61.38008
62.08395	19604.29	61.62757	19603.99	62.62115	19603.28	61.62134	19602.05	61.63199	19603.32	61.61961	19602.95	61.63096
62.3437	19604.07	61.87302	19604.15	62.87455	19603.1	61.87428	19603.09	61.8788	19603.38	61.87266	19602.34	61.88015
62.58394	19605.56	62.12693	19605.68	63.12139	19603.76	62.1247	19602.35	62.12589	19604.64	62.12021	19602.72	62.1313
62.83295	19605.1	62.37357	19604.92	63.37517	19603.31	62.37433	19602.56	62.37826	19602.71	62.37201	19604.06	62.38074
63.08536	19607.22	62.62788	19604.27	63.62151	19603.4	62.62199	19603.94	62.63205	19603.57	62.61993	19604.12	62.63215
63.33658	19603.8	62.87445	19603.41	63.87433	19602.32	62.87365	19603.44	62.87942	19604.15	62.87319	19604.79	62.87926
63.58416	19603.88	63.12742	19606.85	64.12306	19603.1	63.12403	19603.04	63.12623	19603.8	63.12196	19604.52	63.13299
63.83458	19605.82	63.37301	19606.42	64.37373	19603.95	63.37567	19603.12	63.37887	19604.4	63.37299	19604.07	63.38176
64.08582	19605.14	63.62943	19606.93	64.6198	19602.03	63.62287	19603.14	63.63269	19604.02	63.61972	19603.43	63.63171
64.33658	19606.09	63.87376	19603.32	64.8725	19604.68	63.87345	19603.86	63.87865	19603.26	63.87399	19602.53	63.8791
64.58303	19605.36	64.12983	19604.83	65.12043	19601.35	64.12736	19603.55	64.12757	19603.61	64.12161	19602.58	64.13174
64.83267	19605.25	64.37563	19606.5	65.37116	19602.98	64.37941	19602.04	64.37902	19602.7	64.37432	19602.9	64.38141
65.0847	19606.19	64.62445	19604.86	65.619	19602.89	64.62653	19602.44	64.63255	19603.47	64.61848	19603.21	64.63222
65.33475	19605.55	64.87132	19608.15	65.87368	19602.61	64.87206	19602.25	64.87652	19602.67	64.87373	19603.05	64.87809
65.58402	19606.09	65.12856	19604.89	66.12189	19603.17	65.1256	19603.89	65.12437	19603.55	65.11972	19604.87	65.13034
65.83286	19608.7	65.3748	19604.9	66.37259	19602.92	65.3756	19604.07	65.37823	19604.76	65.37209	19604.65	65.38097
66.08522	19603.54	65.62608	19604.8	66.61817	19600.49	65.63822	19602.14	65.63263	19604.21	65.61884	19603.77	65.63309
66.33655	19605.51	65.87174	19605.27	66.87177	19603.2	65.87267	19603.2	65.87876	19602.82	65.87405	19601.6	65.879
66.58513	19607.23	66.12774	19604.13	67.12244	19602.11	66.12421	19602.53	66.12384	19603.42	66.12162	19603.6	66.13082
66.8354	19604.39	66.37561	19605.6	67.37387	19601.6	66.37576	19602.71	66.37752	19601.78	66.37339	19603.56	66.38041
67.08301	19604.96	66.62852	19604.83	67.61975	19602.37	66.62304	19603.48	66.63393	19604.45	66.6196	19604.31	66.63474
67.33406	19605.71	66.87362	19606.27	67.87152	19601.78	66.87332	19603.76	66.87987	19601.25	66.87183	19603.63	66.8803
67.58504	19605.95	67.12647	19607.07	68.12067	19603.93	67.1254	19604.71	67.12656	19601.9	67.12018	19602.42	67.13009
67.83609	19602.12	67.40284	19604.18	68.37557	19603.32	67.37411	19603.61	67.37755	19604.22	67.37448	19604.83	67.38028
68.08559	19605.43	67.62687	19605.59	68.62094	19603.55	67.62454	19602.82	67.63306	19604.18	67.62149	19603.79	67.63727
68.33558	19605.09	67.87302	19604.24	68.87314	19603.62	67.87496	19601.53	67.87916	19604.52	67.87166	19603.11	67.88277
68.58886	19604.7	68.12813	19606.54	69.12169	19602.6	68.12734	19601.52	68.12672	19603.02	68.12128	19603.31	68.13316
68.83537	19605.54	68.37309	19605.71	69.37287	19601.99	68.37437	19602.77	68.37994	19602.98	68.37588	19603.39	68.37971
69.08679	19604.18	68.62542	19605.54	69.62105	19602.79	68.6226	19602.88	68.63245	19603.49	68.62288	19600.92	68.63491

69.33425	19603.52	68.87277	19602.98	69.87468	19601.26	68.87565	19602.22	68.87842	19603.9	68.87432	19603.92	68.88185
69.58457	19604.61	69.12991	19607.54	70.12272	19602.13	69.12765	19602.62	69.1282	19604.62	69.12111	19601.86	69.13249
69.83583	19606.24	69.37665	19604.31	70.37355	19602.35	69.37487	19601.9	69.37943	19605.28	69.37389	19603.79	69.37969
70.08715	19605.37	69.62754	19605.92	70.61967	19603.3	69.62301	19602.94	69.63611	19603.6	69.6228	19603.88	69.63405
70.3344	19605.41	69.872	19605.84	70.87409	19602.86	69.87493	19603.33	69.87888	19604.81	69.87484	19603.41	69.88097
70.58513	19603.96	70.1273	19603.92	71.12339	19602.62	70.14841	19602.45	70.12581	19605.82	70.12126	19604.06	70.13183
70.83517	19604.25	70.37565	19604.25	71.37543	19601.83	70.37577	19604.2	70.37968	19605.24	70.37246	19603.14	70.38184
71.08808	19605.25	70.62742	19605.99	71.62207	19602.78	70.62548	19602.59	70.63495	19603.48	70.62142	19603.22	70.63458
71.3364	19605.96	70.87282	19603.2	71.87354	19603.59	70.87543	19602.93	70.88015	19602.67	70.87572	19603.28	70.88172
71.58562	19604.32	71.12823	19605.62	72.12124	19600.73	71.12739	19602.64	71.12642	19604.09	71.12221	19604.66	71.13235
71.83432	19604.22	71.37537	19605.98	72.37388	19603.17	71.37557	19602.25	71.3793	19603.55	71.37391	19604.47	71.38248
72.08591	19606.28	71.62985	19605.41	72.62298	19602.72	71.62541	19600.29	71.63471	19603.84	71.61997	19604.32	71.6367
72.33773	19606.43	71.87489	19607.82	72.87628	19601.68	71.8779	19601.57	71.88183	19604.57	71.87414	19604.39	71.88152
72.58684	19603.48	72.13002	19606.44	73.12208	19602.28	72.12517	19602.74	72.12883	19603.32	72.12432	19605.21	72.1329
72.83519	19603.61	72.37446	19606.3	73.37728	19604	72.37616	19603.4	72.37914	19602.3	72.3744	19602.89	72.38262
73.08606	19606.11	72.63046	19608.4	73.62383	19601.62	72.62539	19602.6	72.63448	19604.21	72.62115	19604.7	72.63587
73.33809	19605.87	72.87535	19603.09	73.87682	19600.66	72.87679	19604.04	72.88119	19602.62	72.8741	19602.39	72.88136
73.58765	19607.27	73.13167	19603.8	74.12466	19602.88	73.12753	19602.36	73.13003	19603.89	73.12272	19603.81	73.13327
73.83643	19605.72	73.37799	19604.9	74.37419	19602.12	73.37652	19603.14	73.38119	19603	73.37394	19604.65	73.38262
74.0857	19605.79	73.63015	19606.91	74.62358	19601.74	73.62602	19604.17	73.63383	19603.81	73.62342	19602.25	73.63682
74.33712	19605.63	73.87583	19607.82	74.87695	19601.68	73.87726	19603.5	73.88077	19603.34	73.87523	19603.3	73.88281
74.58743	19604.65	74.13048	19608.14	75.12524	19600.08	74.12783	19602.65	74.12977	19603.57	74.12186	19603.58	74.13192
74.83763	19606.49	74.37643	19607.8	75.37506	19601.6	74.37651	19601.94	74.38139	19603.78	74.37523	19604.09	74.38127
75.087	19604.47	74.62923	19607.65	75.62273	19602.95	74.62533	19602.39	74.63701	19603.48	74.62489	19603.03	74.63621
75.33735	19604.57	74.87556	19606.94	75.8774	19601.36	74.87923	19603.45	74.88104	19604.18	74.87622	19602.4	74.88326
75.58728	19607.2	75.13143	19606.08	76.12539	19601.08	75.12935	19602.53	75.12905	19602.4	75.1235	19602.8	75.13538
75.83856	19604.89	75.37814	19607.88	76.37695	19600.77	75.37667	19602.96	75.38092	19602.74	75.37419	19604.43	75.38216
76.088	19604.06	75.63003	19603.63	76.62291	19602.42	75.62715	19605.03	75.63727	19601.81	75.62453	19605.01	75.63676
76.3375	19605.84	75.87591	19602.49	76.87533	19601.7	75.87718	19601.9	75.88151	19603.9	75.87637	19604.47	75.88271
76.58696	19604.71	76.13123	19602.2	77.12516	19601.5	76.12892	19602	76.12935	19603.83	76.12377	19602.83	76.13615
76.83729	19605.05	76.381	19607.98	77.37722	19600.33	76.37833	19603.92	76.38138	19601.39	76.37463	19604.56	76.3847
77.08845	19604.06	76.62741	19605.52	77.62444	19602.3	76.62754	19601.06	76.63701	19602.08	76.62231	19603.46	76.64515
77.33878	19602.85	76.87541	19604.34	77.87654	19599.62	76.87665	19603.65	76.88454	19601.92	76.87674	19604.92	76.88375
77.58701	19603.76	77.13105	19605	78.12528	19600.89	77.13021	19603.32	77.12822	19605.65	77.1245	19603.08	77.13477
77.83712	19605.38	77.37905	19605.96	78.38003	19601.62	77.37862	19602.2	77.38138	19604.87	77.37621	19604.24	77.38402
78.08898	19607.86	77.62898	19603.32	78.62543	19600.09	77.62864	19603.38	77.63643	19603.44	77.62242	19604.19	77.64062
78.34091	19604.82	77.87545	19604.91	78.87804	19601.06	77.87812	19603.51	77.88196	19603.39	77.87639	19603.48	77.88209
78.58883	19605.01	78.13256	19604.39	79.12377	19600.95	78.12859	19603.12	78.12939	19604.05	78.12443	19604.02	78.13848
78.83751	19603.99	78.37978	19603.3	79.37636	19601.42	78.37968	19602.16	78.38161	19602.83	78.37691	19603.41	78.38383
79.08784	19604.54	78.63103	19605	79.62467	19601.67	78.62821	19603.41	78.63886	19603.78	78.62505	19603.3	78.63983
79.33924	19607.28	78.87817	19607.77	79.87771	19601.59	78.87904	19603.04	78.88352	19603.17	78.87764	19602.65	78.88429
79.59037	19606.32	79.13259	19606.17	80.12784	19600.94	79.13384	19602.79	79.13102	19602.55	79.12313	19602.65	79.13678
79.83899	19604.65	79.38222	19603.64	80.37775	19600.36	79.38084	19602.62	79.38492	19603.44	79.37731	19601.98	79.38403
80.08759	19603.86	79.63183	19604.38	80.62484	19601.2	79.62662	19601.92	79.6371	19603.94	79.62613	19602.54	79.64037
80.34005	19604.93	79.87817	19606.94	80.87785	19598.81	79.87884	19602.33	79.88353	19603.42	79.8789	19604.24	79.88651
80.59024	19604.62	80.13272	19604.67	81.12746	19601.19	80.13121	19602.84	80.13078	19604.29	80.12455	19602.94	80.13777
80.84012	19603.96	80.38191	19605.25	81.37707	19601.15	80.38273	19602.02	80.38642	19602.42	80.37695	19603.83	80.38814
81.0885	19604.74	80.63025	19605.14	81.62435	19600.14	80.62751	19601.96	80.63791	19601.95	80.62645	19603.33	80.64041
81.3451	19603.39	80.87705	19607.22	81.87904	19601.8	80.87843	19602.63	80.88275	19603.21	80.87967	19603.06	80.88534
81.58936	19603.22	81.13374	19604.89	82.12676	19600.47	81.13049	19601.86	81.12994	19603.53	81.12726	19604.38	81.13855
81.8405	19603.56	81.3821	19605.75	82.37904	19601.36	81.38095	19602.73	81.3834	19603.68	81.37934	19603.75	81.38708
82.09039	19606.14	81.63241	19604.94	82.62551	19602.42	81.62923	19602.23	81.63936	19604.1	81.62543	19604.21	81.64047
82.33857	19604.49	81.8761	19608.29	82.87772	19601.58	81.88611	19601.44	81.88714	19603.27	81.87778	19604.28	81.88502
82.58961	19606.26	82.13367	19607.12	83.12821	19600.73	82.1325	19601.14	82.13089	19602.05	82.1265	19603.96	82.13717
82.84018	19606.94	82.38123	19605.85	83.38284	19600.91	82.3806	19603.16	82.3828	19604.2	82.37995	19603.15	82.38717
83.09051	19608.74	82.63307	19606.02	83.62693	19598.58	82.62928	19602.66	82.64053	19603.71	82.62669	19602.69	82.64398
83.34036	19600.94	82.87797	19605.01	83.8769	19600.74	82.87988	19602.56	82.88519	19603.85	82.87863	19603.22	82.88628
83.58935	19607.12	83.13192	19604.22	84.12654	19600.41	83.13296	19602.54	83.13167	19602.47	83.12598	19602.1	83.13891
83.84051	19605.18	83.38255	19605.63	84.37958	19600.57	83.38009	19603.39	83.38464	19603.54	83.37772	19601.5	83.38604
84.09112	19603.94	83.63275	19603.74	84.62892	19601.46	83.6291	19603.93	83.63876	19603.63	83.6272	19603.18	83.642
84.34343	19606.99	83.87874	19603.41	84.88078	19600.93	83.87998	19602.79	83.88522	19603.2	83.87932	19601.81	83.88727
84.59007	19601.7	84.13589	19605.38	85.12844	19600.42	84.13475	19602.7	84.13232	19601.56	84.1251	19602.53	84.13895
84.83973	19604.45	84.38071	19604.74	85.37958	19601.46	84.38053	19601.09	84.38541	19602.76	84.37804	19604.2	84.38542
85.08989	19604.75	84.63192	19603.09	85.62874	19601.75	84.62903	19601.5	84.64105	19603.03	84.62865	19603.71	84.64113
85.34108	19604.37	84.87911	19605.45	85.88036	19601.03	84.88168	19602.41	84.88482	19602.3	84.87951	19602.41	84.88698
85.5913	19604.62	85.13819	19605.37	86.12974	19600.91	85.1343	19601.47	85.13059	19603.99	85.12701	19603.27	85.13883
85.84048	19606.02	85.38726	19605.24	86.37977	19601.36	85.38148	19601.66	85.38495	19602.38	85.37817	19605.23	85.38772
86.08955	19603.97	85.6329	19604.31	86.62641	19599.25	85.62849	19602.75	85.63968	19604.11	85.62886	19602.28	85.64159
86.34087	19605.61	85.87904	19605.3	86.88126	19599.37	85.88101	19602.41	85.8857	19602.53	85.87915	19603.23	85.88654
86.5923	19604.19	86.13755	19606.33	87.12984	19599.84	86.14191	19601.6	86.13057	19602.83	86.12941	19602.54	86.13842

86.84269	19605.34	86.38503	19606.72	87.38134	19600.49	86.38631	19602.31	86.38549	19601.86	86.37717	19603.18	86.38886
87.08972	19603.99	86.63396	19605.18	87.62809	19600.33	86.62986	19600.46	86.64065	19601.65	86.62812	19602.9	86.64284
87.34312	19605.35	86.87916	19603.19	87.87866	19600.4	86.8799	19601.27	86.88761	19603.31	86.88033	19602.76	86.88612
87.59232	19607.21	87.13817	19608.26	88.1294	19599.47	87.13548	19600.73	87.13294	19601.01	87.12955	19602	87.13866
87.84357	19605.41	87.40363	19605.18	88.38432	19598.81	87.38271	19602.44	87.38529	19600.75	87.37977	19602.03	87.38807
88.09194	19602.5	87.63843	19603.06	88.63035	19599.22	87.62993	19601.56	87.6404	19601.36	87.62847	19603.14	87.64578
88.34307	19604.86	87.8852	19605.22	88.87917	19600.03	87.87982	19601.59	87.88668	19602.62	87.8806	19601.87	87.88846
88.5921	19605.63	88.13715	19606	89.12944	19598.5	88.13555	19601.39	88.13346	19602.22	88.12856	19603.08	88.1402
88.84333	19602.82	88.38437	19607.09	89.37978	19599.06	88.38485	19600.75	88.38667	19603.32	88.38152	19600.44	88.38665
89.09369	19605.63	88.63582	19606.4	89.62833	19598.29	88.6318	19602.27	88.64016	19603.44	88.6308	19602.58	88.64276
89.34332	19601.88	88.88103	19603.4	89.88076	19598.51	88.88144	19600.91	88.88786	19601.44	88.88097	19602.86	88.88889
89.59183	19604.53	89.13642	19607.32	90.12967	19598.65	89.13646	19600.65	89.13507	19603.83	89.12801	19602.4	89.14145
89.84282	19603.99	89.38257	19606.95	90.38182	19600.23	89.38347	19601.25	89.38768	19604.06	89.38044	19603.5	89.38785
90.09323	19605.6	89.63456	19605.49	90.62827	19599.82	89.63288	19601.39	89.64195	19604.47	89.6301	19602.17	89.64433
90.3443	19605.87	89.88093	19603.47	90.88135	19599.35	89.88574	19600.95	89.88763	19603.84	89.88064	19603.32	89.89029
90.59162	19603.52	90.13707	19604.95	91.13266	19599.66	90.13667	19599.98	90.13425	19603.11	90.1279	19601.72	90.13949
90.84209	19605.24	90.38522	19603.98	91.38221	19599.48	90.38251	19600.57	90.38883	19602.13	90.37997	19602.5	90.38772
91.09495	19606.26	90.63571	19605.84	91.62964	19598.96	90.63301	19602.52	90.64245	19601.31	90.63086	19601.51	90.6463
91.345	19606.58	90.8824	19604.18	91.88202	19599.03	90.88476	19600.36	90.88863	19601.37	90.88149	19600.11	90.8888
91.59311	19607.56	91.13722	19602.74	92.13291	19598.82	91.13757	19600.92	91.13269	19601.58	91.12949	19602.06	91.1413
91.84192	19604.15	91.38798	19605.21	92.38172	19600.33	91.38383	19600.95	91.38868	19602.73	91.38122	19600.81	91.39064
92.09228	19604.84	91.63447	19604.9	92.62838	19598.73	91.63227	19601.83	91.64157	19600.97	91.62952	19601.17	91.64675
92.34473	19605.88	91.88103	19606.95	92.88052	19598.93	91.88359	19601.31	91.8904	19602.15	91.88326	19601.85	91.88941
92.5942	19601.64	92.14107	19605.25	93.13239	19599.95	92.1376	19600.7	92.14017	19602.23	92.13008	19600.75	92.14359
92.84441	19604.28	92.38781	19602.98	93.38498	19599.91	92.38304	19600.51	92.38914	19602.73	92.381	19602.22	92.39106
93.09278	19602.5	92.63635	19605.22	93.62984	19597.96	92.6312	19599.67	92.64299	19602.2	92.62944	19600.71	92.64806
93.34309	19602.74	92.88018	19604.35	93.88112	19598.4	92.88369	19600.6	92.88851	19602.2	92.88392	19602.5	92.89257
93.59281	19603.11	93.13928	19606.19	94.13199	19598.13	93.13795	19601.81	93.13579	19601	93.13104	19601.55	93.14406
93.84413	19603.71	93.38715	19604.79	94.3831	19597.7	93.3852	19599.89	93.39051	19602.28	93.38198	19601.87	93.39107
94.09563	19602.46	93.63743	19604.98	94.63156	19597.65	93.63374	19601.81	93.64263	19601.63	93.63042	19600.65	93.64615
94.34378	19605.66	93.88219	19604.11	94.88309	19597.39	93.88402	19600.08	93.88847	19602.57	93.8816	19601.22	93.89025
94.59223	19604.11	94.13801	19603.6	95.13108	19598.22	94.13717	19602.42	94.13603	19601.77	94.12963	19602.61	94.14063
94.8433	19602.89	94.38705	19603.74	95.3822	19600.31	94.38536	19600.19	94.39093	19601.39	94.39479	19602.75	94.39091
95.09475	19603.28	94.63895	19605.32	95.6316	19598.91	94.63358	19599.91	94.6428	19599.79	94.63187	19600.99	94.64735
95.34633	19603.44	94.88339	19600.78	95.88422	19599.38	94.88586	19602.28	94.88905	19601.56	94.88413	19601.28	94.89131
95.59312	19604.7	95.14123	19605.23	96.13382	19598.55	95.13711	19598.78	95.13617	19601.83	95.12966	19600.58	95.14241
95.84325	19605.24	95.38719	19605.43	96.38327	19598.41	95.38522	19601.82	95.39127	19601.28	95.38242	19601.74	95.39005
96.09385	19604.96	95.64042	19602.63	96.6314	19599.62	95.63508	19600.06	95.64228	19601.36	95.63253	19600.97	95.65514
96.34487	19601.5	95.88416	19602.77	96.88344	19598.37	95.885	19600.65	95.88895	19600.68	95.88373	19600.85	95.89507
96.59603	19605.09	96.13957	19604.49	97.13322	19596.97	96.13824	19598.95	96.13555	19600.11	96.13031	19601.14	96.14414
96.84342	19602.94	96.3888	19602.41	97.38455	19599.38	96.38466	19600.46	96.39095	19600.71	96.38295	19602.05	96.39013
97.09399	19604.24	96.63809	19604.12	97.62929	19598.69	96.63403	19600	96.6432	19600.98	96.6317	19600.04	96.64548
97.34621	19603.71	96.8848	19606.51	97.88325	19597.99	96.88508	19599.54	96.89099	19602.03	96.88423	19598.78	96.89267
97.59664	19608.18	97.13974	19603.27	98.13422	19598.71	97.14004	19599.06	97.13574	19600.61	97.13162	19601.15	97.1443
97.84638	19601.01	97.38862	19603.6	98.38748	19598.79	97.38506	19601.78	97.3913	19601.75	97.38188	19601.77	97.39257
98.09355	19603.56	97.63791	19604.33	98.63267	19597.88	97.63348	19600.51	97.6426	19600.91	97.62993	19600.75	97.64728
98.34451	19605.73	97.88401	19604.68	98.88308	19597.91	97.88655	19600.25	97.88997	19599.99	97.8842	19600.29	97.89217
98.59625	19601.97	98.14224	19602.96	99.13693	19597.46	98.1387	19599.78	98.13664	19599.95	98.13258	19601.84	98.14668
98.84684	19604.91	98.38968	19602.18	99.38541	19599.65	98.38701	19598.93	98.39156	19599.96	98.38301	19599.37	98.39246
99.09571	19603.49	98.63923	19605.28	99.63286	19598.54	98.63481	19601.42	98.64309	19601.22	98.63028	19599.06	98.6502
99.34581	19601.55	98.8839	19603.29	99.88477	19594.67	98.88564	19598.19	98.89149	19600.22	98.88566	19600.96	98.8932
99.59608	19602.65	99.14265	19603.99	100.1333	19597.06	99.14395	19600	99.14051	19602.41	99.1382	19599.86	99.1464
99.84709	19604.13	99.3909	19601.44	100.3865	19597.74	99.38691	19598.2	99.39136	19600.09	99.385	19599.88	99.39161
100.0968	19603.73	99.63984	19603.62	100.635	19597.13	99.63775	19598.78	99.64838	19598.97	99.63063	19600.99	99.65622
100.3504	19600.77	99.88197	19600.23	100.8881	19596.77	99.88638	19599.57	99.89465	19598.48	99.88599	19601.87	99.89925
100.5967	19603.92	100.144	19606.06	101.136	19596.68	100.1395	19598.96	100.1377	19600.31	100.1333	19599.79	100.1454
100.8514	19600.79	100.3919	19605.08	101.3868	19597.25	100.3909	19598.86	100.3931	19601.45	100.3875	19599.07	100.3933
101.0992	19603.74	100.6419	19603.59	101.6348	19598.09	100.6384	19599.82	100.6481	19601.61	100.6341	19599.39	100.6521
101.3492	19602.44	100.8857	19600.98	101.8881	19597.57	100.8889	19598.8	100.8944	19598.89	100.8862	19600.18	100.8986
101.5978	19604.47	101.1431	19602.52	102.1365	19597.5	101.1414	19597.72	101.1418	19599.64	101.1346	19601.59	101.1487
101.8478	19604.17	101.3921	19604.28	102.3985	19597.62	101.3899	19601.94	101.393	19596.87	101.3855	19599.19	101.395
102.0986	19602.1	101.6421	19605.49	102.6345	19598.5	101.6383	19599.83	101.6477	19598.47	101.6362	19597.77	101.6554
102.3502	19601.9	101.8878	19601.49	102.8869	19596.02	101.89	19599.67	101.8961	19599.1	101.8871	19598.04	101.897
102.6009	19602.14	102.1431	19603.56	103.1364	19595.15	102.1427	19598.47	102.1416	19599.66	102.134	19600.62	102.147
102.8491	19602.17	102.3909	19604.01	103.3901	19594.96	102.3887	19596.52	102.3935	19598.11	102.3883	19598.62	102.3998
103.0978	19602.62	102.6433	19603.95	103.6357	19597.03	102.6395	19597.97	102.6505	19599.19	102.6363	19598.94	102.6549
103.3494	19603.22	102.888	19604.64	103.8878	19596.38	102.8899	19599.5	102.8949	19598.98	102.889	19597.07	102.8973
103.6	19603.5	103.1426	19606.1	104.1354	19599.15	103.1432	19599.2	103.1417	19598.65	103.1344	19598.73	103.1467
103.8509	19601.96	103.392	19601.84	104.3879	19596.01	103.3929	19599.88	103.3954	19598.79	103.3875	19601.23	103.3957
104.0988	19601.88	103.6423	19603.8	104.636	19597.23	103.6394	19598.82	103.6488	19599.44	103.6366	19600.97	103.653

104.3483	19603.42	103.8876	19603.14	104.889	19597.76	103.891	19599.03	103.8941	19599.56	103.89	19600.06	103.899
104.6004	19601.05	104.1443	19603.66	105.1364	19595.14	104.1415	19598.04	104.1411	19600.06	104.1384	19599.28	104.1484
104.851	19602.37	104.3934	19602.92	105.3889	19595.83	104.3905	19597.79	104.3943	19600.23	104.387	19596.61	104.3951
105.1007	19602.49	104.6431	19605.1	105.6379	19596.1	104.6417	19597.72	104.6489	19596.77	104.6362	19599.23	104.6551
105.3498	19603.41	104.8889	19602.3	105.8901	19595.46	104.891	19599.53	104.8958	19598.66	104.8913	19599.45	104.8987
105.6001	19598.3	105.1456	19603.78	106.1374	19594.08	105.1432	19597.33	105.1413	19599.37	105.1388	19598.66	105.152
105.8497	19599.84	105.3955	19603.3	106.3882	19595.21	105.3915	19598.84	105.3967	19598.11	105.3917	19599.14	105.3993
106.1008	19603.86	105.645	19601.08	106.6362	19595.64	105.6392	19597.94	105.6492	19599.17	105.636	19599.92	105.657
106.3511	19604.08	105.8875	19601.93	106.8902	19596.34	105.8908	19597.54	105.8971	19598.65	105.8892	19598.32	105.897
106.6001	19601.7	106.1455	19602.32	107.1388	19595.74	106.1435	19599.91	106.1414	19598.73	106.1388	19599.16	106.1502
106.8504	19603.01	106.3996	19600.85	107.4044	19595.05	106.3915	19598.16	106.3964	19598.97	106.3894	19599.01	106.4006
107.1006	19602.8	106.6435	19602.08	107.6359	19596.41	106.6397	19598.16	106.6514	19599.01	106.6374	19598.04	106.6549
107.3522	19603.37	106.8902	19600.28	107.8884	19597.34	106.8918	19598.7	106.8991	19596.5	106.8905	19598.84	106.8984
107.6033	19603.41	107.1457	19602.01	108.1393	19593.73	107.1452	19598.33	107.1442	19599.46	107.1386	19597.36	107.1533
107.8505	19601.82	107.4246	19603.74	108.3932	19597.14	107.3928	19596.96	107.3948	19597.97	107.3901	19597.81	107.3976
108.1011	19601.85	107.6485	19602.14	108.638	19595.94	107.6403	19598.28	107.6513	19598.29	107.6377	19598.65	107.6565
108.3521	19603.94	107.8899	19602.45	108.8889	19594.04	107.8916	19598.54	107.8979	19598.58	107.8926	19598.61	107.9003
108.6041	19598.25	108.1451	19601.79	109.1382	19594.62	108.1443	19597	108.1437	19598.5	108.1369	19598.72	108.1517
108.8505	19602.58	108.3963	19601.99	109.3904	19594.41	108.3928	19598.39	108.3979	19598.47	108.3892	19598.46	108.3971
109.1008	19603.76	108.6483	19602	109.6393	19596.76	108.6419	19597.49	108.6519	19596.7	108.6361	19597.46	108.6574
109.3528	19599.25	108.8913	19602.52	109.8899	19595	108.8919	19597.53	108.8971	19598.49	108.8926	19597.58	108.8989
109.6035	19599.23	109.1487	19600.44	110.1379	19595.98	109.1456	19597.56	109.1432	19599.26	109.1381	19598.15	109.1532
109.8534	19602.42	109.401	19601.78	110.3923	19594.04	109.3941	19598.04	109.3971	19597.64	109.3908	19598.03	109.3977
110.1009	19601.87	109.6436	19601.82	110.6387	19594.87	109.6423	19598.71	109.6629	19597.23	109.6368	19596.92	109.6576
110.3516	19600.32	109.8903	19599.84	110.892	19596.12	109.8934	19598.22	109.9025	19597.97	109.9071	19597.4	109.8988
110.6047	19603.06	110.1476	19601.44	111.1396	19594.42	110.1459	19597.5	110.1434	19598.01	110.1409	19598.55	110.1523
110.8541	19599.86	110.3983	19602	111.3919	19593.6	110.3944	19597.24	110.397	19595.83	110.3899	19597.66	110.3989
111.1017	19598.93	110.6476	19601.39	111.64	19596.28	110.6427	19596.3	110.6528	19595.48	110.6394	19598.57	110.6565
111.3513	19602.74	110.8915	19599.43	111.8927	19594.43	110.8948	19598.84	110.8994	19597.59	110.8923	19597.26	110.8996
111.6039	19600.06	111.1451	19600.48	112.1403	19594.29	111.1463	19596.17	111.1435	19597.86	111.1382	19596.75	111.1529
111.8541	19602.57	111.3999	19602.93	112.392	19597.17	111.3944	19595.68	111.3979	19597.8	111.4067	19598.44	111.3992
112.1032	19602.42	111.6468	19601.97	112.6376	19594.92	111.6417	19596.21	111.6536	19596.78	111.6393	19596.83	111.6579
112.3539	19598.44	111.8922	19599.26	112.8927	19594.39	111.8936	19594.84	111.9014	19596.96	111.8927	19597.04	111.8993
112.6034	19598.78	112.148	19601.77	113.1399	19592.94	112.1477	19596.78	112.1454	19596.17	112.1405	19595.85	112.1548
112.8542	19601.23	112.398	19602.02	113.3966	19593.23	112.396	19595.54	112.3992	19598.29	112.391	19598	112.4007
113.1038	19599.64	112.6483	19602.07	113.639	19595.22	112.6432	19595.47	112.6565	19599.12	112.6405	19596.44	112.6601
113.3537	19600.88	112.8924	19600.83	113.8929	19596.1	112.8931	19594.92	112.8992	19596.33	112.8912	19597.65	112.8983
113.6057	19599.7	113.1476	19600.83	114.1399	19594.52	113.148	19595.76	113.145	19597.99	113.1389	19596.38	113.1534
113.8542	19602.52	113.3992	19600.51	114.3952	19595.04	113.3956	19597.17	113.3994	19597.36	113.3916	19596.52	113.401
114.1047	19602.25	113.6468	19601.72	114.6398	19594.8	113.6445	19595.13	113.6545	19595.64	113.6411	19599.57	113.6599
114.3548	19601.63	113.8928	19600.8	114.8916	19593.32	113.8951	19596.43	113.8997	19597.71	113.894	19597.33	113.8996
114.6044	19599.45	114.1501	19601.68	115.1402	19592.76	114.1476	19596.76	114.1441	19596.73	114.1385	19597.41	114.1516
114.8559	19600.65	114.4004	19599.39	115.3949	19593.87	114.397	19594.9	114.3986	19596.49	114.3936	19599.58	114.3992
115.1033	19600.42	114.6471	19599.77	115.6421	19592.86	114.6439	19595.09	114.655	19596.66	114.6407	19596.02	114.66
115.3565	19601.67	114.8924	19601.95	115.8934	19595.24	114.8994	19597.17	114.9006	19597.47	114.8937	19596.41	114.9022
115.6086	19600.87	115.1507	19599.87	116.1409	19593.33	115.1492	19594.68	115.1453	19596.56	115.1412	19597.62	115.1554
115.8544	19599.72	115.3998	19598.03	116.3949	19594.04	115.3962	19596.9	115.3995	19596.5	115.3922	19596.22	115.3989
116.1034	19598.44	115.6482	19599.91	116.6418	19595.48	115.6434	19595.08	115.6559	19595.61	115.6412	19597.45	115.6603
116.3555	19599.57	115.8917	19601.02	116.8945	19594.45	115.8968	19596.51	115.9014	19595.66	115.8955	19595.91	115.9005
116.6054	19600.53	116.149	19600.93	117.1411	19592.82	116.15	19596.41	116.1457	19596.73	116.142	19595.52	116.1546
116.8552	19600.2	116.4001	19599.29	117.3938	19593.48	116.3968	19598.12	116.3989	19596.27	116.3936	19596.91	116.4028
117.1046	19596.33	116.6489	19598.6	117.6405	19593.9	116.6431	19595.37	116.6556	19596.28	116.6406	19596.85	116.6603
117.3561	19598.26	116.8941	19600.13	117.8953	19594.4	116.8972	19595.28	116.9032	19594.87	116.8951	19597.18	116.9016
117.6034	19599.96	117.1521	19600.51	118.1431	19593.5	117.1487	19596.45	117.1472	19597.2	117.1413	19597.03	117.1556
117.8553	19599.8	117.3975	19600.52	118.3983	19593.65	117.396	19595.19	117.4019	19596.11	117.3934	19594.08	117.4023
118.1057	19598.53	117.6507	19601.23	118.6403	19593.32	117.6433	19597.61	117.6558	19595.41	117.642	19596.24	117.6604
118.3586	19599.27	117.8981	19600.43	118.8954	19594.73	117.8957	19595.78	117.9015	19594.54	117.8931	19595.61	117.9015
118.6038	19599.74	118.1631	19600.02	119.1447	19593.73	118.1496	19595.4	118.1481	19595.3	118.1416	19598.2	118.1582
118.8567	19599.16	118.401	19595.4	119.3975	19593.85	118.397	19596.71	118.4021	19594.42	118.3932	19597.26	118.4026
119.1057	19599.94	118.648	19599.98	119.6428	19593.6	118.6446	19596.23	118.6554	19595.85	118.6419	19595.89	118.6615
119.3577	19600.1	118.8942	19600.62	119.8938	19594.35	118.8976	19598.08	118.9013	19595.44	118.8975	19595.71	118.903
119.6075	19599.87	119.1526	19602.61	120.1437	19592.62	119.1516	19595.32	119.1474	19594.71	119.1415	19596.29	119.1566
119.8576	19599.89	119.4015	19601.35	120.4065	19592.58	119.3977	19594.79	119.4014	19595.7	119.3945	19595.69	119.4029
120.1092	19600.52	119.6562	19600.01	120.6419	19592.54	119.6461	19594.44	119.6555	19594.77	119.6432	19595.36	119.6604
120.3566	19600.43	119.8954	19598.68	120.8936	19594.07	119.8994	19596.79	119.903	19595.68	119.8977	19595.7	119.9031
120.6064	19596.72	120.1501	19598.84	121.1423	19592.92	120.1496	19595.53	120.1471	19595.53	120.1427	19596.58	120.1567
120.8546	19600.53	120.4	19600.85	121.3928	19593.23	120.3978	19593.02	120.4028	19594.89	120.3955	19595.24	120.4025
121.1055	19598.3	120.6457	19601.37	121.6415	19595.13	120.6444	19595.65	120.6538	19596.08	120.6396	19596.41	120.6596
121.3543	19600.93	120.8912	19600.33	121.8948	19594.73	120.8964	19595.36	120.9002	19596.76	120.8926	19595.41	120.901
121.6045	19600.41	121.1498	19602.3	122.1406	19593.2	121.1478	19594.57	121.1449	19595.73	121.1407	19593.68	121.1555

121.8542	19600.83	121.402	19601.29	122.3923	19590.45	121.3944	19594.75	121.3991	19595.83	121.3933	19594.05	121.401
122.1038	19597.95	121.6474	19601.62	122.6425	19593.89	121.6429	19596	121.6536	19594.79	121.6508	19596.76	121.6602
122.3592	19600.05	121.8921	19598.54	122.8946	19593.64	121.8966	19595.07	121.9031	19595.75	121.8945	19596.64	121.9006
122.6069	19600.2	122.1499	19599.61	123.144	19592.73	122.1484	19592.06	122.1466	19595.12	122.1419	19597.03	122.1558
122.8562	19599.81	122.3983	19600.77	123.3974	19594.9	122.3955	19594.36	122.4018	19596.48	122.3953	19596.05	122.4019
123.1053	19598.57	122.6472	19601.32	123.6414	19592.34	122.6435	19595.01	122.654	19596.16	122.6399	19596.29	122.6589
123.3667	19601.54	122.8923	19598.38	123.8949	19592.86	122.8951	19593.49	122.9001	19596.35	122.8931	19597.2	122.9006
123.6069	19599.66	123.1508	19597.67	124.1437	19593.58	123.1491	19597.83	123.1465	19596.02	123.1409	19595.72	123.1576
123.8552	19599.97	123.3986	19600.1	124.4017	19591.58	123.3956	19593.57	123.4008	19595.98	123.3936	19594.69	123.4015
124.1057	19596.28	123.6492	19599.07	124.642	19594.38	123.6453	19596.32	123.6542	19593.78	123.6421	19593.17	123.6609
124.3559	19598.88	123.8949	19600.17	124.8928	19594.16	123.8964	19593.38	123.8996	19596.72	123.8933	19595.74	123.9006
124.6052	19598.51	124.1507	19598.71	125.1424	19592.57	124.1491	19595.37	124.1473	19595.64	124.1415	19594.96	124.1563
124.8549	19599.82	124.3994	19600.52	125.3955	19592.48	124.399	19595.82	124.4018	19594.21	124.394	19594.98	124.4017
125.1076	19599.14	124.6487	19598.62	125.6426	19593.37	124.6451	19595.76	124.6536	19597.63	124.6426	19594.6	124.6599
125.3587	19598.95	124.8946	19599.76	125.8954	19594.08	124.8987	19595.22	124.9001	19595.55	124.895	19593.69	124.9047
125.6084	19596.94	125.1496	19600.41	126.1424	19593.76	125.1492	19594.2	125.1468	19595.98	125.1428	19595.76	125.1563
125.8553	19597.54	125.3992	19600.18	126.394	19592.86	125.408	19593.69	125.4009	19596.68	125.3943	19596.33	125.4023
126.104	19599.1	125.6525	19602.29	126.643	19594.08	125.6511	19595.14	125.6561	19594.7	125.6409	19594.58	125.661
126.3568	19598.51	125.8952	19601.51	126.8962	19591.03	125.8973	19593.74	125.9032	19595.27	125.8952	19595.67	125.9032
126.6067	19596.15	126.1509	19598.1	127.1441	19592.52	126.1496	19594.07	126.1477	19595.7	126.1448	19596.49	126.1551
126.8583	19596.22	126.4011	19600.11	127.3958	19593.49	126.3975	19595	126.4008	19593.92	126.3977	19594.07	126.4007
127.1083	19597.86	126.649	19599.95	127.6418	19591.93	126.6448	19596.01	126.657	19595.47	126.6412	19595.65	126.6625
127.3578	19596.88	126.8966	19599.66	127.894	19591.47	126.8996	19597.53	126.904	19596.85	126.8967	19594.35	126.9034
127.6055	19597.62	127.1518	19596.53	128.1441	19594.89	127.1509	19596.05	127.149	19596.47	127.1455	19595.43	127.1579
127.8599	19599.22	127.415	19599.66	128.4005	19590.56	127.3973	19595.69	127.4011	19596	127.3983	19594.53	127.4027
128.1078	19599.01	127.6506	19598.64	128.6447	19593.29	127.6468	19592.58	127.6553	19594.71	127.6427	19593.32	127.6605
128.3582	19595.89	127.8937	19599.17	128.8955	19592.03	127.8969	19594.15	127.9024	19594.41	127.8945	19593.82	127.9038
128.6067	19598	128.1524	19600.21	129.1439	19592.39	128.1518	19593.93	128.1488	19592.73	128.1446	19592.8	128.159
128.8571	19598.85	128.4011	19598.73	129.3961	19592.93	128.3987	19595.08	128.4044	19595.3	128.3953	19593.16	128.403
129.107	19598.39	128.6531	19599.55	129.6438	19590.67	128.6465	19594.84	128.6561	19596.88	128.6526	19596.29	128.6622
129.3584	19597.9	128.8977	19597.92	129.8974	19593.22	128.9002	19592.76	128.9018	19595.73	128.8954	19594.28	128.9074
129.6068	19598.96	129.1515	19597.19	130.1435	19590.66	129.152	19595.38	129.1487	19595.23	129.147	19594.54	129.1565
129.8608	19599.94	129.4027	19599.02	130.3965	19591.15	129.3983	19595.07	129.4011	19596.15	129.3956	19594.62	129.4041
130.1085	19602.35	129.6516	19598.16	130.6459	19591.35	129.6485	19596.16	129.6567	19594.61	129.6455	19594.18	129.6624
130.3594	19598.94	129.8965	19598.6	130.8998	19593.5	129.8994	19597.27	129.9046	19592.36	129.8974	19594.68	129.9042
130.6086	19600.15	130.1507	19600.52	131.1456	19590.28	130.1541	19594.98	130.148	19594	130.1451	19595.2	130.1563
130.8608	19599.75	130.4023	19597.66	131.4099	19590.68	130.4052	19595.25	130.4028	19596.16	130.3974	19596.35	130.4031
131.1079	19598.51	130.6535	19598.8	131.6454	19591.48	130.6483	19593.09	130.6573	19593.81	130.6442	19592.06	130.6625
131.3588	19599.16	130.8975	19597.91	131.8975	19590.82	130.8993	19594.93	130.9053	19592.63	130.8981	19594.07	130.9073
131.6081	19595.56	131.1532	19598.35	132.1499	19590.49	131.1529	19594.77	131.1478	19595.41	131.1462	19592.76	131.157
131.8604	19595.82	131.4046	19597.81	132.398	19591.81	131.3992	19592.4	131.4031	19592.73	131.3979	19593.47	131.4025
132.1068	19598.69	131.6518	19596.57	132.645	19591.19	131.6483	19593.67	131.6564	19594.37	131.6444	19593.53	131.6671
132.3604	19596.99	131.8967	19598.08	132.8974	19591.48	131.8999	19595.84	131.908	19593.76	131.8977	19594.9	131.9052
132.6075	19596.98	132.1539	19595.88	133.1457	19593.24	132.1538	19594.6	132.1501	19594.17	132.1472	19593.83	132.16
132.8595	19597.98	132.4032	19597.65	133.4013	19589.72	132.4009	19592.41	132.4038	19595.55	132.3978	19597.33	132.4051
133.1096	19596.29	132.6532	19596.94	133.6461	19591.35	132.6499	19591.86	132.6572	19592.88	132.6449	19594.27	132.6629
133.3651	19596.05	132.8957	19595.85	133.8974	19591.47	132.8993	19596.55	132.9042	19593.89	132.8988	19593.9	132.9189
133.6075	19598.83	133.1537	19598.88	134.1474	19591.71	133.1544	19592.89	133.1522	19594.07	133.147	19592.37	133.1715
133.8626	19594.53	133.4048	19598.98	134.4017	19590.73	133.4	19594.92	133.4069	19595.14	133.3995	19593.47	133.4076
134.1106	19597.64	133.6576	19600.33	134.6475	19591.73	133.6499	19594.22	133.6632	19594.23	133.646	19594.05	133.6659
134.3606	19598.42	133.8966	19599.32	134.8969	19590.57	133.9012	19592.9	133.9064	19594.95	133.8981	19595.43	133.9065
134.6081	19598.82	134.1541	19597.35	135.1466	19591.81	134.1528	19594.2	134.1511	19595.32	134.1454	19592.9	134.1585
134.8603	19598.24	134.4045	19596.55	135.3998	19590.83	134.4004	19593.83	134.405	19592.32	134.3979	19592.92	134.4104
135.1123	19598.51	134.6527	19597.44	135.6475	19591.93	134.6498	19593.17	134.6572	19593.01	134.6459	19594.81	134.6664
135.3613	19596.94	134.8985	19598.28	135.902	19589.93	134.9023	19594.01	134.906	19593.72	134.8999	19596.2	134.913
135.6088	19598.26	135.1536	19595.74	136.1464	19591.06	135.1542	19592.28	135.1503	19594.65	135.1469	19593.71	135.1671
135.8593	19596.19	135.4035	19597.19	136.399	19592.01	135.3996	19593.28	135.4066	19594.71	135.401	19592.57	135.4096
136.11	19597.17	135.6545	19597.78	136.6483	19590.57	135.65	19594.65	135.6582	19595.23	135.6458	19594.4	135.6705
136.3623	19598.31	135.8979	19596.78	136.8998	19591.43	135.9016	19593.14	135.9078	19592.96	135.8988	19592.36	135.9127
136.6103	19596.38	136.1578	19596.95	137.1487	19593.07	136.1528	19593.43	136.151	19592.67	136.1476	19593.61	136.1677
136.8592	19595.9	136.4082	19598.12	137.3978	19590.46	136.4017	19592.75	136.405	19594.58	136.4008	19592.8	136.4107
137.1107	19594.55	136.6536	19598.03	137.6481	19591.6	136.6509	19592.55	136.6589	19592.58	136.646	19595.08	136.6689
137.3623	19596.03	136.8999	19595.35	137.8999	19591.38	136.9019	19592.11	136.9086	19595.21	136.8988	19594.99	136.922
137.6107	19594.83	137.1534	19597.32	138.1484	19591.38	137.1555	19594.2	137.1522	19591.78	137.1472	19593.85	137.1696
137.8601	19596.03	137.4052	19597.69	138.4133	19594.53	137.4026	19592.58	137.4056	19592.06	137.4003	19593.78	137.4061
138.1104	19596.92	137.6528	19599.34	138.6477	19591.26	137.6501	19595.35	137.6572	19595.54	137.646	19594.92	137.6661
138.3635	19595.34	137.8986	19596.61	138.8991	19591.76	137.9023	19592.75	137.9063	19592.87	137.8988	19592.43	137.9065
138.6143	19595	138.1572	19597.28	139.1493	19591.81	138.1556	19593.21	138.1526	19592.37	138.1467	19593.77	138.1636
138.8609	19596.54	138.4063	19595.93	139.4001	19592.85	138.4023	19595.09	138.4051	19593.18	138.4041	19593.25	138.407
139.1108	19597.64	138.6543	19596.07	139.648	19591.02	138.6517	19594.97	138.6576	19594.59	138.6473	19592.66	138.6713

139.3636	19595.89	138.8986	19597.24	139.8982	19591.56	138.9023	19592.52	138.9088	19593.72	138.9003	19593.56	138.9059
139.6105	19596.11	139.1565	19598.68	140.1484	19591	139.1573	19593.43	139.1533	19594.66	139.1464	19592.14	139.1602
139.8616	19599.03	139.4074	19596.11	140.4011	19591.66	139.4023	19591.63	139.406	19593.38	139.405	19594.28	139.4086
140.1116	19597.5	139.6545	19598.06	140.6491	19591.2	139.652	19590.93	139.6597	19591.57	139.6487	19592.64	139.6668
140.3635	19594.53	139.8988	19599.85	140.9015	19591.81	139.9042	19593.54	139.9083	19593.8	139.9025	19593.19	139.9082
140.6099	19599.67	140.1593	19596.74	141.1474	19592.12	140.1542	19591.52	140.153	19594.37	140.1463	19594.34	140.1596
140.8612	19596.06	140.4096	19598.61	141.4014	19591.55	140.4055	19591.22	140.4061	19595.58	140.4036	19594.55	140.409
141.1119	19596.81	140.6576	19596.91	141.6498	19591.79	140.6519	19594.21	140.6594	19592.73	140.6483	19592.86	140.6684
141.3655	19596.88	140.9009	19597.61	141.9008	19590.84	140.9035	19594.58	140.9084	19594.08	140.9012	19595	140.9087
141.6203	19597.81	141.1585	19597.05	142.1484	19592.27	141.1584	19592.13	141.1532	19593.24	141.1486	19593.66	141.1613
141.8616	19594.43	141.4092	19599.46	142.3998	19591.85	141.4035	19593.33	141.4077	19592.38	141.4037	19592.79	141.4086
142.1116	19596.15	141.6555	19598.23	142.6484	19591.29	141.6533	19592.73	141.6594	19592.4	141.6488	19593.99	141.668
142.3867	19594.85	141.9002	19595.84	142.9005	19589.91	141.9212	19592.95	141.9155	19591.64	141.9013	19592.24	141.9102
142.6122	19596.78	142.1575	19596.84	143.152	19590.19	142.1613	19593.64	142.1541	19592.71	142.1501	19594.97	142.1596
142.8611	19595.07	142.4068	19597.96	143.4041	19591.32	142.4046	19591.13	142.4083	19593.02	142.4051	19593.47	142.4103
143.1104	19595.92	142.6598	19596.66	143.6485	19591.54	142.6519	19593.38	142.6624	19590.9	142.6483	19593.21	142.6687
143.3655	19598.9	142.9012	19596.6	143.9019	19590.54	142.904	19591.77	142.9084	19593.11	142.9004	19592	142.909
143.6124	19593.98	143.1571	19596.1	144.1504	19593.05	143.1564	19592.4	143.154	19592.92	143.1495	19593.19	143.1648
143.8635	19594.09	143.4074	19596.24	144.4033	19590.09	143.4063	19593.75	143.407	19592.09	143.4098	19593.71	143.4203
144.1107	19597.15	143.6581	19597.26	144.6483	19590.18	143.6547	19591.47	143.6619	19592.69	143.651	19591.24	143.6751
144.3654	19596.13	143.902	19596.1	144.9014	19590.28	143.9041	19593.06	143.908	19592.56	143.9044	19593.06	143.9179
144.6146	19594.07	144.1616	19597.44	145.1521	19589.27	144.1563	19590.26	144.1528	19593.21	144.1514	19591.8	144.1687
144.8635	19595.94	144.4089	19594.51	145.4036	19591.62	144.405	19592.8	144.4076	19593.15	144.402	19594.33	144.4181
145.1136	19594.92	144.6559	19597.35	145.6494	19590.86	144.6549	19590.49	144.6621	19592.92	144.6507	19593.45	144.6759
145.3673	19594.45	144.9008	19596.35	145.9006	19592.49	144.9073	19593.93	144.9097	19592.58	144.9031	19592.72	144.9185
145.6123	19594.49	145.1582	19598.07	146.1511	19591.41	145.1584	19591.91	145.1551	19592.81	145.1479	19593.87	145.1721
145.8638	19597.92	145.4103	19599.67	146.4047	19591.38	145.4053	19592.18	145.4082	19592.78	145.4035	19591.88	145.4191
146.1145	19595.81	145.6589	19594.11	146.6504	19591.1	145.6539	19591.72	145.66	19592.04	145.6507	19593.45	145.6727
146.366	19594.16	145.9007	19593.17	146.9033	19589.74	145.9053	19592.72	145.9087	19592.92	145.9015	19592.64	145.9203
146.6119	19596.81	146.16	19596.82	147.1496	19592.19	146.1572	19590.04	146.1556	19591.96	146.151	19594	146.1699
146.8642	19598.65	146.4107	19595.65	147.4052	19591.92	146.4049	19593.05	146.4093	19592.32	146.4048	19593.12	146.4236
147.1148	19595.72	146.6578	19597.46	147.6505	19591.58	146.6536	19592.35	146.6609	19592.43	146.6502	19593.8	146.6781
147.3693	19600.87	146.903	19596.34	147.9035	19592.67	146.9051	19592.27	146.9122	19595.05	146.9039	19591.17	146.9272
147.6128	19595.97	147.1573	19597.41	148.1518	19589.54	147.1579	19591.45	147.1566	19592.03	147.1506	19593.63	147.1693
147.8642	19594.95	147.4289	19596.91	148.4108	19589.76	147.4062	19592.12	147.41	19591.93	147.4068	19592.43	147.4216
148.1154	19596.93	147.6579	19596.5	148.6507	19588.02	147.6535	19591.25	147.6619	19590.56	147.6494	19593.01	147.6793
148.3706	19594.96	147.904	19597.11	148.9038	19590.14	147.9063	19594.34	147.9095	19594.73	147.9029	19595.25	147.9151
148.616	19595.13	148.1633	19595.42	149.1533	19591.27	148.1602	19592.87	148.1556	19591.07	148.151	19593.51	148.1703
148.8639	19595.5	148.4102	19595.73	149.4052	19591.22	148.4074	19591.57	148.4093	19591.69	148.4077	19593.05	148.4161
149.115	19594.78	148.6592	19594.73	149.6495	19588.81	148.6579	19592.97	148.6625	19591.26	148.6518	19592.71	148.6834
149.3671	19596.3	148.9042	19596.54	149.9039	19590.97	148.9054	19591.69	148.9108	19593.03	148.9047	19591.59	148.9143
149.6159	19595.1	149.1586	19594.2	150.1523	19590.28	149.1615	19592.14	149.1554	19591.3	149.1515	19592.06	149.1704
149.8651	19595.12	149.4102	19596.64	150.4108	19590.18	149.4072	19593.02	149.41	19593.11	149.4073	19592.56	149.4168
150.1164	19596.8	149.6593	19594.68	150.6542	19588.85	149.6545	19592.97	149.6641	19590.93	149.6515	19590.26	149.6718
150.3705	19595.13	149.9051	19593.76	150.9063	19589.64	149.9134	19591.17	149.911	19591.42	149.9039	19589.93	149.9182
150.6175	19595.26	150.161	19598.1	151.1562	19590.87	150.1654	19592.16	150.1573	19591.75	150.151	19591.29	150.1695
150.8689	19597.38	150.4132	19595.11	151.4091	19590.4	150.413	19592.49	150.4158	19591.39	150.4127	19592.56	150.4242
151.1208	19593.48	150.6624	19592.69	151.6546	19591.21	150.6588	19592.89	150.6666	19593.7	150.6561	19591.86	150.6737
151.3719	19597.33	150.9107	19595.28	151.9094	19591.89	150.9106	19594.4	150.9137	19592.81	150.9084	19594.03	150.9166
151.6185	19593.77	151.1651	19593.58	152.1556	19591.27	151.1624	19592.83	151.1605	19591.16	151.1553	19591.84	151.1786
151.8696	19596.18	151.4158	19596.64	152.4112	19590	151.41	19592.24	151.4152	19591.95	151.4134	19590.7	151.4197
152.1215	19593.33	151.661	19595.99	152.6556	19589.96	151.6588	19592.54	151.6678	19593.4	151.6566	19591.56	151.6766
152.3734	19594.87	151.9086	19594.7	152.9101	19589.1	151.9111	19592.13	151.9171	19592.71	151.9079	19591.43	151.917
152.6186	19598.22	152.1643	19598.98	153.1607	19592.48	152.1631	19592.65	152.1632	19592.16	152.1574	19591.13	152.1716
152.8705	19596.1	152.4134	19596.49	153.4164	19588.81	152.4129	19593.69	152.4155	19591.82	152.4135	19592.93	152.4258
153.1203	19597.51	152.6642	19595.43	153.6551	19591.09	152.6577	19592.77	152.6658	19592.17	152.6543	19592.79	152.6767
153.3756	19595.6	152.9102	19597.19	153.9094	19590.04	152.9126	19592.61	152.9155	19592.97	152.9099	19592.79	152.9171
153.6185	19593.5	153.1651	19593.91	154.1581	19588.89	153.1665	19590.4	153.1611	19591.58	153.1566	19591.91	153.1796
153.8706	19597.24	153.4146	19594.55	154.4128	19590.5	153.4113	19591.72	153.4142	19591.24	153.4147	19590.44	153.4227
154.1216	19594.85	153.6623	19601.5	154.6576	19589.5	153.6602	19591.84	153.6674	19590.32	153.6561	19592.58	153.6756
154.3739	19593.45	153.9102	19599.54	154.9099	19590.33	153.9139	19589.99	153.9161	19592.56	153.9089	19593.28	153.9247
154.6197	19596.28	154.1646	19596.99	155.1571	19588.83	154.1657	19592.14	154.1613	19591.73	154.1567	19592.32	154.1789
154.8685	19597.62	154.4147	19596.44	155.4125	19589.77	154.4127	19592.24	154.4168	19592.3	154.4123	19591.25	154.4168
155.123	19593.87	154.664	19597.82	155.659	19590.05	154.6607	19591.99	154.6695	19591.32	154.6575	19593.1	154.6752
155.3781	19594.64	154.9099	19594.85	155.9124	19590.86	154.9171	19590.59	154.9167	19592.67	154.91	19592.86	154.9262
155.6202	19592.64	155.1661	19595.97	156.1567	19591.72	155.1631	19590.15	155.1657	19591.51	155.1591	19593.42	155.1772
155.8695	19596.72	155.417	19595.03	156.4115	19590.71	155.4125	19591.71	155.4162	19592.19	155.4153	19593.11	155.4286
156.1214	19594.21	155.6635	19596.2	156.6573	19591.52	155.6611	19591.34	155.6679	19591.23	155.6572	19592.03	155.6851
156.3737	19598.4	155.9095	19598.67	156.9121	19590.13	155.9166	19589.99	155.9164	19591.87	155.9101	19592.06	155.9275
156.6213	19596.22	156.1662	19593.56	157.1599	19589.41	156.1666	19590.77	156.166	19593.08	156.1581	19591.53	156.183

156.8701	19592.36	156.4165	19595.89	157.4137	19592.32	156.4145	19592.72	156.4171	19592.04	156.4177	19590.11	156.4288
157.1236	19595.49	156.6656	19595.83	157.6575	19589.68	156.6609	19592.71	156.6672	19592.34	156.6592	19591.91	156.6844
157.3884	19595.89	156.9095	19594.71	157.9114	19591.99	156.9132	19595.27	156.9186	19591.63	156.9097	19591.17	156.9289
157.6253	19595.35	157.1669	19594.62	158.1594	19591.55	157.1647	19590.69	157.1656	19590.33	157.1584	19592.6	157.1811
157.8713	19595.13	157.4152	19597.89	158.4155	19588.83	157.4138	19592.28	157.4189	19591.57	157.4126	19591.44	157.428
158.1213	19596	157.6638	19594.89	158.6578	19590.85	157.6603	19590.91	157.6698	19593.42	157.6582	19591.45	157.6854
158.378	19593.92	157.9082	19593.38	158.9128	19590.06	157.9159	19589.81	157.9174	19592.3	157.9091	19592.97	157.9274
158.6204	19592.64	158.1663	19592.94	159.1596	19590.68	158.1655	19591.8	158.1661	19591.75	158.1591	19590.33	158.1822
158.8705	19594.89	158.4158	19594.68	159.4153	19588.99	158.4148	19590.33	158.4174	19592.78	158.4161	19591.69	158.4279
159.122	19592.54	158.6647	19596.68	159.658	19589.87	158.6618	19591.67	158.6692	19591.2	158.6591	19593.36	158.6862
159.3772	19594.71	158.9101	19595.63	159.9114	19589.87	158.9152	19592.59	158.9175	19590.09	158.91	19592.2	158.9313
159.6217	19594.25	159.1671	19594.41	160.1599	19589.66	159.169	19593.4	159.1658	19591.74	159.1585	19592.16	159.1817
159.8716	19593.3	159.4158	19594.38	160.4161	19590.94	159.413	19590.96	159.4166	19590.21	159.4159	19593.73	159.4284
160.1212	19594.56	159.6646	19593.09	160.6553	19589.75	159.6637	19591.62	159.67	19591.91	159.6591	19590.64	159.6889
160.3769	19596.78	159.912	19595.3	160.9115	19589.61	159.9183	19589.66	159.9188	19591.18	159.9112	19590.49	159.9297
160.6197	19593.3	160.167	19596.46	161.1572	19589.95	160.1658	19592.5	160.1673	19590.62	160.1584	19590.96	160.181
160.8707	19592.11	160.4181	19595.28	161.4121	19590.18	160.4149	19590.47	160.4175	19590.83	160.413	19590.97	160.4284
161.1304	19593.29	160.6617	19595.71	161.6565	19590.52	160.6597	19592.14	160.6663	19591.35	160.6579	19589.54	160.6861
161.3749	19593.8	160.9077	19592.93	161.9105	19592.38	160.9126	19591.9	160.9149	19591.89	160.9121	19591.83	160.9209
161.6225	19590.37	161.1656	19594.13	162.1579	19589.38	161.1635	19592.15	161.1635	19591.23	161.1559	19593.03	161.1777
161.8703	19595.51	161.4161	19594.61	162.4236	19590.53	161.4126	19591.15	161.4156	19592.71	161.4134	19590.94	161.4265
162.1207	19593.79	161.6657	19594.52	162.6577	19590.58	161.6651	19591.33	161.6666	19591.25	161.6563	19589.71	161.682
162.3871	19593.34	161.907	19592.38	162.9105	19588.26	161.9128	19591.19	161.9184	19590.32	161.9101	19591.43	161.9285
162.6219	19597.53	162.1651	19593.3	163.1586	19589.47	162.1644	19590.51	162.1641	19588.92	162.1584	19591.54	162.1794
162.8712	19592.09	162.4147	19597.91	163.415	19588.87	162.4127	19591.15	162.4161	19591.33	162.4151	19591.89	162.4274
163.1208	19594.98	162.6645	19595.16	163.6565	19589.62	162.6623	19589.97	162.6642	19590.22	162.6561	19590.45	162.6866
163.379	19594.68	162.9066	19593.04	163.91	19587.63	162.9124	19592.91	162.9152	19592.26	162.9097	19590.99	162.9288
163.6191	19593.94	163.1642	19595.4	164.1576	19590.9	163.1652	19592.02	163.1641	19591.37	163.1588	19589.91	163.1825
163.8706	19593.09	163.416	19596.03	164.4154	19590.01	163.4119	19592.27	163.4161	19591.04	163.4137	19591.21	163.4267
164.1207	19590.19	163.6646	19594.22	164.6569	19590.04	163.6615	19594.14	163.6679	19590.63	163.6576	19591.54	163.6878
164.3758	19595.56	163.9127	19594.68	164.9105	19589.59	163.9121	19589.79	163.9168	19591.45	163.9129	19591.97	163.9295
164.6199	19595.8	164.1645	19595.17	165.1585	19590.61	164.1648	19591.41	164.1642	19591.42	164.1597	19591.54	164.1797
164.8699	19594.89	164.4161	19594.01	165.4137	19588.86	164.4121	19591.06	164.4167	19591.95	164.4124	19589.65	164.4277
165.1205	19593.13	164.6674	19593.91	165.6606	19589.27	164.6621	19591.4	164.6702	19591.66	164.6579	19591.51	164.686
165.3769	19592.16	164.9106	19592.48	165.9105	19591.06	164.9143	19591.3	164.918	19593.5	164.9117	19591.44	164.9272
165.622	19591.25	165.1686	19594.83	166.1587	19589.72	165.1659	19591.02	165.1612	19590.84	165.1596	19591.95	165.1809
165.8705	19597.02	165.4156	19594.44	166.4127	19590.34	165.4123	19588.26	165.417	19591.82	165.4137	19591.69	165.4291
166.1229	19593.51	165.6647	19598.7	166.6601	19587.82	165.6626	19589.58	165.6669	19591.42	165.657	19592.62	165.686
166.3746	19591.61	165.911	19595	166.9121	19588.41	165.9145	19590.92	165.9175	19590.27	165.9093	19591.86	165.9319
166.6224	19595.04	166.1683	19594.53	167.1583	19590.32	166.164	19590.02	166.1632	19591.41	166.1686	19592.34	166.1803
166.8719	19592.93	166.4202	19594.24	167.4143	19588.76	166.4153	19590.07	166.417	19592.13	166.4166	19591.01	166.4306
167.1202	19596.38	166.6646	19595.24	167.6586	19589.37	166.6632	19591.96	166.6725	19591.68	166.6613	19591.2	166.6877
167.3758	19591.86	166.9099	19596.34	167.9131	19590.28	166.9133	19592.46	166.9196	19590.7	166.9107	19592.3	166.9317
167.6213	19594.41	167.1667	19593.52	168.1604	19590.2	167.1641	19589.53	167.1653	19591.68	167.1608	19591.25	167.1826
167.873	19593.38	167.4471	19594.22	168.4195	19589.04	167.4138	19590.64	167.418	19590.8	167.4171	19590.77	167.4283
168.1321	19591.6	167.6727	19594.86	168.6584	19588.62	167.6621	19591.71	167.6702	19591.96	167.66	19591.39	167.6876
168.3872	19595.79	167.9112	19593.15	168.9127	19590.33	167.914	19592.63	167.9197	19590.71	167.912	19591.25	167.9295
168.6227	19592.62	168.1721	19594.68	169.1607	19587.99	168.1682	19590	168.1635	19591.65	168.1589	19591.47	168.183
168.8718	19593.07	168.4166	19593.18	169.4206	19589.23	168.4134	19589.14	168.418	19590.84	168.4153	19591.86	168.428
169.1234	19593.63	168.6664	19592.2	169.6598	19589.06	168.6643	19590.01	168.6707	19590.47	168.6605	19589.45	168.6904
169.3787	19593.79	168.9114	19592.79	169.914	19589.15	168.9144	19591.65	168.9192	19591.43	168.9132	19590.81	168.9318
169.6229	19592.56	169.1656	19594.87	170.1614	19589.85	169.1651	19591.26	169.1635	19593.62	169.1601	19591.31	169.1831
169.8725	19592.59	169.4183	19595.09	170.416	19590.66	169.414	19590.47	169.4195	19591.79	169.4204	19591.96	169.4302
170.1227	19592.88	169.666	19595.07	170.6596	19589.33	169.663	19590.86	169.6721	19591.22	169.6615	19591.16	169.6927
170.3767	19593.38	169.9124	19594.78	170.9152	19589.6	169.9165	19591.21	169.9349	19590.96	169.9122	19590.96	169.9331
170.6227	19592.26	170.1668	19593.24	171.1616	19589.97	170.1688	19590.1	170.1685	19590.3	170.1762	19590.68	170.1844
170.8744	19596.63	170.4161	19597.75	171.4146	19588.9	170.4144	19589.79	170.4199	19591.11	170.4258	19590.74	170.4306
171.122	19594.81	170.6658	19591.65	171.6603	19590.43	170.6641	19591.04	170.6709	19591.48	170.6616	19590.25	170.6899
171.3787	19595.91	170.9119	19594.48	171.9138	19589.72	170.9141	19590.19	170.9191	19591.45	170.9129	19591.94	170.9312
171.6238	19594.72	171.1698	19593.97	172.1608	19589.58	171.165	19590.8	171.1665	19590.34	171.1606	19590.14	171.1829
171.8756	19593.34	171.4193	19596.63	172.4158	19587.75	171.4153	19589.38	171.42	19590.48	171.4171	19591.13	171.431
172.1222	19594.35	171.6653	19594.55	172.6605	19589.44	171.665	19589.55	171.6685	19590.5	171.662	19590.68	171.6902
172.3812	19594.1	171.9115	19593.68	172.9153	19588.84	171.915	19591.57	171.9224	19591.45	171.912	19593.35	171.9334
172.6243	19590.95	172.1722	19595.16	173.1624	19589.99	172.1705	19589.29	172.1665	19589.64	172.162	19592.07	172.1841
172.8756	19592.57	172.4203	19593.68	173.4215	19589.28	172.4172	19589.94	172.4203	19592.51	172.4187	19590.32	172.4309
173.1244	19594.8	172.6679	19593.89	173.6614	19590.04	172.6636	19590.36	172.6714	19590.98	172.6628	19592.2	172.6866
173.3812	19592.6	172.9115	19598.11	173.9144	19587.96	172.9167	19590.11	172.9195	19589.42	172.913	19591.21	172.9323
173.623	19593.04	173.1704	19589.44	174.1643	19589.15	173.1683	19589.91	173.1663	19590.94	173.1616	19591.02	173.1862
173.8743	19592.67	173.4221	19595.65	174.4177	19588.68	173.4175	19590.9	173.4203	19591.48	173.4194	19589.82	173.4306
174.1248	19595.18	173.6669	19596.48	174.6599	19587.83	173.665	19591.22	173.6706	19590.91	173.6641	19590.78	173.6904

174.3815	19592.07	173.9138	19594.15	174.9134	19589.1	173.917	19589.91	173.9239	19590.87	173.9143	19589.76	173.9335
174.6255	19592	174.1707	19591.79	175.1641	19589.13	174.1689	19590.4	174.1657	19591.57	174.1606	19590.29	174.1835
174.8802	19596.06	174.42	19592.79	175.4176	19589.62	174.4163	19591.43	174.4217	19590.68	174.4189	19591.13	174.4333
175.1256	19591.92	174.6679	19591.98	175.6616	19588.3	174.6661	19589.1	174.6705	19590.81	174.6641	19592.11	174.6898
175.3824	19591.46	174.9163	19594.78	175.9143	19589.08	174.9195	19590.92	174.9202	19591.15	174.9142	19591.55	174.9334
175.6259	19594.59	175.1716	19592.36	176.1631	19590.44	175.1691	19590.29	175.1686	19591.16	175.1633	19590.09	175.1833
175.8762	19593.65	175.4196	19592.76	176.4179	19588.85	175.4163	19589.73	175.4214	19591.63	175.4164	19590.17	175.4336
176.1241	19593.44	175.669	19594.22	176.6627	19588.04	175.6659	19589.04	175.674	19589.84	175.6635	19590.5	175.6917
176.3804	19590.81	175.9145	19593.43	176.9184	19588.78	175.9176	19590.39	175.9205	19590.65	175.9135	19590.64	175.9366
176.626	19593.66	176.1703	19592.32	177.1643	19587.75	176.167	19591.58	176.1683	19589.39	176.162	19590.69	176.186
176.8759	19595.1	176.4208	19596.09	177.417	19588.69	176.4164	19591.27	176.4227	19590.76	176.4211	19592.28	176.4332
177.1254	19592.58	176.6685	19592.81	177.6633	19586.67	176.6662	19589.36	176.6729	19590.4	176.663	19591.71	176.6927
177.3818	19592.45	176.9144	19595.62	177.9168	19589.18	176.9178	19590.71	176.9247	19590.39	176.9158	19590.41	176.9344
177.6263	19594.16	177.1732	19592.93	178.1658	19589.5	177.1679	19590.45	177.1671	19593.31	177.163	19592.04	177.1873
177.8769	19592.79	177.4213	19596.21	178.4226	19587.8	177.4184	19591.64	177.4228	19590.54	177.4197	19590	177.4331
178.1392	19593.17	177.67	19591.62	178.6642	19588.3	177.6662	19591.84	177.6743	19590.74	177.6655	19591.6	177.6921
178.383	19590.88	177.9183	19591.19	178.9181	19589.97	177.9177	19589.74	177.9213	19591.69	177.9149	19591.03	177.9348
178.6249	19592.42	178.1845	19592.23	179.1667	19589.84	178.1717	19590.56	178.1666	19588.45	178.1634	19590.81	178.1871
178.8768	19591.38	178.4331	19590.3	179.4193	19589	178.4189	19590.39	178.4214	19587.38	178.4176	19590.75	178.434
179.1273	19591.56	178.6698	19592.43	179.6639	19588.79	178.6677	19588.62	178.6706	19591.14	178.6644	19591.93	178.6932
179.3828	19595.13	178.9139	19594.29	179.9176	19586.93	178.9205	19589.36	178.922	19589.62	178.9166	19590.34	178.9351
179.6298	19592.15	179.1768	19593.56	180.1675	19588.86	179.1722	19591.76	179.1699	19589.69	179.1636	19591.19	179.1874
179.8782	19591.77	179.4204	19593.66	180.42	19587.65	179.4188	19589.79	179.4239	19589.56	179.4198	19590.87	179.4332
180.13	19594	179.671	19595.36	180.6744	19589.47	179.6673	19590.78	179.6722	19590.04	179.6636	19590.7	179.6901
180.3814	19591.26	179.9175	19591.9	180.919	19589.46	179.9224	19590.32	179.9226	19589.49	179.918	19590.69	179.9319
180.6265	19593.73	180.1722	19592.43	181.1691	19587.75	180.1712	19590.07	180.1697	19591.71	180.1648	19590.79	180.1886
180.8781	19591.01	180.4204	19596.15	181.4215	19589.5	180.4192	19588.92	180.4274	19592.28	180.4199	19589.96	180.4361
181.1275	19593.13	180.67	19593.48	181.6652	19589.89	180.6668	19589.38	180.672	19591.35	180.6639	19590.33	180.6944
181.3843	19591.57	180.9161	19593.23	181.9185	19586.68	180.9203	19589.79	180.9229	19589.34	180.9155	19589.5	180.9369
181.6261	19591.76	181.173	19589.65	182.1682	19590.07	181.1704	19589.85	181.1699	19589.72	181.1671	19590.16	181.1876
181.8775	19593.25	181.4222	19594.84	182.4237	19587.14	181.4193	19590.63	181.4229	19589.5	181.4229	19589.75	181.4364
182.1271	19591.3	181.673	19593.35	182.6651	19589.76	181.6674	19589.52	181.674	19589.27	181.6657	19589.27	181.6932
182.3974	19591.82	181.9176	19591.6	182.9174	19588.88	181.9206	19590.26	181.9279	19589.62	181.9179	19591.76	181.9376
182.6269	19593.87	182.1748	19592.58	183.1686	19588.1	182.1698	19589.02	182.1696	19591.87	182.1656	19589.03	182.1882
182.8773	19591.57	182.4229	19596.15	183.423	19589.64	182.4201	19589.71	182.4241	19588.34	182.4206	19589.61	182.435
183.1292	19592.2	182.6711	19592.14	183.6655	19587.68	182.6685	19590.69	182.672	19590.43	182.6662	19590.91	182.6927
183.3832	19591.75	182.9178	19590.83	183.9199	19587.76	182.9203	19589.56	182.9246	19589.35	182.9179	19589.38	182.9386
183.6278	19592.77	183.1751	19595.27	184.1681	19589.89	183.172	19589.7	183.1719	19590.81	183.1654	19590.87	183.2179
183.8818	19594.52	183.4264	19594.72	184.4226	19589.45	183.4203	19589.44	183.4248	19589.93	183.4235	19591.08	183.4469
184.1275	19593.32	183.6727	19595.17	184.6655	19588.27	183.6683	19590.47	183.6745	19589.6	183.6665	19589.91	183.6875
184.3839	19590.19	183.9166	19593.17	184.9216	19586.88	183.9212	19589	183.9247	19589.58	183.9183	19591.95	183.9366
184.6294	19591.87	184.1768	19593.15	185.1668	19587.12	184.1722	19588.97	184.1735	19590.39	184.167	19590.58	184.1886
184.8797	19592.28	184.4226	19593.98	185.4319	19589.54	184.4202	19590.25	184.4268	19591.12	184.4219	19591.45	184.4381
185.1297	19594.24	184.6733	19591.52	185.6708	19588.29	184.6683	19589.54	184.6723	19590.14	184.6676	19590.84	184.6946
185.3829	19591.9	184.9159	19591.82	185.9216	19587.23	184.9236	19589.84	184.9256	19588.73	184.9188	19590.55	184.9399
185.6295	19592.59	185.1755	19594.23	186.1687	19589.26	185.1731	19589.3	185.1725	19591.23	185.1663	19590.52	185.1884
185.8819	19594.79	185.4238	19591.86	186.4224	19586.66	185.4204	19592.1	185.4282	19589.8	185.4259	19589.39	185.4359
186.1292	19595.46	185.6714	19593.77	186.6656	19586.44	185.6682	19591.08	185.6754	19591.2	185.666	19589.96	185.6951
186.3859	19592.35	185.9171	19593.08	186.9199	19588.52	185.9219	19590.34	185.9351	19590.65	185.9183	19591.19	185.9385
186.6332	19592.74	186.1728	19594.34	187.1687	19589.33	186.1733	19591.42	186.1784	19587.95	186.1663	19590.12	186.1916
186.8797	19592.09	186.4234	19593.2	187.4255	19587.61	186.4228	19590.5	186.4299	19591	186.4249	19591.31	186.4391
187.13	19592.47	186.6726	19590.18	187.6657	19590.55	186.6693	19589.2	186.6818	19591.08	186.6665	19590.11	186.6929
187.387	19594.8	186.9178	19592.29	187.9187	19589.13	186.9216	19589.27	186.9281	19589.25	186.9208	19589.48	186.9392
187.6328	19591.04	187.1753	19594.49	188.1684	19587.33	187.1717	19589.86	187.1782	19590.49	187.1659	19589.2	187.1912
187.8795	19586.98	187.4446	19590.92	188.4275	19588.6	187.4218	19590.01	187.4257	19590.07	187.4231	19590.84	187.4377
188.1292	19595.05	187.6729	19592.93	188.6679	19588.21	187.6715	19590.18	187.6751	19590.07	187.6682	19587.56	187.6949
188.3847	19592.42	187.9198	19591.59	188.9202	19587.52	187.9223	19587.79	187.9272	19591.26	187.9198	19589.79	187.9424
188.6322	19592.08	188.1753	19590.9	189.1675	19588.54	188.1724	19589.3	188.1732	19590.29	188.1675	19590.03	188.1942
188.8802	19593.21	188.4229	19591.49	189.424	19588.18	188.4217	19589.81	188.427	19592.88	188.4257	19589.61	188.4388
189.129	19591.54	188.6734	19593.77	189.6685	19586.66	188.6708	19588.85	188.6744	19589.19	188.6671	19588.63	188.6994
189.3917	19594.22	188.9184	19596.36	189.9221	19589	188.9235	19590.38	188.9269	19589.69	188.9226	19589.78	188.9419
189.6356	19590.85	189.1745	19593.66	190.1671	19587.63	189.1724	19590.22	189.1753	19589.89	189.1673	19591.82	189.1897
189.8836	19593.17	189.4253	19591.6	190.4243	19589.78	189.423	19588.39	189.4267	19590.15	189.426	19591.08	189.4386
190.1312	19593.12	189.6739	19593.15	190.6687	19587.9	189.6714	19589.94	189.6764	19589.58	189.6684	19589.85	189.6962
190.388	19589.91	189.9183	19587.65	190.9232	19589.58	189.9245	19590.5	189.926	19591.78	189.9458	19589.23	189.9395
190.6303	19593.64	190.173	19594.56	191.1707	19589.56	190.174	19591.51	190.1738	19591.16	190.1686	19591.25	190.1907
190.881	19590.45	190.4246	19590.58	191.4287	19588.38	190.4221	19589.62	190.4265	19589.19	190.4263	19590.12	190.4381
191.1318	19591.82	190.6741	19591.01	191.6682	19589.7	190.6703	19587.7	190.6768	19591.35	190.6682	19589.5	190.6963
191.3864	19591.68	190.919	19593.24	191.9211	19588.37	190.9281	19589.56	190.9286	19591.2	190.9208	19589.97	190.9408
191.6449	19592.12	191.1763	19591.12	192.1707	19587.29	191.1752	19590.04	191.1721	19589.87	191.1691	19590.77	191.1904

191.8867	19591.2	191.4294	19593.47	192.4259	19589.59	191.4267	19591.03	191.4321	19590.94	191.4277	19588.81	191.4374
192.1305	19591.9	191.6741	19593.08	192.6699	19587.44	191.6719	19589.98	191.6747	19590	191.6699	19588.53	191.6977
192.389	19592.6	191.9212	19590.97	192.923	19587.98	191.9262	19589.29	191.9289	19590.1	191.9238	19589.68	191.9415
192.6345	19589.28	192.1798	19594.37	193.1695	19588.02	192.1751	19588.81	192.1753	19590.65	192.1711	19590.68	192.1923
192.8845	19592.95	192.4266	19592.77	193.4282	19588.44	192.4244	19589.72	192.43	19588.42	192.4261	19588.93	192.4387
193.1414	19590.33	192.6762	19592.13	193.6702	19587.05	192.6728	19590.66	192.687	19591.69	192.6706	19590.26	192.6957
193.3973	19592.16	192.9212	19591.96	193.9226	19589.42	192.9259	19590.98	192.9387	19591.61	192.9227	19588.62	192.9444
193.6393	19589.93	193.177	19593	194.1708	19588.46	193.1746	19589	193.1748	19589.87	193.1701	19587.69	193.1936
193.8834	19591.65	193.4331	19595.46	194.4247	19589.53	193.424	19590	193.4284	19588.9	193.4253	19590.41	193.4403
194.134	19594.14	193.6761	19592.15	194.6694	19588.72	193.6724	19589.12	193.6793	19589.91	193.6709	19589.41	193.6949
194.3888	19591.32	193.9228	19591.83	194.9235	19588	193.9261	19588.93	193.9274	19590.18	193.9242	19591.17	193.9418
194.6351	19592.86	194.1762	19591.81	195.1705	19586.6	194.1736	19589.53	194.1745	19590.37	194.1725	19591.25	194.1935
194.8842	19590.89	194.4292	19593.76	195.4284	19588.06	194.4257	19589.1	194.4281	19590.27	194.4276	19589.49	194.4409
195.1437	19592.27	194.6753	19591.06	195.6694	19586.96	194.6733	19589.88	194.6782	19591.22	194.6719	19589.84	194.6972
195.3893	19592.46	194.9216	19591.88	195.924	19588.8	194.9282	19589.47	194.9294	19588.49	194.924	19590.18	194.9435
195.6356	19594.17	195.1771	19593.71	196.1722	19589.31	195.176	19589.27	195.1743	19590.4	195.1714	19589.83	195.1933
195.8841	19593.89	195.4281	19592.14	196.4272	19588.01	195.4259	19591.31	195.4277	19590.36	195.4287	19591.5	195.4352
196.1397	19592.39	195.6747	19595.78	196.67	19587.42	195.6737	19588.66	195.6796	19588.87	195.6709	19589.64	195.6958
196.3895	19592.13	195.9223	19590.38	196.9233	19588.7	195.9256	19588.51	195.9291	19589.74	195.9245	19590.71	195.942
196.6335	19591.12	196.1775	19592.4	197.1728	19588.48	196.1762	19588.96	196.1764	19588.59	196.1701	19588.09	196.1947
196.8855	19591.16	196.4308	19591.9	197.4302	19589.17	196.427	19590.51	196.4308	19589.53	196.4283	19591.56	196.4395
197.1338	19592.64	196.6765	19592.33	197.6706	19589.47	196.675	19588.61	196.6781	19589.06	196.6716	19590.07	196.6959
197.3892	19592.76	196.9246	19592.91	197.923	19586.43	196.9255	19590.16	196.9328	19590.16	196.9302	19590.37	196.949
197.6455	19592.62	197.1795	19593.47	198.1813	19588.64	197.1815	19589.46	197.1761	19589.59	197.1706	19589.73	197.2113
197.8888	19591.67	197.4293	19596.71	198.4312	19588.5	197.4281	19589.07	197.4334	19588.5	197.4285	19588.93	197.4419
198.1357	19593.5	197.6888	19592.1	198.6717	19588.42	197.677	19590.79	197.6782	19589.32	197.6722	19588.75	197.6968
198.3909	19592.54	197.9267	19591.92	198.9245	19586.99	197.9281	19587.74	197.9275	19589.09	197.9254	19590.04	197.9448
198.6343	19591.74	198.1814	19589.01	199.1724	19588.34	198.1867	19590.33	198.1847	19589.3	198.1807	19588.28	198.2038
198.8847	19590.27	198.4279	19591.33	199.4296	19587.87	198.4272	19588.24	198.4299	19590.62	198.4299	19588.46	198.4421
199.1345	19588.84	198.6765	19593.17	199.6725	19587.07	198.6739	19588.17	198.6803	19587.95	198.6711	19589.29	198.6964
199.3938	19588.78	198.923	19592.5	199.9263	19587.97	198.9277	19589.35	198.9304	19588.57	198.9254	19587.6	198.9407
199.6372	19590.93	199.1798	19591.58	200.1726	19586.65	199.1744	19588.64	199.1757	19589	199.1699	19588.42	199.196
199.8846	19593.39	199.4274	19590.89	200.4448	19588.01	199.4276	19589	199.4314	19590.02	199.4296	19589.81	199.4455
200.1365	19589.36	199.6764	19591.79	200.6812	19588.79	199.675	19590.55	199.6802	19590.19	199.6716	19589.24	199.6982
200.3932	19590.79	199.9231	19593.38	200.9321	19587.99	199.9293	19589.42	199.9329	19590.17	199.9265	19591.04	199.945
200.6506	19592.38	200.1796	19591.58	201.1808	19587.45	200.1766	19587.84	200.1759	19587.86	200.1739	19589.16	200.1958
200.8906	19592.08	200.4334	19590.32	201.4378	19586.01	200.4333	19588.86	200.4349	19588.89	200.4343	19589.3	200.4467
201.1409	19589.34	200.6818	19594.02	201.6757	19588.67	200.6791	19588.3	200.6835	19589.01	200.6777	19588.53	200.7007
201.3958	19594.09	200.9399	19591	201.9296	19589.73	200.9324	19589.18	200.9349	19589.83	200.9297	19591.81	200.9486
201.6609	19591.95	201.1861	19593.03	202.1782	19587.25	201.1812	19589.05	201.1827	19590.5	201.1772	19589.53	201.2016
201.8996	19591.99	201.4337	19592.88	202.4374	19586.43	201.4334	19588.26	201.4348	19589.06	201.4359	19590.82	201.4467
202.1386	19593.21	201.6816	19593.82	202.6781	19587.92	201.6814	19590.18	201.6828	19589.66	201.6769	19588.93	201.7026
202.4139	19589.55	201.9291	19591.69	202.9295	19588.58	201.934	19590.64	201.9512	19590.27	201.9284	19590.26	201.9495
202.6414	19590.54	202.1815	19592.84	203.1777	19588.11	202.2042	19588.38	202.1818	19589.27	202.1783	19589.45	202.2022
202.892	19592.1	202.4348	19592.36	203.4366	19586.87	202.4366	19588.78	202.4361	19589.21	202.4351	19587.72	202.4471
203.1413	19588.9	202.6847	19589.59	203.6788	19588.59	202.6819	19589.73	202.6883	19589.44	202.6833	19589.8	202.7033
203.3948	19589.84	202.9282	19592.76	203.9323	19587.61	202.933	19587.69	202.9342	19588.87	202.9306	19589.65	202.9486
203.6415	19592.1	203.1833	19589.63	204.179	19588.07	203.1808	19589.94	203.1832	19588.07	203.1774	19588.25	203.2028
203.8911	19590.52	203.4339	19593.34	204.4374	19587.6	203.4333	19588.41	203.4353	19588.82	203.4345	19589.67	203.4482
204.1412	19589.92	203.6832	19590.74	204.6779	19588.87	203.6809	19588.98	203.6895	19589.17	203.678	19590.02	203.7048
204.3959	19592.45	203.9301	19589.3	204.9346	19587.49	203.9323	19588.63	203.9368	19590.78	203.932	19587.18	203.9497
204.64	19592.22	204.1856	19591.76	205.1804	19587.35	204.1837	19589.2	204.1826	19589.07	204.1793	19589.53	204.2029
204.8909	19591.58	204.4374	19592.25	205.4364	19587.67	204.4351	19590.54	204.437	19589.56	204.4338	19588.4	204.4488
205.1414	19592.81	204.6837	19588.86	205.6795	19586.17	204.6826	19591.2	204.6866	19591.52	204.6797	19588.83	204.7073
205.4007	19590.52	204.9297	19590.18	205.9306	19588.13	204.9342	19588.6	204.9388	19588.67	204.931	19588.61	204.9499
205.6407	19593.35	205.1863	19591.19	206.1798	19587.83	205.1841	19587.99	205.1831	19588.13	205.1799	19588.99	205.2022
205.8906	19591.01	205.4342	19590.19	206.439	19588.65	205.4346	19588.54	205.4363	19590.87	205.4373	19589.86	205.449
206.1449	19591.14	205.6819	19590.24	206.679	19587.88	205.6822	19590.59	205.6899	19589.84	205.6785	19587.26	205.7096
206.3976	19590.26	205.9302	19590.51	206.9335	19588.45	205.9328	19590.49	205.9389	19590.87	205.9321	19588.33	205.9518
206.6509	19591.44	206.1862	19594.64	207.1806	19585.55	206.1856	19590.23	206.1848	19588.62	206.1804	19587.69	206.2028
206.894	19591.95	206.4379	19590.77	207.4382	19588.24	206.435	19589.68	206.4373	19587.81	206.4375	19589.93	206.4491
207.1408	19591.29	206.687	19595.08	207.6791	19586.72	206.6829	19588.06	206.6869	19589.5	206.6788	19587.37	206.7067
207.3969	19591.81	206.9307	19589.48	207.9331	19588.47	206.9342	19589.83	206.9403	19588.87	206.9315	19589.55	206.9507
207.6419	19593.07	207.1867	19592.24	208.1805	19587.29	207.1857	19588.75	207.1852	19589.39	207.1814	19588.39	207.2043
207.8932	19591.02	207.4545	19592.21	208.4372	19587.21	207.4339	19589.12	207.4382	19589.47	207.4366	19590.26	207.4489
208.1412	19591.4	207.6865	19590	208.6794	19585.29	207.6848	19587.2	207.6881	19589.12	207.6804	19588.44	207.7059
208.397	19593.91	207.931	19590.45	208.9388	19587.78	207.9347	19588.47	207.9383	19590.35	207.931	19588.32	207.9512
208.6416	19593.1	208.1872	19591.28	209.1829	19586.91	208.1855	19590.01	208.1873	19589.65	208.1797	19589.84	208.2061
208.892	19593.25	208.4336	19588.9	209.4367	19587.7	208.437	19589.64	208.4392	19587.98	208.4371	19590.17	208.4506
209.1447	19591.13	208.6864	19592.49	209.6806	19586.84	208.6828	19588.23	208.6865	19588.93	208.6802	19589.58	208.7082

209.3979	19590.28	208.9291	19589	209.9366	19587.85	208.9362	19589.85	208.9402	19587.88	208.9331	19587.07	208.9489
209.6428	19590.73	209.1856	19591.66	210.1814	19588.19	209.1869	19589.83	209.1863	19589.81	209.1817	19589.92	209.2047
209.894	19589.91	209.4365	19592.27	210.4373	19586.96	209.4358	19588.27	209.4377	19591.57	209.436	19588.88	209.4518
210.1444	19590.94	209.6876	19592.49	210.6792	19587.87	209.6829	19587.75	209.6886	19587.64	209.6814	19589.42	209.7068
210.3978	19590.52	209.9308	19592.36	210.9347	19587.28	209.9386	19587.57	209.9404	19586.63	209.9339	19588.2	209.9493
210.6429	19592.56	210.1874	19592.74	211.1824	19588.48	210.1868	19589.8	210.1857	19587.62	210.184	19588.32	210.207
210.8944	19590.4	210.438	19588.44	211.4386	19588	210.4357	19587.87	210.4406	19589.93	210.4381	19589.18	210.4529
211.1442	19591.19	210.6865	19592.3	211.6811	19587.25	210.6845	19588.74	210.6874	19588.37	210.6804	19589.2	210.7062
211.4005	19591.56	210.9322	19588.44	211.9353	19588.97	210.9356	19589.63	210.9421	19587.44	210.9339	19588.54	210.9533
211.6441	19591.3	211.1876	19594.88	212.1858	19588.3	211.1862	19587.57	211.1895	19589.37	211.1834	19589.1	211.2058
211.8951	19592.1	211.4381	19593.46	212.4418	19587.98	211.4358	19588.83	211.4402	19589.22	211.4398	19589.39	211.4511
212.1458	19590.24	211.6867	19591.38	212.6821	19587.65	211.6842	19587.54	211.6887	19587.62	211.683	19589.57	211.7081
212.3996	19589.94	211.9328	19591.57	212.9354	19584.14	211.9363	19590.09	211.9431	19588.13	211.9325	19590.61	211.9555
212.6434	19588.78	212.188	19590.86	213.1838	19586.98	212.1872	19587.98	212.1881	19589.35	212.1834	19589.6	212.2072
212.8944	19591.09	212.4371	19590.85	213.4402	19586.6	212.4382	19588.94	212.4413	19589.56	212.4397	19590.11	212.454
213.1444	19591.92	212.688	19591.5	213.6835	19588.06	212.6868	19589.08	212.6887	19588.25	212.683	19588.96	212.7073
213.4005	19588.6	212.9328	19589.85	213.9339	19586.42	212.9356	19586.79	212.9411	19588.68	212.935	19590.01	212.9529
213.6448	19592.7	213.188	19592.01	214.1837	19587.21	213.1889	19589.65	213.1875	19588.07	213.1841	19587.74	213.2131
213.8959	19593.28	213.4371	19590.75	214.4398	19586.34	213.4374	19586.74	213.4423	19590.39	213.4391	19589.62	213.4501
214.1458	19588.58	213.6898	19592.11	214.6843	19587.61	213.6856	19587.42	213.693	19587.95	213.6835	19589.05	213.7101
214.4026	19588.68	213.933	19590.19	214.936	19588.09	213.9373	19590.23	213.9426	19587.31	213.9354	19591	213.9506
214.6461	19593.57	214.1907	19590.45	215.185	19587.53	214.1861	19589.26	214.1888	19587.28	214.1852	19586.21	214.2068
214.8987	19590.51	214.4379	19591.28	215.4401	19587.3	214.4378	19588.13	214.4405	19588.95	214.4391	19588.89	214.4539
215.1461	19589.67	214.6889	19590.79	215.6836	19587.51	214.6873	19589.92	214.6902	19589.79	214.6839	19588.29	214.7098
215.4024	19591.98	214.9331	19591.11	215.9384	19589.02	214.9398	19589.02	214.9427	19589.23	214.9358	19588.18	214.9493
215.645	19591.64	215.1892	19591.31	216.1858	19588.37	215.1876	19588.52	215.1878	19587.47	215.1856	19589.15	215.2076
215.8976	19592.73	215.4372	19589.43	216.4406	19586.08	215.4382	19587.77	215.4415	19588.04	215.4426	19589.1	215.4513
216.1473	19588.81	215.6899	19592.38	216.6839	19586.5	215.687	19587.77	215.6905	19590.39	215.6842	19590.31	215.7102
216.4031	19588.74	215.9338	19592.11	216.9408	19587.24	215.9389	19588.59	215.9437	19588.1	215.9359	19589.17	215.9501
216.6478	19590.37	216.1894	19592	217.1858	19590.71	216.1891	19586.61	216.1892	19590.04	216.1869	19589.12	216.207
216.8972	19587.9	216.4404	19591.8	217.4422	19584.75	216.4392	19589.23	216.4441	19586.69	216.4421	19587.79	216.4534
217.1484	19593.2	216.6907	19591.06	217.6859	19587.24	216.689	19588.28	216.6899	19588.57	216.683	19589.33	216.7111
217.4037	19588.49	216.9343	19590.82	217.9375	19587.9	216.9378	19586.14	216.9577	19588.13	216.9365	19590.47	216.9531
217.6453	19590.5	217.1896	19592.57	218.1888	19587.99	217.1888	19589.74	217.1881	19588.22	217.1868	19590.19	217.2088
217.8985	19591.99	217.4396	19592.28	218.4451	19588.33	217.4406	19588.92	217.4429	19589.62	217.4418	19589.34	217.4478
218.15	19589.74	217.6903	19590.25	218.6858	19587.96	217.6875	19588.71	217.6925	19588.36	217.6847	19589.03	217.71
218.4003	19588.82	217.9352	19592.91	218.9381	19585.43	217.9401	19588.64	217.9427	19589.14	217.9361	19587.34	217.9538
218.6491	19590.41	218.1912	19589.61	219.1848	19586.75	218.1886	19588.1	218.191	19588	218.1871	19589.33	218.208
218.8982	19590.79	218.4402	19591.9	219.4411	19587.65	218.4407	19589.02	218.4449	19587.4	218.4425	19588.34	218.4483
219.1499	19592.33	218.6911	19593.36	219.6868	19585.21	218.6896	19587.61	218.6922	19588.68	218.6881	19590.61	218.7111
219.4026	19592.27	218.9359	19589.46	219.9405	19586.78	218.938	19588.37	218.9453	19590.09	218.9371	19590.03	218.9526
219.647	19589.39	219.193	19591.31	220.1856	19587.27	219.1924	19587.2	219.1905	19589.56	219.1864	19588.65	219.2108
219.8979	19589.14	219.4406	19593.34	220.4412	19585.99	219.4385	19588.49	219.4431	19591.25	219.4431	19589.03	219.4533
220.1491	19589.97	219.6944	19590.49	220.6868	19587.06	219.6929	19589.02	219.6912	19587.49	219.6863	19588.7	219.7115
220.4064	19589.47	219.9358	19590.69	220.9386	19588.1	219.9441	19587.43	219.9481	19588.37	219.941	19588.04	219.9572
220.6493	19592.73	220.1906	19589.49	221.1869	19586.65	220.1875	19589.17	220.1918	19589.98	220.1853	19589.8	220.2071
220.8996	19594.41	220.44	19592.38	221.4464	19587.88	220.4579	19588.35	220.445	19587.72	220.4418	19585.14	220.4551
221.1475	19591.11	220.6926	19590.64	221.6871	19586.35	220.6937	19587.96	220.6923	19590.53	220.6877	19589.02	220.7114
221.4022	19592.41	220.9358	19590.83	221.9406	19588.32	220.9407	19589.16	220.945	19585.88	220.9389	19589.52	220.959
221.6484	19590.94	221.1897	19590.91	222.187	19586.03	221.1908	19589.02	221.1924	19586.9	221.1901	19590.15	221.2052
221.8994	19591.61	221.4415	19590.61	222.4426	19586.63	221.4419	19588.52	221.4446	19588.56	221.4482	19586.76	221.4564
222.1494	19590.4	221.6927	19592.94	222.6881	19586.96	221.69	19587.64	221.6931	19588.46	221.6889	19587.21	221.7156
222.4198	19590.1	221.9376	19591.66	222.9386	19585.14	221.9412	19588.43	221.9486	19588.99	221.9375	19589.48	221.9594
222.6471	19589.87	222.1918	19590.83	223.1868	19587.73	222.1918	19588.26	222.1935	19587.72	222.1888	19588.29	222.2064
222.8985	19590.77	222.4414	19588.87	223.4472	19587.07	222.4421	19587.72	222.4447	19588.31	222.4462	19588.22	222.4561
223.1489	19588.5	222.6919	19591.68	223.6884	19585.39	222.6917	19587.83	222.6937	19588.68	222.6875	19589.42	222.7098
223.4071	19587.78	222.9358	19592.32	223.9403	19586.78	222.9408	19588.46	222.9475	19588	222.938	19588.86	222.9536
223.6473	19590.56	223.1906	19591.84	224.193	19587.3	223.1899	19588.99	223.1939	19589.3	223.188	19587.3	223.2128
223.8991	19592.01	223.4405	19593.09	224.4481	19588.4	223.4418	19588.63	223.4445	19588.13	223.4437	19589.68	223.4579
224.1516	19592.08	223.6909	19588.82	224.6893	19587.79	223.6929	19586.49	223.693	19588.28	223.6876	19589.29	223.7082
224.4053	19589.96	223.9369	19590.45	224.9404	19587.7	223.9424	19588.22	223.9474	19589.52	223.9406	19589.56	223.9637
224.6514	19590.78	224.1948	19588.76	225.1884	19588.47	224.1897	19588.93	224.1921	19589.09	224.1873	19589.6	224.2095
224.9056	19588.46	224.4475	19594.72	225.444	19586.87	224.4422	19588.09	224.4462	19588.69	224.4439	19588.06	224.4575
225.1536	19590.7	224.6916	19591.59	225.6914	19589.08	224.6932	19588.49	224.694	19587.23	224.6882	19588.5	224.7056
225.4049	19590.6	224.939	19592.29	225.9418	19585.73	224.9445	19589.67	224.949	19590.19	224.942	19587.04	224.9547
225.6501	19589.99	225.1953	19593.74	226.1896	19588.8	225.1913	19588.81	225.1958	19586.78	225.1909	19588.62	225.2106
225.8986	19591.98	225.4409	19594.32	226.4452	19587.62	225.4426	19589.01	225.4444	19589.04	225.4452	19589.85	225.4585
226.1529	19589.94	225.6915	19590	226.6885	19587.28	225.6951	19587.99	225.6934	19588.47	225.6886	19588.07	225.7102
226.4052	19591.69	225.9379	19590.21	226.9426	19587.06	225.9418	19589.06	225.9491	19588.9	225.9423	19590.06	225.959
226.6503	19591.48	226.1928	19588.88	227.1891	19585.66	226.1921	19588	226.1932	19589.35	226.1894	19589.47	226.2091

226.9004	19589.12	226.4445	19591.42	227.4452	19589.59	226.4443	19588.73	226.4463	19588.63	226.4451	19590.69	226.4583
227.1516	19591.71	226.6929	19590.56	227.6898	19586.88	226.6929	19587.48	226.6962	19587.76	226.6887	19587.51	226.7135
227.405	19592.2	226.9394	19591.12	227.9421	19590.19	226.9416	19586.6	226.9555	19588.74	226.9408	19588.31	226.9618
227.6528	19590.02	227.1939	19592.48	228.1903	19586.72	227.2066	19588.19	227.1951	19589.06	227.1903	19589.14	227.2114
227.9007	19592.48	227.4619	19587.35	228.4499	19585.78	227.4497	19589.18	227.4466	19588.13	227.4477	19587.54	227.4591
228.1523	19588.93	227.7012	19594.53	228.6895	19585.14	227.6933	19586.96	227.6999	19587.1	227.6897	19588.37	227.7136
228.406	19590.24	227.9395	19589.7	228.943	19587.99	227.9433	19589.68	227.9459	19588.95	227.9416	19588.96	227.9621
228.6512	19592.25	228.1939	19589.09	229.1904	19586.49	228.193	19588.22	228.1934	19587.89	228.191	19590.09	228.213
228.8999	19589.74	228.4423	19590.78	229.4495	19586.87	228.4555	19588.06	228.4466	19588.9	228.4481	19588.81	228.4611
229.1551	19590.33	228.6925	19590.44	229.6909	19586.92	228.6967	19589.54	228.6963	19588.9	228.69	19586.25	228.7143
229.4073	19591.03	228.9402	19591.28	229.9429	19586.1	228.9459	19589.31	228.9509	19588.71	228.9407	19589.1	228.9683
229.6521	19588.68	229.1975	19593.12	230.1921	19589.67	229.193	19586.09	229.1921	19587.99	229.1903	19588.44	229.2205
229.9026	19588.56	229.4421	19585.82	230.449	19586.96	229.4452	19587.6	229.4473	19589.37	229.4495	19588.87	229.4692
230.1541	19590.27	229.6938	19590.31	230.6892	19588.1	229.6926	19590.1	229.6971	19588.9	229.6907	19589.45	229.7224
230.4074	19591	229.9406	19591.09	230.9416	19586.26	229.9477	19589.67	229.9509	19588.54	229.9408	19588.9	229.9709
230.6508	19590.8	230.1954	19591.04	231.1915	19587.35	230.1944	19587.93	230.207	19588.34	230.1909	19588.67	230.224
230.9008	19592.57	230.4422	19593.16	231.4497	19586.71	230.4458	19588.09	230.4522	19588.79	230.4632	19588.58	230.475
231.1558	19588.4	230.6944	19590.25	231.6929	19588.25	230.6939	19587.7	230.6937	19589.39	230.7011	19589.16	230.7316
231.4064	19593.9	230.9405	19591.49	231.9445	19585.93	230.9444	19587.74	230.9507	19589.16	230.9419	19589.39	231.218
231.6531	19592.05	231.1948	19590.08	232.1916	19586.28	231.1994	19589.98	231.1917	19589.23	231.1897	19587.86	231.464
231.9018	19589.47	231.4451	19593.2	232.4503	19587.34	231.4477	19587.68	231.4487	19587.68	231.45	19587.51	231.7215
232.1558	19595.06	231.6945	19591.65	232.6906	19586.36	231.7008	19588.43	231.6978	19587.51	231.6919	19588.37	231.9671
232.4069	19590.64	231.9432	19588.2	232.9461	19587.29	231.9538	19587.85	231.9545	19588.21	231.944	19588.67	232.2142
232.654	19589.32	232.1959	19593.17	233.1923	19588.2	232.2012	19588.14	232.1955	19587.18	232.1916	19589.7	232.4624
232.9031	19591.58	232.4444	19589.5	233.4516	19588.86	232.4486	19587.6	232.449	19587.56	232.4506	19588.77	232.719
233.155	19591.97	232.6947	19591.96	233.6904	19586.45	232.6948	19587.4	232.697	19589.07	232.6924	19588.21	232.9654
233.409	19589.05	232.9417	19588.54	233.9454	19588.05	232.9462	19587.58	232.9521	19589.08	232.9424	19587.82	233.2169
233.6551	19589.47	233.198	19591.11	234.1931	19588.94	233.1931	19589.28	233.1942	19587.51	233.1924	19589.26	233.4615
233.9031	19593.72	233.445	19588.99	234.4518	19586.94	233.4459	19587.13	233.4491	19587.52	233.4507	19589.54	233.7224
234.158	19588.4	233.7044	19590.05	234.6918	19587.92	233.6939	19588.3	233.692	19588.1	233.69	19588.19	233.9717
234.4091	19590.98	233.9421	19588.26	234.9462	19587	233.9478	19587.26	233.9516	19588.58	233.9426	19589.83	234.2233
234.6536	19586.01	234.1968	19591.43	235.1926	19586.4	234.1958	19587.95	234.1937	19589.2	234.1918	19588.4	234.4693
234.9047	19589.3	234.4459	19589.11	235.4518	19587.86	234.4489	19587.8	234.4508	19586.96	234.4506	19587.9	234.7237
235.1588	19588.15	234.697	19592.29	235.6912	19587.55	234.6955	19587.7	234.6958	19589.32	234.6933	19587.57	234.9718
235.4096	19585.96	234.9427	19587.44	235.9458	19585.71	234.9496	19589.65	234.9526	19589.36	234.9431	19587.68	235.2219
235.6543	19591.82	235.1974	19588.65	236.1941	19585.26	235.1935	19589.56	235.198	19590.04	235.1953	19587.42	235.4699
235.9035	19592.04	235.4504	19589.14	236.4524	19587.44	235.4496	19587.88	235.4497	19588.06	235.4486	19589.65	235.7206
236.1576	19589.63	235.6952	19590.45	236.6949	19588.45	235.6963	19588.49	235.6995	19587.88	235.6946	19588.71	235.9692
236.4101	19590.04	235.9455	19592.97	236.9459	19586.92	235.9466	19588.83	235.9504	19588.82	235.9432	19588.81	236.2166
236.6556	19592.42	236.1978	19589.73	237.1913	19586.34	236.194	19588.35	236.1986	19589.04	236.1928	19587.65	236.4646
236.9054	19589.95	236.4483	19591.06	237.4528	19586.93	236.4499	19587.26	236.4503	19589.35	236.4501	19587.81	236.717
237.1603	19588.8	236.6949	19592.27	237.697	19587.44	236.6958	19588.27	236.699	19589.57	236.695	19587.84	236.9662
237.4089	19589.32	236.9462	19590.97	237.9466	19587.46	236.9489	19588.68	236.9552	19588.39	236.9466	19587.48	237.2156
237.6644	19589.82	237.199	19588.98	238.193	19586.51	237.1945	19588.58	237.1988	19588.4	237.1934	19589.91	237.4635
237.9033	19587.09	237.4416	19589.02	238.4545	19586.28	237.4655	19586.05	237.4512	19588.17	237.45	19589.07	237.7225
238.1581	19589.26	237.7001	19588.52	238.7012	19586.88	237.7128	19587.5	237.702	19589.5	237.694	19587.96	237.9706
238.4118	19590.21	237.941	19591.57	238.9498	19585.67	237.9631	19588.18	237.9547	19588.74	237.9464	19587.63	238.222
238.651	19590.49	238.2003	19591.48	239.1951	19586.4	238.2115	19588.03	238.1994	19588.58	238.1947	19588.44	238.47
238.9055	19591.03	238.4554	19591.37	239.454	19586.3	238.4598	19588.09	238.4499	19587.03	238.452	19588.6	238.7228
239.1593	19589.17	238.7114	19591.24	239.6972	19585.73	238.7077	19589.76	238.6986	19588.24	238.6948	19587.94	238.9741
239.42	19587.66	238.9529	19591.03	239.9496	19586.19	238.9581	19588.09	238.9532	19588.77	238.9469	19587.76	239.2268
239.6617	19589.49	239.1984	19590.85	240.1937	19586.77	239.2095	19586.8	239.2008	19588.4	239.197	19588.49	239.4769
239.9035	19590.28	239.4455	19589.94	240.4551	19587.01	239.4606	19587.65	239.4515	19588.74	239.4522	19588.17	239.7331
240.1582	19591.29	239.6989	19590.86	240.7063	19587.77	239.7091	19585.71	239.6992	19588.12	239.6999	19589.12	239.9816
240.4107	19588.87	239.942	19589.7	240.9543	19585.88	239.9623	19588.89	239.9544	19588.85	239.9489	19586.76	240.2347
240.6478	19592.26	240.2007	19591.81	241.1906	19587.66	240.2132	19587.48	240.2027	19588.84	240.2002	19588.22	240.7324
240.9012	19588.97	240.4447	19590	241.4567	19584.08	240.4669	19588.36	240.4536	19587.82	240.4544	19588.13	240.9885
241.1569	19590.02	240.6944	19588.25	241.6987	19586.51	240.7136	19588.16	240.6921	19588.25	240.698	19589.2	241.4714
241.4118	19592.2	240.9404	19588.91	241.949	19589.61	240.9517	19587.14	240.953	19587.22	240.9419	19586.82	241.7297
241.6531	19589.17	241.195	19588.28	242.2018	19584.8	241.2123	19587.54	241.197	19589.2	241.1921	19587.49	241.9697
241.9026	19592.49	241.4452	19593.02	242.4529	19587.1	241.4557	19588.4	241.4483	19588.48	241.4586	19588.4	242.2291
242.1555	19590.35	241.6928	19591.82	242.6993	19586.91	241.6977	19586.45	241.6956	19587.61	241.6995	19589.5	242.4707
242.4271	19588.1	241.9419	19590.93	242.9466	19585.08	241.9504	19586.03	241.9527	19585.45	241.9479	19588.51	242.7306
242.6607	19588.35	242.1954	19589.56	243.1916	19586.23	242.1921	19588.33	242.1983	19587	242.1966	19588.9	242.9776
242.9031	19588.19	242.4416	19592.47	243.4512	19586.8	242.4478	19588.65	242.4486	19588.27	242.4553	19587.89	243.2276
243.1557	19589.95	242.6936	19591.57	243.6904	19585.88	242.6929	19588.09	242.6972	19587.4	242.6909	19588.28	243.476
243.4082	19589.79	242.941	19590.37	243.9471	19587.59	242.9554	19590.12	242.9492	19588.76	242.9438	19589.62	243.728
243.6531	19588.98	243.1965	19590.27	244.1925	19586.69	243.2063	19587.35	243.1968	19586.2	243.1912	19587.53	243.9785
243.9044	19590.17	243.4418	19591.4	244.4492	19587.29	243.4484	19586.56	243.4496	19588.97	243.4504	19587.16	244.2276
244.1577	19589.73	243.6958	19589.74	244.6919	19584.43	243.6946	19587.06	243.6986	19588.37	243.6915	19590.62	244.4759

244.4096	19586.88	243.9431	19590.92	244.947	19585.87	243.9474	19588.93	243.9497	19588.29	243.9446	19588.19	244.7239
244.6542	19589.72	244.1983	19590.2	245.1935	19586.49	244.194	19589.72	244.1989	19588.33	244.1919	19589.15	244.9759
244.9039	19589.39	244.4426	19590.42	245.4502	19583.77	244.4477	19586.98	244.4493	19585.59	244.4515	19588.43	245.2271
245.1568	19589.22	244.6952	19591.67	245.6918	19585.87	244.6947	19586.23	244.6989	19587.27	244.6974	19588.56	245.4743
245.4097	19588.74	244.9433	19591.3	245.9461	19585.63	244.9491	19587.56	244.9513	19587.96	244.9426	19589.31	245.7275
245.654	19591.05	245.198	19589.74	246.1929	19587.45	245.1948	19586.2	245.198	19587.98	245.1939	19589.11	245.982
245.9026	19590.01	245.4442	19588.45	246.4513	19586.51	245.4502	19587.41	245.4486	19585.93	245.4476	19588.28	246.2341
246.1584	19591.23	245.6945	19590.89	246.6923	19585.94	245.6958	19586.82	245.6978	19588.57	245.6972	19586.18	246.4823
246.411	19588.66	245.9421	19589.14	246.9474	19586.84	245.9487	19589.35	245.9599	19587.46	245.9451	19588.9	246.7378
246.6538	19588.46	246.197	19590.31	247.1922	19585.23	246.1939	19589.14	246.2001	19587.04	246.1931	19588.25	246.9878
246.9041	19589.41	246.4454	19588.68	247.4499	19587.43	246.446	19587.49	246.4503	19587.8	246.4496	19589.88	247.2381
247.1564	19587.95	246.6977	19589.51	247.6932	19585.53	246.6953	19587.37	246.6979	19586.71	246.693	19587.4	247.4878
247.4095	19589.85	246.9418	19590.65	247.9469	19585	246.9482	19587.55	246.9529	19586.62	246.9459	19586.92	247.7455
247.6559	19587.44	247.196	19588.93	248.1929	19586.5	247.194	19587.02	247.1992	19586.79	247.1935	19587.09	248.235
247.904	19588.41	247.4519	19589.2	248.4674	19586.49	247.4477	19587.99	247.4505	19587.36	247.4489	19587.37	248.4837
248.1569	19589.9	247.7048	19587.66	248.7064	19586.3	247.6962	19586.97	247.6974	19589.11	247.6944	19588.61	248.7448
248.4089	19591.44	247.9419	19587.64	248.9471	19585.54	247.9489	19588.63	247.9559	19588.65	247.9456	19587.79	248.9964
248.6556	19587.67	248.1977	19589.96	249.1934	19586.51	248.1951	19586.36	248.1984	19588.13	248.195	19587.62	249.2486
248.9049	19587.87	248.444	19590.85	249.4517	19586.18	248.4465	19586.9	248.4522	19587.39	248.452	19586.45	249.4992
249.1574	19590.67	248.6967	19588.47	249.6925	19587.16	248.6957	19587.68	248.6987	19585.24	248.6962	19588.62	249.9876
249.4111	19589.24	248.9436	19589.86	249.9472	19586.89	248.9485	19590.19	248.9535	19588.59	248.9475	19586.92	250.2429
249.6548	19589.07	249.1958	19592.26	250.1943	19584.99	249.1952	19588.06	249.1962	19588.75	249.1923	19589	250.4927
249.9065	19588.71	249.444	19590.66	250.452	19584.59	249.45	19587.97	249.4505	19587.36	249.4522	19588.93	250.7575
250.1586	19588.67	249.6964	19588.99	250.6939	19585.3	249.697	19586.94	249.6993	19586.32	249.6993	19587.29	251.2473
250.412	19587.66	249.9439	19588.57	250.9476	19586.73	249.9519	19588.32	249.9545	19587.89	249.9458	19588.54	251.498
250.657	19591.21	250.1997	19593.26	251.1944	19585.49	250.1952	19586.07	250.1947	19588.23	250.1946	19588.14	251.7566
250.9075	19590.2	250.4481	19591.03	251.4534	19586.76	250.4563	19586.93	250.4508	19586.82	250.4496	19587.94	252.0107
251.1603	19588.85	250.6946	19589.87	251.6943	19586.28	250.7019	19588.2	250.7	19587.45	250.6957	19588.4	252.2612
251.4183	19587.21	250.9447	19586.68	251.9471	19586.14	250.9493	19589.11	250.9622	19587.25	250.9467	19588.52	252.7577
251.6584	19589.27	251.1968	19588.66	252.1939	19586.42	251.196	19585.38	251.2054	19589.24	251.1944	19588.45	253.0097
251.9072	19589.98	251.4473	19587.56	252.4522	19586.78	251.4472	19588.49	251.4493	19588.28	251.4511	19589.09	253.2616
252.159	19588.96	251.6974	19592.22	252.694	19584.94	251.6979	19586.7	251.699	19586.25	251.6949	19588.71	253.5159
252.4124	19590.62	251.9465	19590.21	252.9492	19586.65	251.9494	19587.12	251.9544	19587.44	251.9472	19588.46	254.0052
252.6601	19588.53	252.1979	19589.3	253.1942	19586.47	252.1968	19586.85	252.1975	19587.01	252.1948	19588.76	254.259
252.9076	19589.57	252.4461	19589.05	253.4572	19586.02	252.4482	19587.68	252.4536	19586.16	252.4499	19587.58	254.5117
253.1595	19591.43	252.6995	19590.75	253.6995	19586.96	252.6976	19588.34	252.6971	19586.36	252.6978	19587.58	254.7716
253.4147	19587.27	252.9454	19587.66	253.949	19588.08	252.9495	19586.47	252.9545	19587.34	252.949	19588.68	255.2672
253.6591	19587.5	253.2012	19588.84	254.1956	19585.99	253.1989	19587.83	253.1982	19589.04	253.1972	19586.4	255.5214
253.9068	19586.7	253.4465	19587.81	254.4561	19585.92	253.4491	19588.51	253.4531	19588.57	253.4514	19589.3	255.7832
254.161	19589.3	253.6981	19590.62	254.6957	19587.65	253.7	19586.31	253.6973	19586.89	253.6992	19588.59	256.2734
254.4128	19589.16	253.9469	19588.94	254.9492	19587.71	253.9514	19587.6	253.9544	19586.41	253.9499	19588.74	256.5296
254.6597	19590.28	254.2005	19589.18	255.1987	19587.99	254.198	19588.65	254.1974	19587.05	254.1964	19588.69	256.7914
254.9076	19588.27	254.4446	19589.58	255.4555	19587.17	254.4519	19587.79	254.4543	19587.78	254.4523	19588.22	257.3045
255.1615	19588.03	254.6999	19589.1	255.6981	19587.56	254.6971	19588.26	254.6998	19588.11	254.6983	19588.34	257.8039
255.413	19590.7	254.9455	19588.98	255.9494	19587.29	254.9504	19588.46	254.9564	19587.89	254.9506	19587.41	258.0599
255.6593	19591.62	255.2039	19589.91	256.1967	19587.87	255.1973	19587.84	255.1993	19586.44	255.1982	19588.53	258.5536
255.909	19589.62	255.4475	19589.23	256.4555	19586.41	255.4517	19587.41	255.4521	19587.09	255.454	19588.46	258.8152
256.1646	19589.98	255.6999	19589.65	256.6973	19587.52	255.6987	19587.33	255.6971	19587.34	255.7007	19586.33	259.3091
256.4172	19585.16	255.9468	19591.45	256.9506	19586.76	255.9488	19587.81	255.9577	19589.15	255.9483	19588.6	259.5651
256.66	19590.44	256.2058	19588.79	257.1966	19586.57	256.1979	19587.26	256.2036	19586.91	256.2005	19587.12	259.8255
256.9086	19590.88	256.4504	19591.71	257.4561	19587.94	256.4621	19586.88	256.4488	19588.41	256.4508	19588.15	260.3169
257.161	19590.3	256.7006	19590.73	257.7	19586.64	256.7029	19587.99	256.7009	19587.97	256.7013	19587.44	260.5739
257.4136	19590.29	256.9459	19591.54	257.9506	19587.78	256.9504	19587.27	256.9586	19588.85	256.9493	19589.33	260.8366
257.6608	19587.21	257.2018	19589.32	258.1986	19585.86	257.1978	19586.92	257.2043	19588.44	257.1973	19589	261.3288
257.913	19590.18	257.4464	19589.84	258.4595	19588.08	257.4507	19587.48	257.4556	19587.85	257.4523	19588.63	261.5909
258.1656	19587.29	257.7035	19587.08	258.7024	19585.35	257.7013	19588.88	257.7014	19586.78	257.7009	19588.42	262.0898
258.4134	19590	257.9531	19591.95	258.9497	19587.37	257.9511	19587.34	257.9532	19587.62	257.9495	19588.56	262.346
258.6602	19587.21	258.2042	19586.16	259.1988	19585.93	258.1985	19587.72	258.1995	19588.67	258.1976	19586.62	262.8461
258.9087	19587.89	258.4474	19589.58	259.4564	19587.64	258.4515	19588.88	258.4571	19587.6	258.452	19589.62	263.1294
259.165	19588.24	258.709	19590.71	259.7015	19585.9	258.7043	19586.19	258.7037	19587.46	258.7014	19587.46	263.6248
259.4153	19591.54	258.9531	19591.58	259.9514	19586.57	258.9541	19586.68	258.9552	19586.18	258.9503	19588.51	263.8868
259.6607	19591.66	259.203	19588.87	260.1997	19588.56	259.2006	19588.54	259.2023	19588.44	259.1977	19587.61	264.3793
259.9098	19591.95	259.4464	19589.15	260.4568	19586.52	259.4516	19586.67	259.4521	19587.53	259.4554	19587.7	264.6381
260.1645	19589.78	259.7019	19594.74	260.7015	19585.9	259.7006	19587.92	259.6983	19587.35	259.7033	19587.98	264.9015
260.4144	19590.65	259.948	19589.3	260.9524	19586.56	259.9547	19585.97	259.9569	19589.09	259.9541	19588.25	265.3951
260.6612	19589.63	260.2038	19588.82	261.1977	19586.07	260.2014	19588.67	260.2036	19588.41	260.2019	19588.09	265.6548
260.9104	19589.23	260.4468	19589.84	261.4555	19585.92	260.455	19587.54	260.453	19587.49	260.454	19587.56	266.151
261.1628	19588.91	260.7028	19589.73	261.7011	19586.15	260.6977	19587.4	260.7009	19587.16	260.7029	19588.36	266.4069
261.4154	19589.21	260.9479	19589.24	261.9513	19585.12	260.9505	19588.14	260.9586	19588.76	260.9536	19588.74	266.6659
261.661	19590.11	261.2071	19589.6	262.2001	19587.49	261.2009	19586.41	261.2031	19587.94	261.2025	19587.99	267.1615

261.9167	19588.5	261.4515	19590.93	262.4581	19587.61	261.4572	19587.55	261.4527	19586.02	261.455	19586.6	267.4209
262.1703	19588.46	261.7021	19590.44	262.7045	19585.75	261.7031	19587.86	261.7028	19587.91	261.7067	19589.91	267.9224
262.4361	19587.94	261.9487	19589.08	262.9551	19586.41	261.9525	19585.78	261.9671	19588.09	261.9565	19585.04	268.1868
262.9141	19591.06	262.2041	19585.93	263.2036	19586.25	262.2013	19584.42	262.218	19587.1	262.2063	19587.09	268.6786
263.1691	19590.56	262.4488	19589.6	263.461	19586.58	262.4687	19589.28	262.4703	19586.02	262.459	19587.72	268.9418
263.4203	19586.69	262.7014	19591.94	263.7078	19585.65	262.7212	19588.92	262.7175	19587.79	262.7121	19587.97	269.437
263.6681	19591.11	262.946	19589.04	263.9586	19585.28	262.9738	19586.71	262.9706	19588.06	262.9617	19588.37	269.7001
263.9127	19589.29	263.2035	19591.23	264.2072	19587.12	263.4582	19588.03	263.2188	19587.83	263.2176	19587.89	270.1998
264.167	19590.01	263.4482	19590.51	264.4683	19586.23	263.7095	19588.9	263.4758	19585.7	263.4723	19587.97	270.4587
264.4183	19589.13	263.6991	19592.64	264.7178	19583.84	263.9646	19587.19	263.965	19589.04	263.9531	19587.69	270.9589
264.6657	19586.9	263.9478	19588.93	264.9614	19585.73	264.2178	19586.92	264.216	19587.55	264.204	19588.64	271.2255
264.9162	19589.3	264.204	19588.44	265.2028	19585.78	264.4754	19587.44	264.4694	19587.67	264.4602	19589.19	271.7242
265.1657	19592.73	264.4475	19588.24	265.4572	19584.96	264.7264	19584.95	264.7196	19587.35	264.714	19587.38	271.9888
265.4168	19590.04	264.7043	19588.62	265.6993	19587.5	264.9827	19586.73	264.9724	19587.51	264.9668	19586.05	272.4857
265.668	19586.16	264.9496	19590.3	265.9531	19586.65	265.4707	19587.91	265.2198	19587.62	265.2167	19588.54	272.7541
265.9133	19589.49	265.2022	19589.99	266.2008	19587.34	265.7228	19588.28	265.4761	19587.59	265.4748	19588.99	273.2528
266.1689	19586.81	265.4512	19588.63	266.4585	19588.91	265.9786	19587.57	265.7287	19586.52	265.7296	19588.5	273.5167
266.4193	19586.42	265.709	19594	266.7009	19586.91	266.233	19586.88	265.9879	19587.95	266.2161	19585.44	274.0147
266.6672	19590.14	265.9548	19589.34	266.9537	19585.6	266.494	19587.71	266.4816	19588.14	266.4714	19588.05	274.2794
266.9139	19588.61	266.2045	19588.82	267.2026	19586.46	266.9813	19587.28	266.7328	19589.03	266.7246	19587.97	274.7815
267.17	19585.53	266.4492	19588.37	267.4593	19587.33	267.2367	19587.71	266.9926	19587.15	266.9782	19587.37	275.0479
267.4209	19588.55	266.7104	19591.66	267.7	19586.33	267.4933	19588.42	267.2454	19587.66	267.2334	19588.69	275.5451
267.6717	19587.39	266.956	19589.47	267.955	19585.31	267.7467	19587.5	267.7328	19587.76	267.7176	19587.63	275.8069
267.9186	19588.11	267.2096	19591.95	268.2045	19587.37	268.0031	19588.7	267.9891	19586.24	267.9697	19586.19	276.3051
268.1696	19591.58	267.4542	19589.55	268.4584	19587.72	268.4901	19588.09	268.2407	19586.6	268.2218	19587.28	276.5691
268.4226	19587.34	267.7274	19589.01	268.703	19587.31	268.7461	19587.63	268.4981	19587.01	268.4804	19587.93	277.0704
268.6721	19589.69	268.2153	19587.7	268.9585	19587.04	269.0049	19588.26	268.756	19587.56	268.7352	19589.45	277.3351
268.926	19591.15	268.4644	19588.18	269.2051	19587.26	269.4957	19587.22	269.2479	19587.66	268.9904	19588.28	277.8411
269.177	19589.36	268.7262	19589.17	269.4628	19587.35	269.7483	19587.67	269.5062	19587.5	269.4889	19587.68	278.3381
269.4296	19590.04	269.2153	19588.07	269.7046	19586.17	270.0073	19587.97	269.7615	19586.55	269.7451	19588.14	278.6021
269.683	19587.75	269.4651	19592.53	269.9544	19586.35	270.2653	19586.34	270.2579	19587.78	270.2315	19585.76	279.1008
269.9308	19589.72	269.7189	19589.21	270.2033	19586.02	270.7512	19587.75	270.5174	19585.68	270.49	19587.03	279.3613
270.1856	19588.73	269.9667	19586.56	270.4603	19583.97	271.0107	19588.24	271.0209	19587.68	270.7492	19588.09	279.8696
270.4389	19589.42	270.2179	19589.45	270.7042	19586.62	271.2725	19588.09	271.5168	19586.58	271.2353	19587.13	280.3652
270.921	19589.88	270.4698	19588.74	270.9546	19586.17	271.7609	19587.23	271.772	19587.19	271.4934	19586.84	280.6295
271.173	19589.15	270.7276	19588.66	271.2032	19587.16	272.0181	19586.08	272.2671	19587.17	271.7484	19587.5	281.133
271.4269	19594.13	270.9754	19589.52	271.4626	19585.33	272.2781	19587.63	272.5258	19587.15	272.0078	19588.6	281.6305
271.6791	19590.38	271.2307	19589.85	271.7077	19586.55	272.7648	19586.89	272.7834	19588.03	272.5029	19586.81	281.8959
271.9281	19588.58	271.4856	19587.94	271.9571	19586.53	273.024	19587.66	273.2747	19588.24	272.7667	19588.16	282.391
272.179	19590.29	271.9702	19590.42	272.2048	19586.33	273.2842	19587.04	273.5391	19587.47	273.2547	19587.25	282.6451
272.4323	19590.1	272.2236	19591.23	272.4597	19587.85	273.7705	19586.63	274.0368	19586.06	273.5147	19589.47	282.9025
272.685	19585.55	272.4753	19591.21	272.7036	19585.61	274.0292	19588.3	274.2917	19586.46	273.7716	19587.65	283.3973
272.9347	19589.47	272.718	19590.82	272.9575	19585.12	274.2917	19586.15	274.5521	19589.15	274.2607	19586.21	283.6524
273.1865	19588.93	272.9709	19590.21	273.2043	19586.1	274.7791	19588.11	275.0446	19588.27	274.521	19585.86	283.9106
273.4467	19586.51	273.224	19589.18	273.4621	19587.69	275.0384	19589.29	275.3008	19585.96	274.7816	19584.99	284.4075
273.9313	19588.98	273.4745	19591.24	273.7077	19586.71	275.3048	19587.93	275.562	19587.16	275.2695	19588.18	284.6661
274.1896	19585.68	273.7236	19592.1	273.9573	19584.38	275.794	19587.25	276.058	19587.35	275.5282	19587.45	285.1662
274.4461	19588.08	273.9709	19589.41	274.2043	19586.11	276.0534	19586.96	276.3158	19587.02	275.7862	19586.8	285.418
274.7014	19589.37	274.2241	19588.81	274.4599	19584.46	276.3163	19589.08	276.8137	19585.86	276.2745	19588.25	285.9277
275.1892	19589.29	274.4718	19588.38	274.7043	19584.93	276.8028	19586.26	277.0752	19586.68	276.5349	19587.21	286.4242
275.446	19588.77	274.7239	19589.55	274.9595	19586.87	277.0624	19586.47	277.5687	19587.39	276.795	19588.42	286.6774
275.7026	19590.55	274.9714	19588.46	275.2063	19586.8	277.3259	19585.4	277.8358	19587.28	277.2853	19586.96	286.9389
275.9556	19589.13	275.2247	19587.2	275.4613	19585.2	277.8122	19587.23	278.3286	19586.8	277.5358	19587.68	287.4363
276.4494	19587.49	275.4672	19590.74	275.7081	19587.47	278.0735	19586.45	278.5886	19586.26	277.7847	19589.04	287.6883
276.7044	19588.67	275.7233	19590.12	275.9565	19587.29	278.3382	19587.72	278.8527	19587.68	278.0342	19584.6	287.9447
276.9559	19589.51	275.9758	19586.17	276.2067	19586.3	278.8271	19587.54	279.3478	19588.74	278.285	19586.35	288.4446
277.2124	19589.46	276.2317	19590.75	276.4616	19588.19	279.0904	19587.72	279.61	19588.04	278.5382	19587.87	288.7016
277.7019	19588.08	276.4884	19588.64	276.71	19585.18	279.3574	19587	280.1136	19588.29	278.7904	19586.04	289.2028
277.9552	19591.36	276.9751	19587.94	276.9701	19586.74	279.8457	19586.86	280.6122	19587.6	279.0419	19588.61	289.4564
278.2141	19589.49	277.2304	19588.88	277.2298	19587.72	280.1092	19586.36	280.8824	19586.71	279.293	19587.07	289.9591
278.4747	19587.71	277.4846	19590.5	277.7245	19585.58	280.3757	19587.94	281.3784	19586.51	279.546	19587.67	290.2187
278.9623	19590.49	277.7399	19589.45	277.9872	19585.41	280.8663	19588.78	281.6404	19587.01	279.7974	19586.78	290.72
279.2209	19588.28	277.9935	19589.24	278.2454	19587.43	281.1261	19588.1	282.1344	19587.92	280.0472	19586.28	290.9794
279.48	19588.51	278.4856	19587.89	278.7357	19586.1	281.3947	19585.9	282.3961	19588.04	280.2971	19586.45	291.4775
279.9679	19590.19	278.7427	19588.87	278.9962	19587.52	281.8901	19587.89	282.8963	19587.25	280.7899	19586.98	291.7352
280.2245	19585.1	278.9954	19587.55	279.2539	19584.5	282.15	19586.3	283.1571	19588.43	281.0423	19586.45	292.2368
280.4843	19587.28	279.2576	19587.17	279.743	19585.21	282.4181	19586.51	283.6521	19586.78	281.2951	19586.56	292.4915
280.9703	19589.35	279.751	19587.93	280.0039	19587.85	282.911	19586.08	283.919	19587.82	281.5485	19587.26	292.9913
281.2303	19587.95	280.0077	19590.22	280.2616	19585.98	283.1776	19585.62	284.4175	19587.89	281.8002	19587.86	293.2592
281.4897	19588.43	280.2649	19589.57	280.7513	19587.6	283.6711	19587.42	284.6787	19586.35	282.0537	19587.63	293.7633

281.9779	19588.19	280.755	19585.58	281.0143	19587.64	283.9346	19586.75	285.1773	19587.11	282.5477	19587.57	294.2681
282.2363	19585.02	281.01	19588.87	281.2711	19586.17	284.4313	19588.63	285.4327	19586.86	282.7991	19588.72	294.5365
282.4982	19589.61	281.2693	19589.6	281.7589	19585.26	284.6955	19587.31	285.9346	19588.43	283.0512	19588.11	295.0389
282.9872	19588.91	281.7583	19591.59	282.0193	19585.67	285.1865	19585.45	286.1858	19587.12	283.303	19587.1	295.3073
283.246	19589.2	282.0137	19589.26	282.2786	19586.74	285.4569	19588.49	286.4404	19586.15	283.7997	19586.57	295.8119
283.5274	19587.54	282.273	19587.15	282.7696	19585.99	285.9509	19587.48	286.9426	19587.06	284.0528	19588.43	296.3136
284.0164	19588.05	282.7645	19588.14	283.034	19586.85	286.225	19586.6	287.1956	19586.92	284.306	19589.32	296.5829
284.2772	19588.73	283.0201	19590.29	283.5349	19585.12	286.7165	19586.08	287.6941	19587.41	284.5613	19587.97	297.0879
284.5378	19590.68	283.279	19591.89	283.7955	19586.57	286.9827	19587.51	287.954	19587.11	285.055	19587.93	297.5888
285.0278	19587.61	283.7733	19589.76	284.2867	19584.53	287.4799	19587.59	288.4533	19587.69	285.3113	19588.36	297.8611
285.2875	19589.74	284.0298	19591.61	284.5528	19586.92	287.7427	19588.18	288.7095	19586.21	285.5695	19586.88	298.3646
285.5469	19586.74	284.2985	19589.21	285.0464	19587.77	288.2352	19588.03	289.2071	19587.06	286.0624	19587.58	298.635
286.0367	19588.8	284.7898	19586.94	285.307	19586.7	288.5083	19585.99	289.4601	19585.93	286.3185	19587.18	299.1371
286.296	19585.86	285.0456	19589.2	285.7986	19585.9	289.0037	19587.46	289.7157	19587.07	286.5731	19588.3	299.4042
286.5567	19588.56	285.3032	19590.55	286.0619	19583.69	289.2713	19587.71	290.2125	19586.74	287.0657	19588.82	299.9122
287.0513	19588.84	285.795	19588.06	286.556	19585.07	289.7643	19587.37	290.4912	19586.3	287.3231	19586.52	300.4155
287.3114	19589.58	286.0523	19590.42	286.8174	19587.27	290.0332	19588.12	290.9936	19587	287.5785	19588.35	
287.5706	19591.3	286.31	19590.31	287.3101	19587.23	290.5309	19587.06	291.2478	19586.21	288.0735	19587.34	
288.0619	19590.54	286.8006	19587.92	287.5761	19586.12	290.7961	19587.36	291.7463	19587.47	288.3274	19588.15	
288.3233	19588.24	287.057	19591.44	288.0704	19585.75	291.2934	19586.37	292.007	19585.83	288.5832	19588	
288.5841	19593.96	287.3153	19591.59	288.3359	19586.62	291.5614	19587.62	292.5092	19588.07	289.0799	19585.99	
289.0741	19588.21	287.8198	19590.57	288.8323	19587.5	292.0584	19587.07	292.7636	19586.48	289.3493	19587.56	
289.3364	19588.75	288.0791	19590.51	289.0958	19586.25	292.329	19586.96	293.2597	19588.76	289.8432	19587.46	
289.597	19589.84	288.5738	19590.34	289.5932	19587.15	292.8244	19588.19	293.5163	19587.1	290.1078	19588.85	
290.0905	19590.03	288.8335	19586.19	289.8583	19588.02	293.0928	19586.9	293.772	19588.79	290.6191	19588.06	
290.3488	19588.92	289.3261	19587.15	290.3526	19586.19	293.5888	19586.54	294.2697	19587.78	291.1138	19588.06	
290.6092	19589.14	289.5915	19590.16	290.62	19586.44	293.8565	19585.43	294.5265	19585.33	291.3821	19586.93	
291.1009	19588.07	290.0838	19591.76	291.115	19583.88	294.3569	19589.7	295.0398	19586.41	291.8768	19587.08	
291.3616	19589.29	290.3435	19589.17	291.3795	19586.98	294.6272	19586.46	295.5422	19584.55	292.1448	19586.27	
291.857	19589.53	290.6089	19587.53	291.8754	19586.46	295.1245	19586.75	295.8037	19587.36	292.6389	19588.07	
292.1166	19587.72	291.1015	19589.74	292.1418	19585.5	295.3986	19587.25	296.3009	19584.77	292.9062	19587.26	
292.6084	19589.76	291.3604	19587.5	292.6352	19584.76	295.8957	19587.21	296.5649	19586.56	293.4042	19587.58	
292.8724	19584.15	291.6292	19589.2	292.8996	19584.92	296.1648	19588.49	297.0664	19587.87	293.6713	19588.01	
293.3661	19587.29	292.1245	19587.79	293.4028	19585.52	296.6627	19587.39	297.3329	19587.26	294.166	19586.77	
293.631	19587.04	292.3843	19587.78	293.9008	19587.72	296.932	19587.74	297.8378	19588.52	294.4404	19589.22	
294.1247	19587.67	292.8754	19586.94	294.1694	19583.42	297.434	19587.99	298.3377	19587.32	294.9364	19587.83	
294.3917	19587.59	293.1391	19591.25	294.6638	19587.31	297.7029	19587.87	298.6077	19587.26	295.2058	19587.5	
294.8836	19585.82	293.6392	19586.43	294.9369	19586.16	298.1984	19587.68	299.1087	19586.56	295.7012	19586.14	
295.1453	19587.37	293.8994	19588.75	295.4426	19585.92	298.4711	19586.13	299.3774	19587.13	295.9717	19587.5	
295.6369	19589.06	294.3923	19588.02	295.9409	19586.38	298.968	19586.8	299.8833	19586.48	296.4732	19586.74	
295.9042	19588.64	294.6589	19588.89	296.2062	19585.33	299.2386	19587.34	300.3842	19586.83	296.7443	19589.06	
296.4	19587.06	295.1569	19586.67	296.7048	19586.57	299.7352	19587.15			297.2422	19586.84	
296.662	19590.96	295.4188	19585.02	296.9722	19585.5	300.0082	19587.07			297.5165	19587.69	
297.1587	19589.61	295.9123	19590.61	297.4761	19584.5					298.0131	19588.44	
297.4273	19587.25	296.1785	19587.81	297.9738	19586.85					298.2836	19585.84	
297.9205	19588.75	296.681	19592.09	298.2422	19584.85					298.7814	19586.68	
298.1857	19587.87	297.183	19589.29	298.7364	19584.98					299.0505	19587.84	
298.6827	19590.98	297.445	19587.03	299.0055	19584.87					299.5483	19587.76	
298.947	19589.28	297.9387	19588.73	299.5121	19586.25					299.82	19585.04	
299.4469	19588.53	298.205	19590.33	300.011	19582.96					300.3194	19586.22	
299.7131	19588.84	298.7101	19587.62									
300.2134	19589.45	299.2123	19591.79									
		299.4758	19586.93									
		299.9709	19588.41									
		300.2386	19588.46									

	1.43												
	35												
5	*x 1*		*x 2*		*x 3*		*x 4*		*x 5*		*x 1*		
P (cm^{-1})	t (s)	P (cm^{-1})	t (s)	P (cm^{-1})	t (s)	P (cm^{-1})	t (s)	P (cm^{-1})	t (s)	P (cm^{-1})	t (s)	P (cm^{-1})	
19604.05	0	19592.95	0	19592.52	0	19591.65	0	19592.97	0	19592.67	0	19580.45	
19606.07	0.6595	19593.41	0.65986	19593.77	0.66884	19593.69	0.66642	19592.64	0.66669	19592.59	3.27477	19578.86	
19602.09	0.85409	19591.95	0.85013	19593.41	0.85477	19594.22	0.85497	19593.03	0.85441	19594.43	3.49262	19580.08	
19602.41	1.09904	19590.84	1.10003	19593.6	1.10746	19591.55	1.10461	19592.81	1.10353	19595.03	3.67588	19581.16	
19604.59	1.35242	19593.58	1.34774	19591.34	1.35373	19593.75	1.35252	19593.79	1.35295	19597.12	3.96501	19578.85	
19603.91	1.59974	19594.11	1.5997	19591.42	1.60591	19593.95	1.60581	19592.96	1.60449	19593.57	4.17127	19581.16	
19605.5	1.85099	19592.29	1.84735	19593.25	1.85356	19591.6	1.85283	19593.43	1.85264	19593.51	4.42264	19579.33	
19605.56	2.09785	19594.26	2.0984	19591.47	2.10724	19594.14	2.10364	19591.63	2.10102	19595.11	4.66742	19579.81	
19601.55	2.34973	19591.52	2.34796	19594.4	2.35205	19594.35	2.35239	19594.13	2.35192	19593.12	4.91885	19579.58	
19604.66	2.59833	19592.36	2.60136	19593.34	2.60528	19592.11	2.60515	19593	2.60258	19595.51	5.16961	19578.4	
19603.97	2.85083	19591.44	2.84712	19594.45	2.85447	19591.36	2.85368	19592.68	2.85256	19595.33	5.41724	19580.25	
19604.39	3.09925	19592.41	3.09768	19592.18	3.10626	19592.3	3.10295	19594.72	3.103	19592.4	5.66615	19578.94	
19604.13	3.35282	19591.33	3.34851	19591.38	3.35229	19591.9	3.3527	19593.07	3.35263	19593.35	5.91896	19579.44	
19604.35	3.59831	19591.93	3.59758	19591.18	3.60516	19594.26	3.60343	19593.33	3.6026	19595.48	6.17014	19578.92	
19604.52	3.84951	19591.21	3.84692	19593.37	3.85406	19593.03	3.85291	19592.64	3.85252	19595.08	6.41758	19578.4	
19604.07	4.09903	19592.54	4.0977	19590.34	4.10771	19595.13	4.10562	19593.76	4.1027	19594.96	6.66733	19580.36	
19605.62	4.35167	19592.2	4.34887	19594.74	4.35429	19592.63	4.35441	19593.24	4.35369	19594.38	6.9201	19579.85	
19603.73	4.5998	19591.91	4.59979	19593.47	4.60567	19593.67	4.60427	19593.15	4.60371	19595.09	7.17196	19579.39	
19605.29	4.85066	19592.25	4.85271	19592.35	4.85396	19592.86	4.85743	19594.18	4.85238	19593.8	7.42353	19580.44	
19604.25	5.10614	19592.11	5.10217	19594.06	5.10889	19592.71	5.10387	19593.27	5.10573	19592.22	7.66652	19579.15	
19602.47	5.35441	19590.98	5.34736	19591.48	5.35889	19591.47	5.35421	19592.11	5.3585	19594.35	7.91847	19579.73	
19603.47	5.60004	19592.13	5.59855	19591.28	5.60897	19591.96	5.60446	19593.14	5.60394	19595.73	8.17072	19579.09	
19604.15	5.85378	19590.52	5.84971	19592.61	5.85557	19592.42	5.8589	19593.96	5.85338	19593.96	8.42032	19579.38	
19604.1	6.09982	19590.54	6.09864	19593.52	6.10768	19593.94	6.11002	19594.02	6.10258	19594.21	8.66699	19578.59	
19601.94	6.35118	19590.87	6.3484	19594.26	6.35323	19592.95	6.35339	19594.19	6.35332	19594.51	8.91929	19578.88	
19603.99	6.59989	19592.5	6.59864	19591.42	6.60588	19594.59	6.60532	19594.07	6.6052	19593.28	9.17238	19579.77	
19603.43	6.85264	19590.24	6.85036	19591.28	6.85569	19592.73	6.85461	19592.35	6.85572	19596.27	9.41898	19581.01	
19603.81	7.10039	19592.66	7.09984	19593.32	7.10858	19590.63	7.10473	19594.35	7.11787	19593.92	9.6676	19580.38	
19605.6	7.3515	19593.01	7.34914	19589.81	7.35613	19594.27	7.35437	19593.23	7.36212	19593.94	9.92028	19580.57	
19604.92	7.59999	19593.02	7.59907	19591.41	7.61182	19593.51	7.60979	19591.88	7.60508	19594.56	10.17169	19579.02	
19602.41	7.85178	19591.95	7.85027	19591.74	7.85527	19592.29	7.85335	19592.08	7.85386	19594.26	10.41892	19579.16	
19604.32	8.10006	19591.44	8.09946	19593.89	8.11055	19592.45	8.10319	19592.98	8.10529	19592.6	10.66848	19579.09	
19604.05	8.35291	19591.15	8.3517	19593.62	8.36056	19593.61	8.35498	19593.77	8.35518	19596.15	10.92168	19578.82	
19604.69	8.60105	19590.66	8.59921	19592.84	8.60661	19594.93	8.6077	19595.3	8.60422	19593.6	11.17449	19577.78	
19604.88	8.85176	19592.48	8.84964	19592.52	8.85577	19596.38	8.85507	19592.23	8.85421	19592.53	11.48686	19579.41	
19604.88	9.10119	19592.51	9.09938	19592.02	9.10748	19592.78	9.10538	19592.56	9.10446	19593.89	11.66721	19581.28	
19603.66	9.35225	19592.65	9.35148	19592.7	9.35603	19591.78	9.3564	19594.65	9.35478	19593.9	11.9231	19580.03	
19603.86	9.60119	19593.99	9.60008	19590.57	9.60716	19594.82	9.60749	19593.59	9.60557	19592.5	12.17454	19580.03	
19604.47	9.85207	19592.26	9.84979	19591.8	9.85515	19594.46	9.85646	19593.26	9.85447	19593.78	12.42256	19581.49	
19604.68	10.10018	19592.67	10.09984	19593.04	10.10834	19591.2	10.10553	19592.62	10.10376	19595.55	12.66712	19582.33	
19604.78	10.35215	19592.92	10.3519	19594.89	10.356	19593.87	10.3595	19593.7	10.35512	19594.06	12.92183	19578.74	
19604.9	10.6017	19592.48	10.60156	19591.45	10.60909	19592.91	10.60732	19594.12	10.60917	19594.04	13.17271	19580.39	
19603.85	10.85535	19591.63	10.85008	19592.7	10.85738	19592.8	10.85572	19592.22	10.85619	19593.61	13.42099	19580.64	
19604.69	11.10199	19591.74	11.10034	19593.58	11.10955	19592.71	11.10762	19593.03	11.1047	19594.2	13.66797	19579.73	
19603.21	11.35305	19592.55	11.35066	19591.74	11.35516	19592.29	11.35638	19592.16	11.35511	19594.23	13.92059	19580.23	
19603.22	11.60156	19592.01	11.60147	19591.9	11.60797	19593.27	11.60746	19593.13	11.60635	19593.24	14.17372	19580.23	
19602.85	11.85342	19590.76	11.85203	19593.83	11.85801	19593.44	11.85578	19593.89	11.85665	19596	14.4202	19582.93	
19603.11	12.10352	19590.61	12.10148	19593.19	12.10962	19590.31	12.10609	19594.29	12.1072	19593.99	14.67288	19578.54	
19603.25	12.35371	19591.62	12.35118	19592.93	12.35602	19593.51	12.35703	19596.89	12.35908	19593.6	14.92187	19578.68	
19603.05	12.6023	19591.2	12.60069	19591.29	12.60808	19593.53	12.6076	19592.6	12.60617	19595	15.17956	19581.25	
19603.3	12.85283	19591.96	12.85146	19594	12.85764	19592.87	12.85615	19593.84	12.85655	19593.55	15.42224	19580.21	
19603.26	13.10217	19592.58	13.10833	19592.77	13.11165	19593.01	13.11229	19595.17	13.10823	19593.08	15.66862	19580.45	
19603.48	13.35405	19591.68	13.35133	19593.01	13.35626	19594.3	13.35591	19595.39	13.35556	19594.29	15.92222	19580.45	
19604.41	13.60182	19592.45	13.6008	19593.38	13.60837	19593.21	13.60826	19592.55	13.6065	19594.39	16.17635	19579.37	
19604.84	13.85276	19590.94	13.85096	19591.78	13.85788	19592.17	13.85649	19594.16	13.85508	19594.22	16.42619	19579.16	
19602.89	14.10281	19592.14	14.10128	19596.2	14.11232	19591.86	14.10843	19594.71	14.10654	19594.08	16.66826	19580.5	
19605.33	14.35442	19592.39	14.35263	19591.47	14.35638	19593.91	14.35664	19593.19	14.35618	19593.59	16.9214	19577.4	
19604.52	14.6025	19592.27	14.60139	19591.91	14.60833	19590.79	14.60695	19592.52	14.60834	19594.87	17.176	19577.62	
19602.95	14.85412	19592.36	14.85178	19592.38	14.85704	19592.57	14.85653	19594.74	14.85769	19592.69	17.42304	19579.61	
19603.47	15.1023	19592.34	15.10172	19591.67	15.11111	19593.59	15.10822	19594.72	15.10682	19594.46	17.66948	19580.31	
19602.66	15.3537	19592.59	15.35236	19592.5	15.35759	19591.13	15.35661	19593.78	15.35653	19593.86	17.92202	19580.25	
19603.59	15.60424	19589.98	15.60244	19591.2	15.60967	19593.63	15.60863	19593.81	15.60758	19595.95	18.17895	19579.55	
19604.4	15.86407	19590.67	15.85186	19593.29	15.85778	19593.29	15.86193	19595.88	15.85835	19594.91	18.42282	19578.21	
19604.76	16.10371	19591.58	16.10174	19592.9	16.11247	19594.14	16.10982	19594.83	16.10815	19595.19	18.67013	19580.69	

19603.16	16.35476	19590.34	16.35307	19594.31	16.35751	19593.09	16.35845	19593.2	16.35743	19595.27	18.92335	19580.42
19603.39	16.60277	19591.45	16.60292	19592.38	16.61114	19591.94	16.60886	19594.59	16.60691	19593.98	19.17845	19580.21
19604.33	16.85489	19592.69	16.85322	19591.36	16.85846	19592.87	16.85681	19592.4	16.85691	19595.38	19.42656	19578.67
19604.15	17.10437	19590.96	17.10225	19592.27	17.11189	19591.73	17.10984	19591.69	17.10656	19595.06	19.67182	19579.81
19602.71	17.35414	19592.29	17.35144	19593.34	17.3567	19593.79	17.35844	19591.83	17.35798	19593.48	19.9239	19580.08
19603.46	17.60303	19592.56	17.60282	19593.34	17.61014	19593.83	17.62654	19593.63	17.60729	19592.1	20.17532	19579.86
19604.1	17.85549	19592.17	17.85328	19593.31	17.85952	19593.72	17.85726	19595.44	17.85746	19595.53	20.42369	19580.45
19603.28	18.10393	19592.24	18.10476	19594.27	18.11193	19591.43	18.10809	19595.28	18.10798	19593.55	20.67811	19581.19
19604.03	18.3559	19593.03	18.35229	19592.9	18.35688	19594.92	18.358	19592.9	18.35752	19594.81	20.92488	19578.72
19603.8	18.60453	19591.93	18.60178	19593.18	18.61019	19592.95	18.60998	19593.66	18.60688	19594.06	21.17656	19580.38
19606.27	18.85441	19591.41	18.85203	19594.69	18.85919	19594.02	18.85851	19593.03	18.85818	19595.34	21.42306	19581.94
19604.62	19.10942	19592.46	19.10709	19595.66	19.11351	19593.21	19.10818	19592.12	19.10751	19594.42	21.67166	19580.63
19603.3	19.35589	19593.94	19.35722	19593.7	19.35841	19594.16	19.35725	19594.38	19.35817	19594.08	21.92629	19579.36
19603.84	19.60589	19592.03	19.60314	19592.91	19.6114	19594.35	19.60936	19594.65	19.60962	19593.49	22.17864	19578.43
19602.55	19.85588	19590.71	19.8532	19594.66	19.85894	19594.11	19.85946	19594.32	19.85892	19594.55	22.42923	19580.2
19602.49	20.10373	19592.53	20.10389	19593.07	20.11311	19594.31	20.11077	19594.12	20.10722	19595.33	22.67213	19577.19
19603.73	20.35648	19593.06	20.35518	19593.09	20.36181	19593.12	20.35809	19591.55	20.35907	19593.29	22.92592	19579.3
19604.45	20.60585	19593.36	20.60442	19593.03	20.61726	19591.17	20.61039	19590.96	20.60972	19593.19	23.17857	19579.71
19603.98	20.85972	19592.32	20.85445	19591.8	20.86048	19592.03	20.85897	19592.97	20.85946	19592.12	23.42542	19577.72
19603.34	21.10623	19591.92	21.10439	19592.34	21.11264	19592.85	21.11217	19594.08	21.10906	19595.05	23.67282	19578.47
19604.36	21.35679	19592.67	21.35456	19591.72	21.36179	19592.91	21.36155	19592.9	21.35877	19595.02	23.92701	19581.16
19603.55	21.60587	19590.92	21.60546	19593.58	21.61358	19593.36	21.61119	19592.53	21.60974	19594.43	24.17791	19581.4
19604.23	21.85779	19592.59	21.85563	19591.45	21.86177	19592.19	21.85982	19593.8	21.86026	19594.92	24.42518	19580.6
19603.25	22.10648	19591.84	22.10616	19593.54	22.11496	19591.91	22.11167	19595.53	22.11138	19593.84	24.67245	19579.64
19604.28	22.35754	19591.77	22.3538	19592.37	22.35967	19593.29	22.36102	19594.3	22.36049	19593.14	24.92793	19577.97
19603.79	22.60645	19591.69	22.6045	19595.4	22.61358	19592.77	22.6108	19592.17	22.60998	19594.46	25.17932	19580.47
19603.98	22.85661	19591.51	22.85535	19591.04	22.86216	19591.74	22.8597	19596.25	22.85914	19594.35	25.424	19579.86
19603.9	23.10704	19592.88	23.10597	19591.46	23.11361	19592.67	23.10994	19592.83	23.11102	19593.92	25.67159	19580.44
19603.88	23.35825	19590.77	23.35765	19593.91	23.36002	19591.54	23.36055	19593.6	23.361	19592.18	25.92547	19580.22
19603.22	23.6075	19591.63	23.60619	19593.5	23.61393	19592.31	23.61246	19594.5	23.61051	19593.41	26.18063	19580.06
19606.03	23.85783	19593.13	23.85656	19591.5	23.86206	19593.68	23.86103	19592.28	23.85919	19594.06	26.42493	19579.31
19604.48	24.10678	19590.57	24.10521	19592.4	24.11408	19590.94	24.11106	19593.69	24.11076	19595.06	26.67187	19580.44
19604.13	24.35864	19590.86	24.35582	19591.94	24.36021	19592.11	24.36105	19593.76	24.36077	19595.97	26.9249	19578.8
19605.1	24.60736	19592.36	24.60614	19593.52	24.61222	19592.6	24.61149	19594.04	24.61185	19594.72	27.1901	19578.9
19603.86	24.85803	19590.7	24.85733	19592.08	24.86149	19593.64	24.86052	19593.96	24.85982	19594.01	27.42694	19583.08
19606.47	25.10757	19592.03	25.10407	19593.13	25.11454	19593.46	25.10982	19592.84	25.10901	19593.4	27.67427	19580.49
19604.87	25.3577	19593.01	25.35471	19593.26	25.36108	19594.31	25.35967	19592.63	25.35951	19592.52	27.92748	19579.22
19603.53	25.60696	19591.21	25.60595	19594.62	25.61248	19592.61	25.61182	19593.08	25.61081	19596.4	28.17877	19579.66
19603.72	25.86018	19592.75	25.85558	19591.96	25.8613	19590.77	25.86158	19592.19	25.85986	19592.41	28.42532	19580.85
19603.23	26.10622	19590.29	26.10615	19593.5	26.11367	19591.54	26.11175	19594.13	26.10974	19593.66	28.67383	19579.37
19602.29	26.35744	19591.93	26.35512	19592.08	26.36201	19592.87	26.3596	19593.76	26.35957	19593.71	28.92683	19580.72
19604.83	26.6069	19592.91	26.60455	19592.51	26.6143	19592.29	26.6119	19593.7	26.60989	19594.54	29.17886	19578.84
19603.75	26.85931	19591.74	26.85625	19593.58	26.86166	19593.67	26.86068	19593.74	26.86014	19594.48	29.42738	19581.34
19603.12	27.10912	19590.43	27.10678	19592.63	27.11582	19593.71	27.1118	19592.52	27.11036	19594.51	29.67498	19580.29
19603.41	27.3579	19589.7	27.35477	19592.7	27.36037	19592.06	27.36022	19596.12	27.3605	19595.34	29.92891	19580.83
19604.18	27.60743	19591.83	27.60643	19590.84	27.61425	19593.68	27.6118	19593.87	27.61009	19593.04	30.18123	19579.42
19605.13	27.85861	19593.43	27.85714	19594.67	27.86234	19593.37	27.86149	19593.69	27.8593	19595.2	30.42666	19580.08
19604.33	28.10933	19591.07	28.10722	19593.95	28.11283	19594.39	28.11312	19593.11	28.11016	19595.67	30.67418	19581.05
19603.81	28.35954	19589.95	28.356	19593.22	28.36118	19592.57	28.36188	19592.74	28.36118	19594.51	30.92952	19579.1
19603.31	28.60721	19593.37	28.60755	19592.47	28.61393	19593.81	28.6152	19593.99	28.6125	19594.98	31.18158	19578.82
19602.67	28.85782	19590.67	28.85558	19593.91	28.86419	19593.78	28.86016	19593.61	28.862	19593.66	31.42896	19578.36
19604.43	29.11065	19593.42	29.1068	19593.41	29.11537	19593.51	29.11194	19595.73	29.11055	19593.85	31.67514	19580.4
19604.97	29.35958	19591.96	29.35692	19592.79	29.36139	19593.55	29.36231	19593.15	29.35997	19594.03	31.92821	19581.84
19604.77	29.60988	19592.3	29.60693	19593.79	29.61328	19593.61	29.61297	19594.68	29.61186	19592.5	32.182	19580.51
19605.47	29.85908	19592.1	29.85619	19592.23	29.86328	19593.74	29.86183	19593.51	29.86287	19593.47	32.43502	19579.39
19603.91	30.10904	19591.76	30.10786	19591.72	30.11572	19591.9	30.11293	19592.36	30.11204	19593.45	32.6742	19578.2
19602.93	30.36085	19590.51	30.35868	19592.67	30.36292	19591.35	30.36371	19594.25	30.36147	19594.86	32.92717	19578.59
19603.2	30.61023	19591.35	30.61023	19594.3	30.61716	19595.22	30.61514	19594.32	30.61469	19593.38	33.18489	19581.54
19603.57	30.86411	19592.53	30.85763	19591.86	30.86337	19593.45	30.86264	19592.05	30.86348	19593.91	33.42837	19577.95
19602.4	31.11171	19593.79	31.10783	19595.03	31.11601	19592.86	31.11294	19593.99	31.12196	19595	33.6758	19581.05
19604.7	31.36028	19591.24	31.35763	19594.48	31.3637	19593.64	31.36288	19594.8	31.36211	19595.32	33.93732	19581.09
19605.14	31.60985	19592.36	31.6086	19592.87	31.61642	19593.38	31.61683	19592.95	31.61279	19594.1	34.18351	19579.21
19601.94	31.86204	19590.87	31.85948	19592.9	31.86345	19592.94	31.86817	19594.44	31.8638	19595.68	34.42828	19580.39
19604.26	32.11155	19592.27	32.10895	19592.35	32.11957	19592.17	32.11325	19592.04	32.1129	19593.39	34.67518	19582.34
19603.3	32.36099	19590.8	32.35809	19592.78	32.36398	19592.62	32.36278	19594.33	32.36302	19593.61	34.92933	19583.77
19603.89	32.6085	19591.63	32.60845	19592.99	32.619	19593.04	32.61449	19595.22	32.61232	19596.39	35.18241	19580.35
19603.78	32.86106	19593.13	32.85904	19593.8	32.86491	19591.8	32.86361	19592.53	32.86322	19594.49	35.42869	19582.08
19602.91	33.11214	19591.96	33.11016	19593.59	33.11561	19592.51	33.11408	19594.28	33.11287	19594.36	35.68184	19579.53
19603.79	33.36134	19591.13	33.35798	19594.77	33.36364	19592.1	33.36285	19593.8	33.36338	19595.74	35.93046	19578.09
19604.12	33.60849	19591.92	33.60979	19595.64	33.61612	19593.86	33.62768	19593.98	33.6155	19594.24	36.18362	19579.65

19604.01	33.85973	19592.59	33.85911	19592.24	33.87082	19593.23	33.86442	19593.73	33.86272	19596.93	36.42988	19577.92
19603.52	34.11693	19592.28	34.11082	19593.1	34.12372	19592.88	34.11534	19593.79	34.11276	19593.6	36.6756	19578.59
19604.54	34.36155	19590.72	34.35804	19592.72	34.36389	19593.74	34.3642	19593.11	34.36407	19592.31	36.9342	19579.78
19602.91	34.60983	19591	34.61005	19593.33	34.61487	19592.93	34.61575	19593.81	34.61302	19593.18	37.18506	19579.17
19604.83	34.86115	19591.52	34.86075	19591.96	34.86532	19593.46	34.86247	19592.37	34.86221	19593.47	37.43131	19579.79
19604.77	35.11196	19592.06	35.11085	19593.03	35.11807	19593.85	35.11383	19592.52	35.11378	19595.78	37.67558	19579.93
19602.5	35.35973	19592.24	35.35777	19592.47	35.36415	19593.06	35.36532	19594.07	35.36511	19593.95	37.92818	19580.66
19605.25	35.60966	19590.95	35.61011	19591.2	35.61612	19591.96	35.61705	19593.89	35.61457	19593.78	38.18304	19580.24
19605.49	35.86358	19592.55	35.85976	19591.57	35.86438	19591.98	35.8632	19594.53	35.86432	19594.18	38.4329	19579.31
19603.84	36.11253	19593.41	36.11146	19591.89	36.11727	19593.52	36.11453	19592.33	36.11515	19593.4	38.67737	19578.92
19602.57	36.36107	19591.81	36.3582	19593.31	36.36441	19591.63	36.36432	19593.46	36.36432	19593.66	38.93087	19578.71
19603.69	36.60934	19593.27	36.60871	19594.45	36.61741	19592.35	36.61593	19592.9	36.61637	19595.09	39.18342	19578.22
19603.2	36.86136	19591.63	36.86061	19593.27	36.86579	19592.73	36.86508	19594.68	36.86454	19593.04	39.4314	19577.78
19602.68	37.11306	19593.43	37.11042	19592.89	37.11924	19592.85	37.11682	19592.69	37.11533	19594.47	39.67784	19579.12
19604.72	37.36162	19591.53	37.35884	19591.9	37.36387	19594.42	37.36467	19594.76	37.36607	19592.76	39.93113	19579.9
19604.19	37.60945	19594.6	37.60863	19595.38	37.61808	19593.28	37.61581	19593.45	37.6152	19595.36	40.18399	19580.34
19602.58	37.86115	19593.71	37.86006	19590.88	37.86722	19594.58	37.86484	19591.95	37.87368	19598.19	40.43226	19579.04
19604.37	38.11276	19591.23	38.11567	19595.96	38.11638	19591.85	38.11738	19593.12	38.11616	19595.46	40.67912	19579.39
19603.15	38.36346	19590.85	38.35975	19591.51	38.36429	19592.79	38.36581	19593.82	38.36409	19594.42	40.93126	19580.85
19603.2	38.61226	19590.39	38.60908	19593.67	38.61728	19592.47	38.61661	19591.79	38.6157	19594.26	41.18418	19580.59
19602.78	38.86145	19590.87	38.86009	19590.83	38.86671	19593.35	38.86508	19590.55	38.86908	19594.8	41.43098	19579.31
19603.73	39.12937	19591.46	39.11204	19592.68	39.1187	19590.57	39.1171	19594.47	39.11682	19594.05	41.67893	19581.36
19604.07	39.36237	19590.16	39.36044	19594.42	39.36593	19591.68	39.3664	19592.35	39.36477	19594.32	41.932	19580.2
19603.08	39.61196	19590.39	39.60971	19596.15	39.61785	19591.68	39.6176	19593.17	39.61563	19594.36	42.1855	19580.05
19603.67	39.86299	19591.98	39.8592	19592.09	39.86747	19593.12	39.86439	19593.39	39.86591	19594.32	42.43009	19578.67
19603.42	40.11499	19593.24	40.11122	19592.65	40.11774	19591.33	40.11634	19591.76	40.1162	19593.86	42.67896	19579.36
19603.1	40.3648	19591.5	40.36232	19593.2	40.36755	19592.93	40.36854	19593.25	40.36653	19595.63	42.93312	19579.59
19605.08	40.61404	19592.55	40.61207	19592.18	40.6201	19592.82	40.61888	19591.22	40.61662	19593.49	43.18563	19580.24
19602.88	40.86732	19592.56	40.86096	19592.45	40.86952	19594.06	40.86585	19592.24	40.86524	19594.42	43.43138	19580.73
19602.02	41.11417	19591.7	41.11247	19592.92	41.11866	19592.09	41.11655	19593.32	41.11774	19593.79	43.67876	19578.99
19604.47	41.3632	19592.92	41.36211	19593.52	41.36824	19593.84	41.36774	19591.72	41.36711	19594.45	43.95045	19579.38
19604.04	41.61318	19590.4	41.61297	19592.47	41.62037	19593.14	41.62006	19594.64	41.61857	19592.07	44.20792	19579.91
19604.81	41.86453	19589.69	41.86282	19592.63	41.87069	19591.09	41.8665	19592.62	41.86703	19594.15	44.43576	19578.9
19604.88	42.11875	19593.76	42.11338	19592.21	42.12189	19591.68	42.11821	19592.23	42.11719	19593.83	44.68476	19581.73
19603.62	42.365	19593.16	42.36243	19593.54	42.36672	19593.24	42.36745	19592.7	42.36563	19594.87	44.93248	19579.04
19603.28	42.61342	19594.05	42.61319	19592.05	42.61961	19594.67	42.61979	19594.79	42.61765	19594.48	45.1895	19579.15
19605.02	42.86555	19590.58	42.86363	19593.46	42.86953	19592.97	42.86814	19591.98	42.86831	19593.74	45.43432	19580.88
19604.39	43.1155	19591.68	43.11365	19593.75	43.12111	19595.02	43.1198	19594.2	43.11839	19594.59	45.68039	19579.35
19604.04	43.36524	19592.55	43.36339	19593.05	43.36728	19593.9	43.36655	19592.87	43.36686	19592.61	45.93414	19579.89
19604.1	43.61419	19591.54	43.61379	19592.95	43.62045	19593.2	43.61923	19594.96	43.61791	19594.32	46.18858	19578.03
19603.44	43.86589	19592.03	43.86345	19592.17	43.87088	19593.39	43.86944	19594.18	43.86783	19594.32	46.43442	19579.09
19603.85	44.11747	19593.66	44.11457	19593.28	44.12104	19591.69	44.12004	19595.24	44.12049	19595.04	46.68116	19583.02
19605.08	44.36631	19591.8	44.36312	19593.08	44.36802	19592.28	44.37005	19592.98	44.36798	19592.51	46.93454	19581.95
19602.62	44.61485	19591.83	44.61387	19594.78	44.62016	19592.87	44.61972	19591.58	44.61762	19593.52	47.18846	19576.92
19603.98	44.86607	19591.31	44.86372	19591.69	44.86936	19592.3	44.86933	19594.17	44.86769	19594.96	47.4329	19581.37
19602.61	45.11652	19592.35	45.11546	19590.7	45.12132	19591.45	45.12153	19593.81	45.119	19592.37	47.68103	19579.15
19603.08	45.36526	19593.32	45.36373	19591.28	45.36935	19592.58	45.36966	19594.36	45.36791	19594.23	47.93768	19579.65
19605.15	45.61586	19591.79	45.61405	19591.45	45.62276	19596.2	45.61958	19590.75	45.6195	19593.1	48.18927	19581.43
19604.16	45.87156	19592.47	45.86353	19593.19	45.87055	19592.99	45.86729	19595.31	45.86874	19594.19	48.43404	19579.82
19606.94	46.11827	19594.02	46.11498	19594.15	46.12158	19594.62	46.12136	19592.64	46.11772	19594.13	48.67987	19579.79
19603.48	46.36605	19590.89	46.3649	19591.09	46.36875	19591.48	46.36994	19592.63	46.36835	19593.61	48.93509	19578.95
19603.83	46.61416	19593.06	46.61494	19591.75	46.62194	19592.05	46.62102	19592.63	46.61946	19593.08	49.18912	19581.2
19601.99	46.86812	19592.2	46.86353	19590.8	46.87156	19593.35	46.8681	19595.56	46.87155	19595.5	49.43721	19579.32
19603.94	47.12375	19590.77	47.11326	19592.74	47.12365	19594.43	47.11929	19594.12	47.11945	19594.27	49.68068	19579.12
19603.88	47.36556	19590.77	47.36581	19593.31	47.36926	19594.33	47.36924	19592.45	47.36835	19595.49	49.93236	19581.83
19603.16	47.61433	19592.18	47.6162	19594.93	47.62271	19592.91	47.62049	19591.59	47.6206	19595.47	50.18843	19579.83
19602.88	47.86823	19591.61	47.86343	19590.96	47.87189	19592.4	47.87003	19594.21	47.86983	19593.39	50.43546	19579.62
19605.93	48.11973	19591.6	48.1152	19593.54	48.12167	19593.27	48.12167	19594.23	48.11982	19593.02	50.68016	19579.49
19604.1	48.36716	19592.46	48.36431	19590.99	48.36886	19593.67	48.36907	19594.16	48.36927	19592.99	50.9336	19579.51
19603.13	48.6135	19591.34	48.61531	19591.37	48.62198	19592.84	48.62054	19594.86	48.61992	19592.22	51.19181	19579.21
19603.5	48.86641	19594.28	48.8649	19592.21	48.8707	19592.85	48.86909	19593.25	48.86851	19594.94	51.43426	19581.89
19602.74	49.12204	19592.76	49.11287	19590.31	49.12229	19593.22	49.12102	19594.66	49.11941	19594.35	51.68119	19580.58
19604.28	49.36652	19589.98	49.36309	19594.08	49.36827	19594.82	49.36793	19593.16	49.36885	19592.74	51.93568	19580.62
19601.78	49.61729	19593.2	49.61789	19592.69	49.62339	19593.15	49.62261	19594.6	49.62061	19594.38	52.18557	19578.48
19603.52	49.86586	19593.05	49.86531	19594.05	49.86986	19592.7	49.86948	19592.28	49.86756	19592.23	52.43754	19580.54
19602.53	50.11767	19593.39	50.11391	19593.64	50.12248	19593.61	50.12136	19593.07	50.11952	19595.24	52.68742	19581.5
19603.42	50.36656	19590.74	50.36285	19590.94	50.36963	19591.74	50.36849	19593.49	50.36968	19596.51	52.93828	19580.31
19603.44	50.61556	19593.77	50.61505	19594.93	50.62284	19593.7	50.62199	19592.11	50.62093	19593.2	53.18731	19579.82
19602.67	50.87068	19591.97	50.86604	19593.53	50.87044	19592.73	50.86931	19594.72	50.86975	19592.83	53.43621	19579.46
19602.9	51.11861	19591.57	51.11593	19591.73	51.12191	19593.88	51.12229	19593.26	51.11851	19593.66	53.68348	19579.23

19604.29	51.36648	19592.54	51.36337	19593.24	51.37077	19596.4	51.37181	19593.48	51.3691	19593.85	53.93726	19580.09
19604.6	51.61567	19590.43	51.61437	19592.43	51.62347	19591.26	51.62338	19594.9	51.6199	19594.83	54.18767	19578.4
19603.32	51.86733	19591.83	51.86687	19593.89	51.86979	19593.32	51.86906	19592.61	51.86998	19595.41	54.43523	19578.32
19603.3	52.11866	19592.21	52.11719	19594.34	52.12456	19591.97	52.12107	19593.05	52.12156	19592.75	54.68361	19578.88
19604.71	52.36556	19591.07	52.36569	19593.39	52.37149	19594.96	52.37049	19595.22	52.36817	19594.21	54.93869	19578.45
19603.69	52.61559	19591.92	52.6142	19593.91	52.62327	19592.46	52.62454	19593.05	52.61943	19592.72	55.19159	19579.82
19605.04	52.86791	19592.63	52.86635	19593.95	52.87297	19593.91	52.87063	19592.53	52.86971	19595.08	55.43605	19580.65
19603.78	53.12144	19592.51	53.11685	19592.22	53.12256	19593.58	53.12093	19593.88	53.12153	19595.75	55.6838	19578.95
19603.55	53.36751	19591.69	53.36631	19594.35	53.37002	19593.05	53.37006	19593.4	53.37062	19595.74	55.93596	19581.15
19604.74	53.61547	19591.1	53.61556	19591.74	53.62377	19593.5	53.62218	19593.29	53.61893	19593.12	56.18945	19580.52
19602.95	53.86778	19591.63	53.86635	19593.26	53.87321	19593.5	53.87125	19593.49	53.86992	19592.93	56.43661	19580.46
19604.38	54.11987	19591.62	54.11614	19595.22	54.1233	19594.66	54.12313	19592.8	54.11976	19594.14	56.68305	19580.04
19604.15	54.36869	19592.15	54.36746	19595.26	54.36989	19593.94	54.36951	19592.58	54.37159	19593.91	56.93766	19580.46
19603.66	54.61519	19592.46	54.61687	19593.51	54.62362	19593.93	54.62258	19591.43	54.6218	19594.23	57.19066	19576.48
19602.03	54.86772	19592.4	54.867	19592.96	54.87306	19594.27	54.87013	19594.69	54.86974	19594.95	57.4403	19579.28
19602.67	55.12051	19592.57	55.11503	19591.14	55.12567	19593.24	55.12311	19593.12	55.12019	19595.22	57.68554	19581.47
19602.94	55.36885	19593.41	55.36636	19593.31	55.37108	19593.36	55.37164	19592.34	55.37055	19593.46	57.93537	19579.4
19604.18	55.61808	19591.6	55.61767	19592.96	55.62504	19592.57	55.62389	19595.73	55.62296	19595.2	58.18918	19579.62
19605.38	55.87122	19591.69	55.86717	19592.29	55.87318	19593.4	55.871	19591.72	55.87254	19593.89	58.43672	19579.1
19604.37	56.11911	19592.6	56.11804	19594.55	56.1244	19593.88	56.12224	19592.39	56.12036	19596.15	58.68602	19579.94
19604.39	56.36829	19592.74	56.36632	19594.17	56.37076	19593.2	56.37179	19593.2	56.36952	19597.11	58.93677	19578.63
19603.91	56.61793	19592.83	56.61715	19594.5	56.62354	19591.97	56.62379	19596.22	56.62094	19594.32	59.18766	19579.08
19603.63	56.86934	19591.51	56.86787	19592.22	56.87239	19593.21	56.87063	19592.91	56.87243	19592.61	59.43843	19579.8
19602.51	57.12093	19591.73	57.11724	19593.02	57.12725	19592.34	57.12201	19593.41	57.12256	19592.83	59.68619	19581.9
19602.47	57.3684	19592.89	57.36511	19594.49	57.37308	19592.53	57.37262	19594.15	57.3733	19592.89	59.93761	19580.16
19604.74	57.61702	19592.15	57.61728	19592.59	57.6262	19593.13	57.62655	19593.31	57.62206	19594.55	60.19591	19579.44
19602.4	57.87073	19592.35	57.86832	19593.98	57.8734	19593.87	57.87159	19594.23	57.87162	19594.72	60.43918	19579.88
19602.95	58.12491	19592.06	58.118	19591.79	58.12512	19593.72	58.12262	19592.52	58.12291	19592.68	60.6875	19580.63
19605.46	58.37007	19591.46	58.36745	19590.74	58.37259	19595	58.37214	19593.03	58.37376	19594.54	60.94165	19582.92
19605.01	58.61701	19591.09	58.61776	19593.33	58.62664	19593.78	58.62682	19592.06	58.62301	19593.27	61.1903	19580.19
19605.55	58.86946	19592.35	58.86766	19593.35	58.87525	19592.96	58.8724	19594.27	58.87132	19594.18	61.43758	19580.39
19601.7	59.12846	19592.46	59.11979	19591.88	59.12494	19592.73	59.12272	19591.64	59.12219	19593.56	61.68626	19579.03
19604.72	59.37598	19590.86	59.36637	19590.62	59.372	19592.62	59.37844	19593.06	59.37303	19592.96	61.93982	19579.44
19604.58	59.61835	19593.67	59.6163	19593.13	59.62693	19593.02	59.63344	19593.48	59.62468	19593.32	62.19369	19578.1
19604.99	59.86942	19591.66	59.86728	19592.18	59.87577	19593.46	59.87912	19594.93	59.87268	19593	62.43995	19579.76
19603.07	60.12304	19593	60.11982	19593.64	60.12597	19593.45	60.12505	19595.48	60.12249	19594.21	62.68625	19580.63
19603.73	60.37134	19591.92	60.37199	19592.29	60.37347	19592.54	60.37257	19593.29	60.3752	19593.14	62.94938	19578.58
19602.44	60.62249	19592.12	60.61991	19593.11	60.6272	19592.06	60.62618	19593.57	60.62731	19595.13	63.19375	19580.89
19603.52	60.87425	19592.61	60.86874	19594.94	60.8768	19593.08	60.87374	19591.88	60.8746	19594.32	63.4398	19581.1
19603.59	61.12525	19591.38	61.11908	19593.63	61.12868	19592.92	61.12647	19592.6	61.12516	19593.26	63.68725	19578.76
19604.12	61.37553	19592.93	61.37154	19594.52	61.37495	19593.13	61.37521	19593.95	61.37459	19591.83	63.93985	19581.16
19603.39	61.62071	19591.45	61.62128	19593.98	61.62788	19594.17	61.62715	19593.19	61.62455	19596.01	64.19199	19580.74
19603.27	61.87258	19590.58	61.8697	19595.53	61.87516	19593.46	61.87338	19592.97	61.87539	19594.56	64.44225	19578.52
19603.83	62.12657	19591.64	62.1197	19592.9	62.13028	19591.97	62.12693	19593.58	62.12587	19595.85	64.6907	19581.11
19605.01	62.37237	19591.76	62.36998	19591.04	62.37589	19592.9	62.37628	19595.72	62.37603	19594.53	64.94077	19579.56
19603.84	62.62049	19590.79	62.6215	19594.22	62.62915	19595.67	62.62996	19594.94	62.62476	19592.98	65.20496	19579.9
19603.25	62.87253	19592.25	62.87042	19592.48	62.87718	19594.87	62.87393	19593.81	62.87566	19593.94	65.45234	19579.12
19602.01	63.126	19592.28	63.12233	19592.8	63.12819	19591.26	63.12698	19592.83	63.12606	19595.71	65.68959	19580.58
19603.28	63.37402	19591.94	63.37105	19593.63	63.37687	19594.47	63.37627	19592.25	63.37665	19593.29	65.94763	19579.61
19602.68	63.62288	19591.91	63.62194	19592.85	63.62881	19592.07	63.62971	19593.97	63.62527	19594.08	66.19067	19580.6
19603.47	63.87329	19592.31	63.87081	19593.56	63.87927	19592.39	63.87654	19593.99	63.87462	19595.15	66.44065	19579.3
19604.02	64.1255	19592.47	64.12153	19592.8	64.12957	19592.52	64.12772	19592.91	64.12539	19595.18	66.68815	19581.2
19602.92	64.37297	19591.25	64.37179	19592.79	64.37465	19593.66	64.37492	19592.11	64.37685	19594.44	66.94233	19578.38
19603.92	64.62271	19591.73	64.62201	19595.07	64.627	19592.74	64.62651	19592.34	64.62546	19591.91	67.19029	19578.69
19603.07	64.87266	19592.88	64.86995	19592.47	64.87623	19593.67	64.87574	19594.14	64.8857	19593.41	67.43772	19580.49
19604.22	65.12665	19592.15	65.12277	19591.95	65.12896	19591.37	65.12708	19592.08	65.12367	19594.56	67.69403	19578.49
19603.73	65.37088	19591.48	65.37062	19594	65.37463	19593.51	65.37389	19593.95	65.37486	19593.5	67.94183	19580.85
19605.05	65.61995	19589.86	65.62029	19593.78	65.62779	19592.99	65.62621	19594.51	65.62515	19594.11	68.19344	19579.47
19603.28	65.87554	19591.1	65.87037	19592.77	65.87656	19593.54	65.8751	19592.74	65.87517	19594.62	68.44025	19580.64
19603.07	66.12647	19589.97	66.1222	19593.71	66.129	19592.94	66.12748	19592.23	66.12633	19593.68	68.68613	19581.43
19604.84	66.37287	19592.99	66.37187	19592.23	66.37981	19593.2	66.37576	19591.86	66.37353	19594.23	68.94219	19578.61
19605.16	66.62386	19591.03	66.61962	19594.42	66.62833	19592.5	66.62722	19593.02	66.6253	19593.06	69.20395	19580.06
19602.65	66.87114	19592.34	66.8688	19590.35	66.87526	19593.1	66.87482	19594.59	66.87512	19594.17	69.44186	19581.04
19603.38	67.12626	19591.55	67.12132	19592.13	67.13035	19592.47	67.1268	19594.64	67.12687	19594.39	69.68743	19580.18
19604.09	67.37223	19592.01	67.37126	19594.47	67.37577	19592.61	67.37617	19593.86	67.37641	19594.33	69.93987	19578.92
19604.47	67.62349	19590.45	67.62478	19591.53	67.64612	19593.31	67.62905	19593.52	67.626	19596.6	70.19488	19580.2
19603.79	67.87509	19591.78	67.86882	19592.86	67.88089	19592.01	67.87407	19593.46	67.87561	19592.06	70.44154	19579.72
19603.81	68.12639	19592.65	68.12118	19592.95	68.12821	19594.29	68.12587	19595.23	68.12484	19593.42	70.68829	19579.17
19603.13	68.37303	19592.13	68.37039	19594.5	68.37774	19594.61	68.3745	19593.88	68.37638	19594.68	70.94128	19580.63
19603.24	68.62268	19591.7	68.62262	19594.74	68.62937	19592.37	68.62885	19592.11	68.62677	19593.58	71.19216	19578.2

19603.97	68.87395	19590.46	68.87179	19592.65	68.87783	19594.03	68.87611	19593.31	68.87741	19593.91	71.44041	19580.9
19603.79	69.12856	19589.28	69.12206	19590.23	69.12822	19592.98	69.12674	19593.05	69.1252	19593.74	71.68949	19580.57
19603.78	69.37295	19592.12	69.37106	19591.92	69.37688	19593.47	69.37459	19594.37	69.37527	19594.64	71.94529	19580.04
19602.83	69.62171	19591.66	69.62208	19592.2	69.62984	19591.26	69.62979	19594.6	69.62604	19594.11	72.1913	19578.18
19602.71	69.87464	19591.56	69.87159	19591.98	69.87897	19591.48	69.87764	19593.85	69.87685	19595.21	72.44253	19581.05
19602.41	70.12906	19591.09	70.12285	19593	70.12893	19592.07	70.12941	19592	70.12658	19593.91	72.68866	19580.47
19602.49	70.37355	19594.16	70.37125	19592.51	70.37598	19592.65	70.375	19593.93	70.37507	19592.61	72.94345	19579.29
19604.68	70.62307	19591.39	70.62349	19590.98	70.62847	19591.87	70.63872	19594.9	70.62701	19593.48	73.19412	19578.76
19603.44	70.87605	19592.87	70.87317	19592.49	70.87812	19596.13	70.87703	19594.28	70.87757	19593.74	73.44068	19579.89
19602.51	71.13477	19592.08	71.12499	19593.24	71.1306	19591.27	71.12843	19593.09	71.12826	19593.21	73.68894	19580.59
19604.11	71.37633	19591.54	71.37147	19592.37	71.37657	19591.75	71.37801	19592.52	71.37938	19594.86	73.94386	19582.57
19603.95	71.62345	19592.41	71.62352	19592.98	71.62912	19591.93	71.6295	19593.85	71.62728	19595.06	74.19567	19580.5
19604.01	71.87294	19591.57	71.87288	19591.45	71.87861	19592.7	71.87565	19593.55	71.87642	19594.87	74.44976	19581.09
19603.9	72.13105	19592.18	72.12468	19592.9	72.13261	19593.55	72.12779	19593.88	72.14257	19595.05	74.68884	19580.94
19605.69	72.37516	19592.13	72.37156	19592	72.37873	19592.09	72.37745	19591.33	72.37737	19595.13	74.94398	19580.91
19604.52	72.62423	19591.42	72.62302	19592.29	72.63168	19592.47	72.6318	19592.76	72.62917	19594.15	75.19657	19581.5
19604.76	72.87398	19590.12	72.87204	19593.48	72.87808	19591.64	72.87689	19592.65	72.87776	19594.47	75.44394	19579.39
19603.93	73.13218	19590.94	73.12754	19592.36	73.12986	19592.43	73.12753	19592.11	73.12604	19593.94	75.69112	19578.16
19604.88	73.37436	19592.6	73.3718	19593.33	73.37956	19594.54	73.37639	19592.18	73.37954	19594.48	75.94347	19581.66
19605.23	73.62475	19592.26	73.62471	19593.45	73.63158	19593.47	73.6306	19594.95	73.62812	19593.97	76.19508	19579.34
19604.03	73.87697	19592.54	73.87344	19593.03	73.87955	19594	73.87915	19592.7	73.87912	19593.95	76.44297	19580.55
19602.43	74.12948	19591.36	74.12632	19593.67	74.1296	19593.25	74.12995	19593.18	74.12796	19592.79	76.69124	19580.81
19603.34	74.37475	19590.88	74.37418	19593.27	74.3779	19591.85	74.38715	19592.26	74.37664	19594.73	76.94542	19577.87
19603.77	74.62202	19590.92	74.62326	19592.23	74.63019	19590.9	74.63084	19591.07	74.62814	19592.81	77.19464	19579.58
19603.39	74.87568	19591.66	74.87402	19593.02	74.8798	19592.11	74.87904	19591.93	74.8789	19594.24	77.44493	19578.52
19604.86	75.12982	19591.8	75.12858	19592.74	75.12939	19593.3	75.13011	19593.16	75.12963	19592.7	77.69102	19580.29
19604.59	75.37611	19590.85	75.37485	19593.54	75.37971	19594.02	75.37656	19594.02	75.37737	19593.58	77.94522	19578.89
19603.33	75.62361	19591	75.62437	19593.45	75.63102	19593.3	75.63033	19591.76	75.62964	19594.19	78.19559	19578.91
19603.53	75.87799	19591.44	75.8725	19591.52	75.88044	19592.98	75.8782	19593.5	75.87943	19595.53	78.44356	19578.61
19604.13	76.12951	19592.33	76.12751	19592.81	76.13242	19592.3	76.13127	19593.31	76.12866	19596.82	78.69069	19580.29
19604.14	76.37717	19592.3	76.37546	19592.54	76.37935	19593.96	76.37957	19592.7	76.37582	19595.85	78.95796	19580.11
19604.13	76.62488	19592.41	76.62571	19591.9	76.63122	19592.06	76.6329	19592.52	76.62877	19591.58	79.19601	19578.2
19602.88	76.87647	19592.77	76.87458	19594.56	76.87976	19592.29	76.87932	19592.72	76.87878	19595.5	79.4458	19578.47
19604.77	77.1308	19590.37	77.12887	19593.47	77.13412	19593.06	77.13209	19592.67	77.13035	19594.76	79.69	19577.78
19604.44	77.37654	19591.46	77.37487	19591.19	77.37997	19593	77.37937	19593.34	77.38117	19593.51	79.94812	19577.44
19604.09	77.62577	19590.92	77.62494	19591.91	77.63404	19593.46	77.6327	19593.53	77.63012	19593.41	80.19625	19579.1
19603.66	77.8774	19590.65	77.87514	19590.75	77.88004	19595.37	77.88039	19591.77	77.88113	19594.97	80.44944	19579.83
19603.76	78.12946	19591.96	78.12653	19593.8	78.13216	19593.85	78.13149	19592.49	78.12971	19592.82	80.69151	19578.68
19604.1	78.37567	19589.94	78.37492	19594.87	78.38199	19592.19	78.3807	19593.74	78.37993	19593.63	80.94609	19579.35
19602.73	78.62693	19591.6	78.62465	19593.61	78.63372	19593.9	78.63525	19590.75	78.63223	19595.1	81.19476	19578.63
19604.86	78.87776	19592.46	78.87759	19592.26	78.88166	19593	78.8799	19591.84	78.87781	19594.74	81.44748	19579.09
19604.28	79.13119	19589.33	79.12948	19591.99	79.13445	19593.33	79.12998	19593.01	79.12836	19594.06	81.69263	19580.01
19603.32	79.37587	19589.6	79.3851	19591.58	79.37922	19592.17	79.3898	19590.28	79.37976	19593.6	81.947	19579.5
19603.37	79.6257	19591.22	79.62896	19593.51	79.63174	19593.77	79.64187	19593.26	79.63163	19593.29	82.19302	19578.62
19603.79	79.87854	19591.03	79.87509	19592.43	79.88163	19594.12	79.88033	19593.3	79.88177	19593.37	82.44476	19579.39
19605.18	80.13353	19589.18	80.12999	19592.42	80.13412	19594.36	80.13108	19592.18	80.12958	19595.06	82.69332	19579.2
19604.63	80.38115	19588.77	80.37922	19593.83	80.3822	19594.49	80.38123	19592.46	80.38376	19593.17	82.95214	19579.95
19603.24	80.62479	19589.6	80.62657	19592.28	80.63252	19592.21	80.63506	19593.3	80.63117	19594.57	83.19262	19579.01
19603.26	80.87902	19591.53	80.87456	19592.74	80.88156	19591.96	80.88294	19592.15	80.88301	19594.37	83.44866	19579.47
19603.11	81.14677	19590.09	81.12791	19591.76	81.13512	19592.03	81.13278	19592.75	81.13172	19595.5	83.6931	19578.36
19604.1	81.37886	19591.09	81.37547	19590.41	81.38173	19592.9	81.37819	19592.46	81.3789	19592.29	83.94742	19579.2
19604.34	81.62598	19591.32	81.62755	19593.73	81.63336	19591.74	81.63747	19594.07	81.63353	19594.64	84.19516	19579.68
19602.98	81.87765	19589.62	81.87647	19592.64	81.8828	19592.05	81.88114	19591.73	81.88151	19592.43	84.44835	19578.7
19604.94	82.13173	19589.81	82.12847	19591.78	82.13784	19590.32	82.13344	19592.69	82.12993	19592.83	84.69442	19579.18
19603.9	82.379	19588.35	82.37643	19592.6	82.38318	19589.76	82.37955	19592.01	82.38112	19594.1	84.94908	19577.46
19603.79	82.62731	19590.23	82.62685	19591.22	82.63571	19592.82	82.63713	19593.37	82.63328	19593.55	85.19424	19578.29
19602.7	82.87705	19589.01	82.87621	19592.76	82.88204	19592.94	82.88131	19593.1	82.88027	19593.85	85.4487	19578.28
19602.75	83.13271	19591.68	83.12923	19591.59	83.13279	19592.03	83.13448	19593.52	83.13065	19593.9	85.69485	19577.42
19603.05	83.38029	19590.52	83.37768	19593.27	83.38459	19591.9	83.38133	19592.29	83.38146	19595.53	85.95788	19577.51
19603.98	83.62878	19589.43	83.62729	19594.5	83.63494	19591.26	83.63799	19593.05	83.63428	19594.05	86.19639	19578.74
19603.17	83.87993	19592.32	83.87559	19591.31	83.88497	19592.74	83.88142	19591.45	83.8926	19593.56	86.44907	19578.65
19604.16	84.1333	19590.82	84.13231	19592.49	84.13448	19593.05	84.13363	19594.05	84.12995	19592.44	86.6944	19579.77
19604.53	84.38007	19589.27	84.37896	19593.1	84.38531	19592.87	84.38211	19592.65	84.38189	19592.91	86.94842	19580.25
19602.57	84.62868	19589.27	84.62731	19590.44	84.63634	19591.48	84.63617	19590.64	84.63429	19594.05	87.19874	19580.92
19603.37	84.88125	19590.21	84.87624	19590.86	84.88233	19591.6	84.88228	19591.47	84.88403	19594.47	87.44733	19576.84
19603.9	85.13244	19590.04	85.13075	19591.11	85.1344	19592.38	85.13488	19592.65	85.13094	19593.31	87.69431	19578.58
19603.35	85.38011	19590.63	85.37944	19592.8	85.38206	19591.28	85.38115	19593.52	85.3814	19593.42	87.94944	19579.08
19603.59	85.62699	19590.52	85.62891	19591.62	85.63522	19591.8	85.63844	19592.79	85.63379	19591.81	88.19879	19578.98
19603.25	85.88249	19589.76	85.8756	19592.03	85.88555	19591.92	85.88247	19590.83	85.88449	19594.73	88.44825	19578.16
19603.02	86.13404	19590.79	86.1303	19591.76	86.13622	19590.95	86.13426	19592.92	86.13212	19591.83	88.69309	19578.36

19603.72	86.38083	19591.43	86.37785	19591.75	86.38302	19591.86	86.38142	19592.83	86.38316	19594.46	88.97149	19577.73
19603.64	86.62777	19589	86.62949	19591.74	86.63441	19591.87	86.63926	19591.39	86.63647	19591.31	89.19938	19579.14
19603.08	86.87911	19590.02	86.87793	19592.53	86.88409	19591.14	86.88287	19591.96	86.88399	19592.55	89.45182	19578.71
19602.7	87.13528	19589.12	87.12952	19589.86	87.13938	19593.39	87.13603	19593.05	87.13417	19594.72	89.69539	19578.82
19602.01	87.38116	19589.87	87.37907	19591.42	87.38394	19593.28	87.38358	19593.07	87.38084	19592.7	89.94813	19577.54
19602.9	87.62914	19590.3	87.63186	19592.19	87.63524	19592.1	87.63953	19591.65	87.63194	19595.56	90.20143	19579.03
19602.74	87.87937	19590.21	87.87815	19590.86	87.88382	19591.98	87.8828	19594.47	87.8841	19592.78	90.45015	19579.24
19601.58	88.13431	19589.21	88.13316	19591.69	88.13612	19592.89	88.13609	19591.02	88.13372	19595.27	90.69584	19577.33
19603.42	88.38104	19590.12	88.37949	19591.68	88.38579	19591.15	88.38421	19591.23	88.38247	19592.41	90.9501	19576.17
19603.27	88.62976	19588.51	88.62862	19592.01	88.63645	19589.85	88.64021	19590.87	88.63387	19591.96	91.19563	19576.11
19603.66	88.88188	19591.33	88.87816	19590.66	88.88325	19591.69	88.8814	19591.91	88.88598	19593.19	91.45082	19578.24
19602.51	89.1349	19588.21	89.133	19590.44	89.13641	19592.61	89.13553	19591.27	89.13376	19592.08	91.69728	19577.17
19601.89	89.38103	19587.7	89.38095	19591.8	89.38368	19590.32	89.38467	19591.73	89.38291	19594.72	91.95166	19577.96
19604.17	89.62855	19588.98	89.62895	19591.42	89.63651	19590.81	89.63768	19591.39	89.63423	19591.46	92.19611	19578.36
19602.63	89.8825	19588.8	89.87762	19589.83	89.88544	19591.33	89.88356	19592.16	89.88273	19592.31	92.44989	19578
19605.76	90.13559	19589.38	90.13163	19589.8	90.13624	19592.54	90.13595	19592.99	90.13354	19591.13	92.69655	19578.65
19603.69	90.38237	19588.63	90.38134	19592.32	90.38356	19590.58	90.38315	19590.45	90.38309	19592.36	92.95328	19577.61
19602.82	90.62917	19590.08	90.63288	19590.71	90.63681	19591.58	90.63846	19591.42	90.63584	19592.27	93.1986	19578.98
19602.65	90.8846	19588.7	90.87907	19590.97	90.88577	19591.18	90.88688	19589.06	90.8838	19592.51	93.45062	19577.49
19601.63	91.13665	19590.1	91.1323	19589.74	91.13686	19591.37	91.13642	19592.35	91.13343	19592.16	93.69649	19576.93
19601.7	91.3839	19590.5	91.38191	19590.42	91.38436	19591.43	91.38311	19589.87	91.40588	19591.54	93.95025	19575.05
19601.49	91.6292	19590.4	91.63243	19591.48	91.63677	19589.44	91.64153	19591.98	91.63462	19592.53	94.20198	19575.51
19602.87	91.88	19588.74	91.87944	19590.74	91.88727	19591.61	91.8845	19591.23	91.8864	19592.62	94.45128	19576.29
19601.67	92.13797	19588.76	92.13297	19592.38	92.14097	19591.69	92.13997	19591.66	92.13467	19591.36	94.6951	19577.44
19602.76	92.38462	19587.47	92.38183	19589.72	92.38494	19591.68	92.38435	19590.29	92.38336	19593.84	94.95219	19575.96
19602.15	92.63041	19587.84	92.63194	19587.93	92.63827	19591.7	92.6411	19591.18	92.63443	19591.39	95.20379	19578.14
19602.67	92.88035	19589.1	92.87979	19591.99	92.88697	19589.88	92.88411	19592.83	92.88624	19591.49	95.45265	19576.17
19601.75	93.13615	19590.25	93.13384	19589.54	93.13812	19590.93	93.13851	19591.23	93.1362	19590.93	95.69844	19574.99
19601.36	93.38271	19590.1	93.38159	19590.72	93.38468	19590.46	93.38654	19590.59	93.38344	19591.95	95.95072	19576.71
19602.36	93.63284	19588.15	93.63202	19590.79	93.63734	19589.45	93.64226	19591.87	93.63539	19592.23	96.20143	19577.57
19601.19	93.88467	19588.13	93.88001	19590.7	93.88756	19590.91	93.90037	19590.5	93.88667	19592.62	96.45117	19576.98
19603.19	94.13564	19587.27	94.13642	19589.94	94.13812	19590.91	94.13943	19592.28	94.13677	19591.02	96.69858	19577.93
19602.29	94.38248	19589	94.38178	19590.34	94.38626	19591.41	94.3864	19591.96	94.38612	19593	96.95409	19576.39
19602.38	94.63045	19588.54	94.6305	19590.54	94.64064	19590.48	94.643	19591.68	94.63488	19592.01	97.1981	19578.62
19602.11	94.88295	19588.45	94.8819	19589.45	94.88696	19590.81	94.8868	19590.16	94.88683	19591.9	97.45162	19578.3
19600.86	95.13764	19589.49	95.13838	19589.24	95.13848	19590.29	95.13799	19591.17	95.1356	19590.62	97.69822	19576.71
19602.29	95.38319	19587.52	95.38324	19589.8	95.38639	19590.27	95.38576	19589.85	95.38592	19592.58	97.95299	19576.69
19602.54	95.63159	19588.43	95.63203	19590.5	95.64059	19588.61	95.66756	19590.09	95.63434	19590.17	98.2048	19575.82
19601.62	95.88555	19587.19	95.87978	19590.21	95.8891	19589.53	95.89327	19592.35	95.88721	19589.38	98.45319	19575.44
19603.01	96.13914	19587.91	96.14033	19591.69	96.13803	19589.28	96.13926	19590.94	96.13573	19592.59	98.69822	19577.86
19600.44	96.38527	19587.52	96.38596	19588.15	96.38538	19591.26	96.38623	19590.74	96.38672	19592.47	98.95205	19575.5
19599.92	96.63255	19589.42	96.6316	19589.77	96.64042	19589.45	96.64356	19591.37	96.63733	19590.95	99.20434	19576.91
19601.72	96.88297	19588.15	96.87906	19589.97	96.88894	19591.96	96.88703	19589.69	96.88828	19590.61	99.45448	19575.39
19601.54	97.13829	19588.23	97.13806	19589.42	97.14363	19588.74	97.13974	19591.45	97.13725	19591.8	99.69803	19575.31
19601.03	97.3847	19588.39	97.38486	19589.77	97.38643	19589.47	97.38696	19591.04	97.39209	19590.19	99.95607	19574.93
19601.12	97.63172	19587.63	97.63456	19588.97	97.63952	19590.36	97.6448	19590.13	97.63914	19591.85	100.2069	19577.79
19600.08	97.88359	19587.43	97.88317	19589.67	97.88787	19589.46	97.88515	19589.56	97.89094	19589.46	100.4571	19575.13
19600.2	98.13712	19587.14	98.13799	19588.09	98.13998	19588.93	98.13997	19590.16	98.13612	19590.48	100.7028	19574.88
19600.97	98.3832	19588.63	98.38363	19588.54	98.3871	19590.47	98.38717	19588.98	98.38359	19589.7	100.9559	19576.89
19600.63	98.63492	19586.66	98.63483	19589.33	98.64518	19589.4	98.64451	19590.04	98.6382	19590.19	101.2077	19578.65
19602.08	98.88628	19585.87	98.88324	19587.78	98.88705	19590.51	98.88684	19589.21	98.88836	19589.08	101.4707	19573.72
19600.18	99.14309	19588.58	99.14399	19589.28	99.14319	19589.99	99.14566	19589.56	99.1426	19590.18	101.703	19577.14
19600.84	99.40504	19587.46	99.38325	19588.82	99.38701	19588.09	99.38569	19589.94	99.38491	19589.73	101.9578	19575.79
19600.69	99.6345	19587.79	99.63529	19587.56	99.64145	19589.25	99.64612	19590.51	99.63615	19591.36	102.2063	19573.56
19601.63	99.88605	19587.29	99.88316	19588.24	99.89397	19588.89	99.88715	19590.18	99.88922	19590.03	102.4592	19574.77
19601.22	100.137	19587.71	100.1403	19586.36	100.14	19588.66	100.1416	19587.7	100.1386	19589.94	102.703	19574.24
19600.94	100.3866	19588.08	100.387	19588.16	100.3889	19590.47	100.3899	19587.89	100.3906	19590.36	102.9583	19574.74
19600.13	100.6341	19587.03	100.6347	19589.48	100.6429	19588.58	100.6462	19591.55	100.6396	19590.33	103.2061	19575.95
19600.15	100.8885	19587.6	100.885	19588.08	100.8925	19587.48	100.8897	19589.53	100.8919	19589.61	103.4571	19575.8
19600.61	101.1414	19586.15	101.1428	19589.74	101.1426	19588.97	101.1436	19588.86	101.1392	19588.91	103.7024	19575.09
19601.21	101.388	19587.32	101.3888	19589.01	101.39	19588.96	101.3908	19590.03	101.3905	19589.5	103.9585	19574.27
19600.98	101.6363	19587.27	101.636	19587.89	101.6434	19588.44	101.6486	19587.25	101.6403	19589.52	104.2094	19575.61
19599.29	101.8877	19586.56	101.8848	19587.63	101.8932	19587.33	101.8886	19590.57	101.8913	19590.14	104.4584	19575.48
19599.73	102.1453	19585.92	102.1486	19586.75	102.1464	19589.7	102.1437	19588.13	102.1388	19589.17	104.7031	19574.62
19599.17	102.3883	19586.54	102.3908	19586.56	102.389	19587.49	102.3907	19589.85	102.3897	19590.6	104.9589	19575.08
19599.77	102.6386	19586.74	102.6377	19588.56	102.6428	19589.73	102.6494	19590.27	102.6422	19590.47	105.2131	19574.77
19601.54	102.8884	19585.7	102.8857	19588.29	102.892	19588.46	102.8914	19589.6	102.8956	19590.31	105.4597	19576.52
19597.96	103.1418	19587.06	103.1424	19588.22	103.145	19588.61	103.143	19588.42	103.1397	19588.25	105.7038	19574.5
19600.24	103.3879	19586.8	103.3866	19587.07	103.3924	19588.64	103.3882	19586.75	103.3909	19590.39	105.9583	19574.48
19600.16	103.6383	19586.25	103.6376	19587.35	103.6444	19588.77	103.6482	19587.67	103.6423	19588.56	106.2083	19575.07

19599.69	103.8906	19584.87	103.8869	19587.13	103.8928	19587.87	103.8919	19588.45	103.8946	19590.94	106.4594	19574.79
19598.53	104.1469	19586.59	104.1435	19587.05	104.1433	19587.63	104.1448	19587.31	104.1429	19589.58	106.7045	19573.28
19599.9	104.3935	19586.35	104.3871	19587.83	104.3918	19587.21	104.3901	19588.29	104.3892	19589.95	106.9599	19575.7
19598.52	104.6366	19586.64	104.6376	19586.24	104.6501	19586.66	104.6476	19588.88	104.6416	19589.28	107.2093	19575.28
19598.75	104.889	19584.85	104.8877	19585.86	104.8929	19588.3	104.8918	19587.87	104.8954	19588.73	107.458	19576.34
19597.58	105.1428	19584.71	105.1438	19587.88	105.1455	19587.16	105.1461	19589.62	105.1429	19588.98	107.7046	19575.79
19596.72	105.3882	19586.3	105.3909	19587.27	105.3917	19586.38	105.3914	19587.13	105.3921	19589.1	107.9603	19574.62
19600.97	105.6364	19585.98	105.6388	19588.12	105.6454	19588.98	105.6478	19587.07	105.642	19588.02	108.2063	19574.14
19597.7	105.8914	19583.96	105.8864	19587.23	105.8945	19587.56	105.8916	19587.56	105.8968	19588.89	108.4588	19574.73
19599.39	106.1429	19585.01	106.146	19587.9	106.1471	19586	106.1452	19589.06	106.1425	19588.15	108.705	19575.52
19599.62	106.3915	19586.14	106.3894	19587.08	106.3913	19587.65	106.3924	19588.2	106.3933	19589.65	108.9621	19574.1
19598.82	106.6374	19587.44	106.6384	19587.25	106.6451	19588.03	106.6477	19589.63	106.6452	19588.73	109.2078	19576.18
19598.92	106.8899	19586.48	106.8855	19586.37	106.8938	19586.98	106.8918	19587.95	106.8947	19588.12	109.4618	19575.01
19598.26	107.1454	19586.66	107.1447	19589.35	107.1487	19588.14	107.1462	19588.8	107.1432	19587.71	109.7174	19572.93
19598.13	107.3908	19585.58	107.3886	19587.28	107.3933	19586.62	107.3932	19589.93	107.3933	19589.87	109.9592	19574.92
19598.8	107.6393	19585.55	107.6408	19584.08	107.6462	19587.53	107.6522	19588.39	107.6447	19588.12	110.2153	19574.13
19598.59	107.8901	19586.37	107.8876	19587.38	107.893	19586.57	107.8926	19586.55	107.8957	19586.45	110.4618	19575
19599.48	108.1453	19585.64	108.1461	19588.62	108.1458	19586.15	108.1454	19588.35	108.1422	19588.6	110.7074	19575.5
19598.42	108.3909	19585.31	108.3887	19588.8	108.3977	19586.8	108.3928	19585.87	108.393	19586.48	110.9637	19572.83
19598.74	108.6407	19585.44	108.6406	19587.24	108.6463	19584.72	108.6498	19588.03	108.6436	19587.4	111.2081	19571.69
19599.01	108.892	19585.59	108.89	19586.26	108.894	19587.42	108.8937	19586.57	108.8974	19587.92	111.4635	19574.62
19595.03	109.1449	19585.41	109.1503	19587.54	109.1498	19586.96	109.1455	19587.57	109.1429	19588.64	111.7079	19574.23
19599.31	109.3891	19585.13	109.3893	19586.59	109.3952	19587.05	109.3924	19587.18	109.3922	19588.07	111.9615	19571.92
19596.57	109.639	19586.29	109.6435	19586.55	109.6492	19585.96	109.6507	19587.8	109.6423	19588.78	112.2091	19575.22
19597.47	109.891	19584.96	109.8887	19587.02	109.8963	19586.14	109.8938	19588.39	109.8971	19587.65	112.4606	19574.22
19598.22	110.1467	19585.35	110.1469	19586.25	110.146	19587.33	110.1459	19587.07	110.1434	19587.98	112.7073	19574.93
19599.17	110.3905	19585.77	110.3896	19587.07	110.3938	19586.69	110.393	19585.93	110.3943	19587.12	112.9621	19572.34
19600.29	110.6404	19586.75	110.642	19587.87	110.6478	19586.52	110.6497	19586.58	110.6439	19587.72	113.2101	19574.92
19599.07	110.8938	19586.16	110.8886	19586.1	110.8964	19587.08	110.8935	19585.9	110.8975	19586.94	113.4611	19574.3
19597.39	111.1449	19585.04	111.1489	19585.22	111.1484	19587.28	111.1458	19587.27	111.1426	19588.98	113.708	19573.05
19597.4	111.3919	19585.14	111.3905	19586.16	111.3921	19586.25	111.3953	19586.11	111.395	19587.99	113.9631	19573.79
19596.71	111.6398	19586.54	111.641	19587.39	111.6472	19584.21	111.6523	19588.11	111.6466	19586.92	114.2091	19573.1
19596.67	111.8913	19582.85	111.8892	19585.29	111.8957	19586.07	111.8934	19587.6	111.8979	19585.94	114.461	19574.04
19597.33	112.1467	19584.32	112.1465	19586.83	112.1509	19587.3	112.1463	19586.81	112.1447	19587.19	114.7076	19573.47
19598.4	112.3925	19583.19	112.3929	19586.65	112.394	19587.72	112.395	19584.57	112.3939	19585.66	114.9631	19573.04
19597.6	112.6437	19585.71	112.6452	19585.11	112.6491	19585.55	112.6521	19587.01	112.6455	19587.42	115.2101	19573.93
19597.52	112.891	19586.02	112.8898	19586.29	112.8966	19584.53	112.8944	19586.21	112.8999	19586.66	115.463	19572.49
19597.89	113.1464	19584.65	113.1478	19586.81	113.1468	19586.03	113.1454	19587.98	113.146	19587.01	115.7067	19572.98
19597.53	113.392	19585.51	113.3928	19586.01	113.3966	19586.5	113.3943	19588.09	113.3988	19587.64	115.962	19573.69
19598.55	113.6428	19583.31	113.6465	19586.8	113.6493	19584.38	113.6507	19586.4	113.6454	19587.53	116.2146	19573.52
19597.24	113.8929	19584.53	113.892	19587.27	113.8964	19585.27	113.8953	19587.6	113.9	19586.73	116.4627	19572.76
19595.93	114.1478	19582.55	114.1493	19586.91	114.1471	19582.63	114.1476	19585.84	114.1453	19584.74	116.7062	19572.85
19597.13	114.3922	19583.12	114.3946	19584.84	114.3974	19586.38	114.3941	19586.46	114.3985	19586.11	116.9637	19571.95
19598.59	114.6417	19582.69	114.6447	19585.01	114.6477	19584.66	114.6512	19586.6	114.6446	19585.2	117.2128	19573.26
19598.46	114.894	19583.09	114.8952	19585.83	114.8957	19586.39	114.8972	19583.85	114.8982	19588.47	117.4658	19573.44
19598.48	115.1473	19584.89	115.1483	19584.3	115.1486	19585.72	115.1496	19584.18	115.1469	19587.74	117.7093	19571.94
19594.78	115.3912	19583.49	115.3927	19587.61	115.3982	19587.67	115.3952	19587.71	115.3969	19586.39	117.9688	19574.56
19596.18	115.6424	19584.13	115.6454	19585.03	115.649	19586.81	115.6511	19586.95	115.6472	19586.25	118.2128	19572.47
19596.62	115.896	19584.16	115.8909	19583.98	115.8976	19584.55	115.8966	19587.82	115.9007	19588.19	118.4641	19574.62
19597.13	116.1473	19584.41	116.1498	19584.45	116.1493	19586.43	116.1498	19586.08	116.144	19586.59	118.7103	19574.64
19597.94	116.3947	19583.88	116.3929	19586.23	116.3966	19585.04	116.3971	19586.22	116.3967	19585.2	118.9636	19572.84
19598.24	116.6426	19581.63	116.6463	19585.34	116.6506	19585.42	116.6544	19586.37	116.6471	19588.02	119.2138	19572.6
19597.54	116.8942	19583.06	116.8913	19585.44	116.8999	19583.95	116.8984	19584.83	116.8988	19584.85	119.4644	19574.02
19597.25	117.1482	19584.96	117.1572	19583.36	117.1516	19584.95	117.149	19586.68	117.1481	19587.32	119.7101	19573.25
19597.14	117.3942	19583.77	117.3989	19584.82	117.3958	19585.84	117.3972	19585.9	117.397	19586.7	119.9674	19571.81
19596.55	117.6417	19584.49	117.6466	19586.49	117.65	19585.34	117.6582	19583.19	117.648	19586.05	120.2113	19571.39
19597.44	117.8937	19584.55	117.8931	19584.92	117.8994	19585.7	117.8974	19585.71	117.9031	19587.93	120.4651	19573.64
19596.78	118.1485	19585.67	118.1493	19585.26	118.1513	19585.56	118.1487	19584.67	118.1471	19586.88	120.7073	19572.57
19597.29	118.394	19581.65	118.3946	19587.12	118.3962	19585.15	118.3969	19585.48	118.3964	19586.55	120.9622	19571.19
19596.42	118.6448	19584.65	118.6465	19584.59	118.6486	19584.63	118.6548	19585.44	118.6485	19587.33	121.2097	19573.29
19597.82	118.8947	19582.91	118.8925	19585.34	118.9001	19584.24	118.8976	19586.19	118.9013	19588.24	121.4624	19573
19597.8	119.1488	19583.38	119.1486	19586.69	119.1528	19584.36	119.16	19588.53	119.1483	19586.52	121.7081	19573.14
19596.68	119.3938	19584.04	119.3933	19585.07	119.3966	19585.82	119.3969	19585.51	119.3985	19585.37	121.9622	19571.55
19596.3	119.6451	19583.98	119.6476	19583.88	119.6505	19583.82	119.6535	19584.61	119.6472	19584.78	122.2099	19572.5
19596.68	119.8975	19583.76	119.8938	19585.2	119.9019	19584.57	119.8984	19584.33	119.8987	19587.19	122.4639	19571.67
19596.23	120.1473	19583.28	120.1506	19585.92	120.1514	19585.85	120.152	19585.81	120.1478	19585.37	122.7062	19570.96
19596.18	120.3956	19583.35	120.3948	19584.47	120.3987	19584.35	120.3974	19586.31	120.401	19586.62	122.9623	19572.43
19598.11	120.6413	19581.71	120.6436	19585.44	120.6496	19585.19	120.6525	19584.33	120.6469	19584.41	123.2114	19571.84
19596	120.8953	19582.33	120.8906	19585.68	120.8968	19585.75	120.897	19584.34	120.9022	19585.71	123.468	19573.36
19596.36	121.1479	19583.26	121.1496	19584.88	121.1487	19585.86	121.1486	19587	121.1462	19586.67	123.71	19571.4

19596.59	121.3945	19582.91	121.3925	19585.07	121.3967	19584.2	121.3965	19585.91	121.398	19585.04	123.9621	19570.16
19596.89	121.6414	19583.27	121.6427	19585.37	121.65	19584.71	121.6519	19582.95	121.6522	19586.16	124.211	19571.83
19596.67	121.8931	19584.14	121.8912	19583.91	121.8995	19585.97	121.8966	19584.81	121.9055	19584.79	124.465	19574.27
19596.04	122.1474	19583.29	122.1491	19582.05	122.1518	19584.49	122.149	19586.47	122.1465	19584.05	124.7104	19571.14
19595.33	122.3929	19583.3	122.3927	19584.5	122.4007	19586.67	122.3974	19585.43	122.406	19586.32	124.964	19572.72
19597.48	122.6408	19583.51	122.6429	19584.53	122.6492	19583.02	122.6538	19585.89	122.6541	19586.15	125.2138	19573.71
19595.71	122.892	19583.21	122.8898	19584.8	122.9003	19585.37	122.8962	19585.96	122.9058	19585.58	125.4638	19571.79
19595.97	123.147	19582.71	123.1487	19583.79	123.1503	19585.7	123.1497	19584.44	123.1479	19586.82	125.7106	19572.29
19594.57	123.394	19583.43	123.3953	19583.59	123.395	19584.25	123.3981	19585.35	123.3971	19586.63	125.9662	19573.78
19596.83	123.6428	19582.21	123.6449	19585.49	123.6487	19582.36	123.6552	19584.7	123.6481	19585.14	126.2124	19572.38
19595.33	123.8927	19585.19	123.8907	19582.99	123.8992	19585.68	123.8965	19584.14	123.9027	19585.3	126.4659	19572.7
19595.32	124.1465	19584.37	124.1493	19583.05	124.1511	19584.34	124.1504	19585.56	124.1511	19583.64	126.7103	19571.48
19595.81	124.3934	19582.72	124.3939	19583.76	124.3995	19584.42	124.3988	19587.5	124.3962	19585.89	126.9662	19573.01
19595.87	124.6434	19582.51	124.6467	19585.11	124.6494	19583.89	124.6547	19584.63	124.6594	19586.58	127.2214	19570.13
19595.59	124.8956	19582.95	124.8935	19583.99	124.9021	19583.31	124.8958	19585	124.9027	19584.79	127.4708	19571.19
19595.98	125.1473	19580.65	125.1489	19584.93	125.1498	19584.74	125.1495	19586.58	125.15	19585.75	127.7236	19574.38
19594.38	125.3937	19582.87	125.3932	19583.44	125.3988	19584.4	125.4018	19585.16	125.4036	19584.55	128.0076	19570.55
19595.27	125.6422	19585.1	125.6473	19584.18	125.6513	19583.83	125.6555	19584.77	125.6493	19584.87	128.2372	19571.6
19596.75	125.8971	19582.41	125.8925	19583.13	125.9008	19582.37	125.898	19582.58	125.9076	19583.79	128.4813	19573.42
19594.17	126.1472	19583.38	126.1508	19584.33	126.1523	19581.94	126.149	19584.14	126.1504	19582.29	128.7243	19572.6
19595.2	126.3956	19581.44	126.3948	19585.03	126.3983	19585.58	126.3999	19581.96	126.4001	19583.27	128.9778	19572.96
19597.88	126.6419	19582.26	126.6447	19583.97	126.6525	19586.1	126.6549	19585.06	126.65	19586.67	129.227	19571.62
19595.91	126.895	19582.91	126.8921	19584.57	126.9019	19582.97	126.9022	19583.56	126.9053	19585.8	129.4766	19570.86
19596.89	127.1497	19583.08	127.1521	19584.34	127.1548	19583.67	127.1502	19585.74	127.15	19585.32	129.7338	19569.88
19596.15	127.3954	19584.11	127.3954	19584.82	127.3987	19584.29	127.3979	19585.44	127.3978	19584.89	129.981	19570.27
19596.43	127.6451	19581.61	127.6476	19581.93	127.6516	19583.54	127.6586	19585.58	127.6529	19585.34	130.2246	19571.82
19594.69	127.8956	19581.18	127.895	19586.54	127.9164	19580.43	127.8981	19583.17	127.906	19584.75	130.475	19571.1
19595.4	128.1504	19581.16	128.151	19584.69	128.1577	19583.48	128.1504	19582.12	128.1491	19586.09	130.724	19571.56
19594.15	128.3951	19582.61	128.3974	19584.19	128.3987	19584.89	128.3989	19584.37	128.4	19586.19	130.9783	19572.98
19597.14	128.6457	19581.39	128.6466	19584.06	128.652	19585.08	128.6568	19585.95	128.6506	19585.12	131.2266	19568.78
19592.51	128.8971	19581.02	128.8947	19582.27	128.9018	19583.4	128.8992	19584.55	128.9078	19584.67	131.4668	19569.93
19595.32	129.1487	19581.9	129.1532	19584.57	129.1531	19583.21	129.1541	19584.92	129.1487	19586.32	131.7121	19574.43
19595.79	129.3959	19582.6	129.3949	19582.56	129.4007	19581.9	129.3989	19584.24	129.3984	19584.35	131.9679	19571.04
19595.71	129.6448	19582.81	129.647	19582.97	129.6533	19584.06	129.656	19583.86	129.6505	19582.93	132.2128	19570.24
19596.39	129.8984	19582.26	129.8944	19583.58	129.9024	19581.75	129.899	19583.69	129.9064	19584.6	132.4667	19572.15
19596.25	130.1491	19582.61	130.1535	19583.01	130.1506	19584.72	130.1516	19583.93	130.1506	19586.05	132.7129	19571.9
19594.32	130.3969	19582.54	130.3957	19581.86	130.4003	19584.29	130.3986	19581.75	130.3984	19586.51	132.9683	19570.32
19595.96	130.6441	19583.65	130.6476	19582.21	130.6541	19583.11	130.6581	19582.2	130.6497	19583.77	133.2142	19570.26
19593.29	130.9	19581.1	130.895	19583.25	130.9007	19584.06	130.8998	19584.84	130.9089	19584.96	133.4658	19568.14
19595.85	131.1517	19583.17	131.1532	19584.14	131.1523	19582.92	131.1527	19584.61	131.1537	19583.11	133.7131	19571.19
19594.74	131.3982	19580.98	131.3951	19582.73	131.4006	19582.31	131.3999	19581.17	131.4001	19584.48	133.967	19569.43
19596.02	131.6441	19580.78	131.6485	19583.79	131.6551	19583.67	131.6582	19584.81	131.6499	19586.03	134.2128	19570.77
19594.79	131.8987	19581.78	131.8956	19583.43	131.9028	19583.68	131.8982	19583.61	131.9074	19584.46	134.4679	19570.89
19596.5	132.154	19581.55	132.1535	19583.65	132.1547	19583.8	132.1525	19584.65	132.1526	19583.21	134.7135	19571.46
19594.09	132.3983	19580.41	132.3973	19583.82	132.4	19581.33	132.4009	19582.6	132.4018	19580.77	134.9691	19570.84
19593.64	132.6475	19581.76	132.6478	19582.84	132.6548	19582.21	132.6587	19583.35	132.6517	19584.27	135.2154	19570.96
19595.41	132.8997	19581.84	132.8942	19580.25	132.9045	19582.75	132.902	19582.04	132.9041	19585.35	135.4686	19570.19
19594.37	133.1502	19583.11	133.1555	19581.88	133.1555	19583.45	133.154	19584.67	133.1505	19585.52	135.7133	19570.09
19594.38	133.3982	19581.97	133.3956	19580.45	133.4002	19582.97	133.4039	19582.41	133.4033	19583.34	135.9673	19571.01
19595.62	133.648	19582	133.6485	19584.8	133.6546	19582.96	133.6595	19582.36	133.6523	19583.43	136.2164	19570.1
19595.25	133.9021	19581.28	133.8968	19582.24	133.9033	19583.78	133.901	19583.61	133.9091	19583.03	136.4679	19571.16
19594.26	134.1513	19582.2	134.154	19580.93	134.1541	19582.86	134.1544	19582.48	134.1496	19584.59	136.7133	19570.41
19594.97	134.398	19581.17	134.3961	19580.31	134.4026	19584.15	134.3995	19582.72	134.4006	19584.96	136.9676	19571.42
19595.74	134.6476	19583.09	134.6479	19582.26	134.6549	19583.2	134.6581	19583.28	134.6523	19582.39	137.2157	19570.62
19594.41	134.9011	19580.02	134.8974	19581.95	134.903	19581.83	134.9015	19583.14	134.9089	19583.56	137.4688	19570.51
19593.56	135.1487	19582.15	135.1555	19584.37	135.1544	19582.96	135.1553	19580.28	135.1508	19583.08	137.7139	19571.47
19594.92	135.3991	19582.02	135.4	19581.83	135.4016	19584.3	135.4017	19581.41	135.3993	19583.92	137.9696	19571.68
19594	135.6484	19581.49	135.6482	19581.86	135.6561	19580.68	135.6618	19581.92	135.6505	19585.81	138.2155	19571.2
19595.14	135.903	19581.78	135.8984	19582.02	135.9028	19582.15	135.9017	19583.02	135.9075	19584.41	138.4685	19571.74
19592.39	136.1489	19581.28	136.1554	19581.26	136.1575	19583.26	136.1542	19581.68	136.151	19581.88	138.717	19567.82
19595.56	136.3977	19582.99	136.3987	19582.21	136.4023	19583.65	136.4019	19583.3	136.4024	19584.94	138.9691	19569.1
19591.46	136.6491	19581.74	136.6483	19581.83	136.6551	19583.74	136.6597	19581.21	136.6515	19584.76	139.2155	19573.25
19594.7	136.9033	19581.61	136.8983	19582.3	136.9017	19583.87	136.9019	19585.25	136.9108	19581.31	139.4682	19570.69
19594.73	137.1542	19581.96	137.1546	19583.17	137.1585	19583.32	137.1575	19583.28	137.1523	19584.66	139.7154	19570.47
19594.57	137.3983	19581.18	137.3996	19582.41	137.4026	19582.92	137.4019	19582.35	137.404	19584.21	139.9715	19570.82
19594.91	137.6471	19581.86	137.6499	19580.75	137.657	19583.9	137.6613	19583.29	137.6535	19582.64	140.2166	19569.8
19594.78	137.9007	19580.61	137.8994	19580.65	137.9046	19581.79	137.9	19581.32	137.9081	19584.74	140.4694	19571.27
19592.9	138.1526	19578.57	138.1567	19581.12	138.1558	19582.75	138.1565	19583.99	138.151	19584.36	140.7158	19571.23
19593.23	138.4008	19581.2	138.398	19580.8	138.4017	19581.64	138.4023	19583.17	138.4037	19584.3	140.971	19571.36
19597.02	138.6486	19579.36	138.6514	19581.78	138.6571	19583.46	138.661	19581.91	138.6543	19584.24	141.2179	19570.75

19593.3	138.8996	19580.61	138.9002	19581.42	138.9054	19581.86	138.9015	19582.16	138.908	19583.2	141.4703	19570.38
19595.2	139.1535	19580.24	139.159	19580.99	139.1569	19584.13	139.1536	19583.44	139.1534	19581.47	141.7194	19570.42
19592.51	139.3999	19581.64	139.3979	19580.22	139.4022	19583.62	139.4017	19581.32	139.4035	19582.41	141.9699	19572.06
19597.35	139.6489	19580.13	139.6663	19583.3	139.6551	19583	139.6601	19581.47	139.6552	19584.11	142.2178	19571.42
19593.56	139.9014	19583.11	139.9031	19583.64	139.9061	19580.54	139.9053	19583.92	139.9098	19585.35	142.4712	19568.81
19594.89	140.1521	19580.99	140.1577	19580.37	140.1584	19581.16	140.1559	19583.39	140.1531	19585.14	142.7177	19569.4
19594.18	140.4045	19580.27	140.4002	19580.84	140.4036	19585.94	140.4025	19583.91	140.4061	19584.79	142.9724	19572.03
19594.97	140.6543	19580.58	140.6505	19582.98	140.656	19582.15	140.6633	19582.2	140.6534	19583.82	143.2172	19569.8
19595.92	140.9094	19579.68	140.9	19580.9	140.9048	19582.64	140.9047	19580.66	140.9088	19583.79	143.4711	19570.92
19592.54	141.1534	19580.62	141.1586	19584.37	141.1584	19581.66	141.1547	19582.57	141.1535	19582.41	143.7183	19571.26
19594.79	141.3999	19578.03	141.3991	19582.95	141.4053	19583.13	141.4035	19583.4	141.4053	19584.75	143.9716	19569.29
19594.58	141.6503	19580.4	141.6512	19581.75	141.6562	19584.37	141.6622	19583.32	141.6552	19583.55	144.2184	19568.49
19594.81	141.9017	19581.69	141.8994	19581.86	141.9056	19583.21	141.9056	19579.87	141.9096	19583.25	144.4706	19569.86
19593.26	142.155	19579.24	142.1584	19584.21	142.1602	19583.03	142.1564	19581.56	142.1531	19584.14	144.7198	19567.67
19593.79	142.4001	19581.08	142.4024	19582.95	142.4056	19582.15	142.4035	19582.65	142.4078	19583.82	144.9792	19572.17
19593.85	142.648	19578.73	142.6509	19581.79	142.658	19582.38	142.661	19583.34	142.6557	19581.82	145.2183	19570.67
19595.63	142.9022	19579.99	142.9003	19578.76	142.9055	19582.14	142.9038	19582.86	142.9102	19581.59	145.4706	19571.96
19593.19	143.1564	19578.86	143.1583	19580.4	143.1597	19581.19	143.1585	19582.63	143.1536	19585.18	145.7195	19569.53
19593.03	143.4056	19578.28	143.4033	19581.95	143.4056	19579.99	143.4042	19582.78	143.4058	19582.87	145.9747	19569.62
19593	143.6498	19579.83	143.6528	19582.42	143.6586	19581.47	143.6634	19583.16	143.6551	19578.77	146.2202	19569.68
19593.57	143.9013	19581.79	143.902	19582.52	143.9066	19582.49	143.9027	19582.1	143.9138	19583.22	146.474	19571.65
19592.8	144.1566	19580.8	144.1608	19578.26	144.1569	19580.33	144.1564	19582.08	144.1557	19584.49	146.7186	19569.88
19592.5	144.4051	19580.39	144.3998	19579.21	144.4058	19579.56	144.4049	19581.6	144.4074	19582.29	146.9722	19569.01
19593.76	144.6531	19578.28	144.6536	19581.19	144.6586	19581.05	144.6653	19581.21	144.6582	19582.27	147.2202	19571.38
19595.48	144.9038	19580.66	144.9015	19582.05	144.9084	19582.72	144.9048	19579.96	144.911	19584.43	147.4736	19570.93
19593.83	145.1581	19579.58	145.1613	19581.16	145.1594	19581.79	145.1572	19581.79	145.1546	19582.43	147.7201	19570.61
19593.8	145.4023	19579.96	145.4015	19580.29	145.4041	19581.93	145.4041	19583.63	145.4076	19582.13	147.9737	19568.83
19592.91	145.6509	19581.28	145.6528	19580.91	145.6566	19581.44	145.6635	19580.91	145.6548	19582.98	148.2191	19569.16
19594.7	145.906	19579.42	145.9015	19581.33	145.9107	19579.85	145.9051	19581.12	145.9118	19583.4	148.4726	19568.9
19593.46	146.1574	19580.55	146.159	19580.52	146.1603	19582.45	146.1596	19581.98	146.1547	19584.04	148.7192	19567.57
19593.02	146.4148	19579.06	146.4028	19580.73	146.4088	19581.67	146.4053	19581.31	146.4086	19583.01	148.9732	19570.12
19593.09	146.6509	19581.13	146.654	19579.7	146.6567	19580.49	146.664	19582.89	146.6563	19584.26	149.2252	19570.53
19593.66	146.9031	19580.74	146.8999	19582.79	146.9076	19582.05	146.9083	19581.41	146.915	19582.49	149.4753	19568.77
19593.75	147.1592	19579.11	147.1598	19580.31	147.1615	19581.6	147.1592	19582.19	147.1557	19584.65	149.7227	19571.31
19591.26	147.4046	19578.2	147.4023	19582.54	147.4122	19580.29	147.405	19580.58	147.4081	19582.13	149.9764	19569.66
19594.73	147.6516	19578.62	147.655	19580.74	147.6578	19580.96	147.6656	19581.23	147.6588	19583.68	150.2311	19569.79
19593.17	147.905	19579.04	147.9028	19582.02	147.9082	19582.84	147.9064	19580.19	147.9133	19584.22	150.4803	19568.96
19593.52	148.1559	19579.91	148.1619	19582.65	148.16	19582.5	148.1592	19580.88	148.1577	19582.23	150.7217	19570.12
19594.05	148.4061	19580.27	148.4033	19580.05	148.4069	19580.39	148.4066	19580.55	148.4089	19583.65	150.9777	19570.1
19592.65	148.6516	19582.67	148.6537	19579.9	148.6609	19580.91	148.667	19582.59	148.6581	19581.88	151.2242	19570.89
19594.59	148.9033	19578.82	148.9036	19581.15	148.9072	19580.43	148.9061	19582.07	148.9176	19583.01	151.4784	19569.59
19593.19	149.1566	19579.37	149.1604	19579.27	149.1604	19581.16	149.1603	19580.95	149.1566	19583.12	151.7241	19570.01
19594.96	149.4054	19580.26	149.4064	19581.45	149.4066	19580.59	149.4072	19582.8	149.4098	19582.46	151.9784	19568.93
19591.28	149.6541	19577.59	149.6557	19579.5	149.6604	19583.16	149.6658	19582.91	149.6572	19584.23	152.2265	19570.72
19592.01	149.9065	19579.07	149.9035	19580.07	149.9106	19580.57	149.908	19580.26	149.9141	19580.8	152.4787	19570.81
19593.72	150.1557	19579.06	150.1616	19582.43	150.1607	19579.8	150.1602	19581.05	150.1571	19581.72	152.7222	19570.81
19592.71	150.4072	19578.4	150.4058	19580.14	150.4098	19584.42	150.4101	19582.75	150.4123	19580.7	152.979	19569.37
19592	150.6593	19579.98	150.6586	19582	150.6626	19579.29	150.6691	19581.28	150.6617	19582.15	153.2271	19567.69
19590.65	150.9114	19581.05	150.9053	19579.15	150.9123	19580.91	150.9111	19581.28	150.9164	19581.82	153.4778	19567.25
19592.57	151.1593	19580.24	151.1643	19582.25	151.1645	19580.56	151.1643	19581.34	151.1842	19582.5	153.7229	19570.08
19593.25	151.409	19580.49	151.4075	19582	151.4111	19584.21	151.4091	19580.81	151.414	19581.9	153.9773	19568.63
19592.03	151.6557	19578.11	151.6603	19580.18	151.6639	19581.01	151.6685	19580.08	151.676	19583.66	154.2267	19568.56
19593.13	151.9091	19581.26	151.9075	19580.59	151.9124	19578.77	151.91	19582.31	151.917	19580.03	154.4802	19571.08
19593.43	152.1635	19579.75	152.1646	19579.05	152.1681	19579.55	152.1634	19580.06	152.1594	19583.24	154.7243	19568.53
19591.5	152.4072	19579.32	152.4078	19579.89	152.412	19579.37	152.4101	19580.68	152.4149	19580.73	154.9785	19567.63
19592.81	152.6561	19578.81	152.6578	19580.7	152.6638	19581.73	152.6697	19582.09	152.6635	19583.48	155.2274	19569.62
19591.96	152.9097	19578.5	152.9054	19579.52	152.9114	19582.47	152.9114	19581.6	152.9189	19583.62	155.4807	19570.92
19591.41	153.1619	19580	153.1659	19579.4	153.1642	19581.77	153.1638	19580.56	153.1609	19584.29	155.7262	19570.41
19593.71	153.4097	19579.19	153.4093	19578.84	153.4114	19581.04	153.4114	19580.1	153.4137	19581.9	155.9804	19570.33
19594.38	153.655	19578.87	153.6601	19581.02	153.6648	19580.69	153.6707	19582.75	153.6641	19582.76	156.2269	19569.92
19591.34	153.909	19579.77	153.9069	19582.13	153.9124	19581.49	153.9117	19580.58	153.9197	19581.7	156.4808	19571.1
19591.15	154.1613	19577.86	154.1671	19583.22	154.1629	19580.44	154.1862	19581.53	154.1601	19582.4	156.7255	19569.56
19593.55	154.4108	19580.13	154.4092	19581.1	154.4128	19581.67	154.412	19578.39	154.4136	19581.12	156.9798	19567.62
19593.89	154.6589	19581.2	154.6637	19580.75	154.667	19581.09	154.6717	19578.95	154.6651	19580.31	157.2278	19569.28
19591.22	154.9092	19580.5	154.9077	19579.52	154.9146	19581.29	154.9119	19582.91	154.9204	19582.2	157.4803	19569.07
19591.34	155.1649	19580.49	155.1682	19580.85	155.1645	19581.41	155.1655	19580.36	155.1617	19580.95	157.7257	19568.05
19594.31	155.4125	19579.4	155.4089	19580.25	155.4145	19580.55	155.4147	19581.24	155.415	19581.47	157.9822	19572.11
19591.62	155.6599	19579	155.6615	19580.6	155.6655	19580.81	155.6713	19580.19	155.6644	19582.27	158.2272	19568.89
19592.76	155.9141	19578.96	155.9075	19580.17	155.9156	19578.39	155.9135	19582.24	155.9253	19581.37	158.4782	19570.43
19594.54	156.1643	19582.23	156.168	19578.59	156.1667	19579.89	156.1672	19583.12	156.1696	19582.1	158.7263	19567.67

19592.87	156.4095	19579.33	156.4102	19580.37	156.412	19579.26	156.4141	19580.63	156.4163	19583.17	158.9819	19566.71
19592.58	156.6604	19580.74	156.6621	19581.3	156.6647	19578.37	156.6717	19578.97	156.6643	19583.93	159.2283	19569.99
19592.93	156.9128	19578.78	156.9087	19580.65	156.9151	19581.73	156.9142	19581.11	156.922	19580.16	159.4839	19570.55
19593.35	157.1619	19579.43	157.1693	19579.83	157.1698	19579.46	157.1655	19580.78	157.1641	19580.78	159.7255	19569.44
19593.62	157.4088	19580.96	157.4101	19580.55	157.4131	19580.15	157.4132	19579.83	157.4157	19582.62	159.9808	19567.96
19591.62	157.6591	19579.22	157.6638	19579.93	157.6654	19581.23	157.6731	19579	157.6668	19582.4	160.2292	19570.09
19593.67	157.9135	19579.82	157.9133	19579.89	157.9134	19580.72	157.9134	19581.92	157.9214	19581.45	160.4828	19566.97
19592.39	158.1629	19579.45	158.173	19580.34	158.1676	19581.51	158.1668	19581.19	158.1631	19578.96	160.7251	19570.57
19592.3	158.409	19580.3	158.4107	19581.63	158.4164	19580.96	158.4136	19579.87	158.4143	19584.04	160.9794	19570.36
19590.81	158.66	19579.9	158.662	19580.95	158.6657	19581.32	158.6715	19580.39	158.6676	19580.97	161.2262	19570.1
19592.52	158.9112	19580.47	158.9086	19581.39	158.9154	19581.25	158.9154	19580.1	158.9208	19583.28	161.4813	19569.06
19593.81	159.1664	19579.36	159.17	19579.55	159.1664	19580.43	159.1666	19581.91	159.1635	19580.44	161.7258	19568.39
19593.77	159.4119	19578.97	159.4118	19580.29	159.4161	19580.28	159.4144	19581.76	159.4146	19579.99	161.9816	19569.78
19591.27	159.6848	19577.49	159.6621	19580.57	159.6681	19581.18	159.6745	19579.52	159.6658	19582.87	162.2259	19569.84
19590.5	159.9163	19579.73	159.9097	19580.7	159.9167	19580.97	159.9151	19580.44	159.9219	19581.94	162.4791	19569.24
19591.7	160.185	19579.2	160.17	19581.02	160.1668	19580.16	160.1678	19581.32	160.1656	19582.32	162.7243	19567.07
19592.37	160.4138	19579.41	160.4129	19579.12	160.4165	19580.93	160.4145	19581.98	160.4179	19581.84	162.9828	19567.57
19592.16	160.6586	19580.03	160.6621	19579.45	160.6652	19581.62	160.668	19582.55	160.6656	19581.16	163.2262	19568.23
19592.5	160.9101	19578.44	160.9058	19580.29	160.9152	19579.09	160.9145	19580.91	160.9178	19581.63	163.4812	19567.1
19592.46	161.1618	19578.64	161.1672	19579.75	161.1651	19579.74	161.1641	19581.69	161.1606	19582.51	163.7241	19567.93
19592.12	161.4112	19580.38	161.4098	19578.68	161.4132	19579.72	161.4128	19579.92	161.4152	19579.98	163.9822	19568.34
19590.44	161.6593	19578.77	161.661	19580.15	161.6633	19580.14	161.6686	19579.45	161.662	19581.42	164.2274	19568.33
19592.25	161.9119	19580.37	161.9063	19577.57	161.9163	19580.68	161.9128	19580.58	161.9188	19584.25	164.4822	19570.83
19592.98	162.1593	19578.76	162.1672	19580.45	162.1698	19580.6	162.165	19579.44	162.163	19581.05	164.7253	19568.03
19593.36	162.4089	19578.4	162.409	19580.83	162.4306	19581.56	162.4127	19581.11	162.4136	19579.66	164.9797	19567.51
19592.64	162.6601	19577.07	162.6612	19578.48	162.6661	19579.81	162.6708	19580.95	162.6685	19580.61	165.2277	19567.95
19590.61	162.9126	19578.54	162.9084	19579.83	162.9142	19580.96	162.9144	19580.23	162.9212	19581.8	165.4913	19566.61
19591.19	163.1574	19579.12	163.1667	19579.83	163.1697	19580.13	163.1657	19581.01	163.1601	19580.58	165.7271	19568.15
19590.56	163.409	19579.16	163.4108	19577.78	163.4177	19581.3	163.4112	19582.42	163.4147	19580.76	165.9821	19569.36
19592.26	163.6618	19578.38	163.6608	19580.4	163.6651	19580.76	163.6709	19579.89	163.6657	19580.79	166.2271	19569.99
19591.74	163.9109	19578.32	163.9089	19581.56	163.9142	19580.75	163.9153	19580.7	163.9223	19579.6	166.482	19569.6
19591.87	164.1616	19577.97	164.1663	19579.2	164.1666	19580.04	164.1649	19579.57	164.1638	19582.46	166.7269	19569.4
19591.41	164.408	19579.56	164.4097	19579.79	164.4179	19580.42	164.4144	19581.92	164.4129	19581.63	166.981	19570.16
19592.79	164.6577	19578.66	164.6628	19579.31	164.666	19579.59	164.6725	19580.52	164.6661	19582.89	167.2307	19569.92
19590.25	164.914	19579.39	164.9085	19578.84	164.9155	19579	164.9132	19581.35	164.9226	19579.13	167.4822	19569.51
19591.88	165.1602	19577.83	165.1676	19577.92	165.1649	19580.83	165.1666	19580.85	165.1629	19581.77	167.7264	19566.13
19593.62	165.4118	19576.37	165.4091	19579.15	165.4169	19580.31	165.4143	19579.74	165.4145	19581.23	167.9814	19567.77
19591.61	165.6574	19579.11	165.6602	19579.16	165.6665	19579.79	165.6748	19580.58	165.667	19580.83	168.2283	19567.04
19591.91	165.9116	19578.66	165.9073	19580.76	165.9154	19580.46	165.9154	19579.59	165.9224	19581.4	168.482	19566.89
19591.94	166.1646	19578.25	166.1679	19578.85	166.1676	19581.57	166.1675	19580.01	166.1623	19580.02	168.7269	19570.59
19590.98	166.4122	19577.8	166.4112	19577.08	166.4226	19579.5	166.4133	19579.84	166.4175	19580.69	168.9851	19570.06
19591.28	166.661	19578.87	166.6617	19579.82	166.667	19580.26	166.6716	19583.06	166.6665	19581.07	169.2301	19567.99
19594.21	166.9113	19577.72	166.9071	19578.04	166.9177	19579.66	166.9178	19579.93	166.9209	19582	169.4841	19568.3
19591.05	167.1599	19577.38	167.1674	19578.49	167.1707	19579.79	167.1719	19579.82	167.1642	19583.33	169.727	19566.66
19591.94	167.4099	19578.06	167.4107	19579.67	167.4195	19579.24	167.4158	19581.26	167.4173	19579.21	169.9849	19567.82
19592.09	167.6601	19577.19	167.6628	19578.96	167.6664	19579.35	167.6751	19581.25	167.6725	19581.34	170.2311	19569.47
19592.99	167.912	19577.88	167.9094	19580.5	167.9171	19579.84	167.9166	19579.97	167.9212	19581.03	170.4851	19568.72
19592.2	168.1604	19575.59	168.1686	19579.78	168.1689	19580.36	168.1673	19580.15	168.1626	19580.91	170.7278	19569.53
19589.05	168.4143	19578.49	168.4116	19578.83	168.4215	19580.65	168.4136	19579.99	168.4178	19580.57	170.9826	19566.44
19590.95	168.66	19579.08	168.6624	19578.92	168.6658	19579.09	168.6731	19579.71	168.6696	19579.32	171.2323	19568.03
19592.17	168.9132	19579.14	168.9121	19578.23	168.9155	19578.19	168.9182	19580.59	168.9225	19580.99	171.4841	19568.32
19590.98	169.1616	19578.85	169.1699	19578.57	169.1682	19581.07	169.1677	19580.13	169.1654	19580.07	171.7293	19565.57
19593.94	169.4127	19577.68	169.4125	19581.25	169.4185	19577.13	169.4144	19580.94	169.4186	19580.74	171.9845	19567.94
19589.88	169.6613	19577.71	169.6627	19578.72	169.6674	19578.23	169.6724	19581.13	169.6684	19581.3	172.2303	19566.7
19590.87	169.9131	19576.17	169.9097	19579.36	169.9173	19578.66	169.9187	19580.43	169.9237	19582.32	172.4845	19567.53
19591.24	170.1627	19578.32	170.1687	19577.37	170.166	19580.65	170.1668	19578.92	170.1648	19580.46	172.7295	19567.24
19591.75	170.4144	19578.72	170.4116	19579.95	170.4191	19579.72	170.4171	19577.74	170.4168	19580.37	172.9843	19566.56
19590.86	170.6609	19578.8	170.6637	19579.03	170.6677	19577.39	170.6731	19578.8	170.6699	19581.34	173.231	19567.16
19592.92	170.9159	19577.19	170.9094	19578.87	170.9171	19580.17	170.9192	19580.39	170.9226	19581.1	173.4835	19567.99
19589.46	171.1658	19577.18	171.1696	19581.15	171.1706	19578.49	171.1679	19579.44	171.165	19580.51	173.73	19566.87
19590.82	171.4159	19578.98	171.413	19580.67	171.4199	19578.7	171.4154	19579.95	171.4176	19580.08	173.9848	19567.99
19591.14	171.6608	19578.32	171.6637	19578.65	171.6687	19580.12	171.6739	19578.65	171.6677	19581.42	174.2314	19569.39
19591.8	171.9138	19578.1	171.913	19579.16	171.9184	19579.15	171.9179	19580.69	171.9271	19579.95	174.4855	19566.71
19591.15	172.166	19577.98	172.1702	19579.13	172.171	19581.17	172.1692	19579.02	172.165	19579.69	174.7294	19568.7
19591.43	172.4159	19577.59	172.4135	19579.79	172.4206	19580.62	172.4156	19580.4	172.4175	19580.17	174.9832	19570.06
19593	172.663	19578.04	172.664	19579.53	172.6678	19580.86	172.6988	19579.65	172.6695	19580.49	175.2332	19567.86
19590.36	172.9158	19578.91	172.9112	19578.2	172.919	19577.92	172.9163	19579.47	172.925	19581.21	175.4856	19568.45
19591.36	173.1634	19576.7	173.1695	19579.8	173.1697	19579.91	173.1688	19578.63	173.1684	19579.24	175.731	19565.78
19591.81	173.4146	19576.97	173.4129	19579.67	173.4213	19578.92	173.4196	19578.55	173.4175	19580.5	175.984	19566.21
19591.99	173.6654	19577.85	173.6652	19579.68	173.6681	19577.75	173.6741	19582.2	173.6699	19581.18	176.2324	19567.75

19590.82	173.9162	19578.33	173.9113	19577.61	173.9202	19577.33	173.9168	19580.31	173.9278	19579.78	176.485	19568.86
19592.73	174.1654	19575.8	174.1722	19580.06	174.1742	19577.24	174.1692	19581.69	174.1672	19580.23	176.7295	19565.57
19591.14	174.4138	19577.41	174.4226	19579.58	174.4249	19580.03	174.4181	19579.96	174.4201	19578.06	176.985	19568.58
19590.6	174.6617	19580.77	174.6656	19580.14	174.6698	19579.62	174.6762	19581.41	174.6722	19581.92	177.2407	19568.88
19590.1	174.9151	19576.86	174.9121	19579.37	174.9185	19579.54	174.9163	19579.07	174.926	19580.35	177.4858	19567.97
19591.39	175.166	19579.24	175.1731	19578.02	175.1703	19577.8	175.1691	19579.65	175.1665	19580.27	177.7315	19569.69
19590.45	175.4154	19578.28	175.4142	19577.42	175.4224	19580.36	175.4164	19581.31	175.4171	19581.28	177.9828	19572.17
19591.35	175.663	19577.49	175.6648	19577.17	175.6718	19579.7	175.6731	19580.77	175.6676	19579.05	178.2356	19568.1
19592.98	175.9179	19577.54	175.9122	19578	175.9197	19579.71	175.9202	19579.56	175.9255	19579.64	178.4852	19566.99
19589.33	176.1632	19578.63	176.1706	19576.59	176.1687	19577.19	176.1719	19579.89	176.1684	19580.61	178.7317	19567.43
19591.09	176.4185	19576.12	176.4148	19579.3	176.4209	19578.92	176.4186	19579.52	176.4202	19577.82	178.9862	19565.99
19590.26	176.6648	19577.2	176.6673	19580.87	176.6713	19578.9	176.6737	19578.75	176.6694	19579.03	179.2339	19567.17
19591.22	176.9155	19577.32	176.9119	19580.08	176.9193	19579.73	176.919	19579.03	176.9267	19579.23	179.4873	19567.36
19590.73	177.1654	19577.02	177.1718	19579.55	177.1736	19580.72	177.1688	19581.08	177.1662	19581.51	179.7322	19568.13
19591.48	177.4174	19578.65	177.4151	19578.22	177.421	19579.12	177.4177	19580.55	177.4215	19580.41	179.9879	19565.61
19591.05	177.6648	19576.56	177.6677	19578.12	177.6709	19578.41	177.676	19580.84	177.668	19580.58	180.2353	19565.36
19591.12	177.916	19578.31	177.9134	19577.12	177.9212	19578.55	177.9194	19580.31	177.9263	19581.85	180.4878	19568.2
19591.29	178.1679	19576.94	178.1708	19578.27	178.1715	19580.05	178.1716	19580.44	178.1675	19580.02	180.7322	19565.85
19592.17	178.4165	19575.43	178.4165	19578.32	178.4343	19578.86	178.4176	19580.42	178.4225	19581.31	180.9872	19566.57
19591.32	178.6656	19578.99	178.6683	19578.45	178.6718	19580.45	178.6789	19581.6	178.6711	19580.27	181.2414	19566.49
19593.18	178.9192	19577.96	178.9143	19578.05	178.9213	19577.47	178.9187	19579.74	178.9285	19578.08	181.5024	19568.59
19591.3	179.1656	19576.29	179.1703	19579.03	179.1721	19578.82	179.1719	19578.16	179.1685	19579.12	181.7332	19568.2
19591.08	179.4165	19577.71	179.4184	19577.83	179.4234	19579.35	179.4198	19579.8	179.4199	19579.81	181.9865	19568.41
19591.51	179.6686	19576.76	179.6678	19578.71	179.6734	19579.53	179.6762	19578.82	179.6729	19578.85	182.2359	19566.5
19593.14	179.9191	19579.67	179.9157	19577.75	179.9209	19578.33	179.9216	19579.52	179.9272	19581.1	182.4872	19566.63
19588.11	180.1646	19578.13	180.1751	19579.05	180.172	19580.55	180.1727	19580.65	180.1694	19581.03	182.7349	19567.2
19590.5	180.4193	19577.84	180.4258	19577.31	180.4254	19578.37	180.421	19580.01	180.4224	19580	182.9891	19567.28
19590.13	180.6668	19577.44	180.668	19578.32	180.6731	19579.1	180.6791	19578.43	180.6724	19578.13	183.2365	19568.12
19591.18	180.9211	19577.51	180.9129	19577.51	180.9234	19580.12	180.9185	19578.39	180.9346	19579.54	183.4894	19567.69
19589.86	181.1661	19578.75	181.1718	19577.8	181.1756	19577.73	181.1734	19579.43	181.1808	19578.88	183.7338	19566.58
19590.59	181.4192	19579.1	181.4192	19578.38	181.4228	19578.78	181.4216	19576.53	181.4279	19581.87	183.9876	19567.41
19592.03	181.666	19578.64	181.6697	19577.87	181.6736	19578.72	181.678	19578.64	181.6721	19580.89	184.2376	19567.96
19590.01	181.9184	19575.71	181.9146	19582.06	181.9221	19578.89	181.9213	19578.73	181.9289	19580.71	184.4894	19567.2
19591.44	182.1668	19575.14	182.1731	19578.14	182.1742	19579.51	182.1727	19578.91	182.1705	19581.36	184.7351	19567.4
19588.97	182.4205	19575.7	182.4221	19578.14	182.4235	19579.74	182.4199	19578.75	182.4216	19582.96	184.9875	19566.86
19589.25	182.667	19577.08	182.6683	19577.72	182.6739	19578.38	182.6777	19579.44	182.6736	19581.68	185.2383	19569.48
19590.85	182.9243	19575.39	182.9162	19577.11	182.9246	19579.15	182.9224	19579.1	182.9306	19581.64	185.4918	19568.1
19591.16	183.1644	19577.24	183.1732	19579.75	183.1739	19578.04	183.1744	19578.02	183.1703	19577.86	185.7343	19568.43
19592.52	183.4316	19577.04	183.4189	19577.49	183.4238	19577.88	183.4205	19579.11	183.4239	19578.77	185.9902	19566.27
19591.32	183.6667	19578.07	183.6689	19578.63	183.6724	19580.81	183.6789	19579.03	183.6717	19581.57	186.2361	19566.82
19589.4	183.9198	19576.02	183.9171	19579.36	183.9232	19578.89	183.9225	19579.61	183.9325	19579.75	186.4917	19568.19
19591.73	184.1686	19575.12	184.1744	19579.29	184.1739	19578.76	184.1742	19579.27	184.17	19582.06	186.7341	19567.6
19590.22	184.4174	19575.14	184.4195	19578.37	184.426	19579.11	184.421	19578.29	184.4234	19580.68	186.9881	19568.11
19591.14	184.6673	19577.11	184.6722	19577.18	184.6747	19578.97	184.6784	19578.52	184.6768	19579.76	187.2374	19566.25
19591.27	184.9211	19575.23	184.9209	19579.27	184.9252	19581.35	184.9222	19579.17	184.932	19579.37	187.4916	19567.65
19591.75	185.169	19577.97	185.1731	19577.14	185.1777	19578.07	185.1744	19579.34	185.1712	19578.54	187.7366	19566.55
19590.39	185.4216	19577.1	185.42	19579.97	185.4282	19578.58	185.4203	19579.56	185.4227	19579.44	187.9906	19569.34
19591.02	185.6653	19576.79	185.6699	19577.88	185.6742	19576.98	185.6794	19577.61	185.6733	19577.97	188.2537	19568.44
19590.59	185.9215	19577.68	185.9174	19576.4	185.9222	19577.46	185.9214	19580.28	185.9309	19579.9	188.494	19566.27
19591.13	186.17	19577.44	186.1721	19577.22	186.1739	19579.02	186.1792	19579.4	186.1726	19578.02	188.742	19568.65
19590.18	186.4198	19577.7	186.4203	19577.65	186.4446	19577.64	186.4196	19578.92	186.429	19579.06	188.99	19565.44
19590	186.6698	19577.92	186.6713	19579.13	186.6754	19580.11	186.6802	19580.06	186.6731	19578.25	189.2382	19568.99
19590.68	186.921	19574.76	186.9166	19579.5	186.9238	19578.17	186.9239	19578.91	186.9302	19579.73	189.4926	19567.1
19592.05	187.1703	19577.87	187.1749	19578.67	187.1792	19579.87	187.1745	19579.67	187.1736	19580.48	189.7367	19567.31
19589.78	187.4208	19575.16	187.4203	19578.81	187.4362	19580.24	187.4263	19578.32	187.4255	19580.37	189.9912	19569.07
19591.21	187.6711	19577.73	187.6706	19578.24	187.6778	19578.74	187.6794	19578.17	187.675	19580.82	190.2404	19567.12
19592.13	187.9225	19576.92	187.9172	19579.68	187.9255	19577.8	187.9215	19578.45	187.9331	19583.86	190.4915	19566.72
19589.51	188.1714	19577.01	188.176	19579.56	188.195	19580.22	188.1753	19579.79	188.1726	19577.38	190.7362	19567.82
19590.53	188.4213	19576.52	188.4222	19578.75	188.4312	19577.95	188.4243	19580.34	188.424	19579.15	190.9904	19565.43
19589.26	188.6692	19575.38	188.6744	19578.65	188.6767	19578.69	188.678	19577.1	188.6742	19579.5	191.2397	19564.42
19590.78	188.9233	19577.05	188.9194	19579.49	188.9239	19577.71	188.9233	19578.32	188.9308	19580.31	191.4928	19569.64
19589.49	189.1734	19576.28	189.1753	19577.79	189.1766	19578.45	189.1789	19579.83	189.1721	19579	191.7385	19566.4
19591.85	189.4217	19577.12	189.4199	19578.41	189.4283	19577.24	189.4212	19580.58	189.4291	19580.15	191.9918	19567.81
19591.03	189.67	19575.77	189.6713	19577.78	189.6784	19581.28	189.6855	19579.96	189.6743	19580.48	192.2383	19567.51
19589.95	189.9208	19577.04	189.9181	19578.6	189.925	19576.74	189.9244	19579.3	189.933	19578.75	192.5059	19565.2
19589.68	190.1687	19576.25	190.1759	19578.61	190.1737	19579.2	190.1765	19578.82	190.175	19580.63	192.742	19565.74
19589.39	190.4226	19577.13	190.4227	19576.14	190.4301	19580.3	190.4237	19577.97	190.4269	19579.32	192.9933	19565.64
19590.31	190.6722	19578.39	190.673	19580.15	190.6778	19579.2	190.6796	19579.03	190.6755	19579.05	193.2401	19566.49
19590.3	190.9247	19576.86	190.918	19579.83	190.9258	19579.67	190.9249	19579.38	190.9346	19578.6	193.4938	19566.05
19590.24	191.17	19577.21	191.179	19577.12	191.179	19579.08	191.1772	19577.47	191.1746	19578.28	193.7379	19564.81

19590.88	191.4237	19575.19	191.4204	19579.58	191.4264	19578.31	191.423	19580.71	191.4262	19579.94	193.9932	19568.6
19589.62	191.6711	19577.17	191.6728	19578.23	191.6773	19577.56	191.6831	19577.3	191.6762	19579.27	194.2385	19566.79
19590.04	191.9205	19576.92	191.9204	19578.18	191.9269	19576.97	191.9268	19578.91	191.9346	19578.36	194.4926	19565.23
19591.08	192.1698	19576.51	192.1811	19577.68	192.1811	19579.52	192.1776	19578.19	192.1761	19579.46	194.7383	19564.94
19590.93	192.4212	19576.86	192.4206	19578.15	192.4279	19577.7	192.4238	19577.98	192.4262	19579.3	194.9925	19565.93
19591.32	192.6737	19577.09	192.6743	19576.85	192.6774	19578.15	192.6825	19577.65	192.6762	19579.64	195.2414	19566.66
19591.13	192.9237	19577.87	192.9216	19578.98	192.9269	19578.38	192.9255	19577.76	192.9342	19581.28	195.4944	19566.58
19591.01	193.1688	19577.79	193.1798	19578.22	193.1774	19578.78	193.1783	19576.52	193.1756	19577.22	195.7389	19565.69
19590.73	193.4224	19577.91	193.4213	19579.83	193.4296	19576.06	193.4251	19577.32	193.4262	19579.57	195.9922	19564.92
19591.06	193.6716	19575.52	193.6731	19577.24	193.6792	19577.56	193.6819	19578.8	193.6782	19579.77	196.24	19566.54
19590.14	193.9257	19576.59	193.92	19578.91	193.9246	19578.23	193.9255	19579.61	193.9365	19577.85	196.4951	19565.69
19590.75	194.172	19576.1	194.1774	19578.8	194.1779	19578.94	194.1792	19578.04	194.1748	19580.4	196.7389	19564.61
19592.7	194.4218	19576.7	194.4225	19578.13	194.4288	19578.5	194.428	19578.9	194.427	19578.11	196.9943	19563.16
19592.84	194.6718	19577.7	194.676	19576.33	194.6791	19579	194.6842	19578.67	194.6773	19578.53	197.2405	19566.84
19590.61	194.9241	19577.29	194.9207	19577.14	194.9256	19579.43	194.9242	19578.85	194.9355	19579.3	197.506	19567.97
19589.25	195.17	19576.16	195.1771	19576.87	195.1785	19576.76	195.1778	19578.02	195.1751	19578.41	197.7419	19568.06
19590.51	195.4219	19575.07	195.4236	19577.58	195.4303	19578.76	195.426	19577.7	195.43	19577.44	197.9938	19567.56
19590.1	195.6735	19576.14	195.6749	19576.82	195.6795	19577.76	195.6824	19579.41	195.6777	19578.4	198.2413	19567.07
19589.46	195.9271	19577.35	195.9203	19578.12	195.9285	19575.86	195.9272	19580.47	195.9353	19579.36	198.4956	19567.14
19589.43	196.1713	19577.98	196.1752	19577.53	196.1768	19578.59	196.1793	19580.01	196.1759	19580.41	198.7411	19567.62
19588.57	196.4266	19576.38	196.4246	19578.67	196.4283	19578.46	196.4271	19579.01	196.4278	19578.56	198.9945	19566.07
19591.12	196.6731	19576.65	196.6768	19577.61	196.6786	19578.02	196.6848	19580.01	196.6783	19577.77	199.2421	19566.43
19591.43	196.9262	19577.65	196.9205	19579.97	196.9274	19577.68	196.9267	19577.45	196.9367	19578.75	199.496	19567.63
19591.29	197.173	19577.18	197.1769	19578.58	197.1837	19577.5	197.1798	19577.2	197.1771	19577.88	199.7412	19567.83
19589.85	197.424	19576.01	197.4236	19578.5	197.4292	19576.53	197.4255	19578.31	197.4298	19581.73	199.9971	19565
19589.43	197.6744	19576.68	197.6746	19578.03	197.6775	19580.25	197.6884	19577.35	197.6788	19577.71	200.2421	19569.2
19589.42	197.9265	19576.59	197.9211	19578.59	197.9273	19578.23	197.9276	19578.51	197.938	19577.86	200.5265	19564.76
19590.3	198.1839	19575.21	198.1865	19576.81	198.1885	19577.77	198.189	19578.54	198.1926	19578.09	200.7462	19566.93
19589.31	198.4244	19576.68	198.4235	19575.91	198.4311	19577.83	198.4268	19577.63	198.4282	19578.7	200.9982	19567.81
19591.36	198.6742	19578.3	198.6755	19578.61	198.6806	19577.23	198.6854	19577.8	198.6805	19580.12	201.2481	19567.55
19590.85	198.9248	19576.51	198.9207	19577.3	198.9277	19576.97	198.9272	19579.17	198.9374	19578.27	201.5005	19564.74
19588.8	199.173	19577.28	199.1785	19577.9	199.1853	19577.73	199.1804	19577.93	199.1777	19576.54	201.7465	19565.29
19591.02	199.4288	19576.14	199.4238	19579.07	199.4329	19579.36	199.4289	19578.33	199.4289	19578.89	201.999	19566.51
19591.04	199.6746	19576.6	199.6778	19576.53	199.6785	19577.69	199.6853	19579.25	199.6793	19580.57	202.2471	19566.66
19590.1	199.9271	19576.37	199.936	19578.16	199.9265	19578.8	199.9256	19579.72	199.9374	19578.76	202.5027	19564.63
19590.46	200.172	19577	200.1787	19578.21	200.1808	19578.21	200.1807	19579.63	200.1765	19577.75	202.7463	19564.83
19589.87	200.4422	19578.08	200.4298	19577.99	200.4371	19576.97	200.4366	19577.53	200.4358	19578.47	203.0001	19565.56
19589.82	200.6793	19576.23	200.6805	19577.53	200.684	19577.76	200.6911	19577.55	200.6824	19577.81	203.2479	19566.66
19590.8	200.9383	19576.44	200.9279	19578.47	200.9327	19578.46	200.9327	19578.85	200.941	19579.19	203.5022	19564.9
19590.94	201.1824	19578.72	201.1866	19577.51	201.1866	19579.56	201.184	19576.86	201.1835	19579.1	203.7489	19567.23
19589.72	201.4295	19575.46	201.4296	19576.92	201.4356	19577.65	201.434	19577.14	201.4355	19580.18	204.0012	19566.4
19589.06	201.6805	19577.11	201.6832	19578.19	201.6908	19577.58	201.6903	19578.2	201.6879	19579.27	204.2484	19565.11
19590.48	201.9319	19574.83	201.9297	19579.89	201.9347	19578.73	201.9344	19578.07	201.9422	19578.16	204.5035	19565.73
19591.14	202.1814	19578.11	202.1832	19579.27	202.1871	19577.71	202.1848	19578.27	202.1825	19577.89	204.7481	19566
19590.62	202.4321	19576.56	202.4288	19577.72	202.4371	19578.71	202.4313	19578.11	202.4348	19578.03	205.0024	19566.83
19591.45	202.678	19575.14	202.6823	19579.21	202.6997	19576.41	202.6897	19578.49	202.6851	19578.04	205.2486	19567.79
19590.1	202.9324	19575.56	202.9276	19577.12	202.9347	19579.04	202.9339	19577.04	202.9406	19579.85	205.502	19567.42
19590.87	203.178	19577.33	203.1818	19576.75	203.1856	19579.34	203.1872	19579.02	203.1834	19578.5	205.7479	19565.71
19589.86	203.433	19576.71	203.4297	19577.31	203.4351	19578.32	203.4325	19579.47	203.4363	19578.24	206.0031	19565.59
19589.71	203.6798	19575.44	203.6835	19577.13	203.6845	19577.93	203.6895	19578.74	203.6849	19577.05	206.2506	19568.02
19589.87	203.9319	19577.55	203.9286	19577.54	203.9364	19578.11	203.9342	19578.95	203.9448	19581.61	206.5047	19564.99
19590.25	204.1817	19575.73	204.1843	19579.36	204.194	19577.68	204.1869	19578.91	204.1854	19578.6	206.7496	19566.61
19592.17	204.4318	19574.96	204.4315	19577.06	204.4388	19578.06	204.4363	19577.36	204.4348	19579.68	207.0018	19568.42
19590.23	204.6812	19576.5	204.6839	19576.21	204.6856	19577.93	204.6967	19576.83	204.6873	19578.24	207.2524	19565.93
19590.33	204.9352	19576.12	204.9282	19578.29	204.9354	19578.44	204.9345	19578.33	204.9446	19579.02	207.5054	19566.25
19590.04	205.1799	19576.64	205.1852	19578.47	205.1887	19577.97	205.1856	19578.37	205.1844	19578.75	207.7553	19567.39
19589.09	205.4318	19577.27	205.4303	19578.99	205.4385	19577.84	205.4368	19577.57	205.4375	19578.73	208.0042	19564.59
19590.35	205.6805	19577.26	205.6838	19578.39	205.6868	19577.52	205.692	19578.14	205.6905	19579.58	208.2494	19567.92
19589.76	205.9375	19577.35	205.9285	19575.37	205.9357	19576.78	205.9347	19577.79	205.9467	19578.71	208.5058	19566.79
19590.74	206.1818	19574.72	206.1849	19576.34	206.1872	19577.32	206.1889	19577.84	206.1853	19579.66	208.7496	19564.39
19589.29	206.4315	19574.91	206.429	19578.05	206.4366	19576.37	206.436	19577.94	206.4362	19577.22	209.0044	19564.29
19590.05	206.6806	19576.01	206.6845	19576.3	206.6871	19577.36	206.6917	19577.18	206.6866	19579.7	209.2497	19565.58
19589.86	206.9347	19576.91	206.9301	19576.61	206.9374	19578.65	206.9348	19577.61	206.9435	19577.6	209.5048	19567.59
19590.13	207.1832	19576.8	207.1866	19578.58	207.1939	19577.91	207.1877	19576.92	207.1856	19578.12	209.752	19565.12
19588.51	207.4342	19577.92	207.4344	19576.4	207.4403	19578.44	207.4365	19577.42	207.4359	19578.42	210.0036	19567.5
19589.98	207.6802	19577.52	207.6904	19577.54	207.6863	19575.12	207.6932	19578.02	207.6859	19578.73	210.2505	19566.29
19588.86	207.9342	19576.02	207.9331	19576.34	207.9381	19577.53	207.9362	19577.05	207.9416	19578.06	210.5064	19565.32
19592.57	208.1816	19575.8	208.1852	19576.4	208.1906	19575.14	208.1954	19578.08	208.1867	19579.42	210.751	19564.83
19591.79	208.4367	19575.6	208.4325	19577.3	208.4409	19578.21	208.4357	19577.68	208.4361	19579.91	211.0033	19567.1
19589.03	208.6808	19577.19	208.6837	19575.94	208.6879	19576.65	208.6972	19578.02	208.6871	19577.47	211.2512	19567.05

19588.08	208.9335	19576.58	208.9328	19577.32	208.9371	19577.47	208.9358	19578.19	208.946	19580.81	211.5064	19566.77
19589.49	209.182	19576.99	209.1854	19579.93	209.1897	19576.29	209.1892	19579.38	209.1877	19578.05	211.7505	19565.77
19587.58	209.436	19576.09	209.4321	19577.99	209.4405	19577.97	209.437	19578.85	209.4376	19578.91	212.0049	19566.47
19589.7	209.6845	19576.24	209.6849	19577.75	209.6877	19577.69	209.6953	19579.29	209.6871	19579.83	212.2518	19565.35
19589.8	209.9379	19576.84	209.9311	19577.21	209.9389	19578.77	209.9366	19577.99	209.9427	19579.24	212.5093	19566.14
19588.87	210.1805	19576.36	210.1877	19576.96	210.1919	19577.32	210.1866	19577.92	210.1864	19578.42	212.7504	19566.09
19590.61	210.448	19575.32	210.4325	19576.72	210.441	19578.58	210.4407	19577.58	210.4378	19581.25	213.0047	19566.88
19591.14	210.6838	19575.09	210.6856	19578.69	210.6905	19575.8	210.6933	19577.97	210.6875	19579.18	213.2509	19565.63
19588.76	210.9388	19576.19	210.9301	19575.84	210.9387	19578.13	210.9364	19576.92	210.9445	19579.7	213.5105	19565.54
19588.83	211.1814	19575.95	211.186	19578.52	211.1877	19577.29	211.19	19578.83	211.1877	19579.33	213.7527	19569.52
19588.13	211.4366	19575.7	211.4345	19577.02	211.4396	19575.59	211.4386	19578.46	211.4383	19579.76	214.0053	19563.7
19590.88	211.6837	19575.47	211.6866	19578.67	211.6914	19576.89	211.6933	19578.04	211.6877	19579.54	214.2521	19566.88
19590.16	211.9366	19577.17	211.9316	19577.55	211.94	19577.27	211.9379	19577.54	211.9671	19578.25	214.5086	19565.18
19590.33	212.1834	19575.1	212.1884	19578.25	212.1929	19578.44	212.1898	19578.98	212.1886	19579.43	214.754	19565.53
19589.78	212.4345	19577.52	212.4355	19577.51	212.4455	19579.17	212.4387	19576.75	212.4397	19577.79	215.0043	19565.9
19589.43	212.6835	19575.31	212.6842	19578.32	212.6907	19577.26	212.6959	19577.35	212.6916	19577.58	215.2533	19564.6
19590.44	212.9376	19575.84	212.9338	19578.12	212.9418	19576.18	212.9388	19578.3	212.9446	19580.72	215.5073	19565.69
19589.72	213.1838	19575.91	213.1893	19576.2	213.1929	19578.11	213.1898	19577.38	213.189	19578.53	215.7533	19565.29
19591.21	213.4371	19577.11	213.4345	19575.69	213.4422	19577.97	213.4396	19578.51	213.4385	19578.09	216.0057	19565.98
19589.98	213.6831	19576.23	213.6866	19576.6	213.6882	19576.65	213.6954	19576.21	213.6888	19579.48	216.2547	19566.35
19590.39	213.9381	19576.2	213.9351	19578.28	213.9399	19576.4	213.94	19576.57	213.9475	19577.59	216.5078	19564.16
19589.89	214.1828	19575.89	214.189	19576.53	214.1908	19576.78	214.1916	19577.26	214.1887	19579.36	216.7535	19567.28
19589.56	214.4377	19576.11	214.4377	19578.24	214.4426	19577.17	214.4594	19577.7	214.4418	19579.98	217.0084	19567.26
19588.77	214.6857	19576.05	214.687	19576.45	214.6898	19578.11	214.6955	19576.44	214.6886	19577.86	217.2548	19565.94
19589.43	214.9402	19574.6	214.9333	19578.76	214.9389	19577.95	214.9393	19577.82	214.9451	19580.39	217.5096	19566.28
19590.09	215.1834	19577.34	215.19	19575.9	215.1919	19577.55	215.1915	19576.02	215.1892	19579.31	217.7592	19566.07
19590.63	215.4501	19574.14	215.4344	19575.1	215.4439	19576.68	215.4387	19576.23	215.4396	19578.42	218.0066	19564.88
19588.24	215.6859	19576.98	215.6872	19578.91	215.691	19577.14	215.697	19577.29	215.6909	19577.84	218.2537	19565.2
19590.01	215.9424	19574.71	215.9344	19574.69	215.9449	19577.98	215.9405	19577.65	215.9455	19577.69	218.509	19564.22
19589.24	216.1863	19575.26	216.1918	19575.92	216.1934	19575.49	216.1911	19579.74	216.1903	19576.86	218.756	19565.7
19587.91	216.4367	19577.71	216.4364	19577.56	216.4424	19576.83	216.4393	19578.68	216.4404	19577.94	219.0078	19567.25
19588.55	216.686	19577.64	216.6894	19576.81	216.6921	19577.6	216.6959	19578.15	216.6907	19576.53	219.2541	19566.66
19590.15	216.939	19576.55	216.9378	19576.95	216.9451	19578.44	216.9405	19577.51	216.946	19580.7	219.5095	19562.78
19591.36	217.1842	19577.48	217.1896	19577.93	217.1956	19576.31	217.1913	19576.29	217.1895	19577.09	219.756	19565.91
19590.16	217.4409	19577.33	217.4364	19575.79	217.4438	19575.52	217.4439	19577.78	217.4414	19578.68	220.0094	19566.12
19588.73	217.6846	19574.28	217.6876	19576.36	217.693	19577.86	217.6974	19577.74	217.6912	19577.99	220.2558	19565.18
19589.62	217.9408	19576.86	217.9395	19576.82	217.9418	19578.17	217.9419	19576.3	217.947	19578.72	220.5115	19564.67
19589.41	218.1857	19576.58	218.1908	19577.29	218.1943	19578.71	218.1941	19577.79	218.1908	19577.54	220.7565	19563.59
19590.44	218.4385	19576.98	218.4383	19577.32	218.4433	19577.97	218.4392	19576.53	218.4415	19576.88	221.0084	19565.06
19588.33	218.6847	19574.79	218.6883	19578.82	218.6911	19576.59	218.6976	19577.97	218.6936	19578.05	221.2576	19566.22
19589.49	218.9436	19574.96	218.9345	19576.35	218.9432	19578.3	218.9407	19577.31	218.9482	19577.71	221.5103	19564.98
19590.31	219.1852	19575.28	219.191	19576.92	219.1921	19577.64	219.193	19577.63	219.1909	19578.92	221.7563	19566.11
19588.07	219.4387	19575.69	219.4363	19575.78	219.4454	19576.91	219.4428	19577.03	219.4424	19577.67	222.0085	19564.72
19589.33	219.6867	19576.42	219.6889	19576.86	219.6934	19577.43	219.6988	19579.68	219.6925	19575.12	222.2557	19563.14
19589.11	219.9592	19575.69	219.9353	19576.93	219.9408	19577.91	219.9403	19576.92	219.9479	19579.02	222.516	19565.59
19591.13	220.1904	19574.54	220.1939	19578.14	220.1962	19577.1	220.1921	19576.37	220.1887	19578.82	222.7561	19566.24
19589.14	220.4431	19576.03	220.439	19577.93	220.4462	19576.45	220.44	19575.64	220.4434	19578.82	223.0086	19565.61
19588.37	220.6871	19573.87	220.6908	19576.83	220.693	19578.04	220.6992	19578.28	220.6915	19576.93	223.2576	19565.22
19589.92	220.9434	19576.72	220.9365	19575.42	220.9399	19576.09	220.9421	19577.63	220.9477	19578.93	223.5136	19565.87
19590.32	221.1853	19576.28	221.1925	19575.86	221.1947	19578.11	221.1932	19578.43	221.1894	19580.45	223.757	19567.53
19589.83	221.4408	19574.1	221.4367	19577.39	221.4448	19576.66	221.4418	19578.1	221.4465	19578.92	224.009	19566.17
19589.23	221.6866	19576.38	221.6914	19576.72	221.6994	19577.54	221.6977	19577.82	221.6933	19576.36	224.2578	19565.76
19589.53	221.9424	19575.47	221.9379	19575.31	221.9432	19578.09	221.942	19575.97	221.9499	19578.89	224.5124	19567.12
19589.72	222.1853	19575.97	222.1935	19576.45	222.1962	19576.62	222.1959	19578.38	222.191	19577.2	224.7565	19564.82
19588.87	222.4431	19576.18	222.4393	19575.8	222.4452	19577.47	222.4395	19575.78	222.4437	19578.36	225.0095	19566.24
19591.08	222.6877	19575.51	222.6916	19576.38	222.6941	19577.36	222.6977	19577.99	222.6927	19578.18	225.2599	19564.73
19589.27	222.943	19575.38	222.9389	19577.7	222.9436	19575.22	222.9424	19577.73	222.9493	19577.4	225.5133	19565.84
19589.63	223.1859	19575.45	223.1933	19575.46	223.1993	19576.3	223.1963	19577.86	223.1931	19578.65	225.7593	19565.36
19589.46	223.4405	19576.03	223.4368	19576.38	223.4463	19575.96	223.4417	19578.8	223.4445	19577.55	226.0118	19564.88
19587.82	223.6862	19575.39	223.6904	19575.7	223.6941	19576.34	223.6999	19576.04	223.6928	19580.16	226.2596	19566.21
19590.21	223.9413	19575.7	223.9391	19575.22	223.9434	19575.31	223.9414	19577.75	223.9506	19578.22	226.5121	19565.02
19589.97	224.1871	19576.03	224.1934	19574.09	224.1955	19575.73	224.1956	19579.56	224.1929	19577.58	226.7601	19565.46
19587.71	224.442	19576.23	224.4394	19577.4	224.448	19578.16	224.4436	19576.8	224.4435	19578.23	227.0124	19565.69
19590.02	224.6914	19575.24	224.6912	19578.05	224.695	19578.98	224.7002	19578.63	224.6937	19577.85	227.2597	19566.57
19589.05	224.9443	19575.79	224.9405	19578.58	224.942	19578.15	224.9429	19577.07	224.948	19579.21	227.5126	19565.2
19589.72	225.189	19573.68	225.193	19576.01	225.197	19577.23	225.1948	19576.87	225.1923	19576.6	227.7601	19565.86
19588.86	225.4452	19577.51	225.4397	19576.26	225.4477	19577.49	225.4433	19579.55	225.4443	19576.61	228.0106	19568.18
19589.95	225.6906	19574.24	225.6916	19577.49	225.6971	19576.74	225.7002	19576.6	225.6949	19578.78	228.2598	19565.46
19586.97	225.9433	19577.32	225.9385	19575.73	225.9436	19576.95	225.9434	19578.42	225.9516	19576.06	228.5147	19564.94
19590.31	226.1875	19575.4	226.1947	19575.9	226.1967	19576.06	226.1967	19576.68	226.1929	19578.73	228.7595	19562.15

19588.98	226.4423	19575.3	226.4395	19575.5	226.448	19577.53	226.4434	19578.75	226.446	19579.73	229.0132	19566.29
19588.05	226.6903	19575.24	226.6936	19575.2	226.6977	19576.33	226.702	19578.62	226.6954	19577.8	229.2609	19564.79
19587.77	226.944	19576.35	226.9397	19576.65	226.9458	19577.06	226.9433	19577.43	226.9498	19576.68	229.5158	19566.1
19589.16	227.1887	19576.24	227.1967	19575.5	227.1996	19578.75	227.1964	19576.93	227.2003	19576.43	229.7611	19564.95
19590.41	227.4417	19574.48	227.4397	19576.97	227.4487	19577.36	227.4433	19577.04	227.4467	19579.96	230.0125	19564.19
19588.79	227.6906	19574.96	227.6917	19576.92	227.6968	19577.35	227.7016	19577.19	227.6982	19580.7	230.2598	19563.7
19589.31	227.9445	19574.99	227.941	19577.19	227.9452	19576.62	227.9432	19576.55	227.9505	19578.21	230.5157	19565.44
19588.03	228.1886	19574.13	228.1968	19574.75	228.2037	19577.35	228.1984	19576.54	228.1948	19578.27	230.7608	19564.79
19589.03	228.4441	19572.61	228.441	19574.98	228.4461	19575.77	228.4437	19575.18	228.4461	19577.7	231.0129	19565.37
19589.91	228.6879	19576.79	228.6916	19577.29	228.6947	19576.83	228.7018	19577.52	228.6949	19578.16	231.2597	19565.43
19587.74	228.9442	19575.28	228.9416	19577.31	228.9467	19577.09	228.9405	19576.4	228.9515	19579.5	231.5219	19567.11
19590.15	229.1885	19575.81	229.1966	19578.23	229.1998	19576.74	229.1979	19576.75	229.1905	19579.52	231.7641	19564.06
19590.55	229.4434	19576.24	229.4449	19575.84	229.4521	19576.56	229.4453	19577.65	229.4479	19577.24	232.0133	19566.28
19589	229.6904	19576.59	229.6915	19578.58	229.6919	19574.75	229.7032	19577.22	229.696	19579.45	232.2609	19565.48
19587.72	229.9464	19575.56	229.9446	19577.49	229.9499	19578.04	229.94	19577.53	229.9552	19576.53	232.5186	19565.63
19588.87	230.1911	19575.88	230.196	19575.29	230.1997	19576.12	230.1972	19578.2	230.2023	19577.53	232.7624	19563.73
19588.66	230.446	19575.38	230.4426	19577.82	230.45	19575.93	230.445	19577.41	230.4475	19578.33	233.0143	19567.42
19587.73	230.6892	19574.96	230.693	19574.57	230.6958	19576.56	230.7023	19576.72	230.6976	19577.72	233.261	19567.21
19589.25	230.9471	19575.18	230.9396	19577.08	230.9402	19576.71	230.9418	19578.46	230.9523	19578.05	233.5156	19567.03
19589.74	231.1905	19574.86	231.1971	19576.04	231.2007	19576.04	231.1977	19576.46	231.1971	19577.65	233.7627	19564.01
19589.04	231.4464	19575.41	231.4431	19575.18	231.4449	19577.22	231.4453	19577.24	231.45	19577.69	234.0136	19563.88
19589.2	231.6919	19575.91	231.6955	19577.44	231.6985	19576.32	231.7039	19577.71	231.695	19577.03	234.2628	19566.48
19588.81	231.9456	19575.58	231.9409	19575.34	231.9472	19576.5	231.9441	19577.18	231.9514	19577.8	234.5173	19563.42
19589.3	232.1912	19575.72	232.1985	19575.89	232.1997	19577.06	232.1993	19577.61	232.1908	19576.56	234.7626	19565.85
19590.55	232.4466	19577.1	232.4416	19576.63	232.4464	19578.4	232.442	19576.55	232.4491	19579.07	235.014	19564.54
19587.92	232.6934	19574.33	232.6943	19579.27	232.6973	19576.52	232.7046	19576.71	232.6959	19577.49	235.2614	19565.03
19589.5	232.945	19575.77	232.941	19576.5	232.947	19576.92	232.9443	19577.59	232.9541	19577.85	235.5177	19565.32
19588.1	233.1918	19575.27	233.1966	19576.14	233.1996	19578.22	233.194	19576.62	233.1967	19577.77	235.7626	19565.99
19589.27	233.4571	19575.71	233.4427	19576.41	233.4455	19577.53	233.4475	19575.14	233.4492	19579.29	236.0153	19565.86
19590.29	233.6983	19574.83	233.6936	19575.13	233.6986	19576.92	233.7103	19578.27	233.6967	19578.47	236.2636	19563.53
19589.62	233.946	19572.93	233.9418	19578.29	233.9461	19576.17	233.9515	19576.86	233.9569	19577.96	236.5152	19564.51
19591.02	234.1919	19576.99	234.1993	19574.28	234.2058	19575.27	234.1978	19577.08	234.199	19578.46	236.7622	19563.87
19589.23	234.4462	19575.8	234.4435	19577.78	234.4517	19576.19	234.4473	19575.09	234.4505	19579.03	237.0153	19566.58
19588.05	234.6944	19577.19	234.6943	19576.1	234.6997	19576.94	234.7036	19577.54	234.6943	19577.69	237.2636	19567.05
19589.21	234.9482	19576.11	234.9422	19576.82	234.9479	19577.89	234.9601	19578.36	234.9526	19575.52	237.5287	19564.21
19588.55	235.1942	19574.12	235.2006	19577.2	235.2026	19576.27	235.1985	19577.7	235.196	19577.45	237.7775	19564.7
19588.72	235.4454	19576.11	235.445	19575.1	235.4551	19576.94	235.4515	19576.17	235.4495	19577.47	238.026	19562.75
19589.44	235.6937	19573.35	235.6947	19576.35	235.7003	19576.78	235.7055	19576.1	235.6951	19577.61	238.2723	19564.54
19589.95	235.9493	19574.36	235.9433	19576.47	235.9477	19575.03	235.9478	19575.9	235.9559	19578.29	238.5249	19564.89
19589.31	236.1946	19574.77	236.1966	19575.25	236.2046	19574.89	236.2011	19575.96	236.1992	19581.02	238.7726	19564.07
19590.07	236.4483	19575.15	236.445	19575.78	236.4506	19578.16	236.4475	19577.27	236.4476	19576.86	239.0228	19564.77
19589.11	236.6952	19575.35	236.6982	19575.19	236.7001	19575.89	236.7046	19575.54	236.6991	19578.14	239.2709	19564.71
19589.84	236.9495	19576.71	236.9589	19574.61	236.9499	19577.37	236.9485	19577.61	236.9495	19578.61	239.5264	19563.97
19589.79	237.1949	19576.08	237.2088	19576.03	237.2052	19576.78	237.2006	19577.93	237.1926	19576.6	239.776	19564.33
19587.93	237.4547	19576.51	237.4558	19577.67	237.4522	19576.73	237.4462	19577.18	237.4526	19579.32	240.0263	19564.92
19589.72	237.6942	19575.6	237.7043	19578.95	237.7022	19576.61	237.7032	19579.88	237.6998	19578.04	240.2741	19563.85
19588.07	237.9469	19574.06	237.9491	19576.68	237.9497	19577.84	237.9474	19576.72	237.9524	19576.47	240.5206	19564.35
19588.51	238.1951	19573.56	238.1995	19575.21	238.2024	19576.91	238.2018	19576.03	238.1972	19577.97	240.759	19564
19589.41	238.4473	19574.92	238.4469	19574.66	238.4525	19576.94	238.4499	19576.33	238.4534	19577.64	241.0138	19565.48
19589.22	238.6955	19576.6	238.6976	19575.1	238.7031	19575.68	238.7064	19575.24	238.7	19577.67	241.2624	19565.04
19589.43	238.9498	19574.24	238.943	19577	238.946	19579.2	238.9435	19576.45	238.9532	19579.14	241.5147	19562.94
19589.06	239.1962	19575.77	239.201	19576.11	239.2024	19575.99	239.2015	19578.16	239.2115	19579.03	241.7593	19562.85
19589	239.4489	19577.39	239.4475	19576.64	239.4527	19576.19	239.4482	19575.85	239.4608	19578.92	242.0136	19564.71
19588.25	239.6955	19575.73	239.6971	19576.61	239.7039	19576.44	239.7054	19576.07	239.7076	19578.43	242.2613	19564.97
19587.54	239.9468	19576.09	239.9464	19576.41	239.9502	19575.41	239.9475	19577.2	239.9616	19577.44	242.5186	19566.92
19588.94	240.1948	19574.8	240.2012	19576.93	240.2036	19577.29	240.2023	19576.94	240.2099	19575.54	242.7589	19562.81
19588.29	240.452	19574.67	240.4485	19574.47	240.4579	19575.66	240.4493	19576.85	240.4597	19576.51	243.0137	19564.9
19588.06	240.6963	19574.22	240.6927	19575.82	240.6944	19575.65	240.7011	19577.4	240.7051	19577.02	243.2629	19566.01
19589.94	240.9444	19575.48	240.9402	19574.83	240.9465	19577.47	240.945	19576.58	240.9528	19578.03	243.5175	19567.73
19588.51	241.1908	19575.03	241.2026	19578.03	241.1976	19576.6	241.2006	19577.36	241.1947	19576.13	243.7645	19568.69
19588.26	241.4463	19575.98	241.4421	19576.19	241.4575	19576.79	241.4519	19577.71	241.4477	19578.08	244.0208	19564.69
19588.63	241.698	19575.99	241.6953	19575.88	241.7077	19577.7	241.7054	19576.62	241.7012	19577.65	244.2604	19566.71
19587.76	241.946	19577.2	241.9416	19577.71	241.9511	19575.42	241.9463	19576.8	241.9505	19579.25	244.5194	19563.83
19588.2	242.1926	19577.14	242.204	19575.43	242.2029	19577.01	242.2015	19577.32	242.1971	19576.83	244.7631	19565.13
19587.28	242.4502	19576.01	242.4434	19576.01	242.4505	19575.2	242.4474	19576.78	242.4543	19577.51	245.013	19565.56
19588.32	242.6994	19575.01	242.6937	19574.56	242.7004	19575.89	242.7009	19576.46	242.7001	19576.56	245.2606	19567
19589.77	242.9503	19576.08	242.9415	19574.74	242.9468	19576.57	242.9462	19575.58	242.9508	19578.21	245.5175	19564.38
19588.29	243.1997	19575.49	243.1978	19575.35	243.2	19576.2	243.1982	19577.24	243.1978	19579.81	245.7616	19565.42
19591.17	243.4532	19575.6	243.4441	19577.95	243.4497	19576.62	243.4464	19576.31	243.4489	19577.26	246.0168	19564.6
19588.36	243.6964	19575.51	243.6937	19575.65	243.6991	19577.58	243.7016	19576.79	243.7005	19576.19	246.2609	19564.3

19589.9	243.9471	19575.91	243.9397	19574.42	243.9522	19575.96	243.9473	19577.25	243.9517	19577.13	246.5169	19563.73
19589.15	244.1923	19573.82	244.1983	19574.64	244.1984	19577.55	244.1975	19578.37	244.1976	19576.78	246.7626	19565.3
19588.95	244.4466	19577.32	244.4462	19575.8	244.4516	19576.47	244.4476	19576.12	244.4483	19578.62	247.0161	19564.37
19587.58	244.694	19573.52	244.6955	19576.35	244.7	19577.08	244.7021	19577.29	244.7035	19577.06	247.2623	19565.17
19588.2	244.9474	19575.19	244.9417	19576.57	244.9471	19576.97	244.9468	19574.81	244.9502	19578.21	247.5165	19564.59
19589.7	245.1921	19574.71	245.1984	19575.73	245.2012	19574.94	245.1999	19576.13	245.1969	19578.34	247.7621	19563.59
19589.5	245.4477	19574.25	245.4443	19575.66	245.4561	19576.91	245.4472	19577.31	245.4487	19577.41	248.0226	19562.96
19590.19	245.6924	19574.67	245.6941	19575.65	245.6979	19577.37	245.7016	19576.19	245.6983	19578.68	248.2624	19564.16
19586.74	245.9468	19574.92	245.9449	19575.7	245.9484	19577.92	245.948	19575.51	245.9525	19578.68	248.538	19565.23
19589.48	246.1948	19574.77	246.2016	19574.45	246.2017	19577.97	246.199	19574.38	246.1968	19577.71	248.778	19563.93
19590.42	246.4492	19574.58	246.4428	19576.28	246.4531	19576.69	246.4459	19578.52	246.4498	19578.82	249.0212	19565.74
19590.03	246.6946	19575.29	246.6964	19575.94	246.7029	19576.55	246.7026	19576.82	246.7	19578.5	249.263	19562.91
19587.68	246.9459	19572.61	246.9444	19575.44	246.9468	19577.31	246.9483	19577.46	246.9529	19578.1	249.5304	19567.21
19589.14	247.1921	19576.57	247.2004	19576.25	247.201	19575.09	247.1992	19575.43	247.1958	19577.88	249.7754	19566.43
19589.42	247.4523	19575.62	247.4463	19574.54	247.4537	19575.63	247.447	19575.77	247.4484	19576.87	250.0195	19564.38
19589.39	247.6949	19574.64	247.6965	19574.41	247.6982	19575.74	247.7017	19577.45	247.7051	19577.29	250.263	19565.52
19586.56	247.9476	19574.7	247.9434	19576.4	247.9493	19575.5	247.9492	19576.35	247.9503	19576.53	250.5192	19565.27
19589.14	248.1931	19572.37	248.2004	19575.97	248.2011	19576.63	248.2013	19577.24	248.1928	19577.08	250.7638	19563.08
19589.58	248.4496	19573.92	248.4455	19576.42	248.4709	19578.85	248.4624	19577.03	248.4518	19578.35	251.0168	19565.27
19588.8	248.6949	19574.06	248.6967	19576.16	248.7165	19576.51	248.7071	19575.47	248.7031	19578.86	251.2629	19564.49
19588.4	248.9475	19573.41	248.9429	19575.64	248.9576	19576.07	248.952	19577.68	248.9492	19578.91	251.5177	19565.99
19589.86	249.1944	19575.77	249.2008	19575.82	249.2005	19577.41	249.1962	19576.2	249.1988	19577.72	251.7648	19565.94
19586.65	249.4477	19575.47	249.4456	19576.02	249.4563	19578.14	249.4536	19576.43	249.4519	19577.34	252.0192	19564.62
19587.87	249.6955	19576.83	249.6971	19574.96	249.6988	19579.07	249.7006	19575.18	249.7006	19576.22	252.2634	19563.85
19588.22	249.9495	19576.23	249.9452	19576.05	249.9495	19575.95	249.9498	19575.28	249.9527	19576.24	252.5232	19565.08
19587.79	250.1944	19574.36	250.2012	19574.13	250.2053	19576.4	250.201	19576.35	250.196	19576.51	252.7722	19564.79
19588.34	250.4495	19576.99	250.4457	19574.59	250.4532	19576.23	250.4495	19577.71	250.4509	19577.53	253.0211	19563.74
19590.51	250.6937	19576.11	250.6953	19575.17	250.6949	19576.37	250.7027	19575.97	250.7012	19577.69	253.2699	19562.78
19588.35	250.9497	19576.58	250.9439	19575.13	250.9491	19574.21	250.9447	19575.37	250.9516	19579.6	253.5188	19566.32
19589.04	251.1942	19574.35	251.2	19575.98	251.2019	19575.92	251.2001	19575.64	251.1995	19576.09	253.7766	19566.71
19587.2	251.4586	19573.45	251.4465	19574.77	251.4551	19576.23	251.4512	19575.63	251.4509	19577.79	254.0341	19566.54
19591.09	251.7029	19574.7	251.6986	19577.11	251.7012	19576.31	251.7004	19577.06	251.7022	19577.71	254.2749	19564.71
19587.59	251.9483	19577.77	251.9454	19576.48	251.9499	19577.08	251.9501	19575.21	251.9545	19577.3	254.5282	19565.25
19589.18	252.1957	19576.38	252.202	19575.35	252.2049	19575.59	252.2025	19578.68	252.1987	19577.9	254.7673	19564.97
19588.28	252.4493	19574.3	252.4499	19575.48	252.4516	19577.61	252.4443	19576.25	252.4517	19577.42	255.0187	19566.54
19589.68	252.6992	19573.87	252.6992	19574.49	252.7033	19575.4	252.7056	19577.65	252.7019	19577.11	255.2655	19564.96
19589.73	252.9496	19575.05	252.9454	19576.21	252.948	19577.73	252.9497	19575.26	252.9556	19579.14	255.5378	19563.49
19587.93	253.195	19574.91	253.2023	19576.09	253.2023	19575.39	253.2025	19574.03	253.2004	19577.83	255.7751	19563.65
19587.97	253.4515	19574.74	253.4487	19575.58	253.4521	19576.17	253.4466	19576.17	253.452	19577.79	256.019	19565.1
19587.56	253.6963	19574.99	253.6992	19575.47	253.6976	19575.65	253.7058	19577.7	253.7035	19578.02	256.2648	19563.11
19587.86	253.9504	19574.12	253.9452	19576.77	253.9527	19575.26	253.9505	19575.78	253.9564	19576.21	256.5212	19564.45
19591.01	254.196	19573.62	254.202	19574.37	254.1977	19576.67	254.2043	19576.76	254.1995	19576.76	256.7653	19563.07
19587.89	254.4523	19575.51	254.4469	19575.01	254.4578	19578.36	254.4539	19575.27	254.4532	19578.24	257.0216	19564.43
19586.74	254.6966	19575.36	254.6977	19575.68	254.7069	19577.71	254.7025	19577.24	254.703	19577.67	257.2665	19564.2
19588.37	254.9508	19576.26	254.9466	19576.52	254.952	19576.21	254.9548	19577.4	254.9551	19578.35	257.5222	19563.78
19588	255.1968	19576.81	255.2025	19577.12	255.1978	19575.19	255.2086	19574.05	255.2	19577.29	257.7656	19563.2
19587.02	255.4524	19574.84	255.4626	19575.81	255.4574	19577.8	255.4558	19576.66	255.4523	19578.75	258.0224	19563.69
19587.95	255.7	19575.3	255.7143	19576.31	255.7036	19575.31	255.7085	19575.22	255.7043	19576.11	258.2725	19563.5
19589.03	255.9507	19576.97	255.9593	19575.44	255.9486	19577.53	255.9606	19576.01	255.9554	19578.3	258.522	19565.18
19588.21	256.1953	19574.34	256.2096	19577.32	256.2038	19577.22	256.2128	19577.61	256.202	19576.9	258.7663	19566.72
19587.04	256.4537	19576.88	256.4562	19574.93	256.4545	19576.76	256.4583	19576.17	256.4538	19578.23	259.0187	19563.59
19588.99	256.6993	19574.19	256.7053	19575.83	256.7026	19575.94	256.7096	19575.88	256.7049	19576.57	259.2686	19564.75
19588.47	256.9508	19575.31	256.9492	19576.81	256.9474	19578.69	256.9547	19578.33	256.9593	19577.18	259.5239	19564.52
19587.62	257.1965	19577.18	257.2012	19575.56	257.2164	19575.76	257.2065	19576.41	257.2017	19578.83	259.7666	19563.47
19587.25	257.4529	19575.11	257.448	19576.54	257.4656	19575.95	257.4517	19575.48	257.4576	19577.54	260.0207	19563.15
19588.68	257.699	19574.75	257.7003	19576.93	257.7155	19575.46	257.7009	19575.3	257.7032	19578.83	260.2658	19563.7
19587.72	257.9491	19573.69	257.9474	19575.13	257.9614	19578.02	257.9476	19579.29	257.9576	19578.35	260.5237	19564.14
19589.41	258.1984	19575.95	258.2022	19575.37	258.2101	19575.86	258.2035	19575.85	258.2012	19577.55	260.7672	19564.31
19588.47	258.4538	19574.41	258.4503	19574.15	258.4636	19575.33	258.4499	19576.18	258.4536	19577.79	261.0187	19564.76
19587.48	258.7009	19574.93	258.703	19576.52	258.7112	19577.2	258.7095	19576.96	258.704	19578.41	261.2685	19562.95
19590.44	258.9526	19574.59	258.952	19576.82	258.9588	19577.04	258.9742	19576.16	258.9588	19576.46	261.5228	19565.99
19588.13	259.2041	19573.53	259.2054	19576.83	259.2088	19573.88	259.4575	19575.28	259.2002	19577.8	261.7692	19564.4
19588.7	259.4587	19576.74	259.4534	19576.72	259.4618	19577.21	259.7101	19576.3	259.4542	19576.96	262.0219	19561.9
19586.69	259.7061	19573.31	259.7017	19574.68	259.7133	19576.31	259.9561	19577.54	259.703	19575.85	262.2683	19563.78
19588.78	259.9571	19573.47	259.9486	19575.92	259.9609	19575.17	260.2091	19577.45	259.9584	19578.29	262.5242	19563.28
19589.64	260.2056	19574.56	260.2207	19576.16	260.2128	19575.73	260.4578	19576.93	260.2018	19577.6	262.7675	19564.04
19588.84	260.4697	19571.34	260.4725	19575.7	260.4652	19575.4	260.7103	19577.28	260.4541	19577.31	263.0217	19563.24
19588.9	260.7202	19575.14	260.9545	19574.15	260.7161	19577.58	260.9625	19575.74	260.706	19576.86	263.2687	19564.66
19587.55	260.9729	19573.15	261.2083	19575.31	260.9641	19576.97	261.2174	19575.81	260.959	19575.81	263.5235	19565.56
19588.82	261.4596	19575.34	261.4574	19576.13	261.2183	19575.51	261.4714	19575.73	261.2014	19578.51	263.7676	19565.2

19589.31	261.7069	19574.2	261.7096	19576.4	261.4701	19574.92	261.7257	19576.34	261.4575	19577.56	264.0219	19566.04
19587.82	261.9605	19576.09	261.957	19576.4	261.7217	19576.62	261.9754	19577.34	261.7111	19576.46	264.2712	19564.38
19586.48	262.2071	19574.71	262.2146	19576.24	261.9711	19575.6	262.2279	19577.21	261.9594	19577.68	264.5239	19565.28
19587.22	262.4616	19575.41	262.4655	19576.32	262.2224	19577.5	262.7155	19576.67	262.2	19575.38	264.7689	19564.07
19589.44	262.7111	19576.15	262.7154	19575.75	262.7122	19576.38	262.9713	19577.72	262.4557	19577.46	265.0221	19566.34
19587.81	262.9642	19573.62	262.9628	19574.32	262.9616	19576.34	263.2249	19576.7	262.7038	19578.96	265.2747	19564.24
19588.91	263.2129	19575.28	263.2171	19575.38	263.2338	19575.42	263.474	19576.5	262.9581	19578.18	265.5318	19563.93
19588.41	263.4667	19573.6	263.4673	19573.53	263.7222	19577.49	263.7276	19576.03	263.2096	19577.38	265.7832	19563.24
19589.13	263.7157	19577.24	263.7185	19575.41	263.9732	19577.4	263.9782	19574.46	263.461	19576.57	266.0377	19566.33
19588.52	263.9693	19576.76	263.968	19576.47	264.2255	19576.15	264.2303	19578.05	263.7201	19577.56	266.2916	19566.08
19586.24	264.2195	19576.37	264.2247	19576.12	264.4835	19575.84	264.4829	19578.23	263.9796	19578.46	266.7761	19564.3
19587.57	264.4747	19574.02	264.4775	19574.69	264.7357	19575.12	264.969	19577.35	264.4667	19577.27	267.0325	19565.27
19587.28	264.7244	19576.46	264.9578	19575.24	264.9854	19574.55	265.2208	19575.93	264.721	19577.44	267.2858	19565.85
19588.76	264.9774	19575.01	265.2158	19574.85	265.2391	19576.04	265.4702	19577.5	264.9771	19577.11	267.5429	19563.35
19586.91	265.4664	19574.35	265.4677	19576	265.7295	19575.32	265.7256	19577.47	265.2296	19576.62	267.7962	19562.95
19586.19	265.7168	19574.54	265.7233	19575.81	265.9815	19575.24	265.98	19575.94	265.4828	19576.07	268.284	19564.85
19587.95	265.971	19575.26	265.976	19573.14	266.2388	19576.5	266.2335	19576.58	265.9754	19577.66	268.5446	19565.05
19589.74	266.2233	19573.78	266.2329	19575.66	266.4923	19576.2	266.4868	19577.66	266.2269	19577.19	268.8028	19565.37
19588.53	266.4891	19576.1	266.7197	19574.96	266.9799	19574.63	266.9745	19577.35	266.482	19578.05	269.2911	19565.33
19587.21	266.9746	19573.14	266.9683	19576.17	267.2343	19576.55	267.2279	19575.93	266.7342	19576.59	269.5498	19563.26
19589.14	267.2256	19575	267.2256	19576.19	267.4892	19575.95	267.4799	19577.48	266.9907	19577.66	269.8029	19564.77
19588.29	267.4812	19576.13	267.4777	19575.68	267.7468	19574.74	267.7366	19576.67	267.4802	19577.49	270.0598	19565.17
19587.36	267.7331	19574.77	267.733	19574.89	268.2382	19575.88	267.9904	19574.78	267.735	19578.13	270.5508	19566.1
19587.73	267.987	19574.75	267.9863	19576.74	268.4959	19576.43	268.4783	19577.33	267.993	19577.78	270.8048	19565.74
19589.31	268.2368	19575.29	268.4711	19575.93	268.7522	19575.61	268.7358	19576.93	268.2457	19575.49	271.0627	19564.42
19587.4	268.4963	19574.97	268.7249	19574.36	269.2434	19576.17	268.9892	19575.95	268.7348	19577.66	271.318	19564.35
19588.22	268.9814	19574.48	268.9783	19574.74	269.5011	19575.82	269.245	19576.59	268.9952	19575.57	271.8044	19562.7
19587.46	269.2349	19574.81	269.2361	19575.58	269.7562	19576.51	269.7368	19577.09	269.2497	19578.85	272.0634	19565.48
19587.58	269.4923	19574.62	269.725	19575.15	270.2469	19576.23	269.9931	19576.38	269.7381	19578.4	272.3199	19563.8
19587.83	269.7449	19575.54	269.9787	19574.29	270.5035	19576.24	270.246	19576.43	269.9972	19576.2	272.5781	19565.4
19588.74	270.004	19577.12	270.2366	19574.38	270.7589	19575.98	270.5008	19576.88	270.2514	19577.83	273.0682	19566
19588.31	270.4963	19575.26	270.4923	19575.1	271.0122	19575.18	270.9914	19575.8	270.5095	19577.75	273.3249	19564.75
19589.55	270.7492	19575.03	270.7476	19574.89	271.5054	19574.61	271.2464	19575.13	271.0118	19578.16	273.5848	19563.66
19588.69	271.0053	19575.08	271.2367	19575.78	271.7627	19575.7	271.5012	19578.19	271.2663	19577.55	274.0744	19562.68
19587.35	271.2597	19575.3	271.4919	19574.06	272.0165	19575.91	271.7605	19573.97	271.761	19576.4	274.3319	19566.52
19589.3	271.5208	19575.12	271.75	19574.27	272.5098	19575.64	272.2511	19576.04	272.0276	19577.27	274.5905	19562.46
19586.52	272.008	19574.59	272.004	19575.66	272.768	19576.52	272.5077	19574.27	272.5195	19577.02	275.0844	19562.3
19586.79	272.2656	19574.96	272.4896	19575.45	273.0219	19574.65	272.7677	19576.71	272.7746	19578.58	275.3456	19563.23
19587.39	272.5258	19573.96	272.7458	19573.19	273.5156	19575.42	273.2588	19576.57	273.0373	19579.51	275.8345	19564.98
19587.72	273.0142	19574.14	273.0007	19575.46	273.774	19577.52	273.5215	19576.7	273.5294	19576.61	276.0965	19563.82
19588.94	273.2715	19575.4	273.2652	19574.08	274.0283	19576.48	274.0128	19575.36	273.7857	19577.67	276.3561	19563.36
19586.77	273.5341	19572.78	273.7534	19575.61	274.5232	19576.33	274.2686	19575.66	274.0499	19577.18	276.8473	19563.72
19587.32	274.0225	19574.61	274.0104	19575.52	274.7818	19575.77	274.5357	19577.27	274.5429	19576.64	277.1079	19562.93
19587.89	274.2807	19573.93	274.2741	19574.49	275.2774	19575.94	275.0293	19574.97	274.7991	19577.63	277.3697	19564.87
19587.33	274.5454	19575.07	274.7621	19576.04	275.537	19574.87	275.2866	19575.8	275.06	19577.59	277.861	19564.04
19588.12	275.0334	19576.19	275.0204	19576.79	275.7951	19576	275.5457	19575.51	275.5521	19577.51	278.1191	19563.71
19587.47	275.2938	19573.85	275.2855	19573.76	276.2895	19577.54	276.0397	19576.06	275.8104	19577.9	278.3809	19564
19587.1	275.5545	19573.35	275.7765	19575.91	276.5487	19574.26	276.2973	19576.31	276.0704	19576.41	278.8723	19563.91
19586.01	276.0439	19573.23	276.0325	19575.89	277.0434	19576.73	276.5576	19576.09	276.5679	19578.22	279.1313	19563.27
19590.36	276.3032	19576.42	276.2956	19574.12	277.3024	19576.72	277.0498	19576.41	276.8268	19577.03	279.3949	19566.49
19587.78	276.5629	19574.44	276.786	19574.96	277.5625	19574.65	277.3113	19576.57	277.0894	19578.71	279.8856	19563.04
19587.82	277.0507	19575.08	277.0445	19575.32	278.055	19575.85	277.8086	19575.85	277.5867	19577.55	280.1446	19564.69
19587.88	277.3107	19575.16	277.3101	19576.56	278.3138	19576.41	278.0685	19575.61	277.8457	19575.62	280.4105	19563.25
19588.22	277.5711	19574.29	277.8075	19576.11	278.573	19576.1	278.5622	19575.4	278.3382	19577.77	280.9045	19562.65
19589.4	278.0575	19574.55	278.0649	19576.61	279.0702	19577.27	278.8261	19576.61	278.6013	19577.12	281.1653	19565.46
19588.55	278.3181	19573.17	278.3281	19577.04	279.3282	19576.58	279.3202	19576.34	279.1011	19578.36	281.6561	19563.56
19586.76	278.5785	19573.88	278.8229	19576.12	279.5907	19575.24	279.5807	19575.3	279.359	19576.52	281.9213	19565.21
19586.39	279.0645	19573.85	279.0805	19575.51	280.0855	19576.28	280.0767	19574.54	279.8547	19578.7	282.4207	19563.71
19589.19	279.3259	19574.45	279.345	19575.65	280.3445	19575.56	280.3454	19576.24	280.1194	19576.28	282.6815	19565.74
19587.12	279.5855	19573.64	279.8341	19575.33	280.8401	19575.77	280.8406	19574.72	280.6176	19575.83	283.173	19563.62
19587.29	280.0935	19572.2	280.0918	19574.6	281.1017	19574.81	281.1008	19575.65	280.8767	19577.05	283.4399	19563.93
19587.99	280.5854	19575.85	280.3555	19575.5	281.5981	19576.31	281.5963	19576.24	281.1401	19577.8	283.9346	19564.81
19587.15	280.8477	19574.28	280.8456	19576.44	281.8588	19575.76	281.8613	19577.27	281.635	19576.76	284.1948	19562.26
19588.12	281.341	19574.64	281.1039	19575.98	282.3525	19575.84	282.3532	19574.62	281.8935	19576.45	284.6869	19562.7
19588.41	281.6031	19573.63	281.3669	19575.12	282.6161	19575.17	282.6164	19575.91	282.3931	19576.7	284.953	19564.51
19588.5	282.0935	19575.63	281.8601	19575.48	283.1162	19577.09	283.1129	19576.8	282.6548	19577.29	285.4563	19564.61
19587.59	282.3575	19575.51	282.1245	19577.2	283.377	19575.7	283.372	19575.74	283.1477	19577.55	285.9512	19563.14
19586.31	282.8488	19574.21	282.6134	19577.59	283.8728	19576.09	283.8694	19576.56	283.4092	19579.08	286.2145	19564.95
19587.67	283.1101	19572.89	282.8761	19574.67	284.1348	19575.4	284.1319	19574.8	283.905	19575.9	286.7075	19564.89
19587.7	283.3759	19575.75	283.1365	19575.93	284.6306	19575.18	284.6309	19577.35	284.1688	19576.67	286.9714	19565.53

19586.89	283.8673	19574.81	283.6272	19576.45	284.8832	19574.82	284.885	19576.34	284.6672	19575.68	287.4719	19564.3
19587.25	284.1294	19573.71	283.8866	19575.79	285.1363	19575.71	285.3794	19575.83	284.9308	19577.29	287.7355	19563.37
19588.95	284.6199	19575.02	284.1481	19577.04	285.3894	19576.86	285.6333	19575.51	285.4297	19576.28	288.2286	19563.77
19589.39	284.8873	19574.46	284.6392	19574.19	285.887	19575.73	285.8897	19574.84	285.6906	19576.21	288.4979	19563.42
19587.08	285.382	19572.05	284.9016	19575.97	286.1406	19575.11	286.3838	19576.21	286.1854	19576.57	288.9941	19563.2
19587.18	285.635	19575.81	285.3981	19574.24	286.399	19573.73	286.6391	19575.31	286.446	19577.96	289.2582	19564.91
19587.8	285.8903	19574.63	285.661	19576.06	286.9012	19575.34	286.8949	19575.42	286.9425	19577.54	289.7514	19562.15
19587.56	286.3864	19575.11	286.1528	19575.17	287.1572	19575.38	287.392	19576.76	287.2035	19577.3	290.0169	19563
19588.96	286.6394	19573.26	286.4195	19574.93	287.6554	19574.66	287.6449	19576.53	287.6996	19578.09	290.5167	19562.78
19587.71	286.8941	19574.71	286.9107	19577.13	287.9084	19575	287.8994	19576.44	287.9665	19574.96	290.781	19563.44
19585.9	287.3885	19575.01	287.1734	19575.51	288.1663	19574.14	288.3958	19576.67	288.4636	19577.35	291.2747	19563.82
19587.1	287.6416	19575.46	287.6649	19573.36	288.6652	19575.96	288.6499	19576.12	288.7295	19578.36	291.5455	19562.08
19585.86	287.8995	19574.13	287.9281	19574.85	288.9185	19575.52	288.9055	19575.61	289.2259	19576.47	292.0447	19562.22
19588.91	288.3969	19575.9	288.423	19575.11	289.1725	19574.4	289.4016	19575.33	289.4902	19576.77	292.31	19562.87
19587.06	288.6527	19575.01	288.6875	19574.25	289.6731	19574.57	289.6604	19576.27	289.9958	19577.03	292.8042	19562.31
19589.94	288.9076	19573.37	289.1806	19575.33	289.9263	19575.61	290.1588	19575.32	290.4942	19576.75	293.0717	19563.14
	289.4044	19574.4	289.4485	19574.86	290.1818	19576.82	290.4123	19574.35	290.7577	19575.82	293.5719	19563.66
	289.6579	19572.74	289.9405	19575.73	290.6843	19576.05	290.6691	19575.24	291.2536	19576.57	293.8368	19563.44
	289.9133	19573.41	290.207	19574.29	290.9416	19575.12	291.1674	19575.4	291.5179	19577.27	294.3319	19564.29
	290.4127	19573.12	290.7025	19573.82	291.443	19574.29	291.4221	19575.03	292.0248	19577.03	294.6022	19562.1
	290.6666	19576.34	290.9693	19574.79	291.6994	19576.54	291.6786	19575.56	292.5221	19575.42	295.1002	19564.49
	291.1585	19574.5	291.4628	19574.48	292.2011	19573.24	292.1778	19577.11	292.7854	19577.47	295.3673	19562.42
	291.4169	19575.76	291.729	19575.1	292.4616	19574.81	292.4325	19576.36	293.2847	19576.5	295.8645	19566.03
	291.6707	19576.18	292.2262	19576.81	292.9593	19577.75	292.9326	19575.5	293.5509	19574.95	296.1314	19562.89
	292.164	19573.6	292.4817	19574.51	293.2163	19575.72	293.1896	19575.05	294.0551	19575.66	296.6301	19564.11
	292.4219	19575.08	292.7383	19574.24	293.7156	19576.26	293.6887	19576.63	294.5554	19575.6	296.8995	19565.57
	292.6775	19573.67	293.2363	19574.43	293.9699	19574.34	293.95	19577.45	294.8191	19575.41	297.4125	19562.9
	293.1703	19574.45	293.4932	19575.75	294.4737	19574.49	294.4493	19575.74	295.3177	19577.67	297.9389	19563.75
	293.4282	19572.28	293.984	19575.72	294.9733	19577.42	294.7068	19576.07	295.5844	19577.84	298.4414	19563.23
	293.685	19571.5	294.2427	19577.24	295.2307	19576.45	295.2075	19576.72	296.0886	19576.15	298.7077	19563.06
	294.179	19574.95	294.5004	19576.1	295.7326	19575.23	295.4634	19575.88	296.588	19575.9	299.205	19563.82
	294.4452	19572.2	294.9949	19576.36	295.9986	19574.12	295.9646	19574.88	296.8541	19577.68	299.4772	19564.51
	294.9427	19576.12	295.2535	19576.62	296.5	19576.54	296.2208	19574.49	297.3521	19576.87	299.9771	19562.25
	295.1984	19573.99	295.755	19574.45	296.77	19574.38	296.7216	19575.31	297.6225	19577.43	300.2468	19563.07
	295.694	19574.84	296.0081	19576.22	297.2728	19575.13	296.9932	19574.93	298.1255	19577.73		
	295.9552	19576.49	296.2701	19576.26	297.5436	19574.25	297.4952	19575.14	298.3929	19575.97		
	296.455	19575.06	296.7698	19575.73	298.0469	19576.99	297.7689	19576.79	298.8926	19576.51		
	296.715	19574.61	297.0235	19574.92	298.5491	19573.76	298.2708	19576.57	299.1641	19578		
	297.2098	19574.3	297.5188	19574.22	298.8191	19573.8	298.5402	19575.35	299.6634	19574.98		
	297.4815	19574.39	297.7757	19576.24	299.3201	19577.49	299.0511	19575.15	299.9338	19576.22		
	297.9795	19572.62	298.031	19576.2	299.5933	19574.72	299.5552	19576.09	300.4358	19576.79		
	298.2524	19574.54	298.5263	19575.66	300.096	19575.97	300.0578	19577.85				
	298.7468	19572.03	298.7869	19574.21								
	299.0176	19572.59	299.2876	19575.63								
	299.5171	19575.14	299.5441	19574.56								
	299.7868	19572.53	300.0402	19574.73								
	300.2838	19572.53										

1.43								1.4				
40								4				
x_2		x_3		x_4		x_5		x_1		x_2		x
t (s)	P (cm^{-1})	t (s)	P (cm^{-1})	t (s)	P (cm^{-1})	t (s)	P (cm^{-1})	t (s)	P (cm^{-1})	t (s)	P (cm^{-1})	t (s)
0	19581.44	0	19580.08	0	19579.56	0	19579.71	0	19570.66	0	19568.39	0
0.71925	19582.15	0.68913	19580.44	0.69888	19580.93	0.67232	19582.12	0.67141	19572.33	0.66148	19570.92	0.67653
0.86001	19578.91	0.85677	19581.45	0.84822	19582.22	0.85057	19584.36	0.85689	19572.65	0.8535	19571.21	0.85504
1.10603	19578.69	1.10335	19581.07	1.10123	19581.5	1.1031	19581.43	1.10259	19570.17	1.10242	19572.8	1.10429
1.35036	19578.16	1.35782	19580.91	1.34882	19579.41	1.3467	19584.03	1.35203	19570.07	1.35202	19571.53	1.35153
1.60009	19579.59	1.60231	19579.61	1.59981	19579.92	1.59734	19580.67	1.60306	19569.3	1.60221	19570.8	1.61399
1.84958	19580.45	1.85421	19579.91	1.8471	19580.79	1.84727	19580.94	1.85777	19570.28	1.85213	19570.78	1.85463
2.10238	19579.23	2.10171	19581.67	2.09925	19579.11	2.0986	19582.03	2.10029	19570.9	2.10219	19571.54	2.10322
2.35029	19579.4	2.35685	19580.86	2.3482	19580.21	2.34781	19582.11	2.35117	19571.88	2.35102	19569.89	2.35341
2.60045	19578.6	2.60088	19582.27	2.59846	19583.1	2.59637	19580.89	2.6024	19570.54	2.60214	19572.2	2.60297
2.85042	19582.06	2.85377	19581.49	2.84823	19581.95	2.84749	19582.5	2.85725	19570.89	2.85231	19572.02	2.85327
3.10072	19580.86	3.10199	19578.71	3.09963	19580.44	3.09736	19582.29	3.10075	19572.05	3.1019	19570.45	3.10105
3.35082	19579.27	3.36021	19579.39	3.34844	19580.97	3.34737	19582.36	3.35128	19571.52	3.35221	19568.16	3.35282
3.60132	19579.69	3.60137	19581.19	3.59837	19577.62	3.59826	19582.65	3.60218	19570.75	3.60221	19570.69	3.6041
3.85049	19579.64	3.85306	19579.78	3.84621	19581.27	3.84796	19582.43	3.85689	19570.15	3.85097	19571.42	3.85215
4.10003	19581.75	4.10186	19579.84	4.10015	19579.4	4.09747	19581.61	4.10243	19571.54	4.1019	19569.69	4.10127
4.34938	19580.08	4.35863	19579.51	4.34916	19581.34	4.34831	19582.61	4.35218	19570.96	4.35338	19572.19	4.35258
4.60109	19581.91	4.60287	19579.55	4.59911	19580.08	4.59863	19584.14	4.60373	19571.26	4.60727	19572.82	4.60311
4.85933	19580.82	4.8546	19580.97	4.84709	19582.41	4.85353	19581.01	4.8577	19571.31	4.85715	19569.97	4.85336
5.10701	19580.39	5.10245	19582.04	5.10034	19579.31	5.10398	19584.14	5.10705	19569.19	5.10205	19569.67	5.1035
5.35054	19579.69	5.3607	19578.76	5.35211	19581.45	5.34709	19583.24	5.35235	19570.19	5.35134	19570.92	5.35211
5.60067	19579.27	5.60458	19578.95	5.60364	19579.2	5.59792	19581.86	5.60283	19569.75	5.60405	19570.08	5.60408
5.85041	19580.32	5.85586	19579.16	5.85109	19580.85	5.84888	19582.48	5.85715	19568.17	5.85273	19573.46	5.85419
6.10076	19579.67	6.10243	19579.2	6.0987	19579.4	6.09997	19581.75	6.10236	19569.25	6.10148	19571.14	6.10384
6.35129	19580.29	6.3582	19583.56	6.34833	19580.11	6.34856	19583.18	6.35273	19568.29	6.35232	19569.3	6.35429
6.60199	19578.39	6.60273	19580.68	6.59931	19582.01	6.59847	19581.15	6.60435	19572.69	6.605	19571.45	6.60339
6.8514	19580.6	6.85583	19580.48	6.84858	19580.19	6.84906	19580.86	6.85755	19568.7	6.85442	19570.1	6.85331
7.10103	19582.21	7.10379	19581.87	7.10216	19579.53	7.09924	19583.75	7.10184	19571.91	7.10379	19572.37	7.10634
7.35067	19579.46	7.3601	19579.14	7.34912	19580.38	7.34806	19583.68	7.35187	19569.56	7.35249	19571.12	7.36095
7.6028	19580.19	7.60317	19579.54	7.59971	19582.76	7.59943	19583.13	7.60367	19568.74	7.60472	19570.26	7.60885
7.85176	19581.31	7.85491	19579.74	7.84879	19580.63	7.84953	19581.17	7.85787	19568.73	7.85403	19571.27	7.85352
8.10151	19581.48	8.10359	19582.36	8.10083	19580.35	8.09889	19582.67	8.10383	19571.08	8.10402	19570.67	8.10355
8.35134	19578.89	8.3594	19579.79	8.35098	19582.15	8.34923	19582.94	8.35365	19570.18	8.35454	19570.38	8.35525
8.6014	19580.52	8.60323	19584.02	8.60128	19581.78	8.59898	19580.82	8.6034	19570.58	8.60539	19571.21	8.6042
8.85326	19577.65	8.85499	19581.56	8.84907	19579.41	8.85363	19583.41	8.85761	19570.34	8.85353	19571.66	8.85405
9.10345	19580.75	9.10364	19580.98	9.0998	19579.61	9.10116	19580.28	9.10353	19571.24	9.10368	19570.25	9.10299
9.35145	19581.01	9.36051	19581.29	9.34932	19582.72	9.34871	19580.95	9.35407	19568.92	9.35404	19570.53	9.35436
9.60193	19579.39	9.60479	19582.34	9.60044	19581.27	9.59972	19582.7	9.60478	19572.79	9.60555	19569.49	9.60483
9.85152	19582.32	9.85678	19580.6	9.8496	19581.85	9.84921	19582.34	9.85831	19571.83	9.85481	19570.4	9.85474
10.11025	19581.13	10.10401	19580.95	10.10032	19581.46	10.10179	19581.97	10.10287	19570.91	10.10356	19570.92	10.10458
10.353	19582.2	10.36101	19580.54	10.35024	19582.6	10.3508	19583.8	10.35434	19570.68	10.35462	19571.19	10.35402
10.60568	19582.11	10.60634	19579.78	10.60145	19583.04	10.60033	19582.4	10.60452	19570.56	10.60646	19571.37	10.60532
10.85222	19578.33	10.85868	19579.91	10.85134	19579.44	10.8505	19582.5	10.8597	19569.61	10.85587	19569.88	10.8553
11.10269	19582.37	11.10415	19579.67	11.1038	19579.62	11.10285	19583	11.10485	19570.37	11.10505	19570.12	11.10579
11.35242	19578.23	11.35919	19579.63	11.35177	19580.7	11.35124	19584.02	11.35469	19570.39	11.3542	19571.83	11.3552
11.60441	19581.41	11.60569	19582.07	11.60228	19579.48	11.60137	19583.76	11.60454	19570.47	11.61115	19570.59	11.60549
11.86151	19580.15	11.85809	19578.45	11.85045	19579.63	11.85782	19582.85	11.86235	19571.59	11.86469	19570.72	11.85648
12.10477	19579.38	12.10608	19579.53	12.10207	19582.12	12.10134	19582.56	12.10548	19569.69	12.10787	19569.62	12.10685
12.35266	19582.31	12.35979	19579.87	12.35211	19580.53	12.35098	19581.01	12.35539	19570.51	12.35857	19570.78	12.35676
12.60622	19579.88	12.60469	19581.85	12.60376	19579.8	12.60455	19583.91	12.60374	19570.74	12.60648	19571.93	12.60574
12.85293	19582.53	12.85727	19580.2	12.85271	19580.23	12.85155	19581.66	12.86619	19568.04	12.85548	19569.83	12.8547
13.11024	19579.56	13.10572	19579.96	13.10146	19580.64	13.10142	19581.74	13.10543	19567.88	13.10587	19571.31	13.10476
13.35348	19579.7	13.36008	19578.43	13.3513	19577.81	13.3504	19581.69	13.35538	19571.17	13.3551	19571.96	13.36353
13.60567	19578.05	13.60426	19578.64	13.60252	19582.2	13.601	19580.69	13.60921	19570.75	13.60847	19569	13.60658
13.85314	19581.47	13.85745	19580.31	13.85257	19581.99	13.85202	19581.16	13.85931	19570.39	13.85604	19572.81	13.85585
14.10323	19580.97	14.10701	19579.51	14.10338	19583.21	14.1025	19583.56	14.10523	19571.3	14.1048	19572.25	14.10478
14.3533	19580.96	14.36344	19579.93	14.35135	19580.42	14.35044	19582.66	14.35622	19570.44	14.35462	19570.69	14.35593
14.60517	19581.13	14.61051	19582.85	14.60199	19581.23	14.60098	19581.57	14.60649	19571.07	14.60681	19571.88	14.60604
14.85446	19580.26	14.85859	19580.06	14.85137	19580.32	14.85264	19582.74	14.85909	19570.91	14.85606	19571.27	14.85681
15.10434	19580.49	15.10552	19578.73	15.10448	19580.52	15.10328	19581.98	15.10494	19569.37	15.10626	19570.57	15.10634
15.35404	19580.61	15.36078	19579.86	15.35267	19579.67	15.35084	19582.15	15.3564	19570.25	15.35588	19569.25	15.35602
15.60572	19580.86	15.60758	19581.54	15.60289	19583.17	15.60186	19580.28	15.60759	19569.35	15.60783	19570.9	15.60732
15.85585	19581.33	15.86095	19583.07	15.85089	19580.7	15.85937	19580.14	15.86261	19569.52	15.8572	19570.06	15.85685
16.10664	19580	16.10797	19580.74	16.10496	19579.3	16.1038	19582.33	16.10572	19568	16.10779	19569.99	16.10859

16.3546	19580.83	16.3625	19580.38	16.35408	19581.12	16.35274	19580.37	16.35773	19570.99	16.35708	19570.39	16.35809
16.60504	19579.32	16.60597	19583.25	16.60387	19580.4	16.60243	19579.9	16.60707	19572.54	16.60656	19571.54	16.60769
16.85427	19581.65	16.85908	19580.08	16.85785	19578.98	16.85307	19581.35	16.86055	19571.32	16.85651	19571.51	16.85678
17.10523	19580.25	17.11231	19581.66	17.10417	19579.72	17.10227	19583.73	17.10755	19570	17.10996	19571.38	17.10726
17.35506	19582.77	17.36288	19580.41	17.35285	19581.87	17.35118	19584.05	17.35565	19568.87	17.35674	19569.4	17.35757
17.60492	19580.01	17.60706	19578.94	17.60426	19581.39	17.60226	19583.08	17.6077	19570.17	17.60938	19568.91	17.6085
17.85543	19580.79	17.85969	19580.06	17.85262	19581.09	17.85311	19582.93	17.86235	19568.29	17.85756	19569.01	17.85805
18.10474	19578.15	18.10819	19579.6	18.10287	19580.34	18.10269	19581.77	18.1069	19572.62	18.10673	19571.46	18.10641
18.35694	19579.72	18.36517	19582.75	18.35336	19582.38	18.35169	19581.58	18.35655	19570.45	18.35692	19570.9	18.35683
18.60727	19578.98	18.61152	19579.69	18.60373	19580.94	18.60364	19581.25	18.60746	19567.82	18.60933	19571.66	18.60725
18.85528	19580.28	18.86015	19579.91	18.85309	19580.86	18.85437	19582.47	18.86306	19571.23	18.85775	19572.02	18.85856
19.10566	19580.5	19.10686	19581.07	19.10325	19578.85	19.10432	19582.84	19.1084	19571.09	19.10637	19572.39	19.10895
19.35421	19579.65	19.36258	19580.14	19.35348	19580.29	19.35154	19581.74	19.35813	19568.49	19.35702	19570.74	19.35729
19.60624	19579.61	19.60879	19579.85	19.60493	19578.57	19.60471	19581.78	19.60704	19572.06	19.60933	19568.83	19.60828
19.85612	19580.58	19.86069	19579.66	19.85304	19579.91	19.85317	19580.45	19.86053	19569.38	19.85844	19572.22	19.85784
20.10711	19578.34	20.10791	19580.3	20.10557	19581.23	20.10756	19582.32	20.10805	19569.09	20.10975	19570.67	20.10974
20.35576	19581.28	20.36399	19579.44	20.35441	19581.17	20.35376	19584.09	20.3586	19569.53	20.3578	19570.54	20.36012
20.60583	19580.87	20.60944	19582.56	20.60426	19582.01	20.60414	19581.4	20.60981	19571.37	20.61174	19570.55	20.60869
20.85675	19579.78	20.86175	19580.64	20.85431	19578.93	20.8608	19580.63	20.86396	19567.27	20.85853	19571.46	20.85854
21.1158	19581.69	21.10972	19579.49	21.10545	19579.91	21.10521	19580.59	21.10793	19571	21.10971	19568.93	21.10868
21.35756	19579.08	21.36472	19580.35	21.35572	19582.02	21.35429	19582.77	21.35902	19568.21	21.3592	19571.6	21.35971
21.60682	19582.16	21.60976	19579.87	21.60579	19579.73	21.60609	19580.32	21.60949	19567.98	21.61078	19569.83	21.61054
21.85741	19581.67	21.86064	19581.4	21.85974	19581.02	21.8554	19583.51	21.86458	19571.04	21.85955	19570.95	21.85875
22.10874	19579.46	22.10908	19579.14	22.1061	19581.7	22.1053	19580.62	22.10832	19572.72	22.11352	19570.95	22.10925
22.35746	19580.6	22.36658	19581.14	22.35536	19580.46	22.35519	19580.98	22.35874	19569.13	22.36086	19569.82	22.35971
22.60942	19579.87	22.61055	19580.81	22.60675	19579.98	22.6063	19581.77	22.60994	19571.85	22.61285	19572.78	22.61029
22.85686	19580.81	22.86137	19581.09	22.8556	19578.95	22.85598	19579.65	22.86354	19569.25	22.86017	19573.63	22.86053
23.10747	19580.68	23.10903	19579.62	23.10575	19582.38	23.10553	19580.57	23.10904	19570.65	23.1098	19570.66	23.11016
23.3572	19580.5	23.36462	19579.85	23.35506	19581	23.35415	19582.43	23.35876	19572.76	23.35897	19572.12	23.35971
23.60938	19580.5	23.61126	19583.93	23.60529	19582.07	23.60551	19581.46	23.61012	19570.68	23.61158	19570.19	23.61008
23.85843	19579.36	23.86209	19581.23	23.85535	19580.61	23.85842	19579.48	23.86708	19567.67	23.86016	19573.07	23.86059
24.10887	19579.68	24.10966	19580.07	24.10996	19580.74	24.10716	19579.59	24.11051	19570.91	24.11118	19570.75	24.1114
24.35669	19580.49	24.36538	19579.72	24.35803	19580.72	24.35466	19582.27	24.36034	19570.42	24.35981	19572.46	24.36108
24.60753	19581.44	24.60957	19582.46	24.60735	19582.21	24.60456	19582.1	24.6095	19568.24	24.61112	19571.63	24.60855
24.85918	19581.47	24.86281	19581.45	24.85626	19580.73	24.85721	19581.63	24.86578	19568.15	24.8614	19571.13	24.85899
25.1084	19577.72	25.11041	19580.86	25.10769	19579.76	25.10711	19583.37	25.10973	19571.01	25.10979	19570.91	25.10963
25.35656	19581.31	25.36472	19579.95	25.35614	19581.18	25.35638	19583.45	25.35864	19572.48	25.35991	19571.21	25.36035
25.60834	19579.74	25.61013	19582.06	25.6084	19579.02	25.60773	19581.14	25.60871	19571.22	25.61425	19571.98	25.6113
25.85768	19583.18	25.86194	19581.47	25.85418	19581	25.85732	19581.25	25.87048	19570.12	25.85946	19569.96	25.86018
26.11057	19579.17	26.11135	19582.12	26.10812	19580.69	26.10573	19580.94	26.10976	19570.93	26.11022	19569.52	26.12115
26.35772	19580.41	26.3671	19581.41	26.35694	19581.62	26.35536	19581.95	26.36022	19570.32	26.35932	19570.98	26.36093
26.60696	19580.71	26.6105	19579.84	26.60816	19580.59	26.60827	19583.41	26.6098	19571.18	26.61351	19571.2	26.61089
26.85862	19581.82	26.86216	19581.25	26.85556	19580.42	26.85706	19583.02	26.8646	19572.03	26.85995	19573.48	26.86144
27.10825	19579.34	27.11064	19581.62	27.10628	19580.96	27.10592	19581.74	27.11082	19569.85	27.1117	19571.88	27.11091
27.35933	19580.25	27.36733	19582.57	27.35641	19580.57	27.35525	19581.57	27.36098	19572.51	27.35947	19570.65	27.36048
27.60929	19581	27.6109	19580.38	27.6074	19580.88	27.60599	19582.03	27.61055	19571.06	27.61274	19570.85	27.6119
27.85856	19578.4	27.86266	19579.24	27.85663	19581.7	27.8579	19582.38	27.86373	19568.38	27.86101	19570.06	27.86104
28.10905	19579.87	28.1101	19580.94	28.10765	19578.9	28.10674	19581.85	28.11009	19569.83	28.11158	19569.25	28.11204
28.35846	19580.76	28.3678	19582.46	28.35618	19581.09	28.35525	19583.42	28.36004	19570.31	28.35999	19571.03	28.3607
28.61096	19578.39	28.61107	19579.66	28.60746	19580.38	28.6055	19580.82	28.61136	19568.98	28.61393	19569.44	28.61242
28.85997	19579.54	28.86283	19582.51	28.85613	19582.23	28.86645	19581.06	28.86661	19569.34	28.86056	19570.36	28.86208
29.10851	19580.41	29.11004	19581.57	29.10767	19582.55	29.10717	19582.91	29.11242	19569.08	29.11356	19571.55	29.11201
29.35804	19580.21	29.36688	19579.84	29.35796	19579.07	29.35588	19582.27	29.36116	19570.29	29.36046	19571.49	29.36225
29.61075	19579.84	29.61219	19582.43	29.60744	19579.35	29.60585	19581.28	29.61086	19568.22	29.61132	19570.11	29.61188
29.86033	19578.65	29.86437	19580.15	29.85616	19579.22	29.86614	19580.67	29.87018	19568.67	29.86093	19570.77	29.86103
30.1118	19580.29	30.11057	19580.91	30.10968	19581.24	30.10729	19582.56	30.11222	19571.77	30.11225	19572.93	30.11136
30.35944	19581.86	30.36761	19583.11	30.3585	19579.58	30.3592	19580.56	30.36317	19570.02	30.36334	19571.94	30.36371
30.61001	19582.07	30.61299	19579.37	30.60927	19581.59	30.609	19582.03	30.61197	19569.91	30.61686	19571.19	30.61386
30.86097	19581.66	30.86672	19580.68	30.85737	19578.86	30.85845	19582.6	30.86601	19569.19	30.86247	19572.48	30.8626
31.11082	19581.47	31.1134	19580.93	31.10975	19582.23	31.10802	19582.35	31.11245	19569.48	31.1132	19569.94	31.11207
31.36102	19581.54	31.36871	19580.74	31.36037	19580.33	31.35821	19580.84	31.36233	19570.2	31.36391	19571.57	31.36324
31.61107	19579.13	31.6115	19580.95	31.61023	19580.3	31.60963	19581.85	31.61385	19570.34	31.61677	19571.41	31.61548
31.86103	19582.03	31.86531	19580.01	31.85804	19582.19	31.86025	19583.24	31.86484	19571.3	31.86229	19572.73	31.86469
32.1113	19579.2	32.11303	19580.66	32.11186	19582.53	32.10867	19583.72	32.11194	19570.08	32.11575	19572.82	32.11254
32.36018	19581.24	32.39229	19582.13	32.35961	19581.18	32.35835	19582.03	32.36204	19571.49	32.3634	19570.68	32.36489
32.61108	19578.06	32.61226	19580.91	32.61031	19579.86	32.60817	19582.44	32.61229	19571.31	32.61557	19571.36	32.61237
32.86127	19581.54	32.86495	19581.19	32.8582	19579.44	32.85958	19580.66	32.86625	19571.1	32.8634	19570.92	32.86216
33.10997	19582.23	33.11261	19580.74	33.10841	19579.77	33.1083	19577.81	33.11267	19569.49	33.11605	19571.01	33.11435
33.35913	19580.75	33.36964	19581.32	33.35809	19580.96	33.35765	19584.59	33.36235	19572.15	33.36255	19570.96	33.36351
33.61064	19578.96	33.61323	19580.64	33.61095	19580.37	33.60896	19581.95	33.61152	19570.27	33.61535	19571.87	33.6121

33.86131	19581.1	33.86731	19579.89	33.85928	19581.13	33.8611	19581.21	33.86404	19571.65	33.86228	19571.54	33.86286
34.11201	19578.61	34.11297	19580.13	34.10967	19580.18	34.11054	19583.64	34.11285	19569.6	34.11545	19571.61	34.11383
34.35947	19581.26	34.36885	19580.59	34.35809	19578.84	34.35862	19581.1	34.36213	19570.72	34.36341	19572.56	34.365
34.6112	19579.16	34.61294	19579.79	34.6099	19579.41	34.60825	19582.93	34.61283	19569.47	34.6178	19572.22	34.61391
34.86262	19581.82	34.86651	19579.64	34.85881	19581.31	34.86081	19583.52	34.86488	19570.49	34.86198	19570.5	34.86331
35.11393	19579.82	35.11345	19579.25	35.11051	19580.32	35.10957	19581.51	35.11281	19570.54	35.11356	19571.7	35.11143
35.36205	19580.34	35.36921	19581.47	35.36091	19580.29	35.36137	19582.14	35.36369	19570.68	35.36511	19573.29	35.36365
35.61047	19579.8	35.61373	19580.87	35.60999	19580.56	35.61011	19582.71	35.61392	19569.81	35.6201	19569.47	35.61501
35.86112	19580.38	35.86792	19581.09	35.85856	19581.34	35.85881	19579.54	35.86452	19567.66	35.86359	19571.54	35.86407
36.11198	19581.47	36.11537	19579.41	36.11028	19582.55	36.1101	19582.24	36.11302	19570.77	36.11386	19571.13	36.11492
36.36265	19580.74	36.37042	19581.77	36.35994	19580.49	36.35942	19582.94	36.36319	19570.89	36.36418	19571.71	36.36282
36.61233	19581.01	36.61342	19581.9	36.61279	19580.69	36.60951	19582.3	36.61415	19571.79	36.61933	19570.12	36.61492
36.86141	19578.72	36.86734	19580.41	36.86025	19580.5	36.86477	19581.39	36.86594	19571.01	36.86456	19571.21	36.86428
37.11181	19582.3	37.11381	19579.37	37.11287	19581.61	37.10965	19579.98	37.1132	19571.57	37.11772	19571.27	37.11527
37.36145	19582.89	37.37537	19580.75	37.36106	19581.56	37.35961	19581.19	37.36413	19573.57	37.36359	19572.69	37.3733
37.61304	19579.23	37.61513	19580.26	37.61259	19580.26	37.61008	19583.28	37.61428	19570.2	37.61761	19570.79	37.61605
37.86269	19580.45	37.86618	19579.92	37.86048	19580.75	37.86212	19582.23	37.86965	19571.47	37.86467	19571.44	37.86346
38.11172	19580.34	38.11387	19580.59	38.11158	19580.28	38.11	19583.17	38.11365	19571.43	38.11488	19572.33	38.11406
38.36111	19581.43	38.37087	19580.51	38.36053	19581.19	38.35971	19581.79	38.36399	19570.64	38.36388	19573.52	38.36511
38.61218	19579.01	38.61506	19579.86	38.6125	19579.86	38.6099	19581.54	38.61353	19569.99	38.61836	19572.43	38.61606
38.86324	19579.31	38.86791	19580.38	38.86258	19580.78	38.86209	19581.03	38.86879	19571.87	38.86462	19571.61	38.86527
39.11414	19579.77	39.11396	19579.83	39.11131	19580.52	39.11016	19582.69	39.11437	19570.93	39.11509	19570.29	39.11443
39.36128	19580.94	39.36955	19579.17	39.36066	19582.5	39.35889	19581.19	39.36587	19570.71	39.36523	19570.96	39.36405
39.61297	19580.32	39.61504	19579.59	39.6132	19579.02	39.60943	19582.16	39.6152	19566.58	39.62147	19571.95	39.61563
39.86371	19580.14	39.86857	19581.59	39.86162	19579	39.86189	19582.33	39.86558	19569.08	39.86394	19571.32	39.86571
40.11553	19579.12	40.11532	19578.98	40.11177	19580.55	40.112	19581.62	40.11349	19569.98	40.11504	19571.71	40.11624
40.36434	19580.6	40.37225	19581.13	40.36297	19581.25	40.36158	19582.16	40.36611	19571.86	40.36584	19571.13	40.36704
40.61491	19579.98	40.61605	19580.82	40.61919	19579.56	40.61077	19579.89	40.61767	19568.05	40.62166	19570.97	40.61543
40.8634	19579.26	40.87134	19579.56	40.86154	19579.33	40.86434	19582.15	40.86944	19570.34	40.86651	19571.94	40.86751
41.11484	19579.99	41.1174	19584.58	41.11339	19578.22	41.11251	19583.54	41.115	19570.4	41.11564	19570.13	41.11707
41.36533	19580.01	41.37462	19579.23	41.36367	19581.56	41.36168	19580.28	41.36542	19570.64	41.36548	19571.86	41.3674
41.6152	19580.92	41.61637	19580.6	41.61466	19581.04	41.61207	19584.4	41.6162	19571.55	41.62121	19570.24	41.61681
41.8642	19581.4	41.87007	19579.47	41.86289	19581.11	41.86227	19580.6	41.87192	19571.01	41.86812	19570.22	41.86575
42.11859	19580.02	42.11634	19580.36	42.11309	19580.29	42.11375	19581.62	42.11763	19569.31	42.12067	19571.38	42.11775
42.3652	19581.39	42.3736	19581.68	42.36323	19581.62	42.36244	19580.87	42.36759	19569.93	42.36664	19569.1	42.36984
42.61634	19578.74	42.61781	19580.19	42.61477	19579.22	42.61283	19579.74	42.61673	19570.23	42.62054	19569.6	42.61825
42.86605	19580.49	42.87034	19579.53	42.86409	19582.16	42.86561	19582.95	42.86916	19569	42.86595	19572.21	42.86704
43.11433	19581.96	43.11684	19580.2	43.11285	19580.5	43.1125	19581.9	43.11763	19569.53	43.11853	19570.55	43.11745
43.3645	19579.92	43.37399	19580.56	43.36263	19579.95	43.36109	19582.64	43.36825	19570.7	43.36733	19570.83	43.36876
43.61482	19577.9	43.61778	19579.87	43.6142	19578.87	43.61289	19581.62	43.61579	19571.52	43.62201	19571.01	43.61971
43.86658	19582.67	43.87473	19580.58	43.86414	19579.54	43.86763	19580.48	43.86928	19571.53	43.86657	19570.75	43.86862
44.11614	19580.7	44.11704	19582.07	44.11438	19581.29	44.11448	19579.85	44.11781	19570.07	44.11804	19569.8	44.11713
44.36378	19580.95	44.37521	19579	44.36293	19581.84	44.36221	19583.51	44.36925	19569.61	44.36778	19570.75	44.36886
44.61559	19580.86	44.61779	19578.07	44.61417	19580.12	44.61294	19581.43	44.61706	19568.76	44.62193	19569.32	44.6178
44.86778	19579.9	44.87324	19581.88	44.86395	19582	44.86393	19580.38	44.87015	19568.71	44.86881	19572.29	44.86866
45.11729	19581.18	45.11858	19581.44	45.11773	19581.16	45.1149	19582.07	45.11846	19570.58	45.11783	19568.62	45.11952
45.36422	19579.62	45.37574	19581.18	45.36417	19580.87	45.36527	19581.82	45.36839	19569.18	45.3673	19571.6	45.36961
45.61518	19579.66	45.61777	19580.15	45.61489	19581.16	45.61449	19580.68	45.61858	19569.62	45.63011	19570.73	45.61827
45.86635	19579.04	45.87308	19578.51	45.86508	19583.71	45.86942	19580.37	45.86877	19565.64	45.87178	19570.2	45.8683
46.11832	19580.92	46.12063	19580.24	46.11666	19580.35	46.11439	19582	46.11937	19569.98	46.12047	19570.97	46.11916
46.3666	19581	46.37614	19581.17	46.36925	19578.94	46.36383	19582.5	46.36773	19568.57	46.36817	19571.57	46.3727
46.61512	19580.34	46.61719	19581.09	46.61521	19580.2	46.61612	19580.81	46.61972	19568.88	46.63018	19571.31	46.61917
46.86623	19580.35	46.87265	19580.89	46.86784	19580.23	46.86744	19580.29	46.87235	19568.86	46.86829	19570.5	46.86809
47.11816	19579.58	47.11994	19580.26	47.11503	19579.32	47.11385	19582.18	47.11931	19571.2	47.12247	19571.64	47.11978
47.36703	19581.18	47.3761	19581.22	47.36523	19579.88	47.36321	19581.27	47.36785	19570.82	47.36992	19570.47	47.37172
47.61749	19579.31	47.62051	19580.52	47.61643	19583.14	47.61441	19583.59	47.61809	19570.43	47.62425	19568.87	47.62049
47.86672	19580.27	47.87137	19580.26	47.86342	19582.08	47.86617	19580.82	47.87279	19573.37	47.86828	19571.89	47.86827
48.11699	19580.48	48.11908	19579.43	48.11669	19580.96	48.11492	19581.47	48.1208	19571.65	48.11977	19571.7	48.11974
48.36714	19580.4	48.3766	19580.87	48.36588	19581.22	48.36455	19582.69	48.3679	19571.44	48.36917	19572.6	48.37154
48.61648	19580.02	48.61845	19578.83	48.6165	19579.71	48.61281	19581.58	48.61851	19570.94	48.62784	19570.1	48.61933
48.86692	19579.31	48.87428	19579.77	48.86574	19581.32	48.86619	19582.8	48.86945	19571.44	48.86881	19572.56	48.86897
49.11593	19580.88	49.11829	19581.8	49.11496	19581.83	49.11565	19581.64	49.1196	19570.85	49.11995	19572.1	49.11791
49.36518	19583.25	49.37657	19580.6	49.36507	19579.44	49.36954	19582.16	49.37037	19571.91	49.36713	19569.79	49.36973
49.61917	19578.5	49.62111	19582.78	49.61949	19578.9	49.61655	19580.17	49.6199	19569.45	49.62548	19569.92	49.62294
49.86679	19579.58	49.87204	19582.41	49.87597	19581.5	49.86695	19581.58	49.86819	19572.29	49.86922	19572.32	49.86974
50.12969	19579.21	50.11938	19580.89	50.11613	19580.78	50.11394	19583.99	50.11929	19568.98	50.12268	19570.28	50.11922
50.36617	19579.35	50.37797	19580.24	50.36423	19580.59	50.36394	19583	50.36932	19568.53	50.36996	19573.22	50.36994
50.61719	19579.05	50.61867	19578.97	50.61642	19581.5	50.61565	19583.51	50.61892	19570.23	50.62315	19570.18	50.62037
50.86671	19581.97	50.87331	19580.42	50.86566	19582.54	50.86586	19583.46	50.86964	19571.4	50.86853	19570.91	50.86952
51.11843	19582	51.12143	19579.72	51.11804	19580.46	51.11556	19580.98	51.1205	19569.53	51.12117	19571.31	51.12017

51.36674	19580.82	51.39494	19579.99	51.36605	19579.74	51.36476	19583.14	51.36984	19568.12	51.37074	19571.14	51.38103
51.62018	19580.82	51.62024	19580.83	51.6164	19581.25	51.61674	19581.73	51.6205	19570.23	51.62505	19573.35	51.61908
51.86707	19580.07	51.87188	19582.3	51.86565	19580.9	51.86776	19579.74	51.86956	19571	51.86874	19571.69	51.86921
52.12021	19579.77	52.12043	19579.93	52.11668	19580.06	52.11502	19580.25	52.12118	19566.78	52.12433	19570.95	52.1197
52.36761	19580.12	52.37894	19579.39	52.36707	19582.74	52.36386	19581.82	52.3694	19569.44	52.37095	19570.19	52.3718
52.61883	19579.37	52.61962	19579.91	52.61741	19581.78	52.6161	19580.71	52.62063	19568.13	52.62635	19571.65	52.62055
52.86717	19583.17	52.87205	19580.78	52.86608	19582.49	52.87331	19581.95	52.87108	19567.98	52.86932	19572.54	52.86932
53.11948	19580.46	53.11915	19579.75	53.11637	19580.69	53.11636	19582.28	53.11872	19568.73	53.12107	19570.68	53.11991
53.36765	19580.74	53.37744	19578.98	53.36639	19578.48	53.3635	19581.8	53.36993	19572.22	53.37077	19572.34	53.3704
53.61897	19579.85	53.62078	19579.57	53.61789	19581.67	53.61774	19583.57	53.62061	19569.22	53.62615	19570.79	53.62281
53.86983	19578.65	53.87236	19580.54	53.86847	19580.37	53.87649	19582.81	53.87118	19569.23	53.87064	19569.54	53.87079
54.11999	19579	54.1201	19581.5	54.11742	19580.26	54.11622	19583.05	54.12039	19570.7	54.12068	19570.47	54.12081
54.36696	19581.76	54.37788	19580.76	54.36672	19580.36	54.36549	19583.13	54.37058	19571.67	54.37131	19569.86	54.36955
54.61934	19578.57	54.62138	19578.87	54.61858	19583.79	54.61755	19582.6	54.6199	19567.65	54.62866	19571.64	54.62066
54.8693	19579.38	54.87471	19582.06	54.86922	19579.86	54.86737	19580.76	54.87014	19572.31	54.87144	19572.63	54.87175
55.13063	19580.3	55.11938	19579.98	55.11971	19580.21	55.12835	19581.29	55.12222	19571.11	55.12245	19571.84	55.12141
55.36871	19580.85	55.37916	19581.48	55.3681	19581.79	55.36587	19582.46	55.36963	19568.69	55.37134	19570.4	55.38152
55.62013	19582.44	55.62043	19581.91	55.61783	19579.33	55.61911	19581.9	55.61945	19569.62	55.62761	19571.84	55.62257
55.86924	19581.38	55.87487	19581.31	55.87133	19578.82	55.86973	19580.37	55.87058	19569.79	55.87155	19572.75	55.87007
56.12173	19579.59	56.12098	19579.84	56.11971	19582.33	56.11585	19582.36	56.12143	19571.89	56.12291	19571.28	56.12132
56.36894	19581.8	56.37866	19581.38	56.36809	19581.27	56.36587	19583.43	56.373	19567.12	56.37407	19570.92	56.37241
56.62012	19579.21	56.62051	19580.67	56.61771	19580.3	56.61826	19582.46	56.61991	19572.16	56.62803	19571.96	56.62296
56.86805	19580.8	56.87643	19580.25	56.86921	19582.05	56.87279	19579.82	56.87096	19571.5	56.86993	19571.25	56.8704
57.11976	19582.47	57.1286	19579.74	57.12056	19579.39	57.11755	19580.75	57.12121	19569.37	57.12429	19570.42	57.12129
57.3693	19581.33	57.38099	19581.29	57.36976	19582.78	57.36492	19581.04	57.37296	19569.59	57.37296	19571.49	57.37276
57.6201	19580.42	57.63289	19577.94	57.62036	19579.18	57.62012	19582.71	57.62288	19570.16	57.63197	19571.03	57.62498
57.87032	19580.76	57.8746	19580.64	57.87224	19580.47	57.87295	19582.99	57.87126	19571.95	57.8753	19571.09	57.87148
58.12041	19578.55	58.1223	19579.93	58.11844	19579.91	58.11909	19581.23	58.12058	19570.9	58.12331	19570.21	58.122
58.36812	19579.51	58.38072	19581.28	58.36998	19581.04	58.37728	19581.04	58.37654	19569.46	58.37125	19572.82	58.37288
58.61991	19581.46	58.62177	19579.46	58.62078	19579.84	58.62611	19580.65	58.62217	19570.46	58.62885	19573.07	58.62344
58.87121	19578.38	58.87532	19579.65	58.87146	19580.38	58.88059	19581.26	58.87389	19570.56	58.87421	19569.08	58.8726
59.12357	19576.73	59.12311	19579.52	59.12045	19580.16	59.12665	19583.46	59.12107	19569.32	59.12368	19570.56	59.12344
59.36889	19579.19	59.3806	19580.55	59.36898	19580.75	59.37712	19582	59.37322	19572.18	59.37312	19571.18	59.37285
59.61958	19580.75	59.62184	19578.88	59.62132	19581.35	59.61901	19579.36	59.62196	19572.45	59.62984	19570.31	59.62454
59.8706	19578.75	59.87438	19581.11	59.87216	19580.57	59.87313	19580.61	59.87265	19571.3	59.87161	19569.8	59.87307
60.12259	19579.17	60.12222	19579.65	60.11846	19582.67	60.1176	19581.05	60.12366	19569.46	60.12376	19571.82	60.1228
60.37291	19582	60.38166	19581.04	60.36971	19579.21	60.3691	19580.88	60.37425	19569.82	60.37446	19571	60.37473
60.62063	19579.05	60.62529	19580.3	60.62176	19580.76	60.6211	19581.94	60.62371	19569.59	60.63202	19573.45	60.62518
60.87218	19581.43	60.88068	19580.42	60.87401	19580.68	60.87557	19581.87	60.87351	19572.19	60.87307	19570.02	60.88114
61.12319	19581.71	61.1242	19583.7	61.12261	19581.93	61.11987	19581.03	61.12409	19571.73	61.1266	19571.02	61.12557
61.37231	19579.39	61.38197	19582.37	61.37019	19580.27	61.36826	19578.89	61.37519	19569.86	61.37474	19573.21	61.37648
61.6226	19580.75	61.62578	19580.96	61.62117	19580.3	61.62008	19584.87	61.62452	19570.16	61.63347	19570.2	61.62472
61.87218	19580.84	61.87864	19580.58	61.87231	19581.89	61.8766	19582.14	61.87449	19571.61	61.87528	19571.94	61.87402
62.12424	19581.43	62.12728	19579.51	62.12152	19580.53	62.12187	19582.37	62.12528	19569.59	62.12721	19571.43	62.12653
62.37226	19580.89	62.38313	19580.2	62.37269	19580.24	62.36936	19583.67	62.376	19570.55	62.37399	19572.08	62.3769
62.62425	19581.8	62.62465	19582.06	62.62331	19580.66	62.61973	19580.9	62.62578	19569.6	62.63139	19571.44	62.62527
62.87861	19579.72	62.87828	19579.51	62.8738	19582.17	62.88673	19581.22	62.87478	19568.5	62.87587	19571.48	62.87437
63.12261	19579.99	63.12715	19581.86	63.12373	19581.12	63.12464	19582.01	63.12571	19570.72	63.12755	19572.68	63.12534
63.37223	19579.89	63.38122	19581.28	63.37268	19580.84	63.37179	19580.77	63.37435	19570.99	63.37392	19571.13	63.37693
63.62414	19579.7	63.62443	19582.33	63.62363	19578.98	63.62092	19581.93	63.62607	19567.34	63.63415	19573.01	63.62814
63.87433	19582.23	63.8778	19578.8	63.87275	19581.16	63.87599	19582.48	63.8757	19569.5	63.87559	19572.24	63.87521
64.12457	19577.94	64.12746	19580.98	64.12254	19581.77	64.1215	19581.09	64.12409	19569.28	64.12726	19570.66	64.12655
64.37126	19580.96	64.38569	19579.68	64.37348	19581.23	64.37144	19581.9	64.37599	19571.31	64.3759	19568.91	64.3767
64.62399	19581.42	64.63231	19579.81	64.62151	19579.35	64.6205	19581.81	64.62369	19570.16	64.6309	19570.93	64.62727
64.87376	19580.74	64.87793	19581.59	64.87279	19581.28	64.87781	19581.04	64.87576	19569.66	64.87386	19571.76	64.8751
65.12489	19583.35	65.12581	19579.39	65.12373	19581.29	65.11893	19579.96	65.12504	19568.65	65.12585	19569.77	65.12423
65.37113	19580.26	65.38264	19579.63	65.37124	19582.68	65.36861	19582.07	65.37318	19572.09	65.37511	19573.41	65.37505
65.62225	19580.32	65.62631	19582.34	65.62266	19580.12	65.62042	19582.99	65.62424	19568.99	65.63357	19572.67	65.62657
65.87341	19581.39	65.879	19581.58	65.8734	19582.37	65.87972	19582.81	65.87461	19570.98	65.87358	19571.94	65.87615
66.12368	19579.83	66.12556	19580.83	66.12172	19579.5	66.12167	19580.64	66.12617	19568.52	66.12612	19571.65	66.12675
66.37289	19580.16	66.38231	19579.38	66.37101	19581.26	66.36866	19583.66	66.37592	19569.99	66.37447	19572.68	66.37756
66.62606	19579.97	66.62522	19582.72	66.62264	19581.18	66.6226	19582.87	66.62417	19571.84	66.6552	19571.96	66.62562
66.87227	19579.35	66.8789	19578.69	66.87426	19581.86	66.8765	19582.72	66.87361	19570.86	66.88182	19571.14	66.87455
67.12386	19579.67	67.12556	19579.88	67.12452	19582.47	67.12318	19581.25	67.13642	19571.81	67.12822	19571.26	67.12644
67.37342	19582.16	67.3801	19581.1	67.37151	19579.38	67.37072	19582.99	67.37702	19569.08	67.37453	19571.53	67.37781
67.62444	19581	67.62571	19579.35	67.62205	19580.74	67.62179	19580.85	67.62797	19570.21	67.63237	19571.66	67.62779
67.87474	19580.22	67.87795	19579.2	67.88209	19580.72	67.87744	19580.98	67.87559	19569.5	67.87617	19572.05	67.87432
68.12412	19580.77	68.12676	19580.3	68.12703	19582.77	68.1227	19581.75	68.1238	19570.96	68.12687	19572.42	68.12596
68.37163	19581.42	68.38403	19584.67	68.37234	19581.26	68.37194	19580.59	68.37695	19571.16	68.37461	19570.94	68.37853
68.6246	19578.69	68.62547	19580.09	68.62393	19580.04	68.62103	19582.71	68.62796	19570.89	68.63278	19570.7	68.62886

68.87524	19580.79	68.87966	19579.97	68.87276	19581.88	68.8758	19581.94	68.87717	19572.17	68.87621	19571.28	68.87771
69.12477	19581.77	69.12778	19581.32	69.12331	19579.88	69.1209	19580.95	69.12727	19569.52	69.12889	19569.89	69.12625
69.37212	19581.71	69.38475	19579.39	69.37412	19578.98	69.37212	19580.98	69.37887	19570.39	69.37696	19571.74	69.37651
69.62529	19580.64	69.6256	19582	69.62476	19580.43	69.62325	19580.27	69.62578	19568.81	69.63454	19572.65	69.62819
69.87472	19579.24	69.88011	19580.33	69.87715	19579.76	69.87928	19580.07	69.87733	19570.43	69.87665	19569.36	69.87745
70.12572	19580.43	70.12728	19581.24	70.13624	19582.23	70.12103	19581.34	70.1279	19569.1	70.1283	19570.34	70.12857
70.37264	19580.61	70.38578	19580.92	70.37301	19581.59	70.37093	19581.56	70.37814	19571.13	70.37729	19570.01	70.37728
70.62265	19580.58	70.62573	19580.89	70.62454	19580.43	70.62263	19580.55	70.6264	19570.09	70.63399	19572.01	70.62929
70.87405	19581.43	70.87886	19580.69	70.87643	19582.23	70.87766	19582.46	70.87672	19570.55	70.87574	19570.91	70.87816
71.12649	19579.82	71.12803	19581.54	71.12494	19580.93	71.12291	19580.2	71.12731	19570.3	71.12773	19571.44	71.12842
71.37583	19580.75	71.38504	19579.84	71.37224	19580.45	71.37148	19582.36	71.37902	19571.59	71.3775	19570.44	71.37916
71.6257	19578.65	71.62759	19582.62	71.62371	19580.91	71.6214	19581.68	71.62625	19571.86	71.636	19570.47	71.62884
71.87378	19580.61	71.87958	19578.38	71.87815	19581.92	71.88344	19579.62	71.87537	19571.08	71.87769	19571.34	71.87652
72.12558	19581.01	72.1272	19582.73	72.12509	19578.97	72.12534	19582.18	72.1378	19569.88	72.13145	19571.31	72.12719
72.37624	19579.55	72.386	19581.14	72.37419	19581.28	72.37276	19582.34	72.37762	19569.27	72.3774	19570.68	72.37886
72.62527	19582.65	72.62789	19580.86	72.62492	19578.57	72.62512	19581.55	72.62865	19570.69	72.63618	19570.8	72.6297
72.87509	19580.28	72.88464	19580.55	72.8778	19578.36	72.87854	19581.97	72.8784	19570.29	72.87859	19571.04	72.87838
73.1251	19580.71	73.12718	19581.33	73.12522	19580.36	73.12328	19579.6	73.13374	19570.32	73.13041	19570.05	73.1283
73.37529	19579.76	73.38738	19580.24	73.3745	19580.36	73.37284	19584.08	73.37914	19570.56	73.37688	19571.47	73.37921
73.62659	19579.25	73.62867	19580.17	73.6273	19583.61	73.62569	19582.48	73.62827	19570.52	73.63581	19571.24	73.62999
73.876	19579.85	73.8836	19580.76	73.87732	19578.66	73.88208	19583.04	73.87788	19571.38	73.87795	19571.36	73.87842
74.13647	19580.33	74.12997	19580.39	74.12545	19581.12	74.12362	19581.4	74.1277	19570.31	74.13136	19571.93	74.12724
74.37617	19580.82	74.38846	19581.19	74.37557	19582.03	74.37181	19583.51	74.3818	19570.23	74.37816	19571.53	74.37739
74.62472	19581.45	74.62792	19582.76	74.62652	19581.14	74.62334	19583.41	74.62779	19571.21	74.63673	19571.03	74.62869
74.8768	19582.27	74.88283	19580.28	74.87836	19580.42	74.8799	19581.95	74.87879	19570.33	74.87711	19571.74	74.88047
75.12781	19578.87	75.12983	19579.27	75.12585	19582.07	75.1251	19582.38	75.12929	19572.66	75.1302	19570.62	75.12988
75.37826	19582.07	75.38878	19579.4	75.37464	19579.68	75.37155	19582.61	75.3775	19567.75	75.3802	19570.19	75.38074
75.62441	19578.4	75.62788	19579.87	75.62598	19582.1	75.62367	19582.29	75.62773	19571.59	75.63817	19571.18	75.62952
75.87681	19579.86	75.88375	19583.23	75.87968	19580.19	75.88332	19581.01	75.87848	19569.39	75.8784	19569.98	75.87869
76.12639	19579.82	76.129	19580.07	76.12667	19580.89	76.12622	19582.38	76.12924	19568.07	76.1324	19570.71	76.13025
76.37754	19579.59	76.38807	19580.35	76.37521	19580.66	76.37297	19581.09	76.37959	19572.21	76.37978	19570.02	76.38055
76.62859	19578.35	76.62974	19582.17	76.6256	19578.71	76.62342	19583.24	76.62838	19569.66	76.64064	19570.2	76.62824
76.87922	19578.37	76.88305	19582.89	76.87911	19581.62	76.88236	19581.56	76.87891	19571.89	76.8777	19572.29	76.87848
77.12647	19582.05	77.12875	19578.97	77.12896	19578.32	77.12585	19578.3	77.14003	19570.16	77.13337	19568.31	77.13167
77.37848	19579.49	77.38857	19579.86	77.37548	19580.29	77.37428	19582.28	77.37963	19569.39	77.37884	19569.49	77.38198
77.62846	19580.61	77.63066	19579.88	77.6259	19581.17	77.62331	19583.29	77.63118	19570.34	77.64105	19570.76	77.63105
77.87816	19578.42	77.88096	19580.71	77.87899	19581.29	77.88142	19580.4	77.87965	19569.8	77.88579	19569.77	77.87901
78.12687	19581.04	78.12911	19580.81	78.12833	19580.36	78.12516	19583.18	78.12855	19569.29	78.13181	19566.91	78.12907
78.37707	19580.59	78.38702	19582.47	78.37692	19579.65	78.37591	19581.55	78.37921	19567.17	78.37818	19570.83	78.38044
78.62835	19577.93	78.62993	19581.66	78.62604	19580.75	78.62642	19581.79	78.63046	19571.63	78.64456	19571.69	78.63111
78.87911	19579.44	78.88435	19580.44	78.87818	19581.61	78.88262	19581.46	78.88065	19570.34	78.88088	19571.05	78.88023
79.13984	19582.41	79.12902	19580.58	79.1317	19581.67	79.12527	19582.07	79.12987	19569.3	79.13257	19571.93	79.13036
79.37685	19577.72	79.38497	19582.24	79.37664	19577.44	79.3753	19580.96	79.38093	19568.68	79.37899	19568.13	79.37975
79.62629	19579.49	79.63043	19580.48	79.62892	19580.08	79.62592	19581.29	79.62916	19570.33	79.63909	19570.57	79.63178
79.87713	19579.58	79.88683	19580.14	79.87802	19580.39	79.88357	19583.78	79.88142	19570.65	79.87938	19569.01	79.88166
80.14242	19581.83	80.12999	19579.47	80.1285	19579.41	80.12513	19581.13	80.1329	19569.31	80.13374	19570.2	80.13171
80.39294	19581.7	80.38876	19579.95	80.3801	19581.3	80.37711	19580.53	80.38255	19567.99	80.38413	19572.04	80.39496
80.6307	19581.62	80.63072	19581.02	80.62832	19578.3	80.62509	19582.27	80.63022	19570	80.63849	19571.67	80.62981
80.88265	19579.78	80.88671	19580.11	80.88058	19581.78	80.88895	19582.21	80.88033	19570.87	80.87894	19573.16	80.87976
81.12887	19581.23	81.13173	19581.59	81.12958	19581.24	81.13388	19580.99	81.13189	19569.6	81.13288	19570.04	81.13169
81.38007	19579.2	81.38611	19578.61	81.37655	19580.55	81.37502	19582.61	81.38258	19569.53	81.38205	19571.96	81.38328
81.63545	19581.05	81.63073	19579.48	81.62882	19580.24	81.62641	19581.32	81.63	19571.53	81.64005	19571.07	81.63274
81.88882	19579.4	81.88524	19581.12	81.88263	19580.8	81.88367	19579.6	81.8817	19570.8	81.88889	19570.93	81.88119
82.13877	19581.09	82.13217	19579.44	82.13011	19581.07	82.12713	19579.65	82.13213	19571.79	82.13746	19571.9	82.13134
82.3771	19579.5	82.38541	19577.97	82.37665	19580.23	82.37703	19580.83	82.38281	19569.35	82.38084	19571.09	82.38337
82.63541	19580.33	82.63002	19580.16	82.62873	19581.63	82.62443	19584.12	82.63246	19567.81	82.64034	19568.21	82.63319
82.89162	19579.86	82.88478	19580.18	82.88767	19579.64	82.88173	19583.93	82.88051	19568.73	82.88193	19570.84	82.88441
83.13087	19580.28	83.13281	19580.46	83.13469	19581.11	83.1281	19579.81	83.13119	19567.7	83.13642	19569.06	83.13003
83.37978	19579.71	83.39235	19580.4	83.37917	19581.8	83.3781	19581.57	83.3818	19568.13	83.38275	19569.24	83.38336
83.62936	19579.45	83.63069	19579.68	83.62936	19579.32	83.62903	19580.94	83.63291	19568.08	83.63937	19570.51	83.63316
83.89122	19578.86	83.88668	19580.93	83.88253	19579.68	83.88368	19579.93	83.88232	19567.47	83.8817	19569.15	83.88312
84.13862	19579.06	84.13204	19578.67	84.13065	19579.91	84.12823	19583.02	84.13048	19570.62	84.13501	19570.22	84.13378
84.37791	19579.14	84.39149	19580.89	84.38029	19579.33	84.37861	19582.22	84.38352	19567.99	84.38355	19571.58	84.39544
84.62935	19578.37	84.63271	19580.5	84.63106	19579.74	84.6298	19583.33	84.63421	19569.38	84.6424	19569.34	84.6336
84.88161	19581.87	84.88729	19581.08	84.88183	19579.67	84.88383	19582.14	84.88441	19567.78	84.8825	19570.98	84.88308
85.13191	19580.1	85.13159	19579.41	85.13182	19578.49	85.12766	19580.14	85.13341	19567.12	85.13536	19568.93	85.1343
85.37872	19580.53	85.39042	19580.35	85.37969	19579.24	85.37867	19582.39	85.38094	19567.63	85.38341	19570	85.38632
85.62886	19582.26	85.63265	19580.24	85.62984	19581.3	85.62863	19581.97	85.63373	19568.9	85.64266	19570.71	85.63533
85.88009	19578.74	85.88843	19577.54	85.8819	19580.96	85.8838	19580.78	85.88195	19569.6	85.89187	19568.69	85.88365
86.13172	19580.75	86.13396	19579.56	86.13089	19580.08	86.12739	19581.21	86.1336	19568.42	86.13374	19569.72	86.13423

86.38027	19580.3	86.39056	19581.53	86.37894	19580.24	86.37803	19581.31	86.38435	19568.99	86.38232	19569.93	86.38564
86.62982	19578.84	86.63254	19579.35	86.63046	19579.31	86.63185	19579.35	86.63371	19567.64	86.6413	19571.42	86.63353
86.87948	19578.46	86.88618	19579.5	86.8857	19579.18	86.88425	19580.34	86.88245	19568.2	86.88437	19568.72	86.88344
87.13141	19578.65	87.13419	19580	87.13078	19580.39	87.12799	19580.15	87.13489	19569.31	87.13819	19569.16	87.13395
87.38197	19577.53	87.38971	19580.53	87.37943	19581.55	87.37792	19581.52	87.38591	19569.2	87.38387	19567.99	87.38547
87.6414	19580.39	87.63303	19579.31	87.63011	19579.92	87.62974	19582.07	87.63466	19570.52	87.6411	19570.48	87.63617
87.89085	19577.67	87.88716	19580.25	87.88965	19580.29	87.88583	19579.45	87.88279	19567.14	87.8822	19572.78	87.88844
88.13331	19577.7	88.13439	19580.13	88.13592	19579.55	88.12992	19580.53	88.13239	19569.52	88.13647	19570.09	88.13372
88.37995	19581.15	88.39369	19582.26	88.38071	19581.58	88.38101	19582.31	88.38424	19570.89	88.38472	19571.54	88.38463
88.63169	19578.35	88.63411	19579.2	88.62966	19580.51	88.62826	19580.51	88.63633	19569.31	88.642	19570.53	88.63615
88.88196	19580.01	88.88609	19579.52	88.88412	19579.09	88.88535	19580.77	88.88531	19566.89	88.88304	19571.23	88.88572
89.13156	19578.11	89.13475	19579.94	89.13124	19578.88	89.13027	19580.04	89.13369	19568.35	89.13475	19569.66	89.13502
89.38045	19578.94	89.39405	19578.29	89.38027	19578.16	89.37957	19580.22	89.38831	19567.23	89.38376	19565.93	89.39671
89.63281	19578.88	89.63464	19580.29	89.63079	19581.5	89.62903	19582.34	89.63568	19568.59	89.64143	19570.98	89.6357
89.8825	19577.54	89.88847	19578.24	89.8838	19581.99	89.88687	19577.91	89.88521	19566.7	89.88498	19568.2	89.8863
90.13325	19577.51	90.13278	19579.42	90.13227	19581.27	90.12985	19580.17	90.1342	19570.75	90.13509	19568.61	90.13547
90.38037	19580.13	90.39311	19579.23	90.3821	19581.13	90.37959	19578.7	90.38557	19568.31	90.38348	19569.87	90.38594
90.63196	19577.28	90.63507	19579.25	90.6308	19579.34	90.63252	19581.61	90.63594	19567.81	90.64218	19568.24	90.63469
90.88241	19577.1	90.88888	19578.76	90.88419	19580.63	90.88539	19580.42	90.88765	19566.93	90.89039	19569.58	90.88617
91.13345	19577.64	91.1352	19579.81	91.13262	19579.77	91.12935	19579.89	91.13496	19567.94	91.13574	19570.69	91.13565
91.38067	19577.01	91.39064	19578.23	91.38222	19577.76	91.38071	19579.1	91.38469	19566.71	91.38378	19569.32	91.38766
91.63249	19579.24	91.63541	19579.85	91.63218	19579.65	91.63129	19581.17	91.63556	19567.05	91.64242	19567.99	91.63573
91.88243	19578.87	91.88808	19577.53	91.88645	19579.11	91.8888	19581.34	91.88521	19569.42	91.88569	19569.1	91.88657
92.13363	19577.4	92.13426	19578.89	92.13258	19581.34	92.13155	19578.04	92.13556	19570.28	92.14032	19569.28	92.13595
92.38056	19577.32	92.38827	19579	92.38227	19578.97	92.37993	19581.15	92.3878	19567	92.38426	19570.15	92.38784
92.63241	19575.65	92.63568	19578.42	92.63287	19578.95	92.63193	19580.17	92.63593	19569.06	92.64243	19568.23	92.63754
92.8869	19580.3	92.88773	19578.85	92.88611	19578.92	92.88909	19579.48	92.88734	19566.52	92.88578	19567.09	92.88607
93.13353	19579.27	93.13527	19577.94	93.13407	19578.56	93.13166	19578.76	93.1374	19568.34	93.13845	19569.95	93.13571
93.38312	19577	93.3922	19578.15	93.3802	19578.19	93.37948	19579.64	93.38839	19567.65	93.38566	19569.03	93.38752
93.637	19577.83	93.6349	19576.56	93.63223	19576.87	93.63248	19578.31	93.63756	19568.94	93.64344	19568.22	93.63723
93.88324	19578.87	93.88814	19580.58	93.89281	19577.86	93.88763	19579.29	93.88694	19567.4	93.88592	19569.8	93.88647
94.13375	19578.38	94.13607	19578.08	94.13802	19579.23	94.13361	19581	94.13709	19565.92	94.13896	19569	94.13853
94.3834	19577.66	94.39071	19579.65	94.38193	19576.47	94.38027	19579.76	94.38948	19567.72	94.38595	19569.65	94.3882
94.63497	19578.83	94.63601	19578.47	94.6332	19578.59	94.63098	19580.29	94.63837	19568.85	94.6426	19567.67	94.63805
94.88298	19575.27	94.88787	19581.13	94.89256	19578.58	94.88929	19578.76	94.88796	19569.3	94.88763	19567.87	94.88822
95.13377	19576.19	95.13586	19577.47	95.13328	19577.39	95.13484	19579.52	95.14015	19569.37	95.13839	19566.88	95.13757
95.38381	19578.4	95.38988	19578.71	95.38381	19578.1	95.3818	19577.75	95.38584	19566.88	95.38678	19567.98	95.38909
95.63529	19579.46	95.63603	19578.65	95.63579	19580.18	95.63448	19579.91	95.63857	19566.12	95.64497	19567.48	95.63717
95.88478	19578.49	95.89159	19579.4	95.88698	19576.22	95.88918	19580.66	95.88843	19565.91	95.88879	19568.03	95.8873
96.13372	19577.99	96.13363	19576.87	96.13363	19577.56	96.13424	19581.58	96.13732	19565.93	96.14091	19565.45	96.13958
96.38233	19577.35	96.39067	19578.22	96.38545	19578.17	96.38272	19580.43	96.38536	19565.41	96.38615	19567.54	96.39083
96.63679	19576.02	96.63541	19578.9	96.63505	19578.05	96.63165	19580.52	96.63725	19565.42	96.64631	19565.81	96.63721
96.88582	19578.13	96.88963	19575.42	96.88951	19577.58	96.88799	19580.39	96.88829	19566.86	96.88816	19566.78	96.88662
97.14662	19578.8	97.13836	19576.23	97.13537	19578.68	97.13399	19578.36	97.13769	19566.38	97.14405	19566.2	97.13866
97.3823	19577.25	97.38951	19577.95	97.38302	19576.74	97.38204	19579.31	97.38774	19565.89	97.38903	19567.7	97.39098
97.64753	19576.2	97.63936	19577.48	97.63385	19578.25	97.63284	19578.44	97.63724	19566.55	97.6457	19567.58	97.64046
97.8858	19576.5	97.89072	19576.49	97.89425	19577.94	97.89162	19577.72	97.88604	19567.47	97.88879	19565.64	97.88701
98.13762	19576.62	98.13909	19576.22	98.13935	19577.66	98.13467	19578.94	98.13859	19565.96	98.14058	19567.17	98.13767
98.38398	19576.39	98.39288	19578.1	98.38444	19578.8	98.38349	19579.45	98.38977	19567.93	98.38878	19566.22	98.38906
98.63507	19577.97	98.63611	19578.66	98.6356	19576.67	98.63491	19578.83	98.63988	19567.08	98.64618	19567.86	98.63919
98.88426	19575.88	98.89288	19576.7	98.88918	19577.3	98.89565	19578.62	98.88774	19564.03	98.88768	19569.09	98.8889
99.14122	19576.49	99.14159	19577.69	99.13858	19575.88	99.13867	19577.16	99.1422	19567.28	99.14355	19566.15	99.14298
99.38517	19576.46	99.39264	19576.94	99.38784	19577.2	99.38121	19579.82	99.39205	19567.89	99.38803	19567.67	99.3903
99.6361	19577.13	99.63915	19577.95	99.63667	19576.31	99.6336	19576.94	99.63825	19564.15	99.6476	19567.35	99.63866
99.88477	19574.44	99.89157	19575.95	99.88949	19579.46	99.89215	19578.53	99.88759	19566.55	99.88996	19567.02	99.88954
100.1346	19573.34	100.1373	19578.04	100.1345	19577.27	100.1355	19579.41	100.1369	19567.43	100.1393	19564.91	100.1405
100.3874	19575.21	100.3977	19578.87	100.3884	19575.77	100.3835	19578.35	100.391	19564.61	100.3902	19567.04	100.3941
100.6394	19574.4	100.6402	19576.97	100.6387	19577.53	100.6342	19578.7	100.6412	19566.02	100.6495	19567.84	100.6404
100.8889	19576.78	100.8973	19576.79	100.8924	19577.4	100.8918	19576.54	100.8962	19566.36	100.8924	19566.31	100.8903
101.1368	19574.85	101.1414	19576.66	101.138	19578.36	101.1397	19579.71	101.1411	19566.11	101.1423	19568.1	101.141
101.3867	19575.54	101.3938	19577.75	101.3874	19577.4	101.3858	19577.24	101.3922	19565.18	101.3919	19566.95	101.3951
101.6403	19575.93	101.6414	19576.46	101.6382	19576.22	101.6384	19576.89	101.64	19564.07	101.6497	19567.16	101.6423
101.8913	19576.22	101.8973	19577.3	101.895	19577.22	101.8925	19575.48	101.8935	19565.76	101.8916	19565.94	101.8897
102.1401	19576.75	102.1415	19576.18	102.1384	19576.16	102.138	19577.19	102.1415	19565.86	102.1464	19566.64	102.1409
102.3864	19577.42	102.4003	19576.75	102.388	19576.29	102.3868	19576.64	102.392	19566.86	102.3915	19566.94	102.4055
102.6463	19575	102.6417	19575.43	102.6381	19577.54	102.6378	19576.63	102.641	19564.46	102.6505	19568.12	102.6448
102.89	19576.35	102.8949	19576.63	102.8915	19576.01	102.8966	19577.97	102.8929	19567.32	102.8911	19565.8	102.8929
103.1399	19574.9	103.1424	19576.33	103.138	19575.85	103.137	19576.35	103.1443	19565.4	103.1434	19564.99	103.1442
103.3891	19575.67	103.3962	19577.14	103.3892	19575.82	103.3864	19577.18	103.3951	19565.72	103.3921	19566.43	103.404
103.6376	19575.38	103.6422	19576.06	103.6398	19575.96	103.6376	19577.35	103.644	19565.26	103.6487	19568.69	103.6416

103.889	19575.76	103.8951	19576.64	103.8914	19577.44	103.8949	19576.88	103.8921	19563.73	103.8913	19567.04	103.892
104.1401	19576.63	104.1437	19576.65	104.1405	19577.51	104.1374	19576.47	104.1414	19565.16	104.144	19568.59	104.1421
104.3891	19574.08	104.3987	19574.33	104.3894	19576.06	104.3869	19577.43	104.3963	19564.43	104.393	19563.95	104.3953
104.641	19574.73	104.6422	19576.36	104.6407	19576.65	104.6381	19576.03	104.6435	19565.21	104.648	19566.43	104.6421
104.8897	19576.52	104.8981	19576.46	104.8921	19575.65	104.8949	19576.64	104.8924	19565.71	104.8936	19565.04	104.8918
105.1407	19576.78	105.1417	19576.21	105.1407	19576.97	105.137	19576.64	105.1448	19565.46	105.1452	19568.14	105.144
105.3897	19575.18	105.3952	19576.19	105.3899	19575.37	105.3862	19576.12	105.3937	19564.2	105.3914	19566.87	105.3982
105.6396	19575.78	105.643	19575.4	105.6416	19578.82	105.6381	19577.03	105.6451	19566.61	105.6529	19565.44	105.6436
105.8913	19574.8	105.898	19574.04	105.8946	19575.67	105.8962	19577.1	105.8941	19565.31	105.8947	19564.64	105.8923
106.1399	19574	106.1433	19576.18	106.1406	19576.1	106.1396	19576.42	106.1426	19564.11	106.1459	19563.59	106.1431
106.3889	19574.49	106.4003	19574.78	106.3911	19576.71	106.3875	19575.6	106.3941	19565.73	106.3918	19566.65	106.403
106.6409	19575.58	106.6435	19575.91	106.6408	19575.28	106.6382	19576.95	106.644	19563.49	106.6657	19568.46	106.6452
106.8912	19573.5	106.896	19575.64	106.8968	19575.39	106.8954	19574.02	106.8972	19564.72	106.9037	19563.87	106.8942
107.1423	19576.17	107.1426	19574.41	107.1423	19575.01	107.1398	19575.17	107.1427	19565.59	107.149	19566.24	107.1451
107.389	19576.03	107.3958	19575.55	107.3892	19575.7	107.3868	19578.14	107.3956	19566.74	107.3946	19565.32	107.3964
107.6426	19575.23	107.6434	19577.87	107.6405	19575.51	107.6373	19576.55	107.6437	19564.73	107.6538	19564.53	107.6459
107.8917	19575.21	107.8999	19576.19	107.8962	19574.14	107.8953	19575.71	107.8974	19564.16	107.893	19565.43	107.8956
108.1427	19575.3	108.1422	19575.38	108.1429	19575.56	108.1406	19576.97	108.145	19565.23	108.1468	19567.24	108.1463
108.391	19573.68	108.3965	19575.84	108.3906	19575.55	108.3901	19574.13	108.3927	19564.69	108.3937	19564.33	108.3986
108.6401	19574.4	108.6453	19574.29	108.6399	19576.11	108.6391	19576.59	108.6438	19564.96	108.6547	19564.74	108.6443
108.8909	19575.3	108.9015	19575.63	108.8957	19574.71	108.8977	19574.91	108.8959	19564.17	108.8948	19565.35	108.8955
109.1424	19576.15	109.1445	19576.17	109.1414	19575.46	109.1408	19575.09	109.1445	19563.27	109.1467	19563.92	109.1474
109.3914	19575.7	109.3976	19576.75	109.3911	19575.43	109.3895	19577.68	109.3978	19563.16	109.3936	19565.81	109.4009
109.642	19573.96	109.6461	19574.76	109.6426	19574.26	109.6399	19576.62	109.6427	19565	109.6511	19562.92	109.645
109.8903	19571.78	109.8977	19575.45	109.8971	19575.79	109.8959	19576.88	109.8962	19563.12	109.8965	19563.06	109.8944
110.1414	19574.68	110.1499	19576.17	110.1543	19576.75	110.1397	19575.47	110.1438	19563.52	110.1481	19565.06	110.1465
110.4021	19573.52	110.4002	19574.91	110.3922	19575.27	110.3902	19576.1	110.3974	19563.01	110.3943	19565.7	110.4008
110.6429	19574.54	110.6449	19574.75	110.6432	19574.56	110.6414	19575.86	110.6455	19565.41	110.6528	19562.91	110.647
110.894	19572.71	110.9001	19575.24	110.8956	19575.43	110.8967	19575.72	110.8967	19563.35	110.8948	19563.62	110.8952
111.1451	19573.6	111.1453	19575.33	111.1471	19576.48	111.1402	19573.63	111.1445	19563.37	111.1495	19565.6	111.1466
111.3912	19574.21	111.3984	19576.62	111.3906	19575.43	111.3892	19576.1	111.3961	19564.28	111.3957	19561.79	111.3989
111.6433	19573.95	111.6608	19575.44	111.6424	19575.7	111.6416	19576.9	111.6464	19560.96	111.6521	19564.48	111.6477
111.8954	19574.13	111.9018	19574.46	111.896	19575.49	111.8986	19574.39	111.8998	19566.58	111.8953	19562.61	111.8988
112.1452	19576.94	112.146	19575.21	112.1439	19574.59	112.1424	19576.63	112.1451	19561.69	112.1552	19565.59	112.1468
112.3909	19575.64	112.3978	19574.07	112.3919	19576.24	112.3906	19577.29	112.3942	19565.06	112.3959	19564.98	112.4001
112.6427	19574.35	112.6456	19575.79	112.6428	19572.76	112.6408	19574.97	112.6476	19561.74	112.6519	19564.5	112.6473
112.8941	19573.83	112.9021	19575.33	112.8974	19573.91	112.8974	19574.86	112.8985	19563.23	112.8961	19565.67	112.8968
113.1455	19573.93	113.1473	19575.55	113.1444	19576.09	113.1427	19575.59	113.1462	19566.23	113.1497	19564.47	113.1498
113.3929	19572.46	113.3999	19575.34	113.3935	19572.1	113.391	19575.86	113.3949	19563.78	113.3983	19566.76	113.3994
113.6428	19573.94	113.647	19573.14	113.644	19574.26	113.6404	19576.36	113.6455	19564.62	113.6526	19563.41	113.6459
113.8941	19573.24	113.9012	19572.28	113.8968	19573.7	113.8993	19577.15	113.8996	19563.85	113.8966	19561.67	113.8974
114.1454	19574.7	114.1468	19573.08	114.144	19575.4	114.1426	19575.4	114.1478	19564.09	114.1504	19563.5	114.1495
114.3937	19574.77	114.3999	19576.09	114.3935	19573.73	114.3923	19574.9	114.3995	19560.56	114.3961	19564.34	114.4017
114.6445	19576.31	114.6455	19575.25	114.6443	19575.43	114.6436	19574.17	114.647	19563.47	114.6544	19563.13	114.6483
114.8945	19573.71	114.9019	19573.72	114.8966	19574.64	114.9016	19574.64	114.8998	19563.39	114.8972	19562.07	114.8985
115.1445	19572.85	115.1466	19575.26	115.1418	19573.95	115.1428	19574.65	115.147	19562.82	115.15	19563.76	115.148
115.3932	19573.24	115.4024	19574.33	115.3932	19576.09	115.408	19575.02	115.3985	19564.88	115.3963	19563.97	115.4005
115.647	19571.92	115.6453	19572.95	115.6497	19573.62	115.6447	19573.31	115.648	19563.7	115.6567	19563.04	115.6486
115.8967	19573.7	115.9023	19575.89	115.8988	19573.08	115.9008	19576.49	115.8997	19562.15	115.8981	19563.34	115.8985
116.1458	19572.3	116.1465	19572.16	116.142	19573.13	116.1435	19574.38	116.1459	19563.44	116.152	19563.16	116.148
116.3925	19574.08	116.4045	19574.8	116.3934	19574.21	116.392	19575.06	116.3999	19562.79	116.3967	19563.94	116.4018
116.6444	19575.01	116.6471	19572.72	116.6453	19573.24	116.643	19575.9	116.6483	19564.26	116.6534	19564.38	116.6487
116.8971	19573.69	116.8996	19576.7	116.9013	19574.6	116.9048	19575.53	116.9002	19563.83	116.8983	19563.04	116.8987
117.1479	19572.86	117.1449	19573.76	117.1452	19574.18	117.154	19575.82	117.1485	19561.64	117.1547	19563.84	117.1504
117.3942	19573.38	117.4024	19574.03	117.3936	19575.85	117.3932	19577.07	117.3992	19563.86	117.3987	19564.48	117.4008
117.6464	19572.97	117.6474	19575.45	117.6446	19574.12	117.6427	19575.46	117.6487	19565	117.6543	19562.29	117.6481
117.899	19573.4	117.9013	19574.34	117.8987	19574.31	117.9009	19576.16	117.9003	19561.08	117.8972	19563.42	117.8988
118.1486	19572.86	118.147	19574.5	118.1471	19574.99	118.1443	19573.99	118.1496	19562.16	118.1523	19563.1	118.1493
118.3949	19572.44	118.4002	19573.05	118.3956	19575.75	118.3969	19573.23	118.3986	19562.14	118.3993	19562.82	118.4079
118.6445	19573.36	118.647	19574.24	118.6441	19575.76	118.6446	19574.26	118.6482	19564.09	118.6534	19564.07	118.6499
118.8967	19574.41	118.9019	19575.02	118.9001	19573.25	118.9018	19577.96	118.9017	19563.75	118.8978	19565.28	118.8999
119.1481	19573.27	119.1483	19572.55	119.145	19573.81	119.1445	19575.18	119.1494	19564.78	119.1526	19562.42	119.149
119.3968	19571.97	119.4059	19572.95	119.3948	19575.88	119.3959	19575.36	119.4018	19562.71	119.3981	19563.82	119.4059
119.6453	19574.03	119.6471	19573.2	119.6488	19575.6	119.6428	19574.39	119.6482	19563.97	119.6563	19563.38	119.6503
119.8975	19574.26	119.9017	19572.22	119.9002	19571.77	119.9015	19575.45	119.8992	19564.06	119.8999	19562.7	119.9019
120.1561	19572.8	120.1512	19573.1	120.1464	19574.04	120.1449	19572.81	120.1506	19560.81	120.1543	19561.27	120.1503
120.3961	19574.08	120.4045	19575.19	120.3955	19573.82	120.3963	19576.04	120.406	19563.05	120.3991	19563.25	120.4047
120.6449	19571	120.6469	19572.9	120.6455	19573.89	120.6438	19574.55	120.6475	19561.73	120.655	19565.07	120.6483
120.8976	19572.07	120.901	19573.91	120.8967	19576.31	120.8996	19573.61	120.8978	19562.74	120.8987	19561.73	120.8985
121.1454	19572.78	121.145	19571.8	121.1433	19574.34	121.1457	19572.76	121.1457	19563.71	121.1515	19562.03	121.1574

121.3928	19572.37	121.4008	19574.52	121.3962	19574.42	121.3988	19573.99	121.397	19562.22	121.3974	19562.65	121.4029
121.6438	19571.35	121.6471	19573.05	121.6454	19576.51	121.6484	19575.81	121.647	19562.88	121.6532	19561.92	121.6473
121.8977	19573.17	121.9019	19573.33	121.8984	19572.63	121.9007	19573.79	121.9003	19561.62	121.8976	19561.24	121.8981
122.1479	19571.98	122.146	19571.06	122.1451	19575.58	122.1473	19575.84	122.1477	19563.88	122.1539	19564.51	122.1492
122.3928	19571.98	122.4125	19573.09	122.3932	19573	122.3937	19572.72	122.3968	19563.09	122.3976	19563.65	122.4019
122.6442	19571.57	122.6458	19574.61	122.6463	19573.15	122.6431	19575.43	122.6463	19562.06	122.6552	19561.98	122.6483
122.8956	19573.42	122.9017	19574.88	122.8986	19573.51	122.9017	19572.25	122.8985	19563.19	122.8971	19561.46	122.8979
123.1478	19575.16	123.1473	19573.23	123.1458	19571.8	123.1448	19574.32	123.1503	19561.42	123.1523	19563.6	123.1485
123.3952	19572.15	123.4002	19574.05	123.3935	19574.06	123.3927	19572.66	123.3994	19562.33	123.3987	19562.55	123.405
123.6445	19570.29	123.6465	19573.44	123.6445	19574.26	123.642	19574.7	123.6461	19561.27	123.6519	19562.35	123.6481
123.8967	19571.52	123.9023	19572.55	123.9002	19571.9	123.9011	19573.98	123.8992	19562.81	123.8982	19561.7	123.8987
124.148	19571.54	124.1481	19574.51	124.1458	19573.05	124.1454	19574.49	124.1505	19561.44	124.1509	19562.66	124.149
124.3954	19571.24	124.402	19572.45	124.3952	19573.79	124.3935	19574.19	124.4032	19560.56	124.3978	19562.02	124.4047
124.6472	19573.15	124.6462	19571.29	124.6467	19574.75	124.6434	19573.78	124.6477	19562.61	124.6544	19561.18	124.6497
124.8948	19573.2	124.9039	19573.28	124.899	19572.26	124.8993	19572.39	124.9022	19562.37	124.8991	19562.58	124.9002
125.1485	19570.87	125.1482	19574.47	125.1494	19573.48	125.1446	19575.32	125.1523	19561.46	125.1522	19562.93	125.1477
125.4083	19572.06	125.4034	19573.06	125.3994	19573.47	125.3943	19571.13	125.4006	19563.01	125.3991	19561.45	125.4158
125.6579	19572.3	125.6484	19573.25	125.6598	19574.48	125.6444	19574.05	125.649	19560.73	125.6521	19561.83	125.6529
125.9081	19570.46	125.9054	19574.35	125.9019	19573.47	125.9024	19574.52	125.8997	19563.25	125.9002	19562.74	125.8995
126.1602	19572.53	126.1476	19569.3	126.1525	19572.65	126.1456	19574.57	126.1503	19562.34	126.1528	19562.8	126.15
126.3947	19571.02	126.4024	19572.78	126.4033	19574.02	126.3975	19574.44	126.4019	19560.54	126.3993	19561.34	126.4089
126.6472	19573.56	126.6546	19572.28	126.6524	19573.26	126.6459	19573.06	126.6509	19562.77	126.6552	19562.52	126.6497
126.8972	19572.75	126.9078	19572.69	126.9019	19574.82	126.9031	19574.01	126.8997	19563.75	126.8995	19561.24	126.8988
127.1476	19572.95	127.1486	19571.82	127.1456	19573.79	127.1447	19575.41	127.1509	19560.89	127.1549	19561.84	127.1498
127.3948	19573.1	127.4013	19572.62	127.3972	19574.49	127.3948	19573.6	127.4015	19562.03	127.3998	19561.74	127.4078
127.6457	19570.38	127.6487	19572.55	127.6489	19570.42	127.6465	19573.87	127.6494	19562.3	127.6601	19561.2	127.6515
127.8968	19572.92	127.9065	19573.14	127.9014	19573.95	127.902	19574.33	127.8985	19561.18	127.8989	19562.2	127.8992
128.1497	19571.52	128.1501	19574.2	128.1495	19573.33	128.1446	19573.5	128.1488	19563.78	128.1526	19561.32	128.1498
128.3993	19572.88	128.4043	19573.07	128.3959	19571.78	128.3952	19574.84	128.4011	19560.53	128.3994	19562.38	128.4055
128.6461	19571.7	128.6475	19571.45	128.6494	19572.58	128.6456	19574.39	128.6511	19559.87	128.655	19561.86	128.6509
128.9013	19571.2	128.9043	19572.8	128.9	19574	128.9041	19573.26	128.9037	19559.56	128.9016	19562.64	128.9024
129.1512	19571.68	129.1498	19572.86	129.1475	19573.06	129.1479	19574.56	129.1493	19560.84	129.1519	19562.98	129.1506
129.4011	19573.5	129.4027	19572.55	129.3984	19571.8	129.395	19572.73	129.4042	19563.81	129.4004	19561.32	129.4074
129.6513	19572.27	129.6505	19572.58	129.649	19573.39	129.647	19572.84	129.65	19561.17	129.6561	19560.73	129.6518
129.901	19572.35	129.9045	19570.38	129.902	19573.44	129.9042	19572.83	129.9044	19561.25	129.9018	19563.1	129.9024
130.1615	19572.59	130.148	19572.19	130.147	19574.43	130.1472	19572.95	130.1511	19561.6	130.1524	19562.02	130.1514
130.3973	19574.53	130.4056	19573.44	130.3968	19572.32	130.396	19574.28	130.4006	19562.29	130.401	19561.28	130.4087
130.6495	19571.67	130.6497	19571.19	130.6494	19572.06	130.6454	19572.99	130.6496	19563.59	130.6563	19561.81	130.6513
130.9005	19570.78	130.9091	19572.24	130.905	19572.44	130.9029	19575.46	130.9041	19560.92	130.9014	19561.6	130.902
131.1503	19569.18	131.1492	19574.08	131.1505	19572.97	131.1468	19571.23	131.1639	19562.74	131.155	19561.95	131.1523
131.3959	19570.98	131.4036	19571.67	131.3988	19572.03	131.396	19574.01	131.4038	19562.78	131.402	19563.24	131.4086
131.6503	19572.92	131.6517	19572.32	131.6497	19573.26	131.6472	19574.09	131.6523	19562.74	131.6548	19561.88	131.6525
131.8988	19571.52	131.9094	19572.49	131.9011	19571.79	131.9041	19572.69	131.9024	19560.04	131.9134	19560.05	131.9025
132.1533	19569.89	132.1512	19572.32	132.1478	19573.95	132.1477	19573.27	132.1565	19563.26	132.1573	19559	132.1529
132.3983	19571.71	132.4026	19573.49	132.3974	19571.52	132.3973	19571.69	132.404	19561.05	132.401	19561.33	132.4096
132.6471	19573.53	132.6506	19571.43	132.6494	19571.6	132.6506	19573.33	132.6521	19560.65	132.6564	19562.39	132.6526
132.8986	19571.84	132.905	19573.37	132.9043	19573.41	132.9069	19572.42	132.9027	19561.6	132.9033	19560.94	132.903
133.1512	19571.24	133.1567	19572.1	133.1508	19570.57	133.1467	19572.86	133.1666	19560.77	133.1532	19561.47	133.1528
133.399	19570.93	133.4048	19572.43	133.3974	19570.85	133.3961	19572.32	133.4124	19560.59	133.4018	19562.89	133.41
133.6521	19570.72	133.6524	19572.61	133.6488	19571.83	133.6481	19572.51	133.6522	19558.99	133.657	19559.57	133.6539
133.8995	19569.11	133.9069	19571.63	133.9036	19573.08	133.9067	19572.02	133.9041	19562.5	133.9023	19561.04	133.9023
134.1534	19573.04	134.152	19571.28	134.1511	19570.68	134.1491	19571.74	134.1544	19561.16	134.1566	19563.59	134.1625
134.3987	19569.36	134.4064	19571.84	134.3995	19572.32	134.3965	19573.65	134.4061	19560.2	134.4035	19559.75	134.4092
134.6496	19570.4	134.6528	19571.64	134.6501	19570.86	134.6485	19573.24	134.6516	19560.63	134.6577	19561.09	134.6549
134.9014	19572.03	134.9067	19570.84	134.903	19570.63	134.9049	19573.65	134.9053	19559.49	134.9024	19561.99	134.9041
135.155	19571.68	135.1534	19573.11	135.1509	19571.95	135.1505	19571.45	135.1569	19562.01	135.1557	19562.26	135.1537
135.3991	19571.28	135.405	19571.71	135.398	19572.11	135.3966	19572.57	135.4031	19559.75	135.4051	19563.37	135.4087
135.6505	19569.01	135.6526	19572.47	135.6506	19571.26	135.65	19573.22	135.6529	19558.35	135.6583	19560.3	135.655
135.901	19572.93	135.9113	19572.43	135.9045	19571.48	135.9077	19574.37	135.9061	19561.94	135.9043	19562.34	135.9037
136.1543	19570.71	136.152	19567.59	136.1476	19572.33	136.1503	19569.95	136.155	19560.14	136.1545	19562.67	136.155
136.3987	19570.93	136.4064	19574.34	136.3989	19573.82	136.3985	19571.85	136.4039	19561.56	136.4032	19562.44	136.4099
136.6501	19570.65	136.6556	19571.87	136.6507	19572.67	136.6492	19572.42	136.6511	19561.17	136.6593	19559.67	136.6544
136.9025	19570.54	136.9102	19573.74	136.9049	19571.95	136.9054	19571.67	136.9076	19559.75	136.9057	19561.17	136.9049
137.1557	19572.9	137.1542	19570.13	137.1506	19571.8	137.1494	19573.01	137.1557	19559.78	137.1603	19560.94	137.155
137.399	19571.24	137.4069	19571.01	137.3982	19571.76	137.3986	19571.04	137.4055	19560.61	137.4039	19559.76	137.4093
137.6494	19571.3	137.6542	19569.82	137.6519	19571.14	137.6515	19572.19	137.6539	19560.11	137.6596	19561.6	137.6551
137.9018	19571.63	137.9101	19571.58	137.9042	19570.94	137.9056	19572.03	137.9046	19561.66	137.9045	19561.55	137.9059
138.1587	19571.88	138.1562	19573.13	138.1502	19571.32	138.1501	19569.96	138.1552	19561.25	138.1562	19561.85	138.1554
138.4017	19571.73	138.4092	19572.68	138.4005	19572.69	138.3986	19571.34	138.4082	19563.53	138.405	19560.85	138.41
138.6492	19569.99	138.6534	19570.93	138.6503	19569.61	138.6505	19572.14	138.6548	19560.08	138.6605	19558.85	138.655

138.9009	19572.49	138.9075	19571.59	138.9073	19572.71	138.9081	19573.82	138.9062	19560.42	138.9044	19562.66	138.9045
139.1593	19570.67	139.1557	19570.47	139.1509	19571.93	139.1512	19573.05	139.1541	19561.05	139.1565	19561.37	139.1566
139.4021	19571.11	139.4089	19572.13	139.4008	19572.17	139.3996	19570.96	139.4098	19561.64	139.4064	19560.57	139.4117
139.6519	19571.46	139.6548	19568.95	139.6543	19572.21	139.6616	19572.36	139.6551	19560.32	139.6583	19562.4	139.6552
139.9019	19572.41	139.9088	19570.75	139.9074	19570.94	139.9078	19574.05	139.9082	19559.89	139.9052	19562.52	139.9062
140.1587	19569.83	140.1536	19572.58	140.1516	19573.12	140.1519	19573.4	140.1557	19559.86	140.1581	19560.57	140.1566
140.4009	19569.42	140.4066	19571.67	140.4015	19572.18	140.4002	19572.76	140.4051	19558.36	140.4058	19559.7	140.4114
140.6533	19571.01	140.6549	19572.11	140.6536	19572.3	140.6511	19572.42	140.6534	19560.18	140.6624	19560.43	140.6547
140.9026	19570.73	140.9136	19571.88	140.9071	19566.89	140.9059	19570.69	140.9078	19561.15	140.9034	19563.44	140.9068
141.1587	19572.55	141.1568	19573	141.1527	19571.74	141.1521	19572.62	141.1573	19561.02	141.1581	19561.63	141.1582
141.401	19571.44	141.4087	19571.11	141.4021	19570.44	141.4003	19572.39	141.4089	19560.24	141.4079	19562.7	141.4109
141.6612	19573.2	141.6541	19570.17	141.6516	19571.31	141.6549	19574.54	141.6578	19561.81	141.6601	19560.98	141.658
141.9064	19569.87	141.9106	19571.58	141.9074	19571.56	141.9068	19570.89	141.9079	19557.63	141.9054	19560.34	141.9048
142.1653	19572.01	142.1569	19569.82	142.1523	19572.34	142.1513	19572.34	142.1564	19559.07	142.1614	19561.98	142.1569
142.4055	19568.87	142.4108	19572.02	142.4008	19569.87	142.4013	19574.37	142.4096	19560.22	142.4054	19560.32	142.4127
142.6567	19571.73	142.6542	19571.57	142.6534	19571.02	142.6545	19572.95	142.6577	19560	142.663	19560.26	142.6576
142.9106	19570.7	142.9077	19571.06	142.9097	19571.58	142.9074	19571.33	142.9081	19561.26	142.9054	19559.92	142.9081
143.1775	19570.25	143.1577	19571.68	143.1524	19569.08	143.1527	19574.38	143.1566	19560.27	143.1574	19558.52	143.1571
143.4015	19572.1	143.4092	19568.42	143.4026	19572.9	143.4005	19570.36	143.4105	19560.6	143.4063	19560.15	143.4113
143.6536	19569.43	143.6566	19570.26	143.6555	19570.36	143.6552	19572.8	143.6575	19560.26	143.6613	19560.13	143.6575
143.9046	19569.19	143.9104	19570.37	143.9082	19571.06	143.9106	19573.1	143.9067	19558.2	143.9059	19561.12	143.9073
144.1619	19572.07	144.1562	19573.14	144.1545	19571.79	144.1666	19572.54	144.1563	19560.32	144.1645	19559.97	144.1573
144.4028	19571.27	144.407	19570.06	144.4022	19572.9	144.4004	19572.53	144.4156	19560.66	144.4068	19559.79	144.4128
144.6552	19570.94	144.6565	19571.2	144.6542	19569.96	144.6531	19572.22	144.6571	19561.72	144.6626	19559.94	144.6585
144.9039	19570.14	144.9117	19571.45	144.9064	19572.36	144.91	19572.53	144.9103	19560.61	144.9068	19561.03	144.908
145.1609	19569.6	145.1583	19569.26	145.1531	19571.71	145.1536	19572.43	145.1576	19559.92	145.1605	19558.61	145.1576
145.4035	19572.39	145.4311	19569.73	145.4012	19571.2	145.4013	19571.6	145.4099	19560.16	145.4088	19561.41	145.4115
145.6547	19570.9	145.6557	19570.09	145.6559	19571.88	145.6528	19573.97	145.6589	19558.87	145.662	19561.59	145.6589
145.9043	19569.28	145.9114	19571.25	145.9089	19570.92	145.9112	19573.1	145.9093	19557.88	145.9062	19559.94	145.9095
146.1608	19568.34	146.1668	19569.74	146.153	19570.99	146.1541	19571.26	146.1583	19560.13	146.159	19560.72	146.1596
146.404	19571.9	146.4142	19571.53	146.4033	19569.44	146.4022	19572.32	146.4095	19559.64	146.4072	19560.34	146.4159
146.6562	19569.46	146.6615	19571.74	146.6556	19572.34	146.6535	19573.11	146.6583	19560.2	146.6636	19561.82	146.659
146.9043	19569.63	146.9151	19571.59	146.9129	19570.11	146.9105	19572.4	146.9118	19558.32	146.9085	19561.19	146.9081
147.1579	19570.57	147.1627	19571.13	147.1567	19569.04	147.1545	19572.7	147.159	19558.87	147.1635	19558.88	147.1583
147.4045	19568.36	147.4185	19570.54	147.4023	19567.55	147.4015	19570.16	147.4099	19559.15	147.4058	19561.18	147.4148
147.6556	19569.19	147.6572	19569.65	147.6537	19571.24	147.6537	19571.14	147.6589	19559.43	147.6647	19560.03	147.6614
147.907	19569.57	147.9142	19570.43	147.9125	19570.82	147.9128	19571.75	147.9106	19558.94	147.9087	19560.65	147.9092
148.1596	19569.06	148.1599	19570.25	148.1541	19572.1	148.1542	19570.64	148.1584	19560.62	148.1596	19560.74	148.158
148.4032	19570.09	148.4128	19572.06	148.4043	19571.69	148.4063	19572.75	148.4154	19559.97	148.4054	19559.02	148.4128
148.6565	19570.72	148.6578	19570.61	148.656	19568.97	148.6541	19569.61	148.6599	19559.8	148.6649	19558.67	148.6617
148.9071	19570.23	148.9112	19570.89	148.9123	19571.26	148.9088	19570.69	148.9101	19560.5	148.9082	19562.53	148.9104
149.1623	19569.73	149.1587	19569.24	149.1541	19571.34	149.1548	19572.55	149.1585	19557.98	149.16	19559.59	149.1596
149.4046	19569.58	149.4115	19571.51	149.4053	19570.22	149.4036	19571.47	149.413	19560.99	149.4079	19560.26	149.4136
149.6566	19570.51	149.6584	19570.36	149.6594	19570.16	149.6584	19571.25	149.6588	19560.21	149.6656	19559.36	149.6646
149.9056	19570.88	149.9157	19571.85	149.9094	19569.55	149.9126	19573.58	149.912	19559.95	149.9084	19558.52	149.9104
150.1613	19571.38	150.1596	19571.01	150.1557	19572.17	150.1542	19570.42	150.1588	19559.97	150.1619	19560.2	150.1602
150.4089	19567.18	150.4146	19570.57	150.4072	19571.18	150.4071	19572.85	150.4158	19560.38	150.4125	19559.28	150.4169
150.6599	19569.74	150.6614	19571.23	150.6598	19570.71	150.6586	19570.5	150.6631	19559.5	150.6686	19559.58	150.6644
150.9088	19570.24	150.9184	19568.57	150.9147	19568.75	150.9146	19571.92	150.9156	19557.47	150.9128	19560.55	150.9167
151.1642	19569.32	151.1651	19571.28	151.1709	19571.53	151.1573	19571.72	151.1647	19559.93	151.1674	19562.01	151.1639
151.4095	19568.06	151.4175	19570.84	151.4088	19571.33	151.4116	19569.44	151.4148	19560.53	151.4118	19560.13	151.4188
151.6609	19571.86	151.664	19570.79	151.6587	19570.82	151.6595	19573.99	151.6613	19560.03	151.6685	19561.58	151.664
151.9106	19569.25	151.9172	19571.69	151.9147	19570.35	151.9166	19570.25	151.9151	19559.62	151.9132	19560.2	151.9133
152.1657	19570.63	152.1663	19569.33	152.159	19569.42	152.1612	19569.01	152.164	19557.94	152.1692	19558.55	152.1636
152.4091	19570.34	152.4169	19572.57	152.41	19570.63	152.4103	19571.68	152.4187	19558.06	152.4125	19558.23	152.4203
152.661	19569.57	152.6628	19568.7	152.6596	19570.65	152.6601	19571.63	152.6616	19559.86	152.6679	19559.63	152.6676
152.911	19567.89	152.9164	19568.27	152.9155	19571.89	152.9198	19572.2	152.9166	19556.9	152.9137	19558.12	152.9133
153.1662	19571.15	153.1646	19570.99	153.16	19569.22	153.164	19572.61	153.1647	19559.31	153.167	19559.48	153.1658
153.409	19572.49	153.4168	19568.6	153.4095	19568.56	153.409	19571.89	153.4191	19559.2	153.4124	19558.44	153.4213
153.6607	19570.13	153.6644	19571.27	153.6608	19570.5	153.658	19571.29	153.6627	19559.65	153.6696	19560.03	153.6676
153.9119	19568.79	153.918	19571.7	153.9163	19570.77	153.9156	19571.24	153.9167	19559.69	153.9139	19558.05	153.9158
154.1692	19569.87	154.1655	19567.62	154.1604	19568.99	154.1626	19571.81	154.164	19561.88	154.1707	19559.62	154.1638
154.4093	19568.29	154.4166	19567.98	154.4096	19569.61	154.4092	19571.51	154.4188	19559.09	154.4127	19558.69	154.4202
154.6608	19570.71	154.6624	19570.49	154.6622	19568.3	154.66	19569.17	154.6647	19558.48	154.6703	19561.17	154.6672
154.9123	19568.46	154.9209	19569.24	154.9142	19571.62	154.9186	19570.54	154.9159	19559.91	154.9137	19560.57	154.9179
155.1686	19570.59	155.1639	19568.53	155.1606	19569.93	155.1591	19571.5	155.1653	19560.96	155.1663	19560.78	155.1662
155.4106	19571.09	155.4171	19569.97	155.4095	19572.46	155.4119	19571.17	155.4184	19560.22	155.4151	19559.65	155.4199
155.6605	19570.77	155.6657	19572.32	155.6616	19571.57	155.6649	19571.2	155.6643	19558.51	155.6715	19559.88	155.6804
155.912	19571.29	155.9189	19569.59	155.9157	19570.66	155.9177	19571.75	155.9166	19560.27	155.914	19559.25	155.9209
156.167	19570.06	156.1655	19569.71	156.1625	19570.17	156.1763	19570.23	156.1658	19558.81	156.1681	19557.99	156.1671

156.4127	19569.7	156.4189	19568.58	156.4097	19570.68	156.4108	19572.68	156.4187	19557.67	156.4145	19560.23	156.4238
156.662	19571.4	156.6616	19569.21	156.6605	19571.19	156.6613	19569.22	156.665	19560.22	156.673	19559.28	156.667
156.9116	19569.34	156.9171	19570.68	156.9168	19569.37	156.9182	19572.26	156.9182	19558.43	156.9152	19560.93	156.9168
157.1677	19569.17	157.1648	19570.31	157.1629	19572.03	157.1607	19570.81	157.1821	19557.92	157.172	19562.72	157.166
157.4143	19569.71	157.4215	19568.06	157.4126	19569.84	157.4088	19570.89	157.4201	19560.13	157.4152	19559.29	157.4218
157.6625	19568.45	157.6644	19569.84	157.6652	19570.87	157.6606	19569.69	157.6653	19558.02	157.6724	19559.42	157.6689
157.9131	19570.4	157.9209	19570.09	157.9164	19568.91	157.9188	19572.29	157.9184	19559.94	157.9163	19560.67	157.9162
158.1704	19569.39	158.1657	19568.49	158.1624	19569.97	158.1627	19571.64	158.1669	19557.13	158.1694	19560.71	158.1659
158.4119	19569.39	158.4195	19571.51	158.4116	19570.94	158.4101	19572.25	158.4191	19558.36	158.4153	19559.09	158.4229
158.6636	19571.41	158.6666	19570.49	158.6632	19570.25	158.6621	19567.92	158.6659	19558.4	158.674	19559.03	158.6689
158.9138	19568.23	158.9212	19566.9	158.9194	19571.26	158.9186	19572.85	158.9187	19557.97	158.9194	19560.11	158.9168
159.1702	19568.07	159.165	19569.31	159.1763	19571.21	159.1634	19568.7	159.1671	19556.66	159.1697	19557.93	159.1661
159.4139	19570.32	159.4203	19570.05	159.4117	19571.68	159.4119	19568.56	159.4223	19557.59	159.4148	19556.97	159.4315
159.6647	19568.85	159.6654	19567.88	159.6618	19572.76	159.661	19570.98	159.6682	19558.72	159.6756	19558.73	159.6735
159.9152	19570.03	159.9228	19569.43	159.9181	19569.51	159.9205	19569.33	159.9195	19559.49	159.9184	19558.61	159.9175
160.1699	19569.82	160.1657	19571.77	160.1628	19569.21	160.1638	19569.4	160.1696	19558.56	160.1698	19559.25	160.1674
160.4142	19569.35	160.4192	19569.31	160.4105	19569.9	160.4131	19571.26	160.4195	19559.45	160.4151	19559.7	160.4226
160.66	19568.36	160.6627	19568.97	160.6592	19571.33	160.6589	19569.51	160.6637	19560.47	160.6728	19560.94	160.6651
160.9105	19569.75	160.9171	19570.64	160.9132	19569.35	160.9188	19570.49	160.9175	19558.09	160.9132	19558.72	160.9163
161.168	19571.29	161.1671	19569.85	161.1604	19570.64	161.1613	19572.56	161.1644	19558.93	161.1665	19559.11	161.1651
161.4117	19568.89	161.4174	19570.16	161.4098	19569.18	161.4086	19570.92	161.4153	19558.21	161.4145	19559.05	161.4188
161.6639	19571.14	161.6649	19568.96	161.6599	19568.97	161.6611	19572	161.6626	19559.32	161.6702	19560.54	161.6657
161.9107	19568.12	161.9178	19568.92	161.914	19570.64	161.919	19569.83	161.9175	19557.9	161.9141	19557.35	161.9158
162.1694	19570.72	162.1648	19570.47	162.1602	19570.13	162.1729	19568.69	162.165	19559.59	162.1694	19556.39	162.1671
162.4128	19570.69	162.4183	19569.43	162.4105	19570.03	162.4117	19571.04	162.4163	19559.81	162.4136	19558.16	162.4227
162.6629	19569.58	162.664	19567.62	162.6624	19570.39	162.6607	19571.34	162.6634	19559.32	162.6757	19558.58	162.6662
162.9122	19568.88	162.9179	19570.52	162.9142	19570.92	162.918	19572.44	162.9177	19558.01	162.915	19559.91	162.9156
163.1757	19567.62	163.1642	19570.43	163.1628	19567.7	163.1618	19571.25	163.1659	19558.27	163.1673	19557.55	163.1645
163.4132	19567.3	163.4198	19571.05	163.4105	19570.33	163.4073	19571.26	163.4162	19556.6	163.414	19557.62	163.4208
163.6621	19569.2	163.665	19569.16	163.6629	19570.61	163.6596	19570.09	163.664	19557.67	163.6712	19559.43	163.666
163.9132	19568.8	163.9185	19568.33	163.9132	19568.35	163.9167	19569.66	163.9183	19558.17	163.9166	19559.15	163.9151
164.1686	19570.38	164.1637	19569.7	164.1668	19568.84	164.1628	19571.27	164.1675	19558.25	164.1668	19558.78	164.1676
164.4125	19569.02	164.4194	19568.3	164.412	19570.87	164.411	19570.02	164.4189	19557.06	164.4141	19560.03	164.428
164.6633	19568.71	164.6636	19570.18	164.6632	19568.05	164.6605	19569.42	164.6668	19558.86	164.6711	19557.24	164.6723
164.9152	19570.95	164.9187	19569.2	164.9168	19570.69	164.9166	19569.45	164.9188	19558.97	164.9152	19559.13	164.9165
165.1692	19569.19	165.1651	19570.02	165.1651	19569.92	165.1619	19570.25	165.1651	19560.56	165.169	19559.03	165.167
165.4123	19569.58	165.4192	19569.33	165.4116	19568.98	165.4117	19569.85	165.4174	19557.05	165.4155	19558.54	165.4209
165.6638	19568.91	165.6637	19570.34	165.6626	19567.94	165.6637	19568.73	165.6665	19557.89	165.6713	19559.66	165.6683
165.9135	19570.11	165.9201	19570.86	165.9166	19571.55	165.9179	19571.33	165.9191	19557.42	165.9155	19559.38	165.9181
166.1735	19568.78	166.1665	19569.4	166.1636	19571.7	166.1639	19571.31	166.1791	19558.41	166.1686	19558.69	166.1669
166.4142	19567.16	166.4206	19568.97	166.411	19568.48	166.4117	19570.58	166.4187	19556.56	166.4157	19561.29	166.4217
166.6634	19568.11	166.6639	19565.96	166.6624	19569.74	166.663	19569.76	166.6663	19556.97	166.6704	19559.44	166.667
166.9121	19569.86	166.9209	19569.27	166.9175	19567.41	166.9173	19570.58	166.9192	19556.74	166.927	19557.51	166.9179
167.1695	19567.08	167.1661	19570.17	167.1636	19568.95	167.1746	19570.07	167.17	19560.19	167.1751	19558.24	167.1687
167.4136	19566.86	167.4198	19568.41	167.4123	19570.23	167.41	19571.63	167.4182	19556.92	167.4151	19558.25	167.4236
167.6642	19568.58	167.665	19570.48	167.6641	19570.38	167.6662	19568.05	167.6664	19559.82	167.6723	19560.01	167.6692
167.9134	19568.96	167.9208	19569.95	167.9163	19570.79	167.9179	19569.68	167.9188	19557.38	167.9166	19557.69	167.9174
168.1706	19570.35	168.1653	19570.06	168.1697	19568.28	168.1636	19570.24	168.1719	19561.48	168.1698	19557.99	168.1675
168.4134	19566.77	168.4203	19569.56	168.412	19566.88	168.4124	19568.99	168.4194	19557.08	168.4165	19559.58	168.4252
168.6646	19568.61	168.6652	19570.41	168.6655	19568.38	168.6638	19571.25	168.67	19556.2	168.6736	19558.19	168.6709
168.9147	19569.01	168.92	19570.37	168.9168	19569.57	168.917	19571.65	168.9195	19556.93	168.9169	19558.88	168.918
169.1722	19569.85	169.1668	19569.9	169.1652	19570.32	169.1653	19568.68	169.1685	19556.78	169.1703	19560.73	169.1683
169.4147	19570.65	169.421	19570.31	169.4136	19566.91	169.4122	19570.65	169.4226	19559.05	169.4154	19558.52	169.4298
169.6658	19565.9	169.6674	19569.16	169.6649	19568.17	169.6651	19567.41	169.6675	19557.05	169.672	19558.62	169.6751
169.9158	19568.73	169.9203	19569.16	169.9152	19568.64	169.9193	19568.62	169.9206	19556.85	169.9183	19558.03	169.9198
170.1747	19568.04	170.1668	19570.34	170.1738	19570.95	170.1629	19569.68	170.1711	19557.1	170.1707	19557.26	170.168
170.4125	19566.17	170.4206	19569.14	170.424	19570	170.4123	19570.91	170.4206	19558.06	170.4161	19557.34	170.4255
170.6841	19566.82	170.6664	19569.39	170.6646	19568.62	170.6652	19570.12	170.6677	19557.68	170.6745	19558.98	170.6687
170.9162	19570.3	170.9246	19568.59	170.9152	19571.57	170.9196	19570.19	170.9215	19558.77	170.9174	19558.88	170.9203
171.1716	19568.66	171.1694	19569.22	171.165	19570.22	171.1658	19568.6	171.1704	19558.75	171.1702	19557.81	171.1685
171.4155	19568.41	171.4238	19569.98	171.4121	19569.97	171.4137	19570.8	171.4202	19558.36	171.4176	19556.97	171.426
171.6662	19569.37	171.6671	19569.11	171.6653	19571.91	171.6661	19570.34	171.6678	19560.92	171.673	19558.27	171.6693
171.915	19568.5	171.9421	19568.65	171.9201	19569.63	171.9171	19570.21	171.9207	19554.83	171.9185	19558.86	171.9194
172.1708	19567.93	172.172	19569.05	172.1652	19568.06	172.1645	19569.78	172.1715	19556.27	172.1732	19557.83	172.1686
172.4145	19568.74	172.4237	19566.69	172.4135	19568.98	172.4143	19570.47	172.4198	19556.67	172.4186	19560.8	172.4271
172.6644	19567.38	172.6689	19567.35	172.6645	19569.2	172.6645	19568.85	172.6688	19558.06	172.6748	19558.37	172.6692
172.9163	19568.48	172.922	19569.3	172.9186	19567.49	172.9198	19569.13	172.9218	19559.46	172.9193	19558.32	172.9193
173.1748	19568.88	173.169	19568.76	173.1662	19570.48	173.1664	19569.77	173.1718	19555.72	173.1718	19557.55	173.1699
173.4138	19569.14	173.4239	19570.6	173.4127	19568.1	173.4133	19568.87	173.4225	19557.51	173.4196	19558.25	173.4258
173.6692	19568.01	173.6685	19567.95	173.6658	19569.17	173.6642	19570.36	173.6709	19557.8	173.6744	19559.55	173.6709

173.9165	19566.24	173.9278	19568.53	173.9178	19569.79	173.922	19570.26	173.9222	19557.89	173.9185	19557.38	173.9208
174.1727	19569.17	174.1705	19567.87	174.1647	19569.57	174.167	19570.77	174.1715	19557.38	174.1743	19559.57	174.1688
174.4148	19568.4	174.4241	19566.78	174.4166	19566.94	174.4144	19568.85	174.4232	19556.26	174.4182	19559.31	174.4254
174.6678	19567.83	174.6695	19567.43	174.6672	19569.4	174.6652	19570.48	174.6737	19558.03	174.6747	19558.33	174.671
174.9178	19565.63	174.9252	19567.02	174.9183	19568.93	174.9193	19569.08	174.9212	19558.29	174.9207	19557.32	174.9228
175.1728	19568.8	175.1709	19568.08	175.1652	19566.74	175.1671	19571.24	175.1727	19557.67	175.1721	19558.79	175.1704
175.4141	19568.47	175.4239	19567.51	175.4137	19570.17	175.4151	19570.27	175.4214	19556.78	175.4207	19560.27	175.4319
175.6692	19569.04	175.669	19566.92	175.6665	19568.71	175.6869	19569.92	175.6703	19558.04	175.68	19557.56	175.6714
175.9184	19565.47	175.9239	19570.61	175.9185	19566.69	175.92	19568.05	175.9227	19558.61	175.9209	19560.48	175.9233
176.1733	19567.99	176.1713	19566	176.1681	19570.2	176.1671	19571.72	176.1713	19556.1	176.1725	19557.47	176.1717
176.4158	19568.16	176.4257	19568.5	176.4153	19568.63	176.4166	19568.67	176.4251	19558.05	176.4216	19554.75	176.4274
176.667	19570.06	176.6694	19569.95	176.6661	19566.91	176.6694	19568.71	176.6787	19558.94	176.7007	19558.89	176.6708
176.9173	19567.7	176.923	19568.14	176.9188	19569.1	176.9242	19569.86	176.9223	19557.9	176.9338	19558.54	176.9228
177.1725	19567.05	177.1713	19568.9	177.1685	19567.51	177.1671	19571.98	177.1719	19556.77	177.1759	19558.51	177.1718
177.4171	19566.91	177.4253	19569.02	177.4178	19568.21	177.4144	19569.34	177.4213	19555.63	177.4202	19557.41	177.4282
177.6675	19568.23	177.6698	19566.51	177.6666	19567.96	177.6674	19570.89	177.6734	19556.25	177.6768	19558.64	177.6708
177.9195	19567.73	177.9258	19568.49	177.9184	19569.78	177.9228	19568.08	177.9226	19558.05	177.9228	19558.2	177.9239
178.1754	19569.28	178.1715	19569.22	178.1665	19568.09	178.1687	19569.93	178.1747	19558.96	178.1739	19560.23	178.1709
178.4177	19568.22	178.4249	19568.38	178.4172	19566.96	178.4189	19569.07	178.4279	19556.73	178.4204	19556.23	178.4293
178.6696	19568.85	178.6705	19568.85	178.6692	19568.63	178.6678	19568.5	178.6738	19556.66	178.6756	19558.27	178.6727
178.9186	19567.78	178.9247	19567.85	178.9186	19567.45	178.9219	19572.78	178.924	19557.59	178.921	19559.13	178.9229
179.1751	19568.9	179.1718	19566.09	179.1668	19566.86	179.1689	19569.87	179.1739	19558.46	179.1738	19557.68	179.173
179.4179	19568.12	179.4246	19568.72	179.4164	19568.09	179.4167	19569.48	179.4263	19558.11	179.4213	19557.4	179.4333
179.6711	19567.71	179.6704	19567.74	179.6693	19569.84	179.6739	19570	179.6735	19556.33	179.6771	19558.18	179.6807
179.9172	19568.05	179.9256	19568.13	179.917	19567.68	179.9258	19567.45	179.924	19555.79	179.9244	19557.47	179.924
180.1745	19567.78	180.1724	19566.53	180.1698	19567.57	180.1694	19568.81	180.1745	19557.52	180.1771	19558.87	180.1721
180.419	19570.3	180.4255	19568.03	180.4185	19568.72	180.4165	19571.01	180.4251	19557.61	180.4209	19557.7	180.4294
180.671	19568	180.6692	19566.35	180.6682	19567.96	180.6709	19570.27	180.6731	19556.45	180.6759	19557.37	180.6737
180.9188	19567.98	180.9287	19570.18	180.9219	19567.42	180.9221	19570.01	180.9257	19555.2	180.9254	19559.14	180.9255
181.174	19567.02	181.1728	19567.21	181.1782	19569.4	181.169	19569.48	181.1755	19558.5	181.1761	19557.45	181.1739
181.4185	19566.08	181.4262	19569.56	181.4191	19568.06	181.4177	19570.71	181.4253	19557.92	181.4235	19558.94	181.4432
181.6714	19568.05	181.6713	19567.56	181.6692	19567.31	181.6695	19570.6	181.6725	19557.06	181.677	19557.19	181.679
181.921	19566.68	181.9247	19569.44	181.9196	19567.71	181.9223	19569.89	181.9253	19558.4	181.9236	19558	181.9239
182.1768	19567.53	182.1723	19567.06	182.1683	19568.01	182.1814	19571.56	182.176	19555.58	182.179	19558.41	182.175
182.418	19567.62	182.4246	19568.01	182.4215	19568.66	182.4182	19570.64	182.4251	19560.5	182.4248	19558.41	182.4299
182.6721	19567.12	182.6729	19569.35	182.6706	19570.28	182.6711	19568.28	182.6744	19558.31	182.6801	19557.16	182.6752
182.9219	19566.68	182.9294	19569.05	182.9192	19569.67	182.9223	19570	182.9266	19558.28	182.9232	19558.56	182.9257
183.1773	19567.37	183.172	19569.15	183.1706	19569.61	183.1722	19569.79	183.1801	19556.05	183.1772	19558.74	183.1754
183.4192	19567.11	183.4275	19568.71	183.4193	19567.75	183.4173	19567.25	183.4255	19555.55	183.4243	19557.58	183.4341
183.6722	19564.95	183.6743	19568.38	183.6707	19568.77	183.6698	19568.31	183.6738	19556.96	183.6757	19555.94	183.6827
183.9209	19568.63	183.9252	19568.86	183.9206	19567.1	183.929	19571.85	183.9264	19555.94	183.9261	19556.86	183.928
184.1767	19567.54	184.1728	19567.69	184.1726	19570.15	184.1728	19570.66	184.178	19555.75	184.1774	19557.51	184.1736
184.4193	19567.72	184.4261	19565.58	184.4196	19566.68	184.419	19569.49	184.4275	19556.11	184.4231	19556.87	184.4309
184.6731	19566.95	184.6735	19569.7	184.6702	19569.05	184.6694	19567.53	184.6734	19558.35	184.6784	19556.74	184.6743
184.9204	19567.61	184.9272	19567.94	184.9221	19569.42	184.9235	19566.32	184.9271	19556.71	184.9388	19558.44	184.9256
185.1767	19565.95	185.1745	19567.88	185.1708	19568.12	185.1718	19570.25	185.179	19556.63	185.1778	19557.42	185.1751
185.4199	19566.9	185.4302	19567.31	185.4203	19567.93	185.4199	19569.57	185.4273	19554.28	185.4241	19555.34	185.4303
185.6729	19567.78	185.6747	19567.05	185.6718	19565.09	185.6717	19567.99	185.6763	19558.9	185.6798	19558.07	185.6747
185.9216	19568.79	185.9287	19568.99	185.9224	19567.35	185.9239	19569.11	185.9277	19558.89	185.9372	19554.88	185.9272
186.177	19570.14	186.1752	19568.99	186.1714	19566.76	186.1722	19566.57	186.1814	19556.47	186.1787	19558.72	186.1741
186.4204	19569.04	186.4326	19567.39	186.4211	19564.82	186.42	19570.31	186.4259	19556.92	186.4264	19558.66	186.435
186.6736	19564.85	186.6745	19567.05	186.67	19567.4	186.6728	19569.72	186.6765	19556.61	186.678	19558.54	186.6746
186.9225	19568.14	186.9283	19565.54	186.9217	19566.8	186.926	19569	186.9266	19555.56	186.9256	19560.4	186.9253
187.1791	19566.88	187.1748	19566.66	187.1733	19568.27	187.1733	19570.28	187.1791	19556.85	187.184	19559.85	187.1743
187.4204	19567.27	187.4283	19565.59	187.4217	19567.47	187.4202	19566.62	187.4274	19555.49	187.429	19557.49	187.4315
187.6756	19566.84	187.6786	19565.1	187.6735	19566.88	187.6718	19570.29	187.6737	19556.15	187.6794	19557.69	187.6769
187.9224	19567.44	187.9306	19568.77	187.9226	19570.7	187.9264	19571.32	187.9278	19554.98	187.9273	19557.41	187.9259
188.1779	19567.64	188.1749	19566.31	188.1735	19567.86	188.1723	19569	188.1777	19557.82	188.1833	19557.89	188.1758
188.4214	19566.6	188.4275	19566.51	188.4219	19568.46	188.4209	19567.06	188.4264	19556.33	188.4251	19556.47	188.4309
188.6754	19566.7	188.6763	19567.01	188.6734	19567.12	188.6719	19569.62	188.6758	19557.01	188.6812	19558.8	188.678
188.9205	19566.02	188.9298	19567.8	188.9219	19566.82	188.9262	19568.27	188.9283	19553.99	188.9266	19557.84	188.9285
189.1761	19566.8	189.1752	19565.36	189.1736	19569.19	189.1743	19568	189.1782	19555.22	189.1791	19558.58	189.1857
189.4231	19568.06	189.4287	19568.23	189.4214	19566.96	189.4211	19569.22	189.4292	19556.93	189.4267	19557.48	189.4343
189.6739	19566.9	189.6769	19565.63	189.6722	19567.91	189.6735	19571.7	189.677	19558.43	189.6799	19556.23	189.6816
189.9227	19567.14	189.9289	19567.78	189.9246	19567.67	189.9246	19567.24	189.9288	19556.91	189.9279	19556.65	189.9279
190.1801	19564.76	190.1769	19566.49	190.1734	19566.15	190.1731	19567.78	190.1795	19555.56	190.1777	19556.88	190.1786
190.4227	19568.04	190.4302	19568.3	190.4233	19569.88	190.4219	19569.76	190.4283	19554.96	190.426	19556.34	190.4318
190.6734	19568.49	190.6778	19565.92	190.6741	19570.2	190.6732	19567.41	190.6755	19558.03	190.6824	19555.74	190.68
190.9239	19567.46	190.9316	19567.82	190.9262	19570.26	190.9251	19570.62	190.9299	19555.55	190.9309	19557	190.9301
191.1804	19568.32	191.1784	19567.1	191.1743	19567.77	191.1729	19570.27	191.1805	19557.1	191.1848	19556.6	191.1793

191.4235	19566.84	191.4309	19566.62	191.4241	19568.63	191.421	19569.16	191.4283	19557.76	191.4261	19557.54	191.4322
191.677	19568.12	191.6807	19565.69	191.6736	19567.19	191.6741	19569.18	191.6802	19556.07	191.6818	19556.39	191.679
191.9227	19569.8	191.9295	19566.93	191.9254	19567.66	191.9249	19569.04	191.9301	19555.94	191.9276	19557.51	191.9283
192.1793	19566.26	192.1771	19568.58	192.1738	19567.94	192.1729	19567.16	192.1836	19557.24	192.1826	19555.87	192.1788
192.4241	19567.82	192.4286	19566.89	192.4239	19569.42	192.4217	19568.62	192.4395	19556.12	192.4272	19555.12	192.437
192.6745	19566.28	192.6771	19566.88	192.6753	19567.87	192.6739	19569.51	192.6791	19556.48	192.6815	19556.54	192.6783
192.9238	19567	192.9297	19565.93	192.9258	19567.84	192.9276	19570.5	192.9299	19556.09	192.9271	19554.95	192.9275
193.1789	19566.91	193.1776	19566.38	193.177	19568.03	193.1742	19567.38	193.1917	19554.12	193.1806	19554.13	193.1791
193.4232	19565.84	193.4304	19567.86	193.4236	19567.57	193.4229	19570.64	193.4444	19556.48	193.4272	19558.12	193.4375
193.6765	19565.69	193.6773	19568.4	193.6755	19566.54	193.6732	19569	193.6824	19557.65	193.682	19555.47	193.6882
193.9252	19565.65	193.9343	19565.82	193.9238	19570.42	193.928	19569.29	193.934	19558.79	193.9277	19557.07	193.9292
194.1811	19566.52	194.1769	19566.98	194.1867	19565.2	194.1741	19569.07	194.1812	19555.71	194.1808	19556.95	194.1805
194.4228	19566.06	194.4315	19567.27	194.4223	19566.15	194.4239	19567.58	194.4332	19558.31	194.4269	19558.09	194.4352
194.6772	19567.67	194.6782	19566.26	194.6749	19566.75	194.6742	19568.21	194.6784	19558.53	194.6838	19558.02	194.6801
194.9269	19568.73	194.9314	19566.6	194.9287	19567.99	194.9265	19568.73	194.9319	19556.45	194.9274	19557.07	194.9303
195.182	19569.3	195.1793	19566.28	195.1765	19565.37	195.174	19570.41	195.1802	19555.9	195.1805	19556.4	195.1809
195.424	19568.26	195.4311	19566.58	195.4258	19567.04	195.4243	19567.63	195.4328	19557.5	195.4278	19554.3	195.4355
195.6751	19566.22	195.6787	19567.83	195.6742	19565.49	195.6762	19569.42	195.6785	19554.82	195.6835	19555.36	195.6789
195.926	19567.94	195.9322	19567.18	195.9253	19567.45	195.9258	19568.76	195.9324	19556.11	195.9279	19556.99	195.9312
196.1819	19567.26	196.1789	19567.45	196.1748	19567.29	196.1739	19568.48	196.1811	19555.92	196.1814	19555.26	196.1813
196.4245	19567.47	196.4327	19566.91	196.4241	19567.99	196.424	19571.53	196.4314	19556.33	196.4282	19554.43	196.4358
196.6765	19566.59	196.6818	19568.53	196.6747	19566.46	196.6754	19571.28	196.6795	19557.46	196.6822	19556.53	196.68
196.9258	19567.8	196.9322	19567.82	196.9261	19569.53	196.9265	19568.63	196.9319	19554.74	196.9294	19558.78	196.9291
197.1819	19566.89	197.1799	19567.63	197.1759	19567.81	197.1762	19567.05	197.1831	19555.33	197.1852	19556.02	197.1819
197.4255	19565.59	197.4335	19567.96	197.4251	19566.94	197.4265	19569.95	197.4323	19556.43	197.4284	19557.16	197.436
197.6782	19565.52	197.6805	19566.32	197.6764	19565.39	197.6753	19568.81	197.6818	19556.51	197.6834	19557.48	197.6834
197.9267	19566.83	197.9357	19564.98	197.9271	19567.41	197.9334	19567.9	197.9342	19552.12	197.9299	19556.74	197.9304
198.189	19567.29	198.1886	19566.66	198.1837	19567.09	198.1869	19568.4	198.1939	19556.83	198.1931	19556.32	198.1908
198.4262	19569.07	198.4324	19569.63	198.4249	19568.44	198.4255	19567.8	198.433	19555.7	198.4298	19558.69	198.4351
198.6783	19567.54	198.6807	19567.87	198.6755	19568.99	198.6779	19567.99	198.6794	19558.69	198.6826	19557.91	198.6828
198.93	19564.14	198.9333	19569.17	198.9283	19565.33	198.9315	19569.39	198.9339	19555.57	198.9313	19558.15	198.9312
199.1827	19566.67	199.18	19566.24	199.1798	19567.2	199.176	19567.44	199.1863	19556.33	199.1837	19556.04	199.1829
199.4247	19566.66	199.4343	19567	199.4257	19568.88	199.4257	19571.73	199.4347	19555.03	199.4306	19557.4	199.4374
199.6792	19569.63	199.6823	19566.08	199.6763	19565.92	199.6773	19567.91	199.6811	19554.02	199.683	19558.7	199.6837
199.9427	19564.57	199.9332	19565.91	199.9287	19566.71	199.931	19568.33	199.9349	19559.41	199.9304	19557.76	199.934
200.1847	19567.22	200.1814	19569.56	200.1782	19566.3	200.1772	19567.75	200.1823	19555.73	200.1841	19556.07	200.1841
200.4294	19568.47	200.4426	19569.72	200.4288	19567.19	200.4333	19566.41	200.4381	19554.65	200.4339	19557.35	200.4418
200.6834	19566.48	200.6843	19566.06	200.6811	19566.91	200.6795	19569	200.6844	19558.14	200.6898	19557.34	200.686
200.934	19568.43	200.9368	19567.71	200.9344	19564.97	200.9342	19567.81	200.9377	19558.98	200.9346	19557.29	200.9379
201.1894	19567.46	201.1863	19567.2	201.1863	19567.32	201.1817	19568.43	201.1905	19555.89	201.1875	19560.51	201.1873
201.4312	19567.1	201.4381	19567.66	201.4315	19568.33	201.4316	19567.66	201.4389	19557.55	201.4344	19555.71	201.4424
201.6817	19565.08	201.7026	19565.88	201.6825	19567.55	201.6808	19566.07	201.69	19556.57	201.6881	19557.66	201.6871
201.9334	19564.63	201.9394	19566.29	201.9322	19566.19	201.9338	19569.25	201.938	19555.05	201.9358	19557.89	201.9361
202.1885	19566.74	202.1852	19567.13	202.1806	19569.55	202.1816	19569.21	202.1887	19557.03	202.1896	19557.83	202.1889
202.4319	19568.64	202.4388	19566.46	202.4303	19566.93	202.4324	19566.85	202.4384	19556.58	202.4365	19557.23	202.4435
202.6836	19565.17	202.6865	19565.56	202.6845	19566.16	202.6831	19567.94	202.6844	19557.24	202.69	19558.75	202.6862
202.9318	19565.59	202.9389	19567.81	202.9329	19568.14	202.9327	19565.25	202.9402	19555.97	202.9384	19558.31	202.9387
203.1869	19567.02	203.1859	19566.26	203.1943	19571.04	203.1818	19565.01	203.1902	19554.95	203.1931	19557.61	203.1888
203.4325	19569.19	203.4386	19566.59	203.4293	19569.37	203.4327	19569.61	203.4394	19556.4	203.435	19556.94	203.442
203.6848	19568.35	203.6872	19565.66	203.6835	19566.99	203.6841	19566.91	203.6939	19553.16	203.6881	19557.25	203.6859
203.936	19567.83	203.9398	19568.05	203.9347	19565.83	203.9356	19567.89	203.939	19555.96	203.9381	19556.27	203.9391
204.194	19566.42	204.1856	19566.92	204.1821	19567.02	204.1851	19568.17	204.1924	19554.44	204.1906	19558.29	204.1867
204.4316	19565.18	204.4408	19566.09	204.4327	19566.39	204.4316	19567.38	204.441	19554.72	204.4408	19557.79	204.4444
204.685	19566.6	204.6875	19566.21	204.6821	19568.8	204.6838	19566.76	204.6896	19555.44	204.6907	19558.43	204.6891
204.936	19562.72	204.9387	19565.42	204.9359	19566.83	204.9365	19569.89	204.94	19557.71	204.94	19557.89	204.9374
205.1896	19568.14	205.1851	19567.32	205.1845	19569.57	205.1832	19568.16	205.1888	19556.86	205.1911	19556.34	205.1864
205.4335	19566.8	205.4406	19568.97	205.4322	19565.78	205.4347	19570.26	205.4406	19555.57	205.4368	19556.42	205.4465
205.6854	19566.83	205.6872	19565.58	205.6854	19567.03	205.6838	19568.75	205.6893	19554.58	205.6937	19556.38	205.7016
205.9365	19567.35	205.9414	19568.21	205.9321	19570.55	205.9348	19568.38	205.9413	19554.16	205.9378	19556.38	205.9404
206.1917	19567.56	206.1858	19567.12	206.1837	19565.75	206.1837	19566.64	206.1903	19556.73	206.191	19557	206.1882
206.433	19563.92	206.4405	19568.06	206.4335	19567.69	206.437	19568.27	206.4402	19555.35	206.4385	19559.08	206.4467
206.6845	19565.56	206.6884	19568.63	206.687	19568.12	206.6841	19569.26	206.6906	19555.14	206.6909	19555	206.689
206.9355	19567.08	206.9401	19567.72	206.9341	19568.2	206.9373	19567.14	206.9437	19559.06	206.9408	19558.27	206.9407
207.1911	19566.2	207.1877	19568.16	207.1844	19568.26	207.184	19569.42	207.1933	19556.53	207.1918	19557.55	207.1902
207.4338	19567.35	207.4414	19568.61	207.4335	19567.22	207.4341	19569.1	207.4416	19555.7	207.4388	19558.68	207.4467
207.6852	19566	207.7028	19567.94	207.6858	19570.1	207.6851	19569.11	207.6877	19556.25	207.6903	19555.21	207.6897
207.9349	19566.95	207.9442	19567.38	207.939	19565.76	207.9353	19567.52	207.9409	19553.67	207.9387	19554.91	207.94
208.1976	19566.26	208.1872	19567.29	208.1851	19567.34	208.1837	19567.11	208.1933	19556.88	208.1928	19556.82	208.1896
208.4447	19565.61	208.4408	19565.24	208.434	19566	208.4387	19567.5	208.4422	19554.63	208.438	19558.85	208.445
208.6876	19567.49	208.689	19567.15	208.6876	19565.34	208.6866	19567.28	208.6881	19555.81	208.6958	19556.44	208.689

208.9361	19565.99	208.9433	19565.17	208.9376	19567.83	208.9374	19567.37	208.9411	19556.73	208.9391	19555.96	208.9416
209.1918	19568.22	209.1881	19566.22	209.1848	19568.28	209.1826	19568.5	209.1927	19555.85	209.1931	19557.62	209.1912
209.4345	19567.55	209.441	19565.72	209.4339	19566.75	209.436	19568.47	209.4437	19556.88	209.4395	19554.34	209.4477
209.6866	19566.21	209.6903	19567.61	209.6868	19566.41	209.6838	19571.33	209.6901	19554.06	209.6915	19556.72	209.6902
209.9377	19569.7	209.9436	19567.71	209.9351	19569.09	209.9373	19568.73	209.9426	19556.29	209.9398	19558.52	209.942
210.1942	19565.9	210.1882	19565.5	210.187	19566.65	210.1851	19568.16	210.1917	19553.93	210.1916	19556.6	210.1898
210.434	19565.28	210.4425	19565.46	210.4345	19563.61	210.4351	19567.56	210.4419	19555.37	210.4391	19555.57	210.446
210.6847	19568.51	210.687	19564.59	210.687	19567.85	210.6875	19569.11	210.6892	19555.71	210.6935	19558.31	210.6903
210.9376	19568.94	210.9442	19566.43	210.9366	19564.65	210.9404	19568.9	210.9423	19555.56	210.9393	19556.68	210.9419
211.1943	19565.74	211.1891	19567.36	211.1868	19566.78	211.192	19568.06	211.1936	19554.48	211.1955	19556.77	211.1901
211.4353	19566.7	211.443	19565.39	211.4362	19566.45	211.4361	19569.63	211.4427	19557.6	211.4408	19555.38	211.4479
211.686	19567.14	211.6901	19566.22	211.6885	19569.75	211.6857	19568.15	211.6907	19555.65	211.6941	19552.66	211.6908
211.9355	19563.29	211.944	19566.37	211.9369	19566.91	211.9378	19568.78	211.9426	19554.69	211.9411	19556.14	211.9419
212.1927	19565.8	212.1916	19569.11	212.1848	19567.15	212.1861	19568.85	212.1932	19554.84	212.1973	19553.18	212.1899
212.4385	19566.22	212.442	19567	212.4406	19566.16	212.4373	19569.01	212.4437	19556.65	212.443	19556.79	212.449
212.6876	19566.05	212.6925	19565.88	212.6876	19564.89	212.6865	19566.1	212.6897	19554.21	212.6949	19556.06	212.6917
212.9378	19566.05	212.944	19566.35	212.9391	19567.79	212.9382	19569.53	212.954	19558.6	212.9407	19556.08	212.9414
213.1925	19567.11	213.1904	19563.81	213.1872	19569.57	213.1858	19567.02	213.198	19555.37	213.1929	19555.8	213.1948
213.4387	19564.86	213.4446	19565.88	213.4365	19567.58	213.437	19568.1	213.4478	19559.18	213.4406	19558.81	213.4497
213.6892	19564.05	213.6911	19565.95	213.69	19567.38	213.688	19567.5	213.6911	19554.82	213.6936	19557.16	213.6913
213.9414	19566.69	213.9476	19565.8	213.9368	19564.96	213.9417	19567.34	213.9455	19557.01	213.9421	19556.3	213.9438
214.1993	19564.38	214.1922	19567.75	214.1885	19567.69	214.1865	19568.84	214.2017	19557.18	214.1985	19557.62	214.1926
214.4529	19565.67	214.4456	19565.41	214.4366	19567.84	214.4348	19566.87	214.4514	19556.45	214.4411	19557.66	214.4489
214.6882	19567.41	214.6921	19565.37	214.6899	19566.64	214.692	19567.14	214.6902	19555.12	214.695	19556.91	214.6924
214.9385	19566.82	214.946	19567.7	214.9373	19568.74	214.9441	19566.16	214.9451	19553.39	214.9451	19555.62	214.9433
215.1958	19566.72	215.1927	19566.35	215.1886	19566.61	215.1874	19567.27	215.1966	19555.25	215.1967	19553.7	215.1915
215.438	19567.08	215.4429	19566.26	215.437	19566.29	215.4377	19570	215.4446	19556.94	215.4427	19555.47	215.449
215.6895	19564.14	215.6938	19567.15	215.6905	19570.13	215.6886	19567.48	215.6929	19553.6	215.6949	19557.3	215.6932
215.9391	19566.59	215.9459	19568.66	215.9385	19566.39	215.9417	19570.06	215.9522	19557.29	215.9427	19557.71	215.9443
216.1944	19566.37	216.1916	19564.32	216.1879	19566.26	216.188	19568.31	216.2078	19555.87	216.196	19558.09	216.1921
216.4374	19565.69	216.4466	19565.62	216.4373	19566.98	216.4378	19567.45	216.4499	19555.51	216.4423	19555.01	216.4513
216.69	19565.23	216.6919	19565.85	216.6909	19567.34	216.6943	19568.04	216.6925	19556.32	216.6965	19555.76	216.6929
216.9373	19567.26	216.948	19565.26	216.9386	19566.03	216.9397	19566.4	216.9469	19554.41	216.9448	19554.15	216.943
217.197	19568.01	217.1928	19566.17	217.1884	19564.54	217.1881	19566.95	217.2049	19558.28	217.1986	19556.35	217.1926
217.4398	19564.35	217.4457	19567.4	217.4376	19567.17	217.4369	19567.51	217.4419	19555.14	217.4437	19557.33	217.4499
217.6889	19563.86	217.6962	19565.63	217.689	19565.91	217.69	19568.61	217.6929	19555.38	217.6954	19556.83	217.6944
217.9393	19565.14	217.9467	19565.79	217.9396	19566.94	217.9409	19566.07	217.9462	19556.78	217.9459	19556.05	217.9444
218.1974	19567.77	218.1932	19565.21	218.1868	19567.79	218.1876	19567.84	218.1997	19559.69	218.2001	19557.17	218.1938
218.4391	19565.49	218.4451	19567.46	218.4383	19566.57	218.4379	19567.93	218.4648	19555.25	218.447	19555.34	218.4518
218.6896	19564.69	218.6963	19565.96	218.6902	19567.75	218.6892	19566.53	218.7052	19555.18	218.6995	19558.68	218.6943
218.94	19563.62	218.9479	19565.23	218.939	19566.33	218.9403	19567.37	218.942	19554.6	218.9447	19559.03	218.9452
219.1988	19564.84	219.1935	19566.32	219.1878	19565.73	219.189	19564.77	219.1998	19554.09	219.198	19557	219.1942
219.4387	19564.88	219.4468	19566.68	219.4383	19569.02	219.4374	19567.15	219.4454	19554.38	219.4436	19555.87	219.4515
219.6909	19565.28	219.6964	19565.79	219.6908	19565.31	219.6905	19567.07	219.6943	19556.97	219.6997	19556.85	219.6943
219.94	19565.97	219.9488	19568.35	219.9391	19567.08	219.942	19571.15	219.9514	19556.69	219.9439	19557.49	219.9479
220.198	19564.37	220.1929	19565.82	220.19	19564.78	220.1897	19567.66	220.1987	19557.35	220.1959	19555.67	220.1992
220.4401	19565.87	220.4456	19565.77	220.4376	19566.79	220.4389	19568.42	220.4476	19555.98	220.4462	19556.8	220.4515
220.6911	19565.75	220.696	19565.14	220.6903	19566.6	220.6918	19566.36	220.6987	19555.57	220.6976	19553.77	220.6955
220.9418	19565.34	220.9481	19566.11	220.9419	19566.29	220.9505	19566.15	220.9473	19557.41	220.9442	19556.34	220.9475
221.199	19566.87	221.1943	19565.88	221.191	19566.24	221.1951	19565.98	221.2065	19557.51	221.1963	19556.03	221.1958
221.4416	19566.16	221.4464	19566.55	221.4403	19567.44	221.4394	19568.9	221.4465	19556.33	221.4465	19557.83	221.4526
221.6936	19566.61	221.6952	19564.72	221.6886	19566.13	221.6915	19566.39	221.6993	19552.21	221.6969	19555.09	221.696
221.9401	19563.32	221.9468	19567.61	221.9409	19567.51	221.9456	19565.59	221.9477	19554.47	221.9454	19553.94	221.9489
222.199	19565.81	222.1945	19566.28	222.1921	19564.83	222.1908	19567.52	222.2026	19556.06	222.1998	19556.15	222.1955
222.4427	19566.91	222.4476	19564.65	222.4407	19566.93	222.4435	19568	222.4464	19555.15	222.446	19554.79	222.4551
222.6944	19566.1	222.6957	19566.21	222.6897	19565.54	222.6938	19567.92	222.6947	19555.29	222.6983	19556.83	222.6934
222.9421	19566.3	222.9493	19563.66	222.9414	19565.33	222.942	19564.56	222.949	19556.02	222.9487	19555.03	222.9492
223.1992	19566.03	223.1959	19565.15	223.1902	19565.01	223.1908	19565.13	223.2006	19553.3	223.1978	19553.68	223.1974
223.4433	19567.82	223.4485	19568.09	223.4407	19566.25	223.4397	19566.09	223.4494	19557.41	223.4477	19555.46	223.4543
223.6937	19567.16	223.6982	19565.63	223.6917	19565.8	223.6932	19567.45	223.6958	19554.13	223.7019	19554.21	223.6929
223.9416	19565.59	223.9464	19565.1	223.9407	19565.58	223.9432	19569.24	223.9493	19553.51	223.9525	19558.18	223.9505
224.1984	19565.38	224.1973	19564.78	224.1903	19564.76	224.191	19569.02	224.2021	19556.09	224.1948	19554.82	224.1994
224.4421	19566.43	224.449	19565.44	224.4424	19564.74	224.4388	19566.57	224.448	19554.52	224.452	19554.71	224.4534
224.6942	19564.29	224.7087	19567.53	224.6925	19566.71	224.6919	19567.6	224.6954	19554.75	224.6982	19555.96	224.6941
224.9436	19566.05	224.9531	19564.89	224.9423	19567.75	224.9422	19568.98	224.9484	19556.05	224.949	19555.89	224.9498
225.201	19563.78	225.1925	19567.95	225.1935	19565.33	225.1926	19567.21	225.2017	19553.7	225.2005	19554.83	225.198
225.4412	19566.24	225.4501	19564.79	225.4407	19564.77	225.4411	19566.85	225.4437	19554.85	225.4483	19556.67	225.4529
225.6956	19566.3	225.7003	19563.57	225.6925	19567.91	225.6961	19568.45	225.6959	19556.93	225.6985	19555.82	225.6934
225.9433	19566.98	225.9526	19565.82	225.9441	19569	225.9416	19566.57	225.9485	19552.38	225.9436	19554.75	225.9594
226.2016	19564.91	226.194	19564.25	226.1926	19566.93	226.1955	19568.4	226.2001	19553.71	226.1977	19552.9	226.2017

226.442	19565.5	226.4491	19564.56	226.4426	19567.55	226.4412	19569.36	226.4517	19555.09	226.4498	19555.21	226.4472
226.6942	19566.53	226.6981	19566.37	226.6936	19567.3	226.6956	19566.98	226.6926	19555.91	226.7006	19554.49	226.6979
226.9443	19564.91	226.9514	19566.05	226.9448	19564.99	226.9425	19567.15	226.9498	19555.6	226.9488	19556.55	226.9486
227.1995	19564.77	227.1961	19566.49	227.1951	19567.01	227.1922	19567.07	227.2008	19555.28	227.2196	19555.18	227.1978
227.445	19565.73	227.4494	19565.61	227.4427	19564.53	227.4425	19565.23	227.4514	19556.16	227.4733	19557	227.457
227.6944	19565.04	227.6996	19566.54	227.6936	19564.44	227.6957	19566.9	227.6955	19554.2	227.7209	19555.09	227.7008
227.9433	19563.59	227.9492	19565.35	227.9427	19566.78	227.945	19568.45	227.9497	19554.59	227.9711	19557.2	227.949
228.2018	19564.63	228.1977	19564.61	228.2059	19564.73	228.1927	19568.01	228.2017	19557.1	228.2214	19555.02	228.1973
228.4489	19567.18	228.4496	19566.53	228.4461	19566.65	228.4413	19566.79	228.4467	19554.8	228.4692	19555.14	228.4567
228.6973	19566.15	228.7003	19565.1	228.6937	19566.38	228.6949	19565.75	228.6981	19555.3	228.7166	19555.5	228.6985
228.9438	19566.3	228.9473	19565.6	228.9465	19567	228.944	19564.04	228.9502	19555.29	228.9697	19556.55	228.9506
229.2002	19565.31	229.1996	19566.23	229.1921	19566.56	229.1928	19566.59	229.1977	19555.93	229.2156	19557.15	229.1959
229.444	19563.89	229.4498	19567.18	229.4423	19566.38	229.4425	19566.45	229.4525	19552.77	229.461	19555.06	229.456
229.6963	19567.72	229.7001	19567.01	229.6958	19568.39	229.6969	19567.63	229.6982	19555.93	229.7068	19554.34	229.6998
229.9438	19567.1	229.9508	19564.96	229.9471	19565.88	229.9443	19567.34	229.9508	19553.15	229.9535	19554.62	229.9486
230.2012	19564.12	230.194	19566.36	230.1974	19567.3	230.1944	19565.43	230.1998	19552.75	230.2005	19554.83	230.1941
230.4436	19565.81	230.4504	19567.02	230.4511	19566.71	230.444	19567.91	230.4458	19556.23	230.4447	19554.21	230.4548
230.6968	19567.55	230.6963	19565.84	230.7118	19566.15	230.6961	19565.43	230.6992	19554.88	230.701	19556.05	230.6993
230.9565	19563.89	230.9513	19566.71	230.9596	19565	230.9465	19565.49	230.9466	19555.17	230.9495	19555.77	230.9523
231.208	19566.38	231.1957	19564.69	231.2062	19565.59	231.1949	19566.15	231.2027	19555.18	231.2001	19556.34	231.1972
231.4502	19565.53	231.4503	19564.66	231.457	19565.62	231.4459	19565.03	231.4563	19555.22	231.4446	19554.85	231.4559
231.6976	19567.5	231.6972	19567.89	231.7078	19565.47	231.6955	19567.76	231.7018	19554.64	231.7043	19556.88	231.7011
231.9462	19564.13	231.9496	19566.73	231.9574	19567.39	231.9441	19566.69	231.9503	19555.32	231.9551	19555.66	231.9479
232.2034	19565.72	232.2156	19567.92	232.2032	19567.43	232.1963	19566.48	232.2012	19556	232.2112	19556.4	232.1999
232.4573	19563.65	232.466	19563.62	232.4477	19565.81	232.4458	19568.6	232.4488	19555.58	232.4608	19557.74	232.451
232.7125	19565.09	232.713	19567.08	232.6964	19564.98	232.697	19566.49	232.6954	19554.36	232.7087	19557.37	232.6979
232.9677	19566.24	232.9618	19565.21	232.9451	19566.89	232.9473	19565.81	232.9519	19553.38	232.9589	19556.21	232.9527
233.2225	19565.32	233.2082	19565.67	233.1956	19567.15	233.1942	19564.72	233.2052	19553.78	233.2073	19552.91	233.1995
233.4722	19565	233.4566	19565.2	233.448	19565.55	233.4434	19568.71	233.4541	19555.34	233.4538	19557.5	233.4509
233.7188	19563.78	233.7053	19565.26	233.6962	19567.75	233.6981	19567.5	233.6995	19554.27	233.7025	19556.37	233.7014
233.9756	19566.42	233.9554	19566.23	233.9473	19566.63	233.9488	19568.89	233.948	19556.7	233.9533	19556.47	233.949
234.2341	19565.44	234.2029	19565.48	234.1951	19566.19	234.1959	19566.74	234.2047	19554.21	234.2024	19554.55	234.1978
234.7056	19565.52	234.452	19567.36	234.4465	19565.31	234.4451	19566.74	234.4582	19554.83	234.4482	19556.63	234.4602
234.957	19564.38	234.704	19566.73	234.6975	19564.94	234.6959	19567.38	234.7045	19551.84	234.7007	19554.87	234.7085
235.2157	19566.3	234.9552	19564.46	234.9473	19567.56	234.946	19566.13	234.9545	19553.58	234.9501	19556.11	234.9574
235.4648	19564.82	235.2032	19566.29	235.1974	19565.17	235.2004	19566.57	235.2067	19554.22	235.2	19556.55	235.2048
235.7113	19567.3	235.4511	19563.39	235.4456	19568.69	235.4472	19567.13	235.4569	19552.55	235.4466	19554.99	235.4573
235.9582	19564.93	235.7034	19565.58	235.6971	19569.81	235.6982	19565.9	235.6981	19555.77	235.7042	19554.51	235.7061
236.214	19563.24	235.9542	19564.4	235.9464	19564.96	235.9751	19567.96	235.953	19555.46	235.9601	19554.94	235.9549
236.4577	19565.21	236.2029	19566.54	236.2014	19566.71	236.4542	19566.25	236.2049	19553.87	236.2164	19556.69	236.2047
236.7078	19563.39	236.4527	19568.62	236.4454	19565.64	236.7048	19565.66	236.4575	19554.11	236.4663	19554.01	236.4613
236.9583	19564.97	236.6981	19566.33	236.6987	19565.22	236.954	19568.82	236.6968	19552.7	236.7189	19556.26	236.7139
237.2145	19565.04	236.9535	19564.3	236.9457	19568.82	237.2059	19566.77	236.9544	19554.35	236.9728	19552.89	236.9641
237.4578	19563.29	237.2008	19564.49	237.196	19565.15	237.454	19565.04	237.206	19555.12	237.2257	19553.72	237.2045
237.7079	19564.4	237.4529	19567.2	237.4445	19565.78	237.7055	19565.52	237.4524	19554.42	237.7085	19557.07	237.4594
237.9589	19565.2	237.7041	19565.88	237.6969	19565.43	237.9558	19566.77	237.7021	19554.68	237.9595	19556.89	237.703
238.2164	19565.84	237.953	19566.01	237.9498	19563.95	238.2033	19568.65	237.9552	19556.11	238.2118	19553.71	237.9531
238.4601	19565.28	238.2035	19567.13	238.1994	19566.36	238.4528	19565.03	238.2052	19555.15	238.4613	19555.44	238.202
238.7073	19565.46	238.4534	19565.56	238.4473	19567.48	238.7048	19566.9	238.4577	19554.34	238.7162	19557.03	238.4599
238.9597	19565.87	238.7043	19566.53	238.6978	19564.69	238.9565	19566.96	238.6994	19554.82	238.9639	19556.9	238.7034
239.2148	19564.09	238.9554	19565.58	238.9493	19567.1	239.2051	19564.44	238.9552	19556.58	239.2129	19555.07	238.9541
239.4617	19562.96	239.2018	19565.24	239.1999	19567.51	239.4534	19566.68	239.2034	19554.46	239.4575	19553.31	239.2033
239.7095	19567.82	239.4552	19564.25	239.4476	19565.64	239.7041	19565.96	239.4575	19554.78	239.7069	19553.55	239.4579
239.9574	19563.71	239.7038	19562.74	239.6984	19566.97	239.9562	19566.94	239.7005	19554.48	239.954	19556.63	239.701
240.2146	19565.41	239.9557	19563.79	239.9527	19565.98	240.2045	19568.52	239.9566	19553.4	240.2098	19556.17	239.9549
240.4604	19564.97	240.2007	19565.92	240.2031	19566.53	240.4535	19566.77	240.2044	19554.06	240.4516	19554.99	240.2031
240.7047	19566.22	240.455	19564.98	240.4491	19565.43	240.7001	19569.23	240.4577	19554.06	240.7044	19556.45	240.4601
240.9547	19564.57	240.6973	19564.01	240.6947	19567.1	240.9486	19566.63	240.6948	19553.96	240.9646	19554.48	240.6996
241.2099	19564.94	240.9517	19564.85	240.9439	19565.74	241.2011	19566.6	240.9503	19553.85	241.2025	19553.98	240.9503
241.4548	19562.83	241.1985	19565.5	241.1936	19566.77	241.4494	19566.93	241.2121	19552.91	241.4597	19556.85	241.1987
241.7038	19563.61	241.4516	19566.21	241.4436	19565.03	241.7022	19565.86	241.4531	19555.72	241.7096	19554.33	241.4667
241.9558	19565.53	241.7123	19566.45	241.6961	19568.5	241.9505	19567.95	241.7018	19555.67	241.9563	19556.56	241.711
242.2101	19565.89	241.9498	19563.17	241.9433	19568.4	242.1997	19567.26	241.9538	19554.61	242.2089	19555.13	241.9507
242.4564	19565.83	242.2068	19566.43	242.1977	19564.31	242.4475	19564.26	242.2042	19553.91	242.4555	19556.04	242.1987
242.7058	19566.6	242.4597	19564.35	242.4451	19564.43	242.701	19566.19	242.455	19555.65	242.7052	19554.44	242.4591
242.956	19565.16	242.7102	19565.47	242.6963	19567.36	242.9489	19568.04	242.6988	19555.43	242.9569	19557.19	242.701
243.2116	19565.48	242.9569	19566.53	242.9446	19563.89	243.1999	19568.34	242.9519	19555.34	243.2071	19554.07	242.9524
243.458	19564.51	243.2103	19565.03	243.1963	19566.88	243.4485	19564.45	243.2039	19554.82	243.4565	19554.15	243.2011
243.7057	19565.68	243.4547	19564.56	243.444	19564.62	243.703	19567.87	243.4537	19553.2	243.7095	19553.75	243.4613
243.9572	19566.28	243.7086	19565.46	243.6948	19565.6	243.9489	19567.53	243.6999	19552.71	243.9567	19553.44	243.7008

244.2102	19567.49	243.9631	19565.08	243.9442	19569	244.2002	19567.28	243.9513	19554.29	244.2118	19554.25	243.9516
244.457	19564.29	244.1989	19565.29	244.1967	19567.08	244.4502	19567.91	244.2039	19557.11	244.4564	19555.44	244.2012
244.7076	19563.4	244.4556	19564.84	244.4445	19567.53	244.7017	19565.98	244.4545	19552.51	244.7087	19557.01	244.4561
244.9557	19563.83	244.7204	19567.65	244.6957	19565.97	244.9504	19566.16	244.7006	19554.21	244.9619	19557.06	244.7103
245.2127	19564.38	244.9625	19564.78	244.9444	19565.98	245.2002	19567.22	244.9533	19553.82	245.2082	19556.99	244.9534
245.4576	19562.66	245.1986	19566.42	245.1972	19565.86	245.4493	19567.34	245.2035	19554.72	245.4535	19557.05	245.2007
245.7101	19563.71	245.4531	19566.96	245.4445	19565.64	245.7003	19567.16	245.4532	19553.7	245.7064	19556.2	245.4564
245.9573	19565.2	245.707	19563.66	245.6999	19567.82	245.9516	19567.51	245.7055	19552.17	245.9573	19553.87	245.7012
246.2106	19565.21	245.9558	19565.55	245.9436	19565.93	246.2018	19566.03	245.9537	19552.17	246.2103	19556.78	245.9544
246.4572	19564.74	246.2032	19565.23	246.1959	19564.84	246.4519	19564.16	246.2052	19554.51	246.4528	19555.33	246.2027
246.7064	19567.34	246.451	19564.84	246.4458	19563.47	246.7017	19567.03	246.4557	19555	246.7107	19556.89	246.4594
246.9598	19563.21	246.7083	19564.53	246.7003	19567.49	246.9529	19564.58	246.7068	19553.82	246.9559	19555.41	246.7019
247.213	19564.77	246.9552	19565.24	246.9457	19566.02	247.2011	19564.87	246.9541	19553.59	247.2083	19556.01	246.9523
247.4594	19566.44	247.2	19565.04	247.1972	19565.35	247.4509	19565.65	247.2071	19553.97	247.4568	19554.97	247.202
247.7067	19565.07	247.4549	19567.65	247.4449	19568.58	247.7029	19568.51	247.4491	19554.71	247.7072	19557.65	247.4573
247.9588	19565.43	247.7058	19563.68	247.6985	19564.45	247.954	19566.21	247.7026	19553.41	247.9566	19556.53	247.7027
248.2128	19563.9	247.9537	19565.21	247.9442	19566.38	248.201	19568.12	247.9551	19555.91	248.2073	19556.95	247.9529
248.4634	19565.11	248.2001	19565.05	248.1974	19566.82	248.4509	19568.15	248.202	19555.27	248.4584	19555.84	248.2014
248.7089	19565.32	248.4577	19565.83	248.4449	19564.65	248.7031	19566.72	248.4564	19554.48	248.7102	19555.62	248.4582
248.9574	19562.96	248.7067	19565.17	248.6984	19566.84	248.9543	19566.37	248.7	19554.54	248.958	19553.68	248.7021
249.2132	19566.45	248.9559	19567.5	248.9454	19567.65	249.203	19565.58	248.9566	19555.07	249.2111	19555.68	248.9552
249.4608	19563.39	249.2025	19564.2	249.1974	19564.42	249.4517	19565.86	249.2062	19554.68	249.4574	19555.89	249.1997
249.7092	19565.55	249.4522	19567.28	249.4452	19565.63	249.7037	19567.18	249.4547	19554.78	249.7095	19557.49	249.4608
249.9585	19565.41	249.708	19563.08	249.6971	19566.21	249.9663	19564.81	249.6998	19554.92	249.9579	19555.7	249.7129
250.2136	19565.04	249.9579	19563.05	249.9447	19567.99	250.2171	19564.85	249.9543	19556.56	250.209	19554.06	249.9603
250.4597	19564.12	250.2056	19564.33	250.2084	19565.95	250.4623	19566.39	250.2028	19554.96	250.4523	19556.22	250.2054
250.7193	19562.51	250.457	19563.72	250.4525	19564.47	250.7101	19566.35	250.4525	19554.69	250.7093	19553.07	250.4543
250.9678	19565.49	250.7079	19565.04	250.6985	19565.67	250.9588	19565.83	250.6975	19551.83	250.9581	19555.38	250.7034
251.218	19567.01	250.9609	19564.4	250.9465	19565.76	251.2075	19568.34	250.9571	19552.95	251.2166	19555.05	250.9513
251.4622	19562.5	251.2099	19563.22	251.202	19564.59	251.4555	19564.37	251.2061	19553.85	251.4733	19556.11	251.2036
251.7118	19564	251.4618	19565.55	251.445	19566.22	251.7023	19567.28	251.4567	19553.96	251.7211	19555.63	251.4526
251.9592	19563.87	251.7137	19564.62	251.6991	19566.39	251.9529	19567.44	251.703	19556.4	251.9716	19557.39	251.6988
252.2137	19564.71	251.9663	19564.38	251.9483	19565.35	252.2042	19567.58	251.9532	19553.04	252.2233	19552.38	251.9565
252.4651	19564.55	252.2158	19566.04	252.206	19566.02	252.452	19566.07	252.2045	19553.65	252.4722	19554.86	252.202
252.712	19563.62	252.4699	19563.76	252.452	19563.99	252.7075	19566.54	252.4536	19554.74	252.7246	19553.51	252.458
252.9609	19568.75	252.7217	19563.86	252.6981	19564.73	252.9566	19567.68	252.7004	19552.12	252.9759	19555.37	252.7047
253.2154	19564.25	252.9767	19565.3	252.9467	19567.87	253.2045	19567.49	252.9555	19552.78	253.2277	19554.92	252.9519
253.4642	19563.87	253.4638	19563.23	253.1997	19564.52	253.453	19565.95	253.2064	19555.7	253.7139	19555.1	253.2035
253.7118	19563.13	253.7175	19564.95	253.4478	19565.13	253.704	19565.22	253.4572	19555.77	253.9642	19555.98	253.4563
253.9609	19566.98	253.9712	19564.58	253.7002	19564.84	253.957	19565.74	253.7151	19554.38	254.2131	19553.07	253.7039
254.2164	19565.15	254.2209	19563.88	253.947	19565.49	254.2059	19567.98	253.9711	19553.38	254.4613	19554.08	253.9523
254.4626	19565.06	254.4757	19564.37	254.2004	19567.87	254.4538	19564.73	254.2307	19554.17	254.7128	19555.35	254.1989
254.7127	19562.89	254.7288	19566.42	254.4489	19565.6	254.7042	19565.18	254.7166	19554.08	254.9615	19556.31	254.4649
254.9637	19566.44	254.9813	19565.78	254.7	19567.15	254.9552	19566.94	254.9682	19553.57	255.2113	19556.27	254.7231
255.2216	19563.78	255.4697	19564.41	254.948	19565.4	255.2115	19565.9	255.2207	19554.87	255.4592	19554.45	255.2126
255.4762	19562.07	255.7227	19565.33	255.2035	19568.25	255.4579	19565.34	255.4725	19552.24	255.7129	19555.43	255.4661
255.7265	19564.74	255.9782	19564.96	255.4529	19565.65	255.7084	19565.3	255.7256	19554.46	255.9624	19556.2	255.7132
255.9898	19562.64	256.2338	19564.4	255.7024	19565.75	255.9583	19565.2	255.9787	19557.27	256.213	19554.52	255.9654
256.4739	19564.35	256.4865	19565.46	255.9495	19567.91	256.2107	19567.09	256.2306	19554.57	256.4618	19556.58	256.2146
256.7256	19563.4	256.977	19565.52	256.2078	19564.79	256.4605	19564.19	256.4829	19556.69	256.719	19554.9	256.4667
256.9755	19563.23	257.2292	19566.03	256.4552	19566.85	256.7143	19566.62	256.7312	19554.6	256.9705	19555.78	256.716
257.2304	19566.78	257.4826	19564.24	256.7063	19564.36	256.9619	19566.79	256.9847	19553.9	257.2217	19554.59	256.975
257.4808	19564.69	257.7459	19565.09	256.9542	19566.87	257.2116	19568.23	257.4723	19553.99	257.4695	19555.89	257.2249
257.7293	19565.96	258.2347	19563.66	257.2145	19565.68	257.4593	19566.79	257.7243	19552.92	257.7207	19555.7	257.4815
257.9823	19564.65	258.4894	19566.12	257.4631	19564.68	257.7085	19568.61	257.9751	19555	257.9712	19555.37	257.9694
258.2451	19566.35	258.744	19565.14	257.7119	19565.58	257.957	19565.83	258.2298	19553.95	258.225	19555.12	258.2186
258.7279	19565.33	259.0058	19566.29	257.9628	19566.62	258.2083	19565.84	258.4836	19552.95	258.4754	19554.35	258.4749
258.9801	19562.66	259.498	19565.22	258.2254	19564.68	258.4564	19563.98	258.735	19552.47	258.7304	19554.25	258.7239
259.237	19564.44	259.7528	19567.52	258.7076	19566.21	258.71	19567.21	258.9893	19556.6	259.2227	19556.73	258.9757
259.4887	19566.86	260.0086	19566.85	258.9554	19565.42	258.9606	19566.65	259.4848	19554.53	259.4728	19553.55	259.2258
259.7384	19568.16	260.2636	19564.58	259.2208	19564.72	259.2142	19565.16	259.7378	19554.6	259.7233	19555.9	259.4862
259.9898	19561.71	260.7567	19565.65	259.4721	19564.23	259.4642	19566.5	259.9919	19554.05	259.9739	19557.44	259.9763
260.2455	19563.8	261.0163	19563.65	259.7297	19564.65	259.7181	19564.72	260.2464	19554.46	260.2314	19553.85	260.2273
260.4977	19564.67	261.274	19564.74	260.2119	19565.14	259.97	19563.65	260.7383	19554.56	260.4837	19551.41	260.483
260.7567	19564.37	261.7662	19564.38	260.4612	19565.17	260.2251	19565.18	260.9925	19552.21	260.9713	19555.11	260.7339
261.2457	19565.37	262.0222	19566.56	260.7131	19566.44	260.4764	19565.52	261.2481	19555.75	261.2262	19554.55	260.9889
261.5061	19563.49	262.2793	19564.26	260.9651	19565.68	260.7286	19569.29	261.5027	19552.74	261.4776	19552.02	261.2414
261.7571	19562.5	262.7728	19563.83	261.2196	19565.44	261.2121	19568.88	261.7571	19554.23	261.7321	19555.98	261.7306
262.0141	19563.31	263.0301	19562.93	261.4703	19566.17	261.465	19564.36	262.2488	19555.54	261.9876	19552.68	261.9848
262.4969	19565.45	263.2869	19565.23	261.7237	19566.36	261.715	19565.62	262.5069	19554.49	262.4762	19555.01	262.2363

262.7491	19565.31	263.7777	19565.88	261.9777	19565.64	261.9692	19563.26	262.7611	19552.35	262.7306	19556.04	262.4938
263.0042	19564.09	264.0354	19565.43	262.2384	19565.63	262.2278	19566.56	263.2517	19553.63	262.9845	19555.5	262.7483
263.2594	19563.32	264.2907	19566.67	262.7229	19564.07	262.4806	19566.08	263.5087	19553.03	263.2386	19555.4	263.2361
263.5157	19564.4	264.7943	19565.7	262.9735	19565.12	262.7318	19565.47	263.7634	19555.26	263.4921	19554.55	263.4939
263.7673	19564.15	265.2853	19566.86	263.2293	19565.32	263.2159	19566	264.0211	19551.68	263.9818	19555.31	263.7477
264.2572	19562.43	265.5434	19564.29	263.4807	19564.35	263.4657	19568.26	264.515	19553.08	264.2399	19552.92	264.004
264.5152	19564.26	265.8107	19564.53	263.7351	19567.07	263.7257	19566.43	264.7695	19552.54	264.4917	19555.44	264.5031
264.77	19566.6	266.3026	19565.84	263.9909	19564.96	263.9839	19568.84	265.0258	19554.57	264.7468	19552.36	264.7585
265.0294	19565.57	266.5642	19564.92	264.2474	19566.22	264.2381	19568.88	265.5191	19553.52	265.2432	19554.81	265.2473
265.5168	19564.8	267.0613	19565.2	264.7313	19565.13	264.7216	19566.67	265.778	19554.26	265.4961	19557.16	265.5075
265.7731	19565.38	267.3181	19565.1	264.9855	19564.05	264.9832	19565.96	266.0336	19553.84	265.7541	19554.62	265.7606
266.0282	19562.98	267.8106	19564.36	265.2452	19565.2	265.2374	19565.22	266.5261	19553.55	266.2474	19555.09	266.0197
266.2951	19565.79	268.0692	19564	265.4975	19564.51	265.4914	19566.41	266.7833	19552.72	266.5023	19554.57	266.5163
266.7844	19566.17	268.3261	19563.51	265.7512	19564.97	265.9787	19566.65	267.0418	19554.77	266.7617	19556.19	266.7704
267.0482	19563.14	268.8181	19565.1	266.2352	19564.35	266.2356	19566.16	267.5346	19554.98	267.256	19555.08	267.0279
267.5402	19565.1	269.0765	19563.94	266.4884	19565.49	266.4936	19568.22	267.7913	19552.84	267.5132	19555.56	267.523
267.8002	19563.77	269.3363	19563.31	266.7435	19566.81	266.7493	19564.91	268.0508	19554.7	267.7747	19556.72	267.7797
268.0632	19562.73	269.8303	19564.47	266.9989	19564.76	267.2359	19564.22	268.5446	19549.71	268.2662	19555.6	268.0426
268.5544	19563.64	270.09	19565.09	267.2535	19564.57	267.4918	19566.44	268.8012	19552.88	268.521	19554.98	268.5397
268.8139	19564.9	270.5925	19564.82	267.51	19566.54	267.7453	19568.79	269.0623	19553.24	268.7824	19553.86	268.7946
269.0718	19564.18	270.8539	19563.66	268.0001	19563.6	268.0078	19566	269.5597	19550.97	269.2771	19554.16	269.0533
269.5706	19565.06	271.3488	19564.05	268.2558	19565.81	268.4958	19565.25	269.8179	19553.53	269.5322	19555.42	269.5499
269.8287	19562.4	271.613	19566.87	268.5125	19564.95	268.7503	19566.26	270.3105	19554.17	269.7943	19555.33	269.8063
270.3214	19566.21	272.1087	19565.43	268.7696	19564.51	269.0102	19565.56	270.5717	19554.22	270.2869	19554.75	270.3033
270.5827	19566.55	272.3686	19562.13	269.2551	19565.67	269.267	19565.87	271.0686	19555.48	270.5482	19555.24	270.5642
271.077	19563.9	272.8724	19563.04	269.5126	19563.18	269.7519	19565.21	271.3379	19552.72	271.0401	19556.46	271.0607
271.341	19564.16	273.3676	19564.25	269.769	19566.3	270.0129	19566.74	271.831	19554.5	271.3007	19551.69	271.3181
271.8314	19563.15	273.6319	19562.41	270.0292	19564.77	270.2699	19563.65	272.0915	19555.04	271.5614	19552.21	271.8113
272.0909	19563.02	274.1262	19564.66	270.517	19565.23	270.7596	19564.81	272.5916	19552.22	272.0537	19556.64	272.0719
272.3537	19564.42	274.3874	19565.22	270.7745	19565.78	271.0197	19566.6	272.8504	19554.76	272.3149	19554.37	272.5754
272.8407	19564.04	274.8823	19563.9	271.0368	19564.1	271.2773	19567.57	273.3424	19554.06	272.813	19553.77	273.0707
273.1007	19562.8	275.145	19566.43	271.5255	19566.31	271.7658	19566.53	273.6018	19552.86	273.0731	19554.5	273.3285
273.3619	19565.38	275.6432	19563.38	271.7826	19565.94	272.0273	19569.85	273.8615	19555.78	273.566	19554.73	273.5914
273.8495	19565.65	275.9112	19564.23	272.0445	19568.17	272.2853	19566.7	274.3531	19555.57	273.8303	19555.13	274.0869
274.1123	19565.09	276.4067	19565.28	272.5336	19566.61	272.7718	19565.26	274.6168	19551.91	274.3245	19555.08	274.3455
274.3717	19563.66	276.6704	19562.87	272.7899	19564.16	273.0373	19565.46	275.1142	19554.42	274.5833	19552.44	274.8384
274.8649	19562.22	277.1635	19565.06	273.0536	19566.46	273.2959	19566.01	275.3731	19555.34	275.0769	19555.82	275.1018
275.1275	19565.54	277.426	19563.55	273.5428	19564.93	273.7815	19565.16	275.8682	19554.86	275.3381	19555.56	275.6004
275.6208	19563.46	277.9244	19563.95	273.8005	19566.26	274.0453	19566.66	276.1329	19554.05	275.8373	19557.12	275.8604
275.8873	19566.74	278.1881	19564.57	274.0617	19564.34	274.3043	19564.99	276.6288	19554.6	276.0986	19554.97	276.3603
276.377	19565.01	278.6893	19563.82	274.5522	19566.32	274.7953	19562.71	276.8921	19554.13	276.5924	19553.76	276.6249
276.6379	19564.18	278.9525	19565.17	274.8143	19565.05	275.0597	19566.71	277.3903	19552.92	276.8566	19556.6	277.1228
276.8991	19564.22	279.454	19564.76	275.0802	19564.76	275.5512	19567.03	277.6547	19555.74	277.3529	19556.48	277.3862
277.3895	19565.95	279.7229	19564.52	275.5713	19565.2	275.811	19566.37	278.1704	19553.97	277.6151	19554.45	277.8821
277.6501	19565.02	280.2181	19564.74	275.8302	19562.97	276.0763	19566.39	278.6666	19550.51	278.11	19554.75	278.1487
277.9168	19562.69	280.4944	19565.03	276.0966	19565.37	276.5682	19565.78	278.9312	19551.89	278.3743	19553.85	278.6473
278.4083	19565.31	280.9973	19564.05	276.5869	19564.54	276.8292	19568.13	279.437	19554.05	278.8729	19556.63	278.9093
278.677	19566.71	281.2628	19564.64	276.8469	19564.75	277.0941	19564.05	279.9347	19553.22	279.1357	19554.1	279.4084
279.171	19565	281.7635	19566.99	277.117	19562.83	277.5856	19565.2	280.199	19552.71	279.6314	19555	279.6731
279.4324	19562.73	282.0318	19565.61	277.6083	19565.89	277.8457	19567.96	280.6929	19553.1	279.8958	19555.38	280.1711
279.9226	19565.6	282.5274	19563.91	277.8688	19565.63	278.1106	19565.33	280.9571	19554.78	280.3923	19555.39	280.436
280.188	19564.7	282.7934	19564.16	278.1361	19565.87	278.6041	19565.67	281.4545	19553.7	280.6539	19556.12	280.9335
280.6782	19563.28	283.2952	19564.39	278.6283	19565.36	278.8647	19567.07	281.7182	19555.77	281.15	19555.39	281.1954
280.9388	19567.48	283.5603	19565.45	278.8886	19565.67	279.3542	19565.85	282.2203	19555.97	281.4132	19553.56	281.6915
281.2057	19564.36	284.065	19563.15	279.1581	19565.53	279.6181	19567.29	282.4878	19553.38	281.9136	19555.04	281.9531
281.6955	19564.84	284.3321	19564.87	279.6493	19564.79	280.1172	19564.38	282.9875	19554.74	282.1796	19553.64	282.4563
281.9575	19564.75	284.8364	19565.21	279.9091	19566.04	280.3837	19567.9	283.2548	19556.2	282.6823	19556.4	282.9547
282.2253	19561.59	285.3385	19563.83	280.1748	19564.63	280.8749	19565.34	283.7514	19553.34	282.9488	19554.16	283.2187
282.7188	19563.26	285.6013	19564	280.6675	19565.17	281.1412	19563.45	284.0171	19552.81	283.4438	19552.7	283.7151
282.9809	19564.48	286.1035	19566.78	280.9291	19565.89	281.6353	19565.47	284.5161	19552.95	283.7104	19556.05	283.9794
283.4842	19562.19	286.3598	19565.71	281.42	19567.5	281.8967	19566.1	284.7793	19552.56	284.2109	19551.58	284.4819
283.9784	19563.11	286.8715	19567.78	281.6847	19566.8	282.3883	19564.65	285.28	19552.93	284.4752	19555.67	284.7458
284.2458	19564.06	287.3765	19565.09	282.1825	19564.56	282.6528	19565.78	285.545	19552.97	284.9747	19556.14	285.2445
284.7392	19562.79	287.6352	19563.16	282.4449	19564.24	283.1482	19565.32	286.0447	19552.94	285.2403	19554.49	285.5124
285.0037	19565.82	288.1353	19563.72	282.9352	19565.23	283.4091	19566.47	286.3009	19554.94	285.7452	19554.71	286.0136
285.497	19565.71	288.3934	19566.17	283.2201	19564.22	283.9021	19566.38	286.7976	19551.97	286.2496	19553.48	286.2788
285.7619	19563.93	288.8945	19564.27	283.7134	19564.46	284.1699	19563.47	287.0544	19554.03	286.7494	19554.46	286.7755
286.2585	19562.63	289.15	19563.83	283.9663	19566.08	284.6631	19564.43	287.5548	19553.11	287.0154	19554.9	287.0425
286.5125	19563.52	289.6575	19563.55	284.2206	19566.91	284.9262	19566.95	287.8109	19553.76	287.5414	19557.45	287.5451
286.7681	19563.12	290.1574	19563.21	284.4716	19565.95	285.4185	19567.07	288.3111	19552.02	288.0391	19555.1	287.8005

287.2636	19561.64	290.4146	19566.08	284.7257	19565.49	285.683	19566.59	288.5672	19552.1	288.3136	19552.53	288.3002
287.5175	19563.88	290.9254	19565.38	285.2193	19563.77	286.1794	19566.28	289.0682	19553.33	288.819	19554.06	288.5618
287.7738	19566.11	291.4265	19562.82	285.4733	19565.53	286.4439	19567.04	289.3283	19555.15	289.3216	19554.25	289.0641
288.279	19562.94	291.6883	19563.03	285.727	19564.83	286.9368	19564.92	289.8269	19552.82	289.5755	19554.07	289.3206
288.5436	19563.62	292.1929	19564.55	285.9853	19565.29	287.2056	19566.15	290.0855	19552.14	290.0732	19554.14	289.8185
289.042	19564.23	292.6988	19564.36	286.4768	19564.19	287.6988	19567.52	290.5878	19551.97	290.3329	19551.99	290.0765
289.3013	19563.87	293.1998	19565.42	286.7319	19564.84	287.9644	19565.41	290.8471	19555.34	290.8363	19556.79	290.581
289.7942	19563.69	293.4613	19564	286.985	19565.83	288.4586	19565.73	291.3471	19553.49	291.3471	19553.08	291.0831
290.0527	19563.08	293.9839	19565.63	287.2434	19564.21	288.7127	19566.28	291.6042	19551.84	291.8506	19553.58	291.3407
290.3125	19565.74	294.4903	19564.74	287.7392	19564.66	288.9677	19565.2	292.106	19554.16	292.3542	19553.38	291.8396
290.8061	19564.33	294.9931	19563.05	287.9955	19565.49	289.4641	19566.32	292.3641	19555.02	292.8578	19551.79	292.0979
291.0787	19564.28	295.2563	19564.21	288.4895	19562.81	289.7205	19565.67	292.8651	19554.93	293.3588	19555.96	292.6037
291.5765	19564.11	295.7628	19565.28	288.7463	19565.22	289.9768	19566.21	293.1252	19554.65	293.6209	19553.07	293.108
291.8324	19563.13	296.2651	19564.02	289.003	19563.43	290.4726	19563.46	293.6265	19552.89	294.1242	19552.31	293.6128
292.3318	19564.19	296.5249	19565.43	289.4963	19564.71	290.727	19565.07	293.8846	19555.76	294.3833	19553.37	294.1161
292.5974	19564.15	297.0289	19561.99	289.7564	19564.54	290.9814	19565.25	294.3887	19555.13	294.8887	19556.94	294.6218
293.0954	19566.32	297.5328	19565.6	290.0129	19564.6	291.4785	19565.38	294.8892	19553.42	295.3898	19553.46	295.1248
293.3561	19563.81	298.0351	19564.96	290.5094	19566.27	291.7334	19565.93	295.1537	19551.88	295.8988	19555.72	295.6341
293.8518	19562.72	298.3066	19565.59	290.7787	19564.57	291.9907	19566.99	295.6599	19555.81	296.4004	19554.47	296.1387
294.111	19563.49	298.8178	19564.18	291.294	19566.53	292.4902	19565.61	295.9192	19551.64	296.6607	19556.2	296.6445
294.6079	19563.83	299.3275	19564.09	291.7903	19565.39	292.7449	19566.57	296.4214	19551.51	297.1637	19555.69	297.1488
294.8646	19563.28	299.8488	19565.7	292.0514	19566.07	293.2425	19564.54	296.6803	19556.05	297.4228	19554.63	297.6534
295.3667	19564.25	300.3584	19564.42	292.5487	19565.48	293.4999	19564.85	297.1871	19553.78	297.9267	19554.59	298.1571
295.6323	19564.38			292.8053	19563.96	293.9965	19564.8	297.6917	19554.09	298.4292	19550.36	298.6622
296.1322	19566.74			293.313	19564.64	294.2539	19566.67	298.1971	19553.86	298.6965	19553.99	299.1665
296.391	19563.06			293.8084	19564.52	294.7504	19565.6	298.6978	19553.51	299.1997	19553.18	299.6724
296.8858	19564.58			294.0692	19565.33	295.0109	19566.12	298.9699	19554.71	299.7017	19553.46	300.1757
297.1469	19562.76			294.5672	19564.83	295.509	19563.97	299.4762	19552.1	299.9643	19553.94	
297.6475	19563.61			294.8242	19566.48	295.7685	19565.27	299.9863	19553.2	300.4691	19553.85	
297.9077	19565.21			295.3179	19565.4	296.2846	19565.31	300.4906	19552.08			
298.4058	19563.9			295.5808	19564.5	296.78	19564.72					
298.6672	19563.65			296.0821	19565.08	297.0432	19566.84					
299.168	19564.73			296.3514	19564.9	297.5441	19565.11					
299.4305	19563.86			296.8477	19565.26	297.8019	19565.4					
299.9314	19565.09			297.1294	19565.93	298.298	19566.88					
300.198	19562.21			297.6292	19564.33	298.561	19566.39					
				297.8999	19565.63	299.0645	19566.16					
				298.4031	19563.57	299.5667	19564.47					
				298.8986	19565.09	299.825	19563.89					
				299.1734	19564.43	300.322	19564.48					
				299.6732	19565.99							
				299.9422	19563.98							
				300.4393	19564.68							

43					1.43							
5					50							
x_3	x_4		x_5		x_1		x_2		x_3		x_4	
P (cm^{-1})	t (s)	P (cm^{-1})	t (s)	P (cm^{-1})	t (s)	P (cm^{-1})	t (s)	P (cm^{-1})	t (s)	P (cm^{-1})	t (s)	P (cm^{-1})
19566.83	0	19570.97	0	19569.36	0	19561.17	0	19560.47	0	19562.07	0	19561.89
19571.22	0.68054	19569.09	0.73334	19571.8	0.67116	19559.07	0.66355	19560.97	0.66148	19561.36	0.66087	19560.42
19570.03	0.84928	19569.94	0.89218	19569.61	0.857	19560.06	0.85111	19560.14	0.85302	19562.15	0.84903	19562.27
19571.55	1.10017	19571.98	1.10132	19569.93	1.10373	19560.12	1.1005	19562.01	1.10669	19562.71	1.10515	19561.63
19570.14	1.34719	19569.25	1.34831	19570.28	1.35302	19560.56	1.34954	19561.1	1.35283	19560.32	1.34687	19562.24
19570.51	1.59985	19572.23	1.60447	19573.24	1.60326	19559.66	1.59981	19560.63	1.60561	19562	1.5991	19563.21
19571.31	1.84778	19569.55	1.84852	19570.98	1.85334	19560.05	1.84715	19559.8	1.85097	19561.54	1.84695	19561.8
19571.93	2.09806	19572.76	2.09865	19569.81	2.1017	19561.33	2.09883	19560.06	2.105	19561.23	2.10732	19561.85
19570.92	2.34671	19571.2	2.3482	19570.28	2.35317	19558.86	2.34902	19561.93	2.3517	19560.22	2.35194	19561.76
19571.35	2.60001	19571.14	2.60253	19573.96	2.60293	19561.42	2.60907	19560.55	2.60412	19559.34	2.59851	19561.92
19570.07	2.84794	19572.01	2.84914	19569.74	2.85286	19559.91	2.84659	19561.71	2.85275	19560.62	2.85162	19562.46
19570.88	3.09824	19570.98	3.09895	19570.67	3.1026	19559.94	3.10257	19560.5	3.10358	19563.79	3.1022	19563.08
19571.08	3.34689	19569.47	3.34893	19571.88	3.35418	19560.83	3.34841	19560.12	3.35221	19560.13	3.34758	19562.24
19571.04	3.59859	19571.82	3.60682	19571.57	3.60386	19559.64	3.60036	19559.74	3.60323	19560.23	3.59901	19560.31
19568.3	3.84877	19571.93	3.84727	19571.22	3.85209	19561.41	3.84816	19561.11	3.85253	19559.35	3.84637	19561.08
19570.65	4.09995	19570.17	4.09842	19572.15	4.10198	19561.4	4.1008	19559.34	4.10499	19560.99	4.10241	19561.66
19572.87	4.34746	19572.47	4.34938	19570.65	4.35276	19562.56	4.34853	19563.28	4.35313	19561.51	4.35021	19562.26
19570.32	4.5981	19570.08	4.60623	19572.87	4.60858	19561.74	4.60343	19561.37	4.60494	19561.48	4.60458	19558.95
19572.21	4.85276	19570.26	4.84798	19570.87	4.85583	19560.25	4.84915	19560.72	4.85225	19559.65	4.8483	19561.98
19572.11	5.10326	19570.62	5.10345	19571.25	5.10962	19559.16	5.10105	19562.9	5.1089	19561.11	5.10278	19561.85
19570.25	5.34832	19568.56	5.35213	19571.31	5.35671	19561.99	5.3495	19560.3	5.35264	19561.65	5.34646	19563.13
19573.09	5.59918	19571.96	5.60595	19570.58	5.60316	19561.03	5.60143	19562.05	5.60834	19563.42	5.59804	19562.45
19568.2	5.84804	19572.06	5.84988	19572.57	5.85235	19559.36	5.84753	19560.54	5.85371	19560.27	5.84746	19561.29
19569.69	6.09943	19573.13	6.09827	19572.01	6.10292	19559.88	6.10013	19559.34	6.10581	19559.31	6.10204	19561.23
19568.99	6.34932	19568.29	6.34835	19569.32	6.35546	19561.95	6.34845	19562.78	6.35381	19558.81	6.3476	19562.51
19569.92	6.60148	19570.7	6.60485	19573.08	6.60609	19560.02	6.60155	19563.48	6.60625	19560.94	6.60051	19562.24
19573.89	6.85007	19570.38	6.85047	19572.86	6.85485	19561.62	6.84935	19561.1	6.8542	19561.82	6.84908	19562.16
19571.68	7.09906	19570.39	7.09894	19572.89	7.1028	19559.78	7.10131	19561.4	7.10515	19558.78	7.10243	19561.58
19568.74	7.34803	19570.48	7.34904	19571.12	7.35368	19560.9	7.34825	19560.5	7.35298	19562.69	7.34821	19563.65
19570.33	7.6006	19569.54	7.60461	19571.52	7.60602	19560.17	7.60083	19560.86	7.60534	19562.94	7.59842	19562.35
19570.82	7.8504	19572.78	7.84977	19573.7	7.85468	19559.94	7.84977	19562.36	7.85404	19562.74	7.84853	19560.62
19570.56	8.0993	19572.57	8.10009	19571.26	8.10483	19562.37	8.10259	19559.14	8.10729	19560.91	8.10777	19561.84
19570.95	8.34879	19573.1	8.35116	19571.77	8.35465	19558.94	8.34929	19561.35	8.35332	19560.43	8.34903	19562.28
19570.77	8.60043	19572.25	8.6044	19570.85	8.60525	19558.29	8.60108	19560.22	8.60529	19559.55	8.60081	19564.25
19570.96	8.85038	19569.03	8.84915	19570.71	8.85491	19560.01	8.84954	19561.63	8.85364	19559.57	8.84902	19560.47
19572.98	9.09986	19571.18	9.09969	19571.69	9.10659	19560.43	9.10214	19560.43	9.11214	19561.46	9.10583	19559.73
19572.43	9.3482	19570.77	9.35065	19573.61	9.3561	19560.68	9.35039	19561.42	9.3544	19561.9	9.34961	19560
19572.36	9.59961	19571.6	9.605	19570.53	9.60553	19560.55	9.60374	19560.67	9.60609	19561.03	9.60088	19562.92
19570.96	9.85532	19569.73	9.85033	19571.15	9.85372	19562.38	9.84908	19562.97	9.85312	19562.8	9.8492	19564.13
19572.41	10.10076	19570.39	10.10027	19569.46	10.10505	19558.6	10.10138	19560.95	10.111	19562.15	10.10743	19561.46
19572.64	10.35042	19571.07	10.35151	19571.48	10.35621	19559.46	10.35063	19561.73	10.3548	19559.72	10.35016	19562.11
19570.49	10.60129	19569.66	10.6094	19569.15	10.60701	19559.37	10.60345	19562.68	10.6074	19561.64	10.60118	19562.86
19570.39	10.85024	19570.69	10.85228	19571.53	10.85468	19558.93	10.85173	19562.92	10.85384	19561.6	10.84927	19561.52
19572.64	11.10075	19568.01	11.10075	19570.08	11.10602	19560.98	11.10244	19561.69	11.1084	19562.67	11.10971	19562.65
19571.58	11.35058	19571.57	11.3506	19570.69	11.35592	19559.07	11.34989	19560.83	11.35464	19561.74	11.35141	19563.04
19570.27	11.60248	19572.25	11.60619	19570.92	11.60867	19562.38	11.60417	19560.29	11.60749	19561.43	11.60341	19561.52
19570.53	11.85248	19569.96	11.85177	19572.26	11.85596	19559.96	11.85935	19561.77	11.85553	19561.3	11.84985	19562.37
19569.88	12.10106	19571.06	12.10302	19570.94	12.10535	19560.14	12.10451	19560.91	12.10797	19561.76	12.1074	19563.92
19571.66	12.3497	19572.64	12.35215	19571.65	12.35737	19560.67	12.35081	19561.22	12.35475	19562.18	12.34992	19559.17
19569.34	12.60303	19570.39	12.60706	19571.48	12.60942	19559.74	12.60365	19562.34	12.60832	19562.33	12.6032	19561.41
19572.15	12.85174	19571.89	12.85079	19571.61	12.85604	19560.97	12.85131	19561.44	12.85523	19559.54	12.85144	19561.89
19570.06	13.1022	19569.72	13.10235	19570.46	13.10588	19560.52	13.10587	19561.42	13.10835	19561.85	13.10804	19564.22
19571.12	13.35023	19570.77	13.3526	19574.25	13.35728	19558.36	13.35124	19559.64	13.35469	19562.38	13.34983	19560.62
19570.04	13.60153	19571.14	13.60599	19568.14	13.60893	19560.45	13.60354	19560.25	13.60715	19560.45	13.60203	19561.79
19570.72	13.85124	19571.98	13.8515	19573.14	13.8557	19558.89	13.85033	19558.04	13.85577	19560.58	13.85019	19561.76
19571.99	14.10275	19570.41	14.10124	19569.16	14.10761	19559.47	14.10387	19561.45	14.10971	19560.16	14.11404	19561.15
19571.02	14.35045	19571.57	14.35328	19572.37	14.35815	19560.23	14.35293	19563.77	14.35541	19561.09	14.35056	19563.18
19571.76	14.60047	19571.67	14.60755	19572.33	14.60845	19558.25	14.60478	19561.18	14.60748	19561.03	14.60117	19561.89
19571.73	14.8515	19572.61	14.85214	19572.83	14.85619	19560.12	14.85076	19558.96	14.85468	19561.04	14.8511	19562.01
19572.53	15.10328	19570.01	15.10105	19572.26	15.10684	19561.92	15.1034	19561.53	15.10932	19561.35	15.10965	19561.18
19571.13	15.35219	19570.64	15.35256	19571.8	15.36023	19561.04	15.3539	19563.49	15.3566	19560.1	15.35259	19562.77
19572.49	15.60423	19570.53	15.60916	19572.45	15.60925	19559.41	15.60643	19560.49	15.60903	19560.3	15.6043	19562.79
19569.16	15.85253	19572.27	15.85346	19571.61	15.85623	19560.45	15.85221	19561.13	15.85678	19561.86	15.8513	19562.79
19570.84	16.10293	19569.84	16.10196	19570.27	16.10826	19561.08	16.10508	19561.02	16.10985	19559.9	16.11017	19561.94

19568.07	16.35248	19571.43	16.35256	19570.62	16.35883	19560.44	16.35303	19559.21	16.35657	19559.38	16.35278	19560.15
19571.87	16.60245	19570.18	16.60722	19569.08	16.60945	19557.93	16.60486	19560.72	16.60921	19561.06	16.60542	19560.46
19569.26	16.85491	19569.01	16.854	19569.8	16.8569	19560.01	16.85286	19560.18	16.85681	19559.07	16.85168	19562.37
19570.76	17.10211	19570.34	17.10315	19572.61	17.10785	19559.01	17.10461	19558.98	17.10999	19563.08	17.10454	19563.07
19572.08	17.35204	19568.78	17.35233	19571.36	17.35916	19558.69	17.35234	19561.24	17.3569	19561.49	17.35155	19561.52
19571.63	17.60324	19570.27	17.60887	19571.34	17.6133	19558.96	17.60494	19561.37	17.60855	19562.3	17.60417	19560.79
19569.16	17.85469	19571.17	17.85393	19571.81	17.85852	19560.23	17.85278	19558.92	17.85781	19560.56	17.85233	19561.28
19571.33	18.10402	19567.75	18.10405	19571.46	18.10758	19560.68	18.10628	19558.75	18.10994	19561.74	18.10538	19562.1
19570.53	18.35174	19571.46	18.35378	19570.92	18.35841	19560.58	18.35246	19561.35	18.357	19558.85	18.35161	19562.53
19570.93	18.60237	19571.93	18.60885	19571.37	18.61446	19561.72	18.60544	19561.29	18.60902	19560.77	18.604	19561.71
19570.5	18.85392	19571.28	18.8537	19569.76	18.86218	19562.66	18.85377	19559.57	18.85671	19559.94	18.85288	19561.66
19572.24	19.10496	19571.13	19.10494	19571.25	19.10955	19560.69	19.10686	19560.31	19.11062	19562.09	19.10379	19562.9
19570.13	19.35247	19569.04	19.3544	19571.12	19.35818	19559.28	19.35323	19559.89	19.35778	19563.26	19.35248	19562.33
19572.13	19.60376	19569.19	19.60847	19572.43	19.61108	19557.66	19.60587	19560.77	19.60903	19560.57	19.60626	19562.29
19569.34	19.8544	19572.21	19.85367	19572.08	19.85815	19558.86	19.85334	19561.22	19.8566	19561.53	19.85313	19562.64
19571.7	20.10481	19569.74	20.10326	19571.24	20.10892	19560.43	20.10584	19559.78	20.11368	19560.48	20.10742	19562.11
19570.32	20.35391	19571.72	20.35495	19572.89	20.36054	19560.96	20.35562	19559.71	20.35978	19562.24	20.35332	19561.47
19571.27	20.60339	19569.03	20.60782	19569.55	21.05404	19558.72	20.60807	19562.26	20.6112	19561.04	20.60599	19563.79
19571.43	20.85464	19571.21	20.85396	19571.28	21.4213	19557.98	20.85421	19561.54	20.85793	19562.9	20.85395	19563.42
19572.69	21.10424	19572.5	21.10364	19573.58	21.66629	19561.12	21.10716	19562.26	21.11172	19562.37	21.10849	19561.3
19570.52	21.35474	19568.58	21.355	19570.06	21.91617	19561.56	21.35505	19560.77	21.35955	19562.17	21.35404	19561.57
19569.85	21.60633	19570.19	21.61063	19570.69	22.1659	19559.89	21.61134	19560.92	21.61093	19560.71	21.60604	19561.66
19571.7	21.85472	19571.91	21.85651	19571.21	22.43186	19561.2	21.85511	19561.85	21.85973	19560.11	21.85463	19562.36
19570.95	22.10525	19569.34	22.10452	19570.58	22.67	19559.9	22.10856	19559.96	22.11313	19562.95	22.11113	19562.96
19569.59	22.35434	19570.98	22.35545	19569.53	22.91698	19560.74	22.35427	19561.18	22.35861	19561.15	22.35508	19561.81
19571.56	22.60692	19570.77	22.61109	19572.14	23.16661	19560.08	22.60913	19561.58	22.61663	19561.64	22.61088	19561.41
19571.65	22.85614	19571.68	22.85712	19569.83	23.41695	19560.77	22.85625	19560.63	22.8588	19561.02	22.85424	19562.73
19569.52	23.10552	19571.29	23.10649	19572.86	23.66567	19561.03	23.10902	19563.43	23.11389	19560.45	23.10893	19561.3
19570.25	23.35461	19571.43	23.35669	19572.03	23.91668	19560.06	23.35532	19561.31	23.36106	19560.61	23.35562	19561.48
19571.23	23.60523	19571.98	23.61019	19572.28	24.16728	19559.82	23.60854	19560.43	23.61165	19561.52	23.6075	19562.39
19570.27	23.85706	19570.73	23.85697	19572.11	24.41819	19558.7	23.85684	19561.35	23.8593	19560.03	23.85563	19561.92
19570.82	24.10699	19567.69	24.10795	19567.45	24.66628	19559.26	24.10906	19563.31	24.11302	19562.76	24.10797	19561.93
19571.77	24.35438	19572.87	24.35713	19572.89	24.91548	19560.49	24.35673	19560.26	24.36075	19562.89	24.35534	19562.18
19572.89	24.60763	19571.12	24.61192	19572.09	25.1662	19560.04	24.60859	19559.04	24.61226	19561.45	24.60705	19561.03
19571.41	24.85717	19570.21	24.85623	19572.37	25.41667	19559.77	24.85707	19558.72	24.8584	19559.2	24.85618	19563.18
19572.39	25.1067	19570.07	25.10633	19570.04	25.66785	19558.31	25.10806	19560.59	25.11222	19560.82	25.11031	19560.23
19573.34	25.35408	19571.82	25.35696	19571.07	25.91656	19559.3	25.35648	19560.29	25.36082	19561.68	25.35376	19563.53
19569.1	25.6044	19571.57	25.61198	19570.93	26.16544	19562.42	25.60948	19559.58	25.61283	19558.66	25.60662	19562.04
19570.93	25.85719	19572.38	25.85632	19569.32	26.41705	19561.39	25.85571	19562.51	25.85986	19561.51	25.85545	19562.56
19572.07	26.10673	19572.58	26.1059	19570.6	26.66916	19559.55	26.11617	19560.68	26.11242	19560.26	26.1121	19560.96
19570.83	26.35659	19569.87	26.35617	19570.17	26.9172	19561.5	26.35785	19560.21	26.36128	19560.84	26.35551	19562.86
19571.5	26.60642	19570.24	26.61353	19570.09	27.16629	19558.37	26.60994	19559.68	26.61261	19560.27	26.60678	19559.92
19572.35	26.85682	19570.26	26.85649	19572.37	27.41595	19560.26	26.85644	19560.65	26.86097	19560.18	26.85635	19561.88
19568.09	27.10594	19572.02	27.10826	19571.32	27.66651	19558.86	27.1103	19562.94	27.11266	19561.34	27.11079	19562.04
19571.18	27.35532	19571.44	27.35596	19570.63	27.91804	19560	27.3553	19561.03	27.36006	19561.47	27.35654	19561.84
19569.47	27.60619	19571.31	27.61197	19572.07	28.16783	19558.01	27.60907	19561.58	27.61298	19561.75	27.6079	19563.41
19573.4	27.85821	19570.46	27.85677	19569.77	28.41775	19559.88	27.85678	19562.07	27.86132	19562.91	27.85665	19561.96
19571.18	28.10687	19570.28	28.10736	19572.17	28.67861	19561	28.1105	19561.98	28.11389	19562.6	28.11402	19560.02
19570.92	28.35543	19570.16	28.35743	19571.91	28.91818	19561.1	28.35704	19559.43	28.36179	19560.94	28.35623	19560.59
19571.35	28.60889	19569.9	28.61146	19572.56	29.16807	19559.85	28.60951	19560.45	28.6125	19561.87	28.61266	19562
19569.45	28.85839	19570.44	28.85713	19571.53	29.41835	19561.26	28.85617	19561.7	28.86147	19561.01	28.85546	19563.77
19573.44	29.10695	19569.08	29.10678	19570.64	29.6703	19561.02	29.11639	19560.98	29.11497	19560.3	29.11305	19562.65
19570.85	29.35726	19569.5	29.35789	19572.48	29.91841	19559.19	29.35823	19561.83	29.3628	19562.97	29.35834	19562.06
19570.9	29.60607	19572.32	29.61185	19570.7	30.16734	19558.99	29.61103	19561.79	29.61406	19563.35	29.60888	19560.98
19570.91	29.85792	19568.06	29.85973	19570.04	30.41821	19561.07	29.85726	19560.29	29.86011	19561.11	29.85564	19562.97
19570.78	30.10909	19572.1	30.10677	19572.69	30.66964	19559.99	30.10927	19560.44	30.11395	19563.21	30.11021	19561.98
19572	30.35693	19570.69	30.35865	19569.79	30.91989	19560.8	30.35704	19562.07	30.36284	19560.23	30.35793	19562.43
19571.41	30.61074	19572.04	30.61724	19569.1	31.17023	19561.59	30.61194	19560.66	30.61589	19563.13	30.6104	19563
19572.38	30.85951	19570.83	30.85904	19571.65	31.41998	19560.57	30.86013	19560.57	30.86302	19562.33	30.85823	19562.94
19571.55	31.11001	19570.45	31.10801	19571.04	31.6694	19561.42	31.112	19561.74	31.1139	19563.08	31.11447	19562.78
19571.93	31.35887	19571.81	31.35875	19571.95	31.91945	19561.77	31.35802	19561.44	31.36263	19559.37	31.3583	19560.32
19568.36	31.60822	19571.87	31.6158	19571.56	32.17075	19561.42	31.61036	19563.61	31.61633	19561.36	31.61084	19561.98
19569.3	31.85958	19571.72	31.86612	19571.21	32.4207	19560.16	31.85907	19562.97	31.86353	19560.18	31.85825	19562.1
19571.11	32.10923	19568.41	32.11088	19571.04	32.6724	19562.08	32.1133	19561.43	32.11476	19560.68	32.11183	19562.31
19572.96	32.35883	19570.1	32.36001	19570.78	32.91881	19560.05	32.35917	19561.13	32.36295	19560.33	32.35949	19562.75
19571.71	32.60824	19569.88	32.61152	19572.23	33.17007	19561.47	32.61069	19562.1	32.61484	19564.25	32.60821	19561.75
19571.06	32.85842	19569.03	32.8588	19572.03	33.42054	19559.69	32.85757	19560.82	32.86303	19562.37	32.85808	19562.13
19569.2	33.10917	19571	33.10964	19572.13	33.6706	19559.72	33.11323	19560.93	33.11615	19560.61	33.11334	19561.33
19571.38	33.35836	19571.6	33.36016	19574.97	33.91839	19561.21	33.36024	19561.57	33.36162	19561.28	33.35796	19562.83
19570.67	33.60995	19569.37	33.61471	19568.66	34.1688	19561.12	33.61333	19560.38	33.61461	19562.73	33.60982	19560.99

19571.6	33.85928	19570.19	33.86639	19572.9	34.42037	19560.61	33.85833	19560.34	33.86279	19560.17	33.85795	19563.64
19570.29	34.10927	19572.52	34.10805	19571.08	34.67122	19560.38	34.11126	19561.81	34.11624	19560.46	34.11328	19563.3
19571.96	34.35765	19572.93	34.35907	19571.4	34.91937	19558.05	34.35995	19562.82	34.36259	19561.86	34.35944	19561.14
19569.16	34.60974	19572.01	34.61569	19570.79	35.16902	19560.37	34.61278	19562.7	34.61404	19560.45	34.60986	19562.74
19572.71	34.85986	19569.96	34.86039	19570.72	35.42071	19563.42	34.86166	19561.59	34.86296	19561.17	34.8572	19561.26
19570.31	35.10966	19570.9	35.10919	19573.91	35.67294	19561.6	35.11189	19560.51	35.11782	19560.5	35.1122	19561.36
19571.01	35.35735	19568.43	35.35795	19571.35	35.92088	19561.56	35.35902	19561.74	35.36528	19561.55	35.36151	19561.23
19569.68	35.60887	19569.32	35.61895	19568.46	36.16927	19558.9	35.61198	19561.48	35.616	19560.93	35.61191	19563.87
19573.51	35.86013	19571.06	35.86129	19572.64	36.41956	19561.03	35.86012	19561.73	35.86232	19557.86	35.85846	19561.06
19570.32	36.11046	19569.58	36.11114	19570.9	36.6703	19560.66	36.11294	19562.37	36.1158	19562.23	36.11311	19560.73
19573.06	36.35754	19570.79	36.3606	19570.37	36.92036	19559.38	36.3591	19560.5	36.36548	19561.75	36.35863	19562.5
19571.22	36.60906	19573.28	36.61435	19572.37	37.17105	19559.59	36.61305	19560.73	36.61653	19563.25	36.61113	19563.07
19570.79	36.86011	19570.16	36.8611	19572.33	37.42082	19559.96	36.86088	19560.85	36.86433	19561.02	36.85982	19561.84
19571.31	37.11162	19572.76	37.11053	19571.76	37.67041	19561.07	37.11589	19561.78	37.11601	19564.18	37.11224	19561.94
19573.92	37.35959	19571.41	37.36154	19571.36	37.92034	19561.29	37.35921	19562.38	37.36556	19560.57	37.35974	19561.93
19570.92	37.60914	19571.84	37.61426	19572.18	38.17126	19559.65	37.61357	19560.43	37.61732	19559.92	37.61239	19561.98
19571.64	37.85967	19571.41	37.86917	19572.83	38.42167	19559.96	37.86171	19562.73	37.86445	19559.43	37.8601	19562.39
19572.12	38.111	19573.24	38.10955	19569.91	38.67321	19560.5	38.11743	19561.44	38.11763	19561.7	38.11398	19560.96
19572.22	38.36014	19572.14	38.3607	19570.18	38.92045	19562.05	38.35931	19559.68	38.36345	19558.56	38.35968	19562.5
19569.77	38.6121	19569.71	38.61492	19571.41	39.17114	19562.46	38.61354	19560.11	38.61706	19560.38	38.61377	19562.05
19570.8	38.86033	19571.82	38.86161	19570.6	39.42194	19560.01	38.86164	19562.19	38.86506	19564.57	38.8611	19562.15
19568.75	39.11226	19572.9	39.11031	19570.94	39.67357	19560.09	39.11426	19562.55	39.11834	19560.64	39.11357	19563.43
19571.06	39.35947	19571.54	39.36002	19569.85	39.92195	19561.06	39.35982	19559.88	39.36449	19560.64	39.35981	19562.92
19569.49	39.61146	19570.04	39.61599	19571.53	40.17056	19561.8	39.61415	19560.35	39.61685	19560.34	39.61138	19561.9
19573.26	39.86378	19569.41	39.8627	19571	40.42267	19559.39	39.86291	19560.53	39.86525	19560.78	39.86026	19564.06
19570.05	40.12096	19571.03	40.11176	19571.62	40.67612	19559.4	40.11665	19561.78	40.11954	19560.89	40.11575	19563.19
19570.92	40.36934	19568.96	40.36176	19571.36	40.92364	19560.16	40.36106	19562.31	40.36785	19560.72	40.36234	19561.25
19572.83	40.61793	19571.81	40.61771	19572.21	41.17381	19560.46	40.61443	19561.3	40.61951	19560.25	40.61256	19562.82
19570.93	40.86566	19571.99	40.86182	19571.35	41.42721	19562.75	40.87197	19560.45	40.86486	19560.59	40.86114	19562.46
19569.82	41.1165	19569.13	41.11258	19572.09	41.67426	19560.57	41.13844	19563.3	41.11939	19562.38	41.11616	19563.16
19571.01	41.37401	19570.23	41.36339	19571.16	41.92387	19559.17	41.36771	19560.2	41.36759	19563.27	41.36377	19562.54
19570.17	41.61492	19568.57	41.61523	19571.96	42.17432	19558.35	41.62325	19562.11	41.61987	19558.76	41.61477	19563.02
19569.47	41.86334	19568.5	41.86237	19570.52	42.4245	19559.53	41.86374	19561.29	41.86638	19562.61	41.86051	19563.58
19570.42	42.11632	19569.69	42.11813	19569.98	42.67559	19562.28	42.11816	19561.36	42.11887	19560.97	42.11614	19561.07
19569.34	42.36204	19569.94	42.36285	19571.42	42.92421	19560.14	42.36461	19561.76	42.36857	19560.66	42.36246	19561.1
19571.19	42.61274	19571.99	42.6188	19570.35	43.17472	19562.78	42.61641	19560.52	42.61998	19562.81	42.61767	19562.77
19571	42.86361	19570.9	42.86482	19572.21	43.42448	19562.49	42.86442	19561.65	42.86849	19560.88	42.86169	19560.01
19570.04	43.11481	19570.73	43.11508	19571.79	43.67561	19560.27	43.12153	19560.84	43.11927	19563.01	43.11708	19562.57
19571.3	43.36429	19570.64	43.36222	19570.69	43.92338	19561.16	43.3642	19561.15	43.3675	19561.2	43.36253	19560.61
19570.92	43.61561	19569.68	43.61784	19572.22	44.17404	19561.31	43.61693	19562.65	43.62011	19560.36	43.61384	19562.45
19570.75	43.86395	19571.12	43.86316	19571.23	44.42557	19560.59	43.86419	19562.32	43.86835	19563.03	43.86305	19562.08
19568.02	44.11417	19569.36	44.11506	19573.03	44.67702	19560.19	44.11729	19561.66	44.12148	19561.38	44.12196	19560.16
19570.95	44.36327	19569.37	44.36472	19571.24	44.92451	19559.58	44.36566	19560.53	44.36793	19560.75	44.36378	19563.16
19570.73	44.61736	19570.01	44.61457	19569.01	45.17402	19560.42	44.61842	19561.3	44.62007	19563.25	44.61525	19561.62
19571.45	44.86464	19572.02	44.87248	19573.01	45.42461	19559.8	44.86573	19562.22	44.86857	19560.49	44.86377	19560.42
19571.94	45.11645	19572.41	45.11415	19570.53	45.67616	19559.67	45.11841	19561.33	45.12187	19561.68	45.11843	19561.84
19571.87	45.36202	19570.17	45.36448	19573.35	45.9256	19562.09	45.36453	19561.12	45.3689	19560.96	45.36442	19561.81
19569.95	45.61401	19570.66	45.62076	19570.87	46.17638	19560.21	45.61735	19561.52	45.62079	19561.73	45.61667	19559.52
19572.21	45.86428	19570.99	45.86409	19572.8	46.42423	19560.45	45.8663	19560.45	45.86727	19560.89	45.86418	19560.88
19571.39	46.11569	19569.93	46.11542	19570.38	46.68462	19556.79	46.12384	19561.45	46.12163	19561.99	46.11715	19560.12
19571.64	46.36678	19571.06	46.3652	19570.44	46.92693	19557.85	46.3638	19561.95	46.37805	19560.57	46.36609	19561.41
19572.75	46.61866	19570.66	46.61872	19570.68	47.17663	19560.24	46.61648	19561.56	46.62213	19560.92	46.6167	19561.53
19570.45	46.86687	19571.23	46.86533	19569.5	47.4272	19559.96	46.86619	19562.96	46.87056	19562.66	46.86375	19561.84
19571.83	47.11757	19570.97	47.11595	19573.39	47.67603	19559.94	47.12312	19559.88	47.12098	19561.95	47.11853	19562.35
19569.29	47.36699	19569.2	47.36427	19572.09	47.92486	19559.04	47.36456	19561.46	47.36808	19560.69	47.36619	19562.54
19569.82	47.62388	19569.4	47.61725	19572.27	48.17498	19561.13	47.61814	19561.16	47.62272	19560.75	47.61822	19562.56
19570.03	47.87146	19573.08	47.866	19571.09	48.42596	19559.97	47.86527	19560.53	47.8693	19561.66	47.86426	19562.05
19571.25	48.11982	19571.44	48.11713	19573.76	48.67996	19559.42	48.12611	19561.31	48.12115	19561.33	48.11729	19562.67
19571.71	48.3692	19568.16	48.36693	19573.41	48.92356	19558.6	48.36651	19560.46	48.36787	19560.09	48.36566	19562.5
19570.37	48.62438	19571.05	48.61501	19573.46	49.17343	19560.56	48.61629	19560.94	48.62107	19559.97	48.61721	19563.31
19571.75	48.86537	19569.49	48.87351	19571.23	49.42442	19558.52	48.86475	19563.18	48.86873	19560.59	48.8637	19562.28
19570.39	49.11836	19570.94	49.11593	19571.13	49.67809	19559.48	49.12188	19561.55	49.12142	19559.99	49.11615	19562.2
19570.57	49.3665	19571.24	49.36567	19571.28	49.92568	19556.49	49.36539	19561.55	49.36808	19560.97	49.36317	19561.59
19570.75	49.62453	19571.72	49.61968	19572.01	50.1775	19561.09	49.61925	19560.23	49.62324	19561.47	49.61878	19560.77
19572.43	49.86962	19568.93	49.86384	19572.08	50.42488	19558.68	49.86405	19559.62	49.8691	19561.85	49.86427	19562.21
19569.38	50.12197	19571.53	50.11438	19570.94	50.67647	19560.94	50.13306	19560.66	50.12268	19561.5	50.11826	19561.17
19571.24	50.37064	19572.93	50.36717	19572.54	50.92596	19561.35	50.36884	19561.46	50.36903	19562.22	50.36371	19562.06
19570.11	50.62237	19571.07	50.61879	19571.69	51.17617	19559.25	50.62083	19561.46	50.62112	19561.75	50.61848	19561.58
19570.25	50.86971	19567.33	50.86705	19569.19	51.42609	19561.59	50.8657	19561.62	50.86861	19561.65	50.86459	19561.22
19568.37	51.12187	19568.71	51.11639	19571.05	51.67966	19560.9	51.1202	19561.6	51.12198	19562.52	51.11899	19562.38

19571.07	51.37671	19572.72	51.37193	19571.42	51.92456	19561.07	51.36641	19561.66	51.36948	19560.78	51.3649	19562.04
19570.66	51.6192	19569.41	51.62439	19573.93	52.17582	19559.55	51.625	19562.6	51.62308	19559.94	51.61795	19561.98
19570.61	51.87673	19572.7	51.86594	19572.57	52.42657	19559.87	51.86593	19562.6	51.86919	19560.81	51.86418	19560.13
19570.26	52.12239	19572.21	52.11739	19572.83	52.67913	19561.32	52.12218	19561.1	52.1242	19561.06	52.11696	19562.9
19572.72	52.37232	19567.58	52.36477	19572.67	52.92628	19561.98	52.36418	19562.98	52.37482	19560.61	52.36631	19563.2
19569.08	52.62125	19570.95	52.6191	19572.9	53.17536	19561.91	52.61884	19561.12	52.62833	19561.57	52.62252	19561.67
19569.08	52.87207	19570.74	52.86563	19570.34	53.42664	19559.6	52.86841	19560.37	52.87679	19561.61	52.86589	19562.06
19572.88	53.12293	19568.59	53.11855	19572.28	53.67922	19559.29	53.12291	19562.14	53.12559	19560.24	53.11606	19562.31
19572.64	53.38021	19571	53.36764	19570.48	53.92654	19561.54	53.36609	19560.09	53.37006	19561.35	53.36641	19562
19572.46	53.62484	19568.86	53.61703	19570.86	54.17732	19562.9	53.61902	19559.97	53.62442	19560.7	53.61935	19561.66
19572.05	53.87444	19567.91	53.86528	19569.06	54.42568	19559.42	53.86619	19560.94	53.87004	19562.02	53.86567	19561.5
19569.82	54.11791	19570.39	54.11847	19573.28	54.67743	19561.46	54.12516	19562.63	54.12207	19561.81	54.11953	19561.7
19571.2	54.36563	19568.87	54.36824	19572.66	54.92685	19563.31	54.36781	19560.16	54.37	19562.07	54.36487	19562.01
19571.92	54.62004	19572.82	54.62287	19571.51	55.17741	19561.52	54.61945	19561.35	54.6253	19558.98	54.61944	19562.85
19569.84	54.86827	19571.94	54.86689	19572.85	55.42741	19558.57	54.86629	19560.78	54.87189	19561.48	54.8662	19561.49
19570.52	55.11863	19571.99	55.11878	19572.42	55.67791	19560.92	55.12261	19560.97	55.12473	19560.59	55.12104	19562.29
19573.28	55.3658	19570.97	55.36574	19571.67	55.92722	19561.23	55.36748	19562.35	55.37046	19559.55	55.36552	19562.13
19572.9	55.61728	19571.57	55.62539	19572.48	56.18093	19559.19	55.61941	19560.88	55.62533	19560.57	55.61958	19559.49
19570.77	55.86717	19570.28	55.86698	19571.93	56.42889	19559.41	55.86646	19562.6	55.87172	19560.31	55.86685	19562.01
19571.61	56.11852	19571.28	56.12073	19572.4	56.68063	19560.13	56.12693	19559.89	56.12501	19560.34	56.11959	19561.03
19569.82	56.36661	19571.68	56.36779	19573.71	56.92721	19560.3	56.36873	19558.42	56.37056	19563.02	56.36818	19562.55
19570.76	56.61854	19570.47	56.6172	19573.44	57.17692	19561.14	56.62179	19560.61	56.62515	19562.44	56.61914	19562.3
19570.82	56.86635	19570	56.8765	19572.08	57.42714	19559.96	56.86728	19559.73	56.87097	19560.57	56.86529	19562.9
19570.89	57.11812	19570.66	57.11877	19572.75	57.68084	19560.78	57.128	19562.68	57.12471	19562.32	57.11902	19561.75
19571.34	57.36722	19570.8	57.36809	19571.3	57.92958	19559.77	57.36911	19560.05	57.37201	19563.35	57.36694	19558.99
19572.02	57.61803	19571.44	57.62239	19573.26	58.17788	19560.37	57.62319	19561	57.62509	19559.1	57.62074	19563.05
19568.92	57.86947	19572.3	57.86877	19571.93	58.42642	19560.2	57.86706	19561.98	57.87148	19561.65	57.86765	19561.36
19568.97	58.12706	19570.6	58.11849	19574.1	58.67964	19559.48	58.12603	19559.63	58.12576	19563.92	58.12094	19561.18
19572.3	58.36606	19571.15	58.36762	19573.01	58.92948	19558.24	58.36796	19561.31	58.37224	19562.69	58.36768	19561.43
19570.8	58.62031	19571.57	58.6214	19573.13	59.17898	19559.73	58.62144	19560.25	58.62591	19560.75	58.62092	19561.76
19571.66	58.87776	19571.09	58.86941	19569.41	59.42954	19560.48	58.86896	19561.41	58.87115	19559.13	58.87628	19560.86
19571.36	59.12023	19571.46	59.11977	19570.48	59.6809	19559.99	59.12608	19561.02	59.12563	19563.81	59.12984	19561.88
19569.81	59.36597	19569.51	59.36936	19569.4	59.92848	19559.79	59.36673	19563.88	59.38402	19562.2	59.36687	19560.99
19572.44	59.61993	19570.44	59.61753	19571.3	60.17886	19561.88	59.62084	19561.4	59.6265	19560.01	59.61996	19561.32
19569.94	59.86869	19569.99	59.86877	19571.84	60.43088	19561.49	59.87023	19560.31	59.87298	19558.47	59.86838	19560.39
19571.75	60.11996	19568.19	60.12064	19574.05	60.68691	19561.41	60.12945	19559.56	60.12611	19563.01	60.12206	19563.69
19572.17	60.36955	19571.05	60.37128	19571.17	60.93257	19560.86	60.36988	19561.35	60.37524	19563.08	60.36899	19562.52
19571.55	60.62104	19570.78	60.623	19572.57	61.17969	19560.54	60.6235	19561.54	60.62888	19562.34	60.6225	19560.74
19571.71	60.87053	19568.87	60.87983	19570.99	61.43134	19560.99	60.87124	19562.7	60.87504	19560.45	60.8698	19562.39
19569.05	61.12119	19568.27	61.12055	19570.6	61.68338	19561.58	61.132	19559.49	61.12723	19560.82	61.12513	19560.82
19569.94	61.3702	19569.67	61.37092	19569.83	61.93205	19562.09	61.37157	19560.22	61.37356	19560.31	61.37081	19561.18
19572.45	61.62198	19571.06	61.6237	19570.72	62.17991	19559.53	61.6241	19562.2	61.62696	19562.08	61.62279	19564.05
19571.36	61.87086	19571.08	61.87059	19570.14	62.43079	19561.05	61.87017	19559.17	61.87519	19560.54	61.86986	19563.88
19568.31	62.12134	19571.6	62.11956	19571.96	62.68495	19559.17	62.13748	19561.69	62.12813	19563.07	62.12701	19560.96
19571.26	62.36965	19570.51	62.37066	19572.58	62.93341	19562.49	62.37511	19561.94	62.37617	19562.24	62.37181	19562.91
19570.98	62.62339	19569.28	62.62402	19571.23	63.18092	19559.21	62.62638	19560.85	62.62721	19562.08	62.62562	19561.03
19572.26	62.87236	19570.95	62.87237	19571.65	63.43175	19559.53	62.87214	19561.83	62.87391	19561.41	62.86852	19561.75
19569.41	63.12276	19570.8	63.12297	19571.81	63.68514	19559.77	63.13183	19561.35	63.12835	19560.84	63.12531	19563.21
19572	63.36954	19570.03	63.37092	19569.64	63.93315	19561.95	63.37328	19561.62	63.37653	19561.75	63.37175	19561.37
19571.59	63.62102	19570.43	63.62221	19569.11	64.18261	19560.33	63.628	19560.52	63.62943	19559.93	63.62538	19561.43
19572.51	63.87271	19569.2	63.87172	19570.5	64.4313	19562.52	63.87235	19562.86	63.87377	19562.9	63.87073	19562.91
19570.08	64.12363	19571.74	64.12311	19568.53	64.68573	19558.54	64.13208	19561.62	64.12765	19560.39	64.1252	19562.51
19568.71	64.3723	19571.86	64.37332	19572.19	64.93498	19561.27	64.37264	19561.45	64.37593	19561.62	64.3716	19561.13
19570.75	64.62123	19569.36	64.61856	19571.13	65.18169	19561.83	64.62609	19562.31	64.62783	19562.2	64.62145	19562.36
19572.04	64.87095	19569.66	64.87147	19571.81	65.43171	19559.33	64.87143	19562.11	64.87452	19561.76	64.87059	19562.56
19571.17	65.12296	19569.88	65.12363	19571.31	65.68289	19558.93	65.13103	19561.42	65.12813	19562.12	65.12293	19564.1
19569.46	65.37066	19571.39	65.37248	19570.42	65.93097	19560	65.37103	19559.61	65.37439	19561.74	65.37156	19562.14
19571.3	65.61937	19570.54	65.62212	19572.85	66.18195	19561.35	65.62508	19560.38	65.62865	19560.94	65.62278	19564.97
19572.37	65.87041	19572.11	65.87842	19572.45	66.43186	19560.89	65.873	19558.54	65.87589	19561.11	65.86936	19563.82
19571.67	66.12284	19570.39	66.12056	19571.74	66.68657	19558.52	66.12831	19561.08	66.12692	19561.94	66.12622	19562.37
19570.21	66.37194	19570.92	66.37169	19571.88	66.93246	19559.57	66.37083	19561.29	66.37453	19562.74	66.37099	19559.22
19570.4	66.62194	19569.76	66.62409	19572.41	67.18024	19560.94	66.62541	19559.95	66.62784	19560.85	66.6256	19559.01
19569.4	66.87129	19569.61	66.87087	19572.89	67.43114	19561.58	66.87185	19559.97	66.87667	19560.79	66.86987	19561.1
19571.44	67.12323	19571.67	67.12263	19569.82	67.68643	19560.25	67.131	19561.45	67.12922	19562.18	67.12318	19561.8
19571.12	67.37342	19571.35	67.37269	19571.73	67.9327	19560.21	67.37232	19561.38	67.37586	19561.96	67.37202	19559.16
19572.33	67.62469	19570.96	67.6304	19572.06	68.18106	19559.44	67.62565	19559.75	67.62771	19559.37	67.62591	19562.01
19570.11	67.87145	19569.38	67.87783	19571.22	68.43065	19560.84	67.87214	19561.11	67.87525	19562.62	67.87067	19561.64
19571.15	68.12236	19570.38	68.12197	19573.26	68.6959	19561.58	68.13208	19560.52	68.1296	19563.03	68.12282	19562.36
19571.56	68.37138	19570.35	68.3711	19570.3	68.93396	19561.95	68.37435	19561.03	68.37708	19559.71	68.37044	19561.87
19571.21	68.6235	19568.59	68.62242	19570.09	69.18263	19559.84	68.62556	19561.13	68.62961	19561.58	68.6239	19562.37

19571.22	68.88346	19570.88	68.87316	19571.26	69.43306	19561.01	68.8775	19558.2	68.87528	19560.25	68.87176	19562.12
19570.87	69.12301	19570.68	69.12249	19569.73	69.68579	19560.76	69.13341	19559.97	69.12771	19561.9	69.12591	19561.63
19570.34	69.37149	19571.58	69.37118	19572.21	69.93136	19560.79	69.37368	19561.5	69.37702	19559.72	69.36996	19561.35
19568.77	69.62296	19571.33	69.6234	19570.44	70.18268	19560.91	69.62661	19561.54	69.62997	19560.67	69.6246	19560.85
19570.15	69.87282	19571.07	69.87339	19571.27	70.43285	19562.33	69.87322	19561.17	69.87619	19559.46	69.87208	19563.86
19571.17	70.12359	19571.22	70.1238	19570.88	70.68634	19559.65	70.13085	19560.44	70.12951	19560.67	70.12601	19561.54
19571.5	70.37341	19568.36	70.37196	19573.12	70.93251	19560.61	70.37319	19561.78	70.37727	19560.31	70.37323	19560.88
19570.57	70.62375	19568.89	70.62667	19570.68	71.18264	19562.04	70.62864	19562.32	70.63038	19563.09	70.62716	19560.73
19570.84	70.87234	19570.95	70.8722	19571.07	71.43401	19560.22	70.87354	19560.16	70.87695	19562.77	70.8715	19562.75
19571.84	71.12511	19572.12	71.12471	19569.34	71.68778	19557.65	71.12954	19560.75	71.12919	19560.72	71.12691	19562.05
19570.49	71.37388	19571.87	71.37411	19571.39	71.9331	19559.12	71.37335	19562.6	71.37684	19560.39	71.37438	19561.71
19568.81	71.62498	19572.95	71.62286	19571.89	72.18226	19557.86	71.62776	19561.26	71.62924	19561.39	71.62688	19561.75
19569.6	71.87298	19570.97	71.87267	19571.17	72.43332	19560.22	71.88161	19559.82	71.87928	19561.46	71.87315	19563.12
19570.56	72.12369	19571.03	72.12584	19571.26	72.68743	19559.21	72.14172	19560.5	72.13019	19562.47	72.1248	19562.42
19571.62	72.37464	19571.98	72.37456	19572.53	72.93368	19560.37	72.37273	19560.55	72.37815	19562.41	72.38359	19560.98
19571.97	72.62539	19572.42	72.62834	19572.25	73.18413	19559.63	72.62865	19561.83	72.62966	19559.84	72.62606	19561.29
19570.48	72.87523	19572.29	72.87341	19572.4	73.43315	19560.24	72.87485	19560.98	72.87763	19562.07	72.87492	19561.87
19570.93	73.12348	19570.72	73.12445	19571.53	73.68882	19561.12	73.13491	19561.56	73.131	19561.37	73.12796	19559.55
19568.65	73.37372	19569.17	73.37478	19571.16	73.9354	19561.04	73.37433	19561.35	73.37963	19562.23	73.37472	19560.46
19570.07	73.62624	19572.16	73.627	19571.02	74.18466	19560.75	73.62673	19561.34	73.62987	19562.1	73.62685	19564.57
19573.27	73.8855	19570.08	73.87413	19572.38	74.43399	19561.66	73.87368	19560.04	73.87771	19564.07	73.87488	19561.24
19571.07	74.12552	19571.23	74.1232	19571.92	74.68735	19559.16	74.13193	19560.35	74.13132	19560.28	74.12771	19562.44
19571.61	74.37265	19571.53	74.37554	19569.86	74.93415	19557.82	74.37615	19561.94	74.37909	19560.02	74.37432	19562.94
19570.07	74.62436	19571.86	74.62787	19570.96	75.18561	19558.52	74.6294	19561.1	74.6321	19562.03	74.62658	19562.53
19571.71	74.87426	19573.64	74.87657	19571.47	75.4355	19560.39	74.87394	19561.49	74.87878	19560.75	74.8745	19560.95
19572.49	75.12575	19570.65	75.12546	19571.07	75.68969	19558.41	75.13195	19561.49	75.1321	19561.45	75.12869	19561.92
19569.53	75.37673	19571.23	75.3755	19569.83	75.93466	19558.94	75.37566	19559.48	75.37974	19559.86	75.37571	19559.48
19571.68	75.62624	19572.89	75.63149	19570.97	76.18505	19559.9	75.62926	19561.53	75.63253	19559.69	75.62642	19561.57
19569.36	75.87454	19570.91	75.87544	19571.87	76.43555	19560.94	75.87659	19562.28	75.8825	19561.28	75.87331	19560.42
19569.36	76.12558	19571.89	76.12596	19570.56	76.69236	19557.97	76.13473	19560.89	76.12999	19562.05	76.12652	19561.77
19571.06	76.37518	19568.42	76.37555	19571.41	76.93511	19559.24	76.37453	19561.45	76.38083	19561.06	76.37716	19560.27
19570.2	76.62736	19568.59	76.62452	19572.38	77.18374	19560.2	76.6294	19561.98	76.63216	19561.41	76.62808	19561.86
19570.45	76.87437	19570.72	76.88851	19570.83	77.43447	19560.57	76.87735	19561.29	76.88107	19560.27	76.87513	19563.09
19568.74	77.12559	19570.48	77.1257	19570.12	77.7015	19560.65	77.1316	19561.78	77.1326	19560.67	77.12588	19563.23
19571.28	77.37359	19571.31	77.37686	19570.87	77.94098	19560.87	77.37638	19559.69	77.37815	19561.32	77.37535	19560.91
19572.46	77.62644	19572.43	77.62736	19571.88	78.18595	19561.24	77.62871	19563.11	77.63182	19560.59	77.62949	19561.3
19572.5	77.88669	19568.51	77.87427	19572.29	78.4343	19558.22	77.87613	19559.52	77.88065	19562.49	77.87691	19561.93
19571.95	78.12743	19568.93	78.12652	19570.81	78.69168	19559.84	78.13495	19561.04	78.13282	19560.09	78.12785	19561.09
19572.61	78.37413	19570.2	78.3762	19572.65	78.93695	19560.73	78.37731	19559.68	78.37923	19564.01	78.37501	19562.44
19569.3	78.62606	19571.63	78.62992	19571.16	79.18723	19560.04	78.63096	19561.95	78.63192	19561.66	78.62874	19560.67
19570.13	78.87597	19570.29	78.8781	19572.5	79.43685	19557.83	78.87662	19561.68	78.88157	19562.58	78.87669	19563.62
19572.36	79.12707	19571.02	79.1259	19571.19	79.68956	19558.91	79.14139	19562.11	79.13274	19559.89	79.12825	19562.34
19571.03	79.3759	19571.39	79.37691	19570.28	79.93544	19558.47	79.38297	19561.39	79.38067	19560.63	79.37552	19561.24
19571.46	79.62695	19571.01	79.6286	19571.21	80.18819	19558.45	79.6309	19559.99	79.63245	19563.58	79.62879	19560.94
19571.09	79.8773	19569.03	79.87797	19573.49	80.43975	19559.12	79.87727	19560.09	79.87991	19562.21	79.87471	19562.98
19571.18	80.12848	19568.45	80.12561	19569.56	80.69291	19560.38	80.13532	19563.22	80.13433	19562.92	80.12737	19562.17
19571.43	80.3777	19569.48	80.38054	19571.57	80.93563	19560.02	80.37947	19560.66	80.38511	19562.74	80.38026	19560.68
19570.68	80.62916	19570.09	80.63147	19570.16	81.18773	19560.35	80.63119	19557.8	80.63463	19561.01	80.63013	19562.66
19570.7	80.87604	19570.23	80.88325	19568.87	81.43773	19561.18	80.87759	19559.89	80.88098	19560.81	80.87377	19561.83
19571.09	81.1282	19571.95	81.12974	19572.15	81.69315	19558.25	81.13579	19562.43	81.13247	19561.19	81.13107	19562.27
19567.94	81.37627	19570.36	81.37602	19570.84	81.93743	19558.68	81.37729	19559.02	81.38255	19561.92	81.37889	19562.35
19569.89	81.63044	19570.3	81.62714	19571.81	82.18646	19558.68	81.63166	19559.09	81.63378	19560.82	81.63003	19560.23
19566.96	81.88829	19569.95	81.8761	19573.38	82.43699	19558.57	81.87853	19559.25	81.88187	19560.01	81.8764	19560.4
19570.53	82.12826	19573.16	82.12811	19570.93	82.70946	19559.73	82.14107	19561.3	82.13446	19561.74	82.13007	19559.91
19571.31	82.37451	19567.99	82.3776	19570.39	82.9393	19556.22	82.37737	19561.01	82.38101	19561.94	82.37768	19561.62
19569.27	82.62954	19570.19	82.62762	19571.7	83.18966	19558.57	82.63011	19559.84	82.63451	19560.16	82.63054	19559.83
19569.29	82.87824	19570.38	82.87734	19571.11	83.43644	19560.31	82.88984	19561.5	82.89239	19559.89	82.87684	19560.82
19566.97	83.13327	19568.72	83.12861	19568.83	83.69488	19560.74	83.14164	19559.73	83.1356	19562	83.13253	19561.14
19568.55	83.37891	19570.14	83.37824	19572.11	83.93793	19561.17	83.3825	19558.99	83.3807	19560.33	83.37755	19563.27
19570.27	83.62991	19568.89	83.63045	19571.36	84.19002	19560.12	83.63883	19561.08	83.63395	19559.23	83.63039	19560.71
19572.15	83.87864	19568.59	83.87798	19569.89	84.43822	19558.12	83.8804	19560.66	83.88312	19560.73	83.87687	19561.52
19571.26	84.13077	19570.19	84.13041	19572.76	84.69185	19558.25	84.14203	19561.46	84.1354	19563.22	84.13263	19560.78
19571.26	84.37741	19569.08	84.38007	19570.44	84.9386	19559.63	84.3884	19560.39	84.38257	19560.9	84.37948	19562.3
19570.18	84.62867	19570.45	84.62968	19569.91	85.18956	19558.17	84.63393	19560.48	84.6342	19560.47	84.63021	19560.69
19571.93	84.87777	19567.79	84.87795	19567.21	85.43877	19558.67	84.88593	19559.46	84.88433	19561.39	84.87707	19559.13
19570.11	85.1297	19567.66	85.12831	19570.24	85.69284	19559.87	85.13925	19560.28	85.13735	19561.17	85.13193	19560.18
19569.63	85.37776	19572.93	85.37846	19569.8	85.93868	19557.01	85.3822	19561	85.38504	19561.11	85.38103	19560.35
19570.43	85.63035	19570.52	85.63333	19571.09	86.19107	19557.08	85.63234	19559.57	85.63675	19558.97	85.63214	19560.35
19572.61	85.87919	19570.78	85.87932	19569.87	86.43909	19559.64	85.8818	19559.29	85.88255	19559.46	85.87686	19561.41
19568.58	86.12905	19570.5	86.12987	19570.24	86.69556	19558.7	86.14046	19560.13	86.13613	19560.74	86.132	19559.59

19569.8	86.37769	19570.55	86.37739	19569.46	86.9389	19557.97	86.38258	19560.55	86.38761	19562.76	86.37976	19561.47
19568.61	86.6301	19569.35	86.62894	19569.17	87.18985	19556.93	86.63001	19562.93	86.63635	19561.49	86.63265	19561.64
19568.73	86.88071	19569.29	86.88098	19568.05	87.43912	19557.76	86.87779	19559.44	86.88399	19560.83	86.87907	19563.29
19570.38	87.13064	19570.77	87.13259	19569.89	87.69585	19558.37	87.14508	19561.83	87.13491	19558.44	87.13214	19559.38
19567.65	87.37666	19570.59	87.38132	19572.53	87.94253	19558.07	87.38564	19560.66	87.38214	19560.2	87.37867	19560.77
19570.72	87.63248	19570.52	87.631	19569.92	88.19027	19556.84	87.63289	19561.06	87.63596	19559.94	87.63246	19561.88
19570.96	87.87991	19570.52	87.87873	19568.85	88.43886	19560.22	87.88019	19561.78	87.88328	19559.62	87.87956	19559.39
19568.29	88.13106	19570	88.13146	19569.46	88.69695	19559.19	88.14167	19558.11	88.13696	19561.65	88.13214	19560.26
19570.27	88.37845	19569.54	88.38074	19570.6	88.93967	19557.35	88.38018	19559.24	88.38347	19561.27	88.37809	19559.4
19572.62	88.63061	19570.1	88.62912	19567.43	89.19237	19560.49	88.63457	19559.66	88.63567	19560.85	88.65415	19560.53
19570.37	88.87929	19567.4	88.87879	19571.67	89.44068	19557.84	88.88002	19560.19	88.88303	19559.72	88.88999	19562.82
19568.06	89.13078	19569.28	89.13275	19568.65	89.69671	19559.66	89.14085	19560.67	89.13682	19559.34	89.13483	19562.02
19568.33	89.38116	19569.33	89.38078	19570.49	89.93951	19559.13	89.38046	19559.89	89.38599	19560.94	89.37986	19561.37
19567.25	89.63411	19569.23	89.63115	19571.4	90.19198	19557.75	89.63497	19559.31	89.63786	19560.62	89.63253	19560.18
19569.83	89.87937	19568.63	89.87876	19570.82	90.44147	19558.17	89.8806	19559.46	89.88546	19558.65	89.87828	19558.16
19569.91	90.13058	19568.56	90.13094	19569.22	90.6968	19558.96	90.13918	19559.5	90.1379	19560.67	90.13467	19560.37
19569.37	90.37884	19568.19	90.38186	19569.35	90.94017	19556.92	90.37925	19559.21	90.38443	19562.42	90.3821	19560.54
19568.82	90.63422	19569.6	90.63574	19570.5	91.19096	19556.96	90.63354	19559.74	90.63799	19560.32	90.63696	19559.33
19569.22	90.88177	19570.57	90.88173	19571.33	91.44103	19559.74	90.88074	19559.66	90.88624	19560.52	90.87895	19560.82
19566.75	91.1306	19570.26	91.13079	19571.02	91.69461	19558.36	91.13923	19559.99	91.13913	19561.28	91.13473	19560.29
19568.11	91.37946	19566.71	91.38171	19569.38	91.94209	19557.33	91.3819	19558.21	91.38524	19557.41	91.3814	19559.6
19568.36	91.63235	19567.54	91.63246	19570.29	92.19233	19556.44	91.63524	19559.96	91.63803	19561.42	91.63482	19558.84
19570.23	91.88061	19569.09	91.88105	19570.82	92.44008	19555.77	91.87985	19558.77	91.88553	19560.1	91.88116	19560.14
19569.2	92.13244	19568.82	92.13249	19570.61	92.69594	19556.44	92.15551	19559.09	92.13827	19559.54	92.13335	19558.56
19568.48	92.37918	19567.68	92.37905	19569.18	92.94193	19556.89	92.38249	19559.69	92.38659	19559.55	92.38132	19560.38
19568.11	92.63346	19568.13	92.63266	19567.42	93.19414	19556.63	92.63648	19558.38	92.639	19559.43	92.63528	19560
19568.3	92.88207	19566.45	92.88235	19568.75	93.44303	19556.8	92.8807	19558.07	92.88502	19559.95	92.88154	19560.42
19569.5	93.13235	19566.62	93.13464	19569.35	93.6983	19558.02	93.14041	19560.45	93.13742	19557.58	93.13726	19560.4
19571.12	93.38077	19567.78	93.38299	19570.24	93.94116	19558.68	93.38599	19558.19	93.39509	19558.59	93.38169	19561.55
19569	93.63393	19568.34	93.63357	19567.89	94.19198	19555.61	93.64386	19560.49	93.64531	19559.43	93.63414	19559.4
19569.93	93.88189	19569.36	93.88061	19571.49	94.44218	19557.79	93.88322	19557.44	93.88624	19559.84	93.88154	19560.84
19568.21	94.13351	19566.31	94.13442	19568.83	94.69861	19557.04	94.14046	19558.92	94.1385	19560.26	94.13798	19559.14
19568.73	94.38109	19569.22	94.38339	19568.28	94.94246	19554.81	94.38224	19558.79	94.38565	19559.69	94.38188	19559.54
19570.33	94.63569	19568.76	94.63565	19569.59	95.19267	19558.7	94.63531	19558.26	94.63947	19558.63	94.63459	19560.49
19569.33	94.88171	19568.42	94.88113	19566.63	95.44166	19555.97	94.88217	19559.21	94.88757	19557.34	94.88199	19559.38
19567.6	95.1327	19568.46	95.13312	19567.72	95.70674	19555.47	95.15395	19558.14	95.14159	19560.87	95.13606	19559
19569.31	95.38167	19566.06	95.38341	19570.1	95.94585	19557.21	95.39347	19559.31	95.38689	19557.65	95.38446	19557.82
19569.69	95.63552	19568.96	95.64123	19569.97	96.19332	19557	95.63556	19558.43	95.64109	19558.98	95.63611	19559.56
19568.06	95.89504	19567.13	95.88271	19569.28	96.44345	19556.41	95.88135	19559.37	95.88768	19559.14	95.88034	19557.87
19567.84	96.13345	19567.2	96.13213	19568.39	96.69731	19556.22	96.14265	19556.87	96.14278	19558.36	96.13561	19558.57
19568.54	96.38099	19569.38	96.38198	19568.68	96.94299	19556.38	96.38437	19557.22	96.38685	19558.6	96.38486	19558.43
19567.15	96.63547	19567.53	96.63751	19568.38	97.19321	19557.11	96.63755	19559.64	96.64031	19559.01	96.6362	19558.48
19566.23	96.88386	19568.67	96.88422	19568.77	97.44438	19555.65	96.88167	19557.76	96.8876	19558.71	96.88137	19560.38
19566.27	97.13447	19568.74	97.13364	19568.35	97.70126	19558.99	97.14362	19557.95	97.14224	19557.54	97.13533	19558.22
19565.65	97.37998	19567.82	97.38139	19565.71	97.94431	19557.78	97.38469	19556.68	97.38887	19557.96	97.38355	19559.54
19569.07	97.63397	19566.49	97.6369	19567.77	98.19462	19556.88	97.63729	19558.69	97.63953	19557.85	97.63762	19558.18
19569.36	97.88395	19567.96	97.88383	19567.57	98.44982	19556.62	97.88475	19558.57	97.88641	19559.65	97.88353	19558.14
19567.75	98.13516	19567.17	98.13533	19567.28	98.70153	19556.12	98.14176	19558.51	98.14176	19558.27	98.13749	19559.44
19567.4	98.38292	19567.84	98.38318	19568.88	98.94344	19557.02	98.38221	19557.32	98.39033	19558.75	98.38554	19560.48
19566.93	98.63299	19567.15	98.63538	19565.68	99.19411	19558.05	98.63869	19556.22	98.64105	19556.86	98.63649	19556.23
19566.41	98.88448	19566.08	98.88344	19567.34	99.44564	19557.64	98.8838	19559.22	98.88701	19559.91	98.88416	19557.06
19565.9	99.13921	19565.98	99.14002	19568.36	99.70241	19556.62	99.14978	19558.79	99.14802	19558.65	99.14093	19556.48
19566.57	99.38382	19569.89	99.38454	19567.75	99.94573	19556.51	99.3825	19557.4	99.38891	19555.17	99.38496	19556.63
19566.06	99.63669	19568.52	99.63698	19569.4	100.1935	19556.61	99.6387	19557.55	99.64235	19557.79	99.63818	19555.62
19568.56	99.88474	19568.59	99.88417	19570.26	100.4466	19555.75	99.88407	19558.51	99.88824	19557.7	99.88268	19555.69
19564.78	100.1341	19566.75	100.1356	19565.28	100.7104	19554.71	100.1432	19555.62	100.1435	19558.73	100.1379	19558.29
19565.97	100.3849	19564.89	100.3873	19568.62	100.9521	19553.71	100.3868	19558.38	100.3912	19556.16	100.3886	19558.21
19567.32	100.6381	19565.08	100.6423	19567.87	101.197	19554.86	100.6388	19556.44	100.6443	19555.88	100.6405	19556.36
19565.07	100.8886	19567.56	100.8857	19566.7	101.4474	19556.63	100.885	19556.88	100.8916	19558.16	100.8856	19558.31
19568.6	101.1375	19565.03	101.1385	19569.4	101.7035	19557.55	101.1459	19556.31	101.1468	19555.81	101.139	19557.3
19566.67	101.3844	19565.79	101.387	19565.83	101.9478	19557.73	101.3867	19556.49	101.3913	19557.51	101.3881	19557.33
19567.94	101.6379	19567.96	101.6408	19567.42	102.1974	19556.3	101.6424	19557.95	101.6444	19557.2	101.6414	19558.56
19564.87	101.8874	19564.6	101.8863	19565.25	102.4514	19556.37	101.8851	19558.23	101.892	19558.29	101.8875	19556.03
19565.36	102.1385	19566.21	102.1383	19566.93	102.7067	19553.37	102.1455	19555.38	102.1463	19558.09	102.1398	19555.56
19566.27	102.3855	19566.9	102.3868	19567.52	102.9479	19552.36	102.387	19556.73	102.3921	19554.73	102.3894	19557.23
19567.67	102.6378	19566.9	102.6393	19565.93	103.1983	19556.91	102.642	19554.69	102.6441	19554.41	102.641	19556.31
19564.35	102.8884	19565.29	102.8879	19566.25	103.4503	19555.08	102.8882	19557.79	102.8921	19558.57	102.8874	19558.52
19565.82	103.1384	19565.99	103.1402	19567.28	103.7079	19554.36	103.1483	19555.8	103.1454	19557.2	103.1425	19554.87
19566	103.3871	19566.44	103.3874	19565.91	103.9472	19554.83	103.3889	19558.81	103.4059	19554.2	103.3877	19557.67
19566.48	103.641	19565.08	103.6402	19565.11	104.1977	19557.21	103.6419	19557.77	103.6456	19558.32	103.6403	19556.39

19564.23	103.8888	19566.62	103.8886	19567.5	104.4492	19554.97	103.8896	19556.02	103.8922	19558.14	103.8876	19557.78
19563.34	104.1373	19566.7	104.1416	19568.1	104.7038	19557.08	104.1476	19557.93	104.1452	19556.05	104.1424	19558.44
19565.24	104.3859	19566.19	104.3883	19566.04	104.9507	19554.83	104.3865	19556.46	104.3933	19556.59	104.3887	19557.15
19564.56	104.6384	19563.12	104.6391	19566.21	105.1969	19555.8	104.6436	19556.48	104.6464	19553.89	104.6402	19558.12
19565.7	104.8905	19565.35	104.888	19565.27	105.4492	19554.07	104.888	19556.79	104.8927	19554.66	104.888	19558.17
19565.56	105.1415	19565.01	105.1418	19565.58	105.7067	19553.45	105.1483	19556.11	105.1459	19558.01	105.144	19556.6
19563.55	105.3865	19564.18	105.3899	19565.55	105.9507	19554.24	105.3876	19557.23	105.3925	19555.94	105.3896	19556.86
19565.7	105.6403	19564.94	105.6448	19564.46	106.2046	19555.34	105.6432	19558.03	105.646	19554.86	105.6428	19556.9
19566.32	105.8896	19565.56	105.8876	19564.83	106.4497	19553.34	105.8887	19555.78	105.893	19556.55	105.8875	19555.05
19567.12	106.1409	19565.77	106.1413	19566.15	106.7049	19553.14	106.15	19553.28	106.1489	19556.83	106.1408	19554.62
19564.46	106.3873	19564.34	106.3888	19566.31	106.9496	19554.19	106.3907	19556.2	106.3934	19555.35	106.3903	19555.29
19564.52	106.64	19565.04	106.6415	19566.35	107.2003	19554.32	106.6455	19556.12	106.6463	19554.88	106.6441	19555.71
19563.5	106.8918	19566.6	106.8878	19565.18	107.4516	19554.65	106.8879	19556.17	106.8927	19555.88	106.8894	19555.9
19564.71	107.1417	19566.83	107.1409	19562.85	107.7088	19554.48	107.1485	19557.8	107.1463	19555.22	107.1401	19555.74
19563.61	107.389	19562.66	107.3897	19564.68	107.95	19554.14	107.3906	19557.22	107.3945	19554.71	107.3896	19557.11
19566.76	107.6396	19565.1	107.6424	19566.57	108.2002	19552.28	107.6467	19555.11	107.6472	19555.7	107.6439	19554.76
19565.28	107.8909	19563.29	107.8896	19565.99	108.4513	19552.62	107.8877	19556.04	107.8927	19553.89	107.8908	19554.53
19564.3	108.1413	19565.14	108.1417	19565.35	108.7083	19554.19	108.1464	19554.7	108.1472	19552.65	108.1426	19555.26
19565.07	108.3885	19565.88	108.3892	19564.52	108.9504	19555.48	108.391	19556.9	108.3947	19553.15	108.3901	19556.41
19564.49	108.6416	19565.69	108.6419	19563.67	109.2016	19553.14	108.6449	19555.75	108.6473	19555.09	108.6434	19554.6
19565.69	108.8917	19562.83	108.8917	19563.69	109.4518	19556.93	108.8902	19553.7	108.8951	19554.74	108.8903	19554.92
19565.85	109.1404	19565.69	109.1431	19564.74	109.7122	19552.86	109.152	19553.52	109.1482	19555.75	109.1456	19552.98
19564.87	109.3894	19563.88	109.3919	19565.35	109.9578	19553.84	109.3912	19554.91	109.3934	19556.07	109.3955	19554.8
19566.04	109.6416	19564.49	109.6418	19563.42	110.2007	19554.77	109.6474	19556.08	109.6472	19555.48	109.6438	19555.47
19564.16	109.9028	19563.32	109.8901	19566.25	110.4505	19556.99	109.8908	19555.66	109.8961	19554.94	109.8889	19557.48
19565.65	110.1435	19563.9	110.1428	19565.71	110.7081	19553	110.1518	19556.79	110.1503	19557.1	110.1428	19555.9
19564.85	110.3891	19563.14	110.3907	19566.03	110.9526	19554.27	110.391	19554.7	110.3953	19555.43	110.3934	19557.19
19563.11	110.6401	19565.93	110.6475	19565.14	111.2028	19554.96	110.6474	19554.39	110.649	19553.86	110.6436	19553.94
19563.6	110.8926	19563.88	110.8931	19566.44	111.4502	19554.73	110.8908	19553.68	110.896	19554.97	110.8912	19558.32
19564.6	111.1427	19563.14	111.1442	19564.96	111.7067	19553.36	111.1511	19556.14	111.1511	19554.82	111.1439	19555.15
19563.17	111.3901	19564.88	111.3917	19564.09	111.9517	19554.01	111.3908	19557.46	111.3978	19555.33	111.3952	19554.73
19564.37	111.6424	19564.07	111.6591	19567.59	112.2034	19554.83	111.6454	19554.71	111.6484	19555.42	111.645	19555.6
19563.28	111.8924	19564.01	111.8953	19563.54	112.4527	19552.92	111.8905	19554.14	111.896	19552	111.8917	19555.49
19564.5	112.1429	19563.69	112.1428	19565.19	112.7084	19554.67	112.156	19552.26	112.1493	19553.48	112.1463	19555.68
19564.5	112.3911	19566.38	112.3916	19564.08	112.9514	19552.43	112.3933	19551.74	112.3969	19555.06	112.3927	19554.63
19565.73	112.644	19563.75	112.6454	19564.18	113.2037	19552.42	112.6455	19554.85	112.6486	19553.44	112.6457	19554.19
19565.61	112.8938	19564.09	112.8919	19564.85	113.4521	19551.32	112.8905	19553.18	112.8959	19555.79	112.892	19555.39
19564.08	113.1434	19564.03	113.1436	19565.67	113.7114	19554.83	113.1541	19554.97	113.1491	19554.12	113.1447	19557.58
19563.99	113.3927	19563.08	113.3917	19565.38	113.9528	19557.15	113.3932	19555.43	113.3978	19554.07	113.3936	19552.57
19562.36	113.6455	19565.38	113.6457	19564.08	114.2033	19552.43	113.6474	19553.98	113.6493	19555.62	113.6451	19554.44
19564.59	113.8955	19562.11	113.8938	19567.57	114.4532	19550.12	113.89	19552.12	113.8949	19555.21	113.8922	19554.06
19563.57	114.1439	19564.67	114.1435	19562.79	114.7107	19555.5	114.152	19554.17	114.1489	19554.14	114.1456	19554.06
19562.08	114.3914	19564.84	114.392	19563.72	114.9535	19554.01	114.3938	19552.27	114.398	19552.37	114.3954	19554.13
19563.79	114.6466	19564.04	114.6446	19562.83	115.2021	19552.68	114.6483	19555.96	114.651	19555.73	114.6475	19555.58
19564.11	114.8962	19563.09	114.8938	19565.03	115.4524	19555.9	114.8943	19555.92	114.8973	19556.39	114.891	19554.76
19564.16	115.142	19563.59	115.1464	19564.83	115.7099	19553.34	115.1546	19556.31	115.1521	19555.46	115.1463	19553.33
19564.55	115.3927	19563.46	115.3954	19563.67	115.9548	19553.81	115.3946	19554.22	115.3982	19553.97	115.3947	19556.19
19563.01	115.6446	19564.53	115.6506	19564.68	116.2046	19552.05	115.648	19554.39	115.6494	19552.79	115.647	19553.09
19562.95	115.8957	19564.64	115.8934	19563.62	116.4528	19554.42	115.8927	19552.18	115.8983	19553.23	115.8942	19553.68
19564.6	116.1442	19562.96	116.1455	19566.66	116.7097	19552.13	116.1549	19551.64	116.1517	19554.84	116.1448	19554.7
19562.9	116.3923	19562.88	116.394	19561.87	116.9538	19553.18	116.394	19553.31	116.3982	19554.84	116.3954	19552.95
19562.56	116.6433	19563.49	116.6451	19565.37	117.2046	19552.6	116.6472	19552.17	116.6496	19552.3	116.6467	19554.77
19561.5	116.8956	19562.74	116.8954	19562.83	117.4538	19550.7	116.8936	19554.92	116.8979	19555.04	116.8953	19554.24
19561.98	117.1441	19562.85	117.1456	19562.71	117.7105	19551.44	117.1543	19552.23	117.1529	19554.83	117.1493	19553.76
19563.82	117.3938	19562.75	117.3929	19565.27	117.9545	19552.98	117.3959	19552.44	117.4018	19555.35	117.3956	19554.27
19564.04	117.6439	19562.64	117.6469	19562.2	118.2054	19553	117.6473	19552.98	117.6506	19553.86	117.6475	19551.29
19564.13	117.8966	19562.36	117.8958	19563.04	118.4552	19551.14	117.8941	19551.26	117.8977	19551.12	117.8944	19554.73
19563.46	118.1445	19563.33	118.1474	19564.49	118.7134	19553.06	118.1547	19555.38	118.1519	19553.52	118.1499	19556.3
19562.28	118.4046	19564.1	118.3933	19565.06	118.9558	19552.57	118.3959	19553.16	118.4007	19553.74	118.3967	19552.33
19561.19	118.6459	19563.63	118.6455	19565.06	119.2038	19554.77	118.6489	19554.77	118.6517	19554.84	118.6472	19553.9
19563.52	118.9067	19562.63	118.896	19563.16	119.4541	19553.51	118.8939	19553.67	118.8976	19553.97	118.8933	19554.76
19563.85	119.1444	19564.22	119.147	19562.92	119.7159	19552.88	119.1538	19554.72	119.1523	19553.57	119.1479	19554.59
19562.98	119.3933	19563.6	119.3955	19563.51	119.9634	19554.21	119.3979	19552.79	119.4016	19553.65	119.4099	19551.14
19561.59	119.6465	19563.28	119.6456	19562.49	120.2058	19550.7	119.6492	19555.06	119.6525	19551.45	119.6498	19554.46
19563.02	119.8985	19562.1	119.8971	19563.02	120.4549	19552.89	119.8943	19554.37	119.8997	19553.6	119.895	19555.45
19563.09	120.1472	19563.11	120.1483	19561.84	120.7106	19553.08	120.154	19552.57	120.1559	19553.28	120.1475	19555.37
19562.08	120.3937	19564.29	120.396	19563.54	120.9542	19551.09	120.3995	19554.23	120.4014	19555.07	120.3969	19553.96
19562.54	120.6445	19565.04	120.6482	19564.78	121.2052	19551.77	120.6479	19556.07	120.651	19551.61	120.6473	19552.65
19562.07	120.8951	19562.36	120.895	19562.32	121.4529	19553.19	120.8921	19553.71	120.8972	19553.96	120.8928	19552.17
19564.04	121.148	19561.12	121.1461	19563.22	121.7106	19550.86	121.151	19553.56	121.1508	19553.38	121.149	19552.84

19563.48	121.3914	19563.37	121.3925	19563.19	121.9544	19550.79	121.3951	19552.21	121.3998	19552.76	121.3954	19553.51
19562.1	121.6438	19562.53	121.6457	19563.42	122.2038	19553.89	121.6473	19552.5	121.65	19555.51	121.6482	19555.22
19561.25	121.8944	19562.59	121.9124	19563.18	122.4544	19551.66	121.8931	19552.81	121.8973	19556.74	121.8931	19552.69
19564.5	122.1454	19563.02	122.144	19562.87	122.7126	19553.25	122.1526	19552.49	122.152	19554.17	122.1483	19552.14
19560.27	122.3928	19562.21	122.3919	19562.76	122.9538	19551.98	122.3934	19554.15	122.411	19553.12	122.3953	19554.48
19562.02	122.6457	19561.22	122.6461	19564.46	123.2047	19552.51	122.6474	19549.94	122.6525	19555.19	122.6475	19554.65
19561.72	122.895	19563.72	122.8978	19560.88	123.4561	19551.46	122.8941	19553.51	122.8969	19556.12	122.894	19551.53
19563.3	123.1432	19561.79	123.1447	19564.4	123.7153	19553.5	123.157	19553.9	123.154	19554.29	123.1473	19552.64
19562.87	123.3921	19562.24	123.3928	19560.83	123.9541	19552.15	123.3934	19551.54	123.4007	19553.05	123.4077	19554.08
19563.08	123.6461	19562.19	123.6475	19561.18	124.2058	19552.96	123.6472	19554.69	123.6532	19554.17	123.6492	19551.97
19563.33	123.8991	19563.69	123.8968	19561.78	124.4558	19550.5	123.8944	19552.34	123.8981	19551.91	123.8927	19554.5
19562.9	124.1451	19562.11	124.1486	19563.34	124.7166	19550.7	124.1516	19550.52	124.153	19552.78	124.1473	19552.06
19560.68	124.3919	19559.37	124.3919	19564.53	124.9631	19551.51	124.3938	19551.34	124.4002	19552.42	124.3955	19553.41
19562.31	124.6455	19562.1	124.6459	19562.59	125.205	19551.87	124.6476	19551.1	124.6532	19555.68	124.6494	19553.48
19563.32	124.8958	19561.43	124.8952	19561.82	125.4541	19551.86	124.8967	19553.48	124.899	19551.73	124.8938	19553.66
19562.76	125.1463	19563.61	125.1494	19562.3	125.7123	19552.48	125.1532	19552.51	125.1531	19551.79	125.1458	19552.31
19562.31	125.3926	19561.4	125.4057	19563.91	125.9559	19553.27	125.3952	19553.49	125.3983	19550.16	125.3954	19552.97
19561.86	125.6465	19562.46	125.6538	19562.39	126.2067	19552.24	125.6487	19553.29	125.6537	19554.09	125.6493	19552.48
19562.63	125.8966	19562.61	125.8937	19559.88	126.4544	19553.27	125.8964	19552.08	125.9002	19552.62	125.8943	19552.9
19560.59	126.1469	19561.46	126.1475	19562.72	126.7137	19551.95	126.1545	19552.04	126.1541	19552.62	126.1479	19554.14
19562.07	126.3938	19562.95	126.3946	19562.53	126.9559	19551.53	126.3955	19552.52	126.4157	19551.48	126.3979	19551.75
19560.74	126.6454	19561.42	126.6488	19562.54	127.2068	19551.17	126.6479	19551.96	126.6529	19552.13	126.6501	19551.46
19562.06	126.8985	19561.9	126.895	19561.52	127.4559	19550.34	126.8949	19551.61	126.9001	19554.58	126.8991	19552.06
19561.69	127.1464	19560.45	127.1478	19560.34	127.717	19550.2	127.156	19552.81	127.1545	19551.94	127.1483	19553.08
19562.78	127.3946	19562.01	127.395	19562.54	127.9566	19552.54	127.3974	19553.08	127.4026	19550.68	127.3966	19553.75
19563.04	127.6448	19561.91	127.6493	19561.4	128.2088	19552.44	127.649	19552.76	127.6542	19551.84	127.6503	19551.61
19562.66	127.8969	19561.47	127.8965	19560.67	128.457	19553.31	127.8961	19551.89	127.8996	19552.5	127.896	19552.71
19561.27	128.1461	19562.97	128.1492	19564.4	128.7164	19551.49	128.1539	19551.26	128.1529	19552.58	128.1484	19551.49
19561.95	128.3964	19559.4	128.3976	19562.85	128.9557	19550.58	128.3972	19552.77	128.4019	19553.3	128.3981	19550.16
19562.26	128.6473	19563.35	128.6479	19562.1	129.2087	19552.74	128.6511	19551	128.6537	19551.99	128.6502	19554.16
19561.48	128.908	19562.49	128.8979	19563.26	129.4609	19553.18	128.898	19552.26	128.9025	19553.22	128.8972	19553.8
19561.36	129.147	19560.96	129.1474	19562.73	129.7175	19554.04	129.1561	19552.87	129.155	19550.5	129.1512	19554.28
19561.74	129.3952	19560.5	129.3963	19562.05	129.9631	19549.86	129.3976	19553.78	129.401	19552.09	129.3972	19553.03
19560.97	129.6483	19561.04	129.6488	19560.72	130.2077	19552.19	129.6492	19554.57	129.6536	19551.14	129.6492	19551.87
19562.24	129.9005	19561.65	129.8968	19562.09	130.4573	19550.49	129.8985	19549.29	129.9031	19554.61	129.8958	19552.67
19561.64	130.1469	19561.09	130.1501	19562.97	130.7163	19550.37	130.16	19553.39	130.1579	19552.21	130.1514	19552.83
19561.44	130.3971	19561.17	130.4003	19561.7	130.9584	19551.45	130.4082	19550.57	130.4033	19552.49	130.3978	19553.48
19560.83	130.6514	19561.12	130.6532	19560.17	131.2061	19553.82	130.6512	19554.03	130.6539	19553.68	130.6508	19551.61
19560.49	130.9008	19561.46	130.8965	19561.81	131.4566	19553.83	130.898	19552.47	130.9022	19551.48	130.8987	19552.44
19562.31	131.1483	19561.61	131.1497	19561.07	131.7168	19551.34	131.1558	19551.49	131.1558	19551.81	131.1518	19551.42
19560.71	131.3959	19559.7	131.3987	19563.18	131.9587	19552.48	131.4022	19552.39	131.4041	19552.61	131.3999	19553.08
19561.14	131.6488	19561.69	131.65	19562.11	132.2105	19552.54	131.6517	19551.16	131.6579	19552.61	131.6509	19552.07
19561.52	131.9037	19561.65	131.8965	19560.23	132.4581	19550.99	131.8989	19552.5	131.9011	19552.37	131.8959	19553.32
19559.32	132.1501	19562.59	132.15	19562.44	132.7167	19551.38	132.1711	19551.06	132.155	19553.22	132.152	19554.5
19560.95	132.3972	19561.29	132.3986	19560.71	132.9588	19553.26	132.404	19551.02	132.4051	19550.84	132.399	19551.9
19562.66	132.6496	19561.05	132.6518	19562.36	133.2101	19550.56	132.6515	19552.45	132.6567	19551.3	132.6519	19552.15
19560.98	132.8998	19561.55	132.8979	19562.98	133.4595	19549.82	132.8981	19552.97	132.902	19551.1	132.898	19553.28
19561.98	133.1503	19562.03	133.1497	19560.07	133.7171	19552.31	133.1602	19552.25	133.1545	19552.19	133.1506	19553
19559.28	133.3983	19558.31	133.3966	19561.71	133.9571	19552.04	133.3977	19554.2	133.4035	19551.44	133.4	19549.18
19561.44	133.6491	19563.63	133.6502	19562.92	134.2104	19551.62	133.6525	19550.51	133.6561	19551.41	133.6526	19553.9
19560.73	133.9004	19560.38	133.8987	19562.39	134.4605	19553.39	133.8993	19552.02	133.9055	19553.51	133.8989	19554.37
19561.94	134.1493	19560.93	134.1516	19562.62	134.7166	19553.68	134.1573	19551.83	134.1565	19549.68	134.1532	19552.57
19561.89	134.3971	19561.72	134.3982	19559.74	134.9595	19551.22	134.4099	19553.02	134.4027	19550.99	134.3993	19551.88
19563.48	134.6499	19560.51	134.6563	19562.61	135.2099	19551.22	134.6519	19555.4	134.6568	19551.61	134.6526	19551.89
19560.11	134.9028	19559.44	134.9091	19560.47	135.4588	19551.5	134.8992	19551.49	134.9041	19551.46	134.8978	19551.87
19558.64	135.1501	19561.97	135.153	19560.82	135.7165	19550.15	135.1547	19550.88	135.1608	19554.94	135.153	19552.89
19562.8	135.3998	19559.87	135.3986	19561.81	135.9618	19552.6	135.4003	19551.93	135.405	19550.37	135.4	19551.7
19562.3	135.6497	19561.33	135.6554	19562.1	136.2098	19551.46	135.6526	19553.26	135.6562	19552.37	135.6525	19551.29
19562.93	135.9052	19561.97	135.8994	19562.61	136.4597	19551.51	135.8992	19552.88	135.9044	19552.03	135.8982	19552.32
19561.84	136.1528	19560.8	136.1524	19559.35	136.7173	19549.54	136.1602	19551	136.1578	19552.98	136.1541	19551.47
19561.58	136.3984	19559.59	136.3997	19562.22	136.9607	19551.72	136.4016	19550.81	136.4069	19551.3	136.4024	19552.28
19560.82	136.6496	19562.03	136.6515	19561.43	137.2121	19549.73	136.6542	19550.84	136.6576	19551.42	136.6539	19551.05
19560.72	136.9007	19561.09	136.8979	19560.68	137.4603	19550.45	136.8986	19553.06	136.9038	19552.64	136.8988	19553.21
19560.94	137.1519	19561.23	137.1528	19560.23	137.7207	19553.37	137.1588	19551.08	137.1615	19549.1	137.1552	19552.95
19561.2	137.4087	19561.2	137.4005	19562.85	137.97	19549.52	137.4016	19550.06	137.4063	19551.2	137.4011	19552.87
19561.88	137.6522	19560.34	137.6522	19561.19	138.2122	19550.79	137.6541	19551.92	137.6603	19552.26	137.655	19552.06
19559.33	137.9125	19561.72	137.8986	19561	138.4611	19551.99	137.8989	19551.71	137.9055	19550.54	137.8987	19552.02
19561.91	138.1521	19561.16	138.153	19560.85	138.7189	19551.82	138.1569	19552.85	138.1575	19552.51	138.154	19552.04
19562.24	138.4007	19559.36	138.4007	19559.4	138.9609	19552.04	138.4011	19550.63	138.4053	19551.88	138.4015	19551.91
19561	138.6508	19560.58	138.6533	19560.57	139.2126	19551.39	138.654	19552.72	138.6581	19551.75	138.6544	19550.46

19559.45	138.9031	19560.3	138.9009	19560.17	139.4653	19549.43	138.9017	19554.88	138.9076	19553.37	138.9011	19551.59
19559.43	139.1523	19560.48	139.1538	19564.44	139.7194	19549.6	139.1607	19552.09	139.1578	19552.66	139.1515	19551.84
19561.1	139.399	19560.79	139.3988	19561.36	139.9717	19552.96	139.4009	19550.91	139.4052	19550.65	139.4021	19552.54
19560.9	139.6535	19559.61	139.6537	19561.27	140.2116	19549.88	139.6537	19554.19	139.6592	19551.74	139.6548	19549.55
19560.5	139.9039	19557.87	139.9017	19559.88	140.4624	19551.1	139.9008	19550.98	139.9061	19551.64	139.8996	19553.24
19560.24	140.1535	19559.45	140.1555	19560.66	140.7199	19550.7	140.1605	19551.02	140.1628	19552.48	140.1538	19550.54
19559.4	140.4025	19561.64	140.4054	19561.31	140.9632	19550.24	140.4004	19554.57	140.4092	19549.82	140.4027	19551.14
19561.32	140.6533	19560.13	140.6564	19561.79	141.2135	19549.63	140.6542	19550.36	140.6594	19551.8	140.6549	19552.94
19560.64	140.9034	19561.04	140.9028	19560.84	141.4615	19550.38	140.9108	19551.33	140.9068	19550	140.9009	19550.15
19561.9	141.1529	19562.21	141.1548	19562.13	141.7182	19549.34	141.1634	19550.63	141.1601	19551.16	141.1561	19553.17
19560.78	141.4017	19559.83	141.4006	19561.99	141.9645	19552.19	141.4012	19550.88	141.4076	19552.19	141.4029	19550.54
19561.61	141.6538	19560.65	141.6544	19557.78	142.2158	19551.97	141.6548	19551.03	141.6615	19548.91	141.6552	19552.88
19561.69	141.9149	19562.07	141.9028	19560.83	142.4626	19550.49	141.902	19549.99	141.9063	19551.97	141.9004	19549.86
19560.54	142.1526	19561.85	142.1563	19559.37	142.7189	19550.6	142.161	19551	142.16	19550.76	142.1566	19551.25
19559.98	142.4014	19561.37	142.4013	19562.46	142.9789	19550.47	142.4025	19552.42	142.4084	19550.45	142.4029	19549.79
19560.5	142.6544	19560.22	142.6548	19558.98	143.2148	19550.51	142.6553	19551.69	142.6622	19550.81	142.6555	19550.53
19560.72	142.9071	19560.76	142.9047	19560.79	143.4626	19549.81	142.9033	19550.17	142.9064	19551.28	142.9014	19551.78
19560.15	143.1544	19561.72	143.1542	19561.11	143.7225	19549.82	143.1627	19549.01	143.1733	19552.4	143.1598	19554.36
19559.9	143.402	19560.79	143.4013	19558.6	143.964	19551.06	143.4029	19551.9	143.4084	19552.34	143.4034	19549.54
19561.85	143.6522	19561.02	143.6553	19561.38	144.2143	19547.21	143.6557	19549.33	143.6627	19551.95	143.6575	19553.07
19558.63	143.907	19560.54	143.9043	19561.44	144.4629	19551.81	143.9023	19551.01	143.9078	19551.1	143.9016	19550.54
19559.57	144.1547	19561.03	144.1554	19561.79	144.7231	19549.16	144.1608	19552.49	144.1618	19551.07	144.1571	19551.62
19563.31	144.4024	19561.06	144.4023	19560.62	144.9726	19551.8	144.4042	19551.55	144.4091	19551.2	144.4029	19549.54
19560.6	144.6527	19558.67	144.6558	19561.49	145.2122	19552.42	144.6553	19551.21	144.6627	19550.95	144.6568	19551
19558.23	144.9067	19560.14	144.9032	19561.93	145.463	19551.15	144.903	19552.82	144.9101	19551.05	144.9026	19551.93
19559.92	145.1549	19561.71	145.1568	19558.67	145.7196	19551.38	145.1629	19552.14	145.1637	19549.56	145.1564	19550.9
19560.35	145.4053	19560.37	145.4033	19560.42	145.9655	19550.93	145.4049	19549.56	145.4223	19550.38	145.4036	19550.16
19562.07	145.6557	19561.24	145.6581	19558.02	146.2157	19550.63	145.6575	19551.81	145.664	19551	145.6548	19551.41
19559.99	145.9077	19559	145.9033	19561.09	146.4628	19551.81	145.9039	19550.43	145.907	19551.2	145.9027	19551.11
19560.79	146.157	19559.2	146.1566	19559.71	146.7209	19548.19	146.1702	19549.05	146.1607	19552.28	146.1581	19551.05
19561.85	146.4042	19559.34	146.4036	19556.58	146.9658	19549.55	146.4042	19551.08	146.41	19549.91	146.4076	19552.04
19562.3	146.6553	19556.97	146.6549	19561.33	147.2366	19550.34	146.6573	19550.18	146.6621	19549.76	146.6576	19551.26
19559.57	146.9091	19560.26	146.9047	19561.26	147.4648	19549.87	146.9046	19547.97	146.9069	19549.04	146.9029	19551.32
19559.16	147.1539	19558.51	147.1574	19558.74	147.7229	19550.86	147.162	19548.46	147.1605	19548.92	147.1581	19552.15
19559.03	147.4031	19558.62	147.4041	19560.46	147.9662	19550.36	147.4064	19549.93	147.4108	19550.16	147.4079	19551.23
19560.53	147.6567	19561.59	147.6571	19561.04	148.2169	19549.98	147.6578	19549.51	147.6618	19549.08	147.6584	19551.38
19559.67	147.9107	19562.05	147.9044	19561.89	148.4662	19550.53	147.9036	19552.17	147.9093	19552.59	147.9032	19552.56
19560.27	148.1551	19560.76	148.1565	19559.16	148.723	19548.96	148.1627	19550.54	148.1635	19548.79	148.1567	19551.59
19559.35	148.4023	19561.13	148.403	19559.38	148.966	19549.17	148.4073	19549.25	148.4232	19551.56	148.4056	19550.83
19559.44	148.657	19559.27	148.6568	19560.26	149.2175	19551.59	148.6573	19552.87	148.6637	19548.4	148.6575	19551.36
19561.37	148.9063	19559.53	148.9098	19561.75	149.465	19550.02	148.9052	19551.45	148.909	19553.76	148.9055	19551.67
19560.6	149.1557	19561.12	149.1581	19561.45	149.7214	19550.62	149.1639	19551.5	149.1634	19550.4	149.1685	19549.33
19559.66	149.4042	19561.26	149.4041	19559.55	149.968	19549.91	149.4055	19550.94	149.4126	19550.35	149.4055	19550.63
19559.96	149.656	19558.77	149.6566	19561.66	150.2161	19550.43	149.6572	19549.38	149.6632	19551.36	149.6577	19549.8
19560.42	149.9205	19560.98	149.9067	19560.02	150.4677	19550.86	149.905	19550.11	149.9075	19550.82	149.9041	19549
19558.26	150.1569	19559.34	150.1627	19560.45	150.727	19549.32	150.1665	19550.67	150.1641	19549.29	150.1583	19551.36
19559.92	150.4076	19559.1	150.4077	19560.71	150.9704	19548.93	150.41	19552.33	150.4168	19551.58	150.4092	19550.8
19561.01	150.6603	19560.24	150.6644	19559.42	151.2217	19550.11	150.6628	19549.81	150.6661	19549.65	150.6599	19549.75
19559.97	150.9134	19560.36	150.9084	19560.12	151.4675	19552.14	150.9088	19550.65	150.9136	19550.02	150.9095	19550.43
19561.55	151.1598	19560.09	151.1612	19560.93	151.7267	19549.62	151.1688	19550.37	151.1691	19549.4	151.1624	19549.87
19558.56	151.4065	19560.38	151.4087	19561.94	151.9712	19548.96	151.4105	19550.52	151.4154	19549.36	151.414	19551.46
19559.46	151.6589	19559.23	151.6607	19559.59	152.2228	19549.89	151.6643	19551.27	151.6676	19551.74	151.6662	19549.07
19560.32	151.9135	19558.59	151.9138	19559.73	152.4685	19547.83	151.9103	19549.42	151.9136	19551.37	151.9079	19550.1
19559.17	152.161	19559.55	152.1616	19559.9	152.7285	19550.27	152.1721	19548.33	152.1694	19551.05	152.1627	19551.2
19558.51	152.4107	19559.45	152.4089	19560.6	152.9719	19548.98	152.4124	19550.5	152.417	19549.3	152.4114	19549.7
19557.8	152.6603	19559.64	152.6623	19561.04	153.2237	19549.17	152.6636	19550.28	152.6687	19548.57	152.6625	19551.37
19560.71	152.914	19560.86	152.911	19561.44	153.4718	19549.99	152.9088	19550.36	152.9144	19550.23	152.9084	19550
19558.05	153.1596	19560.75	153.1627	19559.5	153.7271	19549.83	153.172	19551.12	153.1661	19551.28	153.1628	19550.02
19559.51	153.4104	19559.21	153.4101	19557.84	153.9721	19550.56	153.4107	19549.8	153.4185	19550.41	153.4116	19551.81
19560.73	153.6617	19559.86	153.6651	19560.17	154.223	19549.67	153.6642	19550.3	153.668	19549.78	153.6639	19550.77
19559.39	153.9247	19560.14	153.928	19561.92	154.4703	19550.48	153.9084	19548.64	153.9137	19549.1	153.9115	19551.9
19559.32	154.1634	19558.54	154.1617	19560.08	154.7257	19551.16	154.1701	19549.78	154.1695	19547.72	154.1637	19550.67
19559.89	154.4106	19558.86	154.4106	19558.47	154.9719	19548.63	154.4117	19550.04	154.4164	19549.54	154.4116	19550.77
19558.86	154.6617	19558.76	154.6631	19560.19	155.2231	19552.37	154.6649	19549.31	154.6704	19549.46	154.6632	19550.79
19560.26	154.9154	19558.42	154.9112	19559.34	155.4712	19549.39	154.9094	19549.35	154.9146	19550.33	154.912	19550.59
19561.73	155.1611	19561.43	155.1641	19561.05	155.7299	19550.78	155.1673	19551.87	155.1718	19550.06	155.1646	19551.46
19558.8	155.41	19560.57	155.4109	19561	155.9717	19548.12	155.4107	19547.23	155.4344	19550.09	155.4127	19552.47
19556.03	155.6618	19555.21	155.6681	19559.77	156.2226	19549.21	155.664	19551.33	155.6714	19549.38	155.664	19551.75
19558.72	155.917	19558.49	155.9114	19559	156.4709	19550.39	155.9118	19550.12	155.9167	19549.32	155.912	19550.05
19559.15	156.1624	19559.35	156.1638	19560.16	156.7289	19549.03	156.1693	19549.67	156.1703	19551.11	156.1654	19550.26

19559.35	156.4112	19558.01	156.4117	19559.37	156.9727	19550.08	156.4128	19549.67	156.4197	19549.51	156.4146	19550.63
19558.73	156.6633	19558.89	156.6641	19561.17	157.2266	19549.31	156.6637	19550.47	156.6674	19549.64	156.6644	19550.34
19561.1	156.9169	19559.15	156.9128	19555.94	157.4717	19548.72	156.9109	19550.83	156.9157	19550.35	156.9124	19550.72
19560.59	157.1629	19559.01	157.1639	19557.74	157.7282	19551.12	157.1728	19551.82	157.1704	19549.81	157.1656	19549.03
19561.07	157.4121	19559.62	157.4132	19560.13	157.9734	19549.55	157.4128	19551.91	157.4209	19548.28	157.4124	19549.71
19559.06	157.664	19560.42	157.6649	19558.85	158.2234	19548.66	157.6674	19548.99	157.6696	19548.49	157.6643	19551.08
19557.79	157.917	19559.68	157.9138	19559.85	158.4716	19551.44	157.9099	19550.41	157.9162	19548.05	157.9123	19549.1
19558.3	158.1619	19558.75	158.1633	19560.3	158.7308	19549.63	158.1722	19550.53	158.1707	19550.43	158.1638	19548.93
19560.57	158.4127	19560.08	158.4121	19557.85	158.9718	19548.96	158.4137	19552.12	158.4216	19549.84	158.414	19550.02
19559.45	158.6661	19554.08	158.6646	19558.91	159.2237	19547.65	158.6671	19550.8	158.6711	19548.19	158.6657	19554.34
19559.02	158.9164	19555.23	158.9135	19558.61	159.4732	19548.72	158.9124	19551.39	158.9184	19549.74	158.9138	19550.95
19557.29	159.1633	19556.56	159.1636	19560.15	159.7302	19550.2	159.1722	19547.21	159.1696	19550.72	159.1652	19550.35
19559.29	159.4156	19558.52	159.4131	19560.27	159.983	19547.91	159.4129	19548.59	159.4211	19550.67	159.4154	19551.31
19558.68	159.6636	19558.77	159.6664	19559.4	160.2241	19549.86	159.666	19549.8	159.6697	19549.61	159.6658	19551.69
19559.5	159.9163	19559.76	159.9138	19559.98	160.4726	19550.55	159.9135	19548.16	159.9195	19550.57	159.9136	19549.15
19557.77	160.1628	19557.71	160.1648	19559.94	160.7273	19549.77	160.1725	19548.67	160.1721	19548.89	160.1674	19550.54
19557.85	160.4121	19559.99	160.4149	19561.34	160.9697	19550.48	160.415	19549.55	160.4309	19548.59	160.4135	19548.93
19558.29	160.6601	19556.29	160.6649	19560.18	161.2216	19548.2	160.663	19548.97	160.6705	19549.64	160.6634	19550.16
19561.12	160.9142	19557.73	160.9094	19559.93	161.4705	19549.29	160.9096	19549.07	160.9144	19548.24	160.9103	19549.72
19555.16	161.1614	19560.44	161.1617	19558.49	161.7257	19548.96	161.1708	19551.24	161.1681	19548.73	161.1644	19549.65
19559.97	161.41	19558.49	161.4118	19560.12	161.9706	19549.26	161.4125	19548.97	161.421	19550.46	161.4119	19548.6
19561.19	161.6602	19558.53	161.6626	19557.42	162.2235	19548.14	161.6664	19549.23	161.6709	19550.17	161.6628	19551.78
19560.41	161.9138	19558.94	161.9108	19560.45	162.4714	19548.56	161.9107	19549.18	161.9155	19548.32	161.9107	19547.14
19559.99	162.1618	19558.68	162.1631	19559.7	162.726	19550.92	162.1689	19547.23	162.1698	19547.11	162.1616	19552.51
19559.93	162.4095	19557.95	162.4113	19557.69	162.9701	19548.79	162.4134	19550.86	162.42	19549.73	162.4122	19548.51
19557.71	162.6624	19557.22	162.6623	19561.25	163.2213	19548.78	162.664	19549.41	162.6686	19550.13	162.6637	19549.99
19559.06	162.9149	19558.65	162.9114	19559.16	163.4715	19549.51	162.912	19550.17	162.9162	19549.57	162.9108	19551.79
19557.86	163.1631	19559.25	163.1641	19558.65	163.7307	19550.27	163.1702	19549.35	163.1697	19548.67	163.1626	19552.88
19560	163.4099	19561.12	163.4136	19558.28	163.9733	19550.15	163.4249	19550.02	163.42	19550.08	163.4123	19550.3
19559.31	163.6626	19558.66	163.663	19559.39	164.2216	19548.26	163.6637	19550.19	163.6693	19548.94	163.6636	19551.25
19558.08	163.9219	19561.48	163.9116	19558.13	164.4712	19548.33	163.9111	19550.68	163.9171	19550.66	163.9103	19548.93
19559.16	164.1624	19558.61	164.1629	19558.61	164.7295	19549.27	164.1685	19548.37	164.1698	19550.9	164.1642	19550.01
19557.86	164.4108	19559.5	164.415	19556.64	164.9725	19547.79	164.4137	19552.15	164.4212	19549.3	164.4122	19549.13
19559.47	164.6629	19559.19	164.6635	19559.9	165.2229	19549.69	164.664	19549.84	164.671	19550.51	164.664	19547.87
19557.16	164.9143	19559.11	164.913	19559.36	165.4709	19548.92	164.9107	19549.04	164.9175	19548.31	164.9114	19550.16
19558.01	165.1625	19559.43	165.1632	19559.43	165.7279	19547.33	165.1695	19548.67	165.1715	19548.19	165.1652	19549.47
19556.51	165.4104	19558.48	165.4137	19557.88	165.9721	19548.89	165.4132	19548.84	165.4203	19549.38	165.4246	19549.97
19559.86	165.6633	19557.4	165.6673	19558.6	166.226	19546.88	165.6641	19547.96	165.6709	19550.36	165.6657	19550.94
19557.5	165.9161	19558.55	165.9132	19558.88	166.4722	19546.85	165.9131	19550.7	165.9181	19549.68	165.9115	19550.61
19558.55	166.1633	19556.78	166.1645	19557.81	166.7279	19547.46	166.1702	19550.93	166.1715	19548.77	166.1655	19548.23
19558.91	166.4094	19558.27	166.4137	19557.85	166.9723	19549.7	166.4151	19548.75	166.422	19549.08	166.4149	19549.3
19557.28	166.6626	19561.09	166.6649	19561.45	167.2253	19549.62	166.6665	19549.31	166.6711	19547.47	166.6658	19548.71
19556.38	166.9169	19559.65	166.9136	19559.27	167.473	19548.47	166.912	19548.48	166.9188	19549.78	166.9124	19549.6
19558.97	167.163	19556.92	167.1663	19557.05	167.7302	19548.76	167.1703	19546.89	167.1704	19549.03	167.1656	19548.12
19558.22	167.4108	19558.77	167.4136	19561.18	167.9768	19549.32	167.4157	19548.12	167.4202	19549.06	167.4152	19549.62
19556.55	167.662	19558.72	167.6664	19559.59	168.2268	19550.26	167.6657	19549.41	167.6703	19547.46	167.6658	19549.69
19558.57	167.916	19559.58	167.9131	19558.81	168.4722	19546.98	167.9123	19548.59	167.9178	19550.89	167.9133	19550.51
19559.57	168.1643	19558.08	168.1671	19560.08	168.7331	19550.28	168.1735	19547.98	168.1726	19546.9	168.1683	19549.54
19558.52	168.4129	19556.63	168.4141	19555.33	168.9755	19547.82	168.4158	19549.61	168.4305	19546.25	168.4144	19549.67
19558.83	168.6647	19557.19	168.6655	19557.46	169.2285	19547.1	168.6666	19551.34	168.6736	19547.42	168.666	19549.4
19558.42	168.9179	19555.3	168.9194	19560	169.4754	19550.11	168.9151	19548.86	168.918	19549.49	168.9127	19551.6
19558.32	169.1634	19557.32	169.1664	19559.42	169.7301	19549.37	169.1716	19551.27	169.1741	19548.51	169.1676	19548.86
19556.93	169.4132	19557.28	169.413	19558.87	169.9748	19549.08	169.4157	19548.63	169.4212	19547.96	169.4269	19550.26
19557.81	169.6643	19558.93	169.6662	19557.36	170.2298	19548.99	169.6669	19549.87	169.6724	19550.53	169.6686	19549.41
19559.97	169.9166	19559.07	169.9157	19559.88	170.4772	19550.03	169.9141	19550.45	169.9178	19547.36	169.9125	19548.83
19557.22	170.1629	19558.12	170.1672	19556.76	170.7299	19548.21	170.1713	19547.71	170.1743	19550.46	170.1757	19548.53
19558.57	170.4121	19557.55	170.4151	19559.37	170.9747	19548.71	170.4169	19548.1	170.4245	19548.66	170.4166	19550
19560.04	170.6654	19559.19	170.6701	19558.61	171.2289	19549.42	170.6673	19548.78	170.6734	19550.87	170.6668	19550.69
19559.06	170.9181	19559.55	170.9161	19558.3	171.4748	19547.01	170.9134	19547.2	170.9181	19548.79	170.9133	19550.85
19557.86	171.1658	19557.65	171.1659	19559.48	171.7319	19548.57	171.1723	19549.71	171.1719	19549.1	171.1678	19549.38
19559.41	171.4114	19556.96	171.4143	19560.72	171.9753	19551.46	171.4168	19547.82	171.4243	19550.83	171.4167	19548.94
19558.64	171.6668	19558.8	171.6679	19557.14	172.2291	19546.3	171.6673	19549.57	171.674	19549.84	171.6667	19549.12
19558.8	171.9205	19557.22	171.9229	19559.85	172.4745	19545.81	171.9139	19549.03	171.9186	19547.28	171.9144	19548.23
19557.14	172.1647	19558.1	172.1713	19556.3	172.7303	19548.17	172.174	19549.57	172.1764	19549.1	172.1686	19548.06
19558.02	172.4144	19557.25	172.4163	19558.86	172.9755	19549.74	172.4177	19549.4	172.4232	19547.79	172.4189	19549.14
19559.84	172.6673	19559.12	172.6682	19560.57	173.2281	19548.9	172.6698	19547.37	172.6735	19548.98	172.6674	19549.24
19557.34	172.9204	19557.96	172.9179	19559.49	173.4753	19548.28	172.9154	19547.97	172.9192	19548.45	172.9138	19548.86
19559.42	173.1639	19558.51	173.169	19558.49	173.7333	19550.05	173.1753	19546.29	173.1734	19549.31	173.1697	19549.04
19559.39	173.4155	19558.46	173.4144	19560.16	173.9757	19547.69	173.4185	19547.39	173.4245	19548.61	173.4184	19549.5
19556.02	173.6679	19557.53	173.6707	19557.61	174.2275	19547.6	173.6717	19549.66	173.6735	19545.95	173.6697	19551.05

19560.17	173.9212	19556.91	173.9226	19556.68	174.4759	19548.24	173.9163	19549.09	173.9191	19547.48	173.9149	19551.77
19557.67	174.1649	19554.61	174.1709	19559.53	174.7308	19549.99	174.1742	19548.09	174.1741	19546.68	174.1698	19549.14
19557.92	174.4145	19557.73	174.4157	19558.17	174.9765	19547.6	174.4207	19549.5	174.4264	19550.49	174.4176	19547.6
19554.17	174.6672	19558.2	174.6686	19558.33	175.2291	19548.94	174.6694	19549.26	174.6759	19547.03	174.6692	19549.46
19558.56	174.9204	19556.52	174.9175	19558.75	175.4774	19547.95	174.9165	19547.1	174.919	19549.27	174.9144	19549.76
19559.89	175.166	19558.03	175.1702	19556.44	175.7324	19547.69	175.1735	19548.15	175.1748	19548.97	175.1688	19550.68
19557.21	175.4167	19558.05	175.4149	19556.16	175.9789	19549.84	175.4171	19548.12	175.4271	19547.9	175.4195	19551.19
19555.76	175.6664	19560.7	175.6709	19559.37	176.2294	19548.63	175.6694	19548.94	175.6761	19548.52	175.6689	19548.15
19556.8	175.9205	19556.38	175.9181	19555.91	176.4773	19548.11	175.9155	19548.1	175.9218	19549.81	175.9155	19549.73
19555.72	176.1682	19556.32	176.168	19557.82	176.7318	19548.28	176.1743	19548.91	176.175	19548.17	176.1716	19548.95
19558.71	176.4152	19557.64	176.4162	19558.59	176.9915	19546.5	176.4166	19549.57	176.4329	19548	176.4207	19550.2
19558.79	176.6697	19556.11	176.6711	19555.51	177.2297	19547.12	176.67	19548.25	176.6765	19547.8	176.6695	19549.81
19556.57	176.921	19557.44	176.9177	19558.73	177.4782	19548.19	176.9163	19548.49	176.9213	19546.69	176.9175	19550.58
19558.84	177.1672	19556.49	177.1732	19557.39	177.7334	19547.75	177.1737	19550.23	177.1759	19550.64	177.1722	19548.84
19557.62	177.4164	19555.63	177.4171	19559.46	177.9779	19548.23	177.4182	19547.06	177.425	19546.96	177.4197	19550.84
19557.42	177.6693	19557.76	177.6722	19560.28	178.2288	19548.44	177.6722	19546.95	177.675	19549.43	177.6701	19549.29
19555.52	177.9204	19556.25	177.9193	19558.39	178.4791	19549.43	177.9167	19550.01	177.9203	19548.52	177.9158	19549.88
19555.56	178.1669	19554.24	178.1697	19555.85	178.7379	19548.75	178.177	19551.16	178.1748	19548.08	178.1725	19549.17
19559.35	178.4165	19558.1	178.4184	19559.74	178.9792	19547.7	178.4189	19550.09	178.4273	19548.02	178.4221	19550.02
19559.7	178.6815	19557.96	178.6716	19557.25	179.2299	19548.82	178.6727	19549.47	178.6752	19549.26	178.6701	19547.78
19558.76	178.9228	19557.01	178.9312	19558.85	179.4825	19546.84	178.9173	19549.22	178.922	19549.31	178.9189	19549.77
19559.2	179.1676	19557.94	179.1721	19560.48	179.7323	19549.09	179.1753	19550.5	179.1774	19549.76	179.1731	19548.34
19557.77	179.4161	19557.27	179.419	19559.48	179.9779	19547.29	179.4182	19549.26	179.4279	19548.28	179.4215	19548.01
19558.36	179.6717	19558.42	179.6726	19558.71	180.2301	19546.54	179.6723	19548.26	179.676	19549.19	179.6709	19549.11
19557.76	179.9235	19555.74	179.9196	19558.62	180.4798	19547.53	179.9181	19546.86	179.9231	19549.26	179.9165	19548.88
19556.23	180.169	19558.31	180.1697	19560.38	180.7353	19547.46	180.176	19549.1	180.1795	19547.84	180.1725	19549.88
19559.31	180.4166	19557.7	180.4185	19558.77	180.9801	19547.23	180.4199	19548.04	180.4371	19549.35	180.4209	19549.38
19559.5	180.671	19558.09	180.6757	19558.18	181.2306	19547.55	180.6729	19547.88	180.6784	19551.08	180.6719	19545.47
19557.26	180.9257	19557	180.9215	19558.22	181.4815	19545.9	180.9189	19548.23	180.9229	19547.04	180.9182	19549.16
19559.75	181.1698	19558.43	181.1714	19557.38	181.7353	19546.65	181.1776	19548.07	181.1778	19546.76	181.1726	19551.24
19558.93	181.4169	19554.69	181.4194	19556.05	181.9944	19548.44	181.42	19548.8	181.4281	19548.68	181.4228	19548.83
19557.98	181.6729	19558.29	181.6732	19556.93	182.2311	19549.27	181.6734	19548.65	181.6768	19547.85	181.6709	19550.16
19556.07	181.9273	19556.63	181.921	19557.39	182.4805	19548.45	181.9186	19548.01	181.9233	19549.66	181.9203	19549.69
19558.25	182.1696	19558.45	182.1727	19559.39	182.7339	19550.48	182.1779	19549.96	182.1774	19548.66	182.1728	19549.22
19558.73	182.4185	19560.04	182.4188	19553.07	182.9802	19548.34	182.4199	19547.33	182.4294	19548.63	182.4345	19546.46
19554.42	182.674	19556.82	182.6744	19558.53	183.2315	19549.02	182.6737	19547.85	182.6778	19545.94	182.6718	19548.94
19556.57	182.9236	19557.23	182.9223	19557.76	183.4798	19547.04	182.9198	19548.07	182.9239	19550.22	182.9185	19547.75
19555.46	183.1683	19556.48	183.1715	19556.73	183.7372	19545.12	183.1764	19547.01	183.1786	19548.84	183.1739	19547.61
19556.88	183.4204	19555.59	183.42	19556.99	183.9811	19548.06	183.4221	19551.3	183.4285	19548.16	183.424	19548.73
19556.71	183.6726	19557.88	183.6749	19558.21	184.2331	19547.89	183.6742	19547.31	183.6771	19549.14	183.6731	19550.28
19557.85	183.9278	19556.75	183.9241	19560.06	184.4804	19547.48	183.9187	19548.33	183.9245	19549.33	183.918	19550.54
19557.25	184.169	19559.76	184.1724	19557.7	184.7347	19550.35	184.177	19547.78	184.1792	19548.16	184.1761	19548.4
19558.87	184.4171	19557.7	184.4185	19557.01	184.9804	19547.65	184.4218	19546.36	184.4287	19549.5	184.4229	19547.53
19557.46	184.6718	19555.93	184.673	19559.05	185.2336	19547.36	184.6745	19549.73	184.6788	19546.64	184.6745	19548.18
19557.72	184.9257	19556.92	184.923	19557.44	185.4817	19547.85	184.9215	19545.78	184.9255	19548.44	184.9198	19549.35
19555.28	185.1706	19554.7	185.173	19556.83	185.7378	19546.62	185.179	19549.14	185.1793	19548.29	185.1749	19549.42
19557.78	185.4207	19558.21	185.419	19558.23	185.9819	19545.04	185.4211	19548.95	185.4287	19549.76	185.426	19548.2
19558.05	185.6753	19557.79	185.6771	19557.81	186.235	19546.68	185.6746	19547.3	185.6786	19548.65	185.6731	19547.61
19557.5	185.9279	19556.9	185.9236	19560.04	186.4814	19546.72	185.9209	19547.42	185.9252	19548.31	185.9202	19548.83
19557.08	186.1725	19557.51	186.1745	19557.51	186.7359	19548.91	186.1767	19546.09	186.1828	19547.71	186.1751	19548.5
19557.74	186.4193	19558.47	186.4202	19558.94	186.982	19547.79	186.4228	19547.25	186.4314	19549.4	186.4258	19549.12
19557.53	186.6728	19557.04	186.6748	19557.48	187.2341	19549.05	186.6764	19547.98	186.6793	19547.16	186.6732	19549.71
19557.69	186.9285	19555.27	186.9243	19557.96	187.4816	19550.17	186.9211	19546.55	186.927	19547.4	186.9198	19547.62
19556.54	187.171	19555.27	187.1731	19557.7	187.7369	19548.54	187.1796	19545.28	187.1804	19548.46	187.1761	19548.4
19557.35	187.4197	19557.64	187.4209	19557.13	187.9828	19546.04	187.4232	19549.37	187.4316	19549.11	187.4276	19548.47
19558.68	187.6728	19559.31	187.6746	19556.95	188.2337	19547.61	187.6747	19548.19	187.6801	19548.47	187.6736	19550.35
19557.21	187.9254	19557.58	187.9228	19556.55	188.4825	19544.88	187.9214	19547.39	187.9266	19547.8	187.9202	19545.81
19557.58	188.1715	19558.31	188.1751	19557.57	188.7394	19547.83	188.1806	19548.91	188.1808	19548.06	188.1745	19551.09
19556.79	188.4219	19555.89	188.4213	19558.76	188.9831	19549.19	188.4223	19549.91	188.4393	19548.46	188.4259	19548.84
19557.27	188.6725	19555.74	188.6749	19559.56	189.2355	19550.58	188.6756	19547.04	188.683	19549.13	188.6749	19549.11
19557.02	188.9268	19556.53	188.9219	19557.97	189.4841	19547.21	188.9206	19546.44	188.9266	19547.86	188.9224	19550.64
19555.74	189.1713	19557.45	189.1742	19558.57	189.7373	19548.23	189.1787	19548.18	189.1829	19548.47	189.1762	19549.08
19559.2	189.4203	19556.09	189.4222	19558.15	189.983	19545.46	189.4224	19547.07	189.4315	19548.23	189.4259	19548.42
19559.52	189.6733	19555.48	189.6758	19557.88	190.2359	19547.03	189.6753	19545.93	189.6809	19547.92	189.6765	19548.56
19556.4	189.9274	19556.83	189.9256	19557.45	190.4843	19548.83	189.9238	19548.45	189.9276	19546.48	189.9205	19549.21
19559.91	190.1742	19558.36	190.175	19557.2	190.7362	19549.65	190.1763	19548.39	190.1826	19548.25	190.1732	19549.87
19557.41	190.4209	19558.27	190.4221	19557.7	190.9831	19547.96	190.4252	19550.25	190.4334	19549.14	190.4261	19548.42
19558.63	190.6738	19557.24	190.678	19558.41	191.2347	19549.13	190.6768	19548.82	190.6809	19547.93	190.6744	19547.36
19558.54	190.927	19557.63	190.9274	19559.87	191.4839	19548.94	190.9227	19546.75	190.9273	19548.63	190.9237	19550.03
19556.79	191.1739	19557.32	191.1749	19556.84	191.737	19547.84	191.1789	19549.74	191.1797	19546.72	191.1752	19548.6

19558.21	191.4224	19555.76	191.4234	19556.04	191.9839	19548.75	191.4369	19547	191.4354	19550.23	191.4264	19550.9
19555.73	191.6737	19556.16	191.6753	19557.36	192.2356	19545.77	191.6768	19546.48	191.6813	19548.22	191.6767	19549
19557.88	191.9285	19554.2	191.9278	19554.6	192.4858	19546.19	191.9236	19548.01	191.9289	19549.98	191.9231	19547.49
19555.5	192.1736	19558.29	192.1745	19557.32	192.7387	19548.44	192.1819	19547.44	192.1819	19547.59	192.1774	19546.48
19557.13	192.4231	19555.35	192.4242	19555.04	192.9836	19545.49	192.4411	19548.78	192.4318	19547.81	192.428	19548.21
19555.79	192.6755	19558.42	192.6754	19558.02	193.2354	19548.27	192.6834	19551.67	192.6854	19549.89	192.6764	19547.95
19555.25	192.9284	19555.32	192.9378	19556.08	193.4855	19547.5	192.9231	19548.46	192.9295	19547.21	192.9221	19549.23
19555.86	193.1724	19557.2	193.1767	19558.48	193.7414	19547.52	193.1804	19548.65	193.1814	19546.82	193.1777	19547.52
19559.81	193.4238	19558.22	193.4235	19558.85	193.9837	19547.08	193.4259	19547.22	193.4333	19547.32	193.4304	19547.62
19556.34	193.6762	19555.24	193.6781	19557.42	194.2363	19549.43	193.6787	19545.88	193.6804	19549.64	193.6765	19550.46
19555.52	193.9292	19558.2	193.9267	19555.75	194.4853	19548.68	193.9234	19548.5	193.9288	19550.28	193.9231	19547.47
19554.66	194.1745	19554.8	194.1775	19558.2	194.7417	19548.39	194.1809	19548.15	194.1838	19547.72	194.1761	19548.13
19557.22	194.4242	19558.55	194.4242	19559.04	194.9842	19548.24	194.4428	19547.65	194.4354	19547.84	194.4286	19547.57
19556.58	194.6753	19556.75	194.6755	19558.42	195.2371	19549.53	194.6788	19549.13	194.6827	19547.18	194.6779	19546.34
19558.82	194.9297	19555.69	194.9295	19556.92	195.4856	19546.39	194.9262	19546.14	194.9294	19548.25	194.9225	19547.33
19555.8	195.182	19557.61	195.1765	19559.47	195.7395	19547.75	195.1822	19546.23	195.1837	19546.36	195.1763	19549.36
19555.76	195.4226	19556.28	195.4255	19556.08	195.9974	19549.26	195.426	19547.88	195.436	19546.77	195.431	19548.87
19554.71	195.6753	19557.63	195.6795	19556.84	196.2373	19547.58	195.6782	19547.04	195.6836	19546.94	195.6774	19549.13
19556.15	195.9291	19556.28	195.93	19556.82	196.4867	19547.22	195.9254	19548.54	195.9379	19548.58	195.9236	19549.99
19557.01	196.1753	19556.29	196.178	19554.13	196.7416	19550.13	196.1816	19547.5	196.1837	19546.78	196.1901	19547.54
19554.94	196.4228	19559.15	196.4252	19557.31	196.9853	19547.02	196.4262	19549.2	196.436	19546.98	196.4312	19549.04
19556.44	196.6757	19556.29	196.6778	19559.37	197.2372	19545.66	196.6791	19549.93	196.6832	19546.31	196.678	19547.12
19555.26	196.9313	19554.09	196.9289	19555.01	197.4863	19545.74	196.926	19547.83	196.9416	19548.3	196.925	19549.92
19558.42	197.1763	19557.82	197.178	19556.2	197.743	19550.19	197.1806	19549.09	197.1832	19547.21	197.1795	19550.26
19558.88	197.4242	19557.49	197.4253	19557.27	197.9861	19548.25	197.4266	19548.05	197.4353	19546	197.4422	19548.16
19554.89	197.6764	19557.67	197.679	19557.78	198.2384	19545.02	197.6793	19548.47	197.6875	19546.27	197.6796	19549.52
19556.02	197.9305	19559.08	197.9334	19558.14	198.4873	19547.89	197.9251	19548.49	197.9316	19547.29	197.9256	19546.58
19558.16	198.1985	19557.43	198.1867	19557	198.7516	19548.42	198.1912	19547.46	198.1932	19549.4	198.1899	19549.03
19555.84	198.4253	19554.95	198.4257	19555.76	198.987	19544.44	198.4272	19546.03	198.4364	19547.76	198.4325	19548.86
19555.69	198.6774	19555.62	198.6805	19556.47	199.2387	19548.86	198.6796	19548.12	198.6856	19548.34	198.6775	19549.81
19557.98	198.9326	19555.39	198.9317	19557.27	199.4873	19547.08	198.929	19548.15	198.9328	19549.17	198.9246	19549.43
19553.39	199.1765	19559.47	199.1801	19556.9	199.7409	19545.75	199.1824	19548.35	199.1855	19547.92	199.1787	19547.47
19557.02	199.4237	19555.72	199.4275	19557.54	199.9989	19548.86	199.4274	19547.76	199.4406	19546.08	199.4309	19548.55
19554.95	199.6782	19555.9	199.6791	19556.59	200.2406	19546.18	199.6813	19547.75	199.6854	19547.21	199.6799	19548.15
19556.72	199.9322	19559.89	199.9293	19558.75	200.4904	19547.82	199.927	19547.6	199.931	19547.09	199.9255	19549.2
19556.18	200.1776	19558.2	200.1784	19556.79	200.7473	19549.68	200.1944	19547.77	200.1849	19547.7	200.1782	19547.79
19555.04	200.43	19554.93	200.4307	19557.19	200.9915	19547.66	200.4328	19547.65	200.4414	19548.47	200.4355	19546.72
19557.47	200.6814	19554.27	200.6855	19557.73	201.2443	19548.64	200.6869	19545.01	200.6895	19547.07	200.6843	19546.91
19557.13	200.9369	19554.72	200.9356	19555.71	201.4915	19546	200.9334	19548.04	200.9354	19547.49	200.9309	19547.66
19557.19	201.1826	19556.37	201.1847	19556.71	201.7495	19548.24	201.1899	19547.28	201.189	19548.24	201.1857	19545.76
19554.85	201.4309	19556.56	201.4314	19559.08	201.9953	19547.2	201.4371	19546.55	201.4438	19547.67	201.4366	19549.27
19557.43	201.6811	19556.61	201.684	19556.44	202.2444	19549.14	201.6849	19547.49	201.6889	19548.55	201.6828	19548.77
19556.32	201.9381	19554.9	201.9381	19556.58	202.4916	19545.87	201.9326	19547.37	201.9365	19547.88	201.9351	19546.75
19554.81	202.1826	19555.48	202.1844	19558.37	202.7468	19544.21	202.1918	19547.55	202.1903	19549.1	202.1851	19546.79
19556.39	202.4327	19556.04	202.4301	19554.43	202.9941	19546.75	202.4332	19547.52	202.4421	19547.73	202.4383	19549.13
19554.23	202.6835	19556.89	202.6867	19557.24	203.2674	19546.8	202.6862	19546.61	202.6893	19547.82	202.6856	19548.36
19556.24	202.9405	19557.99	202.9361	19559.37	203.4929	19549.52	202.9322	19547.05	202.9357	19548.25	202.9307	19548.56
19557.85	203.187	19558.59	203.1848	19556.93	203.7499	19545.66	203.1905	19546.04	203.1915	19545.8	203.1858	19548.57
19557.81	203.4309	19558.84	203.4322	19557.92	203.9928	19547.29	203.4319	19545.95	203.4557	19546.91	203.4387	19548.18
19556.63	203.6852	19553.51	203.6838	19557.9	204.2453	19548.43	203.6864	19545.31	203.6901	19547.46	203.6871	19547.6
19557.39	203.9394	19557.59	203.9373	19558.17	204.4932	19546.71	203.9361	19546.79	203.9367	19548.92	203.9315	19547.79
19555.14	204.1828	19556.5	204.1848	19556.32	204.7479	19546.68	204.1909	19549.06	204.1899	19549.46	204.1884	19546.35
19557.29	204.4308	19555.28	204.4329	19555.52	204.9937	19547.49	204.4454	19548.22	204.4431	19546.19	204.4375	19547.28
19555.84	204.6857	19556.69	204.6854	19556.95	205.2453	19546.31	204.6852	19546.9	204.6916	19547.71	204.6849	19548.32
19559.13	204.9389	19555.32	204.9384	19554.75	205.4941	19547.84	204.9333	19547.08	204.9386	19548.16	204.9323	19544.92
19560.36	205.1834	19555.3	205.1865	19557.13	205.7491	19547.7	205.1912	19549.47	205.1917	19548.48	205.1871	19546.77
19558.2	205.4319	19557.33	205.4514	19557.93	205.9955	19546.27	205.4336	19548.4	205.4433	19547.04	205.4393	19548.17
19557.47	205.6832	19556.67	205.6933	19556.61	206.2463	19547.98	205.6868	19547.03	205.6916	19549.18	205.686	19546.51
19557.47	205.945	19555.59	205.9475	19556.08	206.494	19545.67	205.9343	19544.37	205.9389	19547.82	205.9326	19547.22
19556.68	206.1921	19555.77	206.1916	19557.93	206.7502	19547.95	206.1909	19550.21	206.1927	19548.83	206.1868	19547.81
19557.81	206.4349	19554.9	206.4363	19557.63	206.9943	19546.11	206.4346	19548.19	206.4443	19547.74	206.441	19548.81
19557.45	206.687	19556.04	206.6868	19556.7	207.2469	19547.05	206.6864	19547.64	206.6924	19546.63	206.6861	19546.7
19555.19	206.9402	19556.81	206.9423	19556.73	207.4941	19546.61	206.9347	19546.32	206.94	19544.53	206.9324	19548.87
19555.4	207.1862	19558.95	207.1904	19556.92	207.7498	19547.94	207.1907	19546.58	207.1936	19546.81	207.1872	19548.43
19559.09	207.4333	19556.24	207.4413	19554.9	207.9966	19548.54	207.4343	19548.47	207.4461	19548.24	207.4391	19548.05
19557.79	207.6854	19559.56	207.6935	19555.86	208.2465	19547.5	207.6886	19549.2	207.6927	19550.04	207.6864	19548.33
19556.52	207.9402	19558.06	207.9437	19555.81	208.4939	19549	207.9348	19546.76	207.9398	19546	207.9349	19547.7
19556.15	208.186	19556.48	208.1911	19554.74	208.7526	19544.87	208.1926	19548.65	208.1933	19547.54	208.1865	19548.06
19557.48	208.4331	19556.68	208.4392	19555.59	208.9971	19546.9	208.4365	19548.76	208.4457	19547.86	208.4421	19548.07
19557.44	208.6858	19557.28	208.7187	19557.07	209.2473	19548.48	208.6896	19547.22	208.6934	19546.77	208.6888	19547.69

19556.64	208.9382	19555.43	208.9695	19554.78	209.4957	19546.1	208.937	19545.43	208.9395	19547.26	208.9339	19548.75
19555.6	209.1859	19556.31	209.2048	19555.57	209.7537	19548.11	209.1916	19547.45	209.1931	19546.52	209.203	19549.37
19558.22	209.4351	19552.9	209.439	19556.2	210.0118	19546.82	209.4371	19547.65	209.4623	19546.45	209.4548	19548.19
19555.13	209.684	19554.22	209.6901	19554.14	210.2498	19546.32	209.6884	19550.07	209.6982	19547.36	209.6909	19548.41
19556.31	209.939	19557.71	209.9434	19556.23	210.4969	19547.36	209.9377	19547.91	209.9439	19544.89	209.9342	19547.82
19557.21	210.1862	19555.16	210.1933	19557.06	210.7537	19546.53	210.1939	19547.19	210.1944	19547.77	210.1877	19547.37
19554.64	210.4355	19555.99	210.4373	19555.08	210.9978	19545.94	210.4368	19548.72	210.4475	19545.6	210.4389	19548.62
19555.6	210.6872	19554.93	210.6946	19555.05	211.2513	19546.88	210.689	19549.19	210.6942	19547.35	210.6894	19547.75
19555.33	210.9406	19556.19	210.9478	19557.55	211.4969	19544.72	210.937	19547.73	210.9411	19547.39	210.9384	19550.12
19557.46	211.1852	19555.61	211.1901	19556.35	211.7538	19546.42	211.194	19549.16	211.1945	19548.2	211.1893	19545.37
19555.77	211.4353	19557.39	211.4372	19557.19	211.9965	19546.5	211.4378	19547.29	211.4472	19546.1	211.4419	19547.94
19556.85	211.6889	19557.33	211.6887	19556.69	212.2497	19548.61	211.6904	19547.69	211.6938	19548.6	211.6883	19548.45
19557.73	211.944	19555.22	211.9396	19556.88	212.4967	19546.65	211.9388	19546.99	211.9404	19546.48	211.936	19546.82
19557.54	212.187	19554.56	212.1897	19555.16	212.7535	19547.44	212.1932	19547.16	212.1954	19546.7	212.19	19550.37
19557.21	212.4354	19558.01	212.4363	19556.16	212.9968	19547.53	212.4409	19550.22	212.4476	19547.46	212.4426	19547.59
19557.79	212.6864	19556.2	212.6912	19556.38	213.2511	19547.53	212.6911	19546.33	212.6948	19548.52	212.6885	19548.03
19555.25	212.9409	19557.05	212.9423	19555.69	213.4985	19546.35	212.9374	19546.81	212.9409	19546.78	212.9373	19547.99
19554.97	213.1873	19558.3	213.189	19558.15	213.7551	19546.73	213.1948	19549.74	213.1959	19549.56	213.1908	19546.9
19556.12	213.4357	19556.17	213.4377	19555.77	213.9968	19544.34	213.4379	19548.8	213.4482	19546.76	213.4419	19548.98
19558.96	213.688	19557.52	213.6903	19557.8	214.2497	19545.66	213.6934	19546.2	213.6956	19547.86	213.6885	19544.48
19556.88	213.9419	19555.53	213.9439	19557.68	214.4976	19545.1	213.9391	19548.23	213.9413	19548.08	213.9367	19546.31
19553.56	214.191	19557.82	214.1906	19557.17	214.7523	19545.95	214.1961	19547.46	214.1957	19546.04	214.1951	19548.08
19556.62	214.4371	19555.91	214.4367	19555.1	214.9988	19548.41	214.439	19549.1	214.4483	19547.34	214.4465	19548.4
19557.35	214.688	19556.67	214.6899	19555.09	215.2512	19546	214.6895	19543.96	214.6953	19545.78	214.6921	19545.67
19556.19	214.9423	19556.25	214.9406	19557.61	215.4974	19547.29	214.9393	19549.94	214.9441	19547.66	214.9378	19547.36
19554.68	215.1886	19556.34	215.1906	19556.21	215.7615	19546.45	215.1928	19547.36	215.1996	19546.76	215.1912	19547.55
19556.17	215.4432	19553.48	215.4388	19556.36	215.9948	19547.46	215.4384	19545.4	215.4478	19548.44	215.4424	19547.39
19555.65	215.6888	19556.49	215.6958	19556.49	216.2499	19545.89	215.691	19545.57	215.6973	19546.28	215.6911	19547.45
19554.69	215.9427	19555.35	215.9422	19558.59	216.4988	19547.56	215.9395	19546.47	215.9437	19546.11	215.9385	19549.96
19556.26	216.1902	19555.48	216.1901	19556.25	216.7549	19546.35	216.1962	19548.83	216.1968	19545.85	216.1892	19546.58
19557.18	216.4378	19554.28	216.4381	19556.04	217.0009	19544.95	216.4398	19546.23	216.4493	19545.87	216.4422	19547.82
19558.91	216.6922	19554.34	216.6937	19555.72	217.2544	19548.56	216.6909	19547.49	216.6947	19548.1	216.6922	19545.99
19557.06	216.9455	19554.88	216.9422	19558.12	217.4984	19544.81	216.9379	19546.96	216.948	19545.37	216.9434	19547.08
19554.6	217.1901	19556.7	217.1916	19555.11	217.7559	19544.99	217.194	19544.85	217.1963	19547.38	217.1955	19547.84
19553.54	217.4415	19552.28	217.4386	19555.46	217.999	19547.42	217.4397	19546.82	217.4499	19546.42	217.4605	19546.41
19555.1	217.6901	19554.99	217.6926	19554.78	218.2515	19547.17	217.6921	19545.32	217.696	19547.87	217.7042	19548.13
19554.6	217.9445	19557.62	217.9443	19555.13	218.4991	19546.69	217.9391	19546.95	217.9436	19546.73	217.939	19546.9
19553.81	218.1909	19553.45	218.1924	19555.36	218.7583	19545.03	218.1962	19547.32	218.1982	19545.77	218.1958	19547.46
19553.72	218.4384	19552.81	218.4388	19554.37	219.0002	19548.08	218.4392	19548.65	218.4483	19546.33	218.4455	19547.17
19556.76	218.691	19554.06	218.6922	19556.79	219.2534	19545.84	218.6925	19545.65	218.6978	19544.02	218.6934	19547.21
19556.06	218.9481	19555.36	218.9434	19555.41	219.4998	19544.58	218.9399	19547.09	218.9443	19546.4	218.9383	19546.19
19554.26	219.1896	19555.04	219.1928	19555.23	219.7564	19547.66	219.1954	19546.62	219.1978	19548.21	219.1914	19544.8
19557.22	219.4388	19556.45	219.4398	19558.1	220.0008	19546.34	219.4392	19545.83	219.4497	19548.58	219.4441	19548.61
19554.18	219.6916	19554.67	219.6925	19557.67	220.2525	19545.52	219.6939	19544.88	219.6972	19548.76	219.6938	19547.18
19557.74	219.9608	19557.45	219.9446	19556.43	220.4997	19543.34	219.9403	19546.36	219.9451	19547.92	219.9434	19547.26
19555.85	220.2007	19556.94	220.1936	19556.34	220.7551	19544.69	220.1944	19547.66	220.1975	19544.14	220.1947	19549.12
19555.22	220.4414	19555.77	220.4416	19554.26	221.0003	19545.8	220.4426	19549.33	220.4556	19547.81	220.4441	19544.42
19553.39	220.6916	19555.03	220.6972	19552.83	221.2518	19548.64	220.6932	19545.99	220.702	19547.61	220.6929	19546.88
19555.14	220.9475	19554.76	220.9441	19557.12	221.5015	19544.09	220.941	19548.9	220.9461	19545.51	220.9421	19546.97
19557.22	221.193	19554.12	221.1931	19556.26	221.7568	19548.9	221.1955	19547.72	221.1977	19547.42	221.1948	19547.61
19555.61	221.4397	19555.77	221.4407	19554.74	221.9955	19546.9	221.4412	19546.09	221.4528	19545.07	221.4479	19548.37
19555.2	221.6904	19555.7	221.6935	19555.24	222.253	19544.23	221.6929	19547.61	221.7024	19546.33	221.6935	19548.49
19555.52	221.9454	19555.11	221.9449	19558.72	222.501	19546.41	221.941	19546.76	221.9463	19545.93	221.939	19547.86
19555.71	222.1942	19557.32	222.1942	19556.02	222.7606	19546.4	222.1993	19547.97	222.1997	19545.38	222.1955	19547.49
19556.97	222.4414	19557.24	222.4407	19558.47	222.9978	19546.34	222.4444	19546.58	222.4521	19545.12	222.4455	19548.19
19555.2	222.6941	19553.94	222.6956	19554.79	223.254	19544.25	222.6947	19546.24	222.7005	19547.86	222.6988	19548.23
19555.93	222.945	19555.32	222.9461	19554.95	223.5018	19548.56	222.9415	19546.28	222.9459	19546.34	222.9411	19545.64
19556.67	223.1947	19554.95	223.2003	19557.29	223.7586	19545.93	223.1989	19547.24	223.2031	19548.15	223.193	19547.41
19554.53	223.4416	19554.08	223.4408	19557.04	223.997	19548.1	223.443	19543.39	223.4529	19548.2	223.4484	19545.66
19553.81	223.6932	19554.1	223.6935	19556.59	224.2551	19547.47	223.6958	19549.29	223.7005	19545.2	223.6962	19547.37
19555.38	223.9466	19555.73	223.9476	19557.05	224.5015	19548.34	223.9418	19548.85	223.9461	19546.94	223.9397	19547.86
19553.61	224.1925	19556.38	224.1952	19556.95	224.757	19545.14	224.1985	19547.26	224.2	19545.66	224.1957	19548.24
19551.21	224.4439	19556.92	224.4416	19557.24	225.0014	19547.05	224.4425	19548.06	224.4525	19547.05	224.4474	19548.22
19554.11	224.6929	19554.49	224.6945	19555.59	225.2546	19544.44	224.6938	19547.09	224.7002	19547.77	224.6938	19547.84
19554.28	224.9474	19555.48	224.9466	19555.56	225.5023	19549.56	224.9426	19546.42	224.9477	19545.66	224.9426	19547.89
19555.75	225.1937	19556.38	225.1965	19556.12	225.7594	19547.74	225.2004	19547.21	225.2033	19548.48	225.1938	19545.95
19554.82	225.4403	19555.16	225.4427	19554.18	226.0034	19543.45	225.4463	19548.57	225.4572	19546.58	225.4565	19546.19
19557.39	225.6938	19555.81	225.6963	19556.5	226.2559	19546.36	225.6956	19545.54	225.7016	19546.39	225.7033	19547.17
19556.21	225.9511	19558.14	225.9478	19558.8	226.5032	19546.99	225.9454	19543.86	225.9487	19545.36	225.9472	19546.98
19556.3	226.1938	19555.81	226.1959	19556.12	226.7581	19546.12	226.1955	19547.82	226.2024	19547.49	226.1936	19546.44

19556.45	226.4443	19554.36	226.4431	19557.71	226.9997	19549.84	226.4452	19547.34	226.4523	19545.51	226.4508	19547.95
19554.65	226.6935	19555.23	226.6978	19556.88	227.2577	19546.2	226.6954	19547.46	226.7004	19546.09	226.6977	19545.91
19555.83	226.9475	19558.72	226.9471	19556.55	227.5007	19547.62	226.9439	19547.56	226.9487	19547.93	226.9428	19546.95
19555.72	227.1961	19554.14	227.1964	19556.23	227.7588	19546.79	227.2008	19545.15	227.2025	19547.33	227.1963	19547.06
19555.06	227.4443	19556.55	227.4449	19554.09	228.0019	19547.57	227.4475	19548.2	227.4532	19543.2	227.4474	19547.36
19553.61	227.6961	19556.41	227.6964	19557.85	228.2556	19549.03	227.6967	19546.62	227.7008	19545.58	227.6974	19546.64
19556.64	227.949	19553.78	227.9474	19553.86	228.5026	19547	227.9443	19543.58	227.9533	19545.49	227.9428	19547.8
19557.11	228.1943	19555.1	228.1962	19557.25	228.7611	19546.7	228.2009	19545.49	228.2018	19546.06	228.1972	19548.82
19556.83	228.4418	19556.38	228.4438	19554.12	229.004	19546.04	228.4486	19547.81	228.4542	19545.76	228.4485	19546.47
19556.84	228.6974	19555.76	228.6986	19555.79	229.2568	19547.7	228.6986	19545.51	228.7023	19547.37	228.695	19545.77
19559.34	228.9478	19555.66	228.9522	19556.02	229.5023	19544.57	228.9438	19546.16	228.9481	19547.18	228.9476	19546.93
19555.43	229.1966	19554.72	229.1974	19554.66	229.7608	19546.16	229.1999	19547.41	229.203	19547.19	229.1958	19542.92
19555.08	229.4422	19554.53	229.4459	19557.51	230.0049	19547.11	229.4485	19545.48	229.4529	19543.44	229.4502	19548.41
19555.12	229.6941	19556.42	229.6958	19558.38	230.2563	19544.58	229.698	19547	229.7013	19545.74	229.6968	19546.74
19557.36	229.9502	19556.37	229.9491	19556	230.4997	19547.58	229.9436	19545.64	229.9502	19546.29	229.9464	19548.78
19557.27	230.199	19558.91	230.1966	19555.21	230.7605	19547.09	230.2008	19546.08	230.2017	19546.77	230.1998	19545.17
19554.33	230.4415	19556.51	230.4452	19555.27	231.0066	19544.42	230.4485	19548.64	230.4567	19543.85	230.4506	19545.59
19555	230.6959	19553.41	230.7055	19555.38	231.2511	19543.47	230.6976	19545.89	230.7024	19547.96	230.6963	19546.37
19555.24	230.956	19556.06	230.9495	19556.55	231.506	19544.66	230.9447	19546.12	230.9476	19546.38	230.9429	19548.6
19558.71	231.2094	19555.96	231.1992	19556.79	231.758	19546.84	231.2037	19546.78	231.2043	19545.98	231.1977	19545.28
19554.35	231.4541	19555.76	231.4455	19555.22	232.0074	19545.66	231.4497	19547.26	231.4545	19547.98	231.45	19546.32
19556.55	231.7013	19555.13	231.6993	19554.77	232.2544	19547.45	231.6986	19547.09	231.7032	19547.99	231.6988	19549.35
19553.96	231.9518	19557.14	231.9601	19555.47	232.506	19544.18	231.9462	19546.81	231.9491	19544.3	231.9439	19548.38
19559.46	232.2	19555.81	232.2205	19555.79	232.7574	19546.37	232.2004	19546.27	232.206	19546.33	232.1976	19546.37
19553.52	232.4461	19556.56	232.4599	19556.2	233.0207	19546.42	232.4509	19547.21	232.458	19549.11	232.4514	19547.45
19555.46	232.6957	19555.19	232.701	19555.61	233.27	19544.29	232.6999	19543.34	232.7071	19545.62	232.6997	19547.93
19554.27	232.9539	19555.33	232.9512	19554.76	233.516	19545.8	232.947	19547.44	232.9541	19544.71	232.946	19547.57
19554.69	233.2015	19559.31	233.1998	19555.3	233.7708	19548.64	233.2028	19546.56	233.2035	19545.72	233.1996	19544.66
19555.2	233.4493	19554.98	233.4459	19554.34	234.0182	19548.12	233.4502	19545.48	233.4564	19544.1	233.451	19547.99
19555.38	233.6977	19555.27	233.699	19557.19	234.266	19547.28	233.6992	19545.72	233.7077	19548.8	233.6991	19547.57
19559.19	233.9533	19553.55	233.9506	19555.52	234.5158	19545.65	233.9469	19545.82	233.9576	19548.1	233.9475	19545.85
19553.04	234.2024	19553.79	234.2001	19556.49	234.7662	19545.66	234.2053	19547.34	234.2087	19547.32	234.1982	19546.4
19554.38	234.4523	19554.41	234.4467	19556.85	235.0137	19544.2	234.4577	19547.35	234.4611	19548.6	234.4511	19546.3
19555.61	234.7012	19554.09	234.6997	19554.53	235.2618	19546.79	234.7085	19547.59	234.712	19546.69	234.6995	19547.56
19552.62	234.9571	19556.98	234.9511	19557.47	235.5089	19544.38	234.9568	19546.69	234.9597	19547.6	234.9458	19547.67
19554.21	235.2048	19556.81	235.2015	19558.33	235.7616	19546.22	235.2117	19547.03	235.2125	19548.75	235.1999	19547.15
19555.85	235.4538	19555.64	235.4558	19556.81	236.0129	19546.04	235.4607	19545.37	235.4653	19548.24	235.4524	19546.89
19557.99	235.7046	19554.4	235.7078	19557.01	236.254	19546.09	235.7147	19544.85	235.7188	19545.48	235.6991	19546.47
19555.15	235.957	19555.26	235.9599	19554.68	236.5083	19544.6	235.9622	19546.69	235.9694	19545.19	235.9473	19546.19
19555.34	236.2107	19556.1	236.2104	19556.81	236.7623	19546.76	236.2151	19543.96	236.2249	19544.93	236.1995	19549.4
19556.74	236.4599	19556.76	236.4584	19554.27	237.0054	19546.48	236.4682	19546.04	236.4804	19547.49	236.4547	19544.7
19554.96	236.7118	19555.99	236.7104	19555.6	237.2622	19547.39	236.7216	19546.45	236.961	19544.5	236.7004	19546.35
19551.97	236.9686	19555.81	236.9647	19557.48	237.5041	19548.81	236.973	19545.06	237.2135	19547.35	236.9503	19545.95
19555.72	237.2205	19554.31	237.2173	19555.41	237.7631	19547.02	237.2323	19546.42	237.4681	19547.53	237.2023	19545.69
19555.72	237.4813	19555.23	237.4659	19558.13	238.0054	19545.77	237.7104	19546.37	237.7222	19547.13	237.4542	19545.18
19557.38	237.9636	19552.88	237.7188	19554.06	238.2602	19546.83	237.9609	19545.48	237.9727	19546.04	237.7014	19547.67
19555.91	238.2169	19555	237.9744	19556.7	238.5052	19547.68	238.2187	19546.5	238.226	19545.73	237.952	19546.83
19555.5	238.4658	19555.93	238.2287	19555	238.7655	19545.85	238.4709	19545.64	238.4797	19544.93	238.2041	19546.62
19556.52	238.725	19554.89	238.7071	19554.55	239.0087	19544.41	238.724	19547.16	238.7332	19549.03	238.4572	19547.88
19555.71	238.9937	19553.93	238.9639	19556.7	239.2609	19546.88	238.9741	19548.68	238.9842	19546.56	238.7019	19545.99
19553.56	239.4703	19555.91	239.2171	19554.3	239.5068	19543.8	239.2304	19545.93	239.4721	19546.64	238.9483	19547.37
19555.64	239.7233	19553.86	239.4674	19556.39	239.7647	19546.71	239.4841	19545.91	239.7277	19549.36	239.2011	19547.09
19555.4	239.9736	19555.47	239.722	19554.96	240.0101	19546.48	239.7379	19545.59	239.9807	19546.51	239.453	19547.83
19555.42	240.22	19555.87	239.9851	19558.41	240.2621	19547.81	240.2206	19545.26	240.2348	19546.36	239.7013	19547.88
19555.8	240.4704	19554.44	240.463	19555.67	240.5008	19547.01	240.4705	19546.84	240.4912	19546.91	239.9486	19546.95
19554.46	240.7222	19555.48	240.7221	19558.88	240.7718	19545.68	240.7127	19546.31	240.9689	19544.08	240.2005	19546.79
19554.12	240.9787	19555.3	240.9733	19553.73	241.0302	19546.62	240.9597	19546.14	241.2206	19546.09	240.4621	19549.26
19555.44	241.2154	19554.84	241.223	19556.07	241.2711	19544.26	241.2181	19546.84	241.4777	19546.39	240.7083	19546.25
19554.8	241.4682	19557.64	241.4624	19555.77	241.5036	19548.08	241.461	19547.89	241.7218	19546.88	240.944	19548.58
19553.44	241.7157	19557.22	241.7126	19555.27	241.7623	19545.96	241.7135	19547.8	241.9686	19545.98	241.2005	19548.11
19555.48	241.9706	19553.9	241.9653	19552.59	242.0064	19546.52	241.9609	19546.03	242.2242	19544.87	241.4567	19546.53
19554.76	242.2174	19555.67	242.2142	19558.8	242.2578	19546.11	242.2179	19545.46	242.4709	19546	241.7029	19544.26
19554	242.4676	19557.44	242.462	19555.83	242.5054	19545.93	242.4619	19547	242.7226	19547.13	241.9487	19546.04
19554.35	242.7191	19554.26	242.7154	19556.82	242.7614	19546.41	242.7157	19543.98	242.9707	19545.99	242.2038	19548.75
19557.81	242.9773	19555.04	242.9699	19557.11	243.0066	19546.71	242.9617	19545.83	243.2227	19546.88	242.4537	19544.5
19553.53	243.2169	19555.32	243.2184	19555.64	243.2578	19545.37	243.2176	19546.64	243.4789	19547.08	242.7008	19547.95
19558.04	243.4669	19556.69	243.463	19554.54	243.5059	19544.94	243.4631	19549.6	243.7283	19545.18	242.9481	19547.83
19556.32	243.7183	19555.73	243.7129	19554.96	243.7629	19545.23	243.715	19544.21	243.969	19547.18	243.1963	19549.46
19553.83	243.9724	19556.5	243.9686	19555.8	244.0081	19547.04	243.9619	19546.34	244.2272	19545.39	243.4496	19549.73
19554.87	244.218	19555.38	244.2142	19554.41	244.2604	19544.75	244.2182	19545.93	244.474	19546.4	243.6985	19548.96

19556.1	244.467	19554.58	244.4622	19553.4	244.5079	19547.34	244.4619	19548.02	244.7176	19546.11	243.9515	19544.78
19556.86	244.7223	19555.84	244.7156	19555.06	244.7615	19544.31	244.7139	19545.58	244.972	19545.07	244.2004	19546.97
19553.95	244.973	19555.67	244.9675	19555.7	245.008	19544.15	244.9646	19546.78	245.2232	19545.03	244.4493	19547.08
19555.7	245.2187	19555.76	245.2141	19557.19	245.2603	19547.45	245.2176	19546.48	245.4732	19546.55	244.6967	19547.89
19555.43	245.4669	19554.34	245.4643	19555.72	245.5068	19546.27	245.4638	19547.26	245.7184	19544.89	244.9467	19547.11
19555.28	245.7164	19554.96	245.7194	19555.78	245.7624	19545.9	245.7136	19546.86	245.97	19545.29	245.1984	19546.56
19556.11	245.9725	19555.57	245.9704	19554.51	246.0082	19546.25	245.9616	19543.55	246.2232	19547.24	245.4497	19548.34
19554.57	246.2179	19556.24	246.2216	19555.47	246.261	19544.72	246.2176	19545.06	246.4692	19547.82	245.6984	19546.14
19554.43	246.4675	19555.68	246.4749	19553.84	246.5068	19547.89	246.4647	19544.82	246.7237	19549.43	245.9489	19544.32
19554.28	246.719	19555.6	246.7252	19555.7	246.7644	19545.45	246.7158	19544.69	246.9678	19548.73	246.1997	19548.71
19553.68	246.9795	19555.94	246.9864	19555.53	247.0074	19544.86	246.9624	19543.72	247.2253	19545.76	246.4545	19548.46
19557.58	247.2193	19555.53	247.247	19554.7	247.2606	19544.1	247.2189	19547.81	247.4777	19545.45	246.6993	19546.38
19556.2	247.4703	19555.1	247.7312	19555.49	247.5081	19547.58	247.4639	19544.83	247.7289	19546.46	246.9469	19547.1
19556.04	247.7193	19554.35	248.0092	19553.71	247.7648	19545.92	247.7165	19547.11	247.9741	19544.62	247.2006	19544.52
19556.25	247.9812	19553.12	248.4859	19555.67	248.0103	19546.59	247.9624	19546.02	248.2258	19547.02	247.4541	19548.49
19555.49	248.2193	19556.67	248.7411	19555.6	248.2556	19547.01	248.2164	19544.81	248.4747	19544.39	247.6997	19547.03
19556.14	248.4697	19556.94	248.9894	19553.05	248.5074	19545.34	248.4763	19547.99	248.7253	19545.07	247.9487	19548.09
19553.48	248.7202	19552.13	249.2391	19556.86	248.7632	19544.88	248.7147	19546.96	248.9688	19545.33	248.1986	19547.06
19555.32	248.9739	19555.24	249.4894	19558.2	249.0093	19545.62	248.964	19546.68	249.2284	19545.71	248.453	19545.71
19557.06	249.2198	19555.04	249.7371	19557.04	249.2627	19547.25	249.2199	19547.27	249.4787	19544.2	248.7006	19546.9
19554.1	249.4698	19555.64	249.9902	19552.85	249.507	19547.29	249.4653	19542.29	249.7257	19547.02	248.9475	19546.94
19555.12	249.7202	19556.46	250.2371	19554.63	249.7614	19549.03	249.7156	19546.44	249.9709	19545.38	249.201	19548.92
19554.18	249.9816	19553.62	250.486	19556.9	250.0107	19547.28	249.9629	19545.86	250.2258	19545.96	249.4519	19545.24
19555.6	250.2296	19554	250.7422	19554.02	250.2574	19547.65	250.2245	19544.75	250.4756	19543.41	249.7045	19546.48
19556.62	250.4731	19553.08	250.9906	19557.19	250.5096	19545.35	250.4668	19544.28	250.7236	19543.15	249.9494	19545.37
19553.26	250.7215	19553.44	251.2389	19555.26	250.7583	19546.57	250.7166	19545.94	250.9711	19545.98	250.1988	19546.69
19555.5	250.9757	19551.64	251.4913	19556.36	251.0183	19547.39	250.964	19544.95	251.2263	19544.75	250.468	19547.7
19553.05	251.2206	19555.3	251.739	19555.12	251.2671	19545.71	251.2214	19544.36	251.4844	19543.93	250.7221	19549.42
19556.2	251.4745	19554.74	251.9905	19554.44	251.5129	19543.6	251.4658	19549.87	251.7455	19546.98	250.9701	19546.35
19553.93	251.7218	19553.09	252.2398	19554.99	251.7622	19546.96	251.7185	19545.11	252.2348	19547.36	251.2187	19545.62
19556.04	251.9774	19554.85	252.4878	19555.35	252.0113	19546.81	251.9664	19544.33	252.4887	19546.92	251.4703	19546.64
19556.21	252.2229	19552.71	252.7395	19555.4	252.2596	19544.02	252.2202	19545.69	252.738	19547.15	251.7169	19545.52
19554.95	252.4758	19555.6	252.9911	19553.89	252.5068	19547.08	252.4669	19546.99	252.9891	19545.11	251.9635	19543.84
19555.91	252.7231	19553.95	253.2424	19554.51	252.7625	19546.36	252.7278	19544.99	253.24	19545.87	252.2129	19545.65
19557.07	252.9803	19555.06	253.4903	19556.45	253.0118	19546.43	252.9705	19546.4	253.4918	19545.46	252.4638	19548.2
19552.66	253.2272	19554.53	253.7411	19553.54	253.2646	19545.79	253.2195	19543.19	253.7419	19544.95	252.7141	19545.25
19552.27	253.4732	19553.98	253.9913	19555.34	253.5118	19545.95	253.4761	19545.11	253.9899	19546.64	252.9715	19546.02
19553.89	253.7236	19554.74	254.242	19555.16	253.7634	19544.89	253.7185	19547.57	254.2404	19544.91	253.221	19543.62
19553.7	253.9761	19554.75	254.4891	19551.88	254.0158	19546.14	253.9642	19544.65	254.4936	19544.7	253.4733	19547.55
19555.84	254.2233	19557.93	254.7412	19555.12	254.2676	19546.88	254.2183	19544.58	254.7449	19547.12	253.7228	19546.45
19554.05	254.4703	19555.15	254.9916	19556.1	254.5158	19547.99	254.4678	19548.62	254.9943	19545.16	254.2074	19544.49
19552.41	254.7246	19552.06	255.2424	19554.68	254.7659	19544.39	254.7177	19546.97	255.2491	19544.56	254.462	19547.66
19554.11	254.9768	19552.86	255.4951	19558.49	255.0163	19544.56	254.9666	19545.27	255.7362	19545.78	254.7117	19547.27
19554.75	255.2305	19556.61	255.7477	19556.89	255.2667	19545.6	255.2388	19546.54	255.9851	19544.33	254.961	19547.8
19553.21	255.4795	19557.34	256.0065	19553.96	255.5151	19544.08	255.4926	19547.86	256.2427	19545.6	255.2111	19548.26
19555.22	255.7285	19553.78	256.2619	19553.47	255.7662	19545.37	255.9725	19545.32	256.4957	19546.77	255.463	19547.12
19556.87	255.9813	19553.78	256.7463	19556.68	256.0176	19546.63	256.2274	19546.03	256.7456	19545.18	255.7169	19544.97
19556.64	256.23	19555.73	256.9983	19556.28	256.2694	19546.12	256.4823	19544.1	256.9964	19546.3	255.9666	19546.31
19557.48	256.4778	19555.9	257.2492	19551.96	256.5181	19544.02	256.7336	19545.82	257.2533	19546.58	256.2164	19545.48
19557.29	256.7297	19558.33	257.498	19553.81	256.7734	19544.9	256.9809	19545.38	257.7421	19546.76	256.4684	19546.65
19555.29	256.9844	19554.65	257.7485	19556.71	257.0246	19546.78	257.2357	19547.48	257.991	19546.42	256.7196	19547.31
19556.08	257.2333	19554.21	258.0021	19556.48	257.2769	19544.98	257.4862	19546.22	258.2434	19545.43	256.9686	19545.73
19554.51	257.4871	19554.86	258.2543	19555.01	257.7725	19545.21	257.738	19546.2	258.4995	19546.54	257.2218	19548.33
19555.58	257.7358	19554.34	258.5033	19557.09	258.2848	19543.64	257.9912	19546.8	258.7509	19544.96	257.4765	19545.44
19555.77	257.9893	19551.97	258.7546	19554.51	258.5393	19544.21	258.2471	19546.29	259.0024	19545.25	257.9612	19546.38
19556.8	258.2443	19556.62	259.007	19556.42	258.7953	19546.1	258.7319	19545.4	259.261	19547.69	258.2154	19546.95
19556.09	258.4957	19554.63	259.2588	19555.24	259.0448	19544.84	258.9823	19545.7	259.7501	19544.93	258.4701	19545.94
19554.79	258.9836	19553.35	259.5088	19555.54	259.2994	19543.71	259.2369	19544.98	260.0014	19546.05	258.732	19547.2
19552.88	259.2332	19555.35	259.7637	19554.74	259.7921	19544.91	259.4936	19545.83	260.2597	19543.44	258.9821	19545.61
19555.62	259.4817	19554.34	260.0185	19555.01	260.0468	19546.39	259.7466	19548.85	260.5167	19544	259.233	19548.08
19553.12	259.7326	19553.59	260.4997	19555.38	260.3008	19546.2	259.9965	19545.75	261.0069	19545.41	259.4878	19548.43
19556.92	259.9874	19553.09	260.7603	19556.29	260.5508	19543.28	260.2526	19545.93	261.2622	19545.23	259.9691	19546.17
19554.83	260.2369	19553.74	261.0142	19557.25	260.8138	19548.95	260.736	19545.54	261.5184	19544.24	260.2225	19545.12
19554.2	260.4882	19555.61	261.2687	19554.02	261.3036	19543.65	260.9875	19545.06	261.7723	19544.62	260.4781	19546.66
19557.41	260.739	19554.37	261.5209	19553.27	261.5549	19547.07	261.2462	19547.83	262.2628	19545.65	260.7307	19545.53
19553.57	260.9961	19557.03	261.7754	19552.56	261.8105	19546.36	261.4988	19544.82	262.5189	19548.47	260.9813	19545.18
19554.06	261.2468	19555.14	262.2629	19555.31	262.0663	19541.9	261.7515	19546.81	262.7714	19548.19	261.2353	19545.17
19555.59	261.4987	19553.24	262.5132	19555.2	262.3261	19542.62	262.2455	19545.82	263.0222	19546.39	261.4896	19548.16
19555.48	261.7493	19553.13	262.7707	19553.97	262.819	19546.17	262.5022	19545.95	263.2777	19544.7	261.9759	19550.64
19556.31	262.2349	19553.34	263.0267	19555.24	263.0773	19545.58	262.7567	19545.07	263.7765	19547.01	262.2297	19546.33

19555.08	262.4925	19555.02	263.2821	19553.59	263.3352	19543.52	263.0102	19544.69	264.0304	19547.04	262.4865	19547.16
19554.87	262.7464	19555.34	263.7741	19556.01	263.8341	19546.14	263.4961	19546.88	264.2876	19546.07	262.7385	19545.98
19556.14	263.0034	19554.27	264.0312	19555.57	264.0879	19546.11	263.7492	19546.29	264.7841	19544.53	262.9918	19546.11
19553.39	263.4876	19555.96	264.2873	19556.72	264.3446	19545.99	264.0039	19545.31	265.0375	19542.81	263.2452	19546.22
19552.58	263.7421	19552.74	264.7739	19555.17	264.8416	19546.02	264.2615	19546.73	265.296	19546.26	263.501	19544.52
19554.48	264.0011	19553.47	265.0291	19556.55	265.0963	19545.03	264.5179	19548	265.7898	19545.91	263.9847	19546.91
19552.41	264.253	19552.99	265.2848	19554.1	265.3521	19544.24	265.005	19545.73	266.0434	19547.07	264.2385	19548.06
19553.97	264.5061	19557.69	265.5383	19556.74	265.8474	19545.28	265.2645	19545.46	266.3003	19546.69	264.496	19545.29
19555.76	264.7595	19557.46	265.7987	19553.12	266.103	19543.98	265.52	19546.18	266.7936	19545.9	264.7533	19547.81
19554.12	265.2434	19555.76	266.2884	19554.77	266.3619	19544.7	265.7801	19545.13	267.0477	19544.97	265.0091	19545.48
19554.78	265.5029	19555.18	266.544	19554.7	266.8603	19546.01	266.2761	19547.28	267.3065	19545.07	265.4981	19548.85
19557.04	265.7613	19554.74	266.8013	19554.39	267.122	19546.04	266.5315	19544.12	267.8028	19545.56	265.7561	19543.85
19554.42	266.0208	19554.21	267.0577	19555.35	267.6119	19547.14	266.7892	19545.81	268.0595	19547.4	266.0161	19544.91
19554.93	266.5124	19552.63	267.5442	19556.26	267.8794	19546.93	267.2795	19542.35	268.3183	19543.52	266.5084	19544.42
19555.16	266.7655	19556.05	267.8033	19552.85	268.3718	19542.37	267.5346	19546.36	268.8111	19545.16	266.7674	19546.89
19555.88	267.0253	19559.19	268.06	19553.77	268.6257	19544.42	267.7937	19545.62	269.0622	19545.47	267.0226	19547.61
19553.9	267.5129	19553.64	268.3181	19555.34	268.8871	19546.69	268.2861	19547.22	269.3153	19544.47	267.5141	19543.42
19553.56	267.7671	19556.12	268.8061	19554.05	269.3812	19546.19	268.543	19545.48	269.5644	19544.08	267.7718	19547.82
19553.7	268.0292	19557.84	269.0632	19555.42	269.6349	19544.24	268.8006	19545.18	269.8126	19545.25	268.0273	19546.89
19555.64	268.518	19555.12	269.3207	19555.19	269.8971	19546.21	269.2909	19544	270.0604	19545.78	268.5185	19548.24
19555.32	268.781	19552.37	269.5768	19558.17	270.3918	19546.9	269.5476	19545.91	270.3104	19543.41	268.7795	19545.6
19556.14	269.0516	19555.77	270.066	19556.14	270.6466	19545.36	269.8037	19546.78	270.5615	19546.26	269.0352	19548.15
19553.19	269.5468	19551.68	270.324	19556.06	270.9098	19543.73	270.0625	19545.75	270.8121	19547.44	269.5394	19546.49
19552.97	269.8051	19555.08	270.5807	19555.04	271.405	19545.01	270.5527	19544.97	271.0593	19545.01	270.028	19548.6
19553.98	270.2912	19553.23	271.0705	19555.75	271.6611	19543.89	270.8112	19547.65	271.3102	19544.28	270.2848	19545.28
19551.72	270.5499	19556.36	271.3285	19555.11	271.9238	19544.81	271.0687	19544.52	271.5641	19544.93	270.5603	19547.03
19554.12	270.8081	19555.95	271.5849	19555.51	272.4189	19545.48	271.5589	19543.63	271.8166	19547.6	271.0487	19547.44
19555.98	271.3004	19553.78	272.0737	19554.9	272.6794	19544.51	271.8165	19545.27	272.0669	19547.23	271.3058	19545.2
19554.84	271.5595	19554.78	272.3247	19556.07	273.1731	19545.93	272.0756	19542.56	272.3208	19547.56	271.5687	19545.02
19552.88	271.8217	19556.04	272.5751	19554.51	273.4322	19546.96	272.5668	19545.49	272.5734	19546.29	272.0583	19547.95
19556.26	272.3083	19555.05	272.8259	19556.37	273.6896	19546.19	272.8239	19546.22	273.0714	19545.68	272.3155	19546.11
19554.33	272.5687	19552.41	273.0757	19554.84	274.1837	19546.02	273.0861	19544.26	273.3216	19544.35	272.5768	19546.36
19553.18	272.8278	19554.74	273.3257	19553.91	274.4467	19544.13	273.5862	19544.73	273.5752	19545.29	273.067	19546.11
19557.53	273.316	19556.24	273.5737	19554.32	274.9439	19548.35	273.8448	19545.26	273.8262	19545.97	273.3265	19547.45
19556.32	273.578	19553.55	273.8288	19554.43	275.2045	19547.65	274.3403	19547.01	274.0767	19546.81	273.6022	19544.78
19553.66	273.8386	19554.88	274.0821	19553.61	275.6977	19544.53	274.6	19544.75	274.3272	19544.87	274.0938	19546.06
19552.52	274.3284	19552.78	274.3325	19555.26	275.9718	19544.51	275.0905	19544.62	274.5925	19545.58	274.3523	19547.89
19555.06	274.592	19555.75	274.5819	19553.63	276.4674	19547.02	275.3545	19546.25	275.088	19544.75	274.6168	19546.08
19555.13	275.0879	19552.98	274.8394	19555	276.7281	19546.06	275.8419	19544.86	275.3405	19545.36	275.1096	19547.88
19555.27	275.3479	19554.43	275.3298	19554.2	277.2249	19546.73	276.1046	19542.84	275.6043	19548.93	275.3806	19548.46
19554.28	275.8364	19555.81	275.5793	19554.17	277.4873	19544.44	276.3692	19546.26	276.1002	19545.67	275.8699	19546.52
19555.15	276.1087	19555.18	275.8339	19554.73	277.9846	19545.95	276.8584	19544.32	276.3506	19546.68	276.1317	19544.12
19555.44	276.6016	19553.71	276.0849	19555.5	278.2472	19544.16	277.1208	19544.52	276.605	19545.68	276.3965	19546.57
19555.06	276.861	19557.47	276.3354	19554.64	278.7441	19545.32	277.3846	19543.73	276.8557	19545.69	276.8883	19545.88
19552.89	277.1286	19553.67	276.5859	19554.2	279.0085	19546.4	277.8754	19543.1	277.1116	19544.69	277.1563	19544.91
19555.71	277.6208	19553.02	276.8397	19557.45	279.5043	19545.31	278.1429	19544.09	277.6131	19543.59	277.6506	19546.34
19553.05	277.8815	19553.51	277.1049	19555.23	279.7667	19544.95	278.6353	19546.48	277.8656	19547.57	277.9143	19546.73
19554.24	278.3701	19554.69	277.5957	19556.48	280.2656	19547.18	278.8951	19544.61	278.3623	19543.09	278.41	19547.85
19554.27	278.6358	19555.52	277.8577	19554.66	280.5259	19546.58	279.16	19545.65	278.6175	19544.86	278.6717	19546.04
19552.79	279.1299	19556.4	278.1094	19554.94	281.0359	19547.62	279.654	19544.23	278.8684	19545.44	279.1655	19547.13
19555.9	279.389	19554.04	278.5988	19557.54	281.5324	19546.5	279.913	19547.59	279.123	19545.57	279.4315	19547.37
19555.28	279.8799	19554.78	278.8559	19554.71	281.7976	19542.98	280.4101	19544.89	279.6214	19546.12	279.922	19545.91
19552.29	280.1459	19553.81	279.1098	19555.75	282.2952	19545.6	280.6745	19545.54	279.8726	19546.88	280.1859	19546.34
19554.68	280.6375	19555.61	279.3634	19552.38	282.5593	19547.69	281.1683	19546.67	280.1291	19545.31	280.6824	19547.6
19553	280.9088	19553.87	279.8588	19555.28	283.0588	19545.54	281.4353	19543.52	280.6281	19544.07	280.9435	19545.9
19553.22	281.3997	19553.91	280.1155	19554.02	283.326	19546.98	281.9267	19545.43	280.8904	19544.81	281.441	19545.49
19555.49	281.665	19553.19	280.3693	19551.72	283.8311	19543.6	282.1903	19546.03	281.3903	19543.2	281.7043	19546.65
19556.45	282.1593	19554.38	280.8649	19552.33	284.3366	19545.81	282.6882	19543.75	281.6568	19545.09	282.199	19545
19552.68	282.4205	19553.23	281.117	19553.04	284.8398	19545.5	282.9483	19544.5	282.1597	19543.73	282.4654	19544.83
19556.39	282.9175	19556.11	281.3714	19557.74	285.3394	19545.69	283.4488	19544.38	282.4283	19546.64	282.956	19545.81
19556.49	283.1841	19555.4	281.6253	19555.99	285.6044	19541.99	283.7138	19546.41	282.9261	19545.43	283.2219	19545.91
19555.16	283.6779	19553.6	282.1193	19554.99	286.1022	19544.74	284.2165	19544.08	283.1918	19545.41	283.7184	19546.06
19556.53	283.9488	19554.52	282.3724	19555.56	286.3585	19545.67	284.4909	19545.49	283.6916	19546.97	283.9797	19546.5
19555.81	284.4397	19553.49	282.6262	19552.61	286.8638	19544.86	284.9847	19544.82	283.9559	19544.06	284.4773	19546.22
19554.47	284.7057	19554.22	282.8854	19554.55	287.3648	19547.41	285.2526	19544.88	284.459	19543.25	284.7415	19544.89
19551.94	285.1993	19555.62	283.3793	19555.92	287.62	19547.65	285.7558	19544.79	284.9574	19545.87	285.2357	19545.85
19553.99	285.4634	19552.29	283.6457	19556.05	288.1196	19545.32	286.0192	19544.11	285.2265	19545.94	285.5	19546.8
19553.29	285.9617	19554.68	284.1443	19554.5	288.3772	19543.46	286.5136	19547.34	285.727	19547.03	285.9918	19548.04
19556.99	286.2294	19553.96	284.408	19554.26	288.8817	19543.86	286.7837	19542.71	285.9918	19546.14	286.2472	19546.73
19553.84	286.7237	19554.31	284.9049	19555.45	289.3817	19545.26	287.2822	19543.04	286.4944	19544.71	286.5054	19546.05

19553.04	286.9961	19552.27	285.1728	19555.08	289.6382	19546.25	287.5479	19545.44	286.7613	19546.99	286.9985	19543.05
19553.27	287.4911	19556.03	285.6689	19556.11	290.1396	19545.27	288.0449	19545.4	287.2625	19546.36	287.2545	19546.66
19552.28	287.7565	19554.1	285.9385	19554.87	290.4007	19544.19	288.3175	19544.12	287.7667	19546.84	287.5145	19545.11
19555.16	288.2511	19553.65	286.4319	19554.8	290.9065	19544.83	288.8142	19545.39	288.0326	19543.5	288.009	19546.99
19555.5	288.5171	19555.64	286.6985	19555.39	291.4092	19543.65	289.0805	19543.91	288.5362	19543.57	288.2658	19545.9
19553.38	289.0159	19554.56	287.1939	19553.79	291.9197	19542.03	289.577	19545.24	289.0336	19544.91	288.5282	19544.38
19553.68	289.2816	19553.13	287.4592	19556.74	292.4187	19546.18	289.8443	19544.32	289.302	19544.93	289.0227	19545.5
19554.37	289.7748	19555.16	287.9576	19556.13	292.6748	19546.09	290.344	19544.88	289.8036	19544.12	289.2784	19546.44
19553.63	290.0356	19554.25	288.2259	19555.34	293.1776	19545	290.605	19545.54	290.0712	19544.28	289.5398	19542.99
19552.3	290.531	19554.41	288.7205	19556.21	293.4364	19543.64	291.1018	19545.29	290.5772	19546.84	290.0396	19543.17
19553.8	290.7868	19554.74	288.9917	19554.15	293.9413	19546.48	291.3619	19547.39	291.0779	19543.79	290.3011	19546.33
19552.99	291.0492	19551.88	289.4866	19556.31	294.4423	19544.49	291.857	19546.13	291.3454	19546.67	290.7971	19543.02
19555.7	291.5436	19553.61	289.7556	19555.3	294.6994	19546.19	292.1154	19544.34	291.8459	19546.54	291.057	19545.32
19553.13	291.8006	19556.87	290.2531	19554.9	295.2004	19543.56	292.6192	19543.37	292.1158	19546.38	291.5573	19544.32
19555.39	292.0638	19554.06	290.5195	19555.21	295.4588	19545	293.1186	19545.55	292.6201	19545.74	291.8136	19546.17
19552.68	292.5638	19554.82	291.0198	19552.62	295.9681	19546.1	293.3861	19547.3	293.1207	19545.24	292.3096	19545.37
19555.13	292.8217	19552.92	291.2901	19552.25	296.469	19544.54	293.8818	19547.53	293.6273	19545.29	292.5703	19545.6
19555.75	293.3157	19553.77	291.7884	19554.45	296.7284	19546.43	294.1431	19545.97	294.129	19541.57	293.0659	19544.01
19556.51	293.5741	19553.09	292.0629	19554	297.2306	19544.97	294.6431	19545.01	294.6365	19545.26	293.3231	19546.17
19552.89	294.0806	19553.09	292.5674	19554.36	297.4882	19545.12	294.9016	19544.78	295.1404	19547.1	293.5852	19545.86
19555.14	294.3371	19555.01	293.0667	19555.74	297.9953	19544.77	295.4049	19544.96	295.6474	19545.85	294.0832	19543.62
19553.56	294.8369	19555.68	293.3352	19554.94	298.4978	19543.67	295.9014	19546.14	296.1512	19546.6	294.3403	19545.61
19556.04	295.1027	19555.88	293.8332	19554.34	298.7732	19543.96	296.1638	19544.85	296.6546	19544.64	294.8369	19546.91
19553.8	295.6027	19553.03	294.1156	19553.83	299.2769	19544.77	296.6638	19544.41	297.1579	19545.89	295.0971	19544.44
19553.97	295.8612	19556.13	294.6099	19554.07	299.7821	19546.17	296.9239	19545.29	297.6625	19544.65	295.6024	19546.92
19553.86	296.3567	19554.88	294.8866	19553.47	300.2859	19542.27	297.4241	19546.44	298.1659	19546.12	295.8595	19546.22
19556.95	296.6179	19552.71	295.3857	19556.11			297.6856	19544.57	298.6711	19544.27	296.3555	19548.7
	297.1241	19553.17	295.6571	19556.27			298.1882	19546.21	299.1756	19545.85	296.6323	19546.51
	297.6239	19554.01	296.1563	19554.44			298.4526	19543.42	299.6841	19547.25	297.1339	19545.45
	297.8839	19556.4	296.426	19555.83			298.9513	19546.84	300.1901	19545.81	297.4067	19546.47
	298.3801	19555.39	296.9292	19553.22			299.2137	19546.9			297.9041	19546.39
	298.6427	19551.86	297.4267	19555.2			299.7144	19546.21			298.1779	19544.5
	299.1612	19553.03	297.6995	19554.05			299.9745	19546.97			298.6777	19545.97
	299.6603	19551.04	298.1988	19553.1			300.4769	19545.43			298.9483	19544.38
	299.9232	19555.05	298.7031	19554.16							299.4503	19549.15
	300.4215	19553.56	298.9782	19556.69							299.7287	19544.96
			299.4764	19555.21							300.229	19544.1
			299.7503	19553.46								
			300.2558	19555.13								

		1.43										
		52.5										
x_5		x_1		x_2		x_3		x_4		x_5		x
t (s)	P (cm^{-1})	t (s)	P (cm^{-1})	t (s)	P (cm^{-1})	t (s)	P (cm^{-1})	t (s)	P (cm^{-1})	t (s)	P (cm^{-1})	t (s)
0	19560.9	0	19554.41	0	19557.18	0	19557.38	0	19555.8	0	19556.99	0
0.65995	19562.98	0.66364	19554.04	0.66956	19553.81	0.65933	19555.17	0.66699	19554.51	0.67098	19556.72	2.79788
0.84906	19559.77	0.8485	19553.13	0.85442	19556.38	0.85305	19554.84	0.85815	19557.3	0.86614	19556.09	3.27253
1.09983	19560.74	1.09968	19555.35	1.10335	19554.15	1.10081	19555.76	1.10735	19557.77	1.10732	19554.25	3.49022
1.34732	19561.59	1.34923	19554.07	1.3527	19553.87	1.35379	19556.61	1.35941	19554.74	1.35297	19557.47	3.67526
1.59907	19563.54	1.59655	19553.22	1.60731	19557.44	1.59743	19553.63	1.6097	19554.31	1.60473	19557.25	3.94912
1.84815	19558.03	1.8468	19555.16	1.85322	19555.43	1.85023	19557.53	1.85686	19555.08	1.85191	19558.9	4.16277
2.09793	19559.79	2.09711	19556.09	2.10301	19556.09	2.09651	19556.54	2.10499	19555.47	2.10485	19557.36	4.40865
2.34778	19560.8	2.34932	19555.65	2.35171	19554.18	2.34904	19554.67	2.36784	19557.32	2.35296	19556.57	4.72328
2.60137	19560.86	2.59722	19554.95	2.6053	19553.96	2.59686	19555.67	2.61697	19557.17	2.60577	19557.56	4.91446
2.8468	19562.17	2.84719	19555.01	2.85248	19556.6	2.85009	19555.9	2.86167	19558.33	2.84797	19556.89	5.16524
3.09856	19561.31	3.09672	19555.11	3.10257	19555.89	3.09789	19556.77	3.11178	19555.09	3.10235	19556.23	5.41103
3.34783	19563.16	3.3526	19554.86	3.35278	19554.59	3.34944	19554.38	3.3589	19554.73	3.3575	19554.81	5.6575
3.60111	19563.86	3.59914	19554.2	3.60751	19554.45	3.59651	19554.26	3.61079	19554.93	3.60803	19557.15	5.90821
3.8471	19563.64	3.84853	19555.04	3.85174	19556.32	3.85367	19557.98	3.85758	19555.46	3.85135	19555.86	6.16184
4.09841	19560.32	4.09784	19557.35	4.10274	19556.9	4.09732	19557.64	4.11042	19555.93	4.10618	19556.47	6.41019
4.34742	19562.84	4.34801	19555.48	4.35354	19554.73	4.35472	19555.45	4.35745	19556.03	4.35272	19557.47	6.66094
4.60594	19560.23	4.59766	19554.2	4.60788	19554.22	4.59893	19556.33	4.60956	19558.43	4.61318	19555.04	6.90946
4.85518	19562.56	4.85258	19557.29	4.85296	19555.75	4.84966	19554	4.86185	19556.84	4.85629	19557.89	7.15747
5.10402	19561.69	5.10271	19554.65	5.1032	19555.69	5.09724	19555.94	5.10735	19555.25	5.10967	19555.81	7.40861
5.34852	19562.93	5.35026	19553.39	5.35687	19556.52	5.34946	19556.33	5.36889	19555.4	5.35356	19555.89	7.6581
5.60068	19561.61	5.59799	19553.62	5.61125	19557.01	5.59869	19556.44	5.6161	19557.25	5.60934	19556.21	7.9116
5.84871	19562.5	5.84747	19552.02	5.85376	19553.09	5.85076	19558.63	5.85893	19554.29	5.85267	19555.43	8.16033
6.09987	19562.81	6.09837	19553.49	6.10373	19553.45	6.0976	19554.93	6.10978	19553.97	6.10539	19557.3	8.40876
6.34958	19561.13	6.35256	19554.11	6.3545	19556.8	6.34941	19551.9	6.37939	19556.24	6.35372	19558.44	8.65933
6.60427	19561.3	6.59896	19555.99	6.60648	19552.38	6.59749	19555.13	6.60974	19553.53	6.61325	19554.57	8.91079
6.85038	19562.35	6.84931	19554.66	6.85422	19554.41	6.85149	19555.74	6.86619	19558.03	6.85335	19554.9	9.15869
7.09951	19564.82	7.09888	19553.79	7.10426	19554.94	7.0989	19556.47	7.10919	19557.85	7.10717	19558.5	9.41053
7.34803	19560.34	7.35102	19554.77	7.35473	19556.69	7.35074	19554.39	7.3608	19554.84	7.35334	19556.88	9.65837
7.60337	19563.79	7.60024	19552.93	7.60749	19555.91	7.5982	19555.81	7.61188	19555.38	7.60145	19557.18	9.90988
7.85013	19561.42	7.84881	19554.12	7.85997	19554.67	7.85072	19554.54	7.86034	19554.8	7.85375	19553.84	10.16203
8.10046	19562.52	8.09951	19554.8	8.10341	19554.86	8.10206	19554.19	8.1049	19558.94	8.11206	19555.42	10.41326
8.3486	19562.37	8.35177	19554.5	8.35539	19555.39	8.352	19552.96	8.36411	19555.27	8.35709	19555.98	10.66209
8.60267	19562.16	8.60062	19554.47	8.60895	19557.36	8.59894	19555.31	8.61104	19558.34	8.60673	19556.65	10.91015
8.85052	19561.7	8.85022	19553.32	8.85466	19556.1	8.85129	19556.54	8.85831	19556.51	8.85391	19554.86	11.15894
9.10219	19563.21	9.10011	19553.82	9.10464	19554.4	9.0983	19553.09	9.10904	19554.24	9.10721	19556.09	11.41607
9.34855	19560.49	9.35167	19553.87	9.35893	19555.35	9.35251	19554.2	9.36057	19552.2	9.36199	19556.28	11.66028
9.60371	19561.68	9.59887	19553.9	9.6078	19556.74	9.59908	19554.26	9.61279	19556.84	9.61028	19556.59	11.91069
9.84956	19561.76	9.84975	19554.13	9.85519	19556.04	9.85538	19555.18	9.8543	19554.71	9.85387	19556.33	12.15939
10.10086	19561.75	10.10121	19552.61	10.10567	19553.33	10.10802	19556.91	10.10886	19553.06	10.11048	19554.29	12.41067
10.35151	19562.47	10.35294	19554.49	10.3556	19557.32	10.3515	19552.86	10.35995	19555.15	10.35099	19556.49	12.66106
10.6061	19564.21	10.59928	19557.69	10.60996	19555.53	10.5999	19555.13	10.61191	19556.7	10.61018	19555.95	12.91102
10.84974	19562.04	10.85062	19553.31	10.85459	19554.5	10.85272	19555.35	10.85788	19556.32	10.85401	19558.41	13.15954
11.10029	19561.14	11.10195	19553.53	11.10567	19552.96	11.10063	19556.03	11.10866	19555	11.11023	19556.76	13.41241
11.34986	19562.65	11.35313	19555.54	11.35665	19553.91	11.35215	19556.34	11.363	19555.64	11.35786	19558.03	13.6604
11.60594	19561.48	11.60085	19557.03	11.60904	19558.93	11.60052	19555.41	11.61163	19556.55	11.60801	19556.2	13.91268
11.85141	19560.98	11.85019	19554.11	11.8557	19554.49	11.85331	19554.77	11.85916	19556.48	11.85901	19554.5	14.1605
12.10181	19562.88	12.10067	19554.6	12.10532	19555.63	12.10275	19555.8	12.11115	19556.36	12.10767	19557.68	14.4147
12.35012	19560.81	12.35764	19555.22	12.35694	19556.92	12.3531	19554.84	12.3596	19556.28	12.35635	19556.2	14.65981
12.61165	19561.18	12.60297	19557.64	12.60959	19553.07	12.60041	19556.77	12.62042	19554.05	12.61073	19557.25	14.91267
12.85176	19562.79	12.85319	19553.9	12.85613	19553.65	12.85253	19556.22	12.85927	19554.51	12.85466	19553.07	15.1624
13.10268	19563.19	13.10199	19555.59	13.1052	19555.41	13.10104	19555.63	13.11259	19554.97	13.10804	19555.51	15.41291
13.35104	19561.98	13.35252	19554.01	13.35571	19555.46	13.3539	19555.74	13.36205	19555.55	13.35863	19556.72	15.66129
13.605	19563.92	13.60003	19552.84	13.61102	19554.09	13.60033	19556.34	13.61339	19555.13	13.60641	19556.12	15.91222
13.85067	19560.18	13.85129	19553.71	13.85599	19553.5	13.85195	19556.82	13.8609	19555.17	13.85893	19555.39	16.1616
14.10269	19561.86	14.10234	19555.61	14.10597	19555.26	14.10048	19555.65	14.11238	19554.32	14.10806	19557.47	16.41454
14.35163	19561.96	14.35322	19553.81	14.35711	19558.14	14.3539	19555.25	14.36312	19555.97	14.35608	19557.17	16.66141
14.60509	19562.15	14.60104	19553.84	14.60948	19556.16	14.60274	19556.28	14.6151	19556.67	14.60814	19557.43	16.9123
14.85073	19560.99	14.85172	19555.55	14.85469	19556.62	14.85358	19555.15	14.85935	19556.54	14.85907	19554.3	17.16028
15.10325	19563.31	15.10198	19556.65	15.10648	19556.83	15.09997	19556.27	15.11258	19553.91	15.1094	19556.78	17.41215
15.35222	19563.81	15.3635	19555.36	15.35892	19554.35	15.35423	19556.75	15.36672	19556.2	15.35828	19557.5	17.66125
15.60723	19563.21	15.60563	19556.73	15.61088	19555.78	15.60214	19555.79	15.61452	19554.62	15.61088	19556.67	17.91536
15.85216	19562.48	15.85304	19556.93	15.8568	19554.53	15.85451	19555.83	15.86219	19555.03	15.85981	19557.93	18.16177
16.10345	19562	16.10218	19555.37	16.10907	19558.5	16.10376	19554.14	16.11297	19556.4	16.10961	19557.81	18.41195

16.3532	19563.29	16.35458	19556.67	16.35816	19554.4	16.35403	19557.75	16.36373	19557.02	16.35868	19557.44	18.66159
16.60701	19562.69	16.60122	19556.17	16.61053	19554.4	16.6012	19554.91	16.61591	19553.11	16.61179	19555.4	18.91385
16.85139	19561.34	16.85343	19553.2	16.85719	19554.29	16.85408	19555.74	16.86284	19556.72	16.86451	19555.05	19.16115
17.10254	19562.43	17.10302	19553.05	17.10621	19554.7	17.10184	19556.78	17.11173	19553.18	17.11325	19557.26	19.41147
17.35187	19562.33	17.35601	19553.98	17.35735	19555.69	17.35523	19553.97	17.36275	19557.49	17.35588	19556.63	19.66165
17.60897	19562.88	17.60189	19555.55	17.60912	19555.49	17.60291	19555.85	17.61538	19555.26	17.60993	19558.61	19.91322
17.85364	19561.42	17.85342	19555.08	17.85757	19553.66	17.85414	19555.63	17.86269	19555.02	17.87185	19556.43	20.16281
18.10501	19562.53	18.1032	19554.89	18.10688	19557.99	18.10171	19553.68	18.11355	19557.5	18.11388	19554.84	20.41422
18.35212	19561.17	18.35591	19552.26	18.36231	19557.28	18.35493	19554.35	18.3633	19557	18.35902	19553.68	20.66872
18.60843	19563.12	18.6023	19553.61	18.61136	19556.52	18.60385	19556.46	18.61468	19555.67	18.61111	19557.64	20.91426
18.85437	19562.97	18.85264	19554.93	18.85773	19555.36	18.85498	19556.91	18.86258	19554.78	18.85742	19555.55	21.16413
19.10467	19561.65	19.10392	19554.62	19.10806	19553.81	19.10191	19553.1	19.11459	19557.6	19.11151	19556.9	21.41442
19.35176	19560.24	19.35713	19556.56	19.35648	19555.77	19.35589	19556.49	19.36331	19553.55	19.35803	19557.06	21.66263
19.60764	19562.08	19.60234	19554.63	19.61005	19557.19	19.60361	19555.41	19.61586	19554.91	19.61203	19557.41	21.91325
19.85363	19561.18	19.8534	19554.19	19.85865	19554.92	19.85542	19554.29	19.86268	19557.32	19.85848	19558.01	22.16394
20.10502	19562.06	20.10483	19556.77	20.10913	19556	20.10397	19555.5	20.11248	19554.33	20.11067	19554.87	22.41514
20.35354	19560.95	20.35946	19558.63	20.3588	19555.25	20.35597	19556.21	20.3658	19555.84	20.37436	19555.94	22.66399
20.60842	19564.79	20.60522	19553.49	20.60975	19555.1	20.60511	19555.53	20.61755	19556.03	20.62016	19557.53	22.91465
20.85476	19562.68	20.85389	19553.28	20.86578	19555.36	20.85836	19558.63	20.86279	19554.13	20.86547	19557.13	23.16376
21.10626	19560.59	21.1052	19556.74	21.10874	19552.99	21.10503	19554.92	21.11406	19555.4	21.13597	19554.34	23.41722
21.35464	19562.52	21.35947	19556.4	21.35937	19555.04	21.3576	19556.98	21.36511	19554.34	21.36517	19558.31	23.66516
21.60973	19559.54	21.60604	19552.04	21.61326	19553.33	21.60419	19554.49	21.61788	19556.72	21.60656	19556.27	23.9154
21.85603	19563.45	21.85596	19556.17	21.86248	19557.75	21.85615	19553.38	21.8645	19555.24	21.86253	19556.08	24.16731
22.10609	19560.46	22.10506	19554.71	22.10854	19556.46	22.10474	19555.6	22.11294	19557.96	22.12173	19557.52	24.41544
22.35546	19561.77	22.35746	19557.01	22.3604	19554.21	22.35763	19556.55	22.36584	19554.61	22.35531	19559.25	24.66307
22.61151	19561.99	22.62628	19553.66	22.61243	19555.54	22.6062	19553.47	22.62028	19555.75	22.62045	19554.83	24.91518
22.85469	19561.48	22.85622	19555.98	22.86095	19554.94	22.85734	19555.32	22.86674	19557.67	22.86617	19555.08	25.16312
23.10709	19564.67	23.1068	19554.72	23.10875	19555.44	23.10428	19554.89	23.11456	19555.6	23.10787	19556.78	25.41397
23.35526	19561.76	23.36015	19556.45	23.35878	19555.44	23.35764	19553.89	23.36504	19556	23.35601	19554.19	25.66353
23.61145	19562.9	23.60641	19555.7	23.61369	19556.78	23.60573	19553.82	23.61909	19554.51	23.61946	19557.06	25.91539
23.85537	19562.28	23.85639	19552.32	23.86129	19554.55	23.8582	19557.04	23.86716	19556.28	23.86004	19557.16	26.16347
24.10735	19562.44	24.10781	19553.5	24.11099	19554.6	24.10662	19555.52	24.11744	19557.54	24.11225	19558.23	26.41362
24.35516	19563.61	24.35939	19555.41	24.35979	19557.46	24.35838	19555.43	24.36614	19555.14	24.35875	19557.03	26.66388
24.61173	19561.41	24.60459	19555.21	24.61086	19555.88	24.60554	19554.41	24.61936	19555.2	24.60865	19555.26	26.91575
24.85877	19560.71	24.85673	19555.44	24.86001	19554.71	24.86093	19555.71	24.86477	19557.31	24.86335	19558.74	27.16461
25.10742	19561.22	25.1062	19556.34	25.11057	19555.85	25.10575	19554.2	25.11674	19557.07	25.11601	19558.11	27.41625
25.35466	19561.26	25.36069	19556.36	25.3616	19556.41	25.35822	19555.36	25.36787	19554.69	25.35674	19554.59	27.66291
25.61092	19562.24	25.60622	19555.79	25.61155	19553.46	25.60403	19556.19	25.61953	19555	25.60949	19556.28	27.91397
25.85606	19561.97	25.85883	19554.11	25.86117	19555.56	25.858	19553.03	25.87819	19555.12	25.86367	19556.6	28.16451
26.10918	19562.41	26.1059	19554.69	26.11112	19555.64	26.10548	19555.08	26.11871	19555.19	26.1087	19555.31	28.4207
26.35451	19561.68	26.36053	19558.33	26.36047	19557.26	26.35761	19553.54	26.36626	19555.33	26.36213	19556.54	28.66535
26.61219	19561.28	26.60645	19552.83	26.61206	19556.56	26.60443	19554.24	26.61974	19555.67	26.61171	19558.66	28.91496
26.85628	19562.84	26.85584	19554.22	26.86205	19555.41	26.85754	19554.24	26.86648	19554.43	26.86178	19556.66	29.16636
27.10902	19563.65	27.10629	19555.84	27.11164	19554.45	27.10516	19554.22	27.11582	19555.29	27.12029	19555.77	29.41543
27.35532	19560.72	27.36602	19555.63	27.3619	19556.04	27.35851	19555.74	27.36627	19556.05	27.356	19556.45	29.66589
27.61271	19561.34	27.60936	19553.91	27.61419	19558.21	27.60593	19555.73	27.6207	19555.7	27.61034	19555.65	29.91697
27.85556	19563.39	27.85712	19556.58	27.86057	19556.83	27.85793	19554.53	27.86856	19554.67	27.86347	19556.91	30.16424
28.10901	19563.93	28.106	19556.35	28.11027	19555.12	28.10468	19554.73	28.11773	19554.33	28.11852	19555.92	30.4162
28.35655	19561.7	28.35982	19555.18	28.36132	19556.59	28.35878	19557.75	28.36752	19556.98	28.35783	19558.52	30.66609
28.6144	19559.86	28.60784	19555.06	28.61224	19553.86	28.60586	19555.25	28.61996	19553.26	28.61896	19556.99	30.91827
28.8558	19562.79	28.85802	19556.17	28.8627	19555.32	28.85886	19554.67	28.86817	19556.86	28.85602	19558.71	31.16684
29.10935	19562.05	29.1081	19556.1	29.11075	19554.71	29.10695	19557.68	29.12013	19556.74	29.11188	19554.61	31.4204
29.35746	19561.27	29.35938	19553.89	29.36148	19553.31	29.35904	19556.6	29.36828	19554.02	29.36104	19558.04	31.66494
29.61362	19562.25	29.60577	19553.62	29.61491	19557.39	29.60572	19555.61	29.62044	19555.94	29.61592	19557.66	31.91669
29.85711	19560.86	29.85662	19552.1	29.86234	19554.11	29.85896	19556.97	29.86789	19555.4	29.85909	19558.13	32.16787
30.10963	19562.5	30.10875	19555.15	30.1127	19556.57	30.10715	19554.68	30.11809	19555.05	30.11188	19556.19	32.41873
30.35858	19562.06	30.36381	19552.51	30.36194	19557.58	30.3608	19553.83	30.36981	19555.51	30.36063	19557.48	32.66465
30.61483	19563.65	30.60801	19555.4	30.6141	19554.93	30.60728	19553.99	30.62414	19556.4	30.61597	19555.61	32.91623
30.85885	19561.74	30.85748	19555.18	30.86292	19557.26	30.85942	19555.02	30.86871	19555.3	30.86248	19555.83	33.17055
31.11003	19561.8	31.10797	19555.04	31.11327	19555.84	31.10809	19556.29	31.12167	19557.26	31.12155	19558.49	33.42547
31.35742	19561.9	31.36727	19556.13	31.36326	19555.75	31.36103	19556.54	31.37224	19555.34	31.3573	19556.63	33.66764
31.61423	19562.81	31.61175	19555.15	31.61319	19554.37	31.60896	19556.63	31.62257	19557.61	31.61987	19555.74	33.91897
31.86077	19563.35	31.85929	19555.29	31.87254	19556.84	31.86043	19554.33	31.87031	19556.77	31.86733	19557.04	34.16485
32.11168	19562.55	32.10885	19556.73	32.11353	19557.17	32.10678	19555.72	32.12084	19556.35	32.11584	19557.45	34.41755
32.35805	19561.69	32.36171	19555.31	32.36357	19556.24	32.36105	19556.63	32.37176	19557.09	32.36458	19554.7	34.66666
32.61459	19561.19	32.60861	19556.65	32.61473	19553.27	32.60749	19555.23	32.62389	19556.11	32.615	19557.27	34.91884
32.85925	19561.25	32.85872	19550.77	32.8617	19556.34	32.86037	19554.93	32.86896	19552.29	32.86652	19554.42	35.16655
33.11099	19561.87	33.10987	19557.09	33.11312	19558.1	33.10696	19554.35	33.11771	19557.05	33.11579	19555.56	35.41711
33.35814	19562.58	33.36164	19554.01	33.36268	19557.27	33.36155	19554.5	33.3709	19554.88	33.36496	19556.88	35.66635
33.61377	19563.79	33.60821	19555.55	33.61523	19553.65	33.60751	19554.72	33.62561	19556.59	33.61664	19555.9	35.91897

33.85858	19558.5	33.85824	19555.34	33.8618	19555.46	33.86048	19556.1	33.87091	19554.84	33.86591	19555.98	36.16675
34.11077	19563.14	34.11019	19555.78	34.11175	19554.1	34.10967	19554.79	34.11882	19554.24	34.11662	19556.83	36.4168
34.35868	19563.56	34.36451	19555.56	34.36316	19558.87	34.36356	19554.56	34.37082	19555.96	34.36626	19558.08	36.66677
34.61544	19561.86	34.60844	19555.45	34.61487	19553.87	34.60873	19556.24	34.63267	19555.43	34.6188	19555.42	36.91939
34.85783	19562.33	34.85895	19553.95	34.8641	19556.39	34.86096	19556.61	34.87222	19556.44	34.86622	19553.8	37.16747
35.1111	19563.33	35.10949	19555.31	35.11279	19554.13	35.10916	19557.55	35.1233	19558.62	35.11886	19557.22	37.41814
35.36027	19561.19	35.36309	19555.67	35.36249	19554.67	35.3623	19557.49	35.36878	19557.39	35.36488	19556.39	37.6664
35.61555	19562.09	35.61079	19555.99	35.61308	19557.58	35.60914	19557.23	35.62707	19556.1	35.61768	19559.2	37.91985
35.85806	19560.41	35.85863	19553.31	35.86391	19557.3	35.86137	19555.88	35.87104	19554.76	35.86529	19556.88	38.16786
36.11056	19561.48	36.10951	19555.68	36.11465	19554.97	36.108	19556.11	36.12074	19554.28	36.11881	19559.53	38.42119
36.35941	19561.09	36.36638	19554.04	36.36478	19554.97	36.3614	19555.45	36.37121	19555.5	36.36547	19557.51	38.66813
36.61929	19561.85	36.61116	19554.48	36.61503	19554.47	36.60966	19555.16	36.63045	19556.41	36.61676	19554.88	38.91778
36.86646	19561.88	36.86013	19553.93	36.8656	19556.8	36.8629	19555.53	36.86898	19557.72	36.86644	19554.48	39.16685
37.11061	19560.45	37.10949	19554.28	37.11418	19556.17	37.10815	19554.28	37.11995	19557.33	37.11959	19555.76	39.42636
37.35791	19560.72	37.36508	19553.54	37.36454	19554.01	37.3621	19556.41	37.37098	19557.35	37.36746	19556.09	39.66969
37.61819	19561.16	37.61062	19553.87	37.61715	19556.18	37.61066	19553.67	37.62679	19556.35	37.61803	19555.99	39.91811
37.86151	19562.24	37.86106	19556.53	37.86389	19554.71	37.86338	19554.87	37.87287	19555.63	37.86454	19558.22	40.16732
38.11225	19561.65	38.11264	19555.98	38.11304	19555	38.10962	19555.22	38.1244	19555.86	38.11787	19555.87	40.42177
38.35878	19561.79	38.36488	19556.38	38.36433	19553.75	38.36193	19556.69	38.37138	19555.12	38.36849	19557.3	40.67044
38.61553	19562.85	38.6098	19556.81	38.61669	19554.94	38.60933	19554.58	38.62948	19554.86	38.62065	19557.08	40.92229
38.8603	19561.7	38.86132	19556.19	38.86522	19554.78	38.86403	19555.32	38.87233	19555.7	38.86733	19559.42	41.16961
39.11183	19562.44	39.1116	19554.69	39.11424	19556.27	39.11039	19554.91	39.12212	19556.56	39.1189	19555.97	41.42064
39.35975	19563.24	39.36581	19553.79	39.36379	19556.08	39.36415	19555.3	39.37126	19554.45	39.36688	19552.64	41.66987
39.62938	19562.13	39.61186	19554.33	39.61578	19556.44	39.60922	19555.67	39.62986	19557.15	39.62174	19553.07	41.92198
39.8656	19562.8	39.86063	19552.63	39.86639	19558.13	39.86468	19557.2	39.87118	19556	39.86689	19557.17	42.17
40.11295	19561.81	40.11168	19553.89	40.11644	19555.16	40.11184	19554.37	40.12187	19555.98	40.12214	19556.23	42.41957
40.3616	19561.24	40.36673	19557.25	40.36588	19557.65	40.36484	19554.87	40.3756	19554.14	40.36194	19556.02	42.66947
40.6207	19560.82	40.63135	19555.38	40.61606	19556.17	40.61071	19556.4	40.62998	19556	40.62141	19558.73	42.92159
40.86125	19563.55	40.86223	19552.87	40.86673	19555.96	40.86443	19557.88	40.8727	19553.93	40.86952	19557.74	43.17008
41.1141	19562.74	41.11241	19554.56	41.1177	19557.71	41.11146	19553.38	41.12478	19555.08	41.12133	19555.74	43.42499
41.36216	19561.76	41.36726	19554.73	41.36861	19556.47	41.36467	19556.05	41.37523	19558.26	41.36738	19556.06	43.66936
41.61863	19562.44	41.61314	19554.39	41.61687	19554.63	41.612	19556.03	41.63177	19555.11	41.62204	19558.6	43.92789
41.86396	19559.89	41.86233	19554.86	41.86743	19556.83	41.86518	19557.29	41.875	19557.33	41.86992	19554.48	44.17007
42.11366	19564.77	42.11218	19555.08	42.11664	19555.47	42.11239	19556.95	42.12409	19556.04	42.12123	19555.54	44.42554
42.3621	19562.24	42.3677	19554.13	42.37813	19558.69	42.36537	19554.68	42.37656	19557.39	42.37141	19558.1	44.67715
42.62014	19564.52	42.61323	19553.48	42.6184	19554.91	42.61325	19555.04	42.63159	19555.81	42.62204	19556.93	44.92123
42.86605	19563.37	42.86392	19554.41	42.86719	19554.37	42.86819	19554.86	42.87558	19556.28	42.87017	19558.6	45.17164
43.11551	19563.48	43.11428	19553.17	43.11686	19555.16	43.1135	19557.13	43.12435	19557.82	43.1224	19555.32	45.42897
43.36199	19562.86	43.36762	19556.56	43.36691	19557.66	43.3647	19554.51	43.37498	19556.17	43.3708	19556.01	45.67175
43.6211	19560.45	43.61305	19556.32	43.61803	19554.7	43.62918	19553.17	43.6318	19556.1	43.62221	19556.08	45.92299
43.86494	19562.63	43.86443	19555.66	43.86881	19554.23	43.86661	19552.35	43.87507	19555.17	43.87077	19557.91	46.16994
44.11552	19561.76	44.11424	19555.3	44.11849	19557.24	44.11355	19554.11	44.11945	19556.26	44.12899	19556.1	46.42241
44.36438	19561.71	44.36961	19558.15	44.36655	19556.51	44.36707	19557.99	44.37547	19555	44.37027	19555.78	46.67154
44.62173	19563	44.6138	19557.04	44.61985	19554.59	44.61248	19553.51	44.62989	19555.73	44.62173	19557.08	46.92386
44.86434	19563.14	44.86364	19556.34	44.86855	19555.44	44.86641	19555.76	44.87421	19554.88	44.87261	19558.67	47.17181
45.11631	19562.93	45.11526	19556.27	45.11889	19557.25	45.11447	19557.34	45.12631	19556.1	45.12376	19557.76	47.42285
45.36404	19561.64	45.36994	19557.88	45.36868	19556.81	45.36649	19553.72	45.37572	19555.69	45.37222	19555.53	47.67114
45.62213	19561.39	45.61488	19554.63	45.61901	19555.85	45.61311	19553.89	45.62989	19554.93	45.62159	19554.33	47.92357
45.86439	19562.9	45.86319	19554.33	45.87875	19556.5	45.8664	19554.73	45.87544	19557.85	45.87294	19553.87	48.17244
46.11613	19563.07	46.11431	19556.25	46.11881	19555.85	46.11674	19553.32	46.12605	19555.87	46.12258	19556.45	48.42613
46.36412	19563.88	46.36926	19556.26	46.36924	19553.84	46.36768	19555.46	46.37601	19556.54	46.37134	19557.51	48.66881
46.62238	19561.83	46.6169	19555.43	46.61895	19555.95	46.61383	19555.33	46.63755	19557.38	46.62404	19556.42	48.92206
46.86576	19562.57	46.86427	19554.18	46.86984	19557.71	46.8663	19554.62	46.87563	19557.6	46.86998	19557.21	49.17121
47.11646	19559.82	47.11496	19554.18	47.11897	19556.9	47.11513	19553.85	47.12658	19555.18	47.12547	19554.81	49.42427
47.3656	19563.38	47.37105	19556.09	47.36955	19556.07	47.36811	19554.01	47.37438	19555.05	47.37175	19556.07	49.67053
47.62235	19560.49	47.61588	19553.69	47.6459	19557.36	47.61592	19555	47.62823	19554.54	47.62689	19557.17	49.92206
47.86626	19561.2	47.86545	19556.36	47.8688	19557.62	47.86845	19555.27	47.8772	19555.73	47.87105	19557.24	50.17149
48.1167	19559.7	48.11444	19556.71	48.12009	19557.46	48.11461	19552.83	48.12612	19555.17	48.12566	19556.9	50.42475
48.36572	19561.02	48.37232	19555.1	48.37088	19558.8	48.36754	19555.4	48.37662	19556.44	48.3705	19556.14	50.67189
48.64021	19562.87	48.61391	19553.85	48.61925	19556.53	48.61446	19555.7	48.63143	19556.28	48.62556	19555.2	50.92262
48.86442	19560.96	48.86519	19553.95	48.86825	19557.47	48.86684	19554.21	48.87652	19554.66	48.87241	19556.91	51.17109
49.11671	19561.85	49.11562	19553.68	49.11851	19555.74	49.11448	19555.08	49.11934	19557.07	49.12426	19555.73	51.42316
49.365	19562.18	49.37065	19555.17	49.36883	19555.39	49.36637	19557.4	49.37781	19556.56	49.37041	19557.46	51.67229
49.62449	19562.9	49.61678	19555.24	49.62204	19557.75	49.61628	19553.81	49.63721	19553.61	49.62651	19555.34	51.93753
49.86433	19562.32	49.86485	19557.47	49.86793	19554.46	49.86626	19554.32	49.87787	19555.34	49.87355	19557.44	52.17124
50.11746	19564.62	50.11539	19552.52	50.11837	19555.56	50.11481	19557.36	50.1269	19555.77	50.12624	19558.02	52.4273
50.36584	19563.3	50.37316	19554.88	50.36854	19557.92	50.36694	19556.24	50.37751	19556.98	50.37395	19556.88	52.6773
50.62393	19564.63	50.62183	19557.39	50.61985	19555.35	50.61367	19556.74	50.63465	19555.58	50.62661	19556.57	52.93086
50.86496	19563.67	50.86645	19555.72	50.86988	19555.82	50.86755	19555.83	50.87667	19556.9	50.87218	19558.19	53.17135
51.11775	19562.46	51.11422	19556.79	51.11925	19556.49	51.11532	19555.9	51.12457	19555.96	51.12393	19555.57	53.42349

51.36592	19561.48	51.37005	19554.39	51.36877	19555.24	51.36804	19559.43	51.37689	19556.4	51.37219	19555.66	53.67193
51.62618	19561.3	51.6145	19554.54	51.62001	19554.49	51.61382	19555.79	51.6315	19554.95	51.62694	19555.8	53.92507
51.86707	19561.42	51.86669	19555.84	51.87068	19556.15	51.86827	19556.25	51.87685	19557.88	51.87089	19554.83	54.17349
52.11749	19563.84	52.11632	19555.01	52.12197	19554.98	52.11466	19556.54	52.13191	19553.38	52.12637	19556.89	54.42262
52.36537	19562.44	52.37096	19553.57	52.37108	19554.21	52.36806	19555.9	52.37939	19556.54	52.37357	19554.99	54.67205
52.62768	19563.07	52.61647	19555.15	52.6192	19554.54	52.6156	19555.03	52.63623	19557.77	52.62684	19557.83	54.93216
52.86744	19561.85	52.86513	19553.12	52.86936	19555.44	52.86768	19556.99	52.87778	19558.1	52.87424	19557.35	55.17861
53.11828	19562.25	53.11615	19557.33	53.12113	19557.26	53.11533	19555.53	53.12976	19554.54	53.12379	19555.76	55.42808
53.3655	19564.02	53.37497	19554.13	53.37096	19555.31	53.36867	19555.67	53.38042	19554.62	53.37235	19558.37	55.67613
53.62596	19562.12	53.62246	19555.98	53.61965	19554.17	53.61603	19557.61	53.63641	19553.75	53.62772	19556.18	55.9245
53.86675	19560.96	53.866	19555.03	53.8693	19556.06	53.86939	19554.63	53.87775	19556.26	53.87282	19557.38	56.18181
54.12003	19563.68	54.11645	19555.86	54.12132	19555.67	54.11531	19555.35	54.1295	19557.05	54.12599	19556	56.42516
54.36604	19561.38	54.37071	19559.24	54.37131	19554.57	54.3696	19554.57	54.38029	19556.74	54.3736	19558.42	56.67321
54.62575	19560.36	54.61688	19556.22	54.62113	19554.76	54.618	19556.36	54.63551	19555.11	54.62681	19555.83	56.92338
54.86749	19561.74	54.86683	19554.95	54.87083	19554.76	54.86918	19555.25	54.87834	19556.6	54.87523	19555.42	57.17421
55.11762	19561.72	55.1159	19555.22	55.11972	19558.05	55.11716	19555.16	55.1283	19555.69	55.12602	19558.32	57.42585
55.36751	19563.76	55.37319	19554.45	55.3708	19557.76	55.36907	19555.78	55.37985	19553.71	55.37331	19555.29	57.67508
55.62627	19560.2	55.61672	19555.04	55.62156	19556.33	55.61617	19553.3	55.6419	19555.21	55.62748	19554.61	57.92534
55.86745	19563.44	55.86775	19553.97	55.87363	19553.6	55.86926	19553.91	55.8781	19555.98	55.87526	19556.78	58.17207
56.11861	19562.01	56.11775	19553.67	56.12109	19553.96	56.11762	19555.69	56.13006	19556.82	56.12553	19557.52	58.42419
56.36669	19561.8	56.37391	19554.66	56.3698	19557.53	56.371	19554.04	56.37575	19556.24	56.37581	19555.85	58.67376
56.627	19561.53	56.61612	19554.73	56.62138	19555.45	56.61622	19555.85	56.6456	19555.02	56.62641	19557.48	58.92603
56.86856	19560.97	56.86705	19553.89	56.87531	19553.55	56.86928	19554	56.87991	19556.03	56.87475	19555.78	59.17623
57.11945	19561.66	57.11816	19555.53	57.12248	19555.09	57.11813	19554.64	57.12963	19555.36	57.12593	19557.08	59.42426
57.3671	19562.86	57.37555	19554.94	57.37303	19553.93	57.37072	19556.23	57.38145	19555.04	57.3736	19558.13	59.67332
57.62622	19562.34	57.61614	19555.22	57.62064	19554.41	57.6179	19557	57.63299	19556.86	57.62663	19555.61	59.92493
57.8677	19562.92	57.86833	19555.21	57.8727	19556.81	57.86867	19554.47	57.87919	19556.63	57.87617	19556.91	60.17468
58.11943	19561.8	58.11919	19552.8	58.12296	19554.29	58.11708	19556.29	58.13238	19556.86	58.12555	19556.91	60.43536
58.3663	19562.27	58.37783	19557.08	58.37296	19555.13	58.36989	19556.82	58.37754	19554.91	58.37519	19555.61	60.67467
58.62666	19563.94	58.62616	19557.64	58.62281	19557.68	58.62022	19555.68	58.64011	19557.93	58.63058	19557.11	60.93693
58.86954	19561.86	58.86896	19556.33	58.87532	19556.3	58.87093	19553.51	58.88161	19555.57	58.87778	19556.15	61.17702
59.11877	19561.45	59.11777	19553.75	59.12132	19554.92	59.11851	19553.96	59.13257	19556.9	59.12825	19555.98	61.42627
59.36711	19561.48	59.37372	19554	59.37254	19556.79	59.37167	19553.12	59.38248	19558.12	59.37623	19556.62	61.6747
59.62645	19562.64	59.62004	19552.79	59.62351	19554.48	59.6181	19555.23	59.6382	19557.13	59.62773	19556.97	61.92695
59.87053	19562.06	59.87037	19555.31	59.87351	19557.35	59.8714	19554.29	59.88202	19555.03	59.87687	19556.23	62.17747
60.12162	19559.17	60.1179	19555.82	60.12156	19555.8	60.12046	19556.29	60.13083	19555.2	60.12805	19554.96	62.42889
60.36848	19562.08	60.37617	19557.21	60.37397	19559.21	60.37421	19555.97	60.38288	19556.07	60.37773	19555.65	62.67504
60.6292	19562.69	60.62027	19554.8	60.62486	19556.32	60.61874	19555.94	60.64049	19558.43	60.63167	19552.31	62.92672
60.87174	19562.7	60.8721	19554.41	60.87579	19557.04	60.8725	19552.8	60.8811	19558.13	60.87684	19556.94	63.17518
61.1233	19563.09	61.12178	19556.2	61.12419	19555.12	61.12075	19554.37	61.13334	19558.06	61.14636	19556.33	63.42989
61.36961	19562.46	61.37692	19558.15	61.37298	19553.31	61.37251	19555.97	61.3822	19553.11	61.37906	19552.56	63.67877
61.62947	19563.19	61.62015	19554.22	61.63116	19555.59	61.62066	19554.69	61.64172	19557.23	61.62961	19556.57	63.92777
61.87209	19561.13	61.87128	19553.4	61.8815	19558.09	61.87253	19556.68	61.88392	19555.13	61.87709	19557.73	64.17608
62.12424	19562.35	62.12201	19554.22	62.12516	19554.88	62.12043	19555.48	62.13315	19553.79	62.13202	19556.44	64.42994
62.36969	19560.85	62.37652	19557.29	62.37383	19554.96	62.38485	19557.27	62.38259	19554.84	62.37831	19554.66	64.67833
62.62784	19562.18	62.62128	19556.67	62.62499	19553.77	62.62917	19556.82	62.64141	19556.83	62.63277	19555.24	64.93028
62.87117	19561.62	62.8727	19551.89	62.88477	19557.16	62.87403	19553.06	62.88081	19555.78	62.87883	19558.63	65.19346
63.12343	19563.5	63.12163	19557.04	63.12636	19555.88	63.12271	19553.44	63.13137	19555	63.13271	19556.29	65.43802
63.37139	19560.65	63.37942	19554.99	63.38019	19555.49	63.3729	19555.11	63.38042	19554.11	63.38084	19556.68	65.67888
63.62889	19560.25	63.62224	19554.17	63.62487	19558.43	63.62094	19556.78	63.63794	19557.92	63.63264	19553.9	65.93175
63.87108	19561.76	63.87387	19553.85	63.87502	19553.31	63.87489	19554.63	63.87822	19555.37	63.87921	19556.57	66.17677
64.12352	19561.94	64.1222	19556.26	64.12568	19558.79	64.12352	19556	64.13439	19554.92	64.13194	19555.99	66.42607
64.37071	19563.33	64.37969	19553.37	64.37607	19555.1	64.37446	19553.52	64.38208	19552.74	64.37891	19556.52	66.67728
64.6294	19562.82	64.61993	19554.07	64.62449	19556.27	64.61971	19554.6	64.63664	19556.68	64.62908	19557.48	66.9274
64.87072	19561.79	64.87184	19555.08	64.87365	19554.4	64.87326	19552.67	64.8769	19556.19	64.87951	19557.48	67.17649
65.12312	19562.57	65.12192	19555.55	65.12372	19553.42	65.12117	19556.39	65.1287	19556.33	65.12952	19558.19	67.4261
65.3705	19562.43	65.37686	19555.03	65.37514	19556.03	65.37582	19554.48	65.37892	19555.44	65.37865	19557.1	67.67667
65.62561	19561.48	65.62021	19557.27	65.62503	19555.9	65.61921	19555.05	65.63617	19555.34	65.62867	19556.74	67.92819
65.87043	19564.02	65.87275	19558.29	65.87471	19557.82	65.87376	19555.89	65.87884	19556.91	65.87866	19555.18	68.17702
66.12447	19561.99	66.12185	19556.88	66.12454	19559.64	66.12212	19554	66.13264	19555.13	66.13038	19558	68.42789
66.37121	19561.31	66.37754	19554.43	66.37528	19555.72	66.37441	19556.52	66.37988	19556.54	66.37863	19557.99	68.67659
66.62735	19562.43	66.62063	19553.47	66.62804	19556.86	66.62131	19558.3	66.63686	19558.31	66.63142	19555.91	68.92649
66.87223	19561.79	66.87087	19554.65	66.87913	19554.94	66.87396	19556.24	66.88111	19556	66.87936	19555.84	69.17803
67.12271	19563.65	67.1222	19554.87	67.12625	19555.03	67.12054	19555.32	67.13149	19555.64	67.12995	19557.72	69.43337
67.37098	19561.89	67.38011	19553.77	67.37525	19557.26	67.37486	19557.13	67.3792	19557.4	67.37984	19557.72	69.67786
67.62783	19562.66	67.62958	19554.73	67.62709	19555	67.62126	19554.74	67.63703	19555.52	67.63181	19553.35	69.92952
67.87286	19563.38	67.87049	19557.27	67.88629	19555.82	67.87586	19555.5	67.88013	19552.41	67.87973	19558.06	70.17735
68.12451	19560.74	68.12094	19557.94	68.12676	19554.17	68.1217	19556.01	68.13183	19555.08	68.13202	19555.25	70.4322
68.36999	19562.15	68.3765	19555.37	68.37706	19557.03	68.37707	19555.2	68.3826	19554.96	68.37871	19557.03	70.67735
68.63022	19561.24	68.62191	19557.03	68.62566	19556.78	68.62219	19554.54	68.63742	19556.33	68.63125	19555.53	70.92755

68.87388	19562.19	68.87208	19556.59	68.87574	19554.41	68.87453	19556.19	68.87913	19554.7	68.87941	19558.59	71.17672
69.12373	19562.07	69.12249	19553.86	69.12604	19554.93	69.12447	19555.3	69.13248	19559.05	69.13241	19554.25	71.42919
69.37134	19561.79	69.3802	19555.46	69.37833	19554.57	69.37551	19556.7	69.38186	19556.18	69.37427	19558.8	71.68009
69.62884	19562.69	69.62218	19554.67	69.62741	19556.29	69.62098	19555.71	69.64137	19558.2	69.63275	19557.85	71.93287
69.8721	19564.14	69.8725	19553.8	69.87581	19556.12	69.87542	19553.63	69.88687	19557.83	69.88102	19554.7	72.17718
70.12476	19563.18	70.12412	19556.46	70.12706	19556.43	70.1239	19554.63	70.13253	19555.52	70.13485	19555.75	72.42822
70.37303	19563.44	70.37884	19556.23	70.3778	19557.1	70.37892	19556.62	70.3842	19556.04	70.38113	19556.53	72.67783
70.63129	19564.04	70.62229	19554.67	70.62768	19553.71	70.62134	19556.18	70.63518	19556.37	70.63513	19555.73	72.93069
70.87335	19564.68	70.87301	19553.59	70.87795	19557.16	70.87493	19556.04	70.88228	19554.94	70.88297	19556.17	73.17722
71.12524	19560.64	71.12411	19556.11	71.12576	19553.45	71.12262	19555.03	71.13253	19555.21	71.13551	19555.78	73.43168
71.37295	19563.43	71.37882	19554.98	71.37703	19558.07	71.37918	19554.75	71.39343	19554.68	71.37717	19556.99	73.6772
71.63271	19561.32	71.62235	19554.78	71.63486	19556.16	71.62337	19557.41	71.63687	19555.95	71.63295	19556.19	73.93068
71.8741	19562.33	71.87236	19554.76	71.88084	19555.81	71.87504	19555.35	71.88137	19556.15	71.88254	19558.1	74.17914
72.12669	19563.26	72.12382	19555.94	72.12765	19554.7	72.12312	19557.18	72.13198	19555.71	72.13523	19555.71	74.43088
72.37355	19562.3	72.38218	19555.9	72.37599	19554.86	72.37647	19554.88	72.38217	19553.04	72.3826	19554.97	74.67819
72.63014	19562.14	72.63145	19557.15	72.62727	19554.32	72.62301	19556.05	72.63916	19557.26	72.63371	19558.04	74.9293
72.8754	19563.3	72.87232	19554.49	72.87663	19556.46	72.8769	19555.61	72.88336	19556.85	72.88212	19555.69	75.1789
73.12616	19562.22	73.12405	19553	73.12969	19554.97	73.12195	19555.45	73.13332	19554.3	73.13462	19554.76	75.43187
73.37313	19562.09	73.38012	19554.82	73.38039	19555.65	73.37557	19553.65	73.38493	19555.15	73.38076	19557.3	75.67983
73.63304	19561.42	73.62457	19555.04	73.62732	19553.32	73.62401	19554.46	73.64101	19558.85	73.63432	19555.94	75.93086
73.87488	19562.55	73.87435	19554.77	73.878	19555.16	73.8769	19556.2	73.88428	19554.83	73.88411	19555.35	76.17843
74.1266	19563.47	74.12291	19553.46	74.12904	19556.09	74.12446	19556.41	74.13745	19556.14	74.13471	19559.74	76.43223
74.37284	19562.81	74.38021	19553.35	74.38137	19555.81	74.37956	19557.2	74.38373	19555.62	74.38116	19555.09	76.67945
74.63475	19563.51	74.62323	19555.58	74.63	19558.17	74.62359	19554.54	74.64291	19555.09	74.63397	19557.63	76.92878
74.87649	19563.04	74.87429	19554.01	74.87896	19555	74.87711	19554.7	74.88106	19556.27	74.88442	19554.7	77.17766
75.12769	19560.64	75.12548	19557.11	75.12881	19553.93	75.12552	19555.69	75.13302	19555.51	75.13577	19555.53	77.43136
75.37423	19561.24	75.38242	19553.89	75.38079	19556.07	75.37619	19552.95	75.38225	19555.61	75.38294	19556.37	77.68096
75.631	19561.95	75.64755	19554.22	75.63043	19554.95	75.62311	19553.2	75.63758	19553.5	75.63426	19556.03	77.9351
75.8756	19561.84	75.87474	19555.76	75.87999	19554.87	75.8777	19553.76	75.88314	19556.89	75.88265	19558.06	78.18264
76.12806	19562.39	76.12679	19555.84	76.12922	19555.16	76.1262	19554.04	76.13112	19554.32	76.13721	19554.75	78.43232
76.37633	19562.94	76.38196	19554.48	76.3788	19556.55	76.38107	19556.67	76.38444	19555	76.3811	19555.47	78.68042
76.63087	19562.29	76.63312	19556.15	76.63014	19556.11	76.62259	19555.71	76.6444	19556.41	76.63307	19554.56	78.93345
76.87563	19561.39	76.87375	19556.16	76.88375	19554.18	76.87694	19554.66	76.88579	19555.18	76.88408	19555.06	79.17947
77.1316	19561.79	77.12584	19556.87	77.13049	19553.77	77.12535	19556.13	77.13434	19556.32	77.13471	19554.08	79.42954
77.37479	19561.24	77.382	19553.47	77.37933	19555.38	77.38168	19555.43	77.38585	19555.15	77.38386	19557.54	79.6799
77.63474	19564.16	77.62546	19552.29	77.63007	19555.17	77.62437	19554.05	77.64052	19556.74	77.63566	19557.34	79.93201
77.87575	19561.77	77.87491	19556.22	77.89291	19553.45	77.87821	19555.86	77.88562	19558.63	77.88405	19557.39	80.18123
78.12813	19561.94	78.12467	19553.6	78.1319	19554.72	78.12581	19553.13	78.1364	19556.75	78.1362	19560.37	80.43417
78.37617	19563.97	78.3829	19553.33	78.38177	19553.46	78.37907	19554.13	78.38556	19557.73	78.37905	19558.21	80.68025
78.63528	19562.13	78.62832	19557.1	78.632	19555.53	78.62599	19555.65	78.64565	19555.82	78.63606	19553.04	80.93348
78.87629	19562.48	78.87723	19553.63	78.88287	19554.7	78.8782	19556.09	78.8845	19558.22	78.88626	19558.54	81.19053
79.12926	19562.38	79.12604	19553.41	79.13277	19555.75	79.13711	19555.39	79.13571	19554.37	79.14264	19557.01	81.43248
79.37503	19560.76	79.38064	19555.69	79.38215	19554.51	79.38013	19553.62	79.38539	19554.62	79.38418	19557.72	81.68059
79.63336	19560.63	79.62633	19553.83	79.63195	19554.67	79.62694	19556.05	79.64221	19557.9	79.63703	19555.88	81.93216
79.87746	19560.53	79.87616	19554.77	79.88181	19551.91	79.88137	19555.8	79.88487	19557.48	79.8858	19557.01	82.18094
80.13137	19561.46	80.12802	19554.62	80.1311	19555.76	80.12705	19555.79	80.13622	19555.16	80.13794	19560.21	82.43545
80.3763	19563.03	80.38386	19553.89	80.38575	19555.88	80.38223	19557.08	80.38995	19557.66	80.38583	19554.45	82.68111
80.63372	19562.78	80.6364	19554.27	80.63201	19555.82	80.62575	19555.37	80.64487	19556.14	80.63819	19557.04	82.93194
80.87762	19561.93	80.87504	19554.93	80.88348	19556.75	80.87844	19555.44	80.88458	19555.11	80.88332	19552.98	83.18086
81.13018	19562.13	81.12692	19554.22	81.13318	19557.32	81.12791	19554.33	81.1358	19556.61	81.13721	19556.62	83.43316
81.37467	19562.35	81.3816	19554.33	81.38231	19555.6	81.38248	19556.6	81.38592	19555.2	81.38401	19554.36	83.68291
81.63472	19563.6	81.62925	19554.88	81.63062	19556.73	81.62713	19554.67	81.64474	19555.37	81.6362	19555.79	83.93417
81.88289	19563.13	81.87669	19555.51	81.88386	19553.17	81.87887	19553.06	81.88262	19558.16	81.88484	19558.72	84.1813
82.12968	19560.99	82.12772	19552.91	82.13275	19556.36	82.12696	19554.3	82.13363	19558.62	82.13744	19557.06	84.43346
82.378	19564	82.38265	19555.65	82.38284	19554.91	82.38124	19554.16	82.38732	19557.29	82.39111	19556.72	84.68219
82.63558	19561.16	82.62859	19552.99	82.63045	19554.7	82.62748	19554.72	82.64606	19554.52	82.63682	19555.49	84.93651
82.87715	19561.76	82.87829	19552.73	82.88161	19554.03	82.88012	19556.2	82.88596	19553.74	82.88337	19556.46	85.18131
83.12922	19560.56	83.12875	19553.16	83.13206	19554.47	83.12878	19553.71	83.13627	19555.78	83.1349	19557.45	85.43191
83.3778	19559.18	83.38298	19554.66	83.38303	19557.14	83.38187	19557.15	83.38783	19553.47	83.38318	19557.15	85.68173
83.63736	19561.61	83.62919	19556.41	83.63225	19553.87	83.62763	19554.64	83.6435	19557.32	83.63414	19555.98	85.94397
83.87805	19560.69	83.87925	19555.75	83.88051	19556.46	83.88348	19554.9	83.88752	19556.92	83.88478	19558.15	86.18442
84.1304	19561.01	84.12921	19555.2	84.13189	19552.95	84.12856	19555.46	84.13896	19554.64	84.13344	19556.03	86.43426
84.37821	19561.32	84.38298	19554.49	84.38342	19556.88	84.38333	19554.91	84.38743	19554.43	84.3811	19555.19	86.6816
84.63693	19561.96	84.62927	19554.74	84.63276	19554.37	84.62666	19555.94	84.6451	19553.65	84.63493	19553.67	86.93579
84.88517	19561.06	84.87967	19555.02	84.88416	19554.53	84.88221	19553.79	84.88911	19554.78	84.88284	19554.25	87.18339
85.1333	19561.78	85.13087	19558.64	85.13175	19553.41	85.13068	19555.59	85.13979	19554.65	85.13693	19557.62	87.43465
85.37692	19562.16	85.38201	19553.42	85.38288	19553.68	85.38159	19555.22	85.38904	19555.89	85.38426	19552.62	87.68165
85.63888	19560.26	85.62722	19552.65	85.63224	19555.26	85.62738	19555.28	85.64525	19555.33	85.63555	19555.16	87.93688
85.88154	19560.05	85.87886	19555.23	85.89413	19553.37	85.88662	19556	85.88566	19555.53	85.884	19557.06	88.18341
86.13096	19560.36	86.13276	19551.98	86.13293	19552.94	86.12963	19554.01	86.16709	19555.34	86.13658	19554.35	88.43901

86.37714	19561.42	86.38367	19555.17	86.38446	19555.6	86.38452	19553.53	86.38884	19552.19	86.38313	19556.69	88.68207
86.63503	19560.23	86.62879	19555.77	86.63225	19556.77	86.62752	19553.96	86.64535	19554.73	86.63598	19554.57	88.93494
86.88059	19560.38	86.87867	19551.58	86.88698	19552.32	86.88084	19554.28	86.8864	19553.69	86.88432	19555.69	89.18469
87.13072	19562.11	87.13142	19556	87.13427	19553.36	87.12856	19552.97	87.14066	19554.43	87.13724	19554.84	89.4374
87.3775	19560.63	87.38547	19553.64	87.38568	19553.67	87.3827	19555.08	87.38828	19552.68	87.38466	19555.28	89.68401
87.63746	19561.69	87.63032	19552.15	87.63394	19555.19	87.62968	19554.62	87.64495	19553.26	87.63634	19555.1	89.93576
87.88045	19561.56	87.87913	19555.15	87.884	19555.88	87.88256	19553.48	87.88686	19554.54	87.88618	19555.15	90.18263
88.13228	19560.89	88.13158	19552.9	88.13209	19554.65	88.13001	19553.41	88.13885	19553.73	88.13894	19555.16	90.43659
88.37909	19561.88	88.38536	19552.05	88.38502	19553.9	88.38155	19556.07	88.3896	19553.72	88.38364	19555.35	90.6856
88.63641	19561.3	88.63179	19553.78	88.63464	19553.56	88.63012	19555.05	88.64508	19554.97	88.6364	19554.58	90.93808
88.88099	19559.64	88.88069	19554.31	88.8851	19554.52	88.88342	19554.7	88.89036	19553.8	88.88573	19555.93	91.1828
89.13996	19560.32	89.13051	19554.85	89.13238	19555.11	89.13115	19555.31	89.13693	19553.59	89.13815	19554.84	91.4346
89.38779	19559.75	89.38605	19552.77	89.38453	19554.57	89.38347	19552.74	89.38825	19555.64	89.38271	19554.34	91.68559
89.63965	19562.36	89.62957	19554.12	89.63462	19556.2	89.62853	19552.81	89.64605	19553.58	89.6368	19555.88	91.93724
89.88233	19561.07	89.88049	19554.21	89.88532	19552.26	89.88344	19554.85	89.88988	19553.5	89.885	19556.56	92.18388
90.13138	19560.11	90.13103	19552.66	90.13566	19553.9	90.13101	19553.25	90.1423	19555.42	90.13811	19556.37	92.43466
90.37884	19559.81	90.38519	19551.75	90.38364	19553.35	90.38389	19554.71	90.39093	19553.17	90.3831	19552.79	92.68533
90.64114	19559.71	90.62906	19554.57	90.63472	19554.39	90.63135	19554.93	90.64733	19553.88	90.63886	19554.08	92.93791
90.88306	19562.34	90.8809	19552.33	90.88591	19554.54	90.88291	19553.58	90.88957	19555.07	90.88496	19552.62	93.18563
91.13248	19561.14	91.13152	19553.28	91.13618	19555.43	91.13445	19552.61	91.1413	19553.41	91.13917	19557.23	93.43761
91.38052	19561.86	91.38702	19554.04	91.38787	19551.86	91.38352	19552.79	91.39463	19553.71	91.38533	19552.92	93.68493
91.63864	19560.11	91.62973	19553.67	91.63573	19553.13	91.63248	19552.67	91.64759	19553.52	91.63781	19551.61	93.93656
91.88387	19558.01	91.88103	19551.75	91.89019	19553.85	91.88349	19552.63	91.88971	19553.78	91.88477	19552.49	94.1865
92.13299	19561.11	92.12994	19553.76	92.13505	19552.4	92.14257	19551.75	92.1442	19554.2	92.14145	19552.07	94.43716
92.37937	19558.87	92.38504	19551.58	92.38809	19554.53	92.38494	19552.17	92.40154	19552.91	92.38502	19554.21	94.68502
92.63801	19560.98	92.63171	19552.21	92.63729	19553.42	92.63187	19553.1	92.65065	19554.03	92.63911	19553.96	94.93755
92.88371	19560.82	92.8797	19552.24	92.88671	19551.86	92.88452	19553.66	92.89536	19554.25	92.88419	19554.34	95.18673
93.13496	19559.02	93.13231	19554.72	93.13486	19551.58	93.13233	19554.74	93.14476	19552.75	93.13856	19554.6	95.43819
93.38126	19558.59	93.38756	19551.35	93.38627	19553.72	93.3837	19553.77	93.39659	19553.53	93.38951	19552.87	95.68578
93.63691	19558.01	93.6333	19553.6	93.63717	19552.47	93.6319	19552.4	93.65048	19554.08	93.63893	19553.92	95.93771
93.88417	19561.14	93.88257	19552.04	93.89815	19552.73	93.88477	19551.81	93.89598	19552.27	93.88653	19551.1	96.18613
94.14172	19559.22	94.13138	19551.85	94.13663	19552.28	94.13441	19550.96	94.14588	19551.65	94.13993	19553.11	96.44037
94.38172	19557.72	94.38854	19551.91	94.38803	19551.94	94.3883	19553.85	94.39648	19551.42	94.38602	19556.12	96.68507
94.63779	19561.7	94.63304	19553.24	94.63538	19552.79	94.63171	19552.03	94.65162	19551.24	94.63848	19554.07	96.93835
94.88378	19557.15	94.88827	19552.2	94.88723	19552.62	94.8842	19554.05	94.89639	19552.15	94.88744	19552.06	97.18524
95.13572	19560.2	95.13372	19551.51	95.13595	19554.11	95.13528	19552.62	95.14741	19551.49	95.13975	19554	97.4404
95.38216	19559.11	95.38484	19552.1	95.38935	19553.03	95.38626	19551.94	95.39537	19553.86	95.38622	19554.35	97.68791
95.63992	19557.3	95.631	19553.32	95.63629	19552.6	95.63376	19552.44	95.65278	19553.76	95.63661	19552.69	97.93877
95.88277	19558.54	95.88349	19551.97	95.88808	19553.36	95.88548	19552.32	95.89552	19551.95	95.88423	19552.78	98.18509
96.13569	19558.66	96.13478	19552.33	96.13726	19553.86	96.14576	19551.83	96.14824	19552.9	96.14047	19550.73	98.43781
96.38314	19560.26	96.38758	19551.1	96.38964	19552.8	96.38802	19553.55	96.39715	19553.18	96.39032	19551.62	98.688
96.64053	19560.67	96.64598	19551.35	96.63924	19552.54	96.63568	19551.28	96.65241	19552.04	96.63989	19554.25	98.9386
96.88506	19559.41	96.88369	19550.88	96.89161	19551.24	96.88724	19555.34	96.89797	19552.31	96.88724	19554.2	99.18508
97.13484	19559.8	97.13277	19554.12	97.13684	19553.15	97.13679	19551.93	97.14431	19552.59	97.14146	19553.39	99.43707
97.38151	19556.87	97.3894	19551.36	97.39133	19553.24	97.38558	19551.26	97.39805	19552.95	97.38763	19552.67	99.68669
97.64196	19556.45	97.63375	19549.3	97.63872	19550.94	97.63328	19553.09	97.65341	19551.19	97.63996	19554.21	99.94011
97.88488	19558.63	97.88314	19551.09	97.88789	19551.54	97.88492	19554.13	97.90547	19552.24	97.88894	19553.34	100.1864
98.13693	19557.7	98.13391	19554.32	98.13706	19552.53	98.13605	19551.31	98.14929	19553.49	98.14153	19551.27	100.4392
98.38375	19558.15	98.39025	19549.81	98.38867	19549.3	98.38624	19551.48	98.3979	19550.11	98.38706	19550.99	100.6889
98.64077	19559.66	98.63578	19551.05	98.63801	19550.92	98.63199	19553.74	98.65277	19553.14	98.64059	19551.8	100.9417
98.88453	19558.44	98.88505	19550.83	98.89747	19550.27	98.88636	19552.96	98.8975	19552.34	98.8888	19553.14	101.1904
99.1415	19559.21	99.13787	19551.13	99.14266	19552.11	99.14151	19550.28	99.15376	19554.54	99.1492	19551.12	101.4434
99.38354	19559.15	99.39128	19551.59	99.38977	19553.03	99.38974	19551.54	99.39778	19550.92	99.38986	19555.15	101.6888
99.64823	19556.01	99.63484	19552.84	99.63702	19553.53	99.63363	19550.36	99.65253	19552.77	99.64099	19551.44	101.9423
99.89646	19557.54	99.88498	19552.89	99.89145	19551.47	99.88605	19550.53	99.89785	19549.32	99.89199	19550.42	102.1914
100.139	19558.1	100.1363	19551.42	100.1399	19552.29	100.1361	19552.01	100.1483	19552.64	100.1441	19549.81	102.442
100.386	19558.18	100.3919	19550.19	100.3925	19547.75	100.3914	19552.04	100.4017	19551.06	100.3909	19551.81	102.6886
100.6464	19559.64	100.6474	19552.68	100.6403	19551.04	100.6383	19551.31	100.6563	19552.46	100.6452	19552.12	102.9415
100.8882	19556.64	100.8988	19551.37	100.8928	19550.9	100.8897	19550.85	100.9003	19553.79	100.8917	19553.34	103.1923
101.1386	19557.99	101.1388	19551.18	101.1416	19549.59	101.1399	19551.47	101.1539	19550.49	101.1428	19554.55	103.4481
101.3861	19556.87	101.3945	19552.05	101.3939	19549.85	101.3898	19551.01	101.4005	19551.2	101.3913	19551.82	103.6886
101.6437	19560.55	101.6387	19550.65	101.6405	19551.41	101.6375	19551.4	101.6577	19552.38	101.6438	19550.38	103.9415
101.8887	19556.91	101.8862	19551.84	101.8943	19550.97	101.8919	19550.83	101.901	19551.17	101.8925	19552.81	104.1916
102.1419	19557.19	102.137	19552.61	102.1421	19551.35	102.1402	19549.83	102.1569	19552.1	102.1445	19551.68	104.4442
102.3855	19555.38	102.3941	19548.63	102.3963	19550.76	102.3919	19551.37	102.3998	19551.45	102.3916	19551.73	104.691
102.6445	19555.33	102.638	19550.44	102.642	19550.52	102.6411	19551.03	102.6558	19552.91	102.6465	19550.87	104.943
102.8881	19557.54	102.8874	19552.08	102.8943	19551.79	102.8947	19552.01	102.9011	19550.66	102.8931	19551.28	105.1899
103.1398	19555.05	103.1378	19549.99	103.1415	19551.5	103.1422	19550.21	103.1525	19552.2	103.1461	19550.67	105.4432
103.388	19559.5	103.3936	19551.81	103.3947	19549.38	103.3903	19550.88	103.4019	19552.5	103.3912	19552.3	105.6913
103.6462	19556.25	103.6378	19550.2	103.642	19550.2	103.6403	19550.38	103.657	19551.87	103.6452	19550.67	105.9442

103.8884	19556.95	103.8887	19550.78	103.8942	19550.57	103.906	19550.46	103.9032	19552.34	103.8927	19550.07	106.1917
104.1403	19557.56	104.1371	19551.47	104.1416	19549.21	104.1409	19548.97	104.1532	19551.05	104.1439	19551.14	106.4432
104.3886	19558.55	104.3917	19549.92	104.3927	19548.77	104.3926	19551.08	104.4038	19549.98	104.3928	19553.5	106.6923
104.6544	19556.18	104.6395	19548.82	104.6431	19551.15	104.6391	19551.62	104.6553	19551.05	104.6465	19549.62	106.944
104.8894	19557.62	104.8892	19548.69	104.8943	19549.89	104.8913	19550.08	104.9026	19550.66	104.8954	19551.62	107.1916
105.1413	19556.67	105.139	19549.94	105.1437	19550.13	105.1521	19549.77	105.1534	19547.84	105.1497	19550.88	107.445
105.3885	19555.74	105.3932	19551.47	105.3934	19548.84	105.3923	19551.23	105.4042	19550.12	105.3934	19553.82	107.6925
105.6477	19556.01	105.6495	19550.45	105.6409	19548.92	105.6385	19548.85	105.658	19551.98	105.6453	19550.69	107.9466
105.8921	19556.45	105.8878	19549.93	105.8968	19550.4	105.8944	19549.87	105.9052	19550.05	105.8897	19549.27	108.1927
106.1414	19558.68	106.1381	19547.67	106.1433	19549.48	106.1437	19551.37	106.1515	19549.68	106.1469	19549.69	108.4435
106.3883	19556.42	106.3948	19549.71	106.4003	19552.24	106.3944	19549.17	106.403	19552.1	106.3931	19550.31	108.6918
106.6481	19556.39	106.6393	19551.72	106.6442	19550.07	106.6411	19547.79	106.6588	19552.05	106.6482	19549.18	108.9459
106.8913	19555.42	106.8882	19550.26	106.8968	19550.4	106.8929	19551.43	106.9038	19551.53	106.895	19551.8	109.2063
107.1426	19555.91	107.1405	19551.54	107.1435	19549.18	107.143	19550.95	107.1536	19551.21	107.1475	19551.68	109.4454
107.3883	19555.56	107.3955	19549.55	107.3958	19549.06	107.3941	19548.67	107.4037	19549.24	107.3946	19550.47	109.6915
107.6512	19556.94	107.6401	19549.66	107.6447	19549.53	107.6391	19551.16	107.6614	19551.39	107.6476	19549.03	109.9445
107.8915	19554.46	107.8903	19550.45	107.9089	19549.59	107.8926	19548.64	107.9123	19549.91	107.895	19549	110.1938
108.1416	19555.94	108.1391	19549.33	108.1424	19549.33	108.1539	19550.55	108.1549	19549.86	108.1503	19549.8	110.4624
108.3896	19558.47	108.3963	19550.51	108.3957	19549.4	108.3974	19548.12	108.4035	19549.08	108.3949	19550.36	110.6915
108.6467	19557	108.6402	19551.12	108.646	19550.82	108.6417	19549.53	108.6613	19550.51	108.647	19548.38	110.9443
108.891	19555.55	108.8924	19548.47	108.9094	19550.96	108.8935	19549.82	108.9041	19551.33	108.8931	19549.8	111.1955
109.1427	19556.12	109.1414	19550.56	109.145	19548.69	109.1458	19548.61	109.1547	19550.2	109.1547	19549.7	111.4458
109.3899	19557.72	109.3955	19551.17	109.3954	19548.06	109.4112	19548.45	109.4068	19551.9	109.3966	19550.76	111.6927
109.6531	19555.22	109.6397	19548.09	109.6446	19549.96	109.6424	19551.35	109.6584	19549.31	109.6519	19548.41	111.9444
109.8929	19555.16	109.8919	19550.19	109.8969	19550.55	109.8937	19549.33	109.9037	19550.61	109.8961	19549.37	112.1952
110.1434	19555.25	110.1426	19548.46	110.1464	19550.86	110.1449	19548.27	110.1557	19549.96	110.1487	19551.01	112.4481
110.3894	19555.56	110.395	19548.51	110.3966	19550.25	110.3927	19548.49	110.4044	19549.7	110.395	19548.23	112.6952
110.6481	19554.84	110.6405	19548.58	110.6447	19548.44	110.64	19550.3	110.6612	19547.97	110.649	19549.59	112.9456
110.8924	19556.09	110.8922	19547.13	110.8977	19545.47	110.8944	19548.59	110.9068	19548.26	110.8983	19550.27	113.1943
111.1478	19555	111.1435	19548.13	111.1452	19547.39	111.1458	19547.14	111.1581	19550.6	111.1506	19548.46	113.4488
111.3897	19553.89	111.3948	19548.69	111.3985	19548.52	111.3957	19549.02	111.4058	19550.9	111.397	19548.98	113.6945
111.647	19557.05	111.6411	19550.04	111.6464	19548.89	111.6393	19549.66	111.6622	19547.49	111.6475	19549.46	113.9468
111.8934	19555.8	111.8906	19548.91	111.8997	19547.77	111.8955	19548.54	111.9058	19549.56	111.9004	19549.52	114.198
112.1458	19556.36	112.1433	19547.91	112.1458	19546.98	112.1485	19549.94	112.1738	19548.48	112.1542	19553.22	114.4471
112.3898	19554.14	112.3959	19548.79	112.3987	19547.16	112.396	19547.9	112.4101	19549.4	112.4003	19551.84	114.6949
112.6542	19554.64	112.6424	19548.69	112.6485	19547.57	112.6402	19547.52	112.6604	19549.58	112.6537	19550.4	114.9462
112.8943	19555.61	112.8909	19547.91	112.8988	19548.24	112.8951	19548.5	112.907	19549.05	112.9016	19547.78	115.197
113.1462	19556.83	113.1423	19547.78	113.1468	19547.29	113.1443	19547.67	113.1584	19552.17	113.1535	19551.47	115.4495
113.3906	19555.76	113.3972	19549.41	113.3977	19548.45	113.3953	19548.04	113.4079	19552.49	113.4035	19552.04	115.6937
113.6473	19555.81	113.6434	19549.67	113.6461	19546.85	113.6391	19548.91	113.6602	19548.13	113.6545	19549.81	115.9463
113.8935	19553.71	113.8933	19547.18	113.9018	19551.56	113.8955	19549	113.9059	19551.94	113.9105	19549.28	116.1965
114.1461	19556.52	114.1417	19545.59	114.147	19548.34	114.1446	19550.62	114.158	19548.7	114.1547	19551.16	116.4486
114.3919	19554.69	114.3991	19549.52	114.3993	19548.46	114.3988	19549.27	114.4092	19548.22	114.4034	19548.36	116.6961
114.6469	19554.01	114.6452	19548.68	114.6464	19548.66	114.6436	19549.47	114.6595	19547.97	114.6537	19550.73	116.9473
114.8949	19552.88	114.8939	19546.56	114.8996	19550.81	114.896	19548.41	114.9073	19550.09	114.9033	19549.15	117.1948
115.148	19553.86	115.1429	19546.35	115.1481	19551.09	115.1457	19548.47	115.1553	19548.92	115.1594	19549.98	117.4498
115.3934	19553.08	115.3983	19545.11	115.3993	19547.99	115.395	19549.12	115.4088	19548.95	115.4036	19548.16	117.6977
115.6502	19554.71	115.6458	19546.81	115.6491	19546.59	115.6426	19547.6	115.6644	19547.39	115.6552	19549.86	117.9543
115.894	19555.87	115.8954	19548.06	115.8987	19547.78	115.897	19550.38	115.9061	19552.19	115.9034	19547.92	118.1942
116.1474	19553.26	116.1454	19548.38	116.1483	19549.32	116.1467	19550.78	116.1575	19550.88	116.1572	19546.45	118.4517
116.3939	19556.57	116.3945	19549.04	116.4009	19547.57	116.3994	19546.17	116.4083	19547.37	116.4048	19549.79	118.696
116.6513	19557.44	116.6558	19548.4	116.6487	19546.93	116.6423	19547.35	116.6665	19548.57	116.6544	19546	118.9501
116.8942	19554.42	116.8938	19548.74	116.9035	19547.99	116.8971	19549.1	116.9084	19548.57	116.9048	19547.63	119.1954
117.1464	19554.19	117.145	19548.6	117.1516	19548.44	117.1495	19548.54	117.1575	19549.21	117.1536	19548.24	119.4493
117.3935	19555.06	117.3995	19549.39	117.4007	19548.35	117.403	19548.42	117.4099	19549.3	117.4044	19550.39	119.6964
117.6498	19556.8	117.6437	19547.01	117.6502	19546.91	117.6449	19547.95	117.6654	19549.36	117.6561	19549.03	119.9498
117.896	19553.68	117.8927	19545.7	117.9017	19547.75	117.8978	19548.84	117.9075	19549.91	117.9055	19547.28	120.1984
118.1482	19555.12	118.1448	19549.1	118.1494	19549.1	118.1584	19547.98	118.1598	19546.7	118.1552	19548.87	120.4521
118.3928	19553.07	118.3968	19547.98	118.3994	19550.59	118.4003	19547.11	118.4105	19548.74	118.4032	19547.59	120.6942
118.6537	19553.41	118.646	19550.64	118.6502	19548.02	118.6453	19546.5	118.6635	19551.12	118.6551	19549.99	120.9466
118.8963	19554.19	118.8955	19548.33	118.902	19549.46	118.8971	19546.93	118.9102	19548.65	118.9049	19548.65	121.2046
119.149	19555.08	119.1435	19550.34	119.1496	19549.64	119.1493	19547.46	119.1585	19548.45	119.1547	19549.25	121.4497
119.3931	19553.81	119.3968	19547.23	119.4002	19545.79	119.4021	19547.95	119.4087	19546.86	119.4065	19550.84	121.693
119.6494	19554.97	119.6459	19546.25	119.6488	19547.98	119.6444	19548.97	119.6649	19549.19	119.6569	19547.27	121.9473
119.8968	19554.09	119.8969	19546	119.9026	19545.7	119.898	19548.57	119.9107	19548.53	119.9053	19547.37	122.1968
120.1508	19553.23	120.1461	19547.3	120.1493	19548.73	120.1501	19549.24	120.1623	19548.9	120.1588	19549.92	122.4511
120.3937	19556.51	120.4014	19547.97	120.4009	19549.59	120.3982	19549.58	120.4122	19547.29	120.4056	19549.24	122.6944
120.6489	19552.94	120.6559	19549.02	120.6468	19549.19	120.6412	19547.88	120.6639	19546.52	120.6563	19550.61	122.9472
120.895	19552.38	120.8932	19547.18	120.9006	19547.54	120.8969	19548.43	120.9067	19550.19	120.9032	19549.91	123.1977
121.1456	19554.56	121.1431	19548.55	121.1479	19547.6	121.1482	19544.67	121.1597	19546.72	121.1585	19548.39	123.4482

121.393	19552.01	121.3967	19549.01	121.4005	19547.42	121.398	19548.15	121.4101	19549.91	121.4273	19549.15	123.6944
121.6524	19555.44	121.6464	19547.4	121.6515	19549.33	121.6409	19546.29	121.6631	19546.13	121.6572	19547.93	123.9473
121.8943	19553.58	121.8931	19548.14	121.902	19547.62	121.9	19545.71	121.9075	19548.1	121.9028	19546.66	124.1967
122.147	19554.93	122.1439	19545.42	122.1476	19547.82	122.1462	19547.45	122.1568	19545.41	122.1537	19549.3	124.4497
122.3934	19553.93	122.3976	19546.42	122.4018	19547.55	122.3988	19549.61	122.4089	19546.8	122.4009	19548.25	124.6953
122.6486	19555.14	122.6454	19549.02	122.6504	19546.6	122.6428	19548.05	122.662	19550.21	122.6547	19548.11	124.9492
122.8966	19554.15	122.8938	19546.8	122.9002	19546.89	122.8976	19546.46	122.908	19548.74	122.9045	19547.79	125.1965
123.1467	19551.98	123.1431	19550.54	123.1471	19545.88	123.1471	19546.25	123.161	19547.82	123.1544	19548.96	125.4504
123.3926	19554.63	123.3981	19548.22	123.4017	19549.13	123.3996	19549.8	123.4082	19548.72	123.4025	19547.87	125.6969
123.6504	19554.93	123.6467	19544.62	123.6511	19545.34	123.6449	19545.75	123.6655	19548.54	123.6585	19547.51	125.9481
123.8956	19555.56	123.8955	19548.44	123.9121	19545.25	123.898	19546.6	123.9083	19546.37	123.9054	19549.06	126.1971
124.1494	19553.27	124.1441	19546.74	124.1506	19547.15	124.1494	19548.02	124.158	19549.57	124.1566	19547.01	126.4504
124.3925	19551.05	124.3964	19548.05	124.3994	19548.85	124.3977	19547.89	124.4095	19548.11	124.4034	19548.95	126.6992
124.6504	19552.91	124.6476	19547.63	124.6507	19547.37	124.6443	19548.96	124.6634	19549.1	124.655	19545.97	126.9493
124.8961	19554.81	124.8967	19547.01	124.9016	19548.3	124.8994	19546.89	124.9097	19546.86	124.9047	19546.62	127.2039
125.1486	19551.69	125.1456	19547.16	125.1492	19547.04	125.1488	19549.51	125.1622	19547.81	125.1597	19549.42	127.4561
125.394	19552.07	125.3948	19546.4	125.4011	19547.85	125.397	19547.11	125.4081	19547.44	125.4059	19547.94	127.7109
125.6482	19553.75	125.6476	19545.15	125.6513	19546.06	125.6445	19546.9	125.6634	19548.33	125.6565	19545.73	127.9666
125.896	19554.92	125.8957	19547.1	125.901	19547.27	125.8982	19547.49	125.9074	19548.94	125.9064	19550.82	128.2127
126.1489	19554.41	126.1459	19548.04	126.1493	19548.6	126.1504	19546.6	126.161	19547.63	126.1566	19547.99	128.4746
126.394	19554.51	126.3991	19548.22	126.4031	19547.79	126.398	19548.17	126.4088	19547.56	126.4037	19549.13	128.7104
126.6503	19554.51	126.6489	19546.27	126.6502	19547.48	126.6471	19547.43	126.6641	19546.45	126.6558	19548.66	128.9624
126.8969	19552.45	126.8945	19547.08	126.9037	19545.75	126.8984	19547.71	126.9102	19548.7	126.906	19547.21	129.2098
127.1484	19555.32	127.1469	19548.11	127.1519	19548.72	127.1507	19546.65	127.1605	19547.71	127.1558	19546.41	129.4636
127.3943	19553.25	127.3978	19547.58	127.4026	19546.74	127.3999	19547.08	127.4111	19547.3	127.4046	19547.19	129.7116
127.6501	19553.31	127.6497	19545.41	127.6506	19547.71	127.6449	19545.55	127.667	19547.74	127.6578	19548.75	129.9628
127.8976	19551	127.8956	19546.11	127.9127	19545.98	127.8996	19547.83	127.9093	19550.63	127.906	19544.97	130.2112
128.1511	19554.05	128.1464	19547.21	128.1509	19547.72	128.1522	19548.68	128.1624	19549.25	128.1598	19547.05	130.4649
128.396	19553.27	128.3983	19546.52	128.4022	19547.67	128.3996	19548.5	128.4104	19547.04	128.4047	19546.66	130.7128
128.6515	19552.52	128.649	19546.06	128.6523	19547.37	128.6466	19544.5	128.6692	19548.07	128.6577	19546.11	130.9631
128.8977	19552.32	128.8968	19546.35	128.9079	19543.2	128.8996	19548.39	128.911	19546.8	128.9074	19547.19	131.2117
129.1479	19553.72	129.147	19547.41	129.1515	19547.16	129.1506	19547.47	129.1622	19547.77	129.1573	19548.18	131.4546
129.3949	19553.1	129.3991	19545.94	129.4017	19546.47	129.4002	19546.33	129.4127	19547.43	129.4056	19547.45	131.6986
129.6516	19551.63	129.6509	19544.65	129.6501	19546.02	129.6466	19549.25	129.6661	19546.39	129.6576	19546.67	131.9564
129.8971	19554.13	129.9014	19543.93	129.9036	19547.72	129.9017	19546.83	129.9113	19548.9	129.9167	19545.31	132.2055
130.1488	19553.07	130.147	19545.57	130.1512	19547.32	130.1519	19545.76	130.1635	19549.74	130.1591	19546.71	132.454
130.3958	19551.97	130.3989	19545.51	130.4043	19547.54	130.3992	19547.26	130.4108	19549.87	130.405	19548.31	132.6993
130.653	19552.98	130.6474	19546.91	130.651	19544.61	130.6465	19543.98	130.6641	19546.1	130.6602	19550.56	132.9525
130.8983	19551.79	130.8985	19547.77	130.9044	19548.76	130.905	19546.38	130.9115	19547.47	130.9083	19546.08	133.1981
131.153	19551.93	131.1487	19545.76	131.151	19548.27	131.1533	19547.2	131.1611	19547.47	131.1614	19546.88	133.4539
131.3996	19552.22	131.4008	19545.73	131.4065	19546.03	131.4005	19545.43	131.4115	19545.97	131.4067	19547.71	133.6991
131.6509	19553.8	131.6474	19546.52	131.6523	19545.48	131.6457	19546.32	131.6662	19547.28	131.6591	19547.01	133.9519
131.8994	19552.36	131.8984	19544.88	131.9113	19546.83	131.9012	19548.26	131.9113	19544.58	131.9084	19549.29	134.2001
132.1515	19554.77	132.1496	19547.8	132.1524	19545.71	132.1513	19547.51	132.1636	19546.5	132.1583	19547.3	134.4561
132.3969	19551.24	132.4013	19543.54	132.4029	19545.18	132.403	19547.25	132.4135	19547.38	132.408	19545.7	134.6993
132.653	19554.31	132.6501	19543.22	132.6525	19546.88	132.6472	19545.13	132.6673	19547.24	132.6575	19547.01	134.9532
132.9009	19551.56	132.8967	19546.09	132.907	19545.2	132.8993	19545.52	132.9109	19545.59	132.909	19545.81	135.1991
133.1517	19554.11	133.1485	19546.5	133.1534	19545.97	133.1523	19545.12	133.162	19543.13	133.1615	19546.5	135.4536
133.3992	19554.91	133.4011	19547.46	133.4056	19544.46	133.4007	19548.29	133.4155	19546.53	133.4088	19545.84	135.6982
133.6561	19552.45	133.6504	19544.86	133.6531	19547.23	133.648	19546.19	133.664	19545.86	133.6596	19547.8	135.9527
133.8996	19554.13	133.8993	19545.75	133.9082	19546.75	133.9013	19548.4	133.913	19544.55	133.9081	19546.08	136.2002
134.1516	19552.24	134.1489	19547.62	134.1533	19545.79	134.1533	19547.31	134.1628	19547.04	134.1626	19549.34	136.4569
134.4026	19551.73	134.4019	19546.36	134.4041	19544.55	134.406	19545.32	134.4156	19544.85	134.4105	19546.76	136.6997
134.6517	19553.05	134.6491	19547.12	134.6515	19546.04	134.6506	19546.99	134.6714	19549.28	134.6582	19546.97	136.9503
134.8995	19551.22	134.8997	19548.13	134.91	19544.41	134.9027	19543.76	134.9115	19549.02	134.91	19549.61	137.1986
135.1511	19551.34	135.1506	19548.92	135.1538	19547.65	135.1534	19547.18	135.1663	19547.29	135.1621	19544.22	137.4562
135.4003	19550.65	135.4005	19543.84	135.4059	19545.59	135.4017	19548.79	135.413	19546.97	135.4112	19548.77	137.7007
135.6543	19551.02	135.6638	19543.8	135.6551	19546.2	135.6486	19548.09	135.6699	19544.93	135.6665	19547.25	137.9549
135.9032	19552.53	135.9002	19543.43	135.906	19545.91	135.903	19544.18	135.9135	19548.18	135.9143	19546.85	138.1995
136.1516	19552.94	136.1492	19547	136.1528	19546.59	136.1574	19547.29	136.1643	19548.4	136.1682	19546.58	138.4561
136.401	19553.38	136.4018	19544.21	136.4069	19545.91	136.4046	19546.11	136.4143	19546.6	136.4093	19548	138.7007
136.6588	19553.59	136.6483	19545.02	136.6543	19543.08	136.6491	19547.25	136.6706	19545.98	136.6616	19548.06	138.9542
136.9012	19552.23	136.9003	19547.16	136.9108	19546.4	136.9049	19544.68	136.9146	19545.18	136.9099	19546.57	139.2011
137.1521	19552.49	137.1512	19546.65	137.1527	19544.63	137.1545	19547.22	137.1663	19547.76	137.1612	19546.28	139.4573
137.3998	19553.93	137.4016	19546.18	137.4069	19547.11	137.4065	19548.28	137.413	19545.99	137.4082	19543.96	139.6998
137.6506	19551.76	137.6496	19546.39	137.6542	19546.22	137.6486	19547.56	137.6726	19544.11	137.6639	19547.37	139.9532
137.9016	19552.26	137.899	19546.11	137.9214	19545.53	137.9043	19545.99	137.9142	19546.94	137.9083	19547.14	140.2038
138.1525	19551.45	138.1497	19543.96	138.1545	19547.35	138.1609	19545.76	138.1692	19546.98	138.1619	19546.4	140.4564
138.399	19555.01	138.4039	19545.88	138.405	19546.94	138.4083	19544.66	138.4151	19547.41	138.4099	19547.94	140.7102
138.6544	19550.92	138.6518	19545.82	138.6552	19547.35	138.6504	19546.04	138.67	19543.53	138.662	19546.94	140.9542

138.9138	19551.11	138.9004	19544.01	138.9094	19546.15	138.9031	19546.63	138.9142	19549.43	138.9118	19545.94	141.2014
139.1533	19554.37	139.1507	19546.68	139.1556	19542.82	139.1564	19546.31	139.1665	19546.83	139.1608	19545.88	141.4588
139.4011	19553.51	139.4014	19544.35	139.4077	19543.85	139.409	19547.96	139.4151	19548.48	139.4101	19547.72	141.7036
139.6588	19552.32	139.652	19545.95	139.6557	19547.04	139.6506	19546.29	139.6692	19546.28	139.6644	19547.36	141.9536
139.9021	19551.03	139.9031	19546.95	139.9103	19545.16	139.9062	19544.27	139.9142	19547.12	139.9125	19546.66	142.2022
140.1517	19553.02	140.1509	19545.27	140.1546	19546.97	140.1562	19544.1	140.1675	19546.02	140.1646	19545.34	142.4604
140.4009	19550.26	140.399	19546.56	140.4085	19546.98	140.4041	19546.8	140.415	19546.9	140.4112	19547.03	142.7149
140.6581	19553.21	140.6519	19543.86	140.6559	19544.63	140.6509	19547.1	140.671	19545.6	140.6655	19545.52	142.9602
140.9022	19551.83	140.902	19547.07	140.9187	19548.12	140.9038	19544.04	140.9159	19544.51	140.9125	19547.6	143.2022
141.1547	19551.76	141.1514	19547.19	141.1556	19545.09	141.1596	19548.51	141.1669	19546.51	141.1636	19548.34	143.4567
141.3991	19551.13	141.4027	19546.7	141.4076	19544.41	141.4034	19544.14	141.4162	19545.21	141.4117	19545.46	143.703
141.6561	19551.46	141.6512	19544.31	141.6558	19544.25	141.6497	19547.44	141.6706	19547.25	141.6634	19547.13	143.9554
141.9019	19553.85	141.9012	19547.34	141.9128	19544.82	141.9045	19545.49	141.9152	19547.02	141.9126	19545.92	144.2026
142.1544	19553.25	142.152	19542.24	142.1553	19546.44	142.1646	19544.08	142.1671	19546.84	142.1645	19547.87	144.4606
142.4018	19551.93	142.4038	19545.52	142.411	19547.93	142.4108	19544.8	142.4166	19546.36	142.4123	19547.47	144.7051
142.6568	19552.49	142.6529	19544.45	142.6568	19545.37	142.6519	19547.12	142.6711	19544.85	142.6625	19545.28	144.9563
142.9055	19553.75	142.9023	19542.55	142.913	19546.87	142.9054	19546.77	142.9157	19545.58	142.9144	19543.88	145.2037
143.1555	19552.7	143.1521	19545.53	143.1564	19547.85	143.1579	19544.15	143.1664	19543.57	143.1629	19546.55	145.4581
143.4018	19551.9	143.4044	19544.73	143.4096	19545.96	143.4058	19545.8	143.4178	19545.77	143.4109	19547.36	145.7048
143.6572	19551.53	143.6521	19543.8	143.6567	19546.1	143.6537	19544.36	143.6709	19542.32	143.6659	19546.24	145.9552
143.9043	19551.43	143.904	19544.25	143.9118	19544.87	143.9062	19544.59	143.9157	19548.64	143.9143	19546.32	146.2046
144.1545	19553.94	144.1529	19541.95	144.1573	19543.2	144.1595	19545.55	144.1694	19546.76	144.1591	19546.69	146.4618
144.4022	19549.98	144.4046	19546.03	144.4103	19546.15	144.4093	19547.16	144.4164	19544.26	144.413	19546.95	146.7042
144.658	19551.98	144.6533	19544.92	144.6577	19545.23	144.6525	19546.87	144.6733	19546.5	144.6645	19546.03	146.9557
144.9043	19550.23	144.9049	19543.86	144.9124	19546.06	144.9068	19545.75	144.9173	19544.83	144.9127	19548.38	147.2049
145.156	19551.15	145.1541	19547.38	145.1563	19546.28	145.1592	19544.24	145.1703	19546.26	145.1664	19544.94	147.4607
145.401	19553.91	145.4021	19545.01	145.4104	19548.01	145.4088	19544.95	145.4175	19546.73	145.4117	19547.87	147.7047
145.6566	19551.04	145.6518	19544.14	145.6575	19546.28	145.6529	19544.98	145.674	19545.05	145.664	19548.06	147.955
145.9046	19552.45	145.9057	19544.24	145.9123	19546.41	145.9091	19545.51	145.9207	19543.07	145.9146	19546.95	148.2048
146.1564	19550.99	146.1533	19544.28	146.1604	19542.97	146.1577	19545.71	146.1693	19545.86	146.1642	19545.23	148.4601
146.4028	19550.91	146.4081	19544.07	146.4103	19544.41	146.4069	19545.7	146.4377	19545.17	146.4119	19545.14	148.7112
146.6579	19550.3	146.6542	19545.29	146.6569	19545.85	146.6543	19544.75	146.6717	19541.37	146.665	19548.29	148.9596
146.9049	19552.26	146.9041	19544.14	146.9194	19546.13	146.9098	19543.42	146.9171	19544.63	146.9201	19546.23	149.2066
147.1564	19548.41	147.1544	19545.86	147.1585	19547.21	147.1669	19544.14	147.17	19544.86	147.1652	19547.42	149.4644
147.4027	19551.09	147.4069	19545.16	147.4111	19545.57	147.4115	19545.72	147.4177	19545.17	147.4134	19547.03	149.7139
147.6585	19551.83	147.6545	19543.26	147.6606	19546.42	147.6532	19544.96	147.6703	19546.29	147.6653	19545.33	149.9594
147.9055	19550.39	147.9069	19543.59	147.9119	19543.36	147.9075	19544.13	147.9168	19546.25	147.9172	19547.89	150.2047
148.1572	19552.83	148.1546	19546.32	148.1591	19547.66	148.1625	19546.16	148.1707	19542.58	148.1645	19546.17	150.4646
148.4031	19551.6	148.4055	19546.27	148.4131	19542.96	148.4073	19546.57	148.4179	19544.69	148.4129	19545.91	150.7091
148.6598	19551.31	148.6542	19544.72	148.6592	19543.81	148.6562	19548.14	148.6762	19548.54	148.6662	19547.14	150.9615
148.9065	19549.64	148.907	19543.34	148.9134	19543.71	148.9074	19545.48	148.9194	19546.84	148.9192	19546.19	151.208
149.1581	19547.82	149.1555	19544.49	149.1578	19546	149.1603	19545.09	149.1705	19543.13	149.1676	19545.46	151.4626
149.4033	19549.43	149.4052	19545.03	149.4126	19544.89	149.4085	19547.63	149.4203	19545.42	149.4146	19546.1	151.7098
149.6607	19552.28	149.6547	19546.71	149.6603	19544.85	149.6548	19545.56	149.6683	19546.32	149.6688	19546.14	151.9611
149.9055	19550.53	149.9072	19541.85	149.913	19546.26	149.9094	19544.38	149.9171	19547.37	149.9197	19544.39	152.2091
150.1588	19552.5	150.1559	19545.7	150.159	19546.79	150.161	19545.48	150.1688	19544.27	150.1683	19545.86	152.4662
150.4068	19549.64	150.4105	19544.93	150.4143	19546.32	150.4119	19544.13	150.4174	19545.43	150.4193	19545.92	152.7105
150.6631	19551.95	150.6573	19544.57	150.6627	19544.77	150.6571	19543.47	150.6674	19545.16	150.6712	19544.32	152.9614
150.9095	19553.89	150.9087	19543.6	150.9166	19544.89	150.9118	19543.63	150.9139	19545.85	150.9234	19544.83	153.2091
151.1615	19550.4	151.1586	19544.82	151.1628	19545.98	151.1715	19544.57	151.1692	19545.37	151.1688	19544.24	153.4668
151.4069	19550.43	151.4122	19546	151.4169	19543.23	151.4185	19546	151.4204	19545.53	151.4186	19545.8	153.7101
151.6641	19553.11	151.6615	19543.97	151.6623	19544.12	151.6596	19545.65	151.669	19545.86	151.6704	19544.44	153.9611
151.9109	19549.74	151.9109	19544.07	151.9198	19543.88	151.9163	19543.62	151.9151	19545.59	151.9208	19547.19	154.2113
152.1625	19547.33	152.1582	19542.72	152.1649	19545.63	152.1645	19546.44	152.1687	19547.23	152.1725	19548.44	154.4659
152.4084	19551.58	152.41	19544.85	152.4156	19544.41	152.4143	19545.69	152.4171	19543.47	152.419	19542.37	154.7121
152.6639	19551.59	152.6603	19545.26	152.664	19544.01	152.6615	19545.35	152.6709	19546.38	152.6712	19545.78	154.966
152.9099	19551.79	152.9118	19546.66	152.9169	19546.31	152.9123	19546.24	152.9178	19544.24	152.9246	19545.68	155.211
153.1635	19550.95	153.1591	19545.32	153.1637	19544.07	153.1668	19544.38	153.1787	19545.97	153.1733	19546.32	155.4659
153.4086	19551.47	153.4095	19543.04	153.4152	19543.7	153.4137	19545.11	153.4207	19544.95	153.4204	19543.33	155.7125
153.6653	19550.82	153.6733	19545.38	153.6646	19545.6	153.6591	19544.55	153.6676	19544.72	153.6661	19548.07	155.9643
153.9103	19551.19	153.9102	19543.57	153.918	19545.58	153.913	19545.63	153.9138	19544.27	153.9228	19545.67	156.2102
154.1623	19552.54	154.1598	19545.06	154.1637	19543.76	154.1666	19544.96	154.1675	19544.93	154.1714	19544.41	156.4657
154.4095	19549.27	154.4118	19542.74	154.4178	19547.08	154.4137	19544.59	154.416	19545.68	154.423	19545.25	156.7119
154.6649	19550.06	154.6584	19543.02	154.6638	19541.48	154.6598	19547.01	154.6666	19545.29	154.6712	19543.39	156.9644
154.9115	19550.97	154.9124	19544.02	154.9191	19545.28	154.9164	19543.33	154.9147	19543.86	154.9349	19544.71	157.2125
155.1648	19549.24	155.165	19544.6	155.1646	19543.94	155.166	19542.34	155.1662	19547.23	155.1778	19544.61	157.4647
155.4095	19550.67	155.4092	19544.32	155.4165	19542.98	155.4161	19545.28	155.4206	19544.71	155.422	19546.09	157.7119
155.6887	19548.66	155.6626	19542.83	155.6667	19542.12	155.6598	19545.99	155.6745	19547.42	155.6715	19544.57	157.9651
155.913	19551.86	155.9093	19544.11	155.9327	19542.94	155.9136	19544.24	155.9192	19543.56	155.9233	19542.8	158.2118
156.1647	19551.17	156.1602	19544.46	156.1667	19544.81	156.1668	19544.58	156.1677	19543.66	156.1722	19544.83	158.4713

156.4113	19548.47	156.41	19543.9	156.4174	19545.99	156.4147	19543.81	156.4158	19542.18	156.4231	19543.47	158.7097
156.6659	19551.19	156.6625	19543.57	156.6656	19543.38	156.6612	19546.11	156.6721	19544.88	156.6712	19545.33	158.9638
156.9126	19549.28	156.9175	19543.44	156.9226	19542.65	156.9155	19544.97	156.9186	19546.27	156.9231	19544.36	159.2125
157.165	19551.01	157.1616	19543.57	157.1655	19544.29	157.1671	19543.95	157.1674	19546.14	157.1739	19545.99	159.4673
157.4129	19550.84	157.4125	19543.49	157.418	19542.18	157.4146	19543.28	157.4171	19545.02	157.4208	19547.03	159.7124
157.6655	19550.75	157.6748	19542.74	157.6652	19544.21	157.6623	19543.92	157.6687	19547.47	157.6731	19544.59	159.9641
157.9124	19550.4	157.9126	19546.16	157.9217	19542.45	157.9182	19543.75	157.9179	19545.44	157.9251	19545.74	160.2111
158.1654	19550.91	158.1628	19544.12	158.1664	19544.18	158.1673	19543.63	158.17	19544.24	158.1761	19548.8	160.467
158.4117	19547.77	158.4123	19545.43	158.4187	19546.37	158.4152	19542.98	158.4181	19545.05	158.4223	19546.21	160.7097
158.6687	19550.61	158.6631	19541.53	158.6678	19546	158.6627	19543.34	158.6698	19547.09	158.6735	19547	160.9619
158.9131	19549.54	158.9114	19541.77	158.9214	19543.83	158.9161	19544.17	158.9304	19546.93	158.9256	19544.34	161.2087
159.1651	19552.18	159.1637	19546.2	159.167	19543.98	159.1689	19543.59	159.1691	19545.61	159.1745	19544.86	161.4652
159.4122	19548.39	159.4133	19546.04	159.4197	19543.88	159.4178	19543.88	159.4178	19543.24	159.4239	19543.17	161.7099
159.6695	19550.99	159.6625	19545.41	159.6678	19543.07	159.6645	19541.71	159.6731	19544.73	159.677	19545.64	161.9647
159.9138	19551.32	159.9135	19542.49	159.9257	19543.08	159.9177	19543.17	159.9176	19545.79	159.925	19544.55	162.2084
160.184	19552.15	160.1631	19541.83	160.1679	19545.42	160.1683	19545.63	160.1671	19544.8	160.1736	19544.5	162.4679
160.4148	19552.9	160.4146	19542.86	160.4208	19543.38	160.4188	19543.01	160.42	19544.88	160.422	19545.86	162.7103
160.6649	19550.51	160.6601	19545.15	160.667	19543.46	160.6591	19543.38	160.6688	19545.26	160.6715	19545.78	162.9643
160.9124	19550.37	160.9097	19542.86	160.9197	19543.69	160.912	19546.65	160.9167	19545.13	160.9255	19542.62	163.21
161.1626	19550.02	161.18	19543.01	161.1661	19545.2	161.1694	19541.94	161.1654	19546.04	161.1707	19544.73	163.4759
161.4068	19550.05	161.4119	19542.41	161.4174	19544.49	161.4194	19544.55	161.4159	19544.26	161.4181	19544.57	163.7086
161.6647	19551.1	161.6616	19542.64	161.664	19543.24	161.66	19544.77	161.6727	19543.24	161.6717	19546.41	163.9632
161.9105	19550.13	161.9112	19541.28	161.9225	19545.18	161.9134	19544.47	161.916	19543.81	161.925	19543.02	164.2107
162.1631	19548.51	162.1613	19542.37	162.1672	19543.49	162.1652	19543.8	162.1675	19546.67	162.174	19545.73	164.4665
162.4115	19551.07	162.4086	19542.66	162.4175	19542.97	162.423	19543.08	162.4164	19545.44	162.42	19546.02	164.7098
162.6647	19550.98	162.6609	19544.58	162.6645	19540.31	162.662	19543.6	162.6738	19546.59	162.6723	19543.45	164.9634
162.9116	19549.79	162.9129	19542.59	162.9225	19541.17	162.9136	19545.17	162.9191	19545.81	162.9239	19545.54	165.2118
163.1623	19550.12	163.1624	19544.19	163.1661	19542.13	163.165	19544.35	163.1692	19544.58	163.1733	19543.3	165.4696
163.4106	19550.04	163.4096	19545.06	163.4155	19542.93	163.4153	19543.71	163.4148	19542.06	163.4204	19546.49	165.7108
163.6653	19549.18	163.6627	19544.04	163.6654	19544.49	163.6623	19540.46	163.671	19544.82	163.6727	19545.88	165.9643
163.9124	19550.45	163.9118	19545.04	163.9187	19543.24	163.9144	19543.97	163.9184	19546.15	163.9246	19546.36	166.2112
164.1633	19546.81	164.163	19541.33	164.1664	19543.51	164.182	19543.1	164.1681	19546.73	164.1742	19544.97	166.4684
164.4114	19549.41	164.4134	19543.83	164.423	19543.11	164.4201	19540.96	164.417	19545.25	164.4208	19545.72	166.7105
164.6637	19552.67	164.6605	19542.42	164.6672	19545.88	164.6612	19543.85	164.6699	19546.73	164.6711	19545.55	166.9645
164.9122	19550.08	164.9112	19545.59	164.9222	19542.51	164.9146	19542.49	164.921	19544.23	164.9235	19546.14	167.2111
165.1644	19549.42	165.1644	19543.6	165.1665	19543.9	165.1667	19542.2	165.1677	19542.68	165.1731	19543.72	167.4692
165.4103	19551.96	165.4116	19544.66	165.4181	19542.44	165.4171	19543.26	165.4212	19545.8	165.4219	19545.43	167.7113
165.6658	19547.83	165.6615	19541.6	165.666	19541.18	165.6612	19544.66	165.6713	19542.34	165.674	19542.32	167.9672
165.9132	19552.42	165.9116	19542.85	165.9278	19545.22	165.915	19541.98	165.9168	19543.5	165.9222	19544.74	168.2116
166.1669	19549.99	166.163	19542.83	166.1674	19543.79	166.1695	19545.52	166.1683	19543.08	166.176	19544.76	168.4675
166.4122	19550.89	166.4124	19541.61	166.4192	19541.28	166.419	19543.84	166.4178	19545.71	166.4181	19544.31	168.7131
166.666	19549.55	166.6622	19544.51	166.6677	19546.25	166.6616	19540.97	166.6765	19544.08	166.6755	19545.82	168.9661
166.9143	19547.64	166.9127	19543.42	166.9391	19546.94	166.916	19543.5	166.9172	19543.98	166.9273	19545.46	169.2121
167.1664	19552.47	167.1645	19539.67	167.1684	19541.77	167.1672	19544.42	167.1668	19544.1	167.1755	19542.43	169.4708
167.412	19549.62	167.4127	19542.25	167.4198	19541.94	167.4182	19544.16	167.4173	19545.44	167.4228	19545.1	169.7124
167.673	19551.58	167.6626	19542.81	167.6712	19541.33	167.6623	19544.16	167.675	19544.28	167.6764	19544.77	169.9668
167.9143	19549.86	167.9131	19543	167.9224	19542.66	167.9185	19543.02	167.9179	19545.57	167.9307	19547.64	170.2136
168.1654	19552.34	168.1635	19542.27	168.1811	19546.93	168.1678	19544.62	168.185	19546.29	168.1764	19548.64	170.4701
168.4122	19549.77	168.4137	19543.28	168.4197	19540.7	168.4214	19545.3	168.4173	19543.9	168.423	19545.34	170.7113
168.6647	19550.48	168.6654	19544.66	168.6672	19545.77	168.6628	19544.47	168.6788	19544.68	168.6745	19543.06	170.9674
168.9137	19549.25	168.9147	19541.2	168.9227	19543.54	168.9182	19542.45	168.921	19543.05	168.9299	19541.95	171.2154
169.1698	19548.52	169.1658	19540.98	169.167	19544.2	169.1688	19544.39	169.1701	19541.76	169.1771	19545.53	171.4705
169.4136	19548.56	169.4142	19542.44	169.4205	19546.11	169.4204	19544.34	169.4244	19543.55	169.4177	19542.96	171.7133
169.6696	19550.04	169.6769	19541.14	169.6674	19544.27	169.664	19542.03	169.6721	19543.99	169.6742	19544.79	171.967
169.9148	19548.63	169.9148	19544.14	169.9245	19543.07	169.9172	19544.79	169.9346	19543.59	169.9196	19544.62	172.2145
170.167	19550.9	170.1656	19545.86	170.1688	19541.02	170.1684	19544.27	170.1755	19544.11	170.1735	19544.21	172.4737
170.4131	19551.28	170.4144	19542.09	170.42	19545.54	170.4185	19542.11	170.4269	19542.37	170.4249	19546.27	172.7143
170.6666	19549.62	170.6653	19543.07	170.6698	19542.85	170.6633	19545.03	170.6794	19545.28	170.6774	19544.67	172.9685
170.9155	19548.27	170.9142	19545.49	170.9263	19543.49	170.9177	19542.55	170.925	19544.32	170.9274	19544.58	173.2125
171.1672	19549.26	171.1652	19541.2	171.1674	19542.43	171.1706	19540.33	171.1746	19545.23	171.1752	19543.48	173.4706
171.4133	19549.89	171.4151	19542.44	171.421	19544.33	171.4183	19544.43	171.4225	19545.5	171.4255	19545.39	173.7152
171.6663	19548.21	171.6651	19542.59	171.6692	19544.7	171.6634	19542.55	171.6724	19543.57	171.6822	19544.11	173.9692
171.9155	19548.68	171.9145	19540.57	171.9263	19542.16	171.9182	19541.76	171.9223	19543.32	171.9269	19545.75	174.2158
172.1678	19549.89	172.1653	19543.84	172.168	19541.91	172.1707	19543.4	172.172	19540.61	172.1736	19542.52	174.4705
172.4135	19549.01	172.4145	19543.77	172.4231	19540.33	172.4215	19544.06	172.4255	19545.25	172.4241	19541.89	174.7132
172.6679	19551.6	172.6676	19542.51	172.6681	19544.6	172.6636	19543.16	172.6731	19544.44	172.669	19544.03	174.9697
172.9161	19549.41	172.916	19541.83	172.9258	19544.32	172.9168	19544.78	172.9229	19543.23	172.9199	19544.72	175.2144
173.1689	19551.77	173.1658	19540.71	173.1705	19541.14	173.1704	19542.58	173.1764	19544.72	173.1725	19543.88	175.4745
173.416	19548.77	173.414	19541.76	173.4254	19541.2	173.4188	19542.83	173.4223	19544.75	173.4181	19544.01	175.7132
173.6687	19549.96	173.6658	19542.23	173.6707	19542.94	173.6636	19542.95	173.6756	19545.79	173.6738	19543.15	175.9731

173.9178	19549.4	173.9161	19543.16	173.926	19543.65	173.9194	19542.14	173.9248	19544.83	173.9258	19542.28	176.2181
174.1681	19550.57	174.1657	19540.61	174.1699	19543.21	174.1703	19543.29	174.1743	19542.13	174.1782	19542.42	176.4797
174.416	19550.51	174.4142	19540.01	174.4226	19543.33	174.4188	19543.55	174.4239	19545.17	174.4254	19545.82	176.7247
174.667	19548.44	174.6669	19542.48	174.6696	19542.34	174.6645	19544.98	174.674	19542.5	174.676	19543.84	176.9734
174.9173	19549.15	174.9162	19543.88	174.9269	19540.9	174.9186	19541.25	174.9256	19545.22	174.9284	19543.54	177.2232
175.1684	19550.66	175.1672	19543.11	175.1713	19544.31	175.1716	19545.99	175.1758	19543.91	175.1808	19543.53	177.4728
175.4152	19547.55	175.4158	19544.16	175.4283	19541.57	175.4195	19544.04	175.4238	19542.9	175.4254	19544.75	177.7146
175.6694	19550.15	175.6655	19545.52	175.6722	19543.53	175.6664	19543.04	175.6761	19544.7	175.6795	19542.79	177.9685
175.9173	19550.47	175.9165	19542.02	175.929	19544.21	175.9192	19541.12	175.9239	19543.74	175.9242	19545.49	178.2157
176.1686	19550.82	176.1663	19542.47	176.1708	19542.51	176.1722	19542.9	176.1784	19543.9	176.1762	19543.22	178.4748
176.4175	19547.16	176.4151	19544.73	176.4227	19540.3	176.4185	19543.22	176.4237	19543.77	176.4248	19543.16	178.7157
176.671	19549.31	176.6662	19541.93	176.671	19542.46	176.6655	19542.67	176.6749	19544.09	176.6776	19543.59	178.9704
176.918	19550.38	176.9166	19541.38	176.9295	19541.95	176.9193	19543.37	176.9243	19542.11	176.9425	19543.69	179.2147
177.1687	19549.9	177.1689	19541.28	177.1704	19542.99	177.1709	19542.09	177.1754	19543.92	177.1853	19545.33	179.4745
177.4163	19548.81	177.4169	19544.32	177.4241	19542.16	177.4188	19541.02	177.4259	19542.32	177.4218	19541.57	179.7187
177.6688	19549.83	177.6681	19541.96	177.671	19543.93	177.6675	19542.43	177.6743	19543.13	177.6787	19544.85	179.9689
177.9197	19547.22	177.9185	19542.19	177.9297	19541.36	177.9216	19540.65	177.928	19544.47	177.9307	19540.43	180.2158
178.1695	19549.49	178.1678	19545.34	178.1718	19543.51	178.1728	19543.85	178.1792	19543.02	178.1745	19545.73	180.4726
178.4157	19551.81	178.4163	19540.09	178.4267	19541.7	178.4198	19544.95	178.4247	19541.55	178.4216	19544.29	180.7175
178.6699	19547.8	178.6685	19545.8	178.6716	19542.18	178.6673	19543.96	178.6764	19544.1	178.6777	19544.61	180.9704
178.9204	19552.15	178.9187	19540.93	178.9296	19542.94	178.9212	19545.79	178.9261	19544.73	178.9263	19540.44	181.2294
179.17	19550.18	179.1673	19542.02	179.1727	19544.16	179.1728	19541.87	179.1806	19541.95	179.1801	19544.58	181.4861
179.4162	19548.93	179.4169	19542.96	179.4256	19540.93	179.4239	19546.39	179.4289	19543.15	179.4279	19540.56	181.7174
179.6729	19548.96	179.6673	19545.27	179.6713	19542.7	179.6677	19544.8	179.6764	19542.44	179.6797	19543.65	181.9704
179.9198	19548.37	179.9188	19543.57	179.9304	19544.52	179.9227	19540.44	179.9374	19541.81	179.934	19543.1	182.2194
180.1711	19548.93	180.1686	19542.53	180.1726	19541.38	180.1726	19539.23	180.1792	19542.02	180.1804	19542.5	182.4798
180.4171	19549.86	180.4176	19543.14	180.4265	19543.31	180.4212	19543.66	180.4298	19542.33	180.4277	19544.45	182.7179
180.6682	19549.94	180.6672	19542.39	180.673	19540.27	180.6689	19542.29	180.6787	19541.78	180.6805	19542.48	182.9687
180.9199	19549.27	180.9182	19541.07	180.932	19542.99	180.9214	19542.9	180.9269	19545.65	180.9406	19543.4	183.2172
181.1723	19547.77	181.1708	19542.84	181.1722	19539.87	181.1741	19542.85	181.1765	19545.93	181.1836	19542.24	183.4749
181.4181	19552.07	181.4188	19541.87	181.4267	19542.66	181.422	19544.84	181.427	19543.4	181.4284	19541.4	183.7186
181.672	19551	181.6738	19544.7	181.674	19542.99	181.6688	19540.83	181.6812	19544.73	181.7169	19542.88	183.9704
181.9217	19550.51	181.9186	19541.7	181.9315	19541.11	181.9207	19543.89	181.9312	19542.63	181.9344	19544.52	184.2177
182.1721	19549.3	182.1693	19544.61	182.1725	19540.96	182.1734	19544.86	182.1769	19545.4	182.184	19543.49	184.4776
182.4182	19546.7	182.4188	19544.43	182.4269	19542.88	182.4242	19544.06	182.4272	19543.27	182.4281	19545.46	184.7186
182.6736	19548.17	182.6703	19541.55	182.673	19542.55	182.6712	19543.75	182.6822	19543.45	182.6806	19541.99	184.9723
182.9209	19548.08	182.9197	19541.92	182.9304	19542.49	182.9227	19543.75	182.9289	19544.47	182.9333	19540.84	185.2186
183.1713	19549.94	183.1689	19542.08	183.1727	19545.2	183.1741	19542.64	183.1813	19543.55	183.7774	19545.13	185.4799
183.4182	19549.26	183.4267	19544.27	183.4266	19542.99	183.4248	19539.23	183.4276	19545.96	184.0178	19542.45	185.7207
183.6715	19549.57	183.6698	19541.62	183.6743	19540.45	183.6705	19544.57	183.6782	19543.9	184.2729	19542.44	185.9727
183.9211	19549.29	183.9197	19542.48	183.9328	19542.62	183.9221	19543.84	183.9394	19542.49	184.516	19542.91	186.2207
184.1737	19548.89	184.1718	19541.16	184.1745	19542.4	184.1765	19541.92	184.1846	19542.55	184.7677	19540.28	186.4782
184.4184	19549.76	184.4189	19542.26	184.4274	19544.21	184.4323	19542.19	184.4288	19544.02	185.0182	19540.5	186.7183
184.673	19550.24	184.6696	19541.74	184.6732	19542.16	184.6694	19542.86	184.6804	19541.67	185.2877	19542.94	186.9703
184.9239	19550.5	184.9212	19542.49	184.9313	19542.93	184.9258	19541.72	184.9277	19544.87	185.5181	19540.72	187.2214
185.1774	19548.05	185.1715	19542.51	185.1739	19542.99	185.1768	19541.62	185.1805	19541.62	185.7692	19538.68	187.4778
185.4213	19549.3	185.4217	19541.96	185.4276	19544.07	185.4258	19541.67	185.4358	19545.2	186.0213	19541.3	187.72
185.6723	19547.35	185.6735	19540.83	185.6756	19543.18	185.6721	19540.02	185.6795	19545.18	186.271	19542.03	187.9715
185.9361	19551.64	185.9215	19542.61	185.9313	19543.39	185.9269	19542.88	185.9272	19543.9	186.5179	19543.32	188.2213
186.1741	19549.46	186.1697	19540.7	186.1746	19543.74	186.1761	19543.2	186.1791	19542.09	186.771	19541.86	188.4777
186.4207	19549.77	186.4219	19543.14	186.4284	19542.84	186.4271	19544.39	186.4284	19541.77	187.0183	19540.84	188.7229
186.673	19547.94	186.6713	19542.28	186.6749	19542.98	186.6723	19542.3	186.6808	19547.21	187.2742	19539.1	188.9797
186.9226	19547.49	186.9211	19541.91	186.9354	19544.98	186.9258	19542.7	186.9283	19542.29	187.519	19542.99	189.2257
187.1738	19548.77	187.1719	19539.85	187.1748	19542.56	187.1774	19542.23	187.181	19541.75	187.7705	19540.93	189.4767
187.4219	19547.96	187.4221	19541.09	187.4315	19543.92	187.4261	19544.29	187.4289	19541.2	188.0185	19542.32	189.7209
187.6778	19550.9	187.6717	19541.35	187.6759	19540.77	187.6766	19544.47	187.6821	19544.01	188.2729	19541.99	189.9738
187.9229	19549.97	187.9223	19541.4	187.9321	19539.86	187.9253	19540.36	187.9295	19543.68	188.5198	19540.88	190.2205
188.1741	19548.3	188.1713	19542.23	188.1746	19542.9	188.18	19540.87	188.1841	19540.43	188.7734	19543.24	190.4796
188.4225	19550.05	188.4219	19543.23	188.4291	19545.81	188.4274	19543.66	188.4319	19543.53	189.0206	19540.19	190.7206
188.6735	19547.7	188.6756	19542.36	188.6759	19540.45	188.6727	19540.87	188.6795	19543.69	189.2731	19542.01	190.973
188.9239	19550.27	188.9225	19540.29	188.9317	19542.31	188.924	19543.52	188.9426	19543.76	189.5202	19545.84	191.2213
189.1741	19548.49	189.1719	19541.74	189.1765	19543.46	189.1759	19542.84	189.1829	19542.87	189.7729	19543.31	191.4823
189.4213	19549.05	189.4229	19539.74	189.4306	19540.91	189.4261	19541.19	189.4318	19541.57	190.0219	19543.04	191.7218
189.6752	19548.98	189.6737	19541.17	189.6778	19543.33	189.6751	19543.94	189.6807	19542.5	190.273	19542.31	191.9745
189.9242	19549.16	189.925	19543.86	189.9336	19542.36	189.9242	19541.39	189.9306	19545.58	190.5204	19545.29	192.2323
190.1765	19548.57	190.1719	19540.14	190.1774	19540.66	190.177	19541.6	190.1847	19542.68	190.7726	19541.86	192.4884
190.4225	19550.16	190.4234	19539.82	190.4338	19541.52	190.4247	19543.76	190.4378	19546.43	191.022	19543.01	192.7202
190.6748	19548.36	190.6739	19541.25	190.6788	19543.07	190.6745	19540.96	190.6852	19545.17	191.2733	19547.36	192.9751
190.9252	19547.39	190.9231	19542.16	190.9342	19540.07	190.9253	19543.24	190.9294	19544.97	191.5214	19543.14	193.2221
191.1763	19548.43	191.1729	19540.52	191.1777	19540.31	191.1806	19542.43	191.1799	19543.92	191.7734	19540.39	193.479

191.4228	19547.29	191.4242	19538.38	191.4311	19542.58	191.4258	19538.93	191.431	19540.29	192.0253	19542.49	193.7279
191.6771	19548.34	191.6746	19539.39	191.6811	19541.47	191.6745	19544.09	191.6826	19543.24	192.2729	19541.63	193.9736
191.9241	19548.31	191.9223	19541.9	191.936	19540.61	191.9261	19541.2	191.9324	19542.07	192.5211	19542.88	194.223
192.1756	19546.28	192.1728	19541	192.1777	19542.45	192.1791	19539.55	192.1872	19542.73	192.7724	19545.19	194.4819
192.4233	19549.8	192.4236	19543.07	192.4298	19542.54	192.4257	19542.96	192.4312	19544.05	193.023	19543.99	194.723
192.6793	19548.22	192.6784	19542.13	192.6771	19540.11	192.676	19541.79	192.6832	19543.28	193.274	19541.05	194.976
192.9239	19549.76	192.9243	19543.03	192.9366	19543.31	192.9266	19540.53	192.933	19545.9	193.5206	19545.04	195.2211
193.1765	19545.98	193.1726	19539.65	193.1795	19543.52	193.1777	19544.03	193.1841	19545.06	193.7724	19542.71	195.4803
193.4242	19549.26	193.4244	19542.19	193.4312	19541.95	193.4257	19540.98	193.4329	19541.64	194.0234	19545.39	195.7229
193.6773	19546.95	193.6895	19542.93	193.6772	19543.49	193.6751	19541.7	193.6828	19544.42	194.286	19541.39	195.9772
193.9263	19548.97	193.9252	19542.86	193.9356	19539.65	193.9265	19544.76	193.934	19541.89	194.5223	19542.59	196.2251
194.179	19548.87	194.1735	19539.77	194.179	19545.17	194.1793	19542.16	194.1839	19543.27	194.7756	19544.99	196.4796
194.4245	19547.39	194.4244	19541.57	194.4332	19543.78	194.4295	19543	194.4332	19541.33	195.0266	19543.23	196.7226
194.6765	19546.7	194.676	19540.08	194.6826	19541.47	194.6754	19541.76	194.6831	19539.91	195.2755	19541.51	196.9768
194.9264	19549.14	194.9247	19541.9	194.9435	19542.65	194.9281	19541.98	194.9335	19541.52	195.5221	19542.99	197.2258
195.1784	19548.36	195.1765	19542.64	195.181	19543.83	195.1806	19542.78	195.1853	19540.88	195.7738	19542.32	197.4818
195.4237	19546.76	195.4242	19541.22	195.4317	19542.97	195.4272	19541.42	195.4344	19541.33	196.0254	19542.77	197.7218
195.6776	19545.86	195.6757	19541.85	195.6783	19540.61	195.6759	19543.08	195.6847	19545.01	196.2763	19542.46	197.9745
195.9412	19549.61	195.9257	19543.34	195.9361	19542.48	195.9275	19539.72	195.9329	19539.66	196.5227	19543.95	198.2282
196.1774	19546.63	196.1752	19544.61	196.1781	19540.78	196.1822	19539.47	196.1858	19541.89	196.7723	19543.62	198.4829
196.4252	19549.77	196.4257	19540.31	196.4331	19540.52	196.4277	19540.86	196.4351	19541.93	197.0358	19541.69	198.7254
196.6778	19549.45	196.6776	19540.44	196.6806	19543.58	196.6756	19541.11	196.6855	19542.83	197.2758	19543.97	198.9766
196.9261	19547.47	196.9272	19538.73	196.9364	19540.39	196.9297	19541.67	196.9358	19544.21	197.5261	19545.28	199.2262
197.1817	19547.18	197.1748	19540.44	197.1807	19538.46	197.1807	19541.52	197.1832	19542.75	197.7762	19543.72	199.4811
197.4256	19548.35	197.4264	19542.86	197.4335	19543.37	197.4273	19541.56	197.4349	19542.48	198.0242	19542.24	199.7241
197.6774	19550.95	197.6764	19541.75	197.6793	19538.38	197.679	19543.29	197.6865	19543.62	198.2762	19543.67	199.976
197.9266	19548.84	197.927	19541.61	197.9377	19543.52	197.929	19542.06	197.9389	19542.43	198.5229	19542.11	200.2274
198.1867	19549.77	198.1851	19541.8	198.1888	19542.26	198.1925	19540.18	198.1959	19543.96	198.7845	19543.03	200.4954
198.4266	19548.1	198.4247	19540.5	198.4322	19542.59	198.4308	19541.2	198.4384	19542.83	199.0257	19541.14	200.7293
198.6783	19549.31	198.6891	19539.76	198.6818	19543.88	198.6779	19542.97	198.7146	19539.71	199.2769	19542.69	200.9805
198.9271	19547.27	198.9265	19540.07	198.9358	19540.26	198.9301	19541.01	198.9309	19542.66	199.524	19539.67	201.2292
199.1799	19548.97	199.1786	19541.83	199.1811	19543.17	199.1812	19541.02	199.1978	19542.8	199.7749	19542.98	201.4868
199.4258	19547.33	199.4257	19540.48	199.4341	19541.31	199.4337	19541.58	199.4338	19542.51	200.0263	19542.34	201.7288
199.6792	19547.03	199.6772	19540.5	199.681	19543.86	199.6775	19540.2	199.6826	19542.47	200.283	19543.99	201.9827
199.9293	19548.14	199.9276	19541.01	199.9378	19538.91	199.9298	19537.91	199.9355	19541.92	200.5289	19546.02	202.2289
200.1802	19549.54	200.1767	19538.36	200.1807	19543.65	200.1815	19540.1	200.1942	19541.71	200.7825	19542.69	202.4895
200.4297	19547.55	200.4317	19541.63	200.4379	19542.06	200.4343	19542.57	200.4388	19543.14	201.0442	19543.58	202.7301
200.6843	19549.82	200.6802	19542.85	200.6855	19542.73	200.6825	19542.79	200.6903	19540.86	201.2827	19543.97	202.9821
200.9456	19548.97	200.9305	19540.51	200.942	19540.82	200.9343	19541.61	200.9743	19541.76	201.5321	19543.54	203.2312
201.1854	19545.92	201.1844	19540.45	201.1857	19539.85	201.1879	19542.31	201.2351	19542.44	201.7837	19543.89	203.4861
201.4304	19550.25	201.4299	19538.87	201.4394	19539.12	201.4351	19540.78	201.7023	19541.23	202.0307	19541.87	203.7354
201.6835	19550.17	201.6813	19540.93	201.6878	19542.2	201.6835	19538.07	201.9521	19545.37	202.2814	19541.73	203.9913
201.9319	19548.7	201.9319	19542.86	201.9425	19544.69	201.9348	19540.64	202.1988	19541.56	202.5314	19541.1	204.2302
202.1855	19548.13	202.1813	19538.69	202.1866	19542.63	202.1877	19541.41	202.4494	19540.16	202.7817	19540.96	204.4913
202.4354	19547.68	202.4316	19538.05	202.4393	19543.64	202.4372	19543.56	202.6964	19544.58	203.0323	19542.46	204.7313
202.6843	19547.34	202.6866	19538.44	202.6864	19540.47	202.6826	19540.63	202.9473	19544.84	203.2821	19542	204.9837
202.9332	19547.66	202.9341	19539.72	202.9442	19541.3	202.9368	19541.22	203.1992	19542.28	203.5307	19545.13	205.2307
203.185	19546.94	203.1832	19540.71	203.1877	19538.5	203.1874	19540.42	203.4481	19544.4	203.7824	19542.47	205.4902
203.4315	19547.52	203.4317	19541.24	203.4392	19541.06	203.4354	19543.44	203.6991	19542.78	204.0334	19543.63	205.7303
203.6847	19547.26	203.6856	19542	203.6876	19542.33	203.6834	19541.41	203.948	19541.31	204.2831	19544.78	205.9813
203.938	19550.45	203.9331	19538.11	203.9437	19542.03	203.9352	19543.76	204.198	19540.29	204.5311	19541.51	206.2317
204.1862	19547.85	204.1844	19542.82	204.1865	19544.26	204.1882	19543.01	204.4481	19541.27	204.7827	19542.35	206.4886
204.4324	19547.31	204.4327	19540.17	204.439	19540.68	204.4386	19538.99	204.6958	19544.82	205.0335	19543.18	206.7304
204.684	19548.35	204.6835	19542.38	204.6875	19538.36	204.6828	19542.72	204.9486	19540.18	205.2851	19542.89	206.9835
204.9356	19548	204.9354	19539.15	204.9428	19542.24	204.9368	19540.83	205.1997	19540.16	205.5333	19541.36	207.2318
205.188	19547.9	205.185	19541.34	205.186	19543.56	205.1887	19542.45	205.4469	19541.52	205.784	19540.08	207.4898
205.4327	19549.57	205.434	19543.25	205.439	19540.72	205.4366	19543.21	205.6978	19543.26	206.031	19541.65	207.7309
205.6862	19549.71	205.6839	19542.37	205.6928	19544.25	205.6838	19542.42	205.9488	19540.76	206.2854	19543.34	207.9823
205.9475	19548.01	205.9383	19540.99	205.9472	19538.39	205.9383	19541.5	206.1992	19543.03	206.5324	19544.4	208.2309
206.1874	19547.5	206.1893	19540.42	206.1883	19541.47	206.1888	19540.97	206.4479	19541.49	206.7848	19543.06	208.4911
206.4341	19547.96	206.4341	19540.51	206.4392	19540.56	206.4361	19538.86	206.6982	19543.19	207.0336	19541.55	208.7326
206.6858	19548.87	206.6866	19539.89	206.6883	19542.53	206.6847	19540.92	206.962	19541.72	207.2867	19538.44	208.9831
206.9363	19546.62	206.9398	19539.57	206.9481	19542.39	206.9385	19542.48	207.2133	19542.78	207.532	19541.67	209.2318
207.1868	19546.5	207.1864	19541.82	207.1913	19540.73	207.1886	19541.55	207.4595	19543.05	207.7824	19541.95	209.4897
207.4338	19550.07	207.4348	19539.21	207.4411	19540.31	207.4373	19542.79	207.6974	19541.03	208.0337	19543.76	209.7338
207.6884	19548.1	207.6891	19541.92	207.6896	19539.07	207.6844	19540.4	207.9573	19542.4	208.2845	19542.49	209.9842
207.9361	19549.76	207.9337	19542.97	207.9531	19540.81	207.9399	19542.26	208.2199	19541.04	208.5339	19542.06	210.2316
208.1899	19547.77	208.1857	19542.98	208.1961	19540.81	208.1883	19542.2	208.5399	19542.64	208.7833	19541.88	210.4901
208.4363	19548.68	208.4351	19540.36	208.4418	19541.59	208.4388	19539.73	209.0103	19541.42	209.0342	19543.71	210.7331
208.6892	19548.01	208.6864	19541.9	208.6902	19542.79	208.6859	19542.48	209.2578	19544.8	209.2862	19540.1	210.9914

208.9376	19545.75	208.9339	19538.71	208.9473	19539.15	208.9378	19539.61	209.508	19542.29	209.5349	19543.87	211.2334
209.1878	19545.94	209.1837	19539.39	209.1879	19538.65	209.1885	19541.66	209.7583	19545.62	209.7861	19542.89	211.4899
209.4347	19546.6	209.4346	19540.11	209.4413	19543.21	209.4396	19543.77	210.0072	19542.13	210.035	19545.02	211.7348
209.6874	19546.59	209.6863	19539.52	209.6902	19543.25	209.6861	19541.13	210.2624	19542.9	210.2873	19541.33	211.9862
209.97	19546.9	209.9357	19541.15	209.9464	19541.37	209.9411	19540.83	210.5177	19543.41	210.5367	19543.75	212.2325
210.1997	19547.86	210.1878	19541.65	210.1898	19543.03	210.1947	19543.29	210.7654	19543.36	210.7888	19540.87	212.4931
210.4356	19549.3	210.4362	19541.61	210.4416	19541.04	210.4393	19541.15	211.0152	19542.17	211.0357	19540.38	212.7346
210.6878	19549.39	210.7	19540.99	210.6915	19542.02	210.6855	19541.81	211.2646	19540.47	211.2866	19541.4	212.9861
210.937	19549.26	210.937	19541.13	210.9472	19541.23	210.9398	19543.55	211.5128	19540.45	211.5355	19545.53	213.2342
211.1881	19548.53	211.1852	19540.66	211.1901	19541.43	211.1903	19540.81	211.7891	19541.91	211.7881	19540.62	213.496
211.4341	19546.56	211.4352	19541.43	211.446	19542.38	211.4392	19540.29	212.2638	19544.88	212.0359	19542.7	213.7344
211.6893	19545.76	211.6888	19542.21	211.6915	19542.04	211.6861	19543.91	212.5216	19541.93	212.2886	19540.86	213.9863
211.9386	19550.68	211.9372	19541.36	211.9499	19541.35	211.939	19541.84	212.7771	19543.29	212.5355	19544.48	214.2367
212.1883	19546.02	212.1863	19541.17	212.1904	19540.08	212.1914	19541.85	213.024	19543.34	212.7864	19538.7	214.4963
212.4356	19547.07	212.4355	19543.48	212.4434	19543.21	212.4397	19539.27	213.2671	19539.31	213.042	19541.28	214.7361
212.6885	19549.42	212.689	19539.82	212.6919	19543.96	212.6873	19541.75	213.5254	19540.65	213.2869	19542.44	214.9861
212.9383	19547.3	212.9376	19540.81	212.9472	19541.89	212.9416	19543.29	213.7596	19543.16	213.5357	19544.24	215.2354
213.1876	19548.31	213.1868	19541.48	213.1916	19539.61	213.1911	19542.78	214.0141	19544.5	213.7872	19541.08	215.4938
213.4355	19546.97	213.4394	19541.29	213.4432	19540.69	213.4423	19540.32	214.2649	19542.53	214.0379	19541.15	215.7358
213.6903	19549.15	213.7084	19542.4	213.6912	19540.67	213.6865	19540.2	214.5184	19538.65	214.2887	19543.05	215.9875
213.9394	19551.51	213.9426	19539.17	213.9492	19539.69	213.9437	19542.53	214.7719	19542	214.536	19541.9	216.2352
214.1905	19547.11	214.1872	19540.38	214.1905	19539.07	214.1918	19541.99	215.0224	19542.04	214.7872	19540.44	216.4941
214.4366	19548.85	214.4379	19540.29	214.4464	19541.43	214.4446	19540.53	215.2694	19540.01	215.041	19542.46	216.7356
214.6893	19547.11	214.6881	19540.38	214.6912	19540.73	214.695	19540.72	215.5163	19541.56	215.2889	19543.66	216.9886
214.9396	19546.64	214.9401	19542.37	214.9506	19540.46	214.941	19543.25	215.7723	19544.17	215.5358	19539.16	217.2355
215.1926	19547.7	215.1876	19540.66	215.194	19539.62	215.1914	19540.06	216.0117	19542.31	215.7876	19542.47	217.4924
215.4372	19549.18	215.4397	19543.8	215.4507	19541.18	215.44	19539.76	216.2619	19542.96	216.0402	19541.18	217.7369
215.6913	19548.74	215.6927	19538.12	215.6948	19540.24	215.6885	19538.69	216.5176	19540.78	216.2914	19542.6	217.9879
215.9383	19547.41	215.9384	19543.57	215.949	19540.94	215.9428	19540.23	216.7681	19539.39	216.5383	19540.28	218.2352
216.1921	19547.91	216.1888	19541.43	216.1917	19538.03	216.1907	19540.67	217.0196	19542.35	216.7865	19542.19	218.4966
216.442	19548.79	216.4368	19542.27	216.4437	19541.93	216.442	19542.43	217.2651	19541.3	217.0377	19543.32	218.7362
216.6926	19549.05	216.6907	19538.25	216.6919	19539.23	216.6886	19543.5	217.5176	19541.72	217.2909	19543.4	218.9898
216.94	19547.74	216.9409	19540.93	216.9499	19537.98	216.9423	19543.2	217.7669	19542.58	217.5436	19539.64	219.2352
217.1911	19548.04	217.1914	19542.7	217.1929	19539.5	217.1937	19542.3	218.0201	19541.58	217.7958	19539.53	219.5145
217.439	19547.04	217.439	19540.01	217.4454	19545.17	217.4406	19541.53	218.2631	19541.98	218.0437	19542.76	219.7377
217.6915	19547.51	217.6956	19540.45	217.6919	19538.59	217.6902	19543.07	218.5156	19543.73	218.293	19541.58	219.9894
217.9405	19549.44	217.941	19539.17	217.9517	19542.54	217.948	19539.05	218.7688	19539.76	218.5368	19540.4	220.2383
218.1922	19548.92	218.1892	19541.2	218.1956	19542.9	218.1926	19539.47	219.015	19539.63	218.7885	19542.54	220.4969
218.4397	19548.01	218.4412	19542.07	218.4441	19539.83	218.442	19541.49	219.2633	19539.84	219.0404	19541.94	220.7364
218.6939	19545.81	218.6925	19539.63	218.6937	19542.23	218.6888	19540.76	219.5231	19540.77	219.2899	19540.4	220.9895
218.9413	19548.43	218.9389	19540.5	218.9562	19539.39	218.9431	19541.98	219.77	19540.46	219.5377	19542.49	221.2364
219.1931	19548.97	219.1909	19540.32	219.1957	19543.38	219.1924	19542.34	220.0218	19541.44	219.7881	19541.49	221.4957
219.44	19546.51	219.4405	19538.46	219.4465	19542.81	219.442	19541.43	220.2652	19539.96	220.0413	19541.44	221.736
219.6945	19549.2	219.6927	19540.88	219.6921	19540.75	219.6913	19540.69	220.5193	19541.57	220.2911	19542.34	221.9916
219.9541	19548.92	219.9412	19539.21	219.9528	19541.86	219.9437	19542.27	220.7733	19542.64	220.5402	19541.69	222.238
220.1942	19548.86	220.1911	19538.42	220.1918	19541.08	220.1964	19540.96	221.0193	19541	220.7899	19541.75	222.4969
220.4594	19548.61	220.4399	19539.68	220.4473	19539.87	220.4531	19541.01	221.2669	19541.52	221.0405	19540.62	222.7381
220.6977	19549.29	220.6966	19540.87	220.6937	19539.08	220.6894	19541.62	221.5176	19541.06	221.2936	19539.88	222.9896
220.9415	19547.66	220.9472	19540.19	220.9526	19538.43	220.9425	19541.55	221.7678	19543.23	221.5404	19539.94	223.2377
221.1935	19548.8	221.1897	19538.26	221.1938	19542.25	221.1955	19539.95	222.0187	19544.05	221.7913	19539.76	223.4985
221.4414	19548.33	221.4601	19538.64	221.4478	19537.9	221.4437	19543.1	222.2651	19544.62	222.0401	19541.13	223.7387
221.6918	19547.97	221.7114	19539.9	221.6953	19540.97	221.6903	19541.25	222.5177	19542.72	222.2915	19540.07	223.9892
221.9415	19549.05	221.9509	19539.29	221.9541	19541.13	221.9443	19539.63	222.7712	19542.9	222.54	19542.07	224.2383
222.1939	19547.32	222.1913	19541.14	222.1914	19541.27	222.1953	19542.38	223.0214	19539.66	222.7911	19544.02	224.4959
222.4418	19548.07	222.4427	19541.29	222.4668	19540.75	222.4439	19540.62	223.2796	19541.62	223.0495	19541.16	224.7386
222.693	19547.52	222.6909	19538.69	222.7036	19539.71	222.6909	19542.83	223.5228	19540.64	223.3031	19539.13	224.9909
222.9418	19548.17	222.9425	19541.35	222.9487	19542.15	222.9475	19541.28	223.7657	19539.58	223.5532	19543.41	225.2392
223.1941	19548.09	223.1913	19541	223.195	19542.2	223.1975	19540.51	224.0218	19542.76	223.8052	19539.76	225.4973
223.4431	19547.77	223.4416	19540.57	223.4504	19538.93	223.4473	19542.92	224.2712	19541.72	224.0751	19541.97	225.7401
223.6929	19546.48	223.6963	19540.77	223.6963	19539.62	223.6929	19542.11	224.5237	19540.34	224.5523	19543.84	225.9924
223.9433	19547.61	223.9411	19540.37	223.9532	19540.1	223.9451	19543.19	224.7685	19542.32	224.8041	19541.64	226.2409
224.1974	19546.29	224.1917	19541.96	224.1912	19540.61	224.1959	19541.42	225.0164	19540.51	225.0574	19541.55	226.4991
224.4426	19547.38	224.4415	19540.39	224.4495	19535.87	224.4646	19541.04	225.2676	19541.87	225.3086	19542.87	226.7405
224.6953	19548.94	224.6973	19540.71	224.6975	19539.36	224.7043	19541.91	225.5195	19539.87	225.5586	19540.18	226.9932
224.9438	19547.15	224.9468	19542.49	224.9548	19541.57	224.9486	19541.69	225.7704	19539.71	225.809	19542.11	227.2405
225.195	19548.93	225.1935	19540.4	225.1967	19542.29	225.1962	19544.45	226.0129	19540.89	226.0603	19544.46	227.4996
225.4437	19548.49	225.4425	19538.92	225.4488	19539.22	225.4471	19541.35	226.2651	19541.59	226.3146	19539.54	227.7401
225.6957	19546.97	225.6949	19542.25	225.6963	19540.59	225.6926	19542.94	226.5173	19540.66	226.566	19543.21	227.9929
225.9479	19547.8	225.9442	19537.64	225.9551	19540.01	225.9462	19540.76	226.7814	19540.25	226.8178	19539.58	228.2409
226.1958	19545.17	226.1931	19539.12	226.1942	19540.18	226.1994	19541.42	227.0254	19542.63	227.0812	19542.52	228.5018

226.4438	19547.67	226.4421	19541.7	226.4522	19538.33	226.4468	19541.06	227.2678	19540.4	227.5588	19540.36	228.7393
226.6952	19548.69	226.6931	19538.94	226.6919	19538.27	226.6935	19538.18	227.5177	19541.22	227.8136	19539.79	228.9934
226.9466	19546.73	226.947	19541.74	226.9577	19540.94	226.9456	19541.27	227.7706	19542.71	228.0666	19539.5	229.2406
227.1955	19549.74	227.1949	19539.11	227.1986	19541.14	227.1972	19539.38	228.0307	19541.22	228.3187	19543.28	229.4994
227.4435	19546.68	227.4464	19537.59	227.4511	19542.11	227.4473	19543.87	228.2761	19537.26	228.5692	19540.1	229.742
227.6973	19547.9	227.6966	19541.36	227.6951	19539.11	227.698	19542.02	228.5204	19539.38	228.8218	19544.04	229.9923
227.9487	19547.74	227.9529	19538.14	227.9549	19541.86	227.9462	19540.22	228.7665	19541.04	229.0884	19541.01	230.2424
228.195	19546.74	228.1965	19541.42	228.1948	19541.28	228.1991	19540.24	229.0166	19543.33	229.5714	19542.23	230.4997
228.443	19547.78	228.4472	19538.62	228.4687	19539.56	228.446	19542.53	229.2669	19541.8	229.8254	19543.05	230.7438
228.698	19545.78	228.6956	19538.47	228.7115	19540.9	228.696	19541.53	229.5186	19541.01	230.0864	19540.06	230.9932
228.9468	19549.58	228.9441	19539.17	228.9649	19540.2	228.9479	19539.22	229.7728	19540.41	230.3406	19541.68	231.2405
229.1957	19547.53	229.1955	19541.36	229.2073	19539.9	229.2015	19542.29	230.0166	19543.42	230.5948	19542.81	231.5004
229.4434	19548.45	229.4454	19542.53	229.4525	19539.64	229.4587	19540.61	230.2711	19539.86	231.0774	19541.57	231.7427
229.6978	19547.28	229.6967	19539.95	229.6963	19538.06	229.702	19542.12	230.5243	19542.11	231.3294	19538.5	231.9941
229.949	19548.11	229.9474	19541.73	229.9565	19539.03	229.9491	19539.65	230.773	19540.52	231.5838	19542.24	232.2426
230.1961	19549.62	230.1937	19541.73	230.1994	19540.43	230.2005	19539.36	231.018	19542.06	231.8418	19538.9	232.503
230.4438	19548.19	230.4524	19539.56	230.4507	19540.94	230.4462	19540.8	231.2672	19541.91	232.0986	19545.28	232.7395
230.6973	19549.53	230.7015	19539.24	230.6952	19543.09	230.6941	19539.14	231.528	19541.01	232.5807	19541.33	232.9942
230.9488	19547.47	230.9477	19542.95	230.9607	19541.47	230.9486	19541.02	231.7804	19540.12	232.8366	19541.26	233.2415
231.1983	19547.14	231.1951	19540.39	231.2048	19541.15	231.2014	19539.24	232.0303	19541.69	233.0932	19542.08	233.5008
231.4431	19547.37	231.4481	19539.97	231.4545	19540.06	231.4478	19538.88	232.2795	19541.28	233.349	19542.76	233.7443
231.6986	19547.77	231.6966	19541.22	231.6996	19539.69	231.6952	19538.97	232.5364	19540.42	233.8363	19543.42	233.995
231.949	19548.52	231.9472	19540.17	231.957	19541.25	231.9475	19541.7	232.795	19543.5	234.0988	19541.98	234.2423
232.2	19546.87	232.1966	19541.79	232.2027	19541.17	232.1994	19538.66	233.2755	19541.18	234.3555	19539.88	234.5032
232.4463	19547.41	232.4463	19539.01	232.4501	19538.67	232.4484	19542.35	233.5398	19541.42	234.6138	19539.68	234.7394
232.6966	19548.02	232.6971	19540.38	232.696	19538.94	232.6954	19539.11	233.8003	19538.52	235.0991	19541.92	234.9955
232.9499	19547.16	232.9468	19539.45	232.9588	19540.67	232.9479	19540.5	234.2905	19544.08	235.3537	19542.79	235.2452
233.1997	19547.3	233.1958	19541.19	233.2052	19540.68	233.2007	19542.78	234.5517	19541.92	235.6106	19541.26	235.5027
233.446	19545.78	233.4483	19539.19	233.4533	19542.27	233.45	19540.09	235.0441	19543.84	235.8717	19541.26	235.7429
233.6983	19548.91	233.6965	19540.72	233.7046	19538.91	233.696	19541.99	235.2858	19541.67	236.3575	19541.6	235.9959
233.9494	19546.88	233.9473	19541.32	233.9591	19541.33	233.9495	19540	235.5495	19541.01	236.6147	19542.62	236.2452
234.1968	19547.05	234.1964	19540.94	234.203	19538.52	234.2005	19540.06	235.8007	19541.84	236.8832	19542.71	236.5029
234.4457	19548.11	234.4464	19537.56	234.4536	19540.19	234.451	19539.1	236.0513	19540.94	237.3651	19542.64	236.7409
234.6986	19547.7	234.6991	19540.44	234.6994	19536.75	234.699	19538.71	236.2935	19540.09	237.6255	19537.73	236.998
234.9537	19546.2	234.9484	19539.13	234.956	19540.75	234.9513	19542.67	236.5454	19543.34	237.8872	19538.12	237.244
235.2027	19545.82	235.1967	19539.03	235.2025	19539.81	235.2044	19542.66	236.7966	19538.18	238.377	19541.47	237.5025
235.4495	19547.52	235.4478	19540.48	235.4519	19539.98	235.456	19539.46	237.0519	19541.19	238.6386	19541.31	237.7419
235.7011	19545.81	235.7	19539.8	235.7005	19539.63	235.7051	19543.09	237.3016	19540.02	238.9015	19539.8	237.9947
235.953	19546.51	235.9508	19537.9	235.958	19540.34	235.9559	19540.54	237.5497	19541.89	239.3855	19540.27	238.2454
236.2025	19548.83	236.1989	19540.31	236.2056	19540.71	236.2037	19539.69	237.814	19541.25	239.6475	19539.72	238.5042
236.4512	19547.3	236.4461	19539.48	236.4567	19543.02	236.4512	19539.37	238.2943	19542.17	239.9131	19540.45	238.7437
236.7025	19547.33	236.7146	19540.19	236.705	19539.93	236.6967	19537.81	238.5493	19542.64	240.4002	19540.96	239.0021
236.9562	19547.11	236.9688	19539.5	236.9634	19539.33	236.95	19541.42	238.8018	19539.89	240.6644	19540.76	239.2387
237.2085	19546.98	237.2226	19539.86	237.2124	19543.44	237.2018	19538.96	239.0629	19541.13	240.9233	19539.73	239.5032
237.4571	19544.82	237.4743	19542.61	237.4649	19539.71	237.4502	19540.12	239.308	19541.07	241.4024	19540.85	239.7434
237.7085	19548.1	237.728	19539.36	237.7137	19540.67	237.7014	19540.81	239.555	19539.58	241.6553	19540.24	239.9979
237.962	19550.61	238.2098	19538.14	237.971	19542.63	237.9527	19541.1	239.8056	19539.74	241.9558	19539.31	240.2481
238.215	19544.71	238.4664	19540.46	238.2209	19538.97	238.2021	19539.24	240.0541	19542.69	242.4461	19541.74	240.506
238.4645	19548.07	238.7217	19539.89	238.4736	19537.98	238.4506	19538.82	240.3043	19543.45	242.6966	19539.99	240.7423
238.7175	19546.06	238.9802	19538.01	238.7236	19537.71	238.6996	19542.66	240.5463	19541.47	242.9481	19541.09	240.9937
238.9707	19547.87	239.224	19541.08	238.9824	19542.47	238.9511	19541.84	240.8022	19539.83	243.196	19540.76	241.2431
239.2242	19546.02	239.4684	19540.27	239.4665	19540.86	239.2021	19542.46	241.05	19539.7	243.4424	19541.16	241.5138
239.4757	19546.92	239.7143	19540.55	239.7164	19539.44	239.4654	19541.09	241.2938	19539.22	243.6886	19543.25	241.7526
239.9554	19547.03	239.9595	19540.72	239.9763	19538.61	239.7095	19541.01	241.5455	19541.82	243.9444	19541.21	241.9929
240.2121	19547.12	240.2039	19538.5	240.2316	19540.42	239.9552	19539.32	241.7958	19539.37	244.1929	19542.42	242.2406
240.4634	19548.62	240.4578	19542.42	240.4884	19540.33	240.2032	19540.56	242.044	19543	244.4419	19543.55	242.5061
240.7137	19546.69	240.7003	19538.71	240.9766	19539.46	240.4533	19540.61	242.2954	19539.2	244.6913	19540.73	242.7446
240.9566	19545.17	240.9516	19540.13	241.2264	19541.18	240.6945	19540.33	242.5455	19541.6	244.9439	19539.57	242.9971
241.2005	19547.82	241.2008	19540.22	241.4717	19539.93	240.948	19541.37	242.8015	19542.15	245.2008	19539.08	243.2443
241.4506	19547.43	241.4486	19538.64	241.7156	19538.35	241.1989	19540.61	243.0474	19537.39	245.453	19540.86	243.5018
241.7017	19546.58	241.7139	19538.93	241.9731	19538.49	241.4466	19540.98	243.2941	19540.5	245.7039	19541.67	243.7438
241.9509	19546.32	241.9542	19538.36	242.2128	19542.45	241.6944	19542.01	243.5456	19541.07	245.9581	19540.73	243.9949
242.2011	19548.55	242.2062	19540.77	242.4681	19539.64	241.9471	19541.97	243.8016	19541.84	246.2088	19540.99	244.2414
242.4484	19545.5	242.4536	19540.17	242.7147	19539.82	242.1982	19539.66	244.0471	19540.3	246.4616	19540.88	244.5045
242.7029	19547.5	242.7046	19539.85	242.9737	19539.04	242.448	19541.53	244.2907	19538.85	246.9493	19538.51	244.7427
242.9533	19546.93	242.9532	19540	243.2149	19539.95	242.6946	19540.27	244.5455	19541.25	247.2046	19542.15	244.9954
243.2015	19547.42	243.2016	19538.06	243.4723	19540.65	242.9504	19540.18	244.7996	19539.71	247.4549	19540.5	245.243
243.4475	19547.78	243.4528	19539.25	243.7144	19540.25	243.1977	19538.17	245.0468	19540.51	247.7058	19541.05	245.5021
243.7009	19546.71	243.7045	19540.55	243.9755	19538.95	243.4506	19538.64	245.2945	19541.28	247.9625	19542.5	245.7449
243.952	19547.88	243.9567	19539.32	244.2176	19542.72	243.6962	19537.96	245.5449	19541.21	248.2148	19539.61	245.9959

244.2039	19546.98	244.2062	19539.24	244.4713	19538.98	243.9488	19541.71	245.798	19540.89	248.4646	19538.25	246.245
244.4494	19546.14	244.4513	19538.97	244.7123	19540.36	244.2004	19543.06	246.0479	19540.07	248.716	19539.44	246.5023
244.7038	19548.84	244.706	19540.46	244.9747	19538.54	244.4534	19541.8	246.2953	19542.08	249.1991	19540.55	246.7443
244.953	19546.78	244.9535	19539.88	245.2195	19537.01	244.6965	19540.16	246.5538	19540.58	249.4481	19543.45	246.9959
245.2025	19545.74	245.2025	19540.97	245.4706	19540.55	244.9488	19539.68	246.8045	19541.23	249.704	19542.95	247.2435
245.4495	19548.28	245.4514	19538.53	245.7123	19541.63	245.2019	19541.44	247.0538	19542.47	249.9587	19541.66	247.5024
245.704	19544.99	245.7049	19537.58	245.9779	19541.82	245.4604	19540.16	247.3095	19540.42	250.2111	19540.38	247.7495
245.9533	19547.88	245.9523	19540.37	246.2195	19539.5	245.7024	19540.94	247.5693	19541.66	250.4619	19542.87	247.9945
246.2016	19548.31	246.2039	19538.87	246.4731	19540.59	245.9516	19540.9	248.0665	19539.21	250.714	19538.79	248.2442
246.4504	19543.31	246.4555	19539.05	246.7186	19539.2	246.1993	19543.11	248.3222	19540.77	250.9709	19541.23	248.5032
246.7046	19545.74	246.7054	19540.42	246.9758	19538.43	246.4515	19541.09	248.5752	19538.91	251.4544	19542.55	248.7448
246.9636	19548.29	246.9547	19539.69	247.2191	19538.97	246.698	19538.74	249.0656	19542.34	251.7067	19538.55	248.9975
247.2034	19545.85	247.2059	19540.23	247.4686	19538.85	246.9502	19540.32	249.3176	19540.32	251.9636	19541.79	249.2439
247.4502	19546.91	247.4526	19541.92	247.7165	19539.57	247.2022	19542.4	249.574	19542.43	252.2093	19537.64	249.5031
247.7038	19547.49	247.7049	19539.42	247.9764	19541.18	247.4508	19537.02	249.8279	19542.77	252.4551	19541.68	249.7455
247.9564	19545.54	247.9566	19538.14	248.2239	19537.35	247.6962	19541.01	250.081	19540.24	252.7082	19539.15	249.9967
248.204	19546.81	248.207	19539.73	248.4723	19540.54	247.9512	19540.65	250.5713	19543.04	252.959	19540.05	250.2455
248.4514	19547.04	248.4556	19537.22	248.7141	19540.12	248.2022	19541.57	250.826	19543.46	253.2075	19541.11	250.505
248.7027	19547.41	248.7074	19538.01	248.9769	19540.59	248.4519	19541.93	251.0806	19541.31	253.4571	19541	250.7464
248.9548	19549.28	248.9602	19537.44	249.2222	19542.54	248.6979	19542.33	251.3304	19542.02	253.7083	19543.27	250.9979
249.2022	19548.15	249.2058	19539.09	249.4699	19540.22	248.951	19538.34	251.5763	19541.89	253.9625	19539.88	251.2441
249.4503	19547.95	249.4541	19539.21	249.7165	19539.87	249.1991	19538.4	251.8257	19540.31	254.2107	19542.01	251.5053
249.7048	19546.36	249.7189	19542.78	249.9741	19540.87	249.4506	19542.45	252.0886	19539.44	254.4558	19539.6	251.7449
249.9551	19544.13	249.9609	19537.35	250.2193	19538.95	249.699	19540.72	252.577	19541.22	254.7072	19541.3	252
250.2023	19547.43	250.2071	19540.8	250.4668	19537.4	249.9516	19540.16	252.8294	19540.64	254.9614	19541.51	252.2472
250.4508	19546.97	250.4588	19539.96	250.7189	19537.91	250.2026	19542.26	253.0938	19540.74	255.2119	19540.73	252.5085
250.7067	19543.11	250.7111	19539.95	250.9743	19539.08	250.4503	19541.02	253.5813	19541.95	255.4609	19539.87	252.7489
250.9572	19549.39	250.9658	19535.7	251.2196	19541.58	250.6984	19539.69	253.8318	19541.78	255.7099	19543.37	252.9988
251.2042	19547.97	251.2127	19539.81	251.4723	19538.88	250.9517	19540.4	254.0851	19542.09	255.96	19543.13	253.2511
251.4508	19544.94	251.4583	19538.34	251.7212	19538.91	251.2007	19537.29	254.3345	19539.44	256.2106	19541.09	253.5056
251.7053	19546.93	251.7068	19537.73	251.9782	19537.15	251.451	19539.85	254.5866	19538.55	256.4578	19541.93	253.7597
251.9579	19548.03	251.9568	19538.1	252.2167	19539.76	251.7004	19540.51	254.84	19541.12	256.7095	19539.41	254.0091
252.2045	19545.72	252.2069	19539.29	252.4772	19539.31	251.9518	19539.17	255.1053	19541.73	256.9628	19542.32	254.2489
252.4508	19547.27	252.454	19538.83	252.7184	19540.87	252.2079	19539.37	255.596	19541.76	257.2123	19539.28	254.5043
252.7034	19547.44	252.709	19542.03	252.9783	19538.92	252.4569	19538.51	255.8551	19541.66	257.4584	19541.42	254.747
252.9561	19545.1	252.9562	19539.37	253.2162	19541.49	252.7044	19539.39	256.1171	19541.07	257.7089	19541.2	254.9977
253.2056	19545.65	253.207	19538.72	253.4738	19538.65	252.9528	19539.42	256.6105	19542.09	257.962	19540.38	255.248
253.4532	19550.21	253.4567	19541.13	253.7187	19540.57	253.2052	19538.01	256.8619	19540.77	258.2179	19540.19	255.5048
253.7053	19548.97	253.7089	19540	253.9767	19538.89	253.457	19538.46	257.1255	19538.39	258.4691	19541.15	255.7489
253.9561	19545.39	253.9583	19537.69	254.216	19542.05	253.7045	19539.82	257.6133	19541.42	258.7192	19541.89	255.9993
254.208	19545.6	254.2131	19538.87	254.4753	19537.67	253.9646	19538.31	257.8681	19543.39	258.9742	19540.77	256.2462
254.4543	19544.64	254.463	19540.38	254.7204	19537.23	254.2153	19537.07	258.1359	19539.44	259.462	19540.06	256.5049
254.7058	19545.42	254.7144	19539.63	254.9763	19538.42	254.4665	19538.12	258.6253	19541.63	259.7137	19541.5	256.7484
254.9571	19545.94	254.9637	19540.68	255.2187	19539.25	254.7143	19540.37	258.8835	19540.96	259.9672	19540.92	257.0008
255.2055	19546.13	255.2142	19539.85	255.4722	19539.86	254.9671	19540.72	259.1456	19539.64	260.2218	19541.42	257.247
255.4534	19548.42	255.4608	19538.64	255.7251	19537.39	255.2194	19540.21	259.6375	19540.54	260.4704	19539.73	257.5079
255.7067	19548.27	255.7171	19539.9	255.9785	19539.01	255.4721	19539.09	259.8986	19539.9	260.7205	19540	257.7472
255.9615	19546.49	255.9677	19538.98	256.2178	19541.26	255.721	19537.94	260.1507	19540.05	260.9741	19540.42	258.0018
256.213	19546.46	256.2171	19539.78	256.4739	19538.47	255.9732	19540.88	260.6496	19540.23	261.4669	19542.66	258.257
256.4616	19545.36	256.4668	19539.99	256.7234	19539.31	256.4567	19539.99	260.9033	19538.36	261.7206	19541.5	258.508
256.7156	19547.39	256.7177	19539.64	256.983	19539.81	256.706	19540.33	261.1731	19539.73	261.9764	19539.57	258.7487
256.9676	19545.75	256.9664	19536.85	257.2257	19539.38	256.9571	19539.88	261.6624	19540.17	262.23	19542.15	259.0006
257.2182	19545.12	257.2172	19538.27	257.4744	19542	257.2092	19539.38	261.9204	19543.04	262.4818	19542.72	259.2484
257.4683	19547.16	257.4664	19539.45	257.7279	19541.47	257.4611	19541.83	262.1835	19540.13	262.9807	19542.34	259.5083
257.7243	19544.97	257.7186	19539.97	257.9759	19542.31	257.7122	19540.92	262.6738	19539.66	263.2353	19539.65	259.7496
258.2129	19544.7	257.9702	19540.1	258.2221	19537.91	257.9652	19537.64	262.9416	19539.79	263.7268	19542.1	260.0018
258.4613	19546.23	258.2214	19540.01	258.4747	19541.59	258.2187	19539.36	263.437	19541.65	263.9993	19542.39	260.2509
258.7128	19548.04	258.484	19538.39	258.7217	19541.01	258.4718	19539.93	263.6933	19539.71	264.4909	19540.67	260.5074
258.9663	19549.49	258.9672	19539.6	258.9787	19539.09	258.7229	19539.22	263.9596	19539.83	264.7521	19541.4	260.7501
259.2172	19548.11	259.2178	19539.39	259.2212	19539.71	258.9751	19542.33	264.4497	19542.95	265.2518	19540.06	261.0022
259.4662	19545.54	259.468	19539.23	259.476	19536.14	259.2314	19538.83	264.703	19541.71	265.5114	19541.48	261.2474
259.7185	19549.13	259.722	19539.38	259.7204	19538.65	259.7142	19536.68	264.9594	19543.12	266.008	19542.21	261.508
259.9732	19546.27	259.9768	19538.68	259.9789	19540.82	259.9688	19540.47	265.2181	19540.31	266.2756	19543.35	261.75
260.2248	19545.31	260.2286	19539.7	260.2219	19538.96	260.2221	19542.01	265.7078	19538.77	266.7715	19541.46	262.0039
260.4761	19545.87	260.4822	19539.17	260.4758	19539.16	260.4781	19539.82	265.9701	19542.01	267.0393	19540.66	262.2479
260.7294	19547.64	260.7351	19536.37	260.7176	19536.57	260.7306	19536.63	266.2338	19541.19	267.5303	19541.41	262.5088
260.9909	19548.06	260.9889	19539.22	260.9855	19537.62	260.9842	19542.38	266.7232	19541.17	267.7934	19540.91	262.7502
261.4737	19545.32	261.4743	19539.94	261.2356	19541.9	261.4721	19541.41	266.9793	19541.33	268.2871	19539.67	263.0041
261.7296	19546.88	261.7298	19538.47	261.4807	19539.61	261.7254	19539.55	267.2368	19540.3	268.5505	19538.93	263.2492
261.9868	19548.36	261.9843	19539.69	261.7252	19539.4	261.9825	19539.66	267.7343	19541.24	269.0456	19541.74	263.5104

262.2437	19545.06	262.2406	19540.63	261.979	19538.46	262.2388	19539.75	268.0012	19539.65	269.3173	19538.51	263.75
262.496	19548.49	262.4936	19540.56	262.222	19534.88	262.7223	19537.95	268.5038	19541.36	269.809	19542.03	264.0035
262.9846	19546.08	262.98	19538.34	262.4769	19539.42	262.984	19540.07	268.7592	19540.14	270.0764	19541.6	264.2523
263.2415	19546.18	263.234	19538.94	262.7202	19537.89	263.2446	19540.97	269.259	19542.08	270.5696	19544.07	264.5088
263.4943	19545.94	263.4915	19540.38	262.9803	19540.54	263.7292	19539.78	269.5177	19537.4	270.8347	19540.97	264.7501
263.7514	19545.36	263.7529	19540.47	263.2348	19538.9	263.9861	19538.79	270.0113	19542.56	271.3301	19541.94	265.0052
264.2364	19546.11	264.2386	19540.64	263.4905	19537.93	264.2418	19540.55	270.2661	19541.16	271.5922	19543.21	265.2471
264.49	19545.17	264.4971	19539.23	263.9867	19540.63	264.4974	19541.43	270.5251	19541.3	272.0905	19539.06	265.5061
264.7473	19545.54	264.7546	19538.42	264.2424	19537.98	264.9846	19538.6	271.0233	19539.54	272.3528	19542.7	265.7489
265.0032	19547.15	265.0151	19540.09	264.4985	19538.65	265.2418	19539.19	271.2813	19539.98	272.8478	19540.38	266.0047
265.2593	19548.4	265.5046	19539.1	264.9931	19537.83	265.4978	19540.85	271.7791	19541.18	273.1181	19540.68	266.2464
265.7444	19544.76	265.7807	19539.55	265.2485	19544.13	265.7514	19536.01	272.0409	19538.46	273.6129	19542.06	266.5101
266.0261	19546.8	266.266	19537.72	265.7421	19540.47	266.2417	19540.98	272.5357	19540.27	273.8798	19541.66	266.7541
266.5173	19547.69	266.5268	19541.93	266.0103	19537.53	266.5031	19541.99	272.7947	19537.63	274.3809	19539.3	266.9975
266.7726	19547.67	266.791	19541.55	266.5036	19539.55	266.7587	19539.75	273.2902	19540.27	274.6468	19541.25	267.2503
267.037	19546.21	267.277	19541.33	266.7592	19540.26	267.2522	19540.79	273.5489	19540.54	275.1471	19538.58	267.5092
267.5231	19546.59	267.54	19539.2	267.0246	19540.02	267.5094	19539.59	274.0527	19540.97	275.4134	19540.87	267.7529
267.78	19547.1	267.7966	19540.77	267.5181	19538.36	267.7652	19541.43	274.5477	19539.28	275.9143	19541.76	268.0032
268.0404	19547.62	268.2831	19539.75	267.7713	19538.7	268.2566	19538.27	274.8083	19541.2	276.1837	19540.53	268.2487
268.5258	19545.08	268.5451	19542.33	268.032	19540.1	268.5156	19541.17	275.3077	19541	276.6795	19539.69	268.5132
268.7885	19546.1	268.8024	19539.5	268.526	19538.55	268.7723	19538.18	275.5782	19537.86	276.9518	19539.31	268.7578
269.047	19546.65	269.2883	19540.82	268.78	19535.77	269.2625	19539.31	276.0829	19539.16	277.4508	19540.06	269.0049
269.5355	19547.44	269.5551	19540	269.0417	19536.84	269.5223	19539.82	276.5772	19539.64	277.7172	19540.24	269.2496
269.7958	19545.88	269.8135	19538.55	269.5362	19538.63	269.7799	19538.26	276.8408	19538.97	278.2171	19541.55	269.51
270.068	19546.11	270.3053	19537.46	269.7956	19538.47	270.2717	19538.45	277.3396	19538.31	278.4829	19542.41	269.7548
270.5561	19545.88	270.5687	19535.86	270.294	19536.1	270.5317	19540.4	277.601	19542.53	278.9848	19542.58	270.0027
270.8243	19543.17	270.8283	19539.78	270.5534	19540.3	270.791	19539.06	278.0975	19540.87	279.2538	19539.99	270.2494
271.3161	19545.2	271.3157	19539.91	271.0501	19540.9	271.2836	19540.27	278.3568	19539.36	279.7523	19539.08	270.5139
271.5754	19546.45	271.5784	19539.04	271.3084	19538.25	271.5429	19537.96	278.8578	19541.12	280.0231	19540.28	270.7606
271.837	19544.71	271.8386	19538.34	271.8045	19538.57	271.8018	19541.93	279.1233	19540.33	280.5169	19541.63	271.0084
272.3239	19546.45	272.3263	19539.53	272.0683	19542.15	272.2939	19539.89	279.6192	19540.65	280.7858	19540.92	271.2539
272.5865	19545.73	272.5971	19539.78	272.5657	19537.81	272.5559	19540.96	279.8925	19538.88	281.2886	19539.9	271.512
272.8472	19545.7	273.0887	19536.87	272.8234	19537.1	273.0433	19538.14	280.3869	19542.23	281.7875	19540.31	271.7583
273.3334	19545.08	273.3453	19538.64	273.3175	19538.97	273.3055	19538.33	280.6468	19540.42	282.06	19539.79	272.0062
273.5949	19549.73	273.6123	19538.75	273.5784	19539.6	273.5676	19539.97	281.1446	19539.86	282.5554	19539.56	272.254
273.8611	19546.64	274.1039	19538.84	274.076	19538.47	274.0566	19541.25	281.4053	19542.04	282.8246	19541.45	272.5082
274.35	19549.09	274.3634	19539.66	274.337	19538.8	274.3209	19540.17	281.9057	19541.77	283.3213	19541.24	272.7564
274.6119	19545.33	274.8567	19537.8	274.8303	19536.77	274.8121	19540.19	282.1825	19540.14	283.5912	19539.99	273.0046
274.8776	19547.18	275.1185	19538.57	275.0941	19538.43	275.072	19536.3	282.6778	19541.65	284.0951	19539.32	273.2588
275.3657	19546.17	275.3792	19537.69	275.5897	19538.97	275.5692	19540.3	282.947	19541.48	284.597	19541.25	273.5165
275.6313	19545.69	275.869	19537.94	275.8498	19535.9	275.8346	19541.13	283.4422	19537.7	284.8666	19540.24	273.7657
276.1383	19547.94	276.131	19537.97	276.3452	19540.44	276.3322	19537.67	283.7048	19539.43	285.3653	19539.03	274.0145
276.6333	19546.64	276.3932	19537.3	276.6061	19538.01	276.5948	19537.4	284.2069	19540.19	285.8681	19542.44	274.2618
276.8977	19546.42	276.8839	19537.67	277.1028	19539.56	277.0876	19538.83	284.4726	19539.58	286.1419	19542.11	274.5386
277.3864	19543.93	277.1477	19537.33	277.3646	19540.53	277.3567	19540.09	284.9835	19541.93	286.6419	19541.67	275.0243
277.6499	19545.6	277.645	19540.45	277.8594	19540.2	277.8506	19539.79	285.4789	19540.23	286.9158	19539.87	275.2746
277.9147	19547.18	277.906	19537.35	278.1421	19539.22	278.1111	19540.25	285.7327	19542.02	287.4111	19539.46	275.5286
278.4085	19548.67	278.3979	19538.7	278.6381	19539.56	278.6028	19539.67	285.9926	19540.37	287.6847	19540.49	275.7768
278.6731	19547.1	278.6645	19538.14	278.9045	19541.76	278.8675	19540.31	286.4941	19543.56	288.1869	19540.16	276.0268
279.1738	19545.81	279.1572	19538.2	279.403	19539.22	279.3636	19538.22	286.7442	19537.51	288.4613	19543.1	276.2767
279.4352	19544.89	279.419	19540.55	279.663	19537.84	279.6244	19541.01	287.0118	19539.38	288.9678	19540.01	276.5329
279.9308	19545.85	279.9153	19538.43	280.1603	19538.58	280.1181	19542.32	287.512	19538.48	289.4684	19539.45	276.7836
280.1953	19546.19	280.1773	19538.42	280.4282	19536.77	280.3869	19541.23	287.7644	19541.91	289.7423	19537.84	277.0355
280.703	19547.62	280.6738	19537.65	280.9268	19538.71	280.8802	19541.54	288.2742	19539.37	290.2487	19538.62	277.2868
281.2073	19546.8	280.9351	19539	281.1877	19539.24	281.1429	19538.62	288.7707	19539.73	290.5194	19540.57	277.5423
281.4707	19547.07	281.4268	19539.29	281.6848	19537.9	281.633	19539.81	289.0284	19540.44	291.027	19541.25	278.0276
281.9653	19547.32	281.7147	19538.65	281.9533	19537.12	281.8968	19541.03	289.5325	19541.43	291.5278	19541.11	278.2786
282.2296	19547.69	282.205	19539.47	282.4523	19539.1	282.3937	19536.13	290.0405	19540.53	291.802	19538.72	278.5342
282.7224	19546.8	282.4718	19538.79	282.7138	19536.41	282.6556	19540.43	290.544	19541.43	292.3025	19542.01	278.7846
282.9876	19548.36	282.9648	19534.39	283.211	19538.74	283.147	19540.02	291.048	19540.13	292.5764	19541.99	279.0387
283.4793	19546.31	283.2271	19538.75	283.4756	19537.52	283.4153	19539.07	291.5476	19540.15	293.0833	19541.11	279.2895
283.7438	19545.51	283.7225	19538.89	283.9757	19538.5	283.9105	19537.55	291.8086	19538.94	293.5864	19540.8	279.5484
284.2408	19545.67	283.9884	19538.63	284.2384	19538.44	284.1767	19539.3	292.3087	19539.3	293.8595	19540.34	280.0345
284.5075	19547.87	284.482	19536.79	284.7367	19536.9	284.6849	19540.03	292.5659	19539.36	294.3558	19540.29	280.2896
285.0027	19547.32	284.7496	19538.28	285.0072	19538.76	285.177	19542.01	293.0799	19541.93	294.6419	19538.88	280.547
285.2692	19543.87	285.2429	19537.14	285.5104	19537.92	285.4468	19541.5	293.5788	19539.1	295.1469	19540.6	280.7983
285.7632	19548.5	285.5085	19539.97	286.0145	19536.51	285.9427	19540.41	293.8373	19541.37	295.649	19539.43	281.0509
286.0316	19546.76	286.0072	19540.3	286.517	19538.73	286.208	19538.3	294.3441	19540.59	295.9265	19543.75	281.3071
286.5273	19546.26	286.2728	19538.08	286.7814	19538.7	286.7074	19536.57	294.8525	19539.43	296.4285	19539.5	281.7911
286.7958	19545.93	286.7697	19537.68	287.2794	19537.21	286.9656	19541.01	295.3572	19538.42	296.7041	19538.98	282.0448

287.2923	19545.16	287.0366	19539.39	287.5461	19539.59	287.4612	19539.29	295.8604	19541.09	297.2087	19538.39	282.3001
287.559	19545.97	287.5336	19538.86	288.0536	19537.21	287.7158	19538.09	296.364	19539.3	297.7124	19539.88	282.5589
288.056	19546.14	287.8021	19538.77	288.5631	19538.16	287.9739	19539.67	296.869	19539.28	298.2177	19540.01	282.8131
288.3222	19546	288.2957	19538.46	289.0656	19539.18	288.471	19538.2	297.3734	19538.75	298.7227	19541.16	283.3016
288.8227	19544.65	288.5686	19537.22	289.3304	19540.19	288.7311	19540.02	297.8803	19540.67	299.2405	19541.82	283.5599
289.1474	19546.34	289.0656	19538.89	289.8275	19538.18	289.23	19542.1	298.3813	19540.28	299.7434	19542.03	283.8156
289.6606	19543.81	289.3209	19539.12	290.0973	19540.54	289.489	19539.01	298.6513	19539.65	300.0202	19541.38	284.071
290.1915	19549.43	289.8194	19538.43	290.5976	19538.81	289.9854	19539.51	299.1553	19542.39			284.5613
290.693	19545.06	290.0786	19538.22	290.863	19539	290.2471	19540.9	299.6574	19539.81			284.8149
290.9837	19546.33	290.5793	19536.7	291.3613	19538.35	290.7418	19539.13	299.9297	19539.56			285.0708
291.4842	19546.62	290.8366	19537.49	291.6318	19541.07	291.0006	19540.56	300.4326	19540.49			285.3297
291.9944	19548.44	291.3299	19541.81	292.1345	19538.82	291.5077	19539.3					285.8207
292.5009	19545.28	291.5916	19537.13	292.4017	19539.69	292.0059	19539.88					286.0773
293.0231	19546.73	292.0903	19540.04	292.9086	19539.94	292.2672	19541.75					286.3397
293.5313	19546.78	292.3456	19539.69	293.4144	19538.28	292.7633	19538.32					286.8308
294.0368	19544.63	292.8538	19539.98	293.92	19540.97	293.0221	19537.11					287.0863
294.542	19545.71	293.3492	19536.53	294.4232	19539.75	293.5231	19538.73					287.3467
295.0574	19546.37	293.6134	19540.13	294.9266	19539.8	293.7801	19542.47					287.8372
295.5685	19545.5	294.1155	19539.4	295.4276	19538.79	294.2809	19540.52					288.0929
296.0729	19546.13	294.3771	19538.49	295.696	19539.89	294.5431	19539.13					288.3557
296.6329	19547.2	294.8722	19538.04	296.1962	19540.15	295.0402	19538.5					288.848
297.1464	19548.83	295.1325	19538.21	296.4678	19539.18	295.3018	19540.92					289.106
297.6521	19548.82	295.6364	19537.74	296.9777	19536.77	295.7979	19535.81					289.3691
298.1677	19544.45	296.1352	19540.42	297.4821	19537.78	296.0593	19538.58					289.8627
298.6783	19545.19	296.3942	19539.16	297.9881	19538.79	296.5555	19539.57					290.1189
299.1844	19547.91	296.8913	19540.52	298.4925	19536.44	296.8162	19539.16					290.3838
299.6871	19546.33	297.1518	19538.19	299.0088	19538.21	297.3179	19536.91					290.8777
299.9535	19545.31	297.6562	19540.34	299.5173	19539.42	297.5771	19538.95					291.136
300.4561	19546.74	298.1567	19539.79	300.0254	19539.44	298.0788	19539.32					291.3995
		298.415	19537.45			298.3477	19538.94					291.8936
		298.9124	19538.96			298.8476	19538.96					292.151
		299.1733	19537.57			299.1179	19541.82					292.4158
		299.6778	19537.37			299.614	19540.96					292.9101
		300.1765	19537			299.8878	19537.5					293.1693
						300.3924	19542.04					293.662
												293.9265
												294.4226
												294.6836
												295.1755
												295.4411
												295.9369
												296.1971
												296.6912
												296.9554
												297.4537
												297.7166
												298.209
												298.4749
												298.969
												299.229
												299.7222
												299.9848
												300.4849

1.43												
55												
1	x_2		x_3		x_4		x_5		x_1		x_2	
P (cm^{-1})	t (s)	P (cm^{-1})	t (s)	P (cm^{-1})	t (s)	P (cm^{-1})	t (s)	P (cm^{-1})	t (s)	P (cm^{-1})	t (s)	P (cm^{-1})
19551.06	0	19551.77	0	19552.49	0	19551.34	0	19555.69	0	19546.13	0	19549.89
19548.91	0.71574	19550.78	0.83949	19550.1	0.67458	19552.01	0.67834	19554.64	0.66085	19548.2	0.66128	19547.4
19551.69	0.8568	19550.8	1.19174	19552.86	0.85126	19550.98	0.85015	19551.36	0.85427	19549.07	0.85334	19549.55
19549.57	1.10424	19552.67	1.43902	19551.58	1.10211	19549.52	1.09814	19550.03	1.1068	19545.43	1.10307	19547.94
19549.73	1.35011	19551.07	1.69193	19551.12	1.35016	19554.36	1.3461	19553.98	1.35037	19545.11	1.35362	19547.15
19551.5	1.60059	19550.7	1.94126	19551.8	1.60181	19551.63	1.5973	19554.29	1.60481	19546.31	1.61532	19548.8
19551.52	1.84757	19552.61	2.19004	19551.49	1.85077	19552.11	1.84726	19551.51	1.85289	19546.4	1.851	19548.36
19551.1	2.09887	19553.55	2.43955	19552.64	2.09889	19551.49	2.09821	19552.81	2.10401	19547.76	2.0989	19548.28
19549.2	2.34861	19549.87	2.68973	19551.64	2.34926	19554.62	2.346	19553.56	2.35033	19548.59	2.35167	19546.59
19550.52	2.59894	19549.32	2.9411	19551.6	2.60054	19553.4	2.59576	19554.31	2.60376	19547.29	2.60101	19548.77
19549.17	2.85085	19549.62	3.1916	19552.92	2.85117	19552.19	2.8462	19554.68	2.85408	19548.25	2.85258	19545.29
19551.01	3.09959	19551.47	3.43946	19552.69	3.10077	19553.05	3.09654	19552.93	3.10628	19544.66	3.0991	19545.18
19548.28	3.34925	19549.63	3.69008	19550.53	3.35082	19552.36	3.34783	19553.45	3.35052	19548.14	3.35431	19548.81
19551.07	3.60006	19550.12	3.93948	19551.97	3.60033	19550.11	3.59671	19554.07	3.60359	19548.02	3.60129	19546.52
19550.06	3.84809	19550.56	4.19208	19549.43	3.85034	19553.7	3.84626	19553.58	3.85421	19549.76	3.85258	19546.59
19553.37	4.09929	19551.05	4.44107	19551.33	4.10128	19551.58	4.09601	19554.53	4.10631	19549.01	4.10142	19548.03
19550.04	4.34922	19551.55	4.69091	19551.97	4.35148	19552.31	4.34726	19552.27	4.35189	19547.34	4.35382	19548.02
19549.53	4.60503	19555.66	4.94043	19552.87	4.60043	19552.69	4.59736	19549.96	4.60448	19547.17	4.60042	19547.29
19551.71	4.85481	19550.86	5.19626	19552.63	4.8505	19551.6	4.84682	19552.99	4.85765	19547.03	4.85361	19546.72
19553.95	5.09973	19550.17	5.44404	19552.2	5.10184	19551.02	5.10184	19554.01	5.10901	19548.17	5.10639	19547.34
19551.47	5.34918	19553.04	5.69112	19550.72	5.35533	19551.07	5.35129	19553.39	5.35203	19547.79	5.35691	19545.42
19550.02	5.6	19550.09	5.93982	19552.3	5.60732	19552.55	5.59687	19555.45	5.60498	19549.31	5.60022	19544.13
19551.22	5.84973	19550.15	6.19202	19552.46	5.85164	19549.71	5.84709	19553.78	5.85145	19546.58	5.8524	19547.38
19550.77	6.09995	19551.16	6.44083	19552.31	6.10042	19552.52	6.09742	19552.49	6.10581	19549.2	6.10149	19545.29
19550.66	6.34939	19551.47	6.69231	19553.2	6.35036	19551.47	6.34824	19554.17	6.35184	19544.66	6.35628	19546.62
19549.86	6.61038	19552	6.94344	19553.13	6.60252	19551.97	6.59724	19553.11	6.60598	19546.18	6.60194	19547.79
19550.47	6.84985	19552.36	7.19184	19552.06	6.85359	19553.39	6.84796	19554.96	6.85347	19545.81	6.85442	19548.56
19551.51	7.10071	19553.25	7.44025	19551.31	7.10209	19552.55	7.09773	19554.81	7.10675	19546.45	7.10101	19547.53
19552.54	7.35014	19551.92	7.69084	19551.93	7.35143	19550.31	7.34887	19555.28	7.35165	19550.26	7.35672	19546.79
19550.44	7.60042	19550.38	7.94205	19550.09	7.60222	19550.99	7.59755	19555.51	7.60465	19546.42	7.60196	19548.01
19550.73	7.84936	19552.02	8.19373	19552.84	7.85103	19549.9	7.84732	19553.08	7.85408	19546.25	7.85457	19546.24
19551.06	8.10097	19551.86	8.4419	19552.35	8.10238	19553.2	8.0988	19554.86	8.10871	19546.69	8.1017	19548.2
19550.07	8.35044	19552.48	8.69172	19551.22	8.35314	19552.65	8.3491	19555.1	8.35285	19548.06	8.35838	19545.72
19549.73	8.60058	19551.07	8.94157	19550.22	8.60078	19555.14	8.59769	19553.88	8.60528	19545.33	8.6028	19548.22
19550.28	8.85075	19551.15	9.19313	19550.2	8.85155	19552.65	8.84794	19551.77	8.85311	19547.88	8.85476	19546.08
19553.04	9.10079	19550.19	9.46081	19553.26	9.10244	19551.69	9.09885	19554.59	9.1089	19549.08	9.10269	19546.97
19548.65	9.35011	19550.86	9.6928	19552.36	9.35316	19553.78	9.34971	19553.23	9.35496	19547.05	9.35569	19546.92
19550.36	9.60223	19554.77	9.94122	19550.2	9.60257	19554.7	9.59894	19555.41	9.60488	19546.56	9.6019	19548.5
19550.69	9.85131	19552.62	10.19362	19554.59	9.85188	19551.38	9.84803	19551.57	9.85255	19545.47	9.85503	19547.5
19551.49	10.1038	19547.19	10.44333	19551.38	10.10215	19551.24	10.0985	19558.37	10.10845	19547.35	10.10339	19546.37
19550.77	10.35091	19552.85	10.69325	19553.67	10.35305	19550.55	10.34924	19553.44	10.35425	19547.42	10.35897	19546.6
19552.75	10.60151	19551.99	10.94264	19550.89	10.60343	19553.16	10.60079	19550.37	10.60688	19546.73	10.60273	19548.32
19549.86	10.85129	19550.67	11.19493	19551.72	10.85246	19551.06	10.84986	19554.78	10.85647	19550.67	10.85486	19547.65
19553.15	11.10615	19551.01	11.44306	19551.8	11.10281	19552.25	11.09884	19553.03	11.10809	19547.43	11.10483	19547.09
19550.23	11.35145	19550.63	11.69529	19552.84	11.35288	19550.47	11.34949	19551.8	11.35429	19546.41	11.35987	19547.6
19550.3	11.60145	19552.02	11.94389	19552.45	11.60413	19551.59	11.59981	19553.72	11.60815	19548.09	11.60345	19547.39
19550.04	11.85112	19551.73	12.1944	19549.47	11.85489	19549.87	11.85057	19553.93	11.85447	19546.65	11.85505	19547.41
19548.86	12.10386	19551.66	12.44235	19551.06	12.10297	19551.49	12.10081	19553.23	12.10779	19549.27	12.10385	19548.37
19551.03	12.3528	19553.8	12.69282	19551.72	12.35281	19551.33	12.34968	19552.65	12.35413	19547.75	12.35607	19545.87
19548.87	12.60364	19550.19	12.94443	19549.8	12.60484	19550.23	12.60032	19554.05	12.60849	19547.24	12.60431	19549.18
19552.85	12.85143	19552.3	13.1948	19551.18	12.85396	19552.93	12.85073	19554.34	12.85417	19548.44	12.85479	19548.12
19550.71	13.10647	19551.72	13.44319	19552.63	13.10461	19551.64	13.09999	19553.47	13.11588	19547.07	13.10266	19545.14
19550.4	13.35107	19552.4	13.69303	19551.5	13.35314	19553.52	13.34979	19552.67	13.35366	19545.07	13.35682	19545.91
19551.46	13.60174	19551.78	13.9429	19552.39	13.6025	19553.36	13.59975	19555.93	13.6079	19546.93	13.60398	19546.82
19553.73	13.85165	19550.71	14.19443	19552.86	13.85331	19554.37	13.84933	19552.35	13.85519	19547.4	13.85563	19547.17
19550.46	14.10398	19551.01	14.44337	19552.01	14.10628	19554.6	14.10032	19551.51	14.11028	19548.02	14.1029	19548.66
19550.32	14.35103	19551.88	14.70197	19553.52	14.3542	19553.38	14.35023	19553.38	14.35402	19548.04	14.35858	19547.05
19550.56	14.60114	19550.94	14.94258	19551.33	14.60281	19552.42	14.60023	19552.93	14.6132	19546.44	14.60468	19548.53
19549.99	14.85318	19552.14	15.19534	19551.22	14.85312	19549.7	14.84935	19551.8	14.85497	19547.2	14.85614	19545.54
19549.77	15.10521	19550.59	15.44479	19549.87	15.1047	19554.76	15.09915	19551.62	15.111	19550.03	15.10339	19547.56
19552.72	15.35253	19552.36	15.69494	19553.33	15.35488	19551.44	15.3508	19554.33	15.3559	19548.29	15.35771	19549.68
19549.19	15.602	19549.35	15.944	19551.88	15.60455	19551.64	15.60166	19553.38	15.60926	19548.34	15.60445	19550.12
19549.23	15.85207	19550.96	16.19473	19551.05	15.85382	19552.14	15.85119	19553.97	15.85457	19546.24	15.85773	19545.45
19550.6	16.1048	19550.87	16.44454	19549.76	16.10595	19552.56	16.10033	19553.5	16.11236	19549.27	16.10489	19548.03

19548.27	16.35361	19551.62	16.69538	19551.83	16.35552	19552.3	16.35091	19553.84	16.35614	19547.04	16.35989	19546.66
19550.34	16.60245	19550.22	16.94454	19553.54	16.60574	19551.88	16.60162	19551.95	16.60919	19551.04	16.60444	19546.93
19551.15	16.85154	19550.02	17.19515	19552.52	16.85356	19553.92	16.8516	19552.77	16.85534	19544.21	16.85748	19546.78
19551.15	17.10437	19551.83	17.44338	19551.73	17.10532	19553.18	17.10141	19554.05	17.1112	19546.94	17.1062	19546.82
19552.33	17.35268	19551.04	17.69791	19552.93	17.35472	19552.23	17.35092	19553.71	17.35492	19548.01	17.35949	19548.34
19549.7	17.60307	19551.11	17.94765	19551.58	17.60673	19553.62	17.60051	19553.83	17.60948	19548.75	17.6039	19549.17
19550.68	17.85257	19550.29	18.19735	19551.77	17.8548	19551.62	17.85258	19554.05	17.8558	19545.89	17.85732	19548.9
19549.46	18.10399	19551.27	18.44544	19550.86	18.1042	19552.31	18.10198	19555.59	18.10926	19547.63	18.10585	19547.28
19550.67	18.35529	19553.28	18.6946	19550.75	18.35491	19552.19	18.35079	19554.94	18.35467	19548.95	18.36132	19548.37
19550.84	18.60418	19550.76	18.94646	19553.74	18.60488	19553.71	18.60058	19551.16	18.61019	19543.11	18.6046	19547.68
19551.42	18.85328	19550.81	19.19627	19554.53	18.8562	19552.57	18.85169	19552.18	18.85639	19548.52	18.85803	19546.27
19550.05	19.1069	19549.3	19.44742	19551.71	19.1052	19551.84	19.10236	19552.17	19.11198	19548.33	19.10521	19546.53
19550.54	19.35277	19553.38	19.69682	19553.33	19.35451	19553.4	19.35096	19554.75	19.35528	19547.05	19.35895	19546.13
19550.4	19.60294	19551.57	19.94481	19552.96	19.60527	19552.84	19.60123	19551.85	19.6094	19548.4	19.60693	19546.19
19552.48	19.85388	19550.15	20.19678	19552.91	19.85605	19550.6	19.85077	19553.11	19.85674	19547.52	19.85846	19547.46
19551.22	20.10689	19552.21	20.44639	19551.81	20.10752	19550.76	20.10329	19551.45	20.11182	19546.71	20.10517	19546.48
19550.92	20.35437	19551.65	20.69765	19552.1	20.35753	19552.39	20.3537	19552.95	20.35814	19548.66	20.36033	19546.57
19550.53	20.60368	19550.37	20.94558	19554.43	20.60555	19552.38	20.60227	19554.33	20.61137	19549.08	20.60781	19550.32
19552.01	20.85435	19551.15	21.19738	19551	20.85603	19552.42	20.85237	19553.42	20.85629	19544.83	20.86124	19548.93
19550.94	21.10644	19551.3	21.44679	19553.37	21.10652	19551.85	21.10284	19552.72	21.11718	19547.11	21.10697	19546.64
19550.75	21.35559	19550.2	21.69767	19551.23	21.35686	19552.53	21.35335	19554.33	21.35699	19546.76	21.36166	19547.96
19550.59	21.60526	19550.96	21.94594	19550.24	21.60781	19549.62	21.60382	19553.52	21.61168	19547.26	21.60744	19545.71
19551.2	21.85485	19551.28	22.19739	19550.78	21.856	19552.82	21.85325	19552.04	21.85774	19545.58	21.86231	19547.3
19549.94	22.10651	19550.65	22.44735	19552.77	22.10624	19553.66	22.10405	19554.22	22.11129	19546.2	22.10804	19547.53
19551.12	22.35525	19551.13	22.69997	19551.71	22.35771	19552.44	22.35264	19553.16	22.35737	19550.2	22.36412	19546.81
19550.36	22.60606	19552.55	22.94744	19551.1	22.60892	19551.41	22.60458	19553.81	22.6103	19550.03	22.60738	19548.24
19548.81	22.87116	19551.96	23.19807	19550.03	22.85631	19552.96	22.8546	19552.3	22.85901	19546.48	22.86167	19547.82
19550.64	23.10597	19551.56	23.44584	19552.33	23.10608	19552.3	23.10377	19554.9	23.11327	19547.33	23.10873	19547.44
19547.08	23.35665	19551.74	23.69866	19553.03	23.35719	19550.51	23.35316	19554.5	23.35732	19545.73	23.36227	19546.39
19550.26	23.60628	19551.71	23.9491	19552.19	23.60854	19552.09	23.60444	19552.99	23.61101	19546.75	23.60749	19545.88
19551.14	23.85694	19553.37	24.19891	19553.48	23.8571	19553.83	23.85437	19553.98	23.85797	19548.55	23.86146	19546.15
19549.77	24.10924	19550.06	24.44656	19551.84	24.10705	19552.15	24.10414	19553.29	24.11383	19548.07	24.10896	19549.55
19549.68	24.35538	19550.89	24.69584	19553.55	24.35775	19551.9	24.3536	19553.03	24.35928	19546.51	24.36353	19546.85
19549.79	24.60487	19551.08	24.95017	19552.57	24.60805	19552.21	24.60242	19553.04	24.61142	19548.9	24.60696	19547
19551.37	24.85745	19552.99	25.19904	19552.44	24.85743	19552.16	24.85662	19551.3	24.85746	19546.36	24.86065	19546.89
19550.59	25.10785	19549.04	25.44805	19553.01	25.10636	19551.94	25.10457	19552.74	25.11259	19544.74	25.10729	19547.42
19549.6	25.35543	19552.55	25.69667	19551.81	25.35683	19553.31	25.35299	19553.63	25.35869	19548.12	25.36345	19546.67
19550.99	25.6051	19550	25.9461	19551.29	25.60811	19552.27	25.60282	19554.44	25.61226	19544.55	25.60786	19547.62
19550.18	25.85633	19550.38	26.19857	19552.41	25.85784	19554.05	25.85338	19554.01	25.85788	19548.86	25.86034	19543.93
19550.96	26.10948	19550.33	26.44835	19550.17	26.12073	19553.35	26.10516	19555.54	26.11318	19546.56	26.10823	19546.11
19550.42	26.35614	19552.37	26.69664	19551.64	26.35703	19551.91	26.35391	19552.14	26.35831	19550.16	26.36251	19546.03
19549.6	26.60609	19551.04	26.94756	19552.05	26.60687	19550.67	26.60276	19552.07	26.61793	19545.57	26.60873	19547.6
19551.15	26.85619	19550.83	27.19916	19553.63	26.8579	19551.6	26.85386	19552.57	26.85863	19546.36	26.86033	19546.55
19550.32	27.10733	19551.41	27.44922	19550.4	27.10867	19552.24	27.10444	19552.28	27.11445	19546.9	27.10698	19546.9
19549.86	27.35673	19551.78	27.69932	19552.94	27.35802	19552.67	27.35477	19552.69	27.35767	19546.84	27.36415	19548.97
19550.05	27.6062	19551.56	27.94854	19552.46	27.60931	19550.13	27.60406	19555.48	27.61184	19547.22	27.60806	19545.52
19549.9	27.85609	19552.72	28.19831	19552.89	27.85703	19551.57	27.85338	19553.62	27.86011	19543.96	27.86169	19545.39
19550.66	28.1106	19551.84	28.44831	19550.89	28.10782	19554.62	28.10538	19553.49	28.11392	19550.01	28.10771	19548.78
19550.13	28.35616	19551.09	28.69992	19552.87	28.35868	19553.99	28.35572	19554.61	28.35787	19547.85	28.36278	19548.79
19549.67	28.60653	19549.69	28.9492	19551.83	28.60855	19551.87	28.60487	19553.1	28.61093	19546.14	28.60884	19548.15
19550.75	28.85759	19552.88	29.19863	19553.65	28.85817	19550.69	28.85347	19555.25	28.85898	19544.81	28.86167	19545.37
19550.24	29.10944	19548.97	29.44815	19550.74	29.10818	19551.34	29.10432	19554.44	29.11585	19546.99	29.10864	19548.37
19548.71	29.35545	19550.15	29.69869	19552.2	29.3603	19551.15	29.35527	19555.66	29.359	19547.5	29.36428	19546.94
19550.72	29.60657	19550.75	29.95005	19552.12	29.61028	19553.28	29.60542	19554.17	29.61262	19547.63	29.60863	19548.54
19550.91	29.85869	19551.57	30.19955	19552.53	29.85818	19553.1	29.85556	19552.83	29.85896	19548.19	29.86211	19547.35
19552.4	30.10855	19552.5	30.44941	19551.79	30.10794	19553.53	30.105	19551.89	30.11461	19546.91	30.11017	19548.68
19551.03	30.35742	19550.78	30.69907	19551.45	30.35984	19552.48	30.35494	19553.7	30.36085	19545.31	30.36449	19546.64
19550.45	30.60805	19550.78	30.95032	19551.8	30.61073	19551.57	30.60719	19553.45	30.61498	19548.3	30.61014	19548.01
19551.14	30.85855	19551.44	31.20185	19551.48	30.85991	19551.75	30.85819	19553.83	30.85894	19548.8	30.86407	19547.56
19551.74	31.1119	19551.57	31.45148	19554.52	31.10949	19551.16	31.10596	19553.45	31.11482	19546.06	31.11128	19548.62
19549.72	31.35853	19550.92	31.70081	19552.36	31.36006	19550.79	31.35585	19552.08	31.36093	19547.14	31.36611	19549.17
19550.1	31.60702	19551.36	31.94985	19551.59	31.61095	19552.98	31.60672	19553.07	31.61578	19546.59	31.60977	19549.14
19552.33	31.85939	19550.95	32.20235	19552.14	31.86067	19553.88	31.85798	19552.85	31.86048	19549.18	31.8634	19549.21
19551.08	32.11239	19551.73	32.45112	19553.45	32.11798	19554.47	32.10759	19552.88	32.11564	19548.91	32.1109	19549.17
19550.41	32.36028	19551.75	32.69879	19553.76	32.36156	19550.25	32.35664	19553.05	32.3605	19545.55	32.36796	19547.72
19550.79	32.60837	19551.81	32.94872	19552.59	32.61089	19552.11	32.61512	19555.04	32.61443	19548.99	32.61093	19545.78
19550.47	32.85797	19551.31	33.20018	19551.5	32.85973	19551.39	32.85616	19552.08	32.86066	19548.06	32.86303	19546.71
19550.69	33.11035	19549.73	33.45179	19553.87	33.11142	19552.99	33.1078	19554.53	33.11529	19549.64	33.1097	19548.67
19550.13	33.35812	19553.73	33.70028	19550.72	33.36156	19549.87	33.35724	19551.32	33.35882	19545.47	33.36763	19549.31
19550.89	33.60882	19549.5	33.94877	19551.51	33.61012	19552.67	33.60622	19552.03	33.6141	19547.89	33.61165	19548.88

19551.22	33.85832	19553.54	34.20038	19554.43	33.85899	19551.07	33.85565	19553.32	33.86046	19548.96	33.86479	19547.28
19550.44	34.11163	19550.6	34.45036	19550.27	34.11061	19551.4	34.10671	19550.73	34.11523	19547.33	34.11028	19548.61
19550.4	34.35728	19551.96	34.70262	19551.53	34.36055	19551.32	34.35682	19549.78	34.3603	19548.05	34.36457	19548.39
19550.81	34.60891	19549.96	34.9506	19552.8	34.61049	19550.07	34.60716	19555.11	34.61571	19547.8	34.61053	19547.66
19550.45	34.85953	19551.72	35.20031	19551.91	34.85937	19551.92	34.85574	19556.16	34.86104	19548.07	34.86393	19550.65
19550.64	35.11296	19550.42	35.45222	19551.17	35.10916	19552.13	35.10623	19552.88	35.11673	19548.14	35.10982	19547.47
19551.44	35.35802	19552.49	35.70124	19551.14	35.3614	19551.27	35.35719	19553.33	35.36271	19547.34	35.36644	19548.4
19549.25	35.60775	19550.39	35.95066	19552.99	35.61125	19552.41	35.60782	19554	35.61522	19546.87	35.61226	19549.14
19549.63	35.85952	19549.5	36.20043	19551.48	35.86137	19553.45	35.85821	19554.96	35.86089	19545.09	35.86524	19547.74
19551.89	36.11311	19549.79	36.45108	19552.65	36.11091	19550.79	36.10718	19553.85	36.11601	19545.45	36.11203	19548.85
19549.9	36.35965	19554.2	36.70067	19551.01	36.3606	19552.45	36.35588	19552.7	36.36279	19548.47	36.36878	19548.86
19550.51	36.61215	19551.24	36.95114	19553.58	36.61028	19551.61	36.61033	19552.21	36.61577	19547.04	36.61016	19546.94
19551.52	36.85934	19552.41	37.20276	19552.28	36.86138	19551.99	36.85975	19552.54	36.86097	19548.28	36.86463	19547.13
19549.76	37.11288	19552.21	37.45085	19550.38	37.11288	19550.43	37.10697	19553.57	37.11612	19545.32	37.11195	19547.67
19548.21	37.36172	19550.56	37.7014	19553.98	37.36074	19551.71	37.35613	19550.81	37.36298	19546.55	37.36964	19545
19549.69	37.60973	19551.1	37.95085	19550.44	37.61202	19553.13	37.60683	19551.21	37.6158	19545.86	37.61021	19547.76
19550.99	37.86018	19552.47	38.20157	19553.75	37.86204	19550.48	37.85835	19554.6	37.86183	19545.56	37.86459	19545.2
19553.14	38.11344	19551.89	38.4504	19551.33	38.11328	19552.96	38.10945	19553.68	38.11745	19544.59	38.11234	19546.69
19549.99	38.36064	19551.03	38.70246	19550.59	38.36127	19553.47	38.3582	19554.42	38.36218	19547.87	38.371	19548.65
19550.19	38.61088	19551.04	38.95156	19551.22	38.61077	19550.12	38.60714	19552.5	38.6165	19547.1	38.61233	19548.94
19551.77	38.85933	19551.72	39.20153	19550.84	38.86135	19550.85	38.85825	19554	38.86282	19548.4	38.86525	19547.06
19550.46	39.11468	19550.83	39.45129	19552.58	39.11222	19551.4	39.10893	19554.73	39.11798	19546.88	39.11126	19545.8
19551.24	39.36031	19552.46	39.70217	19552.3	39.3629	19552.23	39.35842	19553.43	39.36215	19547.4	39.36979	19548.55
19550.97	39.61179	19549.18	39.95347	19552.15	39.61072	19553.95	39.60813	19552.86	39.6151	19546.72	39.6134	19547.14
19552.27	39.86076	19551.46	40.20189	19551.7	39.8612	19551.6	39.85687	19554.26	39.86226	19548.81	39.86696	19548.06
19551.22	40.1158	19550.67	40.45178	19551	40.11219	19552.9	40.10786	19553.08	40.13663	19546.3	40.11284	19547.72
19551.08	40.36118	19552.18	40.70351	19552.48	40.36333	19551.38	40.36016	19554.42	40.36506	19549.12	40.37016	19547.3
19550.07	40.61169	19552.64	40.95357	19553.92	40.61259	19552.33	40.61001	19551.27	40.62226	19546.26	40.61357	19545.6
19551.06	40.86252	19552.43	41.20527	19550.61	40.86187	19549.76	40.85909	19555.45	40.86289	19547.23	40.86766	19548.42
19550.38	41.11857	19552.5	41.45213	19552.54	41.11324	19552.01	41.10934	19554.15	41.11968	19548.12	41.11479	19546.08
19551.03	41.36063	19553.81	41.70328	19552.63	41.36389	19552.8	41.36023	19553.58	41.36458	19543.16	41.37073	19548.65
19549.94	41.61107	19551.2	41.95377	19552.11	41.61458	19549.98	41.61147	19552.89	41.62787	19547.69	41.61375	19548.44
19549.93	41.86139	19550.78	42.2051	19551.17	41.86465	19551.02	41.86281	19552.1	41.86299	19548.87	41.86816	19547.72
19550.87	42.12169	19550.46	42.45358	19551.22	42.11334	19553.15	42.11024	19552.15	42.11925	19546.35	42.11606	19548.61
19550.28	42.36201	19554.07	42.70248	19553.17	42.36324	19553.03	42.36078	19550.68	42.36414	19547.92	42.37198	19545.35
19550.59	42.61083	19552.28	42.95301	19551.23	42.61643	19550.46	42.61004	19553.28	42.61885	19546.97	42.61427	19547.21
19549.94	42.86361	19550.75	43.20531	19553.01	42.86706	19551.9	42.86092	19554.3	42.86506	19546.96	42.8689	19547.19
19550.59	43.11676	19550.28	43.45501	19553.31	43.11526	19554.12	43.11137	19551.94	43.11923	19548.44	43.11567	19546.26
19551.15	43.36277	19550.39	43.70353	19552.44	43.36402	19553.22	43.36067	19552.78	43.36398	19548.79	43.37273	19546.26
19550.2	43.61187	19551.92	43.95316	19551.36	43.61405	19553.28	43.60954	19553.98	43.61916	19545.38	43.61513	19547.03
19547.6	43.86182	19552.3	44.20524	19553.45	43.8654	19554.84	43.86076	19554.51	43.86506	19546.28	43.8684	19548.25
19549.02	44.1228	19551.43	44.45491	19551.89	44.11553	19554.03	44.11139	19554.43	44.12014	19549.14	44.11585	19548.58
19551.75	44.36718	19549.56	44.70651	19551.07	44.3637	19552.62	44.3633	19553.27	44.36406	19548.44	44.37454	19546.59
19553	44.61319	19551.34	44.95518	19550.88	44.61553	19553.93	44.61126	19553.45	44.6203	19545.84	44.61558	19546.19
19549.56	44.86226	19550.74	45.2053	19551.14	44.86566	19553.93	44.86062	19552.61	44.86667	19547.25	44.86917	19548.91
19549.71	45.11841	19550.43	45.45537	19552	45.11625	19552.51	45.1108	19553.5	45.12165	19546.83	45.11444	19547.13
19549.88	45.3629	19549.47	45.70588	19553.24	45.3642	19552.41	45.36251	19555.79	45.36596	19548.04	45.37271	19548.48
19552.08	45.61361	19552.25	45.95949	19552.52	45.614	19552.76	45.6123	19555.24	45.61953	19544.69	45.61594	19547.29
19552.86	45.86364	19552	46.20686	19551.29	45.86649	19554.87	45.86187	19552.41	45.86565	19548.58	45.86957	19549.6
19549.26	46.1182	19549.88	46.45404	19551.95	46.11647	19552.54	46.11104	19555.12	46.12163	19548.94	46.11653	19545.3
19550.82	46.36384	19551.91	46.70553	19553.6	46.36477	19552.61	46.36295	19551.72	46.36628	19547.75	46.37258	19548.68
19550.91	46.61513	19551.63	46.95563	19548.27	46.61382	19552.35	46.61275	19552.87	46.62073	19545.67	46.61496	19550.25
19549.61	46.86377	19550.2	47.2066	19553.53	46.86522	19550.29	46.86259	19552.55	46.86469	19547.6	46.87129	19548.17
19549.97	47.12148	19550.32	47.45498	19552.77	47.11599	19551.96	47.11065	19553.4	47.12112	19547.2	47.11725	19547.88
19548.94	47.36356	19552.54	47.70551	19550.23	47.36586	19549.65	47.36155	19554.56	47.36853	19547.49	47.37469	19548.22
19550.28	47.61424	19552.26	47.95542	19552.29	47.61721	19549.55	47.61233	19553.96	47.62149	19548.68	47.6147	19548.92
19551.88	47.86537	19551.66	48.20604	19552.13	47.86495	19553.8	47.86229	19550.93	47.86522	19547.71	47.87025	19548.39
19549.48	48.12119	19552.64	48.45619	19552.3	48.11562	19550.99	48.11379	19554.35	48.12144	19549.03	48.11696	19548.05
19550.12	48.36262	19552.09	48.70535	19552.36	48.36601	19552.29	48.36182	19553.98	48.36739	19547.33	48.37429	19547.7
19550.61	48.61331	19552.59	48.95303	19552.19	48.61553	19552.03	48.6111	19553.57	48.62056	19546.68	48.61514	19547.99
19550.97	48.86426	19551.87	49.20603	19549.66	48.86447	19551.87	48.86208	19553.08	48.86597	19547.55	48.86917	19547.75
19550.56	49.11891	19552.27	49.45489	19550.58	49.11392	19556.11	49.11194	19554.22	49.11801	19546.42	49.11536	19547.47
19551.84	49.36387	19551.33	49.70711	19550.07	49.36444	19555.69	49.36051	19553.79	49.36508	19546.18	49.37779	19548.1
19551.75	49.6157	19552.44	49.95683	19551.43	49.61713	19550.35	49.61374	19553.84	49.62152	19548.1	49.61762	19548.48
19552.67	49.86373	19551.87	50.20465	19552.47	49.86542	19552.89	49.8626	19551.8	49.86684	19546.15	49.86931	19544.83
19550.07	50.12038	19549.4	50.45452	19551.48	50.11631	19552.5	50.11279	19553.84	50.12336	19546.52	50.1152	19546.21
19552.41	50.36654	19550.42	50.70623	19554.2	50.36393	19551.92	50.36199	19553.23	50.36577	19549.43	50.37235	19546.98
19550.67	50.61316	19552.61	50.95632	19551.68	50.6155	19551.44	50.61094	19551.67	50.62018	19545.28	50.61699	19546.31
19551.32	50.86365	19553.04	51.20544	19549.61	50.8661	19550.58	50.86238	19552.55	50.8656	19546.5	50.87004	19549.29
19550.1	51.11932	19550.78	51.45429	19551.61	51.11584	19550.68	51.11331	19553.6	51.12264	19546.92	51.11521	19546.35

19550.86	51.3642	19550.16	51.70709	19552.04	51.36578	19551.98	51.36268	19555	51.36694	19546.66	51.37322	19548.46
19551.77	51.61505	19551.08	51.95657	19553.74	51.61515	19552	51.61207	19549.86	51.62174	19547.8	51.61618	19548.05
19549.68	51.86337	19551.09	52.20661	19553.19	51.86732	19552.45	51.86211	19551.85	51.86502	19546.49	51.87024	19547.42
19550.13	52.11989	19550.64	52.45571	19551.33	52.11664	19550.99	52.11263	19554.14	52.12176	19549.41	52.11667	19545.09
19550.17	52.3653	19551.59	52.70622	19552.18	52.36558	19551.76	52.36361	19551.83	52.36726	19545.68	52.37316	19548.41
19550.05	52.6154	19553.01	52.95605	19549.47	52.61774	19552.34	52.61335	19553.23	52.62076	19545.92	52.6168	19547.02
19550.3	52.86539	19552.73	53.20774	19552.84	52.86631	19552.85	52.86818	19551.96	52.86559	19549.6	52.87283	19546.02
19550.94	53.1201	19550.35	53.45691	19552.81	53.11705	19554.3	53.11269	19551.26	53.12359	19549.05	53.11759	19548.7
19550.73	53.36542	19550.41	53.7073	19552.33	53.36767	19551.59	53.36411	19553.48	53.36812	19547.75	53.37586	19548.14
19551.09	53.61585	19552.22	53.9555	19551.96	53.61746	19553.37	53.61462	19552.13	53.62345	19545.73	53.61634	19546.24
19550.41	53.86523	19553.35	54.20704	19551.66	53.86674	19552.87	53.86254	19551.84	53.86698	19548.11	53.86987	19547.72
19551.32	54.12278	19550.65	54.45652	19549.7	54.11701	19550.58	54.11229	19552.37	54.12072	19548.45	54.11793	19546.61
19550.22	54.36408	19550.55	54.70746	19552	54.36801	19551.17	54.36311	19554.26	54.36746	19549.22	54.37422	19547.68
19551	54.61535	19550.77	54.95608	19550.59	54.61884	19553.07	54.61438	19554.69	54.62314	19548.09	54.61767	19546.55
19549.39	54.86535	19549.89	55.20598	19553.44	54.86649	19552.72	54.86491	19556.11	54.86782	19548.46	54.87054	19546.61
19551.49	55.12271	19553.53	55.4582	19552.71	55.11611	19551.89	55.11325	19551.59	55.12297	19547.04	55.11703	19547.6
19549.68	55.36905	19550.02	55.71036	19552.21	55.3671	19550.99	55.36273	19554.21	55.36733	19548.12	55.37675	19547.97
19552.16	55.61428	19549.83	55.95936	19551	55.61867	19551.85	55.61351	19551.94	55.62461	19548.18	55.61953	19548.55
19551.55	55.86507	19551.53	56.20766	19552.69	55.86813	19551.37	55.86411	19551.89	55.86876	19546.95	55.87201	19547.47
19549.94	56.12109	19551.1	56.45715	19550.88	56.11875	19551.75	56.11438	19553.43	56.12475	19546.76	56.1165	19547.32
19550.2	56.36574	19551.16	56.70827	19552.74	56.3666	19551.41	56.3622	19553.99	56.36687	19549.36	56.37894	19545.97
19548.75	56.61619	19552.68	56.9603	19548.08	56.61816	19552.12	56.61339	19552.93	56.62308	19546.61	56.61837	19548.27
19549.71	56.8657	19553.02	57.21104	19553.13	56.86793	19549.97	56.86463	19553.87	56.86944	19546.54	56.87476	19546.82
19550.42	57.1224	19552.11	57.45733	19555	57.11888	19553.07	57.11538	19552.91	57.1247	19547.19	57.11867	19546.36
19549.9	57.36792	19551.95	57.70792	19548.21	57.36662	19552.58	57.36365	19552.27	57.36788	19546.16	57.37872	19548.43
19549.91	57.61617	19550.59	57.96069	19552.8	57.62009	19555.08	57.61268	19553.56	57.62356	19546.75	57.61848	19545.84
19551.41	57.86699	19551.38	58.20988	19552.62	57.86901	19553.15	57.86463	19553.35	57.86828	19544.45	57.87267	19546.33
19549.09	58.12236	19550.75	58.4568	19553.07	58.11901	19550.88	58.11588	19553.22	58.12514	19547	58.11876	19546.78
19550.54	58.36681	19554.82	58.70801	19550.98	58.36778	19552.53	58.36467	19556.63	58.36956	19547.29	58.37601	19547.29
19551.14	58.61722	19551.12	58.95935	19553.93	58.61906	19551.3	58.61319	19553.1	58.62489	19547.38	58.61782	19547.09
19551.26	58.86719	19553.85	59.21013	19551.95	58.86842	19551.46	58.86441	19555.54	58.86826	19546.75	58.87201	19547.74
19549.63	59.12089	19551.34	59.459	19552.07	59.11909	19552.79	59.1157	19554	59.12395	19547.47	59.11893	19545.5
19551.11	59.36512	19553.13	59.70761	19553.47	59.36948	19551.99	59.36547	19555.08	59.36956	19547.09	59.37741	19547.06
19552.39	59.61751	19551.72	59.9577	19553.23	59.6184	19554.1	59.61553	19552.75	59.62496	19544.81	59.61758	19548.21
19551.36	59.8668	19550.66	60.20935	19550.94	59.86703	19550.46	59.86503	19552.62	59.87022	19547.44	59.87279	19546.7
19551.27	60.14256	19551.75	60.4609	19551.83	60.11902	19551.55	60.1144	19553.56	60.12411	19545.71	60.11994	19547.4
19552.45	60.36659	19552.5	60.71206	19552.21	60.37052	19552.84	60.36805	19553.24	60.3719	19548.04	60.37989	19547.83
19552.03	60.61725	19552.87	60.95974	19552.17	60.61988	19549.6	60.61832	19552.4	60.6276	19547.91	60.62004	19546.66
19550.94	60.86976	19548.93	61.20971	19551.59	60.86929	19551.9	60.86566	19552.85	60.8701	19546.68	60.87962	19544.55
19550.07	61.1253	19551.4	61.46035	19551.8	61.12043	19552.76	61.11715	19553.75	61.12629	19549.85	61.12141	19547.06
19549.37	61.3691	19552.49	61.71372	19555.29	61.37005	19551.74	61.36865	19556.95	61.37055	19548.46	61.3813	19544.5
19549.62	61.61749	19553.72	61.96071	19550.08	61.62174	19551.76	61.6194	19552.46	61.62741	19546.79	61.6209	19549.74
19550.47	61.86813	19551.6	62.21158	19552.52	61.87147	19552.57	61.86678	19554.05	61.87188	19548.35	61.88678	19548.02
19547.88	62.12523	19553.17	62.46049	19552.79	62.12069	19552.93	62.11726	19553.11	62.12676	19546.66	62.12064	19547.68
19552.44	62.36954	19549.95	62.71191	19552.32	62.36939	19554.09	62.36643	19553.64	62.37124	19546.18	62.38271	19547.13
19552.32	62.61908	19551.33	62.96208	19550.75	62.62214	19551.43	62.62035	19553.29	62.62751	19544.47	62.62167	19547.38
19549.21	62.8763	19552.59	63.2122	19555.48	62.87169	19552.05	62.87042	19551.99	62.87282	19547.32	62.87585	19548.36
19551.49	63.12565	19552.87	63.45986	19549.73	63.12195	19552.39	63.11682	19554.64	63.12791	19546.16	63.12142	19548.42
19549.68	63.3704	19552.68	63.71308	19553.04	63.37052	19552.69	63.3682	19551.27	63.37098	19547.26	63.38318	19547.88
19549.53	63.61862	19551.32	63.96277	19552.48	63.62077	19550.18	63.61837	19552.18	63.62685	19547.37	63.62351	19546.38
19550.89	63.86952	19550.95	64.21314	19550.36	63.87385	19552.47	63.86903	19552.49	63.8726	19548.75	63.87755	19547.26
19548.57	64.12607	19550.98	64.46096	19551.21	64.12317	19553.87	64.119	19553.71	64.12951	19541.97	64.12135	19547.41
19550.71	64.37098	19550.77	64.71086	19552.08	64.37251	19551.41	64.36754	19555.22	64.37212	19547.85	64.38603	19549.28
19550.69	64.61914	19551.21	64.96143	19550.9	64.61991	19550.7	64.61811	19554.17	64.62536	19547.7	64.62286	19548.96
19550.15	64.868	19549.73	65.21181	19552.28	64.87039	19551.76	64.86838	19554.87	64.87053	19547.35	64.87699	19546.59
19550.34	65.12432	19551.63	65.46106	19555.02	65.12205	19552.86	65.11923	19553.7	65.12644	19548.52	65.12055	19546.05
19551.98	65.36986	19552	65.71049	19550.99	65.37174	19553.5	65.36659	19553.38	65.37193	19548.35	65.3811	19547.34
19549.76	65.61955	19552.25	65.96158	19551.01	65.62039	19551.09	65.61712	19552.66	65.62613	19545.96	65.62128	19546.74
19550.48	65.86909	19550.25	66.21381	19550.47	65.87072	19551.82	65.86714	19552.99	65.87021	19545.86	65.8764	19547.05
19550.4	66.1244	19551.45	66.45895	19549.57	66.12221	19552	66.12017	19554.65	66.12731	19546.57	66.1221	19545.41
19548.54	66.3684	19551.94	66.71379	19553.22	66.37182	19552.75	66.36943	19551.33	66.37188	19547.24	66.38214	19545.76
19549.17	66.62035	19548.42	66.9608	19553.58	66.62125	19553.24	66.61754	19553.34	66.62758	19548.18	66.62123	19546.92
19551.32	66.87024	19552.65	67.21288	19551.11	66.87152	19551.19	66.87158	19554.07	66.87195	19543.67	66.87733	19548.23
19550.43	67.12769	19551.31	67.46228	19551.89	67.12202	19552.08	67.1172	19550.37	67.12772	19546.96	67.12264	19546.53
19551.68	67.36896	19551.8	67.71135	19552.5	67.37164	19551.28	67.36854	19553.61	67.37142	19547.55	67.40108	19547.05
19551.94	67.61869	19551.33	67.96158	19551.92	67.62327	19550.96	67.61901	19552.86	67.6273	19546.3	67.61991	19545.08
19548.69	67.87301	19550.52	68.21169	19552	67.87132	19551.82	67.86777	19551.23	67.87379	19548.09	67.87763	19548.75
19550.84	68.12621	19552.38	68.46244	19552.02	68.12293	19551.99	68.11742	19554.95	68.12667	19549.62	68.12268	19545.4
19552.06	68.3698	19551.03	68.71383	19552.04	68.37202	19550.94	68.36842	19554.45	68.3711	19547.71	68.38573	19547.64
19552.66	68.618	19550.12	68.9723	19551.87	68.62258	19551.38	68.6191	19554.62	68.62515	19545.65	68.62297	19548.71

19551.56	68.8699	19552.73	69.21324	19551.66	68.87144	19551.68	68.86871	19552.25	68.87381	19545.34	68.8774	19546.96
19548.75	69.13481	19552.11	69.46098	19552.04	69.12187	19553.88	69.11756	19554.89	69.12754	19547.39	69.12141	19548.45
19549.37	69.3776	19551.72	69.71219	19551.62	69.37195	19551.9	69.36889	19554.26	69.37332	19545.32	69.38263	19549.4
19548.86	69.61968	19553.53	69.96444	19550.75	69.62273	19551.27	69.61948	19551.1	69.62813	19548.14	69.6232	19547.72
19551.41	69.86921	19553.11	70.21333	19552.4	69.87329	19552.79	69.86957	19552.88	69.87218	19546.46	69.87799	19548.07
19550.01	70.12608	19552.46	70.46167	19551.62	70.12185	19550.42	70.11772	19551.95	70.12947	19546.37	70.12169	19547.75
19552.55	70.3824	19553.32	70.7135	19550.57	70.37072	19550.71	70.36758	19552.87	70.3732	19545.53	70.38478	19548.33
19549.32	70.62122	19553.1	70.96378	19551.18	70.62163	19551.25	70.6188	19551.13	70.62878	19545.49	70.62382	19546.4
19548.95	70.8692	19552.67	71.21429	19551.3	70.87404	19549.7	70.8689	19554.3	70.87117	19546.89	70.87877	19548.92
19550.07	71.12731	19550.8	71.46114	19551.86	71.12376	19554.44	71.12037	19553.02	71.12952	19544.75	71.12221	19546.63
19550.6	71.37169	19550.32	71.71183	19552.01	71.3708	19552.06	71.37314	19554.11	71.37355	19547.7	71.38783	19547.03
19549.63	71.62102	19551.55	71.96442	19550.17	71.62241	19552.73	71.61842	19552.8	71.62879	19544.49	71.62487	19546.5
19549.87	71.87176	19549.58	72.21469	19553.03	71.8729	19552.79	71.87045	19552.88	71.87375	19545.34	71.88043	19547.02
19549.4	72.12601	19551.64	72.46253	19551.34	72.12432	19553.38	72.1199	19551.69	72.12889	19544.74	72.12304	19549.08
19551.39	72.37213	19550.38	72.71261	19552.88	72.37395	19551.6	72.3676	19554.48	72.37334	19545.37	72.38091	19547.29
19549.76	72.62232	19552.12	72.96467	19553.56	72.62441	19551.29	72.61913	19551.63	72.62987	19546.38	72.62418	19549.58
19549.12	72.87485	19551.05	73.21438	19552.02	72.87304	19552.81	72.87139	19551.18	72.8746	19543.41	72.88015	19550.09
19550	73.13044	19548.49	73.46425	19551.12	73.12517	19551.85	73.11984	19553.82	73.1299	19547.31	73.12454	19546.65
19551.18	73.37276	19551.39	73.7152	19550.44	73.37363	19552.18	73.36802	19552.46	73.38147	19548.62	73.38326	19546.82
19549.54	73.62075	19552.19	73.96343	19549.77	73.62206	19551.97	73.61877	19552.67	73.62979	19546.78	73.62424	19547.72
19550.33	73.87105	19552.07	74.21388	19554.29	73.87349	19555.96	73.86858	19552.96	73.87518	19547.68	73.87976	19545.9
19550.49	74.1281	19553.53	74.46413	19551.85	74.12422	19552.51	74.12054	19552.03	74.13211	19544.72	74.12509	19549.09
19548.72	74.37376	19549.71	74.71497	19551.42	74.37483	19555.67	74.3706	19555.03	74.37327	19547.69	74.38759	19546.47
19548.32	74.62102	19550.81	74.96513	19553.49	74.62453	19552.49	74.61981	19554.54	74.62956	19544.5	74.62453	19549.17
19551.01	74.88145	19550.36	75.21384	19552.57	74.87401	19552.6	74.86945	19554.57	74.87918	19547.3	74.87915	19546.7
19551.12	75.13345	19550.22	75.46644	19551.47	75.12378	19552.71	75.12049	19551.45	75.13103	19547.34	75.12462	19544.76
19550.78	75.37576	19552.65	75.7175	19552.44	75.3741	19550.06	75.37148	19553.74	75.37418	19546.41	75.38383	19548
19550.42	75.6232	19552.28	75.96563	19551.54	75.62437	19551.91	75.6234	19556.1	75.62744	19546.52	75.62345	19546.41
19549.69	75.87188	19551.46	76.21484	19551.27	75.8757	19552.05	75.86977	19552.19	75.87373	19545.37	75.88012	19547.4
19550.67	76.12862	19550.8	76.46429	19551.81	76.1259	19552.98	76.12066	19553.85	76.13098	19547.1	76.12452	19547.93
19549.24	76.3727	19550.97	76.71641	19551.94	76.37338	19551.11	76.37095	19551.31	76.37569	19544.94	76.38608	19548.49
19551.56	76.62397	19550.6	76.9678	19553.62	76.62508	19550.91	76.62129	19551.19	76.63027	19547.8	76.62552	19548.87
19549.15	76.87382	19552.68	77.21567	19551.42	76.87682	19550.43	76.87055	19554.29	76.8745	19546.34	76.87918	19548.13
19551.23	77.12788	19552.92	77.46259	19551.01	77.1259	19551.5	77.11946	19551.72	77.13126	19546.99	77.12535	19548.47
19549.76	77.37394	19552.11	77.71671	19551.6	77.37441	19550.17	77.37079	19552.89	77.37598	19544.84	77.3875	19543.63
19551.03	77.62406	19550.75	77.97693	19551.17	77.62692	19551.5	77.63335	19552.6	77.62948	19548.42	77.6264	19545.75
19548.76	77.87582	19549.89	78.21707	19553.66	77.87629	19549.85	77.87351	19553.16	77.87541	19545.59	77.88091	19546.29
19549.97	78.12796	19551.59	78.46332	19551.19	78.12673	19554.28	78.12049	19551.3	78.13151	19547.62	78.12447	19547.65
19551.73	78.37248	19552.41	78.71309	19553.03	78.37584	19552.72	78.3701	19554.11	78.37582	19545.88	78.38382	19548.07
19550.23	78.62445	19552.13	78.96515	19552.31	78.62457	19551.42	78.62111	19554.74	78.6311	19547.39	78.62742	19547.67
19552.67	78.87522	19551.06	79.2166	19552	78.88085	19551.58	78.87185	19555.14	78.87829	19545.89	78.88118	19546.7
19550.23	79.13067	19551.87	79.47218	19552.5	79.12634	19553.54	79.12273	19552.76	79.13314	19546.85	79.12515	19547.03
19552.96	79.37264	19549.56	79.71498	19554.46	79.37547	19551.8	79.37005	19554.32	79.37476	19548.31	79.38505	19546.36
19552.04	79.62363	19551.04	79.96549	19550.78	79.62401	19553.05	79.62124	19552.91	79.63029	19546.53	79.62604	19544.77
19549.08	79.87392	19552.18	80.21722	19552.05	79.87553	19552.93	79.87168	19552.29	79.8792	19545.9	79.88259	19547.44
19549.83	80.13067	19550.91	80.46726	19550.86	80.12647	19549.28	80.12293	19551.8	80.1349	19545.7	80.12723	19546.01
19552.61	80.3758	19553.32	80.71474	19552.11	80.3787	19554.59	80.37465	19552.78	80.38033	19546.48	80.38757	19546.58
19549.82	80.62363	19551.23	80.9667	19553.52	80.6264	19553.71	80.62272	19552.89	80.63092	19548.29	80.62589	19549.46
19546.99	80.87335	19551.89	81.21657	19552.42	80.87572	19552.81	80.87385	19552.76	80.8774	19547.81	80.88223	19546.91
19549.7	81.13024	19552.99	81.46675	19551.81	81.12556	19552.39	81.12285	19553.75	81.13413	19545.71	81.12671	19549.08
19549.71	81.37495	19549.84	81.71688	19551.59	81.37591	19550.4	81.37138	19552.13	81.37856	19544.93	81.38645	19547.29
19547.78	81.62368	19552.25	81.97601	19552.05	81.62665	19551.16	81.6231	19553.29	81.6324	19544.79	81.62525	19548.4
19550.63	81.8723	19550.48	82.21753	19549.54	81.87827	19552.27	81.8735	19552.27	81.87682	19547.22	81.88206	19547.12
19549.28	82.13192	19549.9	82.46498	19551.25	82.12682	19550.68	82.12141	19551.99	82.13346	19547.48	82.12666	19548.32
19550.42	82.37576	19552.79	82.71838	19553.74	82.37532	19550.06	82.37146	19554.04	82.37896	19545.77	82.3869	19547.81
19549.67	82.62563	19552.38	82.96774	19551.23	82.63024	19549.16	82.62368	19554.78	82.63243	19546.43	82.62849	19549.84
19550.57	82.87606	19550.84	83.22618	19552.65	82.8817	19552.34	82.87522	19553.38	82.87829	19547.23	82.88232	19547.23
19551.7	83.14564	19549.73	83.46632	19551.89	83.13239	19550.35	83.12227	19556.06	83.13141	19546.37	83.1261	19548.92
19550.09	83.37357	19551.76	83.71831	19550.97	83.37687	19551.28	83.37167	19549.62	83.37765	19546.86	83.38765	19546.1
19549.11	83.62476	19553.81	83.96808	19550.06	83.62542	19551.61	83.62293	19555.46	83.63286	19546.69	83.62928	19544.87
19550.74	83.87786	19547.81	84.21876	19551.33	83.87734	19552.91	83.87581	19553.36	83.87749	19546.77	83.88366	19548.21
19550.79	84.13058	19551.43	84.46608	19550.23	84.12878	19552.62	84.12402	19553.92	84.13426	19546.55	84.12618	19547.3
19549.26	84.37447	19551.61	84.71697	19549.63	84.37856	19551.68	84.37139	19552.06	84.37728	19546.26	84.38804	19546.41
19549.11	84.62588	19552.19	84.96792	19548.57	84.62686	19548.53	84.62326	19551.61	84.63441	19544.34	84.6297	19547.13
19549.45	84.87571	19551.06	85.22015	19552.41	84.87707	19551.85	84.88654	19552.55	84.87879	19545.46	84.88425	19547.4
19548.13	85.13216	19552.73	85.4686	19549.87	85.1294	19553.01	85.12976	19556.16	85.13674	19545.18	85.12607	19548.86
19550.31	85.37439	19552.07	85.71887	19550.94	85.37812	19550.87	85.37394	19551.84	85.37627	19545.52	85.38725	19547.43
19549.25	85.62443	19552	85.96718	19551.61	85.62723	19550.45	85.62207	19552.24	85.63084	19546.63	85.62962	19544.24
19549.32	85.8752	19550.03	86.21966	19548.66	85.87685	19550.75	85.87444	19553.69	85.87936	19546.22	85.88381	19546.97
19547.22	86.13282	19548.94	86.46894	19550.02	86.12781	19551.26	86.12501	19552.06	86.13547	19543.64	86.12966	19545.93

19548.77	86.37577	19549.68	86.71805	19552.67	86.39152	19552.96	86.37508	19552.22	86.37864	19546.55	86.38749	19547.43
19552.26	86.62605	19551.98	86.96865	19551.5	86.62839	19552.71	86.62417	19554.06	86.63361	19548.52	86.62978	19546.67
19550.44	86.87514	19551.73	87.21768	19550.71	86.87901	19551.25	86.87546	19552.14	86.87785	19547.68	86.88416	19545.71
19549.27	87.13329	19549.02	87.46914	19551.11	87.12939	19550.34	87.12439	19552.79	87.13612	19543.93	87.12919	19545.55
19548.63	87.37748	19549.93	87.71778	19551.16	87.37802	19551.66	87.37666	19553.09	87.37944	19543.53	87.38931	19546.87
19548.96	87.62819	19552.01	87.9679	19551.85	87.63253	19551.08	87.62516	19551.3	87.6322	19545.96	87.62936	19545.92
19548.09	87.87935	19552.04	88.21882	19549.28	87.87964	19549.12	87.88635	19554.34	87.87738	19545.32	87.88437	19544.64
19548.29	88.13597	19550.12	88.46981	19551.11	88.12938	19550.73	88.12268	19551.95	88.13522	19545.46	88.12949	19548.03
19549.15	88.37644	19547.89	88.71963	19549	88.37719	19549.3	88.37425	19550.49	88.37947	19542.02	88.39102	19546.56
19549.98	88.62873	19549.77	88.97017	19550.56	88.62933	19549.52	88.62621	19551.31	88.6347	19546.58	88.63019	19545.09
19548.19	88.87792	19549.08	89.21842	19550.86	88.8806	19550.22	88.87776	19551.6	88.87906	19545.16	88.88276	19548.65
19551.42	89.13256	19548.82	89.46889	19551.61	89.12979	19551.12	89.12488	19552.91	89.13409	19544.39	89.13417	19545.43
19549.5	89.37716	19550.66	89.72043	19550.87	89.37791	19552.83	89.37479	19552.9	89.38029	19542.75	89.3893	19544.66
19548.45	89.62848	19549.67	89.96972	19549.9	89.62817	19547.9	89.62507	19551.59	89.63616	19543.92	89.6309	19544.39
19548.44	89.87793	19551.33	90.21791	19550.54	89.87966	19553.1	89.87662	19549.34	89.88094	19545.93	89.8841	19545.9
19548.34	90.1336	19551.05	90.46779	19549.92	90.13149	19550.97	90.12715	19550.87	90.13816	19545.85	90.12879	19544.57
19547.92	90.3766	19549.7	90.71943	19551.36	90.37793	19552.6	90.37497	19553.38	90.37854	19544.71	90.3889	19545.29
19549.21	90.62713	19550.86	90.97024	19549.64	90.62865	19550.09	90.62537	19552.07	90.63484	19545.26	90.63065	19545.14
19547.51	90.87698	19552.34	91.22032	19550.81	90.88007	19551.57	90.87754	19550.75	90.88049	19545.35	90.88625	19545.99
19548.01	91.13456	19550.73	91.46788	19551.4	91.13181	19551.71	91.12703	19552.49	91.13683	19545.93	91.12912	19544.34
19546.28	91.37796	19550.36	91.71956	19549.67	91.37855	19551.04	91.37664	19551.57	91.38064	19544.04	91.3916	19545.77
19549.39	91.62914	19549.09	91.97126	19551.2	91.62961	19551.91	91.62841	19550.24	91.635	19544.99	91.63212	19541.85
19548.13	91.8758	19548.89	92.22243	19549.99	91.88074	19550.21	91.87546	19552.61	91.88105	19545.48	91.88599	19543.02
19546.32	92.13465	19549.87	92.46816	19550.16	92.13213	19552.79	92.1245	19553.06	92.13547	19548.2	92.12998	19545.17
19547.66	92.37952	19551.13	92.71976	19549.1	92.38059	19550.28	92.37652	19554.8	92.38166	19544.93	92.38969	19543.93
19548.51	92.63043	19548.96	92.97094	19550.62	92.63241	19550.52	92.62664	19550.99	92.63547	19543.12	92.63007	19546.03
19548.55	92.88113	19550.87	93.22288	19548.13	92.87921	19551.33	92.87697	19553.01	92.87935	19542.11	92.88579	19543.67
19550.26	93.13336	19551.4	93.47025	19550	93.13306	19550.98	93.12604	19553.63	93.1371	19544.5	93.13132	19546.71
19549.12	93.3779	19548.18	93.7201	19550.73	93.38053	19552.03	93.37575	19550.34	93.38198	19545.37	93.39043	19544.31
19549.12	93.63072	19550.42	93.96999	19549.46	93.63101	19550.14	93.62695	19552.48	93.63749	19543.71	93.62922	19544.41
19548.92	93.87951	19552.6	94.223	19550.03	93.87886	19552.42	93.87811	19553.37	93.87993	19544.82	93.88602	19545.95
19547.49	94.13627	19549	94.47063	19550.11	94.13127	19549.65	94.12806	19553.1	94.13762	19546.2	94.13119	19545.68
19548.16	94.37823	19550.12	94.721	19548.34	94.38183	19549.99	94.37546	19551.94	94.38323	19543.48	94.39166	19545.36
19547.84	94.62998	19549	94.97037	19548.21	94.63123	19549.45	94.62803	19550.2	94.63689	19545.51	94.63119	19545.32
19551.83	94.87935	19548.48	95.22327	19549.85	94.88186	19548.2	94.87947	19552.38	94.88103	19540.36	94.8856	19544.77
19548.01	95.13575	19547.1	95.47171	19547.62	95.13338	19548.2	95.12778	19550.34	95.13941	19546.37	95.13059	19545.42
19546.26	95.38045	19550.66	95.72268	19550.52	95.38032	19551.28	95.37648	19550.88	95.38123	19545.14	95.39351	19542.93
19547.06	95.62797	19550.39	95.9722	19550.03	95.63305	19551.03	95.62677	19550.6	95.63737	19543.74	95.63185	19542.82
19548.71	95.87878	19547.15	96.22364	19549.72	95.88218	19550.1	95.87843	19551.15	95.88322	19542.84	95.88722	19544.14
19549.37	96.13578	19549.77	96.47146	19548.2	96.13416	19549.95	96.12793	19552.8	96.13621	19544.26	96.13014	19543.16
19548.72	96.37994	19548.21	96.72199	19548.64	96.37909	19549.87	96.37784	19551.89	96.38178	19542.97	96.39317	19546.18
19547.4	96.62948	19547.37	96.97233	19549.53	96.63115	19548.34	96.62744	19550.37	96.63702	19544.66	96.63266	19541.96
19547.12	96.87805	19547.04	97.22239	19548.56	96.88367	19550.44	96.8793	19553.28	96.8847	19541.66	96.88783	19544.27
19546.87	97.13791	19548.13	97.4706	19550.07	97.13559	19549.43	97.1284	19550.9	97.13925	19544.45	97.13238	19543.27
19547.68	97.38149	19547.26	97.72238	19549.89	97.38131	19548.71	97.37958	19549.93	97.38066	19542.36	97.39233	19545.14
19546.02	97.64228	19548.29	97.97384	19547.64	97.63465	19550.44	97.62934	19549.76	97.63664	19543.14	97.63289	19542.9
19550.25	97.88326	19548.85	98.22288	19549.13	97.88173	19549.18	97.87837	19551.02	97.88429	19542.86	97.88994	19543.04
19547.81	98.13546	19548.85	98.46994	19548.2	98.13665	19550.27	98.12772	19550.9	98.13986	19542.76	98.13338	19543.9
19549.96	98.38505	19548.01	98.72327	19548.12	98.38345	19547.69	98.38032	19550.49	98.3828	19542.18	98.39417	19544.87
19548.23	98.631	19548.7	98.97125	19547.26	98.63191	19548.43	98.63055	19550.23	98.63863	19543.11	98.63264	19544.94
19547.35	98.88087	19546.42	99.22746	19547.92	98.88062	19548.3	98.87944	19552	98.88324	19544.34	98.88895	19545.93
19546.75	99.14096	19547.99	99.47746	19549.12	99.13935	19548.66	99.13238	19550.55	99.14399	19544.69	99.1377	19544.76
19548.62	99.37877	19548.26	99.73304	19546.5	99.38819	19547.12	99.37868	19550.2	99.38329	19540.36	99.39385	19544.08
19546.41	99.63065	19544.86	99.97293	19549.93	99.63404	19550.97	99.62981	19551.05	99.63617	19544.45	99.63128	19544.09
19548.68	99.88186	19547.94	100.2237	19547.95	99.88411	19548	99.88001	19549.12	99.88358	19544.54	99.888	19544.52
19546.79	100.1375	19548.31	100.4749	19548.86	100.1358	19548.38	100.1276	19548.49	100.1405	19545.08	100.1345	19542.82
19546.41	100.3839	19547.22	100.7342	19548.49	100.3842	19546.77	100.3804	19549.1	100.3862	19542.19	100.3947	19541.59
19548.6	100.6308	19547.53	100.9765	19547.47	100.6358	19546.88	100.6318	19548.72	100.6407	19544.26	100.6369	19543.79
19548.82	100.8822	19547.24	101.2251	19546.78	100.8874	19547.78	100.8832	19551.09	100.8846	19540.53	100.8901	19543.76
19547.88	101.1406	19547.99	101.4742	19548.8	101.1385	19546.37	101.1327	19548.64	101.1436	19542.56	101.1352	19543.62
19545.27	101.3853	19548.22	101.7262	19549.07	101.3838	19548.64	101.3821	19548.29	101.3873	19543.93	101.4029	19542.35
19544.68	101.6323	19548.25	101.9775	19546.81	101.6346	19550.18	101.6315	19548.14	101.6444	19540.32	101.6478	19541.54
19545.7	101.8832	19546.76	102.2249	19547.26	101.8872	19549.52	101.884	19549.38	101.8864	19543.77	101.8917	19545.49
19546.99	102.1412	19547.59	102.4772	19548.47	102.1395	19549.27	102.1332	19548.27	102.143	19543.81	102.1359	19541.13
19544.61	102.3839	19547.43	102.7255	19546.81	102.3845	19548.54	102.382	19547.87	102.3866	19543.59	102.3959	19542.75
19545.57	102.6399	19547.76	102.9757	19545.01	102.6377	19550.76	102.6309	19549.96	102.6421	19540.72	102.6376	19540.67
19547.01	102.8893	19547.25	103.2259	19547.26	102.887	19547.91	102.882	19549.98	102.888	19541.17	102.8925	19544.85
19546.61	103.14	19547.43	103.4774	19546.36	103.141	19547.61	103.1331	19551.88	103.1451	19540.9	103.1351	19544.86
19546.49	103.3843	19546.3	103.728	19548.06	103.3905	19546.94	103.383	19550.62	103.385	19542.78	103.3955	19541.55
19546.53	103.6348	19545.42	103.9764	19546.35	103.6343	19548.54	103.6317	19550.13	103.641	19541.93	103.6364	19542.29

19546.75	103.8841	19548.08	104.2273	19548.56	103.8853	19547.91	103.8845	19548.22	103.8931	19541.62	103.8941	19543.52
19547.71	104.1394	19547.33	104.4764	19547.79	104.1393	19549.26	104.1322	19549.64	104.1423	19540.13	104.1368	19541.73
19547.67	104.3838	19548.65	104.7277	19547.63	104.3867	19551.22	104.3826	19549.69	104.3871	19540.84	104.397	19540.91
19545.57	104.6348	19548.25	104.9781	19547.69	104.6387	19547.39	104.6341	19547.85	104.6416	19542.74	104.6354	19542.34
19546.19	104.8848	19547.83	105.226	19548.56	104.8857	19548.68	104.8834	19549.2	104.8874	19542.88	104.893	19542.8
19544.76	105.1395	19547.67	105.4767	19547.45	105.151	19547.49	105.1378	19548.22	105.1436	19541.84	105.1381	19541.63
19545.74	105.3835	19547.33	105.7271	19544.42	105.3875	19547.71	105.384	19548.57	105.3876	19540.06	105.3975	19544.57
19546.74	105.6344	19546.11	105.9793	19546.75	105.6369	19547.03	105.6341	19549.23	105.641	19544.21	105.6375	19543.94
19547.1	105.886	19547.47	106.2294	19545.91	105.889	19547.77	105.8846	19548.47	105.8861	19540.59	105.894	19541.66
19546.63	106.1403	19543.89	106.4753	19547.89	106.1408	19545.37	106.1336	19546.77	106.1454	19540.18	106.1372	19542.18
19547.56	106.3851	19543.35	106.7296	19547.07	106.3856	19549.24	106.383	19549.7	106.3876	19540.59	106.3995	19542.82
19545.37	106.6351	19545.6	106.9802	19547.77	106.6361	19548.6	106.6343	19547.12	106.6436	19542.29	106.6383	19540.69
19544.01	106.8872	19548.23	107.2294	19546.13	106.8878	19547.17	106.8855	19550.02	106.8857	19542.44	106.8936	19541.11
19545.17	107.1415	19546.25	107.4753	19545.99	107.1408	19546.04	107.1351	19548.36	107.1447	19541.33	107.1377	19541.2
19544.84	107.3851	19548.02	107.7293	19546.41	107.3887	19546.27	107.3825	19549.57	107.3882	19543.32	107.398	19542.55
19544.92	107.6346	19547.58	107.9804	19546.79	107.6395	19548.84	107.6327	19547.82	107.6434	19540.8	107.6383	19542.41
19546.03	107.89	19546.7	108.2293	19545.74	107.8867	19548.79	107.8845	19551.21	107.887	19540.29	107.8943	19540.8
19546.9	108.1428	19547.7	108.4783	19546.56	108.1399	19547.58	108.136	19550.41	108.1442	19541.34	108.1377	19540.29
19544.47	108.3861	19546.68	108.7277	19546.72	108.3883	19543.37	108.3859	19550.41	108.3887	19544.07	108.3988	19542.89
19543.57	108.635	19545.67	108.9792	19549.66	108.6382	19546.06	108.6323	19548.29	108.6437	19540.35	108.6395	19542.08
19542.83	108.8851	19546.87	109.2288	19545.74	108.8887	19544.51	108.8834	19549.13	108.8902	19542.7	108.896	19541.34
19544.94	109.1424	19546.36	109.4793	19545.17	109.1406	19545.71	109.1352	19549.42	109.147	19539.73	109.1387	19540.79
19545.62	109.3874	19546.72	109.7285	19546.26	109.3882	19545.71	109.3848	19548.13	109.3889	19541.85	109.398	19540.8
19544.11	109.6365	19546.68	109.9827	19548.44	109.6392	19549.17	109.6346	19546.29	109.6444	19538.95	109.6398	19540.04
19541.75	109.8877	19547.53	110.228	19547.3	109.8888	19545.23	109.8873	19546.41	109.8898	19540.57	109.8956	19541.35
19546.35	110.1426	19542.59	110.4791	19546.39	110.1414	19547.19	110.1369	19547.3	110.1473	19540.85	110.1378	19539.71
19545.73	110.3857	19547.07	110.733	19547.74	110.3873	19544.66	110.3863	19546.23	110.3922	19542.99	110.3993	19539.56
19542.24	110.6376	19546.42	110.9869	19548.95	110.6387	19547.57	110.636	19547.11	110.6432	19540.13	110.6438	19542.54
19544.43	110.8896	19544.77	111.2273	19545.89	110.8904	19548.53	110.8889	19548.71	110.8877	19540.18	110.8966	19542.69
19543.94	111.1433	19545.95	111.4799	19547.53	111.1429	19545.76	111.1366	19548.7	111.1462	19539.36	111.1395	19541.02
19542.71	111.3862	19546.7	111.7313	19546.08	111.3874	19545.33	111.3863	19547.15	111.3903	19541.94	111.4012	19539.68
19540.8	111.6368	19542.42	111.9811	19546.3	111.6398	19544.82	111.636	19547.59	111.6453	19539.92	111.6465	19541.22
19546.02	111.8879	19544.78	112.23	19545.06	111.8905	19546.3	111.8872	19545.21	111.8881	19540.09	111.8963	19540.93
19545.25	112.1455	19545.26	112.479	19545.02	112.1431	19546.76	112.1367	19547.74	112.1465	19538.52	112.1407	19539.38
19546.67	112.3975	19545.68	112.7302	19545.89	112.3883	19544.91	112.3849	19546.03	112.3898	19538.45	112.3991	19540.02
19546.1	112.6359	19546.07	112.982	19545.55	112.6416	19545.49	112.6358	19545.6	112.6465	19541.75	112.6386	19541.44
19541.49	112.8944	19544.49	113.2301	19545.21	112.8924	19543.71	112.8878	19547.12	112.8909	19540.23	112.8966	19538
19543.61	113.1445	19544.35	113.4797	19545.68	113.1435	19542.33	113.1459	19544.73	113.1483	19538.61	113.1405	19540.06
19542.69	113.3882	19545.71	113.7316	19543.63	113.3936	19549.17	113.3853	19547.96	113.3892	19541.4	113.4005	19538.97
19542.51	113.6368	19545.88	113.9816	19545.19	113.6387	19547.47	113.6368	19548.49	113.6447	19541.76	113.6392	19540.46
19545.66	113.8896	19543.33	114.2321	19545.61	113.8911	19547.71	113.8895	19546.49	113.8921	19539.38	113.8963	19541.52
19545.75	114.1464	19546.23	114.4812	19547.48	114.1468	19547.17	114.1377	19549.47	114.1483	19543.28	114.1417	19539.21
19544.85	114.389	19546.9	114.7313	19547.17	114.3929	19546.17	114.3864	19549.55	114.3914	19541.85	114.4008	19540.96
19543.86	114.6385	19545.91	114.9838	19545.99	114.6386	19544.22	114.636	19547.6	114.646	19540.44	114.6402	19540.7
19541.68	114.891	19546.78	115.2323	19545.73	114.8895	19546.4	114.8879	19547.32	114.8916	19539.37	114.8952	19541.73
19544.37	115.1425	19545.75	115.4827	19544.58	115.1444	19546.14	115.1374	19547.66	115.1486	19539.52	115.1408	19540.72
19543.73	115.3893	19544.04	115.7309	19543.52	115.3914	19546.02	115.3976	19545.63	115.3935	19541.91	115.4028	19538.94
19544.54	115.6388	19546.06	115.983	19545.16	115.6415	19546.11	115.6409	19545.79	115.6459	19539.31	115.6413	19540.53
19544.32	115.8903	19546.5	116.2323	19544.96	115.8908	19545.15	115.8886	19546.43	115.8905	19538.25	115.8975	19541.01
19544.11	116.1455	19544.45	116.4836	19545.16	116.1463	19545.43	116.1365	19546.87	116.1479	19539.11	116.1407	19539.36
19544.72	116.3877	19545.9	116.7309	19545.34	116.3931	19543.64	116.3882	19550.28	116.3927	19539.29	116.4015	19541.83
19543.94	116.6384	19544.15	116.9844	19547.2	116.6428	19546.83	116.6373	19545.83	116.6465	19540.02	116.6427	19540.2
19543.64	116.8913	19542.63	117.2322	19546.19	116.893	19545.62	116.9012	19545.85	116.8923	19538.78	116.8977	19541.41
19542	117.1493	19543.96	117.4824	19543.53	117.1461	19545.77	117.1375	19546.58	117.1486	19540.03	117.1425	19539.45
19544.34	117.389	19545.79	117.7324	19544.46	117.3935	19544.81	117.3885	19547.89	117.3938	19539.15	117.4018	19540.33
19542.7	117.6385	19545.64	117.9853	19542.13	117.6441	19544.28	117.6384	19544.2	117.6469	19539.1	117.6438	19540.85
19541.72	117.8943	19542.35	118.2311	19545.84	117.8927	19544.17	117.8917	19548.44	117.8926	19537.83	117.8985	19537.84
19542.96	118.15	19543.04	118.4933	19543.27	118.1484	19544.7	118.1376	19548.49	118.1489	19538.48	118.1414	19541.11
19544.33	118.3905	19542.81	118.7335	19548.32	118.392	19545.53	118.3865	19546.29	118.3923	19539.13	118.4042	19540.28
19544.48	118.6393	19542.03	118.9886	19543.62	118.6411	19547.46	118.6367	19549.08	118.6477	19541.19	118.6427	19539.8
19545	118.8902	19543.24	119.236	19547.35	118.8917	19546.27	118.8925	19545.85	118.8939	19539.41	118.8988	19538.77
19543.17	119.1461	19543.21	119.4814	19545.72	119.1482	19543.89	119.1383	19546.02	119.1517	19539.46	119.1432	19542.59
19544.08	119.3906	19545.83	119.7324	19547.35	119.3945	19543.78	119.388	19547.5	119.3941	19539.91	119.4043	19540.01
19542.82	119.6417	19543.47	119.9853	19545.12	119.643	19545.04	119.6384	19548.35	119.6468	19539.8	119.6427	19540.51
19542.26	119.8895	19543.09	120.2346	19543.31	119.8962	19545.42	119.8903	19547.07	119.8944	19539.23	119.8991	19541.99
19541.75	120.1449	19544.26	120.4834	19546.01	120.1469	19545.1	120.1392	19546.62	120.1499	19538.96	120.144	19539.86
19542.43	120.3909	19544.29	120.7292	19545.94	120.3941	19545.23	120.3908	19546.89	120.3951	19537.94	120.4033	19538.6
19543.25	120.6387	19544.14	120.9825	19547.08	120.6422	19547.34	120.6371	19546.53	120.6457	19540.2	120.6402	19541
19540.17	120.8887	19543.01	121.231	19543.67	120.8913	19543.99	120.8901	19548.97	120.8898	19537.93	120.8946	19537.81
19541.65	121.1431	19545.41	121.4817	19545.32	121.1425	19547.04	121.1365	19546.08	121.1472	19539.34	121.1412	19540.43

19543.21	121.389	19542.02	121.7322	19546.21	121.391	19544.83	121.3866	19549.14	121.3919	19539.13	121.4045	19538.53
19544.65	121.639	19542.27	121.9826	19544.33	121.6429	19545.86	121.6389	19546.7	121.6476	19538.97	121.6422	19539.2
19541.91	121.8897	19544.49	122.2303	19543.96	121.8908	19546.83	121.8909	19548	121.89	19539.82	121.8959	19541.09
19542.65	122.1455	19545.35	122.4814	19546.68	122.1473	19543.82	122.1377	19545.16	122.1463	19539.61	122.1542	19537.87
19542.51	122.4002	19544.06	122.7331	19542.27	122.3942	19542.28	122.3883	19544.63	122.3927	19540.11	122.4216	19539.51
19541.36	122.6402	19544.31	122.9851	19541.36	122.6436	19545.34	122.6387	19546.68	122.6489	19535.44	122.6437	19542.5
19540.79	122.8943	19544.49	123.2316	19546.04	122.8906	19541.68	122.8905	19544.02	122.8924	19537.66	122.8983	19539.81
19544.4	123.1455	19544.66	123.4795	19544.71	123.1455	19542.6	123.1374	19545.78	123.15	19540.51	123.1414	19537.93
19543.2	123.3902	19543.03	123.7335	19541.78	123.3933	19543.34	123.3885	19544.48	123.393	19538.86	123.4023	19539.96
19541.68	123.6388	19542.28	123.9872	19546.5	123.6424	19543.24	123.6385	19547.18	123.6466	19540.49	123.6422	19539.07
19542.94	123.8906	19546.88	124.2338	19542.62	123.8908	19545.8	123.8934	19545.99	123.8926	19542.56	123.8985	19539.46
19544.68	124.1454	19545.72	124.4808	19546.24	124.1455	19542.64	124.1391	19542.72	124.1499	19539.13	124.1455	19539.77
19542.35	124.3902	19541.77	124.7335	19542.95	124.3931	19544.52	124.3878	19544.56	124.3935	19538.73	124.405	19535.72
19542.46	124.6397	19540.08	124.9845	19545.72	124.6432	19542.41	124.6384	19543.31	124.6488	19539.99	124.6417	19539.18
19542.9	124.8894	19543.88	125.2334	19544.14	124.8926	19548.05	124.8942	19545	124.8939	19540.16	124.8976	19537.65
19540.83	125.1448	19543.87	125.481	19544.78	125.1459	19542.25	125.1398	19543.26	125.154	19539.19	125.144	19539.44
19541.02	125.4029	19544.25	125.7316	19544.67	125.3952	19544.26	125.3886	19546.3	125.3944	19539.66	125.4063	19539.71
19541.18	125.6527	19544.39	125.9962	19544.97	125.6441	19543.24	125.6382	19546.86	125.6471	19538.9	125.6415	19538.28
19542.83	125.9031	19542.91	126.2336	19543.32	125.8928	19545.61	125.8928	19543.44	125.8948	19537.77	125.8981	19540.31
19542.51	126.1569	19543.17	126.4833	19544.74	126.1467	19545.51	126.1391	19546.65	126.1495	19538.52	126.1452	19538.56
19543.46	126.3904	19545.53	126.7372	19543.82	126.3921	19544.8	126.3901	19545.62	126.3934	19539.38	126.4059	19538.82
19541.38	126.6408	19544.59	126.9864	19542.25	126.6435	19544.46	126.6375	19545.17	126.6489	19540.09	126.6413	19537.75
19541.03	126.8925	19542.97	127.232	19543.12	126.8946	19543.83	126.893	19546.98	126.8946	19537.44	126.8987	19538.65
19543.16	127.1463	19542.76	127.4823	19545.4	127.1525	19542.9	127.1389	19545.04	127.1501	19537	127.1444	19538.53
19542.55	127.3892	19540.63	127.7342	19542.25	127.3918	19545.65	127.3911	19547.62	127.3943	19539.14	127.4054	19538.28
19544.26	127.641	19544.01	127.9879	19543.89	127.6457	19542.04	127.64	19546.37	127.6497	19538.41	127.6441	19537.23
19539.06	127.8956	19543.93	128.2335	19542.35	127.8951	19545.34	127.8945	19546.74	127.8944	19538.67	127.8979	19539.66
19543.9	128.1469	19544	128.4859	19547.78	128.1507	19543.81	128.1396	19546.6	128.1515	19538.45	128.146	19537.96
19540.41	128.3895	19542.07	128.7339	19542.43	128.3929	19544.32	128.3904	19546.22	128.395	19537.75	128.4054	19538.96
19542.54	128.6419	19541.72	128.9884	19544.37	128.6464	19544.3	128.6403	19545.02	128.6495	19538.12	128.6431	19537.37
19540.43	128.8915	19541.91	129.2362	19542.78	128.8963	19542.97	128.8936	19546.81	128.8927	19540	128.8991	19537.65
19542.06	129.147	19542.27	129.4826	19545.11	129.1509	19543.09	129.141	19544.25	129.1513	19540.11	129.1449	19537.62
19541.81	129.3917	19545.15	129.7349	19546.07	129.3944	19545.22	129.3914	19545.99	129.3944	19538.26	129.4048	19536.96
19540.22	129.6404	19542.51	129.9884	19546.15	129.6435	19546.49	129.6412	19545.03	129.6514	19536.66	129.6448	19538.83
19540.52	129.8929	19539.5	130.2358	19544.14	129.8925	19544.47	129.894	19544.79	129.8948	19537.9	129.9	19535.59
19539.92	130.1505	19544.15	130.486	19544.33	130.1493	19543.18	130.1403	19543.99	130.1531	19536.59	130.1455	19536.85
19541.65	130.3925	19543.24	130.7339	19541.48	130.3938	19544.79	130.3921	19544.75	130.395	19537.29	130.4052	19539.29
19541.31	130.6426	19541.79	130.9994	19544.16	130.6447	19544.69	130.6408	19543.91	130.6511	19538.07	130.6452	19539.82
19538.82	130.8908	19542.65	131.2363	19542.48	130.8958	19544.08	130.8947	19546.17	130.8974	19538.35	130.8999	19538.22
19540.49	131.1491	19545.45	131.4863	19542.65	131.1494	19545.95	131.1405	19544.13	131.1542	19535.55	131.1473	19538.36
19541.74	131.3923	19542.22	131.7401	19542.53	131.4114	19542.03	131.3903	19544.92	131.3952	19536.75	131.4066	19538.33
19542.27	131.6422	19539.61	131.9879	19542.88	131.6459	19543.79	131.6393	19545.77	131.6501	19539.25	131.6445	19539
19539.08	131.8935	19543.34	132.2349	19543.14	131.8963	19542.92	131.8943	19543.79	131.8955	19535.49	131.9007	19537.33
19543.05	132.1487	19542.02	132.4836	19543.26	132.1498	19543.86	132.1441	19541.78	132.1541	19536.63	132.1477	19538.5
19541.44	132.392	19541.09	132.7385	19543.63	132.3948	19544.98	132.3943	19544.71	132.3934	19537.92	132.4074	19538.16
19539.91	132.6422	19541.12	132.9906	19544.06	132.6501	19543.91	132.6401	19541.76	132.651	19537.83	132.65	19539.28
19541.36	132.896	19544.15	133.2386	19542.94	132.896	19540.99	132.8966	19545.76	132.8967	19539.58	132.9062	19537.88
19542.15	133.1486	19541.48	133.4852	19541.25	133.1522	19544.37	133.1406	19544.54	133.1535	19538.49	133.1478	19538.39
19538.54	133.3957	19545.64	133.7382	19542.99	133.3954	19540.69	133.3928	19545.51	133.3969	19536.13	133.4068	19537.79
19541.38	133.6423	19543.68	133.9905	19543.51	133.6457	19542.82	133.643	19542.04	133.6639	19537.7	133.6466	19536.47
19541.53	133.8931	19543.24	134.2379	19544.53	133.897	19544.06	133.8982	19542.76	133.8957	19536.72	133.9015	19538
19542.42	134.1509	19543.07	134.4838	19543.04	134.1519	19544	134.1412	19544.55	134.1527	19539.84	134.1482	19541.16
19542.23	134.3938	19543.48	134.7392	19543.5	134.3942	19541.88	134.3919	19545.43	134.3978	19539.57	134.4077	19539.19
19541.52	134.6415	19541.11	134.9921	19541.06	134.6459	19542.99	134.644	19543.68	134.6537	19538.12	134.6473	19536.04
19542.63	134.8939	19544.39	135.2381	19543.64	134.8955	19542.34	134.8957	19545.61	134.8964	19535.71	134.9022	19537.44
19539.49	135.1518	19541.11	135.486	19544.44	135.1521	19546.09	135.1406	19543.66	135.154	19537.23	135.1466	19539.53
19538.83	135.3967	19542.27	135.7413	19544.77	135.3973	19546.68	135.3965	19547.62	135.3984	19535	135.4067	19539.25
19540	135.6449	19540.51	135.9955	19544.18	135.6458	19544.27	135.6428	19545.66	135.6552	19537.66	135.6511	19536.32
19541.7	135.8939	19541.9	136.2377	19541.66	135.8966	19541.63	135.8964	19546.88	135.8964	19535.64	135.9019	19537.09
19539.49	136.1517	19540.62	136.4862	19543.55	136.1516	19542.84	136.1433	19543.47	136.1541	19536.64	136.1474	19538.58
19540.8	136.394	19542.38	136.7397	19544.05	136.3975	19542.7	136.3934	19544.05	136.3971	19536.12	136.4102	19536.93
19539.02	136.6445	19542.56	136.9919	19544.06	136.6481	19540.98	136.6436	19544.6	136.6525	19538.89	136.6499	19539.4
19540.35	136.8958	19541.48	137.2389	19543.45	136.8973	19541.87	136.8958	19545.49	136.8991	19537.46	136.903	19538.74
19541.63	137.1495	19541.49	137.4867	19542.66	137.1519	19542.7	137.1424	19545.22	137.155	19535.29	137.1544	19535.94
19541.63	137.3934	19541.89	137.7407	19543.47	137.3992	19543.89	137.3917	19546.31	137.3975	19537.18	137.4089	19538.36
19538.72	137.6444	19543.79	137.9918	19544.05	137.6512	19543.94	137.6426	19544.84	137.6542	19537.18	137.6461	19537.97
19540.74	137.898	19542.07	138.2581	19543.54	137.8968	19542.52	137.8977	19543.29	137.9003	19538.43	137.9025	19537.98
19543.47	138.1502	19543.03	138.4907	19542.1	138.1526	19543.59	138.1441	19547.8	138.1576	19536.56	138.1475	19537.37
19543.49	138.3944	19541.72	138.7397	19544.26	138.3985	19542.32	138.3926	19545.13	138.3966	19538.94	138.4069	19538.12
19540.33	138.6438	19541.06	138.9934	19542.61	138.6504	19543.31	138.6419	19545.58	138.6531	19535.12	138.6468	19535.52

19541.22	138.8953	19541.18	139.2392	19542.79	138.8969	19543.36	138.8991	19544.42	138.8991	19539.48	138.9025	19537.81
19538.7	139.1511	19544.24	139.4862	19539.95	139.1535	19541.01	139.1441	19545.48	139.1562	19538.43	139.146	19536.28
19543.32	139.3956	19541.39	139.742	19546.18	139.398	19544.24	139.3944	19544.86	139.3986	19536.04	139.4074	19537.81
19542.12	139.6456	19540.3	139.9954	19541.6	139.6516	19543.69	139.6441	19542.34	139.6531	19539.84	139.6489	19536.48
19541.06	139.8944	19542.28	140.2376	19542.61	139.8987	19542.66	139.8989	19545.05	139.9009	19537.05	139.9042	19537.15
19540.58	140.1531	19540.33	140.4863	19544.88	140.1528	19541.32	140.145	19546.81	140.157	19534.91	140.1468	19538.29
19540.78	140.3973	19540.43	140.7389	19542.46	140.4017	19542.73	140.3987	19542.34	140.4016	19537.52	140.4094	19538.46
19539.52	140.6456	19541.77	140.9924	19542.28	140.6505	19542.08	140.6445	19543.66	140.6579	19536.13	140.6493	19538.7
19540.61	140.8947	19540.78	141.2391	19544.38	140.8993	19541.14	140.9	19543.56	140.8992	19538.34	140.9037	19536.52
19542.26	141.1499	19540.58	141.4887	19543.1	141.1545	19541.34	141.1449	19544.3	141.1557	19536.98	141.1473	19537.14
19541.82	141.3972	19540.75	141.7389	19541.16	141.3974	19542.32	141.396	19543.65	141.3996	19536.54	141.4088	19539.8
19538.4	141.6515	19542.78	141.9931	19544.76	141.6506	19545.19	141.6447	19545.47	141.6558	19537.68	141.6483	19537.05
19540.11	141.9007	19541.08	142.2387	19542.74	141.8992	19540.93	141.8988	19545.5	141.9008	19537.04	141.9036	19535.61
19540.41	142.1551	19540.6	142.4896	19542.48	142.1561	19542.25	142.1443	19542.91	142.1574	19535.82	142.1569	19538.95
19538.55	142.3991	19543.75	142.7386	19542.07	142.3975	19542.04	142.3971	19542.92	142.3981	19536.58	142.4156	19537.25
19540.26	142.6498	19541.5	142.9935	19542.41	142.6541	19544.59	142.6465	19542.49	142.6551	19536.16	142.6552	19537.25
19538.83	142.9096	19540.55	143.2397	19543.49	142.9003	19544.32	142.9204	19546.45	142.9017	19538.94	142.9072	19537.56
19540.2	143.1489	19544.21	143.4899	19543.85	143.1559	19541.96	143.1442	19545.28	143.157	19536.87	143.1527	19537.5
19542.49	143.4066	19539.99	143.7404	19542.27	143.401	19543.23	143.3941	19541.33	143.3992	19536.08	143.4172	19536.29
19538.94	143.6457	19540.67	143.9957	19540.73	143.6514	19542.77	143.6445	19542.36	143.6548	19536.42	143.6537	19537.08
19542.07	143.8989	19541.83	144.2405	19543.14	143.8991	19540.12	143.8997	19545.2	143.9005	19537.72	143.912	19537.9
19541.23	144.1514	19542.86	144.49	19542.05	144.1551	19542.6	144.1484	19542.42	144.1583	19536.86	144.1505	19535.18
19541.88	144.3954	19541.58	144.7405	19542.27	144.4005	19538.82	144.3948	19544.26	144.4014	19536.08	144.4109	19537.25
19538.96	144.6468	19540.25	144.9958	19539.92	144.6505	19544.42	144.6445	19545.54	144.6556	19538.23	144.6506	19534.67
19541.55	144.8988	19541.79	145.2385	19542.33	144.8981	19545.19	144.9007	19544.5	144.9007	19537.85	144.9059	19538.82
19537.61	145.1521	19540.18	145.49	19543.54	145.1555	19540.73	145.1455	19544.92	145.1576	19537.07	145.1507	19536.52
19539.38	145.3974	19541.11	145.7417	19539.31	145.4005	19541.7	145.397	19543.33	145.4044	19537.88	145.4146	19537.47
19537.24	145.6455	19541.23	145.9951	19542.23	145.6512	19544.03	145.6477	19543.35	145.6592	19535.68	145.6508	19537.23
19541.1	145.8999	19540.66	146.2414	19542.24	145.9023	19541.52	145.9005	19546.11	145.9003	19536.4	145.9059	19536.22
19539.02	146.1519	19541.02	146.4875	19540.34	146.1555	19541.85	146.1469	19543.19	146.159	19535.62	146.1495	19536.4
19540.7	146.3983	19539.48	146.74	19543.63	146.3993	19539.79	146.3965	19543.83	146.4011	19538.71	146.411	19535.91
19538.96	146.6468	19540.44	146.9946	19543.1	146.6677	19543.41	146.6471	19544.91	146.6566	19533.5	146.6509	19537.61
19537.47	146.9007	19540.69	147.2416	19540.77	146.9014	19543	146.9005	19542.81	146.9027	19536.56	146.9077	19534.91
19540.13	147.1522	19542.48	147.4908	19541.06	147.1589	19542.52	147.1456	19543.07	147.1592	19537.25	147.1493	19535.85
19538.15	147.3997	19543.42	147.7418	19543.56	147.3993	19540.83	147.3973	19544.62	147.4002	19533.73	147.4141	19539.01
19540.19	147.6478	19539.58	147.9956	19539.85	147.6553	19541.36	147.6487	19543.65	147.6575	19534.47	147.6507	19535.47
19539.12	147.9036	19540.72	148.2427	19541.79	147.9016	19543.84	147.901	19544.47	147.9025	19537.15	147.9058	19537.14
19539.47	148.1538	19541.72	148.4911	19543.06	148.1576	19541.43	148.1466	19544.15	148.1599	19540.36	148.1509	19538.37
19539.33	148.4	19542.81	148.7449	19540.83	148.402	19543	148.3964	19543.91	148.4024	19537.3	148.4137	19534.52
19540.03	148.6497	19543.39	148.9942	19542.13	148.6515	19540.17	148.6481	19543.58	148.6577	19535.35	148.6532	19536.23
19539.53	148.8994	19540.24	149.2408	19541.05	148.9019	19540.82	148.9032	19541.29	148.9024	19536.15	148.9066	19537.33
19541.88	149.1528	19541.33	149.4895	19540.61	149.1584	19542.72	149.1475	19541.46	149.1682	19534.88	149.152	19536.65
19539.17	149.4	19542.76	149.7434	19543.4	149.401	19542.59	149.398	19542.4	149.4006	19534.77	149.4151	19538.28
19539.07	149.6493	19540.46	149.9964	19541.37	149.6541	19543.5	149.647	19543.06	149.6577	19539.18	149.6502	19535.41
19537.17	149.9012	19539.45	150.2413	19539.12	149.902	19542.68	149.9041	19544.68	149.9036	19537.64	149.907	19538.04
19538.59	150.153	19540.33	150.4935	19542.09	150.1567	19544.09	150.1482	19546.96	150.161	19534.58	150.1517	19539.39
19538.44	150.4028	19541.65	150.7465	19539.33	150.4049	19542.92	150.4	19542.33	150.4043	19537.42	150.4173	19537.32
19540.37	150.6517	19540.45	151.0008	19540.86	150.6543	19543.96	150.6538	19543.59	150.6594	19538.56	150.6559	19534.86
19538.86	150.9038	19541.97	151.2448	19539.97	150.9038	19540.63	150.907	19544.39	150.906	19536.63	150.912	19537.61
19537.42	151.1566	19542.23	151.4935	19542.33	151.1596	19539.96	151.1517	19543.73	151.1638	19535.25	151.155	19537.6
19537.51	151.4028	19538.32	151.7483	19544.18	151.4046	19541.98	151.4007	19545.23	151.4083	19534.49	151.4179	19537.01
19539.57	151.6521	19543.58	152.0029	19541.09	151.6568	19540.55	151.6547	19543.58	151.6605	19534.28	151.6567	19537.45
19540.67	151.904	19540.31	152.2468	19543.45	151.9055	19540.02	151.9068	19541.14	151.9071	19533.04	151.9102	19537.73
19538.25	152.159	19540.31	152.4941	19541.02	152.161	19542.11	152.1508	19543.24	152.163	19537.7	152.154	19537.44
19539.12	152.4043	19540.55	152.7483	19542.48	152.4046	19541.15	152.4034	19542.26	152.4083	19538.95	152.4187	19537
19540	152.6519	19538.9	153.0004	19543.19	152.659	19542.03	152.6528	19542.52	152.6612	19535.5	152.6559	19536.77
19538.56	152.9087	19539.7	153.2471	19542.82	152.9076	19540.72	152.906	19545.21	152.9065	19536.89	152.9113	19538.05
19538.94	153.1619	19539.62	153.4947	19543.64	153.16	19540.7	153.1493	19542.83	153.1646	19537.21	153.1571	19537.03
19539.31	153.4076	19541.39	153.7467	19542.82	153.4061	19541.32	153.4006	19543.52	153.4064	19535.65	153.416	19537.84
19540.07	153.6581	19541.6	154.0004	19544.05	153.6575	19540.76	153.6574	19540.6	153.6618	19536.27	153.6573	19537.58
19540.12	153.9168	19539.52	154.2468	19541.26	153.9064	19543.54	153.9094	19542.67	153.906	19537.51	153.9113	19538.3
19539.67	154.1645	19539.34	154.4965	19542.16	154.1619	19542.68	154.1508	19545.03	154.1659	19539.47	154.1576	19538.46
19539.34	154.4113	19539.92	154.7475	19540.51	154.406	19541.02	154.402	19542.76	154.4075	19535.42	154.4172	19537.97
19538.42	154.6641	19541.16	155.0016	19542.8	154.6598	19541.47	154.6552	19544.95	154.6626	19535.78	154.6575	19536.97
19538.62	154.9124	19539.89	155.246	19543.09	154.9075	19543.04	154.9091	19541.87	154.9074	19538	154.9115	19539.52
19539.22	155.1634	19539.13	155.4968	19542.19	155.1636	19540.93	155.1516	19543.58	155.165	19534.49	155.1577	19538.05
19539.93	155.4107	19539.73	155.747	19542.3	155.4048	19541.23	155.4042	19543.97	155.4091	19534.88	155.4205	19540.08
19539.38	155.6597	19540.81	156.0042	19541.27	155.6566	19539.55	155.654	19543.19	155.6632	19534.85	155.6696	19536.17
19540.07	155.9105	19540.53	156.2464	19541.58	155.908	19539.82	155.9093	19542.91	155.908	19534.59	155.9122	19535.55
19537.84	156.1656	19540.65	156.4965	19539.72	156.1635	19539.22	156.1537	19541.67	156.1665	19536.27	156.1571	19536.53

19539.18	156.4155	19541.32	156.7476	19538.35	156.4055	19539.36	156.4007	19545.38	156.4066	19535.05	156.4203	19535.89
19539.12	156.6608	19541.65	157.0072	19541.37	156.6558	19538.47	156.6558	19542.24	156.6618	19535.35	156.6595	19535.98
19538.77	156.9165	19539.86	157.2474	19541.79	156.9079	19538.43	156.9098	19543.05	156.9099	19536.18	156.9137	19536.55
19537.21	157.167	19540.23	157.4985	19541.43	157.1626	19538.11	157.1533	19540.86	157.1688	19536.49	157.1558	19534.94
19539.86	157.4118	19538.72	157.7471	19541.94	157.4083	19539.42	157.4033	19543.25	157.4063	19536.05	157.4201	19537.83
19538.72	157.662	19537.3	158.0028	19541.48	157.6621	19540.5	157.6508	19540.04	157.6622	19536.28	157.6572	19534.93
19538.46	157.9159	19537.75	158.2493	19538.53	157.9077	19539.14	157.9103	19541.68	157.908	19535.03	157.9135	19538.65
19539.67	158.162	19537.88	158.4973	19541.69	158.1629	19544.45	158.1563	19543.31	158.1661	19533.08	158.1583	19535.88
19539.7	158.4113	19538.74	158.7468	19540.96	158.4073	19541.07	158.405	19541.37	158.4086	19534.18	158.4205	19536.83
19536.86	158.6546	19538.82	159.006	19542.11	158.6604	19541.22	158.654	19542.28	158.6665	19534.72	158.6577	19534.25
19539.96	158.9078	19542.26	159.2495	19542.75	158.9094	19538.43	158.9099	19542.68	158.9093	19534.11	158.9134	19536.91
19538.6	159.1595	19539.55	159.4968	19540.21	159.1647	19541.89	159.1547	19541.88	159.1671	19536.93	159.1594	19536.49
19538.74	159.4172	19540.38	159.7471	19539.55	159.4087	19541.65	159.4048	19541.75	159.4094	19535.65	159.4186	19535.59
19537.97	159.6571	19539.16	160.0054	19542.59	159.6601	19541.69	159.6543	19542.65	159.6635	19535.81	159.6601	19536.68
19537.63	159.9049	19540.01	160.2495	19540.55	159.9115	19539.24	159.9112	19544.71	159.9106	19534.03	159.9132	19538.42
19538.34	160.1592	19537.42	160.4968	19542.39	160.1647	19539.6	160.1532	19543.63	160.1672	19535.04	160.1598	19534.1
19540.28	160.4142	19540.01	160.7452	19538.9	160.4079	19541.94	160.4076	19542.66	160.4088	19533.75	160.4204	19537.7
19538.45	160.6588	19539.45	161.0028	19543.98	160.6574	19540.93	160.6522	19544.06	160.6605	19535.4	160.6566	19537.78
19536.3	160.9034	19537.19	161.2453	19538.98	160.9096	19543.02	160.9078	19543.57	160.9103	19534.34	160.9118	19536.62
19538.13	161.1563	19539.92	161.4956	19540.97	161.1621	19540.34	161.1535	19545.57	161.1653	19537.06	161.1563	19540.93
19538.36	161.4062	19538.48	161.747	19542.21	161.4047	19540.17	161.4017	19542.06	161.4065	19535.91	161.4189	19537.99
19539.59	161.6562	19542.91	162.0051	19541.88	161.6567	19540.3	161.6519	19539.24	161.6647	19535.48	161.6562	19535.22
19536.98	161.9048	19541.08	162.2461	19540.85	161.9075	19538.4	161.9101	19543.92	161.9093	19536.3	161.9142	19540.84
19540	162.1573	19539.85	162.4958	19541.38	162.1617	19540.09	162.1534	19541.61	162.166	19535.91	162.1564	19535.96
19538.81	162.4052	19539.12	162.7511	19540.28	162.4062	19540.92	162.4024	19544.78	162.4076	19536.45	162.4166	19535.95
19539.23	162.6546	19537.14	163.0058	19538.71	162.6597	19542.38	162.6512	19540.35	162.6606	19535.75	162.6573	19536.83
19537.57	162.9085	19540.48	163.2486	19541.43	162.908	19539.09	162.9078	19541.06	162.9093	19537.09	162.9119	19537.77
19538.2	163.1587	19540.11	163.4956	19542.2	163.1621	19542.94	163.1545	19544.16	163.1676	19537.22	163.1554	19537.38
19538.97	163.4034	19536.49	163.7466	19541.58	163.4069	19541.35	163.4039	19541.96	163.4078	19533.98	163.417	19535.93
19538.71	163.6539	19540.26	164.0037	19541.07	163.6564	19540.99	163.6523	19542.42	163.6667	19533.86	163.6569	19534.9
19537.48	163.9067	19539.35	164.2475	19540.72	163.9075	19541.13	163.9092	19542.34	163.9086	19534.68	163.9129	19535.63
19538.01	164.1573	19538.96	164.4983	19540.39	164.1614	19542.62	164.1546	19544.09	164.1671	19535.17	164.1569	19536.01
19536.64	164.405	19538.83	164.7473	19541.03	164.4083	19540.57	164.4048	19542.45	164.4091	19535.51	164.4179	19534.82
19539.07	164.6528	19536.9	165.0023	19541.25	164.6596	19541.14	164.6541	19541.52	164.6641	19534.25	164.6577	19539.12
19538.75	164.9082	19541.12	165.2484	19542.11	164.908	19541.11	164.9107	19541.69	164.9097	19534.99	164.9123	19535.61
19533.97	165.1573	19542.91	165.4964	19540.65	165.1657	19541.29	165.1553	19542.89	165.1655	19534.45	165.1588	19535.66
19536.58	165.406	19539.38	165.7474	19540.64	165.4105	19541.88	165.4074	19542.52	165.4112	19535.37	165.4197	19531.74
19538.36	165.6535	19537.82	166.0034	19539.87	165.6589	19537.44	165.6538	19542.73	165.6633	19535.38	165.6586	19535.19
19536.75	165.9059	19538.01	166.2485	19544.25	165.9107	19540.69	165.91	19541.07	165.9087	19534.19	165.9136	19535.53
19538.47	166.1583	19539.77	166.4971	19539.34	166.1644	19540.53	166.1519	19542.65	166.1671	19534.17	166.1589	19535.15
19536.83	166.4057	19538.99	166.7489	19542.72	166.4075	19541.38	166.4048	19539.17	166.41	19534.56	166.4197	19537.49
19538.57	166.6548	19539.72	167.0097	19538.88	166.6616	19539.16	166.6547	19540.05	166.665	19536.01	166.6605	19536.41
19536.37	166.9095	19539.79	167.2465	19539.44	166.909	19541.57	166.9134	19538.86	166.9108	19535.94	166.9137	19540.47
19540.24	167.1573	19539.98	167.5002	19541.99	167.1667	19538.85	167.155	19542.27	167.17	19533.48	167.1594	19536.83
19537.64	167.4059	19541.04	167.7518	19539.88	167.4081	19539.38	167.4046	19541.54	167.4076	19532.86	167.4226	19533.04
19538.3	167.6574	19538.97	168.0029	19542.24	167.6627	19540.1	167.6554	19543.68	167.6628	19537.32	167.6602	19535.56
19540.58	167.9098	19541.29	168.2537	19542.12	167.9105	19541.78	167.9128	19543.75	167.9109	19535.41	167.9143	19536.92
19538.95	168.1556	19540.16	168.499	19538.25	168.1672	19540.08	168.1563	19542.32	168.1705	19536.5	168.1582	19534.35
19537.74	168.4091	19539.66	168.7489	19542.18	168.4093	19539.78	168.4044	19542.09	168.4099	19536.54	168.4192	19538.35
19537.61	168.6569	19540.77	169.0057	19540.52	168.6585	19539.89	168.6551	19545.18	168.6629	19534.76	168.6608	19536.77
19538.51	168.9091	19537.64	169.2512	19541.73	168.9095	19540.33	168.9171	19542.57	168.9123	19535.32	168.9155	19534.81
19539.99	169.159	19537.41	169.5	19539.69	169.1736	19538.66	169.1556	19544.04	169.1673	19535.38	169.1584	19534.46
19538.4	169.4065	19538.26	169.7478	19540.76	169.4171	19539.05	169.404	19542.6	169.4093	19535.56	169.4194	19537.14
19538.44	169.6568	19538.93	170.0073	19540.16	169.6598	19540.85	169.6545	19541.99	169.6646	19535.45	169.6617	19535.14
19536.76	169.9082	19540.09	170.2518	19539.94	169.908	19539.05	169.9111	19542.18	169.9115	19534.15	169.9145	19535.18
19535.76	170.1605	19538.92	170.5002	19542.35	170.1667	19538.94	170.1572	19542.65	170.1664	19534.79	170.1576	19535.23
19535.52	170.4061	19539.35	170.7493	19541.4	170.4101	19540.45	170.4089	19542.14	170.4096	19534.4	170.4187	19536.33
19535.53	170.6559	19540.5	171.0039	19543.41	170.6615	19539.32	170.655	19542.39	170.6658	19535.06	170.6612	19534.4
19539.92	170.9071	19537.16	171.2577	19540.7	170.9088	19539.94	170.9119	19538.83	170.9113	19533.01	170.9156	19536.67
19535.27	171.1609	19539.84	171.5017	19540.96	171.1648	19541.17	171.1578	19543.85	171.1648	19534.82	171.1615	19534.31
19537.36	171.4075	19537.09	171.7521	19540.2	171.4097	19541.48	171.4066	19544.96	171.4099	19533.34	171.4192	19535.48
19538.84	171.6586	19539.29	172.0055	19539.99	171.6629	19541.04	171.6552	19540.94	171.6653	19531.66	171.6639	19537.08
19539.1	171.9064	19539.29	172.2486	19540.02	171.9111	19540.73	171.9112	19541.83	171.9135	19533.25	171.915	19535.06
19537.36	172.1571	19536.8	172.4996	19538.1	172.1689	19541.73	172.1569	19543.29	172.1691	19534.38	172.1614	19536.27
19538.07	172.4079	19537.57	172.7512	19542.19	172.4213	19540.07	172.409	19542.5	172.4119	19533.76	172.4198	19536.19
19537.81	172.6597	19541.32	173.0064	19541.04	172.6671	19538.24	172.6581	19542.43	172.6663	19534.27	172.6606	19535.56
19539.38	172.9132	19539.95	173.2539	19539.19	172.9117	19539.37	172.9188	19542.83	172.9118	19534.12	172.915	19534.51
19538.04	173.1567	19537.27	173.5028	19539.22	173.1671	19540.19	173.1614	19543.96	173.1703	19534.4	173.1613	19534.66
19537.85	173.4076	19540.46	173.7508	19539.52	173.4143	19537.89	173.4193	19542.48	173.4098	19535.43	173.4214	19537.02
19536.89	173.6595	19540.52	174.0048	19542.6	173.6632	19539.93	173.6589	19541.82	173.6655	19534.17	173.663	19537.12

19537.83	173.9096	19539.25	174.2534	19542.87	173.9122	19541.63	173.9148	19542.53	173.9133	19534.65	173.9153	19536.88
19535.46	174.1583	19538.58	174.5	19540.81	174.1687	19538.23	174.1577	19540.93	174.1715	19533.96	174.1624	19533.07
19534.93	174.4077	19539.29	174.7526	19536.21	174.414	19538.7	174.4075	19541.33	174.4109	19534.39	174.4206	19536.03
19538.22	174.6585	19538.46	175.0077	19540.29	174.664	19541.95	174.6581	19542.65	174.6661	19535.35	174.6621	19534.58
19537.41	174.9113	19539.14	175.2527	19540.97	174.9104	19538.4	174.9196	19540.15	174.9122	19536.21	174.918	19537.08
19538.49	175.1591	19539.04	175.5016	19541.31	175.1684	19540.96	175.1566	19540.19	175.1726	19534.61	175.1593	19535.62
19539.37	175.4097	19540.21	175.7603	19541.11	175.4128	19540.77	175.4103	19541.33	175.4122	19533.38	175.4225	19535.63
19535.08	175.659	19539.43	176.0073	19540.43	175.6624	19540.87	175.658	19539.39	175.6661	19534.28	175.6637	19535.31
19540.04	175.9119	19537.89	176.2528	19540.85	175.9104	19541.79	175.9148	19543.02	175.9121	19536.24	175.9165	19534.86
19538.18	176.1592	19539.33	176.5017	19540	176.1684	19539.34	176.1582	19541.73	176.1708	19536.02	176.1619	19534.93
19536.45	176.4097	19537.19	176.7541	19541.09	176.4125	19542.48	176.407	19540.53	176.4127	19534.91	176.4216	19536.6
19537.4	176.6602	19537.91	177.0074	19540.06	176.6645	19540.1	176.6584	19542.44	176.6671	19536.17	176.6624	19535.07
19537.84	176.9111	19537.78	177.2529	19541.47	176.9123	19539.37	176.915	19537.96	176.9129	19533.69	176.9174	19535.25
19540.27	177.1595	19538.65	177.5006	19540.07	177.1704	19541.84	177.1634	19541.23	177.1712	19531.72	177.1627	19535.34
19538.27	177.4104	19538.17	177.7542	19540.34	177.4129	19539.43	177.4069	19541.53	177.414	19534.84	177.4466	19535.3
19537.92	177.6611	19539.58	178.0076	19540.16	177.6668	19538.42	177.6582	19540.52	177.6698	19535.48	177.661	19535.41
19536	177.9145	19540.56	178.2538	19538.94	177.9124	19537.84	177.9149	19540.46	177.9135	19535.15	177.9172	19536.98
19536.36	178.1601	19537.19	178.503	19540.42	178.1698	19540.17	178.1586	19542.14	178.172	19534.49	178.1662	19536.07
19536.6	178.4107	19538.52	178.7539	19541.76	178.412	19539.53	178.4084	19540.65	178.4141	19533.27	178.4245	19535.26
19535.36	178.6639	19538.97	179.0113	19540.83	178.6646	19539.73	178.6585	19542.55	178.6695	19534.4	178.6647	19537.83
19537.68	178.9103	19537.47	179.2539	19538.49	178.9143	19537.77	178.9155	19542.84	178.9145	19534.12	178.9187	19535.32
19538.45	179.1594	19534.9	179.5011	19538.68	179.1717	19538.67	179.1601	19541.91	179.1785	19533.93	179.1625	19534.1
19537.86	179.4132	19537.33	179.7549	19540.03	179.4116	19539.74	179.409	19540.11	179.4182	19535.71	179.4238	19535.68
19536.46	179.6625	19536.9	180.0142	19541.98	179.6643	19537.95	179.6611	19541.99	179.6681	19532.86	179.6655	19534.16
19538.83	179.9118	19537.64	180.2543	19539.57	179.9159	19538.83	179.9154	19542.35	179.9172	19533.88	179.9196	19536.99
19536.71	180.1599	19538.26	180.5021	19536.97	180.1747	19539.76	180.1594	19540.09	180.1755	19534.33	180.1635	19535.24
19536.6	180.4106	19538.69	180.7548	19543.32	180.4139	19540.4	180.4171	19542.29	180.4137	19533.03	180.4229	19533.92
19536.43	180.6634	19536.55	181.0081	19539.2	180.6656	19540.32	180.6608	19541.01	180.6679	19533.15	180.6633	19536.92
19539.15	180.9151	19538.48	181.2574	19541.73	180.914	19542.09	180.9163	19540.88	180.9144	19534.23	180.9179	19536.18
19538.67	181.1624	19539.7	181.5035	19539.76	181.1714	19538.65	181.1602	19540.98	181.1757	19532.99	181.1644	19533.69
19538.03	181.4111	19538.86	181.7564	19541.83	181.4172	19538.72	181.4116	19540.64	181.4141	19533.09	181.4265	19535.16
19536.33	181.664	19535.92	182.0176	19539.8	181.6648	19539.76	181.6619	19540.12	181.6693	19533.82	181.6641	19536.89
19536.83	181.9124	19538.66	182.2559	19539.7	181.9143	19540.31	181.9169	19540.61	181.9152	19533.09	181.9192	19533.65
19535.86	182.1618	19535.84	182.5022	19540.01	182.1729	19539.23	182.1609	19541.1	182.1743	19534.39	182.1649	19537.7
19537.18	182.4112	19538.37	182.7571	19538.88	182.4154	19537.94	182.4108	19541.31	182.4157	19532.25	182.4405	19535.28
19537.97	182.6649	19536.64	183.0098	19538.59	182.6692	19539.58	182.6638	19541.93	182.6711	19534.61	182.6642	19536.2
19537.03	182.9173	19539.52	183.2592	19540.93	182.9142	19539.04	182.9224	19540.92	182.9163	19535.04	182.9196	19536.09
19536.85	183.1616	19536.48	183.5046	19540.13	183.1705	19541.53	183.1588	19543.26	183.1752	19533.27	183.1653	19534.91
19538.69	183.4124	19539.88	183.7575	19542.53	183.4145	19541.14	183.4096	19540.18	183.4157	19535.13	183.4235	19537.84
19538.81	183.6634	19539.11	184.0101	19539.74	183.6704	19540.2	183.6621	19540.84	183.6719	19534.91	183.6635	19534.95
19536.13	183.915	19539.83	184.2579	19541.22	183.9165	19539.48	183.922	19540.33	183.9166	19535.47	183.92	19535.9
19536.94	184.1628	19539.19	184.504	19542.14	184.1721	19540.81	184.1666	19541.29	184.1752	19533.04	184.1659	19536.03
19538.43	184.4122	19535.23	184.7565	19536.86	184.4161	19540.06	184.4101	19541.85	184.4164	19534.02	184.4272	19533.24
19536.86	184.6641	19539.06	185.0101	19539.5	184.6683	19540.84	184.6619	19540.83	184.6719	19534.23	184.6659	19537.18
19534.99	184.9143	19536.22	185.2634	19537.89	184.9171	19539.53	184.9176	19543.82	184.9158	19534.85	184.9206	19536.56
19537.02	185.164	19538.44	185.5033	19539.34	185.1887	19538.7	185.164	19543.81	185.1748	19536.18	185.1649	19535.78
19537.86	185.4117	19536.5	185.7563	19541.58	185.4159	19538.31	185.4132	19540	185.4174	19532.97	185.4251	19535.92
19534.61	185.6644	19536.78	186.012	19538.4	185.6681	19539.1	185.6624	19541.29	185.6701	19533.97	185.6667	19536.14
19535.72	185.9167	19537.59	186.2589	19541.77	185.9165	19541.92	185.9179	19544.56	185.9174	19533.39	185.9226	19534.01
19535.77	186.1666	19540.05	186.5118	19540.73	186.1749	19540.54	186.163	19540.64	186.1765	19533.35	186.1676	19535.23
19537.59	186.4136	19536.94	186.7591	19538.08	186.4151	19541.98	186.4126	19539.65	186.4175	19536.54	186.4237	19534.75
19537.57	186.6635	19537.8	187.0106	19538.88	186.6676	19540.25	186.6626	19542.75	186.6726	19534.97	186.6661	19534.76
19537.26	186.9177	19536.32	187.2573	19542.52	186.9189	19537.96	186.9204	19543.71	186.921	19534.34	186.9233	19533.81
19536.62	187.1645	19538.13	187.5061	19539.65	187.1781	19539.97	187.1638	19538.05	187.1782	19532.82	187.1697	19534.61
19538.19	187.4149	19537.76	187.7564	19540.7	187.4168	19540.48	187.4126	19540.26	187.4166	19535.12	187.429	19535.89
19537.48	187.6642	19538.7	188.0123	19537.2	187.6711	19539.72	187.6653	19540.75	187.6732	19531.02	187.667	19533.73
19538.19	187.9217	19536.78	188.2585	19539.05	187.917	19538.33	187.9172	19539.3	187.919	19532.77	187.9221	19533.66
19539.77	188.1646	19535.84	188.5078	19536.92	188.1745	19538.34	188.1638	19541.62	188.1773	19531.25	188.1681	19535.76
19539.15	188.4162	19537.87	188.7577	19538.96	188.4169	19540.96	188.413	19540.17	188.4187	19535.84	188.4267	19534.49
19536.94	188.665	19537.93	189.012	19540.65	188.6689	19538.21	188.664	19543.94	188.6713	19534.62	188.6663	19534.39
19538.14	188.917	19536.84	189.2581	19537.92	188.9169	19537.69	188.92	19538.75	188.9185	19536.11	188.9242	19532.19
19536.99	189.1643	19536.88	189.5058	19537.91	189.1769	19539.52	189.164	19540.02	189.1773	19534.96	189.1685	19535.03
19539.07	189.418	19537.2	189.7588	19539	189.4178	19538.31	189.4139	19542.88	189.4192	19533.94	189.4272	19533.26
19539.29	189.664	19535.25	190.0152	19538.07	189.6674	19538.26	189.669	19540.19	189.6731	19531.96	189.6679	19535.31
19537.45	189.9175	19540.36	190.2578	19538.82	189.9177	19539.33	189.9241	19536.88	189.9191	19534.45	189.9242	19537.03
19535.58	190.1644	19536.45	190.5075	19540.13	190.1778	19540.72	190.1646	19543.04	190.1768	19534.37	190.1681	19534.49
19535.79	190.4157	19536.84	190.7611	19538.47	190.4179	19536.83	190.4168	19537.41	190.4175	19532.84	190.4307	19534.06
19538.5	190.666	19538.39	191.0148	19537.62	190.6686	19539.71	190.6656	19540.89	190.6725	19534.75	190.6677	19530.73
19537.78	190.9179	19536.85	191.2596	19541.21	190.9185	19539.26	190.9197	19542.15	190.9206	19533.96	190.9254	19535.27
19535.05	191.1658	19537.53	191.5059	19541.75	191.1755	19540.57	191.165	19542.93	191.178	19532.42	191.1675	19534.96

19536.36	191.416	19539.14	191.7594	19537.73	191.4179	19538.23	191.4127	19540.12	191.419	19531.83	191.4257	19535.09
19534.06	191.6658	19537.27	192.0153	19542.73	191.6699	19540.59	191.6652	19538.23	191.6725	19532.72	191.6708	19539.23
19537.56	191.918	19536.44	192.2575	19537.59	191.9213	19540.71	191.9206	19541.93	191.9214	19534.85	191.9242	19532.7
19536.64	192.167	19539.44	192.5071	19540.7	192.1767	19537.36	192.1672	19540.09	192.1816	19533.93	192.1712	19534.94
19535.94	192.4154	19536.94	192.7595	19537.57	192.4166	19537.7	192.4142	19540.92	192.4182	19535.37	192.4288	19531.89
19536.16	192.6653	19538.14	193.0134	19540.53	192.6725	19536.73	192.6659	19539.25	192.6738	19534.15	192.6754	19535.34
19534.18	192.9201	19537.24	193.2589	19540.87	192.9189	19540.54	192.9205	19541.28	192.9194	19533.66	192.9238	19535.8
19535.63	193.1661	19541.31	193.5082	19537.91	193.1799	19537.98	193.1669	19540.71	193.1789	19532.65	193.1695	19533.19
19535.47	193.4187	19535.91	193.7606	19539.95	193.4302	19539.19	193.4139	19539.68	193.4213	19529.37	193.4263	19534.52
19535	193.6668	19538.46	194.0157	19541.6	193.6739	19538.83	193.6666	19541.35	193.6787	19533.9	193.6673	19533.08
19535.83	193.9177	19538.05	194.2607	19538.52	193.9179	19539.37	193.9197	19539.88	193.9326	19532.47	193.9288	19533.26
19535.17	194.1666	19536.67	194.5079	19539.83	194.175	19536.98	194.1678	19541.73	194.1773	19531.82	194.173	19534.35
19538.5	194.4181	19536.54	194.7618	19542.49	194.4199	19539.57	194.4173	19543.55	194.4195	19531.71	194.4302	19534.64
19536.48	194.6672	19538.08	195.0149	19539.56	194.6705	19536.43	194.6672	19540.71	194.6738	19536.08	194.6687	19534.28
19536.72	194.9187	19538.02	195.2642	19538.24	194.9185	19539.44	194.9208	19540.19	194.9206	19535.34	194.9252	19534.85
19535.22	195.1669	19538.9	195.5091	19539.45	195.1778	19539.93	195.1685	19543.29	195.1786	19533.15	195.1703	19535.25
19537.55	195.4191	19538.56	195.7585	19540.12	195.4195	19538.42	195.4188	19543.49	195.4199	19535.14	195.4307	19532.84
19535.67	195.6677	19539.1	196.0144	19540.62	195.6752	19537.98	195.6679	19536.92	195.6757	19534.03	195.6687	19535.23
19538.88	195.9204	19535.67	196.2614	19537.73	195.9198	19538.73	195.9226	19540.74	195.9211	19532.54	195.9259	19535.83
19538.4	196.1668	19536.76	196.5097	19539.05	196.1766	19537.74	196.1698	19540.7	196.1773	19535.19	196.1796	19536.01
19535.45	196.4168	19537.85	196.7619	19537.4	196.4216	19537.96	196.415	19537.57	196.421	19536.22	196.4292	19538.37
19538.01	196.668	19537.7	197.0169	19537.61	196.6715	19536.88	196.6677	19540.01	196.6755	19531.9	196.6715	19533.53
19535.28	196.9218	19536.99	197.2611	19538.72	196.9207	19537.3	196.9244	19539.27	196.9217	19533.2	196.9276	19535.15
19536.14	197.1706	19537.97	197.5102	19540.64	197.1791	19538.2	197.1677	19538.24	197.1785	19532.51	197.1704	19534.51
19536.51	197.4183	19535.75	197.7629	19539.6	197.4205	19540.26	197.4177	19539.68	197.4233	19534.36	197.428	19536.64
19535.59	197.6677	19537.52	198.0152	19538.46	197.6746	19539.77	197.6664	19542.37	197.6757	19535.33	197.6709	19534.65
19537.07	197.9247	19537.11	198.2614	19538.77	197.9216	19537.9	197.928	19540.25	197.9227	19530.96	197.9274	19536.28
19537.27	198.1791	19538.33	198.54	19539.95	198.1872	19539.8	198.1775	19540.14	198.189	19536.46	198.1809	19531.83
19536.05	198.4172	19537.71	198.7657	19538.46	198.4204	19540.2	198.4166	19540.38	198.4212	19533.57	198.4257	19534.23
19536.6	198.6689	19539.91	199.018	19540.95	198.6729	19537.8	198.6666	19540.02	198.6778	19534.44	198.6712	19532.09
19536.36	198.9246	19536.95	199.2618	19537.14	198.9205	19538.26	198.9295	19540.46	198.9252	19532.02	198.9276	19535.88
19536.65	199.1688	19539.17	199.5164	19537.89	199.1786	19537.86	199.1686	19539.31	199.184	19534	199.1732	19533.69
19537.11	199.4194	19536.04	199.7625	19538.48	199.4224	19540.03	199.4173	19541.59	199.4215	19534.88	199.4278	19534.5
19535.63	199.6708	19536.67	200.0173	19539.06	199.6734	19540.09	199.672	19540.03	199.6765	19534.19	199.6709	19533.13
19537.48	199.9225	19536.39	200.2644	19541.5	199.9202	19538.63	199.9229	19540.26	199.9255	19531.58	199.9275	19534.37
19537.58	200.1705	19537.43	200.5133	19540.24	200.1792	19538.91	200.1686	19538.41	200.1808	19530.98	200.1728	19532.38
19536.76	200.4244	19537.1	200.765	19537.63	200.4274	19539.14	200.4248	19542.19	200.4342	19533.88	200.4347	19534.65
19536.38	200.6736	19537.02	201.0232	19538.35	200.6786	19539.57	200.6738	19540.31	200.6812	19534.08	200.678	19532.64
19535.76	200.9281	19538.74	201.2651	19539.06	200.9261	19539.52	200.9294	19537	200.9264	19532.65	200.9308	19536.79
19535.73	201.173	19537.44	201.515	19538.47	201.1819	19538.34	201.1725	19538.9	201.1837	19534.53	201.1772	19536.98
19536.83	201.4373	19537.73	201.7662	19539.99	201.4265	19541.27	201.4239	19540.69	201.4272	19533.79	201.4327	19534.76
19533.81	201.6736	19536.62	202.0213	19539.38	201.6771	19538.1	201.6719	19538.94	201.6823	19532.06	201.6763	19531.67
19538.29	201.9274	19537.19	202.2674	19539.28	201.929	19539.26	201.9304	19538.87	201.9272	19534.45	201.9338	19534.92
19536.51	202.173	19539.65	202.5148	19539.18	202.1843	19538.17	202.1716	19540.23	202.1855	19531.82	202.1776	19531.21
19534.84	202.424	19536.86	202.7657	19539.58	202.4286	19539.32	202.4257	19538.22	202.4299	19532.57	202.4354	19537.92
19536.31	202.6735	19537.16	203.022	19541.45	202.6794	19540.43	202.6766	19538.81	202.6822	19530.37	202.6753	19532.64
19534.18	202.9312	19538.97	203.2714	19538.71	202.9273	19540.77	202.9291	19536.28	202.9301	19533.19	202.9322	19531.01
19537.44	203.1734	19538.01	203.5167	19538.49	203.1809	19542.54	203.1732	19541.28	203.1857	19530.92	203.1827	19533.01
19535.52	203.4235	19537.36	203.7689	19541.66	203.428	19539.59	203.4226	19539.38	203.4293	19535.93	203.4365	19534.55
19533.6	203.6869	19536.67	204.0229	19537.81	203.6773	19540.67	203.6727	19538.8	203.6824	19535.1	203.6754	19536.3
19532.86	203.93	19536.4	204.2679	19539.12	203.9274	19539.46	203.9354	19539.76	203.9284	19533.54	203.9335	19532.77
19536.57	204.1746	19536.15	204.5178	19539.36	204.1881	19537.85	204.181	19541.92	204.1877	19534.67	204.1782	19531.63
19535.78	204.4278	19537.48	204.7689	19539.38	204.4313	19537.46	204.4233	19540.38	204.4273	19532.92	204.4353	19533.19
19537.25	204.6739	19536.22	205.0228	19538.12	204.6782	19539.27	204.6743	19540.16	204.6824	19534.51	204.6783	19534.17
19535.16	204.9277	19535.39	205.269	19538.01	204.9357	19536.58	204.9346	19539.48	204.9301	19533.89	204.9316	19534.31
19537.63	205.1741	19536.61	205.5179	19537.83	205.1852	19539.31	205.1735	19538.69	205.1868	19534.94	205.1777	19535.18
19535.23	205.4256	19537.16	205.7668	19540.68	205.4296	19541.46	205.4264	19540.46	205.4298	19532.52	205.4373	19533.74
19535.41	205.6749	19537.5	206.0248	19538.96	205.687	19540.02	205.6744	19540.04	205.6871	19534.76	205.6804	19535.13
19535.76	205.9272	19534.96	206.2683	19538.24	205.9364	19539.1	205.9309	19539.72	205.9288	19531.53	205.932	19534.12
19540.47	206.1732	19536.8	206.5169	19540.62	206.1866	19538.39	206.1734	19540.37	206.1996	19530.97	206.1789	19534.6
19536.09	206.4304	19538.48	206.767	19540.11	206.4278	19538.57	206.4249	19539.02	206.4313	19535.44	206.4359	19533.04
19536.48	206.6767	19536.2	207.0224	19536.56	206.6807	19537.45	206.6735	19539.65	206.6829	19535.74	206.6781	19535.76
19534.87	206.9296	19538.22	207.2681	19539.78	206.9465	19539.87	206.9311	19540.01	206.9311	19533.63	206.9335	19533.65
19536.16	207.1732	19537.03	207.5182	19540.16	207.1853	19538.86	207.1732	19537	207.1866	19534.17	207.1791	19532.52
19536.12	207.4265	19537.07	207.7678	19540.37	207.4313	19537.12	207.4228	19540.31	207.4298	19533.29	207.4354	19533.98
19536.83	207.6757	19538.14	208.0236	19538.22	207.6832	19535.94	207.6737	19541.83	207.6841	19535.74	207.6796	19535.01
19536.42	207.9329	19537.73	208.2688	19536.19	207.9308	19539.33	207.9319	19541.17	207.9337	19532.89	207.9342	19533.48
19535.02	208.1782	19540.7	208.5185	19537.56	208.185	19540.01	208.1757	19538.61	208.1846	19534.54	208.1798	19534.63
19535.9	208.4263	19537.48	208.7691	19539.59	208.4325	19539.22	208.4248	19540	208.4312	19535.46	208.4347	19535.45
19536.41	208.6856	19536.91	209.0235	19537.09	208.6799	19539.36	208.6768	19542.94	208.6825	19532.29	208.6803	19531.87

19536.73	208.9326	19536.04	209.2708	19539.58	208.9284	19537	208.9304	19538.84	208.9311	19531.9	208.9347	19532.95
19536.92	209.1757	19535.51	209.5177	19538.66	209.186	19539.77	209.1768	19541.59	209.1869	19533.91	209.1817	19533.49
19535.96	209.4284	19540.77	209.7718	19540.14	209.4309	19539.24	209.4265	19539.69	209.4296	19531.55	209.4387	19535.1
19535.96	209.6751	19538.16	210.024	19538.65	209.6805	19539.96	209.6752	19539.14	209.6843	19532.15	209.6807	19535.54
19536.36	209.9288	19534.56	210.2703	19541.35	209.9284	19539.76	209.9312	19537.91	209.9322	19534.85	209.9339	19533.16
19535.58	210.1762	19537.68	210.5183	19537.92	210.1852	19540.19	210.1771	19536.65	210.1887	19535.47	210.1833	19534.72
19533.18	210.4433	19536.69	210.7728	19540.45	210.432	19539.29	210.4295	19539.13	210.431	19532.27	210.4403	19536.27
19536.98	210.6774	19537.39	211.0243	19537.84	210.6807	19538.55	210.6781	19541.17	210.685	19531.72	210.6806	19533.4
19535.86	210.9291	19536.39	211.2718	19537.9	210.93	19540.91	210.9353	19540.36	210.931	19532.02	210.9342	19534.99
19538.95	211.1782	19535.66	211.518	19540.05	211.1872	19539.72	211.1782	19540.44	211.1884	19532.47	211.1814	19532.2
19535.89	211.4284	19534.93	211.7745	19536.96	211.4319	19540.64	211.4276	19540.7	211.4343	19532.94	211.4398	19532.96
19534.65	211.6771	19537.12	212.0248	19538.62	211.6828	19538.08	211.6759	19539.94	211.6853	19531.6	211.6812	19532.8
19535.05	211.9309	19540.75	212.2696	19540.06	211.9305	19539.04	211.9326	19541.92	211.9313	19534.46	211.9355	19537.21
19536.53	212.1776	19534.07	212.5197	19539.57	212.1878	19536.41	212.1764	19540.28	212.1873	19533.82	212.1827	19532.88
19533.42	212.4296	19534.62	212.7724	19539.75	212.4331	19538.78	212.4297	19541.49	212.4322	19532.25	212.4402	19531.77
19536.37	212.6776	19536.84	213.0249	19538.27	212.6863	19536.36	212.6791	19537.52	212.6873	19533.49	212.6828	19532.11
19536.79	212.9321	19534.82	213.2721	19537.66	212.9307	19536.61	212.9328	19538.95	212.9308	19532.75	212.9374	19537.56
19534.24	213.1796	19535.68	213.5219	19539.93	213.1871	19538.48	213.1768	19538.4	213.1891	19530.21	213.1828	19532.58
19535.37	213.4293	19537.28	213.7712	19538.99	213.433	19538.48	213.4273	19539	213.4331	19533.76	213.438	19535.73
19535.13	213.679	19536.16	214.0275	19537.52	213.6841	19536.9	213.6769	19538.86	213.6855	19533.25	213.6807	19534.83
19534.94	213.9325	19538.66	214.271	19536.88	213.93	19538.25	213.9343	19542.34	213.9355	19532.64	213.9369	19532.1
19538.18	214.1803	19536.63	214.5203	19539.39	214.1989	19537.96	214.1789	19536.99	214.1886	19534.68	214.1841	19532.71
19536.37	214.4301	19535.62	214.7728	19536.72	214.4334	19536.44	214.4289	19540.79	214.4321	19534.59	214.4367	19532.93
19534.56	214.6783	19536.89	215.0249	19537.44	214.6833	19535.72	214.6788	19539.98	214.6864	19534.75	214.6838	19533.07
19535.88	214.9333	19535.18	215.2733	19539.88	214.9312	19538.75	214.9356	19538.43	214.9328	19532.12	214.9371	19533.82
19536.57	215.1906	19538.24	215.5195	19538.91	215.1904	19537.33	215.1787	19539.06	215.1905	19532.9	215.1856	19534.66
19533.88	215.4313	19538.38	215.7711	19539.59	215.4334	19536.98	215.4328	19538.4	215.4321	19533.52	215.4415	19532.81
19535.76	215.6792	19536.2	216.0281	19540.69	215.6843	19537.82	215.6765	19539.79	215.6879	19530.81	215.6844	19536.26
19536.17	215.9313	19537.07	216.2721	19540.77	215.9328	19537.55	215.9332	19539.3	215.933	19534.74	215.9389	19534.92
19536.46	216.1799	19537.37	216.5198	19537.16	216.1881	19537.9	216.1796	19538.63	216.19	19535.91	216.1855	19534.41
19537.78	216.4307	19538.03	216.7743	19538.93	216.4317	19538.2	216.4306	19539.26	216.434	19535.34	216.4404	19533.79
19534.73	216.6792	19537.14	217.0328	19537.51	216.6851	19539.36	216.6778	19541.21	216.6867	19533.52	216.6813	19533.45
19538.2	216.9375	19535.76	217.285	19538.12	216.933	19537.92	216.9356	19537.2	216.9347	19533.38	216.9373	19534.45
19535.11	217.1808	19537.78	217.5201	19537.84	217.1882	19540.14	217.1794	19539.05	217.1908	19532.75	217.1849	19533.43
19535.07	217.4346	19536.85	217.7742	19538.84	217.4323	19537.06	217.4291	19539.51	217.4322	19532.93	217.4458	19534.24
19538.44	217.6812	19536.34	218.027	19540.3	217.6868	19538.41	217.6776	19539.99	217.6881	19531.78	217.6828	19536.27
19536.51	217.9377	19535.29	218.272	19538.39	217.9338	19536.68	217.9363	19538.98	217.9325	19533.33	217.9365	19535.58
19534.41	218.1866	19534.78	218.5234	19537.43	218.1975	19537.26	218.1791	19539.55	218.1917	19531.95	218.1855	19533.81
19536.62	218.4314	19535.61	218.775	19538.93	218.432	19538.74	218.4308	19539.49	218.4337	19531.91	218.4408	19534.43
19534.47	218.6795	19538.55	219.0289	19538.37	218.6871	19537.38	218.6806	19537.24	218.6891	19534.84	218.6843	19531.97
19537.23	218.934	19537.77	219.277	19538.28	218.9323	19535.75	218.9383	19541.47	218.9345	19533.06	218.9408	19534.14
19536.16	219.1823	19536.11	219.5232	19536.96	219.1878	19537.52	219.178	19539.67	219.1899	19532.09	219.1891	19529.39
19536.36	219.4319	19537.52	219.7771	19538.27	219.4353	19537.3	219.4294	19538.48	219.4358	19533.64	219.4417	19533.78
19535.75	219.6815	19538.49	220.0283	19537.53	219.6868	19537.43	219.6805	19540.09	219.6885	19532.36	219.6849	19535.94
19534.49	219.9353	19537.44	220.2759	19537.79	219.9354	19538.02	219.9365	19539.63	219.9359	19532.97	219.9399	19534.35
19533.95	220.1827	19536.43	220.5226	19537.88	220.1899	19537.5	220.1804	19537.73	220.1933	19530.38	220.1873	19536.03
19535.6	220.435	19536.52	220.7774	19537.83	220.4341	19539.23	220.4316	19538.59	220.4341	19533.36	220.4416	19532.79
19535.54	220.682	19534.84	221.0289	19540.52	220.6849	19538.41	220.6804	19537.38	220.691	19532.31	220.6871	19533.65
19536.19	220.9354	19533.6	221.2761	19538.77	220.9331	19539.5	220.9366	19539.3	220.9345	19535.08	220.9429	19534.22
19534.31	221.1838	19536.27	221.5266	19538.75	221.1937	19538.11	221.1834	19539.34	221.1913	19532.49	221.1859	19535.13
19534.34	221.4346	19535.11	221.7772	19538.5	221.4339	19537.18	221.4404	19538.85	221.4349	19533.99	221.4401	19533.39
19537.65	221.6823	19536.08	222.0288	19536.6	221.6864	19538.09	221.6831	19537.67	221.6911	19532.86	221.6857	19534.15
19534.58	221.9333	19534.48	222.2764	19537.79	221.9332	19536.26	221.9369	19537.43	221.936	19530.37	221.9395	19533.53
19536.24	222.1838	19534.83	222.5232	19538.05	222.1899	19538.2	222.1834	19539.1	222.1917	19530.14	222.1862	19531.45
19535.13	222.434	19535.82	222.7766	19538.44	222.4344	19536.4	222.4312	19541.07	222.4352	19532.42	222.4432	19534.98
19533.63	222.683	19534.32	223.0294	19538.04	222.6906	19538.29	222.6819	19540.37	222.6944	19533.87	222.6881	19531.04
19536.23	222.938	19536.41	223.2775	19538.13	222.9352	19537.17	222.9427	19538.97	222.9388	19533.25	222.9384	19532.83
19535.22	223.1848	19537.36	223.5242	19538.12	223.1906	19538.19	223.183	19539.32	223.1946	19534.95	223.1858	19533.7
19535.38	223.4344	19534.93	223.7763	19539.37	223.4346	19536.31	223.4311	19538.37	223.4355	19532.73	223.4446	19534.09
19535.51	223.683	19536.51	224.0299	19535.88	223.6882	19536.96	223.6814	19539.72	223.6899	19531.12	223.6862	19531.63
19533.33	223.9357	19538.39	224.2756	19538.71	223.9347	19536.97	223.9409	19541.69	223.9365	19533.42	223.9391	19532.2
19536.41	224.1844	19539.03	224.5242	19536.7	224.1946	19535.63	224.1808	19538.94	224.1976	19532.81	224.1866	19535.58
19536.36	224.4351	19539.67	224.7864	19541.67	224.4354	19539.18	224.4344	19541.66	224.4356	19533.16	224.4441	19533.02
19536.56	224.6826	19536.56	225.0297	19540.1	224.6915	19538.39	224.6857	19538.77	224.69	19532.66	224.6875	19532.62
19536.86	224.9378	19537.39	225.2791	19540.71	224.9351	19537.52	224.9381	19537.37	224.9377	19529.29	224.9429	19534.54
19535.78	225.1871	19534.95	225.5243	19538.13	225.1909	19536.58	225.1832	19537.43	225.1956	19535.31	225.1872	19533.31
19535.29	225.4363	19534.68	225.7773	19537.12	225.4387	19537.43	225.4339	19537.19	225.437	19533.96	225.4446	19531.7
19535.83	225.6829	19537.12	226.0305	19535.72	225.6891	19539.18	225.6829	19537.94	225.6913	19531.88	225.6872	19533.36
19530.57	225.9377	19537.72	226.2774	19539.94	225.9354	19537.6	225.9409	19540.76	225.9378	19534.08	225.942	19534.35
19536.06	226.1845	19536.81	226.525	19538.66	226.1918	19535.98	226.1867	19541.95	226.1959	19533.16	226.1876	19533

19534.4	226.437	19536.03	226.7778	19539.2	226.4375	19537.82	226.4323	19539.3	226.4373	19532.56	226.4425	19535.78
19533.39	226.6835	19536.89	227.0319	19541.01	226.689	19537.38	226.686	19540.12	226.6941	19532.96	226.6871	19533.8
19535.52	226.9378	19533.63	227.2779	19539.49	226.9383	19537.06	226.9395	19540.52	226.9374	19532.32	226.9418	19534.97
19535.85	227.1842	19533.02	227.5271	19538.53	227.1936	19537.58	227.1863	19543.19	227.1955	19532.76	227.1879	19533.26
19536.37	227.4377	19537.2	227.782	19537.02	227.437	19538.56	227.4346	19539.72	227.4377	19534.12	227.4464	19534.49
19533.67	227.6846	19535.55	228.031	19540.31	227.6917	19537.53	227.6844	19539.7	227.6923	19530.59	227.6807	19534.06
19534.93	227.9402	19539.24	228.2794	19537.16	227.9354	19538.33	227.9402	19538.92	227.9389	19534.47	227.9408	19530.44
19534.69	228.186	19532.36	228.5316	19539.3	228.1936	19537.62	228.1837	19536.76	228.1947	19532.36	228.187	19533.12
19535.59	228.4382	19537.64	228.78	19540.44	228.4389	19537.62	228.434	19536.34	228.4381	19531.12	228.4453	19530.75
19538.33	228.6842	19537.78	229.0319	19536.2	228.6904	19538.51	228.6848	19541.85	228.6929	19532.3	228.688	19532.43
19535.43	228.9371	19533.6	229.2784	19536.13	228.9386	19536.79	228.9414	19537.11	228.9405	19531.27	228.9411	19534.17
19534.57	229.1848	19536.71	229.5256	19539.58	229.1921	19536.17	229.1857	19537.47	229.1981	19533.01	229.1887	19530.9
19536.24	229.437	19534.07	229.7809	19540.8	229.438	19536.22	229.4344	19538.04	229.4343	19532.53	229.445	19533.57
19538.06	229.6861	19534.04	230.0331	19535.85	229.6911	19537.01	229.6916	19540.36	229.6932	19530.65	229.6884	19534.72
19535.17	229.9379	19535.3	230.2786	19540.85	229.9376	19536.26	229.9418	19542.39	229.9389	19531.22	229.9427	19534.35
19533.55	230.1845	19535.07	230.5296	19535.68	230.1972	19537.04	230.1937	19539.2	230.1976	19534.6	230.1883	19531.61
19533.93	230.4396	19535.96	230.7804	19539.2	230.4425	19537.58	230.4358	19538.68	230.4414	19531.39	230.4457	19535.29
19535.46	230.685	19534.77	231.032	19539.6	230.6911	19540.62	230.691	19538.4	230.6961	19533.03	230.6827	19533.18
19536.03	230.9386	19536.25	231.2791	19540.23	230.9397	19536.66	230.9432	19537.2	230.9416	19532.98	230.9446	19533.84
19536.57	231.1859	19534.51	231.5268	19539.21	231.1955	19534.13	231.1871	19537.58	231.1965	19531.92	231.1889	19531.7
19533.89	231.4399	19536.03	231.7823	19538.88	231.4405	19538.8	231.4374	19539.13	231.4402	19533.8	231.4471	19532.29
19537.65	231.6864	19535.85	232.0321	19539.57	231.6922	19537.81	231.6886	19537.59	231.6943	19531	231.6926	19534.06
19535.91	231.9393	19536.93	232.2807	19538.39	231.9381	19538.98	231.9423	19541.08	231.9381	19530.54	231.9435	19531.2
19536.98	232.1862	19537	232.5262	19538.5	232.194	19537.18	232.1866	19542.22	232.1972	19533.77	232.1903	19534.14
19535.9	232.4525	19534.48	232.7778	19539.36	232.4399	19538.25	232.4347	19541.91	232.4382	19530.96	232.4465	19532.37
19533.83	232.6887	19536.49	233.0392	19537.56	232.6941	19535.32	232.686	19539.35	232.6949	19531.76	232.7048	19534.26
19535.74	232.9426	19535.19	233.277	19539.13	232.9398	19536.3	232.9422	19537.93	232.9396	19532.07	232.944	19530.69
19534.84	233.1854	19536.27	233.5303	19538.5	233.1968	19537.06	233.1878	19539.92	233.1984	19532.75	233.1904	19536.01
19534.64	233.4417	19536.98	233.7778	19538.29	233.4408	19538.95	233.4513	19539.99	233.4394	19530.63	233.4455	19532.32
19536.56	233.6878	19537.01	234.034	19539.29	233.6956	19534.24	233.6984	19538.76	233.6937	19532.56	233.6928	19534.66
19533.58	233.94	19536.08	234.281	19537.03	233.9383	19538.26	233.9416	19538.21	233.9417	19531	233.9435	19531.67
19534.97	234.1865	19536.6	234.528	19539.12	234.1959	19537.69	234.1904	19539.15	234.1999	19533.14	234.1922	19534.33
19536.37	234.4391	19535.59	234.7793	19537.47	234.444	19536.49	234.4399	19538.62	234.4387	19532.36	234.447	19530.96
19535.66	234.6863	19533.78	235.0357	19535.54	234.6924	19535.94	234.6866	19541.54	234.6965	19535.15	234.6864	19535.35
19535.17	234.9411	19536.76	235.2813	19537.43	234.9433	19537.49	234.9441	19538	234.9419	19533.44	234.944	19532.89
19535.38	235.1869	19535.67	235.5292	19537.67	235.1942	19537.75	235.1878	19539.1	235.1985	19529.09	235.1913	19534.11
19535.13	235.4411	19535.36	235.7823	19538.99	235.4393	19538.71	235.4391	19538.88	235.4409	19534.09	235.4471	19532.54
19535.86	235.6879	19537.94	236.0378	19539.05	235.6937	19535.9	235.6891	19539.06	235.696	19534.4	235.6935	19533.26
19535.44	235.9406	19535.14	236.2799	19536.55	235.9449	19536.52	235.9445	19538.25	235.9447	19533.23	235.9445	19533.47
19535.87	236.1875	19534.19	236.53	19537.12	236.1946	19540.05	236.1914	19539.18	236.2015	19533.34	236.1921	19533.88
19534.68	236.442	19534.84	236.7829	19536.78	236.4396	19539.29	236.4386	19540.84	236.4423	19531.52	236.4503	19532.11
19533.88	236.6882	19536.8	237.0352	19538.06	236.693	19538.69	236.6911	19540.48	236.6975	19532.77	236.6948	19534.34
19534.71	236.9417	19535.76	237.281	19538.49	236.9431	19537.62	236.9452	19539.98	236.9458	19532.33	236.9443	19532.3
19537.08	237.1889	19534.68	237.529	19535.89	237.1941	19538.96	237.1911	19535.44	237.1976	19533.41	237.1919	19533.12
19533.8	237.4439	19535	237.783	19539.37	237.4406	19537.38	237.4399	19538.85	237.4419	19530.21	237.4492	19532.56
19534.16	237.6878	19533.66	238.0361	19537.42	237.6965	19538.21	237.6916	19538.63	237.6996	19534.05	237.6945	19532.05
19533.59	237.944	19537.22	238.2821	19539.51	237.9423	19536.92	237.9476	19540.08	237.9416	19534.75	237.9479	19534.2
19535.67	238.188	19536.1	238.5324	19538.64	238.1975	19538.51	238.191	19540.07	238.2007	19531.04	238.1952	19533.13
19537.89	238.443	19536.22	238.7824	19536.55	238.4376	19538.2	238.4401	19541.21	238.4418	19532.98	238.4494	19531.96
19534.95	238.6891	19537.26	239.0391	19540.86	238.6938	19537.8	238.6938	19540.48	238.7139	19533.32	238.6929	19532.97
19533.34	238.9403	19536.71	239.2795	19540.11	238.9404	19537.94	238.9455	19539.71	238.959	19531.5	238.9472	19533.41
19535.43	239.1874	19535.59	239.5288	19538.66	239.1976	19536.84	239.1894	19538.21	239.2008	19534.04	239.194	19531.77
19533.85	239.4447	19534.96	239.783	19539.04	239.4433	19536.58	239.4391	19541.17	239.4414	19532.84	239.4492	19531
19534.98	239.6888	19534.53	240.0367	19537.92	239.694	19537.07	239.6938	19539.11	239.696	19531	239.6899	19534.54
19534.24	239.9403	19535.65	240.2849	19537.3	239.942	19537.25	239.9467	19540.72	239.9453	19533.94	239.9473	19531.36
19535.69	240.1895	19535.89	240.5254	19537.49	240.1975	19538.38	240.1918	19540.39	240.2027	19531.32	240.1931	19532.85
19535.86	240.4457	19535.68	240.7802	19538.66	240.4421	19537.65	240.4467	19539.93	240.4424	19532.74	240.4468	19531.69
19534.19	240.6847	19536.66	241.0349	19539.54	240.6904	19540.33	240.6868	19539.45	240.6911	19532.63	240.6921	19535.08
19534.93	240.9374	19534.65	241.2824	19538.13	240.9372	19537.23	240.9433	19538.18	240.9397	19533.85	240.9426	19530.57
19535.16	241.185	19539.37	241.5282	19536.51	241.1967	19538.24	241.1884	19537.19	241.2104	19532.78	241.1902	19535.1
19533.83	241.4463	19533.9	241.7768	19538.14	241.438	19538.05	241.437	19539.94	241.4383	19533.85	241.444	19532.93
19536.37	241.6911	19537.24	242.0357	19541.74	241.6902	19537.05	241.6867	19537.21	241.6916	19533.52	241.7036	19534.99
19534.8	241.9428	19535.86	242.2803	19536.22	241.9395	19535.91	241.9401	19538.76	241.9423	19533.55	241.9444	19534.35
19535.36	242.1941	19535.63	242.5284	19538.47	242.1923	19537.78	242.1882	19540.58	242.1977	19528.95	242.195	19533.44
19534.63	242.443	19537.3	242.7826	19536.66	242.4384	19537.32	242.436	19540.29	242.4411	19532.41	242.4497	19531.32
19534.89	242.6902	19535.33	243.0355	19536.87	242.6932	19535.95	242.6893	19537.12	242.6927	19532.08	242.7041	19530.47
19534.26	242.9451	19536.17	243.283	19539.22	242.9398	19535	242.9419	19538.8	242.94	19535.84	242.9418	19532.15
19536.56	243.1877	19534.88	243.5297	19540.39	243.1958	19538.18	243.1872	19540.31	243.1985	19532.85	243.1934	19532.45
19537.18	243.4428	19536.53	243.7801	19536.74	243.4383	19539.39	243.4364	19539.47	243.4389	19532.09	243.4456	19530.66
19533.92	243.6854	19536.55	244.0353	19536.43	243.6915	19535.88	243.6887	19538.28	243.6959	19531.9	243.6889	19532.41

19534.18	243.9417	19534.63	244.28	19538.92	243.942	19538.72	243.9442	19537.78	243.9416	19530.25	243.9428	19530
19536.76	244.1882	19535.63	244.5283	19536.95	244.1927	19538.67	244.1908	19539.87	244.1977	19531.98	244.1912	19530.35
19533.49	244.442	19536.13	244.779	19536.88	244.4386	19537.17	244.4367	19537.59	244.4393	19530.4	244.4474	19535.43
19535.5	244.6881	19535.53	245.0351	19537.18	244.6911	19537.98	244.6932	19537.66	244.6951	19530.17	244.6906	19531.75
19536.28	244.9398	19535.83	245.2817	19537.33	244.9399	19537.71	244.9428	19539.82	244.9692	19535.29	244.9434	19536.77
19535.45	245.1874	19537.09	245.5281	19538.84	245.1943	19536.44	245.1873	19538.89	245.2086	19535.23	245.1905	19530.12
19534.95	245.4428	19536.74	245.7802	19536.83	245.441	19539.82	245.4416	19538.01	245.4397	19535.89	245.4519	19531.66
19535.43	245.6881	19535.95	246.0354	19536.64	245.6905	19539.07	245.6878	19536.79	245.6963	19533.24	245.6922	19534.48
19532.85	245.9417	19538.1	246.2823	19538.05	245.9399	19536.51	245.9439	19539.35	245.9429	19535.58	245.944	19533.12
19534.62	246.1867	19535.88	246.529	19540.97	246.1944	19536.27	246.1889	19535.39	246.1992	19532.43	246.1916	19531.44
19536.3	246.4425	19535.66	246.7832	19536.23	246.4412	19539.05	246.4396	19537.54	246.4419	19534.56	246.4462	19531.56
19535.1	246.6882	19536.77	247.0352	19537.71	246.6928	19536.55	246.6893	19538.48	246.6951	19535.12	246.6942	19532.13
19533.43	246.9404	19536.39	247.2816	19539.54	246.9452	19539.31	246.9413	19539.95	246.9431	19534.01	246.9461	19533.72
19538.03	247.1872	19536.32	247.5292	19538.12	247.1965	19536.43	247.1885	19538.26	247.1978	19533.51	247.1901	19531.02
19533.72	247.4444	19535.72	247.7826	19535.93	247.4417	19537.95	247.4353	19539.75	247.4408	19533.19	247.4469	19531.86
19536.58	247.6918	19535.17	248.0357	19536.18	247.7019	19537.18	247.6866	19539.47	247.6952	19532.46	247.6945	19531.31
19534.34	247.9433	19536.12	248.2824	19537.74	247.9418	19536.48	247.9423	19541.55	247.9432	19532.38	247.9452	19531.56
19535.63	248.1902	19535.44	248.5302	19539.21	248.1941	19538.07	248.1892	19536.53	248.1976	19532.25	248.1908	19532.33
19533.44	248.4434	19536.25	248.7819	19536.42	248.4419	19535.49	248.4367	19536.87	248.4411	19534.36	248.446	19532.64
19534.14	248.6878	19535.07	249.0347	19538.2	248.6925	19535.63	248.6885	19537.21	248.6961	19530.27	248.6953	19531.53
19534.56	248.9419	19536.71	249.2849	19536.29	248.944	19536.18	248.9424	19536.09	248.9438	19531.72	248.9455	19532.39
19535.73	249.1894	19535.28	249.5324	19536.86	249.1964	19535.79	249.1882	19537.42	249.2011	19532.26	249.1927	19533.66
19532.63	249.4454	19534.02	249.7813	19538.97	249.4411	19535.09	249.4374	19537.4	249.4432	19533.87	249.4462	19531.55
19535.53	249.6885	19537.48	250.0364	19537.71	249.6953	19535.88	249.6868	19537.74	249.6956	19533.14	249.6966	19530.98
19536.74	249.9411	19535.18	250.283	19537.13	249.9423	19537.32	249.9467	19539.68	249.9465	19533.75	249.9462	19535.01
19535.83	250.1906	19534.86	250.5306	19536.29	250.1945	19535.6	250.1889	19540	250.1988	19530.58	250.1929	19533.5
19533.42	250.4576	19536.73	250.7818	19537.2	250.443	19537.03	250.4389	19540.83	250.4436	19534.31	250.446	19528.93
19534.37	250.696	19537	251.0394	19534.18	250.6947	19538.25	250.6887	19538.19	250.6955	19531.09	250.6936	19531.29
19532.84	250.9447	19534.24	251.293	19537.97	250.9476	19539.64	250.9444	19540.16	250.9463	19533.45	250.9465	19531.83
19536.11	251.1897	19535.11	251.5526	19539.22	251.1952	19536.92	251.1901	19540.82	251.2015	19533.84	251.1935	19529.38
19534.3	251.4575	19534.92	251.7935	19537.14	251.4458	19538.95	251.4384	19537.34	251.4431	19533.25	251.4469	19533.28
19532.59	251.6955	19535.64	252.0336	19536.46	251.6943	19538.43	251.69	19536.87	251.6961	19532.65	251.6948	19531.49
19536.47	251.9436	19536.15	252.2827	19535.93	251.9431	19534.77	251.9444	19539.52	251.9457	19534.68	251.9465	19532.6
19535.09	252.1897	19535.24	252.5316	19538.97	252.1964	19537.89	252.1884	19538.77	252.2013	19531.33	252.1931	19532.49
19534.05	252.4462	19534.87	252.7839	19538.6	252.4423	19537.24	252.4391	19539.6	252.4435	19533.76	252.4507	19531.86
19533.49	252.6898	19534.12	253.0348	19540.71	252.6981	19538.89	252.692	19536.73	252.6954	19530.88	252.6975	19530.89
19534.79	252.9455	19534.77	253.2834	19536.07	252.9446	19536.52	252.9462	19537.16	252.9451	19533	252.947	19531.87
19534.06	253.1907	19533.2	253.5335	19539.42	253.1997	19535.5	253.1906	19540.93	253.203	19533.41	253.1989	19528.21
19535.34	253.4473	19534.92	253.7847	19537.58	253.4442	19536.69	253.4387	19540.8	253.4443	19531.12	253.4492	19531.82
19534.13	253.6915	19537.11	254.0393	19537.94	253.698	19536.29	253.6907	19535.71	253.6968	19530.11	253.697	19533.66
19534.26	253.9445	19537.31	254.2857	19538.47	253.9462	19536.4	253.9521	19538.09	253.9529	19531.11	253.9473	19528.21
19535.86	254.1913	19533.14	254.5316	19537.34	254.1978	19537.54	254.1922	19536.63	254.2106	19529.55	254.1943	19535.16
19531.94	254.4458	19534.77	254.7859	19536.61	254.4428	19536.17	254.439	19537.13	254.4477	19530.94	254.4482	19531.43
19532.74	254.6916	19538.64	255.0368	19539.16	254.6951	19537.75	254.6911	19538.83	254.6989	19532.6	254.6965	19534.21
19536.05	254.9448	19535.38	255.2848	19539.2	254.9439	19535.16	254.9446	19538.63	254.9459	19533.94	254.9495	19531.2
19535.11	255.1909	19534.59	255.5374	19537.76	255.1954	19535.4	255.1903	19538.81	255.2026	19532.14	255.2012	19534.03
19535.28	255.4485	19534.37	255.785	19536.69	255.4434	19537.7	255.4402	19537.82	255.4385	19534.84	255.4549	19534.13
19535.72	255.6926	19535.84	256.0375	19537.6	255.6961	19539.24	255.6919	19539.02	255.6989	19530.9	255.7112	19532.6
19536.98	255.9445	19536.92	256.299	19539.67	255.9438	19537.86	255.9463	19537.25	255.9474	19531.61	255.9482	19533.79
19535.84	256.1922	19534.13	256.534	19536.9	256.1974	19538.93	256.1922	19538.43	256.2084	19534.62	256.2102	19532.03
19534.04	256.4456	19534.43	256.7831	19537.8	256.4521	19538.69	256.4401	19541.79	256.4459	19533.14	256.4517	19530.63
19533.36	256.6931	19537.98	257.0375	19538.34	256.6998	19535.51	256.6924	19537.56	256.6975	19530.9	256.7002	19532.25
19536.46	256.946	19534.71	257.2868	19538.69	256.9701	19536.87	256.9484	19540.4	256.9407	19535.16	256.9485	19531.83
19534.3	257.1916	19534.69	257.5318	19540.53	257.2112	19538.11	257.1933	19539.41	257.2037	19535.18	257.1951	19531.62
19535.48	257.4493	19536.89	257.7864	19537.29	257.4555	19536.11	257.4421	19541.48	257.4418	19529.84	257.4516	19533.1
19534.38	257.6937	19535.7	258.0408	19537.37	257.6989	19537.76	257.6925	19540.82	257.7012	19533.33	257.7004	19533.03
19535.06	257.9479	19533.43	258.2854	19537.12	257.9458	19538.79	257.9478	19537.65	257.9456	19532.78	257.9493	19535.3
19534.49	258.192	19533.23	258.5376	19538.36	258.1987	19537.18	258.1925	19537.98	258.2037	19534.23	258.1962	19532.94
19536.43	258.4489	19533.24	258.8007	19536.55	258.4453	19538.03	258.4415	19537.53	258.4427	19533.15	258.4517	19530.48
19537.27	258.6938	19534.11	259.0332	19537.14	258.6979	19535.57	258.6918	19538.95	258.6993	19532.24	258.6994	19530.11
19534.98	258.9516	19536.8	259.2871	19538.33	258.9465	19535.76	258.9473	19541.69	258.9497	19535.85	258.9497	19534.91
19534.82	259.2045	19534.99	259.5331	19539.03	259.2005	19535.47	259.1924	19538.89	259.2072	19530.7	259.1979	19529.63
19537.37	259.4552	19536.73	259.7854	19537.19	259.4413	19537.93	259.4411	19538.25	259.4513	19531.73	259.4504	19531.77
19535.07	259.6972	19532.99	260.0384	19536.05	259.6959	19538.07	259.6939	19537.9	259.6975	19532.5	259.698	19531.59
19535.69	259.9454	19535.63	260.2877	19537.61	259.9452	19534.78	259.9459	19538.54	259.9473	19531.82	259.9509	19533.17
19536.99	260.1936	19539.01	260.5334	19539.52	260.1987	19538.89	260.1921	19538	260.2031	19531.09	260.1974	19533.87
19537.11	260.4496	19536.56	260.7867	19539.41	260.4452	19536.4	260.4443	19537.56	260.4476	19530.86	260.4495	19530.55
19530.17	260.6932	19534.22	261.0394	19539.18	260.6972	19535.93	260.6947	19541.44	260.6942	19530.81	260.6985	19532.72
19535.58	260.9474	19535.13	261.2876	19540.11	260.944	19535.57	260.9459	19536.32	260.9481	19533.04	260.9496	19534.12
19536.06	261.1936	19535.58	261.535	19537.47	261.1994	19536.3	261.1933	19538.37	261.2059	19531.33	261.1985	19532.21

19535.4	261.4484	19536.82	261.7864	19538.88	261.4405	19540.12	261.442	19540.9	261.4474	19530.67	261.4546	19532.49
19537.12	261.6942	19533.93	262.0398	19536.66	261.6977	19538.22	261.6945	19533.82	261.7002	19532.65	261.6982	19533.72
19535.69	261.9465	19535.21	262.2876	19539.8	261.945	19538.88	261.9467	19538.95	261.9479	19531.31	261.95	19533.13
19536.25	262.1931	19534.15	262.5344	19537.2	262.2021	19536.91	262.1957	19541.14	262.2066	19531.23	262.1967	19533.02
19534.38	262.4497	19536.5	262.7883	19536.36	262.4444	19534.73	262.4431	19540.33	262.4641	19531.1	262.4537	19534.71
19534.71	262.6968	19538.01	263.0436	19538.1	262.7007	19536.75	262.6949	19538.16	262.7082	19530.45	262.697	19535.19
19533.76	262.9493	19537.95	263.2877	19538.12	262.946	19538.14	262.9488	19538.61	262.9546	19528.39	262.9516	19531.82
19535.3	263.1934	19535.73	263.5373	19537.26	263.2095	19534.9	263.1943	19539.72	263.2074	19529.81	263.1948	19534.14
19533	263.4504	19535.12	263.7875	19535.93	263.4627	19536.87	263.4442	19541.51	263.449	19530.53	263.4552	19531.95
19532.21	263.7051	19534.62	264.0396	19536.08	263.7148	19537.62	263.6944	19538.2	263.6992	19529	263.6999	19532.11
19535.62	263.9648	19535.43	264.2884	19537.14	263.9592	19537	263.9485	19540.91	263.9481	19531.66	263.9463	19531.55
19536.61	264.2074	19535.87	264.5361	19536.94	264.208	19538.67	264.1941	19539.74	264.2092	19534.98	264.1963	19531.78
19534.61	264.4591	19535.7	264.7902	19537.62	264.4516	19537.5	264.4433	19536.34	264.454	19529.61	264.4524	19533.17
19535.05	264.7042	19537.66	265.0411	19536.68	264.6959	19537.41	264.6942	19539.5	264.7008	19532.96	264.6979	19533.9
19534.39	264.9491	19536.91	265.2863	19538.82	264.9477	19534.57	264.9491	19540.3	264.9446	19532.39	264.9517	19529.74
19533.52	265.196	19534.32	265.5363	19538.96	265.2011	19536.55	265.1957	19540	265.2084	19531.54	265.1935	19530.3
19534.19	265.4517	19536.86	265.7928	19536.18	265.4458	19535.2	265.4462	19540.22	265.4524	19534.06	265.4532	19535.35
19533.74	265.6989	19534.68	266.0437	19536.81	265.7015	19536.11	265.6945	19538.51	265.7022	19530.22	265.7175	19530.07
19534.31	265.948	19537.23	266.2913	19537.01	265.9458	19537.79	265.9483	19539.36	265.9467	19531.63	265.97	19534.45
19533.41	266.1955	19537.19	266.5358	19537.33	266.2027	19538.25	266.1942	19537.21	266.2113	19531.84	266.2178	19532.35
19531.92	266.4552	19535.05	266.7902	19535.71	266.4475	19537.39	266.4438	19537.38	266.4549	19531.46	266.4656	19532.06
19534.52	266.7003	19535.5	267.0405	19538.1	266.7005	19534.89	266.6941	19538.9	266.7025	19531.87	266.718	19532.91
19531.98	266.9511	19535.76	267.2901	19537.27	266.9463	19539.03	266.9501	19541.64	266.9485	19531.4	266.967	19533.24
19537.02	267.196	19534.29	267.5374	19537.28	267.2286	19535.92	267.2018	19537.58	267.2081	19529.94	267.2157	19527.62
19534.71	267.4578	19535.19	267.7895	19540.23	267.7158	19538.15	267.4462	19536.39	267.4537	19532.71	267.4714	19532.27
19535.75	267.7043	19536.89	268.0359	19537.95	267.9641	19537.36	267.6955	19542.16	267.7032	19531.19	267.7329	19530.7
19534.54	267.9564	19535.42	268.2897	19535.55	268.2138	19538.89	267.9502	19538.86	267.9489	19532.09	268.223	19528.99
19535.32	268.2132	19535.76	268.5373	19537.49	268.4604	19537.49	268.1993	19540.36	268.2064	19532.9	268.4846	19532.19
19532.97	268.473	19536.23	268.7861	19538.5	268.71	19536.62	268.4489	19539.34	268.4519	19534.07	268.7385	19532.24
19534.18	268.738	19535.4	269.0409	19537.07	268.9568	19535.15	268.6951	19538.19	268.7007	19534.21	268.9925	19532.29
19535.48	269.2185	19534.95	269.2872	19536.27	269.2146	19536.22	268.9493	19537.29	268.9495	19531.44	269.4887	19531.14
19537.06	269.4718	19536.48	269.5336	19536.63	269.4667	19537.7	269.1981	19536.97	269.2048	19530.2	269.7439	19532.72
19532.75	269.7231	19533.91	269.7907	19535.65	269.7249	19536.67	269.4485	19539.4	269.45	19531.42	269.9975	19530.97
19534.89	269.9786	19535.77	270.0394	19537.14	269.9753	19537.04	269.6961	19537.4	269.7002	19530.49	270.4895	19532.94
19535.7	270.226	19535.55	270.2893	19535.87	270.2271	19536.46	269.9511	19540.27	269.9485	19529.68	270.7421	19530.62
19534.11	270.4819	19536.6	270.536	19538.63	270.4751	19537.29	270.2006	19538.05	270.2045	19530.44	270.9934	19531.65
19534.39	270.738	19533.78	270.7898	19535.9	270.7254	19536.54	270.4574	19537.54	270.4524	19531.56	271.2428	19533.56
19534.29	270.9906	19538.83	271.0398	19535.03	270.9739	19537.11	270.7055	19539.87	270.7026	19532.26	271.4971	19533.38
19532.86	271.2407	19534.23	271.2881	19537.53	271.2271	19539.75	270.9612	19541.14	270.9535	19532.89	271.7567	19532.73
19533.92	271.4958	19533.95	271.5337	19535.65	271.4751	19539.31	271.2105	19538.48	271.2118	19531.49	272.242	19532.61
19536.52	271.9802	19536.74	271.7898	19535.06	271.7301	19538.75	271.4621	19537.62	271.4601	19530.81	272.5026	19531.71
19535.62	272.2301	19535.01	272.0403	19539.57	271.9776	19537.08	271.7103	19537.53	271.711	19530.49	272.7523	19531.67
19534.63	272.4851	19536.09	272.2891	19536.88	272.4665	19534.16	271.9624	19537.17	271.9592	19533.62	273.0035	19531.73
19533.15	272.7355	19535.45	272.5378	19537.76	272.7209	19538.08	272.2128	19541.66	272.2167	19532.33	273.2517	19532.79
19532.82	272.9925	19537	272.7908	19537.78	272.968	19537.56	272.465	19538.5	272.4648	19529.27	273.5068	19529.96
19534.75	273.243	19535.5	273.0419	19537.86	273.2237	19536.73	272.7133	19537.66	272.7162	19531.36	273.7612	19531.42
19534.43	273.4989	19535.75	273.2909	19536.8	273.4724	19534.62	272.9666	19539.63	272.9692	19532.71	274.2473	19531.74
19533	273.9821	19534.06	273.5401	19538.96	273.7265	19534.8	273.2229	19537.91	273.2281	19533.3	274.5021	19533.36
19534.29	274.2319	19534.63	273.7934	19537.01	273.9744	19539.15	273.7032	19537.61	273.7161	19533.94	274.7551	19533.18
19533.52	274.4895	19535.12	274.0491	19538.24	274.2284	19535.79	273.9608	19538.72	273.9763	19532.04	275.0074	19530.95
19535.37	274.7371	19536.12	274.3004	19537.21	274.4773	19536.91	274.2198	19539.64	274.2357	19533.46	275.258	19532.86
19534.1	274.9927	19534.84	274.5484	19539.27	274.731	19539.62	274.4749	19538.17	274.7212	19534.23	275.5223	19530.93
19533.38	275.2434	19533.86	274.7988	19537.26	274.9809	19539.6	274.7246	19537.17	274.9749	19533.74	276.0117	19531.43
19534.56	275.5012	19536.64	275.053	19537.66	275.2375	19535.12	274.9815	19540.06	275.2377	19533.28	276.2605	19533.66
19534.72	275.7525	19535.27	275.3039	19539.17	275.7271	19536.32	275.4651	19536.99	275.4871	19533.56	276.5145	19532.36
19535.77	276.2314	19536.18	275.5549	19536.06	275.9781	19535.81	275.7165	19537.17	275.74	19531.52	276.7749	19531.14
19533.33	276.4896	19536.12	275.8054	19537.07	276.2319	19537.5	275.9734	19538.82	276.2334	19529.83	277.0294	19532.84
19533.23	276.7426	19536.87	276.0588	19538.41	276.4834	19536.73	276.2242	19538.97	276.4838	19535.61	277.5191	19533.24
19536.39	277.0011	19535.37	276.5479	19536.72	276.7373	19537.29	276.4794	19539.54	276.7369	19532.04	277.7734	19530.19
19533.92	277.2528	19533.66	276.7992	19535.86	277.2327	19535.51	276.7318	19537.35	276.9888	19530.96	278.0269	19530.57
19534.64	277.5205	19536.57	277.0515	19536.64	277.5008	19536.92	276.9899	19538.1	277.2511	19532.05	278.2851	19531.5
19534.65	278.0187	19534.51	277.3055	19536.92	277.9853	19539.22	277.4702	19537.09	277.7429	19530.39	278.7718	19535.88
19534.36	278.2701	19536.72	277.5576	19537.07	278.2428	19537.05	277.7223	19539.37	277.9985	19533.92	279.026	19535.92
19532.76	278.5272	19534.19	277.8113	19537.94	278.4929	19535.99	277.9788	19540.14	278.259	19532.76	279.279	19531.19
19533.45	279.0118	19537.15	278.0731	19538.34	278.7491	19536.24	278.2308	19537.4	278.7461	19534.63	279.5445	19533.43
19534.86	279.2731	19537.55	278.5621	19535.95	279.0017	19538.58	278.4839	19539.64	278.9988	19533.02	280.0341	19532.04
19534.76	279.5306	19536.05	278.8277	19535.31	279.2609	19535.84	278.7351	19537.54	279.2611	19531.44	280.2905	19530.77
19531.96	280.0204	19535.75	279.3141	19537.96	279.7519	19534.32	278.9939	19541.04	279.5152	19532.82	280.5489	19531.18
19533.82	280.2743	19536.23	279.5684	19539.14	280.0036	19537.4	279.4759	19539.74	280.0037	19533.15	281.0376	19530.65
19535.45	280.5345	19534.74	279.8229	19535.46	280.263	19536.29	279.7261	19539.86	280.2651	19532	281.2926	19532.56

19533.71	281.0232	19534.7	280.0788	19537.94	280.5145	19538.61	279.9865	19537.11	280.5202	19532.03	281.5519	19532.31
19533.15	281.281	19534.5	280.3309	19534.44	280.9996	19537.61	280.2458	19537.02	281.0072	19530.93	282.0465	19530.45
19537.48	281.5407	19534.35	280.8173	19537.99	281.2566	19536.41	280.5133	19540.03	281.2698	19529.95	282.3021	19531.43
19531.59	282.0302	19534.77	281.0751	19536.84	281.5097	19535.73	281.0084	19540.67	281.5224	19532.64	282.5624	19530.67
19534.46	282.2886	19536.68	281.3276	19539	281.768	19538.12	281.2609	19536.31	281.7772	19532.16	283.0545	19534.52
19534.14	282.5496	19534.73	281.5834	19537.88	282.26	19535.02	281.5242	19537.37	282.2717	19531.89	283.3096	19527.79
19531.86	283.0387	19535.34	282.0799	19536.39	282.5122	19535.75	282.0167	19537.87	282.5299	19533.14	283.5707	19532.14
19535.31	283.2991	19537.34	282.3341	19537.8	282.7718	19536.1	282.2701	19538.52	282.7849	19533.5	284.0646	19529.92
19532.52	283.558	19535.15	282.5875	19538.14	283.2642	19534.49	282.5281	19537.27	283.2807	19532.31	284.3211	19533.36
19534.48	284.047	19535.34	282.8419	19536.17	283.5166	19537.17	283.0191	19538.34	283.5393	19529.9	284.5823	19533.99
19533.99	284.2949	19535.08	283.3305	19535.48	283.7774	19538.14	283.2768	19537.56	284.0277	19530.49	285.072	19533.58
19536.08	284.5464	19536.01	283.5881	19538.24	284.2715	19537.76	283.536	19537.12	284.2892	19531.95	285.3271	19532.31
19535.88	284.7918	19534.44	283.8401	19536.15	284.5244	19535.88	284.0275	19537.91	284.5439	19531.96	285.5896	19532.18
19531.35	285.0514	19535.96	284.0996	19537.04	284.7882	19537.22	284.282	19538.93	285.0329	19530.8	286.0807	19529.56
19533.41	285.3006	19534.09	284.591	19538.58	285.2832	19536.34	284.5482	19538.49	285.2961	19530.47	286.3408	19529.52
19535	285.5508	19535.48	284.8442	19537.01	285.5382	19535.1	285.0391	19538.18	285.551	19532.23	286.6161	19529.4
19534.77	285.7971	19535.2	285.1039	19537.09	285.798	19535.83	285.2935	19537.07	286.0428	19528.35	287.106	19532.22
19533.93	286.0473	19536.52	285.5945	19536.25	286.2941	19535.78	285.5558	19537.76	286.3046	19532.35	287.395	19529.7
19535.33	286.2965	19534.15	285.8483	19538.38	286.5489	19537.98	286.0468	19540.86	286.7935	19528.83	287.8923	19532.26
19534.59	286.5478	19534.14	286.1102	19537.32	286.812	19535.5	286.3008	19537.84	287.0398	19532.39	288.1545	19532.89
19533.56	286.798	19534.36	286.6018	19534.36	287.3056	19535.27	286.5511	19538.94	287.2998	19533.85	288.647	19533.56
19535.19	287.0507	19534.83	286.856	19535.47	287.5621	19534.55	286.8013	19542.16	287.544	19532.81	288.9079	19532.25
19534.54	287.3049	19535.26	287.1182	19537.31	288.0569	19536.76	287.0548	19536.34	287.7927	19530.87	289.4033	19531.63
19533.55	287.5603	19534.29	287.6124	19539.73	288.3194	19536.76	287.3011	19539.8	288.0439	19532.11	289.6639	19532.33
19534.4	287.8114	19537.44	287.8683	19536.94	288.8104	19536.51	287.5529	19540.39	288.2953	19530.43	290.1544	19530.16
19534.11	288.3035	19534.58	288.1321	19540.31	289.0706	19536.82	287.7975	19538.98	288.5421	19531.88	290.418	19529.49
19533.64	288.5577	19536.13	288.6315	19538.1	289.3222	19536.3	288.0513	19537.93	288.7902	19531.07	290.9121	19531.55
19533.07	288.8047	19535.98	288.888	19536.92	289.5674	19536.07	288.2976	19537.94	289.0428	19532.1	291.1754	19532.4
19531.53	289.0627	19534.17	289.3772	19540.21	289.8177	19536.96	288.549	19536.3	289.2977	19531.78	291.6741	19531.68
19533.41	289.315	19534.47	289.6416	19538.07	290.0659	19537.34	288.795	19539.47	289.5468	19530.18	291.9357	19531.13
19536.5	289.8006	19533.71	290.1392	19538.61	290.3196	19535.29	289.0497	19536.35	289.7958	19529.54	292.4334	19528.68
19529.95	290.0554	19534.3	290.3972	19537.57	290.5663	19537.42	289.2966	19537.96	290.059	19533.13	292.6985	19530.89
19534.99	290.3063	19535.69	290.887	19534.94	290.8167	19535.81	289.5488	19535.63	290.5557	19530.58	293.1975	19529.29
19533.99	290.5642	19534.84	291.1509	19537.37	291.0634	19533.84	289.7954	19541.92	290.8033	19529.58	293.4723	19534.98
19535.13	290.8176	19532.65	291.6494	19537.62	291.3164	19534.79	290.0497	19536.74	291.0528	19532.92	293.9715	19530.72
19533.32	291.0707	19534.67	291.9097	19534.73	291.5643	19537.56	290.2985	19538.96	291.3087	19531.68	294.2357	19531.77
19534	291.5628	19534.78	292.4011	19536.73	291.8159	19536.82	290.5524	19537.13	291.5599	19535.14	294.7341	19531.67
19535.27	291.8201	19533.42	292.6647	19538.2	292.0626	19536.76	290.8	19539.57	291.8101	19532.43	295.0003	19532.34
19534.55	292.0783	19534.7	293.1647	19538.68	292.3164	19534.07	291.0545	19540.5	292.3176	19530.38	295.4972	19531.07
19532.83	292.5729	19534.19	293.4212	19536.28	292.5675	19538.14	291.5488	19540.54	292.8096	19532.68	295.7652	19529.45
19532.47	292.8248	19534.38	293.9124	19538.38	292.8378	19538.14	291.7962	19536.95	293.0611	19530.14	296.2579	19530.78
19534.39	293.0786	19535.92	294.1801	19536.24	293.3419	19534.02	292.056	19536.48	293.3147	19531.62	296.5283	19531.07
19534.83	293.3374	19534.75	294.6777	19536.77	293.838	19535.92	292.3044	19538.87	293.5645	19532.53	297.0299	19532.23
19533.57	293.8228	19533.94	294.9369	19536.07	294.0873	19536.45	292.5583	19537.41	293.8125	19531.08	297.2966	19531.05
19533.3	294.0771	19534.24	295.4282	19537.72	294.3427	19536.82	292.8067	19537.41	294.0631	19531.21	297.7925	19531.09
19533.28	294.3384	19535.84	295.6933	19538.31	294.5913	19535.83	293.0627	19539.56	294.3164	19531.67	298.0557	19533.07
19535.67	294.5917	19535.51	296.1957	19537.91	294.8456	19536.93	293.5532	19538.5	294.5673	19529.23	298.5566	19531.61
19533.37	295.0834	19536.31	296.455	19538.72	295.3428	19534.62	293.8171	19537.36	294.8183	19531.44	298.8202	19533.56
19532.31	295.3427	19535.55	296.9497	19536.34	295.5912	19535.21	294.3053	19537.83	295.0714	19531.98	299.3148	19532.6
19533.47	295.5965	19534.39	297.2156	19536.05	295.8437	19534.51	294.5595	19535.46	295.5666	19531.05	299.5814	19532.92
19534.7	296.09	19536.19	297.711	19537.07	296.0944	19538.37	294.8074	19537.57	295.8176	19532.1	300.081	19530.88
19533.03	296.3589	19534.96	297.973	19537.01	296.3515	19536.98	295.0636	19538.92	296.0703	19533.25		
19532.19	296.8465	19535.46	298.4657	19535.91	296.8482	19537.66	295.3119	19534.78	296.3335	19529.76		
	297.1151	19536.64	298.731	19535.2	297.0983	19536.73	295.5682	19540.49	296.8257	19528.9		
	297.6101	19534.48	299.2283	19536.82	297.3533	19535.55	295.8192	19537.61	297.0798	19530.99		
	297.8778	19535.59	299.4897	19537.93	297.6057	19535.74	296.0772	19538.03	297.3356	19533.29		
	298.3749	19536.46	299.985	19536.86	298.1027	19539.61	296.573	19538.16	297.5893	19530.44		
	298.6407	19533.75	300.2505	19536.27	298.357	19534.31	296.822	19538.26	298.0867	19533.65		
	299.1331	19535.46			298.6071	19539.71	297.0783	19538.25	298.3543	19531.08		
	299.401	19533.43			298.8615	19535.84	297.329	19539.58	298.8489	19533.01		
	299.8907	19535.92			299.1133	19536.35	297.5844	19538.37	299.1158	19528.94		
	300.1603	19534.24			299.6069	19536.09	298.0797	19538.96	299.613	19529.1		
					299.8612	19534.54	298.3412	19538.27	299.8774	19531.05		
					300.1134	19534.11	298.8291	19539.87	300.3871	19528.1		
							299.0989	19536.7				
							299.5941	19539.39				
							299.8571	19537.47				
							300.3456	19541.96				

1.43						1.43						
57.5						60						
x $_3$		*x* $_4$		*x* $_5$		*x* $_1$		*x* $_2$		*x* $_3$		*x*
t (s)	P (cm^{-1})	t (s)	P (cm^{-1})	t (s)	P (cm^{-1})	t (s)	P (cm^{-1})	t (s)	P (cm^{-1})	t (s)	P (cm^{-1})	t (s)
0	19547.81	0	19547.19	0	19548.23	0	19540.4	0	19539.52	0	19541.64	0
0.69199	19548.88	0.7352	19546.63	0.77482	19549.39	0.66611	19540.35	0.70409	19543.74	0.66002	19540.42	0.66673
0.86229	19550.22	0.87673	19547.62	1.13237	19550.3	0.84794	19541.5	0.84982	19540.64	0.85311	19541.06	0.85706
1.10713	19548.57	1.10204	19551.47	1.37967	19551.16	1.10114	19541.47	1.09833	19543.6	1.10245	19542.51	1.10418
1.35218	19547.57	1.34942	19549.73	1.62484	19548.69	1.34694	19540.27	1.34621	19540.76	1.35717	19540.45	1.35332
1.60396	19548.3	1.60122	19548.58	1.87561	19548.82	1.59987	19539.59	1.59626	19539.58	1.6018	19542.79	1.60371
1.85108	19546	1.85061	19550.12	2.12898	19549.12	1.8457	19540.57	1.84598	19541.06	1.85229	19539.67	1.86298
2.10226	19548.68	2.10327	19548.05	2.37808	19548.1	2.11917	19539.13	2.09652	19542.26	2.10033	19541.43	2.10351
2.35437	19547.92	2.35001	19548.04	2.62644	19548.77	2.34729	19538.27	2.34607	19543.03	2.35885	19544.65	2.35182
2.60252	19546.76	2.60032	19548.2	2.87807	19549.42	2.60514	19542.21	2.59554	19540.06	2.60142	19541.65	2.6042
2.8518	19548.63	2.85069	19546.13	3.12981	19545.45	2.84706	19540.64	2.8464	19541.16	2.85251	19543	2.85572
3.10114	19546.17	3.10362	19547.39	3.37819	19549.54	3.09577	19539.86	3.09612	19545.35	3.09986	19542.53	3.10053
3.35759	19546.9	3.35018	19547.84	3.62659	19547.9	3.34716	19541.56	3.34693	19539.59	3.35706	19538.33	3.35079
3.60502	19545.14	3.60182	19545.81	3.87726	19546.62	3.60272	19537.38	3.59618	19542.54	3.60115	19543.29	3.60217
3.85222	19548.27	3.85073	19547.54	4.13098	19548.74	3.84604	19540.13	3.84628	19542.45	3.85249	19542.16	3.85481
4.10433	19544.34	4.10271	19547.66	4.37973	19548.62	4.09538	19541.04	4.09683	19542.01	4.10083	19542.17	4.10156
4.35377	19546.59	4.35104	19546.77	4.6257	19549.94	4.34711	19540.45	4.34768	19544.44	4.35747	19541.16	4.35159
4.60258	19548.19	4.60048	19547.31	4.87839	19549.78	4.60251	19540.32	4.5972	19541.62	4.60224	19539.13	4.60118
4.86147	19548.01	4.85445	19545.51	5.13368	19545.98	4.84722	19541.63	4.84609	19544.46	4.85379	19542.29	4.8579
5.11761	19546.09	5.10594	19548.16	5.37915	19551.37	5.09532	19542.29	5.09625	19543.24	5.10698	19544.88	5.10231
5.3594	19549.23	5.35161	19548.72	5.6263	19549.3	5.34728	19541.94	5.35107	19542.17	5.36007	19540.88	5.35087
5.60287	19545.88	5.60159	19547.54	5.87718	19549.3	5.60159	19540.91	5.60308	19541.66	5.60171	19541.96	5.60074
5.85214	19547.56	5.85056	19545.5	6.13068	19550.73	5.84854	19539.72	5.84765	19540.45	5.85279	19539.81	5.85589
6.10285	19545.85	6.1034	19548.96	6.37838	19548.13	6.09693	19538.86	6.09647	19541.58	6.10241	19544.32	6.10205
6.35314	19545.55	6.35173	19548.78	6.62655	19550.2	6.34802	19541.37	6.34661	19542.11	6.3632	19540.89	6.35235
6.60432	19550.05	6.60251	19545.19	6.87854	19547.92	6.60095	19542.25	6.59709	19539.84	6.60152	19540.96	6.60217
6.85414	19548.29	6.85205	19548.85	7.13127	19547.81	6.84871	19539.51	6.84865	19540.28	6.85311	19543.37	6.85691
7.10568	19545.95	7.10352	19546.64	7.37842	19550.1	7.09786	19540.64	7.09788	19541.73	7.10142	19540.65	7.10178
7.35318	19546.26	7.35051	19547.29	7.62719	19547.52	7.34693	19539.56	7.3467	19542.65	7.35582	19542.8	7.35979
7.60281	19546.73	7.60106	19546.85	7.88378	19547.29	7.60339	19536.52	7.59663	19541	7.60151	19543.36	7.61087
7.85719	19547.53	7.85267	19548.03	8.13378	19549.65	7.85494	19541.12	7.84747	19543.2	7.85357	19541.59	7.85721
8.1059	19546.33	8.10465	19547.33	8.37794	19551.16	8.0968	19541.79	8.09836	19540.52	8.10204	19539.58	8.10459
8.35581	19544.19	8.3515	19548.26	8.62589	19548.37	8.34897	19540.97	8.34856	19539.41	8.35897	19542.64	8.35952
8.60747	19546.65	8.60142	19549.63	8.87932	19549.16	8.60156	19541.23	8.5976	19542.7	8.60213	19542.72	8.60342
8.85688	19547.7	8.85262	19548.62	9.13211	19548.99	8.86892	19539.78	8.84712	19543.98	8.85465	19543.45	8.85698
9.11425	19548.88	9.10455	19548.19	9.38074	19548.6	9.09745	19539.69	9.09802	19544.13	9.10255	19542.05	9.10433
9.35204	19547.57	9.35315	19547.41	9.62643	19549.12	9.34751	19540.18	9.34868	19539.72	9.36104	19544.66	9.35663
9.60297	19550.22	9.60171	19546.91	9.88303	19547.07	9.60375	19538.83	9.59803	19543.4	9.60197	19542.2	9.60636
9.85425	19547.74	9.85133	19545.96	10.13311	19547.87	9.84887	19540.22	9.85204	19542.91	9.8549	19542.17	9.85793
10.1056	19549.88	10.10591	19549.45	10.37922	19547.79	10.09716	19542.61	10.09805	19542.91	10.10303	19542.31	10.10501
10.3543	19548.58	10.35386	19548.38	10.62824	19549.86	10.34857	19542.02	10.3495	19542.43	10.36348	19542.12	10.35917
10.60753	19547.93	10.60355	19548.98	10.88032	19548.17	10.60741	19540.41	10.59991	19543.24	10.60329	19541.83	10.60958
10.85693	19547.81	10.85261	19548.93	11.13483	19549.39	10.84995	19537.43	10.84864	19542.8	10.85479	19542.44	10.85987
11.10897	19548.42	11.10546	19548.84	11.38004	19546.6	11.09936	19540.52	11.09829	19538.13	11.10453	19541.92	11.10396
11.35558	19549.11	11.3531	19546.83	11.62704	19547.67	11.3478	19541.45	11.34973	19542.91	11.36139	19543.63	11.35308
11.60796	19549.36	11.60337	19548.97	11.87981	19547.92	11.60276	19541.41	11.60007	19540.87	11.60367	19543.64	11.60336
11.85957	19547.16	11.85447	19545.36	12.12981	19551.8	11.84963	19538.46	11.84953	19541.5	11.85509	19544.83	11.85836
12.10989	19549.85	12.10726	19547.84	12.38233	19548.48	12.1001	19541.46	12.0988	19540.81	12.1038	19540.96	12.10529
12.36078	19547.43	12.35414	19546.05	12.62744	19549.25	12.34899	19541.4	12.34888	19539.78	12.36116	19542.21	12.35427
12.61063	19549.58	12.60386	19546.24	12.87912	19548.96	12.60379	19541.27	12.59945	19539.03	12.60301	19543.96	12.60501
12.85837	19545.6	12.85333	19549.98	13.13194	19548.48	12.85091	19538.99	12.8492	19543.15	12.85498	19539.69	12.85773
13.11077	19545.83	13.10723	19548.4	13.38096	19549.34	13.09926	19541.92	13.09878	19541.92	13.10341	19541.97	13.10415
13.35908	19548.79	13.35337	19548.57	13.62965	19548.18	13.34978	19541.92	13.3483	19542.09	13.36141	19542.17	13.36079
13.60825	19549.68	13.60295	19546.91	13.87983	19550.03	13.60534	19540.1	13.60084	19537.47	13.605	19541.69	13.60492
13.86078	19548.34	13.85368	19545.24	14.12964	19549.38	13.8494	19538.87	13.84928	19541.68	13.85586	19545.42	13.86166
14.11055	19549.66	14.11178	19546.46	14.38036	19549.4	14.09851	19539.73	14.09984	19542.15	14.10446	19542.14	14.10355
14.36038	19546.19	14.35702	19548.85	14.62886	19550.53	14.34899	19539.31	14.3493	19539.4	14.36135	19540.3	14.35501
14.61018	19549.48	14.60429	19549.74	14.88129	19549.62	14.60483	19541.82	14.59957	19540.47	14.60381	19541.44	14.60477
14.8607	19547.07	14.85319	19546.47	15.13008	19550.18	14.84962	19540.32	14.84992	19541.28	14.85676	19540.35	14.86083
15.11114	19546.48	15.10867	19546.77	15.38202	19549.96	15.09884	19539.5	15.10023	19540.49	15.10511	19544.15	15.10404
15.3631	19545.2	15.3558	19549.78	15.62973	19549.22	15.34959	19542.88	15.35412	19540.97	15.36102	19542.79	15.35448
15.6111	19547.38	15.60612	19548.29	15.8816	19549.46	15.60529	19541.78	15.60202	19542.74	15.60427	19541.6	15.60515
15.86005	19549.54	15.8555	19546.47	16.13432	19550.4	15.85292	19541.51	15.85085	19539.19	15.85767	19543.49	15.8632
16.11097	19550.37	16.10873	19548.23	16.382	19549.45	16.10072	19539.73	16.10652	19542.15	16.1068	19541.97	16.10638

16.36036	19547.3	16.35464	19548.42	16.62932	19549.49	16.35002	19539.64	16.35179	19541.26	16.36825	19544.39	16.35629
16.6096	19547.09	16.60539	19548.42	16.88041	19548.53	16.60375	19539.83	16.60039	19540.61	16.60398	19540.31	16.60439
16.86072	19548.72	16.85598	19549.72	17.13293	19549.11	16.85067	19541.01	16.84959	19541.56	16.85556	19541.88	16.85905
17.1084	19548.22	17.10977	19547.91	17.38284	19545.53	17.10105	19540.99	17.09978	19539.2	17.10739	19541.99	17.10546
17.36119	19547.42	17.35363	19550.4	17.62943	19548.77	17.35014	19543	17.35107	19539.35	17.3631	19541.67	17.35537
17.61066	19546.44	17.60371	19545.52	17.88298	19548.87	17.60424	19540.85	17.60068	19542.55	17.60428	19541.51	17.60648
17.86107	19548.29	17.85521	19548.43	18.13199	19548.45	17.85143	19538.84	17.8498	19543.08	17.85651	19540.84	17.85958
18.11156	19547.91	18.11048	19547.6	18.38388	19549.95	18.10054	19541.86	18.09977	19539.66	18.10638	19541.47	18.10614
18.361	19549.16	18.35605	19548.19	18.63061	19547.08	18.35194	19541.27	18.35077	19541.31	18.36444	19543	18.35707
18.61221	19546.99	18.60414	19547.7	18.88186	19547.58	18.60807	19542.78	18.61103	19541.21	18.60554	19542.97	18.60491
18.86335	19548.12	18.8547	19548.31	19.13276	19548.45	18.85767	19539.11	18.85162	19541.73	18.85718	19541.77	18.86119
19.11358	19548.01	19.1111	19544.73	19.38162	19549.88	19.10008	19542.75	19.10016	19541.35	19.10671	19542.6	19.10598
19.36228	19548.29	19.3562	19547.53	19.63059	19548.47	19.35035	19540.55	19.35088	19543.74	19.36592	19540.03	19.36164
19.61201	19548.31	19.60572	19548.65	19.88336	19551.23	19.60499	19541.6	19.602	19542.65	19.60596	19543.68	19.61233
19.86506	19547.95	19.8543	19548.94	20.13229	19550.47	19.85284	19542.63	19.85206	19541.87	19.85708	19540.82	19.86128
20.11334	19546.37	20.10874	19547.74	20.38363	19550.86	20.10209	19542.05	20.10133	19539.9	20.1065	19541.85	20.10603
20.36294	19549.91	20.35719	19549.55	20.63188	19547.97	20.35174	19543.3	20.35169	19539.78	20.36395	19542.92	20.35766
20.61121	19549.3	20.60733	19547.32	20.8833	19547.47	20.60589	19539.5	20.60153	19541.48	20.60584	19543.89	20.60767
20.86425	19548.16	20.85554	19548.19	21.1337	19546.51	20.85396	19541.7	20.85271	19543.3	20.85857	19540.01	20.86208
21.11444	19548.18	21.11216	19548.27	21.38192	19550.76	21.10306	19539.74	21.10291	19538.23	21.10665	19542.56	21.10764
21.36547	19546.78	21.35663	19549.09	21.63184	19548.1	21.3521	19541.47	21.35201	19543.31	21.36465	19543.05	21.36135
21.60797	19548.34	21.60647	19549.18	21.88283	19548.44	21.60728	19543.51	21.60201	19541.89	21.60815	19540.87	21.60757
21.86459	19549.98	21.86693	19547.58	22.13397	19548.04	21.85354	19540.49	21.85317	19540.92	21.86004	19545.33	21.86391
22.11122	19548.56	22.11991	19547.72	22.39753	19547.49	22.10234	19540.5	22.10396	19541.76	22.10637	19542.98	22.10791
22.36548	19548.85	22.35611	19547.85	22.63195	19547.34	22.35355	19541.08	22.35345	19540.41	22.36453	19543.71	22.35795
22.61066	19546.18	22.60686	19549.12	22.88289	19547.08	22.60934	19538.55	22.60266	19542.34	22.60805	19543.54	22.60917
22.86178	19549.52	22.85827	19547.69	23.13529	19547.71	22.8548	19540.72	22.85414	19542.8	22.85982	19541.84	22.86315
23.11095	19544.47	23.11259	19549.83	23.3831	19549.02	23.10247	19539.91	23.10452	19541.03	23.10822	19541.67	23.10845
23.36105	19548.97	23.3586	19548.82	23.63344	19549.3	23.3527	19538.77	23.35387	19539.45	23.36421	19545.24	23.35852
23.61288	19549.02	23.60716	19547.81	23.88441	19551.01	23.60899	19539.77	23.60319	19541.29	23.60752	19542.72	23.60764
23.86468	19549.13	23.85747	19549.91	24.1332	19549.66	23.85617	19539.74	23.85265	19542.38	23.85889	19542.46	23.86422
24.1274	19548.69	24.11608	19549.53	24.38385	19549.76	24.10283	19542.17	24.10491	19538.96	24.10725	19543.12	24.10811
24.36174	19545.24	24.36065	19549.1	24.63356	19548.69	24.35324	19540.76	24.35462	19541.62	24.36543	19542.38	24.35925
24.61566	19546.79	24.60814	19548.11	24.88454	19548.98	24.61998	19540.75	24.60368	19542.35	24.607	19544.58	24.6078
24.86324	19548.45	24.86108	19546.96	25.13496	19548.93	24.85754	19542.06	24.85392	19540.1	24.86056	19543.12	24.86195
25.11292	19547.39	25.11122	19548.12	25.38298	19548.24	25.1024	19541.53	25.10315	19543.13	25.10798	19544.2	25.10715
25.36237	19551.36	25.35613	19548.24	25.63142	19549.4	25.3522	19538.18	25.35324	19541.56	25.36333	19543.43	25.35825
25.61298	19550.38	25.6084	19546.25	25.8838	19549.16	25.60871	19540.98	25.60548	19542.1	25.60713	19539.98	25.60894
25.86382	19549.34	25.85866	19547.87	26.13478	19549.17	25.85427	19540.88	25.85402	19541.5	25.86737	19544.59	25.8635
26.11331	19549.59	26.11299	19546.16	26.38721	19549.88	26.10406	19540.26	26.10282	19542.89	26.10894	19540.96	26.10839
26.36119	19548.66	26.35787	19549.16	26.63179	19548.08	26.35308	19540.39	26.35235	19539.35	26.36684	19540.92	26.35808
26.61586	19546.47	26.60821	19547.46	26.88425	19548.6	26.60805	19539.28	26.60375	19541.08	26.60775	19542.62	26.60959
26.86301	19549.14	26.85813	19546.71	27.13395	19550.08	26.85508	19541.55	26.85397	19542.62	26.86022	19542.12	26.86472
27.11415	19548.45	27.11341	19547.75	27.38742	19549.52	27.10413	19539.09	27.10409	19542.63	27.10837	19542.8	27.10801
27.36201	19549.28	27.35834	19546.87	27.63362	19549.2	27.35431	19542.56	27.35287	19541.4	27.36676	19543.09	27.35808
27.61582	19550.5	27.60804	19548.9	27.88428	19548.6	27.609	19537.87	27.60385	19540.35	27.60753	19538.17	27.61099
27.8601	19549.32	27.85802	19546.11	28.13446	19550.2	27.85578	19540.41	27.85505	19539.75	27.86023	19541.32	27.86507
28.11679	19548.68	28.1172	19547.35	28.38822	19547.95	28.10305	19541.77	28.10438	19542.02	28.10885	19541.82	28.10944
28.36302	19545.43	28.36196	19546.68	28.63353	19550.18	28.3538	19539.81	28.35351	19540.79	28.36559	19541.05	28.35838
28.6168	19547.95	28.60851	19547.53	28.88606	19548.05	28.60913	19537.73	28.60375	19540.92	28.6105	19543.94	28.60844
28.86598	19546.9	28.85898	19548.67	29.13369	19548.21	28.85556	19539.06	28.85399	19540.87	28.86181	19544.34	28.86484
29.11677	19545.62	29.11132	19546.17	29.39189	19550.05	29.10393	19540.8	29.10543	19540.79	29.10795	19543.02	29.11054
29.3663	19546.47	29.35851	19546.29	29.63626	19548.77	29.35413	19540.79	29.35447	19541.63	29.36562	19542.02	29.36003
29.616	19547.69	29.60875	19546.7	29.88523	19548.49	29.60951	19539.99	29.60438	19539.07	29.60972	19542.75	29.60866
29.86823	19547.13	29.85832	19547.96	30.13611	19550.46	29.85673	19540.63	29.85335	19544.43	29.86279	19542.77	29.86462
30.1169	19547.11	30.11185	19548.06	30.38865	19550.93	30.10332	19539.95	30.10484	19542.49	30.10929	19539.8	36.03149
30.36645	19547.55	30.36	19550.05	30.63482	19548.78	30.35563	19540.38	30.35595	19542.12	30.36726	19543.2	36.46023
30.61624	19547.05	30.6116	19547.29	30.88712	19548.48	30.61375	19539.62	30.60635	19540.75	30.61065	19541.41	36.67108
30.86641	19549.14	30.86031	19550.09	31.13701	19549.06	30.85755	19541.56	30.85481	19540.11	30.86331	19538.8	36.9194
31.11892	19547.89	31.11664	19547.06	31.38799	19549.13	31.10564	19543.02	31.10548	19544.58	31.11025	19543	37.16771
31.36612	19546.31	31.35934	19547.08	31.63445	19549.08	31.35452	19540.94	31.35554	19541.88	31.36695	19541.49	37.41675
31.6166	19546.93	31.60991	19546.42	31.88718	19549.51	31.6109	19540.43	31.60639	19541.19	31.60993	19539.48	37.67061
31.8693	19548.7	31.86134	19548.74	32.13678	19547.8	31.85752	19537.88	31.85752	19540.77	31.86392	19541.14	37.91925
32.11803	19546.82	32.11794	19547.31	32.38998	19548.28	32.10738	19541.22	32.10553	19543.79	32.11198	19539.45	38.1697
32.36843	19547.61	32.35951	19545.73	32.63493	19548.84	32.35541	19538.77	32.35505	19540.31	32.36774	19542.68	38.41558
32.61801	19547.24	32.60854	19547.46	32.88541	19549.07	32.60942	19540.89	32.60542	19541.79	32.60875	19540.49	38.66999
32.87405	19547.03	32.85986	19547.61	33.13449	19548.48	32.85952	19540.27	32.85708	19542.04	32.8624	19543.54	38.92051
33.11652	19548.02	33.11556	19548.22	33.38818	19547.19	33.10601	19539.6	33.10566	19542.31	33.11056	19545.12	39.1704
33.3655	19550.51	33.35998	19548.46	33.63715	19547.72	33.356	19541.76	33.35486	19544.96	33.36725	19542.33	39.417
33.61337	19548.07	33.60988	19547.11	33.88759	19547.6	33.60979	19542.49	33.60683	19542.9	33.60955	19542.31	39.67054

33.86922	19547.54	33.85944	19548.97	34.13633	19550.07	33.85599	19542.02	33.85749	19543.96	33.86297	19541.15	39.92035
34.12063	19548.59	34.11772	19548.92	34.393	19550.23	34.10527	19542.42	34.10661	19541.16	34.11006	19539.48	40.17041
34.36426	19548.82	34.36102	19545.94	34.63481	19548.14	34.35691	19540	34.35508	19542.09	34.36826	19541.11	40.41902
34.61802	19546.74	34.61027	19546.7	34.888	19549.38	34.61043	19541.4	34.60476	19541.31	34.61114	19541.48	40.67236
34.86966	19547.16	34.86011	19547.68	35.13662	19547.47	34.85705	19539.79	34.85832	19545.28	34.86275	19544.17	40.92027
35.11832	19548.01	35.11565	19547.18	35.38633	19548.97	35.10524	19538.82	35.10641	19540.24	35.10958	19542.98	41.17112
35.36939	19545.14	35.36236	19548.4	35.63463	19550.51	35.35683	19539.47	35.3562	19545.79	35.37342	19542.75	41.41935
35.61852	19547	35.61114	19546.32	35.88664	19548.12	35.61315	19539.94	35.60604	19542.94	35.61174	19541.72	41.67204
35.86945	19551.32	35.85986	19549.44	36.13859	19549.57	35.85713	19540.33	35.85748	19541.41	35.86419	19542.22	41.92052
36.11821	19548.07	36.1167	19547.5	36.38804	19550.25	36.105	19541.34	36.10758	19542.09	36.11062	19542.29	42.16932
36.36951	19546.87	36.36354	19547.19	36.63461	19549.26	36.35656	19540.67	36.35754	19541.32	36.36876	19541.8	42.41929
36.61605	19548.23	36.61222	19545.61	36.88684	19550.26	36.61416	19539.24	36.60595	19543.2	36.61066	19540.02	42.67347
36.86899	19550.55	36.86155	19548.06	37.13854	19548.4	36.85778	19541.84	36.85808	19540.85	36.86441	19546.99	42.92232
37.1155	19550.58	37.1187	19550.03	37.39013	19547.19	37.10655	19540.51	37.1071	19540.94	37.11213	19541.17	43.17152
37.36807	19547.17	37.36074	19549.9	37.63643	19546.84	37.35603	19540.4	37.35795	19540.28	37.37121	19541.4	43.41866
37.61905	19549.63	37.61166	19548.27	37.88836	19550.57	37.61115	19542.05	37.60766	19539.8	37.61094	19543.32	43.67328
37.86894	19548.53	37.86339	19546.5	38.13776	19549.46	37.85878	19541.83	37.85776	19540.76	37.86215	19539.95	43.92342
38.11764	19547.6	38.11736	19547.4	38.38905	19548.06	38.10734	19539.65	38.10608	19543.04	38.1113	19542.19	44.17209
38.36701	19544.59	38.36212	19546.79	38.63638	19549.28	38.35617	19540	38.35651	19543.08	38.373	19544.07	44.41836
38.61273	19550.2	38.61098	19549.47	38.88953	19548.62	38.61156	19540.38	38.61084	19538.92	38.61257	19542.25	44.67313
38.86471	19549.49	38.86169	19547.31	39.13746	19549.09	38.85864	19540.59	38.85913	19541.66	38.86535	19542.43	44.92271
39.11806	19546.6	39.1158	19548.53	39.38827	19549.38	39.10788	19542.63	39.10711	19542.32	39.11156	19542.12	45.17252
39.36517	19548.21	39.36321	19546.93	39.63655	19548.97	39.35866	19539.34	39.3564	19545.05	39.3733	19544.23	45.41955
39.61364	19548.32	39.61185	19546.16	39.88914	19549.59	39.61471	19541.18	39.60733	19539.51	39.61264	19540.49	45.67515
39.86525	19548.2	39.86108	19549.39	40.13677	19547.88	39.85841	19540.76	39.85845	19540.98	39.86506	19542.2	45.93222
40.11735	19547.77	40.11837	19547.4	40.38877	19548.45	40.10746	19540.84	40.10827	19542.71	40.11121	19542.12	46.17491
40.36861	19547.22	40.36447	19547.58	40.63803	19549.55	40.35846	19539.52	40.35833	19543.29	40.37278	19542.6	46.42135
40.61488	19548.16	40.61332	19550.95	40.8913	19546.85	40.61554	19541.14	40.60823	19541.27	40.61472	19542.76	46.67437
40.86875	19548.67	40.86299	19546.8	41.14022	19550.18	40.86157	19541.03	40.86006	19542.65	40.86739	19541.57	46.92401
41.1203	19547.57	41.12086	19542.95	41.38926	19549.78	41.10799	19539.11	41.11087	19542.8	41.11271	19541.96	47.17317
41.36721	19548.12	41.36566	19549.15	41.6381	19547.67	41.35856	19537.75	41.3601	19538.79	41.37283	19539.87	47.42159
41.61557	19546.77	41.6152	19547.67	41.89022	19549.11	41.61394	19540.76	41.60781	19542.59	41.61552	19543.05	47.67574
41.86378	19547.69	41.86276	19549.51	42.13995	19547.96	41.86151	19541.33	41.85794	19540.09	41.86777	19541.67	47.92446
42.11852	19546.11	42.11932	19547.35	42.39155	19550.29	42.11032	19541.05	42.11271	19540.37	42.11369	19542.16	48.17326
42.36478	19547.78	42.36424	19546.86	42.63753	19550.03	42.35901	19540.43	42.36115	19537.99	42.37219	19540.52	48.42165
42.61824	19547.69	42.6149	19544.68	42.88883	19549.79	42.61392	19542.08	42.61053	19542.23	42.61466	19540.4	48.6743
42.86809	19548.8	42.86344	19545.96	43.13883	19549.43	42.86282	19541.07	42.85962	19541.53	42.86784	19539.97	48.92324
43.11881	19549.03	43.11926	19546.88	43.3927	19551.89	43.11089	19539.51	43.10953	19541.55	43.11459	19541.67	49.17122
43.36835	19547.61	43.36382	19547.45	43.64011	19547.28	43.35925	19538.9	43.36057	19541.48	43.37145	19545.67	49.42002
43.61798	19549.56	43.61532	19549.92	43.89012	19546.55	43.61389	19541.43	43.61256	19539.35	43.61326	19540.54	49.67409
43.86421	19547.71	43.86532	19545.93	44.13872	19548.3	43.86159	19542.47	43.86028	19542.86	43.86758	19542.05	49.92597
44.12076	19546.19	44.12079	19546.38	44.39237	19547.89	44.11049	19542.34	44.11031	19542.71	44.11488	19542.81	50.17209
44.36853	19549.45	44.36359	19546.4	44.64067	19548.17	44.36178	19540.63	44.35951	19542.22	44.37615	19542.9	50.41994
44.61978	19546.23	44.61505	19549.39	44.89221	19548.07	44.61504	19540.51	44.61103	19542.19	44.61377	19540.54	50.67447
44.867	19548.4	44.86463	19547.68	45.13972	19549.33	44.86244	19541.6	44.86137	19542.52	44.86818	19541.38	50.92575
45.11727	19548.89	45.1211	19547.99	45.38982	19547.85	45.11042	19541.17	45.11084	19541.6	45.11547	19540.6	51.17425
45.3657	19547.09	45.36551	19549.35	45.63971	19549.87	45.36139	19543.22	45.35963	19539.12	45.37561	19541.13	51.41994
45.61465	19547.74	45.61449	19547.39	45.89351	19549.11	45.61759	19539.76	45.61055	19542.79	45.61474	19541.65	51.67651
45.86849	19547.76	45.86438	19547.58	46.13931	19549.75	45.86397	19539.87	45.86153	19542.55	45.86771	19543.01	51.93696
46.12256	19548.07	46.12377	19548.68	46.39132	19548.42	46.11088	19542.27	46.1113	19540.92	46.11522	19543.25	52.17687
46.36986	19548.21	46.3658	19548.33	46.64084	19548.62	46.36053	19541.39	46.36022	19542.68	46.37932	19541.56	52.42314
46.61757	19548.35	46.61619	19546.89	46.89343	19551.22	46.61686	19538.27	46.61055	19540.48	46.61564	19541.2	52.67567
46.86832	19547.22	46.86477	19545.87	47.14047	19549.17	46.86377	19541.79	46.86131	19543.01	46.8682	19542.56	52.92376
47.12256	19548.03	47.12196	19547.83	47.39275	19549.32	47.11059	19541.63	47.11278	19540.85	47.11521	19544.22	53.17468
47.36552	19547.55	47.36549	19545.95	47.63981	19551.18	47.36189	19540.5	47.36257	19543.73	47.37798	19542.44	53.42245
47.62121	19548.08	47.617	19548.05	47.89153	19548.57	47.61736	19539.41	47.61252	19542.04	47.61642	19543.18	53.6746
47.87024	19548.89	47.86488	19549.33	48.14021	19549.41	47.86533	19540.95	47.86099	19541.04	47.86934	19544.4	53.92683
48.12049	19549.57	48.12131	19547.51	48.39402	19550.35	48.11107	19541.4	48.11151	19541.34	48.11676	19543.67	54.1772
48.36935	19547.29	48.366	19546.59	48.64035	19548.45	48.36035	19542.16	48.3632	19540.1	48.37866	19545.62	54.42297
48.61894	19546.24	48.61608	19549.46	48.89029	19549.21	48.61668	19543.63	48.61289	19541.94	48.61664	19540.39	54.67659
48.86915	19549.5	48.86552	19546.91	49.1456	19548.74	48.86333	19541.1	48.86032	19540.95	48.86882	19539.92	54.92486
49.12206	19545.9	49.1229	19551.64	49.39248	19548.53	49.11272	19538.42	49.10968	19539.92	49.11528	19541.3	55.17487
49.36893	19546.98	49.36434	19548.23	49.64184	19548.41	49.36804	19540.53	49.36124	19540.39	49.37233	19541.03	55.42255
49.62384	19548.39	49.61664	19545.6	49.89604	19550.15	49.62232	19541.33	49.61504	19541.69	49.61769	19539.49	55.69625
49.87067	19548.79	49.86709	19545.91	50.13999	19549.41	49.86567	19539.55	49.86123	19542.22	49.8695	19541.52	55.92698
50.12245	19549.24	50.12209	19546.7	50.39106	19548.55	50.12016	19543.97	50.11009	19542.9	50.11677	19542.12	56.17501
50.36932	19546.97	50.3659	19547.37	50.64151	19548.01	50.36061	19538.62	50.36243	19544.04	50.37352	19539.53	56.42235
50.61588	19547.52	50.61439	19548.31	50.89308	19550.34	50.6156	19541.98	50.6121	19538.72	50.61492	19542.71	56.67886
50.87045	19546.73	50.8654	19549.6	51.14065	19545.79	50.86273	19540.86	50.86195	19541.03	50.86886	19540.73	56.92762
51.12134	19549.56	51.12309	19547.34	51.3909	19549.41	51.11145	19543.2	51.11123	19541.37	51.11796	19541.18	57.17531

51.37008	19547.51	51.3665	19548.61	51.64151	19547.82	51.36355	19541.28	51.36055	19540.95	51.37832	19543.64	57.42141
51.61371	19546.84	51.61563	19548.56	51.89374	19552.66	51.61818	19543.26	51.61174	19542.25	51.61601	19541.43	57.68
51.871	19548.17	51.86547	19547.37	52.14095	19550.52	51.86199	19540.16	51.86232	19544.46	51.87043	19542.14	57.93299
52.12146	19545.91	52.12207	19550.31	52.40148	19548.24	52.11206	19544.13	52.112	19539.22	52.11623	19542.45	58.17905
52.37122	19547.05	52.36715	19548.36	52.64145	19548.78	52.36386	19541.56	52.36142	19541.06	52.37963	19544.6	58.42423
52.62137	19545.22	52.61644	19550.43	52.89185	19548.76	52.61843	19542.59	52.61209	19539.96	52.61719	19541.5	58.67728
52.87049	19547.6	52.86562	19546.9	53.14206	19551.81	52.8661	19542.24	52.86211	19541.14	52.86959	19545.31	58.94067
53.12315	19546.32	53.12182	19548.07	53.39263	19546.73	53.11184	19541.59	53.11347	19542.63	53.11627	19544.22	59.18049
53.3714	19547.24	53.36706	19546.86	53.64183	19548.55	53.3613	19540.95	53.36159	19541.04	53.37839	19543.75	59.424
53.62092	19551.22	53.61759	19548.08	53.89292	19548.35	53.61723	19539.16	53.61414	19542.12	53.61816	19540.5	59.67877
53.87006	19544.36	53.8658	19549.32	54.142	19549.75	53.86371	19539.26	53.86271	19541.88	53.87121	19543.23	59.93049
54.12988	19547.99	54.12381	19547.83	54.39348	19546.49	54.11233	19538.85	54.11449	19540.16	54.11826	19544.36	60.17549
54.37213	19548.64	54.36801	19549.2	54.64082	19545.09	54.36258	19539.52	54.36244	19542.32	54.37912	19541.96	60.42548
54.61944	19546.41	54.61811	19547.47	54.89314	19550.65	54.61814	19541.73	54.61179	19541.56	54.61668	19542.28	60.67997
54.87225	19548.14	54.86698	19548.1	55.14157	19551.85	54.86534	19540.07	54.86355	19545.23	54.87188	19542.41	60.93057
55.1239	19549.27	55.12323	19547.72	55.39521	19547.33	55.11317	19541.8	55.11504	19541.8	55.11818	19539.23	61.17914
55.36648	19548.46	55.36815	19547.21	55.64213	19549.69	55.36273	19539.7	55.36255	19542.69	55.37632	19541.87	61.42389
55.62471	19546.41	55.61955	19549.5	55.89205	19547.78	55.6133	19539.21	55.6128	19541.34	55.61693	19541.24	61.68062
55.8653	19550.46	55.86925	19547.01	56.14255	19548.11	55.86545	19540.48	55.86236	19540.37	55.87077	19543.84	61.92961
56.11899	19548.46	56.12613	19547.53	56.39519	19549.51	56.11413	19538.23	56.11515	19542.97	56.11877	19541.54	62.17935
56.36372	19546.16	56.36652	19548.03	56.64485	19548.75	56.36434	19541.79	56.36426	19542.59	56.38186	19539.41	62.42742
56.61742	19547.36	56.61895	19550.78	56.89605	19547.35	56.61735	19542	56.61389	19541.94	56.61792	19543	62.68386
56.86908	19546.79	56.86861	19547.98	57.14157	19550.69	56.86433	19541.58	56.86211	19540.36	56.87083	19541.92	62.93041
57.12108	19546.85	57.1252	19548.18	57.39398	19548.15	57.11316	19540.68	57.11429	19544.48	57.11867	19541.95	63.17971
57.37001	19546.39	57.36744	19547.8	57.64433	19546.99	57.36497	19541.56	57.36409	19540.1	57.3804	19544	63.42699
57.61457	19547.72	57.61946	19548.3	57.89671	19551.03	57.61754	19540.29	57.6146	19541	57.61988	19541.56	63.6831
57.86625	19545.98	57.86859	19547.72	58.14366	19549.49	57.86732	19539.9	57.86338	19541.89	57.87224	19542.82	63.93186
58.12093	19547.31	58.12815	19547.18	58.39384	19547.2	58.11352	19540.67	58.11427	19540.4	58.11707	19542.91	64.18
58.3696	19544.55	58.37578	19550.44	58.64268	19550.44	58.36466	19541.81	58.36361	19542.62	58.37839	19540.51	64.42734
58.62972	19546.98	58.61872	19549.31	58.89638	19549.16	58.61557	19540.4	58.61773	19542.17	58.61948	19541	64.68349
58.86597	19550.06	58.86744	19547.48	59.14391	19548.58	58.86593	19541.92	58.86394	19540.72	58.87258	19543.02	64.93157
59.12356	19547.86	59.12414	19546.61	59.39669	19547.91	59.11507	19539.37	59.11436	19539.71	59.11866	19542.72	65.17753
59.36649	19547.61	59.36841	19547.61	59.6423	19548.66	59.36422	19541.66	59.3643	19541.43	59.38088	19540.95	65.42562
59.61777	19545.55	59.62158	19549.33	59.89626	19548.67	59.61607	19539.56	59.6159	19540.26	59.62059	19543.88	65.68214
59.86951	19547.26	59.86915	19549.16	60.15084	19548.6	59.86656	19541.87	59.86597	19541.45	59.87344	19541.3	65.93332
60.12592	19548.52	60.12508	19549.47	60.39683	19546.82	60.11538	19542.07	60.11538	19542.77	60.12022	19542.58	66.17814
60.37095	19548.75	60.36988	19547.48	60.64431	19548.18	60.36689	19540.21	60.36522	19539.69	60.37939	19542.67	66.42983
60.62515	19547.3	60.62034	19547.99	60.89728	19548.57	60.61734	19541.62	60.61564	19540.75	60.62165	19542.22	66.68333
60.872	19548.54	60.87177	19549.58	61.14594	19549.07	60.86864	19542	60.86772	19542.11	60.87407	19539.7	66.9339
61.12489	19546.74	61.13151	19547.04	61.39825	19549.31	61.11655	19542.14	61.11793	19541.3	61.12118	19543.63	67.18008
61.3708	19547.47	61.37093	19549.15	61.64652	19549.45	61.36819	19541.74	61.36572	19538.24	61.3823	19540.84	67.42533
61.62308	19547.81	61.62106	19547.66	61.89661	19547.84	61.61752	19539.92	61.61551	19539.91	61.62218	19542.49	67.68433
61.86711	19548.1	61.87109	19548.33	62.14607	19548.07	61.86803	19542.98	61.86593	19541.8	61.87439	19542.31	67.93501
62.12167	19546.76	62.12989	19548.26	62.39802	19551	62.11736	19539.98	62.11793	19541.17	62.12134	19543.56	68.18007
62.3683	19546.02	62.37228	19546.36	62.64648	19548.15	62.3677	19538.56	62.36763	19540.08	62.38119	19542.71	68.42682
62.61922	19546.85	62.62143	19547.11	62.89893	19548.67	62.61895	19541.23	62.61758	19541.54	62.62239	19541.97	68.68234
62.86911	19548.29	62.87042	19546.35	63.14509	19547.85	62.86783	19542.9	62.86677	19544.15	62.875	19542.21	68.93436
63.12189	19549.43	63.12663	19549.16	63.39612	19548.2	63.11613	19543.08	63.1169	19542.45	63.12658	19545	69.18075
63.36853	19548.11	63.37571	19548.89	63.64653	19548.49	63.36793	19542.05	63.36739	19540.14	63.38424	19541.97	69.42809
63.62182	19547.84	63.62286	19547.36	63.89913	19547.65	63.62131	19539.46	63.62087	19539.68	63.62356	19541.31	69.68463
63.86975	19547.41	63.87289	19546.88	64.14633	19548.81	63.86858	19538.84	63.86835	19541.58	63.87573	19542.02	69.93321
64.12644	19545.98	64.13395	19548.81	64.39616	19548.35	64.11643	19540.47	64.11701	19542.64	64.12206	19541.73	70.18058
64.36749	19549.97	64.37108	19549.4	64.64535	19548.36	64.36876	19539.86	64.36645	19540.86	64.38343	19542.94	70.42742
64.61732	19548.02	64.62045	19548.03	64.89798	19549.23	64.62201	19541.75	64.61724	19538.14	64.62195	19542.24	70.68274
64.86884	19546.71	64.87644	19547.97	65.14663	19549.5	64.86869	19540.52	64.86753	19541.12	64.87578	19541.11	70.93443
65.12327	19548.92	65.12645	19547.49	65.39783	19550.37	65.1164	19540.98	65.11592	19542.04	65.12032	19541.25	71.18079
65.36724	19548.24	65.37056	19545.75	65.64507	19547.88	65.36747	19539.56	65.36501	19541.71	65.3816	19540.58	71.42762
65.61767	19549.1	65.62321	19545.02	65.8974	19547.02	65.61814	19540.86	65.61667	19541.48	65.62144	19541.9	71.68311
65.86828	19547.21	65.87034	19547.32	66.14647	19546.31	65.87009	19539.12	65.86693	19541.13	65.87639	19544.71	71.93681
66.12269	19549.08	66.13011	19548.49	66.39887	19548.22	66.11716	19538.94	66.11648	19541.07	66.12178	19541.17	72.18181
66.36873	19546.11	66.37386	19546.8	66.64494	19552.39	66.36572	19540.39	66.36545	19541.22	66.38387	19543.83	72.42774
66.61688	19545.5	66.62418	19547.74	66.89698	19547.11	66.62053	19540.74	66.6159	19541.26	66.62097	19540.98	72.68571
66.86781	19547.24	66.87048	19545.58	67.14541	19548.3	66.87036	19541.98	66.86755	19541.78	66.87664	19541.15	72.93423
67.12528	19548.14	67.12801	19545.81	67.39911	19547.42	67.11849	19541.28	67.11937	19542.04	67.1223	19543.33	73.18197
67.36862	19547.88	67.37658	19548.21	67.64796	19549.14	67.36697	19540.7	67.36726	19540.18	67.38252	19543.19	73.42883
67.61864	19546.89	67.62189	19545.33	67.89633	19546.9	67.61861	19539.18	67.61664	19540.58	67.62066	19543.37	73.68301
67.86882	19547.25	67.87104	19549.65	68.14789	19548.33	67.87027	19541.51	67.8665	19540.94	67.87448	19541.86	73.93702
68.12344	19548.89	68.13428	19546.96	68.39735	19547.77	68.11842	19541.28	68.11853	19541.69	68.12384	19542.72	74.18148
68.36967	19548.13	68.37223	19548.83	68.6476	19551.02	68.36927	19538.87	68.36881	19541.88	68.38241	19540.76	74.42926
68.61958	19549.46	68.62254	19548.27	68.8988	19545.01	68.62075	19539.35	68.62106	19539.49	68.62189	19542.45	74.68441

68.8679	19547.23	68.87646	19548.79	69.14536	19548.35	68.86843	19538.75	68.86608	19542.13	68.8763	19541.86	74.93584
69.12379	19548.41	69.12722	19547.34	69.39968	19546.06	69.1178	19539.81	69.11707	19539.05	69.1234	19541.07	75.18332
69.36815	19548.93	69.37143	19545.97	69.64717	19548.97	69.36981	19543.22	69.36685	19542.38	69.38087	19540.33	75.4294
69.61953	19548.97	69.62523	19548.12	69.89959	19546.31	69.62065	19540.71	69.61871	19541.56	69.62212	19540.92	75.68445
69.87004	19548.31	69.87381	19546.97	70.15285	19547.27	69.87	19541.67	69.86892	19544.3	69.87644	19541.92	75.93975
70.12785	19546.88	70.13788	19548.54	70.39767	19550.01	70.11764	19540.29	70.12651	19542.21	70.12226	19543.21	76.18315
70.36839	19546.59	70.38985	19549.71	70.64712	19550.62	70.3683	19541.45	70.36712	19543.95	70.38323	19540.53	76.42926
70.61798	19546.08	70.62909	19546.98	70.90047	19545.92	70.62297	19541.51	70.61848	19540.93	70.6238	19540.35	76.68565
70.87093	19547.71	70.89304	19547.17	71.14786	19550.04	70.87035	19540.37	70.86939	19540.87	70.87704	19542.57	76.9384
71.12496	19546.59	71.13505	19549.03	71.39919	19549.01	71.12	19538.98	71.11869	19540.23	71.12207	19542.17	77.18409
71.36984	19548.15	71.37277	19548.94	71.64778	19548.27	71.36867	19541.79	71.3672	19542.61	71.38202	19542.96	77.42893
71.61826	19547.81	71.62299	19548.16	71.90092	19549.05	71.6198	19539.86	71.61884	19539.28	71.62312	19540.25	77.69279
71.86906	19547.84	71.87381	19547.84	72.14807	19547.13	71.87116	19539.72	71.86866	19542.07	71.87958	19539.31	77.93887
72.12385	19550.28	72.12964	19547.03	72.40001	19548.78	72.11817	19541.28	72.11871	19541.63	72.12437	19541.65	78.18467
72.37371	19547.31	72.37357	19546.82	72.64692	19550.32	72.36963	19543.14	72.36818	19541.1	72.3843	19542.23	78.4305
72.6258	19545.6	72.62226	19547.46	72.90227	19550.72	72.61957	19542.03	72.61953	19543.21	72.62269	19542.86	78.68394
72.86866	19548.34	72.87285	19548.68	73.14898	19547.06	72.87373	19541.37	72.86997	19543.99	72.87794	19543.77	78.93706
73.12589	19548.07	73.12967	19547.15	73.39989	19549.16	73.11984	19539.65	73.12	19538.22	73.12355	19540.62	79.18518
73.37095	19549.01	73.3738	19546.09	73.64903	19548.22	73.36854	19542.44	73.37003	19542.96	73.38634	19540.2	79.43207
73.62169	19549.86	73.62466	19550.31	73.90156	19549.24	73.6182	19539.42	73.62173	19541.27	73.62515	19543.89	79.68782
73.8706	19547.21	73.87228	19548.12	74.15297	19549.25	73.87274	19542.05	73.86822	19541.63	73.87784	19541.41	79.93752
74.12669	19547.44	74.12934	19549.38	74.40063	19548.95	74.12023	19541.13	74.11907	19541.6	74.12461	19540.55	80.18475
74.37091	19546.42	74.3754	19545.57	74.64965	19546.33	74.37078	19540.62	74.37105	19542.78	74.38457	19545.69	80.43368
74.62125	19545.31	74.62502	19548	74.90128	19550.06	74.61868	19540.89	74.62166	19540.38	74.62523	19540.78	80.68932
74.87244	19548.23	74.87604	19548.06	75.14834	19550.62	74.87271	19537.74	74.86798	19541.16	74.87797	19540.06	80.93725
75.12683	19547.34	75.13407	19549.6	75.40023	19549.6	75.12044	19540.75	75.11843	19541.81	75.12348	19541.23	81.18531
75.37091	19549.2	75.37519	19546.89	75.64944	19547.65	75.37291	19537.33	75.36988	19542.63	75.38518	19538.39	81.4325
75.6215	19545.53	75.62621	19550.51	75.90253	19550.73	75.6196	19540.4	75.62157	19541.46	75.626	19540.17	81.68689
75.8721	19549.12	75.87635	19549.63	76.14867	19546.65	75.87269	19538.16	75.87015	19539.2	75.8791	19541.38	81.93873
76.12722	19546.29	76.13339	19547.41	76.4	19547.99	76.11928	19539.97	76.11855	19543.81	76.12481	19541.2	82.18602
76.37155	19547.14	76.38066	19544.91	76.64953	19547.71	76.37183	19540.85	76.3709	19541.95	76.38578	19543.37	82.43188
76.62157	19544.77	76.62729	19549.51	76.90147	19545.44	76.62106	19540.44	76.62209	19543.38	76.62484	19543.29	82.68926
76.87259	19547.68	76.88501	19545.73	77.14975	19547.23	76.87137	19540.63	76.87092	19539.14	76.87926	19541.98	82.93899
77.12936	19547.3	77.13205	19546.98	77.39893	19547.99	77.12046	19539.39	77.11998	19543.17	77.12694	19541.74	83.18592
77.37064	19547.18	77.37485	19547.46	77.64895	19550.92	77.37341	19541.33	77.37041	19540.8	77.38533	19542.51	83.43099
77.62191	19546.57	77.62478	19549.22	77.902	19552.39	77.62275	19540.69	77.62159	19541.93	77.62427	19543.88	83.68791
77.8719	19548.17	77.87721	19546.14	78.15163	19549.86	77.87444	19539.26	77.87144	19544.41	77.88064	19542.12	83.93829
78.12842	19546.84	78.1336	19548.53	78.40327	19545.64	78.12002	19541.05	78.1232	19540.63	78.12678	19542.47	84.18691
78.37307	19551.24	78.37817	19546.35	78.65069	19547.61	78.37432	19541.33	78.37301	19542.43	78.38688	19542.97	84.43295
78.62046	19547.06	78.63432	19545.2	78.90082	19549.92	78.6238	19540.39	78.62442	19542.22	78.62584	19543.5	84.68874
78.8712	19549.71	78.87799	19548.2	79.14995	19550.71	78.872	19541.45	78.86894	19541.1	78.88063	19541.89	84.93847
79.12978	19547.8	79.13353	19547.83	79.42518	19547.61	79.11971	19539.44	79.12017	19541.05	79.12654	19543.32	85.18744
79.37298	19548.91	79.37736	19548.71	79.65112	19546.56	79.37144	19543.36	79.37216	19543.43	79.38323	19541.68	85.43355
79.62388	19550	79.63607	19546.41	79.90315	19548.73	79.62377	19539.95	79.6224	19542.53	79.62897	19542.17	85.68845
79.87157	19548.31	79.87886	19545.75	80.14978	19546.72	79.87486	19541.3	79.87107	19542.9	79.87932	19540.28	85.9391
80.12721	19546.19	80.1329	19547.6	80.40311	19546.87	80.12298	19538.81	80.12039	19541.63	80.1257	19542.35	86.19297
80.37559	19545.35	80.3804	19550.17	80.65079	19547.97	80.37286	19540.44	80.37377	19542.81	80.40699	19542.02	86.43316
80.62342	19547.82	80.62745	19548.65	80.90425	19547.76	80.62145	19540.07	80.62283	19541.51	80.62737	19542.59	86.69081
80.87195	19548.25	80.88407	19548.52	81.15044	19546.91	80.87587	19539.74	80.87171	19540.19	80.88404	19543.15	86.94157
81.12776	19547.4	81.1367	19549.04	81.40062	19549.57	81.12366	19540.13	81.12093	19543.58	81.12549	19541.83	87.18744
81.37281	19548.25	81.37951	19546.89	81.65071	19548.34	81.37538	19540.35	81.37213	19543.22	81.38763	19537.44	87.43322
81.62383	19548.93	81.62665	19547.32	81.90333	19548.63	81.62048	19541.39	81.62491	19542.67	81.62721	19541.63	87.68951
81.87363	19546.9	81.8782	19546.76	82.15193	19547.89	81.87515	19537.16	81.87315	19541.27	81.88392	19541.03	87.94157
82.12909	19546.06	82.15107	19549.08	82.40201	19549.74	82.12245	19541.9	82.12188	19542.18	82.12924	19542.67	88.19019
82.37379	19547.76	82.39451	19546.88	82.65066	19547.87	82.37378	19541.44	82.37193	19540.15	82.38388	19537.99	88.43431
82.62467	19546.84	82.62702	19546.86	82.90215	19547.87	82.62383	19539.63	82.62257	19541.65	82.62541	19541.06	88.691
82.87446	19547.19	82.88274	19544.74	83.15142	19549.13	82.87572	19542.31	82.87294	19541.68	82.88262	19542.07	88.94348
83.12945	19545.27	83.13184	19546.33	83.40345	19547.18	83.12122	19539.99	83.12389	19539.49	83.12818	19543.1	89.19028
83.37386	19546.85	83.38606	19547.64	83.64952	19548.41	83.37491	19540.87	83.37207	19539.62	83.38886	19542.5	89.43402
83.62324	19546.37	83.62634	19549.31	83.90429	19550.67	83.62482	19539.95	83.62517	19538.54	83.62671	19541.56	89.68989
83.87489	19548.96	83.87685	19546.64	84.1514	19548.12	83.87631	19538.93	83.8722	19538.69	83.883	19541.21	89.9413
84.14323	19547.05	84.13367	19549.01	84.40376	19548.41	84.12137	19538.94	84.12413	19541.21	84.12717	19539.91	90.19018
84.38169	19546.67	84.38062	19545.94	84.65327	19547.73	84.37378	19541.51	84.3743	19541.82	84.38745	19541.53	90.43568
84.62293	19548.08	84.62924	19547.02	84.90183	19545.89	84.62512	19540.18	84.62371	19542.47	84.63239	19542.17	90.68996
84.87341	19549.03	84.8767	19547.39	85.15194	19549.68	84.89653	19541.71	84.87319	19541.13	84.88205	19540.14	90.94206
85.1314	19546.64	85.13376	19548.68	85.40568	19546	85.12293	19540.87	85.12424	19540.77	85.1268	19542.22	91.19057
85.37799	19547.82	85.37834	19546.76	85.65331	19546.95	85.37586	19538.74	85.37496	19539.73	85.38472	19539.78	91.43543
85.62455	19547.39	85.62869	19549.21	85.90474	19547.65	85.62358	19538.94	85.62531	19540.43	85.62946	19540.75	91.69097
85.8732	19546.53	85.87803	19548.41	86.15124	19547.45	85.87855	19541.83	85.87204	19539.71	85.88746	19541.22	91.94193
86.13113	19549.29	86.13407	19545.45	86.40281	19545.98	86.1238	19538.68	86.12368	19540.88	86.1375	19542.08	92.18984

86.37733	19543.59	86.37887	19547.39	86.65406	19547.94	86.37596	19540.08	86.3746	19543.3	86.38791	19543.86	92.43669
86.6247	19547.29	86.62818	19547.76	86.9055	19548.57	86.62272	19537.68	86.62568	19542.05	86.62951	19540.62	92.69493
86.8742	19545.24	86.8795	19547.74	87.15495	19548.01	86.87869	19539.72	86.8737	19539.34	86.88323	19540.43	92.94287
87.13222	19549.14	87.13171	19546.87	87.4031	19545.39	87.12377	19541.46	87.1233	19539.7	87.12808	19541.28	93.19151
87.37754	19548.97	87.378	19546.91	87.65334	19547.94	87.37625	19537.4	87.37408	19539.03	87.38807	19541.39	93.43582
87.6263	19546.23	87.6291	19548.84	87.90674	19548.38	87.62308	19539.11	87.62517	19541	87.63014	19543.24	93.69118
87.87505	19547.71	87.87983	19545.55	88.15445	19549.36	87.89947	19536.79	87.87586	19541.59	87.8846	19539.87	93.94474
88.13208	19547.42	88.13373	19546.92	88.40427	19547.94	88.12513	19538.73	88.12359	19542.22	88.12778	19541.57	94.19087
88.37843	19546.16	88.37808	19545.49	88.65298	19545.84	88.38401	19542.86	88.37398	19541.13	88.38695	19540.71	94.43636
88.62733	19550.12	88.62816	19548.56	88.9136	19546.37	88.63133	19538.21	88.62893	19541.62	88.63029	19540.9	94.69466
88.87854	19548.44	88.87914	19548.67	89.15501	19546.69	88.8775	19539.95	88.8752	19538.65	88.88525	19542.76	94.94443
89.13532	19545.29	89.13884	19546.11	89.40563	19544.89	89.12437	19538.33	89.12426	19540.55	89.12943	19539.67	95.19062
89.37685	19545.68	89.38593	19548.1	89.65418	19547.26	89.38081	19538.94	89.3731	19541.75	89.38992	19537.55	95.43764
89.63007	19547.51	89.633	19546.19	89.90623	19548.69	89.62614	19540.29	89.6242	19540.65	89.62993	19540.64	95.69383
89.8759	19545.56	89.87853	19545.95	90.15574	19545.76	89.8803	19538.81	89.8751	19543.02	89.88579	19540.79	95.94532
90.13182	19544.49	90.1353	19545.99	90.4069	19547.14	90.12494	19540.7	90.12638	19541.59	90.131	19538.83	96.19159
90.37747	19546.15	90.38113	19544.2	90.6534	19545.81	90.37922	19539.46	90.37509	19539.3	90.39173	19540.61	96.43694
90.62623	19546.23	90.63159	19547.87	90.90643	19545.59	90.62652	19540.07	90.6233	19539.58	90.63004	19539.79	96.69425
90.87741	19544.32	90.8784	19549.4	91.15471	19547.88	90.87982	19540.74	90.87464	19540.2	90.88382	19540.78	96.94505
91.13341	19546.35	91.13706	19547.92	91.40605	19546.68	91.12466	19539.62	91.12545	19538.04	91.13104	19539.45	97.19059
91.37771	19545.31	91.38058	19545.94	91.65537	19545.08	91.38008	19543.24	91.37686	19539.92	91.39098	19540.25	97.43679
91.62752	19545.73	91.63207	19547	91.90525	19545.96	91.62627	19540.48	91.62496	19539.61	91.6314	19541.45	97.69568
91.87859	19545.03	91.88247	19548.37	92.15532	19546.42	91.87905	19541.34	91.87354	19540.56	91.8872	19539.03	97.94413
92.13501	19547.4	92.13826	19544.71	92.40766	19549.45	92.12419	19537.51	92.12553	19539.59	92.13008	19540.41	98.19175
92.3819	19545.57	92.38009	19544.9	92.65521	19544.22	92.37806	19537.28	92.37601	19538.55	92.38917	19538.56	98.4378
92.62852	19544.91	92.63018	19546.91	92.90713	19546.75	92.62622	19538.08	92.62711	19540.21	92.63158	19539.91	98.70359
92.87766	19544.13	92.88114	19543.36	93.15467	19545.27	92.88233	19540.3	92.8774	19540.31	92.88553	19539.34	98.94479
93.13517	19545.35	93.13537	19546.77	93.40814	19546.66	93.12793	19540.25	93.13291	19541.6	93.13017	19539.99	99.19284
93.37836	19546.04	93.37947	19548.69	93.65811	19545.53	93.37755	19540.08	93.37696	19540.74	93.3907	19538.23	99.43971
93.62743	19545.79	93.63017	19544.88	93.90948	19547.56	93.62601	19538.35	93.63136	19537.64	93.63209	19540.49	99.69699
93.87868	19548.24	93.88087	19548.88	94.1554	19544.61	93.88221	19538.84	93.87635	19538.13	93.88648	19540.39	99.94407
94.1356	19545.26	94.13698	19546.95	94.40545	19544.74	94.13365	19538.34	94.12457	19541.15	94.13047	19542.1	100.1926
94.38006	19542.26	94.38347	19544.9	94.65676	19547.44	94.38025	19540.55	94.37673	19539.53	94.39235	19539.26	100.4413
94.62941	19544.99	94.63061	19545.11	94.90967	19544.38	94.62653	19540.11	94.62788	19541.37	94.63263	19537.43	100.7
94.87784	19545.15	94.88062	19544.94	95.15647	19546.04	94.8797	19538.43	94.87764	19537.12	94.88742	19537.46	100.9518
95.13386	19544.17	95.13727	19541.68	95.4063	19544.86	95.12628	19538.19	95.12612	19540.9	95.12976	19539.92	101.1982
95.3791	19545.72	95.38321	19545.72	95.65597	19545.27	95.37857	19537.1	95.3768	19540.16	95.39137	19538.57	101.4402
95.63066	19543.29	95.63324	19544.45	95.90894	19543.44	95.62704	19535.84	95.62865	19538.98	95.63276	19537.25	101.6991
95.87842	19545.01	95.87926	19545.58	96.15885	19546.77	95.88142	19537.48	95.87815	19540.73	95.88727	19538.03	101.948
96.13526	19544.8	96.1394	19545.6	96.40723	19545.59	96.12602	19538.71	96.12637	19539.45	96.13458	19540.33	102.1938
96.3782	19544.37	96.3819	19545.71	96.65511	19543.4	96.3785	19539.19	96.37662	19538.12	96.39013	19539.21	102.4427
96.62971	19546.26	96.63313	19545.57	96.90905	19544.87	96.62809	19539.29	96.64444	19538.95	96.63347	19537.58	102.6986
96.88103	19546.07	96.88156	19546.51	97.15799	19544.5	96.88054	19537.08	96.88034	19541.02	96.88853	19541.19	102.9496
97.13517	19545.86	97.13784	19545.85	97.40822	19547.21	97.12882	19540.31	97.12746	19540.03	97.13315	19538.37	103.1964
97.37909	19545	97.38211	19547.34	97.65992	19545.04	97.3802	19536.58	97.3809	19539.26	97.38845	19537.07	103.4428
97.63074	19546.77	97.63453	19546.25	97.9094	19544.98	97.62634	19538.58	97.63028	19541.56	97.63185	19537.62	103.7
97.88112	19544.2	97.88283	19542.78	98.15815	19545.49	97.88366	19536.53	97.87855	19539.75	97.88802	19538.32	103.9479
98.13663	19544.1	98.13907	19543.77	98.41096	19544.75	98.12842	19536.74	98.12885	19535.06	98.13452	19537.06	104.1959
98.37954	19545.19	98.38119	19544.57	98.65796	19542.18	98.38296	19537.26	98.3792	19541.18	98.39418	19539.99	104.4426
98.6286	19545.41	98.63333	19544.39	98.90909	19545.74	98.62937	19537.11	98.63517	19538.94	98.63298	19538.64	104.6993
98.87913	19545.74	98.88328	19544.99	99.15777	19544	98.88188	19539	98.87826	19536.19	98.8885	19540.05	104.9488
99.14522	19547.26	99.14422	19547.67	99.4156	19546.9	99.13372	19538	99.13226	19538.63	99.13884	19540.77	105.1998
99.38135	19544.51	99.38342	19543.49	99.65793	19545.05	99.38038	19537.09	99.37879	19538.29	99.39268	19538.07	105.4434
99.62986	19545.12	99.63164	19544.71	99.91176	19544.19	99.62884	19534.87	99.63148	19539.75	99.63454	19536.93	105.6999
99.87952	19544.96	99.88272	19543.43	100.1572	19543.6	99.88181	19538.5	99.8795	19537.82	99.88789	19536.37	105.9505
100.1382	19542.68	100.1393	19542.48	100.4103	19545.41	100.1281	19536.58	100.1272	19540.96	100.1319	19540.02	106.197
100.3834	19544.68	100.3867	19545.75	100.6607	19543.67	100.3832	19536.41	100.3804	19536.77	100.3925	19536.41	106.4426
100.6321	19543.96	100.6357	19543.59	100.9142	19544.38	100.6317	19536.18	100.6333	19538.19	100.6376	19537.56	106.701
100.8807	19544.36	100.8852	19544.66	101.1588	19546.9	100.8857	19536.34	100.8826	19540.46	100.8917	19539.65	106.9498
101.14	19542.62	101.1424	19544.9	101.4096	19543.26	101.1298	19535.91	101.1311	19537.27	101.1356	19536.08	107.1975
101.3824	19542.98	101.3856	19543.95	101.6637	19542.86	101.3828	19537.1	101.3787	19536.82	101.3938	19535.98	107.4428
101.6341	19540.45	101.6355	19543.79	101.9137	19545.63	101.6319	19536.84	101.6339	19536.3	101.6378	19537.53	107.7008
101.8814	19541.53	101.8856	19543.33	102.1617	19544.99	101.8869	19534.42	101.8826	19539.51	101.8924	19538.39	107.9511
102.1398	19541.4	102.143	19542.07	102.4138	19545.04	102.1308	19536.96	102.132	19535.42	102.1379	19537.66	108.1962
102.3825	19543.57	102.3866	19543.79	102.6582	19543.59	102.3818	19534.05	102.381	19536.74	102.3938	19538.19	108.4439
102.6335	19543.58	102.638	19541.32	102.9122	19543.48	102.6333	19535.5	102.6325	19538.91	102.6386	19536.7	108.7002
102.8831	19541.22	102.8862	19541.37	103.1616	19542.23	102.8875	19534.49	102.8826	19537.85	102.8938	19537.97	108.9497
103.1397	19541.55	103.1417	19544.48	103.4125	19542.18	103.1322	19536.78	103.1321	19538.48	103.1382	19538.46	109.1967
103.3829	19544.1	103.3865	19546.09	103.6605	19545.5	103.3895	19538.25	103.3819	19535.03	103.3947	19534.77	109.4452
103.6338	19544.01	103.6366	19542.28	103.912	19543.97	103.6322	19537.87	103.637	19537.44	103.6366	19535.11	109.7012

103.8848	19545.09	103.8868	19543.28	104.1598	19545.04	103.8871	19536.2	103.8812	19537.6	103.8922	19535.49	109.9671
104.1444	19545.39	104.1407	19540.44	104.4127	19540.44	104.1321	19537.42	104.1313	19538.38	104.138	19534.88	110.1987
104.3823	19543.33	104.3928	19542.96	104.6634	19542.91	104.3884	19535.13	104.3827	19537.35	104.3974	19535.98	110.4452
104.6327	19539.72	104.6369	19545.22	104.9135	19545.14	104.633	19534.51	104.6348	19536.54	104.6382	19536.88	110.7003
104.8849	19545.45	104.8877	19542.55	105.1603	19545.87	104.8862	19538.18	104.8821	19536.28	104.8922	19536.77	110.9518
105.1436	19544.18	105.1399	19541.52	105.4142	19544.32	105.1329	19536.46	105.1313	19536.09	105.1388	19536	111.1982
105.3849	19541.11	105.3871	19541.48	105.6658	19542.12	105.3866	19536.42	105.3816	19536.35	105.3954	19537.47	111.4447
105.6334	19544.09	105.6363	19540.73	105.9151	19545.55	105.6339	19537.68	105.635	19536.08	105.6392	19534.03	111.7012
105.8844	19542.25	105.8874	19544.8	106.1608	19541.29	105.8859	19536.65	105.8831	19539.08	105.8937	19537.77	111.9532
106.1421	19541.3	106.144	19542.73	106.413	19542.9	106.1322	19537.79	106.1314	19537.76	106.1384	19536.69	112.1995
106.3845	19542.62	106.3874	19541	106.6609	19545.01	106.3858	19537.37	106.3819	19537.61	106.398	19537.32	112.4452
106.6352	19542.12	106.6366	19543.73	106.9147	19541.47	106.6344	19535.72	106.6337	19536.55	106.6386	19536.26	112.7033
106.8841	19541.67	106.889	19542.39	107.1625	19543	106.8886	19534.41	106.8844	19535.94	106.8925	19535.2	112.9506
107.1441	19540.34	107.1449	19543.13	107.4116	19543.01	107.132	19535.74	107.1349	19534.95	107.1396	19537.19	113.2004
107.3857	19544.17	107.3895	19542.96	107.6607	19542.94	107.3851	19534.04	107.3818	19537.63	107.3989	19533.84	113.4451
107.6375	19543.68	107.6388	19541.42	107.914	19543.03	107.6343	19534.77	107.6345	19536.77	107.6395	19536.12	113.7022
107.8841	19542.46	107.891	19542.1	108.1637	19542.7	107.8873	19537.2	107.8858	19536.38	107.8957	19536.76	113.9544
108.1432	19541.2	108.1426	19544.79	108.4155	19541.84	108.1308	19538.3	108.1343	19536.63	108.1409	19537.25	114.2007
108.3861	19542.24	108.389	19542.88	108.6611	19544.11	108.3864	19537.15	108.3848	19535.63	108.3988	19534.05	114.4457
108.6387	19541.3	108.6395	19545.29	108.9139	19542.39	108.6348	19535.8	108.6379	19534.4	108.6376	19537.62	114.702
108.8847	19541.37	108.8879	19541.58	109.1634	19539.86	108.8881	19534.85	108.8842	19536.89	108.8935	19537.12	114.9517
109.1489	19541.99	109.1425	19543.03	109.4167	19543.31	109.1311	19534.57	109.1343	19538.27	109.1385	19534.81	115.2009
109.3864	19541.32	109.3885	19544.52	109.6639	19540.82	109.385	19533.46	109.3843	19536.24	109.3995	19535.65	115.4467
109.6388	19541.48	109.6388	19544.86	109.9149	19543.4	109.6355	19535.49	109.6378	19535.97	109.6392	19538.41	115.7033
109.8868	19541.76	109.8899	19543.5	110.1623	19542.95	109.8891	19537.78	109.8834	19535.75	109.8943	19534.4	115.9508
110.1431	19542.61	110.1439	19541.93	110.4144	19540.81	110.1342	19534.04	110.1344	19535.29	110.1381	19534.76	116.2009
110.3863	19539.37	110.3892	19541.05	110.6642	19542.44	110.3851	19535.55	110.3843	19536.86	110.396	19538.53	116.4463
110.6392	19543.84	110.6379	19541.33	110.9171	19543.33	110.6342	19536.16	110.6388	19532.44	110.6415	19534.72	116.7031
110.8921	19541.79	110.8892	19544.36	111.1619	19540.07	110.8914	19535.51	110.884	19533.56	110.8956	19536.35	116.9545
111.1447	19542.95	111.1484	19542.35	111.418	19540.98	111.1351	19536.48	111.1332	19535.53	111.1499	19535.24	117.2033
111.3862	19539.66	111.3906	19541.54	111.6645	19542.91	111.3875	19536.42	111.3845	19536.56	111.4039	19535.13	117.4464
111.6378	19540.68	111.6397	19542.79	111.9187	19540.04	111.6357	19535.3	111.6386	19536.84	111.6425	19536.48	117.705
111.8874	19541.08	111.8895	19542.59	112.1646	19543.33	111.8895	19533.46	111.8852	19535.47	111.8964	19534.8	118.3299
112.1458	19542.37	112.1427	19542.7	112.4166	19542.37	112.1347	19536.36	112.1349	19536.29	112.1404	19533.1	118.7692
112.387	19539.73	112.3908	19541.81	112.6637	19540.89	112.3874	19536.08	112.3852	19536.72	112.3973	19537.67	118.9995
112.6382	19543.2	112.6416	19540.75	112.9172	19541.88	112.6375	19533.75	112.6387	19534.49	112.6399	19535.79	119.2685
112.8881	19540.07	112.8916	19541.8	113.1653	19543.98	112.8904	19536.94	112.8866	19536.3	112.8958	19535.02	119.4945
113.1478	19539.16	113.1438	19540.84	113.4176	19544.38	113.1355	19534.66	113.1348	19534.13	113.1419	19534.93	119.7465
113.3964	19540.2	113.3898	19541.73	113.6642	19542.68	113.3885	19531.47	113.3846	19535.49	113.4034	19532.46	120.0004
113.6397	19543.69	113.6405	19539.26	113.9157	19542.73	113.6366	19534.66	113.6409	19533.55	113.6456	19535.26	120.2528
113.8889	19542.56	113.8932	19543.74	114.1651	19545.02	113.8892	19533.59	113.8864	19537.21	113.897	19537.01	120.4891
114.1507	19542.08	114.1451	19541.51	114.4181	19544.1	114.1342	19535.56	114.1358	19536	114.144	19534.04	120.7456
114.39	19538.45	114.3896	19541.98	114.666	19541.04	114.3877	19536.46	114.3851	19536.6	114.4015	19536.35	120.9966
114.6383	19542.65	114.6404	19543.09	114.917	19541.89	114.6366	19536.47	114.6363	19535.46	114.6438	19533.05	121.246
114.8879	19540.41	114.8922	19543.16	115.1633	19543.46	114.8914	19534.98	114.8879	19537.41	114.8953	19532.85	121.4915
115.1443	19538.32	115.1456	19542.79	115.4186	19542.11	115.1365	19534.93	115.1367	19533.99	115.1456	19534.07	121.7464
115.3873	19540.04	115.3918	19541.49	115.6658	19540.92	115.389	19532.57	115.3861	19535.11	115.4023	19537.06	121.9953
115.64	19540.39	115.6403	19541.6	115.9192	19539.77	115.6394	19535.06	115.6413	19536.06	115.6433	19532.04	122.2457
115.8885	19543.49	115.8908	19542	116.1652	19539.97	115.8907	19533.09	115.8859	19536.68	115.8965	19535.38	122.4902
116.1472	19539.11	116.1492	19540.51	116.4169	19538.28	116.1356	19532.78	116.1372	19533.8	116.1469	19536.51	122.7474
116.3874	19541.04	116.3927	19542.21	116.6658	19543	116.3895	19534.04	116.3871	19534.88	116.3993	19536.13	122.9995
116.6386	19541.24	116.644	19540.16	116.9191	19541.72	116.6354	19534.13	116.6407	19533.87	116.6557	19532.22	123.247
116.8899	19539.31	116.8905	19538.89	117.1655	19539.6	116.8904	19535.12	116.8875	19533.91	116.8971	19534.19	123.4888
117.1468	19540.83	117.1495	19541.99	117.4179	19540.9	117.138	19534.54	117.1354	19536.15	117.1406	19533.32	123.7463
117.389	19539.87	117.392	19541.1	117.6652	19541.26	117.3894	19535.06	117.3874	19535.62	117.3997	19534.04	124.0009
117.6391	19540.2	117.6426	19538.6	117.9193	19541.85	117.6378	19535.83	117.6404	19535.99	117.6568	19531.91	124.2456
117.8906	19538.97	117.8929	19539.48	118.1671	19541.33	117.8938	19536.25	117.8885	19536.81	117.8999	19535.28	124.4916
118.1483	19536.85	118.1455	19541.27	118.4186	19541.01	118.1383	19532.5	118.1358	19536.01	118.1429	19536.04	124.7477
118.3877	19539.4	118.3941	19540.98	118.665	19541.9	118.3921	19537.02	118.3863	19535.1	118.4005	19535.85	124.9967
118.6388	19539.68	118.6426	19542.15	118.9197	19540.57	118.6382	19533.18	118.6449	19534.33	118.6427	19535.75	125.2448
118.8904	19541.65	118.8929	19541.56	119.1681	19542.4	118.893	19533.64	118.8902	19535.85	118.8988	19535.05	125.4917
119.1508	19539.21	119.1436	19541.26	119.4204	19538.49	119.1371	19534.77	119.1392	19534.46	119.1553	19534.36	125.7471
119.3993	19540.97	119.3932	19539.89	119.6657	19542.16	119.3895	19533.21	119.3859	19534.47	119.4043	19534.38	125.9999
119.6384	19538.99	119.6438	19541.73	119.9187	19541.13	119.6382	19534.17	119.6399	19536.68	119.6443	19529.28	126.2458
119.8895	19540.51	119.8937	19538.95	120.1676	19538.6	119.893	19534.63	119.8914	19536.9	119.9001	19536.19	126.4908
120.1472	19543.05	120.1457	19540.49	120.4228	19540.75	120.1402	19532.95	120.1399	19536.36	120.1447	19535.67	126.7476
120.3922	19537.99	120.3935	19541.14	120.6647	19539.97	120.3905	19532.91	120.3893	19534.3	120.4049	19533.82	127.0013
120.638	19537.08	120.639	19539.11	120.9179	19540.15	120.6343	19532.57	120.6389	19535.75	120.6478	19533.34	127.2466
120.8865	19540.67	120.8913	19540.14	121.1644	19543.9	120.8919	19535.68	120.8887	19532.11	120.8984	19534	127.4954
121.1482	19539.23	121.1474	19544.49	121.4213	19543.09	121.1366	19536.26	121.1375	19535.35	121.1419	19534.82	127.7478

121.3889	19537.9	121.393	19539.44	121.6663	19540.81	121.391	19533.71	121.3865	19533.89	121.4005	19536.63	127.999
121.6399	19540.34	121.6424	19542.87	121.9186	19539.57	121.6349	19532.09	121.6395	19535.04	121.6433	19535.5	128.2462
121.8884	19539.41	121.8896	19538.57	122.1671	19540.43	121.8917	19533.49	121.8886	19533.02	121.9058	19536.4	128.4911
122.1457	19540.8	122.1473	19540.61	122.4183	19540.91	122.1364	19533.74	122.1375	19535.43	122.1416	19537.66	128.7477
122.3909	19541.12	122.3942	19537.34	122.6663	19538.33	122.3894	19533.11	122.3875	19532.83	122.3992	19534.59	128.9999
122.6395	19538.35	122.6429	19541.57	122.9185	19540.49	122.636	19533.98	122.6427	19537.05	122.6471	19536.37	129.2479
122.8887	19538.16	122.8906	19540.12	123.1653	19537.93	122.8916	19535.39	122.8893	19532.98	122.9022	19532.04	129.4917
123.1465	19541.36	123.144	19541.1	123.4193	19542.63	123.1363	19533.12	123.1389	19536.4	123.1418	19532.48	129.748
123.3898	19538.39	123.3936	19538.62	123.6685	19540.75	123.3901	19534.27	123.3868	19533.82	123.4006	19534.05	129.9983
123.6399	19537.43	123.6447	19542.27	123.924	19541.14	123.6384	19532.33	123.6453	19536.32	123.6427	19537.15	130.2482
123.8904	19538.76	123.8952	19542.37	124.1666	19539.5	123.8924	19535.09	123.8896	19533.58	123.8979	19532.75	130.4948
124.1524	19538.71	124.1445	19541.21	124.4183	19542.48	124.1358	19533.28	124.1377	19532.92	124.1428	19533.91	130.7494
124.3897	19538.91	124.3926	19541.47	124.6679	19539.53	124.3905	19533.12	124.3854	19534.22	124.3997	19534.57	131.0013
124.6398	19537.59	124.6432	19539.91	124.9216	19542.01	124.6376	19534.69	124.6443	19534.25	124.6465	19534.53	131.2505
124.8922	19537.92	124.893	19539.49	125.1673	19538.47	124.8919	19533.26	124.8907	19532.82	124.9	19537.18	131.4936
125.1479	19538.82	125.1472	19539.71	125.4204	19538.61	125.1357	19533.74	125.1377	19534.8	125.1444	19534.98	131.7473
125.3901	19539.11	125.3932	19538.78	125.6665	19539.83	125.388	19535.85	125.3999	19532	125.4013	19533.79	132.0052
125.642	19538.59	125.6411	19540.02	125.9202	19541.92	125.6386	19534.19	125.6485	19533.38	125.6437	19534.49	132.2503
125.8922	19539.82	125.8924	19540	126.1686	19540.61	125.8944	19534.69	125.8909	19534.36	125.8991	19534.41	132.495
126.1498	19539.02	126.1489	19538.68	126.4228	19543.01	126.1381	19532.46	126.1383	19534.13	126.1432	19534.11	132.751
126.3925	19539.73	126.3956	19540.57	126.6654	19541.91	126.3876	19531.47	126.3885	19533.97	126.4019	19533.12	132.9995
126.6398	19538.81	126.641	19538.43	126.9203	19537.01	126.6376	19532.5	126.6413	19531.73	126.6416	19535.98	133.2489
126.8926	19539.67	126.8923	19540.32	127.1684	19540.22	126.8941	19530.86	126.8878	19534.34	126.8991	19530.98	133.4948
127.1482	19541.04	127.1463	19540.33	127.423	19539.51	127.1388	19530.44	127.1397	19533.85	127.143	19532.71	133.751
127.3929	19540.34	127.3939	19537.43	127.6672	19540.4	127.3894	19532.57	127.3895	19531.41	127.4032	19533.24	134.0056
127.6393	19540.45	127.6444	19537.83	127.9192	19539.78	127.6375	19534.32	127.6465	19533.09	127.6442	19530.9	134.2516
127.8925	19537.66	127.8935	19539.1	128.1686	19539.46	127.8969	19534.17	127.8884	19532.67	127.9017	19532.69	134.4954
128.1494	19541.45	128.1468	19540.71	128.4234	19543.61	128.1397	19535.08	128.138	19534.37	128.1477	19535.12	134.7499
128.3931	19540.19	128.3937	19537.74	128.6689	19537.84	128.3936	19533.33	128.3886	19532.31	128.4033	19534.78	135.0009
128.6419	19536.58	128.6444	19539.28	128.9213	19538.93	128.6377	19531.86	128.646	19533.41	128.6451	19534.67	135.2511
128.8919	19537.87	128.8929	19538.31	129.1675	19540.74	128.8968	19530.6	128.8912	19534.51	128.8983	19532.86	135.498
129.1497	19537.95	129.1479	19540.41	129.4227	19538.7	129.1397	19533.3	129.1391	19533.38	129.1437	19532.7	135.755
129.3916	19539.4	129.3948	19537.05	129.6697	19540.01	129.3934	19531.73	129.3881	19530.82	129.4063	19533.67	136.0045
129.643	19539.42	129.6457	19538.77	129.92	19538.5	129.641	19532.46	129.6436	19530.04	129.6456	19530.45	136.2512
129.8936	19537.64	129.8947	19541.06	130.1675	19540.32	129.8965	19530.6	129.8906	19531.05	129.9004	19534.87	136.4979
130.1503	19537.59	130.1421	19538.62	130.4335	19539.87	130.1394	19533.55	130.1423	19535.73	130.1436	19533.03	136.7516
130.391	19537.78	130.3965	19541.22	130.6695	19539.36	130.3938	19532.49	130.4	19532.89	130.4006	19533.63	137.0024
130.6421	19538.9	130.6471	19539.81	130.9227	19540.65	130.6402	19532.95	130.6431	19529.81	130.6483	19531.3	137.2502
130.8957	19537.81	130.8948	19539.41	131.1669	19540.37	130.8981	19532.56	130.8907	19532.75	130.9002	19533.54	137.4968
131.1508	19536.72	131.1497	19539.09	131.4209	19539.91	131.1394	19530.64	131.1406	19532.79	131.1466	19532.58	137.7525
131.3923	19537.47	131.3939	19538.17	131.6699	19538.49	131.3927	19533.54	131.3913	19532.23	131.4031	19534.49	138.0041
131.6441	19539.05	131.6452	19541.29	131.9237	19540.58	131.6409	19531.76	131.6436	19532.29	131.6455	19533.32	138.2521
131.8945	19538.27	131.8953	19539.46	132.1696	19541.09	131.8985	19532.99	131.8898	19537.84	131.9027	19535.69	138.4961
132.1487	19540.39	132.1458	19538.56	132.4228	19540.03	132.1386	19532.68	132.1415	19530.92	132.1462	19534.17	138.7539
132.3921	19540.43	132.3957	19539.67	132.6696	19539.75	132.3918	19533.15	132.3911	19532.56	132.4031	19532.42	139.0019
132.6429	19537.72	132.6447	19539.25	132.9223	19540.22	132.6403	19529.69	132.6464	19535.64	132.645	19530.42	139.2511
132.8952	19536.27	132.8957	19539.67	133.1702	19538.22	132.8989	19532.18	132.8903	19532.58	132.9012	19531.61	139.4989
133.1505	19538.14	133.146	19538.41	133.4253	19540.75	133.1408	19533.95	133.1411	19530.11	133.1451	19531.95	139.7535
133.3919	19537.61	133.3965	19538.68	133.6701	19540.27	133.3935	19529.96	133.3906	19531.47	133.4047	19530.72	140.004
133.6421	19537.16	133.6458	19538.77	133.9228	19538.87	133.6387	19534.11	133.6498	19533.26	133.6457	19533.41	140.2606
133.8966	19537.42	133.8946	19538.14	134.1704	19537.26	133.8975	19531.21	133.8917	19533.49	133.9016	19533.78	140.4957
134.152	19539.04	134.1464	19538.44	134.4271	19540.16	134.1437	19533.21	134.139	19534.42	134.1476	19533.59	140.7521
134.3944	19538.1	134.3964	19538.48	134.6701	19539.57	134.392	19530.98	134.392	19533.91	134.4057	19532.47	141.0073
134.643	19538.04	134.6465	19539.58	134.9229	19540.53	134.6393	19532.57	134.6479	19532.06	134.6629	19536.1	141.2551
134.8948	19537.65	134.8955	19536.53	135.1704	19539.47	134.9002	19531.02	134.892	19532.8	134.9042	19529.4	141.4992
135.1534	19539.83	135.1483	19540.19	135.4441	19539.86	135.1417	19530.05	135.1407	19534.21	135.1458	19533.86	141.7544
135.3947	19536.32	135.3973	19538.68	135.6728	19538.87	135.3965	19534.56	135.3897	19533.36	135.4053	19533.57	142.0033
135.6443	19537.55	135.6473	19538.2	135.9234	19542.94	135.64	19534.45	135.6474	19531.56	135.6471	19534.42	142.2516
135.8933	19537.28	135.898	19538.55	136.1695	19540.36	135.8986	19529.27	135.8922	19532.09	135.9015	19534.04	142.498
136.1514	19537.96	136.1524	19537.55	136.4247	19537.31	136.1416	19532.19	136.1426	19530.16	136.1451	19534.12	142.7549
136.3953	19539.05	136.4107	19536.61	136.6729	19539.72	136.3937	19532.69	136.3918	19532.46	136.4031	19532.42	143.006
136.6492	19538.34	136.6512	19536.73	136.9237	19540.5	136.6409	19532.24	136.6458	19532.55	136.6476	19531.94	143.252
136.8978	19539.63	136.8992	19538.26	137.1698	19536.64	136.8976	19532.89	136.8927	19532.52	136.9104	19531.57	143.4969
137.1533	19538.2	137.148	19537.85	137.4245	19537.97	137.1424	19531.17	137.1425	19535.14	137.147	19531.06	143.7547
137.3943	19540.59	137.3968	19541.32	137.6731	19538.64	137.3953	19533.14	137.3927	19531.38	137.4035	19532.03	144.0048
137.6445	19536.75	137.6477	19539.04	137.9259	19539.56	137.6425	19532.76	137.6471	19531.23	137.6466	19531.73	144.2547
137.8961	19538.96	137.898	19538.3	138.1703	19538.09	137.8973	19531.52	137.8912	19532.48	137.9042	19531.08	144.4982
138.1533	19537.21	138.1505	19539.6	138.4291	19537.94	138.1434	19532.4	138.1429	19532.07	138.1482	19534.2	144.7536
138.3951	19538.88	138.3967	19538.07	138.674	19541.13	138.3983	19530.44	138.3921	19532.43	138.407	19532.01	145.0049
138.645	19538.24	138.6497	19537.21	138.9244	19537.97	138.6439	19531.64	138.6534	19535.01	138.6474	19532.41	145.253

138.8959	19540.36	138.8984	19538.6	139.1722	19540.39	138.9006	19531.47	138.893	19533.77	138.9045	19532.33	145.4989
139.1536	19537.96	139.1501	19537.94	139.4236	19538.93	139.1411	19532.83	139.1431	19531.07	139.15	19532.14	145.7553
139.3994	19536.69	139.3986	19538.92	139.6731	19538.43	139.3956	19533.25	139.3928	19536.2	139.4068	19534.15	146.0043
139.6449	19535.24	139.6476	19536.26	139.9238	19538.12	139.6434	19531.88	139.6493	19532.83	139.6498	19530.29	146.2543
139.8959	19538.41	139.8972	19539.26	140.1725	19538.82	139.8985	19533.06	139.8957	19529.63	139.9047	19530.35	146.4991
140.1532	19538.51	140.1488	19539.03	140.4373	19540.75	140.1425	19530.93	140.1453	19534.67	140.1489	19533.72	146.7551
140.4007	19538.46	140.3985	19536.21	140.6727	19537.52	140.3966	19532.08	140.3944	19532.22	140.4069	19533.88	147.0075
140.6467	19537.99	140.6513	19536.64	140.9246	19538.62	140.6441	19531.46	140.6488	19533.61	140.6528	19529.12	147.2527
140.8953	19538.47	140.9007	19537.9	141.1722	19538.8	140.9098	19532.4	140.895	19532.95	140.9062	19531.95	147.4977
141.1543	19536.65	141.1508	19538.45	141.4283	19538.74	141.145	19529.63	141.1459	19529.26	141.1483	19533.87	147.7569
141.3981	19537.62	141.3997	19538.68	141.6744	19539.41	141.3972	19529.22	141.3925	19534.49	141.4069	19530.97	148.0083
141.6467	19538.12	141.6501	19538.36	141.9255	19541.92	141.6439	19530.67	141.6518	19531.08	141.6523	19530.28	148.2536
141.8954	19539.68	141.8999	19540.21	142.1727	19539.86	141.9016	19531.37	141.8941	19534.99	141.9062	19532.23	148.4997
142.1533	19538.85	142.1483	19539.27	142.4263	19539.08	142.1446	19532.36	142.1469	19532.93	142.1489	19531.45	148.7556
142.398	19537.92	142.4123	19537.17	142.6745	19537.83	142.3984	19529.97	142.3948	19534.44	142.4068	19530.75	149.0062
142.6466	19534.53	142.6557	19537.5	142.9262	19538.65	142.6446	19530	142.6495	19532.19	142.6521	19532.5	149.2543
142.8962	19534.73	142.9006	19533.32	143.1726	19540.19	142.8999	19532.51	142.8951	19533.28	142.907	19531.3	149.503
143.1538	19536	143.1501	19539.1	143.4241	19538.36	143.1436	19533.71	143.1475	19534.86	143.1494	19532.03	149.756
143.3965	19537.81	143.3984	19536.23	143.6734	19538.62	143.3986	19532.62	143.3962	19530.76	143.4053	19532.42	150.0117
143.6476	19539.09	143.651	19539.8	143.9277	19537.43	143.6453	19530.26	143.6571	19532.22	143.6519	19530.09	150.2596
143.898	19536.25	143.8997	19537.17	144.1747	19539.31	143.9017	19532.8	143.8942	19531.18	143.9063	19531.93	150.5051
144.1566	19537.89	144.1507	19538.63	144.4263	19539.09	144.1457	19532.47	144.1478	19532.65	144.1518	19533.57	150.7588
144.4125	19539.6	144.3994	19536.09	144.6736	19538.28	144.3982	19534.36	144.3948	19531	144.4096	19529.49	151.0137
144.6536	19538.31	144.6504	19537.23	144.9278	19539.7	144.6465	19530.65	144.6552	19531.05	144.6523	19529.07	151.2585
144.8986	19537.52	144.9001	19539.64	145.1745	19536.86	144.9016	19530.05	144.895	19532.41	144.9091	19530.64	151.5036
145.1535	19537.14	145.1529	19536.22	145.4275	19536.9	145.1641	19528.53	145.1463	19533.18	145.1506	19529.99	151.761
145.4006	19537.14	145.4004	19537.96	145.6747	19538.77	145.3988	19533.58	145.3942	19532.15	145.4088	19531.67	152.0123
145.6497	19536.7	145.6505	19540.42	145.9268	19537.34	145.6479	19531.71	145.6505	19531.79	145.6522	19530.51	152.2593
145.8987	19534.83	145.8994	19536.61	146.1763	19536.91	145.901	19534.41	145.8963	19530.03	145.9075	19530.36	152.5037
146.1551	19535.66	146.1557	19538.44	146.4308	19539.45	146.146	19528.77	146.1468	19532.06	146.1568	19531.8	152.7603
146.4006	19537.99	146.4019	19539.1	146.6748	19538.45	146.3978	19532.24	146.3944	19529.07	146.4169	19530.86	153.0114
146.6505	19536.29	146.6518	19535.93	146.9279	19537.1	146.6444	19528.42	146.6556	19534.49	146.6522	19533.08	153.2616
146.8984	19536.71	146.8993	19537.58	147.1767	19537.9	146.9016	19530.31	146.8976	19532.36	146.9071	19533.97	153.5071
147.1568	19538.79	147.1499	19537.28	147.4297	19539.17	147.147	19528.5	147.1497	19532.76	147.1518	19533.75	153.7608
147.3997	19537.36	147.4006	19538.63	147.6767	19535.98	147.3984	19531.39	147.3973	19532.89	147.4096	19532.44	154.0159
147.6484	19536.57	147.6528	19535.16	147.9275	19538.85	147.6452	19530.1	147.6546	19534.7	147.6545	19533.15	154.2608
147.8987	19537.09	147.9002	19536.89	148.1733	19539.59	147.9033	19533.56	147.9025	19531	147.9089	19529.3	154.5072
148.1584	19536.99	148.1516	19538.47	148.4305	19539.61	148.1507	19531.71	148.1534	19531.11	148.1525	19532.28	154.762
148.4007	19538.7	148.4026	19536.31	148.6762	19538.69	148.4016	19531.91	148.4049	19531.51	148.4094	19530.35	155.015
148.6494	19537.19	148.653	19536.72	148.9311	19536.13	148.6503	19530.33	148.6551	19531.75	148.6537	19529.36	155.2594
148.9012	19535.87	148.9014	19535.99	149.1737	19539.43	148.9032	19530.9	148.897	19532.87	148.9082	19528.31	155.5063
149.1608	19537.83	149.1505	19537.02	149.43	19539.28	149.1462	19530.55	149.1458	19530.81	149.1515	19530.52	155.7608
149.4	19535.4	149.4026	19536.1	149.6776	19536.57	149.4008	19529.95	149.3954	19528.99	149.4131	19528.24	156.0136
149.6504	19537.74	149.6525	19537.37	149.9318	19539.58	149.6483	19531.18	149.6521	19532.33	149.6535	19531.77	156.2645
149.8999	19535.22	149.9034	19538.03	150.1756	19536.4	149.9114	19530.59	149.8999	19532.55	149.9094	19530.81	156.5055
150.1582	19536.11	150.1527	19536.89	150.4306	19539.37	150.1478	19532.2	150.1501	19529.45	150.1536	19530.9	156.7598
150.4025	19537.8	150.4045	19537.15	150.6803	19536.81	150.4034	19530.83	150.4004	19531.3	150.413	19531.42	157.0153
150.6532	19538.14	150.6549	19536.62	150.9332	19538.04	150.6545	19529.55	150.6563	19533.06	150.6577	19529.15	157.2599
150.9052	19537.12	150.9067	19537.77	151.1786	19536.47	150.9097	19531.85	150.9009	19532	150.9113	19531.18	157.5081
151.1637	19536.61	151.1581	19539.72	151.4331	19537.58	151.1528	19529.34	151.1509	19531.34	151.1543	19530.5	157.7673
151.4021	19537.48	151.4052	19536.07	151.6793	19536.17	151.4029	19532.42	151.4019	19532.77	151.4118	19531.47	158.0156
151.6529	19533.61	151.6552	19537.62	151.9325	19536.04	151.6526	19532.87	151.6589	19531.53	151.6594	19530.45	158.2644
151.9056	19536.44	151.9101	19537.99	152.182	19540.74	151.9071	19530.36	151.9022	19527.22	151.9128	19532.26	158.5059
152.1629	19538.35	152.1624	19539.24	152.4348	19538.19	152.1569	19528.63	152.1509	19531.38	152.1555	19530.95	158.7631
152.4012	19536.66	152.4053	19537.66	152.6802	19537.73	152.4051	19532.03	152.402	19533.94	152.4118	19532.57	159.0141
152.6525	19538.1	152.6556	19538.81	152.9366	19537.98	152.6516	19533.19	152.6582	19529.75	152.6559	19528.8	159.2636
152.9045	19536.56	152.9054	19539.47	153.1831	19537.74	152.9119	19529.44	152.9013	19530.68	152.9128	19528.15	159.5063
153.1619	19537.43	153.1574	19539.29	153.4366	19536.76	153.1532	19531.29	153.1507	19530.67	153.1604	19531.28	159.7619
153.4054	19536.48	153.4065	19536.55	153.6835	19537.85	153.4049	19534.18	153.4016	19527.86	153.4149	19529.22	160.0156
153.6535	19536.17	153.6576	19536.36	153.9327	19536.2	153.6514	19531.89	153.6642	19531.55	153.6578	19531.06	160.2624
153.9043	19534.9	153.9059	19536.75	154.181	19538.3	153.9161	19528.46	153.9014	19532.38	153.913	19530.94	160.5064
154.1626	19537.2	154.157	19536.63	154.4364	19537.13	154.1555	19531.55	154.1503	19532.45	154.1585	19532.87	160.7644
154.4043	19535.45	154.4081	19538.92	154.6818	19539.05	154.4053	19533.27	154.403	19533.66	154.4149	19531.93	161.0132
154.6536	19535.43	154.6587	19538.06	154.9337	19537.2	154.6529	19530.23	154.6602	19530.5	154.6551	19534.06	161.2599
154.9056	19536.89	154.9062	19537.45	155.1807	19538.68	154.9055	19530.6	154.9024	19530.11	154.9124	19528.83	161.5109
155.1638	19534.89	155.1567	19536.91	155.4348	19538.61	155.1544	19530.36	155.1532	19530.96	155.1578	19531.75	161.7748
155.4068	19538.82	155.407	19539.12	155.681	19536.26	155.4079	19527.82	155.4006	19531.72	155.4139	19529.19	162.0278
155.6587	19536.66	155.6573	19537.04	155.9367	19537.48	155.6548	19530.25	155.6583	19529.87	155.657	19530.49	162.2713
155.9039	19536.83	155.9065	19536.9	156.1842	19536.19	155.9115	19531.03	155.9037	19531.03	155.9133	19531.85	162.505
156.1636	19535.94	156.1591	19541.86	156.435	19537.49	156.1555	19531.18	156.1531	19531.8	156.1572	19530.87	162.7602

156.4054	19535.53	156.4069	19539.64	156.6822	19537.58	156.405	19531.59	156.4018	19531.33	156.4131	19531.55	163.0136
156.6546	19535.37	156.6587	19537.71	156.9407	19538.01	156.6543	19529.47	156.6583	19529.88	156.6605	19533	163.2588
156.9041	19535.01	156.9098	19536.08	157.1813	19535.73	156.9076	19529.99	156.9038	19531.88	156.9161	19533.63	163.5067
157.1634	19536.3	157.1564	19535.58	157.4345	19538.07	157.1534	19529.05	157.154	19530.11	157.1612	19529	163.7629
157.405	19536.18	157.4058	19537.44	157.6817	19539.22	157.4052	19529.11	157.4043	19533.18	157.4166	19529.56	164.0133
157.6562	19535.53	157.6568	19538.48	157.9379	19538.96	157.6528	19530.15	157.658	19531.68	157.6637	19530.61	164.2618
157.9062	19538.41	157.9109	19535.5	158.1814	19536.58	157.9128	19527.64	157.9044	19529.37	157.917	19529.09	164.5059
158.1671	19536.76	158.158	19535.91	158.4352	19536.97	158.1577	19530.42	158.1537	19530.53	158.1578	19531.77	164.7613
158.4059	19536.48	158.4083	19537.54	158.6815	19536.44	158.4082	19529.33	158.4049	19530.89	158.4151	19531.16	165.0267
158.6558	19537.66	158.6576	19536.77	158.9374	19538.88	158.6527	19530.46	158.6646	19531.22	158.6621	19532.72	165.2612
158.9075	19536.48	158.9089	19537.29	159.1825	19537.88	158.9147	19533.09	158.9036	19532.27	158.9167	19527.82	165.5072
159.1665	19535.77	159.1597	19534.46	159.4376	19536.43	159.1541	19530.55	159.154	19530.78	159.1589	19531.81	165.7645
159.4079	19535.28	159.4121	19538.45	159.6813	19535.83	159.4063	19529.17	159.4036	19532.75	159.4191	19530.66	166.0151
159.6579	19534.4	159.6586	19535.99	159.9367	19538	159.6553	19528.11	159.6641	19529.77	159.6605	19530.52	166.2599
159.9075	19535.8	159.9132	19537.07	160.1829	19536.88	159.9114	19529.11	159.9152	19531.95	159.9151	19530.42	166.5078
160.1658	19536.66	160.1597	19537.23	160.4394	19534.86	160.1547	19529.88	160.1541	19529.2	160.1593	19529.13	166.7624
160.4074	19533.59	160.4095	19536.08	160.6784	19539.23	160.41	19529.04	160.4069	19527.62	160.4142	19528.6	167.0154
160.6558	19536.69	160.6556	19537.32	160.9344	19536.87	160.6532	19533.56	160.6564	19532.14	160.6562	19532.61	167.2628
160.9034	19534.36	160.9069	19534.21	161.18	19537.61	160.908	19529.55	160.9036	19530.04	160.9146	19529.71	167.5064
161.1622	19536.73	161.1598	19535.84	161.4353	19536.78	161.1508	19529.61	161.1518	19530.78	161.1563	19527.58	167.7638
161.4041	19535.8	161.4056	19537.37	161.6824	19535.91	161.4058	19530.64	161.402	19533.91	161.4141	19529.52	168.0142
161.6546	19534.95	161.6575	19535.01	161.9348	19537.95	161.6525	19531.32	161.6583	19529.47	161.6571	19533.32	168.2619
161.9049	19537.9	161.9083	19537.21	162.1819	19538.48	161.9083	19527.99	161.903	19530.78	161.9152	19529.85	168.5086
162.1626	19536.06	162.1581	19536.38	162.4348	19537	162.1518	19530.23	162.1524	19527.71	162.1691	19532.25	168.7642
162.4054	19535.87	162.4063	19535.56	162.6811	19537.89	162.4057	19530.32	162.4038	19530.36	162.415	19529.62	169.0138
162.6548	19535.08	162.6562	19535.09	162.9345	19534.91	162.6534	19527.61	162.6577	19532.32	162.6568	19532.09	169.2611
162.9043	19535.38	162.9128	19535.17	163.1798	19535.89	162.9123	19531.41	162.9022	19530.99	162.9156	19530.04	169.5089
163.1643	19534.16	163.1559	19537.11	163.4338	19535.67	163.1521	19528.71	163.1525	19530.9	163.1573	19529.13	169.7657
163.405	19536.51	163.4062	19536.42	163.6825	19538.13	163.4054	19531.53	163.4043	19530.02	163.4153	19529.65	170.0143
163.6572	19535.01	163.6557	19535.4	163.9339	19538.92	163.6526	19528.71	163.6618	19531.8	163.6582	19531.81	170.2637
163.9051	19534.92	163.9096	19538.01	164.1802	19538.33	163.9095	19528.01	163.9029	19528.2	163.915	19528.82	170.506
164.1627	19536.85	164.1578	19536.17	164.4346	19538.15	164.1534	19529.09	164.1532	19529.79	164.1581	19531.02	170.7644
164.4046	19535.67	164.4122	19535.39	164.683	19538.35	164.4057	19528.4	164.4056	19531.02	164.4176	19530	171.0157
164.6593	19534.79	164.6576	19536.63	164.9358	19536.86	164.6541	19531.17	164.6621	19532.18	164.6724	19531.16	171.2635
164.9052	19537.07	164.9093	19536.76	165.1808	19536.15	164.9109	19527.55	164.9033	19527.56	164.9182	19530.14	171.5085
165.1621	19537.13	165.1597	19535.84	165.4346	19537.84	165.1538	19532.64	165.1524	19531.5	165.1566	19526.63	171.7654
165.4075	19534.96	165.4082	19534.73	165.6814	19537.98	165.4049	19531.55	165.406	19530.66	165.4171	19529.62	172.0154
165.6565	19536.56	165.6563	19535.94	165.9369	19535.87	165.6538	19532.4	165.6612	19530.56	165.6611	19531.93	172.2649
165.9056	19536.99	165.9093	19534.03	166.1827	19536.11	165.9104	19530.92	165.9061	19534.46	165.9153	19531.51	172.5093
166.1638	19536.1	166.1608	19536.08	166.436	19538.56	166.1526	19530.47	166.1538	19530.5	166.158	19528.38	172.7647
166.406	19536.04	166.4093	19535.15	166.6817	19534.35	166.4077	19530.44	166.4036	19527.55	166.4152	19526.26	173.0181
166.6566	19538.66	166.658	19538.57	166.9369	19536.03	166.6546	19529.59	166.6589	19530.36	166.66	19532.9	173.2642
166.9097	19535.26	166.9108	19535.36	167.1827	19535.55	166.912	19528.66	166.9051	19529.25	166.9157	19531.35	173.5096
167.163	19534.04	167.1598	19538.33	167.4363	19539.18	167.1529	19529.09	167.1553	19528.89	167.1597	19528.41	173.7651
167.4064	19536.18	167.4099	19536.3	167.6828	19536.18	167.4085	19530.63	167.4054	19529.53	167.4179	19527.9	174.0171
167.6545	19536.62	167.6603	19537.12	167.9354	19536.58	167.6544	19531.67	167.6631	19528.64	167.6585	19528.92	174.2644
167.9073	19535.3	167.91	19535.46	168.1839	19535.57	167.9151	19530.56	167.9029	19528.14	167.9157	19529.59	174.5106
168.1626	19535.51	168.1587	19536.01	168.4386	19535.46	168.1552	19529.42	168.154	19529.63	168.1605	19527.99	174.7671
168.4086	19536.51	168.4108	19538.73	168.6821	19535.94	168.409	19528.71	168.4056	19528.27	168.417	19532.67	175.0209
168.6587	19537.18	168.6594	19537.14	168.9362	19537.99	168.6544	19530.16	168.6652	19530.72	168.6605	19533.04	175.2662
168.9055	19533.54	168.9104	19533.77	169.184	19537.56	168.9098	19532.7	168.9079	19530.73	168.9177	19530.97	175.5099
169.1681	19535.47	169.1585	19537.24	169.439	19535.48	169.1537	19529.44	169.1537	19527.83	169.1596	19528.88	175.766
169.4084	19537.14	169.4088	19534.7	169.6823	19535.97	169.4074	19531.03	169.4055	19528.51	169.4192	19530.12	176.0189
169.6596	19536.07	169.6624	19534.22	169.936	19537.85	169.6556	19529.73	169.6634	19534.38	169.6613	19531.65	176.2663
169.9081	19536.06	169.9105	19534.74	170.1843	19535.04	169.9132	19529.87	169.9064	19529.79	169.9187	19530.53	176.5106
170.1643	19535.72	170.1599	19533.41	170.4395	19536.53	170.1558	19529.75	170.1575	19531.69	170.1588	19528.48	176.7685
170.4097	19537.18	170.4105	19538.74	170.6846	19536.42	170.4083	19527.3	170.4038	19530.45	170.4184	19529.32	177.0191
170.6594	19536.15	170.661	19537.74	170.937	19536.97	170.6546	19527.34	170.6613	19528.02	170.6624	19530.72	177.2688
170.9112	19534.21	170.9097	19537.22	171.1836	19538.47	170.9119	19530.16	170.9064	19528.31	170.9167	19526.97	177.5111
171.164	19537.28	171.1621	19534.51	171.4384	19533.89	171.1577	19529.23	171.1565	19527.88	171.1615	19530.67	177.7678
171.4089	19535.78	171.4088	19535.45	171.6845	19537.63	171.4094	19530.46	171.4062	19530.01	171.4177	19530.34	178.0175
171.6606	19534.26	171.6618	19537.92	171.9387	19537.4	171.6578	19527.27	171.6635	19531.31	171.6627	19529.15	178.271
171.9088	19537.33	171.9112	19534.89	172.1855	19537.03	171.9134	19528.63	171.9059	19528.74	171.9188	19531.64	178.514
172.1633	19531.51	172.1619	19537.02	172.4386	19536.6	172.1562	19526.49	172.1564	19528.49	172.1593	19528.49	178.7673
172.4106	19536.52	172.4103	19534.66	172.6861	19538.47	172.4132	19528.58	172.4061	19532.15	172.4158	19528.03	179.0184
172.6597	19534.42	172.6584	19536.87	172.9382	19535.06	172.6577	19529.27	172.6649	19529.85	172.6616	19528.27	179.2693
172.9089	19534.43	172.9111	19537.61	173.184	19535.54	172.9202	19531.22	172.906	19530.81	172.9182	19529.81	179.5257
173.1651	19536.46	173.1615	19537.26	173.4386	19536.84	173.1547	19529.79	173.1602	19529.24	173.1619	19528.59	179.772
173.4209	19535.91	173.4107	19534.1	173.6854	19537.04	173.411	19532.49	173.4065	19528.35	173.4182	19529.51	180.0217
173.6605	19533.67	173.6621	19538.41	173.9398	19538.9	173.6595	19530.06	173.6665	19533.16	173.6609	19529.3	180.2672

173.9099	19536.16	173.9114	19536.04	174.1865	19537.18	173.9137	19530.45	173.907	19531.01	173.9176	19528.54	180.5122
174.1721	19535.66	174.1617	19537.48	174.438	19536.64	174.1617	19529.05	174.1556	19528.7	174.1629	19527.36	180.7691
174.4166	19536.03	174.4122	19537.53	174.6852	19535.23	174.4093	19527.53	174.407	19527.31	174.4201	19527.3	181.0207
174.659	19534.96	174.6609	19536.23	174.9401	19535.98	174.6571	19529.65	174.6654	19527.07	174.6633	19528.76	181.2695
174.9092	19534.44	174.9118	19535.85	175.1877	19538.68	174.9138	19527.69	174.9083	19527.77	174.9176	19528.11	181.5131
175.1686	19535.55	175.1647	19534.85	175.4405	19536.22	175.1576	19529.72	175.1573	19531.45	175.1623	19529.4	181.767
175.4125	19535.98	175.4118	19537.61	175.685	19534.89	175.412	19526.22	175.4077	19528.16	175.4193	19532.19	182.0192
175.6604	19534.17	175.6617	19535.49	175.9385	19537.54	175.6574	19529.91	175.6663	19527.45	175.6654	19532.7	182.2681
175.9087	19536.04	175.9125	19536.06	176.1879	19535.28	175.917	19530.95	175.908	19529.92	175.9181	19529.22	182.5151
176.1655	19536.45	176.1655	19535.47	176.4414	19536.17	176.1582	19528.69	176.1589	19533.8	176.1611	19529.2	182.7718
176.4122	19534.97	176.4127	19536.2	176.686	19535.41	176.4123	19528.58	176.4086	19532.18	176.4214	19526.71	183.0191
176.665	19535.75	176.6628	19536.2	176.9416	19535.7	176.6595	19530.61	176.6659	19527.97	176.6642	19533.03	183.27
176.9094	19534.13	176.9131	19537.14	177.1886	19536.46	176.9141	19529.53	176.9079	19530.14	176.919	19528.08	183.5157
177.1681	19532.86	177.1618	19537.39	177.442	19535.44	177.1589	19530.42	177.1595	19527.98	177.1624	19525.92	183.7707
177.4149	19536.22	177.4118	19537.05	177.6865	19537.36	177.416	19530.34	177.4091	19531.26	177.4198	19531.69	184.0213
177.6641	19533.66	177.6612	19537.49	177.9405	19534.46	177.6585	19530.4	177.6682	19529.47	177.6648	19528.05	184.2705
177.912	19536.06	177.9148	19538.55	178.1878	19535.3	177.9172	19530.44	177.9072	19529.64	177.9193	19530.16	184.5147
178.1665	19535.64	178.1743	19535.99	178.4423	19534.13	178.1569	19528.37	178.158	19531.39	178.1617	19527.22	184.7702
178.4113	19534.39	178.4121	19534.47	178.6877	19534.62	178.414	19531.12	178.4088	19528.97	178.4191	19528.81	185.02
178.6632	19534.84	178.6615	19538.76	178.9416	19535.34	178.6606	19527.12	178.6697	19531.58	178.6659	19528.56	185.2702
178.9132	19537.93	178.9138	19533.37	179.1889	19537.65	178.919	19528.82	178.9096	19527.52	178.9199	19526.42	185.5153
179.1693	19536.2	179.1661	19533.63	179.4431	19535.5	179.1568	19528.29	179.1587	19525.55	179.1639	19530.56	185.7744
179.4128	19534.24	179.4133	19533.7	179.6882	19537.09	179.4128	19530.74	179.4078	19532.09	179.4209	19529.74	186.0234
179.6627	19534.59	179.6619	19536.12	179.9417	19534.31	179.6606	19528.81	179.6666	19528.51	179.668	19525.9	186.2709
179.9126	19530.92	179.9162	19537.35	180.1902	19536.85	179.9201	19528.34	179.9094	19528.87	179.9226	19526.73	186.5167
180.166	19535.39	180.1658	19537.12	180.4424	19536.54	180.1611	19526.38	180.1596	19530.76	180.1633	19527.65	186.7701
180.4133	19533.32	180.4166	19534.94	180.6879	19534.99	180.4137	19528.33	180.4088	19526.97	180.4203	19529.38	187.0221
180.6626	19536.25	180.6633	19536.42	180.9415	19540.18	180.6607	19527.8	180.6722	19528.38	180.6649	19528.5	187.2691
180.9127	19535.37	180.9157	19533.85	181.1888	19534.57	180.9182	19529.81	180.9115	19530.17	180.9237	19528.76	187.5173
181.1689	19536.43	181.1689	19536.31	181.4434	19535.61	181.1593	19528.85	181.1614	19528.69	181.1656	19531.22	187.7725
181.4136	19535.96	181.4153	19534.77	181.6878	19536.16	181.413	19529.3	181.4089	19531.44	181.4204	19529.29	188.023
181.6619	19535.48	181.6638	19536.03	181.942	19533.57	181.6613	19530.72	181.6691	19531.34	181.6662	19529.84	188.2721
181.9128	19533.85	181.9192	19532.45	182.1916	19534.7	181.9197	19530.58	181.9097	19528.44	181.9206	19528.88	188.5158
182.1678	19534.69	182.1667	19535.74	182.4448	19536.72	182.1597	19528.11	182.1603	19528.21	182.1648	19529.34	188.7727
182.4123	19534.58	182.4146	19532.32	182.6892	19534.45	182.4132	19529.85	182.4096	19529.32	182.4207	19526.76	189.0215
182.6642	19535.6	182.6672	19535.17	182.9419	19536.57	182.6605	19527.54	182.6695	19528.81	182.6656	19530.98	189.2703
182.9129	19534.34	182.9157	19534.77	183.1896	19536.13	182.9192	19528.06	182.9104	19533.13	182.9231	19529.33	189.5156
183.1679	19535.15	183.1655	19537.08	183.4449	19536.91	183.1591	19528.79	183.1613	19529.76	183.1666	19526.84	189.772
183.4121	19535.37	183.4162	19534.21	183.6891	19535.19	183.4132	19528.88	183.4128	19526.39	183.4238	19529.92	190.0279
183.6664	19536.7	183.6645	19536.06	183.9418	19536.88	183.6606	19528.89	183.6731	19529.85	183.6678	19528.79	190.2701
183.9144	19537.04	183.9169	19531.25	184.1922	19537.18	183.9191	19532	183.9103	19530.63	183.9213	19528.69	190.5171
184.1747	19536.28	184.1675	19534.87	184.4453	19535.93	184.1607	19527.15	184.161	19530.34	184.1666	19529.07	190.774
184.4158	19534.73	184.4146	19532.07	184.6908	19535.01	184.4143	19528.48	184.412	19533.58	184.424	19527.83	191.0235
184.6646	19534.57	184.6639	19536.59	184.9412	19535.57	184.6615	19529.82	184.673	19528.89	184.6679	19526.65	191.2711
184.9139	19532.67	184.916	19534.77	185.1913	19537.3	184.9191	19527.95	184.9108	19527.97	184.9209	19528.25	191.5163
185.1688	19535.46	185.1694	19534.78	185.4458	19534.91	185.161	19526.78	185.1604	19530.23	185.166	19531.25	191.772
185.4145	19535.94	185.4152	19531.92	185.6897	19537.13	185.414	19528.33	185.4134	19530.82	185.4222	19528.27	192.0253
185.6673	19534.45	185.6649	19536.53	185.9427	19536.85	185.6628	19528.44	185.6701	19528.34	185.6668	19530.71	192.2713
185.9154	19536.11	185.9166	19536.44	186.1903	19536.14	185.918	19525.27	185.9135	19529.84	185.9234	19527.06	192.5177
186.1707	19533.89	186.1709	19534	186.4468	19536.51	186.1621	19530.23	186.1607	19527.88	186.1661	19529.61	192.7709
186.4145	19532.92	186.417	19535.32	186.6946	19534.85	186.4162	19527.19	186.4149	19528.37	186.4216	19528.87	193.025
186.668	19535.09	186.6648	19533.6	186.9434	19539.11	186.6615	19529.03	186.672	19527.12	186.6691	19530.86	193.2708
186.9151	19535.33	186.9153	19533	187.1912	19535.74	186.9191	19526.6	186.912	19528.08	186.9271	19529.81	193.5183
187.1688	19530.9	187.1697	19535.65	187.4481	19537.13	187.1616	19529.02	187.1633	19529.09	187.179	19528.53	193.7739
187.4161	19533.92	187.4175	19536.2	187.6914	19536.65	187.4146	19526.48	187.4124	19527.15	187.4233	19529.25	194.0241
187.6657	19535.5	187.6682	19536.67	187.9441	19534.28	187.6664	19528.95	187.6715	19528.99	187.668	19525.96	194.2726
187.9166	19534.06	187.9163	19532.72	188.1957	19533.69	187.9199	19531.18	187.9139	19528.16	187.9223	19526.48	194.5178
188.1721	19533.41	188.1704	19535.61	188.4461	19535.86	188.1637	19526.76	188.163	19532.87	188.1672	19525.7	194.7725
188.4162	19534.15	188.4175	19533.79	188.6911	19536.04	188.4166	19528.19	188.4164	19528.35	188.4246	19527.31	195.0251
188.6664	19533.16	188.6714	19536.95	188.9443	19535.44	188.6641	19530.93	188.6718	19527.8	188.6693	19531.88	195.2734
188.9157	19534.5	188.9173	19536.61	189.1925	19535.66	188.9183	19530.3	188.9136	19526.94	188.9223	19527.84	195.5212
189.1724	19536.8	189.1693	19534.52	189.4489	19535.81	189.1616	19527.61	189.1663	19528.51	189.1689	19524.57	195.7739
189.4164	19534.56	189.4169	19535.08	189.6935	19535.34	189.4177	19530.86	189.4135	19527.81	189.4247	19527.15	196.0261
189.6684	19535.4	189.6688	19535.83	189.9443	19535.95	189.6651	19526.72	189.6723	19526.34	189.6701	19528.25	196.2727
189.9162	19533.97	189.9209	19534.93	190.1935	19535.1	189.9216	19527.95	189.914	19530.38	189.9239	19528.6	196.5216
190.1733	19533.98	190.1706	19534.23	190.4662	19534.92	190.1629	19528.8	190.1679	19529.22	190.1715	19531	196.7746
190.416	19533.98	190.4174	19535.26	190.7072	19536.89	190.4171	19527.83	190.4142	19529.81	190.4252	19526.49	197.0266
190.6679	19532.3	190.6674	19535.34	190.9448	19532.86	190.6647	19529.36	190.6861	19532.03	190.6705	19528.89	197.2729
190.9168	19536.53	190.9204	19536.36	191.1918	19535.31	190.9206	19528.27	190.9137	19530.45	190.9251	19527.49	197.5189
191.1706	19535.38	191.1735	19535.51	191.4489	19536.6	191.1635	19525.28	191.1634	19527.76	191.1699	19528.64	197.7754

191.4171	19537.41	191.4201	19536.32	191.6936	19536.85	191.4212	19528.57	191.4145	19527.98	191.4253	19526.98	198.0259
191.6701	19537.57	191.6677	19534.75	191.9449	19531.76	191.6645	19527.52	191.6723	19531.65	191.6759	19533.17	198.2751
191.9177	19531.7	191.919	19533.12	192.1929	19536.16	191.922	19530.98	191.9146	19529.48	191.9253	19527.1	198.5218
192.1815	19536.04	192.1682	19536.81	192.4488	19536.11	192.1671	19526.27	192.1665	19527.9	192.1729	19529.04	198.7751
192.4198	19533.85	192.4193	19535.41	192.6931	19533.78	192.4197	19528.4	192.4139	19531.31	192.4255	19529.51	199.025
192.6718	19535.28	192.6663	19535.21	192.9452	19533.59	192.6657	19529.55	192.6732	19530.28	192.67	19529.86	199.2728
192.9269	19534.75	192.9192	19535.15	193.192	19536.21	192.9273	19525.03	192.9147	19530.38	192.9254	19527.79	199.522
193.1738	19534.88	193.1701	19533.26	193.4473	19534.73	193.1633	19530.07	193.1662	19527.24	193.1689	19527.14	199.7768
193.422	19535.15	193.4205	19534.42	193.6943	19533.27	193.4183	19532.48	193.4144	19529.22	193.4263	19525.21	200.0323
193.6694	19531.7	193.6728	19538.32	193.9497	19533.84	193.6669	19526.55	193.6731	19531.92	193.6724	19527.18	200.2911
193.9191	19534.68	193.9178	19532	194.1916	19537.2	193.924	19529.31	193.9151	19528.19	193.9235	19529.73	200.5253
194.173	19535.25	194.1704	19536.18	194.4479	19535.7	194.1676	19529.24	194.1685	19528.65	194.1702	19528.64	200.7803
194.4189	19537.4	194.4341	19534.51	194.6942	19536.35	194.4181	19526.33	194.417	19526.92	194.4296	19527	201.0309
194.6696	19534.74	194.6721	19535.03	194.9481	19538.03	194.6697	19526.77	194.6728	19531.26	194.6734	19525.86	201.2806
194.9262	19534.68	194.9255	19534.56	195.1937	19534.49	194.9223	19527.8	194.915	19530.42	194.9255	19526.1	201.5267
195.1742	19534.86	195.1695	19532.9	195.4484	19533.5	195.1647	19529.85	195.1661	19530.22	195.1688	19529.64	201.7781
195.4176	19533.12	195.4188	19533.28	195.6937	19535.92	195.4183	19529.91	195.4182	19527.91	195.4258	19529.93	202.0295
195.67	19533.76	195.671	19535.83	195.948	19534.76	195.6679	19526.86	195.6773	19527.41	195.671	19528.93	202.2775
195.918	19537.54	195.9209	19533.97	196.1954	19538.2	195.9252	19525.96	195.9179	19527.82	195.9264	19531.34	202.5268
196.1769	19535.22	196.1734	19534.05	196.4512	19535.18	196.1643	19526.7	196.1669	19526.4	196.168	19527.09	202.7807
196.4201	19534.08	196.4194	19534.21	196.6932	19534.89	196.4193	19527.66	196.4172	19528.57	196.4272	19531.79	203.0322
196.6723	19533.89	196.6694	19533.76	196.9478	19536.14	196.6668	19530.11	196.6745	19530.02	196.675	19528.52	203.2793
196.918	19535.96	196.9245	19534.51	197.1946	19534.82	196.9245	19527.73	196.9161	19528.78	196.9269	19528.46	203.528
197.1743	19534.68	197.171	19534.49	197.4532	19532.98	197.1661	19526.46	197.1655	19529.13	197.1706	19526.75	203.7817
197.4179	19532	197.4195	19535.74	197.695	19534.5	197.4195	19526.8	197.4172	19528.37	197.4269	19528.15	204.0308
197.6714	19531.92	197.6696	19536.66	197.9471	19535.36	197.6669	19527.94	197.6739	19529.55	197.6712	19529.08	204.2801
197.9201	19535.59	197.9223	19537.32	198.1965	19535.58	197.9257	19525.42	197.917	19531.36	197.9259	19529.07	204.5284
198.1854	19532.25	198.1813	19533.46	198.4596	19535.58	198.1757	19528.47	198.1772	19529.44	198.1802	19526.42	204.792
198.4187	19534.47	198.4232	19534.8	198.6959	19536.15	198.4197	19527.77	198.4166	19527.89	198.428	19529.49	205.0317
198.6725	19536.16	198.6701	19536.25	198.9473	19537.16	198.6674	19529.84	198.68	19530.12	198.6729	19530.8	205.278
198.9209	19535.67	198.9248	19534.64	199.195	19536.49	198.925	19529.8	198.9191	19526.77	198.9278	19529.78	205.5289
199.1753	19535.33	199.1754	19535.95	199.4514	19536.26	199.1653	19525.87	199.17	19527.47	199.1715	19528.58	205.7824
199.4212	19533.46	199.4223	19535.43	199.6954	19533.89	199.4206	19529.41	199.4162	19525.2	199.4312	19528.09	206.0315
199.6718	19533.05	199.6722	19536.88	199.9481	19535.66	199.6689	19527.71	199.6759	19526.68	199.6755	19525.37	206.2809
199.9205	19534	199.9209	19532.26	200.1949	19533.72	199.9274	19525.2	199.9185	19526.57	199.9287	19528.06	206.5271
200.1737	19536.63	200.1717	19535.14	200.456	19535.05	200.1666	19527.46	200.174	19529.57	200.1697	19525.27	206.7822
200.4238	19533.04	200.4289	19535.18	200.7005	19533.79	200.4256	19529.6	200.4212	19528.58	200.432	19526.26	207.0325
200.6766	19532.33	200.6758	19536.39	200.9537	19536.38	200.6729	19527.09	200.6802	19531.87	200.677	19526.83	207.2822
200.928	19533.1	200.927	19536.11	201.1999	19536.53	200.9499	19528.53	200.9213	19527.99	200.9323	19526.61	207.5284
201.1827	19533.17	201.1795	19534.17	201.4585	19535.85	201.1711	19529.19	201.1727	19526.03	201.1761	19530.47	207.7812
201.4279	19533.19	201.4283	19533.31	201.6999	19537.98	201.4261	19525.54	201.4217	19529.81	201.4337	19526.21	208.0328
201.6756	19533.44	201.6759	19534.33	201.9527	19535.72	201.671	19528.63	201.6813	19529.33	201.6784	19526.79	208.2804
201.9252	19533.37	201.9266	19533.37	202.2005	19536.06	201.9297	19527.9	201.9217	19527.14	201.9336	19530.47	208.5301
202.1805	19532.87	202.1799	19535.36	202.4597	19531.95	202.1726	19526.7	202.1721	19529.57	202.1756	19526.44	208.7825
202.4268	19532.91	202.4262	19532.62	202.6996	19534.82	202.4257	19526.12	202.422	19528.52	202.4351	19524.52	209.0326
202.678	19536.03	202.6907	19534.48	202.9534	19535.19	202.6721	19526.26	202.682	19530	202.68	19528.46	209.2822
202.9261	19533.36	202.9358	19533.34	203.2003	19534.03	202.9358	19526.9	202.9247	19531.26	202.9339	19527.03	209.528
203.1815	19535.31	203.1778	19535.22	203.4618	19536.9	203.1719	19528.06	203.1734	19527.76	203.1784	19526.78	209.7825
203.4276	19532.77	203.4268	19537.37	203.703	19536.2	203.4262	19529.83	203.4207	19524.59	203.4346	19526.73	210.0339
203.6825	19533.61	203.6765	19536.33	203.9533	19534.08	203.674	19526.34	203.6824	19527.15	203.6792	19530.22	210.2822
203.9284	19533.98	203.9278	19535.26	204.2018	19535.14	203.9307	19527.39	203.9247	19527.54	203.934	19527.01	210.5304
204.1817	19533.77	204.1783	19533.83	204.4607	19534.94	204.1734	19527.26	204.1775	19531.06	204.1792	19526.25	210.7819
204.4261	19534.01	204.432	19533.59	204.7076	19534.35	204.4267	19524.53	204.4264	19526.87	204.4341	19527.44	211.0302
204.6922	19533.07	204.6769	19533.79	204.9531	19532.39	204.6743	19526.74	204.683	19529.62	204.6828	19528.37	211.2819
204.9338	19536.1	204.9267	19533.5	205.2045	19537.9	204.934	19526.18	204.9253	19527.11	204.9372	19526.32	211.5287
205.1812	19536.44	205.1823	19535.62	205.4606	19536.17	205.1715	19528.43	205.1805	19529.21	205.1754	19528.53	211.7872
205.4251	19535.75	205.4316	19535.51	205.7022	19534.89	205.4457	19525.61	205.424	19527.76	205.4377	19528.65	212.034
205.6772	19532.77	205.6808	19534.05	205.9563	19537.36	205.6751	19529.11	205.6822	19527.42	205.6821	19528.13	212.2844
205.9273	19534.44	205.9276	19533.98	206.2031	19535.19	205.9324	19528.7	205.9261	19527.68	205.9345	19528.65	212.5287
206.1809	19534.78	206.1827	19532.1	206.4598	19537.52	206.1728	19527.85	206.1766	19527.69	206.1785	19531.77	212.7815
206.4274	19532.65	206.4302	19533.52	206.7018	19537.25	206.4272	19528.32	206.4246	19529.67	206.4462	19526.66	213.0332
206.6789	19535.16	206.6792	19534.25	206.9559	19536.06	206.6739	19527.27	206.6829	19530.42	206.6895	19526.95	213.2832
206.9268	19535.34	206.9287	19533.09	207.2021	19534.61	206.9324	19527.67	206.9261	19526.23	206.9333	19527.91	213.5302
207.2024	19533.58	207.1797	19535.16	207.4581	19536.52	207.1734	19529.07	207.1756	19526.08	207.1776	19523.99	213.785
207.4382	19533.05	207.4295	19538.4	207.7025	19536.99	207.4288	19528.18	207.4238	19527.46	207.437	19526.68	214.0363
207.6776	19534.58	207.6792	19535.37	207.9581	19536.67	207.6743	19524.8	207.6815	19529.37	207.6843	19531.44	214.2848
207.9285	19533.38	207.9296	19533.31	208.2048	19535.41	207.936	19528.62	207.9262	19528.29	207.9353	19523.82	214.5311
208.1824	19533.4	208.1817	19531.99	208.4603	19535.61	208.1767	19529.74	208.1753	19528.65	208.1812	19527.58	214.787
208.4258	19534.26	208.4309	19534.83	208.7015	19537.07	208.4358	19530.49	208.4255	19526.81	208.4349	19526.32	215.0336
208.6788	19534.85	208.6796	19533.41	208.9548	19534.41	208.6745	19531.31	208.6845	19529.34	208.6807	19526.16	215.2852

208.9288	19534.12	208.9299	19533.98	209.203	19532.81	208.9348	19526.84	208.9267	19526.29	208.9342	19529.43	215.5311
209.1818	19533.21	209.1806	19532.62	209.4606	19534.56	209.1745	19529.01	209.1761	19526.38	209.1788	19525.86	215.7866
209.4261	19536.53	209.4314	19531.43	209.708	19537.52	209.4277	19527.43	209.4242	19525.45	209.4374	19526.89	216.0385
209.6778	19534.72	209.6792	19536.35	209.9564	19533.78	209.675	19527.74	209.6805	19531.89	209.6825	19524.3	216.2831
209.928	19533.94	209.9304	19534.03	210.2033	19537.16	209.9371	19528.33	209.9272	19525.53	209.9354	19526.79	216.5299
210.1845	19534.15	210.1828	19534.59	210.4586	19533.86	210.1745	19528.11	210.177	19527.38	210.179	19529.85	216.7898
210.4279	19532.25	210.4316	19536.24	210.7047	19531.57	210.4308	19528.04	210.4248	19530	210.4367	19527.16	217.0339
210.6788	19534.24	210.6809	19535.08	210.9596	19534.31	210.6768	19526.79	210.6851	19527.36	210.6847	19526.49	217.2847
210.9295	19530.97	210.9293	19532.57	211.203	19535.55	210.9367	19528.67	210.9287	19530.66	210.9365	19526.23	217.5316
211.1865	19532.24	211.184	19534.14	211.4586	19535.8	211.1792	19529.64	211.1777	19529.03	211.1799	19529.56	217.7869
211.4285	19532.38	211.4328	19532.61	211.7039	19533.56	211.4287	19526.34	211.4272	19528.67	211.4371	19526.89	218.0365
211.6797	19534	211.6803	19532.95	211.958	19534.88	211.6767	19528.22	211.6855	19525.56	211.6834	19527.46	218.2851
211.931	19535.09	211.9303	19534.71	212.2053	19535.81	211.9375	19525.27	211.9289	19528.15	211.9358	19530.25	218.5326
212.1837	19531.8	212.1812	19534.05	212.4669	19536.09	212.1767	19527.35	212.1793	19528.26	212.1799	19530.15	218.7888
212.4346	19533.17	212.434	19535.48	212.7046	19533.68	212.433	19526.9	212.4276	19529.65	212.4343	19525.12	219.0339
212.6793	19532.46	212.6798	19536.35	212.957	19533.46	212.6788	19528.18	212.6876	19529.98	212.6861	19524.66	219.285
212.9292	19533.77	212.9291	19535.07	213.2067	19534.65	212.9411	19528.3	212.9282	19526.83	212.9408	19530.71	219.5324
213.1843	19534.15	213.1814	19533.59	213.4638	19535.21	213.1765	19524.41	213.1787	19527.15	213.1792	19526.34	219.8042
213.4289	19534.22	213.4314	19532.74	213.7082	19534.85	213.4299	19528.85	213.4271	19531.23	213.4386	19529.33	220.0323
213.6812	19533.6	213.681	19534.74	213.9575	19532.82	213.6754	19525.51	213.6879	19529.89	213.6901	19529.22	220.287
213.9307	19534.57	213.9314	19535.02	214.2056	19535.5	213.9384	19527.71	213.9286	19530.13	213.9385	19529.36	220.5305
214.186	19532.43	214.1811	19532.31	214.4638	19534.55	214.1788	19531.04	214.1769	19528.85	214.181	19527.52	220.7891
214.428	19534.1	214.4311	19535.03	214.7066	19535.23	214.4306	19525.26	214.429	19528.84	214.4393	19527.74	221.0366
214.6813	19532.81	214.6822	19532.26	214.9613	19534.03	214.6762	19526.36	214.6874	19529.17	214.6841	19526.51	221.2887
214.9319	19532.47	214.9321	19536.06	215.205	19537.06	214.9358	19527.58	214.9281	19529.41	214.9364	19526.46	221.5341
215.1847	19533.67	215.1827	19534.56	215.4632	19534.85	215.1769	19528.43	215.1783	19526.41	215.1821	19525.35	221.79
215.4287	19531.84	215.4311	19534.88	215.7134	19535.41	215.4303	19527.51	215.4279	19528.36	215.4401	19524.3	222.0365
215.6813	19533.28	215.6811	19533.26	215.9661	19535.72	215.6781	19526.2	215.6871	19526.34	215.6861	19526.85	222.2881
215.9311	19532.15	215.9311	19532.57	216.2073	19535.64	215.9363	19525.59	215.9274	19525.71	215.9391	19526.94	222.5345
216.1842	19530.14	216.1865	19534.63	216.4642	19534.35	216.1779	19527.06	216.1799	19526.57	216.1842	19524.65	222.7894
216.4313	19533.59	216.4324	19535.77	216.7095	19537.43	216.4302	19526.54	216.4287	19529.47	216.4386	19524.76	223.0392
216.682	19533.13	216.6835	19535.29	216.9611	19535.54	216.678	19528.24	216.6875	19528.66	216.6863	19528.09	223.2891
216.9306	19534.55	216.9306	19534.64	217.2074	19533.65	216.9407	19526.23	216.9347	19527.2	216.9381	19527.64	223.5341
217.1845	19534.52	217.1841	19534.57	217.4656	19533.23	217.1776	19526.75	217.1801	19525.39	217.1811	19527.96	223.7903
217.4297	19534.71	217.4327	19535.37	217.7069	19536.9	217.4339	19532.7	217.4283	19528.68	217.438	19525.63	224.0382
217.6838	19533.86	217.6831	19533.56	217.9596	19535.93	217.6788	19529.21	217.6855	19529.31	217.6881	19525.03	224.2894
217.9351	19531.63	217.9327	19534.31	218.2076	19531.36	217.9398	19526.16	217.9274	19528.78	217.9394	19525.82	224.5374
218.1865	19534.16	218.1866	19533.89	218.4649	19536.46	218.1782	19526.6	218.18	19531.68	218.1842	19526.73	224.7914
218.4311	19534.21	218.4345	19536.06	218.7092	19535.23	218.432	19527.33	218.4279	19528.06	218.4388	19529	225.0381
218.6821	19532.33	218.6832	19533.59	218.96	19534.51	218.6792	19526.25	218.6897	19526.33	218.6875	19529.53	225.2885
218.9344	19534.97	218.9333	19534.32	219.2073	19534.01	218.9396	19528.05	218.9297	19529.87	218.9398	19526.24	225.5337
219.1907	19531.03	219.1844	19532.84	219.4639	19536.33	219.1787	19527.89	219.1813	19531.09	219.1825	19526.82	225.7905
219.4332	19534.47	219.4336	19533.5	219.7094	19535.22	219.4331	19528.2	219.4301	19527.7	219.4425	19527.83	226.0402
219.6832	19533.49	219.6842	19533.45	219.9596	19531.96	219.6814	19528.43	219.6867	19527.48	219.6881	19527.58	226.2901
219.9345	19534.16	219.9337	19533.95	220.2073	19534.56	219.9398	19528.02	219.9296	19525.34	219.9402	19529.85	226.5357
220.1866	19536.97	220.1851	19533.97	220.4647	19534.4	220.1788	19527.02	220.18	19528.88	220.1849	19526.21	226.7909
220.434	19530.57	220.4343	19533.43	220.7098	19533.71	220.4328	19525.44	220.4293	19528.13	220.4414	19529.26	227.0402
220.6839	19532.71	220.6841	19534.1	220.962	19536.53	220.6781	19526.31	220.6881	19525.71	220.688	19530.56	227.2894
220.9358	19534.49	220.9352	19533.89	221.2081	19534.57	220.9428	19529.38	220.9307	19527.17	220.9383	19525.13	227.5372
221.1867	19534.65	221.1882	19532.42	221.4631	19533.52	221.1839	19526.74	221.1817	19528.45	221.1843	19525.98	227.7923
221.4304	19534.03	221.4354	19535.63	221.7105	19534.99	221.4337	19526.11	221.4308	19526.96	221.4408	19522.79	228.0411
221.6851	19532.97	221.6881	19533.9	221.9619	19536.64	221.6786	19527.72	221.689	19528.51	221.6906	19527.43	228.2905
221.9349	19533.06	221.9351	19534.16	222.2074	19535.07	221.943	19529.24	221.9322	19526.95	221.94	19524.77	228.5364
222.1875	19533.88	222.1879	19534.46	222.4641	19534.95	222.1805	19527.35	222.1852	19527.48	222.185	19525.44	228.7929
222.4326	19533.72	222.4359	19532.35	222.7116	19535.88	222.4344	19527.03	222.4311	19527.53	222.4412	19528.18	229.0405
222.6846	19533.76	222.6843	19535.14	222.9633	19533.31	222.6817	19524.01	222.6892	19527.68	222.6882	19524.05	229.292
222.9334	19532.58	222.9345	19531.61	223.2096	19535.62	222.944	19527.74	222.9324	19527.03	222.9396	19527.18	229.5352
223.1874	19532.16	223.1869	19534.85	223.4665	19534.88	223.179	19527.79	223.1841	19526.97	223.1848	19527.68	229.7923
223.4344	19533.86	223.4364	19535.67	223.7102	19533.43	223.4334	19527.19	223.4321	19528.61	223.44	19527.75	230.0405
223.6879	19534.3	223.6879	19535.28	223.9627	19534.88	223.6789	19526.1	223.691	19527.18	223.6908	19525.82	230.2914
223.9348	19535.42	223.9379	19534.58	224.2091	19532.64	223.9429	19524.84	223.9316	19528.16	223.9398	19526.87	230.5399
224.1913	19533.69	224.1879	19535.22	224.4678	19534.1	224.1821	19529.24	224.1836	19528.31	224.1857	19526.41	230.7921
224.4367	19531.72	224.439	19532.44	224.7104	19534.67	224.4336	19528.17	224.4322	19525.32	224.4437	19525.41	231.0418
224.685	19533.44	224.6878	19535.11	224.9615	19533.93	224.68	19528.07	224.6876	19526.48	224.6909	19527.11	231.2898
224.9347	19533.45	224.939	19534.77	225.21	19534.87	224.9438	19527.41	224.9329	19528.89	224.9405	19527.67	231.5375
225.1917	19534.72	225.1874	19534.67	225.4662	19533.57	225.1802	19526.58	225.1864	19525.55	225.1869	19527.74	231.7927
225.4344	19533.35	225.4369	19535.16	225.711	19532.17	225.4352	19526.89	225.4308	19528.29	225.4415	19528.17	232.0426
225.6863	19533.35	225.6888	19533.14	225.9612	19533.78	225.6838	19528.12	225.6897	19528.38	225.6915	19526.21	232.2942
225.9351	19532.57	225.9358	19533.99	226.2104	19532.06	225.9414	19527.72	225.9335	19526.01	225.943	19526.21	232.5374
226.1899	19535.27	226.1897	19533.2	226.4685	19532.59	226.1809	19528.63	226.1879	19527.96	226.1856	19528.18	232.792

226.4348	19530.38	226.4376	19535.97	226.7126	19533.95	226.4389	19527.46	226.4318	19527.54	226.4417	19529.88	233.0426
226.6863	19533.27	226.688	19533.23	226.9617	19535.16	226.6819	19525.43	226.6908	19527.3	226.6916	19526.99	233.2966
226.9346	19533.87	226.9372	19534.16	227.2118	19536.84	226.9429	19525.23	226.9325	19525.99	226.9427	19527.56	233.5424
227.1913	19532.11	227.1886	19533.2	227.4662	19533.57	227.1804	19526.85	227.188	19529.9	227.1858	19529.34	233.7948
227.437	19534.86	227.4398	19535.34	227.7118	19532.87	227.4368	19525.62	227.4361	19527.49	227.4424	19528.13	234.0418
227.6874	19533.72	227.687	19533.69	227.9646	19534.84	227.6819	19525.61	227.6906	19526.99	227.6908	19524.08	234.2908
227.9387	19535.72	227.9359	19531.1	228.2108	19534.85	227.9443	19525.96	227.9318	19529.83	227.9433	19527.81	234.5376
228.1929	19535.19	228.1891	19533.93	228.4668	19533.98	228.1829	19526.07	228.1862	19529.67	228.1866	19526.71	234.7933
228.4363	19534.15	228.4427	19535.23	228.7128	19534.98	228.4373	19525.88	228.4335	19526.31	228.4414	19527.02	235.0442
228.6945	19535.37	228.689	19533.9	228.9658	19534	228.6819	19527.42	228.6941	19528.45	228.6935	19526.51	235.2925
228.9364	19532.99	228.9363	19534.2	229.2135	19535.59	228.945	19528.08	228.9335	19527.86	228.9418	19524.86	235.5382
229.1949	19532.41	229.1899	19532.62	229.4694	19533.51	229.1828	19525.77	229.1885	19527.96	229.1906	19526.21	235.7963
229.4362	19534.81	229.4424	19534.08	229.7121	19534.31	229.4383	19526.92	229.4369	19525.71	229.4443	19525.03	236.0438
229.6883	19534.92	229.6904	19530.76	229.9647	19533.8	229.6822	19531.11	229.6898	19530.75	229.695	19526.52	236.2926
229.938	19532.96	229.9362	19530.06	230.2129	19534.85	229.9445	19524.56	229.9345	19527.51	229.9438	19526.88	236.5394
230.1923	19533.21	230.1893	19534.07	230.4733	19533.96	230.1839	19526.87	230.1889	19526.55	230.1881	19526.12	236.7945
230.4371	19532.07	230.4403	19532.6	230.7122	19532.93	230.4377	19527.44	230.4354	19528.12	230.4458	19526.68	237.0424
230.6895	19530.65	230.6897	19535.06	230.9657	19537.64	230.6852	19530.28	230.6947	19528.81	230.6943	19525.15	237.2939
230.9381	19532.74	230.9379	19534.53	231.2126	19536.71	230.9454	19526.56	230.9352	19526.99	230.9429	19527.87	237.5384
231.1965	19531.51	231.193	19535.17	231.4713	19535.58	231.1851	19527.89	231.1885	19526.15	231.1879	19526.86	237.7931
231.4373	19534.22	231.4393	19535.29	231.7128	19533.97	231.4381	19524.98	231.4347	19527.08	231.4438	19527.67	238.0466
231.6875	19533.51	231.6906	19533.26	231.966	19532.78	231.6838	19526.89	231.6929	19527.76	231.6942	19525.3	238.2974
231.9364	19534.69	231.9388	19531.22	232.2128	19535.05	231.946	19526.1	231.9343	19525.66	231.9429	19527.68	238.5415
232.197	19533.28	232.1904	19532.12	232.4701	19533.12	232.1842	19526.81	232.1872	19526.23	232.1885	19525.86	238.794
232.4369	19535.61	232.4415	19533.19	232.7119	19534.04	232.4389	19525.09	232.4345	19526.93	232.4456	19527.64	239.0426
232.6878	19533.17	232.6917	19531.69	232.9656	19532.74	232.6845	19527.1	232.6907	19529.68	232.6952	19524.65	239.2937
232.9378	19530.2	232.9397	19534.22	233.2148	19532.33	232.9485	19528.32	232.9359	19528.51	232.944	19526.34	239.5417
233.1953	19531.54	233.191	19531.05	233.4721	19536.17	233.1846	19527.66	233.1882	19528.76	233.1895	19525.91	239.8095
233.4399	19532.89	233.4419	19533.88	233.7142	19533.67	233.4382	19527.14	233.4354	19525.62	233.4461	19527.46	240.0478
233.6894	19533.49	233.6901	19530.79	233.9658	19533.25	233.6832	19528.53	233.6964	19529.63	233.6973	19525.79	240.2942
233.9381	19530.32	233.9388	19534.16	234.2144	19536.94	233.9446	19527.18	233.9362	19527.84	233.9454	19526.32	240.5376
234.1962	19534.74	234.1915	19530.43	234.4712	19534.52	234.1896	19530.01	234.1879	19529.29	234.1918	19527.86	240.7937
234.4394	19531.72	234.443	19533.1	234.7135	19532.56	234.4384	19526.42	234.4371	19527.41	234.4479	19524.95	241.0411
234.6912	19533.42	234.6902	19534.67	234.9669	19534.33	234.6844	19526.98	234.6946	19525.3	234.6956	19527.45	241.2909
234.941	19534.58	234.9409	19532.42	235.2128	19535.46	234.9457	19529.07	234.938	19526.37	234.9462	19524.82	241.5369
235.1965	19535.29	235.1972	19534.82	235.4717	19536.4	235.1856	19525.34	235.1889	19528.23	235.19	19529.45	241.7922
235.4385	19531.18	235.444	19530.75	235.7167	19533.51	235.4386	19524.5	235.4364	19526	235.4449	19527.16	242.0407
235.6916	19534.23	235.6916	19531.08	235.9686	19535.74	235.6835	19526.08	235.7022	19524.24	235.6955	19527.35	242.2905
235.9411	19529.75	235.9406	19533.63	236.2149	19534.76	235.9477	19526.54	235.941	19523.98	235.9451	19527.14	242.5377
236.1971	19532.95	236.1952	19532.61	236.4731	19536.82	236.1881	19527.19	236.1888	19526.07	236.1903	19524.62	242.7936
236.4415	19533.58	236.4401	19533.76	236.7091	19534.69	236.4392	19527.2	236.4385	19527.86	236.4474	19526.34	243.0442
236.6916	19534	236.6932	19534.25	236.971	19532.79	236.6858	19527.15	236.6931	19525.15	236.6938	19526.26	243.2926
236.9396	19533.18	236.9411	19532.54	237.2156	19533.18	236.9469	19525.34	236.9395	19528.03	236.9455	19524.09	243.5485
237.2022	19535.41	237.1944	19533.91	237.4748	19533.46	237.1879	19525.05	237.1887	19527.71	237.192	19526.6	243.8017
237.4449	19532.62	237.4445	19531.75	237.7129	19537.98	237.4403	19529.5	237.4376	19525.16	237.4472	19524.87	244.0437
237.6921	19532.58	237.6995	19535.72	237.9696	19534.47	237.6874	19525.26	237.693	19525.58	237.6986	19525.32	244.2926
237.939	19530.96	237.9391	19535.21	238.2186	19536.65	237.9508	19526.9	237.9391	19529.74	237.9457	19528.51	244.5383
238.1966	19533.06	238.1941	19533.55	238.4739	19533.5	238.1938	19528.28	238.1877	19528.17	238.1949	19530.46	244.7927
238.4391	19533.98	238.4471	19531.05	238.7152	19532.85	238.4403	19525.9	238.436	19529.77	238.4452	19527.62	245.0403
238.6918	19530.7	238.6921	19531.89	238.9719	19535.77	238.6869	19526.51	238.6959	19528.64	238.6964	19528.24	245.2908
238.9412	19532.78	238.9416	19532.29	239.2168	19533.36	238.949	19525.81	238.9377	19527.78	238.9478	19527.08	245.5381
239.1994	19530.75	239.1943	19534.93	239.4756	19534.21	239.1886	19526.71	239.1918	19529.31	239.1857	19528.66	245.7929
239.4431	19533.22	239.4452	19534.13	239.7167	19535.4	239.4421	19527.21	239.4377	19526.52	239.4481	19526.76	246.0438
239.6923	19532.42	239.6923	19531.01	239.9706	19533.72	239.6869	19528.43	239.6942	19528.36	239.6924	19526.91	246.2913
239.9413	19529.42	239.9413	19532.94	240.2167	19533.09	239.9479	19526.65	239.9383	19526.63	239.9486	19526.91	246.5371
240.199	19534.13	240.1958	19532.86	240.4754	19535.12	240.1889	19525.81	240.1898	19527.03	240.1873	19525.15	246.7935
240.4469	19530.88	240.4418	19534.78	240.7125	19534.6	240.4415	19527.46	240.4371	19526.52	240.4476	19525.38	247.0421
240.6876	19531.94	240.6874	19531.38	240.9661	19533.11	240.6829	19528	240.6913	19526.22	240.6869	19527.67	247.2929
240.9371	19533.42	240.9386	19528.39	241.2143	19533.97	240.9458	19526.54	240.9344	19527.26	240.9467	19530.62	247.5382
241.2026	19533.28	241.1945	19535.51	241.4799	19534.66	241.185	19526.53	241.1862	19527.62	241.1886	19528.86	247.7925
241.4364	19533.9	241.4542	19532.6	241.7144	19534.69	241.4371	19526.73	241.434	19531.12	241.4443	19525.61	248.042
241.689	19532.84	241.6951	19532.06	241.9705	19534.5	241.6837	19526.81	241.6892	19526.2	241.6943	19527.91	248.2921
241.9385	19533.14	241.9379	19534.45	242.217	19532.23	241.9441	19529.98	241.934	19525.38	241.9431	19524.55	248.5395
242.1923	19532.56	242.1928	19533.89	242.4731	19534.41	242.1865	19525.55	242.1864	19527.33	242.1887	19525.25	248.7965
242.4371	19534.95	242.4444	19532.65	242.7129	19532.81	242.4382	19525.56	242.4377	19528.38	242.446	19525.46	249.0429
242.6874	19530.62	242.6898	19533.07	242.9696	19535.9	242.6838	19526.39	242.6912	19526.77	242.6936	19524.61	249.2948
242.9378	19530.57	242.9372	19535.12	243.2129	19534.48	242.9452	19527.52	242.9358	19529.5	242.9429	19526.99	249.5389
243.1924	19531.48	243.1905	19533.66	243.4718	19533.84	243.1856	19526.63	243.1871	19530.38	243.1891	19526.24	249.7943
243.4382	19531.82	243.4456	19534.35	243.7155	19534.57	243.4414	19526.72	243.4355	19528.77	243.4659	19524.72	250.0456
243.6925	19532.48	243.6902	19531.57	243.9685	19534.04	243.6844	19528.64	243.6932	19530.44	243.7012	19525.33	250.2941

243.9384	19531.65	243.9379	19533.96	244.2132	19534.13	243.9555	19526.66	243.942	19526.43	243.9441	19526.02	250.5401
244.1956	19530.99	244.1956	19533.34	244.4736	19535.45	244.1906	19526.75	244.1929	19527.38	244.1913	19527.5	250.7941
244.443	19533.43	244.4436	19535.67	244.7187	19535.8	244.4434	19525.75	244.4408	19527.37	244.4457	19526.82	251.0415
244.6884	19534.57	244.6888	19531.85	244.9684	19535.53	244.6914	19524.8	244.7032	19527.79	244.6957	19523.5	251.2951
244.9389	19533.35	244.9407	19530.53	245.2139	19534.45	244.9479	19525.14	244.9452	19528.81	244.9457	19530.03	251.5401
245.1947	19534.96	245.1919	19535.51	245.4734	19534.14	245.1899	19527.29	245.1872	19526.78	245.1895	19526.78	251.795
245.4375	19529.65	245.4423	19532.12	245.7147	19535.11	245.4411	19527.02	245.4423	19526.41	245.4442	19526.4	252.0445
245.689	19533.11	245.69	19531.55	245.9702	19535.16	245.689	19525.57	245.6951	19528.2	245.6954	19525.71	252.2945
245.9387	19533.84	245.9417	19534.79	246.2148	19533.86	245.9456	19528.98	245.9397	19526.8	245.947	19524.65	252.5416
246.193	19532.26	246.1931	19532.24	246.4737	19532.41	246.1864	19529.63	246.1914	19529.04	246.1908	19526.15	252.7962
246.4389	19533.84	246.4443	19532.13	246.714	19533.13	246.4406	19525.75	246.4381	19529.03	246.4465	19525.42	253.0439
246.6904	19532.8	246.689	19534.17	246.9696	19535.71	246.6888	19527.64	246.6945	19527.45	246.6963	19525.22	253.2966
246.9394	19532.3	246.9408	19534.26	247.2164	19535.4	246.949	19523.6	246.9372	19528.46	246.947	19527.01	253.5425
247.1938	19533.03	247.1937	19530.47	247.4765	19535.79	247.1868	19527.82	247.1913	19526.34	247.1901	19525.39	253.7968
247.4404	19532.36	247.4433	19534.77	247.7145	19535.27	247.4412	19526.99	247.4383	19526.07	247.4458	19527.06	254.0414
247.6898	19532.66	247.6911	19531.62	247.9782	19534.25	247.6945	19526.35	247.6938	19528.63	247.6946	19524.09	254.2945
247.9432	19535.63	247.9464	19531.5	248.2151	19533.81	247.953	19525.52	247.937	19528.19	247.9471	19523.97	254.541
248.1946	19533.28	248.1933	19533.33	248.4745	19534.03	248.1903	19527.44	248.1881	19529.94	248.1922	19525.4	254.7968
248.4382	19532.98	248.4449	19533.59	248.7149	19534.86	248.4445	19527.26	248.4384	19526.51	248.4468	19529.21	255.0481
248.6917	19533.87	248.6919	19533.2	248.9689	19534.45	248.6887	19525.09	248.6954	19525.7	248.6952	19529	255.2908
248.9397	19532.18	248.9419	19533.02	249.2142	19533.02	248.9485	19525.16	248.9381	19526.25	248.947	19526.92	255.5443
249.199	19533.75	249.1956	19533.9	249.4769	19532.01	249.188	19524.85	249.1894	19530.43	249.1918	19529.36	255.7956
249.4407	19532.41	249.4512	19532.64	249.7185	19533.21	249.4439	19525.37	249.4387	19528.04	249.4484	19526.41	256.0425
249.6917	19533.21	249.7081	19534.41	249.9702	19533.44	249.689	19527.44	249.6941	19528.82	249.6969	19527.54	256.2952
249.9399	19532.95	249.9425	19532.36	250.2156	19533.77	249.9484	19524.45	249.9382	19526.63	249.9459	19522.32	256.543
250.1969	19535.89	250.1944	19535.31	250.4749	19535.58	250.189	19525.14	250.1911	19527.84	250.1905	19526.04	256.7976
250.4406	19529.62	250.4437	19534.5	250.717	19533.28	250.4419	19525.96	250.4448	19524.59	250.4488	19525.12	257.0457
250.6909	19531.5	250.6934	19537.34	250.9853	19534.63	250.6894	19526.16	250.7055	19525.62	250.6967	19527.5	257.2962
250.9401	19532.36	250.9449	19536.06	251.2193	19534.59	250.9487	19523.84	250.95	19530.33	250.9472	19528.36	257.5437
251.1965	19532.35	251.1959	19532.09	251.4742	19535.69	251.1898	19525.62	251.1896	19527.54	251.1909	19527.58	257.7964
251.4421	19534.02	251.4469	19534.18	251.7162	19535.21	251.4424	19526.76	251.4438	19527.37	251.4463	19527.66	258.0474
251.6916	19533.5	251.6934	19531.1	251.9702	19533.76	251.6898	19525.76	251.6926	19529.02	251.6972	19524.25	258.2968
251.9401	19531.3	251.9444	19534	252.2167	19533.19	251.9494	19525.7	251.94	19528.37	251.9478	19526.92	258.5439
252.1996	19533.79	252.1939	19530.98	252.475	19534.68	252.1861	19527.62	252.191	19524.94	252.1967	19527.91	258.7978
252.4405	19533.19	252.4459	19532.34	252.716	19534.98	252.4412	19526.79	252.4401	19527.88	252.447	19525.2	259.0456
252.6919	19531.94	252.6942	19533.77	252.9692	19535.59	252.6877	19525.13	252.6948	19527.1	252.6966	19527.34	259.2969
252.9429	19533.22	252.9436	19534.08	253.2213	19537.37	252.9498	19527.39	252.9399	19529.47	252.9495	19525.51	259.544
253.2002	19532.22	253.1943	19534.84	253.476	19532.45	253.188	19528.51	253.191	19525.95	253.1944	19526.93	259.8027
253.4445	19531.21	253.4493	19535.01	253.7162	19536.09	253.4429	19526.29	253.4407	19526.77	253.4499	19525.74	260.0506
253.6922	19534.26	253.695	19533.94	253.9694	19533.91	253.6888	19523.94	253.7009	19526.29	253.6997	19526.67	260.2974
253.9431	19533.31	253.9442	19534.52	254.2158	19534.73	253.9481	19527.08	253.9398	19526.59	253.948	19527.57	260.5433
254.1967	19531.33	254.195	19535.32	254.4765	19534.5	254.1928	19526.66	254.1907	19527.45	254.1938	19526.12	260.7993
254.4443	19530.11	254.4469	19532.64	254.7161	19533.3	254.4434	19525.38	254.4422	19528.05	254.4508	19524.38	261.0415
254.6953	19531.74	254.6936	19532.5	254.9716	19529.55	254.6881	19526.25	254.6947	19526.1	254.699	19527.17	261.3011
254.9433	19532.61	254.9449	19534.58	255.2166	19534.07	254.9496	19526.84	254.9385	19528.51	254.948	19525.56	261.5417
255.1962	19533.38	255.1961	19534.5	255.4806	19537.14	255.1888	19527.75	255.1923	19525.57	255.1955	19526.52	261.7979
255.4445	19534.15	255.4485	19531.63	255.7244	19535.38	255.4439	19526.67	255.4395	19529.33	255.4509	19526.09	262.0474
255.6948	19533.82	255.6937	19532.95	255.9705	19535.26	255.6896	19525.75	255.6955	19528.24	255.701	19525.89	262.2959
255.9429	19531.31	255.9442	19534.96	256.218	19535.55	255.9492	19528.17	255.9418	19526.44	255.9507	19525.19	262.5495
256.1994	19532.06	256.1995	19532.72	256.4776	19535.18	256.1907	19527.3	256.1919	19526.89	256.194	19527.41	262.7979
256.4446	19531.53	256.4479	19530.99	256.7129	19533.27	256.4429	19525.39	256.444	19526.08	256.4489	19527.4	263.0502
256.695	19531.99	256.697	19533.73	256.9715	19535.24	256.6912	19526.09	256.6945	19524.4	256.7014	19526	263.2986
256.942	19532.02	256.9453	19534.31	257.2203	19534.59	256.9519	19526.55	256.945	19528	256.9493	19527.55	263.5425
257.2019	19533.18	257.1967	19530.75	257.4778	19535.71	257.1938	19527.36	257.1921	19526.54	257.1952	19523.53	263.8018
257.4449	19529.87	257.448	19530.18	257.7147	19537.1	257.4471	19527.52	257.4412	19528.65	257.4478	19527.03	264.0528
257.695	19531.19	257.6954	19532.8	257.9704	19535.05	257.6901	19528.5	257.6962	19529.01	257.6997	19525.9	264.3002
257.9437	19534.06	257.9449	19532.47	258.2198	19533.98	257.97	19524.95	257.9411	19527.25	257.9534	19522.65	264.5438
258.1969	19532.87	258.1964	19533.13	258.4774	19535.06	258.2076	19527.62	258.1928	19527.42	258.1947	19525.73	264.8021
258.4525	19533.36	258.4478	19530.4	258.7154	19537.23	258.4507	19527.5	258.4402	19526.15	258.4503	19525.67	265.0478
258.7039	19531.66	258.6976	19535.58	258.9762	19533.32	258.6887	19528.77	258.7005	19527.37	258.6994	19526.32	265.3002
258.9469	19532.17	258.9452	19533.67	259.2158	19533.6	258.9539	19525.78	258.9415	19527.85	258.9501	19526.43	265.5434
259.2006	19535.39	259.196	19534.43	259.4774	19533.53	259.1968	19526.93	259.1947	19525.62	259.1962	19526.12	265.8013
259.4489	19529.78	259.4431	19534.45	259.7181	19531.83	259.4484	19524.8	259.4413	19528.1	259.4524	19524.75	266.045
259.6938	19534.96	259.696	19535.22	259.9745	19533.95	259.6908	19524.96	259.6982	19527.37	259.7004	19526.85	266.3025
259.943	19532.98	259.9456	19534.04	260.2204	19535.55	259.9504	19525.89	259.9407	19525.9	259.9505	19525.48	266.5456
260.2001	19532.2	260.1969	19534.7	260.4795	19533.16	260.1908	19529.09	260.1941	19525.72	260.1967	19525.36	266.8022
260.4493	19532.3	260.4678	19532.81	260.7204	19535.68	260.4456	19526.17	260.4422	19528.75	260.4524	19526.76	267.0507
260.6957	19533.96	260.7092	19532.04	260.9739	19534.89	260.6908	19525.6	260.696	19526.77	260.7029	19524.24	267.3055
260.9427	19533.04	260.9502	19530.57	261.222	19535.51	260.9517	19527.15	260.9426	19526.02	260.9506	19524.4	267.5512
261.1979	19533.93	261.1942	19533.91	261.4792	19532.9	261.1967	19525.08	261.1933	19524.84	261.1959	19524.52	267.8043

261.4665	19533.81	261.4492	19531.51	261.7187	19534.27	261.4459	19526.94	261.4408	19529	261.4518	19528.56	268.0542
261.712	19529.91	261.698	19534.08	261.9735	19534.34	261.6911	19525.96	261.6972	19526.92	261.7026	19524.78	268.3074
261.9559	19534.37	261.9449	19532.88	262.2231	19534.61	261.9524	19526.13	261.9416	19528.31	261.9529	19526.24	268.5566
262.2041	19531.07	262.1986	19535.2	262.4799	19533.16	262.1928	19527.34	262.1932	19527.1	262.1972	19524.16	268.8095
262.4565	19534	262.4499	19533.92	262.7206	19534.95	262.4454	19525.56	262.4426	19526.05	262.4517	19526.46	269.0554
262.7027	19534.29	262.6965	19533.66	262.9732	19535.52	262.6934	19525.04	262.6977	19526.25	262.7034	19526.81	269.3097
262.9489	19531.18	262.9627	19532.5	263.2218	19533.9	262.9528	19524.59	262.9429	19530.01	262.9504	19529.9	269.5571
263.1998	19534.95	263.2167	19531.78	263.481	19532.24	263.1934	19525.35	263.195	19527.62	263.196	19526.05	269.8067
263.4489	19533.14	263.4652	19531.54	263.7225	19533.2	263.4469	19523.53	263.4526	19528.01	263.4526	19524.74	270.0528
263.7023	19533.13	263.71	19533.78	263.9743	19534.77	263.6927	19525.81	263.7071	19527.48	263.7011	19523.6	270.3048
263.9479	19530.74	263.9557	19532.3	264.2207	19533.22	263.9517	19525.6	263.9593	19525.32	263.9489	19526.79	270.5507
264.2009	19531.31	264.2	19532.9	264.4799	19537.17	264.1971	19527.8	264.2063	19527.26	264.1969	19526.48	270.803
264.449	19532.66	264.4469	19536.25	264.7222	19533.13	264.4456	19526.58	264.4499	19527.15	264.4552	19526.41	271.0521
264.6991	19533.61	264.6986	19535	264.9736	19535.53	264.6927	19524.73	264.7043	19525.23	264.7042	19526.17	271.3063
264.956	19533.84	264.9427	19531.66	265.2226	19533.28	264.9554	19526.99	264.9505	19525.59	264.9532	19525.96	271.5552
265.2106	19533.22	265.2007	19531.68	265.4803	19533.19	265.1996	19526.01	265.1988	19525.2	265.1978	19525.03	271.809
265.4689	19531.57	265.4481	19532.51	265.7216	19533.61	265.4462	19525.7	265.4451	19528.39	265.454	19526.19	272.0579
265.719	19535.22	265.697	19531.37	265.9761	19535.27	265.7112	19525.17	265.7022	19526.92	265.7061	19527.66	272.3142
265.9658	19532.11	265.9485	19529.25	266.2206	19532.71	265.9659	19524.36	265.9469	19526.02	265.9546	19524.4	272.5633
266.2217	19531.3	266.2031	19533.46	266.4809	19532.73	266.2108	19524.65	266.1987	19530.8	266.201	19524.04	272.8169
266.4744	19533.58	266.4507	19534.77	266.724	19532.02	266.4593	19526.63	266.45	19524.25	266.4515	19524.38	273.0706
266.9509	19534.46	266.6949	19532.21	266.9761	19534.78	266.7053	19526.81	266.7002	19527.98	266.708	19524.24	273.553
267.2052	19532.52	266.9474	19531.29	267.221	19534.91	266.966	19526.89	266.9455	19526.52	266.9655	19522.27	273.8068
267.4549	19532.98	267.2026	19530.46	267.4835	19533.87	267.2147	19526.37	267.1994	19524.32	267.2162	19523.33	274.0604
267.7034	19529.99	267.4552	19533.11	267.728	19534.88	267.464	19526.36	267.4452	19526.68	267.4673	19527.36	274.3163
267.9535	19532.28	267.7021	19530.34	267.9744	19533.07	267.71	19525.34	267.7054	19525.94	267.7169	19524.33	274.567
268.2057	19530.26	267.9463	19531.48	268.2232	19535.07	267.9667	19529.72	267.9543	19527.67	267.9682	19528.22	274.8205
268.4557	19532.82	268.2039	19532.36	268.4762	19534.78	268.2141	19522.68	268.2018	19526.49	268.2152	19528.41	275.0723
268.7068	19531.05	268.4543	19533.66	268.7213	19534.69	268.4655	19527.29	268.4508	19526.95	268.4667	19527.52	275.3302
268.9559	19529.95	268.701	19531.25	268.9763	19532.03	268.7012	19524.8	268.7084	19523.74	268.7195	19523.04	275.8394
269.2133	19530.67	268.9468	19531.99	269.2239	19533.75	268.9551	19527.85	268.9562	19526.72	268.9716	19526.9	276.3285
269.4709	19531.4	269.2016	19532.79	269.4776	19534.87	269.1929	19526.7	269.2055	19527	269.221	19522.95	276.5788
269.7213	19533.51	269.4519	19531.92	269.7236	19533.41	269.4481	19525.31	269.4506	19526.88	269.4718	19524.01	276.8353
269.9701	19531.64	269.6999	19531.32	269.9724	19533.21	269.6944	19526.08	269.7112	19525.74	269.9562	19525.6	277.0899
270.23	19531.95	269.948	19533.07	270.2238	19533.74	269.9546	19525.98	269.9595	19527.72	270.2044	19526.78	277.344
270.7107	19530.54	270.2027	19534.61	270.4803	19533.36	270.1944	19527.33	270.209	19528.85	270.4569	19523.55	277.8316
270.9608	19530.62	270.4531	19532.41	270.7265	19532.66	270.449	19525.99	270.4579	19528.35	270.7103	19526.15	278.0864
271.2165	19530.78	270.6999	19533.23	270.9753	19531.61	270.6972	19524.77	270.7137	19527.97	270.9618	19525.05	278.3408
271.4706	19530.72	270.9477	19531.57	271.2253	19533.89	270.9563	19524.5	270.9607	19529.01	271.209	19526.43	278.5979
271.7231	19530.49	271.2004	19531.96	271.481	19533.07	271.1952	19527.36	271.2139	19526.53	271.4631	19525.99	279.0867
271.9733	19533.9	271.4551	19533.45	271.7281	19532.91	271.4503	19526.66	271.464	19528.86	271.7158	19525.88	279.3431
272.2286	19534.22	271.7039	19529.75	271.9785	19532.52	271.6942	19524.84	271.7207	19525.25	271.9671	19526.62	279.5956
272.4869	19530.73	271.9538	19533.21	272.2263	19535.07	271.9555	19525.91	271.9702	19526.95	272.2168	19526.17	279.8515
272.9679	19529.71	272.2052	19532.22	272.4824	19531.46	272.1963	19524.55	272.2241	19526.63	272.4694	19528.19	280.3398
273.2241	19532.94	272.4585	19532.92	272.7291	19533.39	272.4498	19524.73	272.711	19527.95	272.7227	19525.53	280.5929
273.4818	19532.2	272.709	19530.64	272.9798	19534.77	272.6953	19526.85	272.9625	19528.37	272.9766	19523.36	280.8481
273.7354	19533.74	272.9576	19534.04	273.2295	19534.71	272.9562	19528.18	273.2154	19525.65	273.4637	19524.72	281.1052
273.9866	19532.94	273.2101	19529.92	273.4884	19534.46	273.1961	19525.63	273.4641	19526.64	273.7227	19527.26	281.5919
274.2481	19531.56	273.4632	19531.62	273.7395	19535.1	273.4506	19525.02	273.7218	19528.04	273.9776	19524.25	281.8501
274.7367	19530.92	273.7139	19531.07	273.9916	19532.21	273.6972	19527.96	273.9718	19527.52	274.2307	19522.98	282.1045
274.9897	19534.02	273.9634	19530.65	274.2405	19533.06	273.9543	19528.23	274.2303	19523.8	274.7184	19525.53	282.3652
275.2494	19530.56	274.2158	19533.33	274.4999	19532.64	274.1984	19528.49	274.718	19527.02	274.9722	19524.14	282.8565
275.501	19532.77	274.4685	19532.42	274.9872	19530.37	274.4495	19526.81	274.9689	19528.38	275.2233	19524.2	283.1122
275.7576	19530.87	274.7207	19533.26	275.2342	19536.09	274.6969	19527.07	275.2226	19530.68	275.4787	19528.07	283.3678
276.2477	19534.94	274.9751	19533.37	275.4974	19535.44	274.9561	19525.9	275.473	19529.02	275.7446	19525.31	283.6235
276.5015	19530.92	275.4605	19531.59	275.7484	19531.6	275.1952	19527.9	275.7316	19527.28	276.2296	19523.8	284.1171
276.7565	19534.47	275.717	19532.52	275.9991	19534.38	275.4494	19524.37	275.9844	19529.54	276.486	19527.19	284.3735
277.0103	19530.59	275.9686	19533.33	276.2494	19533.85	275.698	19524.19	276.4651	19527.93	276.7399	19524.66	284.6286
277.27	19530.29	276.2254	19532.98	276.5062	19533.52	275.9565	19527.27	276.7268	19525.64	276.9965	19528.54	285.1199
277.7546	19530.28	276.4802	19529.24	276.9925	19534.53	276.1975	19525.12	276.9776	19527.36	277.2507	19527.02	285.3811
278.0093	19533.28	276.9643	19533.2	277.2436	19533.77	276.4516	19527.06	277.2353	19527.57	277.7411	19524.98	285.6392
278.2726	19530.88	277.2232	19532.08	277.5022	19534.06	276.6986	19528.15	277.4876	19526.76	277.9991	19524.26	286.1331
278.5401	19532.58	277.4837	19532.88	277.7544	19533.92	276.9574	19526.05	277.9678	19525.08	278.254	19524.18	286.3907
279.0237	19531.88	277.9732	19533.17	278.0073	19533.25	277.1976	19522.83	278.2266	19529.32	278.7427	19529.54	286.6495
279.286	19532.13	278.2323	19532.46	278.2605	19534.55	277.4519	19525.35	278.479	19525.97	278.9983	19526.76	287.1442
279.5437	19534.97	278.4937	19530.48	278.7464	19535.05	277.6981	19525.21	278.7414	19528.15	279.2544	19523.88	287.4019
280.0277	19529.17	278.9804	19532.75	279.0043	19533.37	277.9576	19523.26	279.2256	19525.35	279.5144	19526.48	287.6639
280.2898	19531.97	279.2351	19530.9	279.2575	19533.91	278.1985	19525.28	279.4782	19526.93	280.0058	19525.58	288.1602
280.5469	19531.29	279.4923	19531.44	279.5167	19532.2	278.4527	19527.28	279.7401	19529.67	280.2638	19525.4	288.4078
281.03	19532.52	279.746	19531.46	280.008	19532.81	278.6989	19527.13	279.9955	19528.86	280.5201	19523.56	288.6554

281.2912	19533.43	279.9985	19533.95	280.2631	19533.31	278.9567	19526.48	280.4768	19526.97	281.0098	19526.95	288.9003
281.5471	19534.63	280.4867	19533.54	280.5211	19535.65	279.1991	19527.07	280.7386	19525.77	281.2643	19524.85	289.157
281.8043	19533.71	280.7434	19535.3	281.0097	19535.25	279.4526	19526.05	280.9919	19523.69	281.524	19528.71	289.4065
282.296	19529.52	280.9963	19531.92	281.2619	19533.81	279.6996	19527.55	281.2494	19528.98	282.0132	19525.59	289.66
282.5504	19532.8	281.2571	19530.5	281.521	19534.83	279.962	19525.67	281.7518	19526.23	282.2678	19526.3	289.906
282.8106	19532.23	281.7539	19531.08	281.7728	19533.54	280.2146	19529.05	282.0118	19527.25	282.5254	19524.52	290.1765
283.302	19532.83	282.0276	19532.26	282.0289	19534.96	280.4701	19526.93	282.4958	19528.72	282.7858	19526.42	290.67
283.5586	19532.76	282.517	19533.54	282.5213	19534.4	280.7291	19527.25	282.7585	19527.61	283.2741	19523.42	290.9177
283.821	19532.41	282.7856	19530.62	282.7774	19536.09	281.2112	19526.85	283.0153	19525.67	283.5326	19528.2	291.1695
284.3138	19531.5	283.2877	19531.29	283.0329	19532.83	281.4692	19524.18	283.4973	19525.78	283.79	19525.86	291.4174
284.5697	19534.8	283.7863	19531.71	283.5275	19530.06	281.7259	19526.06	283.7632	19523.73	284.2783	19526.73	291.668
284.8299	19529.44	284.0484	19531.59	283.7819	19536.36	281.9925	19525.99	284.0156	19525.86	284.5379	19524.9	291.9181
285.3226	19532.39	284.5377	19532.03	284.0395	19534.66	282.4786	19523.88	284.5022	19527.26	284.796	19525.32	292.1715
285.581	19530.36	284.8279	19533.36	284.5331	19532.32	282.7354	19526.49	284.7655	19527.61	285.285	19525.71	292.4207
285.8408	19531.19	285.3246	19531.88	284.7996	19533.52	282.9993	19526.4	285.0251	19527.59	285.5455	19524.59	292.6729
286.3343	19529.96	285.5878	19530.62	285.2883	19533.96	283.4865	19528.72	285.5098	19526.82	285.8027	19528.25	292.9224
286.5906	19531.55	286.078	19531.81	285.5497	19534.79	283.7412	19526.75	285.7759	19525.78	286.2936	19526.8	293.1785
286.8514	19531.51	286.3439	19531.98	285.8064	19536.33	284.0118	19523.78	286.0331	19527.01	286.55	19525.49	293.4282
287.3441	19532.9	286.8358	19531.41	286.2948	19531.18	284.4975	19527.14	286.5159	19525.57	286.8099	19528.92	293.6826
287.6014	19534.07	287.1026	19533.7	286.5575	19532.66	284.7569	19527.69	286.7792	19526.45	287.2984	19525.29	294.1826
287.8616	19531.3	287.6056	19532.95	286.815	19531.53	285.0199	19524.48	287.0374	19526.53	287.5551	19525.73	294.4402
288.3543	19530.06	287.8681	19532.05	287.3052	19535.66	285.5056	19524.5	287.522	19525.24	287.8158	19524.37	294.9374
288.614	19532.12	288.3769	19532.39	287.5667	19536.7	285.7648	19525.63	287.7887	19528.75	288.3059	19525.2	295.1937
288.8746	19528.39	288.8694	19531.99	287.8266	19530.51	286.0349	19526.49	288.045	19528.14	288.5676	19522.96	295.4445
289.3681	19530.54	289.1267	19534.1	288.3202	19531.74	286.5215	19523.77	288.5297	19527.88	288.8337	19527.87	295.6992
289.6271	19533.5	289.391	19534.53	288.5701	19536.12	286.781	19525.16	288.7955	19526.88	289.3276	19525.08	296.1976
289.8879	19532.58	289.8825	19531.78	288.8169	19534.74	287.0431	19524.61	289.0554	19530.23	289.5886	19523.26	296.4538
290.3824	19532.18	290.1381	19533.82	289.0616	19533.83	287.5304	19526.16	289.539	19528.1	290.0802	19522.62	296.9473
290.6387	19532.8	290.4054	19532.25	289.3184	19534.36	287.789	19525.5	289.8037	19526.16	290.3406	19524.55	297.2043
290.903	19531.35	290.8986	19531.86	289.5691	19534.66	288.0526	19528.07	290.0615	19528.02	290.6041	19522.57	297.455
291.3966	19535.17	291.1593	19530.03	289.8167	19533.74	288.5395	19526.49	290.551	19524.99	291.1001	19524.36	297.7133
291.668	19531.55	291.6482	19533.44	290.0697	19534.43	288.7987	19528.24	290.8159	19526.99	291.3638	19526.03	298.2147
292.1567	19531.89	291.9101	19535.13	290.3268	19530.65	289.0617	19526.69	291.0744	19522.63	291.8554	19525.16	298.4662
292.4207	19532.35	292.1685	19532.48	290.5795	19533.41	289.5477	19527.02	291.5608	19526.3	292.1156	19525.35	298.7239
292.6829	19530.59	292.6599	19527.86	291.0686	19532.94	289.8069	19523.9	291.8275	19530.08	292.3793	19524.16	299.2263
293.1725	19529.41	292.9202	19529.05	291.3196	19534.43	290.0722	19523.5	292.3168	19525.02	292.8744	19525.63	299.4783
293.4384	19530	293.1912	19532.94	291.5703	19533.81	290.5629	19523.77	292.5758	19527.02	293.1343	19526.81	299.9836
293.9266	19533.01	293.6834	19534.31	291.8212	19535.32	290.8244	19524.5	292.8427	19531.03	293.6295	19524.22	300.4796
294.181	19530.79	293.943	19529.6	292.0675	19536.38	291.0847	19525.66	293.3329	19526.41	293.8952	19523.1	
294.4344	19529.33	294.2062	19534.14	292.3224	19532.04	291.5709	19527.02	293.5914	19526.47	294.3932	19524.85	
294.6858	19528.51	294.6984	19532.37	292.5735	19532.6	291.8314	19525.45	293.8591	19528.35	294.659	19525.05	
294.9399	19532.03	294.9571	19531.28	292.8233	19531.76	292.0937	19528.2	294.3487	19528.34	295.1497	19523.72	
295.195	19530.91	295.221	19533.25	293.0713	19532.34	292.5809	19524.56	294.609	19528.22	295.4159	19529.77	
295.6847	19529.53	295.7142	19533.94	293.3249	19534.4	292.8439	19525.25	294.8878	19526.58	295.9116	19521.69	
295.9363	19533.17	295.9753	19530.79	293.5786	19532.42	293.1097	19524.04	295.3782	19524.52	296.1714	19529.28	
296.1922	19533.04	296.4699	19531.48	293.8324	19535.74	293.598	19524.52	295.6398	19526.74	296.6677	19526.64	
296.4477	19531.93	296.7233	19533.38	294.3322	19532.75	293.8631	19526.45	295.9043	19529.95	296.9315	19526.59	
296.7017	19530.88	296.9748	19534.46	294.5875	19531.32	294.3496	19526.12	296.3938	19522.51	297.429	19525.96	
297.1939	19531.75	297.2294	19531.6	295.0827	19533.44	294.6128	19527.5	296.6567	19525.52	297.6954	19525.93	
297.4514	19532.21	297.7242	19531.45	295.335	19533.8	294.8808	19524.37	296.9218	19527.18	298.1875	19527.4	
297.7063	19532.47	297.9737	19529.74	295.5908	19532.31	295.3694	19523.39	297.4116	19524.64	298.4778	19524.01	
298.1986	19531.11	298.2276	19530.83	295.8422	19533.42	295.6297	19524.87	297.6736	19526.19	298.9727	19521.43	
298.4535	19531.5	298.4828	19533.34	296.0937	19534.01	295.8968	19526.81	298.1679	19528.35	299.2366	19525.68	
298.7072	19531.73	298.9746	19533.83	296.3481	19533.66	296.3867	19527.28	298.4319	19527.07	299.7326	19522.74	
298.9616	19530.78	299.2278	19533.94	296.8442	19534.21	296.6488	19529.15	298.9213	19527.26	299.9952	19526.46	
299.4541	19532.31	299.4833	19535.47	297.0944	19533.89	296.9207	19527.4	299.1871	19523.84	300.492	19525.66	
299.7082	19531.02	299.7356	19530.9	297.3499	19532.37	297.4116	19525.64	299.6789	19526.9			
299.9613	19533.42	299.9893	19530.86	297.6044	19532.32	297.6739	19524.94	299.9437	19528.48			
300.2185	19531.88	300.4861	19533.63	298.0976	19532.37	298.1626	19529.33	300.4333	19525.61			
				298.3532	19533.64	298.4259	19525.16					
				298.6108	19535.1	298.6888	19525.21					
				299.1043	19534.88	299.1819	19524.61					
				299.3592	19533.6	299.4531	19525.94					
				299.6148	19533.5	299.9505	19527.55					
				300.1139	19534.84	300.2146	19525.98					

			1.43									
			62.5									
$_4$	x_5		x_1		x_2		x_3		x_4		x_5	
P (cm^{-1})	t (s)	P (cm^{-1})	t (s)	P (cm^{-1})	t (s)	P (cm^{-1})	t (s)	P (cm^{-1})	t (s)	P (cm^{-1})	t (s)	P (cm^{-1})
19544.66	0	19543.01	0	19530.16	0	19530	0	19535.83	0	19533.59	0	19536.32
19546.54	0.70199	19543.68	0.66517	19529.02	0.66924	19533.19	0.6554	19531.35	0.65799	19534.46	0.65874	19534.69
19544.94	0.8583	19545.08	0.85418	19530.27	0.85325	19532.93	0.84777	19533.3	0.85689	19531.15	0.85428	19535.8
19541.2	1.10427	19543.04	1.10267	19531.79	1.10461	19532.6	1.10169	19534.59	1.10222	19535.76	1.10276	19537.75
19544.17	1.35314	19546.81	1.35012	19530.62	1.35318	19534.09	1.34754	19531.53	1.35463	19532.93	1.35224	19538.48
19541.91	1.60234	19544.94	1.60235	19532.82	1.60665	19530.59	1.59674	19534.72	1.60045	19534.5	1.60267	19532.19
19542.56	1.85424	19544.23	1.8542	19532.85	1.85134	19533.64	1.84499	19534.68	1.85506	19532.25	1.85063	19534.69
19544.92	2.10302	19546.65	2.10079	19531.14	2.10237	19533	2.09685	19531.11	2.10176	19533.15	2.10115	19534.73
19542.31	2.35455	19546.76	2.35043	19532	2.35154	19531.57	2.34696	19533.06	2.35376	19537.03	2.35276	19534.54
19542.66	2.60211	19544.71	2.60161	19531.28	2.60921	19530.84	2.59735	19533.06	2.60203	19535.31	2.60281	19538.17
19542.63	2.85377	19544.48	2.85859	19533.92	2.85274	19532.8	2.8465	19531.71	2.8542	19532.3	2.85873	19536.64
19540.42	3.10234	19543.79	3.10378	19532	3.10292	19533.77	3.09591	19531.97	3.10062	19532.84	3.10048	19537.27
19543.05	3.35617	19545.72	3.35082	19530.84	3.35341	19530.68	3.34629	19533.52	3.35493	19535.16	3.35131	19536.85
19543.48	3.60467	19546.61	3.60128	19530.33	3.60476	19534.89	3.59752	19534.16	3.60199	19533.46	3.61308	19532.95
19545.3	3.85486	19542.7	3.85384	19532.21	3.85206	19531.44	3.84703	19534.85	3.85469	19535	3.85577	19532.82
19540.8	4.10182	19547.7	4.10219	19530.27	4.10412	19532.05	4.09674	19533.82	4.09985	19534.53	4.10317	19539.29
19542.24	4.35456	19543.58	4.35116	19532.87	4.35188	19529.51	4.34567	19535.36	4.35308	19535.55	4.3506	19536.29
19542.73	4.60413	19543.6	4.60599	19530.39	4.6044	19531.36	4.59776	19535.61	4.60694	19534.4	4.60192	19539.67
19543.06	4.86572	19544.81	4.85612	19529.81	4.85245	19531.67	4.84736	19534.81	4.85816	19534.17	4.85227	19535.25
19544.05	5.10815	19544.7	5.10549	19533.46	5.10642	19534.27	5.10063	19534.77	5.10685	19534.82	5.10232	19537.72
19543.69	5.35499	19544.19	5.35591	19531.02	5.35769	19533.8	5.35111	19533.63	5.35371	19532.57	5.35125	19534.6
19542.86	5.60337	19542.41	5.60152	19529.34	5.60399	19532.62	5.59704	19533.31	5.60018	19532.25	5.60118	19533.39
19543.07	5.85476	19544.61	5.85456	19534.03	5.85182	19533.41	5.8459	19533.72	5.85708	19534.26	5.85207	19535.52
19542.6	6.10314	19542.48	6.10212	19533.63	6.10434	19531	6.09802	19534.06	6.10223	19535.21	6.10164	19537.11
19543.49	6.35454	19542.47	6.35222	19532.38	6.35399	19531.97	6.3479	19531.94	6.35592	19533.29	6.35281	19535.39
19542.1	6.6039	19543.17	6.60324	19531.78	6.6081	19533.1	6.5984	19533.26	6.60094	19531.59	6.60173	19534.35
19540.69	6.85507	19546.71	6.85576	19532.3	6.85349	19532.14	6.84941	19534.69	6.85471	19535.94	6.85232	19537.21
19544.85	7.10394	19542.11	7.10223	19532.89	7.10428	19532.27	7.09789	19536.09	7.10573	19532.33	7.10334	19534.42
19542.97	7.35478	19541.81	7.35115	19530.14	7.35313	19530.94	7.34707	19532.36	7.35687	19535.05	7.3523	19534.84
19542.39	7.60404	19546.25	7.60314	19536.84	7.60894	19534.42	7.60099	19532.02	7.60293	19534.11	7.60212	19537.97
19543.08	7.85532	19541.15	7.85665	19531.47	7.85251	19532.52	7.84805	19532.7	7.85592	19532.81	7.85253	19535.81
19544.07	8.10439	19544.45	8.10314	19528.66	8.10406	19534.35	8.09808	19534.27	8.10186	19531.89	8.10139	19533.82
19544.32	8.35864	19544.63	8.35253	19531.53	8.35327	19534.6	8.34788	19534.74	8.35604	19537.65	8.3525	19535.14
19541.63	8.60481	19545.87	8.60324	19528.65	8.60945	19535.18	8.59844	19533.24	8.6032	19535.09	8.60281	19535.66
19542.07	8.85498	19547.7	8.86061	19529.59	8.85263	19530.35	8.84899	19533.42	8.85751	19529.77	8.85376	19534.91
19543.78	9.10407	19545.5	9.10388	19533.33	9.10424	19532.43	9.09916	19534.35	9.1022	19532.66	9.102	19533.9
19544.02	9.35563	19545.58	9.35191	19531.21	9.35302	19532.16	9.34681	19533.48	9.35521	19534.76	9.35209	19535.09
19542.67	9.60502	19544.96	9.60355	19532.26	9.60649	19534.8	9.59725	19533.03	9.60231	19534.91	9.60222	19534.41
19544.12	9.85524	19545.09	9.85562	19530.58	9.85383	19530.22	9.8479	19532.16	9.85859	19535.1	9.85723	19538.11
19543.49	10.10478	19546.81	10.10407	19531.73	10.10601	19534.55	10.09952	19537	10.10298	19533.51	10.10819	19536.71
19542.14	10.35577	19545.47	10.35388	19530.48	10.35323	19531.24	10.34863	19532.87	10.35677	19533.79	10.35456	19536.35
19543.22	10.60679	19543.7	10.60343	19531.68	10.6086	19533.06	10.59933	19537.39	10.60241	19535.01	10.60388	19537.96
19542.05	10.85697	19545.23	10.85692	19531.14	10.85572	19535.07	10.8486	19533.67	10.85603	19536.52	10.85413	19537.87
19542.89	11.10599	19542.97	11.10338	19531.15	11.10695	19534.71	11.10015	19533.96	11.10393	19532.88	11.10326	19536.75
19542.89	11.35648	19542.67	11.35421	19533.36	11.35395	19532.67	11.34897	19531.55	11.35609	19531.39	11.35476	19536.45
19543.36	11.60673	19542.57	11.60507	19528.68	11.60757	19528.51	11.59884	19534.32	11.6035	19534.22	11.60532	19539.76
19542.75	11.8576	19543.32	11.85726	19534.69	11.85316	19536.04	11.84881	19532.05	11.85844	19535.27	11.85554	19536.71
19542.57	12.10577	19545.28	12.10446	19532.7	12.10742	19534.01	12.09979	19531.8	12.10429	19534.42	12.10385	19533.53
19542.9	12.35633	19544.5	12.35431	19533.6	12.3552	19532.04	12.3494	19535.21	12.35745	19535.11	12.35348	19531.53
19542.44	12.60572	19545.3	12.60555	19531.91	12.60853	19533.17	12.60072	19531.96	12.60436	19534.14	12.60382	19535.16
19542.17	12.85731	19547.51	12.85911	19531.91	12.85344	19533.49	12.84979	19533.36	12.85646	19537.48	12.85502	19534.88
19542.57	13.10595	19547.27	13.104	19531.26	13.10685	19535.6	13.10022	19533.65	13.10246	19536.2	13.10433	19536.98
19539.65	13.35714	19545.4	13.35273	19531.09	13.35423	19534.35	13.34899	19532.9	13.37318	19532.92	13.35327	19534.52
19540.38	13.60581	19542.49	13.60596	19534.81	13.60946	19534.27	13.60223	19534.42	13.60424	19534.59	13.60362	19536.06
19541.63	13.85728	19544.71	13.85898	19531.51	13.85411	19532.09	13.84953	19533.03	13.86012	19532.3	13.85483	19536.1
19541.08	14.10648	19546.02	14.10576	19532.91	14.10589	19529.86	14.09991	19531.75	14.10322	19532.96	14.1046	19536.38
19543.12	14.35755	19546.42	14.35343	19531.76	14.3536	19530.86	14.34922	19533.52	14.35689	19536.02	14.35395	19536.07
19545.59	14.60595	19544.48	14.60481	19530.92	14.6118	19535.81	14.59966	19532.62	14.6045	19530.76	14.60401	19534.84
19542.65	14.85696	19547.29	14.85824	19531.1	14.85589	19531.31	14.85054	19532.92	14.86012	19532.93	14.85516	19536.53
19540.47	15.10607	19545.76	15.10644	19531.84	15.1064	19529.9	15.10183	19533.91	15.10487	19536.46	15.10486	19537.15
19544.37	15.35859	19541.12	15.35513	19530.06	15.35517	19531.11	15.34998	19536.44	15.35874	19533.33	15.3573	19534.25
19542.91	15.6083	19545.53	15.60816	19530.43	15.6086	19533.33	15.60035	19534.34	15.60469	19534.11	15.60999	19531.79
19542.11	15.85944	19544.34	15.85894	19530.99	15.85646	19530.17	15.85078	19531.63	15.85809	19535.29	15.85435	19534.56
19543.78	16.10745	19546.28	16.10624	19528.82	16.10869	19533.3	16.10297	19532.32	16.10521	19532.63	16.10457	19534.45

19542.74	16.35877	19543.11	16.35611	19531.05	16.35598	19532.43	16.35146	19536.17	16.35943	19532.87	16.35623	19533.89
19542.62	16.60789	19547.4	16.60727	19531.4	16.60915	19532.29	16.60069	19532.54	16.60507	19533.24	16.60538	19536.16
19540.51	16.85917	19542.56	16.86211	19532.44	16.85587	19534.13	16.84954	19530.8	16.86079	19529.49	16.85516	19535.63
19539.66	17.10805	19544.61	17.105	19532.28	17.1073	19530.84	17.102	19531.46	17.10473	19533.74	17.10458	19538.91
19542.77	17.35673	19542.47	17.35545	19529.64	17.35533	19533.35	17.35177	19533.58	17.35871	19534.04	17.35519	19535.64
19540.84	17.60691	19545.29	17.60695	19530.89	17.60775	19533.98	17.60268	19534.37	17.60642	19534.91	17.60556	19536.08
19541.95	17.85875	19547.06	17.85946	19530.49	17.85571	19533.48	17.84951	19529.77	17.86067	19533.4	17.85655	19538.01
19543.21	18.10742	19543.3	18.10607	19532.24	18.10727	19533.23	18.10077	19530.27	18.10489	19532.84	18.10532	19533.92
19540.12	18.36291	19542.13	18.35429	19531.61	18.35617	19535.17	18.35127	19534.21	18.35848	19536.36	18.35507	19534.43
19542.18	18.61251	19542.68	18.60521	19530.98	18.61086	19531.39	18.6022	19536.36	18.60697	19533.09	18.60467	19535.13
19545.33	18.85916	19541.98	18.86078	19532.46	18.8564	19532.74	18.8512	19533.38	18.85882	19534.33	18.85615	19535.45
19543.04	19.10818	19546.72	19.10767	19532.19	19.10759	19531.49	19.10266	19531.09	19.10564	19534.25	19.10587	19535.9
19540.49	19.35927	19545.57	19.35504	19531.95	19.35705	19532.88	19.35002	19532.33	19.35736	19534.79	19.35609	19536.11
19544.03	19.60704	19545.62	19.61029	19529.92	19.61304	19532.86	19.60305	19531.29	19.60657	19534.93	19.60531	19534.58
19545.99	19.86129	19543.21	19.86128	19531.55	19.85637	19533.15	19.85275	19532.34	19.86214	19531.65	19.85634	19534.51
19542.64	20.10826	19545.27	20.10822	19532.36	20.10729	19533.33	20.10384	19534.82	20.10673	19533.59	20.10586	19535.02
19544.47	20.36062	19544.92	20.35721	19530.49	20.35665	19530.97	20.35147	19533.56	20.36021	19536.84	20.35717	19534.06
19542.45	20.61142	19545.09	20.607	19532.21	20.61186	19533.12	20.60245	19534.41	20.60656	19533.14	20.60829	19534.75
19543.83	20.86023	19544.88	20.86136	19533.83	20.85801	19534.01	20.85214	19533.9	20.86268	19532.64	20.85718	19533.69
19542.84	21.10878	19543.74	21.10896	19532.84	21.10894	19531.24	21.1035	19531.95	21.10768	19533	21.1069	19534.33
19542.66	21.36254	19542.48	21.35917	19531.72	21.35733	19533.29	21.35326	19532.57	21.36034	19532.45	21.35728	19534.29
19544.7	21.60995	19544.14	21.60693	19531.78	21.61269	19533.93	21.62541	19532.89	21.60648	19530.44	21.60829	19535.58
19541.22	21.86068	19544.32	21.8612	19532.27	21.85855	19532.88	21.85172	19535.67	21.86274	19533.95	21.85846	19533.27
19544.5	22.10876	19545.66	22.10887	19532.12	22.111	19531.72	22.10451	19535.42	22.1084	19532.57	22.10829	19534.46
19541.69	22.36189	19543.86	22.35827	19531.79	22.35773	19533.47	22.35329	19534.13	22.3605	19533.54	22.35649	19538.44
19543.6	22.61116	19545.38	22.60824	19529.1	22.60947	19531.99	22.60655	19534.79	22.60687	19534.1	22.60769	19535.63
19545.65	22.86103	19545.52	22.86229	19531.41	22.85789	19532.44	22.85277	19534.13	22.86265	19536.59	22.85994	19533.07
19541.72	23.10873	19545.57	23.10817	19532.04	23.11022	19534.54	23.1042	19533.33	23.10862	19535.25	23.10849	19534.08
19543.62	23.36123	19546.9	23.35758	19531.42	23.3583	19535.1	23.35375	19533.03	23.36504	19533.8	23.35853	19536.21
19545.56	23.61215	19544.95	23.60823	19531.18	23.61554	19532.76	23.60585	19533.61	23.60829	19535.28	23.60706	19536.15
19542.15	23.86254	19542.89	23.86428	19532.81	23.85805	19535.62	23.85413	19532.24	23.86295	19533.1	23.85807	19537.08
19543.35	24.10879	19543.31	24.10872	19534.26	24.11107	19531.99	24.10429	19532.92	24.10791	19535.7	24.10858	19535.48
19544.14	24.36142	19545.05	24.35709	19531.52	24.3587	19531.61	24.35254	19536.8	24.3623	19533.55	24.35937	19535.93
19542.27	24.6114	19543.71	24.60734	19531.42	24.61063	19530.86	24.60374	19533.71	24.60814	19532.41	24.60729	19532.8
19543.47	24.86349	19544.63	24.86276	19532.49	24.85736	19537.05	24.85589	19533.58	24.86286	19531.45	24.85805	19535.17
19543.18	25.11021	19543.62	25.10935	19532.57	25.10921	19532.63	25.10558	19533.41	25.10645	19534.39	25.10827	19533.89
19541.74	25.36121	19544.42	25.3619	19530.55	25.35922	19529.57	25.35329	19534.59	25.36066	19533.07	25.35909	19536.54
19541.29	25.61036	19546.08	25.60768	19530.72	25.61414	19534.48	25.60559	19530.44	25.60901	19534.33	25.6085	19537.29
19544.97	25.86153	19542.71	25.86202	19531.26	25.8572	19533.68	25.85349	19534.65	25.86339	19533.12	25.85778	19536.18
19544.17	26.11009	19545.01	26.10885	19533.97	26.10953	19530.87	26.1057	19533.98	26.10826	19534.69	26.11002	19542.4
19545.15	26.36196	19546.08	26.35825	19530.45	26.35817	19534.6	26.35361	19533.23	26.36174	19531.62	26.35885	19539.09
19543.37	26.61086	19545.05	26.60951	19534.18	26.61476	19535.74	26.60412	19534.38	26.60735	19534.5	26.60822	19535.01
19544.72	26.861	19545.6	26.86245	19532.04	26.85843	19532.08	26.85376	19533.87	26.86356	19533.17	26.85851	19537.72
19542.95	27.11012	19541.92	27.10935	19530.93	27.10977	19533.39	27.10511	19533.45	27.10997	19532.67	27.11009	19535.74
19544.31	27.36199	19544.95	27.35877	19529.83	27.3641	19530.8	27.35439	19534.13	27.3613	19535.19	27.35901	19537.27
19542.94	27.61185	19544.78	27.6087	19531.93	27.61377	19531.86	27.6034	19534.3	27.60804	19534.75	27.6079	19536.57
19542.11	27.86093	19543.2	27.86354	19531.25	27.85965	19532.93	27.85295	19529.87	27.86169	19532.31	27.86565	19539.2
19543.49	28.11002	19545.66	28.10855	19530.28	28.10954	19532.17	28.10498	19534.04	28.10946	19533.76	28.10891	19533.77
19544.11	28.36193	19544.46	28.35854	19530.94	28.35886	19533.28	28.35431	19534.78	28.36293	19533.64	28.36016	19536.02
19543.88	28.61255	19544.45	28.60967	19531.03	28.61368	19534.4	28.6061	19531.36	28.60909	19533.28	28.60952	19536.46
19543.12	28.8628	19542.15	28.8669	19533.42	28.86007	19534.31	28.85341	19534.35	28.86353	19533.53	28.8591	19537.37
19543.52	29.10979	19545.69	29.10845	19532.43	29.11214	19532.29	29.10511	19531.98	29.10987	19533.52	29.10835	19534.9
19545.01	29.36202	19544.3	29.35834	19531.04	29.35865	19531.72	29.35412	19533.75	29.36422	19533.61	29.35985	19535.11
19543.7	29.6132	19543.97	29.61021	19533.53	29.61177	19533.95	29.60602	19533.09	29.60959	19535.41	29.61057	19533.39
19542.38	29.86397	19546.85	29.86637	19531.73	29.86011	19532.29	29.85352	19533.54	29.86489	19531.46	29.86088	19532.29
19540.79	30.10967	19545.62	30.11067	19533.06	30.11245	19533	30.10597	19531.39	30.10877	19535.91	30.10959	19534.15
19544.75	30.36336	19541.75	30.35875	19530.45	30.36212	19532.18	30.35523	19531.87	30.36412	19535.8	30.35933	19535.58
19542.27	30.6122	19545.75	30.61028	19531.56	30.61591	19534.81	30.60731	19532.81	30.61118	19531.8	30.61113	19531.66
19543.58	30.8639	19546.26	30.8661	19531.2	30.86068	19533.71	30.85604	19535.56	30.86569	19533.03	30.86491	19538.62
19541.1	31.11249	19545.48	31.11237	19534.68	31.11289	19530.42	31.10632	19533.52	31.11023	19533.19	31.11771	19532.94
19541.45	31.3629	19545.31	31.36087	19531.74	31.36268	19534.03	31.35465	19532.19	31.36496	19532.37	31.3636	19535.9
19543.36	31.61394	19545.29	31.61036	19533.94	31.61605	19532	31.60952	19533.18	31.61087	19532.91	31.61055	19532.95
19544.2	31.86514	19543.69	31.86624	19532.42	31.85998	19533.07	31.85657	19531.07	31.86717	19535.91	31.86413	19537.3
19543.17	32.11312	19543.87	32.11233	19533.19	32.11247	19532.21	32.10771	19530.79	32.11276	19532.59	32.11346	19537.85
19541	32.36464	19542.95	32.36154	19533.12	32.36292	19532.77	32.3556	19534.68	32.36711	19531.86	32.36147	19535.11
19543.68	32.61052	19544.72	32.61196	19532.84	32.6137	19533.33	32.60528	19536.78	32.60981	19530.42	32.61053	19536.17
19543.16	32.8641	19543.39	32.86489	19529.52	32.85973	19530.42	32.85598	19530.33	32.86429	19533.73	32.8613	19536.8
19540.55	33.11151	19543.74	33.1115	19532.86	33.11217	19532.85	33.10755	19531.64	33.11182	19533.11	33.11118	19534.4
19546.07	33.36474	19546.32	33.36103	19531.55	33.3627	19532.42	33.35478	19534.65	33.36408	19533.35	33.36125	19538.15
19542.34	33.61399	19546.35	33.61241	19534.78	33.61296	19532.64	33.60659	19530.1	33.6103	19535.36	33.61142	19536.57

19543.76	33.86334	19545.25	33.86538	19530.2	33.86069	19531.84	33.85538	19536.15	33.86447	19533.06	33.86129	19535.97
19542.27	34.11239	19545.17	34.11106	19533.72	34.11161	19534.32	34.10826	19532.37	34.11195	19532.59	34.11018	19533.34
19539.96	34.36353	19543.48	34.36055	19531.27	34.36127	19532.19	34.356	19529.54	34.36378	19531.91	34.36089	19536.28
19545.1	34.61578	19543.85	34.61267	19530.27	34.61204	19531.14	34.60659	19531.64	34.61042	19530.5	34.61162	19536.31
19542.5	34.86571	19546.07	34.86814	19531.75	34.86259	19530.17	34.85645	19535.48	34.86589	19533.13	34.86247	19537.83
19541.26	35.11673	19545.25	35.11068	19530.93	35.11419	19532.39	35.10883	19533.32	35.11154	19531.39	35.11002	19535.15
19541.69	35.36399	19545.04	35.36047	19533.57	35.36138	19531.14	35.35641	19534.41	35.36591	19533.06	35.36058	19534.97
19542.28	35.61512	19543.42	35.61219	19531.2	35.61148	19530.22	35.60806	19532.46	35.6119	19532.37	35.61216	19536.77
19542.12	35.86593	19542.72	35.86821	19531.83	35.86157	19530.94	35.85585	19531.36	35.86555	19535.34	35.86277	19534.93
19542.88	36.11472	19546.91	36.11273	19533.9	36.11454	19531.52	36.10879	19531.54	36.11172	19531.84	36.11192	19535.73
19541.77	36.36348	19541.08	36.35976	19529.27	36.36736	19534.63	36.35761	19533	36.36642	19532.14	36.36076	19536.81
19541.35	36.6168	19545.01	36.61067	19531.48	36.61217	19529.45	36.60899	19533.63	36.61207	19534.35	36.61155	19535.13
19543.43	36.86605	19545.29	36.86755	19532.92	36.86116	19531.21	36.85655	19532.9	36.86631	19534.26	36.86343	19536.79
19542.76	37.11445	19544.05	37.11225	19533.57	37.11338	19529.67	37.10743	19534.15	37.11208	19532.09	37.11402	19535.04
19543.67	37.36689	19545.89	37.36171	19530.95	37.36231	19532.28	37.35754	19532.76	37.36702	19533.48	37.36186	19538.15
19544.26	37.61575	19544.17	37.61343	19529.76	37.61367	19533.72	37.60927	19533.59	37.61326	19533.49	37.61132	19538.33
19545.49	37.86532	19545.03	37.86861	19532.06	37.86083	19531.37	37.85802	19531.42	37.8662	19534	37.86255	19532.03
19539.58	38.1143	19544.36	38.11251	19533	38.11434	19531.32	38.10904	19533.39	38.11139	19536.34	38.11297	19536.41
19543.33	38.36629	19545.25	38.36232	19532.82	38.36357	19533.23	38.35701	19531.65	38.3653	19532.97	38.36313	19537.71
19545.95	38.61557	19544.12	38.61195	19530.55	38.6139	19531.66	38.60832	19532.67	38.61202	19535.59	38.61282	19537.86
19542.34	38.86602	19548.3	38.86714	19529.82	38.86162	19531.13	38.85874	19535.2	38.86738	19533.71	38.8633	19536.4
19542.09	39.11356	19547.05	39.11357	19531.46	39.11314	19531.19	39.11098	19532.39	39.11334	19532.34	39.11304	19534.33
19542.49	39.36765	19546.39	39.36256	19529.76	39.36316	19531.95	39.3573	19531.47	39.36562	19532.49	39.36317	19536.98
19541.09	39.61839	19543.84	39.61415	19533.03	39.61508	19532.44	39.60872	19533.55	39.61138	19535.64	39.61318	19534.34
19543.3	39.86577	19542.32	39.86881	19531.23	39.86295	19530.75	39.85955	19534.64	39.8656	19533.59	39.86471	19536.06
19544.17	40.11462	19544.36	40.11242	19531.11	40.11371	19533.21	40.1098	19531.17	40.1137	19534.32	40.11234	19532.87
19541.65	40.37223	19545.37	40.36371	19533.3	40.36389	19530.32	40.35885	19532.34	40.36867	19534.33	40.36492	19534.86
19543.09	40.61845	19545.16	40.61495	19531.82	40.61563	19532.92	40.60961	19534.61	40.61565	19532.54	40.6153	19535.98
19540.75	40.86905	19544.53	40.8703	19529.76	40.86529	19531.09	40.86051	19532.51	40.86819	19534.61	40.86706	19536.64
19543.73	41.11575	19544.37	41.11614	19533.98	41.11736	19531.36	41.11117	19535.01	41.11427	19534.01	41.11379	19536.01
19542.54	41.36651	19546.4	41.36396	19528.99	41.36376	19531.82	41.35844	19535.84	41.36969	19535.71	41.36447	19536.83
19542.04	41.6204	19546.66	41.61554	19532.98	41.61584	19533.32	41.61151	19533.52	41.61975	19533.45	41.61576	19537.59
19541.83	41.86787	19543.98	41.8692	19529.47	41.86491	19533.22	41.86044	19531.81	41.86761	19531.87	41.86812	19533.94
19544.38	42.11731	19544.82	42.11605	19532.51	42.11709	19533.67	42.11194	19532.93	42.11405	19533.45	42.11588	19535.02
19542.1	42.36752	19544.22	42.36516	19534.78	42.36681	19531.81	42.35999	19531.18	42.36805	19537.92	42.36484	19536.95
19540.93	42.61758	19543.23	42.61474	19530.35	42.61977	19531.72	42.60991	19533.54	42.61638	19533.78	42.61478	19536.62
19542.16	42.86737	19547.08	42.86978	19531.58	42.86509	19534.44	42.85968	19531.65	42.8704	19532.73	42.86732	19533.61
19543.38	43.11688	19546.61	43.1151	19532.4	43.11693	19534.99	43.11099	19533.57	43.11429	19538.16	43.11594	19533.49
19544.83	43.37079	19545.35	43.36457	19531.95	43.36607	19532.66	43.36116	19532.13	43.36872	19534.94	43.37065	19538.69
19544.17	43.6191	19546.3	43.61534	19532.7	43.61761	19531.02	43.6117	19532.75	43.61547	19533.29	43.61373	19536.52
19542.67	43.86702	19546.9	43.87112	19530.13	43.86481	19533.53	43.86011	19533.57	43.87061	19531.75	43.86792	19534.91
19540.86	44.11713	19544.12	44.11552	19531.86	44.11683	19534.86	44.10989	19534.38	44.11565	19535.14	44.11586	19537.33
19542.83	44.37171	19546.4	44.3652	19530.86	44.36563	19531.98	44.36029	19531.96	44.368	19533.98	44.36649	19535.7
19541.07	44.62087	19541.97	44.61628	19533.25	44.6193	19532.18	44.61277	19531.32	44.61522	19534.17	44.61603	19531.78
19541.63	44.86879	19545.29	44.872	19531.56	44.86659	19533.52	44.86178	19536.11	44.87007	19530.32	44.86703	19536.3
19543.54	45.11671	19545.69	45.11534	19530.33	45.11674	19532.26	45.11149	19530.68	45.11867	19532.82	45.11426	19534.3
19546.2	45.36874	19541.68	45.36643	19531.58	45.36508	19526.9	45.36047	19531.34	45.37005	19534.27	45.36497	19538.92
19541.48	45.62088	19544.74	45.61708	19530.07	45.61669	19531.73	45.61315	19533.49	45.61564	19532.42	45.61647	19533.53
19543.9	45.86815	19542.92	45.8698	19531.43	45.86655	19533.56	45.86218	19531.8	45.87065	19536.55	45.87109	19534.02
19542.81	46.1172	19546.88	46.11665	19529.83	46.11805	19534.31	46.11111	19534.37	46.11618	19533.18	46.11452	19534.06
19545.06	46.37016	19544.62	46.36654	19533.32	46.36457	19534.54	46.36054	19532.7	46.3709	19533.55	46.36505	19534.95
19540.89	46.62008	19545.44	46.61914	19528.77	46.61651	19532.81	46.61319	19534.71	46.61715	19534.72	46.61671	19535.9
19543.19	46.87094	19544.4	46.87199	19532.24	46.86751	19529.15	46.86231	19532.41	46.87014	19534.8	46.87082	19535.1
19542.51	47.11899	19545.77	47.11635	19529.09	47.11891	19534.11	47.11213	19536.75	47.11623	19533.85	47.11846	19536.57
19542.59	47.36821	19545.03	47.36522	19529.97	47.36952	19531.14	47.36061	19532.64	47.37024	19534.54	47.36517	19538.1
19544.07	47.62294	19545.69	47.61676	19528.98	47.61832	19532.72	47.61333	19533.82	47.61743	19533.04	47.61567	19533.04
19544.29	47.87061	19542.77	47.87165	19536.18	47.868	19532.81	47.86267	19533.56	47.87028	19532.6	47.86726	19537.36
19541.36	48.12078	19544.26	48.11866	19532.11	48.11896	19533.04	48.11374	19535.42	48.11548	19532.79	48.1169	19534.69
19544.15	48.36904	19543.46	48.36633	19528.09	48.3674	19536.61	48.36122	19530.8	48.37093	19532.31	48.36733	19532.11
19542.94	48.62049	19545.83	48.62534	19530.46	48.61612	19532.62	48.61734	19534.89	48.61643	19534.5	48.61467	19535.34
19544.84	48.87119	19543.76	48.87169	19529.18	48.86561	19533.92	48.87162	19532.15	48.87066	19534.87	48.86817	19533.33
19541.17	49.11843	19543.26	49.11779	19531.97	49.11768	19532.71	49.11393	19534.21	49.11544	19534.14	49.11523	19534.76
19542.27	49.37068	19546.12	49.36509	19533.15	49.36633	19532.08	49.36133	19533.93	49.36934	19533.58	49.36765	19532.9
19543.95	49.62199	19547.71	49.61789	19530.38	49.62002	19533.14	49.61376	19534.53	49.61828	19533.43	49.61798	19536.38
19543.32	49.87043	19543.5	49.87211	19526.53	49.86473	19536.34	49.86207	19532.64	49.87094	19530.46	49.86653	19535.03
19540.07	50.11789	19545.47	50.11859	19532.96	50.11797	19530.04	50.12981	19530.81	50.11747	19534.06	50.11643	19534.48
19542.24	50.37287	19543.79	50.36761	19532.1	50.36663	19534.6	50.36233	19534.65	50.3706	19533.78	50.36749	19535.39
19542.3	50.62094	19544.87	50.61763	19532.7	50.61846	19533.24	50.61459	19534.36	50.61557	19533.17	50.61763	19535.91
19542.07	50.87039	19543.7	50.87154	19531.7	50.86835	19531.4	50.85993	19533.04	50.87011	19533.5	50.86876	19536.32
19544.47	51.11985	19542.87	51.11769	19528.9	51.11773	19531.84	51.11238	19530.33	51.11747	19534.95	51.11532	19534.81

19541.8	51.37117	19544.63	51.3668	19533.55	51.36585	19532.68	51.36217	19532.99	51.37156	19533.51	51.36717	19534.79
19545.14	51.62539	19541.48	51.62214	19531.09	51.62051	19534.58	51.61477	19531.98	51.61714	19532.46	51.61765	19535.95
19540.81	51.8709	19543.97	51.87349	19532.98	51.86758	19533.27	51.86133	19531.78	51.87026	19532.63	51.86853	19532.62
19539.72	52.11832	19546.98	52.11746	19530.75	52.11912	19531.94	52.11205	19534.05	52.1173	19533.18	52.11747	19536.87
19543.64	52.36852	19544.55	52.36555	19531.37	52.36623	19534.95	52.3616	19532.96	52.37207	19534.85	52.36669	19534.14
19542.88	52.62283	19545.74	52.61778	19529.5	52.62108	19532.38	52.61944	19534.67	52.61851	19531.67	52.61683	19534.03
19543.67	52.87164	19542.97	52.87342	19535.14	52.86727	19531.71	52.86237	19532.34	52.87142	19534.18	52.87011	19535.91
19540.6	53.1204	19544.91	53.11819	19533.05	53.1205	19531.72	53.11277	19534.23	53.11532	19534.56	53.11827	19535.93
19542.16	53.36943	19543.27	53.36556	19529.54	53.36807	19532.05	53.36132	19534.95	53.37248	19532.34	53.36857	19536.39
19546.64	53.62053	19544.85	53.61788	19530.3	53.62113	19531.92	53.61287	19533.89	53.61789	19533.07	53.61729	19535.81
19542.19	53.87034	19543.88	53.87372	19529.13	53.86841	19535.42	53.86323	19532.1	53.87335	19534	53.87149	19536.91
19543.16	54.12127	19546.49	54.1188	19529.31	54.12339	19533.52	54.11425	19533.78	54.11737	19534.29	54.11824	19536.12
19543.44	54.3712	19542.46	54.36608	19531.88	54.36855	19530.57	54.36086	19530.68	54.37237	19531.09	54.3697	19536.26
19543.25	54.62403	19543.75	54.61768	19533.16	54.62161	19535.04	54.61241	19536.18	54.6179	19535.7	54.61835	19535.37
19540.41	54.87111	19548.5	54.87385	19533.97	54.86739	19534.15	54.86291	19532.84	54.87284	19533.36	54.86962	19534.63
19542.09	55.11912	19544.16	55.12057	19530.02	55.11866	19533.58	55.11537	19534.54	55.11872	19531.32	55.11764	19538
19545.22	55.37107	19543.58	55.3668	19530.86	55.36815	19533.27	55.36303	19533.63	55.37368	19532.37	55.36964	19532.87
19546.49	55.62292	19545.15	55.61852	19532.55	55.62133	19534.15	55.6129	19533.62	55.6175	19533.81	55.61872	19536.22
19546.09	55.87736	19544.5	55.87307	19533.8	55.86874	19535.26	55.86295	19531.84	55.87213	19532.37	55.87258	19534.98
19544.21	56.11905	19545.77	56.12046	19527.89	56.11957	19532.06	56.11498	19531.14	56.11772	19531.86	56.11742	19535.71
19540.61	56.37089	19541.37	56.36786	19529.6	56.36829	19530.49	56.36364	19532.17	56.82208	19531.92	56.36881	19533.11
19547.45	56.62487	19543.28	56.6177	19530.14	56.62351	19534.07	56.61544	19533.97	57.06205	19536.9	56.61894	19537.77
19543.09	56.87276	19541.45	56.8729	19532.15	56.87024	19533.14	56.86172	19531.48	57.31337	19532.34	56.87213	19536.02
19542.68	57.11995	19544.14	57.12001	19529.35	57.12046	19533.35	57.1137	19530.46	57.56493	19533.07	57.1194	19535.1
19543.61	57.37051	19544.27	57.36876	19535.42	57.36798	19533.55	57.36504	19533.35	57.81641	19534.11	57.36886	19533.65
19544.8	57.62543	19543.82	57.62007	19529.92	57.62062	19530.32	57.61638	19531.08	58.06351	19533.84	57.61868	19534.21
19545.6	57.87364	19545.99	57.87386	19529.26	57.86932	19535.37	57.86422	19532.51	58.31432	19533.95	57.87304	19536.96
19544.34	58.1211	19545.99	58.12009	19529.64	58.12197	19531.62	58.11407	19536.59	58.56798	19533.43	58.11938	19535.35
19545.04	58.37159	19545.2	58.36824	19529.31	58.368	19530.82	58.36344	19534.15	58.81674	19535.73	58.36791	19535.89
19541.16	58.62566	19546	58.62125	19531.04	58.62034	19532.48	58.61581	19533.05	59.06221	19533.42	58.61881	19536.28
19543.59	58.87341	19542.42	58.87696	19530.61	58.86885	19531.5	58.86482	19533.83	59.31558	19533.88	58.87239	19535.16
19541.8	59.1219	19541.14	59.12769	19530.81	59.12173	19530.91	59.11667	19534.33	59.56978	19534	59.12738	19534.73
19543.92	59.37131	19544.35	59.37237	19532.4	59.37059	19534.49	59.36275	19535.39	59.81707	19533.49	59.36974	19534.52
19541.24	59.62293	19546.24	59.61947	19529.53	59.62063	19531.92	59.61523	19531.56	60.0622	19537.21	59.6184	19535.59
19544.95	59.8727	19542.48	59.88091	19531.13	59.87956	19533.55	59.86532	19536.14	60.31605	19532.52	59.87427	19535.47
19541.67	60.12197	19545.21	60.12289	19531.04	60.121	19532.62	60.11572	19534.19	60.57228	19533.06	60.12082	19535.15
19545.5	60.37481	19544.74	60.37112	19530.3	60.37083	19535.33	60.36665	19532.62	60.82977	19532.62	60.37188	19533.42
19542.16	60.62842	19544.42	60.62087	19530.24	60.62544	19534.91	60.61789	19532.27	61.06376	19532.66	60.62122	19536.05
19544.19	60.87394	19543.28	60.87612	19533.68	60.88172	19533.16	60.8651	19534.1	61.31582	19531.09	60.87536	19534.65
19543.81	61.12323	19543.28	61.12239	19531.99	61.1245	19533.6	61.11796	19533.27	61.5726	19533.23	61.12028	19538.64
19544.51	61.37771	19544.15	61.3717	19532.6	61.37035	19530.02	61.3671	19534.65	61.81846	19534.55	61.37137	19538.02
19543.94	61.62676	19544.12	61.62338	19530.04	61.6218	19532.12	61.61855	19532.59	62.06526	19534.49	61.62257	19534.44
19543.14	61.87448	19546.98	61.87501	19533.92	61.87277	19532.02	61.86655	19534.76	62.31532	19535.58	61.87693	19538.4
19541.92	62.1221	19541.3	62.12192	19529.97	62.12454	19529.97	62.12011	19537.03	62.5689	19533.24	62.12306	19534.68
19544.74	62.37504	19543.36	62.37111	19530.34	62.37173	19534.74	62.3673	19533.76	62.81905	19534.71	62.37092	19535.12
19545.45	62.63084	19545.63	62.62391	19528.54	62.62231	19533.16	62.61867	19531.3	63.0659	19532.93	62.62505	19534.8
19543.82	62.87648	19544.9	62.87949	19529.51	62.87309	19536.02	62.86622	19533.61	63.3173	19535.1	62.87674	19533.18
19543.42	63.12367	19540.75	63.12306	19529.4	63.12463	19531.89	63.11908	19533.72	63.57024	19533.84	63.12237	19533.25
19543.76	63.3758	19543.7	63.37477	19532.78	63.37441	19534.67	63.3689	19531.95	63.82032	19533.29	63.37118	19535.2
19543.23	63.62951	19543.55	63.62239	19534.24	63.62329	19531.61	63.62032	19533.96	64.06604	19534.02	63.62117	19535.7
19543.9	63.87619	19543.81	63.87795	19532.65	63.87263	19534.3	63.86886	19533.32	64.31678	19535.02	63.87654	19531.54
19542.5	64.12369	19545.05	64.12453	19534.76	64.12618	19534.47	64.11902	19532.88	64.57	19535.88	64.12252	19535.47
19542.74	64.37787	19542.23	64.37124	19530.57	64.37366	19528.46	64.36594	19531.23	64.8177	19534.35	64.3726	19540.33
19541.97	64.62538	19543.51	64.62198	19532.43	64.62385	19531.06	64.61895	19533.47	65.06524	19532.66	64.61998	19535.31
19541.23	64.87442	19545.72	64.87657	19530.19	64.86952	19532.58	64.86688	19535.87	65.31643	19534.32	64.87641	19535.38
19541.56	65.13142	19544.23	65.12404	19531.59	65.12211	19533.09	65.11991	19530.39	65.57121	19530.42	65.12165	19533.68
19543.11	65.37518	19544.7	65.37186	19531.24	65.37326	19532.68	65.36776	19531.55	65.8178	19534.38	65.37181	19534.35
19542.55	65.62792	19542.87	65.62273	19529.54	65.62638	19534.4	65.61755	19535.7	66.0648	19535.32	65.62056	19533.37
19545.34	65.8742	19543.27	65.87692	19532.21	65.87057	19531.01	65.86621	19532.96	66.31771	19534.05	65.87362	19534.19
19543.01	66.12372	19544.51	66.12321	19531.55	66.1236	19533.6	66.11894	19533.43	66.57169	19534.85	66.12229	19534.61
19540.28	66.37685	19544.44	66.37159	19534.52	66.37178	19531.74	66.36745	19532.04	66.81821	19532.09	66.37282	19536.6
19543.98	66.62936	19545.31	66.6222	19532.98	66.62633	19534.26	66.61836	19533.58	67.06379	19531.12	66.62263	19537.5
19542.6	66.8747	19544.43	66.87601	19533.24	66.87291	19529.19	66.86535	19533.62	67.31706	19535.42	66.87747	19537.81
19541.38	67.12292	19545.31	67.12326	19532.05	67.12288	19533.45	67.11894	19534.78	67.57202	19535.46	67.12396	19536.07
19543.84	67.37892	19542.71	67.37187	19531.13	67.37083	19531.95	67.36786	19536.33	67.81948	19534.04	67.37253	19534.14
19545.1	67.62909	19544.58	67.62365	19530.69	67.62319	19533.18	67.61964	19534.72	68.06433	19535.63	67.62424	19535.78
19543.44	67.87507	19543.41	67.87563	19532.49	67.87266	19530.78	67.86679	19532.66	68.31777	19536.56	67.87712	19533.07
19542.42	68.12277	19543.23	68.1228	19530.75	68.12564	19534	68.11774	19535.91	68.5714	19532.69	68.12062	19534.18
19545.7	68.37796	19545.57	68.37149	19535.6	68.37167	19534.41	68.3667	19529.99	68.82122	19534.06	68.37236	19537.35
19543.76	68.62875	19544.1	68.62171	19533.96	68.62303	19533.91	68.61856	19533.21	69.06727	19531.68	68.6239	19535.28

19544.4	68.87789	19543.77	68.8776	19530.79	68.87303	19532.49	68.86825	19533.53	69.31749	19532.58	68.87714	19536.81
19543.55	69.12394	19544.39	69.12471	19532.75	69.12537	19532.57	69.11836	19533.05	69.57126	19533.96	69.12353	19533.56
19542.34	69.37559	19545.12	69.37061	19532.65	69.37289	19529.73	69.36696	19532.62	69.82114	19535.35	69.37176	19534.69
19544.43	69.6295	19545.83	69.62416	19532.14	69.62538	19530.7	69.61921	19535	70.06773	19534.45	69.62246	19537.11
19544.15	69.87848	19542.67	69.87956	19531.17	69.87159	19534.06	69.86959	19533.39	70.31793	19536.33	69.87721	19533.82
19542.44	70.12536	19546.88	70.12572	19532.76	70.12613	19533.19	70.12036	19534.74	70.57153	19533.33	70.12383	19535.55
19544.05	70.37733	19545.18	70.37251	19532.92	70.37309	19532.22	70.36631	19532.38	70.82064	19535.73	70.3735	19533.02
19542.98	70.63026	19545.9	70.62313	19532.28	70.62519	19530.67	70.61856	19532.26	71.06737	19534.15	70.62296	19537.47
19543.42	70.87703	19546.66	70.87806	19529.62	70.87302	19531.11	70.86831	19535.28	71.32035	19534.83	70.87766	19534.28
19543.67	71.12598	19544.13	71.12695	19527.63	71.1241	19533.69	71.12044	19536.06	71.57627	19534.38	71.12418	19535.65
19542.26	71.37917	19545.6	71.37458	19531.66	71.37401	19531.05	71.36957	19532.47	71.82084	19532.54	71.37503	19534.94
19545.68	71.63133	19541.53	71.62398	19530.05	71.6272	19535.1	71.62008	19533.72	72.06679	19534.44	71.62375	19534.12
19542.12	71.87973	19546.65	71.87967	19527.67	71.87247	19534.64	71.86799	19532.82	72.3213	19533.55	71.87801	19534.8
19544.51	72.1271	19543.19	72.12593	19532.81	72.12362	19534.12	72.11949	19535.85	72.57436	19531.97	72.12585	19534.82
19543.71	72.37969	19539.93	72.37456	19530.83	72.37354	19532.07	72.36938	19535.82	72.82142	19532.23	72.37489	19536.01
19543.01	72.63047	19545.55	72.62403	19528.93	72.62879	19535.19	72.6205	19532.32	73.06622	19533.43	72.62622	19533.81
19540.83	72.87781	19544.37	72.87892	19534.66	72.87523	19531.56	72.86917	19535.87	73.31961	19534.61	72.87903	19536.43
19542.75	73.12594	19544.32	73.1258	19529.95	73.12963	19530.72	73.1198	19533.6	73.60092	19535.92	73.12358	19533.04
19541.56	73.37937	19545.8	73.37511	19533.65	73.37262	19530.78	73.36897	19534.8	73.83173	19531.65	73.37342	19535.21
19541.76	73.63207	19543.55	73.62672	19532.36	73.62462	19532.9	73.61976	19533.41	74.06972	19538.22	73.62575	19538.62
19542.32	73.87902	19546.22	73.87868	19533.51	73.87581	19532.13	73.86997	19533.7	74.31803	19534.08	73.87973	19536.84
19541.32	74.12984	19543.86	74.12547	19531.12	74.1282	19531.39	74.11974	19534.51	74.57378	19533.33	74.1231	19535.96
19539.73	74.38437	19542.43	74.37621	19529.4	74.37422	19532.84	74.36951	19535.83	74.82242	19532.68	74.37419	19535.6
19541.84	74.6331	19544.33	74.62591	19532.83	74.62435	19531.93	74.62002	19531.12	75.06921	19532.41	74.62569	19537.34
19541.98	74.87764	19547.44	74.87927	19531.5	74.87529	19531.38	74.87084	19534.04	75.32053	19533.81	74.87942	19536.79
19540.28	75.12518	19544.56	75.12502	19531.26	75.12757	19531.31	75.12134	19532.78	75.57484	19533.58	75.12585	19534.97
19545.29	75.38031	19545.44	75.37554	19529.9	75.3757	19533.81	75.3677	19534.15	75.82153	19532.13	75.37561	19536.5
19543.88	75.63398	19546.14	75.62799	19532.24	75.62722	19532.49	75.61968	19533.08	76.06979	19534.22	75.63426	19532.61
19543.94	75.8786	19544.36	75.88107	19527.48	75.87587	19529.35	75.87253	19533.61	76.32243	19535.95	75.89384	19537.94
19543.06	76.12481	19544.75	76.12577	19533.58	76.12617	19534.44	76.12293	19530.53	76.58001	19534.06	76.12586	19530.05
19545.33	76.37809	19543.44	76.37654	19534.44	76.37577	19531.46	76.36883	19532.24	76.82183	19535.03	76.37536	19537.17
19542.18	76.63364	19546.33	76.62536	19532.55	76.62848	19536.7	76.62883	19532.26	77.06839	19537.78	76.62597	19535.76
19541.21	76.88152	19544.22	76.88272	19531.47	76.87806	19531.31	76.86974	19531.58	77.32036	19530.12	76.88106	19536.76
19542.32	77.12817	19546.42	77.12598	19531.58	77.12605	19536.03	77.12295	19532.26	77.57652	19534.79	77.12881	19534.1
19544.9	77.37918	19545.71	77.37585	19530.61	77.37551	19532.15	77.37249	19534.84	77.82139	19534.61	77.37655	19535.1
19540.43	77.63232	19546.42	77.62557	19531.52	77.62783	19532.34	77.62145	19532.16	78.06829	19534.24	77.62469	19534.5
19542.31	77.8802	19544.84	77.88053	19531.22	77.87718	19530.13	77.86983	19531.46	78.32031	19532.6	77.88011	19535.49
19545.5	78.12861	19544.56	78.12763	19529.93	78.12829	19531.52	78.12152	19532.61	78.57643	19530.96	78.12583	19534.82
19544.16	78.3811	19546.45	78.37651	19532.64	78.37467	19533.51	78.37054	19533.34	78.82616	19535.51	78.37652	19531.49
19542.62	78.63438	19542.47	78.62662	19531.71	78.62634	19528.91	78.62199	19530.26	79.07077	19531.46	78.62714	19533.48
19544.15	78.87932	19546.03	78.87917	19532.17	78.88702	19532.61	78.87054	19533.46	79.32019	19534.78	78.88263	19536.29
19540.45	79.12859	19544.69	79.12902	19530.75	79.13011	19532.06	79.12191	19531.78	79.57574	19535.61	79.12617	19533.32
19541.47	79.38072	19544.97	79.37771	19532.13	79.37676	19530.67	79.37097	19533.63	79.82338	19532.66	79.37574	19534.28
19541.04	79.63501	19544.78	79.62575	19533.18	79.62611	19533.83	79.62374	19532.11	80.07005	19533.18	79.62612	19535.9
19542.05	79.88082	19543.45	79.88777	19535.16	79.87615	19534.82	79.87145	19534.62	80.32256	19533.52	79.88322	19536.68
19540.18	80.12809	19544.58	80.12851	19532.59	80.12744	19530.31	80.12253	19531.16	80.5772	19531.83	80.12642	19534.31
19545.11	80.38308	19545.98	80.37964	19532.3	80.37859	19533.78	80.37277	19532.07	80.82346	19532.36	80.37766	19533.51
19543.1	80.63495	19542.94	80.62855	19531.81	80.62867	19531.85	80.62137	19532.82	81.07176	19530.84	80.62827	19534.88
19541.09	80.88204	19544.62	80.87942	19529.76	80.88176	19530.1	80.87223	19532.25	81.32379	19532.46	80.89345	19530.95
19541	81.12811	19544.41	81.12619	19531.66	81.12772	19533.96	81.12445	19533.31	81.57698	19533.1	81.13505	19534.97
19543	81.38201	19542.51	81.37844	19533.79	81.37651	19530.32	81.3733	19531.06	81.82417	19532.26	81.37754	19534.79
19540.72	81.6352	19542.92	81.62669	19530.54	81.62972	19532.04	81.6221	19536.79	82.06839	19535.32	81.62529	19536.57
19542.02	81.88357	19545.18	81.8799	19531.03	81.8773	19534.45	81.87133	19532.76	82.32352	19531.59	81.88335	19535.39
19542.23	82.12825	19542.61	82.12564	19530.24	82.12742	19531.94	82.1233	19531.97	82.57716	19535.21	82.13183	19537.66
19542.57	82.37966	19543.28	82.37633	19527.92	82.37702	19532.11	82.37416	19533.99	82.8232	19532.12	82.37794	19536.6
19541.79	82.63552	19546.07	82.62914	19531.59	82.62962	19532.15	82.62401	19531.28	83.0695	19534.23	82.62675	19534.22
19540.26	82.8822	19544.17	82.88401	19531.34	82.87758	19534.39	82.87123	19532.16	83.32362	19531.86	82.8821	19540.31
19541.6	83.12963	19541.65	83.12867	19528.99	83.1297	19532.99	83.12364	19532.35	83.57716	19532.57	83.1278	19535.2
19545.31	83.37803	19545.82	83.37671	19530.63	83.3758	19531.62	83.37435	19534.22	83.82688	19533.1	83.37887	19534.85
19539.67	83.63556	19544.21	83.62898	19532.34	83.62828	19533.61	83.62456	19535.59	84.07019	19534.55	83.62824	19532.99
19537.55	83.88227	19546.08	83.88309	19530.35	83.87768	19531.77	83.87159	19532.63	84.32279	19533.83	83.88385	19534.58
19542.82	84.13046	19544.87	84.13008	19531.41	84.13176	19532.3	84.12339	19530.23	84.57804	19533.51	84.12662	19536
19539.88	84.37999	19543.87	84.37752	19530.28	84.37901	19530.48	84.37399	19533.03	84.82642	19534.66	84.37704	19532.98
19540.1	84.63733	19545.06	84.62784	19529.26	84.63403	19535.29	84.62525	19530.47	85.0718	19530.53	84.62884	19537.58
19540.59	84.88259	19546.02	84.88178	19533.28	84.87626	19530.68	84.87502	19532.26	85.32437	19528.95	84.88497	19532.69
19541.33	85.13033	19541.58	85.13014	19532.69	85.13136	19531.88	85.12396	19533.15	85.57848	19531.5	85.12765	19535.98
19543.07	85.3809	19545.02	85.37878	19530.35	85.37866	19533.32	85.37437	19531.05	85.826	19531.05	85.37886	19532.76
19540.56	85.63579	19544.74	85.62836	19531.17	85.6297	19530.81	85.62471	19533	86.073	19533.96	85.62923	19534.81
19541.98	85.88219	19542.41	85.8846	19534.73	85.87961	19531.69	85.87554	19532.78	86.32501	19532.9	85.88663	19535.86
19540.68	86.13104	19545.08	86.13034	19529.8	86.12863	19532.64	86.12349	19531.86	86.57775	19531.62	86.12996	19533.95

19540.03	86.38334	19543.53	86.37883	19533.85	86.37766	19530.49	86.37474	19533.32	86.8256	19530.67	86.37982	19534.76
19542.46	86.63746	19541.52	86.63044	19530.13	86.62993	19532.51	86.62597	19530.96	87.07086	19532.17	86.62904	19533.93
19539.19	86.88175	19542.94	86.88247	19532.83	86.88056	19535.06	86.87539	19534.34	87.32519	19531.79	86.88633	19535.66
19539.23	87.13028	19545.8	87.13015	19532.48	87.13314	19530.14	87.12431	19534.27	87.57976	19533.52	87.13111	19533.28
19542.66	87.38491	19543.66	87.37762	19527.44	87.3785	19531.3	87.37757	19530.79	87.82635	19531.69	87.38001	19534.89
19539.97	87.63823	19540.69	87.63378	19530.33	87.62988	19530.56	87.625	19530.75	88.07227	19529.9	87.63081	19537.62
19539.77	87.8822	19541.79	87.88158	19530.72	87.88006	19531.88	87.87517	19534.48	88.32618	19528.76	87.88617	19533.05
19541.05	88.13076	19542.93	88.12936	19529.4	88.13354	19532.16	88.12658	19532.57	88.57994	19530.84	88.12894	19535.27
19542.54	88.38244	19542.64	88.37861	19530.23	88.38093	19530.78	88.37312	19532.14	88.8281	19533.97	88.37984	19532.26
19540.27	88.6398	19543.9	88.63256	19530.27	88.63029	19532.36	88.62634	19532.85	89.07211	19531.64	88.63027	19534.57
19541.95	88.88423	19540.76	88.88556	19528.15	88.87935	19530.45	88.87643	19532.23	89.32655	19531.88	88.88691	19532.9
19539.05	89.12989	19543.11	89.13082	19531.45	89.13215	19531.17	89.12829	19530.83	89.57998	19532.86	89.12886	19533.23
19541.45	89.3819	19541.64	89.37754	19531.17	89.38055	19531.04	89.37482	19531.69	89.82782	19532.47	89.37928	19532.82
19538.79	89.6387	19541.72	89.63171	19530.93	89.6309	19530.75	89.6239	19531.99	90.07202	19535.5	89.62983	19534.21
19539.79	89.8853	19541.47	89.88079	19529.3	89.8786	19533.59	89.87403	19531.4	90.33209	19531.01	89.88712	19534.6
19538.75	90.13244	19542.21	90.13389	19530.26	90.13114	19528.89	90.12732	19531.76	90.58916	19533.93	90.13172	19536.35
19537.24	90.38211	19542.25	90.379	19527.95	90.38015	19531.13	90.37542	19533.06	90.83147	19531.78	90.38073	19533.4
19541.27	90.63939	19544.63	90.62999	19532.06	90.63312	19528.81	90.62702	19530.07	91.08222	19534.24	90.63024	19531.72
19540.22	90.88513	19543.04	90.88423	19531.01	90.8784	19533.57	90.87363	19529.46	91.32986	19533.31	90.8866	19532.26
19541.76	91.1332	19542.27	91.13395	19530.06	91.1304	19530.94	91.12717	19532.45	91.58077	19532.43	91.1315	19533.66
19541.25	91.38473	19546.26	91.38036	19528.92	91.38006	19531.18	91.3761	19530.45	91.83243	19534.51	91.38089	19530.9
19539.27	91.63932	19542.47	91.63189	19526.86	91.63247	19530.16	91.62822	19531.95	92.07454	19532.55	91.62951	19535.44
19539	91.88377	19541.74	91.88317	19531.38	91.88102	19529.4	91.87552	19531.53	92.3274	19533.16	91.88729	19532.91
19538.77	92.13379	19541.72	92.13388	19528.4	92.14197	19528.86	92.12615	19531.08	92.58114	19529.92	92.13552	19532.64
19541.6	92.38712	19543.35	92.38044	19529.44	92.37932	19528.69	92.37507	19531.24	92.82903	19533.72	92.38211	19535.52
19538.31	92.64368	19543.94	92.62991	19527.17	92.63147	19535.59	92.62918	19530.71	93.07322	19532.43	92.63057	19535.37
19539.06	92.88356	19544.55	92.88255	19529.49	92.883	19529.1	92.87672	19528.82	93.32858	19531.69	92.8873	19531.26
19538.88	93.13303	19543.4	93.13346	19529.94	93.13404	19533.11	93.12878	19531.86	93.58325	19530.79	93.13134	19534.07
19538.47	93.38524	19545.6	93.3812	19528.27	93.37986	19532.38	93.37528	19531.26	93.83171	19532.98	93.38147	19534.47
19539.43	93.65986	19541.76	93.63365	19530.28	93.63269	19527.33	93.62686	19529.5	94.07317	19529.36	93.63241	19533.9
19541.17	93.88657	19543.59	93.88204	19529.43	93.88209	19529.27	93.8772	19532.5	94.3264	19530.87	93.89018	19534.71
19538.83	94.13219	19539.46	94.13246	19529.15	94.13542	19528.61	94.1297	19531.83	94.58189	19531.53	94.13045	19533.17
19541.25	94.38371	19542.13	94.38246	19526.1	94.38103	19532.47	94.37563	19532.75	94.85297	19531.41	94.38096	19528.42
19540.37	94.64127	19540.71	94.63291	19529.16	94.63087	19531.62	94.62655	19529.52	95.07457	19531.99	94.63157	19533.18
19536.25	94.88711	19545.08	94.88265	19529.05	94.88085	19530.22	94.8767	19532.19	95.32623	19530.86	94.88876	19532.78
19540.29	95.13409	19542.85	95.13357	19528.54	95.13506	19529.63	95.12873	19529.56	95.58122	19533.91	95.12956	19531.98
19538.07	95.38351	19541.46	95.382	19528.23	95.38333	19530.25	95.37704	19528.13	95.83234	19531.93	95.38111	19531.98
19538.8	95.64137	19539.71	95.6338	19528.31	95.63304	19530.46	95.62646	19528.92	96.07528	19531.91	95.63291	19533.7
19538.3	95.88752	19542.05	95.88804	19529.67	95.88178	19530.22	95.87595	19530.21	96.32731	19528.25	95.88991	19530.32
19538.81	96.13467	19542.54	96.13254	19527.46	96.13567	19530.28	96.12913	19532.68	96.58206	19531.22	96.13231	19535.24
19540.96	96.38471	19539.4	96.38128	19529.62	96.38344	19531.27	96.37759	19527.08	96.83409	19530.09	96.38177	19532.59
19538.26	96.6402	19543.42	96.63429	19524.5	96.63462	19531.21	96.62706	19530.28	97.07639	19529.23	96.63173	19532.31
19536.66	96.88634	19541	96.88645	19526.97	96.8827	19528.07	96.87703	19531.31	97.32776	19531.59	96.89525	19532.73
19538.78	97.13549	19540.44	97.13451	19529.43	97.13495	19526.61	97.12812	19532.97	97.5841	19528.35	97.13971	19530.77
19537.88	97.38478	19542.01	97.38161	19529.05	97.3826	19528.32	97.3786	19530.96	97.83179	19530.47	97.38158	19532.11
19537.55	97.64233	19539.31	97.63447	19528.85	97.63496	19528.41	97.62907	19531.93	98.0767	19531.17	97.63132	19531.78
19538.27	97.88795	19540.6	97.88525	19527.62	97.88389	19530.45	97.87718	19529.31	98.33016	19532.44	97.89182	19532.41
19538.59	98.1362	19542.64	98.13475	19523.5	98.13688	19527.97	98.12757	19532.33	98.58236	19533.16	98.13361	19531.23
19541.07	98.38715	19538.67	98.3811	19528.26	98.38195	19529.05	98.37738	19529.7	98.83184	19529.84	98.38385	19531.57
19538.86	98.64364	19542.42	98.63515	19529.1	98.63498	19530.25	98.6315	19529.67	99.07562	19530.32	98.63482	19532.16
19539.06	98.88801	19538.63	98.88531	19528.63	98.88451	19528.48	98.8785	19531.66	99.33071	19530.77	98.89132	19529.88
19536.72	99.13766	19542.83	99.13987	19524.74	99.14259	19529.85	99.13488	19527.91	99.59178	19531.83	99.13651	19529.68
19535.24	99.38844	19541.11	99.38131	19526.01	99.38295	19527.33	99.37698	19526.95	99.8404	19532.66	99.38304	19531.16
19539.15	99.64182	19540.21	99.63659	19526.2	99.63708	19530.26	99.63036	19529.6	100.076	19529.54	99.63492	19528.36
19540.01	99.88776	19543.45	99.88553	19528.9	99.88473	19525.84	99.88042	19529.7	100.3318	19531.13	99.89104	19530.64
19538.07	100.1338	19539.75	100.1375	19526.13	100.1366	19532.7	100.1293	19527.56	100.5864	19528.14	100.1322	19531.35
19536.98	100.3937	19543.05	100.3851	19527.72	100.3851	19529.59	100.38	19530.76	100.8359	19531.98	100.3858	19531.64
19537.85	100.644	19540.68	100.639	19530.04	100.6358	19529.37	100.6299	19529.96	101.0788	19533.09	100.6369	19532.93
19539.79	100.8929	19539.26	100.8886	19527.01	100.8883	19528.27	100.8804	19529.68	101.3321	19529.14	100.8943	19532.24
19540.07	101.1377	19540.06	101.1367	19526.76	101.1403	19527.73	101.134	19528.19	101.5851	19530.28	101.1372	19526.54
19536.62	101.3882	19536.62	101.3854	19528.11	101.3857	19529.89	101.3816	19530.38	101.8363	19531.75	101.3848	19530.76
19539.34	101.6442	19540.99	101.6408	19524.06	101.6362	19527.82	101.6314	19530.26	102.0799	19527.57	101.6349	19532.96
19536.68	101.8906	19538.38	101.8867	19524.07	101.8864	19530.22	101.8791	19529.91	102.3326	19527.32	101.8931	19530.09
19535.75	102.1374	19540.92	102.1372	19525.2	102.1397	19531.16	102.1315	19525.11	102.5852	19529.91	102.1401	19532.08
19538.34	102.3906	19539.79	102.3864	19525.77	102.3871	19527.11	102.3814	19526.95	102.8366	19529.78	102.3882	19533.02
19536.66	102.6445	19538.13	102.6394	19528.06	102.6406	19525.42	102.6356	19530.04	103.0803	19529.42	102.6354	19533.88
19536.81	102.8893	19538.42	102.8897	19526.73	102.8867	19528.87	102.8803	19529.38	103.334	19531.29	102.8935	19533.89
19538.46	103.1386	19540.88	103.1366	19529.05	103.1371	19527.61	103.1314	19531.16	103.5885	19526.25	103.1376	19530.34
19535.97	103.3921	19539.79	103.3849	19526.17	103.3872	19525.87	103.3809	19528.06	103.8366	19529.61	103.3873	19532.42
19537.93	103.6452	19540.89	103.6392	19525.71	103.6394	19528.06	103.633	19526.63	104.082	19535.13	103.6369	19533.37

19537.98	103.8904	19539.68	103.9092	19529.19	103.8877	19526.88	103.883	19528.35	104.334	19524.66	103.8926	19530.74
19536.81	104.1371	19538.15	104.1389	19527.57	104.1391	19524.51	104.1314	19529.44	104.5869	19529.42	104.137	19531.04
19536.95	104.391	19538.5	104.3846	19527.46	104.3878	19527.68	104.381	19526.43	104.8351	19528.21	104.3869	19530.79
19535.2	104.646	19540.25	104.6393	19525.66	104.6398	19526.81	104.6339	19530.52	105.0791	19524.92	104.6382	19531.78
19536.32	104.8932	19540.91	104.8914	19528.02	104.8995	19525.21	104.8821	19527.29	105.3348	19527.55	104.8941	19533.74
19540.39	105.1387	19539.89	105.1403	19524.05	105.1406	19529.44	105.1347	19529.16	105.5892	19529.33	105.1378	19527.81
19538.38	105.3909	19540.61	105.3862	19526.38	105.3882	19527.6	105.3817	19527.06	105.8378	19527.4	105.3864	19532.95
19536.44	105.644	19538.24	105.6377	19526.87	105.6386	19527.18	105.634	19526.58	106.0806	19528.51	105.6395	19528.86
19536.52	105.8932	19539	105.8927	19522.03	105.8861	19528.05	105.8829	19529.42	106.3338	19527.24	105.8974	19530.32
19537	106.1393	19539.14	106.141	19526.25	106.1386	19526.13	106.1338	19528.76	106.59	19527.52	106.1385	19531.67
19537.53	106.39	19539.08	106.3881	19523.22	106.3886	19529.13	106.3832	19527.72	106.839	19528.51	106.3868	19529.91
19536.87	106.6458	19539.75	106.6369	19525.51	106.6394	19528.51	106.6367	19529.51	107.0843	19528.49	106.6382	19529.32
19534.65	106.8933	19541.07	106.8902	19522.2	106.8883	19528.08	106.8817	19527.55	107.3339	19526.13	106.8955	19528.13
19536.21	107.1403	19535.95	107.1403	19526.11	107.1411	19529.02	107.1345	19529.84	107.5877	19528.32	107.1405	19529.97
19537.33	107.3927	19537.82	107.3885	19525.76	107.3887	19525.82	107.3832	19529.64	107.8374	19529.41	107.3866	19532.04
19536.29	107.647	19540.57	107.6398	19525.28	107.6403	19525.03	107.6361	19529.82	108.0823	19527.67	107.6369	19530.42
19536.97	107.8922	19538.5	107.8897	19526.53	107.89	19530.24	107.8824	19532.77	108.3356	19526.7	107.8954	19528.87
19534.79	108.141	19538.32	108.1415	19525.94	108.1383	19527.76	108.1328	19527.95	108.5889	19530.93	108.1401	19523.86
19535.51	108.3931	19540.46	108.3888	19522.96	108.3879	19527.09	108.3839	19527.29	108.8393	19532.2	108.3891	19526.36
19535.49	108.6495	19542.35	108.6419	19524.21	108.6402	19526.26	108.6393	19528.49	109.0833	19528.44	108.6369	19529.93
19536.04	108.8916	19536.79	108.893	19527.78	108.9029	19525.44	108.8856	19527.73	109.3364	19527.7	108.8939	19529.03
19537.33	109.1391	19539.36	109.1386	19526.43	109.1415	19527.55	109.1348	19527.95	109.5887	19528.16	109.1384	19529.9
19536.59	109.3952	19538.69	109.3901	19523.54	109.3884	19527.78	109.3828	19529.24	109.8457	19527.71	109.3903	19532
19537.49	109.6516	19536.86	109.6472	19525.24	109.6402	19525.83	109.6386	19530.22	110.082	19528	109.6405	19531
19536.06	109.8928	19538.46	109.898	19524.25	109.8908	19526.52	109.8847	19529.66	110.3373	19525.03	109.8972	19531.71
19535.46	110.1388	19538.48	110.1419	19525.28	110.1415	19527.99	110.1357	19524.22	110.5896	19526	110.1391	19529.37
19535.26	110.3936	19538	110.3884	19524.86	110.3885	19526.2	110.3961	19528.88	110.8387	19527.96	110.3889	19531.11
19536.26	110.6481	19537.51	110.6394	19525.84	110.64	19527.33	110.6356	19527.04	111.0822	19528.07	110.6409	19529.1
19538.36	110.8931	19537.91	110.893	19525.74	110.8901	19525.3	110.8854	19526.53	111.3369	19525.73	110.9017	19531.2
19537.47	111.1398	19536.07	111.1431	19523.27	111.1417	19530.46	111.1369	19530.66	111.5924	19528.65	111.1493	19525.34
19535.23	111.3917	19536.96	111.3888	19523.54	111.3913	19526	111.3845	19526.31	111.8394	19530.49	111.3895	19531.71
19538.16	111.6502	19536.94	111.6411	19525.78	111.6396	19527.78	111.6359	19523.34	112.0842	19526.12	111.638	19527.46
19537.46	111.8961	19537.49	111.8928	19525.09	111.8893	19526.79	111.8852	19526.7	112.3368	19528.79	111.9019	19527.77
19535.92	112.141	19537.29	112.1425	19526.32	112.1424	19526.44	112.1368	19527.17	112.5901	19527.6	112.1461	19531.86
19535.51	112.393	19537.54	112.3898	19527.14	112.3909	19525.31	112.3861	19532.49	112.8408	19527.47	112.3915	19527.83
19536.39	112.6501	19539.62	112.6398	19528.1	112.6407	19526.28	112.6384	19528.8	113.0857	19525.53	112.6413	19529.55
19536.75	112.8957	19538.99	112.8929	19527.45	112.8894	19525.66	112.8843	19524.81	113.3378	19526.76	112.8966	19528.55
19533.97	113.1414	19535.29	113.1425	19525.86	113.1406	19524.52	113.1359	19527.92	113.591	19524.75	113.1413	19527.71
19533.05	113.393	19539.47	113.3906	19524.67	113.3909	19524.47	113.3849	19527.66	113.8409	19528.95	113.3912	19525.68
19535.22	113.6497	19538.29	113.6406	19527.36	113.6429	19523.94	113.6387	19525.26	114.0854	19527.19	113.6449	19531.16
19534.76	113.8949	19537.89	113.8905	19526.44	113.8926	19527.24	113.8867	19527.75	114.3383	19526.35	113.8991	19528.85
19537.17	114.1429	19537.09	114.1421	19522.46	114.143	19523.69	114.1373	19527.95	114.5893	19529.38	114.1412	19527.3
19535.44	114.3955	19537.74	114.3925	19522.04	114.3896	19526.77	114.3863	19526.82	114.8404	19526.55	114.3903	19529.64
19532.34	114.6507	19538.64	114.6408	19522.64	114.6449	19528.2	114.6393	19524.06	115.0843	19527.38	114.6436	19525.12
19536.1	114.8953	19538.01	114.8939	19522.27	114.893	19524.9	114.884	19523.02	115.3392	19527.75	114.8987	19528.41
19534.73	115.1427	19536.05	115.1544	19524.57	115.1454	19525.76	115.1375	19527.45	115.5919	19528.58	115.1439	19527.12
19533.38	115.3958	19534.92	115.392	19524.86	115.3931	19525.28	115.386	19527.8	115.8424	19528.12	115.3915	19528.73
19536.17	115.6498	19538.72	115.6453	19523.87	115.6449	19528.39	115.6412	19524.97	116.086	19526.4	115.642	19527.8
19534.33	115.8986	19535.83	115.8968	19525.67	115.8914	19525.13	115.887	19526.86	116.3388	19528.25	115.8982	19530.02
19535.45	116.1414	19537.9	116.143	19524.49	116.1448	19528.11	116.1379	19526.2	116.5916	19524.63	116.1432	19528.85
19535.98	116.4062	19537.42	116.3904	19527.18	116.3912	19529.33	116.3874	19524.82	116.8417	19529.73	116.3907	19527.05
19534.79	116.6523	19537.33	116.6434	19523.33	116.641	19526.53	116.6413	19524.2	117.0879	19525.7	116.6416	19528.32
19537.14	116.8986	19537.72	116.8958	19522.13	116.8935	19525.65	116.8887	19527.4	117.338	19528.59	116.9007	19528.16
19534.2	117.144	19535.67	117.1449	19523.06	117.143	19525.42	117.1366	19526.78	117.5926	19525.97	117.1473	19526.76
19533.38	117.3981	19536.87	117.392	19520.87	117.3955	19527.22	117.3866	19525.78	117.8424	19527.13	117.3921	19528.85
19535.27	117.6505	19538.64	117.6431	19526.52	117.6422	19525.94	117.6388	19522.59	118.0879	19524.14	117.6426	19528.85
19536.71	117.8982	19537.07	117.8957	19524.78	117.8919	19523.11	117.8892	19522.33	118.3394	19528.41	117.9012	19527.35
19536.24	118.1446	19538.42	118.1458	19526.46	118.1444	19529.49	118.1405	19527.03	118.5911	19528.23	118.1448	19525.63
19535.41	118.3953	19539.45	118.3925	19526.4	118.3955	19524.81	118.3875	19527.05	118.8445	19524.46	118.3932	19526.26
19533.96	118.6586	19536.81	118.6433	19523.11	118.6427	19522.88	118.638	19529.71	119.0872	19523.42	118.6416	19527.97
19535.78	118.9002	19536.17	118.8943	19523.63	118.892	19525.29	118.8893	19524.59	119.3406	19528.47	118.899	19528.53
19535.35	119.1447	19537.98	119.1443	19526.15	119.1444	19525.17	119.1395	19526.02	119.5899	19527.2	119.1444	19528.18
19535.08	119.3987	19535.79	119.3925	19526.02	119.3952	19525.62	119.3885	19523.68	119.842	19525.03	119.3925	19527.11
19536.47	119.6524	19537.61	119.6467	19525.88	119.6479	19526.46	119.6392	19526.37	120.0876	19526.2	119.6436	19525.17
19533.7	119.897	19536.5	119.8958	19521.37	119.8939	19524.95	119.8873	19528.17	120.342	19525.22	119.9025	19529.08
19534.2	120.1457	19537.42	120.1439	19522.64	120.159	19528.17	120.1407	19525.72	120.5932	19523.08	120.1441	19525.71
19536.03	120.3991	19534.68	120.395	19522.66	120.3963	19525.01	120.3902	19527.29	120.8402	19525.42	120.394	19526.84
19533.6	120.65	19537.84	120.6451	19522.88	120.642	19525.76	120.6405	19523.98	121.0874	19528.28	120.6416	19527.91
19534.23	120.8947	19534.81	120.8935	19523.56	120.8945	19524.59	120.8856	19523.57	121.3403	19527.09	120.8993	19527.76
19533.17	121.1425	19536.79	121.1412	19524.7	121.1434	19526.3	121.1375	19525.77	121.5896	19525.08	121.1402	19525.32

19536.76	121.396	19537.71	121.3909	19523.42	121.3917	19523.71	121.3869	19528.27	121.8398	19524.58	121.3912	19531.1
19533.8	121.6486	19539.05	121.6464	19524.32	121.6426	19525.28	121.6377	19525.52	122.084	19525.5	121.6415	19527.77
19535.65	121.8959	19538.39	121.8953	19525.16	121.9042	19524.65	121.8862	19526.67	122.3395	19522.47	121.8985	19529.01
19534.09	122.1408	19538.04	122.1434	19521.71	122.1443	19525.63	122.1373	19528.23	122.5918	19527.57	122.145	19526.66
19534.49	122.3963	19535.87	122.3916	19521.17	122.3917	19523.83	122.3882	19524.08	122.8407	19526.32	122.3934	19526.14
19535.21	122.65	19532.66	122.6429	19525.67	122.6416	19526.14	122.6395	19526.12	123.0856	19525.28	122.6397	19526.17
19536.14	122.8976	19536.4	122.8957	19523.13	122.8931	19525.99	122.8888	19526.63	123.3384	19525.96	122.8991	19529.24
19536.71	123.1423	19536.87	123.1461	19522.33	123.1446	19522.59	123.1386	19524.96	123.5891	19525.21	123.1431	19527.96
19534.8	123.3966	19537.31	123.3918	19524.45	123.3943	19524.85	123.3864	19524.59	123.8421	19522.9	123.3929	19530.52
19534.91	123.6517	19538.02	123.6405	19523.04	123.6427	19524.22	123.6396	19525.25	124.0863	19528.15	123.6429	19528.97
19535.68	123.8991	19537.28	123.8935	19524.05	123.8918	19522.45	123.8889	19527.11	124.3399	19526.8	123.9001	19526.94
19535.08	124.144	19537.96	124.1449	19524.85	124.1462	19524.79	124.1391	19527.18	124.59	19527.06	124.1436	19526.27
19531.51	124.397	19534.45	124.3931	19521.66	124.3936	19524.06	124.3859	19525.67	124.8446	19524.27	124.3928	19526.68
19534.18	124.6524	19536.63	124.6452	19525.23	124.6443	19527.15	124.6387	19521.58	125.0864	19525.33	124.6431	19528.05
19532.82	124.8986	19534.63	124.8926	19523.59	124.8925	19520.68	124.8886	19527.41	125.3403	19525.53	124.9001	19524.3
19532.59	125.1443	19536.18	125.1451	19522.08	125.1443	19525.54	125.1405	19525.04	125.5921	19527.68	125.1428	19531.26
19534.95	125.44	19538.08	125.3925	19526.07	125.3934	19524.33	125.3873	19524.98	125.8416	19526.14	125.3927	19528.83
19535.3	125.6906	19533.76	125.6445	19522.49	125.6453	19525.26	125.6384	19523.88	126.0869	19527.17	125.6445	19525.42
19535.87	125.9097	19533.37	125.897	19522.03	125.8946	19521.43	125.8901	19527.68	126.3414	19526.44	125.9003	19527.16
19532.63	126.1451	19535.9	126.1433	19518.74	126.1428	19525.36	126.1412	19522.86	126.5912	19524.86	126.1435	19527.41
19534.12	126.3964	19538.26	126.3915	19520.22	126.3926	19521.67	126.3892	19528.88	126.8439	19523.01	126.3931	19531.4
19535.36	126.6518	19537.27	126.646	19520.71	126.6472	19525.27	126.6397	19524.03	127.0859	19526.85	126.6449	19526.57
19535.78	126.8983	19535.62	126.8945	19526.56	126.8977	19524.63	126.8893	19522.81	127.3436	19524.19	126.9013	19526.56
19534.32	127.1456	19535.65	127.1457	19528.01	127.1462	19524.96	127.1413	19526.64	127.5937	19524.58	127.1469	19526.31
19532.53	127.3994	19537.23	127.3924	19524.19	127.3921	19525.2	127.3901	19523.56	127.8444	19519.36	127.3934	19525.89
19534.96	127.6539	19537.64	127.6449	19525.2	127.6443	19523.84	127.6395	19525.71	128.0867	19527.39	127.6433	19524.21
19535.15	127.8995	19536.7	127.8939	19522.69	127.8951	19524.15	127.8898	19527.6	128.3418	19527.31	127.901	19524.41
19536.1	128.1456	19536.21	128.1445	19523.47	128.1467	19523.91	128.1386	19523.97	128.5932	19525.92	128.1455	19524.23
19533.89	128.3994	19537.34	128.3933	19520.67	128.3945	19523.13	128.3885	19520.48	128.8462	19522.61	128.3949	19526.54
19534.36	128.6522	19533.66	128.6448	19524.25	128.6457	19524.27	128.6412	19524.27	129.0866	19525.24	128.6448	19523.49
19535.77	128.8969	19534.37	128.8951	19522.83	128.8941	19523.54	128.8911	19522.76	129.3414	19523.31	128.904	19526.48
19532.96	129.1447	19536.71	129.1456	19526.34	129.1461	19523.89	129.1402	19525.48	129.5933	19523.86	129.1444	19525.83
19533.06	129.4	19539.03	129.3932	19519.66	129.3951	19524.5	129.3898	19525.35	129.8468	19528.22	129.395	19526.37
19531.53	129.6516	19537.94	129.6486	19523.2	129.6493	19524.25	129.6426	19523.01	130.089	19525.58	129.6453	19526.88
19534.17	129.8992	19537.45	129.8954	19524.61	129.8948	19525	129.8906	19526.77	130.3419	19522.47	129.9005	19528.83
19531.97	130.1453	19536.83	130.1464	19521.24	130.1472	19523.74	130.1412	19523.8	130.5924	19526.51	130.1442	19526.93
19533.49	130.3993	19535.03	130.3948	19522.57	130.3964	19524.2	130.3895	19521.26	130.8456	19523.63	130.3946	19525.87
19534.12	130.6522	19536.12	130.644	19523.05	130.6479	19522.22	130.641	19525.19	131.0888	19522.02	130.6459	19524.42
19535.67	130.9005	19537.85	130.8943	19523.39	130.9008	19523.19	130.8923	19522.27	131.3419	19528.49	130.9044	19527.82
19533.23	131.1453	19534.56	131.1478	19519.61	131.1477	19524.09	131.1431	19522.78	131.596	19524.94	131.1479	19527.27
19533.57	131.4	19535.14	131.3965	19523.94	131.395	19523.43	131.3894	19524.84	131.846	19524.42	131.397	19525.71
19535.83	131.654	19534.82	131.6489	19524.31	131.6497	19526.91	131.6578	19524.02	132.0899	19524.14	131.6449	19527.48
19533.76	131.9017	19535.9	131.8943	19522.94	131.8976	19524.43	131.8914	19521.94	132.344	19524.48	131.9043	19525.48
19529.51	132.1462	19533.97	132.1455	19520.29	132.1478	19524.27	132.1428	19526.22	132.5933	19526.34	132.1509	19529.01
19535.27	132.3999	19536.61	132.3956	19523.51	132.3961	19524.42	132.3907	19526.84	132.8449	19526.56	132.3966	19526.35
19533.78	132.6541	19536.65	132.6493	19521.86	132.648	19522.41	132.646	19524.16	133.0884	19525.79	132.6443	19526.14
19532.64	132.9021	19537.52	132.894	19519.59	132.8989	19523.21	132.8923	19524.86	133.3445	19525.25	132.9048	19523.33
19532.8	133.1483	19533.79	133.1482	19526.37	133.1502	19524.52	133.1416	19521.65	133.596	19522.18	133.1486	19521.49
19534.79	133.4015	19534.71	133.3959	19525.64	133.397	19524	133.3919	19521.03	133.8621	19523.48	133.3984	19522.6
19533.13	133.6528	19533.24	133.6481	19519.73	133.6479	19523.51	133.6435	19524.9	134.0932	19527.05	133.6457	19523.57
19533.91	133.9018	19532.17	133.8961	19522.96	133.8973	19525.66	133.8899	19524.43	134.3429	19523.83	133.9042	19522.19
19534.48	134.1503	19536.87	134.1471	19522.47	134.1485	19521.97	134.1419	19522.55	134.5935	19527.18	134.1468	19525.88
19533.73	134.3985	19534.69	134.3955	19522.29	134.3964	19523.88	134.3929	19526.58	134.8474	19524.17	134.3968	19524.07
19534.34	134.6539	19532.94	134.6494	19522.31	134.6488	19524.6	134.6449	19522.6	135.0913	19526.16	134.6464	19526.34
19529.14	134.9064	19535.03	134.8982	19523.48	134.8965	19523.77	134.8912	19522.92	135.3449	19524.25	134.9032	19524.13
19533.22	135.1515	19536.05	135.1479	19521.53	135.1481	19522.6	135.1448	19521.69	135.5972	19526.73	135.1475	19527.93
19532.13	135.406	19533.5	135.3959	19525.41	135.3961	19523.82	135.3922	19522.51	135.855	19525.54	135.3991	19527.7
19532.55	135.652	19535.96	135.6481	19521.94	135.65	19524.18	135.6444	19520.72	136.0914	19522.65	135.6475	19526.36
19534.89	135.9033	19532.65	135.8979	19523.3	135.8977	19522.69	135.8934	19522.52	136.3461	19524.63	135.923	19527.12
19534.92	136.1506	19534.44	136.1503	19522.73	136.1488	19524.64	136.1442	19522.96	136.5946	19524.29	136.1555	19529.04
19532.22	136.401	19534.01	136.3968	19519.32	136.3978	19525.13	136.3902	19524	136.8488	19523.47	136.398	19528.17
19534.1	136.6544	19534.34	136.6486	19523.18	136.6485	19523.3	136.6459	19524.93	137.0914	19522.92	136.6484	19528.56
19532.6	136.9013	19536.57	136.8954	19524.84	136.9014	19519.95	136.8925	19524.82	137.3471	19524.95	136.9042	19524.44
19534.78	137.1514	19533.39	137.1488	19521.7	137.1487	19521.69	137.1445	19525.82	137.5987	19524.34	137.1509	19524.2
19532.46	137.4039	19536.41	137.4009	19521.21	137.3963	19524.82	137.3917	19521.19	137.8565	19523.9	137.3976	19525.43
19534.18	137.6555	19532.94	137.651	19523.02	137.6496	19524.86	137.6437	19521.73	138.0912	19524.5	137.6463	19522.29
19533.75	137.9004	19534.36	137.8953	19521.58	137.8992	19522.45	137.8927	19523.86	138.347	19524.85	137.9063	19524.18
19532.73	138.1508	19532.78	138.1481	19521.09	138.157	19523.08	138.1443	19523.54	138.5966	19522.29	138.1491	19525.95
19534.05	138.4023	19536.44	138.3971	19520.18	138.3975	19524.73	138.3937	19523.28	138.8517	19524.78	138.405	19523.94
19533.85	138.6537	19538.18	138.6493	19523.33	138.6506	19522.15	138.6498	19522.59	139.0907	19525.69	138.6516	19524.67

19531.47	138.9025	19533.69	138.8971	19523.71	138.9007	19525.49	138.8917	19523.69	139.3478	19525.52	138.9073	19525.6
19531.82	139.1499	19532.48	139.1499	19523.01	139.1536	19524.36	139.1459	19525.68	139.5959	19521.94	139.1497	19525.54
19533.38	139.4029	19533.49	139.3997	19520.63	139.3993	19520.75	139.393	19523.49	139.8476	19526.58	139.4002	19524.42
19532.56	139.6533	19536.75	139.651	19520.08	139.6509	19522.27	139.6466	19522.15	140.0917	19520.55	139.6467	19528.33
19534.82	139.9094	19534.93	139.8986	19522.84	139.8974	19523.34	139.893	19525.62	140.3491	19523.65	139.9079	19523.87
19534.08	140.1509	19533.13	140.1616	19521.16	140.1488	19522.51	140.143	19523.78	140.5959	19525	140.1493	19525.74
19531.72	140.404	19532.97	140.3972	19522.66	140.3981	19523.78	140.3942	19524.41	140.85	19521.92	140.4003	19524.11
19530.36	140.6566	19535.33	140.6505	19521.51	140.6549	19521	140.6476	19523.94	141.0923	19523.6	140.6501	19525.38
19532.14	140.9025	19535.38	140.8999	19520.81	140.9026	19524.65	140.8929	19525.19	141.3513	19524.94	140.9046	19523.8
19534.11	141.1494	19535.5	141.1539	19521.57	141.1516	19522.28	141.1447	19521.7	141.5967	19522.23	141.1497	19525.92
19532.72	141.4032	19532.96	141.3972	19523.27	141.3973	19524.58	141.3935	19523.12	141.8594	19525.03	141.4022	19526.45
19531.86	141.6587	19533.99	141.6495	19522.89	141.6509	19524.91	141.6507	19521.57	142.0949	19526.66	141.6492	19524.88
19532.93	141.9052	19532.74	141.8991	19521.62	141.9012	19522.17	141.8941	19519.35	142.3521	19525.82	141.9064	19524.61
19531.74	142.1493	19535.75	142.1524	19525.21	142.1519	19523.59	142.1446	19526.17	142.5962	19524.13	142.1525	19525.59
19530.33	142.403	19535.32	142.399	19521.48	142.3997	19521.7	142.3942	19521.79	142.8485	19525.41	142.4013	19526.05
19534.09	142.656	19536.57	142.6485	19523.82	142.6539	19525.19	142.6488	19519.99	143.0914	19525.27	142.6508	19523.42
19533.79	142.9063	19536.85	142.8995	19521.72	142.9055	19526.41	142.8958	19527.32	143.3509	19521.86	142.9067	19525.99
19535.13	143.1498	19534.53	143.1659	19522.44	143.1546	19520.71	143.147	19522.88	143.5975	19525.62	143.1492	19527.28
19533.44	143.4037	19533.49	143.3998	19521.88	143.3993	19520.91	143.3928	19522.76	143.852	19522.88	143.4	19525.3
19532.84	143.6583	19538.36	143.654	19524.66	143.6529	19521.66	143.6485	19522.04	144.0911	19525.86	143.6525	19524.1
19529.64	143.9048	19535.09	143.9011	19521.96	143.9019	19523.88	143.8966	19523.75	144.3479	19521.15	143.9086	19527.71
19530.05	144.1525	19534.93	144.1535	19522.69	144.1545	19522.56	144.1482	19522.88	144.5978	19525.24	144.1519	19525.22
19534.75	144.4073	19536.28	144.4011	19520.49	144.3993	19519.9	144.3955	19522.78	144.8518	19525.08	144.3988	19523.96
19530.56	144.656	19534.99	144.6518	19517.91	144.6539	19524.73	144.6535	19521.47	145.093	19526.5	144.6486	19525.01
19533.43	144.9044	19534.72	144.9021	19522.69	144.8993	19523.25	144.8939	19525.15	145.3489	19520.93	144.9055	19527.99
19531.74	145.1525	19534.08	145.1533	19522.02	145.1524	19520.61	145.1483	19522.18	145.5965	19525.31	145.1519	19527.23
19533.48	145.4056	19533.1	145.4	19521.1	145.4016	19520.06	145.3957	19522.96	145.8514	19519.74	145.4002	19525.45
19531.76	145.6592	19533.76	145.6513	19523.3	145.6505	19524.91	145.6506	19525.57	146.0939	19524.43	145.6499	19525.66
19531.95	145.9036	19532.56	145.9013	19519.95	145.9004	19520.35	145.8951	19521.45	146.3483	19520.55	145.9048	19525.23
19532.35	146.1518	19531.88	146.1575	19520.66	146.1504	19524.02	146.1479	19521.45	146.596	19524.36	146.1519	19526.72
19531.34	146.4072	19532.92	146.3994	19521.68	146.4009	19522.72	146.3963	19523.88	146.8518	19525.19	146.4018	19524.36
19531.52	146.6574	19536.32	146.65	19521.77	146.6558	19520.68	146.6554	19520.13	147.0948	19522.3	146.6539	19523.37
19530.3	146.9051	19533.14	146.9026	19522.48	146.9043	19520.11	146.8959	19521.49	147.3489	19522.4	146.9095	19524.09
19535.73	147.1528	19532.45	147.1536	19521.75	147.1505	19522.81	147.1469	19520.85	147.5994	19521.49	147.1563	19525.93
19531.71	147.4063	19534.88	147.4009	19521.23	147.4005	19520.34	147.3957	19523.63	147.8524	19522.44	147.4023	19523.43
19533.21	147.6751	19536.46	147.654	19524.06	147.652	19520.93	147.6532	19524.1	148.0948	19521.67	147.6529	19524.96
19531.83	147.9079	19533.39	147.9009	19521.99	147.904	19522.45	147.8973	19520.28	148.35	19523.8	147.9064	19527.46
19532.55	148.154	19529.74	148.1581	19519.75	148.1529	19520.94	148.1507	19523.03	148.5989	19525.04	148.1532	19525.5
19530.67	148.4051	19529.14	148.4016	19522.99	148.4006	19521.52	148.3951	19520.83	148.8529	19526.04	148.4024	19524.94
19535.08	148.6632	19535.76	148.653	19519.98	148.6547	19522.72	148.6539	19523.86	149.0934	19523.51	148.6517	19523.53
19531.08	148.9066	19532.93	148.9007	19525.08	148.9042	19520.26	148.8978	19522.67	149.3504	19519.2	148.9074	19524.64
19530.49	149.1547	19537.51	149.1547	19522.91	149.1534	19521.99	149.1499	19521.75	149.6	19522.3	149.1519	19524.9
19530.45	149.4067	19534.15	149.4016	19520.54	149.402	19521.74	149.3953	19521.84	149.8539	19520.09	149.4022	19525.57
19534.38	149.6576	19535.65	149.6543	19519.41	149.6541	19521.19	149.6518	19521.3	150.0949	19520.08	149.653	19525.41
19528.98	149.9073	19534.24	149.9022	19521.21	149.9033	19520.08	149.8976	19525.9	150.3529	19520.31	149.9099	19526.55
19532.58	150.1557	19535.8	150.153	19520.35	150.1553	19519.25	150.1513	19522.18	150.6024	19525.46	150.1544	19522.65
19534.54	150.412	19535.6	150.4069	19520.72	150.4059	19519.25	150.4011	19521.47	150.8739	19520.39	150.4048	19525.41
19531.88	150.6622	19537.83	150.6557	19518.71	150.6594	19521.77	150.653	19525.39	151.0981	19521.77	150.6548	19524.75
19531.62	150.9098	19537.64	150.9044	19519.31	150.9078	19517.82	150.8986	19524.9	151.3554	19524.5	150.9117	19527.65
19532.04	151.1568	19535.66	151.1566	19520.73	151.1557	19522.55	151.153	19522.46	151.5997	19523.28	151.1584	19523.75
19532.83	151.4105	19534.17	151.4066	19521.34	151.4065	19521.77	151.4011	19523.82	151.857	19523.93	151.4068	19526.62
19533.57	151.6624	19535.33	151.658	19522.77	151.6575	19521.5	151.6564	19521.35	152.1006	19522.72	151.655	19525.57
19531.72	151.9107	19533.95	151.9065	19523.41	151.9074	19523.28	151.8994	19524.3	152.3547	19523.08	151.9115	19523.51
19532.44	152.1577	19538.34	152.1592	19522.49	152.1566	19522.76	152.1528	19524.66	152.6003	19523.26	152.161	19522.86
19530.55	152.4105	19536.07	152.4039	19522.97	152.4056	19521.44	152.4013	19521.08	152.8576	19522.93	152.4091	19526.19
19531.96	152.6637	19540.01	152.6595	19520.55	152.6577	19518.13	152.6558	19524.54	153.0996	19522.6	152.6563	19523.58
19531.18	152.9124	19535.68	152.9069	19525.26	152.9087	19522.02	152.9005	19522.5	153.3559	19522.28	152.9149	19523.99
19532.68	153.1582	19535.66	153.1592	19524.04	153.1598	19521.58	153.1536	19521.45	153.603	19519.23	153.1566	19522.79
19530.39	153.4122	19533.93	153.407	19522.01	153.4067	19522.46	153.4012	19518.99	153.8595	19522.44	153.4055	19525.67
19534.39	153.6623	19534.51	153.6558	19521.15	153.6564	19523.16	153.6557	19522.12	154.1044	19524.64	153.658	19528.19
19532.36	153.9121	19534.71	153.907	19520.31	153.909	19519.6	153.9032	19522.73	154.3554	19524.12	153.9127	19525.92
19533.64	154.1597	19536.07	154.1601	19522.8	154.1591	19519.71	154.1535	19522.81	154.6032	19521.56	154.1582	19524.93
19532.52	154.4128	19536.64	154.408	19518.1	154.4069	19520.02	154.4024	19523.18	154.8556	19522.65	154.4067	19526.01
19532.28	154.6646	19534.66	154.6582	19519.09	154.6588	19525.39	154.6565	19526.4	155.1002	19520.98	154.6613	19524.95
19533.76	154.9111	19534.85	154.9061	19519.52	154.9095	19520.95	154.9039	19522.86	155.3539	19522.83	154.9119	19523.61
19532.78	155.1589	19533.82	155.1603	19520.07	155.1589	19523.43	155.1606	19523.18	155.6036	19522.42	155.1571	19523.82
19530.89	155.4129	19534.57	155.4073	19520.88	155.4074	19523.98	155.3998	19525.82	155.8583	19521.41	155.4063	19523.27
19531.5	155.6652	19534.55	155.6584	19522.48	155.659	19522.07	155.6555	19520.7	156.1005	19523.27	155.6591	19523.79
19532.2	155.9114	19535.51	155.9069	19519.18	155.9114	19523.31	155.9046	19519.73	156.356	19522.97	155.9122	19528.39
19531.96	156.1594	19538.8	156.1591	19519.7	156.1572	19520.83	156.1543	19523.41	156.6025	19521.92	156.1633	19523.24

19529.63	156.4148	19534.27	156.409	19522.05	156.4078	19523.38	156.402	19521.29	156.8581	19523.65	156.4088	19524.95
19529.79	156.6622	19537.07	156.6581	19520.5	156.6606	19519.06	156.6721	19520.86	157.1019	19525.67	156.6606	19524.96
19531.79	156.9113	19532.34	156.9069	19522.42	156.9117	19520.56	156.9057	19520.43	157.359	19523.13	156.9137	19524.15
19530.12	157.159	19536.72	157.1579	19523.36	157.159	19520.85	157.1545	19523.59	157.6065	19523.81	157.1627	19522.21
19532.78	157.4151	19533.52	157.4082	19518.8	157.4062	19522	157.4031	19520.52	157.8591	19525.39	157.4075	19522.97
19528.77	157.6644	19536.62	157.6592	19519.12	157.6603	19524.64	157.6571	19523.39	158.1027	19522.49	157.6595	19524.94
19531.32	157.9121	19532.41	157.9087	19517.6	157.9134	19522.22	157.9026	19522.29	158.357	19525.38	157.9141	19522.22
19534.27	158.1581	19539.86	158.1611	19520.38	158.1613	19521.6	158.1543	19524.12	158.6083	19524.23	158.1597	19521.68
19528.58	158.4158	19534.8	158.4084	19520.64	158.4071	19521.71	158.4035	19520.96	158.8615	19526.45	158.4079	19523.83
19529.63	158.6663	19534.7	158.6588	19524.58	158.6592	19521.93	158.6638	19523.63	159.1002	19524.08	158.6593	19526.61
19529.52	158.9136	19533.9	158.9081	19520.53	158.9114	19522.41	158.904	19519.77	159.3584	19522.07	158.9149	19524.32
19531.86	159.1583	19532.26	159.1614	19519.56	159.1615	19522.16	159.1533	19520.84	159.6074	19525.53	159.1611	19527.89
19534.07	159.4151	19533.25	159.4099	19523.17	159.4086	19520.9	159.4031	19519.7	159.8597	19524.89	159.409	19526.18
19529.5	159.6648	19535.2	159.6598	19523.41	159.6618	19522.67	159.6588	19520.34	160.1021	19519.64	159.6609	19521.91
19530.4	159.9147	19534.73	159.9072	19520.9	159.9112	19520.16	159.9052	19519.51	160.3585	19520.92	159.9143	19524.4
19532.56	160.1595	19534.7	160.1599	19521.26	160.1632	19524.43	160.1564	19524.14	160.6041	19520.64	160.1618	19523.53
19531.86	160.4163	19532.96	160.4312	19522.12	160.4107	19522.61	160.4042	19523.35	160.8578	19524.8	160.4099	19521.64
19530.45	160.6618	19534.77	160.6579	19517.84	160.6596	19520.64	160.6543	19523.25	161.1002	19521.59	160.6594	19524.19
19532.84	160.9116	19534.05	160.9067	19520.2	160.9094	19521.19	160.9029	19523.93	161.3555	19523.48	160.9108	19524
19531.1	161.1571	19534.65	161.1581	19522.83	161.159	19521.36	161.1536	19522.88	161.6016	19521.28	161.1571	19522.72
19529.68	161.4151	19534.66	161.4077	19521.11	161.4062	19519.88	161.401	19522.55	161.858	19521.33	161.407	19522.83
19531.25	161.6611	19536.53	161.6574	19522.51	161.6573	19521.23	161.666	19517.85	162.0998	19522.15	161.6574	19527.06
19531.56	161.911	19534.32	161.9049	19522.97	161.91	19521.59	161.9051	19521.79	162.3554	19521.7	161.9193	19521.8
19531.41	162.1582	19533.75	162.1582	19519.67	162.1583	19520.95	162.1545	19525.81	162.6042	19524.44	162.1748	19522.89
19527.3	162.4126	19533.52	162.4078	19518.52	162.4065	19520.12	162.4013	19524.3	162.8583	19523.34	162.4084	19519.99
19530.28	162.6601	19531.42	162.6589	19518.25	162.6574	19521.26	162.6571	19521.26	163.0993	19521.61	162.6612	19525.08
19532.09	162.911	19536.89	162.9062	19519.55	162.9104	19521.19	162.9026	19523.41	163.3559	19522.5	162.9125	19521.91
19529.15	163.1594	19531.17	163.1575	19519.37	163.1592	19520.64	163.1541	19519.3	163.6054	19521.8	163.1593	19523.38
19533.03	163.4132	19532.74	163.4082	19517.46	163.4057	19522.3	163.4022	19522.44	163.8586	19520.4	163.4068	19523.08
19529.77	163.6615	19535.16	163.6587	19520.8	163.6608	19522.17	163.657	19517.48	164.1013	19525.76	163.6587	19524.85
19530.75	163.9123	19534.57	163.9083	19520.16	163.9134	19521.83	163.902	19520.12	164.356	19524.82	163.9153	19524.03
19531.23	164.1593	19534.18	164.1577	19521.23	164.1597	19523.99	164.1532	19521.12	164.6063	19523.52	164.1611	19525.04
19531.53	164.4144	19535.33	164.4091	19522.28	164.4078	19520.03	164.4044	19516.4	164.8581	19521.96	164.4086	19523.23
19529.17	164.6624	19535.39	164.6602	19521.21	164.6619	19521.44	164.6578	19520.63	165.1014	19521.75	164.6574	19524.71
19530.07	164.9113	19534.51	164.909	19519.2	164.9108	19520.6	164.9027	19522.67	165.3553	19522.44	164.9138	19525.58
19531.65	165.1603	19535.18	165.1602	19519.06	165.1593	19522.85	165.1541	19524.73	165.6096	19522.79	165.1627	19522.72
19533.01	165.4136	19533.63	165.4098	19522.34	165.4092	19519.95	165.4044	19520.18	165.8609	19522.55	165.4104	19522.57
19530.24	165.6622	19532.87	165.6608	19520.97	165.6616	19521.2	165.6627	19522.51	166.1029	19524.05	165.6588	19525.29
19530.07	165.9132	19532.46	165.907	19519.39	165.9132	19520.5	165.9052	19521.81	166.3576	19523.09	165.9136	19524.62
19530.61	166.1583	19534.25	166.1588	19517.68	166.1593	19521.29	166.1559	19522.58	166.6	19522.15	166.1601	19523.74
19533.56	166.4133	19534.41	166.4103	19519.6	166.4086	19522.06	166.4025	19521.98	166.8607	19523.75	166.4093	19523.7
19528.6	166.6637	19535.85	166.6612	19522.03	166.6633	19520.02	166.6574	19522.84	167.1019	19520.95	166.6603	19525.34
19532.56	166.9144	19534.68	166.9088	19521.27	166.9133	19520.77	166.9052	19520.69	167.3578	19522.94	166.9134	19524.33
19530.73	167.1592	19535.93	167.159	19521.69	167.1597	19519.48	167.1565	19519.01	167.602	19519.62	167.1641	19521.69
19530.27	167.4139	19533.68	167.4105	19517.85	167.409	19517.86	167.4047	19519.75	167.8612	19522.11	167.4117	19522.25
19529.28	167.6661	19533.48	167.6597	19520.19	167.6613	19519.47	167.6597	19521.27	168.102	19521.63	167.6601	19523.03
19531.2	167.9142	19536.51	167.9079	19522	167.9145	19520.29	167.9039	19523.72	168.3572	19523.96	167.9146	19524.02
19530.23	168.1609	19532.94	168.1606	19518.14	168.1606	19521.93	168.1566	19517.12	168.6048	19520.12	168.1611	19520.12
19531.06	168.4127	19536.73	168.4108	19523.39	168.4085	19519.25	168.4082	19522.87	168.8618	19525.11	168.41	19523.86
19532.04	168.6671	19534.8	168.6627	19521.03	168.6662	19520.86	168.6614	19520.18	169.1028	19523.24	168.6606	19524.38
19532	168.9147	19535.57	168.9097	19523.02	168.914	19522	168.9042	19519.67	169.3604	19526.02	168.9173	19521.22
19532.49	169.161	19536.15	169.1597	19519.41	169.1621	19520.72	169.1574	19521.09	169.6059	19521.52	169.1594	19523.38
19529.49	169.4154	19536.38	169.4091	19517.93	169.4091	19519.55	169.4077	19519.64	169.8605	19523.67	169.4108	19522.7
19531.72	169.6637	19534.69	169.661	19520.87	169.6645	19516.85	169.6623	19520.47	170.1023	19522.55	169.6622	19522.21
19529.51	169.915	19530.76	169.9092	19518.24	169.9117	19522.29	169.9042	19519.72	170.3596	19521.16	169.9181	19522.85
19530.73	170.1618	19533.91	170.1615	19517.59	170.1632	19518.09	170.1564	19522.02	170.6055	19522.93	170.1626	19520.21
19532.56	170.4145	19534.2	170.4101	19518.44	170.4113	19524.03	170.4066	19518.22	170.8627	19522.15	170.4128	19522.56
19531.3	170.6656	19535.55	170.6594	19518.55	170.6621	19521.84	170.6786	19519.26	171.1062	19520.83	170.6616	19526.07
19530.18	170.9156	19534.2	170.9114	19521.44	170.9125	19518.5	170.905	19523.12	171.3624	19519.83	170.9178	19522.71
19530.24	171.1615	19534.91	171.1623	19519.39	171.1626	19522.66	171.1572	19522.55	171.6068	19522.37	171.1632	19523.31
19530.82	171.4157	19532.96	171.4104	19520	171.4121	19518.88	171.4084	19519.33	171.8605	19524.32	171.4111	19522.39
19531.3	171.6687	19533.88	171.6597	19517.25	171.6642	19520.94	171.6632	19520.37	172.1047	19520.31	171.6623	19526.01
19529.67	171.9168	19530.7	171.91	19521.23	171.927	19521.54	171.9057	19520.78	172.3596	19521.51	171.9136	19522.65
19531.21	172.1606	19531.24	172.1629	19518.54	172.1626	19520.84	172.156	19521.4	172.6057	19521	172.1659	19520.57
19530.26	172.4155	19535.05	172.4099	19519	172.4123	19522.57	172.4068	19521.53	172.8628	19518.34	172.4126	19526.5
19533.26	172.6709	19534.55	172.6634	19520.4	172.6651	19519.11	172.6645	19520	173.1046	19520.16	172.662	19522.07
19529.25	172.9166	19534.3	172.9123	19519.1	172.9194	19521.57	172.9066	19520.86	173.3598	19518.92	172.9147	19522.56
19527.95	173.1629	19534.15	173.1615	19517.33	173.1647	19517.3	173.1576	19521.93	173.6076	19522.71	173.1606	19523.02
19531.88	173.4159	19534.15	173.4108	19522.73	173.4106	19518.57	173.4162	19521.96	173.8634	19520.03	173.4108	19524.39
19529.93	173.6675	19532.55	173.6624	19519.24	173.6634	19517.37	173.6618	19519.61	174.1054	19525.66	173.6642	19524.48

19532.14	173.9175	19535.83	173.9128	19522.14	173.92	19521.73	173.9075	19525.6	174.3622	19525.91	173.9191	19522.27
19531.58	174.1642	19530.59	174.1619	19519.81	174.1633	19518.68	174.1588	19521.57	174.6067	19519.78	174.1639	19522.82
19531.86	174.417	19534.63	174.4118	19518.72	174.4106	19518.9	174.4112	19520.34	174.8634	19522.79	174.4145	19524.05
19530.21	174.6662	19532.92	174.6647	19522.28	174.665	19525.31	174.6672	19519.6	175.1042	19523.38	174.6629	19521.19
19531.95	174.9183	19535.43	174.9105	19519.21	174.9154	19516.51	174.9063	19522.17	175.3618	19522.83	174.9196	19522.12
19530.61	175.1653	19532.34	175.1628	19520.82	175.1649	19520.89	175.1589	19520.93	175.6071	19522.26	175.1654	19524.95
19526.47	175.4177	19535.21	175.4105	19521.93	175.4102	19523.46	175.408	19521.83	175.8658	19520.58	175.4123	19522.2
19532.24	175.6684	19533.04	175.6632	19518.47	175.6656	19520.88	175.6711	19518.91	176.1044	19519.65	175.6614	19524.01
19529.65	175.9174	19534.79	175.9121	19522.44	175.9165	19523.8	175.907	19519.22	176.3613	19520.76	175.9168	19524.72
19529.71	176.1635	19528.33	176.1639	19523.24	176.1644	19523.43	176.1576	19524.62	176.6079	19522.47	176.1665	19523.27
19525.9	176.4183	19531.24	176.4117	19520	176.4138	19520.23	176.4083	19523.59	176.8633	19523.71	176.4138	19520.63
19530.33	176.6698	19531.43	176.6645	19521.16	176.6655	19518.91	176.6658	19520.39	177.1085	19519.28	176.6621	19526.02
19530.27	176.9178	19534.49	176.914	19519.52	176.9148	19519.29	176.9086	19521.79	177.3606	19523.82	176.9224	19527.22
19530.52	177.1642	19531.31	177.1654	19517.85	177.1639	19520.18	177.1591	19520.27	177.608	19522.98	177.1672	19523.02
19531.66	177.4173	19537.06	177.4115	19519.03	177.4233	19517.06	177.4097	19522.01	177.8647	19523.14	177.4143	19523.14
19531.34	177.6692	19532.28	177.6624	19521.43	177.6645	19523.5	177.6654	19521.2	178.1054	19519.31	177.6629	19527.35
19531.56	177.9184	19536.02	177.9126	19521.19	177.9295	19521.14	177.9074	19519.69	178.3617	19520.23	177.9201	19523.33
19528.15	178.1625	19534.03	178.1649	19521.36	178.1658	19519.9	178.1601	19520.29	178.604	19519.78	178.1643	19523.95
19532.33	178.4174	19532.89	178.4143	19520.71	178.4132	19521.85	178.4086	19521.98	178.8665	19523.59	178.4192	19519.75
19529.06	178.6684	19533.24	178.6642	19517.91	178.6662	19517.51	178.666	19518.15	179.1069	19521.66	178.6647	19522.76
19529.52	178.9193	19533.45	178.9132	19516.37	178.9189	19518.76	178.9084	19520.64	179.361	19519.49	178.9195	19522.19
19529.55	179.1647	19530.86	179.1642	19518.48	179.1685	19520.59	179.1602	19515.73	179.6081	19520.15	179.1655	19523.97
19532.48	179.4166	19534.21	179.4143	19518.36	179.4126	19520.87	179.4089	19521.2	179.8653	19524.6	179.4176	19520.04
19529.52	179.6678	19530.5	179.6662	19521.61	179.6674	19523.17	179.6667	19520.4	180.1069	19520.64	179.6645	19522.22
19528.51	179.9201	19533.05	179.913	19519.95	179.9198	19520.94	179.9113	19521.19	180.3631	19523.17	179.9254	19523.82
19530.63	180.1647	19530.84	180.1729	19520.31	180.1695	19521.74	180.1623	19520.62	180.6093	19521.13	180.1671	19524.15
19530.29	180.4194	19531.27	180.4172	19522.44	180.4347	19522.92	180.4096	19520.1	180.8658	19519.51	180.4137	19521.94
19528.26	180.6696	19533.14	180.669	19521.72	180.6714	19520.99	180.6678	19520.79	181.1097	19519.35	180.663	19523.35
19530.36	180.9192	19534.41	180.9158	19520.04	180.9174	19520.29	180.9099	19521.65	181.3684	19522.49	180.9201	19525.57
19531.51	181.1662	19532.54	181.1669	19520.78	181.1684	19519.21	181.1629	19523.94	181.6077	19522.3	181.1663	19522.16
19530.41	181.4192	19533.35	181.4138	19517.64	181.4161	19517.95	181.4086	19517.04	181.8659	19522.06	181.4148	19523.74
19528.25	181.6721	19533.45	181.6675	19519.75	181.668	19518	181.6678	19524.33	182.1088	19521.7	181.6641	19521.92
19531.06	181.9189	19533.58	181.9179	19519.67	181.9201	19519.57	181.911	19519.21	182.3623	19525.45	181.9222	19522.8
19528.58	182.1647	19530.81	182.167	19518.83	182.1678	19518.11	182.1625	19515.74	182.6092	19521.45	182.1693	19524.29
19528.88	182.4202	19532.54	182.4142	19521.87	182.4157	19518.18	182.4105	19520.84	182.8671	19520.82	182.4154	19520.66
19529.16	182.669	19531.28	182.6681	19521.7	182.6674	19518.69	182.6683	19520.07	183.1091	19520.54	182.6655	19525.24
19530.67	182.9209	19534.06	182.9151	19520.09	182.9225	19520.38	182.9105	19520.9	183.3636	19521.81	182.9223	19523.08
19530.29	183.1672	19532.57	183.1811	19518.06	183.1692	19519.32	183.1627	19517.46	183.6075	19519.9	183.1674	19523.2
19530.96	183.4195	19529.76	183.4147	19520.09	183.4159	19520.81	183.4117	19522.77	183.8675	19521.18	183.4158	19525.81
19529.6	183.6735	19533.27	183.6676	19517.99	183.6696	19523.58	183.6706	19524.05	184.1082	19521.45	183.6653	19524.02
19530.23	183.9225	19534.1	183.9156	19518.7	183.9202	19520.86	183.9094	19515.5	184.369	19521.88	183.9215	19525.17
19531.82	184.1667	19535.12	184.1679	19518.31	184.1708	19518.09	184.1627	19523.77	184.6096	19522.51	184.1697	19521.31
19532.48	184.4209	19532.62	184.4143	19519.42	184.416	19520.81	184.4111	19519.02	184.8687	19524.6	184.4165	19522.96
19529.04	184.6698	19531.7	184.6675	19518.48	184.6696	19522.52	184.6679	19518.52	185.1087	19521.8	184.6658	19521.27
19529.68	184.9211	19530.04	184.9145	19517.37	184.9217	19520.23	184.9116	19522.39	185.3687	19521.77	184.9195	19524.28
19529.12	185.1677	19529.57	185.1667	19516.6	185.1702	19517.72	185.1636	19520.14	185.6095	19522.87	185.1663	19523.4
19530.14	185.4219	19532.32	185.4184	19517.69	185.4212	19520.46	185.411	19521.5	185.8664	19519.11	185.4158	19520.02
19528.13	185.6832	19532.35	185.6707	19520.63	185.6696	19517.41	185.6924	19519.78	186.1104	19522.16	185.6665	19521.8
19529.03	185.9243	19530.7	185.916	19517.5	185.9227	19517.97	185.9195	19517.86	186.3645	19522.05	185.9223	19525.25
19530.53	186.1681	19530.58	186.1679	19518.87	186.1712	19520.54	186.1644	19519.9	186.6097	19521.77	186.1686	19524.1
19531.01	186.4227	19534.18	186.4198	19522.33	186.4179	19522.49	186.4097	19520.45	186.8678	19521.33	186.4156	19520.45
19533.64	186.6685	19533.74	186.6695	19518	186.6715	19519.56	186.6695	19521.18	187.1117	19519.9	186.6667	19518.92
19528.72	186.9206	19531.77	186.9174	19518.77	186.933	19521.84	186.9122	19519.63	187.365	19519.52	186.9254	19521.42
19530.14	187.1694	19534.66	187.1681	19515.48	187.1718	19519.21	187.1649	19520.8	187.608	19518.49	187.1701	19522.27
19530.77	187.4236	19532.72	187.4157	19517.31	187.4172	19522.9	187.4148	19522.47	187.8677	19524.21	187.4184	19519.59
19530.46	187.6704	19531.66	187.6706	19519.2	187.6709	19517.26	187.6714	19522.21	188.1107	19522.46	187.6656	19525.02
19531.23	187.9218	19534.72	187.918	19518.35	187.924	19518.97	187.9115	19519.36	188.3648	19522.26	187.9222	19523.17
19531.77	188.1686	19533.58	188.1691	19517.96	188.1737	19518.86	188.1642	19519.1	188.6094	19519.05	188.1705	19520.72
19529.84	188.4225	19534.55	188.4177	19518.98	188.4169	19517.9	188.4128	19520.91	188.8681	19525.42	188.4183	19524.53
19533.96	188.6707	19532.57	188.6713	19517.96	188.67	19516.18	188.6676	19519.72	189.1121	19520.29	188.669	19520.51
19526.13	188.9227	19535.3	188.9174	19516.4	188.9228	19518.36	188.9125	19521.3	189.3647	19520.64	188.9204	19520.94
19525.45	189.1693	19533.93	189.1698	19517.5	189.1722	19520.39	189.1637	19519.28	189.6137	19520.66	189.1688	19523.02
19531.82	189.4212	19533.71	189.4198	19522.61	189.4194	19521.29	189.413	19521.6	189.8686	19517.44	189.4179	19523.16
19528.9	189.6713	19534.96	189.6704	19518.07	189.6704	19518.71	189.6744	19519.18	190.1146	19520.06	189.6684	19522.53
19529.05	189.9229	19532.02	189.9167	19515.49	189.9252	19522.51	189.9137	19522.21	190.3665	19520.64	189.9221	19521.78
19525.85	190.1683	19534.01	190.1692	19519.71	190.1712	19518.9	190.165	19518.66	190.6105	19517.52	190.1684	19524.23
19527.73	190.4247	19532.81	190.4183	19516.21	190.4184	19516.98	190.4126	19520.84	190.8737	19523.92	190.4194	19523.39
19529.4	190.6705	19530.32	190.6725	19518.66	190.6702	19520.82	190.6696	19520.25	191.1145	19522.77	190.6693	19523.51
19530.46	190.9218	19532.28	190.9194	19519.36	190.9339	19522.11	190.9146	19518.37	191.3661	19520.54	190.923	19521.22
19532.03	191.1704	19531.96	191.17	19518.34	191.1735	19523.33	191.165	19518.84	191.6123	19524.28	191.1699	19522.98

19532.72	191.4243	19529.76	191.4176	19519.66	191.4195	19517.71	191.4132	19518.29	191.8698	19522.03	191.4192	19523.54
19527.38	191.6705	19532.99	191.6724	19519.48	191.6793	19520.34	191.6733	19518.41	192.1126	19523.8	191.6676	19523.93
19531.84	191.9228	19532.35	191.9206	19517.25	191.9233	19518.74	191.9163	19521.16	192.3683	19517.61	191.9226	19524.6
19531.81	192.17	19536.8	192.1721	19520.67	192.1711	19519.35	192.1655	19520.59	192.6112	19520.52	192.1732	19522.36
19530.12	192.4247	19531.26	192.419	19520.2	192.4188	19514.98	192.4124	19519.83	192.8686	19519.69	192.4212	19524.48
19530.24	192.6716	19530.86	192.6689	19518.11	192.6729	19521.84	192.6728	19519.23	193.1105	19521.76	192.6685	19525.16
19530.03	192.9241	19528.74	192.9209	19516.61	192.9249	19524.89	192.9158	19518.19	193.3677	19519.52	192.9224	19524.19
19528.82	193.1693	19532.36	193.1722	19518.44	193.1704	19519.39	193.1648	19518.69	193.6176	19518.86	193.1715	19522.97
19526.44	193.4255	19531.47	193.4202	19516.93	193.4199	19518.45	193.4174	19521.62	193.8751	19522.73	193.4223	19520.73
19527.41	193.6745	19530	193.6746	19519.08	193.6709	19520.1	193.6743	19520.69	194.1234	19519.04	193.6698	19519.06
19529.25	193.9251	19532.05	193.9199	19515.5	193.9249	19515.9	193.915	19518.87	194.3737	19520.56	193.922	19521.5
19528.54	194.1706	19533.03	194.1695	19519.23	194.172	19517.53	194.1665	19522.28	194.6129	19521.81	194.173	19525.05
19528.19	194.4243	19533.53	194.4203	19516.51	194.4205	19519.67	194.4168	19518.38	194.8714	19521.26	194.4198	19522.49
19528.23	194.6738	19533.76	194.6719	19515.93	194.671	19518.75	194.6724	19522.93	195.113	19518.55	194.6709	19523.98
19529.55	194.9268	19532.74	194.9204	19518.8	194.923	19519.04	194.9146	19520.68	195.3677	19516.77	194.9237	19519.44
19533.33	195.1695	19533.46	195.1714	19518.43	195.1721	19522.89	195.166	19521.29	195.6137	19521.12	195.1718	19519.38
19529.08	195.4256	19534	195.4192	19518.32	195.4204	19521.97	195.4161	19522.99	195.8759	19521.91	195.4209	19520.41
19529.09	195.6745	19534.06	195.6744	19519.37	195.6744	19522.49	195.6751	19520.3	196.1153	19521.99	195.6721	19522.32
19528.9	195.926	19533.3	195.9212	19518.07	195.9241	19519.03	195.9169	19520.12	196.3675	19519.76	195.92	19522.03
19528.64	196.1719	19532.59	196.1713	19520.71	196.1718	19521.31	196.165	19521.37	196.6151	19520.69	196.1927	19521.46
19529.66	196.4247	19533.02	196.4215	19518.63	196.4216	19517.18	196.415	19521.38	196.8726	19524.39	196.4263	19523.93
19529.92	196.6751	19529.81	196.6716	19519.49	196.673	19520.35	196.6749	19520.38	197.1139	19520.76	196.6706	19521.1
19531.77	196.9264	19534.21	196.9217	19516.35	196.9303	19521.27	196.9175	19519.21	197.3689	19521.58	196.9239	19521.58
19529.81	197.1721	19529.86	197.1732	19520.14	197.1729	19517.88	197.1691	19519.95	197.6149	19521.82	197.1747	19523.13
19529.87	197.4273	19534.19	197.4216	19517.65	197.422	19519.63	197.4146	19519.58	197.8715	19521.41	197.4203	19521.61
19533.36	197.6735	19532.63	197.6758	19518.2	197.6742	19518.26	197.6736	19516.47	198.1135	19519.1	197.6728	19521.21
19527.87	197.9266	19531.26	197.9214	19517.83	197.9257	19518.78	197.9172	19522.74	198.369	19523.88	197.9262	19523.8
19530.31	198.1808	19530.29	198.1822	19518.39	198.1837	19518.84	198.1774	19520.84	198.6243	19521.87	198.1816	19522.31
19530.79	198.4297	19533.21	198.4221	19517.37	198.4212	19518.85	198.4186	19519.77	198.873	19520.01	198.4197	19523.37
19530.43	198.6727	19532.28	198.6766	19521.3	198.6758	19517.67	198.6761	19517.75	199.1164	19521.73	198.6737	19526.33
19529.75	198.9269	19531.84	198.9244	19518.31	198.928	19521.05	198.9186	19522.91	199.3688	19519.15	198.9269	19523.11
19531.16	199.1737	19529.55	199.173	19520.58	199.1753	19519.56	199.1687	19522.59	199.6156	19520.26	199.1749	19523.04
19529.88	199.4329	19532.16	199.4224	19520.13	199.4246	19518.57	199.4167	19518.83	199.8716	19521.31	199.4221	19521.99
19530.99	199.6818	19530.07	199.6729	19517.14	199.6756	19515.66	199.6725	19520.95	200.117	19521.61	199.6731	19523.5
19529.38	199.9284	19535.21	199.9206	19514.94	199.9271	19517.03	199.9173	19517.66	200.3735	19520.31	199.9247	19520.44
19532.25	200.1736	19533.72	200.1739	19518.99	200.1737	19518.69	200.1675	19522.09	200.62	19522.71	200.1764	19521.13
19531.85	200.4347	19528.82	200.4374	19517.28	200.4318	19517.8	200.4212	19520.74	200.876	19521.25	200.4279	19524.54
19528.34	200.6821	19531.72	200.6774	19517.49	200.6773	19519.76	200.6779	19518.35	201.119	19520.61	200.6746	19520.47
19528.64	200.9323	19532.06	200.9258	19517.56	200.9302	19519.25	200.9223	19520.98	201.3772	19521.77	200.9345	19518.2
19530.26	201.1766	19532.48	201.1748	19516.86	201.1774	19518.81	201.1747	19520.3	201.6195	19522.34	201.1799	19521.63
19527.54	201.4307	19533.81	201.427	19517.9	201.4292	19519.94	201.4202	19518	201.8805	19521.38	201.4282	19521.63
19525.94	201.6782	19532.08	201.6833	19518.08	201.6797	19517.66	201.6782	19523.5	202.1211	19523.34	201.6777	19519.7
19528.75	201.9335	19531.63	201.9293	19516.07	201.9309	19522.34	201.9234	19518.8	202.3754	19521.79	201.9296	19523.7
19529.73	202.1765	19534.44	202.1777	19519.62	202.1785	19518.53	202.1745	19518.74	202.62	19517.14	202.1835	19522.67
19526.88	202.4308	19533.27	202.4252	19517.48	202.4478	19521.06	202.4213	19520.73	202.8769	19517.24	202.4274	19520.21
19530.7	202.6804	19532.09	202.6794	19520.98	202.681	19521.36	202.6788	19519.29	203.121	19517.93	202.678	19522.96
19528.49	202.9335	19532.2	202.9281	19517.87	202.9357	19521.04	202.9236	19520.28	203.374	19520.97	202.929	19525.01
19528.58	203.1813	19533.69	203.1791	19516.21	203.1798	19518.65	203.1736	19521.73	203.6194	19519.7	203.1817	19524.88
19530.11	203.4475	19531.14	203.4256	19518.35	203.4274	19516.93	203.4213	19523.23	203.8789	19525.12	203.4259	19520.86
19528.75	203.679	19531.58	203.6793	19518.62	203.6808	19523	203.6831	19519.43	204.1233	19521.08	203.6801	19523.41
19527.32	203.935	19533.26	203.9284	19519.75	203.9323	19521.5	203.9231	19519.71	204.3764	19519.11	203.9335	19521.15
19529.96	204.1783	19531.3	204.1785	19517.85	204.1786	19515.59	204.1752	19521.94	204.6196	19520.35	204.1812	19524.41
19531.21	204.4327	19533.2	204.429	19517.81	204.4277	19518.36	204.4219	19518.8	204.8925	19520.3	204.4268	19521.86
19528.02	204.6769	19530.84	204.6806	19516.85	204.6786	19522.5	204.6813	19522.95	205.123	19520.37	204.68	19523.52
19527.36	204.9317	19533.48	204.9272	19518.25	204.9336	19517.03	204.9248	19520.06	205.3789	19522.42	204.9326	19521.99
19530.44	205.1791	19532.33	205.1797	19518.13	205.179	19520.57	205.1763	19517.81	205.6217	19521.95	205.1825	19522.12
19528.38	205.432	19529.34	205.4278	19515.31	205.4356	19519.9	205.4235	19517.03	205.8831	19522.65	205.4271	19519.36
19530.24	205.6833	19533.24	205.686	19515.59	205.6796	19518.57	205.6815	19520.13	206.1233	19522.58	205.6785	19520.79
19529.47	205.9341	19527.77	205.9323	19516.86	205.9321	19519.8	205.9252	19521.91	206.3759	19522.84	205.9314	19521.01
19531.21	206.1792	19533	206.1794	19514.77	206.1796	19519.44	206.1727	19522.79	206.6225	19519.31	206.182	19522.49
19527.08	206.4331	19532.21	206.4276	19515.42	206.4302	19518.15	206.4209	19518.67	206.8803	19521.8	206.43	19520.02
19528.78	206.6805	19530.24	206.6804	19518.52	206.6823	19520.69	206.6808	19519.43	207.1242	19521.63	206.6799	19523.94
19528.98	206.9353	19531.39	206.9289	19519.03	206.9341	19517.19	206.9266	19519.32	207.3766	19522.14	206.9351	19523.22
19528.38	207.1787	19531.1	207.1791	19519.81	207.1794	19516.15	207.1764	19519.99	207.6217	19522.36	207.186	19522.12
19527.9	207.4326	19533.96	207.4299	19518.86	207.4313	19522.95	207.422	19521.88	207.8801	19520.35	207.4288	19524.7
19530.51	207.683	19529.3	207.6788	19515.38	207.6826	19520.84	207.6832	19520.19	208.1257	19519.3	207.6805	19522.87
19529.77	207.934	19531.04	207.9309	19516.06	207.934	19519.85	207.9271	19520.15	208.377	19521.2	207.931	19520.76
19526.27	208.1807	19529.1	208.1844	19518.44	208.1814	19516.58	208.1763	19520.93	208.623	19522.66	208.1836	19522.37
19531.1	208.433	19530.4	208.4273	19520.28	208.4309	19518.69	208.4258	19522.9	208.881	19520.83	208.4292	19521.06
19527.6	208.6828	19534.34	208.6827	19519.8	208.683	19520.54	208.6832	19519.45	209.1241	19523.2	208.6805	19523.07

19530.24	208.9337	19533.62	208.9343	19517.47	208.9381	19517.96	208.9265	19521.46	209.3778	19516.58	208.9324	19523.01
19528.93	209.1806	19533.71	209.1971	19517.93	209.1806	19518.97	209.1774	19518.26	209.6228	19523.06	209.1832	19523.17
19527.48	209.4344	19527.59	209.4293	19513.14	209.4321	19519.97	209.4231	19521.2	209.8813	19520.81	209.4305	19525.37
19532.3	209.6804	19530.09	209.6815	19517.85	209.6837	19518.95	209.6812	19520.43	210.1238	19519.3	209.681	19520.18
19530.46	209.9364	19532.05	209.9315	19517.43	209.9354	19521.32	209.9265	19518.57	210.3774	19519.98	209.9355	19522.19
19527.15	210.1811	19532.84	210.184	19517.01	210.1815	19521.16	210.1762	19519.92	210.6221	19521.21	210.1828	19524.15
19529.91	210.4359	19532.3	210.4303	19517.38	210.4306	19520.08	210.4247	19518.14	210.8816	19521.55	210.4317	19521.32
19530.06	210.68	19530.27	210.6805	19518.89	210.6838	19519.07	210.6842	19520.48	211.1244	19523.43	210.6812	19519.5
19531.93	210.9372	19533.89	210.9332	19517.97	210.9357	19518.41	210.9265	19516.64	211.377	19522.47	210.9359	19521.47
19527.16	211.1823	19535.57	211.1829	19517.11	211.1834	19517.12	211.175	19517.6	211.6227	19520.36	211.184	19520.22
19527.92	211.4345	19530.2	211.4286	19518.83	211.4318	19518.29	211.4275	19518.71	211.881	19519.95	211.4328	19522.54
19529.55	211.6827	19530.59	211.6818	19513.93	211.683	19516.2	211.6859	19517.39	212.1277	19520.73	211.6797	19521.82
19527.76	211.9354	19528.07	211.9367	19517.25	211.9358	19516.94	211.9283	19519.42	212.3781	19523.3	211.9358	19524.53
19530.75	212.1839	19530.04	212.1822	19520.68	212.1832	19522.09	212.177	19518.68	212.6237	19516.78	212.1862	19522.78
19530.75	212.4383	19533.83	212.4309	19517.34	212.4316	19519.76	212.4267	19520.86	212.8843	19518.57	212.4325	19524.77
19530.04	212.6853	19533.6	212.6867	19518.36	212.6835	19519.66	212.6828	19520.48	213.1228	19521.08	212.6822	19523.65
19529.03	212.9354	19528.56	212.9303	19516.88	212.9346	19521.88	212.9275	19518.38	213.3801	19517.23	212.9354	19520.71
19526.66	213.2046	19532.39	213.1839	19516.52	213.1827	19521.36	213.1773	19518.38	213.6245	19521.42	213.1968	19520.83
19528.19	213.453	19532.26	213.4326	19517.91	213.4319	19517.7	213.4279	19516.85	213.8827	19519.13	213.4306	19524.29
19528.42	213.6895	19528.02	213.6831	19518.65	213.6852	19518.12	213.6847	19519.24	214.1256	19521.21	213.684	19521.52
19529.25	213.9377	19535.46	213.9326	19521.88	213.9364	19516.47	213.9293	19517.95	214.3801	19520.08	213.9339	19525.29
19527.95	214.1843	19530.37	214.1832	19519.65	214.1835	19520.39	214.1775	19520.43	214.6244	19521.01	214.1834	19518.45
19528.64	214.4359	19532.78	214.4324	19517.63	214.4334	19519.57	214.4275	19520.82	214.8818	19519.15	214.4308	19520.34
19532.77	214.6855	19532.33	214.7028	19521.84	214.6851	19518.95	214.684	19516.05	215.1253	19521.29	214.6826	19520.48
19527.27	214.9397	19529.77	214.9313	19516.26	214.9369	19523.32	214.9305	19521.43	215.3793	19521.3	214.9378	19524.11
19532.07	215.1854	19531.36	215.1852	19519.16	215.1822	19520.9	215.1794	19516.99	215.6241	19522.23	215.1841	19520.49
19530.12	215.436	19528.71	215.4307	19517.74	215.4314	19519.04	215.4274	19515.44	215.8829	19519.68	215.4337	19520.88
19527.65	215.6937	19529.8	215.6844	19517.72	215.6879	19519.39	215.6828	19519.92	216.1255	19517.32	215.6842	19522.93
19530.54	215.9392	19530.72	215.9324	19518.35	215.9382	19517.3	215.9324	19516.42	216.381	19520.68	215.9376	19518.6
19529.74	216.1841	19532.51	216.1842	19520.86	216.1854	19519.89	216.1802	19519.62	216.6242	19519.39	216.1872	19521.21
19527.93	216.4399	19530.67	216.4325	19518.15	216.433	19519.05	216.4304	19521.08	216.8828	19520.05	216.4324	19520.98
19529.65	216.6857	19533.75	216.6837	19518	216.6874	19519.41	216.6846	19515.69	217.1274	19522.21	216.684	19519.92
19525.71	216.9389	19531.21	216.9308	19515.88	216.9401	19519.06	216.9301	19520.07	217.3815	19519.58	216.9399	19524.07
19527.58	217.1871	19531.78	217.1849	19516.58	217.186	19521.29	217.1798	19517.49	217.6281	19518.38	217.1886	19520.49
19527.69	217.4378	19529.06	217.4376	19519.6	217.4313	19515.61	217.4272	19521.71	217.8834	19518.09	217.4335	19522.05
19527.67	217.686	19531.06	217.6853	19519.59	217.6858	19520.7	217.6859	19520.5	218.126	19521.67	217.6877	19522.27
19530.01	217.9394	19531.13	217.9322	19516.95	217.9387	19519.94	217.9316	19519.25	218.3838	19519.47	217.9364	19521.97
19527.55	218.1861	19529.05	218.1875	19519.72	218.1862	19518.18	218.1813	19516.81	218.6258	19520.95	218.1866	19519.2
19529.49	218.4396	19529.94	218.4318	19515.14	218.4344	19524.17	218.4294	19517.96	218.8847	19519.21	218.4343	19519.24
19527.19	218.6856	19530.39	218.687	19516.28	218.6875	19520.97	218.6896	19520.27	219.1257	19520.5	218.6849	19521.49
19527.29	218.9395	19532.66	218.9322	19516.37	218.939	19517.94	218.9323	19516.82	219.3814	19523.42	218.933	19523.72
19527.12	219.1886	19531.6	219.1855	19516.69	219.1863	19521.95	219.1809	19519.17	219.6273	19521.59	219.1891	19520.89
19529.98	219.4408	19529.99	219.4349	19519.09	219.4348	19518.55	219.4277	19517.2	219.8854	19518.5	219.4339	19523.09
19527.84	219.6859	19528.97	219.6849	19518.15	219.6928	19519.41	219.6857	19517.89	220.1285	19516.41	219.6888	19521.18
19529	219.9388	19529.84	219.9348	19518.81	219.9409	19518.55	219.9305	19521.23	220.3823	19518.76	219.9339	19517.39
19526.92	220.188	19534.36	220.1859	19517.08	220.1857	19515.55	220.1802	19520.55	220.6271	19518.39	220.1884	19519.2
19530.67	220.4401	19531.5	220.4395	19515.67	220.4334	19521.03	220.4301	19519.56	220.8855	19523.26	220.4357	19517.53
19528.06	220.6867	19530.29	220.6859	19517.7	220.688	19520.81	220.6881	19520.31	221.1262	19516.29	220.6865	19520.58
19528.71	220.9394	19530.71	220.9341	19516.31	220.9404	19518.49	220.9302	19521.39	221.3841	19518.64	220.9376	19521.46
19527.24	221.1886	19531.84	221.2019	19515.76	221.1881	19519.57	221.1808	19518.35	221.6321	19521.93	221.1881	19518.39
19527.18	221.4407	19532.93	221.4327	19517.15	221.435	19519.82	221.4282	19519.32	221.8864	19520.97	221.4348	19519.81
19527.58	221.6865	19530.61	221.6857	19519.83	221.6871	19517.17	221.6892	19519.54	222.1288	19521.68	221.6893	19521.39
19531.22	221.9409	19532.68	221.9336	19519.56	221.9399	19517.6	221.9306	19519.64	222.3834	19519.88	221.9377	19519.42
19530.62	222.1887	19532.43	222.1881	19518.5	222.1872	19520.54	222.1824	19520.13	222.6269	19518.42	222.19	19522.46
19529.95	222.4388	19530.24	222.4341	19520.26	222.4351	19517.5	222.4314	19519.07	222.8863	19519.03	222.436	19517.95
19529.21	222.6874	19529.46	222.6856	19517.3	222.6893	19520.26	222.6884	19519.8	223.1291	19520.08	222.6873	19521.26
19528.15	222.9403	19534.34	222.9371	19514.27	222.9422	19519.32	222.9337	19520.44	223.3889	19519.99	222.9371	19523.26
19530.61	223.1889	19528.38	223.187	19518.82	223.1895	19518.36	223.1817	19519.36	223.6265	19518.71	223.1895	19521.98
19530.22	223.4431	19530.6	223.4358	19519.02	223.4352	19520	223.4302	19520.37	223.8866	19520.28	223.436	19521.39
19528.44	223.6891	19528.54	223.6881	19514.38	223.693	19513.65	223.6903	19522.95	224.1309	19520.85	223.6869	19522.24
19528.34	223.9421	19530.1	223.9346	19518.8	223.9409	19519.85	223.9294	19519.45	224.3853	19517.88	223.9424	19520.73
19530.46	224.1885	19533.38	224.1889	19518.66	224.189	19517.95	224.1838	19519.56	224.6275	19517.7	224.19	19523.97
19527.82	224.4467	19530.49	224.4362	19516.84	224.4357	19518.39	224.4322	19517.09	224.8866	19519.58	224.4373	19519.67
19531.93	224.6901	19530.9	224.6872	19518.57	224.6923	19519.27	224.6912	19521.72	225.1295	19521.38	224.6866	19523.12
19530.17	224.9392	19533.39	224.936	19516.6	224.9396	19517.64	224.9309	19519.35	225.384	19519.78	224.9375	19519.25
19527.47	225.1914	19532.18	225.1878	19519.67	225.1887	19520.34	225.1825	19517.08	225.6297	19514.28	225.1912	19524.13
19527.99	225.4426	19529.17	225.4339	19515.97	225.4362	19518.41	225.4306	19517.03	225.8889	19519.06	225.4375	19520.15
19529.96	225.6894	19528.76	225.6887	19517.78	225.6921	19517.35	225.6905	19518.15	226.1309	19520.87	225.6888	19521.12
19530.29	225.9401	19527.06	225.9373	19519.41	225.9426	19516.39	225.9303	19519.7	226.3859	19521.65	225.9375	19520.65
19529.01	226.1892	19533.14	226.1906	19516.61	226.1871	19517.54	226.1815	19519.43	226.6301	19519.2	226.1915	19525.91

19529.53	226.4429	19531.17	226.4346	19512.23	226.4384	19519.9	226.432	19518.19	226.8873	19517.25	226.4382	19521.92
19530.09	226.6888	19531.95	226.6909	19518.55	226.692	19517.19	226.6901	19521.54	227.1297	19519.05	226.6889	19518.92
19530.82	226.9452	19532.05	226.9366	19513.48	226.944	19518.16	226.9318	19520.57	227.3867	19521.93	226.9384	19520.35
19529.49	227.1892	19533.13	227.1961	19517.73	227.1891	19519.62	227.1845	19520.06	227.6291	19520.93	227.1937	19521.41
19528.07	227.4443	19529.18	227.4365	19517.15	227.4362	19519.76	227.4323	19516.78	227.8853	19517.43	227.4389	19520.9
19531	227.6895	19532.27	227.6897	19516.85	227.6916	19517.05	227.6904	19517.81	228.1297	19521.26	227.6889	19523.59
19529.1	227.9427	19530.08	227.9379	19522.08	227.9423	19519.36	227.934	19518.82	228.3848	19520.19	227.9419	19524.09
19530.49	228.1912	19530.71	228.1917	19517.63	228.1913	19517.67	228.1837	19516.83	228.6305	19521.09	228.1913	19522.31
19528.75	228.4423	19529.44	228.4384	19518.43	228.4374	19520.68	228.4352	19518.15	228.8884	19520.31	228.4387	19520.53
19531.7	228.6908	19531.91	228.6885	19518.13	228.6934	19520.87	228.6903	19521.01	229.1314	19518.89	228.6904	19518.27
19526.97	228.9431	19530.8	228.9369	19517.98	228.9466	19516.04	228.935	19521.12	229.3867	19518.47	228.9415	19518.25
19527.09	229.1913	19528.13	229.191	19516.9	229.1895	19520.14	229.1852	19520.48	229.6311	19518.5	229.1914	19520.43
19528.97	229.4432	19529.86	229.4394	19521.18	229.4385	19517.38	229.4326	19521.18	229.8904	19520.02	229.4404	19521.9
19527.43	229.6899	19532.38	229.6903	19516.99	229.6913	19520.44	229.6911	19517.39	230.131	19520.6	229.6906	19519.1
19527.01	229.9436	19530.88	229.9377	19515.14	229.9432	19516.72	229.9332	19519.98	230.3855	19517.76	229.9423	19522.32
19526.2	230.1915	19528.55	230.1932	19518.27	230.1928	19519.64	230.1875	19517.77	230.6317	19518.65	230.2074	19524.12
19527.78	230.4439	19531.22	230.4388	19515.5	230.4389	19520.58	230.4363	19521.69	230.8945	19519.2	230.4433	19520.86
19526.28	230.6968	19530.35	230.6915	19517.82	230.6959	19518.25	230.6933	19519.89	231.1269	19518.26	230.6891	19521.39
19529.1	230.9454	19529.54	230.9401	19515.21	230.9422	19516.65	230.95	19515.42	231.3874	19517.64	230.941	19522.74
19529.14	231.1917	19531.7	231.1935	19516.04	231.1903	19518.03	231.189	19519.92	231.6337	19519.3	231.2048	19522.45
19526.71	231.4475	19531.03	231.4373	19517.39	231.4408	19518.99	231.4352	19519.24	231.889	19520.5	231.4401	19523.73
19527.86	231.696	19531.44	231.6926	19518.24	231.6984	19517.41	231.6931	19518.69	232.1336	19519.5	231.692	19521.34
19527.03	231.9484	19530.53	231.938	19516.69	231.9459	19519.14	231.9342	19516.92	232.3878	19520.51	231.9412	19521.3
19529.74	232.1872	19532.36	232.1943	19519.29	232.1905	19517.69	232.1868	19519.06	232.6329	19519.26	232.1954	19520.62
19530.67	232.445	19529.75	232.4383	19516.27	232.4385	19518.95	232.4345	19518.68	232.89	19517.98	232.4421	19524.67
19526.87	232.6899	19531.74	232.6913	19514.95	232.6927	19519.22	232.6927	19517.77	233.1328	19519.77	232.6921	19523.15
19528.34	232.9435	19534.09	232.9421	19516.81	232.9456	19521.18	232.9336	19517.76	233.388	19520.97	232.9594	19520.31
19526.61	233.196	19529.38	233.1965	19517.3	233.1907	19520.65	233.1878	19516.72	233.6317	19517.79	233.1987	19521.74
19529.77	233.4521	19529.25	233.4385	19515.91	233.4384	19517.5	233.4367	19517.96	233.8934	19516.2	233.4369	19519.97
19526.53	233.69	19530.99	233.6926	19518.76	233.6954	19523.71	233.6932	19518.16	234.1289	19519.21	233.6934	19519.22
19530.53	233.9441	19530.47	233.9394	19515.22	233.946	19517.97	233.9411	19520.41	234.3869	19518.43	233.9411	19520.12
19528.56	234.192	19531.57	234.195	19519.89	234.1923	19519.2	234.1873	19518.62	234.6335	19520.45	234.19	19520.03
19529.21	234.4457	19529.58	234.4399	19517.65	234.4438	19520.83	234.4345	19519.71	234.8948	19520.94	234.4408	19520.62
19524.37	234.6939	19531.18	234.6914	19516.46	234.6915	19519.1	234.6927	19520.54	235.1331	19521.16	234.6937	19524.24
19528.28	234.9447	19529.48	234.9388	19516.74	234.9454	19519.37	234.9345	19518.76	235.3873	19521.97	234.941	19521.45
19528.87	235.1906	19531.06	235.1962	19518.53	235.1922	19520.32	235.1881	19520.3	235.6333	19517.83	235.1954	19518.67
19526.57	235.4448	19529.74	235.4388	19515.51	235.4407	19519.35	235.4343	19520.33	235.8911	19522.49	235.4452	19521.58
19526.28	235.6928	19532.34	235.6939	19515.92	235.6943	19520.96	235.6956	19518.97	236.134	19523.49	235.692	19522.61
19528.48	235.9463	19529.71	235.9425	19514.82	235.9463	19518.23	235.9391	19518.37	236.3874	19520.42	235.9405	19521.09
19523.92	236.1934	19532.05	236.1942	19515.71	236.1934	19519.89	236.1911	19521	236.6332	19518.88	236.1966	19523.06
19528.93	236.4457	19529.98	236.4403	19518.25	236.4421	19518.59	236.4374	19517.91	236.8926	19521.4	236.4418	19519.5
19528.72	236.6913	19532.62	236.6934	19518.39	236.6951	19515.16	236.6972	19518.1	237.1284	19523.75	236.6943	19519.77
19527.2	236.9475	19530.33	236.9405	19515.15	236.9498	19517.89	236.9367	19516.81	237.389	19521.33	236.943	19519.55
19528.71	237.1984	19530.85	237.1954	19520.19	237.1874	19518.19	237.19	19518.4	237.6338	19516.63	237.198	19520.92
19528.56	237.4479	19533.23	237.4394	19516.93	237.4412	19516.89	237.4374	19517.94	237.8913	19519.29	237.438	19522.23
19530.76	237.6936	19531	237.6944	19516.77	237.694	19521.76	237.6975	19517.14	238.1342	19518.66	237.6968	19521.25
19528.59	237.9525	19527.58	237.9409	19515.28	237.9507	19519.28	237.9368	19514.76	238.3898	19520.6	237.9456	19521.99
19527.15	238.1942	19531.31	238.1953	19517.92	238.192	19519.71	238.1892	19518.33	238.6348	19519.31	238.1964	19520.28
19528.34	238.4498	19530.58	238.4408	19515.56	238.4411	19518	238.4394	19521.14	238.8932	19518.68	238.4419	19523.19
19528.05	238.6936	19532.21	238.6928	19517.84	238.6968	19519.39	238.6963	19519.62	239.1313	19518.17	238.6955	19517.61
19529.97	238.9483	19531.34	238.9413	19513.96	238.9499	19518.75	238.9366	19520.26	239.3905	19519.41	238.9417	19521.79
19529.01	239.1952	19529.8	239.1971	19519.92	239.1911	19517.77	239.1902	19518.08	239.6312	19517.85	239.1936	19520.26
19528.09	239.4502	19529.97	239.443	19513.66	239.4407	19516.36	239.4373	19520.19	239.8905	19518.76	239.443	19521.62
19527.34	239.6905	19528.91	239.6948	19515.75	239.6947	19518.88	239.6957	19517.67	240.1389	19520.6	239.6952	19522.07
19526.38	239.9472	19533.72	239.9436	19518.57	239.948	19521.06	239.9375	19517.92	240.3904	19517.92	239.9425	19521.37
19526.51	240.1952	19530.39	240.1964	19514.89	240.1931	19513.52	240.1914	19517.05	240.6308	19519.49	240.195	19520.23
19531.53	240.4456	19531.07	240.4428	19514.33	240.4428	19518.6	240.4393	19520.3	240.8923	19519.58	240.4442	19520.99
19525.41	240.6903	19530.84	240.6902	19514.72	240.7101	19516.93	240.6945	19519.21	241.133	19522.46	240.6915	19519.22
19527.82	240.9566	19531.51	240.9364	19515.26	240.9493	19518.26	240.9462	19518.98	241.3882	19519.8	240.9405	19522.08
19524.47	241.1911	19531.26	241.1956	19518.15	241.1912	19518.32	241.1931	19518.11	241.6353	19517.79	241.1925	19520.99
19528.84	241.4455	19531.21	241.438	19520.08	241.4402	19521.63	241.4384	19518.13	241.8925	19521.67	241.44	19523.65
19529.84	241.6938	19533.81	241.692	19516.6	241.6919	19520.8	241.6939	19516.81	242.1312	19518.99	241.6918	19523.35
19528.2	241.9481	19529.05	241.9389	19514.31	241.9441	19520.21	241.9397	19524.17	242.3867	19521.11	241.9395	19521.76
19525.77	242.1938	19533.29	242.193	19518.57	242.1913	19518.15	242.1871	19516.9	242.6339	19521.33	242.194	19520.52
19524.11	242.4484	19530.37	242.4397	19518.84	242.4391	19518.08	242.4328	19520.35	242.8893	19519.12	242.4402	19521.79
19529.16	242.6909	19529.25	242.6919	19517.88	242.6938	19517.92	242.7096	19517.16	243.1427	19523.22	242.6919	19520.11
19527.43	242.9476	19529.97	242.9394	19517.25	242.9467	19519.24	242.9398	19515.14	243.3892	19518.65	242.9394	19522.4
19523.3	243.1924	19534.51	243.1926	19517.57	243.193	19524.84	243.188	19520.57	243.6322	19517.34	243.1939	19520.33
19527.51	243.4475	19534.26	243.44	19514.01	243.4394	19519.22	243.4351	19519.18	243.8902	19519.84	243.4401	19517.87
19528.36	243.6929	19527.79	243.6933	19519.66	243.6926	19515.22	243.6955	19520.22	244.1332	19519.61	243.6951	19518.34

19526.79	243.9515	19530.39	243.9393	19515.06	243.9464	19518.1	243.9364	19519.73	244.3879	19519.19	243.9393	19522.39
19525.5	244.1936	19527.28	244.1923	19517.91	244.1942	19516.22	244.1884	19518.61	244.6332	19518.88	244.199	19520.47
19528.43	244.4473	19532.17	244.4414	19515.99	244.4392	19517.17	244.435	19520.76	244.8899	19522.65	244.4399	19521.44
19527.18	244.6943	19530.78	244.6919	19514.58	244.6932	19517.45	244.695	19520.59	245.1332	19517.08	244.6945	19520.09
19527.66	244.9461	19532.29	244.9418	19518.2	244.9443	19517.4	244.9337	19518.26	245.3874	19517.41	244.9408	19518.92
19528.57	245.1921	19530.58	245.2062	19516.66	245.1934	19520.91	245.187	19519.76	245.6365	19522.16	245.2095	19518.39
19527.43	245.4473	19529.72	245.4427	19516.83	245.4416	19521.04	245.4352	19519.58	245.892	19523.77	245.4463	19520.79
19529.85	245.6935	19530.7	245.6917	19514.89	245.6959	19522.12	245.6939	19518.31	246.1341	19522.78	245.7012	19521.1
19526.16	245.9568	19525.42	245.939	19519.45	245.9484	19520.14	245.9404	19515.26	246.387	19520.2	245.9469	19520.51
19530.75	246.1923	19530.83	246.1957	19516.43	246.1925	19519.85	246.1864	19519.13	246.635	19520.58	246.1951	19521.15
19528.01	246.4475	19531.6	246.4398	19517.54	246.4431	19521.94	246.4359	19518.04	246.893	19519.33	246.443	19521.32
19525.88	246.7	19533.07	246.6926	19515.01	246.6955	19516.69	246.6944	19516.54	247.1335	19521.07	246.6986	19522.25
19527.36	246.9478	19528.97	246.9404	19515.38	246.9493	19516.47	246.943	19519.88	247.3886	19518.35	246.9428	19518.89
19527.57	247.1926	19532.62	247.1947	19517.59	247.1923	19519.95	247.1874	19520.48	247.6339	19517.46	247.1976	19523.16
19530.13	247.4482	19530.3	247.4428	19516.15	247.4424	19517.51	247.4373	19523.26	247.8926	19519.03	247.4406	19522.54
19528.28	247.6952	19530.16	247.6943	19516.72	247.6956	19516.41	247.697	19517.24	248.1365	19519.75	247.696	19519.39
19528.5	247.9498	19529.73	247.94	19516.68	247.9485	19519.99	247.9376	19519	248.3896	19518.72	247.9417	19520.03
19526.36	248.1935	19527.33	248.1956	19516.58	248.193	19518.76	248.1873	19520.29	248.6367	19522.17	248.1953	19524.01
19529.01	248.4516	19529.28	248.443	19516.35	248.4423	19521.08	248.4383	19516.1	248.8926	19520.96	248.4434	19523.23
19529.19	248.6943	19531.32	248.696	19519.76	248.6935	19519.19	248.6982	19516.63	249.1349	19518.56	248.6941	19522.78
19528.53	248.9485	19530.79	248.9416	19519.62	248.9501	19517.49	248.9365	19518.75	249.3895	19518.8	248.941	19521.49
19526.53	249.1935	19528.91	249.1963	19517.89	249.1947	19518.73	249.1898	19521.09	249.6362	19521.14	249.1959	19520.7
19529.43	249.4542	19531.05	249.4428	19517.93	249.4419	19518.52	249.4374	19519.79	249.8949	19519.84	249.4437	19519.3
19528.99	249.6913	19530.97	249.6945	19519.81	249.6957	19521.1	249.6982	19520.72	250.1348	19522.17	249.6941	19523.07
19526.79	249.9489	19530.85	249.9509	19517.07	249.9492	19519.69	249.9491	19521.96	250.39	19519.18	249.9438	19521.19
19529.6	250.1951	19528.16	250.1969	19516.69	250.1955	19518.24	250.2002	19519.72	250.6348	19519.68	250.1968	19519.55
19529.7	250.4559	19531.66	250.4428	19517.89	250.4421	19517.06	250.4481	19516.28	250.8949	19519.17	250.4427	19520.88
19528.35	250.6915	19531.41	250.6947	19514.57	250.6975	19518.31	250.6973	19519.39	251.135	19522.9	250.695	19520.85
19526.97	250.9521	19528.75	250.9431	19517.91	250.9501	19519.51	250.9377	19520.02	251.3894	19518.1	250.9432	19520.75
19527.34	251.2003	19532.3	251.1967	19514.78	251.1952	19518.5	251.1891	19516.57	251.6383	19520.81	251.2201	19521.22
19526.3	251.4514	19533.25	251.443	19514.8	251.4438	19519.56	251.4394	19517.2	251.8949	19519.29	251.456	19522.19
19529.66	251.6941	19527.75	251.6951	19516.92	251.6956	19519.74	251.6982	19516.62	252.137	19517.93	251.6948	19521.23
19530.83	251.9463	19528.62	251.9433	19513.33	251.9498	19516.91	251.9388	19518.18	252.389	19520.29	251.9455	19521.11
19525.94	252.1944	19529.92	252.1978	19515.54	252.1962	19519.64	252.1896	19516.87	252.6363	19521.11	252.2041	19521.7
19529.15	252.4499	19532.29	252.4419	19515.55	252.4439	19521.19	252.4385	19522.22	252.8953	19517.97	252.4406	19523.32
19531.09	252.6948	19529.84	252.6975	19517.55	252.699	19518.35	252.7007	19519.96	253.1358	19521.15	252.6992	19519.95
19525.73	252.9478	19530.53	252.9431	19520.21	252.9508	19521.08	252.94	19520.1	253.393	19517.59	252.9445	19521.78
19526.81	253.1944	19528.74	253.1983	19516.3	253.1941	19521.12	253.1913	19521.01	253.6353	19517.26	253.1982	19520.03
19528.48	253.4483	19529.85	253.4438	19516.23	253.4432	19515.22	253.4402	19517.64	253.896	19519.33	253.4435	19520.37
19527.25	253.696	19528.67	253.6958	19519.83	253.6969	19518.73	253.6972	19516.25	254.1348	19518.26	253.6977	19520.6
19527.2	253.9491	19528.12	253.9443	19516.06	253.9526	19518.71	253.938	19519.99	254.4055	19517.72	253.9435	19521.96
19528.51	254.1961	19529.78	254.1955	19516.91	254.1943	19518.29	254.1912	19520.98	254.6458	19519.57	254.1976	19520.02
19527.49	254.4471	19530.58	254.4452	19518.91	254.4453	19518.26	254.441	19518.37	254.8905	19519.63	254.4453	19522.53
19525.78	254.6944	19529.56	254.6972	19517.32	254.698	19518.4	254.6977	19517.78	255.1376	19518.31	254.6957	19519.08
19527.86	254.9494	19528.52	254.9457	19518.04	254.9499	19518.39	254.9434	19522.72	255.3922	19517.25	254.944	19522.5
19525.2	255.1956	19529.33	255.2091	19518.79	255.1976	19520.46	255.1927	19519.65	255.637	19520.57	255.203	19523.34
19527.34	255.4491	19529.99	255.4496	19514.87	255.4436	19520.5	255.4413	19518.07	255.8962	19520.57	255.4415	19521.97
19526.74	255.6951	19530.83	255.6994	19518.81	255.6978	19518.62	255.6979	19519.59	256.1336	19519.39	255.6953	19518.64
19527.37	255.9495	19531.05	255.9441	19519.51	255.9515	19518.17	255.9421	19516.17	256.3928	19518.93	255.9453	19518.61
19528.04	256.1948	19526.9	256.1989	19516.52	256.1917	19518.53	256.1966	19520.58	256.6375	19518.37	256.2002	19519.25
19525.18	256.4488	19527.5	256.4442	19518.3	256.4461	19518.5	256.4409	19517.75	256.8993	19518.74	256.46	19521.29
19526.39	256.7005	19532.21	256.6957	19515.77	256.7002	19516.35	256.6991	19518.45	257.1376	19521.23	256.7071	19519.77
19528.86	256.9494	19531.14	256.946	19520.27	256.9668	19515.52	256.9401	19518.11	257.392	19519.87	256.9459	19521.6
19525.78	257.1964	19528.56	257.2095	19516.2	257.2079	19516.17	257.192	19516.97	257.6401	19518.7	257.1974	19521.89
19525.12	257.4489	19531.06	257.4489	19516	257.4452	19519.9	257.4411	19519.03	257.8991	19519.85	257.4456	19520.76
19525.68	257.6993	19533.46	257.7003	19516.13	257.698	19517.75	257.7013	19518.14	258.1382	19519.9	257.6981	19522.78
19530.3	257.9502	19532.85	257.9454	19518.25	257.9543	19516.75	257.9525	19518.38	258.3927	19513.28	257.947	19517.87
19529.98	258.1978	19527.52	258.1985	19517.25	258.196	19521.06	258.1931	19515.1	258.6375	19520.26	258.1987	19520.07
19530.26	258.4536	19527.1	258.4476	19516.87	258.4464	19516.29	258.4413	19516.13	258.8995	19519.2	258.4453	19519.73
19527.78	258.6988	19533.11	258.6984	19514.27	258.6994	19519.69	258.6991	19520.31	259.1393	19517.42	258.698	19520.96
19526.81	258.9526	19530.59	258.9472	19517.22	258.9547	19517.08	258.9417	19517.23	259.3939	19517.62	258.9525	19523.35
19528.44	259.1987	19529.47	259.1987	19517.84	259.196	19521.29	259.1935	19520.06	259.6339	19519.25	259.1993	19521.5
19529.24	259.4713	19532.17	259.447	19516.61	259.4462	19517.99	259.4417	19518.22	259.8982	19523.19	259.447	19517.49
19529.05	259.7065	19529.28	259.6982	19516.65	259.6993	19521.04	259.7011	19520.16	260.1413	19521.19	259.6975	19521.25
19527.03	259.9529	19529.08	259.9462	19518.21	259.9532	19519.27	259.9426	19519.98	260.3929	19519.27	259.948	19521.2
19527.45	260.197	19533.91	260.2003	19517.8	260.1974	19518.96	260.1935	19518.99	260.6372	19517.85	260.196	19519.98
19527.26	260.4499	19530.58	260.4514	19521.14	260.4465	19517.95	260.4431	19516.14	260.9004	19520.03	260.4466	19522.01
19525.19	260.6983	19530.29	260.7042	19517.21	260.6992	19517.42	260.6996	19521.12	261.1382	19519.46	260.6958	19519.46
19529.57	260.9535	19529.35	260.9456	19519.12	260.9528	19522.41	260.9411	19518.9	261.393	19520.91	260.9466	19520.84
19527.97	261.1988	19532.11	261.202	19519.25	261.1977	19517.69	261.1957	19518.6	261.6432	19519.11	261.2031	19521.53

19529.24	261.452	19533.85	261.4425	19516.22	261.4466	19516.93	261.4425	19516.43	261.9029	19515.18	261.4458	19521.58
19527.06	261.6986	19530.23	261.6968	19517.14	261.6986	19516.18	261.7015	19518.03	262.1443	19522.01	261.6993	19519.74
19528.39	261.9513	19533.47	261.9468	19515.53	261.9528	19516.55	261.9419	19520.12	262.39	19519.47	261.9434	19519.26
19527.46	262.2024	19531.7	262.202	19519.33	262.1973	19519.5	262.1943	19516.99	262.6394	19520.51	262.2031	19522.8
19527.32	262.453	19530.78	262.4428	19518.54	262.4471	19516.62	262.4449	19518.1	262.9097	19517.29	262.4466	19519.95
19526.64	262.707	19530.41	262.6995	19516.97	262.7035	19519.25	262.7035	19523.26	263.1543	19518.17	262.6986	19518.14
19528.26	262.9625	19529.15	262.946	19518.16	262.9509	19519.64	262.9565	19517.28	263.4013	19519.87	262.9447	19522.06
19527.52	263.2079	19532.43	263.2033	19516.36	263.1967	19517.23	263.2001	19518.78	263.6441	19521.65	263.2019	19519.97
19525.94	263.4581	19529.31	263.4444	19511.17	263.4532	19518.22	263.4439	19516.43	263.8955	19518.52	263.4458	19519.08
19527.31	263.7026	19530.08	263.7028	19517.59	263.7017	19515.99	263.7035	19518.24	264.1381	19516.75	263.6996	19519.67
19526.76	263.9503	19529.52	263.9471	19516.34	263.9502	19519.06	263.9456	19519.14	264.3957	19519.61	263.9449	19518.66
19526.15	264.1988	19525.81	264.2039	19517.54	264.2009	19518.5	264.1949	19522.54	264.6376	19520.9	264.2077	19520.63
19528.71	264.4522	19530.37	264.4483	19512.39	264.4463	19519.36	264.4436	19518.92	264.8996	19521.59	264.4507	19520.88
19528.54	264.6962	19529.88	264.701	19516.51	264.7022	19518.87	264.6994	19522.31	265.1421	19519.6	264.6975	19519.73
19527.26	264.9562	19529.72	264.9496	19515.94	264.9513	19519.01	264.9421	19512.92	265.3951	19519.17	264.952	19519.8
19527.92	265.2004	19529.88	265.1978	19517.85	265.1967	19518.84	265.1969	19520.73	265.6407	19519.15	265.1997	19522.48
19528.19	265.4557	19528.53	265.4468	19516.09	265.4522	19517.08	265.4444	19519.16	265.9062	19519.38	265.4465	19523.82
19527.14	265.7012	19531.96	265.7027	19518.64	265.6969	19517.94	265.7034	19517.59	266.1493	19519.17	265.7002	19521.89
19528.32	265.9549	19533.04	265.9488	19517.8	265.9544	19519.32	265.9468	19519.36	266.3963	19516.8	265.9473	19522.95
19529.5	266.2018	19528.71	266.2082	19515.85	266.2052	19517.92	266.1962	19519.66	266.6432	19519.13	266.2041	19520.54
19529.98	266.4521	19527.36	266.451	19514.97	266.4506	19515.48	266.4446	19515.41	266.8971	19517.74	266.4499	19520.62
19528.36	266.6976	19531.3	266.6986	19515.4	266.6982	19515.49	266.7021	19520.51	267.1413	19520.17	266.7001	19522.34
19525.11	266.9548	19528.94	266.9477	19514.94	266.9562	19518.88	266.9463	19517.84	267.3967	19519.78	266.9499	19524.03
19527.32	267.1997	19527.7	267.2038	19515.54	267.2055	19521.21	267.1952	19515.93	267.6412	19519.88	267.2049	19518.8
19526.7	267.4546	19531.91	267.4476	19517.29	267.45	19516.92	267.4444	19518.08	267.9022	19522.78	267.4498	19521.19
19528.75	267.7013	19531.24	267.7017	19515.64	267.6983	19518.31	267.7029	19517.34	268.1513	19518.35	267.7009	19522.67
19525.48	267.9504	19530.23	267.9477	19513.83	267.9546	19518.66	267.9496	19520.54	268.3996	19518.86	267.9508	19521.28
19527.81	268.1958	19528.76	268.204	19516.51	268.2037	19517.67	268.196	19518.89	268.6441	19518.8	268.2086	19522.37
19529.51	268.4551	19529.26	268.4487	19517.88	268.4497	19517.27	268.4449	19516.69	268.9004	19518.62	268.4446	19523.29
19529.56	268.7059	19529.44	268.699	19520.11	268.6986	19516.08	268.7037	19517.73	269.1467	19518.11	268.7019	19521.41
19526.7	268.9537	19531.44	268.9505	19515.14	268.9524	19518.73	268.9524	19517.64	269.398	19518.3	268.9543	19518.79
19527.15	269.2028	19530.29	269.2063	19515.19	269.202	19518.45	269.2019	19518.21	269.6437	19516.3	269.2074	19516.66
19529.11	269.4549	19530.38	269.4531	19517.05	269.4503	19516.2	269.4488	19518.21	269.9021	19521.97	269.4523	19520.32
19529.43	269.7079	19530.68	269.7051	19518.52	269.6991	19517.96	269.7023	19513.97	270.1494	19518.26	269.7016	19520.69
19526.96	269.9602	19529.21	269.9532	19515.39	269.9547	19515.79	269.9495	19515.08	270.4022	19519.28	269.9534	19519.89
19530.33	270.2102	19528.73	270.2044	19517.18	270.2065	19516.09	270.1983	19516.87	270.6505	19516.78	270.2062	19520.45
19529.76	270.4611	19530.39	270.4521	19513.52	270.4525	19520.58	270.4454	19518.02	270.9065	19517.09	270.4511	19518.23
19528.36	270.7102	19531.86	270.7226	19516.11	270.7041	19519.92	270.7045	19520.15	271.1548	19520.95	270.7034	19520.63
19528.53	270.9739	19526.74	271.2101	19517.3	270.9558	19516.36	270.9503	19519.41	271.4072	19517	270.9522	19519.01
19530.09	271.4601	19531.75	271.4554	19520.22	271.2056	19519.68	271.1998	19520.05	271.655	19519.8	271.2049	19520.16
19527.14	271.708	19532.51	271.7091	19521.32	271.4545	19517.38	271.4515	19521.23	271.911	19521.82	271.4505	19521.72
19527.82	271.9601	19530.57	271.9577	19517.04	271.708	19517.51	271.7069	19520.71	272.1607	19517.35	271.7041	19522.65
19527.68	272.209	19529.8	272.2105	19516.54	271.9612	19518.67	271.9551	19517.33	272.4131	19521.89	271.9536	19521.65
19526.39	272.4602	19529.98	272.459	19521.2	272.2108	19515.3	272.2044	19518.86	272.6614	19519.5	272.2229	19519.97
19529.05	272.7089	19529.4	272.711	19519.1	272.4577	19519.95	272.4548	19517.88	272.9196	19519.42	272.7079	19521.44
19528.55	272.9606	19528.63	272.9593	19518.18	272.7142	19520.03	272.71	19518.55	273.4058	19519.06	272.9564	19521.4
19528.25	273.2104	19531.28	273.2157	19514.79	272.9706	19517.93	272.9606	19516.87	273.6549	19522.55	273.2127	19520.47
19527.84	273.4658	19530.84	273.4639	19517.55	273.2254	19518.86	273.2096	19515.14	273.9241	19521.06	273.4523	19519.82
19528.83	273.7153	19530.57	273.7157	19518.02	273.7114	19518.33	273.4627	19515.91	274.4108	19517.79	273.7076	19520.25
19526.47	273.9675	19530.38	273.9644	19517.16	273.9652	19517.3	273.7192	19518.49	274.6607	19518	273.9571	19519.52
19526.52	274.222	19530.89	274.2168	19514.74	274.2173	19519.22	273.9749	19519.27	274.9195	19523.87	274.2133	19520.11
19527.82	274.474	19528.99	274.4697	19516.03	274.468	19518.53	274.4524	19517.44	275.1698	19518.8	274.4552	19522.42
19528.26	274.7239	19531.87	274.7241	19515.85	274.7156	19518.21	274.7093	19515.78	275.4254	19514.53	274.7088	19518.83
19528.11	274.9803	19530.27	274.9758	19518.18	274.9731	19518.99	274.9605	19518.11	275.6757	19518.18	274.9571	19520.67
19527.82	275.4681	19530.28	275.4587	19516.04	275.2254	19515.63	275.2124	19519.74	275.9342	19514.03	275.2139	19520.6
19524.87	275.7191	19529.37	275.7109	19519.4	275.4754	19518.86	275.463	19521.72	276.4231	19516.98	275.4561	19519
19528.88	275.9747	19530.04	275.961	19516.27	275.7292	19514.6	275.7252	19517.58	276.6732	19518.81	275.7108	19517.95
19528.55	276.2248	19530.65	276.2171	19518.99	276.2162	19515.34	275.9735	19519.31	276.9308	19518.11	275.9554	19517.12
19524.47	276.4793	19526.09	276.4675	19514.02	276.4683	19519.16	276.2291	19513.98	277.1821	19519.85	276.2151	19517.98
19527.11	276.7309	19530.27	276.7196	19518.12	276.7211	19517.05	276.7164	19521.5	277.4401	19521.65	276.4592	19523.08
19528.13	276.9914	19529.79	276.9696	19515.49	276.9797	19518.98	276.9731	19515.74	277.9321	19521.73	276.7101	19520.17
19523.97	277.4799	19532.24	277.2282	19517.41	277.2344	19519.52	277.2283	19517.84	278.1817	19517.31	276.9574	19523.81
19526.58	277.7323	19528.61	277.4816	19516.01	277.7202	19519.75	277.4807	19517.72	278.4368	19517.43	277.2164	19518.06
19526.69	277.993	19529.67	277.9669	19518.81	277.9787	19515.05	277.7402	19519.22	278.6889	19519.18	277.4577	19518.64
19528.22	278.2452	19527.69	278.2243	19515.95	278.2351	19513.25	278.2224	19517.45	278.9487	19519.97	277.7097	19520.75
19526.61	278.7299	19531.53	278.4754	19519.03	278.4862	19516.88	278.4743	19516.9	279.4408	19516.77	277.9582	19518.76
19527.24	278.986	19530.08	278.7302	19514.59	278.7397	19517.42	278.7282	19515.75	279.6933	19515.88	278.2171	19519.18
19528.18	279.2453	19530.49	278.9828	19515.81	279.2268	19519.33	278.9728	19523.77	279.9547	19521.93	278.4682	19521.62
19526.82	279.5045	19527.94	279.2389	19520.53	279.4802	19518.41	279.223	19516.98	280.2094	19517.36	278.7255	19520.82
19530.62	279.9933	19528.55	279.7291	19519.2	279.7364	19519.42	279.4702	19516.63	280.6964	19515.9	279.2233	19519.2

19526.31	280.248	19529.12	279.9811	19519.29	279.996	19518.82	279.7267	19518.96	280.9552	19519.6	279.4782	19519.5
19528.07	280.5052	19529.9	280.2376	19513.98	280.4817	19519.69	279.9757	19518.13	281.2129	19518.73	279.735	19519.99
19526.4	280.7614	19529.16	280.4903	19515.7	280.7412	19520.25	280.2243	19519.67	281.7021	19521.22	280.2269	19523.04
19526.27	281.2489	19531.12	280.7449	19515.93	280.9986	19517.84	280.4719	19518.23	281.9635	19520.86	280.4809	19522.11
19528.46	281.5076	19529.55	281.2365	19516.99	281.2541	19517.02	280.7276	19518.32	282.2147	19519.78	280.7393	19521.96
19528.12	281.7665	19528.34	281.4907	19517.27	281.7405	19518.1	280.9712	19520.98	282.4737	19517.4	281.2332	19521.63
19530.86	282.2547	19527.73	281.7451	19515.56	282.0011	19519.83	281.2236	19520.36	282.9644	19519.12	281.4876	19521.34
19525.76	282.5143	19529.07	282.0011	19515.73	282.2558	19520.67	281.4725	19515.21	283.2182	19520.15	281.7454	19518.29
19527.7	282.7721	19530.52	282.4883	19513.91	282.5143	19518.28	281.728	19519.15	283.4733	19520.47	282.2408	19519.57
19528.43	283.26	19530.93	282.7447	19519.36	283.009	19518.9	281.9716	19519.94	283.7357	19519.29	282.497	19522.09
19526.88	283.515	19531.61	282.9998	19516.91	283.268	19516.33	282.2246	19517.9	284.2253	19518.7	282.7586	19522.07
19527.13	283.7735	19532.07	283.2549	19516.45	283.7546	19518.27	282.4718	19518.88	284.4841	19516.98	283.2531	19519.36
19526.27	284.0292	19529.48	283.7421	19516.57	284.016	19517.8	282.7285	19516.21	284.7425	19517.64	283.5092	19518.26
19529.6	284.5179	19529.37	283.9948	19515.61	284.2724	19522.21	282.9727	19517.87	285.2319	19518.59	283.768	19519.36
19524.77	284.7774	19531.63	284.2533	19516.64	284.7647	19519.18	283.2232	19518.34	285.4894	19519.15	284.2665	19522.47
19526.76	285.0365	19529.06	284.5101	19516.54	285.0276	19517.4	283.474	19517.03	285.7492	19521.8	284.5231	19521.3
19527.97	285.5247	19530.97	284.9981	19517.52	285.2825	19518.69	283.7272	19517.46	286.2381	19520.07	284.7836	19518.49
19527.35	285.7861	19531.07	285.2567	19516.79	285.7694	19516.71	283.9763	19516.84	286.4964	19521.6	285.2779	19520.79
19529.3	286.0492	19529.05	285.5157	19517.24	286.0318	19518.52	284.2236	19520.87	286.7572	19517.29	285.534	19519.85
19526.44	286.5396	19530.34	286.0045	19518.34	286.2875	19520.18	284.4732	19518.32	287.2464	19516.53	285.7947	19522.35
19526.1	286.7999	19528.19	286.2527	19516.43	286.7771	19517.97	284.7276	19519.9	287.5034	19518.14	286.2892	19521.2
19524.97	287.0584	19529.66	286.5063	19518.44	287.0372	19516.76	284.9739	19525.41	287.7667	19517.2	286.546	19520.94
19524.02	287.5484	19531.13	286.7527	19513.31	287.294	19518.9	285.2263	19520.03	288.2552	19518.12	286.8071	19522.12
19527.01	287.8096	19528.11	287.0051	19516.64	287.7836	19521.03	285.4748	19517.49	288.5132	19518.77	287.3045	19520.26
19528.07	288.0707	19530.51	287.2544	19518.96	288.0488	19519.35	285.7348	19519.53	288.7751	19517.42	287.5655	19519.58
19525.06	288.5582	19528.15	287.5037	19513.54	288.5424	19517.77	285.9797	19517.24	289.2645	19519.53	288.0596	19521.18
19528.25	288.8253	19531.95	287.7508	19515.94	288.8054	19517.84	286.2285	19518.77	289.5212	19516.62	288.3233	19522.36
19528.03	289.3266	19529.71	288.0073	19515.05	289.2949	19517.38	286.4741	19517.53	289.7862	19519.81	288.8167	19519.56
19529.53	289.586	19530.64	288.2575	19516.39	289.5582	19517.43	286.7286	19519.73	290.2766	19520.08	289.0839	19520.25
19526.62	290.077	19531.6	288.5054	19516.65	290.0582	19519.05	286.9757	19520.95	290.5365	19517.59	289.5741	19518.78
19529.05	290.3439	19529.63	288.7519	19515.42	290.3165	19519.95	287.225	19518.22	290.7992	19519.35	289.8355	19521.73
19529.63	290.8445	19530.92	289.0079	19522.58	290.8053	19517.91	287.4735	19518.77	291.2886	19517.86	290.0996	19521.56
19526.19	291.1064	19529.08	289.2588	19518.07	291.0708	19516.68	287.7298	19517.88	291.5478	19520.76	290.59	19520.17
19527.37	291.6046	19528.31	289.5082	19515.06	291.5671	19518.01	287.9785	19516.33	291.813	19518.51	290.8516	19519.73
19525.71	291.8775	19528.32	289.7557	19515.32	291.8254	19515.9	288.2284	19515.35	292.3058	19516.44	291.1161	19521.37
19527.65	292.3753	19530.59	290.003	19513.29	292.3187	19515.77	288.4782	19516.99	292.5532	19518.76	291.6076	19518.02
19529.86	292.6409	19531.87	290.2556	19516.84	292.571	19513.54	288.7319	19520	292.8062	19518.88	291.8711	19520.6
19525.62	293.1333	19528.46	290.506	19514.07	292.8194	19512.56	288.9802	19518.27	293.0553	19520.9	292.3683	19518.82
	293.3969	19530.78	290.7534	19515.19	293.0723	19515.75	289.2319	19514.98	293.3037	19518.24	292.6294	19518.2
	293.8992	19526.55	291.0027	19513.4	293.3214	19520.01	289.4811	19519.7	293.5546	19520.09	293.1267	19519.79
	294.1626	19528.45	291.2584	19516.14	293.5741	19518.13	289.7329	19520.37	293.8099	19518.76	293.3932	19521.76
	294.6538	19529.42	291.5067	19513.2	293.8215	19514.93	289.9815	19519.75	294.0581	19518.23	293.8873	19522.76
	294.9203	19529.42	291.7557	19518.61	294.0763	19520.96	290.2389	19520.1	294.3088	19518.51	294.1569	19517.47
	295.4148	19528.52	292.0058	19516.38	294.3254	19516.1	290.5014	19516.98	294.5587	19512.99	294.6475	19521.23
	295.6743	19526.17	292.257	19517.06	294.5842	19518.5	290.9896	19516.42	294.8143	19517.88	294.9117	19521.02
	296.1678	19528.9	292.5066	19516.56	295.0847	19520.8	291.2656	19520.24	295.0651	19518.1	295.4074	19519.87
	296.4317	19531.22	292.7556	19514.87	295.3346	19517.59	291.7564	19517.9	295.3181	19520.62	295.6697	19520.84
	296.9325	19531.88	293.0061	19515.57	295.5894	19517.7	292.0218	19519.99	295.8159	19519.66	296.1735	19519.28
	297.1963	19527.99	293.2588	19517.84	296.0886	19517.33	292.5176	19520.51	296.074	19521.46	296.6664	19522.18
	297.6926	19530.47	293.5086	19515.7	296.3373	19518.9	292.7839	19519.28	296.5675	19517.53	296.9323	19522.46
	297.9592	19533.16	293.7593	19516.73	296.5927	19518	293.2713	19520.78	296.8243	19515.14	297.4262	19519.41
	298.454	19529.85	294.0117	19516.55	297.0923	19515.77	293.5371	19515.36	297.0757	19519.3	297.6888	19515.99
	298.7161	19528.59	294.2633	19516.3	297.3427	19519.65	294.0259	19518.61	297.3283	19518.81	298.1898	19519.24
	299.2103	19528.72	294.5176	19518.76	297.5986	19517.92	294.2884	19516.93	297.8306	19518	298.4575	19516.53
	299.4762	19528.76	294.7693	19516.17	298.1061	19518.08	294.5553	19519	298.3316	19519.97	298.9513	19519.27
	299.9747	19528.92	295.266	19514.6	298.6072	19516.64	295.0464	19518.63	298.5829	19517.24	299.2253	19517.45
	300.2395	19529.84	295.5183	19517.23	298.8583	19514.17	295.3096	19514.18	298.8435	19519.54	299.7259	19521.32
			295.7697	19514.72	299.1141	19521.82	295.8035	19518.48	299.3401	19520.23	299.9902	19519.76
			296.2706	19516.01	299.6124	19516.8	296.0691	19516.47	299.5932	19519.42	300.4861	19519.27
			296.524	19517.27	299.864	19516.82	296.5627	19519.06	299.8543	19520.01		
			296.7736	19517.36	300.1249	19515.8	296.829	19521.25	300.3497	19519.84		
			297.0266	19515.04			297.3174	19515.73				
			297.5227	19515.35			297.5865	19516.5				
			297.7857	19517.54			298.0785	19517.18				
			298.2844	19517.08			298.3427	19519.68				
			298.5541	19515.74			298.8345	19516.08				
			299.0524	19517.95			299.1039	19517.9				
			299.318	19513.47			299.5977	19519.86				
			299.8117	19517.46			299.8671	19519.08				
			300.0817	19514.6			300.3563	19516.96				

1.43												
65												
x [1]		*x* [2]		*x* [3]		*x* [4]		*x* [5]		*x* [1]		*x*
t (s)	P (cm^{-1})	t (s)	P (cm^{-1})	t (s)	P (cm^{-1})	t (s)	P (cm^{-1})	t (s)	P (cm^{-1})	t (s)	P (cm^{-1})	t (s)
0	19531.58	0	19530.13	0	19527.81	0	19529.81	0	19533.8	0	19528	0
0.65517	19531	0.66422	19529.91	0.65905	19528.58	0.65529	19532.7	0.65757	19529.95	0.66024	19528.04	0.66135
0.857	19530.21	0.85238	19530.75	0.84842	19527.23	0.84879	19529.71	0.85682	19531.36	0.85405	19527.03	0.84869
1.1018	19533.47	1.10312	19532.67	1.09931	19531.28	1.09881	19527.35	1.10313	19529.8	1.09999	19527.03	1.09892
1.35426	19531.84	1.35266	19530.69	1.35128	19532.98	1.34979	19530.28	1.35257	19530.42	1.35271	19526.59	1.34954
1.60508	19529.91	1.60295	19527.59	1.59893	19528.87	1.59865	19527.02	1.60163	19534.03	1.6027	19524.55	1.59927
1.85505	19528.91	1.85007	19532.97	1.84533	19530.51	1.8483	19528.76	1.85293	19530.02	1.85108	19523.43	1.84544
2.10131	19529.54	2.10065	19531.96	2.09826	19529.26	2.09729	19532.93	2.1018	19528.07	2.10146	19529.74	2.09721
2.35459	19530.36	2.3513	19528.49	2.35136	19528.83	2.34942	19528.91	2.35333	19530.75	2.34929	19528.55	2.34696
2.60049	19528.86	2.60272	19532.68	2.59851	19528.59	2.59954	19527.98	2.60085	19530.06	2.60613	19525.42	2.59875
2.85507	19530.46	2.85145	19531.8	2.84526	19530.46	2.85352	19526.79	2.85283	19531.37	2.84992	19526.62	2.84702
3.10134	19531.5	3.09994	19526.52	3.09755	19530.95	3.09653	19527.21	3.10169	19527.69	3.09629	19529.57	3.09811
3.35458	19527.91	3.3525	19529.23	3.35122	19528.06	3.3462	19530.7	3.35735	19533.22	3.34856	19530.25	3.3464
3.60068	19527.93	3.60334	19525.63	3.59969	19529.99	3.59892	19529.61	3.60531	19527.7	3.6037	19526.55	3.60014
3.85478	19528.21	3.85235	19528.81	3.84513	19528.37	3.84679	19526.23	3.85381	19531.32	3.85146	19526.57	3.8467
4.10161	19532.96	4.1003	19532.63	4.0975	19531.17	4.09576	19525.85	4.10141	19534.07	4.09716	19527.06	4.10138
4.35906	19531.75	4.35194	19527.84	4.35121	19528.1	4.3468	19531.22	4.35398	19529.82	4.34891	19526.29	4.34645
4.60077	19532.89	4.60652	19531.54	4.60041	19530.16	4.59713	19529.7	4.60305	19529.9	4.60025	19527.19	4.59909
4.85638	19533.62	4.85643	19529.2	4.84607	19528.94	4.84792	19530.87	4.85733	19527.64	4.84926	19530.23	4.84838
5.10224	19530.66	5.1028	19529.64	5.09846	19529.37	5.10473	19528.92	5.10648	19530.29	5.09913	19525.17	5.10008
5.35459	19533.59	5.35204	19531.36	5.35256	19528.28	5.3491	19531.74	5.35353	19532.8	5.34964	19525.51	5.34711
5.60466	19530.09	5.60225	19529.41	5.60284	19530.89	5.60049	19528.99	5.60201	19531.54	5.60154	19528.42	5.60589
5.85621	19530.23	5.85122	19535.44	5.84823	19530.1	5.85086	19531.28	5.855	19532.25	5.85259	19525.1	5.85163
6.10152	19530.79	6.10221	19528.98	6.098	19530.93	6.09767	19530.23	6.10213	19527.18	6.09707	19530.19	6.09937
6.35594	19534.81	6.35268	19530.16	6.35176	19527.21	6.34833	19532.69	6.35361	19530.76	6.35117	19526.39	6.34765
6.60135	19528.68	6.60207	19531.72	6.60039	19530.51	6.59873	19531.84	6.60274	19530.95	6.60426	19527.28	6.60001
6.85651	19529.28	6.85173	19529.95	6.84836	19531.72	6.84824	19529.22	6.85686	19528.87	6.84984	19524.46	6.84825
7.10091	19531.97	7.10226	19529.21	7.0992	19526.4	7.10262	19530.02	7.10277	19528.85	7.09735	19524.1	7.09726
7.35535	19529.64	7.35382	19527.83	7.35157	19529.26	7.35177	19529.2	7.35476	19530.09	7.355	19524.06	7.34704
7.6026	19535.54	7.60364	19531.88	7.59932	19531.73	7.59794	19528.92	7.60205	19530	7.60556	19530.02	7.60018
7.85768	19529.5	7.85169	19530.57	7.84778	19529.4	7.85311	19531.45	7.85506	19529.32	7.84982	19529.4	7.84843
8.10111	19532.38	8.11064	19533.12	8.09949	19528.37	8.09667	19530.5	8.10406	19531.2	8.09852	19528.94	8.09801
8.35727	19531.08	8.35389	19530.9	8.35554	19528	8.34778	19529.73	8.35736	19528.75	8.35412	19526.38	8.34817
8.60247	19532.53	8.60349	19533.02	8.5997	19526.67	8.59759	19528.1	8.60264	19529.01	8.60267	19525.76	8.5996
8.8575	19531.24	8.85941	19531.98	8.84676	19530.99	8.84942	19531.12	8.85503	19532.25	8.85003	19526.28	8.84877
9.10227	19531.88	9.10198	19530.66	9.09912	19528.81	9.09786	19530.06	9.10381	19531.61	9.09859	19523.97	9.09967
9.35624	19526.22	9.35264	19534.15	9.35363	19529.29	9.34722	19530.34	9.35618	19529.35	9.35211	19528.31	9.34836
9.60157	19528.02	9.60441	19530.74	9.60135	19524.44	9.59878	19529.02	9.60296	19529.89	9.60311	19526.13	9.59995
9.85787	19530.76	9.85323	19528.75	9.84756	19528.71	9.84937	19529.21	9.85465	19528.36	9.85395	19529.48	9.84778
10.10291	19528.61	10.10292	19529.24	10.09925	19526.19	10.09748	19530.73	10.10304	19527.04	10.09979	19524.31	10.09951
10.35865	19534.49	10.35339	19528.5	10.35526	19531.1	10.34793	19530.19	10.36699	19529.85	10.3521	19526.87	10.34952
10.60349	19527.11	10.60553	19537.61	10.60193	19530.71	10.59874	19531.32	10.60483	19531.38	10.60535	19525.12	10.60236
10.85844	19529.54	10.85394	19528.61	10.84853	19529.18	10.84952	19531.84	10.85592	19532.45	10.8519	19529.47	10.84897
11.10394	19526.65	11.1051	19533.9	11.10027	19532.17	11.10096	19530.05	11.10353	19529.83	11.09922	19525.42	11.10151
11.3574	19530.3	11.35438	19530.29	11.35424	19530.63	11.34874	19531.44	11.35718	19532.62	11.35209	19525.59	11.34983
11.60432	19531.27	11.60662	19528.94	11.60185	19526.81	11.5987	19530.12	11.60502	19530.44	11.60597	19526.45	11.60406
11.85846	19531.73	11.85505	19533.27	11.84799	19529.94	11.85527	19530.17	11.85729	19530.24	11.85338	19527.21	11.85033
12.1076	19532.31	12.10524	19530.91	12.10019	19528.86	12.10408	19530.13	12.10323	19531.91	12.10091	19525.24	12.10265
12.35979	19531.89	12.35488	19526.29	12.35397	19528.28	12.34997	19528.79	12.3559	19531.04	12.35352	19524.02	12.34952
12.60385	19529.41	12.60502	19530.07	12.60229	19531.47	12.59895	19529.01	12.60377	19530.6	12.60492	19524.26	12.6059
12.85863	19529.19	12.85424	19529.38	12.84914	19530.72	12.84853	19529.79	12.85621	19533.35	12.85097	19523.69	12.85486
13.10338	19531.62	13.10467	19530.61	13.10133	19528.43	13.10003	19530.92	13.10506	19529.99	13.10015	19526.9	13.10215
13.35986	19533.79	13.35496	19531.91	13.35819	19526.27	13.36229	19528.71	13.35737	19528.31	13.35487	19525.11	13.34998
13.60424	19530.9	13.60606	19533.46	13.60213	19528.14	13.60082	19531.02	13.60416	19532.13	13.60248	19526.28	13.60252
13.86062	19532.14	13.85531	19530.44	13.84933	19533.22	13.85026	19529.36	13.85685	19533.84	13.85237	19529.88	13.84963
14.10368	19532.25	14.10466	19530.15	14.1022	19531.84	14.10055	19529.92	14.10594	19530.34	14.10357	19526.6	14.10287
14.35897	19534.16	14.35501	19529.19	14.35449	19531.74	14.35279	19528.8	14.35743	19532.6	14.35289	19523.66	14.35226
14.60504	19528.91	14.60703	19529.07	14.60223	19529.87	14.60254	19532.44	14.60639	19528.61	14.60263	19526.05	14.60279
14.86079	19528.96	14.85455	19535.07	14.84846	19529.56	14.84954	19533.51	14.85634	19530.63	14.85348	19526.97	14.84971
15.1045	19534.86	15.10442	19532.67	15.10251	19531.41	15.09974	19531.69	15.10433	19529.26	15.09994	19528.02	15.10677
15.36081	19532.6	15.35475	19529.08	15.35574	19530.26	15.35075	19529.81	15.35762	19529.32	15.35421	19528.21	15.35493
15.60448	19527.93	15.60817	19530.5	15.60353	19533.87	15.60182	19528.75	15.6053	19533.68	15.60499	19523.07	15.60417
15.861	19529.96	15.85707	19530.27	15.8498	19527.56	15.85359	19528.61	15.85755	19532.79	15.85384	19528.05	15.85109
16.10616	19532.86	16.10537	19532.64	16.10236	19530.96	16.10082	19531.35	16.10546	19531.1	16.10116	19525.26	16.10156

16.36298	19534.23	16.35477	19530.24	16.35637	19524.86	16.35151	19529.75	16.35952	19529.75	16.35387	19523.87	16.35169
16.6043	19530.74	16.60731	19532.2	16.60374	19525.87	16.60172	19527.72	16.60547	19533.71	16.60331	19528.05	16.60435
16.85964	19533.26	16.85631	19529.57	16.8494	19532.44	16.85126	19526.35	16.85857	19533.12	16.85399	19528.78	16.85103
17.10548	19532.15	17.10651	19535.43	17.10106	19528.21	17.10149	19530.18	17.10449	19534.6	17.10208	19526.73	17.10095
17.3615	19531.16	17.35538	19530.02	17.35609	19529.02	17.35001	19531.74	17.35669	19529.73	17.35399	19524.23	17.35086
17.60527	19533.06	17.60748	19524.75	17.60377	19529.92	17.60118	19530.84	17.60606	19532.65	17.60389	19522.68	17.60387
17.8596	19530.61	17.85618	19531.59	17.85116	19527.29	17.85218	19530.04	17.85962	19532.79	17.8589	19531.24	17.8515
18.10494	19528.5	18.10643	19530.24	18.10281	19531.23	18.10236	19528.87	18.10553	19529.76	18.10183	19527.65	18.1018
18.3606	19529.95	18.35577	19531.86	18.35536	19529.03	18.35077	19527.19	18.35752	19528.57	18.35521	19527.3	18.35132
18.60562	19531.66	18.60632	19532.32	18.60406	19528.18	18.60072	19526.56	18.60678	19529.17	18.60296	19525.17	18.60371
18.85963	19530.09	18.85626	19529.81	18.85195	19528.63	18.85098	19530.03	18.8599	19530.16	18.85854	19523.18	18.85295
19.10463	19529.13	19.1063	19527.27	19.10296	19527.88	19.10155	19532.62	19.10651	19528.95	19.10172	19529.53	19.10305
19.36766	19530.56	19.35676	19534.15	19.35581	19529.6	19.3525	19528.66	19.35697	19529.41	19.35444	19528.42	19.353
19.60715	19529.45	19.61037	19532.73	19.60354	19531.64	19.60306	19530.1	19.60663	19532.11	19.60671	19526.36	19.60377
19.86095	19529.1	19.85548	19530.6	19.85153	19527.99	19.85116	19525.96	19.85857	19530.32	19.85411	19527.97	19.85243
20.1057	19531.55	20.1062	19531.83	20.10331	19533.31	20.10198	19529.11	20.10793	19532.67	20.10367	19530.63	20.10344
20.36125	19528.61	20.3573	19532.48	20.35684	19531.51	20.35289	19527.82	20.35812	19530.13	20.35628	19528.81	20.35466
20.61219	19532.83	20.61096	19530.12	20.60491	19530.12	20.6042	19528.27	20.6067	19531.33	20.60688	19526.21	20.60595
20.86541	19534.31	20.85827	19530.42	20.85253	19529.42	20.85287	19531.23	20.85885	19532.56	20.85521	19531.29	20.85253
21.10777	19535.1	21.10641	19534.79	21.10559	19529.62	21.10297	19529.25	21.1078	19531.28	21.10259	19522.57	21.10398
21.3658	19532.34	21.35718	19529.73	21.35772	19528.18	21.35308	19532	21.3594	19531.21	21.35529	19526.87	21.35435
21.60611	19530.02	21.60937	19530.34	21.60559	19530.35	21.60408	19532.4	21.60731	19530.33	21.60524	19525.95	21.60679
21.8718	19532.62	21.85848	19529.66	21.85144	19526.98	21.85328	19530.41	21.85961	19528.48	21.8561	19527.71	21.85319
22.10867	19530.7	22.10824	19528.08	22.10453	19530.52	22.10406	19529.09	22.10714	19528.84	22.10407	19528.02	22.1053
22.36376	19530.31	22.35758	19528.25	22.37175	19529.37	22.35225	19525.42	22.36121	19532.23	22.35446	19524.04	22.35476
22.60651	19537.2	22.60994	19531.89	22.60639	19530.21	22.60514	19532.2	22.60907	19529.65	22.60579	19529.93	22.60672
22.86052	19531.89	22.85814	19532.41	22.85197	19528.01	22.85526	19527.18	22.85958	19527.92	22.86223	19529.08	22.85413
23.10799	19532.29	23.10919	19528.12	23.10521	19525.86	23.10228	19529.18	23.10681	19531.7	23.10487	19529.13	23.10806
23.3646	19531.54	23.35968	19528.89	23.35923	19529.08	23.3525	19528.57	23.35933	19532.16	23.35804	19529.51	23.35422
23.60757	19530.37	23.61086	19532.42	23.60666	19532.87	23.60499	19530.96	23.60977	19529.05	23.60694	19528.61	23.60718
23.86088	19535.03	23.86014	19529.85	23.85238	19529.6	23.85487	19529	23.86102	19534.26	23.86118	19528.74	23.85542
24.10798	19532.37	24.10847	19533.22	24.10431	19530.75	24.10459	19528.99	24.1075	19528.08	24.10367	19526.71	24.10801
24.36482	19529.52	24.35986	19526.39	24.35862	19528.98	24.35261	19529.42	24.36037	19532.14	24.35699	19524.19	24.35403
24.60738	19532.04	24.61064	19529.64	24.60668	19531.39	24.60262	19531.14	24.60889	19531.04	24.60607	19528.58	24.60625
24.86758	19529.84	24.85793	19531.33	24.85384	19531.34	24.85509	19526.17	24.86083	19530.4	24.85837	19526.46	24.85589
25.11112	19533.2	25.10812	19534.78	25.10428	19531.97	25.10393	19527.27	25.10763	19530.66	25.10579	19522.47	25.10495
25.36924	19528.18	25.35932	19531.26	25.35802	19526.14	25.35416	19527.9	25.36157	19532.29	25.35573	19523.06	25.35588
25.60975	19534.71	25.61137	19528.19	25.6065	19530.16	25.60425	19531.75	25.60821	19530.77	25.608	19524.32	25.60693
25.86304	19530.07	25.85878	19533.24	25.85345	19527.75	25.8532	19530.83	25.86269	19527.12	25.85732	19527.3	25.85345
26.10937	19529.69	26.1082	19533.01	26.10549	19527.95	26.10384	19528.45	26.10783	19529.57	26.10429	19526.5	26.10639
26.36552	19535.73	26.35872	19532.76	26.35808	19529	26.35504	19535.54	26.36049	19530.44	26.35614	19526.27	26.3559
26.60736	19533	26.61202	19534.35	26.60545	19528.8	26.60499	19529.57	26.60934	19528.35	26.60601	19527.37	26.60841
26.86308	19529.78	26.85904	19530.42	26.85333	19526.96	26.85495	19530.27	26.86144	19532.33	26.86735	19526.29	26.85368
27.10969	19531.09	27.10812	19527.95	27.10597	19530.1	27.104	19531.46	27.10881	19528.63	27.10565	19530.04	27.10466
27.36477	19529.37	27.35797	19528.78	27.35829	19529.03	27.35443	19528.36	27.36166	19532.17	27.35806	19529.39	27.35584
27.60779	19530.26	27.61101	19527.41	27.60603	19531.06	27.60482	19532.65	27.60877	19532.45	27.60688	19525.22	27.60892
27.86404	19530.85	27.85999	19531.42	27.85342	19530.57	27.85482	19531.67	27.86242	19528.4	27.85745	19525.22	27.85379
28.10964	19529.97	28.11048	19535.62	28.10614	19526.34	28.10437	19531.06	28.10978	19532.95	28.1044	19528.1	28.10466
28.36666	19532.99	28.35821	19529.32	28.35897	19531.91	28.35413	19529.49	28.36399	19531.06	28.35869	19525	28.3557
28.60831	19531.15	28.61069	19530.83	28.60726	19529	28.60475	19531.31	28.60957	19531.36	28.61016	19531.79	28.60858
28.86383	19529.23	28.86152	19533.01	28.85319	19530.92	28.85678	19533.96	28.86094	19531.22	28.85821	19524.84	28.85568
29.10977	19531.37	29.10963	19534.08	29.10616	19527.32	29.105	19529.67	29.10994	19531.36	29.10436	19527.6	29.10693
29.36554	19529.72	29.36047	19529.91	29.36083	19526.91	29.35386	19530.13	29.36334	19529.35	29.35762	19525.47	29.35434
29.60942	19534.81	29.61145	19528.42	29.60816	19532	29.60468	19527.98	29.61178	19530.01	29.60945	19525.59	29.6077
29.86484	19535.2	29.85866	19531.87	29.8544	19526.91	29.85561	19532.22	29.86549	19531.32	29.85876	19524.18	29.85606
30.10917	19529.64	30.10964	19532.93	30.10611	19529.3	30.10592	19530.21	30.10851	19530.63	30.10692	19528.61	30.10713
30.36763	19537.58	30.3623	19530.77	30.36028	19529.52	30.3566	19527.31	30.36091	19528.14	30.35807	19525.62	30.3567
30.61065	19533.33	30.61402	19530.1	30.61214	19531.06	30.60561	19530.25	30.61166	19531.27	30.60759	19531.68	30.60869
30.86544	19531.08	30.8601	19530.18	30.85696	19529.41	30.85598	19530.46	30.8628	19528.83	30.86019	19527.53	30.85671
31.11087	19532.14	31.11055	19531.26	31.10849	19526.23	31.10733	19530.29	31.10968	19531.42	31.10925	19524.8	31.10941
31.37169	19528.11	31.36169	19531.42	31.36171	19531.59	31.35729	19532.27	31.36086	19528.97	31.35871	19529.82	31.35751
31.61275	19526.69	31.6145	19533.27	31.6109	19532.34	31.60658	19528.92	31.61154	19531.6	31.6065	19525.05	31.60985
31.86686	19530.21	31.86107	19530.19	31.85644	19530.29	31.85613	19534.4	31.86486	19530.88	31.85954	19526.06	31.85824
32.11147	19528.4	32.11022	19526.41	32.10973	19531.99	32.10673	19530.01	32.11152	19530.35	32.1071	19529.96	32.10785
32.367	19533.46	32.36238	19532.64	32.36345	19529.19	32.35745	19531.42	32.36348	19532.4	32.36091	19523.45	32.3567
32.60916	19532.66	32.61538	19532.92	32.60936	19529.99	32.60671	19526.93	32.61058	19532.75	32.61014	19527.49	32.60851
32.86616	19536.18	32.86071	19531.16	32.85536	19527.57	32.85672	19530.54	32.86254	19529.61	32.85955	19525.96	32.85724
33.11237	19530.54	33.10922	19533.8	33.10898	19530.95	33.10533	19529.51	33.11081	19528.43	33.10632	19526.6	33.1095
33.36853	19529.73	33.36028	19531.9	33.35974	19526.52	33.35611	19528.71	33.3647	19529.55	33.3592	19529.03	33.35704
33.61422	19534.57	33.61474	19529.63	33.61095	19529.25	33.60671	19527.82	33.61012	19531.41	33.60977	19526.2	33.60916

33.86494	19529.1	33.8644	19531.19	33.85607	19527.77	33.85777	19530.37	33.86256	19531.05	33.85999	19527.59	33.85703
34.1115	19530.97	34.11	19530.56	34.10793	19529.84	34.10525	19528.19	34.11101	19528.53	34.10847	19526.74	34.10827
34.36825	19532.79	34.36058	19527.3	34.36149	19531.05	34.3562	19531.53	34.36652	19529.01	34.35961	19528.07	34.35879
34.60972	19531	34.61498	19530.01	34.61192	19528.43	34.60665	19531.23	34.61378	19531.65	34.60935	19523.91	34.61139
34.86546	19532.77	34.86264	19532.05	34.85546	19527.54	34.85682	19531.17	34.86316	19531.93	34.85993	19529.09	34.85624
35.11138	19533.13	35.11216	19526.57	35.1082	19530.48	35.10674	19530.29	35.11103	19528.26	35.10956	19528.31	35.10954
35.37233	19530.54	35.36084	19531.4	35.36265	19532.31	35.35586	19532.1	35.36464	19528.39	35.36196	19526.75	35.35744
35.61005	19532.68	35.61353	19528.17	35.61055	19530.34	35.60548	19531.34	35.61259	19530.9	35.61179	19523.58	35.60974
35.86606	19530.2	35.86355	19530.79	35.85534	19530.57	35.85784	19530.68	35.86344	19530.63	35.85919	19524.78	35.85757
36.11172	19528.98	36.11213	19530.21	36.1083	19527.21	36.10748	19531.57	36.11026	19529.77	36.10658	19527.62	36.10805
36.3688	19531.64	36.36181	19531.45	36.36438	19530.17	36.35778	19531.05	36.36475	19530.57	36.3608	19528.88	36.357
36.61184	19533.47	36.61484	19528.16	36.61244	19531.55	36.60658	19530.7	36.61671	19530.3	36.62022	19525.27	36.61107
36.86628	19532.77	36.86377	19531.19	36.85572	19527.9	36.85789	19532.36	36.87258	19532.38	36.86091	19524.17	36.85899
37.11063	19531.15	37.11837	19531.28	37.10845	19527.94	37.10958	19529.81	37.11534	19528.72	37.10855	19527	37.11094
37.37225	19531.1	37.36301	19529.32	37.36239	19533.41	37.35898	19528.74	37.3623	19531.36	37.36053	19522.8	37.35813
37.6171	19527.42	37.61603	19533.03	37.61249	19534.26	37.60728	19533.89	37.61245	19530.08	37.61043	19529.31	37.61205
37.86825	19536.9	37.86284	19530	37.85677	19530.49	37.86657	19531.51	37.86547	19532.92	37.8613	19528.43	37.85843
38.11171	19530.8	38.11289	19528.81	38.10824	19531.44	38.10803	19530.79	38.11926	19531.22	38.10885	19529.06	38.11188
38.36714	19535.03	38.36304	19532.24	38.36139	19525.73	38.3584	19531.11	38.36487	19529.61	38.36131	19524.94	38.35939
38.61204	19532.31	38.61621	19528.81	38.61088	19529.13	38.60786	19528.2	38.61264	19529.85	38.60793	19526.94	38.61066
38.8679	19526.43	38.86604	19533.45	38.85752	19530.1	38.85978	19530.06	38.8643	19531.36	38.86151	19529.12	38.85809
39.11292	19533.34	39.11233	19531.71	39.10978	19530.1	39.10738	19530.48	39.1137	19530.62	39.10893	19527.56	39.11162
39.36938	19528.46	39.36244	19530.97	39.36394	19526.58	39.35915	19531.86	39.36512	19533.74	39.3618	19524.39	39.3599
39.61212	19530.7	39.61693	19530.92	39.60995	19530.12	39.61117	19528.73	39.61246	19528.07	39.60923	19525.75	39.61281
39.86846	19527.51	39.86376	19529.41	39.8572	19529.48	39.85989	19531.15	39.86408	19528.71	39.8605	19528.11	39.85835
40.11348	19531.55	40.11246	19529.7	40.11079	19529.94	40.10801	19532.55	40.11257	19530.38	40.10959	19526.75	40.10916
40.37187	19530.43	40.36413	19533.67	40.36534	19531.64	40.36046	19528.9	40.36805	19531.78	40.3629	19530.82	40.35975
40.61294	19534.72	40.61932	19531.41	40.61309	19530.21	40.61075	19528.83	40.61552	19529.28	40.6129	19524.99	40.61388
40.86894	19530.91	40.86599	19529.18	40.85856	19529.46	40.85954	19528.04	40.86541	19531.72	40.86309	19524.51	40.86065
41.11527	19532.15	41.1156	19531.77	41.11154	19531.24	41.10913	19529	41.11404	19527.68	41.10905	19526.51	41.11147
41.37158	19526.81	41.36328	19528.7	41.36643	19528.87	41.36097	19531.3	41.3674	19533.15	41.36397	19529.16	41.35944
41.61352	19529.86	41.61745	19530.07	41.61368	19530.33	41.6113	19529.37	41.61554	19529.95	41.61365	19527.75	41.6122
41.86834	19532.49	41.86592	19531.76	41.85822	19525.44	41.86284	19531.2	41.8743	19530.73	41.86395	19525.2	41.86127
42.11436	19526.86	42.11621	19528.47	42.11103	19527.97	42.10991	19527.22	42.11379	19529.71	42.1098	19522.35	42.11253
42.37107	19532.06	42.36411	19529.17	42.36499	19531.2	42.36011	19529.22	42.36574	19529.5	42.36558	19525.65	42.36212
42.61513	19532.74	42.61852	19530.87	42.61637	19524.99	42.61089	19532.37	42.61482	19531.18	42.61304	19531.75	42.61431
42.87019	19530.29	42.86516	19531.13	42.85903	19528.94	42.86435	19527.83	42.86817	19527.94	42.86404	19528.38	42.86107
43.1138	19533.75	43.11551	19531.22	43.11182	19529.89	43.11025	19533.62	43.11563	19533.37	43.11172	19528.56	43.1136
43.37153	19531.04	43.36692	19530.67	43.36707	19529.96	43.36051	19531.59	43.36577	19531.98	43.36601	19526.88	43.36245
43.61523	19533.19	43.6194	19526.44	43.61481	19528.69	43.61043	19529.19	43.61546	19529.77	43.61116	19523.54	43.61447
43.8713	19531.05	43.86747	19529.99	43.86038	19531.28	43.86392	19529.12	43.86889	19528	43.87021	19527.84	43.86011
44.11454	19529.07	44.11603	19532.13	44.11336	19528.33	44.11202	19529.54	44.11616	19528.77	44.11108	19524.93	44.11448
44.37038	19530.06	44.36608	19533.48	44.36447	19529.3	44.36025	19530.24	44.36672	19527.86	44.36614	19527.96	44.36235
44.61569	19529.9	44.62124	19527.81	44.61403	19530.43	44.61018	19534.07	44.61504	19529.39	44.61443	19529.28	44.61863
44.87103	19527.47	44.86538	19532.62	44.86008	19529.06	44.86437	19532.54	44.8677	19530.8	44.8653	19524.67	44.86133
45.11588	19528.74	45.11598	19530.87	45.11318	19527.16	45.11283	19526.23	45.11698	19531.6	45.11253	19527.97	45.11153
45.37419	19534.02	45.36677	19529.42	45.36555	19527.79	45.3682	19531.19	45.36677	19532.7	45.36624	19526.66	45.3615
45.61558	19531.68	45.62129	19525.49	45.62816	19528.37	45.6111	19530.34	45.61455	19530.68	45.61356	19535.73	45.61554
45.86899	19530.5	45.86531	19531.16	45.86142	19530.52	45.87179	19527.09	45.86809	19531.26	45.86581	19527.12	45.8629
46.11571	19528.56	46.11458	19532.34	46.11474	19529.12	46.11308	19530.95	46.11648	19531.58	46.11184	19524.45	46.11235
46.37334	19535.77	46.36647	19533.21	46.3666	19528.06	46.36341	19528.89	46.3684	19529.98	46.36546	19525.95	46.36142
46.61633	19533.93	46.6221	19526.06	46.61609	19529.74	46.6126	19528.76	46.61505	19528.73	46.61348	19527.27	46.61625
46.87088	19533.64	46.86859	19531.86	46.85992	19532.55	46.86441	19527.22	46.86754	19534.15	46.86698	19528.35	46.86349
47.11545	19531.65	47.11622	19530.02	47.11425	19531.11	47.11252	19528.41	47.11671	19530.76	47.1131	19525.39	47.11479
47.37537	19528.9	47.37055	19531.19	47.36662	19526.44	47.36227	19530.65	47.36952	19530.22	47.36538	19522.81	47.37159
47.61646	19530.85	47.62247	19529.81	47.61626	19523.59	47.61366	19527.96	47.61524	19530.37	47.6124	19533.04	47.61496
47.86946	19531.34	47.8679	19529.38	47.86063	19527.85	47.86461	19531.56	47.868	19530.26	47.87429	19527.59	47.86214
48.11536	19532.1	48.11646	19528.13	48.11363	19526.95	48.11131	19533.22	48.11722	19528.31	48.11484	19529.06	48.1367
48.37585	19530.05	48.36588	19530.42	48.36426	19525.32	48.36262	19531.21	48.37008	19531.59	48.3672	19528.56	48.36449
48.61583	19528.63	48.6207	19533.25	48.61579	19531.9	48.61273	19529.13	48.61579	19530.17	48.61098	19528.23	48.61485
48.86887	19533.35	48.86895	19530.15	48.8605	19530.93	48.86482	19530.85	48.86754	19532.1	48.86473	19528.3	48.86254
49.11437	19531.84	49.11737	19529.69	49.11268	19529.81	49.1122	19529.23	49.11512	19531.3	49.11156	19531.3	49.11168
49.37712	19533.45	49.3657	19531.96	49.36349	19527.4	49.36099	19531.64	49.36879	19534.88	49.36626	19522.51	49.36169
49.61789	19530.94	49.62298	19532.11	49.61874	19527.1	49.61466	19531.76	49.61988	19530.32	49.61576	19529.37	49.61944
49.87224	19528.03	49.86791	19525.45	49.86161	19529.2	49.86541	19533.19	49.86957	19533.74	49.86614	19525.42	49.86312
50.11645	19527.87	50.11771	19531.01	50.11404	19528.7	50.11275	19529.6	50.11528	19527.72	50.11402	19528.95	50.11319
50.37603	19529.58	50.36609	19533.17	50.36515	19527.83	50.36154	19526.74	50.36717	19531.73	50.36553	19523.92	50.37342
50.61675	19532.47	50.62235	19530.69	50.61482	19527.1	50.61161	19532.1	50.61678	19528.37	50.61363	19525.91	50.61498
50.87185	19530.72	50.86621	19528.59	50.86072	19529.03	50.86697	19528	50.87028	19530.1	50.86728	19527.8	50.86327
51.1173	19531.27	51.11832	19529.04	51.1148	19527.22	51.11323	19532.06	51.11554	19533.16	51.11349	19527.69	51.11629

51.3763	19529.5	51.36767	19527.14	51.36416	19527.15	51.36404	19528.08	51.3668	19527.85	51.36631	19526.31	51.36356
51.61606	19534.03	51.62224	19534.12	51.61523	19528.91	51.61251	19529.21	51.6173	19530.56	51.61192	19525.94	51.61527
51.87277	19532.08	51.86766	19531.16	51.861	19528.2	51.86516	19531.34	51.86969	19529.21	51.87339	19521.85	51.86301
52.11722	19529	52.11794	19530.61	52.11476	19524.5	52.11462	19529.47	52.11634	19529.37	52.1172	19530.38	52.1152
52.37614	19528.4	52.36853	19532.54	52.36446	19529.87	52.36312	19529.45	52.36863	19532.82	52.3671	19528.45	52.36439
52.61616	19527.8	52.6234	19530.07	52.61742	19532.48	52.61443	19531.8	52.61772	19532.28	52.61382	19528.88	52.61465
52.86968	19531.42	52.86691	19528.81	52.86121	19531.7	52.86861	19529.87	52.8699	19531.42	52.8669	19531.17	52.86296
53.11659	19530.62	53.11816	19533.57	53.11492	19527.75	53.11136	19528.78	53.11748	19529.09	53.11353	19524.57	53.11594
53.38149	19529.34	53.36812	19529.04	53.36734	19530.64	53.36242	19530.56	53.37013	19533.48	53.36661	19524.53	53.3651
53.62111	19530.09	53.62253	19531.43	53.61648	19529.21	53.6141	19527.74	53.61718	19530.45	53.61455	19528	53.61771
53.87278	19532.3	53.86932	19528.85	53.8616	19527.68	53.86691	19527.8	53.87029	19530.86	53.86803	19520.44	53.86297
54.11656	19529.6	54.11854	19531.99	54.11409	19529.22	54.11419	19532.59	54.11919	19528.32	54.11446	19531.88	54.11442
54.37859	19531	54.36679	19528.19	54.36469	19528.49	54.36215	19530.05	54.37029	19531.59	54.3673	19526.23	54.36401
54.61919	19531.57	54.62132	19531.1	54.61684	19531.6	54.61361	19534.26	54.61682	19530.9	54.61307	19527.18	54.61641
54.87383	19533.84	54.86842	19532.11	54.86491	19528.84	54.8674	19527.44	54.86919	19528.45	54.87412	19527.64	54.86439
55.11812	19531.21	55.11969	19527.63	55.11644	19527.38	55.1139	19529.95	55.11867	19528.13	55.11653	19530.53	55.11412
55.37498	19530.78	55.36975	19528.65	55.36549	19531.55	55.36317	19530.5	55.37102	19531.61	55.3695	19525.24	55.36357
55.61764	19528.88	55.62203	19532.75	55.61747	19527.55	55.61215	19530.46	55.61906	19529.61	55.61466	19524.07	55.61848
55.87339	19531.41	55.86787	19531.95	55.86426	19529	55.86776	19530.9	55.87192	19532.37	55.86743	19524.38	55.86516
56.11847	19531.28	56.1186	19531.45	56.11645	19530.63	56.11539	19527.29	56.11764	19529.62	56.11442	19526.06	56.11552
56.37651	19530.63	56.36889	19528.59	56.36639	19526.47	56.36552	19531.08	56.37179	19530.56	56.37005	19525.78	56.36455
56.61782	19531.3	56.62658	19531.35	56.61896	19529.26	56.61545	19533.22	56.61896	19530	56.61909	19529.14	56.61858
56.8726	19531.95	56.86737	19530.32	56.86376	19530.43	56.86655	19527.94	56.87292	19532.24	56.86959	19525.92	56.8648
57.12001	19529.73	57.11879	19531.55	57.11737	19527.49	57.11655	19533.08	57.11755	19530.71	57.11466	19527.51	57.11546
57.37827	19530.31	57.36961	19531.1	57.36847	19527.41	57.36488	19528.21	57.36933	19531.27	57.3701	19529.12	57.36504
57.61734	19527.87	57.62464	19527.04	57.61892	19530.39	57.61425	19530.11	57.61933	19529.11	57.61513	19527.77	57.61708
57.87346	19528.8	57.86901	19529.95	57.86315	19526.24	57.86736	19531.19	57.87326	19527.32	57.86956	19527.67	57.86465
58.11861	19532.78	58.12066	19530	58.11754	19529.84	58.11485	19531.66	58.11928	19527.89	58.11685	19527.08	58.1169
58.37853	19529.95	58.3691	19532.94	58.36861	19528.94	58.36636	19528.78	58.36913	19528.4	58.37051	19527.78	58.36543
58.61915	19527.93	58.62509	19530.17	58.62208	19527.04	58.61655	19530.1	58.61896	19533.17	58.61474	19525.65	58.61776
58.87376	19531.18	58.8722	19528.75	58.8649	19530.69	58.86877	19531.04	58.87234	19533.61	58.86847	19530.01	58.86483
59.11833	19532.01	59.11965	19530.21	59.1176	19529.35	59.11524	19531.29	59.11936	19527.92	59.117	19528.49	59.1189
59.37944	19532.12	59.36925	19533.57	59.3687	19526.4	59.36638	19531.28	59.37052	19534.48	59.36974	19532.09	59.36812
59.62044	19528.61	59.62459	19526.26	59.62018	19527.39	59.61664	19530.76	59.61918	19530.67	59.61658	19523.91	59.61902
59.87455	19530.08	59.87013	19528.39	59.86294	19528.53	59.86908	19533.73	59.87159	19528.7	59.86845	19527.2	59.86508
60.11827	19529.17	60.12143	19530.92	60.1174	19529.29	60.11423	19530.12	60.12099	19530.63	60.11824	19529.4	60.11594
60.38499	19532.48	60.37044	19529.2	60.37029	19527.66	60.36681	19531.47	60.37505	19531.64	60.37215	19527.14	60.36755
60.62089	19530.58	60.62512	19530.86	60.62181	19530.44	60.61715	19532.79	60.61996	19529.91	60.61854	19529.81	60.62079
60.87727	19531.13	60.87177	19529.76	60.86614	19526.88	60.87181	19531.79	60.87272	19530.07	60.87187	19521.2	60.8682
61.12014	19529.6	61.12253	19528.8	61.11847	19531.01	61.11662	19527.87	61.12117	19534.18	61.11699	19530.19	61.11763
61.37863	19531.7	61.37212	19532.7	61.36743	19529.27	61.36708	19532.81	61.3752	19530.29	61.37145	19523.52	61.36757
61.62025	19529.05	61.64736	19526.94	61.62212	19526.66	61.61788	19533.1	61.62221	19527.95	61.61803	19528.69	61.62118
61.87741	19531.62	61.87213	19529.7	61.86763	19527.87	61.8722	19532.43	61.8733	19529.24	61.87146	19527.7	61.86914
62.12122	19531.4	62.12339	19530.59	62.12043	19530.98	62.12052	19530.83	62.12224	19531.73	62.11905	19525.75	62.12152
62.37907	19530.66	62.37262	19528.56	62.36542	19532.15	62.36722	19527.73	62.37587	19527.02	62.37155	19528.47	62.36853
62.6226	19534.14	62.62723	19528.74	62.62366	19531.96	62.61734	19529.08	62.62282	19530.39	62.61815	19527.03	62.62204
62.87575	19530.85	62.87221	19530.83	62.86688	19528.46	62.8708	19529.67	62.87475	19531.08	62.87205	19525.85	62.86896
63.12333	19535.35	63.123	19533.17	63.12108	19524.8	63.1177	19532.11	63.12122	19528.22	63.1193	19525.07	63.12215
63.38116	19530.16	63.37333	19529.24	63.37189	19526.45	63.36864	19530.53	63.37436	19529.73	63.37185	19525.44	63.36783
63.62082	19530.71	63.62816	19530.15	63.62181	19529.24	63.61629	19530.41	63.62377	19531.05	63.61994	19528.1	63.62174
63.87567	19530.2	63.8746	19529.21	63.86634	19529.21	63.8714	19527.06	63.87457	19531.4	63.87146	19526.1	63.86955
64.12309	19529.97	64.12376	19529.77	64.1225	19531.36	64.11859	19531.11	64.12219	19533.65	64.11848	19525.44	64.12007
64.38248	19531.73	64.37339	19532.16	64.37148	19527.88	64.36951	19530.53	64.37342	19533.65	64.37331	19528.31	64.37
64.62069	19533.94	64.62694	19532.02	64.62082	19529.66	64.61881	19530.98	64.62262	19529.65	64.61843	19526.1	64.62263
64.87536	19531.98	64.87077	19534.4	64.86557	19528.17	64.87032	19532.76	64.87382	19530.63	64.87137	19528.33	64.86833
65.12047	19533.5	65.12184	19535.42	65.11914	19530.82	65.11743	19529.43	65.11987	19533.25	65.12028	19526.82	65.11857
65.37996	19531.19	65.37157	19531.64	65.3685	19526.8	65.36716	19529.76	65.37436	19528.98	65.37162	19528.45	65.36963
65.62241	19532.64	65.6276	19530.96	65.62173	19530.47	65.61816	19529.95	65.62257	19532.14	65.61764	19526.22	65.62131
65.87906	19524.13	65.87234	19528.97	65.86507	19529.36	65.87378	19530.57	65.88103	19530.11	65.8722	19525.54	65.86956
66.12046	19529.29	66.12117	19532.54	66.11968	19526.94	66.11698	19526.67	66.12302	19529.63	66.11946	19524.54	66.11835
66.38149	19530.95	66.37386	19530.48	66.36826	19533.54	66.36716	19529.68	66.37235	19531.19	66.37202	19530.36	66.36838
66.62164	19530.42	66.62879	19530.24	66.62442	19528.36	66.61887	19532.39	66.62245	19529.41	66.61726	19528.4	66.6219
66.87804	19531.24	66.87305	19531.6	66.8685	19526.28	66.87188	19530.71	66.8732	19532.74	66.87176	19522.61	66.86997
67.12032	19531.03	67.12197	19531.54	67.12048	19528.66	67.11938	19532.85	67.12336	19530.16	67.11924	19529.96	67.1198
67.38098	19535.6	67.37138	19527.81	67.36613	19531.42	67.36801	19529.01	67.37607	19532.79	67.37229	19528.31	67.36941
67.62172	19530.13	67.62773	19532.95	67.62239	19531.97	67.61771	19527.24	67.62192	19532.55	67.61811	19528.4	67.62225
67.87876	19530.43	67.87305	19530.68	67.86807	19527.43	67.87683	19533.95	67.87383	19531.21	67.87266	19525.17	67.86917
68.12293	19530.9	68.12141	19527.52	68.12096	19530.62	68.1198	19527.48	68.12286	19528.64	68.1197	19529.15	68.11964
68.3829	19532.39	68.38071	19529.15	68.37014	19529.06	68.36756	19529.45	68.3768	19530.56	68.3736	19524.3	68.36988
68.62167	19533.6	68.62772	19529.69	68.6224	19531.35	68.61887	19527.79	68.62413	19531.02	68.61985	19525.2	68.6235

68.87776	19529.11	68.87556	19532.19	68.8667	19534.57	68.87507	19530.86	68.87393	19527.85	68.87347	19525.95	68.86846
69.12292	19532.78	69.12312	19530.81	69.12087	19534.37	69.1203	19529.43	69.12286	19532.24	69.11906	19525.82	69.11841
69.38253	19529.63	69.3721	19532.39	69.38247	19532.12	69.37029	19529.38	69.37707	19530.64	69.37446	19527.19	69.36958
69.6225	19532.33	69.62785	19525.21	69.63459	19533.49	69.61802	19528.98	69.62477	19532.44	69.62056	19529.58	69.62399
69.87618	19528.83	69.87344	19537.59	69.87327	19526.18	69.87363	19532.25	69.87509	19528.53	69.87576	19526.02	69.87054
70.12162	19529.05	70.12307	19531.35	70.129	19529.39	70.11974	19530.1	70.12362	19532.56	70.12233	19527.11	70.12168
70.38316	19531.76	70.37171	19529.54	70.37627	19531.14	70.37061	19534.11	70.37625	19534.42	70.37279	19525.21	70.36861
70.62313	19532.31	70.63043	19531.24	70.62759	19529.28	70.61893	19529.45	70.62443	19528.49	70.62178	19526.28	70.62397
70.88029	19530.95	70.87423	19531.82	70.87386	19531.66	70.8771	19529.45	70.87702	19528.85	70.87396	19526.43	70.87078
71.12219	19534.38	71.12513	19529.29	71.12728	19531.95	71.12277	19527.49	71.12386	19530.29	71.12198	19530.07	71.12315
71.38218	19526.2	71.37277	19530.19	71.37664	19527.79	71.37376	19531.1	71.37433	19529.38	71.37463	19526.03	71.37007
71.62496	19530.27	71.62816	19531.63	71.6269	19528.45	71.62103	19530.93	71.62365	19528.83	71.61929	19530.54	71.62297
71.87927	19534.99	71.87339	19527.22	71.87494	19533.69	71.87669	19533.05	71.87686	19532.46	71.87312	19522.6	71.86957
72.12498	19528	72.12515	19532.17	72.12733	19527.79	72.12067	19534.88	72.12619	19530.51	72.12342	19527.02	72.12508
72.38281	19529.53	72.37643	19531.23	72.37303	19529.06	72.36935	19531.48	72.37406	19529.13	72.37675	19528.23	72.37235
72.62364	19529.42	72.63018	19530.67	72.62637	19526.88	72.62106	19532.38	72.62328	19528.42	72.62109	19521.82	72.6249
72.87911	19525.95	72.87331	19531.55	72.87277	19530.9	72.89071	19528.93	72.87623	19527.83	72.87344	19526.17	72.86957
73.12385	19526.24	73.12407	19531.24	73.12622	19531.87	73.12057	19525.51	73.12516	19530.05	73.12153	19523.56	73.12128
73.38395	19530.1	73.37575	19527.84	73.40671	19528.26	73.3692	19530.12	73.37573	19534.06	73.37474	19525.6	73.37151
73.62421	19533.18	73.63227	19529.87	73.62901	19528.68	73.63524	19531.13	73.62338	19530.34	73.62167	19527.26	73.62611
73.8795	19529.91	73.87658	19530.38	73.87116	19530.17	73.87597	19532.08	73.87626	19527.8	73.87526	19524.8	73.86954
74.12462	19530.94	74.12519	19528.16	74.12472	19528.02	74.12398	19529.03	74.12737	19533.41	74.12114	19526.82	74.12102
74.38343	19531.95	74.37556	19529.85	74.37407	19529.93	74.36998	19526.4	74.37757	19531.69	74.37458	19529.21	74.37363
74.62442	19530.37	74.63184	19530.91	74.6297	19527.36	74.62009	19532.17	74.62464	19529.92	74.62141	19524.23	74.62413
74.87922	19529.47	74.87426	19529.41	74.86844	19529.09	74.87734	19526.67	74.8774	19532.31	74.87608	19528.43	74.87198
75.12467	19530.52	75.12425	19532.38	75.12724	19530	75.12176	19529.17	75.12621	19532.28	75.12673	19524.77	75.12257
75.38424	19529.66	75.3764	19525.39	75.37891	19531.29	75.37215	19528.31	75.37786	19532.06	75.37428	19527.34	75.37515
75.62641	19530.19	75.63127	19532.23	75.62884	19528.11	75.6206	19532.59	75.62496	19528.5	75.62125	19521.58	75.63254
75.88116	19528.29	75.87533	19529.95	75.8691	19526.03	75.87603	19528.29	75.87718	19531.93	75.87585	19524.04	75.87231
76.12317	19527.08	76.12473	19526.35	76.12333	19530.79	76.1215	19531.14	76.12484	19530.56	76.12366	19526.99	76.12536
76.38308	19532.44	76.37532	19528.32	76.37033	19531.5	76.37198	19526.16	76.37776	19527.08	76.37622	19523.28	76.37234
76.62707	19527.9	76.63211	19528.53	76.62732	19528.22	76.6229	19529.36	76.62514	19528.79	76.62035	19527.75	76.62538
76.90262	19531.57	76.87701	19532.52	76.87242	19531.84	76.87907	19529.98	76.87802	19529.86	76.87478	19530.56	76.87147
77.12432	19529.74	77.12506	19527.38	77.12413	19531.52	77.12338	19530.46	77.12577	19528.22	77.12351	19525.39	77.12313
77.38426	19526.46	77.37652	19531.32	77.37203	19527.01	77.37206	19528.92	77.37843	19530.52	77.37626	19530.32	77.3738
77.62759	19532.43	77.63231	19528.68	77.6261	19533.05	77.62374	19527.96	77.6256	19533.52	77.62234	19528.01	77.62406
77.88254	19534.47	77.87615	19528.97	77.87106	19531.24	77.87707	19531.6	77.87833	19532.92	77.87487	19527.91	77.87302
78.12533	19529.23	78.12466	19533.48	78.12519	19532.17	78.12083	19530.02	78.12631	19527.25	78.12253	19529.26	78.12678
78.38491	19528.6	78.37732	19528.88	78.371	19527.7	78.37193	19529.46	78.3887	19530.11	78.37684	19525.8	78.37445
78.62779	19533.16	78.63309	19530.34	78.62772	19530.03	78.62356	19529.32	78.62945	19532.69	78.62378	19526.35	78.62682
78.88198	19534.29	78.87937	19530.45	78.87148	19529.24	78.87955	19530.18	78.87846	19530.75	78.87823	19526.61	78.87156
79.12666	19528.54	79.13259	19532.21	79.12659	19527.92	79.12202	19529.61	79.12956	19531.79	79.12343	19527.11	79.12423
79.38847	19529.64	79.3778	19530.66	79.37546	19528.95	79.3732	19529.75	79.37969	19533.53	79.37662	19525.93	79.37423
79.62681	19529.92	79.63893	19533.1	79.62879	19531.73	79.6232	19533.89	79.62763	19529.28	79.62322	19527.43	79.62712
79.88168	19532.12	79.88203	19529.8	79.8716	19529.65	79.88109	19530.83	79.87861	19532.47	79.8776	19526.31	79.87409
80.12873	19532.96	80.12797	19530.1	80.12433	19531.9	80.12292	19529.01	80.12637	19533.45	80.12673	19529	80.12456
80.38839	19527.6	80.37892	19529.4	80.37473	19530.66	80.37436	19528.32	80.38122	19530.6	80.3825	19530.18	80.37617
80.62622	19530.32	80.63289	19531.04	80.63038	19531.14	80.6248	19528.67	80.62761	19529.73	80.62247	19527.85	80.62672
80.88151	19532.24	80.87699	19531.49	80.87136	19527.68	80.87895	19528.96	80.87983	19530.91	80.87739	19525.29	80.87438
81.12759	19530.43	81.12856	19523.33	81.12616	19530.55	81.124	19530.04	81.12638	19529.42	81.12419	19524.61	81.12751
81.38797	19528.11	81.37956	19526.09	81.37273	19528.52	81.3721	19530.66	81.37883	19530.2	81.37878	19531.81	81.37548
81.62772	19532.35	81.63345	19528.23	81.63095	19525.52	81.62131	19532.25	81.62614	19533.73	81.62546	19525.68	81.62764
81.88329	19529.22	81.87654	19534.14	81.8726	19526.28	82.76302	19528.07	81.87967	19531.6	81.87795	19527.13	81.87441
82.12737	19528.87	82.12726	19531.05	82.12501	19527.24	83.14902	19526.9	82.12768	19530.77	82.12561	19532.51	82.12781
82.38617	19530.24	82.3801	19528.69	82.37109	19528.32	83.39874	19532.66	82.37752	19528.88	82.37919	19524.15	82.37459
82.62774	19530.06	82.63435	19528.95	82.64594	19530.82	83.6494	19527.34	82.62622	19530.85	82.6264	19520.63	82.62843
82.88291	19533.32	82.87626	19528.48	82.87536	19529.47	83.90246	19527.42	82.87969	19530.56	82.87836	19524.76	82.87349
83.12622	19527.18	83.1282	19534.05	83.12818	19529.51	84.15	19527.87	83.12845	19530.06	83.12523	19526.05	83.13055
83.38667	19529.64	83.37965	19531.71	83.371	19528.75	84.40102	19529.04	83.37867	19529.54	83.37824	19526.22	83.37587
83.62807	19532.54	83.63627	19529.56	83.63079	19526.73	84.64974	19528.55	83.62744	19530.87	83.62608	19528.09	83.62697
83.88358	19526.85	83.88049	19530.02	83.87367	19526.93	84.90312	19527.42	83.87939	19531.97	83.87946	19531.71	83.87319
84.12751	19530.88	84.12769	19531.97	84.12887	19525.73	85.14921	19528.45	84.12899	19530.98	84.1254	19530.21	84.12611
84.38865	19530.22	84.37892	19531.74	84.37354	19532.16	85.40155	19528.51	84.38001	19526.49	84.37815	19526.41	84.37684
84.62753	19530.91	84.63618	19527.69	84.62978	19527.45	85.65114	19532.39	84.62728	19528.04	84.6243	19525.09	84.6317
84.88514	19529.59	84.87778	19530.06	84.87377	19527.91	85.90325	19530.11	84.89036	19528.93	84.87825	19519.79	84.8734
85.12814	19527.16	85.12909	19530.73	85.12795	19531.22	86.14895	19529.81	85.12798	19532.42	85.12881	19525.93	85.12973
85.38775	19527.79	85.37878	19532.56	85.37354	19527.09	86.39949	19526.3	85.38105	19533.13	85.38127	19525.33	85.37543
85.62781	19526.08	85.64023	19530.87	85.63201	19533.31	86.65126	19529.32	85.6293	19532.7	85.6256	19527.22	85.62847
85.88417	19530.17	85.88678	19527.74	85.8714	19529.28	86.9051	19532.83	85.88134	19528.8	85.87711	19522.71	85.87554
86.12878	19526.2	86.12858	19529.37	86.12569	19530.41	87.15334	19531.51	86.12691	19528.61	86.12412	19526.27	86.13045

86.38744	19533.04	86.37808	19529.29	86.37351	19525.88	87.40238	19529.32	86.37938	19530.24	86.37903	19525.84	86.37716
86.62724	19530.27	86.63739	19526.61	86.62986	19529.35	87.65097	19528.05	86.62951	19530.25	86.62579	19522.08	86.6293
86.88436	19530.44	86.88207	19530.11	86.87304	19531.47	87.90387	19527.48	86.88273	19528.46	86.87814	19525.11	86.87546
87.13012	19527.92	87.13065	19531.07	87.1277	19531.26	88.15127	19528.5	87.12974	19529.76	87.13638	19528.38	87.13076
87.39118	19531.66	87.38094	19529.77	87.37397	19529.07	88.40204	19530.66	87.37945	19528.39	87.38077	19527.8	87.37933
87.62892	19528.71	87.636	19526.06	87.6316	19528.11	88.65232	19529.63	87.6286	19532.01	87.62656	19525.34	87.62911
87.88607	19529.71	87.87984	19529.32	87.8753	19527.22	88.90487	19530.49	87.88283	19529.08	87.88011	19526.54	87.87482
88.12727	19529.82	88.13149	19528.42	88.13419	19530.72	89.15167	19526.19	88.12996	19528.64	88.12515	19526.78	88.13101
88.3876	19528.55	88.3807	19531.6	88.37224	19524.81	89.4027	19531.52	88.38169	19530.33	88.38	19524.45	88.38001
88.63023	19525.57	88.63689	19525.72	88.63104	19527.33	89.65179	19529.58	88.62968	19531.47	88.62788	19529.12	88.63041
88.8884	19529.69	88.88243	19529.32	88.87494	19528.81	89.90535	19529.28	88.88341	19530.58	88.87988	19525.83	88.87429
89.12968	19527.65	89.1299	19529.63	89.13017	19530.54	90.15282	19527.6	89.13146	19531.17	89.12691	19528.09	89.12952
89.38811	19530.37	89.38124	19530.32	89.37346	19523.3	90.40392	19530.09	89.38249	19529.4	89.37995	19528.77	89.37803
89.63004	19530.94	89.63641	19530.16	89.63208	19528.14	90.65209	19528.89	89.6295	19527.67	89.62606	19528.53	89.63115
89.88738	19531.51	89.88092	19527.54	89.87538	19528.14	90.90348	19528.21	89.89285	19529.96	89.88128	19529.08	89.87751
90.1301	19528.96	90.12964	19530.04	90.13004	19529.37	91.15169	19529.23	90.1307	19528.18	90.13066	19527.2	90.1315
90.39008	19530.45	90.38008	19526.93	90.37515	19531.2	91.40156	19528.21	90.38652	19526.99	90.38086	19525.43	90.38095
90.62926	19527.29	90.63836	19529.77	90.633	19530.99	91.65262	19530.1	90.63101	19527.68	90.62761	19526.91	90.63208
90.88546	19531.71	90.88192	19528.33	90.87578	19526.6	91.90916	19531.59	90.8906	19529.19	90.88125	19527.62	90.87784
91.13108	19527.88	91.12991	19525.02	91.13209	19524.49	92.15747	19527.08	91.12978	19527.06	91.12807	19527.04	91.13055
91.39021	19530.86	91.38191	19528.33	91.37607	19530.22	92.40168	19527.54	91.3826	19526.65	91.38102	19529.6	91.37894
91.62889	19530.6	91.63803	19529.32	91.63341	19528.07	92.6542	19525.81	91.63152	19530.09	91.62814	19528.04	91.63165
91.8855	19528.29	91.88172	19529.38	91.87515	19528.72	92.90792	19528.63	91.88578	19527.66	91.88617	19524.93	91.87936
92.1311	19525.21	92.13178	19530.79	92.12875	19526.66	93.155	19530.39	92.12999	19525.55	92.12666	19527.95	92.13187
92.39299	19528.87	92.38145	19526.87	92.37606	19528.66	93.40422	19527.57	92.38338	19528.49	92.3827	19522.64	92.37962
92.64037	19530.73	92.63815	19528.11	92.63404	19525.79	93.65547	19531.93	92.63137	19529.18	92.62781	19522.41	92.63088
92.88842	19525.34	92.88141	19525.58	92.87619	19524.09	93.90959	19527.68	92.88503	19528.37	92.89253	19523.8	92.87701
93.13256	19527.48	93.13428	19529.82	93.12921	19525.97	94.15371	19530.1	93.13144	19527.15	93.12925	19525.6	93.13164
93.39352	19529.42	93.38295	19529.78	93.37536	19528	94.40603	19529.38	93.3845	19529.45	93.38384	19528.22	93.38173
93.64426	19531.39	93.63841	19528.15	93.63354	19525.5	94.65389	19530.04	93.63134	19526.91	93.62855	19524.8	93.63411
93.89425	19530.35	93.885	19527.35	93.87703	19523.49	94.90991	19523.64	93.88593	19525.47	93.88073	19529.75	93.87696
94.12956	19524.51	94.13258	19526.05	94.13109	19527.06	95.1544	19529.66	94.13299	19527.8	94.12861	19522.57	94.13129
94.39074	19528.2	94.38354	19530.34	94.37506	19527.65	95.40152	19524.11	94.38546	19528.69	94.38304	19528.86	94.38135
94.63155	19529.22	94.63939	19524.92	94.634	19530.29	95.65254	19527.21	94.63512	19527.8	94.63161	19527.34	94.63289
94.88829	19525.87	94.88087	19527.86	94.87763	19528.57	95.90989	19527.52	94.88773	19531	94.88395	19522.9	94.87888
95.13243	19529.41	95.13143	19533.52	95.13188	19526.74	96.15609	19526.38	95.132	19531.26	95.13056	19521.11	95.13081
95.38999	19526.49	95.38336	19524.68	95.37624	19527.1	96.40492	19525.18	95.38391	19526.22	95.38513	19530.07	95.39088
95.63027	19526.8	95.64175	19526.03	95.63404	19525.22	96.65491	19525.32	95.63031	19528.1	95.62876	19522.36	95.63541
95.88842	19528.61	95.88398	19528.87	95.87737	19523.73	96.9073	19527.84	95.88772	19528.37	95.88242	19524.51	95.87953
96.13582	19528.2	96.13099	19530.78	96.13365	19526.27	97.15723	19530.08	96.13228	19529.34	96.12871	19522.41	96.13304
96.39236	19530.07	96.38109	19525.29	96.37816	19529.93	97.4047	19525.59	96.38828	19528.25	96.38419	19522.85	96.38322
96.6311	19524.01	96.64449	19527.04	96.63599	19527.7	97.65517	19525.31	96.63029	19528.9	96.62872	19525.45	96.63254
96.88838	19530.74	96.88551	19532.43	96.87693	19525.05	97.9121	19528.21	96.88543	19527.36	96.88493	19521.58	96.87922
97.13398	19528.88	97.13162	19527.92	97.133	19524.19	98.156	19527.65	97.13186	19524.75	97.13077	19527.55	97.13211
97.39405	19528.06	97.38255	19526.06	97.37756	19525.32	98.40481	19528.21	97.38784	19529.01	97.38678	19522.6	97.38233
97.63406	19529.99	97.64452	19529.08	97.63712	19527.81	98.65487	19526.28	97.6323	19525.56	97.62879	19524.02	97.63543
97.88948	19527.12	97.88575	19527.53	97.87677	19528.29	98.90887	19524.88	97.88692	19524.16	97.88467	19527.38	97.87819
98.13201	19529.43	98.13412	19526.11	98.13355	19523.54	99.1578	19525.67	98.13205	19531.22	98.13047	19528.58	98.13164
98.39332	19526.8	98.38255	19526.82	98.37725	19529.07	99.40748	19528.4	98.39772	19527.91	98.38553	19526.11	98.38065
98.63304	19530.08	98.64067	19521.83	98.63481	19526.68	99.65761	19526.94	98.63483	19525.35	98.6284	19523.77	98.63571
98.89165	19525.14	98.88844	19524.4	98.88201	19520.76	99.90892	19526.24	98.88728	19526.92	98.88314	19521.29	98.88063
99.13791	19527.9	99.13786	19523.19	99.14004	19529.74	100.1607	19527.76	99.13615	19524.78	99.13563	19524.97	99.13748
99.39419	19524.55	99.38354	19528.78	99.37827	19526.19	100.4094	19530.68	99.3886	19527.69	99.38523	19524.01	99.38484
99.63379	19527.62	99.64017	19530.97	99.6351	19526.73	100.6589	19528.28	99.63555	19525.05	99.63073	19523.45	99.63325
99.891	19530.95	99.88382	19528.73	99.87901	19523.92	100.9125	19522.22	99.88781	19529.55	99.88352	19524.07	99.88228
100.1332	19526.97	100.1347	19527.59	100.136	19525.34	101.1583	19526.28	100.1318	19525.33	100.1317	19525.43	100.1317
100.3979	19524.79	100.3871	19522.89	100.3813	19523.91	101.4076	19526.37	100.3858	19527.71	100.3885	19529.29	100.3897
100.6347	19527.28	100.6458	19524.38	100.6375	19526.19	101.6572	19525.79	100.6368	19527.72	100.633	19526.61	100.6361
100.8931	19531.75	100.8973	19531.21	100.8802	19522.74	101.9238	19525.15	100.891	19525.77	100.8863	19524.27	100.8824
101.1359	19529.04	101.1357	19527.91	101.1378	19524.65	102.1643	19527.29	101.136	19526.12	101.133	19523.21	101.1351
101.3966	19523.8	101.3876	19525.46	101.3825	19522.46	102.4095	19524.06	101.3893	19528.84	101.3885	19523.88	101.3871
101.6346	19522.92	101.6439	19526.51	101.6381	19526.84	102.6598	19525.46	101.6363	19524.09	101.6317	19525.99	101.6375
101.8915	19524.94	101.8897	19524.13	101.8803	19527.21	102.9145	19526.85	101.8894	19527.18	101.8859	19524.53	101.883
102.137	19524.67	102.1359	19529.42	102.1372	19527.94	103.1589	19523.25	102.1377	19525.77	102.1338	19517.19	102.1352
102.3971	19529.39	102.386	19525.94	102.3812	19523.43	103.4086	19521.87	102.3892	19528.32	102.3889	19527.11	102.3865
102.6374	19525.45	102.6454	19521.84	102.6607	19526.34	103.6575	19525.43	102.6356	19523.94	102.6328	19521.26	102.639
102.8917	19528.26	102.8894	19526.45	102.881	19523.5	103.9118	19525.82	102.8907	19526.82	102.8883	19523.67	102.8842
103.1356	19525.6	103.1373	19527.04	103.1357	19524.56	104.1602	19522.29	103.1368	19527.19	103.134	19524.07	103.1369
103.3989	19522.1	103.3862	19530.03	103.3829	19523.87	104.4083	19524.82	103.3895	19526.16	103.3878	19524.76	103.3892
103.6373	19527.66	103.6445	19522.35	103.6403	19526.24	104.6566	19524.6	103.6369	19523.32	103.6335	19526.13	103.6386

103.8932	19529.67	103.8925	19522.48	103.8829	19524.89	104.9106	19526.06	103.9018	19524.05	103.8871	19526.97	103.8846
104.1356	19523.88	104.1378	19525.98	104.138	19523.29	105.1575	19526.48	104.1385	19523.44	104.1329	19522.9	104.1516
104.3976	19529.64	104.3882	19527.22	104.3808	19525.2	105.4083	19525.72	104.3905	19524.46	104.3874	19522.67	104.3912
104.6367	19525.46	104.6457	19526.23	104.6394	19527.18	105.6587	19524.11	104.6383	19523.66	104.6347	19527.31	104.6392
104.8931	19524.68	104.8873	19523.25	104.8846	19522.6	105.9138	19525.45	104.8911	19528.56	104.8883	19522.99	104.8855
105.1365	19522.16	105.1379	19525.01	105.1379	19524.67	106.1573	19524.52	105.1356	19525.45	105.1358	19521.35	105.1376
105.397	19528.75	105.3874	19524	105.382	19526.87	106.4161	19526.82	105.3869	19524.97	105.3887	19526.62	105.3896
105.6371	19524.15	105.6446	19521.99	105.6386	19523.14	106.6587	19527.03	105.6385	19522.59	105.6351	19523.19	105.6401
105.8929	19523.78	105.8891	19528.49	105.8836	19521.88	106.9137	19523.83	105.8927	19527.53	105.8898	19514.41	105.8856
106.1376	19526.13	106.1366	19521.65	106.1388	19522.44	107.1627	19525.54	106.1369	19527.38	106.1352	19527.28	106.14
106.3984	19524.31	106.3873	19525.95	106.382	19527.75	107.4082	19523.33	106.3889	19522.87	106.3898	19524.06	106.3879
106.6375	19521.07	106.6463	19525.69	106.641	19522.84	107.6583	19524.75	106.6391	19523.9	106.6335	19525.17	106.6391
106.893	19523.9	106.8901	19525.47	106.8833	19525.39	107.9129	19524.21	106.8935	19523.25	106.8885	19524.24	106.8858
107.1376	19526.23	107.139	19526.87	107.1399	19524.58	108.1582	19525.35	107.14	19524.29	107.1356	19523.42	107.1414
107.3989	19527.66	107.3878	19523.21	107.3839	19524.37	108.4084	19524.04	107.3903	19524.61	107.3896	19522.36	107.3883
107.6363	19528.1	107.6464	19524.32	107.6413	19521.44	108.6607	19522.83	107.6377	19524.69	107.6351	19526.52	107.6403
107.8917	19524.03	107.891	19521.11	107.8835	19524.43	108.9158	19526.55	107.894	19525.81	107.8886	19526.03	107.8848
108.1397	19524.34	108.1398	19524.93	108.1391	19521.73	109.1601	19523.95	108.1386	19523.42	108.1345	19522.48	108.1409
108.3983	19523.01	108.3884	19520.51	108.3842	19527.36	109.408	19527.12	108.3896	19527.5	108.3898	19528.09	108.3917
108.6377	19524.13	108.6463	19524.29	108.6428	19520.72	109.6599	19526.73	108.6386	19525.34	108.6371	19523.55	108.6412
108.8933	19528.7	108.8954	19524.39	108.8835	19525.51	109.9141	19525.9	108.8928	19523.86	108.8908	19521.9	108.8853
109.1382	19525.66	109.1395	19525.53	109.1374	19520.33	110.1606	19524.06	109.14	19525.06	109.1343	19520.76	109.1397
109.4064	19528.26	109.3885	19523.29	109.3855	19522.94	110.4104	19526.28	109.3925	19525.46	109.3889	19522.77	109.3912
109.6392	19520.37	109.647	19527.71	109.6434	19526.06	110.6622	19528.59	109.6381	19523.65	109.6381	19521.64	109.6418
109.8937	19525.19	109.893	19525.25	109.8848	19523.28	110.9175	19520.52	109.8938	19522.96	109.901	19526.68	109.8867
110.1377	19526.07	110.1401	19523.39	110.1391	19524.62	111.1574	19520.81	110.1396	19521.14	110.1392	19525.33	110.1391
110.3991	19522.54	110.3876	19523.51	110.3835	19524.84	111.4093	19523.56	110.3928	19524.78	110.3895	19523.95	110.3933
110.6397	19522.11	110.6474	19524.25	110.6423	19523.57	111.6605	19523.79	110.6415	19521.74	110.635	19516.96	110.6424
110.8969	19526.91	110.8913	19526.1	110.8863	19520.42	111.9167	19524.75	110.8932	19524.74	110.8923	19523.41	110.8878
111.1385	19524.64	111.1406	19520.95	111.1402	19526.15	112.1656	19525.03	111.14	19526.05	111.1378	19523.63	111.1403
111.3999	19526.35	111.3914	19529.32	111.3839	19523.13	112.4112	19521.37	111.3918	19522.32	111.3912	19523.77	111.3971
111.6398	19522.48	111.646	19524.39	111.6486	19523.92	112.6605	19524.05	111.6419	19528.92	111.6374	19524.63	111.6418
111.8966	19521.52	111.8902	19521.59	111.8862	19525.65	112.9147	19523.03	111.8956	19522.03	111.8913	19524.94	111.888
112.1406	19525.37	112.1414	19524.88	112.1411	19520.93	113.164	19521.85	112.1387	19526.29	112.1363	19521.48	112.1415
112.4006	19523.65	112.3922	19523.47	112.3839	19522.54	113.4116	19524.17	112.3918	19523.44	112.3913	19529.02	112.3955
112.6387	19525.05	112.6505	19526.27	112.6433	19526.65	113.6629	19524.96	112.6425	19522.52	112.6385	19519.05	112.6417
112.8965	19521.64	112.8953	19525.52	112.8846	19525.78	113.9176	19522.97	112.9013	19522.76	112.8924	19523.45	112.8859
113.1429	19523.31	113.14	19523.89	113.1423	19520.44	114.1589	19523.86	113.141	19523.72	113.1376	19519.85	113.1422
113.4017	19520.93	113.3918	19525.34	113.3864	19525.03	114.4109	19521.99	113.3917	19527.4	113.3911	19524.54	113.3945
113.639	19520.03	113.6487	19526.95	113.6442	19524.32	114.6616	19521.76	113.6493	19520.42	113.6373	19527.58	113.6449
113.896	19524.53	113.8985	19522.46	113.8839	19526.66	114.9185	19522.11	113.9004	19527.22	113.8924	19525.12	113.8879
114.1436	19527.76	114.1407	19521.24	114.1414	19522.86	115.1629	19521.18	114.1446	19524.87	114.1399	19526.27	114.1413
114.4005	19525.02	114.3905	19524.47	114.3866	19523.01	115.4104	19523.31	114.391	19523.61	114.393	19529.2	114.3943
114.6399	19524.09	114.6495	19522.92	114.6453	19521.44	115.661	19522.06	114.6433	19524.65	114.6367	19522.71	114.6433
114.8989	19526	114.8959	19521.71	114.8867	19519.27	115.9156	19522.74	114.9052	19525.3	114.8921	19521.29	114.8895
115.1422	19527.78	115.1421	19524.66	115.1415	19521.57	116.1625	19522.82	115.1457	19520.35	115.1435	19519.03	115.1416
115.4047	19522.12	115.391	19523.05	115.3858	19520.86	116.4114	19521.94	115.3939	19525.05	115.3935	19523.72	115.3928
115.641	19526.81	115.6487	19527.7	115.6515	19522.13	116.6629	19525.24	115.6441	19522.76	115.6483	19519.08	115.6439
115.8963	19527.4	115.8939	19524.57	115.8885	19517.85	116.9198	19522.18	115.8988	19522.74	115.8929	19521.5	115.889
116.1415	19523.64	116.1432	19525.84	116.142	19521.93	117.1654	19521.36	116.1409	19522.22	116.1419	19520.9	116.1434
116.4017	19525.95	116.3922	19521	116.3864	19523.34	117.4118	19520.25	116.3948	19523.09	116.3933	19521.82	116.3972
116.6423	19528.64	116.6505	19524.85	116.6445	19522.69	117.6626	19521.2	116.6489	19523.1	116.6434	19524.46	116.6448
116.9	19527.12	116.8929	19517.62	116.8884	19520.11	117.9188	19524.46	116.9024	19526.39	116.892	19524.83	116.8883
117.1426	19521.81	117.1421	19520.96	117.1435	19524.81	118.1635	19526.04	117.1409	19522.49	117.1393	19522.28	117.1439
117.4036	19524.08	117.3924	19522.91	117.3877	19521.91	118.4129	19519.14	117.3938	19525.24	117.3935	19518.86	117.3927
117.6416	19521.57	117.6513	19522.51	117.6448	19521.56	118.6633	19522.91	117.6469	19520.96	117.6411	19523.08	117.6453
117.8999	19521.54	117.8934	19522.95	117.887	19522.36	118.9192	19521.07	117.8983	19524.36	117.8939	19518.49	117.8893
118.1445	19523.52	118.1427	19519.74	118.1435	19523.19	119.1635	19524.34	118.1435	19524.71	118.1374	19520.66	118.144
118.4063	19527.84	118.3936	19519.06	118.3904	19522.37	119.4144	19520	118.393	19520.98	118.3935	19524.52	118.3938
118.6414	19522.39	118.6513	19525.16	118.6462	19522.58	119.6652	19523.63	118.6423	19523.11	118.641	19522.03	118.6489
118.8968	19525.05	118.8977	19519.82	118.8874	19521.18	119.9204	19518.27	118.8985	19524.5	118.9069	19521.23	118.8908
119.145	19522.89	119.1426	19521.43	119.1437	19520.81	120.1668	19519.34	119.1431	19524.75	119.1403	19523.91	119.1447
119.4017	19519.94	119.3925	19520.22	119.3893	19520.26	120.4123	19525.32	119.3951	19523.05	119.3935	19527.84	119.3947
119.6412	19521.44	119.649	19524.76	119.6471	19521.76	120.6592	19520.84	119.6433	19523.88	119.6439	19522.87	119.6451
119.8978	19527.27	119.8981	19524.37	119.8881	19525.93	120.921	19520.33	119.9012	19522.44	119.8943	19525.4	119.8921
120.1452	19524.51	120.1433	19521	120.1424	19524.62	121.1641	19524.72	120.1442	19519.94	120.1446	19520.16	120.1433
120.4026	19521.37	120.3915	19524.92	120.3891	19523.97	121.4132	19520.6	120.3971	19519.17	120.3955	19527.81	120.3946
120.6387	19522.22	120.6469	19524.66	120.6438	19520.54	121.6605	19520.34	120.6401	19521.07	120.6378	19521.48	120.6445
120.8965	19526.88	120.894	19526.35	120.8888	19521.81	121.9174	19522.07	120.8982	19524.02	120.8927	19522.61	120.8893
121.143	19521.4	121.143	19518.7	121.1428	19519	122.1649	19521.95	121.1414	19523.41	121.1403	19521.81	121.1438

121.4022	19522.81	121.3926	19521.36	121.3865	19519.81	122.4113	19521.27	121.3966	19521.77	121.3943	19522.97	121.396
121.6411	19525.46	121.6513	19523.47	121.6451	19524.44	122.6627	19523.3	121.6427	19523.37	121.6392	19515.67	121.6482
121.896	19523.23	121.8935	19523.31	121.8883	19523.46	122.9209	19520.1	121.9194	19523.55	121.892	19519.81	121.8873
122.1418	19521.2	122.1424	19522.73	122.1419	19520.41	123.1605	19522.89	122.1415	19522.53	122.1372	19519.91	122.143
122.4017	19522.73	122.3926	19519.68	122.3882	19521.47	123.4114	19517.71	122.3931	19522.25	122.3938	19521.81	122.3953
122.6427	19521.11	122.6457	19522.94	122.6431	19520.69	123.6636	19520.91	122.6444	19524.68	122.6412	19519.19	122.6447
122.8989	19530.59	122.8929	19523.72	122.887	19520.39	123.9204	19524.47	122.902	19521.34	122.8937	19522.84	122.8891
123.1417	19518.99	123.1425	19522.39	123.1428	19523.33	124.1642	19519.9	123.1433	19521.13	123.1388	19523.68	123.1429
123.4	19521.94	123.3922	19522.26	123.3899	19520.38	124.4123	19520.65	123.3928	19524.14	123.3934	19519.71	123.394
123.6416	19525.88	123.6471	19524.79	123.644	19517.47	124.6623	19521.67	123.6425	19523.27	123.6404	19521.94	123.6434
123.8981	19522.69	123.8979	19523.85	123.8872	19520.7	124.92	19519.1	123.9007	19522.25	123.8935	19520.45	123.8898
124.1429	19524.25	124.1417	19522.86	124.1416	19520.19	125.1649	19523.8	124.1442	19523.78	124.1399	19525.63	124.1426
124.4008	19522.91	124.3926	19520.7	124.39	19520.89	125.4141	19521.88	124.3951	19521.06	124.3947	19524.63	124.3942
124.6555	19523.35	124.6493	19522.84	124.6447	19519.81	125.6637	19521	124.6417	19522.62	124.6387	19520.14	124.6441
124.8975	19524.26	124.8958	19520.8	124.8877	19522.49	125.9218	19523.46	124.9094	19521.09	124.8952	19520.84	124.8901
125.1427	19523.44	125.1416	19524.67	125.1429	19523.07	126.1644	19521.46	125.1474	19522.03	125.1448	19518.24	125.1451
125.4023	19528.91	125.3934	19525.61	125.3912	19519.89	126.4128	19521.67	125.3963	19521.51	125.3958	19521.64	125.3975
125.6447	19519.74	125.6503	19523.69	125.6478	19520.47	126.6646	19519.51	125.6449	19519.63	125.6421	19518.73	125.6467
125.8993	19523.67	125.8979	19521.59	125.8899	19517.34	126.939	19522.9	125.9008	19521.68	125.8955	19522.16	125.8893
126.1418	19524.81	126.146	19523.04	126.1419	19520.95	127.1695	19520.26	126.1443	19523.76	126.1402	19525.02	126.1439
126.404	19521.65	126.3904	19525.05	126.388	19520.97	127.4146	19522.2	126.396	19521.99	126.3939	19524.64	126.397
126.6448	19524.92	126.6491	19521.99	126.6468	19519.56	127.6618	19525.09	126.6447	19523.5	126.6411	19521.13	126.647
126.9009	19520.51	126.8969	19521.89	126.8893	19524.68	127.9218	19521.96	126.902	19524.05	126.8952	19521.74	126.8897
127.143	19520.55	127.144	19523.51	127.1439	19523.77	128.166	19519.05	127.1436	19522.24	127.1394	19517.58	127.1461
127.4031	19523.5	127.3921	19517.15	127.3904	19522.19	128.4143	19521.6	127.3965	19523.66	127.3954	19520.44	127.396
127.6442	19523.88	127.6511	19518.76	127.6475	19521.09	128.6641	19520.43	127.6453	19522.22	127.6416	19521.2	127.6485
127.9031	19519.57	127.8957	19520.72	127.8903	19518.96	128.9229	19517.92	127.9029	19518.59	127.9064	19520.69	127.8917
128.146	19522.75	128.1443	19520.8	128.1436	19518.59	129.1634	19520.05	128.1447	19526.93	128.1415	19523.37	128.1435
128.4031	19521.79	128.3942	19524.75	128.3913	19523.37	129.4143	19519.61	128.3965	19518.28	128.3949	19525.83	128.4047
128.6431	19522.08	128.6663	19524.45	128.6461	19515.22	129.6657	19520.36	128.6436	19521.9	128.6418	19517.3	128.6555
128.8996	19521.21	128.8979	19520.28	128.8906	19518.92	129.9287	19524.49	128.9002	19519.38	128.8978	19524.87	128.8932
129.1452	19518.81	129.146	19520.2	129.1449	19520.4	130.1722	19521.89	129.1453	19523.87	129.1424	19522.73	129.1461
129.4032	19522.62	129.3951	19519.21	129.3911	19521.34	130.4175	19519.05	129.3968	19522.43	129.3961	19521.19	129.3963
129.6449	19523.72	129.6504	19521.42	129.655	19520.12	130.6637	19523.64	129.6437	19522.45	129.6411	19514.91	129.6478
129.9004	19524.96	129.8959	19522.54	129.8897	19520.56	130.9219	19518.4	129.9018	19521.27	129.8978	19520.27	129.8907
130.1436	19519.55	130.1457	19518.59	130.1458	19516.23	131.169	19521.48	130.1461	19523.01	130.1446	19519.93	130.1482
130.4037	19523.46	130.3956	19519.44	130.391	19523.8	131.4159	19517.69	130.4007	19520.73	130.3979	19516.62	130.3987
130.6457	19520.26	130.6515	19524.7	130.6484	19520.06	131.6667	19519.96	130.645	19521.39	130.643	19521.72	130.6489
130.9027	19523.92	130.8939	19523.65	130.8911	19519.26	131.9237	19519.36	130.9031	19524.37	130.8983	19521.93	130.893
131.1438	19519.55	131.1466	19523.2	131.1455	19520	132.1688	19519.28	131.1458	19521.62	131.143	19518.55	131.1462
131.4053	19525.87	131.3956	19520.84	131.3889	19522.34	132.4149	19519.14	131.3974	19520.53	131.3966	19518.62	131.4003
131.6456	19520.69	131.6515	19516.59	131.6474	19521.28	132.6651	19522.88	131.6479	19520.85	131.6416	19520.78	131.6505
131.9126	19519.42	131.8972	19520.2	131.8905	19515.71	132.9232	19523.82	131.9059	19523.18	131.8973	19519.74	131.8935
132.1447	19526.32	132.144	19521.11	132.1442	19520.6	133.1669	19520.25	132.1448	19521.53	132.142	19515.74	132.1466
132.4059	19521.01	132.396	19525.55	132.3888	19520.42	133.4161	19519.93	132.3967	19521.65	132.3957	19520.93	132.3978
132.6475	19522.58	132.653	19518.09	132.6484	19523.82	133.6685	19519.6	132.6456	19521.68	132.6432	19522.32	132.6504
132.9007	19526.7	132.9007	19519.49	132.8916	19520.75	133.94	19520.12	132.9045	19523.84	132.8985	19522.22	132.8932
133.1479	19521.81	133.1462	19525.04	133.1468	19519.29	134.1668	19520.75	133.1488	19522.41	133.1424	19521.78	133.1483
133.4054	19523.08	133.3942	19520.39	133.3905	19520.27	134.4162	19518.81	133.4079	19520.17	133.3983	19523.63	133.3986
133.6437	19516.78	133.6515	19522.88	133.6502	19516.96	134.6657	19523.67	133.6548	19518.36	133.6434	19519.48	133.6492
133.9026	19527.61	133.9005	19520.24	133.8925	19519.75	134.9226	19519.11	133.9062	19522.12	133.8996	19523.75	133.8931
134.1462	19525.78	134.1477	19523.91	134.1468	19518.43	135.1707	19517.79	134.1478	19521.19	134.1449	19520.98	134.1489
134.4036	19520.55	134.3968	19520.85	134.3898	19515.43	135.4153	19521.32	134.3991	19519.2	134.3979	19523.57	134.4015
134.6465	19525.57	134.6522	19521.21	134.6503	19519.71	135.6655	19517.78	134.646	19520.82	134.6417	19518.61	134.6486
134.9028	19521.52	134.8976	19522.16	134.8926	19517.83	135.9252	19518.44	134.9064	19522.54	134.8996	19517.42	134.8934
135.1466	19522.45	135.1479	19517.47	135.1481	19518.16	136.1688	19523.25	135.1474	19521.12	135.1467	19525.38	135.1485
135.4044	19519.96	135.3991	19520.65	135.3901	19522.51	136.4187	19521.04	135.4013	19520.82	135.3997	19523.05	135.3993
135.6463	19522.96	135.6502	19518.38	135.6495	19518.13	136.6701	19517.13	135.6461	19517.29	135.643	19519.77	135.6689
135.9049	19524.45	135.8995	19524.31	135.8932	19520.62	136.9243	19517.89	135.9048	19520.5	135.9012	19521.23	135.9
136.1455	19525.47	136.1459	19517.97	136.1469	19521.11	137.1721	19521.65	136.148	19518.34	136.1433	19516.81	136.1492
136.4077	19521.95	136.3978	19521.49	136.3915	19518.77	137.4208	19516.44	136.3992	19520.65	136.4001	19519.06	136.3995
136.6465	19518.45	136.6524	19517.83	136.6501	19521.73	137.667	19518.9	136.648	19521.02	136.6452	19522.63	136.6492
136.902	19519.98	136.8958	19522.49	136.8936	19524.32	137.9231	19519.76	136.9058	19524.63	136.9013	19517.35	136.896
137.1494	19522.85	137.1467	19523.9	137.147	19515.85	138.17	19520.04	137.1464	19520.13	137.1432	19521.58	137.1476
137.4009	19526.28	137.3983	19519.39	137.3923	19517.86	138.4177	19520.23	137.3989	19521.4	137.3994	19518.46	137.4005
137.6472	19523.63	137.6522	19518.73	137.6522	19519.21	138.6683	19519.82	137.6477	19522.69	137.6448	19522.18	137.6493
137.9037	19523.21	137.8978	19521.6	137.8925	19521.46	138.9244	19518.89	137.9092	19520.41	137.9014	19525.65	137.895
138.1484	19523.47	138.1483	19522.33	138.1479	19514.09	139.1703	19519.61	138.1493	19521.26	138.1436	19523.56	138.1488
138.409	19519.69	138.3979	19519.74	138.3935	19522.93	139.4219	19520.72	138.4036	19521.06	138.3983	19521.47	138.4016
138.6461	19518.73	138.6536	19518.01	138.6515	19521.8	139.6688	19519.61	138.6499	19520.15	138.6449	19516.57	138.6507

138.9044	19520.67	138.9049	19522.22	138.8929	19522.85	139.926	19519.89	138.9078	19521.49	138.9011	19517	138.8958
139.1479	19521.96	139.1487	19519.68	139.1488	19518.7	140.1718	19520.72	139.1488	19520.01	139.1466	19518.15	139.1523
139.4078	19519.71	139.3967	19518.15	139.3941	19517.62	140.4172	19521.01	139.4001	19522.92	139.4002	19516.38	139.4031
139.6466	19523.86	139.656	19524.57	139.6517	19518.07	140.6682	19520.11	139.6483	19523.12	139.6469	19515.2	139.6506
139.9032	19522.94	139.9018	19525.52	139.8927	19522.21	140.929	19520.09	139.9077	19521.96	139.902	19521.39	139.8954
140.1482	19521.91	140.1511	19522.86	140.1476	19522.65	141.1719	19518.59	140.1496	19518.96	140.1493	19513.83	140.1511
140.4056	19523.52	140.4003	19522.71	140.3933	19520.59	141.4183	19517.84	140.4032	19522.31	140.4012	19523.37	140.4032
140.6489	19522.61	140.6536	19520.29	140.6525	19516.84	141.669	19517.53	140.6499	19522.17	140.6448	19521.17	140.6534
140.9043	19521.98	140.9023	19520.55	140.896	19519.77	141.927	19522.7	140.9126	19520.26	140.9012	19523.6	140.8964
141.1492	19522.84	141.1515	19520.8	141.1504	19517.56	142.1739	19518.97	141.1507	19517.31	141.1463	19520.6	141.1512
141.4085	19527.04	141.4021	19517.32	141.3935	19518.44	142.4201	19518.12	141.3995	19521.44	141.4041	19518.31	141.4023
141.65	19518.52	141.655	19519.41	141.6511	19516.71	142.6697	19519.55	141.6499	19520.46	141.6476	19520.61	141.6507
141.907	19517.65	141.9004	19521.92	141.8958	19518.86	142.9276	19517.91	141.9063	19524.72	141.9112	19520.98	141.8968
142.1468	19524.54	142.1524	19518.37	142.1477	19519.85	143.169	19520.39	142.1471	19521.76	142.1483	19518.42	142.1521
142.4102	19520.86	142.4021	19520.68	142.3955	19520.04	143.4179	19522.19	142.4028	19521.47	142.4007	19524.53	142.4053
142.6531	19522.03	142.6559	19523.77	142.6537	19521.37	143.6696	19519.25	142.6498	19518.18	142.6471	19520.4	142.6527
142.908	19520.87	142.9	19519.46	142.909	19518.12	143.9257	19519.71	142.9088	19520.18	142.9021	19520.25	142.8972
143.1508	19517.8	143.1513	19518.48	143.1521	19521.88	144.1711	19521.44	143.1495	19523.69	143.1473	19517.39	143.1542
143.4087	19522.5	143.4003	19523.91	143.3942	19518.21	144.4195	19519.76	143.4029	19520.9	143.4016	19520.75	143.4044
143.6516	19519.65	143.6529	19519.05	143.6521	19520.66	144.6705	19518.7	143.6501	19521.47	143.6455	19520.06	143.6526
143.9061	19524.01	143.9044	19518.76	143.8972	19516.72	144.9298	19520.73	143.9088	19521.8	143.9036	19520.32	143.897
144.1518	19522.39	144.1536	19520.44	144.1479	19518.99	145.172	19518.04	144.1514	19523.83	144.1469	19522.66	144.1532
144.4084	19520.47	144.4012	19518.39	144.3945	19519.86	145.4211	19519.94	144.403	19521.97	144.4027	19519.09	144.4036
144.649	19524.29	144.6563	19518.18	144.655	19518.72	145.6719	19521.95	144.6518	19520.5	144.6475	19520.14	144.6536
144.9046	19521.16	144.9022	19518.81	144.8973	19517.65	145.9281	19518.14	144.9086	19521.35	144.9027	19520.88	144.8985
145.1518	19521.66	145.1509	19521.31	145.1506	19520.58	146.1715	19521.85	145.1515	19518.48	145.15	19520.17	145.1508
145.4117	19521.07	145.399	19521.9	145.3947	19518.36	146.4198	19520.72	145.4008	19516.58	145.4008	19521.56	145.4039
145.6497	19524.31	145.6564	19521.91	145.654	19518.66	146.6777	19518.72	145.6517	19522.8	145.6503	19518.51	145.6531
145.9064	19520.39	145.9063	19519.77	145.8954	19519.07	146.9268	19521.43	145.908	19519.2	145.9039	19517.76	145.8996
146.151	19524.06	146.152	19520.41	146.1512	19520.53	147.1761	19518.6	146.1509	19519.91	146.1474	19518.3	146.1543
146.4092	19523.99	146.3998	19518.4	146.3953	19520.79	147.4206	19519.51	146.406	19522.02	146.4027	19519.42	146.4072
146.6503	19517.66	146.6545	19518.87	146.6557	19518.73	147.6699	19522.68	146.6503	19522.32	146.6489	19517.32	146.6536
146.9059	19518.96	146.9052	19516.05	146.8971	19519.12	147.928	19521.45	146.9085	19519.23	146.9025	19519.81	146.898
147.1505	19520.8	147.1536	19518.42	147.152	19520.25	148.1748	19518.7	147.1514	19519.68	147.1488	19520.94	147.1543
147.41	19521.51	147.4019	19524.14	147.3963	19518.6	148.4219	19519.71	147.4053	19520.51	147.4171	19521.62	147.403
147.6527	19524.19	147.6534	19520.66	147.6554	19516.71	148.6726	19520.38	147.6522	19519.31	147.6489	19524.15	147.6559
147.9065	19519.11	147.9022	19526.55	147.8957	19518.86	148.9291	19519.84	147.9115	19519.62	147.9033	19516.3	147.9015
148.1505	19523.05	148.153	19519.19	148.1517	19519.94	149.1733	19517.25	148.1553	19523.09	148.151	19522.64	148.1522
148.4087	19519.88	148.4028	19518.42	148.3967	19517.29	149.4195	19520.01	148.4036	19519.93	148.4034	19518.76	148.4034
148.6517	19522.34	148.6571	19524.22	148.655	19518.75	149.6733	19517.56	148.6524	19521.35	148.6501	19522.28	148.6545
148.9085	19517.47	148.9046	19521	148.9035	19518.65	149.9288	19517.46	148.91	19519.58	148.9024	19521.59	148.9022
149.1526	19527	149.1519	19518.15	149.1513	19518.61	150.178	19519.6	149.1522	19520.51	149.1502	19519.52	149.154
149.4072	19522.23	149.4025	19519.06	149.3965	19514.78	150.4261	19523.26	149.4057	19520.7	149.4055	19524.61	149.4079
149.6545	19518.68	149.6575	19518.44	149.6577	19520.5	150.6788	19519.1	149.6535	19520.09	149.6488	19519.28	149.6541
149.9092	19522.24	149.9048	19518.03	149.9	19519.37	150.933	19517.43	149.911	19519.11	149.9043	19515.33	149.9015
150.1527	19523.95	150.1523	19516.22	150.1517	19516.96	151.1789	19519.23	150.1526	19519.62	150.1526	19518.18	150.1572
150.4124	19523.12	150.4064	19519.93	150.4002	19518.93	151.4255	19519.65	150.4095	19519.97	150.4084	19517.37	150.41
150.6561	19520.84	150.6592	19518.8	150.6605	19514.86	151.6772	19520.17	150.6548	19521.1	150.6537	19525.61	150.6593
150.9102	19526.22	150.9062	19520.83	150.9035	19517.86	151.9328	19517.73	150.9114	19519.99	150.908	19516.62	150.9051
151.1569	19523.72	151.1564	19518.24	151.1573	19518.12	152.1822	19519.55	151.1595	19521.26	151.1528	19518.42	151.1599
151.4139	19528	151.4074	19518.63	151.3988	19514.09	152.4298	19518.63	151.4088	19520.41	151.4083	19520.35	151.4125
151.6574	19521.11	151.6602	19516.53	151.6589	19510.55	152.6746	19519.24	151.6575	19525.43	151.6537	19519.9	151.6593
151.9112	19523.09	151.909	19516.71	151.9011	19516.88	152.9331	19519.73	151.9126	19519.54	151.909	19519.27	151.9059
152.1576	19525.29	152.1564	19529.65	152.1581	19518.68	153.1814	19519.6	152.1578	19516.08	152.1525	19516	152.1588
152.4164	19517.5	152.4055	19516.41	152.4005	19518.54	153.4286	19518.58	152.4088	19519.79	152.4082	19519.57	152.4171
152.6607	19522.83	152.6592	19520.09	152.6595	19518.53	153.6769	19520.99	152.6573	19520.23	152.6538	19516.66	152.6661
152.9154	19521.81	152.9089	19516.99	152.9029	19513.09	153.9347	19519.61	152.9162	19521.17	152.9095	19520.55	152.9066
153.1566	19519.48	153.1588	19518.06	153.1583	19520.58	154.1804	19518.47	153.157	19524.69	153.154	19519.68	153.1591
153.4173	19524.7	153.4067	19513.29	153.4018	19517.39	154.4256	19517.22	153.4092	19521.1	153.4073	19517.75	153.4102
153.6576	19516.51	153.6603	19523.51	153.6623	19514.52	154.6804	19516.82	153.6584	19522.74	153.6539	19520.01	153.662
153.9203	19522.62	153.9102	19516	153.9014	19521.26	154.9357	19520.38	153.915	19521.82	153.9068	19519.36	153.9061
154.1624	19521.04	154.1578	19519.77	154.1554	19520.15	155.1796	19519.18	154.1584	19519.25	154.1561	19519.74	154.1594
154.4141	19523.14	154.4089	19521.45	154.4012	19515.79	155.4275	19516.62	154.4057	19517.06	154.414	19520.1	154.412
154.6613	19523.91	154.6662	19517.68	154.6605	19515.87	155.6779	19518.65	154.6572	19520.49	154.6531	19518.54	154.6588
154.9123	19521.3	154.9083	19520.61	154.9007	19520.38	155.9345	19523.04	154.9163	19520.98	154.91	19524.34	154.9065
155.1604	19521.91	155.1569	19518.28	155.1585	19519.48	156.1808	19514.62	155.1587	19518.57	155.1584	19518.83	155.1602
155.4161	19518.74	155.4081	19517.11	155.4012	19519.47	156.4291	19521.09	155.4102	19519.42	155.4096	19518.95	155.4146
155.6631	19521	155.6616	19522.67	155.6618	19518.01	156.6779	19515.32	155.6564	19521.18	155.655	19516.14	155.6617
155.9135	19526.05	155.9101	19515.74	155.9028	19518.49	156.9365	19518.45	155.9182	19520.28	155.9103	19517.99	155.9071
156.1596	19523.78	156.1565	19522.32	156.1588	19520.08	157.1835	19519.83	156.1591	19518.12	156.1537	19517.63	156.1617

156.4168	19518.89	156.4082	19517.03	156.4021	19518.28	157.4307	19517.84	156.4081	19520.09	156.4099	19511.67	156.4141
156.6581	19521.92	156.6623	19520.72	156.6625	19518	157.6791	19519.24	156.6556	19521.07	156.6551	19521.45	156.661
156.9122	19523.55	156.9088	19518.76	156.9031	19517.1	157.9368	19521.84	156.9174	19517.77	156.9096	19518.02	156.9057
157.1587	19523.63	157.1565	19521.65	157.1588	19521.35	158.1799	19518.52	157.1611	19519	157.1585	19517.29	157.1619
157.4141	19522.14	157.4104	19518.67	157.4007	19519.13	158.4278	19521.88	157.4101	19523.08	157.4091	19523.15	157.4113
157.6638	19521.58	157.6633	19520.39	157.6612	19517.47	158.679	19521.41	157.6581	19518.1	157.6557	19519.77	157.6633
157.9144	19520.44	157.9154	19511.88	157.9095	19518.44	158.9368	19519.11	157.9172	19521.37	157.9117	19518.55	157.9066
158.1562	19519.56	158.157	19522.04	158.1597	19518.38	159.1837	19516.18	158.1594	19519.34	158.1575	19513.18	158.1606
158.414	19523.44	158.4069	19520.87	158.4038	19519.36	159.4278	19522.95	158.4104	19514.87	158.4095	19515.72	158.4121
158.6597	19517.95	158.6642	19517.95	158.6611	19523.12	159.6784	19519.7	158.6742	19521.01	158.655	19520.9	158.662
158.9154	19517.05	158.9156	19518.73	158.9047	19518.23	159.9381	19520.65	158.9184	19519.8	158.9103	19518.71	158.9081
159.1598	19523.19	159.1591	19520.43	159.1601	19518.74	160.1799	19518.07	159.1602	19519.06	159.1616	19517.5	159.1599
159.4146	19519.72	159.4092	19523.42	159.4044	19519.4	160.4288	19516.95	159.4113	19520.72	159.413	19519.96	159.4151
159.6597	19522.58	159.6605	19520.51	159.663	19517.95	160.6775	19521.45	159.6587	19519.35	159.6585	19517.54	159.6605
159.9133	19521.15	159.9115	19519.65	159.9028	19520.47	160.9326	19518.82	159.9185	19518.41	159.9108	19524.56	159.9072
160.16	19518.12	160.16	19519.92	160.1576	19515.94	161.1781	19515.41	160.161	19519.28	160.1601	19517.92	160.1633
160.4173	19522.15	160.4159	19516.09	160.4043	19515.96	161.425	19520.18	160.4133	19519.44	160.4101	19517.26	160.4327
160.6582	19520.83	160.66	19519.95	160.6587	19518.67	161.6778	19519.45	160.6566	19519.6	160.6559	19518.02	160.6588
160.9141	19521.74	160.9083	19521.64	160.9012	19517.4	161.9339	19517.3	160.9176	19518.23	160.911	19515.52	160.904
161.1576	19524.76	161.1563	19517.14	161.1586	19518.48	162.1822	19519.09	161.1576	19515.18	161.1551	19522.32	161.1595
161.4131	19519.55	161.4099	19521.15	161.4062	19518.75	162.4265	19516.4	161.41	19520.53	161.4084	19518.08	161.4138
161.6603	19520.52	161.6577	19519.59	161.6719	19516.53	162.6802	19521.35	161.6601	19516.37	161.6559	19520.27	161.6599
161.9133	19522.01	161.9086	19521.08	161.9043	19519.85	162.939	19516.67	161.9169	19520.19	161.9112	19520.74	161.9034
162.1631	19517.99	162.1563	19522.17	162.1579	19516.6	163.1797	19515.03	162.1572	19519.47	162.1574	19517.98	162.1595
162.4151	19522.75	162.4106	19520.14	162.4032	19514.85	163.4284	19517.53	162.4088	19518.74	162.4076	19517.39	162.4119
162.6577	19518.67	162.6621	19514.82	162.6605	19518.91	163.6799	19520.48	162.6582	19518.44	162.6571	19520.67	162.66
162.9124	19519.67	162.9097	19519.16	162.9056	19521.46	163.9351	19520.55	162.9168	19519.71	162.911	19522.15	162.9052
163.1567	19524.36	163.1571	19519.88	163.1588	19519.77	164.1792	19517.25	163.1593	19518.18	163.1617	19515.1	163.1592
163.4116	19518.82	163.4099	19518.11	163.401	19519.75	164.4277	19515.87	163.4103	19520.19	163.4085	19519.12	163.4107
163.6599	19519.84	163.6607	19519.87	163.6638	19517.53	164.6781	19516.36	163.6584	19518.94	163.6544	19521.13	163.6613
163.9133	19521.05	163.9129	19514.06	163.904	19516.85	164.9321	19518.61	163.9159	19520.86	163.9088	19516.76	163.9056
164.1591	19522.98	164.1554	19519.9	164.1591	19518.77	165.1846	19520.48	164.1584	19517.99	164.1576	19517.84	164.1604
164.413	19519.8	164.4104	19522.01	164.4023	19517.25	165.4275	19522.84	164.4117	19518.51	164.4096	19515.96	164.412
164.6596	19521.67	164.6626	19517.78	164.6613	19518.25	165.6786	19516.23	164.6581	19518.39	164.6551	19517.35	164.6597
164.9161	19520.56	164.9102	19518.68	164.9042	19516.57	165.9348	19517.15	164.9171	19523.08	164.9126	19518.33	164.9064
165.1579	19524.92	165.1591	19521.05	165.1586	19518.54	166.18	19518.03	165.1585	19519.42	165.1744	19514.87	165.1607
165.4121	19521.27	165.4103	19517.37	165.4034	19524.15	166.4302	19519.34	165.4117	19520.09	165.411	19519.78	165.4146
165.66	19519.43	165.6612	19514.59	165.6611	19516.82	166.6792	19519.04	165.6598	19517.5	165.6579	19517.43	165.6666
165.9175	19518.85	165.9135	19515.15	165.9068	19518.94	166.9348	19513.92	165.9203	19520.74	165.9161	19512.67	165.9053
166.161	19517.8	166.1579	19521.96	166.1594	19515.78	167.1866	19518.26	166.1601	19516.28	166.157	19522.91	166.1641
166.4123	19520.09	166.412	19518.14	166.4038	19523.59	167.4283	19517.57	166.4152	19519.49	166.4102	19519.16	166.4206
166.6606	19522.16	166.665	19519.2	166.6721	19518.87	167.6806	19514.51	166.6624	19521.68	166.6557	19516.52	166.6725
166.9147	19522.95	166.9102	19521.7	166.9062	19520.68	167.9361	19518.15	166.9192	19518.23	166.9167	19519.71	166.9105
167.1596	19519.91	167.1585	19516.95	167.1587	19512.73	168.1827	19517.98	167.16	19518.2	167.1561	19519.01	167.1646
167.4169	19519.14	167.4144	19511.85	167.4037	19516.23	168.431	19518.2	167.4119	19516.58	167.4123	19520.72	167.4128
167.6587	19520.8	167.6618	19519.37	167.6657	19518.75	168.6785	19522.32	167.6594	19515.81	167.6576	19517.71	167.6622
167.9133	19524.31	167.909	19522.65	167.9123	19519.14	168.9377	19515.92	167.9193	19517.26	167.9163	19518.88	167.9072
168.1601	19523.31	168.1579	19522.61	168.161	19516.6	169.1819	19517.98	168.1591	19519.16	168.1585	19521.27	168.1627
168.415	19523.7	168.4137	19521.2	168.4051	19515.55	169.4341	19514.68	168.4148	19514.7	168.4121	19516.49	168.4136
168.6579	19526.9	168.6657	19520.44	168.6626	19517.3	169.6826	19518.76	168.6614	19519.52	168.6566	19517.14	168.663
168.9146	19524.6	168.915	19518.89	168.9074	19513.27	169.9361	19515.79	168.9192	19517.22	168.9157	19520.37	168.9086
169.1618	19516.1	169.1599	19517.81	169.1599	19518.76	170.1966	19515.71	169.1591	19520.72	169.1588	19516.16	169.1638
169.4171	19522.97	169.4117	19519.23	169.4043	19516.68	170.4302	19520.41	169.4133	19520.12	169.4116	19513.83	169.4155
169.6599	19521.41	169.6639	19521.07	169.6638	19517.52	170.682	19518.35	169.66	19518.08	169.6583	19517.27	169.664
169.9128	19521.4	169.914	19521.61	169.9058	19516.45	170.9373	19515.83	169.9254	19519.83	169.917	19525.88	169.9087
170.16	19524.2	170.1607	19519.64	170.1598	19519.45	171.1843	19519.57	170.1736	19518.59	170.1616	19522.8	170.1649
170.4151	19522.69	170.4118	19519.76	170.4096	19516.19	171.4333	19521.81	170.414	19520.51	170.412	19518.32	170.4218
170.6645	19520.15	170.6629	19520.64	170.6628	19514.62	171.6843	19517.77	170.6605	19518.67	170.6593	19520.63	170.6722
170.9157	19515.68	170.9147	19518.83	170.9067	19519.07	171.9396	19515.83	170.9186	19520.92	170.9225	19513.04	170.909
171.1599	19519.45	171.1649	19519.99	171.1632	19516.43	172.1886	19515.93	171.1604	19519.64	171.1596	19511.62	171.1642
171.4164	19520.06	171.4118	19516.2	171.4065	19516.8	172.4338	19519.32	171.4136	19523.75	171.4135	19519.89	171.4185
171.6611	19517.89	171.6663	19521.47	171.666	19515.11	172.685	19518.57	171.6602	19519.84	171.6593	19515.59	171.6637
171.9187	19515.27	171.9128	19524.2	171.9041	19518.95	172.9413	19516.97	171.9217	19516.11	171.9168	19519.07	171.9092
172.1599	19516.18	172.1616	19519.15	172.1611	19518.16	173.1828	19516.02	172.1625	19519.48	172.1594	19519.86	172.1672
172.4139	19523.96	172.4129	19515.18	172.4063	19521.12	173.4322	19514.36	172.4167	19519.21	172.4133	19519.82	172.4159
172.6672	19521.74	172.662	19520.05	172.6643	19518.39	173.6842	19521.19	172.6619	19517.83	172.6588	19516.18	172.6649
172.9189	19526.06	172.9145	19520.16	172.9057	19516.5	173.9404	19519.93	172.9221	19520.1	172.9142	19517.89	172.9084
173.1604	19520.5	173.1599	19519.33	173.1609	19516.09	174.1845	19517.57	173.1605	19522.73	173.1611	19518.19	173.168
173.4154	19523.8	173.4182	19514.32	173.4075	19516.55	174.4351	19516.61	173.4166	19519.08	173.4142	19519.64	173.4183
173.6628	19518.42	173.6623	19525.03	173.6629	19516.79	174.6868	19518.11	173.6615	19518	173.6583	19517.57	173.6639

173.9161	19516.36	173.9172	19519.88	173.9077	19515.59	174.9399	19518.74	173.921	19516.76	173.9162	19520.71	173.909
174.1621	19518.1	174.1622	19516.18	174.1619	19520.42	175.185	19517.26	174.1616	19518.67	174.1601	19519.35	174.1665
174.4195	19520.06	174.4122	19520.93	174.4075	19516.16	175.4344	19515.86	174.4184	19518.01	174.4151	19519.72	174.4186
174.662	19517.12	174.6673	19515.86	174.6694	19517.92	175.685	19518.31	174.6624	19516.5	174.6605	19519.32	174.6658
174.9162	19519.54	174.9123	19516.08	174.9088	19520.71	175.9381	19518.35	174.9221	19519.64	174.9175	19517.41	174.9098
175.162	19521.25	175.1634	19517.06	175.1618	19513.05	176.185	19517.33	175.1617	19520.55	175.1633	19519.58	175.1642
175.4152	19516.16	175.4126	19516.42	175.4055	19518.24	176.435	19516.06	175.4151	19518.85	175.4152	19520.22	175.4167
175.6625	19517.85	175.6647	19515.4	175.6653	19519.66	176.6843	19513.57	175.6625	19518.27	175.6605	19515.71	175.6666
175.9192	19520.9	175.9155	19515.33	175.9068	19511.27	176.9403	19520.19	175.9235	19522.06	175.9195	19521.74	175.9113
176.1611	19521.53	176.1608	19517.75	176.1624	19518.42	177.1876	19516.82	176.1621	19518.05	176.159	19520.53	176.1691
176.4178	19522.3	176.4119	19514.85	176.408	19514.76	177.4343	19515.68	176.4185	19517.02	176.4147	19515.26	176.42
176.6642	19521.26	176.6643	19518.15	176.6688	19515.2	177.6883	19516.68	176.6613	19517.61	176.661	19520.9	176.6665
176.926	19522.73	176.9172	19521.2	176.911	19517.82	177.94	19520.46	176.9237	19517.77	176.9174	19514.93	176.9106
177.1684	19521.5	177.163	19515.83	177.1627	19516.01	178.1868	19516.68	177.1646	19518.57	177.1607	19519.19	177.1656
177.4165	19522.93	177.412	19519.62	177.408	19516.07	178.4338	19520.73	177.4337	19516.19	177.4155	19518.93	177.4179
177.6631	19518.29	177.6657	19520.63	177.6666	19518.56	178.6855	19519.08	177.6638	19521.45	177.6605	19517.32	177.6669
177.918	19519.61	177.9195	19518.4	177.9076	19512.58	178.937	19515.67	177.9231	19519.19	177.9168	19516.71	177.9099
178.1632	19517.95	178.1656	19519.64	178.1655	19517.79	179.1869	19520.22	178.1646	19521.03	178.1629	19519.66	178.1671
178.4174	19517.36	178.4155	19520.37	178.4083	19517.9	179.4358	19516.13	178.4189	19518.11	178.4173	19517.9	178.4178
178.6619	19520.43	178.6643	19519.49	178.666	19515.67	179.6836	19517.09	178.6625	19519.49	178.661	19521.94	178.6694
178.9185	19523.27	178.9184	19520.31	178.9097	19515.41	179.9414	19518.83	178.9241	19520.79	178.9197	19519.23	178.9123
179.1639	19522.34	179.1629	19513.03	179.1649	19519.44	180.188	19515.08	179.1636	19521.39	179.1618	19513.15	179.1665
179.4194	19517.45	179.4149	19517.02	179.4084	19517.15	180.4346	19518.3	179.4213	19518.75	179.4192	19518.77	179.4184
179.6639	19520.36	179.6664	19521.22	179.6665	19518.71	180.6832	19514.62	179.6641	19518.05	179.664	19513.34	179.6669
179.9198	19519.43	179.9151	19521.01	179.9107	19518.71	180.9417	19515.77	179.9253	19515.97	179.9228	19519.55	179.9131
180.1639	19520.71	180.1678	19516.71	180.1663	19519.68	181.1877	19515.96	180.1638	19516.78	180.1636	19523.11	180.1673
180.4207	19517.64	180.4156	19518.75	180.4087	19517.51	181.4353	19519.96	180.4179	19520.97	180.4203	19514.94	180.4207
180.6652	19522.77	180.6677	19516.67	180.667	19516.55	181.6845	19518.67	180.666	19519.06	180.6626	19518.33	180.6695
180.9215	19519.17	180.9149	19518.39	180.9105	19520.28	181.94	19519.17	180.9265	19517.85	180.9211	19512.98	180.9124
181.1633	19522.6	181.163	19520.23	181.171	19511.33	182.1891	19518.19	181.1796	19519.6	181.163	19516.23	181.1679
181.4176	19518.3	181.4157	19521.42	181.4105	19515.65	182.4361	19518.88	181.4241	19518.43	181.4173	19521.46	181.4203
181.6661	19520.94	181.6689	19517.79	181.6678	19514.57	182.6868	19518.22	181.6636	19517.11	181.6609	19518.86	181.6708
181.9261	19520.69	181.9177	19519.52	181.9098	19520.41	182.9418	19518.22	181.9237	19517.35	181.9213	19518.96	181.914
182.1631	19518.44	182.1649	19517.73	182.166	19513.19	183.1872	19516.75	182.1664	19518.64	182.1628	19519.28	182.1687
182.418	19518.72	182.4144	19515.05	182.4116	19518.82	183.4345	19517.97	182.4216	19522.62	182.4209	19518.89	182.4217
182.6789	19518.88	182.6669	19519.39	182.6695	19514.99	183.6848	19519.13	182.6641	19516.72	182.6623	19518.59	182.669
182.9205	19518.66	182.9166	19519.6	182.9095	19520.17	183.9415	19516.11	182.926	19519.13	182.9201	19517.09	182.9131
183.1661	19521.6	183.1672	19515.38	183.1661	19518.44	184.1898	19517.13	183.1664	19518.28	183.1627	19516.44	183.1711
183.4202	19514.95	183.416	19519.26	183.4127	19517.85	184.4363	19513.03	183.421	19519.42	183.4187	19517.77	183.4193
183.6644	19514.33	183.6657	19521.04	183.6687	19516.22	184.6873	19517.24	183.6689	19520.25	183.6632	19519.13	183.6693
183.9223	19519.72	183.9218	19521.08	183.9112	19515.05	184.9433	19515.94	183.9256	19519.35	183.9222	19518.62	183.913
184.165	19524.02	184.1668	19515.92	184.1671	19518.2	185.1908	19518.66	184.1649	19520.33	184.1625	19519.84	184.1668
184.4208	19518.38	184.4177	19520.15	184.4132	19517.31	185.4389	19519.36	184.421	19519.38	184.4188	19517.94	184.4201
184.6726	19520.65	184.6704	19518.18	184.6696	19521.75	185.6917	19519.53	184.6663	19516.08	184.6638	19517.79	184.6686
184.922	19518.19	184.918	19513.04	184.912	19515.41	185.9418	19513.35	184.9262	19518.15	184.9227	19516.77	184.9144
185.1667	19520.24	185.1663	19519.95	185.1705	19514.24	186.191	19518.39	185.166	19518.84	185.1689	19520.61	185.1687
185.4206	19518.61	185.4173	19516.85	185.4116	19516.15	186.4387	19517.34	185.4191	19519.5	185.4184	19519.62	185.4218
185.6658	19518.71	185.6699	19516.76	185.6705	19515.51	186.6876	19518.86	185.6662	19516.61	185.6634	19517.77	185.6701
185.9232	19520.69	185.9181	19516.36	185.913	19515.71	186.9458	19518.51	185.9279	19517.69	185.9245	19513.53	185.914
186.1645	19516.34	186.168	19517.28	186.1673	19515.36	187.192	19516.8	186.1675	19516.75	186.1652	19523.24	186.1688
186.4195	19520.3	186.4177	19514.88	186.4129	19515.59	187.4377	19520.12	186.4181	19516.08	186.4215	19515.16	186.4249
186.6692	19519.54	186.6694	19521.63	186.6715	19520.35	187.6884	19514.13	186.6681	19516.15	186.6652	19514.01	186.6784
186.924	19518.24	186.918	19518.49	186.9143	19519.12	187.946	19517.72	186.9258	19517.08	186.9215	19519.09	186.9148
187.1672	19520.57	187.1658	19515.14	187.1695	19516.43	188.1915	19518.53	187.1682	19517.05	187.1653	19519.09	187.169
187.4205	19520.81	187.4181	19518.36	187.4128	19515.67	188.4407	19517.29	187.4207	19519.85	187.4198	19519.46	187.4219
187.6656	19521.69	187.6715	19513.89	187.6736	19516.84	188.6891	19517.41	187.667	19519.02	187.6654	19515.62	187.675
187.9231	19518.86	187.9183	19519.63	187.9115	19519.53	188.943	19515.45	187.9272	19515.78	187.9321	19516.14	187.9151
188.1677	19519.62	188.1649	19517.62	188.169	19517.49	189.191	19516.26	188.1685	19519.71	188.1687	19515.72	188.1707
188.4224	19520.91	188.4177	19516.89	188.4153	19515.9	189.4387	19516.25	188.4213	19518.43	188.4215	19515.36	188.4214
188.6661	19516.3	188.6768	19515.65	188.6705	19517.76	189.6886	19518.71	188.6668	19518.15	188.6675	19517.74	188.6715
188.9238	19519.63	188.9305	19522.92	188.914	19512.91	189.9473	19514.7	188.9284	19517.31	188.9275	19513.35	188.916
189.1726	19522.32	189.1675	19518.84	189.1693	19515.85	190.1917	19516.52	189.1695	19517.19	189.1658	19514.34	189.17
189.4221	19517.4	189.4192	19516.86	189.4129	19516.82	190.4386	19515.43	189.4227	19516.37	189.4206	19515.64	189.4235
189.6676	19524.37	189.6759	19516.36	189.6731	19511.42	190.6918	19516.96	189.6724	19517.88	189.6666	19512.6	189.6725
189.9224	19522.39	189.9221	19516.89	189.9133	19512.27	190.9447	19519.23	189.9294	19517.3	189.9269	19519.59	189.9159
190.1664	19520.97	190.1692	19516.37	190.1691	19518.52	191.1922	19515.76	190.169	19516.16	190.1698	19517.65	190.1711
190.4214	19514.84	190.4216	19521.29	190.4134	19519.18	191.4386	19514.84	190.4218	19516.96	190.4199	19518	190.4265
190.668	19521.83	190.6714	19519.84	190.6727	19515.92	191.6906	19517.97	190.6685	19519.79	190.6668	19515.01	190.6788
190.9239	19518.86	190.9224	19515.4	190.9158	19517.41	191.9465	19520.13	190.93	19516.11	190.9251	19517.85	190.9167
191.1701	19516.78	191.168	19515.4	191.1711	19515.71	192.1941	19516.48	191.1679	19520.85	191.1673	19520.48	191.172

191.4218	19520.71	191.419	19515.75	191.4153	19516.38	192.4424	19514.52	191.4199	19517.19	191.4207	19515.41	191.4276
191.67	19516.15	191.6706	19515.87	191.6782	19513.96	192.6914	19515.28	191.67	19513.65	191.6651	19518.17	191.6741
191.9257	19518.03	191.9229	19517.96	191.9169	19518.79	192.9445	19516.41	191.9277	19516.07	191.9254	19517.76	191.9184
192.1723	19519.52	192.1696	19516.94	192.1713	19512.95	193.1921	19517.22	192.1702	19524.72	192.1664	19516.53	192.1737
192.4218	19521.37	192.4202	19521.76	192.4134	19516.21	193.4409	19515.24	192.4217	19520	192.4205	19519.02	192.4249
192.6691	19524.5	192.6706	19517.1	192.6814	19518.47	193.6926	19516.37	192.671	19514.37	192.6687	19520.35	192.673
192.9244	19514.06	192.9227	19516.18	192.9138	19518.05	193.9463	19517.55	192.9286	19520.59	192.927	19519.55	192.9182
193.1696	19515.55	193.1701	19516.78	193.1693	19514.11	194.2144	19515.04	193.1689	19517.33	193.1661	19516.86	193.1737
193.4239	19519.69	193.4229	19517.32	193.4136	19517.69	194.4389	19515.05	193.4232	19514.67	193.4213	19518.56	193.4261
193.6719	19520.3	193.6694	19519.43	193.6733	19517.82	194.6942	19516.95	193.6706	19517.52	193.6682	19515.65	193.673
193.9266	19518.77	193.9265	19515.42	193.9158	19516.13	194.946	19516.38	193.9284	19519.02	193.926	19516.17	193.9182
194.1702	19518.01	194.1696	19518.02	194.1723	19516.62	195.1957	19519.35	194.1714	19517.65	194.171	19514.46	194.1725
194.4244	19517.41	194.4218	19518.86	194.4145	19513.5	195.4419	19515.72	194.4229	19519.01	194.4208	19519.29	194.4261
194.67	19522.96	194.6734	19519.59	194.6723	19518.7	195.6938	19518.01	194.6703	19520.32	194.6687	19509.72	194.6738
194.9248	19517.92	194.9209	19516.59	194.9162	19515.01	195.9463	19517.12	194.9297	19518.9	194.9296	19520.3	194.9158
195.1696	19519.71	195.168	19513.41	195.1717	19516.88	196.1913	19514.97	195.1706	19517.1	195.1729	19516.96	195.1717
195.4232	19519.81	195.4242	19520.02	195.416	19519.81	196.4411	19514.22	195.4284	19516	195.4229	19517.65	195.4246
195.6702	19516.66	195.6696	19514.07	195.6737	19518.22	196.6924	19513.39	195.6701	19518.12	195.6677	19515.61	195.6728
195.9271	19519.03	195.9231	19516	195.9157	19514.27	196.9461	19514.36	195.9296	19516.87	195.9263	19517.1	195.9299
196.1696	19520.09	196.1717	19518.71	196.1699	19516.19	197.196	19513.98	196.1715	19518.06	196.1702	19514.64	196.1818
196.424	19519.7	196.4215	19514.98	196.417	19517.63	197.441	19515.14	196.4246	19519.05	196.4236	19517.97	196.4276
196.6711	19519.37	196.6708	19515.94	196.6771	19516.59	197.6941	19516.08	196.674	19519	196.6712	19515.2	196.6745
196.931	19521.38	196.9244	19523.22	196.9168	19518.26	197.9463	19518.04	196.9347	19516.94	196.9337	19514.6	196.9193
197.172	19522.68	197.1712	19518.87	197.1697	19511.88	198.1944	19514.08	197.1727	19518.22	197.1714	19516.69	197.1759
197.4257	19521.92	197.4228	19515.51	197.4168	19518.76	198.4414	19512.2	197.4238	19515.96	197.4263	19516.54	197.4261
197.6708	19519.09	197.6705	19515.23	197.6762	19520.82	198.6993	19518.37	197.6742	19513.5	197.6709	19521.02	197.6754
197.9289	19521.83	197.9275	19514.27	197.9152	19516.8	198.9461	19513.94	197.9305	19514.12	197.9306	19515.33	197.9191
198.1796	19519.15	198.1795	19514.66	198.1802	19521.01	199.2047	19517.02	198.1794	19519.02	198.179	19519.45	198.1847
198.4239	19517.69	198.4222	19514.64	198.4169	19517.76	199.4404	19516.68	198.4264	19519.21	198.4265	19515.83	198.4281
198.6699	19521.16	198.6703	19519.36	198.6774	19514.23	199.6931	19513.37	198.6741	19522.09	198.6699	19515.84	198.6757
198.9279	19519.23	198.9267	19521.89	198.9182	19520.98	199.9488	19516.49	198.9312	19516.85	198.9292	19518.72	198.9189
199.1715	19516.3	199.1743	19519.36	199.1704	19514.2	200.194	19515.32	199.1723	19514.29	199.1698	19514.96	199.1753
199.4281	19521.6	199.4231	19517.49	199.4176	19516.3	200.446	19516.21	199.4247	19515.37	199.4253	19515.8	199.4264
199.6705	19519.28	199.6719	19515.78	199.6751	19515.73	200.6983	19515.22	199.6758	19515.79	199.6718	19513.99	199.677
199.9254	19519.78	199.9256	19511.48	199.9172	19516.93	200.9527	19516.96	199.9316	19517.46	199.9288	19517.35	199.9203
200.173	19519.89	200.1724	19517.84	200.1767	19515.13	201.2001	19515.34	200.1729	19517.84	200.1728	19513.71	200.177
200.4331	19522.81	200.4319	19522.66	200.4196	19514.72	201.4483	19512.26	200.4302	19517.25	200.4283	19520.67	200.4332
200.6773	19516.68	200.6759	19519.86	200.6806	19519.47	201.6987	19517.18	200.6788	19517.06	200.6769	19515.25	200.6792
200.931	19518.81	200.9296	19514.33	200.923	19516.36	201.9552	19513.92	200.9347	19515.75	200.9321	19511.45	200.9253
201.1764	19518.71	201.1753	19518.59	201.1778	19521.23	202.2035	19514.32	201.1781	19518.96	201.1753	19518.48	201.1809
201.4309	19523.42	201.429	19517.22	201.424	19521.39	202.446	19515.56	201.4305	19516.14	201.4303	19515.54	201.4335
201.6795	19518.4	201.6767	19517.61	201.6813	19519.41	202.698	19516.53	201.6789	19516.84	201.6765	19520.3	201.6826
201.9316	19519.61	201.9297	19515.42	201.9209	19517.06	202.9534	19513.83	201.9361	19515.98	201.9347	19519	201.9266
202.177	19521.1	202.1746	19515.04	202.1771	19519.52	203.2004	19517.74	202.1764	19521.01	202.1778	19517.16	202.1819
202.4313	19518.12	202.4265	19518.4	202.4221	19517.98	203.448	19518.41	202.4313	19518.41	202.4302	19517.29	202.4345
202.6779	19518.84	202.6766	19518.57	202.6806	19513.33	203.6991	19511.87	202.6779	19516.64	202.6789	19513.02	202.681
202.9324	19517.14	202.9305	19514.58	202.9216	19514.84	203.9533	19513.16	202.9364	19514.36	202.9421	19515.27	202.9262
203.1769	19519.56	203.1801	19519.27	203.196	19519.15	204.2015	19517.53	203.1785	19517.73	203.1782	19515.04	203.1817
203.4287	19518.44	203.4276	19516.5	203.4223	19515.75	204.4485	19518.61	203.4293	19520.54	203.4321	19519.44	203.4304
203.6773	19524.17	203.6758	19517.65	203.6817	19518.1	204.699	19512.84	203.6802	19518.77	203.6767	19518.74	203.6803
203.9311	19519.29	203.9333	19517.71	203.9209	19518.44	204.9536	19514.81	203.9366	19517.51	203.935	19519.62	203.9263
204.1778	19516.96	204.1773	19515.14	204.1765	19513.41	205.2007	19515.56	204.1796	19516.75	204.1769	19517.56	204.1823
204.4324	19516.79	204.4311	19517.69	204.4242	19518.53	205.4507	19513.48	204.4313	19518.74	204.4325	19512.76	204.432
204.6773	19523.51	204.6768	19518.72	204.683	19519.17	205.6994	19516	204.679	19515.93	204.6786	19515.66	204.6818
204.9318	19523.57	204.9331	19520.61	204.9225	19516.5	205.9545	19516.99	204.9364	19518.59	204.9329	19513.26	204.9266
205.1791	19519.27	205.1779	19520.78	205.1772	19515.69	206.2008	19512.8	205.1794	19516.44	205.1796	19514.39	205.1818
205.4332	19519.15	205.4301	19520.12	205.4241	19517.63	206.4482	19513.73	205.4291	19518.23	205.4314	19514.37	205.4322
205.6805	19520.65	205.6779	19518.3	205.6815	19516.93	206.6986	19517.54	205.6788	19517.35	205.6787	19513.41	205.6822
205.9315	19518.19	205.9343	19517.96	205.9239	19516.42	206.9544	19514.41	205.937	19517.12	205.9325	19513.52	205.9279
206.179	19519.57	206.18	19510.1	206.1772	19517.7	207.2032	19518.06	206.1837	19516.12	206.177	19516.14	206.1806
206.4321	19516.55	206.4304	19520.44	206.4257	19516.12	207.4503	19516.47	206.4341	19521.51	206.4289	19516.19	206.4372
206.6805	19519.38	206.6806	19520.37	206.6862	19514.53	207.7024	19516.43	206.6799	19516.45	206.6785	19513.7	206.6843
206.9335	19519.04	206.9344	19516.55	206.9237	19516.59	207.9571	19515.22	206.9379	19516.52	206.9381	19516.06	206.9282
207.1803	19515.48	207.1778	19515.64	207.1787	19513.84	208.2021	19515.85	207.1799	19519.38	207.1767	19518.82	207.1831
207.4334	19517.44	207.4317	19516.53	207.4244	19515.88	208.4496	19518.1	207.4341	19517.79	207.4302	19516.69	207.4331
207.6814	19518.86	207.6804	19514.32	207.6818	19513.61	208.7002	19515.51	207.6801	19519.91	207.7019	19516.13	207.6841
207.9363	19521.6	207.9343	19515.53	207.9249	19516.55	208.9573	19513.3	207.9368	19517.08	207.9392	19515.66	207.9268
208.1799	19519.97	208.1788	19513.31	208.1795	19517.32	209.2031	19513.1	208.1808	19521.38	208.1796	19516.72	208.1825
208.4323	19518.01	208.4311	19512.43	208.4264	19514.13	209.4512	19515.49	208.4365	19513.09	208.4333	19520.53	208.4351
208.6839	19518.74	208.679	19521.14	208.6842	19517.78	209.7015	19514.54	208.6791	19517.32	208.6798	19522.57	208.683

208.9354	19518.38	208.9355	19519.96	208.9265	19517.9	209.9528	19513.3	208.9397	19516.65	208.9332	19518.97	208.9322
209.1803	19522.19	209.1791	19517.54	209.1797	19512.36	210.2031	19517.06	209.1857	19519.92	209.1785	19517.01	209.1823
209.4331	19521.89	209.4307	19520.31	209.4259	19514.5	210.4508	19513.09	209.4381	19518.53	209.4356	19518.31	209.4344
209.6813	19520.66	209.6801	19518.29	209.6854	19517.56	210.7061	19518.61	209.6813	19516.71	209.6826	19513.81	209.686
209.9345	19517.02	209.9352	19518.2	209.9276	19516.34	210.9552	19518.24	209.9372	19516.7	209.937	19516.43	209.9301
210.1814	19519.6	210.1799	19514.9	210.1829	19515.83	211.2039	19514.32	210.1812	19517.11	210.1828	19516.93	210.1862
210.4373	19526.78	210.4327	19516.89	210.4287	19516.24	211.4519	19516.02	210.4336	19516.25	210.4356	19514.42	210.4364
210.6801	19518.74	210.6812	19515.16	210.689	19512.93	211.703	19514.88	210.6805	19515.04	210.682	19515.29	210.6858
210.9347	19520.93	210.9368	19515.24	210.9268	19515.6	211.9583	19513.56	210.9378	19518.05	210.9348	19516.83	210.9304
211.1818	19519.36	211.1802	19515.8	211.1806	19519.56	212.2066	19516.23	211.1803	19512.23	211.1804	19514.18	211.1849
211.4341	19516.84	211.4318	19515.6	211.4265	19518.23	212.4523	19515.69	211.4331	19516.64	211.4332	19518.87	211.4386
211.6822	19521.19	211.6839	19513.86	211.6864	19515.13	212.7038	19517.44	211.6811	19518.51	211.6795	19516.57	211.686
211.9364	19517.74	211.935	19518.81	211.9266	19512.28	212.9563	19515.99	211.9396	19518.49	211.939	19517.62	211.9309
212.1797	19522.49	212.1817	19517.72	212.181	19518.41	213.2057	19516.76	212.1818	19517.87	212.1794	19521.23	212.1845
212.4336	19515.54	212.4339	19515.25	212.4258	19517.69	213.4509	19515.01	212.4341	19516.61	212.4339	19512.66	212.4359
212.6822	19519.05	212.6826	19519.44	212.6845	19516.03	213.7035	19514.58	212.682	19512.71	212.6809	19512.6	212.6874
212.9416	19519.17	212.9381	19516.36	212.9265	19512.63	213.9585	19514.48	212.9419	19515.62	212.9395	19519.71	212.9301
213.1811	19518.13	213.1798	19518.65	213.182	19516.08	214.2062	19515.59	213.1817	19518.76	213.1809	19515.5	213.185
213.4336	19516.5	213.4336	19514.95	213.433	19518.46	214.4513	19515.86	213.4362	19517.12	213.4338	19515.09	213.436
213.682	19519.38	213.6834	19521.18	213.6852	19516.51	214.7035	19515.51	213.6807	19517.63	213.6821	19510.54	213.6858
213.9371	19518.11	213.937	19518.89	213.9279	19516.72	214.9585	19514.9	213.9418	19515.75	213.9362	19516.04	213.9313
214.1954	19522.59	214.1828	19517.51	214.1817	19514.92	215.2072	19514.77	214.1838	19519.57	214.1805	19518.81	214.1849
214.4421	19519.2	214.434	19511.23	214.4268	19518.34	215.4534	19514.05	214.436	19514.33	214.435	19514.25	214.4355
214.6838	19519.01	214.6848	19516.48	214.6869	19511.97	215.7058	19517.26	214.6819	19515.47	214.6798	19516.45	214.6867
214.936	19515.03	214.9371	19512.77	214.9261	19516.41	215.9632	19513.5	214.9404	19517.2	214.9459	19516.54	214.9331
215.1826	19517.42	215.1828	19518.17	215.182	19518.52	216.2077	19514.65	215.1819	19517.41	215.1868	19516.08	215.1841
215.4359	19512.86	215.4347	19515.29	215.4286	19515.32	216.4522	19515.66	215.4351	19517.04	215.4357	19513.6	215.4381
215.683	19522.29	215.6825	19511.33	215.69	19521.27	216.7047	19515	215.6838	19521.52	215.6808	19516.54	215.6874
215.9365	19517.48	215.9392	19519.42	215.9281	19524.66	216.9599	19518.15	215.9434	19516.44	215.9415	19514.63	215.9325
216.1814	19523.31	216.1823	19517.33	216.1812	19516.83	217.2079	19516.27	216.1961	19512.59	216.1815	19519.95	216.1877
216.4364	19522.84	216.4339	19514.09	216.4283	19513.47	217.4531	19516.77	216.4328	19518.38	216.4344	19515.36	216.4642
216.6834	19518.4	216.6855	19517.76	216.6901	19508.81	217.7042	19514.89	216.683	19515.73	216.6824	19514.85	216.7069
216.9386	19521.48	216.9368	19517.28	216.9285	19518.08	217.9606	19513.6	216.9485	19517.58	216.9428	19516.78	216.9372
217.1828	19519.96	217.1843	19515.95	217.1817	19516.8	218.2074	19514.54	217.1831	19518.51	217.1836	19520.39	217.1866
217.4355	19519.2	217.4366	19513.81	217.4273	19516.45	218.4542	19514.59	217.4362	19514.85	217.4371	19516.17	217.4364
217.6848	19516.11	217.6846	19518.02	217.6886	19518.35	218.7057	19516.09	217.6817	19515.26	217.6812	19513.68	217.688
217.9382	19518.98	217.9448	19510.99	217.9289	19516.35	218.9583	19511.41	217.9412	19516.68	217.9391	19517.11	217.9327
218.1835	19515.72	218.1817	19519.38	218.1836	19515.72	219.2078	19516.3	218.1858	19519.51	218.1823	19517.51	218.1881
218.4367	19522.61	218.4372	19516.13	218.4264	19518.93	219.4522	19517.07	218.4366	19516.75	218.4378	19515	218.4392
218.687	19519.82	218.6847	19513.98	218.6875	19511.04	219.7061	19518.57	218.6845	19518.8	218.6826	19511.9	218.6874
218.9389	19519.2	218.9427	19521.74	218.9292	19513.17	219.9624	19516.22	218.9631	19517.92	218.941	19517.21	218.9359
219.1843	19521.8	219.1834	19517.94	219.1826	19517.6	220.2093	19515	219.1841	19518.26	219.1839	19513.93	219.1899
219.4389	19519.08	219.4365	19517.65	219.4275	19516.06	220.4538	19517.73	219.4398	19514.42	219.4397	19517.43	219.4391
219.6842	19516.25	219.6848	19515.95	219.6863	19516.74	220.7061	19518.19	219.6859	19517.93	219.685	19517.01	219.6879
219.938	19520.9	219.9374	19514.41	219.933	19518.27	220.9578	19516.86	219.9432	19519.8	219.9417	19509.74	219.9364
220.1839	19520.87	220.1839	19514.66	220.184	19516.94	221.2086	19513.07	220.1851	19517.3	220.1855	19517.46	220.1872
220.44	19517.66	220.4358	19515.85	220.4287	19515.5	221.4553	19514.87	220.4381	19515.09	220.4373	19511.3	220.437
220.6849	19514.7	220.6872	19514.39	220.6893	19512.43	221.7064	19514.2	220.685	19517.6	220.6839	19516.56	220.6968
220.942	19516.07	220.938	19519	220.9304	19515.6	221.9594	19515.52	220.9443	19518.21	220.9484	19519.16	220.938
221.1837	19520.07	221.1848	19513.21	221.1851	19516.39	222.2103	19511.83	221.1856	19517.92	221.1843	19517.76	221.1893
221.448	19520.26	221.4367	19518.73	221.4295	19511.28	222.4541	19514.71	221.4382	19518.58	221.4362	19521.46	221.4435
221.6805	19516.81	221.685	19516.1	221.6927	19517.72	222.7065	19515.38	221.6862	19518.11	221.6843	19520.17	221.6895
221.9438	19516.26	221.9429	19514.48	221.9329	19516.77	222.9602	19515.77	221.9463	19515.27	221.9426	19512.39	221.935
222.1847	19517.21	222.1849	19513.17	222.1827	19520.18	223.2096	19517.26	222.1859	19516.25	222.1865	19515.56	222.1888
222.439	19523.45	222.4356	19516.14	222.4318	19514.05	223.4536	19516.84	222.4368	19518.26	222.4368	19512.55	222.4383
222.6896	19516.23	222.6846	19515.21	222.6908	19516.42	223.7066	19514.24	222.6862	19514.95	222.6851	19519.8	222.6888
222.9415	19521.27	222.9387	19520.28	222.932	19515.4	223.9608	19516.04	222.9455	19514.64	222.9398	19521.53	222.9341
223.1859	19518.36	223.1877	19513.42	223.1845	19515.82	224.2098	19514.43	223.1866	19516.64	223.187	19521.37	223.1882
223.4398	19521.33	223.4366	19519.21	223.4306	19520.11	224.4594	19513.34	223.438	19515.26	223.4426	19513.2	223.4375
223.6868	19515.68	223.686	19516.17	223.6905	19515.66	224.707	19524.1	223.6856	19518.05	223.6851	19521.51	223.692
223.9432	19515.28	223.9414	19518.6	223.9313	19513.8	224.96	19513.4	223.9457	19516.8	223.9438	19518.11	223.9349
224.1866	19522.29	224.1864	19512.78	224.1851	19514.65	225.2086	19516.89	224.1871	19517.48	224.1852	19515.24	224.1904
224.4391	19517.79	224.4397	19517.52	224.4298	19518.57	225.4555	19519.83	224.4395	19513.36	224.4411	19513.87	224.4396
224.6864	19519.19	224.6871	19514.92	224.6911	19513.15	225.7103	19516.1	224.6847	19517.15	224.689	19519.49	224.69
224.9417	19516.02	224.9424	19512.4	224.9329	19515.76	225.9614	19515.29	224.9447	19517.7	224.9443	19516.11	224.9364
225.1859	19514.94	225.1861	19517.76	225.1874	19512.66	226.2102	19515.41	225.187	19516.23	225.1898	19516.8	225.1897
225.441	19522.45	225.4373	19513.67	225.4326	19516.42	226.4565	19514.93	225.4388	19516.88	225.44	19520.69	225.4414
225.6866	19520.23	225.6879	19514.74	225.6926	19514.98	226.7101	19517.93	225.6852	19515.28	225.6876	19521.36	225.6903
225.9407	19515.94	225.94	19519.72	225.9327	19520.23	226.9613	19516.59	225.9444	19515.59	225.9466	19516.5	225.9392
226.1873	19516.96	226.1884	19516.5	226.1912	19519.48	227.2116	19517.24	226.1908	19518.99	226.1865	19513.67	226.1908

226.4425	19517.56	226.4387	19515.29	226.4355	19512.67	227.4599	19513.65	226.4425	19520.4	226.4408	19518.09	226.4444
226.6899	19516.62	226.6869	19512.35	226.6926	19515.35	227.7106	19514.33	226.687	19516.82	226.6865	19518.43	226.6954
226.9412	19517	226.9419	19511.89	226.9319	19517.86	227.962	19513.49	226.9458	19517.89	226.9441	19520.05	226.9374
227.1857	19518.15	227.1909	19520.3	227.1876	19513.87	228.2146	19514.24	227.1865	19513.26	227.1867	19520.3	227.1923
227.4438	19515.58	227.4461	19514.79	227.4333	19516.82	228.4585	19515.41	227.4399	19517.89	227.442	19517.9	227.4463
227.6905	19518.52	227.6873	19515.03	227.692	19514.88	228.711	19515.62	227.6868	19517.4	227.6866	19513.54	227.6904
227.9435	19516.4	227.9441	19515.61	227.9325	19517.55	228.966	19516.22	227.9443	19515.34	227.954	19512.93	227.9378
228.1867	19520.28	228.189	19512.7	228.1869	19515.86	229.2163	19516.45	228.1874	19518.83	228.1972	19517.32	228.1922
228.4407	19519.29	228.4414	19510.17	228.4316	19521.03	229.4618	19513.78	228.4402	19517.38	228.4407	19512.79	228.4444
228.6889	19522.68	228.6886	19516.4	228.6927	19517.23	229.7108	19515.49	228.6887	19519.75	228.6877	19515.24	228.6932
228.9434	19517.9	228.9445	19518.77	228.9334	19519.63	229.9596	19511.21	228.9429	19517.27	228.9459	19514.5	228.9392
229.1891	19521.89	229.1899	19522.04	229.1882	19514.86	230.2142	19515.34	229.188	19516.39	229.1873	19520.54	229.1939
229.4422	19519.85	229.4414	19518.23	229.4323	19513.95	230.4599	19517.49	229.4419	19514.93	229.4414	19513.44	229.4432
229.6882	19520.53	229.6883	19514.58	229.6934	19518.19	230.7108	19517.96	229.6894	19517.62	229.6874	19519.12	229.6935
229.9439	19517.5	229.9462	19517.92	229.9352	19517.91	230.9634	19515.87	229.9453	19516.41	229.9473	19512.09	229.9392
230.188	19520.19	230.1901	19514.03	230.188	19516.3	231.214	19513.08	230.1884	19513.7	230.1916	19516.21	230.1935
230.4415	19520.65	230.4411	19514.68	230.4334	19514.38	231.4608	19515.81	230.4411	19517.94	230.4416	19510.66	230.4473
230.6884	19516.25	230.6932	19514.41	230.6965	19514.11	231.712	19516.28	230.6891	19516.09	230.6888	19509.31	230.6947
230.943	19518.14	230.9372	19516.32	230.9357	19518.72	231.9655	19516.05	230.9464	19521.04	230.946	19513.12	230.9412
231.1917	19519.68	231.191	19513.56	231.1897	19518.34	232.2175	19512.34	231.1905	19517.92	231.1901	19519.13	231.1923
231.4444	19519.57	231.4418	19515.46	231.433	19513.3	232.4605	19516.43	231.4429	19517.44	231.4427	19517.82	231.4442
231.6923	19517.01	231.6899	19517.31	231.6946	19516.57	232.7148	19515.57	231.6893	19514.84	231.6872	19519.31	231.6937
231.943	19519.41	231.9456	19515.96	231.9349	19515.51	232.9644	19518.36	231.9471	19516.64	231.9454	19515.73	231.9439
232.1893	19514.39	232.1917	19516.89	232.1896	19514.36	233.2158	19517.13	232.1899	19518.09	232.1889	19519.66	232.1931
232.4439	19518.55	232.4408	19515.09	232.4358	19515.95	233.4598	19514.08	232.4437	19519.22	232.4438	19517.94	232.4455
232.6908	19518.81	232.6888	19518.45	232.6945	19519.26	233.7119	19514.91	232.6894	19518.7	232.6919	19513.22	232.693
232.9436	19522.15	232.9459	19519.33	232.9345	19515.93	233.9648	19513.79	232.9696	19517.43	232.9448	19512.63	232.9409
233.1882	19515.55	233.1905	19517.55	233.1904	19513.36	234.2163	19515.69	233.2083	19517.87	233.1887	19514.67	233.194
233.4438	19515.76	233.443	19517.42	233.4352	19511.67	234.4612	19514.44	233.4483	19513.94	233.4432	19509.39	233.4445
233.6947	19518.25	233.6912	19515.99	233.6938	19515.27	234.7159	19514.56	233.6898	19519.23	233.6892	19516	233.7106
233.9464	19518.14	233.9502	19515.02	233.9354	19518.34	234.9685	19514.59	233.9496	19515.33	233.9483	19512.39	233.9467
234.1887	19516.24	234.1866	19514.45	234.1879	19514.81	235.2149	19514.58	234.1857	19517.45	234.1912	19515.22	234.195
234.4438	19518.03	234.4424	19517.11	234.436	19517.31	235.4606	19514.81	234.4452	19519.62	234.4426	19516.42	234.4451
234.694	19516.42	234.6944	19522.3	234.698	19516.89	235.7144	19509.36	234.6922	19515.29	234.692	19511.31	234.694
234.9456	19521.01	234.9522	19516.33	234.9366	19517.15	235.9628	19517.78	234.9501	19514.75	234.9467	19519.76	234.9411
235.1871	19520.52	235.1884	19507.69	235.1911	19516.78	236.22	19512.94	235.1921	19515.76	235.1938	19517.55	235.195
235.4422	19521.78	235.4434	19511.87	235.4345	19518.35	236.4624	19516.64	235.4457	19517.69	235.4434	19517.74	235.4428
235.6922	19523.73	235.6907	19514.13	235.6963	19518.73	236.7125	19513.48	235.6917	19517.34	235.6898	19508.9	235.6959
235.9468	19516.62	235.9484	19517.23	235.9381	19517.45	236.9641	19514.54	235.9483	19517.43	235.9499	19515.26	235.9477
236.1905	19513.65	236.1858	19511.88	236.1921	19519.72	237.217	19518.59	236.1898	19512.73	236.1927	19513.78	236.1977
236.4433	19522.09	236.4432	19517.79	236.4359	19517.55	237.4618	19518.87	236.4454	19515.09	236.4448	19515.94	236.4457
236.6918	19523.36	236.6919	19510.57	236.6988	19516.13	237.7126	19514.48	236.6925	19512.27	236.6914	19518.15	236.6952
236.9451	19523.11	236.9508	19514.33	236.9424	19515.23	237.9635	19516.01	236.9494	19518.68	236.9501	19522.8	236.9435
237.1901	19515.97	237.1899	19517.41	237.1917	19512.53	238.2175	19511.87	237.189	19517.5	237.196	19516.88	237.1946
237.4454	19521.82	237.444	19519.25	237.4358	19516.09	238.4622	19517.87	237.4469	19516.02	237.4458	19515.45	237.4448
237.6916	19515.59	237.6955	19514.16	237.6952	19514.56	238.716	19517.95	237.6961	19518.21	237.692	19516.15	237.696
237.945	19518.52	237.9461	19513.12	237.937	19514.12	238.9683	19512.8	237.9483	19518.15	237.9512	19515.55	237.9429
238.1919	19518.7	238.1913	19515.66	238.1909	19517.48	239.2191	19517.14	238.1891	19514.53	238.1945	19520.27	238.195
238.4473	19518.77	238.4463	19518.43	238.4378	19514.17	239.466	19515.15	238.4442	19517.98	238.4444	19517.96	238.4447
238.6916	19519.46	238.6918	19514.99	238.6971	19509.52	239.7124	19515.87	238.6883	19516.86	238.6926	19520.69	238.6966
238.9461	19521.71	238.9504	19516.28	238.9366	19514.21	239.9661	19512.96	238.9501	19515.79	238.9477	19519.34	238.9451
239.1904	19518.38	239.1933	19515.86	239.1953	19512.28	240.2166	19518.23	239.1908	19517.81	239.1918	19519.22	239.1997
239.4474	19520.62	239.4453	19516.67	239.4396	19516.99	240.4653	19513.3	239.4467	19514.46	239.444	19516.82	239.4492
239.691	19523.91	239.6928	19515.88	239.697	19519.94	240.71	19512.2	239.6937	19516.18	239.6926	19524.07	239.6952
239.9473	19514.42	239.9479	19518.31	239.938	19520.1	240.9597	19516.74	239.9524	19515.27	239.9513	19512.62	239.9445
240.1877	19515.13	240.1919	19516.06	240.1912	19512.02	241.2133	19514.33	240.2111	19517.85	240.2035	19513.68	240.1975
240.4475	19518.8	240.4464	19518.06	240.4375	19511.57	241.4588	19515.14	240.4535	19515.12	240.4521	19523.89	240.4478
240.6862	19516.6	240.6885	19518.13	240.6918	19517.62	241.7117	19514.79	240.6909	19519.83	240.6916	19518.89	240.6915
240.9437	19522.72	240.96	19517.8	240.9354	19516.05	241.9659	19516.3	240.9515	19517.38	240.9428	19516.26	240.9497
241.1887	19517.61	241.1894	19515.34	241.202	19516.44	242.2155	19514.86	241.1903	19515	241.1898	19512.43	241.1949
241.4411	19523.54	241.4419	19517.44	241.4412	19518.81	242.4608	19511.75	241.4428	19516.29	241.4423	19520.14	241.4468
241.693	19519.7	241.6944	19513.86	241.7014	19523.48	242.7108	19517.43	241.6888	19517.11	241.6873	19518.07	241.7038
241.9448	19521.08	241.9492	19515.24	241.9409	19513.59	242.9622	19515	241.9467	19516.04	241.9522	19518.97	241.949
242.1883	19520.7	242.188	19514.69	242.1917	19513.27	243.2129	19517.54	242.1924	19516.05	242.1964	19517.91	242.1952
242.4425	19516.73	242.4421	19512.43	242.4432	19514.73	243.4594	19512.06	242.4439	19515.19	242.4423	19522.52	242.4481
242.6954	19517.23	242.6889	19517.84	242.6941	19518.4	243.7105	19512.29	242.6883	19515.35	242.6897	19514.46	242.6949
242.9456	19516.32	242.949	19512.75	242.9401	19515.32	243.9625	19518.67	242.9477	19517.96	242.9467	19512.96	242.944
243.1909	19520.58	243.1911	19518.3	243.1934	19517.58	244.2152	19516.7	243.1906	19518.37	243.1892	19517.7	243.1935
243.443	19515.42	243.4428	19514.62	243.4376	19516.96	244.4595	19514.66	243.4447	19514.35	243.4427	19517.77	243.4435
243.6923	19518.36	243.6902	19518.37	243.6973	19510.1	244.7123	19514.41	243.69	19515.54	243.6926	19512.39	243.693

243.955	19519.94	243.9484	19522.01	243.9401	19517.49	244.9639	19509.78	243.9459	19515.7	243.9513	19516.75	243.9398
244.1915	19516.67	244.1891	19517.99	244.1922	19514.94	245.2161	19512.3	244.1925	19515.6	244.1907	19517.76	244.1991
244.4452	19519.22	244.4433	19514.3	244.4374	19519.6	245.4613	19512.56	244.4438	19519.72	244.4415	19517.95	244.4436
244.6929	19519.98	244.6897	19512.54	244.6972	19517.26	245.7137	19513.06	244.6899	19515.91	244.6913	19522.94	244.6933
244.9438	19513.1	244.9485	19515.17	244.9384	19515.51	245.9667	19512.99	244.9517	19516.76	244.946	19514.23	244.9406
245.1945	19514.18	245.1901	19513.16	245.1908	19513.84	246.219	19514.38	245.1913	19515.87	245.1938	19520.55	245.1966
245.445	19521.36	245.4449	19515.35	245.4401	19514.54	246.4608	19511.25	245.443	19516.72	245.4439	19511.53	245.4437
245.705	19513.92	245.6916	19516.15	245.6986	19516.38	246.7171	19512.43	245.6911	19514.83	245.6887	19516.77	245.6932
245.9467	19518.93	245.9523	19516.86	245.9464	19515.74	246.97	19518.27	245.9482	19518.22	245.9478	19515.11	245.9394
246.1912	19519.57	246.1896	19519.13	246.1912	19519.5	247.2226	19516.03	246.1933	19515.75	246.194	19518.05	246.1976
246.4436	19516.66	246.4452	19517.86	246.4413	19518.15	247.4622	19515.51	246.443	19515.12	246.4458	19516.69	246.4447
246.6952	19514.18	246.6908	19515.93	246.7018	19514.35	247.7149	19516.86	246.691	19514.44	246.6895	19521.74	246.6946
246.9467	19518.25	246.9466	19513.7	246.942	19513.94	247.9695	19513.32	246.9487	19517.84	246.9447	19519.61	246.9408
247.1916	19520.02	247.1904	19515.3	247.193	19515.78	248.2133	19514.74	247.192	19520.06	247.1911	19514.72	247.1945
247.4446	19517.71	247.4453	19516.93	247.4385	19513.28	248.4608	19512.81	247.4451	19518.17	247.4437	19518.1	247.4442
247.6939	19517.47	247.6914	19513.54	247.7098	19516.33	248.7152	19512.13	247.6913	19515.77	247.6911	19518.73	247.6966
247.945	19522.62	247.946	19515.4	247.9531	19517.06	248.9656	19512.6	247.9504	19516.19	247.9457	19518.76	247.9415
248.1901	19523.67	248.1914	19514.07	248.2029	19512.15	249.2173	19513.79	248.1942	19516.89	248.1921	19515.37	248.197
248.4427	19517.46	248.4434	19513.35	248.4369	19516.08	249.4636	19515.72	248.4467	19517.32	248.4443	19516.75	248.4468
248.6945	19523.74	248.6915	19517.1	248.6976	19515.42	249.7155	19516.21	248.6927	19519.15	248.6904	19519.18	248.7036
248.9461	19519.76	248.9613	19520.84	248.966	19519.64	249.9639	19513.16	248.9497	19518.2	248.9485	19516.25	248.9426
249.1919	19520.45	249.1933	19521.16	249.208	19513.35	250.2173	19512.69	249.1918	19517.59	249.1921	19514.08	249.1962
249.4458	19516.45	249.4453	19513.09	249.4457	19517.25	250.4635	19519.63	249.4456	19516.94	249.4442	19519.8	249.4443
249.6957	19521.73	249.6901	19515.13	249.6957	19521.32	250.7172	19517.67	249.6927	19514.89	249.6932	19522.17	249.6959
249.9481	19522.57	249.9482	19512.38	249.9402	19518.28	250.967	19515.26	249.9483	19514.07	249.9476	19511.48	249.9416
250.192	19515.76	250.1921	19517.12	250.1927	19515.71	251.2182	19512.17	250.1931	19513.32	250.198	19516.83	250.1977
250.4449	19519.14	250.4471	19516.49	250.4394	19516.8	251.4642	19510.55	250.4465	19517.39	250.4446	19512.64	250.4463
250.6956	19519.84	250.6919	19514.58	250.6949	19519.81	251.7139	19513.79	250.6932	19515.46	250.6927	19519.22	250.7042
250.9482	19517.15	250.9485	19517.29	250.9388	19512.25	251.9669	19517.7	250.9498	19518.76	250.9474	19515.58	250.9504
251.1912	19517.48	251.1899	19515.93	251.1916	19512.67	252.218	19513.88	251.196	19516.68	251.1951	19511.04	251.1963
251.4458	19513.47	251.4455	19514.77	251.4386	19514.57	252.4649	19515.38	251.4505	19518	251.4455	19513.7	251.4468
251.6967	19521.23	251.6941	19514.87	251.6974	19515.82	252.7177	19513.88	251.6996	19512.67	251.6913	19510.81	251.6972
251.9515	19519.56	251.9479	19519.2	251.9421	19515.18	252.9736	19512.17	251.9491	19514.23	251.9497	19517.28	251.9428
252.1924	19524.32	252.1923	19513.46	252.1928	19514.49	253.2183	19512.3	252.193	19519.19	252.1983	19513.75	252.1983
252.4447	19517.76	252.4444	19514.96	252.4385	19518.05	253.4689	19509.55	252.4464	19518.08	252.4464	19514.63	252.4455
252.6963	19517.84	252.6934	19514.8	252.6986	19511.2	253.7187	19516.84	252.6935	19515.98	252.6924	19519.14	252.6969
252.9479	19516.55	252.9519	19512.55	252.9402	19514.77	253.9651	19514	252.9502	19518.96	252.9539	19517.85	252.9487
253.1941	19517.45	253.1874	19514.34	253.1928	19515.25	254.2192	19514.52	253.1947	19516.83	253.2004	19512.23	253.1986
253.4464	19521.88	253.4481	19518.3	253.4383	19517	254.4781	19510.99	253.4475	19516.07	253.4474	19517.1	253.4477
253.6978	19519.63	253.693	19516.4	253.6996	19514.78	254.7172	19510.76	253.6939	19514.2	253.6935	19517.27	253.6993
253.9491	19512.67	253.9528	19518.01	253.9411	19512.27	254.9693	19517.85	253.9508	19517.06	253.9481	19517.62	253.9437
254.1929	19517.79	254.1891	19515.02	254.1933	19515.92	255.2175	19514.24	254.1905	19518.12	254.196	19515.67	254.1996
254.447	19519.2	254.4471	19519.16	254.4391	19517.64	255.4645	19515.28	254.4485	19515.87	254.4462	19511.71	254.4464
254.6963	19516.8	254.693	19511.32	254.7022	19512.62	255.7176	19512.96	254.6949	19514.42	254.6954	19520.25	254.6971
254.9474	19516.23	254.9499	19518.27	254.9437	19514.2	255.9693	19517.9	254.9521	19518.26	254.9521	19516.23	254.9435
255.1948	19517.2	255.1937	19518.61	255.1954	19512.43	256.2259	19515.3	255.1889	19517.47	255.197	19514.95	255.1984
255.4501	19516.12	255.4479	19516.14	255.438	19515.03	256.4665	19515.13	255.4487	19517.55	255.446	19518.13	255.4469
255.6969	19516.24	255.7002	19515.73	255.6962	19514.1	256.7178	19513.1	255.6949	19519.88	255.6942	19514.97	255.699
255.95	19512.07	255.951	19513.95	255.9418	19511.29	256.9675	19512.43	255.9502	19517.08	255.9499	19518.02	255.9443
256.1933	19518.67	256.1887	19512.85	256.1939	19515.18	257.2208	19514.74	256.2001	19519.64	256.1948	19514.67	256.2113
256.4468	19519.08	256.4471	19513.05	256.4395	19515.08	257.4654	19513.29	256.4479	19516.47	256.4471	19517.18	256.4647
256.6977	19517.8	256.6932	19515.31	256.6979	19514.78	257.7181	19514.85	256.6951	19519.91	256.6937	19517.27	256.7111
256.9502	19517.85	256.9506	19518.11	256.9422	19515.97	257.9659	19519.99	256.9488	19515.01	256.9548	19521.16	256.9563
257.1898	19520.4	257.194	19518.47	257.194	19519.96	258.2212	19513.81	257.1943	19516.15	257.2162	19513.38	257.204
257.4468	19519.02	257.4469	19515.27	257.4409	19515.33	258.4661	19514.44	257.4493	19516.14	257.453	19518.22	257.4569
257.7017	19516.33	257.6944	19513.7	257.6989	19512.27	258.7204	19513.95	257.6967	19518.65	257.6929	19518.12	257.7044
257.9469	19523.43	257.9522	19516.59	257.9403	19514.2	258.9654	19512.75	257.9513	19515.73	257.9533	19520.96	257.9518
258.1951	19521.38	258.193	19516.8	258.1936	19512.75	259.2229	19515.05	258.191	19513.07	258.1941	19514.92	258.2014
258.4468	19514.27	258.449	19516.88	258.4413	19513.19	259.467	19515.74	258.4508	19514.29	258.4464	19519.45	258.4509
258.7027	19515.99	258.6945	19517.76	258.7036	19516.54	259.7194	19519.05	258.694	19516.04	258.696	19516.53	258.6993
258.9489	19517.82	258.9534	19510.69	258.9474	19514.72	259.9681	19515.51	258.9546	19516.63	258.9513	19517.97	258.9458
259.1956	19517.01	259.1916	19513.57	259.1949	19513.84	260.2215	19515.78	259.1966	19516.28	259.1976	19514.91	259.2027
259.4483	19518.75	259.4503	19514.92	259.4426	19521.23	260.4672	19514.3	259.45	19518.31	259.4515	19517.78	259.45
259.6993	19516.52	259.6958	19511.31	259.7001	19517.16	260.7223	19513.73	259.703	19514.99	259.6968	19511.34	259.6998
259.9458	19523.68	259.9517	19516.1	259.9418	19512.04	260.9681	19514.68	259.9507	19517.13	259.953	19522.45	259.9462
260.1961	19524.92	260.1934	19514.98	260.1944	19515.69	261.222	19513.97	260.1921	19512.52	260.2033	19511.96	260.2003
260.4485	19519.43	260.4483	19517.96	260.4411	19517.23	261.4674	19515.06	260.4511	19515.08	260.4537	19516.12	260.4511
260.6985	19519.05	260.6951	19517.1	260.6978	19514.33	261.7224	19515.24	260.6905	19513.1	260.6993	19515.83	260.7004
260.9501	19517	260.9517	19515.02	260.9427	19513.67	261.9705	19513.63	260.9543	19518.49	260.9612	19513.88	260.946
261.1905	19518.66	261.194	19514.17	261.1954	19514.26	262.2262	19514.67	261.1988	19513.3	261.2163	19512.17	261.2021

261.4497	19524.65	261.4492	19515.02	261.4428	19513.44	262.4704	19514.55	261.4529	19516.21	261.4675	19518.76	261.4525
261.695	19518.06	261.694	19515.21	261.7081	19515.47	262.7237	19515.15	261.6976	19517.34	261.7148	19516.55	261.7025
261.9473	19512.14	261.954	19511.04	261.9636	19515.69	262.9704	19511.33	261.9534	19515.72	261.9685	19514.44	261.9514
262.1968	19516	262.1952	19513.03	262.2087	19515.38	263.2212	19511.58	262.1969	19512.75	262.2168	19517.84	262.2067
262.4501	19518.42	262.4498	19511.68	262.4518	19516.27	263.47	19513.09	262.4521	19514.84	262.4672	19514.81	262.4606
262.6966	19518.86	262.6929	19517.53	262.7006	19514.75	263.7209	19513.9	262.6972	19517.27	262.7157	19517.94	262.7115
262.9529	19518.11	262.9565	19515.38	262.9428	19518.24	263.9693	19513.1	262.9527	19516.11	262.9696	19514.2	262.9607
263.1949	19514.8	263.2	19514.35	263.1975	19515.56	264.2238	19513.18	263.1957	19516.32	263.2215	19517.45	263.2123
263.4495	19519.84	263.448	19516.7	263.4556	19515.85	264.4682	19509.56	263.4521	19517.35	263.4727	19513.09	263.4678
263.6979	19513.64	263.6966	19517.59	263.7077	19510.86	264.7233	19515.32	263.6984	19516.63	263.9563	19516.92	263.7203
263.9526	19519.27	263.955	19513.65	263.9507	19512.23	264.9727	19514.07	263.9541	19514.76	264.2062	19512.27	263.9679
264.1962	19517.25	264.1991	19515.18	264.198	19513.12	265.225	19515.62	264.1973	19516.24	264.4592	19516.79	264.2198
264.4511	19519.28	264.45	19518.06	264.4423	19514.5	265.4711	19512.95	264.4516	19515.45	264.7087	19516.72	264.4729
264.6984	19519.7	264.6944	19511.75	264.6986	19513.82	265.7212	19517.1	264.6994	19516.3	264.9635	19515.54	264.9508
264.9535	19520.45	264.9565	19519.43	264.9441	19512.99	265.9724	19513.65	264.9527	19515.8	265.2137	19514.06	265.2072
265.1998	19514.1	265.2028	19516.89	265.1983	19510.85	266.2282	19516.83	265.1985	19516.41	265.4667	19515.92	265.462
265.4495	19515	265.4486	19515.57	265.4438	19518.84	266.4753	19515.48	265.4525	19517	265.7175	19515.16	265.7202
265.6976	19519.3	265.6955	19515.04	265.712	19513	266.7264	19515.41	265.6989	19517.97	265.9731	19514.78	265.9686
265.9528	19517.98	265.9554	19517.01	265.9623	19513.94	266.9731	19516.12	265.9567	19517.23	266.2229	19509.17	266.2218
266.1975	19516.85	266.1995	19515.11	266.2123	19514.26	267.2272	19514.37	266.1944	19514.87	266.4765	19517.58	266.4834
266.4497	19520.47	266.4467	19513.62	266.4567	19514.36	267.474	19512.22	266.4556	19517.36	266.9637	19519.36	266.9635
266.7099	19523.29	266.6986	19513.1	266.7108	19512.17	267.7237	19515.1	266.6979	19515.41	267.2136	19515.06	267.2191
266.9634	19515.98	266.9523	19511.48	266.9553	19514.21	267.9697	19511.39	266.9583	19513.5	267.4688	19513.4	267.4711
267.2082	19518.42	267.1986	19514.3	267.2036	19515.31	268.2307	19514.48	267.1938	19515.05	267.7188	19517.96	267.7261
267.456	19514.96	267.4455	19512.95	267.4494	19511.44	268.4798	19516.27	267.4532	19515.79	267.9903	19518.42	267.9773
267.7058	19518.83	267.7016	19519.48	267.7197	19518.54	268.7301	19512.92	267.6997	19518.05	268.4738	19521.31	268.2323
267.9574	19518.83	267.9536	19519.55	267.9665	19512.39	268.983	19511.36	267.9537	19516.39	268.7251	19514.38	268.4857
268.2036	19518.43	268.1993	19518.06	268.2167	19514.15	269.24	19512.29	268.2119	19519.4	268.9852	19514.78	268.7434
268.4532	19519.39	268.4478	19515.25	268.4619	19513.5	269.4865	19512.47	268.4555	19516.04	269.2369	19519.89	269.2288
268.7028	19515.97	268.6976	19515.44	268.7185	19512.82	269.7373	19514.66	268.6992	19516.39	269.4985	19515.74	269.4873
268.9591	19519.98	268.953	19518.61	268.9687	19515.42	269.9883	19515.49	268.9574	19519.08	269.9932	19517.97	269.7413
269.2095	19519.09	269.1993	19514.21	269.22	19516.83	270.2419	19513.2	269.1982	19513.77	270.2504	19519.11	269.9941
269.4596	19520.14	269.446	19517.02	269.4647	19516.45	270.4911	19512.47	269.4562	19515.93	270.5089	19519.68	270.2492
269.711	19515.97	269.6987	19516.26	269.7177	19516.47	270.7425	19513.27	269.6994	19521.33	270.9969	19518.59	270.7327
269.9644	19525.34	269.9533	19516.34	270.2036	19513.47	270.9941	19515.63	269.9559	19514.69	271.2513	19514.33	270.986
270.2122	19517.38	270.1984	19516.83	270.4494	19515.48	271.4747	19510.09	270.2016	19516.83	271.5101	19517.53	271.2508
270.4681	19517.18	270.4485	19512.32	270.7059	19516.31	271.7289	19515.23	270.4559	19514.58	271.7657	19514.13	271.5138
270.7198	19518.1	270.697	19518.99	270.9544	19514.42	271.9781	19512.81	270.7005	19516.31	272.2506	19519.75	271.998
270.9752	19517.03	270.9608	19516.48	271.2049	19515.21	272.2352	19513.24	270.9555	19516.93	272.5063	19515.71	272.2501
271.4598	19516.5	271.2099	19513.32	271.4528	19513.49	272.4851	19514.52	271.2028	19518.15	272.7642	19519.68	272.5068
271.7227	19517.26	271.4605	19516.73	271.7093	19514.34	272.7388	19513.93	271.4552	19516.87	273.0222	19517.96	272.7636
271.9809	19517.84	271.7104	19511.05	271.958	19518.39	272.993	19514.34	271.7011	19516.71	273.5081	19520.68	273.0157
272.4649	19515.46	271.9695	19518.41	272.2119	19515.16	273.2467	19512.77	271.955	19515.11	273.7634	19517.64	273.2717
272.7167	19516.76	272.2183	19515.03	272.4607	19517.13	273.4963	19512.03	272.2044	19520.85	274.0214	19517.67	273.758
272.972	19518.99	272.4705	19517.57	272.7176	19513.48	273.751	19511.36	272.461	19518.6	274.2775	19513.75	274.0117
273.2224	19514.27	272.7231	19518.19	272.9667	19514.33	274.2386	19513.72	272.7111	19514.88	274.7624	19517.79	274.2673
273.4781	19517.79	273.208	19517.36	273.2202	19513.57	274.4929	19513.71	272.9661	19515.17	275.0218	19512.77	274.5287
273.7287	19519.57	273.4589	19511.43	273.4685	19511.3	274.7525	19515.62	273.2175	19516.24	275.2779	19517.06	275.0129
273.984	19514.84	273.7096	19516.21	273.7272	19516.85	275.0159	19515.16	273.4706	19513.43	275.5364	19513.32	275.2708
274.4862	19522.57	273.9706	19512.37	274.2099	19516.93	275.5044	19513.86	273.7224	19515.36	276.0292	19515.23	275.5319
274.7399	19513.45	274.2206	19514.11	274.4614	19518.75	275.7671	19512.78	274.21	19515.28	276.2865	19514.98	276.0161
275.2253	19520.11	274.4736	19516.01	274.7183	19514.69	276.0294	19513.66	274.4671	19512.8	276.5459	19511.23	276.2724
275.4788	19515.42	274.7252	19517.09	274.9753	19515.71	276.5248	19514.49	274.7173	19516.48	277.0356	19516.87	276.533
275.7306	19516.97	274.9822	19511.01	275.2298	19513.6	276.7813	19512.48	274.9752	19516.44	277.2933	19511.79	276.7911
275.9873	19519.47	275.4691	19514.36	275.4791	19515.32	277.0432	19514.6	275.2267	19512.88	277.5521	19513.33	277.276
276.2384	19516.35	275.7204	19514.38	275.7378	19513.69	277.5249	19515.03	275.7119	19516.05	278.04	19513.99	277.5372
276.4933	19510.81	275.9805	19511.07	276.2219	19515.88	277.782	19513.44	275.9695	19516.19	278.2996	19514.56	277.7943
276.982	19521.03	276.2361	19513.18	276.4722	19517.15	278.0417	19512.13	276.2234	19515.54	278.5597	19514.58	278.2795
277.2327	19514.84	276.7217	19518.01	276.7333	19517.82	278.3023	19513.27	276.4745	19515.07	279.0469	19514.71	278.5416
277.4865	19520.67	276.9789	19513.66	276.9869	19514.47	278.79	19513.15	276.7259	19516.65	279.3053	19520.22	278.8001
277.7431	19519	277.2326	19521.99	277.2453	19513.68	279.0503	19514.39	276.9831	19515.48	279.5658	19518.03	279.2878
278	19509.93	277.4875	19516.42	277.7334	19516.25	279.3313	19515.62	277.471	19514.01	280.071	19517.21	279.551
278.4871	19516.26	277.7416	19517.32	277.986	19517.9	279.8204	19512.83	277.7225	19512.15	280.5591	19519.82	279.8092
278.7404	19516.9	278.2292	19515.18	278.2463	19517.44	280.0795	19511.35	277.9814	19516.08	280.8202	19515.13	280.2966
278.9998	19520.2	278.486	19514.38	278.4995	19513.5	280.3381	19513.22	278.2375	19516.48	281.0843	19515.15	280.5602
279.2523	19517.43	278.7382	19508.77	278.9797	19512.64	280.8231	19513.83	278.7241	19517.71	281.5715	19512.74	280.819
279.5096	19519.34	279	19515.21	279.2395	19518.76	281.0791	19512.69	278.9791	19514.38	281.8346	19518.73	281.309
279.9998	19516.68	279.4899	19513.37	279.4933	19512.98	281.3383	19515	279.2543	19519.07	282.0973	19512.61	281.574
280.2548	19516.72	279.7435	19517	279.7552	19513.93	281.5919	19516.04	279.7411	19514.13	282.5852	19520.83	282.0629
280.5105	19514.73	280.0054	19514.05	280.2416	19517.13	282.0875	19510.82	280.005	19513.21	282.8487	19513.49	282.3224

280.779	19516.52	280.4952	19516.09	280.4937	19515.28	282.3512	19514.95	280.2583	19513.83	283.1109	19514.84	282.5758
281.2672	19518.69	280.7496	19512.75	280.753	19512.01	282.8403	19516.56	280.7498	19513.86	283.5977	19514.57	282.8247
281.524	19520.94	281.0123	19515.27	281.0079	19510.95	283.1012	19513.76	281.0074	19514.42	283.8654	19513.01	283.0753
281.7812	19519.85	281.5022	19515.76	281.2647	19516.31	283.36	19517.5	281.271	19517.86	284.3579	19513.23	283.324
282.0391	19518.14	281.7567	19513.7	281.7528	19514.08	283.8478	19512.31	281.7595	19513.22	284.6212	19514.57	283.5788
282.5341	19515.93	282.0246	19512.59	282.0058	19517.81	284.1008	19513.75	282.0195	19517.62	285.1257	19517.38	284.0712
282.7962	19514.61	282.5168	19511.87	282.263	19517.8	284.35	19514.19	282.2782	19517.93	285.3929	19512.98	284.3222
283.2891	19517.69	282.7711	19514.22	282.5153	19513.6	284.5971	19513.44	282.7646	19516.57	285.8851	19509.85	284.5777
283.5469	19520.56	283.0385	19513.59	282.7782	19515.73	284.8489	19516.33	283.0258	19514.4	286.1457	19516.43	284.828
283.8068	19515.41	283.5297	19513.09	283.2633	19518.42	285.1013	19518.88	283.2874	19514.59	286.4105	19512.66	285.0774
284.2953	19515.08	283.7888	19517.93	283.5191	19518.26	285.3521	19513.71	283.7765	19514.6	286.9025	19518.6	285.328
284.5559	19516.32	284.0651	19517.02	283.7811	19513.42	285.5988	19510.07	284.0388	19518.35	287.1626	19513.75	285.583
284.8158	19520.51	284.556	19516.36	284.2679	19513.54	285.8519	19519.83	284.2989	19513.9	287.4272	19515.47	285.8338
285.3028	19518.15	284.8158	19510.26	284.5212	19510.91	286.1218	19514.82	284.7913	19519.89	287.9237	19518.62	286.0848
285.5635	19519.53	285.3076	19515.15	284.7836	19515.85	286.6164	19510.44	285.0515	19514.97	288.1857	19512.96	286.3355
285.823	19516.82	285.5683	19515.97	285.0403	19515.3	286.8677	19515.87	285.3132	19515.47	288.676	19515.36	286.8254
286.3161	19518.58	285.8294	19516.88	285.5265	19518.88	287.1224	19513.68	285.8058	19514.65	288.9457	19519.55	287.077
286.5779	19514.06	286.3187	19516.61	285.7887	19514.17	287.376	19513.1	286.0689	19516	289.4403	19517.03	287.3314
286.8387	19514.33	286.5781	19514.59	286.0458	19508.78	287.873	19516.53	286.5576	19513.46	289.7041	19511.44	287.5915
287.3295	19512.95	286.8404	19515	286.5303	19513.93	288.1409	19513.12	286.8178	19514.92	290.1947	19519.96	288.081
287.5908	19515.77	287.3296	19516.02	286.7928	19516.43	288.6342	19512.37	287.0794	19516.01	290.461	19515.34	288.3332
287.8546	19519.15	287.5884	19515.55	287.0479	19511.05	288.886	19513.22	287.5686	19514.69	290.9589	19510.32	288.5896
288.3462	19518.99	287.8503	19516.18	287.308	19515.16	289.1397	19511.02	287.8308	19517.75	291.2226	19508.66	288.8426
288.6089	19514.32	288.3393	19512.12	287.7965	19513.95	289.3916	19513.99	288.0931	19515.82	291.7137	19513.66	289.0985
289.0979	19519.36	288.5858	19513.62	288.053	19512.73	289.6387	19512.2	288.5837	19518.65	291.9835	19518.51	289.5949
289.3579	19519.21	288.8464	19514.06	288.316	19513.83	289.8913	19512.35	288.8447	19513.98	292.4752	19514.04	289.8481
289.6242	19516.66	289.0967	19515.76	288.8041	19511.23	290.1454	19513.25	289.1154	19510.95	292.7405	19521.23	290.1012
290.1153	19516.68	289.3452	19517.04	289.062	19514.27	290.6375	19517.52	289.6058	19515.88	293.2319	19509.27	290.3536
290.3785	19514.64	289.5948	19514.85	289.3244	19512.72	290.8885	19512.54	289.8666	19511.13	293.4989	19519.4	290.8439
290.8742	19514.28	289.8476	19515.18	289.8111	19513.79	291.1417	19509.66	290.1305	19516.92	293.9939	19522.44	291.0978
291.1206	19515.3	290.0989	19516.9	290.0616	19519.68	291.3936	19512.02	290.6212	19516.4	294.2587	19515.45	291.3504
291.3716	19519.99	290.3468	19518.43	290.3161	19516.37	291.6411	19515.69	290.8848	19515.1	294.7509	19510.85	291.6111
291.623	19517.8	290.595	19512.1	290.5669	19513.65	291.8946	19512.64	291.3802	19513.57	295.0209	19511.48	292.1055
291.876	19518.76	290.8468	19518.57	290.8173	19515.4	292.1496	19515.41	291.6405	19517.41	295.5151	19516.05	292.3604
292.1241	19519.56	291.0989	19515.49	291.065	19518.77	292.6419	19513.35	292.1358	19517.64	295.7836	19518.45	292.6194
292.3766	19514.52	291.3464	19517.88	291.3156	19513.62	292.8951	19512.73	292.3989	19517.53	296.2743	19518.32	293.1123
292.631	19519.37	291.5965	19514.07	291.5666	19511.9	293.151	19513.63	292.8922	19513.61	296.5496	19517.07	293.3651
292.8865	19517.22	291.8515	19516.72	291.8189	19513.65	293.6434	19512.89	293.1563	19514.72	297.0466	19512.98	293.625
293.3825	19516.94	292.1029	19513.98	292.0673	19510.74	293.906	19512.6	293.6481	19517.96	297.306	19514.25	294.1201
293.6388	19521.63	292.5964	19516.18	292.3159	19509.8	294.1688	19510.62	293.9118	19517.8	297.8056	19517.68	294.3798
293.8943	19515.15	292.8513	19516.37	292.5655	19513.84	294.6588	19513.98	294.4078	19517.77	298.0629	19518.29	294.8723
294.3893	19519.72	293.1072	19515.33	292.8197	19517.35	294.9111	19515.46	294.6668	19511.54	298.5553	19516.61	295.1409
294.643	19519.08	293.6018	19515.72	293.0689	19516.51	295.1656	19512.52	295.1629	19515.95	298.8104	19514.9	295.6436
294.8975	19516.86	293.8577	19517.81	293.3209	19510.02	295.4183	19516.26	295.4268	19516.73	299.0698	19519.89	295.9097
295.3946	19514.81	294.1118	19512.4	293.5715	19517.85	295.6699	19513.83	295.9227	19513.33	299.562	19513.61	296.4035
295.6512	19514.2	294.606	19518.49	293.826	19518.92	295.9248	19512.4	296.1872	19519.67	299.8213	19516.09	296.6762
296.1425	19523.38	294.8609	19517.45	294.0757	19517.64	296.4163	19515.54	296.6784	19517.05	300.0808	19516.19	297.1744
296.3964	19518.48	295.1172	19516.6	294.3271	19514.31	296.668	19513.42	296.9422	19516.07			297.441
296.6716	19514.14	295.6104	19515.12	294.8164	19514.33	296.9242	19515.51	297.439	19516.01			297.9348
297.1643	19518.12	295.8662	19517.29	295.0658	19515.95	297.1845	19513.38	297.7008	19516.32			298.2051
297.4173	19516.45	296.121	19513.69	295.3162	19514.51	297.6751	19514.68	298.1961	19513.89			298.7047
297.6724	19517.99	296.6171	19517.04	295.5654	19516.28	297.9414	19513.96	298.4654	19516.68			298.9708
297.928	19519.16	296.8754	19510.74	295.8199	19514.13	298.4321	19513.91	298.962	19517.3			299.4652
298.4244	19517.08	297.3691	19516.05	296.0699	19515.85	298.6994	19517.03	299.2299	19513.45			299.7483
298.6805	19521.01	297.6218	19515.88	296.3209	19512.77	299.1968	19511.65	299.7227	19515.21			300.2444
299.1773	19515.74	297.8824	19514.89	296.5744	19516.61	299.4631	19513.65	299.9905	19518.32			
299.4318	19516.62	298.3781	19517.05	296.8297	19514.29	299.9569	19512.5	300.4856	19514.54			
299.6879	19510.41	298.6404	19511.78	297.0824	19512.94	300.2304	19513.46					
300.1812	19514.22	299.1376	19516.22	297.5733	19517.29							
		299.3905	19517.93	297.8271	19511.05							
		299.6452	19516.68	298.0794	19516.09							
		300.1411	19518.35	298.343	19514.29							
				298.8341	19510.31							
				299.0889	19520.06							
				299.3426	19510.94							
				299.6009	19521.63							
				300.0913	19512.1							

1.43							1.43-Cycle					
67.5							30					
2	x_3		x_4		x_5		x_1		x_2		x_3	
P (cm^{-1})	t (s)	P (cm^{-1})	t (s)	P (cm^{-1})	t (s)	P (cm^{-1})	t (s)	P (cm^{-1})	t (s)	P (cm^{-1})	t (s)	P (cm^{-1})
19528.2	0	19530.39	0	19530.85	0	19527.54	0	19596.82	0	19598.9	0	19598.36
19529.29	0.66011	19531.27	0.66383	19525.8	0.66031	19524.07	0.68824	19598.58	1.47199	19599.31	1.92747	19598.68
19530.11	0.84829	19528.23	0.85365	19528.05	0.85326	19525.9	0.84971	19597.61	1.85389	19598.38	2.42013	19598.43
19531.12	1.1037	19525.17	1.10735	19527.69	1.10872	19526.97	1.10477	19597.12	2.07258	19597.02	2.52831	19597.79
19534.95	1.34777	19529.47	1.35297	19527.59	1.35848	19526.44	1.35728	19598.1	2.32899	19600.54	2.77918	19597.61
19524.94	1.59852	19524.03	1.60272	19528.91	1.60303	19525.97	1.60459	19596.39	2.57426	19597.05	3.02695	19600.03
19527.71	1.84622	19530.77	1.85131	19527.15	1.85186	19526.08	1.84564	19599.14	2.82508	19598.97	3.27664	19598.89
19534.1	2.10051	19529.02	2.10204	19528.59	2.1001	19526.52	2.10244	19599.23	3.07275	19598.1	3.52738	19598.71
19531.45	2.3469	19527.3	2.35324	19530.15	2.35223	19528.23	2.34771	19597.01	3.32305	19596.62	3.77782	19599.65
19530.5	2.59728	19528.07	2.60218	19527.39	2.60209	19526.89	2.59829	19597.57	3.57449	19599.78	4.02802	19599.1
19531.86	2.84856	19526.49	2.8516	19529.63	2.85068	19526.61	2.84634	19598.21	3.82621	19597.74	4.27871	19599.69
19529.76	3.09766	19530.64	3.10171	19526.9	3.10023	19530.11	3.09761	19598.02	4.07626	19596.15	4.52753	19599.89
19528.53	3.34764	19525.46	3.35204	19525.38	3.35154	19528.04	3.3475	19596.34	4.32457	19598.32	4.78222	19598.57
19528.66	3.59808	19532.21	3.60384	19531.14	3.60207	19525.41	3.6001	19596.46	4.57388	19595.9	5.03187	19599.38
19531.77	3.84751	19529.54	3.85193	19525.21	3.85146	19526.42	3.8463	19598.17	4.8239	19599.19	5.27872	19599.8
19526.46	4.10007	19528.22	4.10456	19526.02	4.10118	19524.46	4.09738	19597.31	5.07366	19598.23	5.52879	19598.23
19533.12	4.34699	19527.68	4.35221	19525.42	4.35215	19529.45	4.34888	19595.25	5.32546	19598.79	5.77883	19601.47
19530.04	4.59724	19525.26	4.60249	19524.03	4.605	19524.55	4.59892	19597.17	5.57434	19599.47	6.02826	19600.3
19531.1	4.84877	19527.66	4.85788	19525.25	4.86096	19525.9	4.84734	19599.36	5.82412	19597.26	6.27846	19598.51
19533.69	5.10069	19525.99	5.10898	19526.54	5.10405	19529.82	5.10243	19595.89	6.07302	19599.13	6.52818	19598.81
19530.57	5.35429	19529.44	5.35252	19527.03	5.35226	19524.49	5.35392	19598.42	6.32372	19600.22	6.78019	19599.36
19534.24	5.60302	19524.73	5.60272	19529.37	5.60165	19528.53	5.60234	19595.59	6.5749	19599.8	7.02927	19598.15
19527.64	5.84939	19530.87	5.85169	19523.81	5.85104	19524.97	5.84781	19595.28	6.82589	19598.66	7.28578	19600.66
19526.72	6.10061	19527.62	6.10278	19526.11	6.10235	19526.08	6.09819	19599.2	7.07415	19598.47	7.5284	19599.35
19530.5	6.34914	19530.77	6.35278	19529.94	6.35321	19529.86	6.34962	19596.07	7.32367	19598.22	7.77899	19600.01
19531.07	6.599	19530.59	6.60387	19523.26	6.60283	19526.85	6.60156	19599.16	7.57451	19599.1	8.02948	19599.92
19529.44	6.84869	19527.55	6.85339	19527.47	6.85316	19530.43	6.84926	19596.43	7.82555	19597.59	8.28061	19597.67
19529.77	7.10018	19530.55	7.10362	19527.76	7.11615	19526.68	7.09935	19595.65	8.07523	19600.12	8.52938	19600.29
19528.85	7.34822	19526.11	7.35215	19527.5	7.35648	19523.64	7.34964	19598.05	8.32451	19598.01	8.77869	19598.2
19526.27	7.59905	19527.44	7.60273	19529.28	7.6034	19527.6	7.60291	19598.9	8.57388	19599.83	9.02887	19599.2
19531.63	7.84939	19525.55	7.85412	19528.6	7.85351	19527.41	7.84902	19596.52	8.82601	19598.18	9.2803	19598.41
19528.12	8.10369	19529.71	8.10887	19528.24	8.10334	19527.27	8.10173	19598.35	9.07633	19599.05	9.53011	19600.84
19529.22	8.35074	19525.9	8.35427	19526.19	8.35383	19525.36	8.34887	19596.67	9.32553	19596.88	9.78005	19599.33
19527.68	8.60006	19526.74	8.60315	19526.41	8.60225	19526.62	8.60244	19600.07	9.57451	19594.88	10.02952	19599.2
19531.11	8.85088	19531.64	8.85321	19528.73	8.85318	19527.05	8.84744	19596.91	9.82547	19599.32	10.27987	19600.88
19530.36	9.10215	19530.65	9.10399	19527.81	9.10589	19527.64	9.10282	19598.33	10.07411	19599.4	10.53082	19599.53
19531.16	9.34946	19529.07	9.35384	19532.25	9.35358	19529.53	9.34968	19597.47	10.3257	19598.34	10.78227	19596.19
19528.93	9.59868	19528.46	9.60467	19529.65	9.60303	19529.38	9.60214	19597.38	10.57779	19600.17	11.03046	19601.83
19531.42	9.8493	19527.22	9.85326	19525.4	9.85349	19525.68	9.84998	19597.07	10.82695	19595.4	11.28029	19600.92
19529.61	10.10345	19529.45	10.1042	19528.13	10.10392	19525.79	10.09904	19597.23	11.07541	19599.28	11.53024	19600.92
19534.41	10.35105	19532.17	10.35486	19527.77	10.3559	19526.13	10.34877	19599.57	11.32609	19598.05	11.78212	19600.51
19528.39	10.60133	19526.54	10.6062	19524.13	10.60513	19527.35	10.60499	19599.08	11.57658	19598.98	12.03196	19600.2
19528.42	10.85042	19530.54	10.85448	19529.83	10.85507	19529.16	10.85164	19597.3	11.82739	19601.45	12.2823	19598.79
19527.11	11.10158	19529.48	11.10491	19525.89	11.10418	19521.87	11.10226	19597.21	12.08845	19598.97	12.53033	19600.45
19530.32	11.35079	19529.07	11.35668	19527.35	11.35554	19526.24	11.35056	19597.16	12.32934	19597.8	12.78028	19599.8
19528.79	11.60098	19528.12	11.60586	19526.77	11.60533	19528.09	11.60386	19596.71	12.57602	19598	13.0307	19598.19
19532.58	11.85114	19529.46	11.85509	19527.98	11.85513	19527.47	11.85234	19596.88	12.82792	19597.03	13.28196	19600.07
19530.68	12.10506	19529.27	12.10573	19524.44	12.10456	19526.79	12.10318	19597.49	13.07693	19596.85	13.53172	19600.41
19532.61	12.35094	19530.19	12.35462	19528.38	12.3542	19525.76	12.35122	19597.64	13.32561	19596.04	13.78142	19597.69
19529.6	12.60096	19530.4	12.60427	19524.94	12.60515	19526.57	12.60261	19594.41	13.57553	19597.17	14.03085	19600.6
19533.25	12.85002	19525.38	12.85488	19527.58	12.85624	19527.97	12.85017	19596.48	13.82771	19598.62	14.28378	19599.41
19525.96	13.10299	19530.05	13.10609	19528.78	13.10393	19530.18	13.10351	19598.52	14.07814	19598.5	14.53165	19599.7
19531.61	13.35075	19528.14	13.3567	19527.53	13.35495	19527.01	13.35153	19598.47	14.32727	19600.76	14.78236	19598.8
19525.15	13.60022	19522.27	13.60527	19526.97	13.60559	19524.83	13.60245	19595.84	14.57614	19597.55	15.03112	19599.93
19530.01	13.85132	19528.25	13.85786	19526.55	13.85685	19530.18	13.8521	19597.64	14.82708	19598.11	15.28498	19598.5
19529.6	14.10176	19528.64	14.10542	19530.29	14.10541	19524.06	14.10107	19596.31	15.07648	19596.32	15.53232	19595.97
19527.81	14.3518	19525.85	14.35587	19528.4	14.35493	19527.94	14.35006	19597.51	15.33296	19598.75	15.7834	19600.29
19528.33	14.60196	19531.62	14.60556	19526.66	14.60529	19525.56	14.60277	19597.96	15.57759	19600.38	16.03453	19599.23
19534.25	14.85036	19527.45	14.8552	19526.63	14.8558	19525.2	14.85292	19599.18	15.82884	19598.73	16.28529	19600.08
19530.47	15.10205	19527.97	15.1056	19528.2	15.10589	19523	15.10283	19596.62	16.07792	19599.1	16.53406	19598.09
19526.48	15.35089	19530.28	15.35632	19530.32	15.35661	19528.43	15.35093	19598.06	16.32758	19598.7	16.78192	19599.1
19527.63	15.6031	19529.17	15.60731	19527.24	15.60475	19524.36	15.60401	19599.13	16.57731	19597.48	17.03236	19600.9
19530.79	15.85275	19524.43	15.85741	19529.3	15.85536	19527.46	15.85226	19596.54	16.8284	19598.94	17.28382	19598.49
19529.31	16.10541	19529.99	16.108	19525.88	16.10749	19524.35	16.10593	19596.88	17.07783	19596.99	17.53299	19601.82

19532.61	16.35147	19526.54	16.35615	19529.83	16.35739	19527.51	16.35207	19598	17.32748	19601.12	17.78243	19599.41
19527.32	16.60187	19529.75	16.60574	19527.83	16.60507	19526.39	16.60293	19598.18	17.57691	19596.6	18.03195	19600.97
19532.68	16.85679	19529.32	16.85578	19525.61	16.85679	19526.17	16.85254	19597.11	17.82838	19598.11	18.28367	19598.95
19524.63	17.10482	19526.65	17.10793	19528.66	17.10554	19528.05	17.10504	19598.97	18.07865	19600.04	18.534	19598.5
19532.08	17.35304	19526.99	17.35854	19524.67	17.3569	19526.65	17.3526	19596.79	18.32916	19598.41	18.78403	19600.51
19528.47	17.60214	19528.99	17.606	19530.36	17.6074	19525.51	17.60423	19597.68	18.5782	19598.24	19.03221	19597.56
19531.91	17.8515	19525.96	17.85688	19529.48	17.85555	19525.83	17.85137	19595.78	18.82819	19598.53	19.28428	19598.32
19530.02	18.10465	19529.36	18.10673	19524.49	18.10568	19524.81	18.10484	19596.66	19.07717	19597.71	19.53352	19599.09
19530.75	18.35299	19526.67	18.35668	19530.92	18.35635	19523.63	18.35165	19597.15	19.32816	19597.42	19.78397	19600.23
19528.34	18.60365	19526.81	18.60762	19526.15	18.60675	19528.03	18.60454	19597.49	19.57888	19599.02	20.03433	19597.67
19527.69	18.85176	19528.24	18.85646	19528.51	18.85787	19523.84	18.85271	19599.67	19.8296	19599.06	20.28609	19601.01
19524.35	19.10198	19525.67	19.10701	19523.93	19.10782	19526.45	19.1075	19595.85	20.07823	19597.45	20.53373	19599.72
19531.04	19.35298	19529.38	19.35687	19525.34	19.35626	19524.72	19.35115	19597.4	20.32835	19597.47	20.78485	19598.93
19529.97	19.60384	19527.7	19.60745	19525.16	19.60644	19524.91	19.60414	19596.35	20.58058	19598.05	21.03459	19598.43
19530.75	19.85337	19527.29	19.85844	19529.14	19.85737	19526.42	19.8533	19598.11	20.83075	19598	21.28648	19600.27
19533.12	20.10225	19528.3	20.10846	19530.3	20.10733	19526	20.10689	19598.19	21.08038	19594.68	21.53502	19599.59
19532.93	20.35344	19525.85	20.3581	19530.93	20.35797	19523.83	20.3528	19598.05	21.33115	19598.93	21.78501	19599.69
19529.12	20.60407	19528.16	20.60841	19524.16	20.60802	19528.6	20.60471	19595.34	21.57876	19597.55	22.03374	19596.47
19530.91	20.85363	19526.94	20.85891	19528.45	20.85736	19526.46	20.85307	19595.88	21.83178	19597.62	22.28588	19599.42
19529.32	21.10476	19533.73	21.10969	19530.23	21.10701	19526.36	21.10796	19597.69	22.0807	19599.26	22.53513	19598.61
19526.94	21.35562	19527.02	21.35858	19524.91	21.35866	19527.58	21.35535	19598.92	22.33844	19598.6	22.78492	19599.97
19531.38	21.60532	19525.22	21.60765	19525.78	21.60908	19529.42	21.60702	19596.3	22.58122	19598.16	23.0356	19598.63
19526.38	21.85546	19529.64	21.85878	19529.95	21.85897	19524.09	21.8535	19597.49	22.83061	19600.83	23.28589	19598.75
19533.53	22.10472	19526.13	22.11005	19526.73	22.10854	19527.37	22.10881	19599.09	23.07964	19599.87	23.53591	19597.3
19531.02	22.35523	19531.03	22.35911	19530	22.35881	19528.7	22.35437	19599	23.32989	19599.18	23.78729	19598
19531.07	22.605	19529.15	22.60858	19526.09	22.61037	19525.11	22.60649	19596.08	23.58061	19598.92	24.03578	19599.57
19533.25	22.85554	19526.15	22.85849	19527.29	22.85931	19522.22	22.8546	19598.08	23.83139	19596.1	24.28542	19599.82
19528.49	23.10429	19528.13	23.10938	19527.03	23.10748	19526.62	23.10963	19596.72	24.07986	19598.78	24.53439	19599.73
19527.55	23.35548	19530.94	23.35994	19527.82	23.35872	19527.21	23.35492	19595.99	24.32963	19600.63	24.787	19598.12
19528.81	23.60566	19526.36	23.60943	19526.41	23.60816	19528.05	23.60705	19597.66	24.58007	19597.59	25.03726	19599.63
19528.8	23.85455	19532.21	23.85823	19524.72	23.85894	19525.18	23.85443	19596.05	24.83194	19597.27	25.28646	19598.08
19530.8	24.10407	19527.44	24.10963	19525.38	24.10958	19528.91	24.10894	19598.7	25.08104	19600.48	25.53467	19601.36
19529.59	24.3552	19529.42	24.36936	19528.34	24.35915	19521.63	24.35756	19597.8	25.33082	19597.34	25.78592	19599.14
19530.27	24.60398	19530.96	24.61047	19528.28	24.61856	19529.43	24.60621	19598.22	25.57965	19598.54	26.04128	19599.98
19528.61	24.85749	19532.9	24.85953	19528.45	24.8592	19530.36	24.85397	19595.98	25.83138	19596.98	26.2865	19599.09
19530.93	25.10485	19526.2	25.10804	19530.11	25.10827	19523.43	25.10755	19596.11	26.08098	19600.21	26.53655	19597.85
19529.47	25.35367	19528.26	25.3584	19529.41	25.3605	19526.77	25.35718	19596.89	26.331	19597.56	26.78557	19598.45
19533.55	25.60452	19529.73	25.60957	19526.29	25.6092	19525.59	25.60814	19596.28	26.58106	19599.1	27.03459	19602.71
19528.6	25.85538	19527	25.85977	19524.7	25.85935	19527.54	25.85337	19595.61	26.83129	19598.83	27.28668	19599.02
19528.71	26.1058	19531.84	26.11021	19527.5	26.10806	19527.54	26.10553	19598.28	27.08014	19600.36	27.53731	19597.88
19531.45	26.35489	19530.17	26.35862	19526	26.35909	19524.92	26.35703	19598.01	27.33064	19597.73	27.79147	19600.6
19528.77	26.60429	19529.51	26.61015	19531.16	26.60973	19524.35	26.60728	19597.32	27.5813	19599.47	28.03566	19598.7
19527.93	26.85406	19525.74	26.85996	19527.66	26.85974	19525.6	26.85488	19598.38	27.83252	19597.57	28.28583	19599.2
19529.81	27.10539	19529.99	27.11108	19527.41	27.12054	19525.47	27.10709	19595.75	28.08183	19598.47	28.53674	19598.97
19528.83	27.35566	19529.46	27.35898	19527.44	27.35931	19528.47	27.35754	19598.17	28.33015	19598.87	28.78764	19598.92
19526.2	27.60493	19531.32	27.61311	19530.36	27.60831	19526.14	27.60699	19596.3	28.58116	19596.91	29.03739	19599.59
19526.61	27.85491	19528.49	27.86034	19528.68	27.85843	19528.46	27.85419	19597.62	28.83207	19599.96	29.28672	19599.61
19532.91	28.10449	19527.62	28.11173	19526.2	28.10975	19526.53	28.10688	19596.87	29.0823	19599.15	29.53619	19600.64
19526.69	28.35639	19531.46	28.36105	19524.73	28.36077	19526.88	28.36041	19598.36	29.33146	19596.61	29.78675	19600.01
19529.43	28.60643	19527.56	28.61022	19524.48	28.61052	19525.7	28.60652	19596.28	29.58047	19597.96	30.03706	19599.35
19527.59	28.85892	19527.95	28.85933	19526.44	28.85925	19532.13	28.85411	19595.01	29.83202	19600.69	30.28912	19597.87
19524.72	29.10519	19528.41	29.11005	19525.48	29.11075	19525.89	29.10799	19596.49	30.08165	19597.42	30.53784	19600.01
19533.35	29.36447	19526.93	29.36047	19526.7	29.35945	19525.17	29.36107	19597.74	30.33876	19598.94	30.7891	19598.24
19529.84	29.60588	19530.96	29.61121	19525.19	29.61012	19527.24	29.61	19595.55	30.58333	19596.46	31.03708	19602.51
19525.57	29.85738	19531.13	29.85943	19526.85	29.86119	19527.22	29.85535	19595.52	30.83412	19600.44	31.289	19599.39
19531.44	30.10707	19532	30.11108	19526.55	30.11172	19530.82	30.10692	19599.26	31.08276	19599.57	31.53882	19599.01
19527.24	30.35777	19526.87	30.36167	19525.53	30.36111	19525.02	30.36028	19595.57	31.33197	19597.38	31.78917	19601.17
19532.63	30.6061	19529.58	30.6119	19528.59	30.61151	19526.97	30.611	19598.48	31.58241	19599.61	32.03814	19598.79
19531.48	30.85833	19529.25	30.86162	19523.67	30.86209	19529.04	30.85764	19598.32	31.83359	19599.72	32.28888	19599.84
19530.34	31.10888	19534.07	31.11218	19525.83	31.11282	19526.19	31.1088	19597.82	32.08453	19598.11	32.53811	19599.94
19531.35	31.35775	19530.56	31.36226	19528.79	31.36313	19526.11	31.36288	19598.5	32.33397	19599.36	32.78911	19599.24
19528.66	31.60721	19532.21	31.61414	19528.91	31.61226	19528.12	31.61165	19596.79	32.58212	19600.28	33.03898	19598.84
19532.26	31.8577	19527.55	31.86263	19525.19	31.86091	19524.51	31.85739	19595.47	32.83372	19597.96	33.28724	19599.03
19529.49	32.10795	19529.49	32.11309	19529.48	32.11293	19526.9	32.10982	19597.48	33.08326	19600.45	33.53607	19599.02
19530.68	32.35907	19525.37	32.36123	19525.81	32.3618	19527.28	32.36164	19595.97	33.33422	19598.14	33.78836	19597.28
19533.26	32.60896	19528.75	32.61137	19524.81	32.61151	19527.27	32.61229	19598.73	33.58346	19598.8	34.03874	19601.97
19528.86	32.85841	19528.21	32.86112	19525.87	32.86137	19525.49	32.85581	19598.6	33.83347	19600.29	34.28822	19598.28
19527.77	33.1063	19531.7	33.11266	19525.15	33.11054	19529.55	33.10987	19599.31	34.08305	19599.81	34.5374	19598.19
19527.92	33.35822	19526.5	33.36058	19526.33	33.36137	19527.46	33.36198	19595.75	34.33293	19597.85	34.78861	19598.37
19528.27	33.60779	19530.01	33.61291	19524.65	33.61114	19524.81	33.61398	19597.06	34.58429	19595.05	35.03895	19599.28

19528.96	33.86212	19529.23	33.86266	19529.84	33.86317	19527.75	33.85601	19597.02	34.83437	19597.51	35.28917	19600.69
19529.21	34.10732	19524.73	34.11328	19529.11	34.11414	19524.81	34.10943	19598.53	35.08277	19598.46	35.53848	19598.52
19528.5	34.35786	19531.21	34.3635	19524.32	34.36376	19525.32	34.35978	19597.18	35.3325	19597.99	35.78753	19598.39
19525.74	34.60816	19527.05	34.61362	19523.69	34.61204	19527.7	34.61313	19592.81	35.58407	19598.63	36.03863	19600.93
19531.72	34.8589	19530.84	34.86198	19529.19	34.86054	19524.86	34.85677	19598.15	35.83548	19599.97	36.29029	19600.57
19525.58	35.10867	19529.92	35.11273	19528.19	35.11266	19525.68	35.10864	19596.83	36.08438	19597.63	36.53928	19597.96
19528.83	35.35764	19531.13	35.36333	19528.99	35.36263	19527.18	35.35956	19597.75	36.33365	19595.33	36.78773	19599.44
19531.59	35.60852	19526.23	35.6145	19528.08	35.61284	19526.99	35.6144	19595.93	36.58386	19597.38	37.037	19597.51
19533.01	35.86199	19530.95	35.86245	19529.03	35.86245	19525.38	35.85757	19593.94	36.83397	19597.04	37.28864	19601.42
19530.09	36.10918	19525.72	36.11204	19526.57	36.11262	19527.54	36.11128	19596.5	37.08492	19598.44	37.54042	19598.16
[truncated: 775,530 more chars]
